# Systematic Identification and Analysis of *Acinetobacter baumannii* Type VI Secretion System Effector and Immunity Components

**DOI:** 10.3389/fmicb.2019.02440

**Published:** 2019-10-30

**Authors:** Jessica M. Lewis, Deanna Deveson Lucas, Marina Harper, John D. Boyce

**Affiliations:** Infection and Immunity Program, Department of Microbiology, Monash Biomedicine Discovery Institute, Monash University, Clayton, VIC, Australia

**Keywords:** *Acinetobacter baumannii*, type VI secretion system, antibacterial toxins, effectors, phylogenetic analysis

## Abstract

Many Gram-negative bacteria use a type VI secretion system (T6SS) for microbial warfare and/or host manipulation. *Acinetobacter baumannii* is an important nosocomial pathogen and many *A. baumannii* strains utilize a T6SS to deliver toxic effector proteins to surrounding bacterial cells. These toxic effectors are usually delivered together with VgrG proteins, which form part of the T6SS tip complex. All previously identified *A. baumannii* T6SS effectors are encoded within a three- or four-gene locus that also encodes a cognate VgrG and immunity protein, and sometimes a chaperone. In order to characterize the diversity and distribution of T6SS effectors and immunity proteins in this species, we first identified all *vgrG* genes in 97 *A. baumannii* strains via the presence of the highly conserved VgrG domain. Most strains encoded between two and four different VgrG proteins. We then analyzed the regions downstream of the identified *vgrG* genes and identified more than 240 putative effectors. The presence of conserved domains in these effectors suggested a range of functions, including peptidoglycan hydrolases, lipases, nucleases, and nucleic acid deaminases. However, 10 of the effector groups had no functionally characterized domains. Phylogenetic analysis of these putative effectors revealed that they clustered into 32 distinct groups that appear to have been acquired from a diverse set of ancestors. Corresponding immunity proteins were identified for all but two of the effector groups. Effectors from eight of the 32 groups contained N-terminal rearrangement hotspot (RHS) domains. The C-terminal regions of these RHS proteins, which are predicted to confer the toxic effector function, were very diverse, but the N-terminal RHS domains clustered into just two groups. While the majority of *A. baumannii* strains contained an RHS type effector, no strains encoded two RHS effectors with similar N-terminal sequences, suggesting that the presence of similar N-terminal RHS domains leads to competitive exclusion. Together, these analyses define the extreme diversity of T6SS effectors within *A. baumannii* and, as many have unknown functions, future detailed characterization of these effectors may lead to the identification of proteins with novel antibacterial properties.

## Introduction

*Acinetobacter baumannii*, is a rapidly emerging, multi-drug resistant, nosocomial pathogen with no clearly defined environmental niche. Many *A. baumannii* strains produce a type VI secretion system (T6SS) that is primarily used for interbacterial competition (Carruthers et al., 2013; Weber et al., 2015; Fitzsimons et al., 2018). The T6SS is a complex nanomachine that can deliver effector proteins into the extracellular environment or directly into eukaryotic or prokaryotic cells (Cianfanelli et al., 2016b). Effectors that target eukaryotic cells generally manipulate the host cell to increase the survival of the invading pathogen, while effectors that target prokaryotic cells generally kill surrounding susceptible bacteria (prey) and thus provide a competitive advantage for the bacteria that delivered the effector (predator) (Cianfanelli et al., 2016b). The ability of a T6SS-positive predator to successfully target and kill another bacterium is dependent on the specificity of the effectors delivered by the predator and/or the particular T6SS components (e.g., specific immunity proteins) produced by the prey cell (Miyata et al., 2013; Alcoforado Diniz and Coulthurst, 2015). Although most T6SS effectors identified to date act either on bacterial or mammalian cells, a small subset of effectors have activity against both cell types (Miyata et al., 2013; Jiang et al., 2014). Additionally, two effectors have recently been identified that are toxic to *Candida albicans*, the first T6SS effectors with direct antifungal activity (Trunk et al., 2018).

The proteins required to assemble the T6SS differ slightly between bacterial species, but usually a core set of 13 proteins is required to form the secretion apparatus, which comprises a baseplate/membrane spanning complex, a contractile sheath, and an injectable inner tube (Zoued et al., 2014). Delivery of effector proteins involves attachment of the effectors onto the tip of the tube that is propelled outside of the cell before disassembly and recycling. This injectable inner tube is comprised of three proteins; the hemolysin co-regulated protein (Hcp), which forms the main tube, the proline-alanine-alanine-arginine repeat (PAAR) protein that helps form the penetrating T6SS tip, and a trimer of valine-glycine-arginine G (VgrG) proteins at the tip (Cianfanelli et al., 2016a).

The T6SS effectors are delivered either as translational fusions with the Hcp, VgrG or PAAR T6SS tip proteins (evolved effectors) (Suarez et al., 2010; Brooks et al., 2013; Schwarz et al., 2014; Cianfanelli et al., 2016b) or as cargo that is non-covalently bound to the inner tube proteins (Hcp, VgrG, or PAAR proteins; cargo effectors) (Durand et al., 2014; Alcoforado Diniz and Coulthurst, 2015; Bondage et al., 2016; Burkinshaw et al., 2018; Fitzsimons et al., 2018). Additional chaperone proteins that aid in the delivery of cargo effectors have also been identified (Liang et al., 2015; Whitney et al., 2015; Bondage et al., 2016; Zepeda-Rivera et al., 2017). The T6SS effectors characterized to date have diverse modes of action including degradation of bacterial cell wall components, lipase, and nuclease activity, activation of the PI3K/Akt signaling pathway, and inhibition of the NLRP3 inflammasome (Hood et al., 2010; Dong et al., 2013; Russell et al., 2013; Jiang et al., 2014; Chen et al., 2017; Fitzsimons et al., 2018). Most VgrG-type evolved effectors have activity against mammalian cells (Pukatzki et al., 2007; Ma et al., 2009; Suarez et al., 2010; Sana et al., 2015), while most PAAR-type evolved effectors also encode an RHS domain and display antibacterial activity (Koskiniemi et al., 2013; Alcoforado Diniz and Coulthurst, 2015). The majority of cargo effectors have antibacterial activity, although the precise mechanism of killing for many of these remains to be determined (Russell et al., 2014).

T6SS-positive bacteria that deliver broadly acting antibacterial effectors must protect themselves from self- and sibling-intoxication. Some antibacterial effectors have a rearrangement hot spot (RHS) N-terminal domain that is predicted to fold around and encapsulate the toxic C-terminal region of the protein (Busby et al., 2013), protecting the host cell from self-, but not sibling-, intoxication. The toxic C-terminal region of these RHS proteins is proceeded by a well conserved cleavage motif, DPxG-(18)-DPxGx, where auto proteolysis acts to release the C-terminal toxic portion (Jackson et al., 2009; Poole et al., 2011; Busby et al., 2013). To protect themselves from both self- and sibling-intoxication, cells also usually express specific immunity proteins that directly neutralize the action of the cognate antibacterial effector (Hood et al., 2010; Russell et al., 2011; Dong et al., 2013; Bernal et al., 2017; Fitzsimons et al., 2018). T6SS immunity proteins are typically small and are encoded immediately downstream of the gene encoding the cognate effector. Immunity proteins have been shown to be essential for the survival of many T6SS-producing bacterial species, highlighting their importance for protection against self-intoxication (Dong et al., 2013; Fu et al., 2013).

The majority of T6SS structural components are highly conserved between species. However, the T6SS produced by *A. baumannii* has diverged slightly from those in other species as there is no known *A. baumannii* homolog of TssJ, a membrane spanning protein crucial for T6SS function in *E. coli* (Aschtgen et al., 2008; Carruthers et al., 2013). Additionally, *A. baumannii* encodes a peptidoglycan hydrolase, TagX, within the main T6SS locus, which is essential for the normal function of the apparatus (Weber et al., 2016). The majority of recent studies on the T6SS have focused either on structural aspects of the T6SS apparatus or on specific effector proteins and their delivery (Unterweger et al., 2015; Kirchberger et al., 2017; Nguyen et al., 2018). However, to our knowledge there have been no studies focused on characterizing the full functional and phylogenetic diversity of toxic effector and immunity pairs delivered by different members of a single bacterial species nor on their distribution within a species.

In this study, we utilize the information from previous reports, showing that currently known *A. baumannii* T6SS effector/immunity pairs are encoded in loci together with a VgrG tip protein (Weber et al., 2013, 2016; Fitzsimons et al., 2018), to identify *A. baumannii* T6SS effector and immunity genes in 97 (96 complete and one draft) *A. baumannii* genomes. Detailed bioinformatic analysis of the gene products indicated that there are 32 different cargo effector families with a diverse range of predicted functions and almost all of these effector families are phylogenetically distinct from each other, as well as from previously characterized effectors in other bacterial species. We identify likely immunity proteins for all but two of the effector groups and use the predicted sub-cellular location of these immunity proteins to predict the cellular compartment where the cognate effector will act. Finally, we also analyze the type and distribution of VgrG, Hcp and putative PAAR tip proteins within the species and search for possible evolved effectors.

## Materials and Methods

### Identification of T6SS Effector Loci in *A. baumannii* Genomes

**FIGURE 1:**
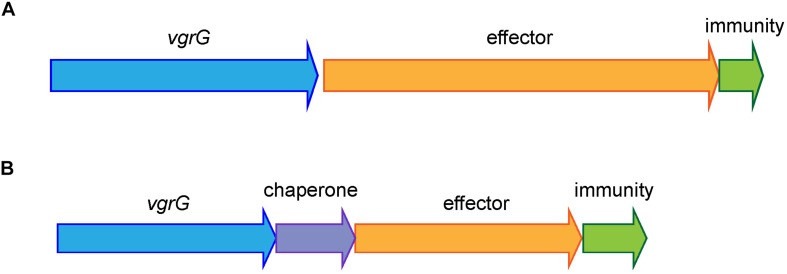
Shematic representation of the common organization of *A. baumannii* T6SS effector loci. The most common arrangement is a three-gene locus comprising a VgrG-encoding gene, followed by a gene encoding the toxic effector and a gene encoding the cognate immunity protein **(A)** and the less common arrangement is a four-gene locus comprising a VgrG-encoding gene, followed by a gene encoding a chaperone then a gene encoding a toxic effector and a gene encoding a cognate immunity protein **(B)**.

All currently identified *A. baumannii* T6SS effectors are encoded in either a three-gene locus, together with a gene encoding a VgrG tip protein and a gene encoding an immunity protein, or in a four-gene locus, with the same two genes encoding VgrG and immunity proteins plus a gene encoding a chaperone protein (Figure 1). Consequently, to identify effector loci, we searched for genes encoding the well conserved VgrG proteins in complete, publicly available *A. baumannii* genomes on the Pathosystems Resource Integration Center (PATRIC) database (96 as of June 2018, Supplementary File S1) as well as one incomplete genome sequence, representing the widely studied *A. baumannii* strain, ATCC 19606. Proteins containing the VgrG-domain were identified using the Protein Family Sorter service tool, with a PATRIC cross-genus families (PGfam) filter (Wattam et al., 2017). TssM proteins and proteins containing a Hcp-domain or a PAAR-domain were identified in a similar manner using PATRIC.

To predict the functions of identified effectors, conserved domains were identified using the NCBI protein Basic Local Alignment Search Tool (BLASTp) or PSI-BLAST, using default parameters against the non-redundant protein sequences database (nr) of the National Center for Biotechnology Information (NCBI) (Altschul et al., 1997, 2005). Sequences representing putative effectors were searched (RStudio, version 3.4.1) for the presence of the lipase motifs, GxSxG (Derewenda and Derewenda, 1991) and HxKxxxxD (Sung et al., 1997); effectors were predicted to function as lipases if they contained one of the above lipase motifs and shared similarity to lipase proteins within four PSI-BLAST iterations using default parameters (excluding the *A. baumannii* taxid 470) (Altschul et al., 1997).

### Phylogenetic Analysis of Protein Sequences

Multiple sequence alignments of protein sequences were generated for each phylogenetic tree using the MUltiple Sequence Comparison by Log-Expectation (MUSCLE, v3.8) algorithm (Edgar, 2004), followed by manual correction using Jalview (v2.10) (Waterhouse et al., 2009). Maximum likelihood (ML) phylogenetic trees were estimated with IQ-TREE (Nguyen et al., 2015), using the best-fit amino acid substitution model (Kalyaanamoorthy et al., 2017). For effector sequences containing an RHS-domain, only the predicted cytotoxic C-terminal domain, following the final D(P/S)xGx cleavage sequence, was included in the alignments (Jackson et al., 2009). Conversely, only the sequence preceding the final D(P/S)xGx of RHS-domain encoding effectors was included in the alignments for the RHS pre-cleavage analysis. Branch distances were determined using the ultrafast bootstrap (UFBoot) method (Hoang et al., 2018). Trees were visualized using the FigTree (v1.4) program^1^.

### Phylogenetic Analysis of Whole Genome Sequences

The *A. baumannii* genome sequences were used to generate a phylogenetic tree using the Harvest package (v1.1.2) (Treangen et al., 2014). Core genome SNPs were aligned using Parsnp and the tree was visualized using Figtree.

### Grouping of T6SS Effectors

Effectors were grouped based on their location on the phylogenetic tree, amino acid sequence similarity, and shared conserved domains. Alignments of effectors were generated using Clustal Omega (Sievers et al., 2011). The level of amino acid identity and coverage between proteins representing different effector groups was determined using the NCBI BLASTp multiple alignment function with default parameters, or the multiple alignment function of Geneious Prime (version 2019.2.1)^2^. Each of the *A. baumannii* effector proteins was given a numeric identifier (1–32) relating to the groupings displayed in the effector phylogenetic tree (Figure 2). The same effector group number was also used for the cognate immunity, VgrG and chaperone (if present) proteins encoded in the same locus as the specific effector gene. A group number of 0 was assigned to loci that encoded a VgrG protein but no effector. A full list of all the protein sequences used in the various comparisons is given in Supplementary File S2, together with the parent strain names, gene locus tags and effector group numbers.

**FIGURE 2:** Maximum likelihood (ML) phylogenetic trees showing T6SS effector groups generated using only *A. baumannii* effector sequences **(A)** and groups generated when sequences representing characterized T6SS effectors from other species were additionally included in the analysis **(B)**. Trees were generated from amino acid sequences using the Probability Matrix from Blocks (revised BLOSUM matrix) with the discrete Gamma model (PMB + F + G4). The scale bars represent the number of amino acid substitutions. All branch support values ≥70 (UFBoot) are shown at major nodes. Putative function and/or targets of effectors are denoted by the different colors. All tips are visible on the *A. baumannii* T6SS effector tree **(A)**. Branches on tree B that contained more than 2 effectors were condensed and are represented by 1–3 tips (annotated with ^∗^). Black names represent *A. baumannii* effectors, blue underlined names represent characterized effectors from other bacterial species **(B)**. See Supplementary Files S3a,b for raw phylogenetic tree data, and Supplementary Files S4a,b for corrected alignments. See Table 1 for details of the characterized effectors.

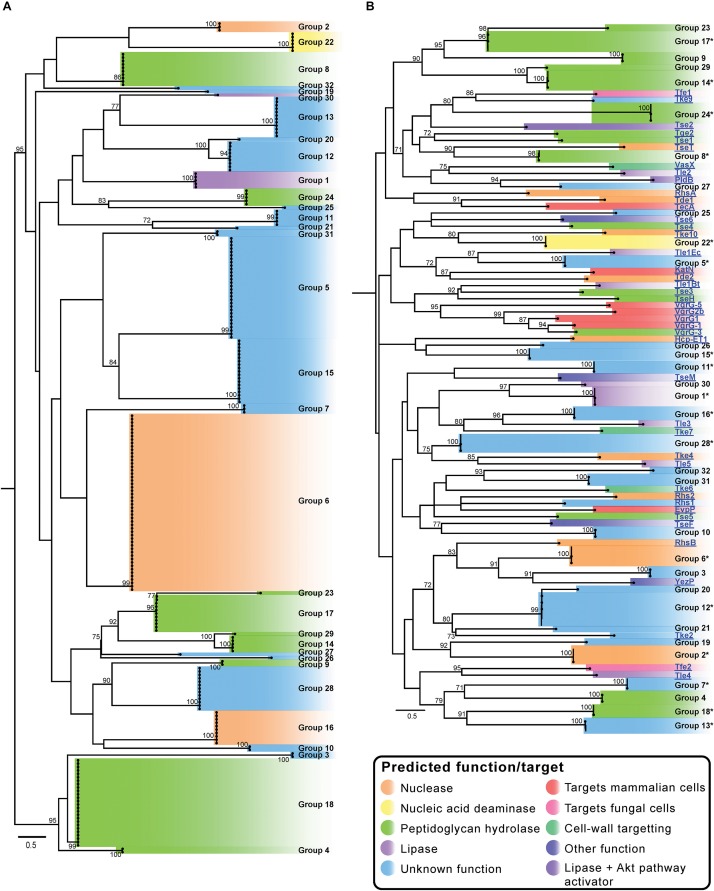



**TABLE 1:** Putative *A. baumannii* T6SS effector protein groups.

**Identifier**	**Previous name**	**Name^a^**	**Size (aa)**	**Domains**	**Lipase motif**	**Number of proteins^c^**	**Predicted function**
1	Tse1	Tle1^AB^	276	No conserved domains	GXSXG	5 (137)	Lipase
2	Tse2	Tde2^AB^	529	Ntox15		3 (47)	DNase
3	Tse3	Tse3^AB^	1001	No conserved domain	GXSXG	2 (30)	Unknown
4	Tse4	Tpe4^AB^	812–848H^b^	LysM, NlpD, Peptidase M23, spore safA		2 (45)	Peptidoglycan hydrolase
5		Tse5^AB^	1565	RhsA, Rhs assoc core, RHS, RHS repeat	GXSXG	30 (923)	Unknown
6		Tde6^AB^	1635	RhsA, Rhs assoc core, RHS, RHS repeat, YD repeat, Tox-GHH		52 (2138)	DNase
7		Tse7^AB^	428	DUF3396	GXSXG	3 (63)	Unknown
8		Tae8^AB^	666–698^b^	COG3179, LysM, spore safA, CHAP, PRK06347		11 (277)	Peptidoglycan amidase
9		Tce9^AB^	532	COG3179, lysozyme-like superfamily, chitinase glyco hydro 19	HXKXXXXD	2 (68)	Chitinase
10		Tse10^AB^	504	DUF2235 (alpha/beta hydrolase), COG3673	GXSXG	2 (60)	Unknown
11		Tse11^AB^	787	No conserved domains	HXKXXXXD	5 (74)	Unknown
12		Tse12^AB^	917	No conserved domains		9 (183)	Unknown
13		Tse13^AB^	364	No conserved domains		12 (161)	Unknown
14		Tpe14^AB^	746	LysM, Peptidase M15_3, PRK13914, PRK06347, Peptidase M23, NlpS, spore safA		5 (113)	Peptidoglycan hydrolase
15	Rhs1	Tse15^AB^	1590	RhsA, Rhs assoc core, RHS, RHS repeat, YD repeat, Bacuni 01323 like		19 (505)	Unknown
16	Rhs2	Tde16^AB^	1623	RhsA, Rhs assoc core, RHS, RHS repeat, AHH		10 (228)	DNase^e^
17	LysM	Tae17^AB^	582	LysM, Amidase 5, PRK06347		11 (184)	Peptidoglycan amidase
18		Tpe18^AB^	674	LysM, NlpD, Peptidase M23, PRK11649, mltD		27 (1102)	Peptidoglycan hydrolase
19		Tse19^AB^	502	No conserved domains		1 (30)	Unknown
20		Tse20^AB^	929	No conserved domains		1 (22^d^)	Unknown
21		Tse21^AB^	1613	RhsA, Rhs assoc core, RHS, RHS repeat, YD repeat, Bacuni 01323 like		1 (40)	Unknown
22		Tme22^AB^	1638	RhsA, Rhs assoc core, RHS, YD repeat, RHS repeat (x2), YwqJ-deaminase, deoxycytidylate deaminase, ComEB		6 (72)	Nucleic acid deaminase
23		Tpe23^AB^	671	LysM, NlpD, PRK11198, spore safA, XkdP		1 (8)	Peptidoglycan hydrolase
24		Tpe24^AB^	798	Peptidase M23, NlpD, PRK11649		5 (91)	Peptidoglycan hydrolase
25		Tse25^AB^	825	No conserved domains		1 (10)	Unknown
26		Tse26^AB^	1602	RhsA, Rhs assoc core, RHS, RHS repeat, YD repeat, Bacuni 01323 like		1 (12)	Unknown
27		Tse27^AB^	1608	RhsA, Rhs assoc core, RHS, RHS repeat, YD repeat, RHS repeat (x2)		1 (13)	Unknown
28		Tse28^AB^	245	No conserved domains		13 (126)	Unknown
29		Tpe29^AB^	715	LysM, NlpD, PRK06347, Hydrolase 2, Peptidase M23, spore safA		1 (121)	Peptidoglycan hydrolase
30		Tle30^AB^	270	Lipase	GXSXG	1 (44)	Lipase
31		Tse31^AB^	450	No conserved domains		2 (38)	Unknown
32		Tse32^AB^	481	No conserved domains		1 (42)	Unknown

*^*a*^Effector name derivations are: Tle, T6SS lipase effector; Tde, T6SS DNase effector; Tse, Type VI secretion system exported; Tpe, T6SS peptidoglycan hydrolase effector; Tae, T6SS peptidoglycan amidase effector; Tce, T6SS chitinase effector; Tme, T6SS nucleic acid deaminase effector. ^*b*^Length varies based on repeat sequence. ^*c*^First number indicates the number of effectors identified in the 97 *A. baumannii* genomes used in this study. The bracketed number indicates the number of proteins with >97% amino acid identity and >99% coverage identified in the NCBI non-redundant database via BLASTp analysis. ^*d*^>95% amino acid identity, >99% coverage via BLASTp. ^*e*^Function confirmed.*

### Analysis of the Cleavage Motif in RHS Effectors

A common RHS-cleavage motif for the RHS-domain-containing effectors was generated using Multiple EM for Motif Elicitation (MEME) from the MEME Suite version 5.0.5 (default parameters) (Bailey and Elkan, 1994), using the 36 amino acids preceding, and including, the common D(P/S)xGx sequence. Three motif sequences were generated; one generated using all available *A. baumannii* RHS sequences identified in this study, one generated using each of the different groups of non-*A. baumannii* RHS effectors shown in Supplementary Table S1, and one generated using single representatives of each of the *A. baumannii* RHS effector groups, and the non-*A. baumannii* RHS effectors.

### Analysis of the Genomic Position and Genetic Context of T6SS Effector Loci

*A. baumannii* whole genomes were aligned and the regions flanking the effector loci were identified and analyzed using Progressive Mauve (Version 1.1.1) (Darling et al., 2010). The analysis included the genomes from *A. baumannii* strains AB307-0294, ATCC 19606, ACICU, R2091, AR_0088, AR_0078, 15A34, 15A5, AF-401, ATCC 17978, ZW85-1, Ab04, 6200, and AB031 as collectively these strains encode all groups of effector loci identified in this study.

### Identification of Signal Peptides and Prediction of Protein Sub-Cellular Location

Signal peptides were identified using the SignalP 4.1 server using default parameters for Gram-negative bacteria (Nielsen, 2017). To predict putative localization of the immunity proteins, the sequences were analyzed using PSORTb (v3.0.2) using the settings for Gram-negative bacteria (Yu et al., 2010). Predicted transmembrane domains were identified in protein sequences using TMHMM (Moller et al., 2001).

## Results and Discussion

### Identification of Genes Encoding Putative VgrG, Chaperone, Effector, and Immunity Proteins in 97 *A. baumannii* Genomes

To comprehensively identify the array of *A. baumannii* T6SS effector and immunity pairs, we analyzed 97 genomes, comprising 96 complete genomes and one draft genome (representing strain ATCC 19606). Firstly, the presence/genomic position of the locus encoding the T6SS structural proteins was identified by searching for the conserved TssM and Hcp domains using the Protein Family Sorter service tool in PATRIC. The integrity of the locus was then assessed by visual inspection and BLAST analysis (Supplementary File S1). BLAST analysis identified TssM and Hcp homologs in 65 of the 97 strains; no strains contained more than a single T6SS structural locus. Further analyses revealed that 11 of the identified T6SS loci contained a truncated or non-functional *tssM* and/or *hcp* gene, both of which are essential for T6SS function. Thus, we predict that 54 (58%) of the *A. baumannii* strains analyzed contain an intact T6SS locus, although it is possible that a small number of these strains are unable to produce functional T6SS structures due to missense mutations in genes encoding critical T6SS proteins. It is also possible that, despite an intact locus, some strains do not produce a functional T6SS due to the presence of a T6SS suppression plasmid (Weber et al., 2015). As a large number of strains lack a functional T6SS, this suggests that under some conditions there may be a fitness burden associated with T6SS activity. This supports previous work that has shown that T6SS activity is suppressed in the presence of large conjugative multidrug resistance plasmids (Weber et al., 2015). However, under *in vitro* growth conditions an AB307-0294 *tssM* mutant grew indistinguishably to the wild-type strain (Fitzsimons et al., 2018).

In all previously identified *A. baumannii* T6SS systems, each effector is encoded in a three- or four-gene locus, downstream of a gene encoding a VgrG tip protein and upstream of a gene encoding the cognate immunity protein; the locus may also contain a gene encoding a chaperone protein (Figure 1) (Weber et al., 2013, 2016; Fitzsimons et al., 2018). Therefore, to identify effectors we first identified the genes encoding proteins containing the highly conserved VgrG domain using the PATRIC Protein Family Sorter, filtering by the PATRIC cross-genus families (PGfam) (Wattam et al., 2017). In total, 272 *vgrG* genes were identified in 95 of the 97 *A. baumannii* genomes (Supplementary File S2). The majority of the strains contained three separately encoded *vgrG* genes, with a maximum of five *vgrG* genes (in *A. baumannii* strain AB031). Two strains, B8300 and ABNIH28, lacked the entire T6SS locus and any genes encoding VgrG-domain containing proteins (Supplementary File S1). In addition to the VgrG domain, all identified *vgrG* genes encoded proteins containing a DUF2345 domain (COG4253), previously reported to be present in all three VgrG proteins produced by *A. baumannii* strain AB307-0294 (Fitzsimons et al., 2018) and commonly found in VgrG proteins produced by other bacterial species (De Maayer et al., 2011). Based on gene length and detailed BLAST analysis, we predict that 25 of the 272 identified *vgrG* genes encode truncated and likely non-functional proteins; a number of these truncated genes showed clear evidence of gene rearrangement including loss of the downstream cognate effector gene (Supplementary File S2).

To identify putative effector, chaperone, and immunity genes, the sequence adjacent to each *vgrG* gene was visually inspected and analyzed using a range of bioinformatic tools. Fifteen strains encoded at least one orphan *vgrG* gene; however, twelve of these were predicted to encode non-functional VgrG proteins as the genes were short and had no discernible effector or immunity genes downstream. Inspection of the region adjacent to the remaining *vgrG* genes allowed for the identification of 244 putative effector genes that were predicted to be intact, based on the alignment and similarity to other bacterial proteins using BLAST analysis; a further 39 effector genes were identified but were excluded from further analysis as they were likely to encode truncated/non-functional proteins. The length of the predicted functional effector proteins varied from 245 amino acids to 1638 amino acids. All effector proteins larger than 1100 amino acids encoded an RHS domain; this domain has commonly been identified in other T6SS effectors (Supplementary Table S1). Our analyses indicate that the 54 *A. baumannii* strains with an intact T6SS locus typically encode two or three effectors with some strains encoding four functional effectors.

In total, 225 putative immunity genes were identified downstream of the putative effector genes. All immunity proteins were shorter than their cognate effector proteins, a pattern that is widely observed across many T6SSs (Hood et al., 2010; Russell et al., 2011; English et al., 2012; Bernal et al., 2017; Liu et al., 2017). A total of thirty-seven loci with apparently intact *vgrG* and effector genes contained no identifiable third gene encoding a predicted cognate immunity protein. However, twenty-nine of these were in strains predicted to lack a functional T6SS indicating that the genes in these *vgrG* loci may be inactive. The remaining eight immunity-gene deficient *vgrG* loci may be either functionally inactive or encode effectors that do not have antibacterial/self/sibling activity.

A fourth gene encoding a putative chaperone protein, ranging in size from 120 to 332 amino acids, was identified immediately following the *vgrG* in 28 of the 244 *vgrG* loci that were predicted to encode functional effectors. Many of the predicted chaperone proteins contained a DUF4123 domain, previously identified in T6SS chaperone proteins produced by other bacterial species (Liang et al., 2015; Unterweger et al., 2015; Steele et al., 2017).

### More Than 30 Distinct T6SS Effector Families Are Encoded by *A. baumannii* Strains

In order to determine the diversity of the identified *A. baumannii* T6SS effectors, the amino acid sequences of all predicted intact effectors were aligned using MUSCLE and a Maximum Likelihood (ML) phylogenetic tree was constructed (Figure 2A) (Edgar, 2004). The effectors separated into 32 clearly discernible groups (Groups 1–32) and each effector locus was assigned a numeric identifier correlating with the appropriate group (a full list of strain names, locus tags and effector group numbers is given in Supplementary File S2). The bootstrap values for the clustering of different effectors within each of the 32 groups was very high (>95%), indicating that closely related effectors are found across many strains. However, the bootstrap values in the deep branches between groups were usually less than 70%, indicating that effector proteins in each of the different groups were highly diverse and polyphyletic in origin (Figure 2A and Supplementary Files S3a,b) making it impossible to infer unambiguous phylogenetic relationships between many of the different groups. There were some exceptions to this; groups 9 and 28, groups 14, 17 and 29, groups 5 and 15, groups 24 and 25, and groups 12 and 20 forming separate but related clusters with high bootstrap support (>80%). However, overall these data strongly suggest that the effector families identified in *A. baumannii* have originated from diverse ancestors, and that the different effectors found within any single strain have also likely arisen from different ancestral sources. Furthermore, we observed very little amino acid similarity between the groups (Supplementary File S5), with only five pairs of effectors sharing more than 19% amino acids identity between them (in comparison to the length of the shortest sequence), confirming that each group represents a unique effector.

An additional ML phylogenetic tree was constructed that included the amino acid sequences of all the predicted *A. baumannii* effectors as well as all functionally characterized T6SS effectors from other species (Supplementary Table S1 and Figure 2B). The previously characterized effectors from other species included those that target eukaryotic cells and those that target prokaryotic cells and these effectors represented both cargo and evolved effectors (effector domains fused to Hcp, VgrG, or PAAR proteins). The effectors clustered into 75 distinct tips in the tree (Figure 2B), representing 32 distinct *A. baumannii* effector groups and 43 groups containing the effectors from other species. These data indicate that *A. baumannii* effectors are phylogenetically distinct from the effectors produced by other bacterial species.

In order to predict putative functions and/or targets for each of the 32 *A. baumannii* effector families, conserved domains in each effector group were identified using BLASTp and/or PSI-Blast (Table 1) (Altschul et al., 2005). Additionally, the effector amino acid sequences were also examined specifically for the presence of the known lipase motifs GxSxG and HxKxxxxD (Derewenda and Derewenda, 1991; Sung et al., 1997). Putative functions were assigned to 15 of the 32 effector families; including lipases (families 1 and 30), amidases (family 8 and 17), chitinases (family 9), general peptidoglycan hydrolases (families 4, 14, 18, 23, 24, 29), nucleases (families 2, 6, 16), and a nucleic acid deaminase (family 22). None of the *A. baumannii* effectors contained domains previously associated with activity against mammalian cells. This finding is consistent with several previous studies on *A. baumannii* that found that all T6SS effectors examined had antibacterial activity (Carruthers et al., 2013; Weber et al., 2013, 2016; Fitzsimons et al., 2018). Twenty of the effector families had five or less members identified in the 97 analyzed genomes and ten families had just a single member. To determine if these effector families were more widely conserved, we used BLASTp to compare each of the effectors against the NCBI non-redundant database, which includes more than 3000 draft *A. baumannii* genomes. Almost all (19 of 20) of these effector families were highly conserved (>97% amino acid similarity, >99% coverage) in at least 10 other strains, with only Tpe23^AB^ conserved in less than 10 strains (8 strains, >95% amino acid similarity, >99% coverage) (Table 1).

The *A. baumannii* effector groups 1, 2, 3, and 4 (Table 1) include four previously identified *A. baumannii* ATCC 17978 effectors; group 1 includes the predicted lipase Tse1; group 2 includes the DNase Tse2; group 3 includes the antibacterial effector Tse3 with unknown function; and group 4 includes the peptidoglycan hydrolase Tse4 (Weber et al., 2016). Additionally, effector groups 15, 16, and 17 (Table 1) include three previously identified *A. baumannii* AB307-0294 effectors; group 15 includes the antibacterial effector of unknown function Rhs1; group 16 includes the DNase Rhs2; and group 17 includes the peptidoglycan hydrolase LysM (Fitzsimons et al., 2018). In this study, we propose the naming of all effectors using a Txe nomenclature, where the “x” indicates predicted function (or the use of an “s” if function is unknown), which has been used widely in the T6SS field (Hood et al., 2010; Shyntum et al., 2014). Thus, Tse1, becomes Tle1^AB^ for T6SS lipase effector group 1, Tse2 becomes Tde2^AB^ for T6SS DNase effector group 2, Tse3 remains Tse3^AB^, Tse4 becomes Tpe4^AB^ for T6SS peptidoglycan hydrolase effector group 4, Rhs1 becomes Tse15^AB^ for T6SS exported, Rhs2 becomes Tde16^AB^ for T6SS DNase effector group 16 and LysM becomes Tae17^AB^ for T6SS amidase effector group 17. For all effector groups with no clear function, we have followed the previously established naming convention of Type VI secretion system exported [group number] (e.g., Group 3 effectors are named Tse3^AB^). Furthermore, the proposed names include a superscript AB for *A. baumannii* to distinguish them from effectors of the same name/type identified in other species (Table 1). We also propose altering the names of the cognate VgrG, chaperone, and immunity proteins according to the naming of the effectors, with VgrG proteins identified with a number corresponding to the cognate effector, chaperone proteins identified as T6SS accessory proteins (Tap) with a number corresponding to the cognate effector and immunity proteins becoming T6SS immunity with an appropriate middle letter determined by the cognate effector function (e.g., VgrG2 and Rhs2I from *A. baumannii* AB307-0294 become VgrG16 and Tdi16^AB^, respectively).

Effector families with related predicted functions were not always observed to cluster closely on the phylogenetic tree, strongly suggesting convergent evolution of effector functions (Figure 2). For example, proteins in groups 4, 8, 14, 17, 18, 23, 24 and 29 all have predicted peptidoglycan hydrolase activity (light green highlighting; Figure 2), but despite sharing similar functions and targets, they separated into three different areas of the phylogenetic tree (Figure 2). Groups 14, 17, 23, and 29 were found to cluster closely (Figures 2A,B) and given the high bootstrap values, are likely to have evolved from a common recent ancestor. Similarly, groups 4 and 18 are also likely phylogenetically related. Other peptidoglycan hydrolase families that were more distal to these groups are likely to have evolved separately. Two large effector groups (6 and 16) both contain proteins with predicted nuclease activity, including the group 16 effector, Tde16^AB^ (formerly Rhs2) that has been confirmed to function as a DNase (Fitzsimons et al., 2018). However, bioinformatic analysis indicates that effector groups 6 and 16 are phylogenetically unrelated; although both groups contain proteins with similar helix-turn-helix (HTH)/EndoVII nucleic acid binding domains (Zhang et al., 2012), the proteins within group 6 contain a GHH active site whereas group 16 proteins contain an AHH active site. These data support the proposition that the two groups of nuclease effectors have evolved independently and were acquired from different ancestral sources.

**FIGURE 3:**
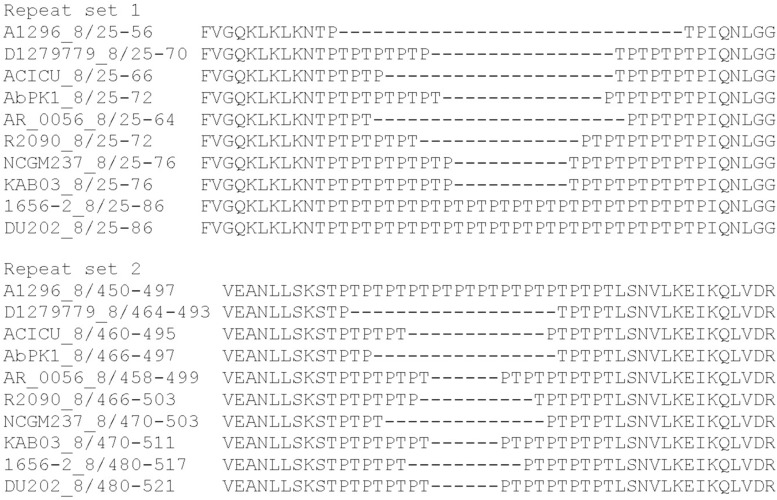
MUSCLE alignment of amino acid repeat sequences present in the *A. baumannii* group 8 effectors encoded by *A. baumannii* strains A1296, D1279779, ACICU, AbPK1, AR_0056, R2090, NCGM, KAB03, 1656-2, and DU202.

Effectors belonging to the groups 4 and 8 have predicted peptidoglycan hydrolase activity and encode uncharacterized repeat regions. The group 4 effectors contain 12 or 24 copies of an alanine-serine-glycine (ASG) repeat. We also identified similar ASG repeats in a number of proteins from other bacterial species, including proteins within the type III secretion system export apparatus, as well as other membrane-associated proteins. The proteins representing group 8 effectors each contain two distinct regions of threonine-proline (TP) repeats; the number of repeats in the first region (set 1) varies from two to 17 copies. The second region (set 2) contains between three and 12 copies (Figure 3). No two effectors in group 8 shared the same number of repeats at both locations. The functions of the ASG and TP repeat sequences found in these proteins are currently unknown.

### T6SS Effectors Are Largely Conserved Between Genetically Related Strains

**FIGURE 4:**
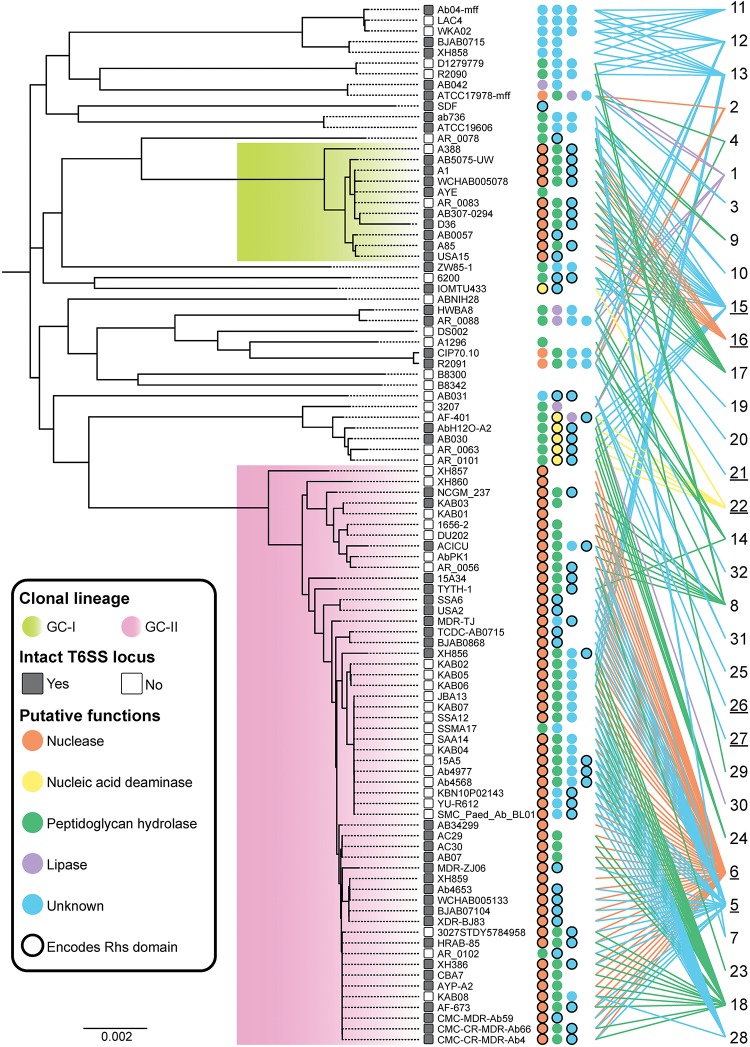
Effector conservation in *A. baumannii*. Distribution of T6SS effectors across the *A. baumannii* strains examined in this study. The ML phylogenetic tree (left) was generated using whole genome sequences representing 97 strains. The scale bar represents the genetic distance between strains as measured by the number of single nucleotide polymorphisms (SNPs). Strains enclosed by the green box include global clone I (GC-I) strains, and strains enclosed by the pink box include global clone II (GC-II) strains. The number of predicted effectors encoded by each strain is indicated by the number of colored circles adjacent to the strain name. The color of each circle represents the predicted function of each effector as follows; orange, nuclease; yellow, nucleic acid deaminase; green, peptidoglycan hydrolase; purple, lipase; blue, unknown function. A black border around a circle indicates that an RHS domain is present within the N-terminal region of the effector. The designated number for each effector group is shown at the far right with an underscore indicating those effector groups that have an N-terminal RHS domain. Colored lines are provided to link each effector group to the strains where a representative is found.

In order to determine whether related *A. baumannii* strains expressed similar groups of effectors, an ML phylogenetic tree of all strains included in these analyses was generated from whole genome core single nucleotide polymorphisms (cgSNPs) (Figure 4). The distribution of effectors across the cgSNP phylogenetic tree was then assessed. The presence or absence of an intact T6SS locus was also noted in order to determine if there was a correlation between specific effector types and loss of T6SS function. Fifty-four strains formed a tight cluster that included known global clonal lineage II (GC-II) strains, eleven formed a cluster including known GC-I strains and 33 strains did not cluster with strains from either global clone lineage (Zarrilli et al., 2013; Hamidian et al., 2019). Particular effector groups were largely found in closely related strains and no effector groups were shared across both GC-I and GC-II lineages. However, many of the effector groups present in strains that did not belong to either GC-lineage were also present in GC-I or GC-II strains.

The effector groups 5, 6, 18, 23 and 28 were found solely in the GC-II lineage, while only effector group 16 was confined to the GC-I lineage. Proteins representing the group 6 nuclease effectors were present in 52 of the 54 genomes representing GC-II strains but were not present in any strains outside that lineage. The majority of the GC-II strains (64%) also encoded predicted peptidoglycan hydrolases belonging to only one of four different peptidoglycan hydrolase groups. A similar pattern was observed in strains from the GC-I lineage, where 10 of the 11 strains (91%) contained the known DNase effector Tde16^AB^, and 9 of the 11 strains (82%) contained the predicted peptidoglycan hydrolase effector Tae17^AB^ (previously LysM from AB307-0294). Thus, the presence of a single T6SS nuclease effector and single peptidoglycan hydrolase effector appears common (observed in 43% of strains). Effectors of unknown function were identified in 76 of the strains (78%) and 23 strains contained more than one effector of unknown function. Thus, *A. baumannii* strains contain a high diversity of T6SS effectors.

### RHS Effectors Have Similar Pre-cleavage Regions but Diverse C-Terminal Toxic Regions

**FIGURE 5:**
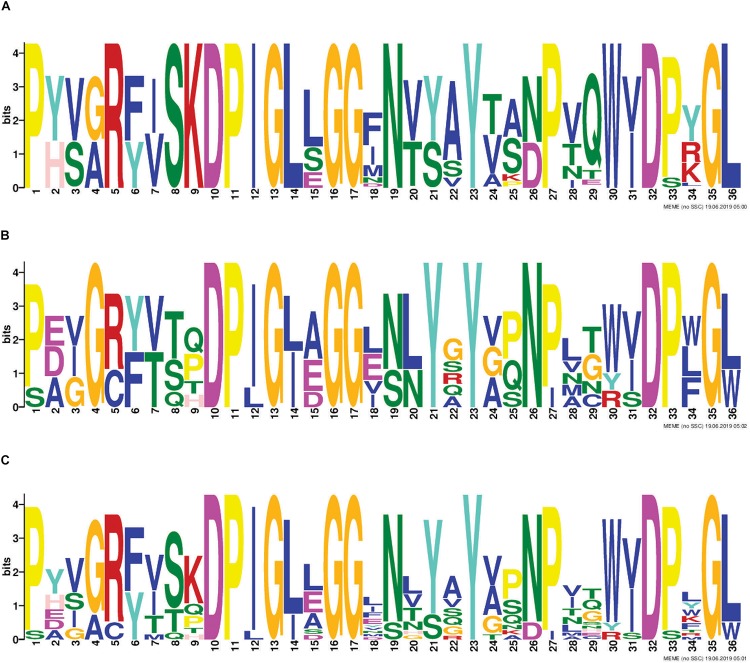
Identification of conserved amino acids surrounding the putative RHS cleavage motif. The 36 amino acids immediately upstream of where the C-terminal toxic domain is predicted to be cleaved from the N-terminal RHS domain of all RHS effectors included in this study, were used for identification of conserved residues using the Multiple EM for Motif Elicitation (MEME) (Bailey and Elkan, 1994). Conserved region in all T6SS-associated *A. baumannii* RHS proteins identified from analysis of; all RHS proteins in the 97 *A. baumannii* genomes **(A)**, representative non-*A. baumannii* RHS proteins **(B)** and in both *A. baumannii* and non-*A. baumannii* RHS proteins **(C)**. The amino acid position is shown on the *X*-axis, and the bit-score showing probability of each of the amino acids at each position is shown on the *Y*-axis.

RHS proteins are a well-recognized class of T6SS effectors. T6SS-associated RHS proteins have a conserved architecture consisting of an N-terminal RHS domain, followed by a DPxG-(18)-D(P/S)xGx cleavage motif, followed by a C-terminal region that is cleaved from the protein and predicted to be solely responsible for effector function (Jackson et al., 2009; Poole et al., 2011; Busby et al., 2013). We identified eight different RHS-type effector families across all *A. baumannii* strains examined (Table 1) and 73% of strains contained at least one RHS family effector (Figure 4). All *A. baumannii* RHS effectors contained a highly conserved predicted cleavage motif (Figure 5) that closely matched the previously identified DPxG-(18)-DPxGx motif. Indeed, the region flanking the known cleavage motif was also highly conserved, and we identified that the sequence DPIGLxGGxNxxxYxxxPxxWxDxxGL was present in every *A. baumannii* RHS effector. Comparison with known RHS effectors from other species identified that the RHS effector cleavage motif could be extended to the sequence DP(I/L)GxxGGxxxxxYxxxxxxxxD(P/S)xG(L/W), with the three partially conserved positions (I/L, P/S and L/W) being most commonly I, P and L, respectively.

**FIGURE 6:**
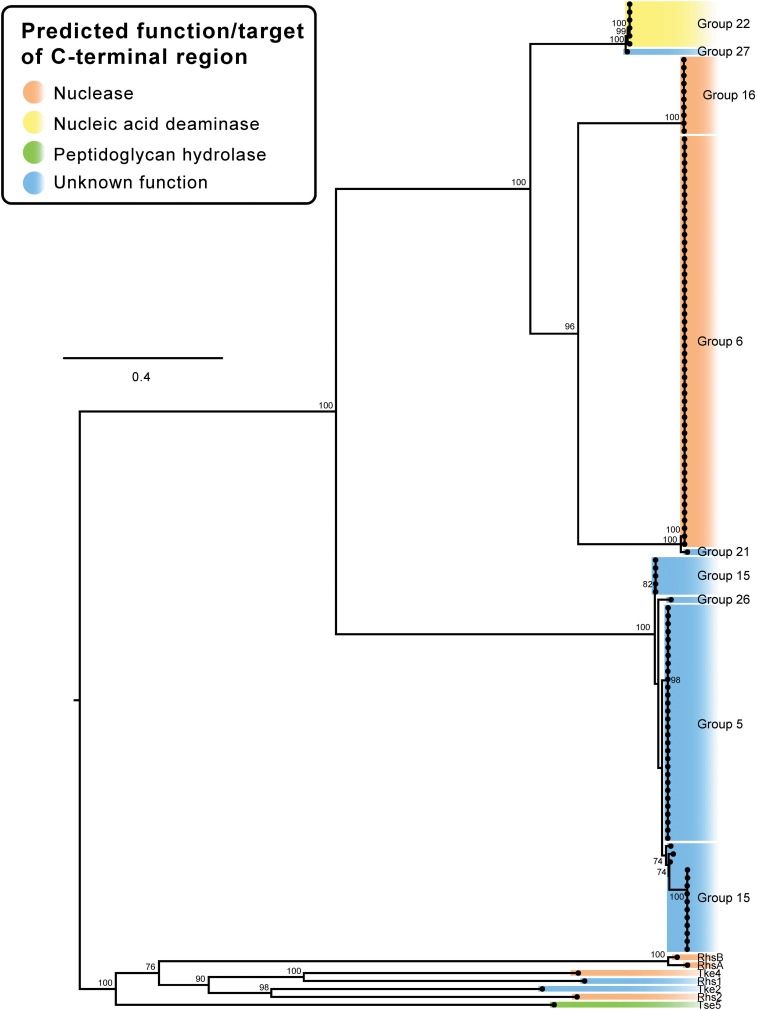
Pre-cleavage region of RHS effectors. ML phylogenetic tree showing the relationship between each of the RHS domain regions from all RHS-family *A. baumannii* effectors. Comparison excludes the C-terminal region encoding the effector (i.e., only the pre-cleavage amino acid sequences were used). Branches have been named according to the corresponding C-terminal effector group. Tree was generated from amino acid sequences using the general ‘variable time’ matrix with discrete Gamma model (VT + F + I + G4). The scale bar represents the number of amino acid substitutions. Branch support values ≥70 (UFBoot) are shown at all major nodes (Supplementary Files S3c, S4c).

To investigate if the RHS-type protein N-terminal regions, containing the RHS domain, and C-terminal regions, encoding the effector domain, had co-evolved, we constructed a ML phylogenetic tree using only the N-terminal region sequence (containing the RHS domain) up to, and including, the final D(P/S)xGx cleavage site. The same regions from seven characterized T6SS RHS proteins from other species were also included. The *A. baumannii* RHS pre-cleavage sequences clustered into four distinct clades that were all very distantly related to the N-terminal regions of the RHS proteins from other species (Figure 6). Clade 1 comprised RHS proteins with C-terminal effectors from groups 22 and 27, clade 2 comprised RHS proteins with C-terminal effectors from group 16, clade 3 contained RHS proteins with C-terminal effectors from groups 6 and 21, and clade 4 contained RHS proteins with C-terminal effectors from group 5, 15, and 26 (Figure 6). While the RHS proteins in clade 2 were all associated with similar C-terminal effectors (all group 16), RHS proteins with N-terminal regions grouped in clades 1, 3 and 4 were associated with a diverse set of effector types. For example, in clade 4, highly similar N-terminal pre-cleavage regions were followed by C-terminal regions representing three divergent effector groups, 5, 15, and 26 (Figures 2, 6). Similarly, clade 1 proteins all shared a highly similar N-terminal region, but the C-terminal regions included effectors from groups 22 and 27 and clade 3 proteins contained C-terminal regions including effectors from groups 6 and 21 (Figures 2, 6). Thus, RHS proteins sharing highly conserved N-terminal regions may be associated with a diverse range of C-terminal effectors. Interestingly, examination of the *A. baumannii* strains that had two separately encoded RHS effectors revealed the pre-cleavage region in each encoded protein belonged to a different RHS clade. This suggests effector proteins with phylogenetically related N-terminal regions cannot function efficiently in the same strain, perhaps because of delivery constraints or because lack of diversity of delivered effectors leads to reduced competitive outcomes.

### T6SS Chaperone Genes Are Associated With Only a Limited Number of Effector Genes

**FIGURE 7:**
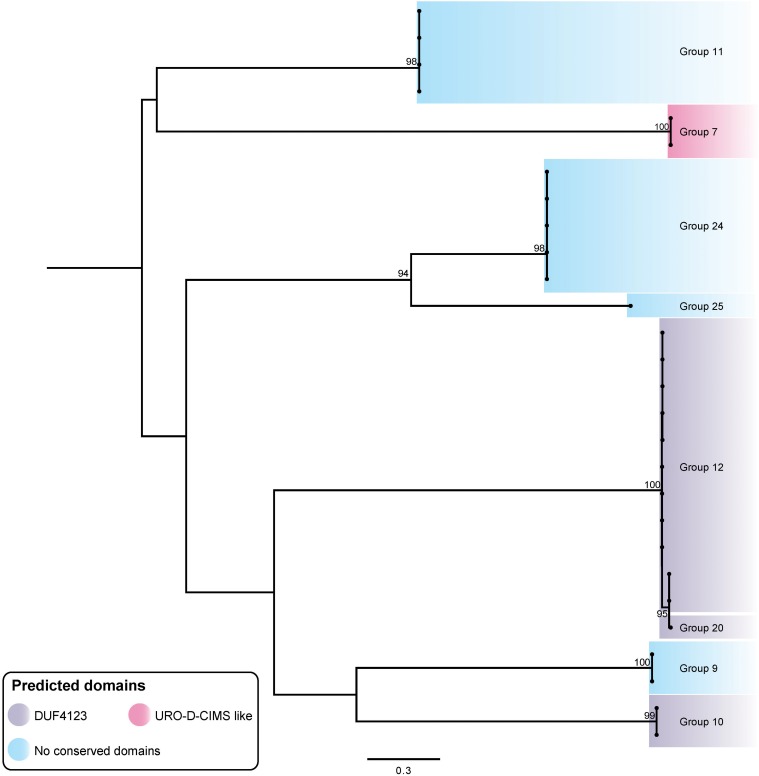
ML phylogenetic tree of *A. baumannii* T6SS putative chaperone proteins. Branches have been named according to the effector loci to which the chaperone proteins correspond. The tree was generated from amino acid sequences using the general ‘variable time’ matrix model (VT + F). The scale bar represents the number of amino acid substitutions. Branch support values ≥70 (UFBoot) are shown at all major nodes (Supplementary Files S3d, S4d).

T6SS chaperone proteins, also called adaptor proteins or effector-associated accessory proteins, have been shown in other bacteria to be crucial for the specific delivery of some evolved and cargo effectors (Miyata et al., 2013; Alcoforado Diniz and Coulthurst, 2015; Liang et al., 2015; Unterweger et al., 2015; Whitney et al., 2015; Bondage et al., 2016; Cianfanelli et al., 2016a; Zepeda-Rivera et al., 2017). T6SS chaperone proteins can directly act to stabilize the effector or can stabilize the effector-VgrG interaction, and it has been proposed that chaperone proteins with a DUF4123 domain may help regulate the amount of a specific effector within the cell (Liang et al., 2015; Unterweger et al., 2017; Zepeda-Rivera et al., 2017). Putative *A. baumannii* chaperone proteins were identified by searching immediately downstream of each *vgrG* for encoded proteins with a chaperone-associated domain (e.g., DUF4123) or by visual inspection of the *vgrG* loci for the presence of four rather than three genes (encoding the VgrG, effector and immunity proteins). The putative chaperone genes identified were typically small and all were located between the *vgrG* and effector gene. A total of 30 putative chaperone genes were identified across the 280 *vgrG* loci examined, and together were associated with eight of the 32 effector groups. The predicted chaperone genes were genetically co-located with effector genes from groups 7, 9, 10, 11, 12, 20, 24 and 25; those associated with group 10, 12, and 20 effectors all contained a DUF4123 domain (Figure 7). Predicted chaperones encoding a URO-D-CIMS-like domain, associated with homocysteine methyltransferases and not previously identified in T6SS chaperones, were only associated with the group 7 effectors (Miyata et al., 2013; Alcoforado Diniz and Coulthurst, 2015; Liang et al., 2015; Unterweger et al., 2015; Whitney et al., 2015; Bondage et al., 2016; Cianfanelli et al., 2016a; Zepeda-Rivera et al., 2017).

In order to assess whether particular chaperones had co-evolved with the effectors with which they were genetically associated, we produced a ML phylogenetic tree of the predicted chaperone proteins (Figure 7). The chaperone proteins clustered into well separated groups with each chaperone group typically being associated with a single effector type (Figures 2, 7), indicating that the chaperones and associated effectors have likely co-evolved. The only exception to this was a set of highly related chaperones genetically associated with effectors from the very closely related groups 12 and 20. These effector groups are located on the same branch tip of the effector tree (Figure 2) and have almost identical sequences for the first 302 N-terminal amino acids (approximately one third of their lengths), indicating that these related effectors are likely to functionally interact with related chaperones.

### Immunity Proteins Group According to Predicted Cellular Localization and Cognate Effector Function

**FIGURE 8:**
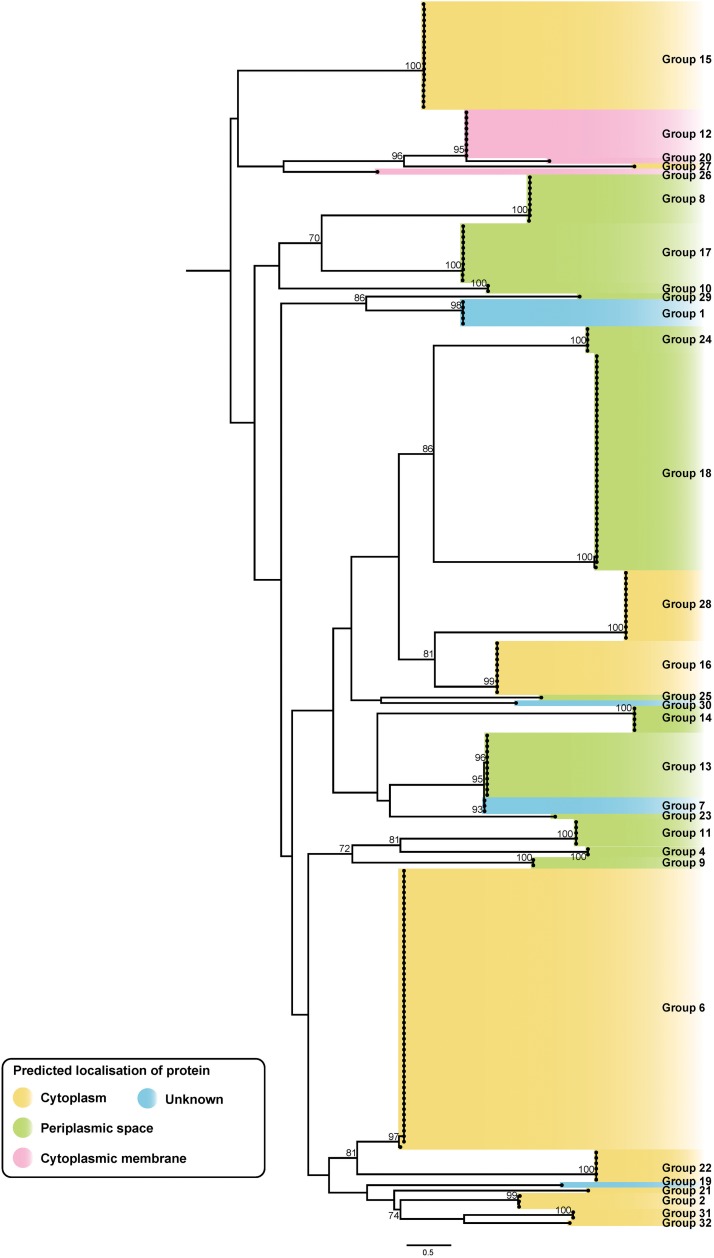
ML phylogenetic tree of putative *A. baumannii* T6SS immunity proteins. The tree was generated from amino acid sequences using the general ‘variable time’ matrix with the discrete Gamma model (VT + F + G4). Branches have been named according to the effector loci to which the immunity proteins correspond. The scale bar represents the number of amino acid substitutions. Branch support values ≥70 (UFBoot) are shown at all major nodes. Colored highlight denotes expected localization of the proteins (Supplementary Files S3e, S4e).

A total of 228 putative immunity proteins were identified across all the *A. baumannii* genomes examined and these were used to generate a ML phylogenetic tree (Figure 8). No conserved functional domains were identified in any of the predicted immunity proteins. Immunity proteins must specifically bind and/or neutralize the activity of their cognate toxin (Hood et al., 2010; Yang et al., 2018) and supporting this relationship, the distribution of the *A. baumannii* immunity proteins closely matched those of their cognate effectors. Indeed, the immunity proteins were always associated with other immunity proteins from the same effector family, with which they were also genetically co-located, and there were only two instances where different immunity protein families were closely phylogenetically related to immunity proteins from other effector families. Firstly, the immunity proteins associated with group 20 effectors clustered closely with immunity proteins associated with group 12 effectors, but as described above, the group 12 and 20 effector proteins are almost identical for the initial 302 amino acids and also share highly related chaperones. Secondly, the immunity proteins associated with group 7 effectors clustered closely with immunity proteins associated with group 13 effectors and shared a very high level of amino acid sequence identity (>95%), despite the effectors from these two groups showing no evolutionary relationship (Figure 2A). This may indicate that proteins belonging to the 7 and 13 effector groups, both with no predicted functional domains, may have a similar fold and/or function, allowing interaction with closely related immunity proteins.

Overall, genes representing 30 of the 32 effector groups co-localized with a gene encoding a putative immunity protein. The two effector groups that lacked a clear cognate immunity protein were the group 3 effectors, represented in two genomes, and the group 5 RHS effectors, represented in 30 genomes; no functional effector domains were identified in either of these effector groups. While it is possible that the group 3 effectors are inactive, and therefore do not require a neutralizing immunity protein, this would seem unlikely for the group 5 effectors as they are widely distributed across the species and are highly conserved. A more likely explanation is that the target of these effectors is absent in these *A. baumannii* strains and therefore the encoded effector protein is non-toxic against the parent strain. This has been observed previously with T6SS effectors that have anti-mammalian or antifungal activity (Chen et al., 2017; Wan et al., 2017; Trunk et al., 2018).

The predicted sub-cellular location of each immunity protein was assessed using SignalP and PSORTb. To date, no signal peptides have been identified in T6SS effector proteins, and it is likely that they are delivered directly to their target location by the T6SS. However, the sub-cellular location of the cognate immunity protein should indicate the location of the effector’s target molecule, as the immunity protein is required in the correct cellular compartment for protection against self- and/or sibling intoxication. Both SignalP and PSORTb algorithms identified signal peptides in proteins representing five of the 30 immunity protein groups and either SignalP or PSORTb predicted signal peptides in proteins representing six other immunity groups. As expected, the immunity proteins associated with all putative nuclease effectors and the nucleic acid deaminase effector were predicted to be cytoplasmic. Signal peptides were predicted by one, or both, programs for seven of the nine immunity protein groups associated with putative peptidoglycan hydrolase effectors. Signal peptides were also detected for four of the immunity protein groups associated with effectors with unknown targets (groups 10, 11, 13 and 25) suggesting that the target of these effectors is accessible from the periplasm. Transmembrane domains of each immunity protein were identified using TMHMM tool. From this analysis, the immunity proteins corresponding to the groups 12, 20, 23, and 26 were all determined to have transmembrane domains and thus were predicted to bind to a cellular membrane. The PSORTb prediction for group 12, 20, and 26 immunity proteins suggested a localization to the cytoplasmic membrane, and this, together with the transmembrane domain data, suggests that the proteins in these three immunity groups all localize to the cytoplasmic membrane. The sub-cellular location of the group 23 immunity proteins could not be predicted. The branches of the immunity protein ML phylogenetic tree were categorized to reflect the likely localization of the proteins (Figure 8), based on the predictions of SignalP, PSORTb, and TMHMM, and on the location of the predicted targets of the cognate effector proteins. Based on the aforementioned criteria, representatives of 10 immunity protein groups were predicted to localize to the cytoplasm, three to the cytoplasmic membrane and 13 to the periplasmic space. The sub-cellular location of proteins belonging to four immunity groups could not be determined.

### *A. baumannii* T6SS VgrG Proteins Share a Common Origin and Cluster Based on the Cognate Effector That They Deliver

**FIGURE 9:**
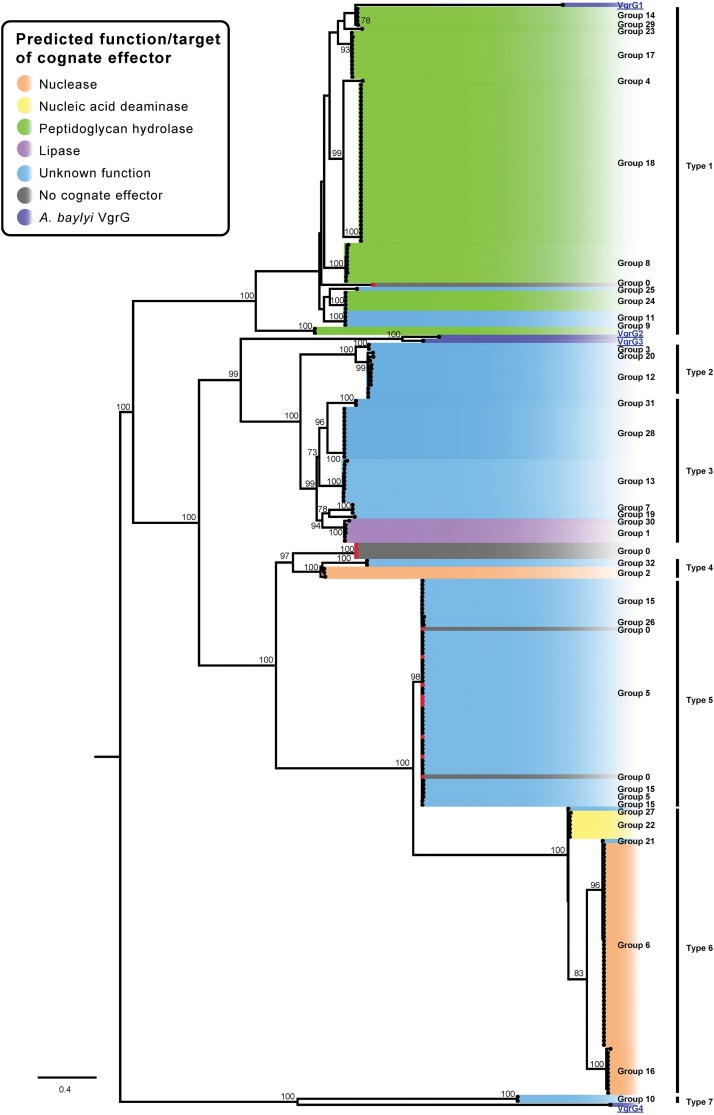
ML phylogenetic tree of *A. baumannii* VgrG proteins. The tree was generated from the VgrG amino acid sequences using the general ‘variable time’ matrix with the discrete Gamma model (VT + F + G4). *A. baylyi* ADP1 VgG proteins are included for comparison. The scale bar represents the number of amino acid substitutions. Branch support values ≥70 (UFBoot) are shown at all major nodes. Black names represent *A. baumannii* VgrG proteins, groups 0–32, blue underlined names represent *A. baylyi* VgrG proteins. *A. baumannii* VgrG proteins that do have a cognate effector encoded downstream are indicated by a red dot at the end of the branch (Supplementary Files S3f, S4f). The different types of closely related VgrG proteins are shown on the right side of the ML phylogenetic tree (Type 1–7).

The amino acid sequences of the identified VgrG proteins were used to generate a VgrG ML phylogenetic tree (Figure 9). No evolved VgrG proteins (i.e., translationally fused to effectors) were identified in the *Acinetobacter* genus. In keeping with the above analyses, the VgrG proteins were named based on the group of the cognate (co-located) effector. A designation of zero was assigned to any VgrG proteins that were encoded within a locus lacking a predicted effector gene. The four characterized VgrG proteins (VgrG1, VgrG2, VgrG3 and VgrG4) from *Acinetobacter baylyi* ADP1, a closely related species, were used as outgroups in the phylogenetic analysis (Figure 9) (Ringel et al., 2017). The *A. baumannii* VgrG proteins generally grouped according to the specific type of effector that each is predicted to deliver, as observed previously when a smaller group of VgrG proteins was analyzed (Fitzsimons et al., 2018). For example, all of the VgrG proteins that were encoded on the same locus as a peptidoglycan hydrolase effector (groups 4, 8, 9, 14, 17, 18, 23, 24 and 29) clustered closely together. This tight cluster also included VgrG proteins that were encoded upstream of genes encoding effectors from group 11 and 25 (Figures 2, 8). The cognate immunity proteins for both the group 11 and group 25 effectors were identified to contain a signal peptide (SignalP) and PSI-BLAST analysis of the group 11 and 25 effectors identified matches with other peptidoglycan hydrolases after more than four iterations. Taken together, these data suggest that the target of both the group 11 and group 25 effectors, members of which lack any clear conserved domains, is likely to be peptidoglycan. VgrG proteins encoded in the same loci as genes encoding RHS effectors (groups 5, 6, 15, 16, 21, 22, 26 or 27) all clustered within closely associated clades (Figure 9). Thus, VgrG proteins that deliver RHS-type effectors are all evolutionarily related, suggesting similar interaction mechanisms between the VgrG tip proteins and RHS effectors.

In a previous study, the VgrG proteins encoded on the same locus as predicted DUF4123-containing chaperones were shown to all cluster together (Fitzsimons et al., 2018). We found that DUF4123-containing chaperones were associated with group 10, 12, and 20 effectors but only the VgrG proteins associated with the group 12 and 20 effectors clustered very closely. The VgrG proteins associated with the group 10 effectors were unrelated and clustered with the VgrG4 from *A. baylyi*, which is encoded in a locus that does not encode a DUF4123-containing chaperone. These data indicate that the VgrG proteins associated with DUF4123-containing chaperones are not closely related and suggest that VgrG proteins are adapted more for interaction with their cognate effector than associated chaperones.

All of the currently identified *Acinetobacter* VgrG proteins, including those from *A. baylyi*, shared a high level of amino acid identity (>53%), suggesting that they have arisen from a common ancestor. This is in contrast to the effectors (Figure 2), which have very low levels of shared amino acid identity (beyond the pre-cleavage region in the case of RHS proteins) and for which our phylogenetic analyses indicate have arisen from a multitude of different ancestors. The strong conservation of the VgrG primary sequence is perhaps unsurprising as VgrG proteins are a crucial structural component of the T6SS apparatus tip and the other structural components are highly conserved within, and between, species (Pukatzki et al., 2007). In contrast to the VgrG proteins, our analyses clearly show that even effectors with similar predicted functions do not cluster together, and therefore are not evolutionarily related. The disparity between the degree of conservation displayed by the VgrG tip proteins and their cognate effectors suggests that the effectors have been acquired independently of the VgrG proteins and have been adapted for delivery by the T6SS following acquisition. A similar relationship has been observed in *Vibrio cholerae* and it has been proposed that some of the current *V. cholerae* T6SS effectors, co-located upstream of a gene encoding a cognate immunity protein, may have replaced historic effectors (Kirchberger et al., 2017). These newly acquired genes are often found in the same locus as additional immunity genes, likely to have been required for the protection of the cell from the historic effector (Kirchberger et al., 2017).

### Effector Loci Are Found at Only a Small Number of Genomic Positions

**FIGURE 10:**
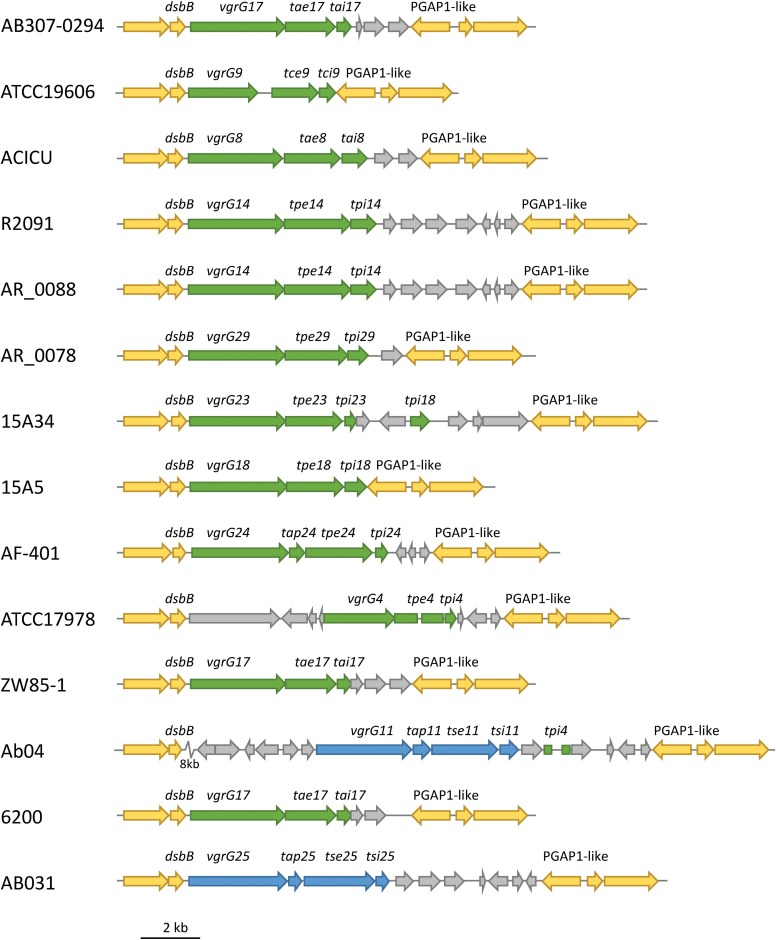
Conserved genomic position of effector loci encoding a Type 1 VgrG protein. The genomic positions of effector loci encoding Type 1 VgrG proteins were identified in 14 *A. baumannii* strains and shown to be present between conserved *dsbB* (upstream) and PGAP1-like (downstream) genes. These loci are predicted to encode peptidoglycan hydrolase effectors (green) and two effectors with no predicted function (blue). Genes conserved across all strains are shown in yellow. Gray genes are additional genes that were not conserved across all strains. Rectangles represent truncated genes. An 8-kbp section of Ab04 (zig zag symbol) was condensed for clarity and contained only non-conserved genes.

In order to identify if the effector loci were found at conserved genomic positions in different strains, we used Mauve (Version 1.1.1) to align selected genomes and identified the relative genomic location of the 32 *A. baumannii* effector loci. We identified different effector loci at only seven distinct genomic locations. Interestingly, effector loci that shared highly related VgrG proteins (Types 1–7; Figure 9) were always encoded at the same genomic location, even across unrelated strains. For example, the loci encoding the Type 1 VgrG proteins (which includes VgrG groups 4, 8, 9, 11, 14, 17, 18, 23, 24, 25 and 29) were always flanked by an upstream gene encoding a disulphide bond formation protein (DsbB), and a downstream gene encoding a PGAP1-like protein (Figure 10). The effectors in these loci are predicted to target peptidoglycan (effector groups 4, 8, 9, 14, 17, 18, 23, 24 and 29) or have unknown functions but are predicted to act in the periplasm (11 and 25). Similarly, the loci encoding type 6 VgrG proteins (which includes VgrG and effector groups 6, 16, 21, 22 and 27), were also always found at a conserved genomic position between a beta lactamase gene and *gltR*, encoding a transcriptional repressor (data not shown). The effectors associated with these loci target DNA (effectors 6, 16 and 22) or have unknown functions but are predicted to act in the cytoplasm (effectors 21 and 27). Taken together, these data indicate that *A. baumannii* may follow the sequential displacement mechanism observed for the T6SS effector loci in *V. cholerae* (Kirchberger et al., 2017).

Additional genes were often found surrounding the effector locus, including, in a few instances, genes encoding orphan immunity proteins. For example, a *tpi*18 homolog, which is predicted to encode an immunity protein for the group 18 effectors, was identified downstream of the *tpe23* locus (Figure 10). Similarly, a *tdi16* homolog was identified downstream of the *tde6* locus (data not shown). Thus, a small number of effector loci encode multiple immunity genes, one for the cognate effector and one for a non-cognate effector. This has been previously observed for *V. cholerae* (Kirchberger et al., 2017) and is likely associated with sequential displacement of incomplete effector locus segments. It is likely that there is some selective advantage to retaining non-cognate immunity genes.

### The *A. baumannii* Hcp Tube and PAAR Tip Proteins Do Not Appear to Encode Fused Functional Effector Domains

Evolved T6SS effectors have been identified in some species outside of the *Acinetobacter* genus. Evolved effectors translationally fused to Hcp proteins have been identified in *Salmonella* sp. and *Enterobacteriaceae* (Ma et al., 2017). We examined the 97 *A. baumannii* genomes for evidence of any genes encoding Hcp proteins translationally fused to effectors (Supplementary File S1). A single *hcp* gene was identified in the core T6SS locus for 66 of the 97 genomes: all shared 100% identity at the amino acid level. This included strain *A. baumannii* AB307-0294, which is known to produce only three antibacterial cargo effectors active against *E. coli* and *A. baylyi*; namely, Tse15^AB^ (Rhs1), Tde16^AB^ (Rhs2) and Tae17^AB^ (LysM) (Fitzsimons et al., 2018). The remaining 31 genomes examined lacked a *hcp* gene entirely or encoded a truncated/non-functional *hcp* gene. Together, these data strongly suggest that *A. baumannii* does not express evolved Hcp effectors.

**FIGURE 11:**
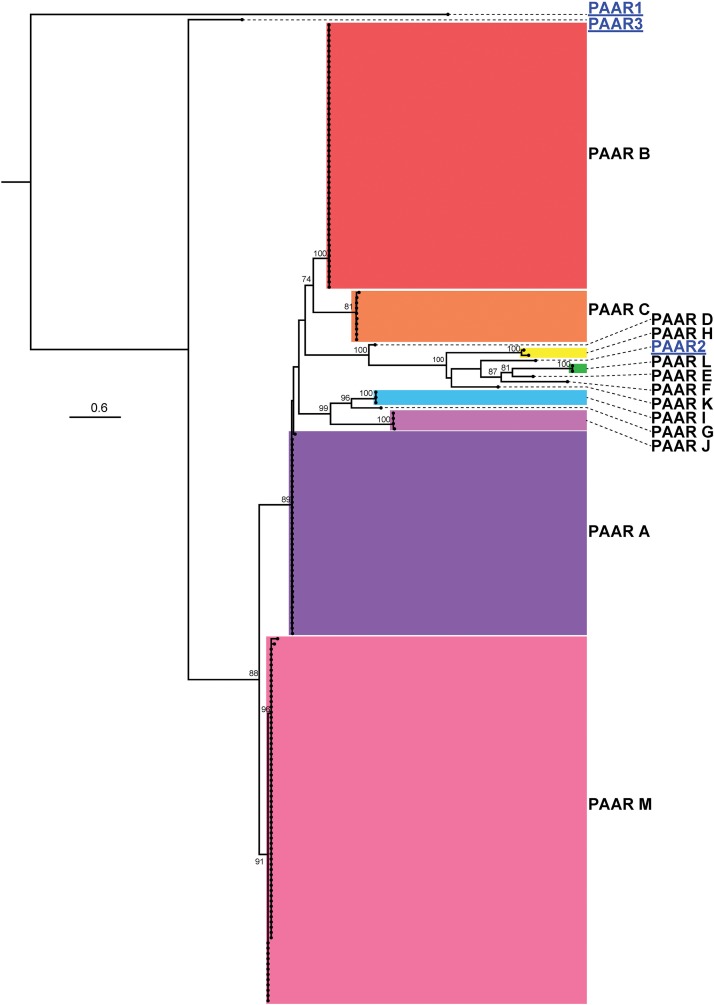
ML phylogenetic tree of *A. baumannii* PAAR proteins with *A. baylyi* ADP1 PAAR proteins used for comparison. The tree was generated from PAAR amino acid sequences using the general ‘variable time’ matrix with the discrete Gamma model (VT + G4). The scale bar represents the number of amino acid substitutions. Branch support values ≥ 70 (UFBoot) are shown at all major nodes. Black names represent *A. baumannii* PAAR proteins, grouped A-M, blue underlined names represent *A. baylyi* PAAR proteins. Separate areas of the tree have been arbitrarily colored to visually define the groups (Supplementary Files S3g, S4g).

Studies on the T6SS in other bacterial species indicate that PAAR proteins form the penetrating spike of the T6SS and can be translationally fused to effectors, often including those containing an RHS domain (Koskiniemi et al., 2013; Whitney et al., 2014; Alcoforado Diniz and Coulthurst, 2015; Bernal et al., 2017). Previous studies on *A. baylyi* ADP1 showed that three PAAR proteins played a role in T6SS action via sharpening of the T6SS tip, but individually these PAAR proteins did not possess any toxicity and thus are unlikely to carry evolved effector domains (Shneider et al., 2013; Ringel et al., 2017). Analysis of the 97 *A. baumannii* genomes predicted that six strains did not encode any PAAR proteins (five of these did not have an intact T6SS structural locus) and 91 strains had between one and four genes predicted to encode PAAR proteins. However, none of the genes encoding PAAR proteins were co-located with a gene encoding a predicted effector; one gene was always encoded within the main T6SS structural locus while any additional *paar* genes were located elsewhere on the genome. The predicted amino acid sequences of the PAAR proteins, ranging in size from 87 amino acids to 280 amino acids, were used to generate an ML phylogenetic tree (Figure 11). The thirteen groups of PAAR proteins identified, designated A-M, formed six distinct clades.

The clade M PAAR proteins were the shortest (87 amino acids) and most abundant. These PAAR proteins were always encoded within the core T6SS locus and were found almost exclusively in strains that also encoded a Hcp protein. The PAAR proteins that belonged to the other groups were all considerably longer (172–280 amino acids), encoded an extended C-terminal region (extended CT), and were encoded by genes located outside of the main T6SS structural locus. However, none of these longer proteins encoded any identifiable conserved domains in the extended CT region. A similar arrangement of *paar* genes was observed previously in *A. baylyi* ADP1, where the smallest PAAR protein (∼90 amino acids) was encoded within the main T6SS locus and the two larger PAAR proteins were encoded together elsewhere on the genome (a pattern not seen in *A. baumannii*). While these larger *A. baylyi* PAAR proteins do not have intrinsic toxic activity, it is proposed that they aid in the secretion of toxic effectors (Ringel et al., 2017).

With respect to strain lineage, the most common group of PAAR proteins was group M, which was encoded within the highly conserved main T6SS structural locus and found in many diverse lineages. With the exception of the group B PAAR proteins, which were encoded solely by GC-II lineage strains, the remaining groups of PAAR proteins were not confined to a single lineage or genome group indicating that the distribution of the PAAR proteins does not correlate with strain lineage or effector distribution. For example, *A. baumannii* AB307-0294 and *A. baumannii* A85, which are both GC-I strains that encode the group 14, 15, and 16 effectors, encoded group M and C, and group M and J PAAR proteins respectively. As the PAAR groups do not correlate with the effector groups in different strains, it is unlikely that PAAR proteins directly interact with and deliver specific effectors. Furthermore, the lack of any functional domains or any PAAR-mediated toxicity in *A. baumannii* strain AB307-0294, suggest that the extended C-terminal region is unlikely to act as an evolved effector. The purpose and diversity of the extended CT region in the *A. baumannii* PAAR proteins remains to be elucidated.

## Conclusion

Bioinformatic analysis of 97 *A. baumannii* genomes allowed us to identify a diverse array of putative T6SS components. We identified genes predicted to encode 272 VgrG proteins, 244 putative effectors and 228 immunity proteins. Detailed bioinformatic analyses indicated that the putative effectors clustered into 32 different groups and that the effectors in these groups were mostly evolutionarily unrelated, indicating that they have likely been acquired from a range of sources and co-opted for delivery by the T6SS. We predict that these effectors, many of unknown function, represent a major resource for the development of novel antibacterial proteins. Two groups of nuclease effectors were widely distributed within each of the major global clone lineages; the group 16 nuclease effectors were restricted to strains within the GC-I lineage and group 6 nuclease effectors were restricted to strains within the GC-II lineage. It is currently unknown why these two nuclease effector groups are GC-lineage restricted, but it is possible that their prevalence has given an evolutionary advantage to these strains and played a role in the global expansion of these clonal groups. We identified many effectors that belonged to the RHS family of proteins and these shared a highly conserved N-terminal region (encoding the RHS domain) but had unrelated C-terminal toxin domains. Similarly, highly conserved VgrG proteins were observed to be associated with diverse range of effectors. These data suggest that common delivery determinants can be used to deliver a diverse range of toxic effectors. Future work will focus on identifying the specific regions in these proteins that drive T6SS effector delivery. While it is unlikely that the effectors identified in this study represent an exhaustive list of the effectors produced by *A. baumannii*, our current analysis provides an excellent base for the future detailed experimental characterisation of these highly diverse effectors.

## DATA AVAILABILITY STATEMENT

All datasets generated for this study are included in the manuscript/Supplementary Files.

## Author Contributions

JL performed all of the bioinformatic analyses, with the exception of the whole genome phylogenetic tree, which was generated by JB. All authors were involved in experimental design and manuscript editing.

## Conflict of Interest

The authors declare that the research was conducted in the absence of any commercial or financial relationships that could be construed as a potential conflict of interest.

## Footnotes

1 http://tree.bio.ed.ac.uk/software/figtree/

2 https://www.geneious.com

## Supplementary Material

TABLE S1 Characterized T6SS effector proteins from various bacterial species (excluding *Acinetobacter* effectors).

FILE S1 *A. baumannii* strains used in this study, amino acid sequence of the T6SS Hcp, TssM and PAAR proteins, and overall presence or absence of the core T6SS locus.

FILE S2 *A. baumannii vgrG* loci identified in this study including the amino acid sequences and locus tags of all VgrG, chaperone, effector and immunity proteins.

FILE S3 Raw ML phylogenetic tree files. **(a–g)** Each sequence has a designated name denoting the strain from where it was derived followed by an underscore then the number of the group the effector region belongs to (based on effector groups, see ML phylogenetic tree shown in Figure 2). **(a)** Nexus file of *A. baumannii* T6SS predicted effectors. **(b)** Nexus file of *A. baumannii* and other characterized T6SS effectors. **(c)** Nexus file of *A. baumannii* RHS pre-D(P/S)xGx cleavage sequences. **(d)** Nexus file of *A. baumannii* predicted chaperone proteins. **(e)** Nexus file of *A. baumannii* predicted immunity proteins. **(f)** Nexus file of *A. baumannii* and *A. baylyi* VgrG proteins. **(g)** Nexus file of *A. baumannii* and *A. baylyi* PAAR proteins.

FILE S4 Corrected amino acid alignment files (fasta format) used for generating the phylogenetic trees. **(a–g)** Each sequence has a designated name denoting the strain from where it was derived followed by an underscore then the number of the group the effector region belongs to (based on effector groups, see ML phylogenetic tree shown in Figure 2). **(a)** Fasta file of *A. baumannii* T6SS predicted effectors. **(b)** Fasta file of *A. baumannii* and other characterized T6SS effectors. **(c)** Fasta file of *A. baumannii* RHS pre-D(P/S)xGx cleavage sequences. **(d)** Fasta file of *A. baumannii* predicted chaperone proteins. **(e)** Fasta file of *A. baumannii* predicted immunity proteins. **(f)** Fasta file of *A. baumannii* and *A. baylyi* VgrG proteins. **(g)** Fasta file of *A. baumannii* and *A. baylyi* PAAR proteins.

FILE S5 *A. baumannii* effector pair-wise amino acid identity matrix. Reported values are the maximum identity of the smallest protein sequence of the pair across the entire sequence length.

FILE S6 Immunity protein predicted sub-cellular location.

FILE S7 *A. baumannii* predicted PAAR proteins.
