# Supplementary material for: Systematic Identification and Analysis of Acinetobacter baumannii Type VI Secretion System Effector and Immunity Components
Source: Front Microbiol. 2019 Oct 30;10:2440. doi: 10.3389/fmicb.2019.02440 (PMC6833914; doi:10.3389/fmicb.2019.02440)
Supplement: TABLE S1 — Characterized T6SS effector proteins from various bacterial species (excluding Acinetobacter effectors). [file Table_1.PDF]

SUPPLEMENTARY TABLE 1 | Characterised T6SS effectors proteins from various bacterial species (excluding *Acinetobacter* effectors).

**Effector**	**Strain**	**Function**	**Reference**
EvpP	*Edwardsiella tarda*	Inhibition of NLRP3 inflammasome	(Chen et al., 2017)
Hcp-ET1	*Escherichia coli*	DNase	(Ma et al., 2017)
KatN	*E. coli*	Mn containing catalase	(Wan et al., 2017)
PldB	*Pseudomonas aeruginosa*	Phospholipase and activation of Akt signalling pathway	(Jiang et al., 2014)
Rhs1	*Serratia marcescens*	Unknown	(Alcoforado Diniz and Coulthurst, 2015)
Rhs2	*S. marcescens*	DNase	(Alcoforado Diniz and Coulthurst, 2015)
RhsA	*Dickeya dadantii*	DNase	(Koskiniemi et al., 2013)
RhsB	*D. dadantii*	DNase	(Koskiniemi et al., 2013)
Tde1	*Agrobacterium tumefaciens*	DNase	(Bondage et al., 2016)
Tde2	*A. tumefaciens*	DNase	(Bondage et al., 2016)
TecA	*Burkholderia cenocepacia*	Deaminating Rho GTPase	(Aubert et al., 2016)
Tfe1	*S. marcescens*	Disrupt plasma membrane potential	(Trunk et al., 2018)
Tfe2	*S. marcescens*	Disrupts nutrient uptake and amino acid metabolism of fungi	(Trunk et al., 2018)
Tge2	*Pseudomonas protegens*	Glycoside hydrolase	(Whitney et al., 2013)
Tke10	*Pseudomonas putida*	Nuclease	(Bernal et al., 2017)
Tke2	*P. putida*	Unknown	(Bernal et al., 2017)
Tke4	*P. putida*	Unknown, probable nuclease	(Bernal et al., 2017)
Tke6	*P. putida*	Colicin pore-forming	(Bernal et al., 2017)
Tke7	*P. putida*	Colicin pore-forming	(Bernal et al., 2017)
Tke9	*P. putida*	Unknown	(Bernal et al., 2017)

Tle1^{Bt}	*Burkholderia thailandensis*	Phospholipase	(Russell et al., 2013)
Tle1^{Ec}	*E. coli*	Phospholipase	(Flaugnatti et al., 2016)
Tle2	*Vibrio cholerae*	Phospholipase	(Russell et al., 2013)
Tle3	*P. aeruginosa*	Phospholipase	(Russell et al., 2013)
Tle4	*P. aeruginosa*	Abhydrolase	(Russell et al., 2013)
Tle5	*P. aeruginosa*	Phospholipase	(Russell et al., 2013)
Tse1	*P. aeruginosa*	Amidase	(Hachani et al., 2014)
Tse2	*P. aeruginosa*	NAD-dependant toxicity	(Hachani et al., 2014)
Tse3	*P. aeruginosa*	Muramidase	(Hachani et al., 2014)
Tse4	*P. aeruginosa*	Periplasm acting	(Whitney et al., 2014)
Tse5	*P. aeruginosa*	Unknown (target is membrane associated)	(Hachani et al., 2014)
Tse6	*P. aeruginosa*	NAD(P+) glycohydrolase	(Whitney et al., 2014)
TseF	*P. aeruginosa*	Bind OMV for iron acquisition	(Lin et al., 2017)
TseH	*V. cholerae*	Cell wall degradation hydrolase	(Altindis et al., 2015)
TseM	*B. thailandensis*	Mn2+ binding protein	(Si et al., 2017)
TseT	*P. aeruginosa*	Nuclease	(Burkinshaw et al., 2018)
VasX	*V. cholerae*	Targets lipids in the bacterial membrane	(Miyata et al., 2011)
VgrG1	*Aeromonas hydrophila*	ADP-ribosyltransferase	(Suarez et al., 2010)
VgrG-1	*V. cholerae*	Actin-crosslinking	(Ma et al., 2009)
VgrG2b	*P. aeruginosa*	Interacts with microtubule	(Sana et al., 2015)
VgrG-3	*V. cholerae*	Peptidoglycan hydrolase	(Brooks et al., 2013)
VgrG-5	*B. thailandensis*	Membrane fusion activity	(Schwarz et al., 2014)
YezP	*Yersinia pseudotuberculosis*	Zinc binding protein	(Wang et al., 2015)

REFERENCES

Alcoforado Diniz, J., and Coulthurst, S.J. (2015). Intraspecies competition in *Serratia marcescens* is mediated by type VI-secreted Rhs effectors and a conserved effector-associated accessory protein. *J Bacteriol* 197(14), 2350-2360.

Altindis, E., Dong, T., Catalano, C., and Mekalanos, J. (2015). Secretome analysis of *Vibrio cholerae* type VI secretion system reveals a new effector-immunity pair. *MBio* 6(2), e00075. doi: 10.1128/mBio.00075-15.

Aubert, D.F., Xu, H., Yang, J., Shi, X., Gao, W., Li, L., et al. (2016). A Burkholderia Type VI Effector Deamidates Rho GTPases to Activate the Pyrin Inflammasome and Trigger Inflammation. *Cell Host Microbe* 19(5), 664-674. doi: 10.1016/j.chom.2016.04.004.

Bernal, P., Allsopp, L.P., Filloux, A., and Llamas, M.A. (2017). The *Pseudomonas putida* T6SS is a plant warden against phytopathogens. *ISME J* 11(4), 972-987.

Bondage, D.D., Lin, J.S., Ma, L.S., Kuo, C.H., and Lai, E.M. (2016). VgrG C terminus confers the type VI effector transport specificity and is required for binding with PAAR and adaptor-effector complex. *Proc Natl Acad Sci U S A* 113(27), E3931-3940.

Brooks, T.M., Unterweger, D., Bachmann, V., Kostiuk, B., and Pukatzki, S. (2013). Lytic activity of the *Vibrio cholerae* type VI secretion toxin VgrG-3 is inhibited by the antitoxin TsaB. *J Biol Chem* 288(11), 7618-7625.

Burkinshaw, B.J., Liang, X., Wong, M., Le, A.N.H., Lam, L., and Dong, T.G. (2018). A type VI secretion system effector delivery mechanism dependent on PAAR and a chaperone-co-chaperone complex. *Nat Microbiol* 3(5), 632-640.

Chen, H., Yang, D., Han, F., Tan, J., Zhang, L., Xiao, J., et al. (2017). The bacterial T6SS effector EvpP prevents NLRP3 inflammasome activation by inhibiting the Ca(2+)-dependent MAPK-Jnk pathway. *Cell Host Microbe* 21(1), 47-58.

Flaugnatti, N., Le, T.T., Canaan, S., Aschtgen, M.S., Nguyen, V.S., Blangy, S., et al. (2016). A phospholipase A1 antibacterial type VI secretion effector interacts directly with the C-terminal domain of the VgrG spike protein for delivery. *Mol Microbiol* 99(6), 1099-1118. doi: 10.1111/mmi.13292.

Hachani, A., Allsopp, L.P., Oduko, Y., and Filloux, A. (2014). The VgrG proteins are "a la carte" delivery systems for bacterial type VI effectors. *J Biol Chem* 289(25), 17872-17884. doi: 10.1074/jbc.M114.563429.

Jiang, F., Waterfield, N.R., Yang, J., Yang, G., and Jin, Q. (2014). A *Pseudomonas aeruginosa* type VI secretion phospholipase D effector targets both prokaryotic and eukaryotic cells. *Cell Host Microbe* 15(5), 600-610.

Koskiniemi, S., Lamoureux, J.G., Nikolakakis, K.C., t'Kint de Roodenbeke, C., Kaplan, M.D., Low, D.A., et al. (2013). Rhs proteins from diverse bacteria mediate intercellular competition. *Proc Natl Acad Sci U S A* 110(17), 7032-7037.

Lin, J., Zhang, W., Cheng, J., Yang, X., Zhu, K., Wang, Y., et al. (2017). A *Pseudomonas* T6SS effector recruits PQS-containing outer membrane vesicles for iron acquisition. *Nat Commun* 8, 14888. doi: 10.1038/ncomms14888.

Ma, A.T., McAuley, S., Pukatzki, S., and Mekalanos, J.J. (2009). Translocation of a *Vibrio cholerae* type VI secretion effector requires bacterial endocytosis by host cells. *Cell Host Microbe* 5(3), 234-243.

Ma, J., Pan, Z., Huang, J., Sun, M., Lu, C., and Yao, H. (2017). The Hcp proteins fused with diverse extended-toxin domains represent a novel pattern of antibacterial effectors in type VI secretion systems. *Virulence* 8(7), 1189-1202.

Miyata, S.T., Kitaoka, M., Brooks, T.M., McAuley, S.B., and Pukatzki, S. (2011). V*ibrio cholerae* requires the type VI secretion system virulence factor VasX to kill *Dictyostelium discoideum*. *Infect Immun* 79(7), 2941-2949. doi: 10.1128/iai.01266-10.

Russell, A.B., LeRoux, M., Hathazi, K., Agnello, D.M., Ishikawa, T., Wiggins, P.A., et al. (2013). Diverse type VI secretion phospholipases are functionally plastic antibacterial effectors. *Nature* 496(7446), 508-512. doi: 10.1038/nature12074.

Sana, T.G., Baumann, C., Merdes, A., Soscia, C., Rattei, T., Hachani, A., et al. (2015). Internalization of *Pseudomonas aeruginosa* strain PAO1 into epithelial cells is promoted by interaction of a T6SS effector with the microtubule network. *MBio* 6(3)**,** e00712. doi: 10.1128/mBio.00712-15.

Schwarz, S., Singh, P., Robertson, J.D., LeRoux, M., Skerrett, S.J., Goodlett, D.R., et al. (2014). VgrG-5 is a *Burkholderia* type VI secretion system-exported protein required for multinucleated giant cell formation and virulence. *Infect Immun* 82(4)**,** 1445-1452. doi: 10.1128/iai.01368-13.

Si, M., Zhao, C., Burkinshaw, B., Zhang, B., Wei, D., Wang, Y., et al. (2017). Manganese scavenging and oxidative stress response mediated by type VI secretion system in *Burkholderia thailandensis*. *Proc Natl Acad Sci U S A* 114(11)**,** E2233-e2242. doi: 10.1073/pnas.1614902114.

Suarez, G., Sierra, J.C., Erova, T.E., Sha, J., Horneman, A.J., and Chopra, A.K. (2010). A type VI secretion system effector protein, VgrG1, from *Aeromonas hydrophila* that induces host cell toxicity by ADP ribosylation of actin. *J Bacteriol* 192(1)**,** 155-168. doi: 10.1128/jb.01260-09.

Trunk, K., Peltier, J., Liu, Y.C., Dill, B.D., Walker, L., Gow, N.A.R., et al. (2018). The type VI secretion system deploys antifungal effectors against microbial competitors. *Nat Microbiol* 3(8)**,** 920-931. doi: 10.1038/s41564-018-0191-x.

Wan, B., Zhang, Q., Ni, J., Li, S., Wen, D., Li, J., et al. (2017). Type VI secretion system contributes to Enterohemorrhagic *Escherichia coli* virulence by secreting catalase against host reactive oxygen species (ROS). *PLOS Pathogens* 13(3)**,** e1006246. doi: 10.1371/journal.ppat.1006246.

Wang, T., Si, M., Song, Y., Zhu, W., Gao, F., Wang, Y., et al. (2015). Type VI secretion system transports Zn2+ to combat multiple stresses and host immunity. *PLoS Pathog* 11(7)**,** e1005020. doi: 10.1371/journal.ppat.1005020.

Whitney, J.C., Beck, C.M., Goo, Y.A., Russell, A.B., Harding, B., De Leon, J.A., et al. (2014). Genetically distinct pathways guide effector export through the type VI secretion system. *Mol Microbiol* 92(3)**,** 529-542. doi: 10.1111/mmi.12571.

Whitney, J.C., Chou, S., Russell, A.B., Biboy, J., Gardiner, T.E., Ferrin, M.A., et al. (2013). Identification, structure, and function of a novel type VI secretion peptidoglycan glycoside hydrolase effector-immunity pair. *J Biol Chem* 288(37)**,** 26616-26624. doi: 10.1074/jbc.M113.488320.
