# Supplementary material for: Systematic Identification and Analysis of Acinetobacter baumannii Type VI Secretion System Effector and Immunity Components
Source: Front Microbiol. 2019 Oct 30;10:2440. doi: 10.3389/fmicb.2019.02440 (PMC6833914; doi:10.3389/fmicb.2019.02440)
Supplement: FILE S3 — Raw ML phylogenetic tree files. (a–g) Each sequence has a designated name denoting the strain from where it was derived followed by an underscore then the number of the group the effector region belongs to (based on effector groups, see ML phylogenetic tree shown in Figure 2). (a) Nexus file of A. baumannii T6SS predicted effectors. (b) Nexus file of A. baumannii and other characterized T6SS effectors. (c) Nexus file of A. baumannii RHS pre-D(P/S)xGx cleavage sequences. (d) Nexus file of A. baumannii predicted chaperone proteins. (e) Nexus file of A. baumannii predicted immunity proteins. (f) Nexus file of A. baumannii and A. baylyi VgrG proteins. (g) Nexus file of A. baumannii and A. baylyi PAAR proteins. [file Data_Sheet_3.pdf]

Supplementary File S3. Nexus tree files

Supplementary File S3a

```
#NEXUS
begin taxa;
	dimensions ntax=244;
	taxlabels
	15A34_23[&!color=#000000]
	15A34_5
	15A34_6[&!color=#000000]
	15A5_18[&!color=#000000]
	15A5_28[&!color=#000000]
	15A5_5
	15A5_6[&!color=#000000]
	1656-2_6[&!color=#000000]
	1656-2_8[&!color=#000000]
	3027STDY5784958_18[&!color=#000000]
	3027STDY5784958_5
	3027STDY5784958_6[&!color=#000000]
	3207_1[&!color=#000000]
	3207_4[&!color=#000000]
	6200_15[&!color=#000000]
	6200_17[&!color=#000000]
	6200_21[&!color=#000000]
	A1296_8[&!color=#000000]
	A1_15[&!color=#000000]
	A1_16[&!color=#000000]
	A1_17[&!color=#000000]
	A388_15[&!color=#000000]
	A388_16[&!color=#000000]
	A388_17[&!color=#000000]
	A85_15[&!color=#000000]
	A85_16[&!color=#000000]
	A85_17[&!color=#000000]
	AB0057_15[&!color=#000000]
	AB0057_16
	AB030_15[&!color=#000000]
	AB030_22[&!color=#000000]
	AB030_24[&!color=#000000]
	AB031_25[&!color=#000000]
	AB031_26[&!color=#000000]
	AB031_27[&!color=#000000]
	AB042_1[&!color=#000000]
	AB042_3
	AB07_18[&!color=#000000]
	AB07_6[&!color=#000000]
	AB307-0294_15[&!color=#000000]
```

AB307-0294_16[&!color=#000000]
AB307-0294_17[&!color=#000000]
AB34299_6[&!color=#000000]
AB5075-UW_15[&!color=#000000]
AB5075-UW_16[&!color=#000000]
AB5075-UW_17[&!color=#000000]
AC29_18[&!color=#000000]
AC29_6[&!color=#000000]
AC30_18[&!color=#000000]
AC30_6[&!color=#000000]
ACICU_5
ACICU_6[&!color=#000000]
ACICU_7[&!color=#000000]
ACICU_8[&!color=#000000]
AF-401_15[&!color=#000000]
AF-401_22[&!color=#000000]
AF-401_24[&!color=#000000]
AF-401_30[&!color=#000000]
AF-673_18[&!color=#000000]
AF-673_5
AF-673_6[&!color=#000000]
AR_0056_5
AR_0056_6[&!color=#000000]
AR_0056_8[&!color=#000000]
AR_0063_15[&!color=#000000]
AR_0063_22[&!color=#000000]
AR_0063_24[&!color=#000000]
AR_0078_15[&!color=#000000]
AR_0078_29[&!color=#000000]
AR_0083_15[&!color=#000000]
AR_0083_16[&!color=#000000]
AR_0083_17[&!color=#000000]
AR_0088_1[&!color=#000000]
AR_0088_12[&!color=#000000]
AR_0088_14[&!color=#000000]
AR_0088_32[&!color=#000000]
AR_0101_15[&!color=#000000]
AR_0101_22[&!color=#000000]
AR_0101_24[&!color=#000000]
AR_0102_18[&!color=#000000]
AR_0102_5
ATCC17978-mff_1[&!color=#000000]
ATCC17978-mff_2[&!color=#000000]
ATCC17978-mff_3
ATCC17978-mff_4[&!color=#000000]
ATCC19606_10[&!color=#000000]
ATCC19606_7[&!color=#000000]

ATCC19606_9[&!color=#000000]
AYE_17[&!color=#000000]
AYP-A2_18[&!color=#000000]
AYP-A2_6[&!color=#000000]
Ab04-mff_11[&!color=#000000]
Ab04-mff_12[&!color=#000000]
Ab04-mff_13[&!color=#000000]
Ab4568_18[&!color=#000000]
Ab4568_28[&!color=#000000]
Ab4568_5
Ab4568_6[&!color=#000000]
Ab4653_5
Ab4653_6[&!color=#000000]
Ab4977_18[&!color=#000000]
Ab4977_28[&!color=#000000]
Ab4977_5
Ab4977_6[&!color=#000000]
AbH12O-A2_15[&!color=#000000]
AbH12O-A2_22[&!color=#000000]
AbH12O-A2_24[&!color=#000000]
AbPK1_6[&!color=#000000]
AbPK1_8[&!color=#000000]
BJAB07104_5
BJAB07104_6[&!color=#000000]
BJAB0715_11[&!color=#000000]
BJAB0715_13[&!color=#000000]
BJAB0868_5
BJAB0868_6[&!color=#000000]
CBA7_18[&!color=#000000]
CBA7_6[&!color=#000000]
'CIP70.10_12'[&!color=#000000]
'CIP70.10_14'[&!color=#000000]
'CIP70.10_2'[&!color=#000000]
'CIP70.10_31'[&!color=#000000]
CMC-CR-MDR-Ab4_18[&!color=#000000]
CMC-CR-MDR-Ab4_5
CMC-CR-MDR-Ab4_6[&!color=#000000]
CMC-CR-MDR-Ab66_18[&!color=#000000]
CMC-CR-MDR-Ab66_5
CMC-CR-MDR-Ab66_6[&!color=#000000]
CMC-MDR-Ab59_5
CMC-MDR-Ab59_6[&!color=#000000]
D1279779_12
D1279779_13[&!color=#000000]
D1279779_8
D36_15[&!color=#000000]
D36_16[&!color=#000000]

D36_17[&!color=#000000]
DU202_6[&!color=#000000]
DU202_8[&!color=#000000]
HRAB-85_18[&!color=#000000]
HRAB-85_5
HRAB-85_6[&!color=#000000]
HWAB8_1[&!color=#000000]
HWBA8_12[&!color=#000000]
HWBA8_14[&!color=#000000]
IOMTU433_15[&!color=#000000]
IOMTU433_22[&!color=#000000]
JBA13_18[&!color=#000000]
JBA13_28[&!color=#000000]
JBA13_6[&!color=#000000]
KAB01_6[&!color=#000000]
KAB02_13[&!color=#000000]
KAB02_18[&!color=#000000]
KAB02_6[&!color=#000000]
KAB03_6[&!color=#000000]
KAB03_8[&!color=#000000]
KAB04_18[&!color=#000000]
KAB04_28[&!color=#000000]
KAB04_6[&!color=#000000]
KAB05_13[&!color=#000000]
KAB05_18[&!color=#000000]
KAB05_6[&!color=#000000]
KAB06_13[&!color=#000000]
KAB06_18[&!color=#000000]
KAB06_6[&!color=#000000]
KAB07_18[&!color=#000000]
KAB07_28[&!color=#000000]
KAB07_6[&!color=#000000]
KAB08_18[&!color=#000000]
KAB08_28[&!color=#000000]
KAB08_6[&!color=#000000]
KBN10P02143_28[&!color=#000000]
KBN10P02143_5
KBN10P02143_6[&!color=#000000]
LAC-4_11[&!color=#000000]
LAC-4_12[&!color=#000000]
LAC-4_13[&!color=#000000]
MDR-TJ_13[&!color=#000000]
MDR-TJ_5
MDR-TJ_6[&!color=#000000]
MDR-ZJ06_5
MDR-ZJ06_6[&!color=#000000]
NCGM237_5

NCGM237_6[&!color=#000000]
NCGM237_8
R2090_12
R2090_13[&!color=#000000]
R2090_8[&!color=#000000]
R2091_12[&!color=#000000]
R2091_14[&!color=#000000]
R2091_2[&!color=#000000]
R2091_31[&!color=#000000]
SAA14_18
SAA14_28[&!color=#000000]
SAA14_6[&!color=#000000]
SDF_15[&!color=#000000]
SMC_Paed_Ab_BL01_28[&!color=#000000]
SMC_Paed_Ab_BL01_5
SMC_Paed_Ab_BL01_6[&!color=#000000]
SSA12_18[&!color=#000000]
SSA12_28[&!color=#000000]
SSA12_6[&!color=#000000]
SSA6_5
SSA6_6[&!color=#000000]
SSMA17_18[&!color=#000000]
SSMA17_28[&!color=#000000]
TCDC-AB0715_5
TCDC-AB0715_6
TYTH-1_14[&!color=#000000]
TYTH-1_5
TYTH-1_6[&!color=#000000]
USA15_15[&!color=#000000]
USA15_16[&!color=#000000]
USA2_5
USA2_6[&!color=#000000]
WCHAB005078_15[&!color=#000000]
WCHAB005078_16[&!color=#000000]
WCHAB005078_17[&!color=#000000]
WCHAB005133_5
WCHAB005133_6[&!color=#000000]
WKA02_11[&!color=#000000]
WKA02_12[&!color=#000000]
WKA02_13[&!color=#000000]
XDR-BJ83_5
XDR-BJ83_6[&!color=#000000]
XH386_18[&!color=#000000]
XH386_5
XH386_6[&!color=#000000]
XH856_13[&!color=#000000]
XH856_18[&!color=#000000]

XH856_5
XH856_6[&!color=#000000]
XH857_6[&!color=#000000]
XH858_11[&!color=#000000]
XH858_13[&!color=#000000]
XH859_6[&!color=#000000]
XH860_6[&!color=#000000]
YU-R612_28[&!color=#000000]
YU-R612_5
YU-R612_6[&!color=#000000]
ZW85-1_17[&!color=#000000]
ZW85-1_19[&!color=#000000]
ZW85-1_20[&!color=#000000]
ab736_10[&!color=#000000]
ab736_7[&!color=#000000]
ab736_9[&!color=#000000]
;
end;

begin trees;
tree tree_1 = [&R] (ab736_10:2.0E-6,ATCC19606_10:2.0E-6,(((((((((AF-
401_30:1.7162,(((Ab04-mff_13:2.0E-6,LAC-4_13:2.0E-
6)[&Bootstrap=99]:0.005609,(((((((BJAB0715_13:0.0,R2090_13:0.0):0.0,XH856_13:0.0):0.0,
KAB02_13:0.0):0.0,KAB05_13:0.0):0.0,XH858_13:0.0):0.0,MDR-TJ_13:0.0):2.0E-
6,D1279779_13:2.0E-6)[&Bootstrap=46]:2.0E-6,KAB06_13:2.0E-6)[&Bootstrap=77]:2.0E-
6)[&Bootstrap=67]:2.0E-
6,WKA02_13:0.002799)[&Bootstrap=100]:2.745606)[&Bootstrap=77]:0.288346,(ZW85-
1_20:0.536526,(((D1279779_12:2.0E-6,R2090_12:2.0E-
6)[&Bootstrap=99]:0.006529,(((AR_0088_12:0.0,R2091_12:0.0):0.0,HWBA8_12:0.0):2.0E-
6,'CIP70.10_12':2.0E-6)[&Bootstrap=95]:2.0E-6)[&Bootstrap=88]:2.0E-6,(Ab04-
mff_12:0.001085,(LAC-4_12:2.0E-6,WKA02_12:2.0E-6)[&Bootstrap=95]:2.0E-
6)[&Bootstrap=97]:0.001084)[&Bootstrap=94]:0.371231)[&Bootstrap=100]:1.844913)[&Boo
tstrap=63]:0.358693,(((3207_1:0.0,HWAB8_1:0.0):2.0E-6,AR_0088_1:2.0E-
6)[&Bootstrap=90]:2.0E-6,(AB042_1:2.0E-6,ATCC17978-mff_1:2.0E-
6)[&Bootstrap=99]:0.003827)[&Bootstrap=100]:1.973745)[&Bootstrap=45]:0.187788,(((((AB
030_24:0.0,AF-401_24:0.0):0.0,AR_0101_24:0.0):2.0E-6,AR_0063_24:2.0E-
6)[&Bootstrap=51]:2.0E-6,AbH12O-A2_24:2.0E-
6)[&Bootstrap=99]:2.416122,AB031_25:3.088864)[&Bootstrap=83]:0.636133)[&Bootstrap=
53]:0.244902,(((((Ab04-mff_11:0.0,XH858_11:0.0):0.0,WKA02_11:0.0):0.0,LAC-
4_11:0.0):2.0E-6,BJAB0715_11:2.0E-
6)[&Bootstrap=99]:2.180093,6200_21:1.483988)[&Bootstrap=72]:1.650951)[&Bootstrap=35
]:0.330894,(((((((ATCC17978-mff_2:0.0,R2091_2:0.0):2.0E-6,'CIP70.10_2':2.0E-
6)[&Bootstrap=100]:1.589527,(((((AB030_22:0.0,AR_0101_22:0.0):0.0,IOMTU433_22:0.0):0.
0,AR_0063_22:0.0):0.0,AF-401_22:0.0):2.0E-6,AbH12O-A2_22:2.0E-
6)[&Bootstrap=100]:2.870637)[&Bootstrap=37]:1.269266,((((((((A1296_8:2.0E-
6,AR_0056_8:2.0E-6)[&Bootstrap=91]:2.0E-6,KAB03_8:2.0E-6)[&Bootstrap=40]:2.0E-
6,(1656-2_8:2.0E-6,DU202_8:2.0E-6)[&Bootstrap=99]:2.0E-6)[&Bootstrap=55]:3.0E-

6,NCGM237_8:2.0E-6)[&Bootstrap=49]:3.0E-6,R2090_8:2.0E-6)[&Bootstrap=57]:2.0E-6,ACICU_8:2.0E-6)[&Bootstrap=59]:2.0E-6,D1279779_8:2.0E-6)[&Bootstrap=77]:2.0E-6,AbPK1_8:0.001479)[&Bootstrap=86]:1.166557)[&Bootstrap=57]:0.21977,AR_0088_32:2.356108)[&Bootstrap=48]:0.319813,(((AB042_3:2.0E-6,ATCC17978-mff_3:2.0E-6)[&Bootstrap=100]:3.946468,(((((((((((((((((((((((((15A5_18:0.0,XH386_18:0.0):0.0,KAB07_18:0.0):0.0,SAA14_18:0.0):0.0,CBA7_18:0.0):0.0,Ab4568_18:0.0):0.0,KAB06_18:0.0):0.0,AB07_18:0.0):0.0,AF-673_18:0.0):0.0,AR_0102_18:0.0):0.0,KAB05_18:0.0):0.0,Ab4977_18:0.0):0.0,KAB08_18:0.0):0.0,HRAB-85_18:0.0):0.0,XH856_18:0.0):0.0,AC29_18:0.0):0.0,CMC-CR-MDR-Ab66_18:0.0):0.0,KAB04_18:0.0):0.0,CMC-CR-MDR-Ab4_18:0.0):0.0,SSA12_18:0.0):0.0,JBA13_18:0.0):0.0,AC30_18:0.0):0.0,AYP-A2_18:0.0):0.0,KAB02_18:0.0):0.0,SSMA17_18:0.0):2.0E-6,3027STDY5784958_18:2.0E-6)[&Bootstrap=99]:0.184693)[&Bootstrap=53]:0.156116,(3207_4:0.00124,ATCC17978-mff_4:2.0E-6)[&Bootstrap=100]:1.12041)[&Bootstrap=95]:0.959495)[&Bootstrap=34]:0.17284,ZW85-1_19:3.019437)[&Bootstrap=41]:0.069252)[&Bootstrap=13]:0.211945,((('CIP70.10_31':2.0E-6,R2091_31:2.0E-6)[&Bootstrap=100]:2.006028,(((((((((((((((((((((((((((((15A34_5:0.0,WCHAB005133_5:0.0):0.0,AF-673_5:0.0):0.0,TYTH-1_5:0.0):0.0,XDR-BJ83_5:0.0):0.0,AR_0056_5:0.0):0.0,USA2_5:0.0):0.0,XH386_5:0.0):0.0,AR_0102_5:0.0):0.0,CMC-CR-MDR-Ab66_5:0.0):0.0,MDR-TJ_5:0.0):0.0,BJAB07104_5:0.0):0.0,YU-R612_5:0.0):0.0,CMC-CR-MDR-Ab4_5:0.0):0.0,MDR-ZJ06_5:0.0):0.0,3027STDY5784958_5:0.0):0.0,NCGM237_5:0.0):0.0,BJAB0868_5:0.0):0.0,Ab4977_5:0.0):0.0,CMC-MDR-Ab59_5:0.0):0.0,Ab4653_5:0.0):0.0,HRAB-85_5:0.0):0.0,ACICU_5:0.0):0.0,SMC_Paed_Ab_BL01_5:0.0):0.0,KBN10P02143_5:0.0):0.0,TCDC-AB0715_5:0.0):0.0,XH856_5:0.0):0.0,SSA6_5:0.0):2.0E-6,15A5_5:2.0E-6)[&Bootstrap=69]:2.0E-6,Ab4568_5:2.0E-6)[&Bootstrap=99]:1.970199,((((((((((((((((((6200_15:0.0,AB307-0294_15:0.0):0.0,A388_15:0.0):0.0,AB030_15:0.0):0.0,AB5075-UW_15:0.0):0.0,AbH12O-A2_15:0.0):0.0,WCHAB005078_15:0.0):0.0,D36_15:0.0):0.0,USA15_15:0.0):0.0,AR_0083_15:0.0):0.0,AR_0063_15:0.0):0.0,AR_0101_15:0.0):0.0,IOMTU433_15:0.0):0.0,AF-401_15:0.0):0.0,A85_15:0.0):0.0,AB0057_15:0.0):0.0,AR_0078_15:0.0):2.0E-6,A1_15:2.0E-6)[&Bootstrap=92]:2.0E-6,SDF_15:0.005707)[&Bootstrap=100]:2.107152)[&Bootstrap=84]:0.280492)[&Bootstrap=60]:0.742627,(((ab736_7:0.0,ATCC19606_7:0.0):2.0E-6,ACICU_7:2.0E-6)[&Bootstrap=100]:2.763117,(((15A34_6:0.0,MDR-ZJ06_6:0.0):0.0,AF-673_6:0.0):0.0,KAB07_6:0.0):0.0,KAB06_6:0.0):0.0,XH856_6:0.0):0.0,YU-R612_6:0.0):0.0,Ab4568_6:0.0):0.0,AYP-A2_6:0.0):0.0,AbPK1_6:0.0):0.0,SAA14_6:0.0):0.0,DU202_6:0.0):0.0,BJAB0868_6:0.0):0.0,SMC_Paed_Ab_BL01_6:0.0):0.0,Ab4653_6:0.0):0.0,BJAB07104_6:0.0):0.0,3027STDY5784958_6:0.0):0.0,Ab4977_6:0.0):0.0,TCDC-AB0715_6:0.0):0.0,AB34299_6:0.0):0.0,XH860_6:0.0):0.0,KAB04_6:0.0):0.0,KAB05_6:0.0):0.0,CMC-CR-MDR-Ab4_6:0.0):0.0,KBN10P02143_6:0.0):0.0,JBA13_6:0.0):0.0,AB07_6:0.0):0.0,AC29_6:0.0):0.0,XH857_6:0.0):0.0,KAB02_6:0.0):0.0,USA2_6:0.0):0.0,NCGM237_6:0.0):0.0,XH859_6:0.0):0.0,KAB03_6:0.0):0.0,ACICU_6:0.0):0.0,SSA12_6:0.0):0.0,XDR-BJ83_6:0.0):0.0,CBA7_6:0.0):0.0,CMC-CR-MDR-

```
Ab66_6:0.0):0.0,KAB01_6:0.0):0.0,AC30_6:0.0):0.0,1656-2_6:0.0):0.0,MDR-
TJ_6:0.0):0.0,WCHAB005133_6:0.0):0.0,HRAB-85_6:0.0):0.0,CMC-MDR-
Ab59_6:0.0):0.0,TYTH-
1_6:0.0):0.0,XH386_6:0.0):0.0,SSA6_6:0.0):0.0,KAB08_6:0.0):0.0,AR_0056_6:0.0):2.0E-
6,15A5_6:2.0E-
6)[&Bootstrap=99]:0.796621)[&Bootstrap=61]:0.455455)[&Bootstrap=15]:0.158425)[&Boot
strap=17]:0.338132,((((((6200_17:2.0E-6,ZW85-1_17:2.0E-6)[&Bootstrap=97]:3.0E-
6,15A34_23:1.820821)[&Bootstrap=77]:0.008632,((((((((A1_17:0.0,AR_0083_17:0.0):0.0,AB
307-0294_17:0.0):0.0,D36_17:0.0):0.0,AB5075-
UW_17:0.0):0.0,WCHAB005078_17:0.0):0.0,AYE_17:0.0):0.0,A85_17:0.0):2.0E-
6,A388_17:2.0E-6)[&Bootstrap=89]:2.0E-
6)[&Bootstrap=96]:0.668719,(AR_0078_29:0.359974,((((AR_0088_14:0.0,R2091_14:0.0):0.0
,HWBA8_14:0.0):2.0E-6,'CIP70.10_14':2.0E-6)[&Bootstrap=100]:0.010277,TYTH-
1_14:0.009855)[&Bootstrap=100]:0.310986)[&Bootstrap=100]:1.688187)[&Bootstrap=92]:0
.292993,AB031_27:1.384105)[&Bootstrap=75]:0.011963,AB031_26:2.993381)[&Bootstrap=
64]:0.523892)[&Bootstrap=51]:0.384815,((ab736_9:2.0E-6,ATCC19606_9:2.0E-
6)[&Bootstrap=100]:1.936605,(((((((((((15A5_28:0.0,KBN10P02143_28:0.0):0.0,KAB04_28:0
.0):0.0,KAB07_28:0.0):0.0,SSMA17_28:0.0):0.0,KAB08_28:0.0):0.0,SSA12_28:0.0):0.0,Ab497
7_28:0.0):0.0,SMC_Paed_Ab_BL01_28:0.0):0.0,SAA14_28:0.0):0.0,JBA13_28:0.0):0.0,YU-
R612_28:0.0):2.0E-6,Ab4568_28:2.0E-
6)[&Bootstrap=100]:1.536672)[&Bootstrap=90]:0.335337)[&Bootstrap=52]:0.191294,(((((((((
A1_16:0.0,WCHAB005078_16:0.0):0.0,AB307-
0294_16:0.0):0.0,AB0057_16:0.0):0.0,AR_0083_16:0.0):0.0,AB5075-
UW_16:0.0):0.0,A85_16:0.0):0.0,D36_16:0.0):0.0,USA15_16:0.0):2.0E-6,A388_16:2.0E-
6)[&Bootstrap=100]:1.978003)[&Bootstrap=100]:2.560129);
end;

begin figtree;
	set appearance.backgroundColorAttribute="Default";
	set appearance.backgroundColour=#ffffff;
	set appearance.branchColorAttribute="User selection";
	set appearance.branchColorGradient=false;
	set appearance.branchLineWidth=2.0;
	set appearance.branchMinLineWidth=0.0;
	set appearance.branchWidthAttribute="Fixed";
	set appearance.foregroundColour=#000000;
	set appearance.hilightingGradient=false;
	set appearance.selectionColour=#2d3680;
	set branchLabels.colorAttribute="User selection";
	set branchLabels.displayAttribute="Branch times";
	set branchLabels.fontName="Adobe Devanagari";
	set branchLabels.fontSize=8;
	set branchLabels.fontStyle=0;
	set branchLabels.isShown=false;
	set branchLabels.significantDigits=4;
	set layout.expansion=496;
	set layout.layoutType="RECTILINEAR";
```

```
set layout.zoom=0;
set legend.attribute="Bootstrap";
set legend.fontSize=10.0;
set legend.isShown=false;
set legend.significantDigits=4;
set nodeBars.barWidth=4.0;
set nodeBars.displayAttribute=null;
set nodeBars.isShown=false;
set nodeLabels.colorAttribute="User selection";
set nodeLabels.displayAttribute="Bootstrap";
set nodeLabels.fontName="Adobe Devanagari";
set nodeLabels.fontSize=8;
set nodeLabels.fontStyle=0;
set nodeLabels.isShown=true;
set nodeLabels.significantDigits=4;
set nodeShapeExternal.colourAttribute="User selection";
set nodeShapeExternal.isShown=true;
set nodeShapeExternal.minSize=10.0;
set nodeShapeExternal.scaleType=Width;
set nodeShapeExternal.shapeType=Circle;
set nodeShapeExternal.size=5.0;
set nodeShapeExternal.sizeAttribute="Fixed";
set nodeShapeInternal.colourAttribute="User selection";
set nodeShapeInternal.isShown=false;
set nodeShapeInternal.minSize=10.0;
set nodeShapeInternal.scaleType=Width;
set nodeShapeInternal.shapeType=Circle;
set nodeShapeInternal.size=4.0;
set nodeShapeInternal.sizeAttribute="Fixed";
set polarLayout.alignTipLabels=false;
set polarLayout.angularRange=0;
set polarLayout.rootAngle=0;
set polarLayout.rootLength=100;
set polarLayout.showRoot=true;
set radialLayout.spread=0.0;
set rectilinearLayout.alignTipLabels=false;
set rectilinearLayout.curvature=0;
set rectilinearLayout.rootLength=100;
set scale.offsetAge=0.0;
set scale.rootAge=1.0;
set scale.scaleFactor=1.0;
set scale.scaleRoot=false;
set scaleAxis.automaticScale=true;
set scaleAxis.fontSize=8.0;
set scaleAxis.isShown=false;
set scaleAxis.lineWidth=1.0;
set scaleAxis.majorTicks=1.0;
```

```
	set scaleAxis.minorTicks=0.5;
	set scaleAxis.origin=0.0;
	set scaleAxis.reverseAxis=false;
	set scaleAxis.showGrid=true;
	set scaleBar.automaticScale=true;
	set scaleBar.fontSize=10.0;
	set scaleBar.isShown=true;
	set scaleBar.lineWidth=1.0;
	set scaleBar.scaleRange=0.0;
	set tipLabels.colorAttribute="User selection";
	set tipLabels.displayAttribute="Names";
	set tipLabels.fontName="Adobe Devanagari";
	set tipLabels.fontSize=8;
	set tipLabels.fontStyle=0;
	set tipLabels.isShown=true;
	set tipLabels.significantDigits=4;
	set trees.order=false;
	set trees.orderType="increasing";
	set trees.rooting=true;
	set trees.rootingType="Midpoint";
	set trees.transform=false;
	set trees.transformType="cladogram";
end;
```

Supplementary File S3b

```
#NEXUS
begin taxa;
	dimensions ntax=287;
	taxlabels
	15A34_23[&!color=#000000]
	15A34_5
	15A34_6[&!color=#000000]
	15A5_18[&!color=#000000]
	15A5_28[&!color=#000000]
	15A5_5
	15A5_6[&!color=#000000]
	1656-2_6[&!color=#000000]
	1656-2_8[&!color=#000000]
	3027STDY5784958_18[&!color=#000000]
	3027STDY5784958_5
	3027STDY5784958_6[&!color=#000000]
	3207_1[&!color=#000000]
	3207_4[&!color=#000000]
	6200_15[&!color=#000000]
	6200_17[&!color=#000000]
	6200_21[&!color=#000000]
	A1296_8[&!color=#000000]
```

A1_15[&!color=#000000]
A1_16[&!color=#000000]
A1_17[&!color=#000000]
A388_15[&!color=#000000]
A388_16[&!color=#000000]
A388_17[&!color=#000000]
A85_15[&!color=#000000]
A85_16[&!color=#000000]
A85_17[&!color=#000000]
AB0057_15[&!color=#000000]
AB0057_16
AB030_15[&!color=#000000]
AB030_22[&!color=#000000]
AB030_24[&!color=#000000]
AB031_25[&!color=#000000]
AB031_26[&!color=#000000]
AB031_27[&!color=#000000]
AB042_1[&!color=#000000]
AB042_3
AB07_18[&!color=#000000]
AB07_6[&!color=#000000]
AB307-0294_15[&!color=#000000]
AB307-0294_16[&!color=#000000]
AB307-0294_17[&!color=#000000]
AB34299_6[&!color=#000000]
AB5075-UW_15[&!color=#000000]
AB5075-UW_16[&!color=#000000]
AB5075-UW_17[&!color=#000000]
AC29_18[&!color=#000000]
AC29_6[&!color=#000000]
AC30_18[&!color=#000000]
AC30_6[&!color=#000000]
ACICU_5
ACICU_6[&!color=#000000]
ACICU_7[&!color=#000000]
ACICU_8[&!color=#000000]
AF-401_15[&!color=#000000]
AF-401_22[&!color=#000000]
AF-401_24[&!color=#000000]
AF-401_30[&!color=#000000]
AF-673_18[&!color=#000000]
AF-673_5
AF-673_6[&!color=#000000]
AR_0056_5
AR_0056_6[&!color=#000000]
AR_0056_8[&!color=#000000]
AR_0063_15[&!color=#000000]

AR_0063_22[&!color=#000000]
AR_0063_24[&!color=#000000]
AR_0078_15[&!color=#000000]
AR_0078_29[&!color=#000000]
AR_0083_15[&!color=#000000]
AR_0083_16[&!color=#000000]
AR_0083_17[&!color=#000000]
AR_0088_1[&!color=#000000]
AR_0088_12[&!color=#000000]
AR_0088_14[&!color=#000000]
AR_0088_32[&!color=#000000]
AR_0101_15[&!color=#000000]
AR_0101_22[&!color=#000000]
AR_0101_24[&!color=#000000]
AR_0102_18[&!color=#000000]
AR_0102_5
ATCC17978-mff_1[&!color=#000000]
ATCC17978-mff_2[&!color=#000000]
ATCC17978-mff_3
ATCC17978-mff_4[&!color=#000000]
ATCC19606_10[&!color=#000000]
ATCC19606_7[&!color=#000000]
ATCC19606_9[&!color=#000000]
AYE_17[&!color=#000000]
AYP-A2_18[&!color=#000000]
AYP-A2_6[&!color=#000000]
Ab04-mff_11[&!color=#000000]
Ab04-mff_12[&!color=#000000]
Ab04-mff_13[&!color=#000000]
Ab4568_18[&!color=#000000]
Ab4568_28[&!color=#000000]
Ab4568_5
Ab4568_6[&!color=#000000]
Ab4653_5
Ab4653_6[&!color=#000000]
Ab4977_18[&!color=#000000]
Ab4977_28[&!color=#000000]
Ab4977_5
Ab4977_6[&!color=#000000]
AbH12O-A2_15[&!color=#000000]
AbH12O-A2_22[&!color=#000000]
AbH12O-A2_24[&!color=#000000]
AbPK1_6[&!color=#000000]
AbPK1_8[&!color=#000000]
BJAB07104_5
BJAB07104_6[&!color=#000000]
BJAB0715_11[&!color=#000000]

BJAB0715_13[&!color=#000000]
BJAB0868_5
BJAB0868_6[&!color=#000000]
CBA7_18[&!color=#000000]
CBA7_6[&!color=#000000]
'CIP70.10_12'[&!color=#000000]
'CIP70.10_14'[&!color=#000000]
'CIP70.10_2'[&!color=#000000]
'CIP70.10_31'[&!color=#000000]
CMC-CR-MDR-Ab4_18[&!color=#000000]
CMC-CR-MDR-Ab4_5
CMC-CR-MDR-Ab4_6[&!color=#000000]
CMC-CR-MDR-Ab66_18[&!color=#000000]
CMC-CR-MDR-Ab66_5
CMC-CR-MDR-Ab66_6[&!color=#000000]
CMC-MDR-Ab59_5
CMC-MDR-Ab59_6[&!color=#000000]
D1279779_12
D1279779_13[&!color=#000000]
D1279779_8
D36_15[&!color=#000000]
D36_16[&!color=#000000]
D36_17[&!color=#000000]
DU202_6[&!color=#000000]
DU202_8[&!color=#000000]
EvpP
HRAB-85_18[&!color=#000000]
HRAB-85_5
HRAB-85_6[&!color=#000000]
HWAB8_1[&!color=#000000]
HWBA8_12[&!color=#000000]
HWBA8_14[&!color=#000000]
Hcp-ET1
IOMTU433_15[&!color=#000000]
IOMTU433_22[&!color=#000000]
JBA13_18[&!color=#000000]
JBA13_28[&!color=#000000]
JBA13_6[&!color=#000000]
KAB01_6[&!color=#000000]
KAB02_13[&!color=#000000]
KAB02_18[&!color=#000000]
KAB02_6[&!color=#000000]
KAB03_6[&!color=#000000]
KAB03_8[&!color=#000000]
KAB04_18[&!color=#000000]
KAB04_28[&!color=#000000]
KAB04_6[&!color=#000000]

KAB05_13[&!color=#000000]
KAB05_18[&!color=#000000]
KAB05_6[&!color=#000000]
KAB06_13[&!color=#000000]
KAB06_18[&!color=#000000]
KAB06_6[&!color=#000000]
KAB07_18[&!color=#000000]
KAB07_28[&!color=#000000]
KAB07_6[&!color=#000000]
KAB08_18[&!color=#000000]
KAB08_28[&!color=#000000]
KAB08_6[&!color=#000000]
KBN10P02143_28[&!color=#000000]
KBN10P02143_5
KBN10P02143_6[&!color=#000000]
KatN
LAC-4_11[&!color=#000000]
LAC-4_12[&!color=#000000]
LAC-4_13[&!color=#000000]
MDR-TJ_13[&!color=#000000]
MDR-TJ_5
MDR-TJ_6[&!color=#000000]
MDR-ZJ06_5
MDR-ZJ06_6[&!color=#000000]
NCGM237_5
NCGM237_6[&!color=#000000]
NCGM237_8
PldB
R2090_12
R2090_13[&!color=#000000]
R2090_8[&!color=#000000]
R2091_12[&!color=#000000]
R2091_14[&!color=#000000]
R2091_2[&!color=#000000]
R2091_31[&!color=#000000]
Rhs1
Rhs2
RhsA
RhsB
SAA14_18
SAA14_28[&!color=#000000]
SAA14_6[&!color=#000000]
SDF_15[&!color=#000000]
SMC_Paed_Ab_BL01_28[&!color=#000000]
SMC_Paed_Ab_BL01_5
SMC_Paed_Ab_BL01_6[&!color=#000000]
SSA12_18[&!color=#000000]

SSA12_28[&!color=#000000]
SSA12_6[&!color=#000000]
SSA6_5
SSA6_6[&!color=#000000]
SSMA17_18[&!color=#000000]
SSMA17_28[&!color=#000000]
TCDC-AB0715_5
TCDC-AB0715_6
TYTH-1_14[&!color=#000000]
TYTH-1_5
TYTH-1_6[&!color=#000000]
Tde1
Tde2
TecA
Tfe1
Tfe2
Tge2
Tke10
Tke2
Tke4
Tke6
Tke7
Tke9
Tle1Bt
Tle1Ec
Tle2
Tle3
Tle4
Tle5
Tse1
Tse2
Tse3
Tse4
Tse5
Tse6
TseF
TseH
TseM
TseT
USA15_15[&!color=#000000]
USA15_16[&!color=#000000]
USA2_5
USA2_6[&!color=#000000]
VasX
VgrG-1
VgrG-3
VgrG-5

VgrG1
VgrG2b
WCHAB005078_15[&!color=#000000]
WCHAB005078_16[&!color=#000000]
WCHAB005078_17[&!color=#000000]
WCHAB005133_5
WCHAB005133_6[&!color=#000000]
WKA02_11[&!color=#000000]
WKA02_12[&!color=#000000]
WKA02_13[&!color=#000000]
XDR-BJ83_5
XDR-BJ83_6[&!color=#000000]
XH386_18[&!color=#000000]
XH386_5
XH386_6[&!color=#000000]
XH856_13[&!color=#000000]
XH856_18[&!color=#000000]
XH856_5
XH856_6[&!color=#000000]
XH857_6[&!color=#000000]
XH858_11[&!color=#000000]
XH858_13[&!color=#000000]
XH859_6[&!color=#000000]
XH860_6[&!color=#000000]
YU-R612_28[&!color=#000000]
YU-R612_5
YU-R612_6[&!color=#000000]
YezP
ZW85-1_17[&!color=#000000]
ZW85-1_19[&!color=#000000]
ZW85-1_20[&!color=#000000]
ab736_10[&!color=#000000]
ab736_7[&!color=#000000]
ab736_9[&!color=#000000]
;
end;

begin trees;
tree tree_1 = [&R] (Ab04-mff_13:2.0E-6,LAC-4_13:2.0E-6,((WKA02_13:2.0E-6,((((((((((((15A34_23:2.112464,(6200_17:2.0E-6,ZW85-1_17:2.0E-6)[&Bootstrap=96]:2.0E-6)[&Bootstrap=98]:0.004665,((((((((A1_17:0.0,WCHAB005078_17:0.0):0.0,A85_17:0.0):0.0,AR_0083_17:0.0):0.0,AB5075-UW_17:0.0):0.0,AYE_17:0.0):0.0,AB307-0294_17:0.0):0.0,D36_17:0.0):2.0E-6,A388_17:2.0E-6)[&Bootstrap=98]:0.00388)[&Bootstrap=96]:0.770015,(ab736_9:2.0E-6,ATCC19606_9:2.0E-6)[&Bootstrap=100]:3.147266)[&Bootstrap=95]:0.38741,(AR_0078_29:0.349061,((((AR_0088_14:0.0,R2091_14:0.0):0.0,HWBA8_14:0.0):2.0E-6,'CIP70.10_14':2.0E-6)[&Bootstrap=100]:0.010453,TYTH-

1_14:0.009875)[&Bootstrap=100]:0.356083)[&Bootstrap=100]:1.85091)[&Bootstrap=90]:0.504709,(((((Tfe1:2.121069,Tke9:2.072308)[&Bootstrap=86]:0.406186,(((AB030_24:0.0,AF-401_24:0.0):0.0,AR_0101_24:0.0):2.0E-6,(AbH12O-A2_24:2.0E-6,AR_0063_24:2.0E-6)[&Bootstrap=51]:2.0E-6)[&Bootstrap=100]:3.492017)[&Bootstrap=80]:0.385533,Tse2:1.684289)[&Bootstrap=50]:0.326974,((Tge2:2.163176,Tse1:2.245637)[&Bootstrap=72]:0.263136,(TseT:2.998452,((A1296_8:3.0E-6,((((((1656-2_8:2.0E-6,DU202_8:2.0E-6)[&Bootstrap=100]:3.0E-6,NCGM237_8:2.0E-6)[&Bootstrap=98]:2.0E-6,R2090_8:2.0E-6)[&Bootstrap=81]:2.0E-6,AbPK1_8:0.001504)[&Bootstrap=85]:2.0E-6,D1279779_8:2.0E-6)[&Bootstrap=77]:2.0E-6,ACICU_8:2.0E-6)[&Bootstrap=84]:0.002593,AR_0056_8:2.0E-6)[&Bootstrap=43]:3.0E-6)[&Bootstrap=46]:0.008878,KAB03_8:0.006443)[&Bootstrap=98]:1.489078)[&Bootstrap=90]:0.606589)[&Bootstrap=43]:0.127849)[&Bootstrap=58]:0.166513,((VasX:2.889966,Tle2:3.089384)[&Bootstrap=75]:0.317949,(PldB:2.692705,AB031_27:1.062622)[&Bootstrap=94]:1.226532)[&Bootstrap=67]:0.494963)[&Bootstrap=71]:0.167143)[&Bootstrap=49]:0.13914,(RhsA:2.021507,(Tde1:2.525794,TecA:1.527218)[&Bootstrap=91]:0.840557)[&Bootstrap=59]:0.498029)[&Bootstrap=48]:0.228818,((((((AB031_25:3.257362,Tse6:2.320016)[&Bootstrap=45]:3.0E-6,Tse4:2.479647)[&Bootstrap=68]:0.31121,(Tke10:2.58826,(((((AB030_22:0.0,AF-401_22:0.0):0.0,AR_0063_22:0.0):0.0,AR_0101_22:0.0):0.0,IOMTU433_22:0.0):2.0E-6,AbH12O-A2_22:2.0E-6)[&Bootstrap=100]:1.540157)[&Bootstrap=80]:0.788953)[&Bootstrap=54]:0.236098,((Tle1Ec:2.397375,((((((((((((((((((((((((((((15A34_5:0.0,XH386_5:0.0):0.0,XDR-BJ83_5:0.0):0.0,NCGM237_5:0.0):0.0,TYTH-1_5:0.0):0.0,ACICU_5:0.0):0.0,SMC_Paed_Ab_BL01_5:0.0):0.0,AR_0102_5:0.0):0.0,USA2_5:0.0):0.0,TCDC-AB0715_5:0.0):0.0,SSA6_5:0.0):0.0,XH856_5:0.0):0.0,BJAB07104_5:0.0):0.0,BJAB0868_5:0.0):0.0,YU-R612_5:0.0):0.0,AF-673_5:0.0):0.0,WCHAB005133_5:0.0):0.0,CMC-MDR-Ab59_5:0.0):0.0,CMC-CR-MDR-Ab4_5:0.0):0.0,HRAB-85_5:0.0):0.0,CMC-CR-MDR-Ab66_5:0.0):0.0,AR_0056_5:0.0):0.0,3027STDY5784958_5:0.0):0.0,MDR-ZJ06_5:0.0):0.0,KBN10P02143_5:0.0):0.0,MDR-TJ_5:0.0):0.0,Ab4977_5:0.0):0.0,Ab4653_5:0.0):2.0E-6,15A5_5:2.0E-6)[&Bootstrap=83]:2.0E-6,Ab4568_5:2.0E-6)[&Bootstrap=100]:1.535228)[&Bootstrap=87]:0.738541,(KatN:2.529631,Tde2:2.415826)[&Bootstrap=87]:0.247282)[&Bootstrap=72]:0.632109)[&Bootstrap=52]:0.164307,(((Tle1Bt:2.4402,Tse3:2.144093)[&Bootstrap=53]:0.055106,TseH:2.821092)[&Bootstrap=92]:0.764342,(VgrG-5:2.97599,(VgrG2b:1.962874,(VgrG1:0.51364,(VgrG-1:0.464796,VgrG-3:0.496909)[&Bootstrap=94]:0.344669)[&Bootstrap=87]:0.436165)[&Bootstrap=99]:1.115995)[&Bootstrap=95]:0.458393)[&Bootstrap=54]:0.417721)[&Bootstrap=31]:0.210972,(Hcp-ET1:2.783099,(AB031_26:2.061922,((((((((((((((((6200_15:0.0,AR_0101_15:0.0):0.0,AR_0083_15:0.0):0.0,AB0057_15:0.0):0.0,AR_0078_15:0.0):0.0,A85_15:0.0):0.0,USA15_15:0.0):0.0,AB307-0294_15:0.0):0.0,IOMTU433_15:0.0):0.0,AF-401_15:0.0):0.0,AB5075-UW_15:0.0):0.0,D36_15:0.0):0.0,WCHAB005078_15:0.0):0.0,AbH12O-A2_15:0.0):0.0,AR_0063_15:0.0):0.0,AB030_15:0.0):0.0,A388_15:0.0):2.0E-6,A1_15:2.0E-6)[&Bootstrap=96]:3.0E-6,SDF_15:0.005521)[&Bootstrap=100]:1.821566)[&Bootstrap=40]:0.188802)[&Bootstrap=32]:0.642754)[&Bootstrap=19]:2.0E-6)[&Bootstrap=31]:0.240972,(((((((Ab04-mff_11:0.0,XH858_11:0.0):0.0,LAC-4_11:0.0):0.0,WKA02_11:0.0):2.0E-6,BJAB0715_11:2.0E-6)[&Bootstrap=100]:2.462589,TseM:1.868024)[&Bootstrap=38]:0.828897,(((AF-

401_30:1.337462,(((3207_1:0.0,HWAB8_1:0.0):2.0E-6,AR_0088_1:2.0E-
6)[&Bootstrap=99]:3.0E-6,(AB042_1:2.0E-6,ATCC17978-mff_1:2.0E-
6)[&Bootstrap=100]:0.003831)[&Bootstrap=100]:1.506334)[&Bootstrap=97]:1.236418,(((((((
((((A1_16:0.0,AB5075-UW_16:0.0):0.0,AB307-
0294_16:0.0):0.0,AR_0083_16:0.0):0.0,D36_16:0.0):0.0,USA15_16:0.0):0.0,AB0057_16:0.0):
0.0,WCHAB005078_16:0.0):0.0,A85_16:0.0):2.0E-6,A388_16:2.0E-
6)[&Bootstrap=100]:1.261397,Tle3:2.474339)[&Bootstrap=96]:0.688638,Tke7:2.437455)[&B
ootstrap=80]:0.433561)[&Bootstrap=44]:0.378625,(((((((((((((15A5_28:0.0,KAB08_28:0.0):0.
0,KAB07_28:0.0):0.0,SSMA17_28:0.0):0.0,SMC_Paed_Ab_BL01_28:0.0):0.0,KBN10P02143_2
8:0.0):0.0,SAA14_28:0.0):0.0,YU-
R612_28:0.0):0.0,Ab4977_28:0.0):0.0,JBA13_28:0.0):0.0,SSA12_28:0.0):0.0,KAB04_28:0.0):2
.0E-6,Ab4568_28:2.0E-
6)[&Bootstrap=100]:0.506656,(Tke4:2.150239,Tle5:2.945455)[&Bootstrap=85]:0.809138)[&
Bootstrap=75]:0.251209)[&Bootstrap=44]:0.182357)[&Bootstrap=25]:0.089128,(((((AR_008
8_32:2.985509,('CIP70.10_31':2.0E-6,R2091_31:2.0E-
6)[&Bootstrap=100]:1.847596)[&Bootstrap=93]:0.66689,Tke6:2.852963)[&Bootstrap=59]:0.
211969,((Rhs2:2.737172,Rhs1:1.832725)[&Bootstrap=53]:3.0E-
6,EvpP:2.362961)[&Bootstrap=67]:0.472445)[&Bootstrap=32]:2.0E-
6,Tse5:2.180239)[&Bootstrap=59]:0.247756,(TseF:1.939889,(ab736_10:2.0E-
6,ATCC19606_10:2.0E-
6)[&Bootstrap=100]:2.713084)[&Bootstrap=77]:0.377851)[&Bootstrap=56]:0.313217)[&Boo
tstrap=32]:0.170357)[&Bootstrap=65]:0.209751,(((RhsB:1.802948,((((((((((((((((((((((((((((((((((
(((((((((((((((((15A34_6:0.0,XH857_6:0.0):0.0,CMC-CR-MDR-
Ab66_6:0.0):0.0,SSA12_6:0.0):0.0,AR_0056_6:0.0):0.0,YU-
R612_6:0.0):0.0,KAB02_6:0.0):0.0,KBN10P02143_6:0.0):0.0,ACICU_6:0.0):0.0,KAB01_6:0.0):
0.0,XH860_6:0.0):0.0,XH859_6:0.0):0.0,WCHAB005133_6:0.0):0.0,Ab4568_6:0.0):0.0,Ab465
3_6:0.0):0.0,SMC_Paed_Ab_BL01_6:0.0):0.0,3027STDY5784958_6:0.0):0.0,KAB03_6:0.0):0.0
,SAA14_6:0.0):0.0,AYP-A2_6:0.0):0.0,SSA6_6:0.0):0.0,TCDC-
AB0715_6:0.0):0.0,XH856_6:0.0):0.0,XH386_6:0.0):0.0,MDR-
TJ_6:0.0):0.0,AB07_6:0.0):0.0,CMC-CR-MDR-Ab4_6:0.0):0.0,TYTH-1_6:0.0):0.0,CMC-MDR-
Ab59_6:0.0):0.0,AC29_6:0.0):0.0,DU202_6:0.0):0.0,KAB06_6:0.0):0.0,KAB04_6:0.0):0.0,KAB
05_6:0.0):0.0,MDR-
ZJ06_6:0.0):0.0,Ab4977_6:0.0):0.0,AB34299_6:0.0):0.0,NCGM237_6:0.0):0.0,AbPK1_6:0.0):0
.0,HRAB-85_6:0.0):0.0,KAB07_6:0.0):0.0,CBA7_6:0.0):0.0,AC30_6:0.0):0.0,1656-
2_6:0.0):0.0,BJAB0868_6:0.0):0.0,AF-
673_6:0.0):0.0,USA2_6:0.0):0.0,BJAB07104_6:0.0):0.0,KAB08_6:0.0):0.0,XDR-
BJ83_6:0.0):0.0,JBA13_6:0.0):2.0E-6,15A5_6:2.0E-
6)[&Bootstrap=100]:1.284469,((AB042_3:2.0E-6,ATCC17978-mff_3:2.0E-
6)[&Bootstrap=100]:2.071364,YezP:1.781435)[&Bootstrap=91]:0.602939)[&Bootstrap=91]:0
.73502)[&Bootstrap=83]:0.411703,((((ZW85-1_20:0.635241,(Ab04-mff_12:0.001074,(LAC-
4_12:2.0E-6,WKA02_12:2.0E-6)[&Bootstrap=99]:2.0E-
6)[&Bootstrap=100]:0.001073)[&Bootstrap=91]:3.0E-
6,(((AR_0088_12:0.0,R2091_12:0.0):0.0,HWBA8_12:0.0):2.0E-6,'CIP70.10_12':2.0E-
6)[&Bootstrap=98]:2.0E-6)[&Bootstrap=92]:0.006466,(D1279779_12:2.0E-6,R2090_12:2.0E-
6)[&Bootstrap=98]:2.0E-
6)[&Bootstrap=99]:1.571821,(6200_21:1.768538,Tke2:2.793465)[&Bootstrap=73]:0.061572
)[&Bootstrap=80]:0.330376)[&Bootstrap=72]:0.363425,(ZW85-1_19:2.398355,((ATCC17978-

mff_2:0.0,R2091_2:0.0):2.0E-6,'CIP70.10_2':2.0E-
6)[&Bootstrap=100]:2.165857)[&Bootstrap=92]:0.662517)[&Bootstrap=59]:0.148227)[&Boo
tstrap=66]:0.271628,(Tfe2:2.259414,Tle4:2.377106)[&Bootstrap=95]:0.737798)[&Bootstrap
=79]:0.427895,(((ab736_7:0.0,ATCC19606_7:0.0):2.0E-6,ACICU_7:2.0E-
6)[&Bootstrap=100]:2.869601,(3207_4:2.0E-6,ATCC17978-
mff_4:0.001228)[&Bootstrap=100]:2.428265)[&Bootstrap=71]:0.357762)[&Bootstrap=91]:0.
398949,((((((((((((((((((((((((15A5_18:0.0,AR_0102_18:0.0):0.0,HRAB-
85_18:0.0):0.0,Ab4568_18:0.0):0.0,XH386_18:0.0):0.0,KAB08_18:0.0):0.0,AYP-
A2_18:0.0):0.0,AC29_18:0.0):0.0,CMC-CR-MDR-Ab66_18:0.0):0.0,CBA7_18:0.0):0.0,CMC-CR-
MDR-
Ab4_18:0.0):0.0,Ab4977_18:0.0):0.0,KAB02_18:0.0):0.0,AB07_18:0.0):0.0,SSMA17_18:0.0):0
.0,KAB06_18:0.0):0.0,AC30_18:0.0):0.0,AF-
673_18:0.0):0.0,KAB04_18:0.0):0.0,XH856_18:0.0):0.0,SSA12_18:0.0):0.0,KAB07_18:0.0):0.0
,KAB05_18:0.0):0.0,JBA13_18:0.0):0.0,SAA14_18:0.0):2.0E-6,3027STDY5784958_18:2.0E-
6)[&Bootstrap=100]:2.23605)[&Bootstrap=100]:2.088139)[&Bootstrap=95]:0.002829,((((((((
BJAB0715_13:0.0,KAB02_13:0.0):0.0,KAB05_13:0.0):0.0,XH858_13:0.0):0.0,XH856_13:0.0):0
.0,MDR-TJ_13:0.0):0.0,R2090_13:0.0):2.0E-6,D1279779_13:2.0E-6)[&Bootstrap=35]:2.0E-
6,KAB06_13:2.0E-6)[&Bootstrap=88]:2.0E-6)[&Bootstrap=100]:0.00567);
end;

```
begin figtree;
	set appearance.backgroundColorAttribute="Default";
	set appearance.backgroundColour=#ffffff;
	set appearance.branchColorAttribute="User selection";
	set appearance.branchColorGradient=false;
	set appearance.branchLineWidth=2.0;
	set appearance.branchMinLineWidth=0.0;
	set appearance.branchWidthAttribute="Fixed";
	set appearance.foregroundColour=#000000;
	set appearance.hilightingGradient=false;
	set appearance.selectionColour=#2d3680;
	set branchLabels.colorAttribute="User selection";
	set branchLabels.displayAttribute="Branch times";
	set branchLabels.fontName="Adobe Devanagari";
	set branchLabels.fontSize=8;
	set branchLabels.fontStyle=0;
	set branchLabels.isShown=false;
	set branchLabels.significantDigits=4;
	set layout.expansion=545;
	set layout.layoutType="RECTILINEAR";
	set layout.zoom=0;
	set legend.attribute=null;
	set legend.fontSize=10.0;
	set legend.isShown=false;
	set legend.significantDigits=4;
	set nodeBars.barWidth=4.0;
	set nodeBars.displayAttribute=null;
```

```
set nodeBars.isShown=false;
set nodeLabels.colorAttribute="User selection";
set nodeLabels.displayAttribute="Bootstrap";
set nodeLabels.fontName="Adobe Devanagari";
set nodeLabels.fontSize=8;
set nodeLabels.fontStyle=0;
set nodeLabels.isShown=true;
set nodeLabels.significantDigits=4;
set nodeShapeExternal.colourAttribute=null;
set nodeShapeExternal.isShown=true;
set nodeShapeExternal.minSize=10.0;
set nodeShapeExternal.scaleType=Width;
set nodeShapeExternal.shapeType=Circle;
set nodeShapeExternal.size=5.0;
set nodeShapeExternal.sizeAttribute=null;
set nodeShapeInternal.colourAttribute=null;
set nodeShapeInternal.isShown=false;
set nodeShapeInternal.minSize=10.0;
set nodeShapeInternal.scaleType=Width;
set nodeShapeInternal.shapeType=Circle;
set nodeShapeInternal.size=4.0;
set nodeShapeInternal.sizeAttribute=null;
set polarLayout.alignTipLabels=false;
set polarLayout.angularRange=0;
set polarLayout.rootAngle=0;
set polarLayout.rootLength=100;
set polarLayout.showRoot=true;
set radialLayout.spread=0.0;
set rectilinearLayout.alignTipLabels=false;
set rectilinearLayout.curvature=0;
set rectilinearLayout.rootLength=100;
set scale.offsetAge=0.0;
set scale.rootAge=1.0;
set scale.scaleFactor=1.0;
set scale.scaleRoot=false;
set scaleAxis.automaticScale=true;
set scaleAxis.fontSize=8.0;
set scaleAxis.isShown=false;
set scaleAxis.lineWidth=1.0;
set scaleAxis.majorTicks=1.0;
set scaleAxis.minorTicks=0.5;
set scaleAxis.origin=0.0;
set scaleAxis.reverseAxis=false;
set scaleAxis.showGrid=true;
set scaleBar.automaticScale=true;
set scaleBar.fontSize=10.0;
set scaleBar.isShown=true;
```

```
	set scaleBar.lineWidth=1.0;
	set scaleBar.scaleRange=0.0;
	set tipLabels.colorAttribute="User selection";
	set tipLabels.displayAttribute="Names";
	set tipLabels.fontName="Adobe Devanagari";
	set tipLabels.fontSize=8;
	set tipLabels.fontStyle=0;
	set tipLabels.isShown=true;
	set tipLabels.significantDigits=4;
	set trees.order=false;
	set trees.orderType="increasing";
	set trees.rooting=true;
	set trees.rootingType="Midpoint";
	set trees.transform=false;
	set trees.transformType="cladogram";
end;
```

Supplementary File S3c

```
#NEXUS
begin taxa;
	dimensions ntax=127;
	taxlabels
	15A34_5
	15A34_6[&!color=#000000]
	15A5_5
	15A5_6[&!color=#000000]
	1656-2_6[&!color=#000000]
	3027STDY5784958_5
	3027STDY5784958_6[&!color=#000000]
	6200_15[&!color=#000000]
	6200_21[&!color=#000000]
	A1_15[&!color=#000000]
	A1_16[&!color=#000000]
	A388_15[&!color=#000000]
	A388_16[&!color=#000000]
	A85_15[&!color=#000000]
	A85_16[&!color=#000000]
	AB0057_15[&!color=#000000]
	AB0057_16
	AB030_15[&!color=#000000]
	AB030_22[&!color=#000000]
	AB031_26[&!color=#000000]
	AB031_27[&!color=#000000]
	AB07_6[&!color=#000000]
	AB307-0294_15[&!color=#000000]
	AB307-0294_16[&!color=#000000]
	AB34299_6[&!color=#000000]
```

AB5075-UW_15[&!color=#000000]
AB5075-UW_16[&!color=#000000]
AC29_6[&!color=#000000]
AC30_6[&!color=#000000]
ACICU_5
ACICU_6[&!color=#000000]
AF-401_15[&!color=#000000]
AF-401_22[&!color=#000000]
AF-673_5
AF-673_6[&!color=#000000]
AR_0056_5
AR_0056_6[&!color=#000000]
AR_0063_15[&!color=#000000]
AR_0063_22[&!color=#000000]
AR_0078_15[&!color=#000000]
AR_0083_15[&!color=#000000]
AR_0083_16[&!color=#000000]
AR_0101_15[&!color=#000000]
AR_0101_22[&!color=#000000]
AR_0102_5
AYP-A2_6[&!color=#000000]
Ab4568_5
Ab4568_6[&!color=#000000]
Ab4653_5
Ab4653_6[&!color=#000000]
Ab4977_5
Ab4977_6[&!color=#000000]
AbH12O-A2_15[&!color=#000000]
AbH12O-A2_22[&!color=#000000]
AbPK1_6[&!color=#000000]
BJAB07104_5
BJAB07104_6[&!color=#000000]
BJAB0868_5
BJAB0868_6[&!color=#000000]
CBA7_6[&!color=#000000]
CMC-CR-MDR-Ab4_5
CMC-CR-MDR-Ab4_6[&!color=#000000]
CMC-CR-MDR-Ab66_5
CMC-CR-MDR-Ab66_6[&!color=#000000]
CMC-MDR-Ab59_5
CMC-MDR-Ab59_6[&!color=#000000]
D36_15[&!color=#000000]
D36_16[&!color=#000000]
DU202_6[&!color=#000000]
HRAB-85_5
HRAB-85_6[&!color=#000000]
IOMTU433_15[&!color=#000000]

IOMTU433_22[&!color=#000000]
JBA13_6[&!color=#000000]
KAB01_6[&!color=#000000]
KAB02_6[&!color=#000000]
KAB03_6[&!color=#000000]
KAB04_6[&!color=#000000]
KAB05_6[&!color=#000000]
KAB06_6[&!color=#000000]
KAB07_6[&!color=#000000]
KAB08_6[&!color=#000000]
KBN10P02143_5
KBN10P02143_6[&!color=#000000]
MDR-TJ_5
MDR-TJ_6[&!color=#000000]
MDR-ZJ06_5
MDR-ZJ06_6[&!color=#000000]
NCGM237_5
NCGM237_6[&!color=#000000]
Rhs1
Rhs2
RhsA
RhsB
SAA14_6[&!color=#000000]
SDF_15[&!color=#000000]
SMC_Paed_Ab_BL01_5
SMC_Paed_Ab_BL01_6[&!color=#000000]
SSA12_6[&!color=#000000]
SSA6_5
SSA6_6[&!color=#000000]
TCDC-0715_5
TCDC-0715_6[&!color=#000000]
TYTH-1_5
TYTH-1_6[&!color=#000000]
Tke2
Tke4
Tse5
USA15_15[&!color=#000000]
USA15_16[&!color=#000000]
USA2_5
USA2_6[&!color=#000000]
WCHAB005078_15[&!color=#000000]
WCHAB005078_16[&!color=#000000]
WCHAB005133_5
WCHAB005133_6[&!color=#000000]
XDR-BJ83_5
XDR-BJ83_6[&!color=#000000]
XH386_5

XH386_6[&!color=#000000]
XH856_5
XH856_6[&!color=#000000]
XH857_6[&!color=#000000]
XH859_6[&!color=#000000]
XH860_6[&!color=#000000]
YU-R612_5
YU-R612_6[&!color=#000000]
;
end;

begin trees;
tree tree_1 = [&R] (AR_0078_15:7.2E-
4,((((((((A1_15:0.0,A85_15:0.0):0.0,D36_15:0.0):0.0,USA15_15:0.0):0.0,WCHAB005078_15:0
.0):0.0,AB0057_15:0.0):0.0,AR_0083_15:0.0):0.0,AB307-0294_15:0.0):2.0E-6,A388_15:2.0E-
6)[&Bootstrap=88]:2.0E-6,(AB5075-UW_15:7.2E-
4,(((((AB031_26:0.032279,(((((AB030_15:0.0,AR_0101_15:0.0):0.0,AR_0063_15:0.0):2.0E-
6,AF-401_15:2.0E-6)[&Bootstrap=45]:2.0E-6,AbH12O-A2_15:2.0E-
6)[&Bootstrap=82]:0.001948,((((((((AB030_22:0.0,AR_0101_22:0.0):0.0,AF-
401_22:0.0):0.0,AR_0063_22:0.0):2.0E-6,AbH12O-A2_22:2.0E-
6)[&Bootstrap=100]:0.00527,IOMTU433_22:0.00528)[&Bootstrap=99]:0.004946,AB031_27:
0.004343)[&Bootstrap=100]:0.241016,((((((((((A1_16:0.0,USA15_16:0.0):0.0,AR_0083_16:0.
0):0.0,WCHAB005078_16:0.0):0.0,AB0057_16:0.0):0.0,AB5075-
UW_16:0.0):0.0,D36_16:0.0):0.0,AB307-0294_16:0.0):2.0E-6,A388_16:2.0E-
6)[&Bootstrap=66]:2.0E-6,A85_16:6.63E-
4)[&Bootstrap=100]:0.26724,((15A34_6:0.0,KAB03_6:0
.0):0.0,ACICU_6:0.0):0.0,DU202_6:0.0):0.0,TYTH-
1_6:0.0):0.0,3027STDY5784958_6:0.0):0.0,KAB05_6:0.0):0.0,AYP-A2_6:0.0):0.0,AF-
673_6:0.0):0.0,TCDC-0715_6:0.0):0.0,MDR-
TJ_6:0.0):0.0,SSA6_6:0.0):0.0,BJAB07104_6:0.0):0.0,Ab4653_6:0.0):0.0,AR_0056_6:0.0):0.0,
KAB02_6:0.0):0.0,XH859_6:0.0):0.0,KAB07_6:0.0):0.0,CMC-CR-MDR-
Ab4_6:0.0):0.0,XH856_6:0.0):0.0,SAA14_6:0.0):0.0,AC29_6:0.0):0.0,BJAB0868_6:0.0):0.0,CM
C-MDR-Ab59_6:0.0):0.0,XH860_6:0.0):0.0,KAB01_6:0.0):0.0,XDR-
BJ83_6:0.0):0.0,SMC_Paed_Ab_BL01_6:0.0):0.0,Ab4977_6:0.0):0.0,JBA13_6:0.0):0.0,AB07_6
:0.0):0.0,YU-
R612_6:0.0):0.0,XH386_6:0.0):0.0,AB34299_6:0.0):0.0,NCGM237_6:0.0):0.0,KAB06_6:0.0):0
.0,Ab4568_6:0.0):0.0,AC30_6:0.0):0.0,AbPK1_6:0.0):0.0,MDR-ZJ06_6:0.0):0.0,1656-
2_6:0.0):0.0,USA2_6:0.0):0.0,WCHAB005133_6:0.0):0.0,XH857_6:0.0):0.0,CMC-CR-MDR-
Ab66_6:0.0):0.0,HRAB-
85_6:0.0):0.0,CBA7_6:0.0):0.0,KAB08_6:0.0):0.0,SSA12_6:0.0):0.0,KAB04_6:0.0):0.0,KBN10P
02143_6:0.0):2.0E-6,15A5_6:2.0E-
6)[&Bootstrap=100]:0.009258,6200_21:0.016553)[&Bootstrap=100]:0.259737)[&Bootstrap=
96]:0.121063)[&Bootstrap=100]:0.491232,(((RhsB:0.022798,RhsA:0.048825)[&Bootstrap=10
0]:1.287428,((Tke4:0.693867,Rhs1:0.709947)[&Bootstrap=100]:0.240357,(Tke2:0.685095,R
hs2:0.77283)[&Bootstrap=98]:0.158316)[&Bootstrap=90]:0.125877)[&Bootstrap=76]:0.1088
81,Tse5:1.107923)[&Bootstrap=100]:0.738946)[&Bootstrap=100]:0.806052)[&Bootstrap=67
]:0.008859)[&Bootstrap=53]:0.009603,(((15A34_5:0.0,USA2_5:0.0):2.0E-6,SSA6_5:2.0E-

```
6)[&Bootstrap=98]:7.17E-4,(((((AF-673_5:0.0,CMC-CR-MDR-Ab66_5:0.0):0.0,CMC-MDR-
Ab59_5:0.0):2.0E-6,CMC-CR-MDR-Ab4_5:2.0E-6)[&Bootstrap=97]:7.17E-
4,BJAB0868_5:7.17E-4)[&Bootstrap=56]:2.0E-
6,((((((((((((((((((((15A5_5:0.0,Ab4568_5:0.0):0.0,NCGM237_5:0.0):0.0,AR_0102_5:0.0):0.0,X
H386_5:0.0):0.0,SMC_Paed_Ab_BL01_5:0.0):0.0,XH856_5:0.0):0.0,TCDC-
0715_5:0.0):0.0,AR_0056_5:0.0):0.0,TYTH-
1_5:0.0):0.0,ACICU_5:0.0):0.0,Ab4653_5:0.0):0.0,KBN10P02143_5:0.0):0.0,XDR-
BJ83_5:0.0):0.0,MDR-ZJ06_5:0.0):0.0,HRAB-85_5:0.0):0.0,BJAB07104_5:0.0):0.0,MDR-
TJ_5:0.0):0.0,Ab4977_5:0.0):0.0,YU-R612_5:0.0):0.0,WCHAB005133_5:0.0):2.0E-
6,3027STDY5784958_5:2.0E-6)[&Bootstrap=50]:2.0E-6)[&Bootstrap=60]:2.0E-
6)[&Bootstrap=98]:0.014439)[&Bootstrap=74]:0.00992,SDF_15:0.012095)[&Bootstrap=74]:
0.006795,6200_15:0.011538)[&Bootstrap=55]:0.001512,IOMTU433_15:0.002171)[&Bootstr
ap=100]:0.04521)[&Bootstrap=90]:2.0E-6);
end;

begin figtree;
	set appearance.backgroundColorAttribute="Default";
	set appearance.backgroundColour=#ffffff;
	set appearance.branchColorAttribute="User selection";
	set appearance.branchColorGradient=false;
	set appearance.branchLineWidth=2.0;
	set appearance.branchMinLineWidth=0.0;
	set appearance.branchWidthAttribute="Fixed";
	set appearance.foregroundColour=#000000;
	set appearance.hilightingGradient=false;
	set appearance.selectionColour=#2d3680;
	set branchLabels.colorAttribute="User selection";
	set branchLabels.displayAttribute="Bootstrap";
	set branchLabels.fontName="Adobe Devanagari";
	set branchLabels.fontSize=8;
	set branchLabels.fontStyle=0;
	set branchLabels.isShown=false;
	set branchLabels.significantDigits=4;
	set layout.expansion=325;
	set layout.layoutType="RECTILINEAR";
	set layout.zoom=0;
	set legend.attribute="Bootstrap";
	set legend.fontSize=10.0;
	set legend.isShown=false;
	set legend.significantDigits=4;
	set nodeBars.barWidth=4.0;
	set nodeBars.displayAttribute=null;
	set nodeBars.isShown=false;
	set nodeLabels.colorAttribute="User selection";
	set nodeLabels.displayAttribute="Bootstrap";
	set nodeLabels.fontName="Arial";
	set nodeLabels.fontSize=9;
```

```
set nodeLabels.fontStyle=0;
set nodeLabels.isShown=true;
set nodeLabels.significantDigits=4;
set nodeShapeExternal.colourAttribute="User selection";
set nodeShapeExternal.isShown=true;
set nodeShapeExternal.minSize=10.0;
set nodeShapeExternal.scaleType=Width;
set nodeShapeExternal.shapeType=Circle;
set nodeShapeExternal.size=5.0;
set nodeShapeExternal.sizeAttribute="Fixed";
set nodeShapeInternal.colourAttribute="User selection";
set nodeShapeInternal.isShown=false;
set nodeShapeInternal.minSize=10.0;
set nodeShapeInternal.scaleType=Width;
set nodeShapeInternal.shapeType=Circle;
set nodeShapeInternal.size=4.0;
set nodeShapeInternal.sizeAttribute="Fixed";
set polarLayout.alignTipLabels=false;
set polarLayout.angularRange=0;
set polarLayout.rootAngle=0;
set polarLayout.rootLength=100;
set polarLayout.showRoot=true;
set radialLayout.spread=0.0;
set rectilinearLayout.alignTipLabels=true;
set rectilinearLayout.curvature=0;
set rectilinearLayout.rootLength=10000;
set scale.offsetAge=0.0;
set scale.rootAge=1.0;
set scale.scaleFactor=1.0;
set scale.scaleRoot=false;
set scaleAxis.automaticScale=true;
set scaleAxis.fontSize=8.0;
set scaleAxis.isShown=false;
set scaleAxis.lineWidth=1.0;
set scaleAxis.majorTicks=1.0;
set scaleAxis.minorTicks=0.5;
set scaleAxis.origin=0.0;
set scaleAxis.reverseAxis=false;
set scaleAxis.showGrid=true;
set scaleBar.automaticScale=true;
set scaleBar.fontSize=10.0;
set scaleBar.isShown=true;
set scaleBar.lineWidth=1.0;
set scaleBar.scaleRange=0.0;
set tipLabels.colorAttribute="User selection";
set tipLabels.displayAttribute="Names";
set tipLabels.fontName="Arial";
```

```
	set tipLabels.fontSize=11;
	set tipLabels.fontStyle=0;
	set tipLabels.isShown=true;
	set tipLabels.significantDigits=4;
	set trees.order=false;
	set trees.orderType="increasing";
	set trees.rooting=true;
	set trees.rootingType="Midpoint";
	set trees.transform=false;
	set trees.transformType="cladogram";
end;
```

Supplementary File S3d

```
#NEXUS
begin taxa;
	dimensions ntax=28;
	taxlabels
	AB030_24[&!color=#000000]
	AB031_25[&!color=#000000]
	AF-401_24[&!color=#000000]
	AR_0063_24[&!color=#000000]
	AR_0088_12[&!color=#000000]
	AR_0101_24[&!color=#000000]
	ATCC19606_10[&!color=#000000]
	ATCC19606_7[&!color=#000000]
	ATCC19606_9[&!color=#000000]
	Ab04-mff_11[&!color=#000000]
	Ab04-mff_12[&!color=#000000]
	AbH12O-A2_24[&!color=#000000]
	BJAB0715_11[&!color=#000000]
	BJAB0715_12[&!color=#000000]
	'CIP70.10_12'[&!color=#000000]
	D1279779_12
	HWBA8_12[&!color=#000000]
	LAC-4_12[&!color=#000000]
	R2090_12
	R2091_12[&!color=#000000]
	WKA02_11[&!color=#000000]
	WKA02_12[&!color=#000000]
	XH858_11[&!color=#000000]
	XH858_12[&!color=#000000]
	ZW85-1_20[&!color=#000000]
	ab736_10[&!color=#000000]
	ab736_7[&!color=#000000]
	ab736_9[&!color=#000000]
;
end;
```

```
begin trees;
	tree tree_1 = [&R] (ab736_7:2.0E-6,ATCC19606_7:2.0E-6,((((((((AB030_24:0.0,AF-
401_24:0.0):0.0,AR_0063_24:0.0):0.0,AR_0101_24:0.0):2.0E-6,AbH12O-A2_24:2.0E-
6)[&Bootstrap=98]:0.559886,AB031_25:0.905092)[&Bootstrap=94]:0.766365)[&Bootstrap=
35]:0.161406,((((((Ab04-mff_12:0.0,WKA02_12:0.0):0.0,XH858_12:0.0):0.0,LAC-
4_12:0.0):2.0E-6,BJAB0715_12:2.0E-
6)[&Bootstrap=72]:0.002331,((((AR_0088_12:0.0,R2091_12:0.0):0.0,HWBA8_12:0.0):2.0E-
6,'CIP70.10_12':2.0E-6)[&Bootstrap=72]:2.0E-6,((D1279779_12:2.0E-6,R2090_12:2.0E-
6)[&Bootstrap=84]:2.0E-6,ZW85-
1_20:0.00639)[&Bootstrap=95]:0.029069)[&Bootstrap=58]:8.6E-
4)[&Bootstrap=100]:1.598852,((ab736_9:2.0E-6,ATCC19606_9:2.0E-
6)[&Bootstrap=100]:1.219148,(ab736_10:2.0E-6,ATCC19606_10:2.0E-
6)[&Bootstrap=99]:1.238006)[&Bootstrap=50]:0.339233)[&Bootstrap=35]:0.362854)[&Boot
strap=45]:0.243433,(((Ab04-mff_11:0.0,WKA02_11:0.0):0.0,XH858_11:0.0):2.0E-
6,BJAB0715_11:2.0E-6)[&Bootstrap=98]:1.082163)[&Bootstrap=100]:2.118993);
end;

begin figtree;
	set appearance.backgroundColorAttribute="Default";
	set appearance.backgroundColour=#ffffff;
	set appearance.branchColorAttribute="User selection";
	set appearance.branchColorGradient=false;
	set appearance.branchLineWidth=2.0;
	set appearance.branchMinLineWidth=0.0;
	set appearance.branchWidthAttribute="Fixed";
	set appearance.foregroundColour=#000000;
	set appearance.hilightingGradient=false;
	set appearance.selectionColour=#2d3680;
	set branchLabels.colorAttribute="User selection";
	set branchLabels.displayAttribute="Branch times";
	set branchLabels.fontName="sansserif";
	set branchLabels.fontSize=8;
	set branchLabels.fontStyle=0;
	set branchLabels.isShown=false;
	set branchLabels.significantDigits=4;
	set layout.expansion=0;
	set layout.layoutType="RECTILINEAR";
	set layout.zoom=0;
	set legend.attribute="Bootstrap";
	set legend.fontSize=10.0;
	set legend.isShown=false;
	set legend.significantDigits=4;
	set nodeBars.barWidth=4.0;
	set nodeBars.displayAttribute=null;
	set nodeBars.isShown=false;
	set nodeLabels.colorAttribute="User selection";
```

```
set nodeLabels.displayAttribute="Bootstrap";
set nodeLabels.fontName="sansserif";
set nodeLabels.fontSize=8;
set nodeLabels.fontStyle=0;
set nodeLabels.isShown=true;
set nodeLabels.significantDigits=2;
set nodeShapeExternal.colourAttribute="User selection";
set nodeShapeExternal.isShown=true;
set nodeShapeExternal.minSize=10.0;
set nodeShapeExternal.scaleType=Width;
set nodeShapeExternal.shapeType=Circle;
set nodeShapeExternal.size=5.0;
set nodeShapeExternal.sizeAttribute="Fixed";
set nodeShapeInternal.colourAttribute="User selection";
set nodeShapeInternal.isShown=false;
set nodeShapeInternal.minSize=10.0;
set nodeShapeInternal.scaleType=Width;
set nodeShapeInternal.shapeType=Circle;
set nodeShapeInternal.size=4.0;
set nodeShapeInternal.sizeAttribute="Fixed";
set polarLayout.alignTipLabels=false;
set polarLayout.angularRange=0;
set polarLayout.rootAngle=0;
set polarLayout.rootLength=100;
set polarLayout.showRoot=true;
set radialLayout.spread=0.0;
set rectilinearLayout.alignTipLabels=false;
set rectilinearLayout.curvature=0;
set rectilinearLayout.rootLength=100;
set scale.offsetAge=0.0;
set scale.rootAge=1.0;
set scale.scaleFactor=1.0;
set scale.scaleRoot=false;
set scaleAxis.automaticScale=true;
set scaleAxis.fontSize=8.0;
set scaleAxis.isShown=false;
set scaleAxis.lineWidth=1.0;
set scaleAxis.majorTicks=1.0;
set scaleAxis.minorTicks=0.5;
set scaleAxis.origin=0.0;
set scaleAxis.reverseAxis=false;
set scaleAxis.showGrid=true;
set scaleBar.automaticScale=true;
set scaleBar.fontSize=10.0;
set scaleBar.isShown=true;
set scaleBar.lineWidth=1.0;
set scaleBar.scaleRange=0.0;
```

```
	set tipLabels.colorAttribute="User selection";
	set tipLabels.displayAttribute="Names";
	set tipLabels.fontName="sansserif";
	set tipLabels.fontSize=8;
	set tipLabels.fontStyle=0;
	set tipLabels.isShown=true;
	set tipLabels.significantDigits=4;
	set trees.order=false;
	set trees.orderType="increasing";
	set trees.rooting=true;
	set trees.rootingType="Midpoint";
	set trees.transform=false;
	set trees.transformType="cladogram";
end;
```

Supplementary File S3e

```
#NEXUS
begin taxa;
	dimensions ntax=226;
	taxlabels
	15A34_23[&!color=#000000]
	15A34_6[&!color=#000000]
	15A5_18[&!color=#000000]
	15A5_28[&!color=#000000]
	15A5_6[&!color=#000000]
	1656-2_6[&!color=#000000]
	1656-2_8[&!color=#000000]
	3027STDY5784958_18[&!color=#000000]
	3027STDY5784958_6[&!color=#000000]
	3207_1[&!color=#000000]
	3207_4[&!color=#000000]
	6200_15[&!color=#000000]
	6200_17[&!color=#000000]
	6200_21[&!color=#000000]
	A1296_8[&!color=#000000]
	A1_15[&!color=#000000]
	A1_16[&!color=#000000]
	A1_17[&!color=#000000]
	A388_15[&!color=#000000]
	A388_16[&!color=#000000]
	A388_17[&!color=#000000]
	A85_15[&!color=#000000]
	A85_16[&!color=#000000]
	A85_17[&!color=#000000]
	AB0057_15[&!color=#000000]
	AB030_15[&!color=#000000]
	AB030_22[&!color=#000000]
```

AB030_24[&!color=#000000]
AB031_25[&!color=#000000]
AB031_26[&!color=#000000]
AB031_27[&!color=#000000]
AB042_1[&!color=#000000]
AB07_18[&!color=#000000]
AB07_6[&!color=#000000]
AB307-0294_15[&!color=#000000]
AB307-0294_16[&!color=#000000]
AB307-0294_17[&!color=#000000]
AB34299_6[&!color=#000000]
AB5075-UW_15[&!color=#000000]
AB5075-UW_16[&!color=#000000]
AB5075-UW_17[&!color=#000000]
AC29_18[&!color=#000000]
AC29_6[&!color=#000000]
AC30_18[&!color=#000000]
AC30_6[&!color=#000000]
ACICU_6[&!color=#000000]
ACICU_7[&!color=#000000]
ACICU_8[&!color=#000000]
AF-401_15[&!color=#000000]
AF-401_22[&!color=#000000]
AF-401_24[&!color=#000000]
AF-401_30[&!color=#000000]
AF-673_18[&!color=#000000]
AF-673_6[&!color=#000000]
AR_0056_6[&!color=#000000]
AR_0056_8[&!color=#000000]
AR_0063_15[&!color=#000000]
AR_0063_22[&!color=#000000]
AR_0063_24[&!color=#000000]
AR_0078_15[&!color=#000000]
AR_0078_29[&!color=#000000]
AR_0083_15[&!color=#000000]
AR_0083_16[&!color=#000000]
AR_0083_17[&!color=#000000]
AR_0088_1[&!color=#000000]
AR_0088_12[&!color=#000000]
AR_0088_14[&!color=#000000]
AR_0088_32[&!color=#000000]
AR_0101_15[&!color=#000000]
AR_0101_22[&!color=#000000]
AR_0101_24[&!color=#000000]
AR_0102_18[&!color=#000000]
ATCC17978-mff_1[&!color=#000000]
ATCC17978-mff_2[&!color=#000000]

ATCC17978-mff_4[&!color=#000000]
ATCC19606_10[&!color=#000000]
ATCC19606_7[&!color=#000000]
ATCC19606_9[&!color=#000000]
AYE_16[&!color=#000000]
AYE_17[&!color=#000000]
AYP-A2_18[&!color=#000000]
AYP-A2_6[&!color=#000000]
Ab04-mff_11[&!color=#000000]
Ab04-mff_12[&!color=#000000]
Ab04-mff_13[&!color=#000000]
Ab4568_18[&!color=#000000]
Ab4568_28[&!color=#000000]
Ab4568_6[&!color=#000000]
Ab4653_18[&!color=#000000]
Ab4653_6[&!color=#000000]
Ab4977_18[&!color=#000000]
Ab4977_28[&!color=#000000]
Ab4977_6[&!color=#000000]
AbH12O-A2_15[&!color=#000000]
AbH12O-A2_22[&!color=#000000]
AbH12O-A2_24[&!color=#000000]
AbPK1_6[&!color=#000000]
AbPK1_8[&!color=#000000]
BJAB07104_18[&!color=#000000]
BJAB07104_6[&!color=#000000]
BJAB0715_11[&!color=#000000]
BJAB0715_12[&!color=#000000]
BJAB0715_13[&!color=#000000]
BJAB0868_18[&!color=#000000]
BJAB0868_6[&!color=#000000]
CBA7_18[&!color=#000000]
CBA7_6[&!color=#000000]
'CIP70.10_12'[&!color=#000000]
'CIP70.10_14'[&!color=#000000]
'CIP70.10_2'[&!color=#000000]
'CIP70.10_31'[&!color=#000000]
CMC-CR-MDR-Ab4_18[&!color=#000000]
CMC-CR-MDR-Ab4_6[&!color=#000000]
CMC-CR-MDR-Ab66_18[&!color=#000000]
CMC-CR-MDR-Ab66_6[&!color=#000000]
CMC-MDR-Ab59_18[&!color=#000000]
CMC-MDR-Ab59_6[&!color=#000000]
D1279779_13[&!color=#000000]
D36_15[&!color=#000000]
D36_16[&!color=#000000]
D36_17[&!color=#000000]

DU202_6[&!color=#000000]
DU202_8[&!color=#000000]
HRAB-85_18[&!color=#000000]
HRAB-85_6[&!color=#000000]
HWAB8_1[&!color=#000000]
HWBA8_12[&!color=#000000]
HWBA8_14[&!color=#000000]
IOMTU433_15[&!color=#000000]
IOMTU433_22[&!color=#000000]
JBA13_18[&!color=#000000]
JBA13_28[&!color=#000000]
JBA13_6[&!color=#000000]
KAB01_6[&!color=#000000]
KAB01_8[&!color=#000000]
KAB02_13[&!color=#000000]
KAB02_18[&!color=#000000]
KAB02_6[&!color=#000000]
KAB03_6[&!color=#000000]
KAB03_8[&!color=#000000]
KAB04_18[&!color=#000000]
KAB04_28[&!color=#000000]
KAB04_6[&!color=#000000]
KAB05_13[&!color=#000000]
KAB05_18[&!color=#000000]
KAB05_6[&!color=#000000]
KAB06_13[&!color=#000000]
KAB06_18[&!color=#000000]
KAB06_6[&!color=#000000]
KAB07_18[&!color=#000000]
KAB07_28[&!color=#000000]
KAB07_6[&!color=#000000]
KAB08_18[&!color=#000000]
KAB08_28[&!color=#000000]
KAB08_6[&!color=#000000]
KBN10P02143_18[&!color=#000000]
KBN10P02143_28[&!color=#000000]
KBN10P02143_6[&!color=#000000]
LAC-4_11[&!color=#000000]
LAC-4_12[&!color=#000000]
LAC-4_13[&!color=#000000]
MDR-TJ_13[&!color=#000000]
MDR-TJ_18[&!color=#000000]
MDR-TJ_6[&!color=#000000]
MDR-ZJ06_18[&!color=#000000]
MDR-ZJ06_6[&!color=#000000]
NCGM237_6[&!color=#000000]
R2090_13[&!color=#000000]

R2090_8[&!color=#000000]
R2091_12[&!color=#000000]
R2091_14[&!color=#000000]
R2091_2[&!color=#000000]
R2091_31[&!color=#000000]
SAA14_28[&!color=#000000]
SAA14_6[&!color=#000000]
SDF_15[&!color=#000000]
SMC_Paed_Ab_BL01_18[&!color=#000000]
SMC_Paed_Ab_BL01_28[&!color=#000000]
SMC_Paed_Ab_BL01_6[&!color=#000000]
SSA12_18[&!color=#000000]
SSA12_28[&!color=#000000]
SSA12_6[&!color=#000000]
SSA6_18[&!color=#000000]
SSA6_6[&!color=#000000]
SSMA17_18[&!color=#000000]
SSMA17_28[&!color=#000000]
TCDC-0715_6[&!color=#000000]
TCDC-AB0715_18[&!color=#000000]
TYTH-1_14[&!color=#000000]
TYTH-1_6[&!color=#000000]
USA15_15[&!color=#000000]
USA15_16[&!color=#000000]
USA2_18[&!color=#000000]
USA2_6[&!color=#000000]
WCHAB005078_15[&!color=#000000]
WCHAB005078_16[&!color=#000000]
WCHAB005078_17[&!color=#000000]
WCHAB005133_18[&!color=#000000]
WCHAB005133_6[&!color=#000000]
WKA02_11[&!color=#000000]
WKA02_12[&!color=#000000]
WKA02_13[&!color=#000000]
WKA02_15[&!color=#000000]
XDR-BJ83_18[&!color=#000000]
XDR-BJ83_6[&!color=#000000]
XH386_18[&!color=#000000]
XH386_6[&!color=#000000]
XH856_13[&!color=#000000]
XH856_18[&!color=#000000]
XH856_6[&!color=#000000]
XH857_6[&!color=#000000]
XH858_11[&!color=#000000]
XH858_12[&!color=#000000]
XH858_13[&!color=#000000]
XH859_18[&!color=#000000]

XH859_6[&!color=#000000]
XH860_6[&!color=#000000]
YU-R612_18[&!color=#000000]
YU-R612_28[&!color=#000000]
YU-R612_6[&!color=#000000]
ZW85-1_17[&!color=#000000]
ZW85-1_19[&!color=#000000]
ZW85-1_20[&!color=#000000]
ab736_10[&!color=#000000]
ab736_7[&!color=#000000]
ab736_9[&!color=#000000]
;
end;

begin trees;
tree tree_1 = [&R]
(6200_21:2.176964,((((((((((((((((KAB01_8:0.0,DU202_8:0.0):0.0,AR_0056_8:0.0):0.0,KAB03_
8:0.0):0.0,AbPK1_8:0.0):0.0,A1296_8:0.0):0.0,1656-2_8:0.0):2.0E-6,ACICU_8:2.0E-
6)[&Bootstrap=98]:0.009627,R2090_8:2.0E-
6)[&Bootstrap=100]:2.326382,(((((((((A1_17:0.0,AYE_17:0.0):0.0,WCHAB005078_17:0.0):0.0
,AB5075-UW_17:0.0):0.0,AR_0083_17:0.0):0.0,D36_17:0.0):0.0,A85_17:0.0):0.0,AB307-
0294_17:0.0):2.0E-6,A388_17:2.0E-6)[&Bootstrap=94]:0.006282,(6200_17:2.0E-6,ZW85-
1_17:2.0E-6)[&Bootstrap=85]:3.0E-
6)[&Bootstrap=100]:1.581729)[&Bootstrap=70]:0.476051,(ab736_10:2.0E-
6,ATCC19606_10:2.0E-
6)[&Bootstrap=100]:2.343454)[&Bootstrap=17]:0.2765,(((6200_15:0.006056,(((((((((((((WKA
02_15:0.0,WCHAB005078_15:0.0):0.0,AB307-
0294_15:0.0):0.0,A388_15:0.0):0.0,A1_15:0.0):0.0,AB5075-
UW_15:0.0):0.0,USA15_15:0.0):0.0,D36_15:0.0):0.0,IOMTU433_15:0.0):0.0,AB0057_15:0.0):
0.0,A85_15:0.0):0.0,SDF_15:0.0):0.0,AR_0078_15:0.0):2.0E-6,AR_0083_15:2.0E-
6)[&Bootstrap=77]:2.0E-
6)[&Bootstrap=65]:0.006052,((((AB030_15:0.0,AR_0063_15:0.0):0.0,AR_0101_15:0.0):0.0,A
F-401_15:0.0):2.0E-6,AbH12O-A2_15:2.0E-6)[&Bootstrap=86]:2.0E-
6)[&Bootstrap=100]:2.081454,((((((((((BJAB0715_12:0.0,R2091_12:0.0):0.0,HWBA8_12:0.0):
0.0,LAC-4_12:0.0):0.0,WKA02_12:0.0):0.0,AR_0088_12:0.0):0.0,'CIP70.10_12':0.0):0.0,Ab04-
mff_12:0.0):2.0E-6,XH858_12:2.0E-6)[&Bootstrap=86]:3.0E-6,ZW85-
1_20:0.93122)[&Bootstrap=95]:0.699859,AB031_27:2.585287)[&Bootstrap=96]:1.351444,A
B031_26:1.053488)[&Bootstrap=47]:0.511148)[&Bootstrap=27]:0.352745)[&Bootstrap=9]:0
.298414,(AR_0078_29:2.394848,(3207_1:2.0E-
6,(((AB042_1:0.0,HWAB8_1:0.0):0.0,ATCC17978-mff_1:0.0):2.0E-6,AR_0088_1:2.0E-
6)[&Bootstrap=64]:2.0E-
6)[&Bootstrap=98]:1.086376)[&Bootstrap=86]:0.953901)[&Bootstrap=8]:0.123555,((((((((AB
030_24:0.0,AR_0063_24:0.0):0.0,AF-401_24:0.0):0.0,AR_0101_24:0.0):2.0E-6,AbH12O-
A2_24:2.0E-6)[&Bootstrap=100]:1.725662,((TCDC-
AB0715_18:0.0,SSA12_18:0.0):0.0,KAB04_18:0.0):0.0,CBA7_18:0.0):0.0,3027STDY5784958_
18:0.0):0.0,WCHAB005133_18:0.0):0.0,SSA6_18:0.0):0.0,AF-673_18:0.0):0.0,CMC-CR-MDR-
Ab66_18:0.0):0.0,KAB07_18:0.0):0.0,YU-

R612_18:0.0):0.0,XH859_18:0.0):0.0,XH386_18:0.0):0.0,Ab4568_18:0.0):0.0,MDR-ZJ06_18:0.0):0.0,JBA13_18:0.0):0.0,BJAB0868_18:0.0):0.0,AB07_18:0.0):0.0,CMC-CR-MDR-Ab4_18:0.0):0.0,AC29_18:0.0):0.0,SMC_Paed_Ab_BL01_18:0.0):0.0,XH856_18:0.0):0.0,KAB06_18:0.0):0.0,KAB05_18:0.0):0.0,Ab4977_18:0.0):0.0,MDR-TJ_18:0.0):0.0,AR_0102_18:0.0):0.0,15A5_18:0.0):0.0,XDR-BJ83_18:0.0):0.0,AC30_18:0.0):0.0,USA2_18:0.0):0.0,AYP-A2_18:0.0):0.0,KBN10P02143_18:0.0):0.0,CMC-MDR-Ab59_18:0.0):0.0,KAB08_18:0.0):0.0,SSMA17_18:0.0):0.0,HRAB-85_18:0.0):0.0,KAB02_18:0.0):2.0E-6,BJAB07104_18:2.0E-6)[&Bootstrap=96]:0.027043,Ab4653_18:0.016792)[&Bootstrap=100]:1.80284)[&Bootstrap=86]:0.391027,((((((((((((((15A5_28:0.0,KAB04_28:0.0):0.0,SSA12_28:0.0):0.0,KAB08_28:0.0):0.0,SMC_Paed_Ab_BL01_28:0.0):0.0,SAA14_28:0.0):0.0,KAB07_28:0.0):0.0,JBA13_28:0.0):0.0,SSMA17_28:0.0):0.0,Ab4977_28:0.0):0.0,YU-R612_28:0.0):0.0,KBN10P02143_28:0.0):2.0E-6,Ab4568_28:2.0E-6)[&Bootstrap=100]:2.144,(((((((((AYE_16:0.0,D36_16:0.0):0.0,A85_16:0.0):0.0,USA15_16:0.0):0.0,WCHAB005078_16:0.0):0.0,A388_16:0.0):0.0,AB307-0294_16:0.0):0.0,AR_0083_16:0.0):0.0,AB5075-UW_16:0.0):2.0E-6,A1_16:2.0E-6)[&Bootstrap=99]:0.698972)[&Bootstrap=81]:0.403947)[&Bootstrap=66]:0.532428,(AB031_25:1.797181,AF-401_30:1.513899)[&Bootstrap=59]:0.333911)[&Bootstrap=47]:0.203613,((15A34_23:1.854401,(((ATCC19606_7:0.0,ACICU_7:0.0):2.0E-6,ab736_7:2.0E-6)[&Bootstrap=93]:0.006229,(KAB06_13:2.0E-6,((((((((((LAC-4_13:0.0,D1279779_13:0.0):0.0,KAB05_13:0.0):0.0,XH858_13:0.0):0.0,R2090_13:0.0):0.0,XH856_13:0.0):0.0,KAB02_13:0.0):0.0,WKA02_13:0.0):0.0,MDR-TJ_13:0.0):0.0,BJAB0715_13:0.0):2.0E-6,Ab04-mff_13:2.0E-6)[&Bootstrap=61]:2.0E-6)[&Bootstrap=96]:0.034771)[&Bootstrap=95]:1.056064)[&Bootstrap=35]:0.139868,((((AR_0088_14:0.0,TYTH-1_14:0.0):0.0,HWBA8_14:0.0):0.0,R2091_14:0.0):2.0E-6,'CIP70.10_14':2.0E-6)[&Bootstrap=100]:2.883851)[&Bootstrap=51]:0.496071)[&Bootstrap=30]:0.460415)[&Bootstrap=50]:0.179748,((((((Ab04-mff_11:0.0,WKA02_11:0.0):0.0,LAC-4_11:0.0):0.0,XH858_11:0.0):2.0E-6,BJAB0715_11:2.0E-6)[&Bootstrap=100]:1.971881,(ATCC17978-mff_4:2.0E-6,3207_4:2.0E-6)[&Bootstrap=100]:2.104716)[&Bootstrap=81]:0.539008,(ab736_9:2.0E-6,ATCC19606_9:2.0E-6)[&Bootstrap=100]:2.030517)[&Bootstrap=72]:0.495131)[&Bootstrap=52]:0.233203,((DU202_6:0.0,XH857_6:0.0):0.0,AB07_6:0.0):0.0,XDR-BJ83_6:0.0):0.0,CMC-CR-MDR-Ab4_6:0.0):0.0,BJAB07104_6:0.0):0.0,SAA14_6:0.0):0.0,1656-2_6:0.0):0.0,KAB07_6:0.0):0.0,KAB08_6:0.0):0.0,Ab4977_6:0.0):0.0,XH859_6:0.0):0.0,AbPK1_6:0.0):0.0,BJAB0868_6:0.0):0.0,XH860_6:0.0):0.0,KBN10P02143_6:0.0):0.0,15A5_6:0.0):0.0,AYP-A2_6:0.0):0.0,AC30_6:0.0):0.0,KAB02_6:0.0):0.0,NCGM237_6:0.0):0.0,KAB01_6:0.0):0.0,TYTH-1_6:0.0):0.0,USA2_6:0.0):0.0,CMC-MDR-Ab59_6:0.0):0.0,KAB03_6:0.0):0.0,MDR-ZJ06_6:0.0):0.0,JBA13_6:0.0):0.0,SSA12_6:0.0):0.0,SMC_Paed_Ab_BL01_6:0.0):0.0,3027STDY5784958_6:0.0):0.0,MDR-TJ_6:0.0):0.0,HRAB-85_6:0.0):0.0,SSA6_6:0.0):0.0,KAB06_6:0.0):0.0,CMC-CR-MDR-Ab66_6:0.0):0.0,KAB04_6:0.0):0.0,AF-673_6:0.0):0.0,XH386_6:0.0):0.0,Ab4568_6:0.0):0.0,CBA7_6:0.0):0.0,AB34299_6:0.0):0.0,AC

```
29_6:0.0):0.0,AR_0056_6:0.0):0.0,ACICU_6:0.0):0.0,15A34_6:0.0):0.0,WCHAB005133_6:0.0)
:0.0,Ab4653_6:0.0):0.0,KAB05_6:0.0):0.0,XH856_6:0.0):2.0E-6,TCDC-0715_6:2.0E-
6)[&Bootstrap=100]:0.056092,YU-
R612_6:0.016316)[&Bootstrap=97]:0.468873,(((((AB030_22:0.0,AR_0101_22:0.0):0.0,AR_00
63_22:0.0):0.0,IOMTU433_22:0.0):0.0,AF-401_22:0.0):2.0E-6,AbH12O-A2_22:2.0E-
6)[&Bootstrap=100]:2.681836)[&Bootstrap=81]:0.316186)[&Bootstrap=42]:0.42758,ZW85-
1_19:2.185038)[&Bootstrap=69]:0.304146,((ATCC17978-mff_2:0.021208,('CIP70.10_2':2.0E-
6,R2091_2:2.0E-
6)[&Bootstrap=99]:0.012612)[&Bootstrap=100]:1.318597,(('CIP70.10_31':2.0E-
6,R2091_31:2.0E-
6)[&Bootstrap=100]:1.223557,AR_0088_32:1.185707)[&Bootstrap=98]:0.714423)[&Bootstr
ap=74]:0.069852);
end;

begin figtree;
	set appearance.backgroundColorAttribute="Default";
	set appearance.backgroundColour=#ffffff;
	set appearance.branchColorAttribute="User selection";
	set appearance.branchColorGradient=false;
	set appearance.branchLineWidth=2.0;
	set appearance.branchMinLineWidth=0.0;
	set appearance.branchWidthAttribute="Fixed";
	set appearance.foregroundColour=#000000;
	set appearance.hilightingGradient=false;
	set appearance.selectionColour=#2d3680;
	set branchLabels.colorAttribute="User selection";
	set branchLabels.displayAttribute="Branch times";
	set branchLabels.fontName="sansserif";
	set branchLabels.fontSize=8;
	set branchLabels.fontStyle=0;
	set branchLabels.isShown=false;
	set branchLabels.significantDigits=4;
	set layout.expansion=700;
	set layout.layoutType="RECTILINEAR";
	set layout.zoom=0;
	set legend.attribute="Bootstrap";
	set legend.fontSize=10.0;
	set legend.isShown=false;
	set legend.significantDigits=4;
	set nodeBars.barWidth=4.0;
	set nodeBars.displayAttribute=null;
	set nodeBars.isShown=false;
	set nodeLabels.colorAttribute="User selection";
	set nodeLabels.displayAttribute="Bootstrap";
	set nodeLabels.fontName="Arial";
	set nodeLabels.fontSize=10;
	set nodeLabels.fontStyle=0;
```

```
set nodeLabels.isShown=true;
set nodeLabels.significantDigits=4;
set nodeShapeExternal.colourAttribute="User selection";
set nodeShapeExternal.isShown=true;
set nodeShapeExternal.minSize=10.0;
set nodeShapeExternal.scaleType=Width;
set nodeShapeExternal.shapeType=Circle;
set nodeShapeExternal.size=5.0;
set nodeShapeExternal.sizeAttribute="Fixed";
set nodeShapeInternal.colourAttribute="User selection";
set nodeShapeInternal.isShown=false;
set nodeShapeInternal.minSize=10.0;
set nodeShapeInternal.scaleType=Width;
set nodeShapeInternal.shapeType=Circle;
set nodeShapeInternal.size=4.0;
set nodeShapeInternal.sizeAttribute="Fixed";
set polarLayout.alignTipLabels=false;
set polarLayout.angularRange=0;
set polarLayout.rootAngle=0;
set polarLayout.rootLength=100;
set polarLayout.showRoot=true;
set radialLayout.spread=0.0;
set rectilinearLayout.alignTipLabels=false;
set rectilinearLayout.curvature=0;
set rectilinearLayout.rootLength=100;
set scale.offsetAge=0.0;
set scale.rootAge=1.0;
set scale.scaleFactor=1.0;
set scale.scaleRoot=false;
set scaleAxis.automaticScale=true;
set scaleAxis.fontSize=8.0;
set scaleAxis.isShown=false;
set scaleAxis.lineWidth=1.0;
set scaleAxis.majorTicks=1.0;
set scaleAxis.minorTicks=0.5;
set scaleAxis.origin=0.0;
set scaleAxis.reverseAxis=false;
set scaleAxis.showGrid=true;
set scaleBar.automaticScale=true;
set scaleBar.fontSize=10.0;
set scaleBar.isShown=true;
set scaleBar.lineWidth=1.0;
set scaleBar.scaleRange=0.0;
set tipLabels.colorAttribute="User selection";
set tipLabels.displayAttribute="Names";
set tipLabels.fontName="Arial";
set tipLabels.fontSize=12;
```

```
	set tipLabels.fontStyle=0;
	set tipLabels.isShown=true;
	set tipLabels.significantDigits=4;
	set trees.order=false;
	set trees.orderType="increasing";
	set trees.rooting=true;
	set trees.rootingType="Midpoint";
	set trees.transform=false;
	set trees.transformType="cladogram";
end;
```

Supplementary File S3f

```
#NEXUS
begin taxa;
	dimensions ntax=276;
	taxlabels
	15A34_23[&!color=#000000]
	15A34_5
	15A34_6[&!color=#000000]
	15A5_18[&!color=#000000]
	15A5_28[&!color=#000000]
	15A5_5
	15A5_6[&!color=#000000]
	1656-2_6[&!color=#000000]
	1656-2_8[&!color=#000000]
	3027STDY5784958_18[&!color=#000000]
	3027STDY5784958_5
	3027STDY5784958_6[&!color=#000000]
	3207_1[&!color=#000000]
	3207_22
	3207_4[&!color=#000000]
	6200_15[&!color=#000000]
	6200_17[&!color=#000000]
	6200_21[&!color=#000000]
	A1296_8[&!color=#000000]
	A1_15[&!color=#000000]
	A1_16[&!color=#000000]
	A1_17[&!color=#000000]
	A388_15[&!color=#000000]
	A388_16[&!color=#000000]
	A388_17[&!color=#000000]
	A85_15[&!color=#000000]
	A85_16[&!color=#000000]
	A85_17[&!color=#000000]
	AB0057_15[&!color=#000000]
	AB0057_16
	AB0057_17
```

AB030_15[&!color=#000000]
AB030_22[&!color=#000000]
AB030_24[&!color=#000000]
AB031_0
AB031_00
AB031_25[&!color=#000000]
AB031_26[&!color=#000000]
AB031_27[&!color=#000000]
AB042_1[&!color=#000000]
AB042_3
AB07_18[&!color=#000000]
AB07_6[&!color=#000000]
AB307-0294_15[&!color=#000000]
AB307-0294_16[&!color=#000000]
AB307-0294_17[&!color=#000000]
AB34299_18
AB34299_6[&!color=#000000]
AB5075-UW_15[&!color=#000000]
AB5075-UW_16[&!color=#000000]
AB5075-UW_17[&!color=#000000]
AC29_18[&!color=#000000]
AC29_6[&!color=#000000]
AC30_18[&!color=#000000]
AC30_6[&!color=#000000]
ACICU_6[&!color=#000000]
ACICU_7[&!color=#000000]
ACICU_8[&!color=#000000]
AF-401_15[&!color=#000000]
AF-401_22[&!color=#000000]
AF-401_24[&!color=#000000]
AF-401_30[&!color=#000000]
AF-673_18[&!color=#000000]
AF-673_5
AF-673_6[&!color=#000000]
AR_0056_5
AR_0056_6[&!color=#000000]
AR_0056_8[&!color=#000000]
AR_0063_15[&!color=#000000]
AR_0063_22[&!color=#000000]
AR_0063_24[&!color=#000000]
AR_0078_15[&!color=#000000]
AR_0078_29[&!color=#000000]
AR_0083_16[&!color=#000000]
AR_0083_17[&!color=#000000]
AR_0088_1[&!color=#000000]
AR_0088_12[&!color=#000000]
AR_0088_14[&!color=#000000]

AR_0088_32[&!color=#000000]
AR_0101_15[&!color=#000000]
AR_0101_22[&!color=#000000]
AR_0101_24[&!color=#000000]
AR_0102_18[&!color=#000000]
AR_0102_5
AR_0102_6
ATCC17978-mff_1[&!color=#000000]
ATCC17978-mff_2[&!color=#000000]
ATCC17978-mff_3
ATCC19606_0
ATCC19606_10[&!color=#000000]
ATCC19606_7[&!color=#000000]
ATCC19606_9[&!color=#000000]
AYE_16[&!color=#000000]
AYE_17[&!color=#000000]
AYP-A2_18[&!color=#000000]
AYP-A2_6[&!color=#000000]
Ab04-mff_11[&!color=#000000]
Ab04-mff_12[&!color=#000000]
Ab04-mff_13[&!color=#000000]
Ab4568_18[&!color=#000000]
Ab4568_28[&!color=#000000]
Ab4568_5
Ab4568_6[&!color=#000000]
Ab4653_18[&!color=#000000]
Ab4653_5
Ab4653_6[&!color=#000000]
Ab4977_18[&!color=#000000]
Ab4977_28[&!color=#000000]
Ab4977_5
Ab4977_6[&!color=#000000]
AbH12O-A2_15[&!color=#000000]
AbH12O-A2_22[&!color=#000000]
AbH12O-A2_24[&!color=#000000]
AbPK1_6[&!color=#000000]
AbPK1_8[&!color=#000000]
BJAB07104_18[&!color=#000000]
BJAB07104_5
BJAB07104_6[&!color=#000000]
BJAB0715_11[&!color=#000000]
BJAB0715_12[&!color=#000000]
BJAB0715_13[&!color=#000000]
BJAB0868_18[&!color=#000000]
BJAB0868_5
BJAB0868_6[&!color=#000000]
CBA7_18[&!color=#000000]

CBA7_6[&!color=#000000]
'CIP70.10_12'[&!color=#000000]
'CIP70.10_14'[&!color=#000000]
'CIP70.10_2'[&!color=#000000]
'CIP70.10_31'[&!color=#000000]
CMC-CR-MDR-Ab4_18[&!color=#000000]
CMC-CR-MDR-Ab4_5
CMC-CR-MDR-Ab4_6[&!color=#000000]
CMC-CR-MDR-Ab66_18[&!color=#000000]
CMC-CR-MDR-Ab66_5
CMC-CR-MDR-Ab66_6[&!color=#000000]
CMC-MDR-Ab59_18[&!color=#000000]
CMC-MDR-Ab59_5
CMC-MDR-Ab59_6[&!color=#000000]
D1279779_12
D1279779_13[&!color=#000000]
D1279779_8
D36_15[&!color=#000000]
D36_16[&!color=#000000]
D36_17[&!color=#000000]
DU202_8[&!color=#000000]
HRAB-85_18[&!color=#000000]
HRAB-85_5
HRAB-85_6[&!color=#000000]
HWAB8_1[&!color=#000000]
HWAB8_32
HWBA8_12[&!color=#000000]
HWBA8_14[&!color=#000000]
IOMTU433_0
IOMTU433_22[&!color=#000000]
JBA13_0
JBA13_18[&!color=#000000]
JBA13_28[&!color=#000000]
JBA13_6[&!color=#000000]
KAB01_6[&!color=#000000]
KAB02_0
KAB02_13[&!color=#000000]
KAB02_18[&!color=#000000]
KAB02_6[&!color=#000000]
KAB03_6[&!color=#000000]
KAB03_8[&!color=#000000]
KAB04_0
KAB04_18[&!color=#000000]
KAB04_28[&!color=#000000]
KAB04_6[&!color=#000000]
KAB05_0
KAB05_13[&!color=#000000]

KAB05_6[&!color=#000000]
KAB06_0
KAB06_13[&!color=#000000]
KAB06_18[&!color=#000000]
KAB06_6[&!color=#000000]
KAB07_0
KAB07_18[&!color=#000000]
KAB07_28[&!color=#000000]
KAB07_6[&!color=#000000]
KAB08_0
KAB08_18[&!color=#000000]
KAB08_28[&!color=#000000]
KAB08_6[&!color=#000000]
KBN10P02143_18[&!color=#000000]
KBN10P02143_28[&!color=#000000]
KBN10P02143_5
KBN10P02143_6[&!color=#000000]
LAC-4_12[&!color=#000000]
MDR-TJ_13[&!color=#000000]
MDR-TJ_18[&!color=#000000]
MDR-TJ_5
MDR-TJ_6[&!color=#000000]
MDR-ZJ06_18[&!color=#000000]
MDR-ZJ06_5
MDR-ZJ06_6[&!color=#000000]
NCGM237_5
NCGM237_6[&!color=#000000]
NCGM237_8
R2090_12
R2090_13[&!color=#000000]
R2090_8[&!color=#000000]
R2091_12[&!color=#000000]
R2091_14[&!color=#000000]
R2091_2[&!color=#000000]
R2091_31[&!color=#000000]
SAA14_0
SAA14_18
SAA14_28[&!color=#000000]
SAA14_6[&!color=#000000]
SDF_15[&!color=#000000]
SDF_16
SMC_Paed_Ab_BL01_18[&!color=#000000]
SMC_Paed_Ab_BL01_28[&!color=#000000]
SMC_Paed_Ab_BL01_6[&!color=#000000]
SSA12_0
SSA12_18[&!color=#000000]
SSA12_28[&!color=#000000]

SSA12_6[&!color=#000000]
SSA6_18[&!color=#000000]
SSA6_5
SSA6_6[&!color=#000000]
SSMA17_0
SSMA17_28[&!color=#000000]
TCDC-AB0715_18[&!color=#000000]
TCDC-AB0715_5
TYTH-1_5
TYTH-1_6[&!color=#000000]
USA15_16[&!color=#000000]
USA2_18[&!color=#000000]
USA2_5
USA2_6[&!color=#000000]
VgrG1Abay
VgrG2Abay
VgrG3Abay
VgrG4Abay
WCHAB005078_15[&!color=#000000]
WCHAB005078_16[&!color=#000000]
WCHAB005078_17[&!color=#000000]
WCHAB005133_18[&!color=#000000]
WCHAB005133_5
WCHAB005133_6[&!color=#000000]
WKA02_11[&!color=#000000]
WKA02_12[&!color=#000000]
WKA02_13[&!color=#000000]
WKA02_15[&!color=#000000]
XDR-BJ83_18[&!color=#000000]
XDR-BJ83_5
XDR-BJ83_6[&!color=#000000]
XH386_18[&!color=#000000]
XH386_5
XH386_6[&!color=#000000]
XH856_13[&!color=#000000]
XH856_18[&!color=#000000]
XH856_5
XH856_6[&!color=#000000]
XH857_6[&!color=#000000]
XH858_11[&!color=#000000]
XH858_12[&!color=#000000]
XH858_13[&!color=#000000]
XH859_18[&!color=#000000]
XH859_5
XH859_6[&!color=#000000]
XH860_6[&!color=#000000]
YU-R612_18[&!color=#000000]

YU-R612_28[&!color=#000000]
YU-R612_5
YU-R612_6[&!color=#000000]
ZW85-1_17[&!color=#000000]
ZW85-1_19[&!color=#000000]
ZW85-1_20[&!color=#000000]
ab736_0
ab736_10[&!color=#000000]
ab736_7[&!color=#000000]
ab736_9[&!color=#000000]
;
end;

begin trees;
tree tree_1 = [&R]
(SDF_16:0.028364,((((((((((A1_16:0.0,WCHAB005078_16:0.0):0.0,USA15_16:0.0):0.0,AB0057_16:0.0):0.0,AB307-0294_16:0.0):0.0,AR_0083_16:0.0):0.0,A85_16:0.0):0.0,D36_16:0.0):0.0,AB5075-UW_16:0.0):3.0E-6,A388_16:3.0E-6)[&Bootstrap=82]:3.0E-6,AYE_16:0.001318)[&Bootstrap=100]:0.011006,((6200_21:0.003089,(1656-2_6:0.001259,((15A34_6:0.0,AR_0102_6:0.0):0.0,KBN10P02143_6:0.0):0.0,XH859_6:0.0):0.0,KAB07_6:0.0):0.0,Ab4653_6:0.0):0.0,XH857_6:0.0):0.0,KAB01_6:0.0):0.0,USA2_6:0.0):0.0,AC30_6:0.0):0.0,MDR-ZJ06_6:0.0):0.0,KAB03_6:0.0):0.0,TYTH-1_6:0.0):0.0,Ab4568_6:0.0):0.0,XH386_6:0.0):0.0,ACICU_6:0.0):0.0,MDR-TJ_6:0.0):0.0,CBA7_6:0.0):0.0,XH860_6:0.0):0.0,3027STDY5784958_6:0.0):0.0,BJAB07104_6:0.0):0.0,KAB06_6:0.0):0.0,KAB02_6:0.0):0.0,XH856_6:0.0):0.0,KAB08_6:0.0):0.0,AYP-A2_6:0.0):0.0,BJAB0868_6:0.0):0.0,SMC_Paed_Ab_BL01_6:0.0):0.0,NCGM237_6:0.0):0.0,SSA12_6:0.0):0.0,AC29_6:0.0):0.0,AB07_6:0.0):0.0,XDR-BJ83_6:0.0):0.0,KAB04_6:0.0):0.0,AR_0056_6:0.0):0.0,SAA14_6:0.0):0.0,KAB05_6:0.0):0.0,SSA6_6:0.0):0.0,AB34299_6:0.0):0.0,WCHAB005133_6:0.0):0.0,JBA13_6:0.0):0.0,HRAB-85_6:0.0):0.0,Ab4977_6:0.0):0.0,AbPK1_6:0.0):0.0,YU-R612_6:0.0):3.0E-6,15A5_6:3.0E-6)[&Bootstrap=94]:3.0E-6,(((AF-673_6:0.0,CMC-CR-MDR-Ab66_6:0.0):0.0,CMC-MDR-Ab59_6:0.0):3.0E-6,CMC-CR-MDR-Ab4_6:3.0E-6)[&Bootstrap=93]:3.0E-6)[&Bootstrap=84]:3.0E-6)[&Bootstrap=100]:0.01088)[&Bootstrap=96]:0.104935,((IOMTU433_22:0.014209,(((((3207_22:0.0,AR_0101_22:0.0):0.0,AR_0063_22:0.0):0.0,AF-401_22:0.0):0.0,AbH12O-A2_22:0.0):3.0E-6,AB030_22:3.0E-6)[&Bootstrap=100]:0.007533)[&Bootstrap=68]:0.002369,(AB031_27:0.004612,(((((VgrG2Abay:0.250151,VgrG3Abay:0.142126)[&Bootstrap=100]:1.101002,(((AB042_3:3.0E-6,ATCC17978-mff_3:3.0E-6)[&Bootstrap=100]:0.089493,(ZW85-1_20:0.036576,((XH858_12:0.026717,BJAB0715_12:0.004443)[&Bootstrap=80]:0.008856,(((D1279779_12:3.0E-6,R2090_12:3.0E-6)[&Bootstrap=99]:0.017783,(((AR_0088_12:0.0,R2091_12:0.0):0.0,HWBA8_12:0.0):3.0E-6,'CIP70.10_12':3.0E-6)[&Bootstrap=99]:0.013339)[&Bootstrap=81]:0.004418,((Ab04-mff_12:0.0,WKA02_12:0.0):3.0E-6,LAC-4_12:3.0E-6)[&Bootstrap=58]:3.0E-6)[&Bootstrap=65]:3.0E-

6)[&Bootstrap=77]:0.003738)[&Bootstrap=99]:0.081242)[&Bootstrap=100]:0.376809,(((('CIP70.10_31':3.0E-6,R2091_31:3.0E-
6)[&Bootstrap=100]:0.195025,(((((((((((15A5_28:0.0,SSMA17_28:0.0):0.0,Ab4977_28:0.0):0.0,JBA13_28:0.0):0.0,SSA12_28:0.0):0.0,KAB04_28:0.0):0.0,SAA14_28:0.0):0.0,KAB07_28:0.0):0.0,KAB08_28:0.0):0.0,KBN10P02143_28:0.0):0.0,YU-
R612_28:0.0):0.0,SMC_Paed_Ab_BL01_28:0.0):3.0E-6,Ab4568_28:3.0E-
6)[&Bootstrap=100]:0.115201)[&Bootstrap=96]:0.055642,(((BJAB0715_13:0.016939,XH858_13:3.0E-6)[&Bootstrap=100]:0.002407,(((((Ab04-
mff_13:0.0,XH856_13:0.0):0.0,WKA02_13:0.0):0.0,KAB05_13:0.0):0.0,KAB06_13:0.0):3.0E-6,KAB02_13:3.0E-6)[&Bootstrap=66]:3.0E-
6)[&Bootstrap=99]:0.007503,((D1279779_13:0.0,R2090_13:0.0):3.0E-6,MDR-TJ_13:3.0E-
6)[&Bootstrap=100]:0.002148)[&Bootstrap=100]:0.163196)[&Bootstrap=73]:0.018137,((((ab736_7:3.0E-6,ACICU_7:3.0E-6)[&Bootstrap=51]:3.0E-6,ATCC19606_7:3.0E-
6)[&Bootstrap=100]:0.15977,ZW85-1_19:0.174063)[&Bootstrap=78]:0.046674,(AF-
401_30:0.033621,(((3207_1:0.0,HWAB8_1:0.0):3.0E-6,AR_0088_1:3.0E-
6)[&Bootstrap=100]:0.00554,(AB042_1:3.0E-6,ATCC17978-mff_1:3.0E-
6)[&Bootstrap=70]:3.0E-
6)[&Bootstrap=99]:0.0034)[&Bootstrap=100]:0.149862)[&Bootstrap=94]:0.03906)[&Bootstrap=99]:0.110373)[&Bootstrap=100]:0.406319)[&Bootstrap=99]:0.284684,(((((((((VgrG1Abay:1.413938,((((AR_0088_14:0.0,R2091_14:0.0):0.0,HWBA8_14:0.0):3.0E-
6,'CIP70.10_14':3.0E-
6)[&Bootstrap=100]:0.014331,AR_0078_29:0.018505)[&Bootstrap=78]:2.18E-
4)[&Bootstrap=40]:0.074422,15A34_23:0.120972)[&Bootstrap=51]:0.04428,(6200_17:0.004117,(ZW85-
1_17:0.006131,((((((((A1_17:0.0,AR_0083_17:0.0):0.0,AB0057_17:0.0):0.0,AB307-
0294_17:0.0):0.0,AB5075-
UW_17:0.0):0.0,A85_17:0.0):0.0,WCHAB005078_17:0.0):0.0,AYE_17:0.0):3.0E-
6,A388_17:3.0E-6)[&Bootstrap=56]:3.0E-
6,D36_17:0.002037)[&Bootstrap=98]:0.002033)[&Bootstrap=98]:0.004048)[&Bootstrap=93]:0.090396)[&Bootstrap=53]:0.057302,(3207_4:0.131588,((((((((((((((((((((((((((((((((((((((15A5_18:0.0,AF-
673_18:0.0):0.0,KAB06_18:0.0):0.0,WCHAB005133_18:0.0):0.0,AC30_18:0.0):0.0,AR_0102_18:0.0):0.0,AYP-A2_18:0.0):0.0,MDR-TJ_18:0.0):0.0,CBA7_18:0.0):0.0,YU-
R612_18:0.0):0.0,KBN10P02143_18:0.0):0.0,AC29_18:0.0):0.0,KAB02_18:0.0):0.0,AB07_18:0.0):0.0,SSA12_18:0.0):0.0,JBA13_18:0.0):0.0,XH856_18:0.0):0.0,XH859_18:0.0):0.0,SAA14_18:0.0):0.0,SMC_Paed_Ab_BL01_18:0.0):0.0,KAB08_18:0.0):0.0,AB34299_18:0.0):0.0,BJAB0868_18:0.0):0.0,Ab4977_18:0.0):0.0,HRAB-
85_18:0.0):0.0,USA2_18:0.0):0.0,KAB07_18:0.0):0.0,MDR-ZJ06_18:0.0):0.0,XDR-
BJ83_18:0.0):0.0,Ab4653_18:0.0):0.0,XH386_18:0.0):0.0,CMC-CR-MDR-
Ab66_18:0.0):0.0,SSA6_18:0.0):0.0,CMC-CR-MDR-Ab4_18:0.0):0.0,CMC-MDR-
Ab59_18:0.0):0.0,KAB04_18:0.0):0.0,Ab4568_18:0.0):0.0,BJAB07104_18:0.0):3.0E-
6,3027STDY5784958_18:3.0E-6)[&Bootstrap=49]:3.0E-6,TCDC-AB0715_18:3.0E-
6)[&Bootstrap=100]:0.120187)[&Bootstrap=99]:0.094307)[&Bootstrap=43]:0.036776,((A1296_8:0.013344,((((((1656-
2_8:0.0,ACICU_8:0.0):0.0,KAB03_8:0.0):0.0,DU202_8:0.0):0.0,AR_0056_8:0.0):3.0E-
6,AbPK1_8:3.0E-6)[&Bootstrap=56]:3.0E-
6,NCGM237_8:0.002219)[&Bootstrap=99]:0.004426)[&Bootstrap=98]:0.005824,(D1279779

_8:3.0E-6,R2090_8:3.0E-6)[&Bootstrap=100]:0.003063)[&Bootstrap=100]:0.146494)[&Bootstrap=42]:0.019608,IOMTU433_0:0.355048)[&Bootstrap=44]:0.013029,((AB031_25:0.185461,((((AB030_24:0.0,AR_0101_24:0.0):0.0,AF-401_24:0.0):0.0,AR_0063_24:0.0):3.0E-6,AbH12O-A2_24:3.0E-6)[&Bootstrap=100]:0.107412)[&Bootstrap=52]:0.039519,(((Ab04-mff_11:0.0,WKA02_11:0.0):0.0,XH858_11:0.0):3.0E-6,BJAB0715_11:3.0E-6)[&Bootstrap=100]:0.145451)[&Bootstrap=61]:0.031681)[&Bootstrap=59]:0.432914,(ab736_9:3.0E-6,ATCC19606_9:3.0E-6)[&Bootstrap=100]:0.402506)[&Bootstrap=100]:0.835561,((ab736_10:3.0E-6,ATCC19606_10:3.0E-6)[&Bootstrap=100]:1.499246,VgrG4Abay:2.131111)[&Bootstrap=100]:1.295222)[&Bootstrap=100]:0.44861)[&Bootstrap=100]:0.524536,((((AB031_0:0.0,ab736_0:0.0):0.0,ATCC19606_0:0.0):3.0E-6,AB031_00:3.0E-6)[&Bootstrap=100]:0.431576,(((AR_0088_32:3.0E-6,HWAB8_32:3.0E-6)[&Bootstrap=100]:0.304766,('CIP70.10_2':3.0E-6,R2091_2:3.0E-6)[&Bootstrap=100]:0.012484)[&Bootstrap=66]:0.012972,ATCC17978-mff_2:0.032918)[&Bootstrap=100]:0.187544)[&Bootstrap=97]:0.115551)[&Bootstrap=100]:0.934502,(((AB5075-UW_15:0.00122,((A85_15:0.0,WCHAB005078_15:0.0):3.0E-6,AB0057_15:3.0E-6)[&Bootstrap=73]:3.0E-6)[&Bootstrap=100]:0.001221,((((A1_15:0.0,AR_0078_15:0.0):0.0,AB307-0294_15:0.0):0.0,D36_15:0.0):3.0E-6,A388_15:3.0E-6)[&Bootstrap=63]:3.0E-6)[&Bootstrap=98]:0.004304,((((SDF_15:0.007361,6200_15:0.00244)[&Bootstrap=73]:0.002452,(AB031_26:0.007405,(((((((((((((((((((((((((((((((((((((JBA13_0:0.0,YU-R612_5:0.0):0.0,BJAB0868_5:0.0):0.0,CMC-CR-MDR-Ab4_5:0.0):0.0,TYTH-1_5:0.0):0.0,Ab4653_5:0.0):0.0,CMC-MDR-Ab59_5:0.0):0.0,SAA14_0:0.0):0.0,WCHAB005133_5:0.0):0.0,MDR-TJ_5:0.0):0.0,15A5_5:0.0):0.0,XH859_5:0.0):0.0,CMC-CR-MDR-Ab66_5:0.0):0.0,15A34_5:0.0):0.0,KAB05_0:0.0):0.0,HRAB-85_5:0.0):0.0,3027STDY5784958_5:0.0):0.0,SSMA17_0:0.0):0.0,KAB04_0:0.0):0.0,KAB08_0:0.0):0.0,XDR-BJ83_5:0.0):0.0,Ab4568_5:0.0):0.0,AF-673_5:0.0):0.0,USA2_5:0.0):0.0,KBN10P02143_5:0.0):0.0,NCGM237_5:0.0):0.0,SSA6_5:0.0):0.0,KAB07_0:0.0):0.0,AR_0056_5:0.0):0.0,MDR-ZJ06_5:0.0):0.0,SSA12_0:0.0):0.0,AR_0102_5:0.0):0.0,KAB06_0:0.0):0.0,XH386_5:0.0):0.0,Ab4977_5:0.0):0.0,BJAB07104_5:0.0):0.0,XH856_5:0.0):3.0E-6,KAB02_0:3.0E-6)[&Bootstrap=74]:3.0E-6)[&Bootstrap=67]:3.0E-6)[&Bootstrap=74]:0.002444,(((((AB030_15:0.0,AR_0063_15:0.0):0.0,AF-401_15:0.0):0.0,AR_0101_15:0.0):3.0E-6,AbH12O-A2_15:3.0E-6)[&Bootstrap=100]:0.006789,TCDC-AB0715_5:3.0E-6)[&Bootstrap=96]:0.001221)[&Bootstrap=96]:0.002447,WKA02_15:0.003665)[&Bootstrap=95]:0.001831)[&Bootstrap=98]:0.057622)[&Bootstrap=100]:1.052101)[&Bootstrap=58]:0.004607)[&Bootstrap=83]:0.126351)[&Bootstrap=100]:0.132291);
end;

begin figtree;
	set appearance.backgroundColorAttribute="Default";
	set appearance.backgroundColour=#ffffff;
	set appearance.branchColorAttribute="User selection";
	set appearance.branchColorGradient=false;

```
set appearance.branchLineWidth=2.0;
set appearance.branchMinLineWidth=0.0;
set appearance.branchWidthAttribute="Fixed";
set appearance.foregroundColour=#000000;
set appearance.hilightingGradient=false;
set appearance.selectionColour=#2d3680;
set branchLabels.colorAttribute="User selection";
set branchLabels.displayAttribute="Branch times";
set branchLabels.fontName="Adobe Devanagari";
set branchLabels.fontSize=8;
set branchLabels.fontStyle=0;
set branchLabels.isShown=false;
set branchLabels.significantDigits=4;
set layout.expansion=504;
set layout.layoutType="RECTILINEAR";
set layout.zoom=0;
set legend.attribute="Bootstrap";
set legend.fontSize=10.0;
set legend.isShown=false;
set legend.significantDigits=4;
set nodeBars.barWidth=4.0;
set nodeBars.displayAttribute=null;
set nodeBars.isShown=false;
set nodeLabels.colorAttribute="User selection";
set nodeLabels.displayAttribute="Bootstrap";
set nodeLabels.fontName="Adobe Devanagari";
set nodeLabels.fontSize=8;
set nodeLabels.fontStyle=0;
set nodeLabels.isShown=true;
set nodeLabels.significantDigits=4;
set nodeShapeExternal.colourAttribute="User selection";
set nodeShapeExternal.isShown=true;
set nodeShapeExternal.minSize=10.0;
set nodeShapeExternal.scaleType=Width;
set nodeShapeExternal.shapeType=Circle;
set nodeShapeExternal.size=5.0;
set nodeShapeExternal.sizeAttribute="Fixed";
set nodeShapeInternal.colourAttribute="User selection";
set nodeShapeInternal.isShown=false;
set nodeShapeInternal.minSize=10.0;
set nodeShapeInternal.scaleType=Width;
set nodeShapeInternal.shapeType=Circle;
set nodeShapeInternal.size=4.0;
set nodeShapeInternal.sizeAttribute="Fixed";
set polarLayout.alignTipLabels=false;
set polarLayout.angularRange=0;
set polarLayout.rootAngle=0;
```

```
	set polarLayout.rootLength=100;
	set polarLayout.showRoot=true;
	set radialLayout.spread=0.0;
	set rectilinearLayout.alignTipLabels=false;
	set rectilinearLayout.curvature=0;
	set rectilinearLayout.rootLength=100;
	set scale.offsetAge=0.0;
	set scale.rootAge=1.0;
	set scale.scaleFactor=1.0;
	set scale.scaleRoot=false;
	set scaleAxis.automaticScale=true;
	set scaleAxis.fontSize=8.0;
	set scaleAxis.isShown=false;
	set scaleAxis.lineWidth=1.0;
	set scaleAxis.majorTicks=1.0;
	set scaleAxis.minorTicks=0.5;
	set scaleAxis.origin=0.0;
	set scaleAxis.reverseAxis=false;
	set scaleAxis.showGrid=true;
	set scaleBar.automaticScale=true;
	set scaleBar.fontSize=10.0;
	set scaleBar.isShown=true;
	set scaleBar.lineWidth=1.0;
	set scaleBar.scaleRange=0.0;
	set tipLabels.colorAttribute="User selection";
	set tipLabels.displayAttribute="Names";
	set tipLabels.fontName="Adobe Devanagari";
	set tipLabels.fontSize=8;
	set tipLabels.fontStyle=0;
	set tipLabels.isShown=true;
	set tipLabels.significantDigits=4;
	set trees.order=false;
	set trees.orderType="increasing";
	set trees.rooting=true;
	set trees.rootingType="Midpoint";
	set trees.transform=false;
	set trees.transformType="cladogram";
end;
```

Supplementary File S3g

```
#NEXUS
begin taxa;
	dimensions ntax=189;
	taxlabels
	15A34_B
	15A34_J
	15A34_M
```

15A5_B
1656-2_B
1656-2_M
3027STDY5784958_A
3027STDY5784958_B
3207_M
6200_A
6200_E
A1268_C
A1268_F
A1_M
A388_J
A388_M
A85_J
A85_M
AB0057_J
AB0057_M
AB030_C
AB030_I
AB030_M
AB042_C
AB042_M
AB07_A
AB07_M
AB307-0294_C
AB307-0294_M
AB34299_A
AB34299_B
AB34299_M
AB5075-UW_M
AC29_A
AC29_B
AC29_M
AC30_A
AC30_B
AC30_M
ACICU_B
ACICU_H
ACICU_M
AF-401_C
AF-401_M
AF-673_A
AF-673_B
AF-673_M
AR_0056_B
AR_0056_M
AR_0063_C

AR_0063_M
AR_0078_C
AR_0078_M
AR_0083_M
AR_0088_I
AR_0088_M
AR_0101_C
AR_0101_M
AR_0102_A
AR_0102_B
ATCC17978-mff_C
ATCC17978-mff_M
ATCC19606_L
ATCC19606_M
AYE_M
AYP-A2_B
AYP-A2_M
Ab04-mff_M
Ab4568_A
Ab4568_B
Ab4653_B
Ab4653_M
Ab4977_A
Ab4977_B
AbH12O-A2_C
AbH12O-A2_M
AbPK1_B
AbPK1_M
B8342_G
BJAB07104_A
BJAB07104_B
BJAB07104_M
BJAB0715_A
BJAB0715_M
BJAB0868_A
BJAB0868_B
BJAB0868_M
CBA7_A
CBA7_B
CBA7_M
'CIP70.10_M'
CMC-CR-MDR-Ab4_A
CMC-CR-MDR-Ab4_B
CMC-CR-MDR-Ab4_M
CMC-CR-MDR-Ab66_A
CMC-CR-MDR-Ab66_B
CMC-CR-MDR-Ab66_M

CMC-MDR-Ab59_A
CMC-MDR-Ab59_B
CMC-MDR-Ab59_M
D36_M
DU202_B
DU202_M
HRAB-85_B
HRAB-85_M
HWAB8_I
HWAB8_M
IOMTU433_M
JBA13_A
JBA13_B
KAB01_A
KAB01_B
KAB01_M
KAB02_A
KAB02_B
KAB03_A
KAB03_B
KAB03_M
KAB04_A
KAB04_B
KAB05_A
KAB05_B
KAB06_A
KAB06_B
KAB07_A
KAB07_B
KAB08_A
KAB08_B
KBN10P02143_B
LAC-4_A
MDR-TJ_B
MDR-TJ_M
MDR-ZJ06_A
MDR-ZJ06_B
MDR-ZJ06_M
NCGM237_B
NCGM237_M
PAAR1
PAAR2
PAAR3
R2090_M
R2091_M
SAA14_A
SAA14_B

```
		SDF_D
		SDF_H
		SDF_K
		SDF_M
		SMC_Paed_Ab_BL01_B
		SSA12_A
		SSA12_B
		SSA6_B
		SSA6_M
		SSMA17_A
		SSMA17_B
		TCDC-0715_B
		TCDC-0715_M
		TYTH-1_A
		TYTH-1_M
		USA15_M
		USA2_B
		USA2_M
		WCHAB005078_M
		WCHAB005133_B
		WCHAB005133_M
		WKA02_A
		WKA02_M
		XDR-BJ83_A
		XDR-BJ83_B
		XDR-BJ83_M
		XH386_A
		XH386_B
		XH386_M
		XH856_A
		XH856_M
		XH857_A
		XH857_B
		XH857_M
		XH858_A
		XH858_M
		XH859_A
		XH859_B
		XH859_M
		XH860_B
		XH860_M
		YU-R612_B
		ZW85-1_M
		ab736_L
		ab736_M
;
end;
```

begin trees;
tree tree_1 = [&R]
(PAAR1:5.392189,PAAR3:0.642689,((15A34_B:0.0,XH857_B:0.0):0.0,HRAB-85_B:0.0):0.0,Ab4653_B:0.0):0.0,CMC-MDR-Ab59_B:0.0):0.0,YU-R612_B:0.0):0.0,CMC-CR-MDR-Ab66_B:0.0):0.0,SMC_Paed_Ab_BL01_B:0.0):0.0,AC29_B:0.0):0.0,KAB02_B:0.0):0.0,SSA6_B:0.0):0.0,AF-673_B:0.0):0.0,CMC-CR-MDR-Ab4_B:0.0):0.0,KAB04_B:0.0):0.0,DU202_B:0.0):0.0,SSA12_B:0.0):0.0,XH386_B:0.0):0.0,KAB03_B:0.0):0.0,AbPK1_B:0.0):0.0,AR_0056_B:0.0):0.0,SAA14_B:0.0):0.0,Ab4977_B:0.0):0.0,JBA13_B:0.0):0.0,CBA7_B:0.0):0.0,AR_0102_B:0.0):0.0,SSMA17_B:0.0):0.0,XDR-BJ83_B:0.0):0.0,KAB08_B:0.0):0.0,KAB06_B:0.0):0.0,3027STDY5784958_B:0.0):0.0,KBN10P02143_B:0.0):0.0,USA2_B:0.0):0.0,XH859_B:0.0):0.0,KAB05_B:0.0):0.0,WCHAB005133_B:0.0):0.0,AB34299_B:0.0):0.0,AYP-A2_B:0.0):0.0,Ab4568_B:0.0):0.0,XH860_B:0.0):0.0,KAB07_B:0.0):0.0,KAB01_B:0.0):2.0E-6,15A5_B:2.0E-6)[&Bootstrap=65]:2.0E-6,((((((((1656-2_B:0.0,MDR-ZJ06_B:0.0):0.0,NCGM237_B:0.0):0.0,MDR-TJ_B:0.0):0.0,BJAB07104_B:0.0):0.0,TCDC-0715_B:0.0):0.0,BJAB0868_B:0.0):0.0,ACICU_B:0.0):2.0E-6,AC30_B:2.0E-6)[&Bootstrap=98]:0.003481)[&Bootstrap=100]:0.187607,((AB307-0294_C:0.029441,(A1268_C:0.00362,((AB042_C:0.0,ATCC17978-mff_C:0.0):2.0E-6,AR_0078_C:2.0E-6)[&Bootstrap=72]:2.0E-6)[&Bootstrap=43]:2.0E-6)[&Bootstrap=71]:0.005027,(AbH12O-A2_C:0.003613,(((AB030_C:0.0,AR_0101_C:0.0):0.0,AR_0063_C:0.0):2.0E-6,AF-401_C:2.0E-6)[&Bootstrap=66]:2.0E-6)[&Bootstrap=89]:0.005874)[&Bootstrap=81]:0.513072)[&Bootstrap=74]:0.096551,(SDF_D:0.074996,((ACICU_H:0.027809,SDF_H:0.085068)[&Bootstrap=100]:0.899257,((PAAR2:0.655804,(((ab736_L:2.0E-6,ATCC19606_L:2.0E-6)[&Bootstrap=100]:0.718107,6200_E:0.247027)[&Bootstrap=81]:0.135082,A1268_F:0.793038)[&Bootstrap=87]:0.243802)[&Bootstrap=56]:0.351602,SDF_K:0.558106)[&Bootstrap=53]:0.060344)[&Bootstrap=100]:0.928599)[&Bootstrap=100]:0.761699)[&Bootstrap=61]:0.07608,((((AB030_I:0.0,HWAB8_I:0.0):2.0E-6,AR_0088_I:2.0E-6)[&Bootstrap=100]:0.292795,B8342_G:0.354294)[&Bootstrap=96]:0.249987,(((A388_J:0.0,AB0057_J:0.0):2.0E-6,A85_J:2.0E-6)[&Bootstrap=80]:2.0E-6,15A34_J:0.014679)[&Bootstrap=100]:0.751393)[&Bootstrap=99]:0.379393)[&Bootstrap=65]:0.064318,LAC-4_A:0.01939)[&Bootstrap=67]:0.020365,((((((((((((((((((((((((((((((6200_A:0.0,KAB04_A:0.0):0.0,CMC-CR-MDR-Ab66_A:0.0):0.0,CMC-CR-MDR-Ab4_A:0.0):0.0,XH386_A:0.0):0.0,CBA7_A:0.0):0.0,XDR-BJ83_A:0.0):0.0,XH859_A:0.0):0.0,Ab4977_A:0.0):0.0,AR_0102_A:0.0):0.0,KAB05_A:0.0):0.0,AF-673_A:0.0):0.0,MDR-ZJ06_A:0.0):0.0,SSA12_A:0.0):0.0,XH858_A:0.0):0.0,XH856_A:0.0):0.0,WKA02_A:0.0):0.0,SSMA17_A:0.0):0.0,TYTH-1_A:0.0):0.0,KAB06_A:0.0):0.0,KAB02_A:0.0):0.0,JBA13_A:0.0):0.0,Ab4568_A:0.0):0.0,SAA14_A:0.0):0.0,KAB07_A:0.0):0.0,CMC-MDR-Ab59_A:0.0):0.0,XH857_A:0.0):0.0,KAB08_A:0.0):2.0E-6,3027STDY5784958_A:2.0E-6)[&Bootstrap=73]:2.0E-6,((BJAB07104_A:0.0,BJAB0868_A:0.0):2.0E-6,BJAB0715_A:2.0E-6)[&Bootstrap=99]:0.003399)[&Bootstrap=70]:0.003447,(((((AB34299_A:0.0,AC30_A:0.0):0.

0,KAB03_A:0.0):0.0,KAB01_A:0.0):2.0E-6,AC29_A:2.0E-6)[&Bootstrap=85]:2.0E-
6,AB07_A:0.003398)[&Bootstrap=70]:0.003356)[&Bootstrap=82]:0.001875)[&Bootstrap=89]
:0.389282,((SDF_M:0.076811,(IOMTU433_M:0.038037,((
((((((TYTH-
1_M:0.0,SSA6_M:0.0):0.0,XH857_M:0.0):0.0,'CIP70.10_M':0.0):0.0,USA2_M:0.0):0.0,CMC-
CR-MDR-Ab4_M:0.0):0.0,MDR-
TJ_M:0.0):0.0,A1_M:0.0):0.0,AB07_M:0.0):0.0,AB34299_M:0.0):0.0,ACICU_M:0.0):0.0,KAB0
3_M:0.0):0.0,XH859_M:0.0):0.0,AC29_M:0.0):0.0,WCHAB005078_M:0.0):0.0,Ab4653_M:0.0
):0.0,AB5075-UW_M:0.0):0.0,HWAB8_M:0.0):0.0,AYP-
A2_M:0.0):0.0,D36_M:0.0):0.0,USA15_M:0.0):0.0,XDR-
BJ83_M:0.0):0.0,R2091_M:0.0):0.0,1656-
2_M:0.0):0.0,AC30_M:0.0):0.0,BJAB0868_M:0.0):0.0,AYE_M:0.0):0.0,XH386_M:0.0):0.0,AR_
0056_M:0.0):0.0,KAB01_M:0.0):0.0,DU202_M:0.0):0.0,AbPK1_M:0.0):0.0,HRAB-
85_M:0.0):0.0,ZW85-1_M:0.0):0.0,CMC-CR-MDR-Ab66_M:0.0):0.0,TCDC-
0715_M:0.0):0.0,AB0057_M:0.0):0.0,A85_M:0.0):0.0,AR_0083_M:0.0):0.0,XH858_M:0.0):0.
0,XH856_M:0.0):0.0,MDR-ZJ06_M:0.0):0.0,XH860_M:0.0):0.0,CMC-MDR-
Ab59_M:0.0):0.0,BJAB07104_M:0.0):0.0,AB307-0294_M:0.0):0.0,AF-
673_M:0.0):0.0,R2090_M:0.0):0.0,BJAB0715_M:0.0):0.0,NCGM237_M:0.0):0.0,A388_M:0.0)
:0.0,CBA7_M:0.0):0.0,AR_0088_M:0.0):0.0,AR_0078_M:0.0):0.0,WCHAB005133_M:0.0):2.0E
-6,15A34_M:2.0E-6)[&Bootstrap=85]:2.0E-6)[&Bootstrap=81]:2.0E-
6)[&Bootstrap=96]:0.038018,((((((((((3207_M:0.0,ab736_M:0.0):0.0,AB042_M:0.0):0.0,ATC
C19606_M:0.0):0.0,WKA02_M:0.0):0.0,AR_0063_M:0.0):0.0,ATCC17978-
mff_M:0.0):0.0,AbH12O-A2_M:0.0):0.0,AF-401_M:0.0):0.0,AR_0101_M:0.0):0.0,Ab04-
mff_M:0.0):2.0E-6,AB030_M:2.0E-6)[&Bootstrap=88]:2.0E-
6)[&Bootstrap=91]:0.105215)[&Bootstrap=88]:0.847763);
end;

```
begin figtree;
	set appearance.backgroundColorAttribute="Default";
	set appearance.backgroundColour=#ffffff;
	set appearance.branchColorAttribute="User selection";
	set appearance.branchColorGradient=false;
	set appearance.branchLineWidth=2.0;
	set appearance.branchMinLineWidth=0.0;
	set appearance.branchWidthAttribute="Fixed";
	set appearance.foregroundColour=#000000;
	set appearance.hilightingGradient=false;
	set appearance.selectionColour=#2d3680;
	set branchLabels.colorAttribute="User selection";
	set branchLabels.displayAttribute="Branch times";
	set branchLabels.fontName="Adobe Devanagari";
	set branchLabels.fontSize=8;
	set branchLabels.fontStyle=0;
	set branchLabels.isShown=false;
	set branchLabels.significantDigits=4;
	set layout.expansion=179;
	set layout.layoutType="RECTILINEAR";
```

```
set layout.zoom=0;
set legend.attribute=null;
set legend.fontSize=10.0;
set legend.isShown=false;
set legend.significantDigits=4;
set nodeBars.barWidth=4.0;
set nodeBars.displayAttribute=null;
set nodeBars.isShown=false;
set nodeLabels.colorAttribute="User selection";
set nodeLabels.displayAttribute="Bootstrap";
set nodeLabels.fontName="Arial";
set nodeLabels.fontSize=10;
set nodeLabels.fontStyle=0;
set nodeLabels.isShown=true;
set nodeLabels.significantDigits=4;
set nodeShapeExternal.colourAttribute=null;
set nodeShapeExternal.isShown=true;
set nodeShapeExternal.minSize=10.0;
set nodeShapeExternal.scaleType=Width;
set nodeShapeExternal.shapeType=Circle;
set nodeShapeExternal.size=5.0;
set nodeShapeExternal.sizeAttribute=null;
set nodeShapeInternal.colourAttribute=null;
set nodeShapeInternal.isShown=false;
set nodeShapeInternal.minSize=10.0;
set nodeShapeInternal.scaleType=Width;
set nodeShapeInternal.shapeType=Circle;
set nodeShapeInternal.size=4.0;
set nodeShapeInternal.sizeAttribute=null;
set polarLayout.alignTipLabels=false;
set polarLayout.angularRange=0;
set polarLayout.rootAngle=0;
set polarLayout.rootLength=100;
set polarLayout.showRoot=true;
set radialLayout.spread=0.0;
set rectilinearLayout.alignTipLabels=false;
set rectilinearLayout.curvature=0;
set rectilinearLayout.rootLength=10000;
set scale.offsetAge=0.0;
set scale.rootAge=1.0;
set scale.scaleFactor=1.0;
set scale.scaleRoot=false;
set scaleAxis.automaticScale=true;
set scaleAxis.fontSize=8.0;
set scaleAxis.isShown=false;
set scaleAxis.lineWidth=1.0;
set scaleAxis.majorTicks=1.0;
```

```
	set scaleAxis.minorTicks=0.5;
	set scaleAxis.origin=0.0;
	set scaleAxis.reverseAxis=false;
	set scaleAxis.showGrid=true;
	set scaleBar.automaticScale=true;
	set scaleBar.fontSize=10.0;
	set scaleBar.isShown=true;
	set scaleBar.lineWidth=1.0;
	set scaleBar.scaleRange=0.0;
	set tipLabels.colorAttribute="User selection";
	set tipLabels.displayAttribute="Names";
	set tipLabels.fontName="Arial";
	set tipLabels.fontSize=12;
	set tipLabels.fontStyle=0;
	set tipLabels.isShown=true;
	set tipLabels.significantDigits=4;
	set trees.order=false;
	set trees.orderType="increasing";
	set trees.rooting=true;
	set trees.rootingType="Midpoint";
	set trees.transform=false;
	set trees.transformType="cladogram";
end;
```
