# Supplementary material for: Systematic Identification and Analysis of Acinetobacter baumannii Type VI Secretion System Effector and Immunity Components
Source: Front Microbiol. 2019 Oct 30;10:2440. doi: 10.3389/fmicb.2019.02440 (PMC6833914; doi:10.3389/fmicb.2019.02440)
Supplement: FILE S4 — Corrected amino acid alignment files (fasta format) used for generating the phylogenetic trees. (a–g) Each sequence has a designated name denoting the strain from where it was derived followed by an underscore then the number of the group the effector region belongs to (based on effector groups, see ML phylogenetic tree shown in Figure 2). (a) Fasta file of A. baumannii T6SS predicted effectors. (b) Fasta file of A. baumannii and other characterized T6SS effectors. (c) Fasta file of A. baumannii RHS pre-D(P/S)xGx cleavage sequences. (d) Fasta file of A. baumannii predicted chaperone proteins. (e) Fasta file of A. baumannii predicted immunity proteins. (f) Fasta file of A. baumannii and A. baylyi VgrG proteins. (g) Fasta file of A. baumannii and A. baylyi PAAR proteins. [file Data_Sheet_4.pdf]

**Supplementary File S4. Corrected alignment files in FastA format.**

**Supplementary file S4a**

```
>ab736_10
---------------------MTQAIQNETRSVRDMLIAAK-----VLAKDGKATESPCKTCRV--------
------------------------------------------------------------------------
---------------------PVWVS---FFFDG------------------------------TGNNKDAD
AATLNQSNVVALFEAHKQDS--------------------------------KNGIEKFYYEGL-GTQFRFD
KYSV---------------------VDSGKITAAARSLQGRKIDITDAEWRKQGYSESGKGVQGALGLGVAL
GIKQRLQK----------------------------------------------------------------
------------AIFELVDYLDKIYTQKGITEINIS----------------------------AFGFSRGA
TEARIFMN-WLQHAPNVTTQGTGSGKKLFYRG----------------------------------------
----KPLKAKFLGI--------------------------------------------FDTVESIGNAAQNK
NP---------------------------------ELYRTRIEDYIEHSMHLVASLEMRQSFPLTPTGKPTA
NTVKGLIHDQKVYPGVHSNVGGGYMPMEQARILGLSRITLHAMYNRACAYGLKFF-----------------
----------------------------------------------------------------------TL
NELNAAKQRK--------------------------------------------------------------
-------------IVFTRFYAFDSKWQQDLNNF---------------------------------------
MAYVKGGT----------------------------------SFEQQMQGQIALY-----------------
-------------------------HQWIREGGYARFIHRKTRERIGRKEKITAITKLNDGLFENIRQALNV
YVPE---------------------------------------------------------GARPYDVIKGR
DRKSTLPKEVIYYFENYVCDSVGGFIAEASDFQAILNDGKAPNYFIPRGIVRPT------------------
----------------------------
>ATCC19606_10
---------------------MTQAIQNETRSVRDMLIAAK-----VLAKDGKATESPCKTCRV--------
------------------------------------------------------------------------
---------------------PVWVS---FFFDG------------------------------TGNNKDAD
AATLNQSNVVALFEAHKQDS--------------------------------KNGIEKFYYEGL-GTQFRFD
KYSV---------------------VDSGKITAAARSLQGRKIDITDAEWRKQGYSESGKGVQGALGLGVAL
GIKQRLQK----------------------------------------------------------------
------------AIFELVDYLDKIYTQKGITEINIS----------------------------AFGFSRGA
TEARIFMN-WLQHAPNVTTQGTGSGKKLFYRG----------------------------------------
----KPLKAKFLGI--------------------------------------------FDTVESIGNAAQNK
```

```
NP---------------------------------ELYRTRIEDYIEHSMHLVASLEMRQSFPLTPTGKPTA
NTVKGLIHDQKVYPGVHSNVGGGYMPMEQARILGLSRITLHAMYNRACAYGLKFF-----------------
----------------------------------------------------------------------TL
NELNAAKQRK--------------------------------------------------------------
-------------IVFTRFYAFDSKWQQDLNNF---------------------------------------
MAYVKGGT----------------------------------SFEQQMQGQIALY-----------------
-------------------------HQWIREGGYARFIHRKTRERIGRKEKITAITKLNDGLFENIRQALNV
YVPE---------------------------------------------------------GARPYDVIKGR
DRKSTLPKEVIYYFENYVCDSVGGFIAEASDFQAILNDGKAPNYFIPRGIVRPT------------------
----------------------------
>AF-401_30
-------------------------------------MNDA-----AIALESFAKQTCGRACVMPPIQP---
------------------------------------------------------------------------
---------------------VVLDV-YKQSFTA------------------------------FVGGAADK
Y----QFLGRPL----------------------------------------DIDFPDDIGAGPTGI-----
-MKS---------------------CMRKFKEASSSQITKAK----FE------------------------
------------------------------------------------------------------------
------------YYGYEEAYSTDSVVKRYRGKLLLN----------------------------LLTDIKNI
LKEEPNTQINLV------------------------------------------------------------
----GHSLGGWNVA-----GLSEELSKAKICTVHTL----------------------ITIDPVGIRLSKSG
VG---------------------------------------------------------------------N
DRARIYYLEPDPVAKKWINIFSQPLKNYRDDYIAI-------------------------------------
----------------------------------------------------------------------LG
GRWNDDDTAK-------------------ANFNSVSTYHHGEA-----------------------------
-----------------SEMFFEKKFLENKMSA---------------------------------------
----------------------------------------SDLLVSELKKVIK-------------------
------------------------------------------------------------------------
------------------------------------------------------------------------
------------------------------------------------------------------------
----------------------------
>3207_1
------------------------MDNVIDANKDIKKQATE-----LLKKAHGKVSNVPPVIPVKLTTYTHS
LTI---------------------------------------------------------------------
```

```
---------------------FIGGA-ADKYVFT------------------------------PGDVPVGW
G--------------DFRPIGPTYFV--------------------------GNYVNNYFATIKGGS-----
------------------------------------------------------------------------
------------------------------------------------------------------------
------------KNAFVKYYGYEEAYVSDTRELTV-----------------------------NTNKDKKL
NAFNDVKNFLKTYPQTQVNIVGH-------------------------------------------------
----SLGGWNAAGL------------------------------------AEILHKNNICKVNVLVTIDPVG
EI---------------------------------------------------------------LSKIGLG
SRTSIYYSRPKPVFNLWISISCDPKSYEWNNDLIAD------------------------------------
----------------------------------------------------------------------LG
GQWSSYPSSN--------------------------------------------------------------
-----------------SSYYYVTRYSHADFRL---------------------------------------
------------------------------------------MMQEKIVNNLSVQ-----------------
------------------------------------------------------------------------
------------------------------------------------------------------------
-----------DILSKELSRIK--------------------------------------------------
----------------------------
>AR_0088_1
------------------------MDNVIDANKDIKKQATE-----LLKKAHGKVSNVPPVIPVKLTTYTHS
LTI---------------------------------------------------------------------
---------------------FIGGA-ADKYVFT------------------------------PGDVPVGW
G--------------DFRPIGPTYFV--------------------------GNYVNNYFATIKGGS-----
------------------------------------------------------------------------
------------------------------------------------------------------------
------------KNAFVKYYGYEEAYVSDTRELTV-----------------------------NTNKDKKL
NAFNDVKNFLKTYPQTQVNIVGH-------------------------------------------------
----SLGGWNAAGL------------------------------------AEILHKNNICKVNVLVTIDPVG
EI---------------------------------------------------------------LSKIGLG
SRTSIYYSRPKPVFNLWISISCDPKSYEWNNDLIAD------------------------------------
----------------------------------------------------------------------LG
GQWSSYPSSN--------------------------------------------------------------
-----------------SSYYYVTRYSHADFRL---------------------------------------
------------------------------------------MMQEKIVNNLSVQ-----------------
```

------------------------------------------------------------------------
------------------------------------------------------------------------
-----------DILSKELSRIK--------------------------------------------------
----------------------------
>HWAB8_1
------------------------MDNVIDANKDIKKQATE-----LLKKAHGKVSNVPPVIPVKLTTYTHS
LTI---------------------------------------------------------------------
---------------------FIGGA-ADKYVFT------------------------------PGDVPVGW
G--------------DFRPIGPTYFV--------------------------GNYVNNYFATIKGGS-----
------------------------------------------------------------------------
------------------------------------------------------------------------
------------KNAFVKYYGYEEAYVSDTRELTV-----------------------------NTNKDKKL
NAFNDVKNFLKTYPQTQVNIVGH-------------------------------------------------
----SLGGWNAAGL------------------------------------AEILHKNNICKVNVLVTIDPVG
EI---------------------------------------------------------------LSKIGLG
SRTSIYYSRPKPVFNLWISISCDPKSYEWNNDLIAD------------------------------------
----------------------------------------------------------------------LG
GQWSSYPSSN--------------------------------------------------------------
-----------------SSYYYVTRYSHADFRL---------------------------------------
------------------------------------------MMQEKIVNNLSVQ-----------------
------------------------------------------------------------------------
------------------------------------------------------------------------
-----------DILSKELSRIK--------------------------------------------------
----------------------------
>AB042_1
------------------------MDNVIDANKDIKKQATE-----LLKKAHGKVSNVPPVIPVKLTTYTHS
LTI---------------------------------------------------------------------
---------------------FIGGA-ADKYVFT------------------------------PGDVPVGW
G--------------DFRPIGPTYFV--------------------------GNYVNNYFATIKGGS-----
------------------------------------------------------------------------
------------------------------------------------------------------------
------------KNAFVKYYGYEEAYVSDTRELTV-----------------------------NTNKDKKL
NAFNDVKNFLKTYPQTQVNIVGH-------------------------------------------------

```
----SLGGWNAAGL------------------------------------AEILHKNNICKVNVLITIDPVG
EI---------------------------------------------------------------LSKIGLG
SRTSIYYSRPKPVFNLWISISCDPKSYEWNNDLIAD------------------------------------
----------------------------------------------------------------------LG
GQWSSYPSSN--------------------------------------------------------------
-----------------SSYYYVTRYSHADFRL---------------------------------------
------------------------------------------MMQEKIVNNLSVQ-----------------
------------------------------------------------------------------------
------------------------------------------------------------------------
-----------DILSKELSRIK--------------------------------------------------
----------------------------
>ATCC17978-mff_1
------------------------MDNVIDANKDIKKQATE-----LLKKAHGKVSNVPPVIPVKLTTYTHS
LTI---------------------------------------------------------------------
---------------------FIGGA-ADKYVFT------------------------------PGDVPVGW
G--------------DFRPIGPTYFV--------------------------GNYVNNYFATIKGGS-----
------------------------------------------------------------------------
------------------------------------------------------------------------
------------KNAFVKYYGYEEAYVSDTRELTV-----------------------------NTNKDKKL
NAFNDVKNFLKTYPQTQVNIVGH-------------------------------------------------
----SLGGWNAAGL------------------------------------AEILHKNNICKVNVLITIDPVG
EI---------------------------------------------------------------LSKIGLG
SRTSIYYSRPKPVFNLWISISCDPKSYEWNNDLIAD------------------------------------
----------------------------------------------------------------------LG
GQWSSYPSSN--------------------------------------------------------------
-----------------SSYYYVTRYSHADFRL---------------------------------------
------------------------------------------MMQEKIVNNLSVQ-----------------
------------------------------------------------------------------------
------------------------------------------------------------------------
-----------DILSKELSRIK--------------------------------------------------
----------------------------
>ATCC17978-mff_2
-------------------------MPDPQNQQFWSKIAER-----PTEIQQQFWYGAEKLAKYLNDNP---
```

```
------------------------------------------------------------------------
---------------------AMYKG-YMTYDTS------------------------------KPDPNLSW
WEKILFYSGQAHYNEAVTHYNTSKVI--------------------------ADQGSWLWGMLK-GD-----
-FNKDP-------------------SMSQIIVGGLISLIPVA----DQVCDIRDLIANFITLSDEKARTKDN
YMALALTSVG--------------------------------------------------------------
------------IIPEVGSAIKTIVKSS------------------------------------RAKDVSKV
KLFKFME--YFEEALTKLKIKCPWGKAPEAWLRSRPWKGIATQAFNTLKNNIDRILSVINKCLIRFNGALKV
ALAKFQATLNHVLN-----TIKKYIDQLCDEVDKAI------------------GRLLPQQPLAMAHAGGGG
KN----------------------------------APTGRYEANVSSGKKTEASHRQKKETPPPPRKMKPK
HVVEPCLEPKKGLRNSWGKKTGKSQAELDAEFDRQLKRQEEGLNRLTVEEYQ--------------------
----------------------------------------------------------------------QN
RQLYEKYKRA--------------------------------------------------------------
-----------------GTGTQQQRIREDMQRQ---------------------------------------
LEESEYKKL---------------------------------KTEQPQLSKGQIE-----------------
-------RMAENNAKKTLEGLDVLHNPDMQLGGFDVKYDPKKPPTLDDFGH---------------------
-------------------------------------------------------------SGVNRSIGSQM
AAKKRLANMDAAAAQAKAKGMGKDAMDVELKRCK--------------------------------------
----------------------------
>CIP70.10_2
-------------------------MPDPQNQQFWSKIAER-----PTEIQQQFWYGAEKLAKYLNDNP---
------------------------------------------------------------------------
---------------------AMYKG-YMTYDTS------------------------------KPDPNLSW
WEKILFYSGQAHYNEAVTHYNTSKVI--------------------------ADQGSWLWGMLK-GD-----
-FNKDP-------------------SMSQIIVGGLISLIPVA----DQVCDIRDLIANFITLSDEKARTKDN
YMALALTSVG--------------------------------------------------------------
------------IIPEVGSAIKTIVKSS------------------------------------RAKDVSKV
KLFKFME--YFEEALTKLKIKCPWGKAPEAWLRSRPWKGIATQAFNTLKNNIDRILSVINKCLIRFNGALKV
ALAKFQATLNHVLN-----TIKKYIDQLCDEVDKAI------------------GRLLPQQPLAMAHAGGGG
KN----------------------------------APTGRYEANVSSGKKTEASHRQKKETPPPPRKMKPK
HVVEPCLEPKKGLRNSWGKKTGKSQAELDAEFDRQLKRQEEGLNRLTVEEYQ--------------------
----------------------------------------------------------------------QN
RQLYEKYKRA--------------------------------------------------------------
-----------------GTGTQQQRIREDMQRQ---------------------------------------
```

```
LEESEYKKL---------------------------------KTEQPQLSKGQIE-----------------
-------RMAENNAKKTLEGLDVLHNPDMQLGGFDVKYDPKKPPTLDDFGH---------------------
-------------------------------------------------------------SGVNRSIGSQM
AAKKRLANMDAAAAQAKAKGMGKDAMDVELKRCK--------------------------------------
----------------------------
>R2091_2
-------------------------MPDPQNQQFWSKIAER-----PTEIQQQFWYGAEKLAKYLNDNP---
------------------------------------------------------------------------
---------------------AMYKG-YMTYDTS------------------------------KPDPNLSW
WEKILFYSGQAHYNEAVTHYNTSKVI--------------------------ADQGSWLWGMLK-GD-----
-FNKDP-------------------SMSQIIVGGLISLIPVA----DQVCDIRDLIANFITLSDEKARTKDN
YMALALTSVG--------------------------------------------------------------
------------IIPEVGSAIKTIVKSS------------------------------------RAKDVSKV
KLFKFME--YFEEALTKLKIKCPWGKAPEAWLRSRPWKGIATQAFNTLKNNIDRILSVINKCLIRFNGALKV
ALAKFQATLNHVLN-----TIKKYIDQLCDEVDKAI------------------GRLLPQQPLAMAHAGGGG
KN----------------------------------APTGRYEANVSSGKKTEASHRQKKETPPPPRKMKPK
HVVEPCLEPKKGLRNSWGKKTGKSQAELDAEFDRQLKRQEEGLNRLTVEEYQ--------------------
----------------------------------------------------------------------QN
RQLYEKYKRA--------------------------------------------------------------
-----------------GTGTQQQRIREDMQRQ---------------------------------------
LEESEYKKL---------------------------------KTEQPQLSKGQIE-----------------
-------RMAENNAKKTLEGLDVLHNPDMQLGGFDVKYDPKKPPTLDDFGH---------------------
-------------------------------------------------------------SGVNRSIGSQM
AAKKRLANMDAAAAQAKAKGMGKDAMDVELKRCK--------------------------------------
----------------------------
>ZW85-1_19
------------------------------VTDFLPSKQDL-----LNALATISSPINKAIKKGQETARDVA
D-----------------------------------------------------------------------
---------------------WLWVVIQGDFAEE------------------------------QTTAQTVT
G----TVISMIPFVDQICDVRDICA---------------------------NCIKIKEDSNNP--------
-WPW---------------------IGLILTLIGLFPVLGSL----FKGIFKVI------------------
------------------------------------------------------------------------
------------LAPVRRFMLRPATKALKFTGGNIY----------------------------KLAEPAIE
```

SGITEMNKFLARPAVKRTIKNSNITNIYKAIAK---------------------------------KIREVK
AGTNKQALLSVFDI-------LIDHLKESVKFIEKY---------------GSKAIGVKAKQMLQSVLDIRN
LA----------------------------------NQKLGEFLKPVQDFLEKLAVRIEKEGDQTFKATTNV
KNIHNFQRIGQNAELKSVLNNKPVWVDIVAKEKFKALSESPLIPKNYPD-----------------------
----------------------------------------------------------------------IS
EMSKNRALRD-------------------AFKTFHDLEPVHLSEGE--------------------------
-----------------ILYRVLDPKSADNSIC---------------------------------------
WMRASEYK----------------------------------KLKSKAD-----------------------
-------WRRYFAVFASWNNNGEVVKYRVPKGGMNVWEGPAASQTFKNSAG---------------------
-------------------------------------KVEKVNSKGEIFVLEGGG------IQIVLDPNDLV
RSNVSKREATPWGYDSGLIGEAKTSMVGVPRLEQNWYGDKK-------------------------------
----------------------------
>ab736_9
--------------------------------------MNK-----LVTITTKFYDKSGNRI----------
------------------------------------------------------------------------
---------------------------INLNVQS------------------------------RYKGSLKA
NSQKTDKLGLFVFQASPNRTVEILAKPP------------------------NQKDYTVFKTIN-SSMNSSE
IHPI---------------------KVQLPKTIDEYKQVKQS----KSTKGIVSTFFKIFDMNGKVMKNFPI
QSRPKGKG----------------------------------------------------------------
------------NSPDKYTNDEGIVEVRSSPNRDIEV---------------------------LVLTSNDT
FVLKSSINSANGSSQPVLIKLDEPYEKFKSASTIKILDR-------------DGSDYIVEKTNVKMLVVENG
KKQLFSISNGKLSL-----------------------------------------QSMIGQKLEFTVYKPDG
KP----------------------------LKTQTYMATRVKNNLIEFHLDVDITKGSTAQNDPEINKKVKV
DILITMEQMKKMWPKALATKMQPILDELNSDLLGYKLDTR--------------------------------
----------------------------------------------------------------------LR
QAHFMAQVRQ-----------------EVGSSFSLREQVEYMG-----------------------------
-----------------PTALKQIGYYRTHPKQ---------------------------------------
------------------------------------------AEIDGYKRG---------------------
----------QGPANGEVIANRMYDDNYRSAKYKLGNTSPGDGWKYL------GRGLKQLTGKNNYQDLTNM
YSTLWP---------------------------GEKVDFVKNPELIEQPKYAVRSAIRFWLKFKLYEVADRG
ANGEQVDAITKVINEATNSYADRRAHFVQARKIFI-------------------------------------
----------------------------
>ATCC19606_9

--------------------------------------MNK-----LVTITTKFYDKSGNRI----------
------------------------------------------------------------------------
---------------------------INLNVQS------------------------------RYKGSLKA
NSQKTDKLGLFVFQASPNRTVEILAKPP------------------------NQKDYTVFKTIN-SSMNSSE
IHPI---------------------KVQLPKTIDEYKQVKQS----KSTKGIVSTFFKIFDMNGKVMKNFPI
QSRPKGKG----------------------------------------------------------------
------------NSPDKYTNDEGIVEVRSSPNRDIEV---------------------------LVLTSNDT
FVLKSSINSANGSSQPVLIKLDEPYEKFKSASTIKILDR-------------DGSDYIVEKTNVKMLVVENG
KKQLFSISNGKLSL-----------------------------------------QSMIGQKLEFTVYKPDG
KP----------------------------LKTQTYMATRVKNNLIEFHLDVDITKGSTAQNDPEINKKVKV
DILITMEQMKKMWPKALATKMQPILDELNSDLLGYKLDTR--------------------------------
----------------------------------------------------------------------LR
QAHFMAQVRQ-----------------EVGSSFSLREQVEYMG-----------------------------
-----------------PTALKQIGYYRTHPKQ---------------------------------------
------------------------------------------AEIDGYKRG---------------------
----------QGPANGEVIANRMYDDNYRSAKYKLGNTSPGDGWKYL------GRGLKQLTGKNNYQDLTNM
YSTLWP---------------------------GEKVDFVKNPELIEQPKYAVRSAIRFWLKFKLYEVADRG
ANGEQVDAITKVINEATNSYADRRAHFVQARKIFI-------------------------------------
----------------------------
>Ab04-mff_11
------------------------MSYSEDFKIFVATINGE-----AENCSETSWKVIAHTMRNRVGFANW-
------------------------------------------------------------------------
---------------------KSWST-IIQIVTK------------------------------TGYDAYTQ
KNSPYKRAKKALDSGDISPKLMSLIKAVEPIFNGTEPDFTGGVVYYYSPKAQAQLHKSDPSNYP-SLVPDFV
VSKS---------------------NPTQQVQIPGTEKDDMR----WYKVLTSIFDVSFVDNAGNPLVGVTV
DVVYNDKKPVP-------------------------------------------------------------
------------LFKDLITDQKGKIKKVRVCNYMGARFKVNGVLVKDKNNKEISLLGDGKNYASVIVVNNGK
GGIKSKTDIHNQESIPGQKQESVKIDSKAEVNNEKKAEVSDNNTKQKNVNFSIKVIDSENKAIPNFSYFLKY
KNAEKKHSVGANGI-------ENNLVALSGEEITVLISGLDSKQEIIRFTAQEGMGEKTIKLNLHTFNILFR
HKDTKKPITNLNLIQKYRN----QIKQKKTDGNGKITVSAMPGFELNYKLRDERNLLTIKVDKNKSLRVIDV
DSSAIEQASKNIKIGTKVVEIAQSKQPVPSSKQKHPVETHDSTPKRDEKVKISTDGHPKTLVNDN-------
----------------------------------------------------------------------GE
TEFIVYTYDQ-------------------KTNQLFSGGNYSIE-----------------------------

----YKGNKKRHSSGIHGIGKKIHKGEIGQKIK---------------------------------------
ITASSGGK---------------------------EFIAFDGNLSRGMKAFELKIDRSVIPSVSDVIISFKG
VNEQSRQAIVSQKTKNVLAYLAKEANMDKLYITSTIRTPRAQAEAMYIKATKYAKPGEDVKKVKAECI----
-----------------------------AKGLGKEATIQKMVEKIVEFQNKGVRVSKHCVSEEQYRKNNII
DLGTNSNGFGTGNTLNSVGKRFKAVCEKALKDGIISGFISADVAGEGAMHIEIIQ-----------------
----------------------------

>BJAB0715_11

------------------------MSYSEDFKIFVATINGE-----AENCSETSWKVIAHTMRNRVGFANW-
------------------------------------------------------------------------
---------------------KSWST-IIQIVTK------------------------------TGYDAYTQ
KNSPYKRAKKALDSGDISPKLMSLIKAVEPIFNGTEPDFTGGVVYYYSPKAQAQLHKSDPSNYP-SLVPDFV
VSKS---------------------NPTQQVQIPGTEKDDMR----WYKVLTSIFDVSFVDNAGNPLVGVTV
DVVYNDKKPVP-------------------------------------------------------------
------------LFKDLITDQKGKIKKVRVCNYMGARFKVNGVLVKDKNNKEISLLGDGKNYASVIVVNNGK
GGIKSKTDIHNQESIPGQKQESVKIDSKAEVNNEKKAEVSDNNTKQKNVNFSIKVIDSENKAIPNFSYFLKY
KNAEKKHSVGANGI-------ENNLVALSGEEITVLISGLDSKQEIIRFTAQEGMGEKTIKLNLHTFNILFR
HKDTKKPITNLNLIQKYRN----QIKQKKTDGNGKITVSAMPGFELNYKLRDERNLLTIKVDKNKSLRVIDV
DSSAIEQASKNIKIGTKVVEIAQSKQPVPSSKQKHPVETHDSTPKRDEKVKISTDGHPKTLVNDN-------
----------------------------------------------------------------------GE
TEFIVYTYDQ-------------------KTNQLFSGGNYSIE-----------------------------
----YKGNKKRHSSGIHGIGKKIHKGEIGQKIK---------------------------------------
ITASSGGK---------------------------EFIAFDGNLSRGMKAFELKIDRSVIPSVSDVIISFKG
VNEQSRQAIVSQKTKNVLAYLAKEANMDKLYITSTIRTPRAQAEAMYIKATKYAKPGEDVKKVKAECI----
-----------------------------AKGLGKEATIQKMVEKIVEFQNKGVRVSKHCVSEEQYRKNNII
DLGTNSNGFGTGNTLNSVGKRFKAVCEKALKDGIISGFISADVAGEGAMHIEIIQ-----------------
----------------------------

>LAC-4_11

------------------------MSYSEDFKIFVATINGE-----AENCSETSWKVIAHTMRNRVGFANW-
------------------------------------------------------------------------
---------------------KSWST-IIQIVTK------------------------------TGYDAYTQ
KNSPYKRAKKALDSGDISPKLMSLIKAVEPIFNGTEPDFTGGVVYYYSPKAQAQLHKSDPSNYP-SLVPDFV
VSKS---------------------NPTQQVQIPGTEKDDMR----WYKVLTSIFDVSFVDNAGNPLVGVTV
DVVYNDKKPVP-------------------------------------------------------------

```
------------LFKDLITDQKGKIKKVRVCNYMGARFKVNGVLVKDKNNKEISLLGDGKNYASVIVVNNGK
GGIKSKTDIHNQESIPGQKQESVKIDSKAEVNNEKKAEVSDNNTKQKNVNFSIKVIDSENKAIPNFSYFLKY
KNAEKKHSVGANGI-------ENNLVALSGEEITVLISGLDSKQEIIRFTAQEGMGEKTIKLNLHTFNILFR
HKDTKKPITNLNLIQKYRN----QIKQKKTDGNGKITVSAMPGFELNYKLRDERNLLTIKVDKNKSLRVIDV
DSSAIEQASKNIKIGTKVVEIAQSKQPVPSSKQKHPVETHDSTPKRDEKVKISTDGHPKTLVNDN-------
----------------------------------------------------------------------GE
TEFIVYTYDQ-------------------KTNQLFSGGNYSIE-----------------------------
----YKGNKKRHSSGIHGIGKKIHKGEIGQKIK---------------------------------------
ITASSGGK---------------------------EFIAFDGNLSRGMKAFELKIDRSVIPSVSDVIISFKG
VNEQSRQAIVSQKTKNVLAYLAKEANMDKLYITSTIRTPRAQAEAMYIKATKYAKPGEDVKKVKAECI----
-----------------------------AKGLGKEATIQKMVEKIVEFQNKGVRVSKHCVSEEQYRKNNII
DLGTNSNGFGTGNTLNSVGKRFKAVCEKALKDGIISGFISADVAGEGAMHIEIIQ-----------------
----------------------------
>WKA02_11
------------------------MSYSEDFKIFVATINGE-----AENCSETSWKVIAHTMRNRVGFANW-
------------------------------------------------------------------------
---------------------KSWST-IIQIVTK------------------------------TGYDAYTQ
KNSPYKRAKKALDSGDISPKLMSLIKAVEPIFNGTEPDFTGGVVYYYSPKAQAQLHKSDPSNYP-SLVPDFV
VSKS---------------------NPTQQVQIPGTEKDDMR----WYKVLTSIFDVSFVDNAGNPLVGVTV
DVVYNDKKPVP-------------------------------------------------------------
------------LFKDLITDQKGKIKKVRVCNYMGARFKVNGVLVKDKNNKEISLLGDGKNYASVIVVNNGK
GGIKSKTDIHNQESIPGQKQESVKIDSKAEVNNEKKAEVSDNNTKQKNVNFSIKVIDSENKAIPNFSYFLKY
KNAEKKHSVGANGI-------ENNLVALSGEEITVLISGLDSKQEIIRFTAQEGMGEKTIKLNLHTFNILFR
HKDTKKPITNLNLIQKYRN----QIKQKKTDGNGKITVSAMPGFELNYKLRDERNLLTIKVDKNKSLRVIDV
DSSAIEQASKNIKIGTKVVEIAQSKQPVPSSKQKHPVETHDSTPKRDEKVKISTDGHPKTLVNDN-------
----------------------------------------------------------------------GE
TEFIVYTYDQ-------------------KTNQLFSGGNYSIE-----------------------------
----YKGNKKRHSSGIHGIGKKIHKGEIGQKIK---------------------------------------
ITASSGGK---------------------------EFIAFDGNLSRGMKAFELKIDRSVIPSVSDVIISFKG
VNEQSRQAIVSQKTKNVLAYLAKEANMDKLYITSTIRTPRAQAEAMYIKATKYAKPGEDVKKVKAECI----
-----------------------------AKGLGKEATIQKMVEKIVEFQNKGVRVSKHCVSEEQYRKNNII
DLGTNSNGFGTGNTLNSVGKRFKAVCEKALKDGIISGFISADVAGEGAMHIEIIQ-----------------
----------------------------
```

>XH858_11
------------------------MSYSEDFKIFVATINGE-----AENCSETSWKVIAHTMRNRVGFANW-
------------------------------------------------------------------------
---------------------KSWST-IIQIVTK------------------------------TGYDAYTQ
KNSPYKRAKKALDSGDISPKLMSLIKAVEPIFNGTEPDFTGGVVYYYSPKAQAQLHKSDPSNYP-SLVPDFV
VSKS---------------------NPTQQVQIPGTEKDDMR----WYKVLTSIFDVSFVDNAGNPLVGVTV
DVVYNDKKPVP-------------------------------------------------------------
------------LFKDLITDQKGKIKKVRVCNYMGARFKVNGVLVKDKNNKEISLLGDGKNYASVIVVNNGK
GGIKSKTDIHNQESIPGQKQESVKIDSKAEVNNEKKAEVSDNNTKQKNVNFSIKVIDSENKAIPNFSYFLKY
KNAEKKHSVGANGI-------ENNLVALSGEEITVLISGLDSKQEIIRFTAQEGMGEKTIKLNLHTFNILFR
HKDTKKPITNLNLIQKYRN----QIKQKKTDGNGKITVSAMPGFELNYKLRDERNLLTIKVDKNKSLRVIDV
DSSAIEQASKNIKIGTKVVEIAQSKQPVPSSKQKHPVETHDSTPKRDEKVKISTDGHPKTLVNDN-------
----------------------------------------------------------------------GE
TEFIVYTYDQ-------------------KTNQLFSGGNYSIE-----------------------------
----YKGNKKRHSSGIHGIGKKIHKGEIGQKIK---------------------------------------
ITASSGGK---------------------------EFIAFDGNLSRGMKAFELKIDRSVIPSVSDVIISFKG
VNEQSRQAIVSQKTKNVLAYLAKEANMDKLYITSTIRTPRAQAEAMYIKATKYAKPGEDVKKVKAECI----
-----------------------------AKGLGKEATIQKMVEKIVEFQNKGVRVSKHCVSEEQYRKNNII
DLGTNSNGFGTGNTLNSVGKRFKAVCEKALKDGIISGFISADVAGEGAMHIEIIQ-----------------
----------------------------
>AB030_24
--MKNGKSINQKSYPLTRYGFICAVSRMESSSDLSLPLTAP-----VNIRASQNKNSRGYIGFFQFGEAA--
------------------------------------------------------------------------
---------------------LIDLGYYKHWNDN-----------------------------SDKTKANDW
TGSWVGHNGVNSLSDFLKSPAKQIQIIGEWIDLLCKRLR-------------NRSFNEYYGKIINGI-----
-------------------------EITESGAIAGAHLVGEGGLGSFLGISGFKGNYKEVDGNNVHISRYIE
MFNHYDLE----------------------------------------------------------SCCSRK
IYII--LKNQIGQIAKNKKVTIESEYSGKFKQS-------------------------------KFIVSAES
DEQGLLPVIIRHPGSKIIIKVDGKQSAVITQASDKKQSIELSVSEDIKIQSVLQKPAEPEPKQEKVVVVEQK
TVDLKKDEVKTNAP-------KDIKFDIAIIESDTNRKITNMRFFIRYKGKIKEHTSDSSGIKTGIIAEEGE
NL-------DILVNGSKGY---QKLKTISITKGMENSCISVPLGLVSVKLKIHDSKGKILKNNKFFVSYRGR
EIEKITDSNGFIQLKMLNAFVYKLLLSNKKPILTLRNDPSISVINVNLNSAATTIQ----------------
----------------------------------------------------------------------MP

RVASNPPSKT-------------------VPAKTKPAKE---------------------------------
-----------------TSSKREESFVDYLIDF----------------------------------IPSLG
KTENVHTE----------------------------------KNGNPLTKQYGSDVVFTIKTINKETGKEEN
LAYSIKYNGAKRTHYTGQDGIGLKKHRGEEGKKIEIVVDVDSKEQVLYTAILKNPMPVIEIRMDKPKDDDSY
LFPLKARTNSYKNGFARFGSNRSGGKRKHAGCDLYAPTGTEIRAMADGKVRVVKGFYSGTDVIEIVHKKHII
RYGEVLAGKSLVKVGDDVKRGQTIGYVGKLTVQVPSMMLHLEMYSNPKDTSPLTVRGNNAYQRRSDLIDPTT
FLDNSTL---------------------
>AbH12O-A2_24
--MKNGKSINQKSYPLTRYGFICAVSRMESSSDLSLPLTAP-----VNIRASQNKNSRGYIGFFQFGEAA--
------------------------------------------------------------------------
---------------------LIDLGYYKHWNDN-----------------------------SDKTKANDW
TGSWVGHNGVNSLSDFLKSPAKQIQIIGEWIDLLCKRLR-------------NRSFNEYYGKIINGI-----
-------------------------EITESGAIAGAHLVGEGGLGSFLGISGFKGNYKEVDGNNVHISRYIE
MFNHYDLE----------------------------------------------------------SCCSRK
IYII--LKNQIGQIAKNKKVTIESEYSGKFKQS-------------------------------KFIVSAES
DEQGLLPVIIRHPGSKIIIKVDGKQSAVITQASDKKQSIELSVSEDIKIQSVLQKPAEPEPKQEKVVVVEQK
TVDLKKDEVKTNAP-------KDIKFDIAIIESDTNRKITNMRFFIRYKGKIKEHTSDSSGIKTGIIAEEGE
NL-------DILVNGSKGY---QKLKTISITKGMENSCISVPLGLVSVKLKIHDSKGKILKNNKFFVSYRGR
EIEKITDSNGFIQLKMLNAFVYKLLLSNKKPILTLRNDPSISVINVNLNSAATTIQ----------------
----------------------------------------------------------------------MP
RVASNPPSKT-------------------VPAKTKPAKE---------------------------------
-----------------TSSKREESFVDYLIDF----------------------------------IPSLG
KTENVHTE----------------------------------KNGNPLTKQYGSDVVFTIKTINKETGKEEN
LAYSIKYNGAKRTHYTGQDGIGLKKHRGEEGKKIEIVVDVDSKEQVLYTAILKNPMPVIEIRMDKPKDDDSY
LFPLKARTNSYKNGFARFGSNRSGGKRKHAGCDLYAPTGTEIRAMADGKVRVVKGFYSGTDVIEIVHKKHII
RYGEVLAGKSLVKVGDDVKRGQTIGYVGKLTVQVPSMMLHLEMYSNPKDTSPLTVRGNNAYQRRSDLIDPTT
FLDNSTL---------------------
>AF-401_24
--MKNGKSINQKSYPLTRYGFICAVSRMESSSDLSLPLTAP-----VNIRASQNKNSRGYIGFFQFGEAA--
------------------------------------------------------------------------
---------------------LIDLGYYKHWNDN-----------------------------SDKTKANDW
TGSWVGHNGVNSLSDFLKSPAKQIQIIGEWIDLLCKRLR-------------NRSFNEYYGKIINGI-----
-------------------------EITESGAIAGAHLVGEGGLGSFLGISGFKGNYKEVDGNNVHISRYIE

MFNHYDLE----------------------------------------------------------SCCSRK
IYII--LKNQIGQIAKNKKVTIESEYSGKFKQS-------------------------------KFIVSAES
DEQGLLPVIIRHPGSKIIIKVDGKQSAVITQASDKKQSIELSVSEDIKIQSVLQKPAEPEPKQEKVVVVEQK
TVDLKKDEVKTNAP-------KDIKFDIAIIESDTNRKITNMRFFIRYKGKIKEHTSDSSGIKTGIIAEEGE
NL-------DILVNGSKGY---QKLKTISITKGMENSCISVPLGLVSVKLKIHDSKGKILKNNKFFVSYRGR
EIEKITDSNGFIQLKMLNAFVYKLLLSNKKPILTLRNDPSISVINVNLNSAATTIQ----------------
----------------------------------------------------------------------MP
RVASNPPSKT-------------------VPAKTKPAKE---------------------------------
-----------------TSSKREESFVDYLIDF----------------------------------IPSLG
KTENVHTE----------------------------------KNGNPLTKQYGSDVVFTIKTINKETGKEEN
LAYSIKYNGAKRTHYTGQDGIGLKKHRGEEGKKIEIVVDVDSKEQVLYTAILKNPMPVIEIRMDKPKDDDSY
LFPLKARTNSYKNGFARFGSNRSGGKRKHAGCDLYAPTGTEIRAMADGKVRVVKGFYSGTDVIEIVHKKHII
RYGEVLAGKSLVKVGDDVKRGQTIGYVGKLTVQVPSMMLHLEMYSNPKDTSPLTVRGNNAYQRRSDLIDPTT
FLDNSTL---------------------
>AR_0101_24
--MKNGKSINQKSYPLTRYGFICAVSRMESSSDLSLPLTAP-----VNIRASQNKNSRGYIGFFQFGEAA--
------------------------------------------------------------------------
---------------------LIDLGYYKHWNDN-----------------------------SDKTKANDW
TGSWVGHNGVNSLSDFLKSPAKQIQIIGEWIDLLCKRLR-------------NRSFNEYYGKIINGI-----
-------------------------EITESGAIAGAHLVGEGGLGSFLGISGFKGNYKEVDGNNVHISRYIE
MFNHYDLE----------------------------------------------------------SCCSRK
IYII--LKNQIGQIAKNKKVTIESEYSGKFKQS-------------------------------KFIVSAES
DEQGLLPVIIRHPGSKIIIKVDGKQSAVITQASDKKQSIELSVSEDIKIQSVLQKPAEPEPKQEKVVVVEQK
TVDLKKDEVKTNAP-------KDIKFDIAIIESDTNRKITNMRFFIRYKGKIKEHTSDSSGIKTGIIAEEGE
NL-------DILVNGSKGY---QKLKTISITKGMENSCISVPLGLVSVKLKIHDSKGKILKNNKFFVSYRGR
EIEKITDSNGFIQLKMLNAFVYKLLLSNKKPILTLRNDPSISVINVNLNSAATTIQ----------------
----------------------------------------------------------------------MP
RVASNPPSKT-------------------VPAKTKPAKE---------------------------------
-----------------TSSKREESFVDYLIDF----------------------------------IPSLG
KTENVHTE----------------------------------KNGNPLTKQYGSDVVFTIKTINKETGKEEN
LAYSIKYNGAKRTHYTGQDGIGLKKHRGEEGKKIEIVVDVDSKEQVLYTAILKNPMPVIEIRMDKPKDDDSY
LFPLKARTNSYKNGFARFGSNRSGGKRKHAGCDLYAPTGTEIRAMADGKVRVVKGFYSGTDVIEIVHKKHII
RYGEVLAGKSLVKVGDDVKRGQTIGYVGKLTVQVPSMMLHLEMYSNPKDTSPLTVRGNNAYQRRSDLIDPTT

FLDNSTL---------------------
>AR_0063_24
--MKNGKSINQKSYPLTRYGFICAVSRMESSSDLSLPLTAP-----VNIRASQNKNSRGYIGFFQFGEAA--
------------------------------------------------------------------------
---------------------LIDLGYYKHWNDN-----------------------------SDKTKANDW
TGSWVGHNGVNSLSDFLKSPAKQIQIIGEWIDLLCKRLR-------------NRSFNEYYGKIINGI-----
-------------------------EITESGAIAGAHLVGEGGLGSFLGISGFKGNYKEVDGNNVHISRYIE
MFNHYDLE----------------------------------------------------------SCCSRK
IYIILLLKNQIGQIAKNKKVTIESEYSGKFKQS-------------------------------KFIVSAES
DEQGLLPVIIRHPGSKIIIKVDGKQSAVITQASDKKQSIELSVSEDIKIQSVLQKPAEPEPKQEKVVVVEQK
TVDLKKDEVKTNAP-------KDIKFDIAIIESDTNRKITNMRFFIRYKGKIKEHTSDSSGIKTGIIAEEGE
NL-------DILVNGSKGY---QKLKTISITKGMENSCISVPLGLVSVKLKIHDSKGKILKNNKFFVSYRGR
EIEKITDSNGFIQLKMLNAFVYKLLLSNKKPILTLRNDPSISVINVNLNSAATTIQ----------------
----------------------------------------------------------------------MP
RVASNPPSKT-------------------VPAKTKPAKE---------------------------------
-----------------TSSKREESFVDYLIDF----------------------------------IPSLG
KTENVHTE----------------------------------KNGNPLTKQYGSDVVFTIKTINKETGKEEN
LAYSIKYNGAKRTHYTGQDGIGLKKHRGEEGKKIEIVVDVDSKEQVLYTAILKNPMPVIEIRMDKPKDDDSY
LFPLKARTNSYKNGFARFGSNRSGGKRKHAGCDLYAPTGTEIRAMADGKVRVVKGFYSGTDVIEIVHKKHII
RYGEVLAGKSLVKVGDDVKRGQTIGYVGKLTVQVPSMMLHLEMYSNPKDTSPLTVRGNNAYQRRSDLIDPTT
FLDNSTL---------------------
>AB031_25
MKKTIKFEKDSKSFETNLAGFIEAVSFKESSSNLLLPLTHP-----INMEAASITNEMGFIGLFQWGEEALY
DLGYYLGDQGISYADIKNTDKNSSLRISWKNQFNSNNWVGKWSGKRNINSKKDFLKHPEIQYEIIKEWIKYL
CNQLRNNNFNEYFGTTIQNIEITESG-AIAGMHL------------------------------VGIGGLGA
F----LGIPKFHGKKQTDGNGTHIKK--------------------------YIENFGGFDLEQ-CC-----
-NRK---------------------IYVTLKDNLGLELKDKE----VIIVSQNKGKYVSGETKVKVKSDENG
NLPVIVRS---------------------------------------------------PNTEIKICADGKE
SNTI--------IQKANEKQKAILSDFKVSSHP-------------------------------ATLEKNST
PQPKPQTNKTPQEVRNEQTQPSTAPEKEKDSSSKDVDFNIQIVEGDS-------------------GKAISN
M---NFFITYKGNI-------KKHTAD-GHGIKQNII--------------AEIGQDIEVSVQGTGHKQVIH
HF-------TANAA----------------------LKNKTVKVSLPVHSFNISVTQDNKPVPNTLFSIFYR
GREISKRTDSRGMLNVRMLTGFVFGFGIKGKSLILSRVEKNTITKA--FTVNGSAVHASKAYEANDAK----

------------------QKQNENLNKHKKEKQEEAAKKQADQAKTQKNSKNEVKQSNTYTENGGKPLTTVS
NQASVTSDTT-------------------NYIIYPNGTIDRINKD---------------------------
-----------------ATGFAAYYYVANNQPI---------------------------------------
LIGKSRIY---------------------------T----ADRWLKKGKKGSGISHLVS-IREIFGDSGSKP
NNKWEKKIGNLNISIHNDTQQQHRYWLSTVAFAAYIGAVCKFG--EKVRFS--GFSDRNGSPGGSSSHLNGE
VGDIGY----------IRVDRNNSVGVNFEMADYDHDASLRFVNILISFGWGKTK------NMLSEHYPAHL
VNKYKLNQGYILPRCSQWTNPRHNNHLHLQGLSVTFNKDE--------------------------------
----------------------------

>6200_17
----------MNKKGLIQFRFVELFTGNNIPNLYHVIKNEK-----GTTIASGMTNSNGLTVMISR------
------------------------------------------------------------------------
---------------------DVGDT-LYVYLKN------------------------------IITGDLKE
K----VRHTVIYKKEIVRVISSKILL--------------------------DNIILAKSTNKP-GN-----
-YRY---------------------LTHKVKKGENLSSISHR----FNCKTSEIVHLNKLKNPDHIDVGQII
KI----------------------------------------------------------------PYKGES
SDSK--------KSPDNKQSKHENNKSSSTQKVN------------------------------SHENNNKN
TSDKPQKRIESKDITDEYTKESGKPMKVATNAT---------------------------------SPCICK
Q---YNLAWGSKVS-------CEFRKRVIKIAQNLW---------------PNDSENMASQLMAVMHLESAG
TF-------SPKIGTFISK----KLTD---------DAKGGYVGLIQFGKFASIDLKVKRSDLAKMSAVQQL
DYVEKYYKLNSAHTKIKNLTGLYLWVNYPKNVKENRLEDEDIVYAAPKDAEV--------------------
----------------------------------------------------------------------TS
KKFLESPYHQ-------------------NPSFMKENEEYKRD-----------------------------
-----------------GKKVIRQGFKNGSTKV---------------------------------------
WEVEQEIK---------------------------K------HLTEGIKSQ---------------------
-------NLEKNYNCAYINQTEIKNTQKINLEKFAEILRKRAKEKSQHQCAKYVRIALEAGGADTSGHPVA-
-----------------------------ASDWGPTLKKIGYKEIPEEFNKPQLG------DIYIITKTDKH
QYGHIAGYDGSQWISDFKQKGHRIYSDHVNYRYFRM------------------------------------
----------------------------

>ZW85-1_17
----------MNKKGLIQFRFVELFTGNNIPNLYHVIKNEK-----GTTIASGMTNSNGLTVMISR------
------------------------------------------------------------------------
---------------------DVGDT-LYVYLKN------------------------------IITGDLKE
K----VRHTVIYKKEIVRVISSKILL--------------------------DNIILAKSTNKP-GN-----

-YRY---------------------LTHKVKKGENLSSISHR----FNCKTSEIVHLNKLKNPDHIDVGQII
KI----------------------------------------------------------------PYKGES
SDSK--------KSPDNKQSKHENNKSSSTQKVN------------------------------SHENNNKN
TSDKPQKRIESKDITDEYTKESGKPMKVATNAT---------------------------------SPCICK
Q---YNLAWGSKVS-------CEFRKRVIKIAQNLW---------------PNDSENMASQLMAVMHLESAG
TF-------SPKIGTFISK----KLTD---------DAKGGYVGLIQFGKFASIDLKVKRSDLAKMSAVQQL
DYVEKYYKLNSAHTKIKNLTGLYLWVNYPKNVKENRLEDEDIVYAAPKDAEV--------------------
----------------------------------------------------------------------TS
KKFLESPYHQ-------------------NPSFMKENEEYKRD-----------------------------
-----------------GKKVIRQGFKNGSTKV---------------------------------------
WEVEQEIK---------------------------K------HLTEGIKSQ---------------------
-------NLEKNYNCAYINQTEIKNTQKINLEKFAEILRKRAKEKSQHQCAKYVRIALEAGGADTSGHPVA-
-----------------------------ASDWGPTLKKIGYKEIPEEFNKPQLG------DIYIITKTDKH
QYGHIAGYDGSQWISDFKQKGHRIYSDHVNYRYFRM------------------------------------
----------------------------
>A1_17
----------MNKKGLIQFRFVELFTGNNIPNLYHVIKNEK-----GTTIASGMTNSNGLTVMISR------
------------------------------------------------------------------------
---------------------DVGDT-LYVYLKN------------------------------IITGDLKE
K----VRHTVIYKKEIVRVTSSKILL--------------------------DNIILAKSTNKP-GN-----
-YRY---------------------LTHKVKKGENLSLISHR----FNCKISEIVHLNKLKNPDHIDVGQII
KI----------------------------------------------------------------PYKGGS
SDSK--------KSPDNKQSKHENNKSSSTQKVN------------------------------SHENNNKN
TSDKPQKRIESKDITDEYTKESGKPMKVATNAT---------------------------------SPCICK
Q---YNLAWGSKVS-------CEFRKRVIKIAQNLW---------------PNDSENMASQLMAVMHLESAG
TF-------SPKIGTFISK----KLTD---------DAKGGYVGLIQFGKFASIDLKVKRSDLAKMSAVQQL
DYVEKYYKLNSAHTKIKNLTGLYLWVNYPKNVKENRLEDEDIVYAAPKDAEV--------------------
----------------------------------------------------------------------TS
KKFLESPYHQ-------------------NPSFMKENEEYKRD-----------------------------
-----------------GKKVIRQGFKNGSTKV---------------------------------------
WEVEQEIK---------------------------K------HLTEGIKSQ---------------------
-------NLEKNYNCAYINHTEIKNTQKINLEKFAEILRKRAKEKSQHQCAKYVRIALEAGGADTSGHPVA-
-----------------------------ASDWGPTLKKIGYKEIPEEFNKPQLG------DIYIITKTDKH

QYGHIAGYDGSQWISDFKQKGHRIYSDHVNYRYFRM------------------------------------
----------------------------
>A388_17
----------MNKKGLIQFRFVELFTGNNIPNLYHVIKNEK-----GTTIASGMTNSNGLTVMISR------
------------------------------------------------------------------------
---------------------DVGDT-LYVYLKN------------------------------IITGDLKE
K----VRHTVIYKKEIVRVTSSKILL--------------------------DNIILAKSTNKP-GN-----
-YRY---------------------LTHKVKKGENLSLISHR----FNCKISEIVHLNKLKNPDHIDVGQII
KI----------------------------------------------------------------PYKGGS
SDSK--------KSPDNKQSKHENNKSSSTQKVN------------------------------SHENNNKN
TSDKPQKRIESKDITDEYTKESGKPMKVATNAT---------------------------------SPCICK
Q---YNLAWGSKVS-------CEFRKRVIKIAQNLW---------------PNDSENMASQLMAVMHLESAG
TF-------SPKIGTFISK----KLTD---------DAKGGYVGLIQFGKFASIDLKVKRSDLAKMSAVQQL
DYVEKYYKLNSAHTKIKNLTGLYLWVNYPKNVKENRLEDEDIVYAAPKDAEV--------------------
----------------------------------------------------------------------TS
KKFLESPYHQ-------------------NPSFMKENEEYKRD-----------------------------
-----------------GKKVIRQGFKNGSTKV---------------------------------------
WEVEQEIK---------------------------K------HLTEGIKSQ---------------------
-------NLEKNYNCAYINHTEIKNTQKINLEKFAEILRKRAKEKSQHQCAKYVRIALEAGGADTSGHPVA-
-----------------------------ASDWGPTLKKIGYKEIPEEFNKPQLG------DIYIITKTDKH
QYGHIAGYDGSQWISDFKQKGHRIYSDHVNYRYFRM------------------------------------
----------------------------
>A85_17
----------MNKKGLIQFRFVELFTGNNIPNLYHVIKNEK-----GTTIASGMTNSNGLTVMISR------
------------------------------------------------------------------------
---------------------DVGDT-LYVYLKN------------------------------IITGDLKE
K----VRHTVIYKKEIVRVTSSKILL--------------------------DNIILAKSTNKP-GN-----
-YRY---------------------LTHKVKKGENLSLISHR----FNCKISEIVHLNKLKNPDHIDVGQII
KI----------------------------------------------------------------PYKGGS
SDSK--------KSPDNKQSKHENNKSSSTQKVN------------------------------SHENNNKN
TSDKPQKRIESKDITDEYTKESGKPMKVATNAT---------------------------------SPCICK
Q---YNLAWGSKVS-------CEFRKRVIKIAQNLW---------------PNDSENMASQLMAVMHLESAG
TF-------SPKIGTFISK----KLTD---------DAKGGYVGLIQFGKFASIDLKVKRSDLAKMSAVQQL

DYVEKYYKLNSAHTKIKNLTGLYLWVNYPKNVKENRLEDEDIVYAAPKDAEV--------------------
----------------------------------------------------------------------TS
KKFLESPYHQ-------------------NPSFMKENEEYKRD-----------------------------
-----------------GKKVIRQGFKNGSTKV---------------------------------------
WEVEQEIK---------------------------K------HLTEGIKSQ---------------------
-------NLEKNYNCAYINHTEIKNTQKINLEKFAEILRKRAKEKSQHQCAKYVRIALEAGGADTSGHPVA-
-----------------------------ASDWGPTLKKIGYKEIPEEFNKPQLG------DIYIITKTDKH
QYGHIAGYDGSQWISDFKQKGHRIYSDHVNYRYFRM------------------------------------
----------------------------

>AB307-0294_17

----------MNKKGLIQFRFVELFTGNNIPNLYHVIKNEK-----GTTIASGMTNSNGLTVMISR------
------------------------------------------------------------------------
---------------------DVGDT-LYVYLKN------------------------------IITGDLKE
K----VRHTVIYKKEIVRVTSSKILL--------------------------DNIILAKSTNKP-GN-----
-YRY---------------------LTHKVKKGENLSLISHR----FNCKISEIVHLNKLKNPDHIDVGQII
KI----------------------------------------------------------------PYKGGS
SDSK--------KSPDNKQSKHENNKSSSTQKVN------------------------------SHENNNKN
TSDKPQKRIESKDITDEYTKESGKPMKVATNAT---------------------------------SPCICK
Q---YNLAWGSKVS-------CEFRKRVIKIAQNLW---------------PNDSENMASQLMAVMHLESAG
TF-------SPKIGTFISK----KLTD---------DAKGGYVGLIQFGKFASIDLKVKRSDLAKMSAVQQL
DYVEKYYKLNSAHTKIKNLTGLYLWVNYPKNVKENRLEDEDIVYAAPKDAEV--------------------
----------------------------------------------------------------------TS
KKFLESPYHQ-------------------NPSFMKENEEYKRD-----------------------------
-----------------GKKVIRQGFKNGSTKV---------------------------------------
WEVEQEIK---------------------------K------HLTEGIKSQ---------------------
-------NLEKNYNCAYINHTEIKNTQKINLEKFAEILRKRAKEKSQHQCAKYVRIALEAGGADTSGHPVA-
-----------------------------ASDWGPTLKKIGYKEIPEEFNKPQLG------DIYIITKTDKH
QYGHIAGYDGSQWISDFKQKGHRIYSDHVNYRYFRM------------------------------------
----------------------------

>AB5075-UW_17

----------MNKKGLIQFRFVELFTGNNIPNLYHVIKNEK-----GTTIASGMTNSNGLTVMISR------
------------------------------------------------------------------------
---------------------DVGDT-LYVYLKN------------------------------IITGDLKE

```
K----VRHTVIYKKEIVRVTSSKILL--------------------------DNIILAKSTNKP-GN-----
-YRY---------------------LTHKVKKGENLSLISHR----FNCKISEIVHLNKLKNPDHIDVGQII
KI----------------------------------------------------------------PYKGGS
SDSK--------KSPDNKQSKHENNKSSSTQKVN------------------------------SHENNNKN
TSDKPQKRIESKDITDEYTKESGKPMKVATNAT---------------------------------SPCICK
Q---YNLAWGSKVS-------CEFRKRVIKIAQNLW---------------PNDSENMASQLMAVMHLESAG
TF-------SPKIGTFISK----KLTD---------DAKGGYVGLIQFGKFASIDLKVKRSDLAKMSAVQQL
DYVEKYYKLNSAHTKIKNLTGLYLWVNYPKNVKENRLEDEDIVYAAPKDAEV--------------------
----------------------------------------------------------------------TS
KKFLESPYHQ-------------------NPSFMKENEEYKRD-----------------------------
-----------------GKKVIRQGFKNGSTKV---------------------------------------
WEVEQEIK---------------------------K------HLTEGIKSQ---------------------
-------NLEKNYNCAYINHTEIKNTQKINLEKFAEILRKRAKEKSQHQCAKYVRIALEAGGADTSGHPVA-
-----------------------------ASDWGPTLKKIGYKEIPEEFNKPQLG------DIYIITKTDKH
QYGHIAGYDGSQWISDFKQKGHRIYSDHVNYRYFRM------------------------------------
----------------------------
>AR_0083_17
----------MNKKGLIQFRFVELFTGNNIPNLYHVIKNEK-----GTTIASGMTNSNGLTVMISR------
------------------------------------------------------------------------
---------------------DVGDT-LYVYLKN------------------------------IITGDLKE
K----VRHTVIYKKEIVRVTSSKILL--------------------------DNIILAKSTNKP-GN-----
-YRY---------------------LTHKVKKGENLSLISHR----FNCKISEIVHLNKLKNPDHIDVGQII
KI----------------------------------------------------------------PYKGGS
SDSK--------KSPDNKQSKHENNKSSSTQKVN------------------------------SHENNNKN
TSDKPQKRIESKDITDEYTKESGKPMKVATNAT---------------------------------SPCICK
Q---YNLAWGSKVS-------CEFRKRVIKIAQNLW---------------PNDSENMASQLMAVMHLESAG
TF-------SPKIGTFISK----KLTD---------DAKGGYVGLIQFGKFASIDLKVKRSDLAKMSAVQQL
DYVEKYYKLNSAHTKIKNLTGLYLWVNYPKNVKENRLEDEDIVYAAPKDAEV--------------------
----------------------------------------------------------------------TS
KKFLESPYHQ-------------------NPSFMKENEEYKRD-----------------------------
-----------------GKKVIRQGFKNGSTKV---------------------------------------
WEVEQEIK---------------------------K------HLTEGIKSQ---------------------
-------NLEKNYNCAYINHTEIKNTQKINLEKFAEILRKRAKEKSQHQCAKYVRIALEAGGADTSGHPVA-
```

```
-----------------------------ASDWGPTLKKIGYKEIPEEFNKPQLG------DIYIITKTDKH
QYGHIAGYDGSQWISDFKQKGHRIYSDHVNYRYFRM------------------------------------
----------------------------
>AYE_17
----------MNKKGLIQFRFVELFTGNNIPNLYHVIKNEK-----GTTIASGMTNSNGLTVMISR------
------------------------------------------------------------------------
---------------------DVGDT-LYVYLKN------------------------------IITGDLKE
K----VRHTVIYKKEIVRVTSSKILL--------------------------DNIILAKSTNKP-GN-----
-YRY---------------------LTHKVKKGENLSLISHR----FNCKISEIVHLNKLKNPDHIDVGQII
KI----------------------------------------------------------------PYKGGS
SDSK--------KSPDNKQSKHENNKSSSTQKVN------------------------------SHENNNKN
TSDKPQKRIESKDITDEYTKESGKPMKVATNAT---------------------------------SPCICK
Q---YNLAWGSKVS-------CEFRKRVIKIAQNLW---------------PNDSENMASQLMAVMHLESAG
TF-------SPKIGTFISK----KLTD---------DAKGGYVGLIQFGKFASIDLKVKRSDLAKMSAVQQL
DYVEKYYKLNSAHTKIKNLTGLYLWVNYPKNVKENRLEDEDIVYAAPKDAEV--------------------
----------------------------------------------------------------------TS
KKFLESPYHQ-------------------NPSFMKENEEYKRD-----------------------------
-----------------GKKVIRQGFKNGSTKV---------------------------------------
WEVEQEIK---------------------------K------HLTEGIKSQ---------------------
-------NLEKNYNCAYINHTEIKNTQKINLEKFAEILRKRAKEKSQHQCAKYVRIALEAGGADTSGHPVA-
-----------------------------ASDWGPTLKKIGYKEIPEEFNKPQLG------DIYIITKTDKH
QYGHIAGYDGSQWISDFKQKGHRIYSDHVNYRYFRM------------------------------------
----------------------------
>D36_17
----------MNKKGLIQFRFVELFTGNNIPNLYHVIKNEK-----GTTIASGMTNSNGLTVMISR------
------------------------------------------------------------------------
---------------------DVGDT-LYVYLKN------------------------------IITGDLKE
K----VRHTVIYKKEIVRVTSSKILL--------------------------DNIILAKSTNKP-GN-----
-YRY---------------------LTHKVKKGENLSLISHR----FNCKISEIVHLNKLKNPDHIDVGQII
KI----------------------------------------------------------------PYKGGS
SDSK--------KSPDNKQSKHENNKSSSTQKVN------------------------------SHENNNKN
TSDKPQKRIESKDITDEYTKESGKPMKVATNAT---------------------------------SPCICK
Q---YNLAWGSKVS-------CEFRKRVIKIAQNLW---------------PNDSENMASQLMAVMHLESAG
```

TF-------SPKIGTFISK----KLTD---------DAKGGYVGLIQFGKFASIDLKVKRSDLAKMSAVQQL
DYVEKYYKLNSAHTKIKNLTGLYLWVNYPKNVKENRLEDEDIVYAAPKDAEV--------------------
----------------------------------------------------------------------TS
KKFLESPYHQ-------------------NPSFMKENEEYKRD-----------------------------
-----------------GKKVIRQGFKNGSTKV---------------------------------------
WEVEQEIK---------------------------K------HLTEGIKSQ---------------------
-------NLEKNYNCAYINHTEIKNTQKINLEKFAEILRKRAKEKSQHQCAKYVRIALEAGGADTSGHPVA-
-----------------------------ASDWGPTLKKIGYKEIPEEFNKPQLG------DIYIITKTDKH
QYGHIAGYDGSQWISDFKQKGHRIYSDHVNYRYFRM------------------------------------
----------------------------

>WCHAB005078_17
----------MNKKGLIQFRFVELFTGNNIPNLYHVIKNEK-----GTTIASGMTNSNGLTVMISR------
------------------------------------------------------------------------
---------------------DVGDT-LYVYLKN------------------------------IITGDLKE
K----VRHTVIYKKEIVRVTSSKILL--------------------------DNIILAKSTNKP-GN-----
-YRY---------------------LTHKVKKGENLSLISHR----FNCKISEIVHLNKLKNPDHIDVGQII
KI----------------------------------------------------------------PYKGGS
SDSK--------KSPDNKQSKHENNKSSSTQKVN------------------------------SHENNNKN
TSDKPQKRIESKDITDEYTKESGKPMKVATNAT---------------------------------SPCICK
Q---YNLAWGSKVS-------CEFRKRVIKIAQNLW---------------PNDSENMASQLMAVMHLESAG
TF-------SPKIGTFISK----KLTD---------DAKGGYVGLIQFGKFASIDLKVKRSDLAKMSAVQQL
DYVEKYYKLNSAHTKIKNLTGLYLWVNYPKNVKENRLEDEDIVYAAPKDAEV--------------------
----------------------------------------------------------------------TS
KKFLESPYHQ-------------------NPSFMKENEEYKRD-----------------------------
-----------------GKKVIRQGFKNGSTKV---------------------------------------
WEVEQEIK---------------------------K------HLTEGIKSQ---------------------
-------NLEKNYNCAYINHTEIKNTQKINLEKFAEILRKRAKEKSQHQCAKYVRIALEAGGADTSGHPVA-
-----------------------------ASDWGPTLKKIGYKEIPEEFNKPQLG------DIYIITKTDKH
QYGHIAGYDGSQWISDFKQKGHRIYSDHVNYRYFRM------------------------------------
----------------------------

>A1_16
----------------------------CSTTLNRNLGGVK-----GDHLQAHHIIPE--------------
------------------------------------------------------------------------

---------------------EIWAK-RKDFLDD------------------------------IGIGGNRD
K------------------------------------------------------------AEN-GV-----
------------------------------------------------------------------------
------------------------------------------------------------------------
------------LMPDSEAKAKQMKR--------------------------------------QLYHCGSH
PIYSAGINQKLGQ-----------------------------------------------------------
------------------------------------------------------------------------
------------------------------------------------------------------------
------------------------------------------------------------------------
----------------------------------------------------------------------IQ
REFESKKITA--------------------------------------------------------------
-----------------SQARDKVANLQSSMRL---------------------------------------
------------------------------------------VLITPGTKPIRLS-----------------
------------------------------------------------------------------------
------------------------------------------------------------------------
------------------------------------------------------------------------
----------------------------
>A388_16
----------------------------CSTTLNRNLGGVK-----GDHLQAHHIIPE--------------
------------------------------------------------------------------------
---------------------EIWAK-RKDFLDD------------------------------IGIGGNRD
K------------------------------------------------------------AEN-GV-----
------------------------------------------------------------------------
------------------------------------------------------------------------
------------LMPDSEAKAKQMKR--------------------------------------QLYHCGSH
PIYSAGINQKLGQ-----------------------------------------------------------
------------------------------------------------------------------------
------------------------------------------------------------------------
------------------------------------------------------------------------
----------------------------------------------------------------------IQ
REFESKKITA--------------------------------------------------------------
-----------------SQARDKVANLQSSMRL---------------------------------------
------------------------------------------VLITPGTKPIRLS-----------------

------------------------------------------------------------------------
------------------------------------------------------------------------
------------------------------------------------------------------------
----------------------------
>A85_16
----------------------------CSTTLNRNLGGVK-----GDHLQAHHIIPE--------------
------------------------------------------------------------------------
---------------------EIWAK-RKDFLDD------------------------------IGIGGNRD
K------------------------------------------------------------AEN-GV-----
------------------------------------------------------------------------
------------------------------------------------------------------------
------------LMPDSEAKAKQMKR--------------------------------------QLYHCGSH
PIYSAGINQKLGQ-----------------------------------------------------------
------------------------------------------------------------------------
------------------------------------------------------------------------
------------------------------------------------------------------------
----------------------------------------------------------------------IQ
REFESKKITA--------------------------------------------------------------
-----------------SQARDKVANLQSSMRL---------------------------------------
------------------------------------------VLITPGTKPIRLS-----------------
------------------------------------------------------------------------
------------------------------------------------------------------------
------------------------------------------------------------------------
----------------------------
>AB0057_16
----------------------------CSTTLNRNLGGVK-----GDHLQAHHIIPE--------------
------------------------------------------------------------------------
---------------------EIWAK-RKDFLDD------------------------------IGIGGNRD
K------------------------------------------------------------AEN-GV-----
------------------------------------------------------------------------
------------------------------------------------------------------------
------------LMPDSEAKAKQMKR--------------------------------------QLYHCGSH
PIYSAGINQKLGQ-----------------------------------------------------------

------------------------------------------------------------------------
------------------------------------------------------------------------
------------------------------------------------------------------------
----------------------------------------------------------------------IQ
REFESKKITA--------------------------------------------------------------
-----------------SQARDKVANLQSSMRL---------------------------------------
------------------------------------------VLITPGTKPIRLS-----------------
------------------------------------------------------------------------
------------------------------------------------------------------------
------------------------------------------------------------------------
----------------------------
>AB307-0294_16
----------------------------CSTTLNRNLGGVK-----GDHLQAHHIIPE--------------
------------------------------------------------------------------------
---------------------EIWAK-RKDFLDD------------------------------IGIGGNRD
K------------------------------------------------------------AEN-GV-----
------------------------------------------------------------------------
------------------------------------------------------------------------
------------LMPDSEAKAKQMKR--------------------------------------QLYHCGSH
PIYSAGINQKLGQ-----------------------------------------------------------
------------------------------------------------------------------------
------------------------------------------------------------------------
------------------------------------------------------------------------
----------------------------------------------------------------------IQ
REFESKKITA--------------------------------------------------------------
-----------------SQARDKVANLQSSMRL---------------------------------------
------------------------------------------VLITPGTKPIRLS-----------------
------------------------------------------------------------------------
------------------------------------------------------------------------
------------------------------------------------------------------------
----------------------------
>AB5075-UW_16
----------------------------CSTTLNRNLGGVK-----GDHLQAHHIIPE--------------

------------------------------------------------------------------------
---------------------EIWAK-RKDFLDD------------------------------IGIGGNRD
K------------------------------------------------------------AEN-GV-----
------------------------------------------------------------------------
------------------------------------------------------------------------
------------LMPDSEAKAKQMKR--------------------------------------QLYHCGSH
PIYSAGINQKLGQ-----------------------------------------------------------
------------------------------------------------------------------------
------------------------------------------------------------------------
------------------------------------------------------------------------
----------------------------------------------------------------------IQ
REFESKKITA--------------------------------------------------------------
-----------------SQARDKVANLQSSMRL---------------------------------------
------------------------------------------VLITPGTKPIRLS-----------------
------------------------------------------------------------------------
------------------------------------------------------------------------
------------------------------------------------------------------------
----------------------------
>AR_0083_16
----------------------------CSTTLNRNLGGVK-----GDHLQAHHIIPE--------------
------------------------------------------------------------------------
---------------------EIWAK-RKDFLDD------------------------------IGIGGNRD
K------------------------------------------------------------AEN-GV-----
------------------------------------------------------------------------
------------------------------------------------------------------------
------------LMPDSEAKAKQMKR--------------------------------------QLYHCGSH
PIYSAGINQKLGQ-----------------------------------------------------------
------------------------------------------------------------------------
------------------------------------------------------------------------
------------------------------------------------------------------------
----------------------------------------------------------------------IQ
REFESKKITA--------------------------------------------------------------
-----------------SQARDKVANLQSSMRL---------------------------------------

------------------------------------------VLITPGTKPIRLS-----------------
------------------------------------------------------------------------
------------------------------------------------------------------------
------------------------------------------------------------------------
----------------------------
>D36_16
----------------------------CSTTLNRNLGGVK-----GDHLQAHHIIPE--------------
------------------------------------------------------------------------
---------------------EIWAK-RKDFLDD------------------------------IGIGGNRD
K------------------------------------------------------------AEN-GV-----
------------------------------------------------------------------------
------------------------------------------------------------------------
------------LMPDSEAKAKQMKR--------------------------------------QLYHCGSH
PIYSAGINQKLGQ-----------------------------------------------------------
------------------------------------------------------------------------
------------------------------------------------------------------------
------------------------------------------------------------------------
----------------------------------------------------------------------IQ
REFESKKITA--------------------------------------------------------------
-----------------SQARDKVANLQSSMRL---------------------------------------
------------------------------------------VLITPGTKPIRLS-----------------
------------------------------------------------------------------------
------------------------------------------------------------------------
------------------------------------------------------------------------
----------------------------
>USA15_16
----------------------------CSTTLNRNLGGVK-----GDHLQAHHIIPE--------------
------------------------------------------------------------------------
---------------------EIWAK-RKDFLDD------------------------------IGIGGNRD
K------------------------------------------------------------AEN-GV-----
------------------------------------------------------------------------
------------------------------------------------------------------------
------------LMPDSEAKAKQMKR--------------------------------------QLYHCGSH

PIYSAGINQKLGQ-----------------------------------------------------------
------------------------------------------------------------------------
------------------------------------------------------------------------
------------------------------------------------------------------------
----------------------------------------------------------------------IQ
REFESKKITA--------------------------------------------------------------
-----------------SQARDKVANLQSSMRL---------------------------------------
------------------------------------------VLITPGTKPIRLS-----------------
------------------------------------------------------------------------
------------------------------------------------------------------------
------------------------------------------------------------------------
----------------------------
>WCHAB005078_16
----------------------------CSTTLNRNLGGVK-----GDHLQAHHIIPE--------------
------------------------------------------------------------------------
---------------------EIWAK-RKDFLDD------------------------------IGIGGNRD
K------------------------------------------------------------AEN-GV-----
------------------------------------------------------------------------
------------------------------------------------------------------------
------------LMPDSEAKAKQMKR--------------------------------------QLYHCGSH
PIYSAGINQKLGQ-----------------------------------------------------------
------------------------------------------------------------------------
------------------------------------------------------------------------
------------------------------------------------------------------------
----------------------------------------------------------------------IQ
REFESKKITA--------------------------------------------------------------
-----------------SQARDKVANLQSSMRL---------------------------------------
------------------------------------------VLITPGTKPIRLS-----------------
------------------------------------------------------------------------
------------------------------------------------------------------------
------------------------------------------------------------------------
----------------------------
>AR_0078_29

```
----------MNKKSLVTIQILDLFGSPISKAQYEVKNQRT-----GQVIAAGTTNSSGCIVEISR------
------------------------------------------------------------------------
---------------------DKGTA-LDVYIKS------------------------------MFKGSMVK
V----QSFVMSKDRMVVKITSPKVLL----------------------------NLKTLTNQGNNGQ-----
-YKR---------------------KTHIVKKGDTLFEIAQK----NHTTVRVLERLNKIDDPNKISIGQVI
KL-------------------------------------------------------------PVHIPASGN
HSHQ--------DKPKHKHSAQSTNKPTASSTKTTP---APAAKRPANPASQTKQENGLPEKAHQLYEDTKK
ALNEAASSVSKILTIDDRSQDGGTPKANTTNICKTNPQCISSGKSELI-REVNIRLAGFGGALPTDEFTELT
ATCIRQFQRDYMGV-PDTGKICGSVLVALDQFYDEY------------------PISSFMGKAACNCGKCSG
FG-------NGKMGVQSGI-----------------NTANEYPGIHRSLIWILKSLNFYLKNEFKDKKINVA
YIESGYRCIENNKKHERTSVNHMGLALDIHFHKNGVRTRELSDMEFIRKNI---------------------
----------------------------------------------------------------------MA
KKMGASEQRI-------------------RDKIYLEPKVFRSG-----------------------------
------EAGATSWVHFDITMFLAKYFNNNMFKT---------------------------------------
KVSDLNGV----------------------------------KLVNLAASSRILSCGGI-VALP--------
-------KSQNNITDELVLSNEDIIDIMKVTETEVIKFKTEKY--FLEQAA--GVVDTIMNRTKSGVWGNSV
RKVVNA------------DRQFSKITGPKSLDPYGSVENMPMSHVSRKVRNFVNS------YLLERANGKKS
IIGENLNYANKYYSDEKNRKAWVDKFHNEAVKNGMILGTGKAIHAHGTVRELRDKMPKPFKIVLPKDFKGI-
----------------------------
>AR_0088_14
----------MNKKSLVTIQILDLFGSPISKAQYVVKNQRT-----GKVIAAGPTNSSGCIVEISQ------
------------------------------------------------------------------------
---------------------DKGTT-LDVYIKS------------------------------MFKGSMVK
V----QSFVMSKDRMVVKITSPKVLL----------------------------DLKTLTNQGNNGQ-----
-YKR---------------------KTHIVKKGDTLFEIAQK----NHTTVRALERLNKIDDPNKISVGQVI
KL-------------------------------------------------------------PVHIPASGN
HSHQ--------DKPKHKHSAQSTNKPTASSAKTTP---APAAKRPANPASQTKQENGLPEKAHQLYEDTKK
ALNEAASSVSKILTVDDRSQDGGTPKANTTNICKTNPQCISSGKSELI-REVNIRLAGFGGALPTDEFTELT
ATCIKQFQRDYMGV-PDTGKICGSVLAALDRFRDEY------------------GIASYFESMKCPCGECSG
FG-------RSRSGNFRFE----MYNKTTRQYVEVYRDAKEYNGMHRSLIWALKAMLFYFKKMPNSAGYKIV
KISSGYRCIDNNWKNRRPTTNHMGCA--LDIVVHNKNNQVVSMSELENQVRIKWFGKYLNTSLGWSPDKFGL
ERISD-----------------------------------------------------------------GA
KTWVHLDVRE-------------------FNKKYKIDKLFSTS-----------------------------
```

----KDGLNGDYLINLFKADEKANKILGCSGIV---------------------------------------
VSPNKNPN------------------------------LNDSSLEDLIRQLGSVISHGE---------GNYE
SYNTGTKNVKGDKVGYSFKNPGKGTVTSKRIQQIIDNAEAKDGNDKNRLFATGKYQTTYYTLQEGIKQGYFT
GSEVYD--------------------ANMQEQVFREFLISKRTKLSQFVRKGIG-------SLRDAQYDAAQ
EWASVAVPKGLPLKNGKISDGNKSYYEKPGQNSSSPESTKMVLEILEKIHRFHKDGK---------------
----------------------------
>CIP70.10_14
----------MNKKSLVTIQILDLFGSPISKAQYVVKNQRT-----GKVIAAGPTNSSGCIVEISQ------
------------------------------------------------------------------------
---------------------DKGTT-LDVYIKS------------------------------MFKGSMVK
V----QSFVMSKDRMVVKITSPKVLL----------------------------DLKTLTNQGNNGQ-----
-YKR---------------------KTHIVKKGDTLFEIAQK----NHTTVRALERLNKIDDPNKISVGQVI
KL-------------------------------------------------------------PVHIPASGN
HSHQ--------DKPKHKHSAQSTNKPTASSAKTTP---APAAKRPANPASQTKQENGLPEKAHQLYEDTKK
ALNEAASSVSKILTVDDRSQDGGTPKANTTNICKTNPQCISSGKSELI-REVNIRLAGFGGALPTDEFTELT
ATCIKQFQRDYMGV-PDTGKICGSVLAALDRFRDEY------------------GIASYFESMKCPCGECSG
FG-------RSRSGNFRFE----MYNKTTRQYVEVYRDAKEYNGMHRSLIWALKAMLFYFKKMPNSAGYKIV
KISSGYRCIDNNWKNRRPTTNHMGCA--LDIVVHNKNNQVVSMSELENQVRIKWFGKYLNTSLGWSPDKFGL
ERISD-----------------------------------------------------------------GA
KTWVHLDVRE-------------------FNKKYKIDKLFSTS-----------------------------
----KDGLNGDYLINLFKADEKANKILGCSGIV---------------------------------------
VSPNKNPN------------------------------LNDSSLEDLIRQLGSVISHGE---------GNYE
SYNTGTKNVKGDKVGYSFKNPGKGTVTSKRIQQIIDNAEAKDGNDKNRLFATGKYQTTYYTLQEGIKQGYFT
GSEVYD--------------------ANMQEQVFREFLISKRTKLSQFVRKGIG-------SLRDAQYDAAQ
EWASVAVPKGLPLKNGKISDGNKSYYEKPGQNSSSPESTKMVLEILEKIHRFHKDGK---------------
----------------------------
>HWBA8_14
----------MNKKSLVTIQILDLFGSPISKAQYVVKNQRT-----GKVIAAGPTNSSGCIVEISQ------
------------------------------------------------------------------------
---------------------DKGTT-LDVYIKS------------------------------MFKGSMVK
V----QSFVMSKDRMVVKITSPKVLL----------------------------DLKTLTNQGNNGQ-----
-YKR---------------------KTHIVKKGDTLFEIAQK----NHTTVRALERLNKIDDPNKISVGQVI
KL-------------------------------------------------------------PVHIPASGN

```
HSHQ--------DKPKHKHSAQSTNKPTASSAKTTP---APAAKRPANPASQTKQENGLPEKAHQLYEDTKK
ALNEAASSVSKILTVDDRSQDGGTPKANTTNICKTNPQCISSGKSELI-REVNIRLAGFGGALPTDEFTELT
ATCIKQFQRDYMGV-PDTGKICGSVLAALDRFRDEY------------------GIASYFESMKCPCGECSG
FG-------RSRSGNFRFE----MYNKTTRQYVEVYRDAKEYNGMHRSLIWALKAMLFYFKKMPNSAGYKIV
KISSGYRCIDNNWKNRRPTTNHMGCA--LDIVVHNKNNQVVSMSELENQVRIKWFGKYLNTSLGWSPDKFGL
ERISD-----------------------------------------------------------------GA
KTWVHLDVRE-------------------FNKKYKIDKLFSTS-----------------------------
----KDGLNGDYLINLFKADEKANKILGCSGIV---------------------------------------
VSPNKNPN------------------------------LNDSSLEDLIRQLGSVISHGE---------GNYE
SYNTGTKNVKGDKVGYSFKNPGKGTVTSKRIQQIIDNAEAKDGNDKNRLFATGKYQTTYYTLQEGIKQGYFT
GSEVYD--------------------ANMQEQVFREFLISKRTKLSQFVRKGIG-------SLRDAQYDAAQ
EWASVAVPKGLPLKNGKISDGNKSYYEKPGQNSSSPESTKMVLEILEKIHRFHKDGK---------------
----------------------------
>R2091_14
----------MNKKSLVTIQILDLFGSPISKAQYVVKNQRT-----GKVIAAGPTNSSGCIVEISQ------
------------------------------------------------------------------------
---------------------DKGTT-LDVYIKS------------------------------MFKGSMVK
V----QSFVMSKDRMVVKITSPKVLL----------------------------DLKTLTNQGNNGQ-----
-YKR---------------------KTHIVKKGDTLFEIAQK----NHTTVRALERLNKIDDPNKISVGQVI
KL-------------------------------------------------------------PVHIPASGN
HSHQ--------DKPKHKHSAQSTNKPTASSAKTTP---APAAKRPANPASQTKQENGLPEKAHQLYEDTKK
ALNEAASSVSKILTVDDRSQDGGTPKANTTNICKTNPQCISSGKSELI-REVNIRLAGFGGALPTDEFTELT
ATCIKQFQRDYMGV-PDTGKICGSVLAALDRFRDEY------------------GIASYFESMKCPCGECSG
FG-------RSRSGNFRFE----MYNKTTRQYVEVYRDAKEYNGMHRSLIWALKAMLFYFKKMPNSAGYKIV
KISSGYRCIDNNWKNRRPTTNHMGCA--LDIVVHNKNNQVVSMSELENQVRIKWFGKYLNTSLGWSPDKFGL
ERISD-----------------------------------------------------------------GA
KTWVHLDVRE-------------------FNKKYKIDKLFSTS-----------------------------
----KDGLNGDYLINLFKADEKANKILGCSGIV---------------------------------------
VSPNKNPN------------------------------LNDSSLEDLIRQLGSVISHGE---------GNYE
SYNTGTKNVKGDKVGYSFKNPGKGTVTSKRIQQIIDNAEAKDGNDKNRLFATGKYQTTYYTLQEGIKQGYFT
GSEVYD--------------------ANMQEQVFREFLISKRTKLSQFVRKGIG-------SLRDAQYDAAQ
EWASVAVPKGLPLKNGKISDGNKSYYEKPGQNSSSPESTKMVLEILEKIHRFHKDGK---------------
----------------------------
```

```
>TYTH-1_14
----------MNKKSLVTIQILDLFGSPISKAQYEVKNQRT-----GQVIAAGTTNSSGCIVEISR------
------------------------------------------------------------------------
---------------------DKGTA-LDVYIKS------------------------------MFKGSMVK
V----QSFVMSKDRMVVKITSPKVLL----------------------------NLKTLTNQGNNGQ-----
-YKR---------------------KTHIVKKGDTLFEIAQK----NHTTVRALERLNKIDDPNKISIGQVI
KL-------------------------------------------------------------PVHIPASGN
HSHQ--------DKSKHKHSAQPVNKPTASSTKTTP---AAAAKRPANPTSQTKQENGLPEKVHQLYEDTKK
ALNEAASSASKILTVDDRSQDGGTPKANTTNICKTNPQCISSGKSELI-REVNIRLAGFGGALPTDEFTELT
ATCIKQFQRDYMGV-PDTGKICGSVLAALDRFRDEY------------------GIASYFESMKCPCGECSG
FG-------RSRSGNFRFE----MYNKTTRQYVEVYRDAKEYNGMHRSLIWALKAMLFYFKKMPNSAGYKIV
KISSGYRCIDNNWKNRRPTTNHMGCA--LDIVVHNKNNQVVSMSELENQVRIKWFGKYLNTSLGWSPDKFGL
ERISD-----------------------------------------------------------------GA
KTWVHLDVRE-------------------FNKKYKIDKLFSTS-----------------------------
----KDGLNGDYLINLFKADEKANKILGCSGIV---------------------------------------
VSPNKNPN------------------------------LNDSSLEDLIRQLGSVISHGE---------GNYE
SYNTGTKNVKGDKVGYSFKNPGKGTVTSKRIQQIIDNAEAKDGNDKNRLFATGKYQTTYYTLQEGIKQGYFT
GSEVYD--------------------ANMQEQVFREFLISKRTKLSQFVRKGIG-------SLRDAQYDAAQ
EWASVAVPKGLPLKNGKISDGNKSYYEKPGQNSSSPESTKMVLEILEKIHRFHKDGK---------------
----------------------------
>15A5_28
------------------------------------MGNNS-----TTSTTTNTANPPTQKVPL--------
------------------------------------------------------------------------
---------------------KPEAE-LHLLVGS-----------------------------AYGEGDRES
P----YGHTAVYIKLKGKDYIY------------------------------DFGRYGKTKPESFGI-----
-FTL---------------------EGANSPRGEGILRIWSS----FDA-----------------------
------------------------------------------------------------------------
------------YIADENSQGKGTSKTRTTH---------------------------------AYGYRIFD
SQAMLVLNYYNNLLKTATLRRNEKYYKS--------------------------------------------
----YALSQDYFAL-----------------------------------APNCTTQSLEATKKAIPSMAKSG
HM----------------------------------------------------------------------
------------------------------------------------------------------------
----------------------------------------------------------------------FV
```

NAEKVLPTTV--------------------------------------------------------------
-----------------KLAFKASKYEMPKYLF---------------------------------------
-------------------------------------------LPDNLNDYLMK------------------
------------------------------------------------------------------------
------------------------------------------------------------------------
-------------SPDVKVDIKNTYHLKK-------------------------------------------
----------------------------
>Ab4568_28
------------------------------------MGNNS-----TTSTTTNTANPPTQKVPL--------
------------------------------------------------------------------------
---------------------KPEAE-LHLLVGS-----------------------------AYGEGDRES
P----YGHTAVYIKLKGKDYIY------------------------------DFGRYGKTKPESFGI-----
-FTL---------------------EGANSPRGEGILRIWSS----FDA-----------------------
------------------------------------------------------------------------
------------YIADENSQGKGTSKTRTTH---------------------------------AYGYRIFD
SQAMLVLNYYNNLLKTATLRRNEKYYKS--------------------------------------------
----YALSQDYFAL-----------------------------------APNCTTQSLEATKKAIPSMAKSG
HM----------------------------------------------------------------------
------------------------------------------------------------------------
----------------------------------------------------------------------FV
NAEKVLPTTV--------------------------------------------------------------
-----------------KLAFKASKYEMPKYLF---------------------------------------
-------------------------------------------LPDNLNDYLMK------------------
------------------------------------------------------------------------
------------------------------------------------------------------------
-------------SPDVKVDIKNTYHLKK-------------------------------------------
----------------------------
>Ab4977_28
------------------------------------MGNNS-----TTSTTTNTANPPTQKVPL--------
------------------------------------------------------------------------
---------------------KPEAE-LHLLVGS-----------------------------AYGEGDRES
P----YGHTAVYIKLKGKDYIY------------------------------DFGRYGKTKPESFGI-----
-FTL---------------------EGANSPRGEGILRIWSS----FDA-----------------------

------------------------------------------------------------------------
------------YIADENSQGKGTSKTRTTH---------------------------------AYGYRIFD
SQAMLVLNYYNNLLKTATLRRNEKYYKS--------------------------------------------
----YALSQDYFAL-----------------------------------APNCTTQSLEATKKAIPSMAKSG
HM----------------------------------------------------------------------
------------------------------------------------------------------------
----------------------------------------------------------------------FV
NAEKVLPTTV--------------------------------------------------------------
-----------------KLAFKASKYEMPKYLF---------------------------------------
-------------------------------------------LPDNLNDYLMK------------------
------------------------------------------------------------------------
------------------------------------------------------------------------
-------------SPDVKVDIKNTYHLKK-------------------------------------------
----------------------------
>JBA13_28
------------------------------------MGNNS-----TTSTTTNTANPPTQKVPL--------
------------------------------------------------------------------------
---------------------KPEAE-LHLLVGS-----------------------------AYGEGDRES
P----YGHTAVYIKLKGKDYIY------------------------------DFGRYGKTKPESFGI-----
-FTL---------------------EGANSPRGEGILRIWSS----FDA-----------------------
------------------------------------------------------------------------
------------YIADENSQGKGTSKTRTTH---------------------------------AYGYRIFD
SQAMLVLNYYNNLLKTATLRRNEKYYKS--------------------------------------------
----YALSQDYFAL-----------------------------------APNCTTQSLEATKKAIPSMAKSG
HM----------------------------------------------------------------------
------------------------------------------------------------------------
----------------------------------------------------------------------FV
NAEKVLPTTV--------------------------------------------------------------
-----------------KLAFKASKYEMPKYLF---------------------------------------
-------------------------------------------LPDNLNDYLMK------------------
------------------------------------------------------------------------
------------------------------------------------------------------------
-------------SPDVKVDIKNTYHLKK-------------------------------------------

----------------------------
>KAB04_28
------------------------------------MGNNS-----TTSTTTNTANPPTQKVPL--------
------------------------------------------------------------------------
---------------------KPEAE-LHLLVGS-----------------------------AYGEGDRES
P----YGHTAVYIKLKGKDYIY------------------------------DFGRYGKTKPESFGI-----
-FTL---------------------EGANSPRGEGILRIWSS----FDA-----------------------
------------------------------------------------------------------------
------------YIADENSQGKGTSKTRTTH---------------------------------AYGYRIFD
SQAMLVLNYYNNLLKTATLRRNEKYYKS--------------------------------------------
----YALSQDYFAL-----------------------------------APNCTTQSLEATKKAIPSMAKSG
HM----------------------------------------------------------------------
------------------------------------------------------------------------
----------------------------------------------------------------------FV
NAEKVLPTTV--------------------------------------------------------------
-----------------KLAFKASKYEMPKYLF---------------------------------------
-------------------------------------------LPDNLNDYLMK------------------
------------------------------------------------------------------------
------------------------------------------------------------------------
-------------SPDVKVDIKNTYHLKK-------------------------------------------
----------------------------
>KAB07_28
------------------------------------MGNNS-----TTSTTTNTANPPTQKVPL--------
------------------------------------------------------------------------
---------------------KPEAE-LHLLVGS-----------------------------AYGEGDRES
P----YGHTAVYIKLKGKDYIY------------------------------DFGRYGKTKPESFGI-----
-FTL---------------------EGANSPRGEGILRIWSS----FDA-----------------------
------------------------------------------------------------------------
------------YIADENSQGKGTSKTRTTH---------------------------------AYGYRIFD
SQAMLVLNYYNNLLKTATLRRNEKYYKS--------------------------------------------
----YALSQDYFAL-----------------------------------APNCTTQSLEATKKAIPSMAKSG
HM----------------------------------------------------------------------
------------------------------------------------------------------------

----------------------------------------------------------------------FV
NAEKVLPTTV--------------------------------------------------------------
-----------------KLAFKASKYEMPKYLF---------------------------------------
-------------------------------------------LPDNLNDYLMK------------------
------------------------------------------------------------------------
------------------------------------------------------------------------
-------------SPDVKVDIKNTYHLKK-------------------------------------------
----------------------------
>KAB08_28
------------------------------------MGNNS-----TTSTTTNTANPPTQKVPL--------
------------------------------------------------------------------------
---------------------KPEAE-LHLLVGS-----------------------------AYGEGDRES
P----YGHTAVYIKLKGKDYIY------------------------------DFGRYGKTKPESFGI-----
-FTL---------------------EGANSPRGEGILRIWSS----FDA-----------------------
------------------------------------------------------------------------
------------YIADENSQGKGTSKTRTTH---------------------------------AYGYRIFD
SQAMLVLNYYNNLLKTATLRRNEKYYKS--------------------------------------------
----YALSQDYFAL-----------------------------------APNCTTQSLEATKKAIPSMAKSG
HM----------------------------------------------------------------------
------------------------------------------------------------------------
----------------------------------------------------------------------FV
NAEKVLPTTV--------------------------------------------------------------
-----------------KLAFKASKYEMPKYLF---------------------------------------
-------------------------------------------LPDNLNDYLMK------------------
------------------------------------------------------------------------
------------------------------------------------------------------------
-------------SPDVKVDIKNTYHLKK-------------------------------------------
----------------------------
>KBN10P02143_28
------------------------------------MGNNS-----TTSTTTNTANPPTQKVPL--------
------------------------------------------------------------------------
---------------------KPEAE-LHLLVGS-----------------------------AYGEGDRES
P----YGHTAVYIKLKGKDYIY------------------------------DFGRYGKTKPESFGI-----

```
-FTL---------------------EGANSPRGEGILRIWSS----FDA-----------------------
------------------------------------------------------------------------
------------YIADENSQGKGTSKTRTTH---------------------------------AYGYRIFD
SQAMLVLNYYNNLLKTATLRRNEKYYKS--------------------------------------------
----YALSQDYFAL-----------------------------------APNCTTQSLEATKKAIPSMAKSG
HM----------------------------------------------------------------------
------------------------------------------------------------------------
----------------------------------------------------------------------FV
NAEKVLPTTV--------------------------------------------------------------
-----------------KLAFKASKYEMPKYLF---------------------------------------
-------------------------------------------LPDNLNDYLMK------------------
------------------------------------------------------------------------
------------------------------------------------------------------------
-------------SPDVKVDIKNTYHLKK-------------------------------------------
----------------------------
>SAA14_28
------------------------------------MGNNS-----TTSTTTNTANPPTQKVPL--------
------------------------------------------------------------------------
---------------------KPEAE-LHLLVGS-----------------------------AYGEGDRES
P----YGHTAVYIKLKGKDYIY------------------------------DFGRYGKTKPESFGI-----
-FTL---------------------EGANSPRGEGILRIWSS----FDA-----------------------
------------------------------------------------------------------------
------------YIADENSQGKGTSKTRTTH---------------------------------AYGYRIFD
SQAMLVLNYYNNLLKTATLRRNEKYYKS--------------------------------------------
----YALSQDYFAL-----------------------------------APNCTTQSLEATKKAIPSMAKSG
HM----------------------------------------------------------------------
------------------------------------------------------------------------
----------------------------------------------------------------------FV
NAEKVLPTTV--------------------------------------------------------------
-----------------KLAFKASKYEMPKYLF---------------------------------------
-------------------------------------------LPDNLNDYLMK------------------
------------------------------------------------------------------------
------------------------------------------------------------------------
```

```
-------------SPDVKVDIKNTYHLKK-------------------------------------------
----------------------------
>SMC_Paed_Ab_BL01_28
------------------------------------MGNNS-----TTSTTTNTANPPTQKVPL--------
------------------------------------------------------------------------
---------------------KPEAE-LHLLVGS-----------------------------AYGEGDRES
P----YGHTAVYIKLKGKDYIY------------------------------DFGRYGKTKPESFGI-----
-FTL---------------------EGANSPRGEGILRIWSS----FDA-----------------------
------------------------------------------------------------------------
------------YIADENSQGKGTSKTRTTH-----------------------------------------AYGYRIFD
SQAMLVLNYYNNLLKTATLRRNEKYYKS--------------------------------------------
----YALSQDYFAL-----------------------------------APNCTTQSLEATKKAIPSMAKSG
HM----------------------------------------------------------------------
------------------------------------------------------------------------
----------------------------------------------------------------------FV
NAEKVLPTTV--------------------------------------------------------------
-----------------KLAFKASKYEMPKYLF---------------------------------------
-------------------------------------------LPDNLNDYLMK------------------
------------------------------------------------------------------------
------------------------------------------------------------------------
-------------SPDVKVDIKNTYHLKK-------------------------------------------
----------------------------
>SSA12_28
------------------------------------MGNNS-----TTSTTTNTANPPTQKVPL--------
------------------------------------------------------------------------
---------------------KPEAE-LHLLVGS-----------------------------AYGEGDRES
P----YGHTAVYIKLKGKDYIY------------------------------DFGRYGKTKPESFGI-----
-FTL---------------------EGANSPRGEGILRIWSS----FDA-----------------------
------------------------------------------------------------------------
------------YIADENSQGKGTSKTRTTH-----------------------------------------AYGYRIFD
SQAMLVLNYYNNLLKTATLRRNEKYYKS--------------------------------------------
----YALSQDYFAL-----------------------------------APNCTTQSLEATKKAIPSMAKSG
HM----------------------------------------------------------------------
```

------------------------------------------------------------------------
----------------------------------------------------------------------FV
NAEKVLPTTV--------------------------------------------------------------
-----------------KLAFKASKYEMPKYLF---------------------------------------
-------------------------------------------LPDNLNDYLMK------------------
------------------------------------------------------------------------
------------------------------------------------------------------------
-------------SPDVKVDIKNTYHLKK-------------------------------------------
----------------------------
>SSMA17_28
------------------------------------MGNNS-----TTSTTTNTANPPTQKVPL--------
------------------------------------------------------------------------
---------------------KPEAE-LHLLVGS-----------------------------AYGEGDRES
P----YGHTAVYIKLKGKDYIY------------------------------DFGRYGKTKPESFGI-----
-FTL---------------------EGANSPRGEGILRIWSS----FDA-----------------------
------------------------------------------------------------------------
------------YIADENSQGKGTSKTRTTH---------------------------------AYGYRIFD
SQAMLVLNYYNNLLKTATLRRNEKYYKS--------------------------------------------
----YALSQDYFAL-----------------------------------APNCTTQSLEATKKAIPSMAKSG
HM----------------------------------------------------------------------
------------------------------------------------------------------------
----------------------------------------------------------------------FV
NAEKVLPTTV--------------------------------------------------------------
-----------------KLAFKASKYEMPKYLF---------------------------------------
-------------------------------------------LPDNLNDYLMK------------------
------------------------------------------------------------------------
------------------------------------------------------------------------
-------------SPDVKVDIKNTYHLKK-------------------------------------------
----------------------------
>YU-R612_28
------------------------------------MGNNS-----TTSTTTNTANPPTQKVPL--------
------------------------------------------------------------------------
---------------------KPEAE-LHLLVGS-----------------------------AYGEGDRES

P----YGHTAVYIKLKGKDYIY------------------------------DFGRYGKTKPESFGI-----
-FTL---------------------EGANSPRGEGILRIWSS----FDA-----------------------
------------------------------------------------------------------------
------------YIADENSQGKGTSKTRTTH---------------------------------AYGYRIFD
SQAMLVLNYYNNLLKTATLRRNEKYYKS--------------------------------------------
----YALSQDYFAL-----------------------------------APNCTTQSLEATKKAIPSMAKSG
HM----------------------------------------------------------------------
------------------------------------------------------------------------
----------------------------------------------------------------------FV
NAEKVLPTTV--------------------------------------------------------------
-----------------KLAFKASKYEMPKYLF---------------------------------------
-------------------------------------------LPDNLNDYLMK------------------
------------------------------------------------------------------------
------------------------------------------------------------------------
-------------SPDVKVDIKNTYHLKK-------------------------------------------
----------------------------
>15A34_23
------MNTELKHKLYNITFTLHDLTHKPIPNLYYEIKNGK-----DLVKKGNTNAQGEITLKY--------
------------------------------------------------------------------------
---------------------VGGNT-LTLFVRK------------------------------DIDNTLKE
I----GKVHTPSKNIKVKLISPKMKF--------------------------DVTLLPHQKQGK--------
-YWR---------------------GTYKVKSGDTLSKIAKE---HHTTISALLALNPNIKSPHEIYQNENI
DIVEGNAD--------------------------------------------------------AIIKLDEN
MTVK--------VPPHKKTGTVSIDRKSKSTSITP-----------------------------QTDTTNKT
AEPKVGQTKLQPPVTKKEQSAASDSKSIPQQKTSTATTQKIEVQQEHNEDKKPVAVASL-------SGCVCK
D---YDLIWGKTTK----FVNCEFRKKVIQICKDMW---------------PNDTKNMANYLMACMHLETGG
SF-------DPAQPNGLGY----------------------YGLIQFGPDVRKDLNNISVEKLTKMSGAQQL
DLVKQHFTRSDRHKLMKSLTDMYLYINYPNALLSGKNKPNDIL-----------------------------
----------------------------------------------------------------------YE
GGSERSAYHA-------------------NPAFMKEKGEYQNI-----------------------------
-FAYRKDKNGNFKLDKNKKKIPIRGFEDGKTYI---------------------------------------
WEVTQEIN---------------------------K------HLTTGLDSKNKENNF---------------
-------ACSIKPVQPIQDICPNCQTKHVDLSASVEWISQFTQPRPNVACARTCVLILKNSKLSSSAGSPTG

LFQVAL-----------------ENSNHSTIEATSNFQNGLNYLDKELNVGHPVMIGVDHKLGYGINEGTTD
HFIVVVGRGCENGKVYYRFYDVGTRHKEKGTSNSNKLYIINGFLRGKTAYNGSTYTMTQVRRN---------
----------------------------
>CIP70.10_31
-------------------------------MVDLNKDDVA-----LITQAIAIGFSPAGVAL---------
------------------------------------------------------------------------
---------------------DVADLARASYNLY------------------------------QEKSDDNY
F---------------------------------------------------EVILCVIGFIPGPGD-----
------------------------------GLKAGLRIVNRKPEILFELIRFIMTHCKIYGNPEQWLSEIIS
DT----------------------------------------------------------------------
------------KIRELIRSGKREALNASNKNIGNRWAKYWIN---------------------QSIEITFN
FLEASISSLVQLLARKVLHWKTKIPKSSADKRHVGGGKGHQNNSAHGTT-----------------------
----YDGKTGQNGN---------------------------------GHGSKGKTLSKVDISKVLSNLEVGG
VG--------EHMADYWVA---------------------NKLAIPAVHDSGQQLTPKLQRPMTKLHIGIHD
QGIDAIWKSDKKNIGIMNTKSYAIIEAKASLSMGKGTGPGSLLNDLDKQQD---------------------
----------------------------------------------------------------------TR
ERHIERASAK--------------------------------------------------------------
-----------------KEKRAEKKLIPKKKFL---------------------------------------
------------------------------------------INQQMSDEWVKKRLRAT-------------
-------SLRNVEIAGFSRHLLYFNYAHPETVEHLNVLAISIQ-----------------------------
-------------------------------------------------------------DNQPVDHSEHL
NEHTPSNFWGAAKIDEALNNRVTRANIKNGHTK---------------------------------------
----------------------------
>R2091_31
-------------------------------MVDLNKDDVA-----LITQAIAIGFSPAGVAL---------
------------------------------------------------------------------------
---------------------DVADLARASYNLY------------------------------QEKSDDNY
F---------------------------------------------------EVILCVIGFIPGPGD-----
------------------------------GLKAGLRIVNRKPEILFELIRFIMTHCKIYGNPEQWLSEIIS
DT----------------------------------------------------------------------
------------KIRELIRSGKREALNASNKNIGNRWAKYWIN---------------------QSIEITFN
FLEASISSLVQLLARKVLHWKTKIPKSSADKRHVGGGKGHQNNSAHGTT-----------------------
----YDGKTGQNGN---------------------------------GHGSKGKTLSKVDISKVLSNLEVGG

```
VG--------EHMADYWVA---------------------NKLAIPAVHDSGQQLTPKLQRPMTKLHIGIHD
QGIDAIWKSDKKNIGIMNTKSYAIIEAKASLSMGKGTGPGSLLNDLDKQQD---------------------
----------------------------------------------------------------------TR
ERHIERASAK--------------------------------------------------------------
-----------------KEKRAEKKLIPKKKFL---------------------------------------
------------------------------------------INQQMSDEWVKKRLRAT-------------
-------SLRNVEIAGFSRHLLYFNYAHPETVEHLNVLAISIQ-----------------------------
-------------------------------------------------------------DNQPVDHSEHL
NEHTPSNFWGAAKIDEALNNRVTRANIKNGHTK---------------------------------------
----------------------------
>ab736_7
------------------------------MSTSETYLNDE-------------------------------
------------------------------------------------------------------------
---------------------EIFELVRDIPLDT---------------------------SIIYGEEDQEF
GICPYITFYIYHQDNEVDEVANKIIDLYEEFENE------------------IIDKPFKLRYRDTGVWKNAN
KW-----------------------RPSRQVMLDEMHESYKK----YFVYFIGATTGDSGGQSARWALQAII
RDNG--------------------------------------------------------------------
------------LRYTSLKISFGDKWFRENKKK-------------------------------WYAFVENC
LIKLNPIQAYSGYEIGSPQSFNCVSPEFETVE----------------------------------------
----RIFSDYFYGL-----------------------------------------DIDHPSNMSFSHDDPSG
LI---------------------------------------YTPSLAAGIRTPTWCFLLSPYWIEKLGLSEE
QIRLKLNDPRIEITKLPDPADPEKYSLWIRLGELSLYPIEEGVPDLL-------------------------
----------------------------------------------------------------------VM
ANELIKPIRC--------------------------------------------------------------
--------------NDLKLTTLDAWDDDPNPRF---------------------------------------
------------------------------------DIDNSPQWIARFDEDSHWPEG---------------
-------KRVNKIHAVLLEQNAIKVLGGEICPKTGEWYSPANNMKKRYFA----------------------
-------------------------------------------------------------EGEIMPEIKDN
PWGETIWYLEVTNKTE--------------------------------------------------------
----------------------------
>ACICU_7
------------------------------MSTSETYLNDE-------------------------------
------------------------------------------------------------------------
```

```
---------------------EIFELVRDIPLDT---------------------------SIIYGEEDQEF
GICPYITFYIYHQDNEVDEVANKIIDLYEEFENE------------------IIDKPFKLRYRDTGVWKNAN
KW-----------------------RPSRQVMLDEMHESYKK----YFVYFIGATTGDSGGQSARWALQAII
RDNG--------------------------------------------------------------------
------------LRYTSLKISFGDKWFRENKKK-------------------------------WYAFVENC
LIKLNPIQAYSGYEIGSPQSFNCVSPEFETVE----------------------------------------
----RIFSDYFYGL-----------------------------------------DIDHPSNMSFSHDDPSG
LI---------------------------------------YTPSLAAGIRTPTWCFLLSPYWIEKLGLSEE
QIRLKLNDPRIEITKLPDPADPEKYSLWIRLGELSLYPIEEGVPDLL-------------------------
----------------------------------------------------------------------VM
ANELIKPIRC--------------------------------------------------------------
--------------NDLKLTTLDAWDDDPNPRF---------------------------------------
------------------------------------DIDNSPQWIARFDEDSHWPEG---------------
-------KRVNKIHAVLLEQNAIKVLGGEICPKTGEWYSPANNMKKRYFA----------------------
-------------------------------------------------------------EGEIMPEIKDN
PWGETIWYLEVTNKTE--------------------------------------------------------
----------------------------
>ATCC19606_7
------------------------------MSTSETYLNDE-------------------------------
------------------------------------------------------------------------
---------------------EIFELVRDIPLDT---------------------------SIIYGEEDQEF
GICPYITFYIYHQDNEVDEVANKIIDLYEEFENE------------------IIDKPFKLRYRDTGVWKNAN
KW-----------------------RPSRQVMLDEMHESYKK----YFVYFIGATTGDSGGQSARWALQAII
RDNG--------------------------------------------------------------------
------------LRYTSLKISFGDKWFRENKKK-------------------------------WYAFVENC
LIKLNPIQAYSGYEIGSPQSFNCVSPEFETVE----------------------------------------
----RIFSDYFYGL-----------------------------------------DIDHPSNMSFSHDDPSG
LI---------------------------------------YTPSLAAGIRTPTWCFLLSPYWIEKLGLSEE
QIRLKLNDPRIEITKLPDPADPEKYSLWIRLGELSLYPIEEGVPDLL-------------------------
----------------------------------------------------------------------VM
ANELIKPIRC--------------------------------------------------------------
--------------NDLKLTTLDAWDDDPNPRF---------------------------------------
------------------------------------DIDNSPQWIARFDEDSHWPEG---------------
```

-------KRVNKIHAVLLEQNAIKVLGGEICPKTGEWYSPANNMKKRYFA----------------------
-------------------------------------------------------------EGEIMPEIKDN
PWGETIWYLEVTNKTE--------------------------------------------------------
----------------------------
>AB042_3
----MNPYTIFEYTIVFYNQRNKELPNVKYSIIFFCEDGTK-----KTYEGATNPKGQTKPIPLNQNGKLHI
FVEGHETIFSPKKTIKPILAEGSNIVEVNDIRLKSNTS----------------------------------
---------------------FLTKA-QYELMQK------------------------------KTSKDLEE
L----RKNAKAVKQKNFFNLSKIRPTFPLDLAEELNERINMSYEEYRKK---NTHIVKKTKYLKFKNYALYR
FVDSAGNGIPIVDYQIFAQGQSRPLINARPGPPDKKGYTQLV----YTHLKSRVTYTLGSARKNSEWYEPIT
CVDKQTIY--------------------------------------------------QIIFPTSIAVTQPD
INHK--------ENMEARQKPPIVINPTTNEVLILPSAVYAEFDKKTKILSDAV----------REVHKSNA
NLIKAIQSRDLDEIKELEKRLNINQEKAIEKINGEFKQTADLREVWVVETTGKTNEKISKYNLKRRYLKVTE
YEELKAKRRNPQTE--VDVTANQYTVQQPAQIRSSF-----EKLSQQLLTVKGNAGSDEKAVYNLIGGLGGE
IAEEYKNSRDVTVTQEAQW----MRMVAGASGEGSISAAPKGVSIKTSGDMSAKWTLFEGVKEWRKFYPCET
GWKLEYDNYDLGTIRFLIGAEISGFSGANLGIAGNLSVDISHQGAAQVIKAVVRPPERSMSQMVDRNKKPMF
QPAQGSLKI-------------------------------------------------------------IG
NNNQENAQNQ-------------------GNISINAFAGVQIQGLLKGAVEWFKPKGDGSGEGEFVAIASAA
AGGGVSAGVGAQGQFQIGYDQTSGNFKILVAAH---------------------------------------
LCWGMGAK------------GVASFVVGTEHLLSYLGFIKSQVAHAGFKTLLYINEAAF-LLAAQVLAYCIG
ENHPVISDINRIAASYGDWIRRLDIDQGRYKTAQNINSSSGKKELLYATPETKGILLYAVTHWTDRTAPIFD
MNVKFSDMKIEFFPTRKTAVINIFKTCISTEEWENTIQHIHPRGNKLTQTQLGKV------EGDIIRFLNYG
NDEKYAEDIIRCLNSGIEYKGTQINAWLQDYLKYRKGAKKIAGSLNYMLVKNQDDNRFKQLEIQQGVWGDSE
EITLIASNLNVLSPFDQDDTDKYETYNV
>ATCC17978-mff_3
----MNPYTIFEYTIVFYNQRNKELPNVKYSIIFFCEDGTK-----KTYEGATNPKGQTKPIPLNQNGKLHI
FVEGHETIFSPKKTIKPILAEGSNIVEVNDIRLKSNTS----------------------------------
---------------------FLTKA-QYELMQK------------------------------KTSKDLEE
L----RKNAKAVKQKNFFNLSKIRPTFPLDLAEELNERINMSYEEYRKK---NTHIVKKTKYLKFKNYALYR
FVDSAGNGIPIVDYQIFAQGQSRPLINARPGPPDKKGYTQLV----YTHLKSRVTYTLGSARKNSEWYEPIT
CVDKQTIY--------------------------------------------------QIIFPTSIAVTQPD
INHK--------ENMEARQKPPIVINPTTNEVLILPSAVYAEFDKKTKILSDAV----------REVHKSNA
NLIKAIQSRDLDEIKELEKRLNINQEKAIEKINGEFKQTADLREVWVVETTGKTNEKISKYNLKRRYLKVTE

```
YEELKAKRRNPQTE--VDVTANQYTVQQPAQIRSSF-----EKLSQQLLTVKGNAGSDEKAVYNLIGGLGGE
IAEEYKNSRDVTVTQEAQW----MRMVAGASGEGSISAAPKGVSIKTSGDMSAKWTLFEGVKEWRKFYPCET
GWKLEYDNYDLGTIRFLIGAEISGFSGANLGIAGNLSVDISHQGAAQVIKAVVRPPERSMSQMVDRNKKPMF
QPAQGSLKI-------------------------------------------------------------IG
NNNQENAQNQ-------------------GNISINAFAGVQIQGLLKGAVEWFKPKGDGSGEGEFVAIASAA
AGGGVSAGVGAQGQFQIGYDQTSGNFKILVAAH---------------------------------------
LCWGMGAK------------GVASFVVGTEHLLSYLGFIKSQVAHAGFKTLLYINEAAF-LLAAQVLAYCIG
ENHPVISDINRIAASYGDWIRRLDIDQGRYKTAQNINSSSGKKELLYATPETKGILLYAVTHWTDRTAPIFD
MNVKFSDMKIEFFPTRKTAVINIFKTCISTEEWENTIQHIHPRGNKLTQTQLGKV------EGDIIRFLNYG
NDEKYAEDIIRCLNSGIEYKGTQINAWLQDYLKYRKGAKKIAGSLNYMLVKNQDDNRFKQLEIQQGVWGDSE
EITLIASNLNVLSPFDQDDTDKYETYNV
>AR_0088_32
--------------------MSTAVAKSPAKTIRVTQKGEQSAADWIKGVLDGSGSSPAQIIVAVVIGC---
------------------------------------------------------------------------
---------------------IPFVG-QGVDVGN------------------------------VIVSIVKI
A--ENPNNKDNWFDLVFNLIAFVPVA--------------------------GDGLKIVFKQLRSGK-----
AMGA---------------------ILDAIPSKTMRGNVEKW----FRNLNWNAYTKELQTTSNKIIDGLID
V-----------------------------------------------------------------------
------------FDSWMTRAVLGQARLKTL----------------------------------VVQLRKMK
TVANRQIEMVMKDLQLAHKKALATPYPNTTAKA---------------------------------------
----PIHKTGSKPL----------------------------------------------NRTSAPHTQQKG
QV----------------------------------LKNTSGNTAKTGSKNTSTKRTSKKRSNKELGSGGEH
ITDYYFVKRKKSRTKINNNGVLYEYHDTGHDGIDHAWYSNS-------------------------------
----------------------------------------------------------------------IG
HKYRITDSKA--------------------------------------------------------------
-----------------TNLASHRKIMTPKAAM---------------------------------------
-----------------------------------D------ALSMGLDVYVKSSKE---------------
-------KKTKGALGKTVSDGAQMSHLWIANKIGSAKITSSHKVKLLEQIEAWKRVEFKPASERVFKNGKGQ
TVA----------------------------------------------------------VKCPYDRSLVT
ITGNQFDHHSQCKGLDEPKCTRSVTSHAITLEFVLPNEMLRE------------------------------
----------------------------
>Ab04-mff_13
------------------------------MSNSLDWNSKT-----NISEHSCKDAHCDCL-----------
```

------------------------------------------------------------------------
---------------------TFWEI-YQQEAIK------------------------------RLSNLQKA
N----NYKLEPGYEARARRIASVYAK--------------------------IFLEIELGGNKN--------
-------------------------LLGRYYWM-GLGAFASK------------------------------
------------------------------------------------------------------------
------------TVATIFKHW-GSLLGYNVGVQEAI----------------------------NLFARGNL
WLFMDIVPWHLA------------------------------------------------------WSASSE
S---FKSCKGTRDT-------STFKFVKPALMNLPW----------------------SSCLPSIKNLKVTK
EINDAFT-----------------------------LLPAIEKAFLNGRDEKSKYRKASQNLFDHLMAIAVQ
EQHNILQVVVWENCSVKFGARMQRWFIGMPDATLVLSSDYSVDAVKKN------------------------
----------------------------------------------------------------------WF
GNYTGSKADQ--------------------------------------------------------------
-----------------LVELKEDVYIAPLKGT---------------------------------------
IAEDYDSR---------------------------M------KWIGKAAEKY--------------------
------------------------------------------------------------------------
-------------------------------------------------------------HRLMLDEKGRP
FLQQELKTISKWGNSKADFKIHSSSNEGKV------------------------------------------
----------------------------
>LAC-4_13
------------------------------MSNSLDWNSKT-----NISEHSCKDAHCDCL-----------
------------------------------------------------------------------------
---------------------TFWEI-YQQEAIK------------------------------RLSNLQKA
N----NYKLEPGYEARARRIASVYAK--------------------------IFLEIELGGNKN--------
-------------------------LLGRYYWM-GLGAFASK------------------------------
------------------------------------------------------------------------
------------TVATIFKHW-GSLLGYNVGVQEAI----------------------------NLFARGNL
WLFMDIVPWHLA------------------------------------------------------WSASSE
S---FKSCKGTRDT-------STFKFVKPALMNLPW----------------------SSCLPSIKNLKVTK
EINDAFT-----------------------------LLPAIEKAFLNGRDEKSKYRKASQNLFDHLMAIAVQ
EQHNILQVVVWENCSVKFGARMQRWFIGMPDATLVLSSDYSVDAVKKN------------------------
----------------------------------------------------------------------WF
GNYTGSKADQ--------------------------------------------------------------
-----------------LVELKEDVYIAPLKGT---------------------------------------

IAEDYDSR---------------------------M------KWIGKAAEKY--------------------
------------------------------------------------------------------------
-------------------------------------------------------------HRLMLDEKGRP
FLQQELKTISKWGNSKADFKIHSSSNEGKV------------------------------------------
----------------------------
>WKA02_13
------------------------------MSNSLDWNSKT-----NISEHSCKDAHCDCL-----------
------------------------------------------------------------------------
---------------------TFWEI-YQQEAIK------------------------------RLSNLQKA
N----NYKLEPGYEARARRIASVYAK--------------------------IFLEIELGGNKN--------
-------------------------LLGRYYWM-GLGAFASK------------------------------
------------------------------------------------------------------------
------------TVATIFKHW-GSLLGYNVGVQEAI----------------------------NLFARGNL
WLFMDIAPWHLA------------------------------------------------------WSASSE
S---FKSCKGTRDT-------STFKFIKPALMNLPW----------------------SSCLPSIKNLKVTK
EINDAFT-----------------------------LLPAIEKAFLNGRDEKSKYRKASQNLFDHLMAIAVQ
EQHNILQVVVWENCSVKFGAWMQRWFIGMPDATLVLSSDYSVDAVKKN------------------------
----------------------------------------------------------------------WF
GNYTGSKADQ--------------------------------------------------------------
-----------------LVELKEDVYIAPLKGT---------------------------------------
IAEDYDSR---------------------------M------KWIGKAAEKY--------------------
------------------------------------------------------------------------
-------------------------------------------------------------HRLMLDEKGRP
FLQQELKTISKWGNSKADFKIHSSSNEGKV------------------------------------------
----------------------------
>BJAB0715_13
------------------------------MSNSLDWNSKT-----NISEHSCKDAHCDCL-----------
------------------------------------------------------------------------
---------------------TFWEI-YQQEAIK------------------------------RLSNLQKA
N----NYKLEPGYEARARRIASVYAK--------------------------IFLEIELGGNKN--------
-------------------------LLGRYYWM-GLGAFASK------------------------------
------------------------------------------------------------------------
------------TVATIFKHW-GSLLGYNVGVQEAI----------------------------NLFARGNL

```
WLFMDIAPWHLA------------------------------------------------------WSASSE
S---FKSCKGTRDT-------STFKFVKPALMNLPW----------------------SSCLPSIKNLKVTK
EINDAFT-----------------------------LLPAIEKAFLNGRDEKSKYRKASQNLFDHLMAIAVQ
EQHNILQVVVWENCSVKFGAWMQRWFIGMPDATLVLSSDYSVDAVKKN------------------------
----------------------------------------------------------------------WF
GNYTGSKADQ--------------------------------------------------------------
-----------------LVELKEDVYIAPLKGT---------------------------------------
IAEDYDSR---------------------------M------KWIGKAAEKY--------------------
------------------------------------------------------------------------
-------------------------------------------------------------HRLMLDEKGRP
FLQQELKTISKWGNSKADFKIHSSSNEGKV------------------------------------------
----------------------------
>D1279779_13
------------------------------MSNSLDWNSKT-----NISEHSCKDAHCDCL-----------
------------------------------------------------------------------------
---------------------TFWEI-YQQEAIK------------------------------RLSNLQKA
N----NYKLEPGYEARARRIASVYAK--------------------------IFLEIELGGNKN--------
-------------------------LLGRYYWM-GLGAFASK------------------------------
------------------------------------------------------------------------
------------TVATIFKHW-GSLLGYNVGVQEAI----------------------------NLFARGNL
WLFMDIAPWHLA------------------------------------------------------WSASSE
S---FKSCKGTRDT-------STFKFVKPALMNLPW----------------------SSCLPSIKNLKVTK
EINDAFT-----------------------------LLPAIEKAFLNGRDEKSKYRKASQNLFDHLMAIAVQ
EQHNILQVVVWENCSVKFGAWMQRWFIGMPDATLVLSSDYSVDAVKKN------------------------
----------------------------------------------------------------------WF
GNYTGSKADQ--------------------------------------------------------------
-----------------LVELKEDVYIAPLKGT---------------------------------------
IAEDYDSR---------------------------M------KWIGKAAEKY--------------------
------------------------------------------------------------------------
-------------------------------------------------------------HRLMLDEKGRP
FLQQELKTISKWGNSKADFKIHSSSNEGKV------------------------------------------
----------------------------
>KAB02_13
```

```
------------------------------MSNSLDWNSKT-----NISEHSCKDAHCDCL-----------
------------------------------------------------------------------------
---------------------TFWEI-YQQEAIK------------------------------RLSNLQKA
N----NYKLEPGYEARARRIASVYAK--------------------------IFLEIELGGNKN--------
-------------------------LLGRYYWM-GLGAFASK------------------------------
------------------------------------------------------------------------
------------TVATIFKHW-GSLLGYNVGVQEAI----------------------------NLFARGNL
WLFMDIAPWHLA------------------------------------------------------WSASSE
S---FKSCKGTRDT-------STFKFVKPALMNLPW----------------------SSCLPSIKNLKVTK
EINDAFT-----------------------------LLPAIEKAFLNGRDEKSKYRKASQNLFDHLMAIAVQ
EQHNILQVVVWENCSVKFGAWMQRWFIGMPDATLVLSSDYSVDAVKKN------------------------
----------------------------------------------------------------------WF
GNYTGSKADQ--------------------------------------------------------------
-----------------LVELKEDVYIAPLKGT---------------------------------------
IAEDYDSR---------------------------M------KWIGKAAEKY--------------------
------------------------------------------------------------------------
-------------------------------------------------------------HRLMLDEKGRP
FLQQELKTISKWGNSKADFKIHSSSNEGKV------------------------------------------
----------------------------
>KAB05_13
------------------------------MSNSLDWNSKT-----NISEHSCKDAHCDCL-----------
------------------------------------------------------------------------
---------------------TFWEI-YQQEAIK------------------------------RLSNLQKA
N----NYKLEPGYEARARRIASVYAK--------------------------IFLEIELGGNKN--------
-------------------------LLGRYYWM-GLGAFASK------------------------------
------------------------------------------------------------------------
------------TVATIFKHW-GSLLGYNVGVQEAI----------------------------NLFARGNL
WLFMDIAPWHLA------------------------------------------------------WSASSE
S---FKSCKGTRDT-------STFKFVKPALMNLPW----------------------SSCLPSIKNLKVTK
EINDAFT-----------------------------LLPAIEKAFLNGRDEKSKYRKASQNLFDHLMAIAVQ
EQHNILQVVVWENCSVKFGAWMQRWFIGMPDATLVLSSDYSVDAVKKN------------------------
----------------------------------------------------------------------WF
GNYTGSKADQ--------------------------------------------------------------
```

-----------------LVELKEDVYIAPLKGT---------------------------------------
IAEDYDSR---------------------------M------KWIGKAAEKY--------------------
------------------------------------------------------------------------
-------------------------------------------------------------HRLMLDEKGRP
FLQQELKTISKWGNSKADFKIHSSSNEGKV------------------------------------------
----------------------------
>MDR-TJ_13
------------------------------MSNSLDWNSKT-----NISEHSCKDAHCDCL-----------
------------------------------------------------------------------------
---------------------TFWEI-YQQEAIK------------------------------RLSNLQKA
N----NYKLEPGYEARARRIASVYAK--------------------------IFLEIELGGNKN--------
-------------------------LLGRYYWM-GLGAFASK------------------------------
------------------------------------------------------------------------
------------TVATIFKHW-GSLLGYNVGVQEAI----------------------------NLFARGNL
WLFMDIAPWHLA------------------------------------------------------WSASSE
S---FKSCKGTRDT-------STFKFVKPALMNLPW----------------------SSCLPSIKNLKVTK
EINDAFT-----------------------------LLPAIEKAFLNGRDEKSKYRKASQNLFDHLMAIAVQ
EQHNILQVVVWENCSVKFGAWMQRWFIGMPDATLVLSSDYSVDAVKKN------------------------
----------------------------------------------------------------------WF
GNYTGSKADQ--------------------------------------------------------------
-----------------LVELKEDVYIAPLKGT---------------------------------------
IAEDYDSR---------------------------M------KWIGKAAEKY--------------------
------------------------------------------------------------------------
-------------------------------------------------------------HRLMLDEKGRP
FLQQELKTISKWGNSKADFKIHSSSNEGKV------------------------------------------
----------------------------
>R2090_13
------------------------------MSNSLDWNSKT-----NISEHSCKDAHCDCL-----------
------------------------------------------------------------------------
---------------------TFWEI-YQQEAIK------------------------------RLSNLQKA
N----NYKLEPGYEARARRIASVYAK--------------------------IFLEIELGGNKN--------
-------------------------LLGRYYWM-GLGAFASK------------------------------
------------------------------------------------------------------------

```
------------TVATIFKHW-GSLLGYNVGVQEAI----------------------------NLFARGNL
WLFMDIAPWHLA------------------------------------------------------WSASSE
S---FKSCKGTRDT-------STFKFVKPALMNLPW----------------------SSCLPSIKNLKVTK
EINDAFT-----------------------------LLPAIEKAFLNGRDEKSKYRKASQNLFDHLMAIAVQ
EQHNILQVVVWENCSVKFGAWMQRWFIGMPDATLVLSSDYSVDAVKKN------------------------
----------------------------------------------------------------------WF
GNYTGSKADQ--------------------------------------------------------------
-----------------LVELKEDVYIAPLKGT---------------------------------------
IAEDYDSR---------------------------M------KWIGKAAEKY--------------------
------------------------------------------------------------------------
-------------------------------------------------------------HRLMLDEKGRP
FLQQELKTISKWGNSKADFKIHSSSNEGKV------------------------------------------
----------------------------
>XH856_13
------------------------------MSNSLDWNSKT-----NISEHSCKDAHCDCL-----------
------------------------------------------------------------------------
---------------------TFWEI-YQQEAIK------------------------------RLSNLQKA
N----NYKLEPGYEARARRIASVYAK--------------------------IFLEIELGGNKN--------
-------------------------LLGRYYWM-GLGAFASK------------------------------
------------------------------------------------------------------------
------------TVATIFKHW-GSLLGYNVGVQEAI----------------------------NLFARGNL
WLFMDIAPWHLA------------------------------------------------------WSASSE
S---FKSCKGTRDT-------STFKFVKPALMNLPW----------------------SSCLPSIKNLKVTK
EINDAFT-----------------------------LLPAIEKAFLNGRDEKSKYRKASQNLFDHLMAIAVQ
EQHNILQVVVWENCSVKFGAWMQRWFIGMPDATLVLSSDYSVDAVKKN------------------------
----------------------------------------------------------------------WF
GNYTGSKADQ--------------------------------------------------------------
-----------------LVELKEDVYIAPLKGT---------------------------------------
IAEDYDSR---------------------------M------KWIGKAAEKY--------------------
------------------------------------------------------------------------
-------------------------------------------------------------HRLMLDEKGRP
FLQQELKTISKWGNSKADFKIHSSSNEGKV------------------------------------------
----------------------------
```

```
>XH858_13
------------------------------MSNSLDWNSKT-----NISEHSCKDAHCDCL-----------
------------------------------------------------------------------------
---------------------TFWEI-YQQEAIK------------------------------RLSNLQKA
N----NYKLEPGYEARARRIASVYAK--------------------------IFLEIELGGNKN--------
-------------------------LLGRYYWM-GLGAFASK------------------------------
------------------------------------------------------------------------
------------TVATIFKHW-GSLLGYNVGVQEAI----------------------------NLFARGNL
WLFMDIAPWHLA------------------------------------------------------WSASSE
S---FKSCKGTRDT-------STFKFVKPALMNLPW----------------------SSCLPSIKNLKVTK
EINDAFT-----------------------------LLPAIEKAFLNGRDEKSKYRKASQNLFDHLMAIAVQ
EQHNILQVVVWENCSVKFGAWMQRWFIGMPDATLVLSSDYSVDAVKKN------------------------
----------------------------------------------------------------------WF
GNYTGSKADQ--------------------------------------------------------------
-----------------LVELKEDVYIAPLKGT---------------------------------------
IAEDYDSR---------------------------M------KWIGKAAEKY--------------------
------------------------------------------------------------------------
-------------------------------------------------------------HRLMLDEKGRP
FLQQELKTISKWGNSKADFKIHSSSNEGKV------------------------------------------
----------------------------
>KAB06_13
------------------------------MSNSLDWNSKT-----NISEHSCKDAHCDCL-----------
------------------------------------------------------------------------
---------------------TFWEI-YQQEAIK------------------------------RLSNLQKA
N----NYKLEPGYEARARRIASVYAK--------------------------IFLEIELGGNKN--------
-------------------------LLGRYYWM-GLGAFASK------------------------------
------------------------------------------------------------------------
------------TVATIFKHW-GSLLGYNVGVQEAI----------------------------NLFARGNL
WLFMDIAPWHLA------------------------------------------------------WSASSE
S---FKSCKGTRDT-------STFKFVKPALMNLPW----------------------SSCLPSIKNLKVTK
EINDAFT-----------------------------LLPAIEKAFLNGRDEKSKYRKASQNLFDHLMAIAVQ
EQHNILQVVVWE-CSVKFGAWMQRWFIGMPDATLVLSSDYSVDAVKKN------------------------
----------------------------------------------------------------------WF
```

```
GNYTGSKADQ--------------------------------------------------------------
-----------------LVELKEDVYIAPLKGT---------------------------------------
IAEDYDSR---------------------------M------KWIGKAAEKY--------------------
------------------------------------------------------------------------
-------------------------------------------------------------HRLMLDEKGRP
FLQQELKTISKWGNSKADFKIHSSSNEGKV------------------------------------------
----------------------------
>AB030_22
---------------------------------NSKKTPAQ-----LTADRAR-------------------
------------------------------------------------------------------------
---------------------------FELGKTS------------------------------GAAADLQV
G---------------------------------------------------NVNITGISGRVG-KN-----
------------------------------------------------------------------------
------------------------------------------------------------------------
------------VHPQLKKALRDVPKEE------------------------------------QAPWHGAC
AEVDAINKALKKG-----------------------------------------------------------
-----MNIEGATID----------------------------------------------------------
------------------------------------------------------------------------
------------------------------------------------------------------------
----------------------------------------------------------------------VV
NINSNDKRHG--------------------------------------------------------------
-----------------------THKPACSSCS---------------------------------------
------------------------------------------NVLKQFGVKSNEK-----------------
------------------------------------------------------------------------
------------------------------------------------------------------------
------------------------------------------------------------------------
----------------------------
>AbH12O-A2_22
---------------------------------NSKKTPAQ-----LTADRAR-------------------
------------------------------------------------------------------------
---------------------------FELGKTS------------------------------GAAADLQV
G---------------------------------------------------NVNITGISGRVG-KN-----
------------------------------------------------------------------------
```

------------------------------------------------------------------------
------------VHPQLKKALRDVPKEE------------------------------------QAPWHGAC
AEVDAINKALKKG-----------------------------------------------------------
-----MNIEGATID----------------------------------------------------------
------------------------------------------------------------------------
------------------------------------------------------------------------
----------------------------------------------------------------------VV
NINSNDKRHG--------------------------------------------------------------
-----------------------THKPACSSCS---------------------------------------
------------------------------------------NVLKQFGVKSNEK-----------------
------------------------------------------------------------------------
------------------------------------------------------------------------
------------------------------------------------------------------------
----------------------------
>AF-401_22
---------------------------------NSKKTPAQ-----LTADRAR-------------------
------------------------------------------------------------------------
---------------------------FELGKTS------------------------------GAAADLQV
G---------------------------------------------------NVNITGISGRVG-KN-----
------------------------------------------------------------------------
------------------------------------------------------------------------
------------VHPQLKKALRDVPKEE------------------------------------QAPWHGAC
AEVDAINKALKKG-----------------------------------------------------------
-----MNIEGATID----------------------------------------------------------
------------------------------------------------------------------------
------------------------------------------------------------------------
----------------------------------------------------------------------VV
NINSNDKRHG--------------------------------------------------------------
-----------------------THKPACSSCS---------------------------------------
------------------------------------------NVLKQFGVKSNEK-----------------
------------------------------------------------------------------------
------------------------------------------------------------------------
------------------------------------------------------------------------

```
----------------------------
>AR_0063_22
---------------------------------NSKKTPAQ-----LTADRAR-------------------
------------------------------------------------------------------------
---------------------------FELGKTS------------------------------GAAADLQV
G---------------------------------------------------NVNITGISGRVG-KN-----
------------------------------------------------------------------------
------------------------------------------------------------------------
------------VHPQLKKALRDVPKEE------------------------------------QAPWHGAC
AEVDAINKALKKG-----------------------------------------------------------
-----MNIEGATID----------------------------------------------------------
------------------------------------------------------------------------
------------------------------------------------------------------------
----------------------------------------------------------------------VV
NINSNDKRHG--------------------------------------------------------------
-----------------------THKPACSSCS---------------------------------------
------------------------------------------NVLKQFGVKSNEK-----------------
------------------------------------------------------------------------
------------------------------------------------------------------------
------------------------------------------------------------------------
----------------------------
>AR_0101_22
---------------------------------NSKKTPAQ-----LTADRAR-------------------
------------------------------------------------------------------------
---------------------------FELGKTS------------------------------GAAADLQV
G---------------------------------------------------NVNITGISGRVG-KN-----
------------------------------------------------------------------------
------------------------------------------------------------------------
------------VHPQLKKALRDVPKEE------------------------------------QAPWHGAC
AEVDAINKALKKG-----------------------------------------------------------
-----MNIEGATID----------------------------------------------------------
------------------------------------------------------------------------
------------------------------------------------------------------------
```

```
----------------------------------------------------------------------VV
NINSNDKRHG--------------------------------------------------------------
-----------------------THKPACSSCS---------------------------------------
------------------------------------------NVLKQFGVKSNEK-----------------
------------------------------------------------------------------------
------------------------------------------------------------------------
------------------------------------------------------------------------
----------------------------
>IOMTU433_22
---------------------------------NSKKTPAQ-----LTADRAR-------------------
------------------------------------------------------------------------
---------------------------FELGKTS------------------------------GAAADLQV
G---------------------------------------------------NVNITGISGRVG-KN-----
------------------------------------------------------------------------
------------------------------------------------------------------------
------------VHPQLKKALRDVPKEE------------------------------------QAPWHGAC
AEVDAINKALKKG-----------------------------------------------------------
-----MNIEGATID----------------------------------------------------------
------------------------------------------------------------------------
------------------------------------------------------------------------
----------------------------------------------------------------------VV
NINSNDKRHG--------------------------------------------------------------
-----------------------THKPACSSCS---------------------------------------
------------------------------------------NVLKQFGVKSNEK-----------------
------------------------------------------------------------------------
------------------------------------------------------------------------
------------------------------------------------------------------------
----------------------------
>A1296_8
--------------------------------MYIVKKGDT-----LTSIANKNGCTVEQIIKLNSIKNKN-
------------------------------------------------------------------------
---------------------LIFVG-QKLKLKN------------------------------TPTPIQNL
G----GKVGIAIVNKKGEPISNFKLKIQVGNNIYSNQLTN------------ARGVVTLPRQKS-GENIIIQ
```

GWSK---------------------TNNSYKLISNRFITENR----NLGIGISLDTIKFETKTDNHKKPINN
NVNNNGQS-----------------------------------------------------------TTTNG
SNSQ--------NNPIARTSVEGCSNCTKITENELK----------------------------QIFTQANS
NDIKKIIDVYTNFSEKFGMNNCLSKAHFFAQVLEEVGKKIEVKDGESLNYHSDILFLSYTKIKRKEYIIEKG
KRVEKMTHGGPFSA-------FRGNRQLCDKYGR-----------------NDNHPADQVMIANIAYANRNG
NG-------DIQSGDGWKY-------------------RGRGIIQITGKDKYDKINKAIKDNYPNVGISIDA
NNINNIYEGTLASMAYWKSFGLSKLATQKQVDIRTQLEVVDSLIDI--------------------------
----------------------------------------------------------------------IN
RDTASRSDRK-------------------KNFEYITAKVFKLNECKNSIVEANLLSKSTPTPTPTPTPTPTP
TPTP---------------------TPTPTPTL---------------------------------------
SNVLKEIK----------------------------------QLVDRNIPYSQTGARGA-------------
-------GSNKNATSVITANDLKGLDCSETVAIYLLKLGVTDK--FYSIHT--GVMLTENDFRKAIRSNKIE
YVVG-----------------------------SKDLNFIPQIGDIFVWRNGGGH------CGIVYDVDRQN
DTVTILEAIGDVGSADENFNINNGGEKKVGCTRTAVYRRSGKALAQHRGWVGYFRPIISGKKI---------
----------------------------
>1656-2_8
--------------------------------MYIVKKGDT-----LTSIANKNGCTVEQIIKLNSIKNKN-
------------------------------------------------------------------------
---------------------LIFVG-QKLKLKNTPTPTPTPTPTPTPTPTPTPTPTPTPTPTPTPTPIQNL
G----GKVGIAIVNKKGEPISNFKLKIQVGNNIYSNQLTN------------ARGVVTLPRQKS-GENIIIQ
GWSK---------------------TNNSYKLISNRFITENR----NLGIGISLDTIKFETKTDNHKKPINN
NVNNNGQS-----------------------------------------------------------TTTNG
SNSQ--------NNPIARTSVEGCSNCTKITENELK----------------------------QIFTQANS
NDIKKIIDVYTNFSEKFGMNNCLSKAHFFAQVLEEVGKKIEVKDGESLNYHSDILFLSYTKIKRKEYIIEKG
KRVEKMTHGGPFSA-------FRGNRQLCDKYGR-----------------NDNHPADQVMIANIAYANRNG
NG-------DIQSGDGWKY-------------------RGRGIIQITGKDKYDKINKAIKDNYPNVGISIDA
NNINNIYEGTLASMAYWKSFGLSKLATQKQVDIRTQLEVVDSLIDI--------------------------
----------------------------------------------------------------------IN
RDTASRSDRK-------------------KNFEYITAKVFKLNECKNSIVEANLLSKSTPTPTPTP------
-------------------------TPTPTPTL---------------------------------------
SNVLKEIK----------------------------------QLVDRNIPYSQTGARGA-------------
-------GSNKNATSVITANDLKGLDCSETVAIYLLKLGVTDK--FYSIHT--GVMLTENDFRKAIRSNKIE
YVVG-----------------------------SKDLNFIPQIGDIFVWRNGGGH------CGIVYDVDRQN

```
DTVTILEAIGDVGSADENFNINNGGEKKVGCTRTAVYRRSGKALAQHRGWVGYFRPIISGKKI---------
----------------------------
>ACICU_8
--------------------------------MYIVKKGDT-----LTSIANKNGCTVEQIIKLNSIKNKN-
------------------------------------------------------------------------
---------------------LIFVG-QKLKLKN--------------------TPTPTPTPTPTPTPIQNL
G----GKVGIAIVNKKGEPISNFKLKIQVGNNIYSNQLTN------------ARGVVTLPRQKS-GENIIIQ
GWSK---------------------TNNSYKLISNRFITENR----NLGIGISLDTIKFETKTDNHKKPINN
NVNNNGQS-----------------------------------------------------------TTTNG
SNSQ--------NNPIARTSVEGCSNCTKITENELK----------------------------QIFTQANS
NDIKKIIDVYTNFSEKFGMNNCLSKAHFFAQVLEEVGKKIEVKDGESLNYHSDILFLSYTKIKRKEYIIEKG
KRVEKMTHGGPFSA-------FRGNRQLCDKYGR-----------------NDNHPADQVMIANIAYANRNG
NG-------DIQSGDGWKY-------------------RGRGIIQITGKDKYDKINKAIKDNYPNVGISIDA
NNINNIYEGTLASMAYWKSFGLSKLATQKQVDIRTQLEVVDSLIDI--------------------------
----------------------------------------------------------------------IN
RDTASRSDRK-------------------KNFEYITAKVFKLNECKNSIVEANLLSKSTPTPTP--------
-------------------------TPTPTPTL---------------------------------------
SNVLKEIK----------------------------------QLVDRNIPYSQTGARGA-------------
-------GSNKNATSVITANDLKGLDCSETVAIYLLKLGVTDK--FYSIHT--GVMLTENDFRKAIRSNKIE
YVVG-----------------------------SKDLNFIPQIGDIFVWRNGGGH------CGIVYDVDRQN
DTVTILEAIGDVGSADENFNINNGGEKKVGCTRTAVYRRSGKALAQHRGWVGYFRPIISGKKI---------
----------------------------
>AR_0056_8
--------------------------------MYIVKKGDT-----LTSIANKNGCTVEQIIKLNSIKNKN-
------------------------------------------------------------------------
---------------------LIFVG-QKLKLKN----------------------TPTPTPTPTPTPIQNL
G----GKVGIAIVNKKGEPISNFKLKIQVGNNIYSNQLTN------------ARGVVTLPRQKS-GENIIIQ
GWSK---------------------TNNSYKLISNRFITENR----NLGIGISLDTIKFETKTDNHKKPINN
NVNNNGQS-----------------------------------------------------------TTTNG
SNSQ--------NNPIARTSVEGCSNCTKITENELK----------------------------QIFTQANS
NDIKKIIDVYTNFSEKFGMNNCLSKAHFFAQVLEEVGKKIEVKDGESLNYHSDILFLSYTKIKRKEYIIEKG
KRVEKMTHGGPFSA-------FRGNRQLCDKYGR-----------------NDNHPADQVMIANIAYANRNG
NG-------DIQSGDGWKY-------------------RGRGIIQITGKDKYDKINKAIKDNYPNVGISIDA
```

NNINNIYEGTLASMAYWKSFGLSKLATQKQVDIRTQLEVVDSLIDI--------------------------
----------------------------------------------------------------------IN
RDTASRSDRK-------------------KNFEYITAKVFKLNECKNSIVEANLLSKSTPTPTPTPTPTP--
-------------------------TPTPTPTL---------------------------------------
SNVLKEIK----------------------------------QLVDRNIPYSQTGARGA-------------
-------GSNKNATSVITANDLKGLDCSETVAIYLLKLGVTDK--FYSIHT--GVMLTENDFRKAIRSNKIE
YVVG-----------------------------SKDLNFIPQIGDIFVWRNGGGH------CGIVYDVDRQN
DTVTILEAIGDVGSADENFNINNGGEKKVGCTRTAVYRRSGKALAQHRGWVGYFRPIISGKKI---------
----------------------------
>D1279779_8
--------------------------------MYIVKKGDT-----LTSIANKNGCTVEQIIKLNSIKNKN-
------------------------------------------------------------------------
---------------------LIFVG-QKLKLKN----------------TPTPTPTPTPTPTPTPTPIQNL
G----GKVGIAIVNKKGEPISNFKLKIQVGNNIYSNQLTN------------ARGVVTLPRQKS-GENIIIQ
GWSK---------------------TNNSYKLISNRFITENR----NLGIGISLDTIKFETKTDNHKKPINN
NVNNNGQS-----------------------------------------------------------TTTNG
SNSQ--------NNPIARTSVEGCSNCTKITENELK----------------------------QIFTQANS
NDIKKIIDVYTNFSEKFGMNNCLSKAHFFAQVLEEVGKKIEVKDGESLNYHSDILFLSYTKIKRKEYIIEKG
KRVEKMTHGGPFSA-------FRGNRQLCDKYGR-----------------NDNHPADQVMIANIAYANRNG
NG-------DIQSGDGWKY-------------------RGRGIIQITGKDKYDKINKAIKDNYPNVGISIDA
NNINNIYEGTLASMAYWKSFGLSKLATQKQVDIRTQLEVVDSLIDI--------------------------
----------------------------------------------------------------------IN
RDTASRSDRK-------------------KNFEYITAKVFKLNECKNSIVEANLLSKS--------------
-------------------------TPTPTPTL---------------------------------------
SNVLKEIK----------------------------------QLVDRNIPYSQTGARGA-------------
-------GSNKNATSVITANDLKGLDCSETVAIYLLKLGVTDK--FYSIHT--GVMLTENDFRKAIRSNKIE
YVVG-----------------------------SKDLNFIPQIGDIFVWRNGGGH------CGIVYDVDRQN
DTVTILEAIGDVGSADENFNINNGGEKKVGCTRTAVYRRSGKALAQHRGWVGYFRPIISGKKI---------
----------------------------
>DU202_8
--------------------------------MYIVKKGDT-----LTSIANKNGCTVEQIIKLNSIKNKN-
------------------------------------------------------------------------
---------------------LIFVG-QKLKLKNTPTPTPTPTPTPTPTPTPTPTPTPTPTPTPTPTPIQNL

G----GKVGIAIVNKKGEPISNFKLKIQVGNNIYSNQLTN------------ARGVVTLPRQKS-GENIIIQ
GWSK---------------------TNNSYKLISNRFITENR----NLGIGISLDTIKFETKTDNHKKPINN
NVNNNGQS-----------------------------------------------------------TTTNG
SNSQ--------NNPIARTSVEGCSNCTKITENELK----------------------------QIFTQANS
NDIKKIIDVYTNFSEKFGMNNCLSKAHFFAQVLEEVGKKIEVKDGESLNYHSDILFLSYTKIKRKEYIIEKG
KRVEKMTHGGPFSA-------FRGNRQLCDKYGR-----------------NDNHPADQVMIANIAYANRNG
NG-------DIQSGDGWKY-------------------RGRGIIQITGKDKYDKINKAIKDNYPNVGISIDA
NNINNIYEGTLASMAYWKSFGLSKLATQKQVDIRTQLEVVDSLIDI--------------------------
----------------------------------------------------------------------IN
RDTASRSDRK-------------------KNFEYITAKVFKLNECKNSIVEANLLSKSTPTPTPTPTPTP--
-------------------------TPTPTPTL---------------------------------------
SNVLKEIK----------------------------------QLVDRNIPYSQTGARGA-------------
-------GSNKNATSVITANDLKGLDCSETVAIYLLKLGVTDK--FYSIHT--GVMLTENDFRKAIRSNKIE
YVVG-----------------------------SKDLNFIPQIGDIFVWRNGGGH------CGIVYDVDRQN
DTVTILEAIGDVGSADENFNINNGGEKKVGCTRTAVYRRSGKALAQHRGWVGYFRPIISGKKI---------
----------------------------

>KAB03_8
--------------------------------MYIVKKGDT-----LTSIANKNGCTVEQIIKLNSIKNKN-
------------------------------------------------------------------------
---------------------LIFVG-QKLKLKN----------TPTPTPTPTPTPTPTPTPTPTPTPIQNL
G----GKVGIAIVNKKGEPISNFKLKIQVGNNIYSNQLTN------------ARGVVTLPRQKS-GENIIIQ
GWSK---------------------TNNSYKLISNRFITENR----NLGIGISLDTIKFETKTDNHKKPINN
NVNNNGQS-----------------------------------------------------------TTTNG
SNSQ--------NNPIARTSVEGCSNCTKITENELK----------------------------QIFTQANS
NDIKKIIDVYTNFSEKFGMNNCLSKAHFFAQVLEEVGKKIEVKDGESLNYHSDILFLSYTKIKRKEYIIEKG
KRVEKMTHGGPFSA-------FRGNRQLCDKYGR-----------------NDNHPADQVMIANIAYANRNG
NG-------DIQSGDGWKY-------------------RGRGIIQITGKDKYDKINKAIKDNYPNVGISIDA
NNINNIYEGTLASMAYWKSFGLSKLATQKQVDIRTQLEVVDSLIDI--------------------------
----------------------------------------------------------------------IN
RDTASRSDRK-------------------KNFEYITAKVFKLNECKNSIVEANLLSKSTPTPTPTPTPTP--
-------------------------TPTPTPTL---------------------------------------
SNVLKEIK----------------------------------QLVDRNIPYSQTGARGA-------------
-------GSNKNATSVITANDLKGLDCSETVAIYLLKLGVTDK--FYSIHT--GVMLTENDFRKAIRSNKIE

YVVG-----------------------------SKDLNFIPQIGDIFVWRNGGGH------CGIVYDVDRQN
DTVTILEAIGDVGSADENFNINNGGEKKVGCTRTAVYRRSGKALAQHRGWVGYFRPIISGKKI---------
----------------------------

>NCGM237_8
--------------------------------MYIVKKGDT-----LTSIANKNGCTVEQIIKLNSIKNKN-
------------------------------------------------------------------------
---------------------LIFVG-QKLKLKN----------TPTPTPTPTPTPTPTPTPTPTPTPIQNL
G----GKVGIAIVNKKGEPISNFKLKIQVGNNIYSNQLTN------------ARGVVTLPRQKS-GENIIIQ
GWSK---------------------TNNSYKLISNRFITENR----NLGIGISLDTIKFETKTDNHKKPINN
NVNNNGQS-----------------------------------------------------------TTTNG
SNSQ--------NNPIARTSVEGCSNCTKITENELK----------------------------QIFTQANS
NDIKKIIDVYTNFSEKFGMNNCLSKAHFFAQVLEEVGKKIEVKDGESLNYHSDILFLSYTKIKRKEYIIEKG
KRVEKMTHGGPFSA-------FRGNRQLCDKYGR-----------------NDNHPADQVMIANIAYANRNG
NG-------DIQSGDGWKY-------------------RGRGIIQITGKDKYDKINKAIKDNYPNVGISIDA
NNINNIYEGTLASMAYWKSFGLSKLATQKQVDIRTQLEVVDSLIDI--------------------------
----------------------------------------------------------------------IN
RDTASRSDRK-------------------KNFEYITAKVFKLNECKNSIVEANLLSKSTPTP----------
-------------------------TPTPTPTL---------------------------------------
SNVLKEIK----------------------------------QLVDRNIPYSQTGARGA-------------
-------GSNKNATSVITANDLKGLDCSETVAIYLLKLGVTDK--FYSIHT--GVMLTENDFRKAIRSNKIE
YVVG-----------------------------SKDLNFIPQIGDIFVWRNGGGH------CGIVYDVDRQN
DTVTILEAIGDVGSADENFNINNGGEKKVGCTRTAVYRRSGKALAQHRGWVGYFRPIISGKKI---------
----------------------------

>R2090_8
--------------------------------MYIVKKGDT-----LTSIANKNGCTVEQIIKLNSIKNKN-
------------------------------------------------------------------------
---------------------LIFVG-QKLKLKN--------------TPTPTPTPTPTPTPTPTPTPIQNL
G----GKVGIAIVNKKGEPISNFKLKIQVGNNIYSNQLTN------------ARGVVTLPRQKS-GENIIIQ
GWSK---------------------TNNSYKLISNRFITENR----NLGIGISLDTIKFETKTDNHKKPINN
NVNNNGQS-----------------------------------------------------------TTTNG
SNSQ--------NNPIARTSVEGCSNCTKITENELK----------------------------QIFTQANS
NDIKKIIDVYTNFSEKFGMNNCLSKAHFFAQVLEEVGKKIEVKDGESLNYHSDILFLSYTKIKRKEYIIEKG
KRVEKMTHGGPFSA-------FRGNRQLCDKYGR-----------------NDNHPADQVMIANIAYANRNG

```
NG-------DIQSGDGWKY-------------------RGRGIIQITGKDKYDKINKAIKDNYPNVGISIDA
NNINNIYEGTLASMAYWKSFGLSKLATQKQVDIRTQLEVVDSLIDI--------------------------
----------------------------------------------------------------------IN
RDTASRSDRK-------------------KNFEYITAKVFKLNECKNSIVEANLLSKSTPTPTPTP------
-------------------------TPTPTPTL---------------------------------------
SNVLKEIK----------------------------------QLVDRNIPYSQTGARGA-------------
-------GSNKNATSVITANDLKGLDCSETVAIYLLKLGVTDK--FYSIHT--GVMLTENDFRKAIRSNKIE
YVVG-----------------------------SKDLNFIPQIGDIFVWRNGGGH------CGIVYDVDRQN
DTVTILEAIGDVGSADENFNINNGGEKKVGCTRTAVYRRSGKALAQHRGWVGYFRPIISGKKI---------
----------------------------
>AbPK1_8
--------------------------------MYIVKKGDT-----LTSIANKNGCTVEQIIKLNSIKNKN-
------------------------------------------------------------------------
---------------------LIFVG-QKLKLKN--------------TPTPTPTPTPTPTPTPTPTPIQNL
G----GKVGIAIVNKKGEPISNFKLKIQVGNNIYSNQLTN------------ARGVVTLPRQKS-GENIIIQ
GWSK---------------------TNNSYKLISNRFITENR----NLGIGISLDTIKFEAKTDNHKKPINN
NVNNNGQS-----------------------------------------------------------TTTNG
SNSQ--------NNPIARTSVEGCSNCTKITENELK----------------------------QIFTQANS
NDIKKIIDVYTNFSEKFGMNNCLSKAHFFAQVLEEVGKKIEVKDGESLNYHSDILFLSYTKIKRKEYIIEKG
KRVEKMTHGGPFSA-------FRGNRQLCDKYGR-----------------NDNHPADQVMIANIAYANRNG
NG-------DIQSGDGWKY-------------------RGRGIIQITGKDKYDKINKAIKDNYPNVGISIDA
NNINNIYEGTLASMAYWKSFGLSKLATQKQVDIRTQLEVVDSLIDI--------------------------
----------------------------------------------------------------------IN
RDTASRSDRK-------------------KNFEYITAKVFKLNECKNSIVEANLLSKSTP------------
-------------------------TPTPTPTL---------------------------------------
SNVLKEIK----------------------------------QLVDRNIPYSQTGARGA-------------
-------GSNKNATSVITANDLKGLDCSETVAIYLLKLGVTDK--FYSIHT--GVMLTENDFRKAIRSNKIE
YVVG-----------------------------SKDLNFIPQIGDIFVWRNGGGH------CGIVYDVDRQN
DTVTILEAIGDVGSADENFNINNGGEKKVGCTRTAVYRRSGKALAQHRGWVGYFRPIISGKKI---------
----------------------------
>15A34_6
-----------------------------APCSLVRYKPDK-----VTPQAGSRQDAID-------------
------------------------------------------------------------------------
```

---------------------RAWSL-EKQLIQT------------------------------TGTGTRDW
S---------------------------------------------------KAELDTILRTPS-GS-----
------------------------GKGHLSSVMSNLGYTGHH------------------------------
------------------------------------------------------------------------
------------INSVKNNGALGESWKGDPRNI-------------------------------VFLENPKH
PNSSPMPNAYNEH-----------------------------------------------------------
----FHSKQGHRGS----------------------------------------------------------
------------------------------------------------------------------------
------------------------------------------------------------------------
------------------------------------------------------------------------
------------------------------------------------------------------------
-----------------TTNVSRGRLIDRQAMI---------------------------------------
---------------------------------------------NQFNKGCSI------------------
------------------------------------------------------------------------
------------------------------------------------------------------------
------------------------------------------------------------------------
----------------------------
>15A5_6
-----------------------------APCSLVRYKPDK-----VTPQAGSRQDAID-------------
------------------------------------------------------------------------
---------------------RAWSL-EKQLIQT------------------------------TGTGTRDW
S---------------------------------------------------KAELDTILRTPS-GS-----
------------------------GKGHLSSVMSNLGYTGHH------------------------------
------------------------------------------------------------------------
------------INSVKNNGALGESWKGDPRNI-------------------------------VFLENPKH
PNSSPMPNAYNEH-----------------------------------------------------------
----FHSKQGHRGS----------------------------------------------------------
------------------------------------------------------------------------
------------------------------------------------------------------------
------------------------------------------------------------------------
------------------------------------------------------------------------
-----------------TTNVSRGRLIDRQAMI---------------------------------------
---------------------------------------------NQFNKGCSI------------------

------------------------------------------------------------------------
------------------------------------------------------------------------
------------------------------------------------------------------------
----------------------------
>1656-2_6
-----------------------------APCSLVRYKPDK-----VTPQAGSRQDAID-------------
------------------------------------------------------------------------
---------------------RAWSL-EKQLIQT------------------------------TGTGTRDW
S---------------------------------------------------KAELDTILRTPS-GS-----
------------------------GKGHLSSVMSNLGYTGHH------------------------------
------------------------------------------------------------------------
------------INSVKNNGALGESWKGDPRNI-------------------------------VFLENPKH
PNSSPMPNAYNEH-----------------------------------------------------------
----FHSKQGHRGS----------------------------------------------------------
------------------------------------------------------------------------
------------------------------------------------------------------------
------------------------------------------------------------------------
------------------------------------------------------------------------
-----------------TTNVSRGRLIDRQAMI---------------------------------------
---------------------------------------------NQFNKGCSI------------------
------------------------------------------------------------------------
------------------------------------------------------------------------
------------------------------------------------------------------------
----------------------------
>3027STDY5784958_6
-----------------------------APCSLVRYKPDK-----VTPQAGSRQDAID-------------
------------------------------------------------------------------------
---------------------RAWSL-EKQLIQT------------------------------TGTGTRDW
S---------------------------------------------------KAELDTILRTPS-GS-----
------------------------GKGHLSSVMSNLGYTGHH------------------------------
------------------------------------------------------------------------
------------INSVKNNGALGESWKGDPRNI-------------------------------VFLENPKH
PNSSPMPNAYNEH-----------------------------------------------------------

----FHSKQGHRGS----------------------------------------------------------
------------------------------------------------------------------------
------------------------------------------------------------------------
------------------------------------------------------------------------
------------------------------------------------------------------------
-----------------TTNVSRGRLIDRQAMI---------------------------------------
---------------------------------------------NQFNKGCSI------------------
------------------------------------------------------------------------
------------------------------------------------------------------------
------------------------------------------------------------------------
----------------------------
>AB07_6
-----------------------------APCSLVRYKPDK-----VTPQAGSRQDAID-------------
------------------------------------------------------------------------
---------------------RAWSL-EKQLIQT------------------------------TGTGTRDW
S---------------------------------------------------KAELDTILRTPS-GS-----
------------------------GKGHLSSVMSNLGYTGHH------------------------------
------------------------------------------------------------------------
------------INSVKNNGALGESWKGDPRNI-------------------------------VFLENPKH
PNSSPMPNAYNEH-----------------------------------------------------------
----FHSKQGHRGS----------------------------------------------------------
------------------------------------------------------------------------
------------------------------------------------------------------------
------------------------------------------------------------------------
------------------------------------------------------------------------
-----------------TTNVSRGRLIDRQAMI---------------------------------------
---------------------------------------------NQFNKGCSI------------------
------------------------------------------------------------------------
------------------------------------------------------------------------
------------------------------------------------------------------------
----------------------------
>AB34299_6
-----------------------------APCSLVRYKPDK-----VTPQAGSRQDAID-------------

------------------------------------------------------------------------
---------------------RAWSL-EKQLIQT------------------------------TGTGTRDW
S---------------------------------------------------KAELDTILRTPS-GS-----
------------------------GKGHLSSVMSNLGYTGHH------------------------------
------------------------------------------------------------------------
------------INSVKNNGALGESWKGDPRNI-------------------------------VFLENPKH
PNSSPMPNAYNEH-----------------------------------------------------------
----FHSKQGHRGS----------------------------------------------------------
------------------------------------------------------------------------
------------------------------------------------------------------------
------------------------------------------------------------------------
------------------------------------------------------------------------
-----------------TTNVSRGRLIDRQAMI---------------------------------------
---------------------------------------------NQFNKGCSI------------------
------------------------------------------------------------------------
------------------------------------------------------------------------
------------------------------------------------------------------------
----------------------------
>Ab4568_6
-----------------------------APCSLVRYKPDK-----VTPQAGSRQDAID-------------
------------------------------------------------------------------------
---------------------RAWSL-EKQLIQT------------------------------TGTGTRDW
S---------------------------------------------------KAELDTILRTPS-GS-----
------------------------GKGHLSSVMSNLGYTGHH------------------------------
------------------------------------------------------------------------
------------INSVKNNGALGESWKGDPRNI-------------------------------VFLENPKH
PNSSPMPNAYNEH-----------------------------------------------------------
----FHSKQGHRGS----------------------------------------------------------
------------------------------------------------------------------------
------------------------------------------------------------------------
------------------------------------------------------------------------
------------------------------------------------------------------------
-----------------TTNVSRGRLIDRQAMI---------------------------------------

---------------------------------------------NQFNKGCSI------------------
------------------------------------------------------------------------
------------------------------------------------------------------------
------------------------------------------------------------------------
----------------------------
>Ab4653_6
-----------------------------APCSLVRYKPDK-----VTPQAGSRQDAID-------------
------------------------------------------------------------------------
---------------------RAWSL-EKQLIQT------------------------------TGTGTRDW
S---------------------------------------------------KAELDTILRTPS-GS-----
------------------------GKGHLSSVMSNLGYTGHH------------------------------
------------------------------------------------------------------------
------------INSVKNNGALGESWKGDPRNI-------------------------------VFLENPKH
PNSSPMPNAYNEH-----------------------------------------------------------
----FHSKQGHRGS----------------------------------------------------------
------------------------------------------------------------------------
------------------------------------------------------------------------
------------------------------------------------------------------------
------------------------------------------------------------------------
-----------------TTNVSRGRLIDRQAMI---------------------------------------
---------------------------------------------NQFNKGCSI------------------
------------------------------------------------------------------------
------------------------------------------------------------------------
------------------------------------------------------------------------
----------------------------
>Ab4977_6
-----------------------------APCSLVRYKPDK-----VTPQAGSRQDAID-------------
------------------------------------------------------------------------
---------------------RAWSL-EKQLIQT------------------------------TGTGTRDW
S---------------------------------------------------KAELDTILRTPS-GS-----
------------------------GKGHLSSVMSNLGYTGHH------------------------------
------------------------------------------------------------------------
------------INSVKNNGALGESWKGDPRNI-------------------------------VFLENPKH

PNSSPMPNAYNEH-----------------------------------------------------------
----FHSKQGHRGS----------------------------------------------------------
------------------------------------------------------------------------
------------------------------------------------------------------------
------------------------------------------------------------------------
------------------------------------------------------------------------
-----------------TTNVSRGRLIDRQAMI---------------------------------------
---------------------------------------------NQFNKGCSI------------------
------------------------------------------------------------------------
------------------------------------------------------------------------
------------------------------------------------------------------------
----------------------------
>AbPK1_6
-----------------------------APCSLVRYKPDK-----VTPQAGSRQDAID-------------
------------------------------------------------------------------------
---------------------RAWSL-EKQLIQT------------------------------TGTGTRDW
S---------------------------------------------------KAELDTILRTPS-GS-----
------------------------GKGHLSSVMSNLGYTGHH------------------------------
------------------------------------------------------------------------
------------INSVKNNGALGESWKGDPRNI-------------------------------VFLENPKH
PNSSPMPNAYNEH-----------------------------------------------------------
----FHSKQGHRGS----------------------------------------------------------
------------------------------------------------------------------------
------------------------------------------------------------------------
------------------------------------------------------------------------
------------------------------------------------------------------------
-----------------TTNVSRGRLIDRQAMI---------------------------------------
---------------------------------------------NQFNKGCSI------------------
------------------------------------------------------------------------
------------------------------------------------------------------------
------------------------------------------------------------------------
----------------------------
>AC29_6

```
-----------------------------APCSLVRYKPDK-----VTPQAGSRQDAID-------------
------------------------------------------------------------------------
---------------------RAWSL-EKQLIQT------------------------------TGTGTRDW
S---------------------------------------------------KAELDTILRTPS-GS-----
------------------------GKGHLSSVMSNLGYTGHH------------------------------
------------------------------------------------------------------------
------------INSVKNNGALGESWKGDPRNI-------------------------------VFLENPKH
PNSSPMPNAYNEH-----------------------------------------------------------
----FHSKQGHRGS----------------------------------------------------------
------------------------------------------------------------------------
------------------------------------------------------------------------
------------------------------------------------------------------------
------------------------------------------------------------------------
-----------------TTNVSRGRLIDRQAMI---------------------------------------
---------------------------------------------NQFNKGCSI------------------
------------------------------------------------------------------------
------------------------------------------------------------------------
------------------------------------------------------------------------
----------------------------
>AC30_6
-----------------------------APCSLVRYKPDK-----VTPQAGSRQDAID-------------
------------------------------------------------------------------------
---------------------RAWSL-EKQLIQT------------------------------TGTGTRDW
S---------------------------------------------------KAELDTILRTPS-GS-----
------------------------GKGHLSSVMSNLGYTGHH------------------------------
------------------------------------------------------------------------
------------INSVKNNGALGESWKGDPRNI-------------------------------VFLENPKH
PNSSPMPNAYNEH-----------------------------------------------------------
----FHSKQGHRGS----------------------------------------------------------
------------------------------------------------------------------------
------------------------------------------------------------------------
------------------------------------------------------------------------
------------------------------------------------------------------------
```

-----------------TTNVSRGRLIDRQAMI---------------------------------------
---------------------------------------------NQFNKGCSI------------------
------------------------------------------------------------------------
------------------------------------------------------------------------
------------------------------------------------------------------------
----------------------------
>ACICU_6
-----------------------------APCSLVRYKPDK-----VTPQAGSRQDAID-------------
------------------------------------------------------------------------
---------------------RAWSL-EKQLIQT------------------------------TGTGTRDW
S---------------------------------------------------KAELDTILRTPS-GS-----
------------------------GKGHLSSVMSNLGYTGHH------------------------------
------------------------------------------------------------------------
------------INSVKNNGALGESWKGDPRNI-------------------------------VFLENPKH
PNSSPMPNAYNEH-----------------------------------------------------------
----FHSKQGHRGS----------------------------------------------------------
------------------------------------------------------------------------
------------------------------------------------------------------------
------------------------------------------------------------------------
------------------------------------------------------------------------
-----------------TTNVSRGRLIDRQAMI---------------------------------------
---------------------------------------------NQFNKGCSI------------------
------------------------------------------------------------------------
------------------------------------------------------------------------
------------------------------------------------------------------------
----------------------------
>AF-673_6
-----------------------------APCSLVRYKPDK-----VTPQAGSRQDAID-------------
------------------------------------------------------------------------
---------------------RAWSL-EKQLIQT------------------------------TGTGTRDW
S---------------------------------------------------KAELDTILRTPS-GS-----
------------------------GKGHLSSVMSNLGYTGHH------------------------------
------------------------------------------------------------------------

```
------------INSVKNNGALGESWKGDPRNI-------------------------------VFLENPKH
PNSSPMPNAYNEH-----------------------------------------------------------
----FHSKQGHRGS----------------------------------------------------------
------------------------------------------------------------------------
------------------------------------------------------------------------
------------------------------------------------------------------------
------------------------------------------------------------------------
-----------------TTNVSRGRLIDRQAMI---------------------------------------
---------------------------------------------NQFNKGCSI------------------
------------------------------------------------------------------------
------------------------------------------------------------------------
------------------------------------------------------------------------
----------------------------
>AR_0056_6
-----------------------------APCSLVRYKPDK-----VTPQAGSRQDAID-------------
------------------------------------------------------------------------
---------------------RAWSL-EKQLIQT------------------------------TGTGTRDW
S---------------------------------------------------KAELDTILRTPS-GS-----
------------------------GKGHLSSVMSNLGYTGHH------------------------------
------------------------------------------------------------------------
------------INSVKNNGALGESWKGDPRNI-------------------------------VFLENPKH
PNSSPMPNAYNEH-----------------------------------------------------------
----FHSKQGHRGS----------------------------------------------------------
------------------------------------------------------------------------
------------------------------------------------------------------------
------------------------------------------------------------------------
------------------------------------------------------------------------
-----------------TTNVSRGRLIDRQAMI---------------------------------------
---------------------------------------------NQFNKGCSI------------------
------------------------------------------------------------------------
------------------------------------------------------------------------
------------------------------------------------------------------------
----------------------------
```

```
>AYP-A2_6
-----------------------------APCSLVRYKPDK-----VTPQAGSRQDAID-------------
------------------------------------------------------------------------
---------------------RAWSL-EKQLIQT------------------------------TGTGTRDW
S---------------------------------------------------KAELDTILRTPS-GS-----
------------------------GKGHLSSVMSNLGYTGHH------------------------------
------------------------------------------------------------------------
------------INSVKNNGALGESWKGDPRNI-------------------------------VFLENPKH
PNSSPMPNAYNEH-----------------------------------------------------------
----FHSKQGHRGS----------------------------------------------------------
------------------------------------------------------------------------
------------------------------------------------------------------------
------------------------------------------------------------------------
------------------------------------------------------------------------
-----------------TTNVSRGRLIDRQAMI---------------------------------------
---------------------------------------------NQFNKGCSI------------------
------------------------------------------------------------------------
------------------------------------------------------------------------
------------------------------------------------------------------------
----------------------------
>BJAB07104_6
-----------------------------APCSLVRYKPDK-----VTPQAGSRQDAID-------------
------------------------------------------------------------------------
---------------------RAWSL-EKQLIQT------------------------------TGTGTRDW
S---------------------------------------------------KAELDTILRTPS-GS-----
------------------------GKGHLSSVMSNLGYTGHH------------------------------
------------------------------------------------------------------------
------------INSVKNNGALGESWKGDPRNI-------------------------------VFLENPKH
PNSSPMPNAYNEH-----------------------------------------------------------
----FHSKQGHRGS----------------------------------------------------------
------------------------------------------------------------------------
------------------------------------------------------------------------
------------------------------------------------------------------------
```

```
------------------------------------------------------------------------
-----------------TTNVSRGRLIDRQAMI---------------------------------------
---------------------------------------------NQFNKGCSI------------------
------------------------------------------------------------------------
------------------------------------------------------------------------
------------------------------------------------------------------------
----------------------------
>BJAB0868_6
-----------------------------APCSLVRYKPDK-----VTPQAGSRQDAID-------------
------------------------------------------------------------------------
---------------------RAWSL-EKQLIQT------------------------------TGTGTRDW
S---------------------------------------------------KAELDTILRTPS-GS-----
------------------------GKGHLSSVMSNLGYTGHH------------------------------
------------------------------------------------------------------------
------------INSVKNNGALGESWKGDPRNI-------------------------------VFLENPKH
PNSSPMPNAYNEH-----------------------------------------------------------
----FHSKQGHRGS----------------------------------------------------------
------------------------------------------------------------------------
------------------------------------------------------------------------
------------------------------------------------------------------------
------------------------------------------------------------------------
-----------------TTNVSRGRLIDRQAMI---------------------------------------
---------------------------------------------NQFNKGCSI------------------
------------------------------------------------------------------------
------------------------------------------------------------------------
------------------------------------------------------------------------
----------------------------
>CBA7_6
-----------------------------APCSLVRYKPDK-----VTPQAGSRQDAID-------------
------------------------------------------------------------------------
---------------------RAWSL-EKQLIQT------------------------------TGTGTRDW
S---------------------------------------------------KAELDTILRTPS-GS-----
------------------------GKGHLSSVMSNLGYTGHH------------------------------
```

```
------------------------------------------------------------------------
------------INSVKNNGALGESWKGDPRNI-------------------------------VFLENPKH
PNSSPMPNAYNEH-----------------------------------------------------------
----FHSKQGHRGS----------------------------------------------------------
------------------------------------------------------------------------
------------------------------------------------------------------------
------------------------------------------------------------------------
------------------------------------------------------------------------
-----------------TTNVSRGRLIDRQAMI---------------------------------------
---------------------------------------------NQFNKGCSI------------------
------------------------------------------------------------------------
------------------------------------------------------------------------
------------------------------------------------------------------------
----------------------------
>CMC-CR-MDR-Ab4_6
-----------------------------APCSLVRYKPDK-----VTPQAGSRQDAID-------------
------------------------------------------------------------------------
---------------------RAWSL-EKQLIQT------------------------------TGTGTRDW
S---------------------------------------------------KAELDTILRTPS-GS-----
------------------------GKGHLSSVMSNLGYTGHH------------------------------
------------------------------------------------------------------------
------------INSVKNNGALGESWKGDPRNI-------------------------------VFLENPKH
PNSSPMPNAYNEH-----------------------------------------------------------
----FHSKQGHRGS----------------------------------------------------------
------------------------------------------------------------------------
------------------------------------------------------------------------
------------------------------------------------------------------------
------------------------------------------------------------------------
-----------------TTNVSRGRLIDRQAMI---------------------------------------
---------------------------------------------NQFNKGCSI------------------
------------------------------------------------------------------------
------------------------------------------------------------------------
------------------------------------------------------------------------
```

----------------------------
>CMC-CR-MDR-Ab66_6
-----------------------------APCSLVRYKPDK-----VTPQAGSRQDAID-------------
------------------------------------------------------------------------
---------------------RAWSL-EKQLIQT------------------------------TGTGTRDW
S---------------------------------------------------KAELDTILRTPS-GS-----
------------------------GKGHLSSVMSNLGYTGHH------------------------------
------------------------------------------------------------------------
------------INSVKNNGALGESWKGDPRNI-------------------------------VFLENPKH
PNSSPMPNAYNEH-----------------------------------------------------------
----FHSKQGHRGS----------------------------------------------------------
------------------------------------------------------------------------
------------------------------------------------------------------------
------------------------------------------------------------------------
------------------------------------------------------------------------
-----------------TTNVSRGRLIDRQAMI---------------------------------------
---------------------------------------------NQFNKGCSI------------------
------------------------------------------------------------------------
------------------------------------------------------------------------
------------------------------------------------------------------------
----------------------------
>CMC-MDR-Ab59_6
-----------------------------APCSLVRYKPDK-----VTPQAGSRQDAID-------------
------------------------------------------------------------------------
---------------------RAWSL-EKQLIQT------------------------------TGTGTRDW
S---------------------------------------------------KAELDTILRTPS-GS-----
------------------------GKGHLSSVMSNLGYTGHH------------------------------
------------------------------------------------------------------------
------------INSVKNNGALGESWKGDPRNI-------------------------------VFLENPKH
PNSSPMPNAYNEH-----------------------------------------------------------
----FHSKQGHRGS----------------------------------------------------------
------------------------------------------------------------------------
------------------------------------------------------------------------

------------------------------------------------------------------------
------------------------------------------------------------------------
-----------------TTNVSRGRLIDRQAMI---------------------------------------
---------------------------------------------NQFNKGCSI------------------
------------------------------------------------------------------------
------------------------------------------------------------------------
------------------------------------------------------------------------
----------------------------
>DU202_6
-----------------------------APCSLVRYKPDK-----VTPQAGSRQDAID-------------
------------------------------------------------------------------------
---------------------RAWSL-EKQLIQT------------------------------TGTGTRDW
S---------------------------------------------------KAELDTILRTPS-GS-----
------------------------GKGHLSSVMSNLGYTGHH------------------------------
------------------------------------------------------------------------
------------INSVKNNGALGESWKGDPRNI-------------------------------VFLENPKH
PNSSPMPNAYNEH-----------------------------------------------------------
----FHSKQGHRGS----------------------------------------------------------
------------------------------------------------------------------------
------------------------------------------------------------------------
------------------------------------------------------------------------
------------------------------------------------------------------------
-----------------TTNVSRGRLIDRQAMI---------------------------------------
---------------------------------------------NQFNKGCSI------------------
------------------------------------------------------------------------
------------------------------------------------------------------------
------------------------------------------------------------------------
----------------------------
>HRAB-85_6
-----------------------------APCSLVRYKPDK-----VTPQAGSRQDAID-------------
------------------------------------------------------------------------
---------------------RAWSL-EKQLIQT------------------------------TGTGTRDW
S---------------------------------------------------KAELDTILRTPS-GS-----

```
------------------------GKGHLSSVMSNLGYTGHH------------------------------
------------------------------------------------------------------------
------------INSVKNNGALGESWKGDPRNI-------------------------------VFLENPKH
PNSSPMPNAYNEH-----------------------------------------------------------
----FHSKQGHRGS----------------------------------------------------------
------------------------------------------------------------------------
------------------------------------------------------------------------
------------------------------------------------------------------------
------------------------------------------------------------------------
-----------------TTNVSRGRLIDRQAMI---------------------------------------
---------------------------------------------NQFNKGCSI------------------
------------------------------------------------------------------------
------------------------------------------------------------------------
------------------------------------------------------------------------
----------------------------
>JBA13_6
-----------------------------APCSLVRYKPDK-----VTPQAGSRQDAID-------------
------------------------------------------------------------------------
---------------------RAWSL-EKQLIQT------------------------------TGTGTRDW
S---------------------------------------------------KAELDTILRTPS-GS-----
------------------------GKGHLSSVMSNLGYTGHH------------------------------
------------------------------------------------------------------------
------------INSVKNNGALGESWKGDPRNI-------------------------------VFLENPKH
PNSSPMPNAYNEH-----------------------------------------------------------
----FHSKQGHRGS----------------------------------------------------------
------------------------------------------------------------------------
------------------------------------------------------------------------
------------------------------------------------------------------------
------------------------------------------------------------------------
-----------------TTNVSRGRLIDRQAMI---------------------------------------
---------------------------------------------NQFNKGCSI------------------
------------------------------------------------------------------------
------------------------------------------------------------------------
```

```
------------------------------------------------------------------------
----------------------------
>KAB01_6
-----------------------------APCSLVRYKPDK-----VTPQAGSRQDAID-------------
------------------------------------------------------------------------
---------------------RAWSL-EKQLIQT------------------------------TGTGTRDW
S---------------------------------------------------KAELDTILRTPS-GS-----
------------------------GKGHLSSVMSNLGYTGHH------------------------------
------------------------------------------------------------------------
------------INSVKNNGALGESWKGDPRNI-------------------------------VFLENPKH
PNSSPMPNAYNEH-----------------------------------------------------------
----FHSKQGHRGS----------------------------------------------------------
------------------------------------------------------------------------
------------------------------------------------------------------------
------------------------------------------------------------------------
------------------------------------------------------------------------
-----------------TTNVSRGRLIDRQAMI---------------------------------------
---------------------------------------------NQFNKGCSI------------------
------------------------------------------------------------------------
------------------------------------------------------------------------
------------------------------------------------------------------------
----------------------------
>KAB02_6
-----------------------------APCSLVRYKPDK-----VTPQAGSRQDAID-------------
------------------------------------------------------------------------
---------------------RAWSL-EKQLIQT------------------------------TGTGTRDW
S---------------------------------------------------KAELDTILRTPS-GS-----
------------------------GKGHLSSVMSNLGYTGHH------------------------------
------------------------------------------------------------------------
------------INSVKNNGALGESWKGDPRNI-------------------------------VFLENPKH
PNSSPMPNAYNEH-----------------------------------------------------------
----FHSKQGHRGS----------------------------------------------------------
------------------------------------------------------------------------
```

------------------------------------------------------------------------
------------------------------------------------------------------------
------------------------------------------------------------------------
-----------------TTNVSRGRLIDRQAMI---------------------------------------
---------------------------------------------NQFNKGCSI------------------
------------------------------------------------------------------------
------------------------------------------------------------------------
------------------------------------------------------------------------
----------------------------
>KAB03_6
-----------------------------APCSLVRYKPDK-----VTPQAGSRQDAID-------------
------------------------------------------------------------------------
---------------------RAWSL-EKQLIQT------------------------------TGTGTRDW
S---------------------------------------------------KAELDTILRTPS-GS-----
------------------------GKGHLSSVMSNLGYTGHH------------------------------
------------------------------------------------------------------------
------------INSVKNNGALGESWKGDPRNI-------------------------------VFLENPKH
PNSSPMPNAYNEH-----------------------------------------------------------
----FHSKQGHRGS----------------------------------------------------------
------------------------------------------------------------------------
------------------------------------------------------------------------
------------------------------------------------------------------------
------------------------------------------------------------------------
-----------------TTNVSRGRLIDRQAMI---------------------------------------
---------------------------------------------NQFNKGCSI------------------
------------------------------------------------------------------------
------------------------------------------------------------------------
------------------------------------------------------------------------
----------------------------
>KAB04_6
-----------------------------APCSLVRYKPDK-----VTPQAGSRQDAID-------------
------------------------------------------------------------------------
---------------------RAWSL-EKQLIQT------------------------------TGTGTRDW

S---------------------------------------------------KAELDTILRTPS-GS-----
------------------------GKGHLSSVMSNLGYTGHH------------------------------
------------------------------------------------------------------------
------------INSVKNNGALGESWKGDPRNI-------------------------------VFLENPKH
PNSSPMPNAYNEH-----------------------------------------------------------
----FHSKQGHRGS----------------------------------------------------------
------------------------------------------------------------------------
------------------------------------------------------------------------
------------------------------------------------------------------------
------------------------------------------------------------------------
-----------------TTNVSRGRLIDRQAMI---------------------------------------
---------------------------------------------NQFNKGCSI------------------
------------------------------------------------------------------------
------------------------------------------------------------------------
------------------------------------------------------------------------
----------------------------
>KAB05_6
-----------------------------APCSLVRYKPDK-----VTPQAGSRQDAID-------------
------------------------------------------------------------------------
---------------------RAWSL-EKQLIQT------------------------------TGTGTRDW
S---------------------------------------------------KAELDTILRTPS-GS-----
------------------------GKGHLSSVMSNLGYTGHH------------------------------
------------------------------------------------------------------------
------------INSVKNNGALGESWKGDPRNI-------------------------------VFLENPKH
PNSSPMPNAYNEH-----------------------------------------------------------
----FHSKQGHRGS----------------------------------------------------------
------------------------------------------------------------------------
------------------------------------------------------------------------
------------------------------------------------------------------------
------------------------------------------------------------------------
-----------------TTNVSRGRLIDRQAMI---------------------------------------
---------------------------------------------NQFNKGCSI------------------
------------------------------------------------------------------------

```
------------------------------------------------------------------------
------------------------------------------------------------------------
----------------------------
>KAB06_6
-----------------------------APCSLVRYKPDK-----VTPQAGSRQDAID-------------
------------------------------------------------------------------------
---------------------RAWSL-EKQLIQT------------------------------TGTGTRDW
S---------------------------------------------------KAELDTILRTPS-GS-----
------------------------GKGHLSSVMSNLGYTGHH------------------------------
------------------------------------------------------------------------
------------INSVKNNGALGESWKGDPRNI-------------------------------VFLENPKH
PNSSPMPNAYNEH-----------------------------------------------------------
----FHSKQGHRGS----------------------------------------------------------
------------------------------------------------------------------------
------------------------------------------------------------------------
------------------------------------------------------------------------
------------------------------------------------------------------------
-----------------TTNVSRGRLIDRQAMI---------------------------------------
---------------------------------------------NQFNKGCSI------------------
------------------------------------------------------------------------
------------------------------------------------------------------------
------------------------------------------------------------------------
----------------------------
>KAB07_6
-----------------------------APCSLVRYKPDK-----VTPQAGSRQDAID-------------
------------------------------------------------------------------------
---------------------RAWSL-EKQLIQT------------------------------TGTGTRDW
S---------------------------------------------------KAELDTILRTPS-GS-----
------------------------GKGHLSSVMSNLGYTGHH------------------------------
------------------------------------------------------------------------
------------INSVKNNGALGESWKGDPRNI-------------------------------VFLENPKH
PNSSPMPNAYNEH-----------------------------------------------------------
----FHSKQGHRGS----------------------------------------------------------
```

```
------------------------------------------------------------------------
------------------------------------------------------------------------
------------------------------------------------------------------------
------------------------------------------------------------------------
-----------------TTNVSRGRLIDRQAMI---------------------------------------
---------------------------------------------NQFNKGCSI------------------
------------------------------------------------------------------------
------------------------------------------------------------------------
------------------------------------------------------------------------
----------------------------
>KAB08_6
-----------------------------APCSLVRYKPDK-----VTPQAGSRQDAID-------------
------------------------------------------------------------------------
---------------------RAWSL-EKQLIQT------------------------------TGTGTRDW
S---------------------------------------------------KAELDTILRTPS-GS-----
------------------------GKGHLSSVMSNLGYTGHH------------------------------
------------------------------------------------------------------------
------------INSVKNNGALGESWKGDPRNI-------------------------------VFLENPKH
PNSSPMPNAYNEH-----------------------------------------------------------
----FHSKQGHRGS----------------------------------------------------------
------------------------------------------------------------------------
------------------------------------------------------------------------
------------------------------------------------------------------------
------------------------------------------------------------------------
-----------------TTNVSRGRLIDRQAMI---------------------------------------
---------------------------------------------NQFNKGCSI------------------
------------------------------------------------------------------------
------------------------------------------------------------------------
------------------------------------------------------------------------
----------------------------
>KBN10P02143_6
-----------------------------APCSLVRYKPDK-----VTPQAGSRQDAID-------------
------------------------------------------------------------------------
```

---------------------RAWSL-EKQLIQT------------------------------TGTGTRDW
S---------------------------------------------------KAELDTILRTPS-GS-----
------------------------GKGHLSSVMSNLGYTGHH------------------------------
------------------------------------------------------------------------
------------INSVKNNGALGESWKGDPRNI-------------------------------VFLENPKH
PNSSPMPNAYNEH-----------------------------------------------------------
----FHSKQGHRGS----------------------------------------------------------
------------------------------------------------------------------------
------------------------------------------------------------------------
------------------------------------------------------------------------
------------------------------------------------------------------------
-----------------TTNVSRGRLIDRQAMI---------------------------------------
---------------------------------------------NQFNKGCSI------------------
------------------------------------------------------------------------
------------------------------------------------------------------------
------------------------------------------------------------------------
----------------------------
>MDR-TJ_6
-----------------------------APCSLVRYKPDK-----VTPQAGSRQDAID-------------
------------------------------------------------------------------------
---------------------RAWSL-EKQLIQT------------------------------TGTGTRDW
S---------------------------------------------------KAELDTILRTPS-GS-----
------------------------GKGHLSSVMSNLGYTGHH------------------------------
------------------------------------------------------------------------
------------INSVKNNGALGESWKGDPRNI-------------------------------VFLENPKH
PNSSPMPNAYNEH-----------------------------------------------------------
----FHSKQGHRGS----------------------------------------------------------
------------------------------------------------------------------------
------------------------------------------------------------------------
------------------------------------------------------------------------
------------------------------------------------------------------------
-----------------TTNVSRGRLIDRQAMI---------------------------------------
---------------------------------------------NQFNKGCSI------------------

------------------------------------------------------------------------
------------------------------------------------------------------------
------------------------------------------------------------------------
----------------------------
>MDR-ZJ06_6
-----------------------------APCSLVRYKPDK-----VTPQAGSRQDAID-------------
------------------------------------------------------------------------
---------------------RAWSL-EKQLIQT------------------------------TGTGTRDW
S---------------------------------------------------KAELDTILRTPS-GS-----
------------------------GKGHLSSVMSNLGYTGHH------------------------------
------------------------------------------------------------------------
------------INSVKNNGALGESWKGDPRNI-------------------------------VFLENPKH
PNSSPMPNAYNEH-----------------------------------------------------------
----FHSKQGHRGS----------------------------------------------------------
------------------------------------------------------------------------
------------------------------------------------------------------------
------------------------------------------------------------------------
------------------------------------------------------------------------
-----------------TTNVSRGRLIDRQAMI---------------------------------------
---------------------------------------------NQFNKGCSI------------------
------------------------------------------------------------------------
------------------------------------------------------------------------
------------------------------------------------------------------------
----------------------------
>NCGM237_6
-----------------------------APCSLVRYKPDK-----VTPQAGSRQDAID-------------
------------------------------------------------------------------------
---------------------RAWSL-EKQLIQT------------------------------TGTGTRDW
S---------------------------------------------------KAELDTILRTPS-GS-----
------------------------GKGHLSSVMSNLGYTGHH------------------------------
------------------------------------------------------------------------
------------INSVKNNGALGESWKGDPRNI-------------------------------VFLENPKH
PNSSPMPNAYNEH-----------------------------------------------------------

----FHSKQGHRGS----------------------------------------------------------
------------------------------------------------------------------------
------------------------------------------------------------------------
------------------------------------------------------------------------
------------------------------------------------------------------------
-----------------TTNVSRGRLIDRQAMI---------------------------------------
---------------------------------------------NQFNKGCSI------------------
------------------------------------------------------------------------
------------------------------------------------------------------------
------------------------------------------------------------------------
----------------------------
>SAA14_6
-----------------------------APCSLVRYKPDK-----VTPQAGSRQDAID-------------
------------------------------------------------------------------------
---------------------RAWSL-EKQLIQT------------------------------TGTGTRDW
S---------------------------------------------------KAELDTILRTPS-GS-----
------------------------GKGHLSSVMSNLGYTGHH------------------------------
------------------------------------------------------------------------
------------INSVKNNGALGESWKGDPRNI-------------------------------VFLENPKH
PNSSPMPNAYNEH-----------------------------------------------------------
----FHSKQGHRGS----------------------------------------------------------
------------------------------------------------------------------------
------------------------------------------------------------------------
------------------------------------------------------------------------
------------------------------------------------------------------------
-----------------TTNVSRGRLIDRQAMI---------------------------------------
---------------------------------------------NQFNKGCSI------------------
------------------------------------------------------------------------
------------------------------------------------------------------------
------------------------------------------------------------------------
----------------------------
>SMC_Paed_Ab_BL01_6
-----------------------------APCSLVRYKPDK-----VTPQAGSRQDAID-------------

```
------------------------------------------------------------------------
---------------------RAWSL-EKQLIQT------------------------------TGTGTRDW
S---------------------------------------------------KAELDTILRTPS-GS-----
------------------------GKGHLSSVMSNLGYTGHH------------------------------
------------------------------------------------------------------------
------------INSVKNNGALGESWKGDPRNI-------------------------------VFLENPKH
PNSSPMPNAYNEH-----------------------------------------------------------
----FHSKQGHRGS----------------------------------------------------------
------------------------------------------------------------------------
------------------------------------------------------------------------
------------------------------------------------------------------------
------------------------------------------------------------------------
-----------------TTNVSRGRLIDRQAMI---------------------------------------
---------------------------------------------NQFNKGCSI------------------
------------------------------------------------------------------------
------------------------------------------------------------------------
------------------------------------------------------------------------
----------------------------
>SSA12_6
-----------------------------APCSLVRYKPDK-----VTPQAGSRQDAID-------------
------------------------------------------------------------------------
---------------------RAWSL-EKQLIQT------------------------------TGTGTRDW
S---------------------------------------------------KAELDTILRTPS-GS-----
------------------------GKGHLSSVMSNLGYTGHH------------------------------
------------------------------------------------------------------------
------------INSVKNNGALGESWKGDPRNI-------------------------------VFLENPKH
PNSSPMPNAYNEH-----------------------------------------------------------
----FHSKQGHRGS----------------------------------------------------------
------------------------------------------------------------------------
------------------------------------------------------------------------
------------------------------------------------------------------------
------------------------------------------------------------------------
-----------------TTNVSRGRLIDRQAMI---------------------------------------
```

---------------------------------------------NQFNKGCSI------------------
------------------------------------------------------------------------
------------------------------------------------------------------------
------------------------------------------------------------------------
----------------------------
>SSA6_6
-----------------------------APCSLVRYKPDK-----VTPQAGSRQDAID-------------
------------------------------------------------------------------------
---------------------RAWSL-EKQLIQT------------------------------TGTGTRDW
S---------------------------------------------------KAELDTILRTPS-GS-----
------------------------GKGHLSSVMSNLGYTGHH------------------------------
------------------------------------------------------------------------
------------INSVKNNGALGESWKGDPRNI-------------------------------VFLENPKH
PNSSPMPNAYNEH-----------------------------------------------------------
----FHSKQGHRGS----------------------------------------------------------
------------------------------------------------------------------------
------------------------------------------------------------------------
------------------------------------------------------------------------
------------------------------------------------------------------------
-----------------TTNVSRGRLIDRQAMI---------------------------------------
---------------------------------------------NQFNKGCSI------------------
------------------------------------------------------------------------
------------------------------------------------------------------------
------------------------------------------------------------------------
----------------------------
>TCDC-AB0715_6
-----------------------------APCSLVRYKPDK-----VTPQAGSRQDAID-------------
------------------------------------------------------------------------
---------------------RAWSL-EKQLIQT------------------------------TGTGTRDW
S---------------------------------------------------KAELDTILRTPS-GS-----
------------------------GKGHLSSVMSNLGYTGHH------------------------------
------------------------------------------------------------------------
------------INSVKNNGALGESWKGDPRNI-------------------------------VFLENPKH

```
PNSSPMPNAYNEH-----------------------------------------------------------
----FHSKQGHRGS----------------------------------------------------------
------------------------------------------------------------------------
------------------------------------------------------------------------
------------------------------------------------------------------------
------------------------------------------------------------------------
-----------------TTNVSRGRLIDRQAMI---------------------------------------
---------------------------------------------NQFNKGCSI------------------
------------------------------------------------------------------------
------------------------------------------------------------------------
------------------------------------------------------------------------
----------------------------
>TYTH-1_6
-----------------------------APCSLVRYKPDK-----VTPQAGSRQDAID-------------
------------------------------------------------------------------------
---------------------RAWSL-EKQLIQT------------------------------TGTGTRDW
S---------------------------------------------------KAELDTILRTPS-GS-----
------------------------GKGHLSSVMSNLGYTGHH------------------------------
------------------------------------------------------------------------
------------INSVKNNGALGESWKGDPRNI-------------------------------VFLENPKH
PNSSPMPNAYNEH-----------------------------------------------------------
----FHSKQGHRGS----------------------------------------------------------
------------------------------------------------------------------------
------------------------------------------------------------------------
------------------------------------------------------------------------
------------------------------------------------------------------------
-----------------TTNVSRGRLIDRQAMI---------------------------------------
---------------------------------------------NQFNKGCSI------------------
------------------------------------------------------------------------
------------------------------------------------------------------------
------------------------------------------------------------------------
----------------------------
>USA2_6
```

-----------------------------APCSLVRYKPDK-----VTPQAGSRQDAID-------------
------------------------------------------------------------------------
---------------------RAWSL-EKQLIQT------------------------------TGTGTRDW
S---------------------------------------------------KAELDTILRTPS-GS-----
------------------------GKGHLSSVMSNLGYTGHH------------------------------
------------------------------------------------------------------------
------------INSVKNNGALGESWKGDPRNI-------------------------------VFLENPKH
PNSSPMPNAYNEH-----------------------------------------------------------
----FHSKQGHRGS----------------------------------------------------------
------------------------------------------------------------------------
------------------------------------------------------------------------
------------------------------------------------------------------------
------------------------------------------------------------------------
-----------------TTNVSRGRLIDRQAMI---------------------------------------
---------------------------------------------NQFNKGCSI------------------
------------------------------------------------------------------------
------------------------------------------------------------------------
------------------------------------------------------------------------
----------------------------
>WCHAB005133_6
-----------------------------APCSLVRYKPDK-----VTPQAGSRQDAID-------------
------------------------------------------------------------------------
---------------------RAWSL-EKQLIQT------------------------------TGTGTRDW
S---------------------------------------------------KAELDTILRTPS-GS-----
------------------------GKGHLSSVMSNLGYTGHH------------------------------
------------------------------------------------------------------------
------------INSVKNNGALGESWKGDPRNI-------------------------------VFLENPKH
PNSSPMPNAYNEH-----------------------------------------------------------
----FHSKQGHRGS----------------------------------------------------------
------------------------------------------------------------------------
------------------------------------------------------------------------
------------------------------------------------------------------------
------------------------------------------------------------------------

-----------------TTNVSRGRLIDRQAMI---------------------------------------
---------------------------------------------NQFNKGCSI------------------
------------------------------------------------------------------------
------------------------------------------------------------------------
------------------------------------------------------------------------
----------------------------
>XDR-BJ83_6
-----------------------------APCSLVRYKPDK-----VTPQAGSRQDAID-------------
------------------------------------------------------------------------
---------------------RAWSL-EKQLIQT------------------------------TGTGTRDW
S---------------------------------------------------KAELDTILRTPS-GS-----
------------------------GKGHLSSVMSNLGYTGHH------------------------------
------------------------------------------------------------------------
------------INSVKNNGALGESWKGDPRNI-------------------------------VFLENPKH
PNSSPMPNAYNEH-----------------------------------------------------------
----FHSKQGHRGS----------------------------------------------------------
------------------------------------------------------------------------
------------------------------------------------------------------------
------------------------------------------------------------------------
------------------------------------------------------------------------
-----------------TTNVSRGRLIDRQAMI---------------------------------------
---------------------------------------------NQFNKGCSI------------------
------------------------------------------------------------------------
------------------------------------------------------------------------
------------------------------------------------------------------------
----------------------------
>XH386_6
-----------------------------APCSLVRYKPDK-----VTPQAGSRQDAID-------------
------------------------------------------------------------------------
---------------------RAWSL-EKQLIQT------------------------------TGTGTRDW
S---------------------------------------------------KAELDTILRTPS-GS-----
------------------------GKGHLSSVMSNLGYTGHH------------------------------
------------------------------------------------------------------------

------------INSVKNNGALGESWKGDPRNI-------------------------------VFLENPKH
PNSSPMPNAYNEH-----------------------------------------------------------
----FHSKQGHRGS----------------------------------------------------------
------------------------------------------------------------------------
------------------------------------------------------------------------
------------------------------------------------------------------------
------------------------------------------------------------------------
-----------------TTNVSRGRLIDRQAMI---------------------------------------
---------------------------------------------NQFNKGCSI------------------
------------------------------------------------------------------------
------------------------------------------------------------------------
------------------------------------------------------------------------
----------------------------
>XH856_6
-----------------------------APCSLVRYKPDK-----VTPQAGSRQDAID-------------
------------------------------------------------------------------------
---------------------RAWSL-EKQLIQT------------------------------TGTGTRDW
S---------------------------------------------------KAELDTILRTPS-GS-----
------------------------GKGHLSSVMSNLGYTGHH------------------------------
------------------------------------------------------------------------
------------INSVKNNGALGESWKGDPRNI-------------------------------VFLENPKH
PNSSPMPNAYNEH-----------------------------------------------------------
----FHSKQGHRGS----------------------------------------------------------
------------------------------------------------------------------------
------------------------------------------------------------------------
------------------------------------------------------------------------
------------------------------------------------------------------------
-----------------TTNVSRGRLIDRQAMI---------------------------------------
---------------------------------------------NQFNKGCSI------------------
------------------------------------------------------------------------
------------------------------------------------------------------------
------------------------------------------------------------------------
----------------------------

>XH857_6
-----------------------------APCSLVRYKPDK-----VTPQAGSRQDAID-------------
------------------------------------------------------------------------
---------------------RAWSL-EKQLIQT------------------------------TGTGTRDW
S---------------------------------------------------KAELDTILRTPS-GS-----
------------------------GKGHLSSVMSNLGYTGHH------------------------------
------------------------------------------------------------------------
------------INSVKNNGALGESWKGDPRNI-------------------------------VFLENPKH
PNSSPMPNAYNEH-----------------------------------------------------------
----FHSKQGHRGS----------------------------------------------------------
------------------------------------------------------------------------
------------------------------------------------------------------------
------------------------------------------------------------------------
------------------------------------------------------------------------
-----------------TTNVSRGRLIDRQAMI---------------------------------------
---------------------------------------------NQFNKGCSI------------------
------------------------------------------------------------------------
------------------------------------------------------------------------
------------------------------------------------------------------------
----------------------------
>XH859_6
-----------------------------APCSLVRYKPDK-----VTPQAGSRQDAID-------------
------------------------------------------------------------------------
---------------------RAWSL-EKQLIQT------------------------------TGTGTRDW
S---------------------------------------------------KAELDTILRTPS-GS-----
------------------------GKGHLSSVMSNLGYTGHH------------------------------
------------------------------------------------------------------------
------------INSVKNNGALGESWKGDPRNI-------------------------------VFLENPKH
PNSSPMPNAYNEH-----------------------------------------------------------
----FHSKQGHRGS----------------------------------------------------------
------------------------------------------------------------------------
------------------------------------------------------------------------
------------------------------------------------------------------------

------------------------------------------------------------------------
-----------------TTNVSRGRLIDRQAMI---------------------------------------
---------------------------------------------NQFNKGCSI------------------
------------------------------------------------------------------------
------------------------------------------------------------------------
------------------------------------------------------------------------
----------------------------
>XH860_6
-----------------------------APCSLVRYKPDK-----VTPQAGSRQDAID-------------
------------------------------------------------------------------------
---------------------RAWSL-EKQLIQT------------------------------TGTGTRDW
S---------------------------------------------------KAELDTILRTPS-GS-----
------------------------GKGHLSSVMSNLGYTGHH------------------------------
------------------------------------------------------------------------
------------INSVKNNGALGESWKGDPRNI-------------------------------VFLENPKH
PNSSPMPNAYNEH-----------------------------------------------------------
----FHSKQGHRGS----------------------------------------------------------
------------------------------------------------------------------------
------------------------------------------------------------------------
------------------------------------------------------------------------
------------------------------------------------------------------------
-----------------TTNVSRGRLIDRQAMI---------------------------------------
---------------------------------------------NQFNKGCSI------------------
------------------------------------------------------------------------
------------------------------------------------------------------------
------------------------------------------------------------------------
----------------------------
>YU-R612_6
-----------------------------APCSLVRYKPDK-----VTPQAGSRQDAID-------------
------------------------------------------------------------------------
---------------------RAWSL-EKQLIQT------------------------------TGTGTRDW
S---------------------------------------------------KAELDTILRTPS-GS-----
------------------------GKGHLSSVMSNLGYTGHH------------------------------

```
------------------------------------------------------------------------
------------INSVKNNGALGESWKGDPRNI-------------------------------VFLENPKH
PNSSPMPNAYNEH-----------------------------------------------------------
----FHSKQGHRGS----------------------------------------------------------
------------------------------------------------------------------------
------------------------------------------------------------------------
------------------------------------------------------------------------
------------------------------------------------------------------------
-----------------TTNVSRGRLIDRQAMI---------------------------------------
---------------------------------------------NQFNKGCSI------------------
------------------------------------------------------------------------
------------------------------------------------------------------------
------------------------------------------------------------------------
----------------------------
>15A34_5
----------------------------ASTNSIGSYGSVK-----SASDTYEKYATSK-------------
------------------------------------------------------------------------
---------------------SLKDV-AERYAAG------------------------------KSVGGSAT
A---------------------------------------------------GVGVGIYANYKGNGE-----
-------------------------STLTMERKVGLGYEYGV----FERNYHYG------------------
------------------------------------------------------------------------
------------ERQVNRGYIEGCAATG------------------------------------ESKYCAGS
HTDKISPYIYKDE-----------------------------------------------------------
----YKSKSKARGI----------------------------------------------------------
------------------------------------------------------------------------
------------------------------------------------------------------------
------------------------------------------------------------------------
------------------------------------------------------------------------
-----------------TISQGKEMVGNPNGPS---------------------------------------
------------------------------------------IIHGPGHSW---------------------
------------------------------------------------------------------------
------------------------------------------------------------------------
-------DLGAHGFPSTY------------------------------------------------------
```

```
----------------------------
>15A5_5
----------------------------ASTNSIGSYGSVK-----SASDTYEKYATSK-------------
------------------------------------------------------------------------
---------------------SLKDV-AERYAAG------------------------------KSVGGSAT
A---------------------------------------------------GVGVGIYANYKGNGE-----
-------------------------STLTMERKVGLGYEYGV----FERNYHYG------------------
------------------------------------------------------------------------
------------ERQVNRGYIEGCAATG------------------------------------ESKYCAGS
HTDKISPYIYKDE-----------------------------------------------------------
----YKSKSKARGI----------------------------------------------------------
------------------------------------------------------------------------
------------------------------------------------------------------------
------------------------------------------------------------------------
------------------------------------------------------------------------
-----------------TISQGKEMVGNPNGPS---------------------------------------
------------------------------------------IIHGPGHSW---------------------
------------------------------------------------------------------------
------------------------------------------------------------------------
-------DLGAHGFPSTY------------------------------------------------------
----------------------------
>3027STDY5784958_5
----------------------------ASTNSIGSYGSVK-----SASDTYEKYATSK-------------
------------------------------------------------------------------------
---------------------SLKDV-AERYAAG------------------------------KSVGGSAT
A---------------------------------------------------GVGVGIYANYKGNGE-----
-------------------------STLTMERKVGLGYEYGV----FERNYHYG------------------
------------------------------------------------------------------------
------------ERQVNRGYIEGCAATG------------------------------------ESKYCAGS
HTDKISPYIYKDE-----------------------------------------------------------
----YKSKSKARGI----------------------------------------------------------
------------------------------------------------------------------------
------------------------------------------------------------------------
```

```
------------------------------------------------------------------------
------------------------------------------------------------------------
-----------------TISQGKEMVGNPNGPS---------------------------------------
------------------------------------------IIHGPGHSW---------------------
------------------------------------------------------------------------
------------------------------------------------------------------------
-------DLGAHGFPSTY------------------------------------------------------
----------------------------
>Ab4653_5
----------------------------ASTNSIGSYGSVK-----SASDTYEKYATSK-------------
------------------------------------------------------------------------
---------------------SLKDV-AERYAAG------------------------------KSVGGSAT
A---------------------------------------------------GVGVGIYANYKGNGE-----
-------------------------STLTMERKVGLGYEYGV----FERNYHYG------------------
------------------------------------------------------------------------
------------ERQVNRGYIEGCAATG------------------------------------ESKYCAGS
HTDKISPYIYKDE-----------------------------------------------------------
----YKSKSKARGI----------------------------------------------------------
------------------------------------------------------------------------
------------------------------------------------------------------------
------------------------------------------------------------------------
------------------------------------------------------------------------
-----------------TISQGKEMVGNPNGPS---------------------------------------
------------------------------------------IIHGPGHSW---------------------
------------------------------------------------------------------------
------------------------------------------------------------------------
-------DLGAHGFPSTY------------------------------------------------------
----------------------------
>Ab4977_5
----------------------------ASTNSIGSYGSVK-----SASDTYEKYATSK-------------
------------------------------------------------------------------------
---------------------SLKDV-AERYAAG------------------------------KSVGGSAT
A---------------------------------------------------GVGVGIYANYKGNGE-----
```

```
-------------------------STLTMERKVGLGYEYGV----FERNYHYG------------------
------------------------------------------------------------------------
------------ERQVNRGYIEGCAATG------------------------------------ESKYCAGS
HTDKISPYIYKDE-----------------------------------------------------------
----YKSKSKARGI----------------------------------------------------------
------------------------------------------------------------------------
------------------------------------------------------------------------
------------------------------------------------------------------------
------------------------------------------------------------------------
-----------------TISQGKEMVGNPNGPS---------------------------------------
------------------------------------------IIHGPGHSW---------------------
------------------------------------------------------------------------
------------------------------------------------------------------------
-------DLGAHGFPSTY------------------------------------------------------
----------------------------
>ACICU_5
----------------------------ASTNSIGSYGSVK-----SASDTYEKYATSK-------------
------------------------------------------------------------------------
---------------------SLKDV-AERYAAG------------------------------KSVGGSAT
A---------------------------------------------------GVGVGIYANYKGNGE-----
-------------------------STLTMERKVGLGYEYGV----FERNYHYG------------------
------------------------------------------------------------------------
------------ERQVNRGYIEGCAATG------------------------------------ESKYCAGS
HTDKISPYIYKDE-----------------------------------------------------------
----YKSKSKARGI----------------------------------------------------------
------------------------------------------------------------------------
------------------------------------------------------------------------
------------------------------------------------------------------------
------------------------------------------------------------------------
-----------------TISQGKEMVGNPNGPS---------------------------------------
------------------------------------------IIHGPGHSW---------------------
------------------------------------------------------------------------
------------------------------------------------------------------------
```

-------DLGAHGFPSTY------------------------------------------------------
----------------------------
>AF-673_5
----------------------------ASTNSIGSYGSVK-----SASDTYEKYATSK-------------
------------------------------------------------------------------------
---------------------SLKDV-AERYAAG------------------------------KSVGGSAT
A---------------------------------------------------GVGVGIYANYKGNGE-----
-------------------------STLTMERKVGLGYEYGV----FERNYHYG------------------
------------------------------------------------------------------------
------------ERQVNRGYIEGCAATG------------------------------------ESKYCAGS
HTDKISPYIYKDE-----------------------------------------------------------
----YKSKSKARGI----------------------------------------------------------
------------------------------------------------------------------------
------------------------------------------------------------------------
------------------------------------------------------------------------
------------------------------------------------------------------------
-----------------TISQGKEMVGNPNGPS---------------------------------------
------------------------------------------IIHGPGHSW---------------------
------------------------------------------------------------------------
------------------------------------------------------------------------
-------DLGAHGFPSTY------------------------------------------------------
----------------------------
>AR_0056_5
----------------------------ASTNSIGSYGSVK-----SASDTYEKYATSK-------------
------------------------------------------------------------------------
---------------------SLKDV-AERYAAG------------------------------KSVGGSAT
A---------------------------------------------------GVGVGIYANYKGNGE-----
-------------------------STLTMERKVGLGYEYGV----FERNYHYG------------------
------------------------------------------------------------------------
------------ERQVNRGYIEGCAATG------------------------------------ESKYCAGS
HTDKISPYIYKDE-----------------------------------------------------------
----YKSKSKARGI----------------------------------------------------------
------------------------------------------------------------------------

------------------------------------------------------------------------
------------------------------------------------------------------------
------------------------------------------------------------------------
-----------------TISQGKEMVGNPNGPS---------------------------------------
------------------------------------------IIHGPGHSW---------------------
------------------------------------------------------------------------
------------------------------------------------------------------------
-------DLGAHGFPSTY------------------------------------------------------
----------------------------
>AR_0102_5
----------------------------ASTNSIGSYGSVK-----SASDTYEKYATSK-------------
------------------------------------------------------------------------
---------------------SLKDV-AERYAAG------------------------------KSVGGSAT
A---------------------------------------------------GVGVGIYANYKGNGE-----
-------------------------STLTMERKVGLGYEYGV----FERNYHYG------------------
------------------------------------------------------------------------
------------ERQVNRGYIEGCAATG------------------------------------ESKYCAGS
HTDKISPYIYKDE-----------------------------------------------------------
----YKSKSKARGI----------------------------------------------------------
------------------------------------------------------------------------
------------------------------------------------------------------------
------------------------------------------------------------------------
------------------------------------------------------------------------
-----------------TISQGKEMVGNPNGPS---------------------------------------
------------------------------------------IIHGPGHSW---------------------
------------------------------------------------------------------------
------------------------------------------------------------------------
-------DLGAHGFPSTY------------------------------------------------------
----------------------------
>BJAB07104_5
----------------------------ASTNSIGSYGSVK-----SASDTYEKYATSK-------------
------------------------------------------------------------------------
---------------------SLKDV-AERYAAG------------------------------KSVGGSAT

A---------------------------------------------------GVGVGIYANYKGNGE-----
-------------------------STLTMERKVGLGYEYGV----FERNYHYG------------------
------------------------------------------------------------------------
------------ERQVNRGYIEGCAATG------------------------------------ESKYCAGS
HTDKISPYIYKDE-----------------------------------------------------------
----YKSKSKARGI----------------------------------------------------------
------------------------------------------------------------------------
------------------------------------------------------------------------
------------------------------------------------------------------------
------------------------------------------------------------------------
-----------------TISQGKEMVGNPNGPS---------------------------------------
------------------------------------------IIHGPGHSW---------------------
------------------------------------------------------------------------
------------------------------------------------------------------------
-------DLGAHGFPSTY------------------------------------------------------
----------------------------
>BJAB0868_5
----------------------------ASTNSIGSYGSVK-----SASDTYEKYATSK-------------
------------------------------------------------------------------------
---------------------SLKDV-AERYAAG------------------------------KSVGGSAT
A---------------------------------------------------GVGVGIYANYKGNGE-----
-------------------------STLTMERKVGLGYEYGV----FERNYHYG------------------
------------------------------------------------------------------------
------------ERQVNRGYIEGCAATG------------------------------------ESKYCAGS
HTDKISPYIYKDE-----------------------------------------------------------
----YKSKSKARGI----------------------------------------------------------
------------------------------------------------------------------------
------------------------------------------------------------------------
------------------------------------------------------------------------
------------------------------------------------------------------------
-----------------TISQGKEMVGNPNGPS---------------------------------------
------------------------------------------IIHGPGHSW---------------------
------------------------------------------------------------------------

----------------------------------------------------------------------
-------DLGAHGFPSTY----------------------------------------------------
---------------------------
>CMC-CR-MDR-Ab4_5
---------------------------ASTNSIGSYGSVK-----SASDTYEKYATSK------------
----------------------------------------------------------------------
--------------------SLKDV-AERYAAG-----------------------------KSVGGSAT
A-------------------------------------------------GVGVGIYANYKGNGE-----
------------------------STLTMERKVGLGYEYGV----FERNYHYG-----------------
----------------------------------------------------------------------
------------ERQVNRGYIEGCAATG----------------------------------ESKYCAGS
HTDKISPYIYKDE---------------------------------------------------------
----YKSKSKARGI--------------------------------------------------------
----------------------------------------------------------------------
----------------------------------------------------------------------
----------------------------------------------------------------------
----------------------------------------------------------------------
----------------TISQGKEMVGNPNGPS--------------------------------------
-----------------------------------------IIHGPGHSW--------------------
----------------------------------------------------------------------
----------------------------------------------------------------------
-------DLGAHGFPSTY----------------------------------------------------
---------------------------
>CMC-CR-MDR-Ab66_5
---------------------------ASTNSIGSYGSVK-----SASDTYEKYATSK------------
----------------------------------------------------------------------
--------------------SLKDV-AERYAAG-----------------------------KSVGGSAT
A-------------------------------------------------GVGVGIYANYKGNGE-----
------------------------STLTMERKVGLGYEYGV----FERNYHYG-----------------
----------------------------------------------------------------------
------------ERQVNRGYIEGCAATG----------------------------------ESKYCAGS
HTDKISPYIYKDE---------------------------------------------------------
----YKSKSKARGI--------------------------------------------------------

```
------------------------------------------------------------------------
------------------------------------------------------------------------
------------------------------------------------------------------------
------------------------------------------------------------------------
-----------------TISQGKEMVGNPNGPS---------------------------------------
------------------------------------------IIHGPGHSW---------------------
------------------------------------------------------------------------
------------------------------------------------------------------------
-------DLGAHGFPSTY------------------------------------------------------
----------------------------
>CMC-MDR-Ab59_5
----------------------------ASTNSIGSYGSVK-----SASDTYEKYATSK-------------
------------------------------------------------------------------------
---------------------SLKDV-AERYAAG------------------------------KSVGGSAT
A---------------------------------------------------GVGVGIYANYKGNGE-----
-------------------------STLTMERKVGLGYEYGV----FERNYHYG------------------
------------------------------------------------------------------------
------------ERQVNRGYIEGCAATG------------------------------------ESKYCAGS
HTDKISPYIYKDE-----------------------------------------------------------
----YKSKSKARGI----------------------------------------------------------
------------------------------------------------------------------------
------------------------------------------------------------------------
------------------------------------------------------------------------
------------------------------------------------------------------------
-----------------TISQGKEMVGNPNGPS---------------------------------------
------------------------------------------IIHGPGHSW---------------------
------------------------------------------------------------------------
------------------------------------------------------------------------
-------DLGAHGFPSTY------------------------------------------------------
----------------------------
>HRAB-85_5
----------------------------ASTNSIGSYGSVK-----SASDTYEKYATSK-------------
------------------------------------------------------------------------
```

```
---------------------SLKDV-AERYAAG------------------------------KSVGGSAT
A---------------------------------------------------GVGVGIYANYKGNGE-----
-------------------------STLTMERKVGLGYEYGV----FERNYHYG------------------
------------------------------------------------------------------------
------------ERQVNRGYIEGCAATG------------------------------------ESKYCAGS
HTDKISPYIYKDE-----------------------------------------------------------
----YKSKSKARGI----------------------------------------------------------
------------------------------------------------------------------------
------------------------------------------------------------------------
------------------------------------------------------------------------
------------------------------------------------------------------------
-----------------TISQGKEMVGNPNGPS---------------------------------------
------------------------------------------IIHGPGHSW---------------------
------------------------------------------------------------------------
------------------------------------------------------------------------
-------DLGAHGFPSTY------------------------------------------------------
----------------------------
>KBN10P02143_5
----------------------------ASTNSIGSYGSVK-----SASDTYEKYATSK-------------
------------------------------------------------------------------------
---------------------SLKDV-AERYAAG------------------------------KSVGGSAT
A---------------------------------------------------GVGVGIYANYKGNGE-----
-------------------------STLTMERKVGLGYEYGV----FERNYHYG------------------
------------------------------------------------------------------------
------------ERQVNRGYIEGCAATG------------------------------------ESKYCAGS
HTDKISPYIYKDE-----------------------------------------------------------
----YKSKSKARGI----------------------------------------------------------
------------------------------------------------------------------------
------------------------------------------------------------------------
------------------------------------------------------------------------
------------------------------------------------------------------------
-----------------TISQGKEMVGNPNGPS---------------------------------------
------------------------------------------IIHGPGHSW---------------------
```

```
------------------------------------------------------------------------
------------------------------------------------------------------------
-------DLGAHGFPSTY------------------------------------------------------
----------------------------
>MDR-TJ_5
----------------------------ASTNSIGSYGSVK-----SASDTYEKYATSK-------------
------------------------------------------------------------------------
---------------------SLKDV-AERYAAG------------------------------KSVGGSAT
A---------------------------------------------------GVGVGIYANYKGNGE-----
-------------------------STLTMERKVGLGYEYGV----FERNYHYG------------------
------------------------------------------------------------------------
------------ERQVNRGYIEGCAATG------------------------------------ESKYCAGS
HTDKISPYIYKDE-----------------------------------------------------------
----YKSKSKARGI----------------------------------------------------------
------------------------------------------------------------------------
------------------------------------------------------------------------
------------------------------------------------------------------------
------------------------------------------------------------------------
-----------------TISQGKEMVGNPNGPS---------------------------------------
------------------------------------------IIHGPGHSW---------------------
------------------------------------------------------------------------
------------------------------------------------------------------------
-------DLGAHGFPSTY------------------------------------------------------
----------------------------
>MDR-ZJ06_5
----------------------------ASTNSIGSYGSVK-----SASDTYEKYATSK-------------
------------------------------------------------------------------------
---------------------SLKDV-AERYAAG------------------------------KSVGGSAT
A---------------------------------------------------GVGVGIYANYKGNGE-----
-------------------------STLTMERKVGLGYEYGV----FERNYHYG------------------
------------------------------------------------------------------------
------------ERQVNRGYIEGCAATG------------------------------------ESKYCAGS
HTDKISPYIYKDE-----------------------------------------------------------
```

----YKSKSKARGI----------------------------------------------------------
------------------------------------------------------------------------
------------------------------------------------------------------------
------------------------------------------------------------------------
------------------------------------------------------------------------
-----------------TISQGKEMVGNPNGPS---------------------------------------
------------------------------------------IIHGPGHSW---------------------
------------------------------------------------------------------------
------------------------------------------------------------------------
-------DLGAHGFPSTY------------------------------------------------------
----------------------------
>NCGM237_5
----------------------------ASTNSIGSYGSVK-----SASDTYEKYATSK-------------
------------------------------------------------------------------------
---------------------SLKDV-AERYAAG------------------------------KSVGGSAT
A---------------------------------------------------GVGVGIYANYKGNGE-----
-------------------------STLTMERKVGLGYEYGV----FERNYHYG------------------
------------------------------------------------------------------------
------------ERQVNRGYIEGCAATG------------------------------------ESKYCAGS
HTDKISPYIYKDE-----------------------------------------------------------
----YKSKSKARGI----------------------------------------------------------
------------------------------------------------------------------------
------------------------------------------------------------------------
------------------------------------------------------------------------
------------------------------------------------------------------------
-----------------TISQGKEMVGNPNGPS---------------------------------------
------------------------------------------IIHGPGHSW---------------------
------------------------------------------------------------------------
------------------------------------------------------------------------
-------DLGAHGFPSTY------------------------------------------------------
----------------------------
>SMC_Paed_Ab_BL01_5
----------------------------ASTNSIGSYGSVK-----SASDTYEKYATSK-------------

```
------------------------------------------------------------------------
---------------------SLKDV-AERYAAG------------------------------KSVGGSAT
A---------------------------------------------------GVGVGIYANYKGNGE-----
-------------------------STLTMERKVGLGYEYGV----FERNYHYG------------------
------------------------------------------------------------------------
------------ERQVNRGYIEGCAATG------------------------------------ESKYCAGS
HTDKISPYIYKDE-----------------------------------------------------------
----YKSKSKARGI----------------------------------------------------------
------------------------------------------------------------------------
------------------------------------------------------------------------
------------------------------------------------------------------------
------------------------------------------------------------------------
-----------------TISQGKEMVGNPNGPS---------------------------------------
------------------------------------------IIHGPGHSW---------------------
------------------------------------------------------------------------
------------------------------------------------------------------------
-------DLGAHGFPSTY------------------------------------------------------
----------------------------
>SSA6_5
----------------------------ASTNSIGSYGSVK-----SASDTYEKYATSK-------------
------------------------------------------------------------------------
---------------------SLKDV-AERYAAG------------------------------KSVGGSAT
A---------------------------------------------------GVGVGIYANYKGNGE-----
-------------------------STLTMERKVGLGYEYGV----FERNYHYG------------------
------------------------------------------------------------------------
------------ERQVNRGYIEGCAATG------------------------------------ESKYCAGS
HTDKISPYIYKDE-----------------------------------------------------------
----YKSKSKARGI----------------------------------------------------------
------------------------------------------------------------------------
------------------------------------------------------------------------
------------------------------------------------------------------------
------------------------------------------------------------------------
-----------------TISQGKEMVGNPNGPS---------------------------------------
```

------------------------------------------IIHGPGHSW---------------------
------------------------------------------------------------------------
------------------------------------------------------------------------
-------DLGAHGFPSTY------------------------------------------------------
----------------------------
>TCDC-AB0715_5
----------------------------ASTNSIGSYGSVK-----SASDTYEKYATSK-------------
------------------------------------------------------------------------
---------------------SLKDV-AERYAAG------------------------------KSVGGSAT
A---------------------------------------------------GVGVGIYANYKGNGE-----
-------------------------STLTMERKVGLGYEYGV----FERNYHYG------------------
------------------------------------------------------------------------
------------ERQVNRGYIEGCAATG------------------------------------ESKYCAGS
HTDKISPYIYKDE-----------------------------------------------------------
----YKSKSKARGI----------------------------------------------------------
------------------------------------------------------------------------
------------------------------------------------------------------------
------------------------------------------------------------------------
------------------------------------------------------------------------
-----------------TISQGKEMVGNPNGPS---------------------------------------
------------------------------------------IIHGPGHSW---------------------
------------------------------------------------------------------------
------------------------------------------------------------------------
-------DLGAHGFPSTY------------------------------------------------------
----------------------------
>TYTH-1_5
----------------------------ASTNSIGSYGSVK-----SASDTYEKYATSK-------------
------------------------------------------------------------------------
---------------------SLKDV-AERYAAG------------------------------KSVGGSAT
A---------------------------------------------------GVGVGIYANYKGNGE-----
-------------------------STLTMERKVGLGYEYGV----FERNYHYG------------------
------------------------------------------------------------------------
------------ERQVNRGYIEGCAATG------------------------------------ESKYCAGS

```
HTDKISPYIYKDE-----------------------------------------------------------
----YKSKSKARGI----------------------------------------------------------
------------------------------------------------------------------------
------------------------------------------------------------------------
------------------------------------------------------------------------
------------------------------------------------------------------------
-----------------TISQGKEMVGNPNGPS---------------------------------------
------------------------------------------IIHGPGHSW---------------------
------------------------------------------------------------------------
------------------------------------------------------------------------
-------DLGAHGFPSTY------------------------------------------------------
----------------------------
>USA2_5
----------------------------ASTNSIGSYGSVK-----SASDTYEKYATSK-------------
------------------------------------------------------------------------
---------------------SLKDV-AERYAAG------------------------------KSVGGSAT
A---------------------------------------------------GVGVGIYANYKGNGE-----
-------------------------STLTMERKVGLGYEYGV----FERNYHYG------------------
------------------------------------------------------------------------
------------ERQVNRGYIEGCAATG------------------------------------ESKYCAGS
HTDKISPYIYKDE-----------------------------------------------------------
----YKSKSKARGI----------------------------------------------------------
------------------------------------------------------------------------
------------------------------------------------------------------------
------------------------------------------------------------------------
------------------------------------------------------------------------
-----------------TISQGKEMVGNPNGPS---------------------------------------
------------------------------------------IIHGPGHSW---------------------
------------------------------------------------------------------------
------------------------------------------------------------------------
-------DLGAHGFPSTY------------------------------------------------------
----------------------------
>WCHAB005133_5
```

```
----------------------------ASTNSIGSYGSVK-----SASDTYEKYATSK-------------
------------------------------------------------------------------------
---------------------SLKDV-AERYAAG------------------------------KSVGGSAT
A---------------------------------------------------GVGVGIYANYKGNGE-----
-------------------------STLTMERKVGLGYEYGV----FERNYHYG------------------
------------------------------------------------------------------------
------------ERQVNRGYIEGCAATG------------------------------------ESKYCAGS
HTDKISPYIYKDE-----------------------------------------------------------
----YKSKSKARGI----------------------------------------------------------
------------------------------------------------------------------------
------------------------------------------------------------------------
------------------------------------------------------------------------
------------------------------------------------------------------------
-----------------TISQGKEMVGNPNGPS---------------------------------------
------------------------------------------IIHGPGHSW---------------------
------------------------------------------------------------------------
------------------------------------------------------------------------
-------DLGAHGFPSTY------------------------------------------------------
----------------------------
>XDR-BJ83_5
----------------------------ASTNSIGSYGSVK-----SASDTYEKYATSK-------------
------------------------------------------------------------------------
---------------------SLKDV-AERYAAG------------------------------KSVGGSAT
A---------------------------------------------------GVGVGIYANYKGNGE-----
-------------------------STLTMERKVGLGYEYGV----FERNYHYG------------------
------------------------------------------------------------------------
------------ERQVNRGYIEGCAATG------------------------------------ESKYCAGS
HTDKISPYIYKDE-----------------------------------------------------------
----YKSKSKARGI----------------------------------------------------------
------------------------------------------------------------------------
------------------------------------------------------------------------
------------------------------------------------------------------------
------------------------------------------------------------------------
```

-----------------TISQGKEMVGNPNGPS---------------------------------------
------------------------------------------IIHGPGHSW---------------------
------------------------------------------------------------------------
------------------------------------------------------------------------
-------DLGAHGFPSTY------------------------------------------------------
----------------------------
>XH386_5
----------------------------ASTNSIGSYGSVK-----SASDTYEKYATSK-------------
------------------------------------------------------------------------
---------------------SLKDV-AERYAAG------------------------------KSVGGSAT
A---------------------------------------------------GVGVGIYANYKGNGE-----
-------------------------STLTMERKVGLGYEYGV----FERNYHYG------------------
------------------------------------------------------------------------
------------ERQVNRGYIEGCAATG------------------------------------ESKYCAGS
HTDKISPYIYKDE-----------------------------------------------------------
----YKSKSKARGI----------------------------------------------------------
------------------------------------------------------------------------
------------------------------------------------------------------------
------------------------------------------------------------------------
------------------------------------------------------------------------
-----------------TISQGKEMVGNPNGPS---------------------------------------
------------------------------------------IIHGPGHSW---------------------
------------------------------------------------------------------------
------------------------------------------------------------------------
-------DLGAHGFPSTY------------------------------------------------------
----------------------------
>XH856_5
----------------------------ASTNSIGSYGSVK-----SASDTYEKYATSK-------------
------------------------------------------------------------------------
---------------------SLKDV-AERYAAG------------------------------KSVGGSAT
A---------------------------------------------------GVGVGIYANYKGNGE-----
-------------------------STLTMERKVGLGYEYGV----FERNYHYG------------------
------------------------------------------------------------------------

```
------------ERQVNRGYIEGCAATG------------------------------------ESKYCAGS
HTDKISPYIYKDE-----------------------------------------------------------
----YKSKSKARGI----------------------------------------------------------
------------------------------------------------------------------------
------------------------------------------------------------------------
------------------------------------------------------------------------
------------------------------------------------------------------------
-----------------TISQGKEMVGNPNGPS---------------------------------------
------------------------------------------IIHGPGHSW---------------------
------------------------------------------------------------------------
------------------------------------------------------------------------
-------DLGAHGFPSTY------------------------------------------------------
----------------------------
>YU-R612_5
----------------------------ASTNSIGSYGSVK-----SASDTYEKYATSK-------------
------------------------------------------------------------------------
---------------------SLKDV-AERYAAG------------------------------KSVGGSAT
A---------------------------------------------------GVGVGIYANYKGNGE-----
-------------------------STLTMERKVGLGYEYGV----FERNYHYG------------------
------------------------------------------------------------------------
------------ERQVNRGYIEGCAATG------------------------------------ESKYCAGS
HTDKISPYIYKDE-----------------------------------------------------------
----YKSKSKARGI----------------------------------------------------------
------------------------------------------------------------------------
------------------------------------------------------------------------
------------------------------------------------------------------------
------------------------------------------------------------------------
-----------------TISQGKEMVGNPNGPS---------------------------------------
------------------------------------------IIHGPGHSW---------------------
------------------------------------------------------------------------
------------------------------------------------------------------------
-------DLGAHGFPSTY------------------------------------------------------
----------------------------
```

```
>Ab4568_5
----------------------------ASTNSIGSYGSVK-----SASDTYEKYATSK-------------
------------------------------------------------------------------------
---------------------SLKDV-AERYAAG------------------------------KSVGGSAT
A---------------------------------------------------GVGVGIYANYKGNGE-----
-------------------------STLTMERKVGLGYEYGV----FERNYHYG------------------
------------------------------------------------------------------------
------------ERQVNRGYIEGCAATG------------------------------------ESKYCAGS
HTDKISPYIYKDE-----------------------------------------------------------
----YKSKSKARGI----------------------------------------------------------
------------------------------------------------------------------------
------------------------------------------------------------------------
------------------------------------------------------------------------
------------------------------------------------------------------------
-----------------TISQGKEMVGNPNGPS---------------------------------------
------------------------------------------IIHGPGHSW---------------------
------------------------------------------------------------------------
------------------------------------------------------------------------
-------DLGAHGFPST-------------------------------------------------------
----------------------------
>6200_21
-----------------------------NFKSVGKIKEER-----ARRQTSSSAPYIKHT-----------
------------------------------------------------------------------------
---------------------MAKCKTQAQNISK------------------------------KEAQYWDM
S----KCESNVAQVTAFRNRLEKEGL--------------------------NKGYKVDLGDNK-GS-----
------------------------------------------------------------------------
------------------------------------------------------------------------
----------------------------------HY----------------------------YIYDAGRN
IGFNEGKET---------------------------------------------------------------
----QYMRIEVS------------------------------------------------------------
------------------------------------------------------------------------
------------------------------------------------------------------------
----------------------------------------------------------------------ST
```

```
NEFHGHPISK--------------------------------------------------------------
-----------------------QRYE---------------------------------------------
------------------------------------------SWQSKT------------------------
------------------------------------------------------------------------
------------------------------------------------------------------------
------------------------------------------------------------------------
----------------------------
>AB031_27
---------------------------NNNPLVGIDLSNKS-----RSEIENILSNSSEVSY----------
------------------------------------------------------------------------
---------------------KGGSP-DKKFMQW------------------------------------KW
N---------------------------------------------------DNGQTAVRLDPP--------
---------------------------------DKVTLYDHA------------------------------
------------------------------------------------------------------------
----------------------------------------------------------------HLYDKNGN
PLDK--------------------------------------------------------------------
------------------------------------------------------------------------
------------------------------------------------------------------------
------------------------------------------------------------------------
------------------------------------------------------------------------
------------------------------------------------------------------------
-----------------DGNIVPRESPDAHIPI---------------------------------------
-------------------------------------------------GDCK-------------------
------------------------------------------------------------------------
------------------------------------------------------------------------
------------------------------------------------------------------------
----------------------------
>15A5_18
-----------------------------MSKIYIVKSGDT-----LWGISKKHHISVKELARINSLSGRMI
H-----------------------------------------------------------------------
---------------------NLRIG-QKIYLQN------------------------------DVNNTNNF
E----TQLKIILMDLSFKPILKATIQLEFDGKKIIRNTKNSIFEDINIQDH-SKGLKVFFKNLN-GT-----
-FDL---------------------IADHKVLPLGRKVLKLT----SRKMKVEGSHYAKDGILNETVNQIMS
```

NLKKVGKP---------------------------------------------------IVESISTTSNKDE
LKNK--------QPPLKIPEEKIEQKRTDNGNSTHI-------------------------I--AAQFTEDN
FLLKPVNNKYRAYIVNAAKRHGFTPHSLAAVIEAEAAKIKKTGEWNTNSKANSSTAAGLTQFLDETWLAMCK
D---KSSLVGQYVM-------NNPKLTIQQKLNLRF---------------NAEMAIDAAAAYAISNFKSSG
LPYQKLT--EPSSIAKFAY----LLHH---------EGATGGKNFVLNTLSQERAKKLLFTQFGKNGAKQAA
DFLNRYKGDAKAAYGAWLRNYIDGHINIYQYVVDKSKTSGINLSTDETIKL---------------------
----------------------------------------------------------------------LK
GQTISTPAPK-------------------ISTTTNNQQVTNVS-----------------------------
-----------------TIEKSESKIRINTSQA---------------------------------------
PTNNVGGD---------------------------N------KWHNPLAD-CKLRTAGL-------------
-------ANAKGATFGKVRNNGTKNHQGVD-----LQANPGTK--IYAVCG--GVIAFAGATGGAYGKVIVL
KVDIND-----------------------LPEKQKKYAQTKLTKNKYVYFFYAHL------SVIDVDKGDPV
DTGEVIGKTGATGNANKMTTISKGAHLHFEARSAPLLGVGLDGRFDPIPFINANLPY---------------
----------------------------

>3027STDY5784958_18

-----------------------------MSKIYIVKSGDT-----LWGISKKHHISVKELARINSLSGRMI
H-----------------------------------------------------------------------
---------------------NLRIG-QKIYLQN------------------------------DVNNTNNF
E----TQLKIILMDLSFKPILKATIQLEFDGKKIIRNTKNSIFEDINIQDH-SKGLKVFFKNLN-GT-----
-FDL---------------------IADHKVLPLGRKVLKLT----SRKMKVEGSHYAKDGILNETVNQIMS
NLKKVGKP---------------------------------------------------IVESISTTSNKDE
LKNK--------QPPLKIPEEKIEQKRTDNGNSTHI-------------------------I--AAQFTEDN
FLLKPVNNKYRAYIVNAAKRHGFTPHSLAAVIEAEAAKIKKTGEWNTNSKANSSTAAGLTQFLDETWLAMCK
D---KSSLVGQYVM-------NNPKLTIQQKLNLRF---------------NAEMAIDAAAAYAISNFKSSG
LPYQKLT--EPSSIAKFAY----LLHH---------EGATGGKNFVLNTLSQERAKKLLFTQFGKNGAKQAA
DFLNRYKGDAKAAYGAWLRNYIDGHINIYQYVVDKSKTSGINLSTDETIKL---------------------
----------------------------------------------------------------------LK
GQTISTPAPK-------------------ISTTTNNQQVTNVS-----------------------------
-----------------TIEKSESKIRINTSQA---------------------------------------
PTNNVGGD---------------------------N------KWHNPLAD-CKLRTAGL-------------
-------ANAKGATFGKVRNNGTKNHQGVD-----LQANPGTK--IYAVCG--GVIAFAGATGGAYGKVIVL
KVDIND-----------------------LPEKQKKYAQTKLTKNKYVYFFYAHL------SVIDVDKGDPV
DTGEVIGKTGATGNANKMTTISKGAHLHFEARSAPLLGVGLDGRFDPIPFINANLPY---------------

```
----------------------------
>AB07_18
-----------------------------MSKIYIVKSGDT-----LWGISKKHHISVKELARINSLSGRMI
H-----------------------------------------------------------------------
---------------------NLRIG-QKIYLQN------------------------------DVNNTNNF
E----TQLKIILMDLSFKPILKATIQLEFDGKKIIRNTKNSIFEDINIQDH-SKGLKVFFKNLN-GT-----
-FDL---------------------IADHKVLPLGRKVLKLT----SRKMKVEGSHYAKDGILNETVNQIMS
NLKKVGKP---------------------------------------------------IVESISTTSNKDE
LKNK--------QPPLKIPEEKIEQKRTDNGNSTHI-------------------------I--AAQFTEDN
FLLKPVNNKYRAYIVNAAKRHGFTPHSLAAVIEAEAAKIKKTGEWNTNSKANSSTAAGLTQFLDETWLAMCK
D---KSSLVGQYVM-------NNPKLTIQQKLNLRF---------------NAEMAIDAAAAYAISNFKSSG
LPYQKLT--EPSSIAKFAY----LLHH---------EGATGGKNFVLNTLSQERAKKLLFTQFGKNGAKQAA
DFLNRYKGDAKAAYGAWLRNYIDGHINIYQYVVDKSKTSGINLSTDETIKL---------------------
----------------------------------------------------------------------LK
GQTISTPAPK-------------------ISTTTNNQQVTNVS-----------------------------
-----------------TIEKSESKIRINTSQA---------------------------------------
PTNNVGGD---------------------------N------KWHNPLAD-CKLRTAGL-------------
-------ANAKGATFGKVRNNGTKNHQGVD-----LQANPGTK--IYAVCG--GVIAFAGATGGAYGKVIVL
KVDIND-----------------------LPEKQKKYAQTKLTKNKYVYFFYAHL------SVIDVDKGDPV
DTGEVIGKTGATGNANKMTTISKGAHLHFEARSAPLLGVGLDGRFDPIPFINANLPY---------------
----------------------------
>Ab4568_18
-----------------------------MSKIYIVKSGDT-----LWGISKKHHISVKELARINSLSGRMI
H-----------------------------------------------------------------------
---------------------NLRIG-QKIYLQN------------------------------DVNNTNNF
E----TQLKIILMDLSFKPILKATIQLEFDGKKIIRNTKNSIFEDINIQDH-SKGLKVFFKNLN-GT-----
-FDL---------------------IADHKVLPLGRKVLKLT----SRKMKVEGSHYAKDGILNETVNQIMS
NLKKVGKP---------------------------------------------------IVESISTTSNKDE
LKNK--------QPPLKIPEEKIEQKRTDNGNSTHI-------------------------I--AAQFTEDN
FLLKPVNNKYRAYIVNAAKRHGFTPHSLAAVIEAEAAKIKKTGEWNTNSKANSSTAAGLTQFLDETWLAMCK
D---KSSLVGQYVM-------NNPKLTIQQKLNLRF---------------NAEMAIDAAAAYAISNFKSSG
LPYQKLT--EPSSIAKFAY----LLHH---------EGATGGKNFVLNTLSQERAKKLLFTQFGKNGAKQAA
DFLNRYKGDAKAAYGAWLRNYIDGHINIYQYVVDKSKTSGINLSTDETIKL---------------------
```

```
----------------------------------------------------------------------LK
GQTISTPAPK-------------------ISTTTNNQQVTNVS-----------------------------
-----------------TIEKSESKIRINTSQA---------------------------------------
PTNNVGGD---------------------------N------KWHNPLAD-CKLRTAGL-------------
-------ANAKGATFGKVRNNGTKNHQGVD-----LQANPGTK--IYAVCG--GVIAFAGATGGAYGKVIVL
KVDIND-----------------------LPEKQKKYAQTKLTKNKYVYFFYAHL------SVIDVDKGDPV
DTGEVIGKTGATGNANKMTTISKGAHLHFEARSAPLLGVGLDGRFDPIPFINANLPY---------------
----------------------------
>Ab4977_18
-----------------------------MSKIYIVKSGDT-----LWGISKKHHISVKELARINSLSGRMI
H-----------------------------------------------------------------------
---------------------NLRIG-QKIYLQN------------------------------DVNNTNNF
E----TQLKIILMDLSFKPILKATIQLEFDGKKIIRNTKNSIFEDINIQDH-SKGLKVFFKNLN-GT-----
-FDL---------------------IADHKVLPLGRKVLKLT----SRKMKVEGSHYAKDGILNETVNQIMS
NLKKVGKP---------------------------------------------------IVESISTTSNKDE
LKNK--------QPPLKIPEEKIEQKRTDNGNSTHI-------------------------I--AAQFTEDN
FLLKPVNNKYRAYIVNAAKRHGFTPHSLAAVIEAEAAKIKKTGEWNTNSKANSSTAAGLTQFLDETWLAMCK
D---KSSLVGQYVM-------NNPKLTIQQKLNLRF---------------NAEMAIDAAAAYAISNFKSSG
LPYQKLT--EPSSIAKFAY----LLHH---------EGATGGKNFVLNTLSQERAKKLLFTQFGKNGAKQAA
DFLNRYKGDAKAAYGAWLRNYIDGHINIYQYVVDKSKTSGINLSTDETIKL---------------------
----------------------------------------------------------------------LK
GQTISTPAPK-------------------ISTTTNNQQVTNVS-----------------------------
-----------------TIEKSESKIRINTSQA---------------------------------------
PTNNVGGD---------------------------N------KWHNPLAD-CKLRTAGL-------------
-------ANAKGATFGKVRNNGTKNHQGVD-----LQANPGTK--IYAVCG--GVIAFAGATGGAYGKVIVL
KVDIND-----------------------LPEKQKKYAQTKLTKNKYVYFFYAHL------SVIDVDKGDPV
DTGEVIGKTGATGNANKMTTISKGAHLHFEARSAPLLGVGLDGRFDPIPFINANLPY---------------
----------------------------
>AC29_18
-----------------------------MSKIYIVKSGDT-----LWGISKKHHISVKELARINSLSGRMI
H-----------------------------------------------------------------------
---------------------NLRIG-QKIYLQN------------------------------DVNNTNNF
E----TQLKIILMDLSFKPILKATIQLEFDGKKIIRNTKNSIFEDINIQDH-SKGLKVFFKNLN-GT-----
```

```
-FDL---------------------IADHKVLPLGRKVLKLT----SRKMKVEGSHYAKDGILNETVNQIMS
NLKKVGKP---------------------------------------------------IVESISTTSNKDE
LKNK--------QPPLKIPEEKIEQKRTDNGNSTHI-------------------------I--AAQFTEDN
FLLKPVNNKYRAYIVNAAKRHGFTPHSLAAVIEAEAAKIKKTGEWNTNSKANSSTAAGLTQFLDETWLAMCK
D---KSSLVGQYVM-------NNPKLTIQQKLNLRF---------------NAEMAIDAAAAYAISNFKSSG
LPYQKLT--EPSSIAKFAY----LLHH---------EGATGGKNFVLNTLSQERAKKLLFTQFGKNGAKQAA
DFLNRYKGDAKAAYGAWLRNYIDGHINIYQYVVDKSKTSGINLSTDETIKL---------------------
----------------------------------------------------------------------LK
GQTISTPAPK-------------------ISTTTNNQQVTNVS-----------------------------
-----------------TIEKSESKIRINTSQA---------------------------------------
PTNNVGGD---------------------------N------KWHNPLAD-CKLRTAGL-------------
-------ANAKGATFGKVRNNGTKNHQGVD-----LQANPGTK--IYAVCG--GVIAFAGATGGAYGKVIVL
KVDIND-----------------------LPEKQKKYAQTKLTKNKYVYFFYAHL------SVIDVDKGDPV
DTGEVIGKTGATGNANKMTTISKGAHLHFEARSAPLLGVGLDGRFDPIPFINANLPY---------------
----------------------------
>AC30_18
-----------------------------MSKIYIVKSGDT-----LWGISKKHHISVKELARINSLSGRMI
H-----------------------------------------------------------------------
---------------------NLRIG-QKIYLQN------------------------------DVNNTNNF
E----TQLKIILMDLSFKPILKATIQLEFDGKKIIRNTKNSIFEDINIQDH-SKGLKVFFKNLN-GT-----
-FDL---------------------IADHKVLPLGRKVLKLT----SRKMKVEGSHYAKDGILNETVNQIMS
NLKKVGKP---------------------------------------------------IVESISTTSNKDE
LKNK--------QPPLKIPEEKIEQKRTDNGNSTHI-------------------------I--AAQFTEDN
FLLKPVNNKYRAYIVNAAKRHGFTPHSLAAVIEAEAAKIKKTGEWNTNSKANSSTAAGLTQFLDETWLAMCK
D---KSSLVGQYVM-------NNPKLTIQQKLNLRF---------------NAEMAIDAAAAYAISNFKSSG
LPYQKLT--EPSSIAKFAY----LLHH---------EGATGGKNFVLNTLSQERAKKLLFTQFGKNGAKQAA
DFLNRYKGDAKAAYGAWLRNYIDGHINIYQYVVDKSKTSGINLSTDETIKL---------------------
----------------------------------------------------------------------LK
GQTISTPAPK-------------------ISTTTNNQQVTNVS-----------------------------
-----------------TIEKSESKIRINTSQA---------------------------------------
PTNNVGGD---------------------------N------KWHNPLAD-CKLRTAGL-------------
-------ANAKGATFGKVRNNGTKNHQGVD-----LQANPGTK--IYAVCG--GVIAFAGATGGAYGKVIVL
KVDIND-----------------------LPEKQKKYAQTKLTKNKYVYFFYAHL------SVIDVDKGDPV
```

DTGEVIGKTGATGNANKMTTISKGAHLHFEARSAPLLGVGLDGRFDPIPFINANLPY---------------
-----------------------------
>AF-673_18
------------------------------MSKIYIVKSGDT-----LWGISKKHHISVKELARINSLSGRMI
H---------------------------------------------------------------------
---------------------NLRIG-QKIYLQN------------------------------DVNNTNNF
E----TQLKIILMDLSFKPILKATIQLEFDGKKIIRNTKNSIFEDINIQDH-SKGLKVFFKNLN-GT-----
-FDL---------------------IADHKVLPLGRKVLKLT----SRKMKVEGSHYAKDGILNETVNQIMS
NLKKVGKP---------------------------------------------------IVESISTTSNKDE
LKNK--------QPPLKIPEEKIEQKRTDNGNSTHI-------------------------I--AAQFTEDN
FLLKPVNNKYRAYIVNAAKRHGFTPHSLAAVIEAEAAKIKKTGEWNTNSKANSSTAAGLTQFLDETWLAMCK
D---KSSLVGQYVM-------NNPKLTIQQKLNLRF---------------NAEMAIDAAAAYAISNFKSSG
LPYQKLT--EPSSIAKFAY----LLHH---------EGATGGKNFVLNTLSQERAKKLLFTQFGKNGAKQAA
DFLNRYKGDAKAAYGAWLRNYIDGHINIYQYVVDKSKTSGINLSTDETIKL---------------------
--------------------------------------------------------------------LK
GQTISTPAPK-------------------ISTTTNNQQVTNVS-----------------------------
-----------------TIEKSESKIRINTSQA---------------------------------------
PTNNVGGD--------------------------N------KWHNPLAD-CKLRTAGL-------------
-------ANAKGATFGKVRNNGTKNHQGVD-----LQANPGTK--IYAVCG--GVIAFAGATGGAYGKVIVL
KVDIND------------------------LPEKQKKYAQTKLTKNKYVYFFYAHL------SVIDVDKGDPV
DTGEVIGKTGATGNANKMTTISKGAHLHFEARSAPLLGVGLDGRFDPIPFINANLPY---------------
-----------------------------
>AR_0102_18
------------------------------MSKIYIVKSGDT-----LWGISKKHHISVKELARINSLSGRMI
H---------------------------------------------------------------------
---------------------NLRIG-QKIYLQN------------------------------DVNNTNNF
E----TQLKIILMDLSFKPILKATIQLEFDGKKIIRNTKNSIFEDINIQDH-SKGLKVFFKNLN-GT-----
-FDL---------------------IADHKVLPLGRKVLKLT----SRKMKVEGSHYAKDGILNETVNQIMS
NLKKVGKP---------------------------------------------------IVESISTTSNKDE
LKNK--------QPPLKIPEEKIEQKRTDNGNSTHI-------------------------I--AAQFTEDN
FLLKPVNNKYRAYIVNAAKRHGFTPHSLAAVIEAEAAKIKKTGEWNTNSKANSSTAAGLTQFLDETWLAMCK
D---KSSLVGQYVM-------NNPKLTIQQKLNLRF---------------NAEMAIDAAAAYAISNFKSSG
LPYQKLT--EPSSIAKFAY----LLHH---------EGATGGKNFVLNTLSQERAKKLLFTQFGKNGAKQAA

```
DFLNRYKGDAKAAYGAWLRNYIDGHINIYQYVVDKSKTSGINLSTDETIKL---------------------
----------------------------------------------------------------------LK
GQTISTPAPK-------------------ISTTTNNQQVTNVS-----------------------------
-----------------TIEKSESKIRINTSQA---------------------------------------
PTNNVGGD---------------------------N------KWHNPLAD-CKLRTAGL-------------
-------ANAKGATFGKVRNNGTKNHQGVD-----LQANPGTK--IYAVCG--GVIAFAGATGGAYGKVIVL
KVDIND-----------------------LPEKQKKYAQTKLTKNKYVYFFYAHL------SVIDVDKGDPV
DTGEVIGKTGATGNANKMTTISKGAHLHFEARSAPLLGVGLDGRFDPIPFINANLPY---------------
----------------------------
>AYP-A2_18
-----------------------------MSKIYIVKSGDT-----LWGISKKHHISVKELARINSLSGRMI
H-----------------------------------------------------------------------
---------------------NLRIG-QKIYLQN------------------------------DVNNTNNF
E----TQLKIILMDLSFKPILKATIQLEFDGKKIIRNTKNSIFEDINIQDH-SKGLKVFFKNLN-GT-----
-FDL---------------------IADHKVLPLGRKVLKLT----SRKMKVEGSHYAKDGILNETVNQIMS
NLKKVGKP---------------------------------------------------IVESISTTSNKDE
LKNK--------QPPLKIPEEKIEQKRTDNGNSTHI-------------------------I--AAQFTEDN
FLLKPVNNKYRAYIVNAAKRHGFTPHSLAAVIEAEAAKIKKTGEWNTNSKANSSTAAGLTQFLDETWLAMCK
D---KSSLVGQYVM-------NNPKLTIQQKLNLRF---------------NAEMAIDAAAAYAISNFKSSG
LPYQKLT--EPSSIAKFAY----LLHH---------EGATGGKNFVLNTLSQERAKKLLFTQFGKNGAKQAA
DFLNRYKGDAKAAYGAWLRNYIDGHINIYQYVVDKSKTSGINLSTDETIKL---------------------
----------------------------------------------------------------------LK
GQTISTPAPK-------------------ISTTTNNQQVTNVS-----------------------------
-----------------TIEKSESKIRINTSQA---------------------------------------
PTNNVGGD---------------------------N------KWHNPLAD-CKLRTAGL-------------
-------ANAKGATFGKVRNNGTKNHQGVD-----LQANPGTK--IYAVCG--GVIAFAGATGGAYGKVIVL
KVDIND-----------------------LPEKQKKYAQTKLTKNKYVYFFYAHL------SVIDVDKGDPV
DTGEVIGKTGATGNANKMTTISKGAHLHFEARSAPLLGVGLDGRFDPIPFINANLPY---------------
----------------------------
>CBA7_18
-----------------------------MSKIYIVKSGDT-----LWGISKKHHISVKELARINSLSGRMI
H-----------------------------------------------------------------------
---------------------NLRIG-QKIYLQN------------------------------DVNNTNNF
```

E----TQLKIILMDLSFKPILKATIQLEFDGKKIIRNTKNSIFEDINIQDH-SKGLKVFFKNLN-GT-----
-FDL---------------------IADHKVLPLGRKVLKLT----SRKMKVEGSHYAKDGILNETVNQIMS
NLKKVGKP---------------------------------------------------IVESISTTSNKDE
LKNK--------QPPLKIPEEKIEQKRTDNGNSTHI-------------------------I--AAQFTEDN
FLLKPVNNKYRAYIVNAAKRHGFTPHSLAAVIEAEAAKIKKTGEWNTNSKANSSTAAGLTQFLDETWLAMCK
D---KSSLVGQYVM-------NNPKLTIQQKLNLRF---------------NAEMAIDAAAAYAISNFKSSG
LPYQKLT--EPSSIAKFAY----LLHH---------EGATGGKNFVLNTLSQERAKKLLFTQFGKNGAKQAA
DFLNRYKGDAKAAYGAWLRNYIDGHINIYQYVVDKSKTSGINLSTDETIKL---------------------
----------------------------------------------------------------------LK
GQTISTPAPK-------------------ISTTTNNQQVTNVS-----------------------------
-----------------TIEKSESKIRINTSQA---------------------------------------
PTNNVGGD---------------------------N------KWHNPLAD-CKLRTAGL-------------
-------ANAKGATFGKVRNNGTKNHQGVD-----LQANPGTK--IYAVCG--GVIAFAGATGGAYGKVIVL
KVDIND-----------------------LPEKQKKYAQTKLTKNKYVYFFYAHL------SVIDVDKGDPV
DTGEVIGKTGATGNANKMTTISKGAHLHFEARSAPLLGVGLDGRFDPIPFINANLPY---------------
----------------------------

>CMC-CR-MDR-Ab4_18
-----------------------------MSKIYIVKSGDT-----LWGISKKHHISVKELARINSLSGRMI
H-----------------------------------------------------------------------
---------------------NLRIG-QKIYLQN------------------------------DVNNTNNF
E----TQLKIILMDLSFKPILKATIQLEFDGKKIIRNTKNSIFEDINIQDH-SKGLKVFFKNLN-GT-----
-FDL---------------------IADHKVLPLGRKVLKLT----SRKMKVEGSHYAKDGILNETVNQIMS
NLKKVGKP---------------------------------------------------IVESISTTSNKDE
LKNK--------QPPLKIPEEKIEQKRTDNGNSTHI-------------------------I--AAQFTEDN
FLLKPVNNKYRAYIVNAAKRHGFTPHSLAAVIEAEAAKIKKTGEWNTNSKANSSTAAGLTQFLDETWLAMCK
D---KSSLVGQYVM-------NNPKLTIQQKLNLRF---------------NAEMAIDAAAAYAISNFKSSG
LPYQKLT--EPSSIAKFAY----LLHH---------EGATGGKNFVLNTLSQERAKKLLFTQFGKNGAKQAA
DFLNRYKGDAKAAYGAWLRNYIDGHINIYQYVVDKSKTSGINLSTDETIKL---------------------
----------------------------------------------------------------------LK
GQTISTPAPK-------------------ISTTTNNQQVTNVS-----------------------------
-----------------TIEKSESKIRINTSQA---------------------------------------
PTNNVGGD---------------------------N------KWHNPLAD-CKLRTAGL-------------
-------ANAKGATFGKVRNNGTKNHQGVD-----LQANPGTK--IYAVCG--GVIAFAGATGGAYGKVIVL

KVDIND-----------------------LPEKQKKYAQTKLTKNKYVYFFYAHL------SVIDVDKGDPV
DTGEVIGKTGATGNANKMTTISKGAHLHFEARSAPLLGVGLDGRFDPIPFINANLPY---------------
----------------------------
>CMC-CR-MDR-Ab66_18
-----------------------------MSKIYIVKSGDT-----LWGISKKHHISVKELARINSLSGRMI
H-----------------------------------------------------------------------
---------------------NLRIG-QKIYLQN------------------------------DVNNTNNF
E----TQLKIILMDLSFKPILKATIQLEFDGKKIIRNTKNSIFEDINIQDH-SKGLKVFFKNLN-GT-----
-FDL---------------------IADHKVLPLGRKVLKLT----SRKMKVEGSHYAKDGILNETVNQIMS
NLKKVGKP---------------------------------------------------IVESISTTSNKDE
LKNK--------QPPLKIPEEKIEQKRTDNGNSTHI-------------------------I--AAQFTEDN
FLLKPVNNKYRAYIVNAAKRHGFTPHSLAAVIEAEAAKIKKTGEWNTNSKANSSTAAGLTQFLDETWLAMCK
D---KSSLVGQYVM-------NNPKLTIQQKLNLRF---------------NAEMAIDAAAAYAISNFKSSG
LPYQKLT--EPSSIAKFAY----LLHH---------EGATGGKNFVLNTLSQERAKKLLFTQFGKNGAKQAA
DFLNRYKGDAKAAYGAWLRNYIDGHINIYQYVVDKSKTSGINLSTDETIKL---------------------
----------------------------------------------------------------------LK
GQTISTPAPK-------------------ISTTTNNQQVTNVS-----------------------------
-----------------TIEKSESKIRINTSQA---------------------------------------
PTNNVGGD---------------------------N------KWHNPLAD-CKLRTAGL-------------
-------ANAKGATFGKVRNNGTKNHQGVD-----LQANPGTK--IYAVCG--GVIAFAGATGGAYGKVIVL
KVDIND-----------------------LPEKQKKYAQTKLTKNKYVYFFYAHL------SVIDVDKGDPV
DTGEVIGKTGATGNANKMTTISKGAHLHFEARSAPLLGVGLDGRFDPIPFINANLPY---------------
----------------------------
>HRAB-85_18
-----------------------------MSKIYIVKSGDT-----LWGISKKHHISVKELARINSLSGRMI
H-----------------------------------------------------------------------
---------------------NLRIG-QKIYLQN------------------------------DVNNTNNF
E----TQLKIILMDLSFKPILKATIQLEFDGKKIIRNTKNSIFEDINIQDH-SKGLKVFFKNLN-GT-----
-FDL---------------------IADHKVLPLGRKVLKLT----SRKMKVEGSHYAKDGILNETVNQIMS
NLKKVGKP---------------------------------------------------IVESISTTSNKDE
LKNK--------QPPLKIPEEKIEQKRTDNGNSTHI-------------------------I--AAQFTEDN
FLLKPVNNKYRAYIVNAAKRHGFTPHSLAAVIEAEAAKIKKTGEWNTNSKANSSTAAGLTQFLDETWLAMCK
D---KSSLVGQYVM-------NNPKLTIQQKLNLRF---------------NAEMAIDAAAAYAISNFKSSG

```
LPYQKLT--EPSSIAKFAY----LLHH---------EGATGGKNFVLNTLSQERAKKLLFTQFGKNGAKQAA
DFLNRYKGDAKAAYGAWLRNYIDGHINIYQYVVDKSKTSGINLSTDETIKL---------------------
----------------------------------------------------------------------LK
GQTISTPAPK-------------------ISTTTNNQQVTNVS-----------------------------
-----------------TIEKSESKIRINTSQA---------------------------------------
PTNNVGGD---------------------------N------KWHNPLAD-CKLRTAGL-------------
-------ANAKGATFGKVRNNGTKNHQGVD-----LQANPGTK--IYAVCG--GVIAFAGATGGAYGKVIVL
KVDIND-----------------------LPEKQKKYAQTKLTKNKYVYFFYAHL------SVIDVDKGDPV
DTGEVIGKTGATGNANKMTTISKGAHLHFEARSAPLLGVGLDGRFDPIPFINANLPY---------------
----------------------------
>JBA13_18
-----------------------------MSKIYIVKSGDT-----LWGISKKHHISVKELARINSLSGRMI
H-----------------------------------------------------------------------
---------------------NLRIG-QKIYLQN------------------------------DVNNTNNF
E----TQLKIILMDLSFKPILKATIQLEFDGKKIIRNTKNSIFEDINIQDH-SKGLKVFFKNLN-GT-----
-FDL---------------------IADHKVLPLGRKVLKLT----SRKMKVEGSHYAKDGILNETVNQIMS
NLKKVGKP---------------------------------------------------IVESISTTSNKDE
LKNK--------QPPLKIPEEKIEQKRTDNGNSTHI-------------------------I--AAQFTEDN
FLLKPVNNKYRAYIVNAAKRHGFTPHSLAAVIEAEAAKIKKTGEWNTNSKANSSTAAGLTQFLDETWLAMCK
D---KSSLVGQYVM-------NNPKLTIQQKLNLRF---------------NAEMAIDAAAAYAISNFKSSG
LPYQKLT--EPSSIAKFAY----LLHH---------EGATGGKNFVLNTLSQERAKKLLFTQFGKNGAKQAA
DFLNRYKGDAKAAYGAWLRNYIDGHINIYQYVVDKSKTSGINLSTDETIKL---------------------
----------------------------------------------------------------------LK
GQTISTPAPK-------------------ISTTTNNQQVTNVS-----------------------------
-----------------TIEKSESKIRINTSQA---------------------------------------
PTNNVGGD---------------------------N------KWHNPLAD-CKLRTAGL-------------
-------ANAKGATFGKVRNNGTKNHQGVD-----LQANPGTK--IYAVCG--GVIAFAGATGGAYGKVIVL
KVDIND-----------------------LPEKQKKYAQTKLTKNKYVYFFYAHL------SVIDVDKGDPV
DTGEVIGKTGATGNANKMTTISKGAHLHFEARSAPLLGVGLDGRFDPIPFINANLPY---------------
----------------------------
>KAB02_18
-----------------------------MSKIYIVKSGDT-----LWGISKKHHISVKELARINSLSGRMI
H-----------------------------------------------------------------------
```

```
---------------------NLRIG-QKIYLQN------------------------------DVNNTNNF
E----TQLKIILMDLSFKPILKATIQLEFDGKKIIRNTKNSIFEDINIQDH-SKGLKVFFKNLN-GT-----
-FDL---------------------IADHKVLPLGRKVLKLT----SRKMKVEGSHYAKDGILNETVNQIMS
NLKKVGKP---------------------------------------------------IVESISTTSNKDE
LKNK--------QPPLKIPEEKIEQKRTDNGNSTHI-------------------------I--AAQFTEDN
FLLKPVNNKYRAYIVNAAKRHGFTPHSLAAVIEAEAAKIKKTGEWNTNSKANSSTAAGLTQFLDETWLAMCK
D---KSSLVGQYVM-------NNPKLTIQQKLNLRF---------------NAEMAIDAAAAYAISNFKSSG
LPYQKLT--EPSSIAKFAY----LLHH---------EGATGGKNFVLNTLSQERAKKLLFTQFGKNGAKQAA
DFLNRYKGDAKAAYGAWLRNYIDGHINIYQYVVDKSKTSGINLSTDETIKL---------------------
----------------------------------------------------------------------LK
GQTISTPAPK-------------------ISTTTNNQQVTNVS-----------------------------
-----------------TIEKSESKIRINTSQA---------------------------------------
PTNNVGGD---------------------------N------KWHNPLAD-CKLRTAGL-------------
-------ANAKGATFGKVRNNGTKNHQGVD-----LQANPGTK--IYAVCG--GVIAFAGATGGAYGKVIVL
KVDIND-----------------------LPEKQKKYAQTKLTKNKYVYFFYAHL------SVIDVDKGDPV
DTGEVIGKTGATGNANKMTTISKGAHLHFEARSAPLLGVGLDGRFDPIPFINANLPY---------------
----------------------------
>KAB04_18
-----------------------------MSKIYIVKSGDT-----LWGISKKHHISVKELARINSLSGRMI
H-----------------------------------------------------------------------
---------------------NLRIG-QKIYLQN------------------------------DVNNTNNF
E----TQLKIILMDLSFKPILKATIQLEFDGKKIIRNTKNSIFEDINIQDH-SKGLKVFFKNLN-GT-----
-FDL---------------------IADHKVLPLGRKVLKLT----SRKMKVEGSHYAKDGILNETVNQIMS
NLKKVGKP---------------------------------------------------IVESISTTSNKDE
LKNK--------QPPLKIPEEKIEQKRTDNGNSTHI-------------------------I--AAQFTEDN
FLLKPVNNKYRAYIVNAAKRHGFTPHSLAAVIEAEAAKIKKTGEWNTNSKANSSTAAGLTQFLDETWLAMCK
D---KSSLVGQYVM-------NNPKLTIQQKLNLRF---------------NAEMAIDAAAAYAISNFKSSG
LPYQKLT--EPSSIAKFAY----LLHH---------EGATGGKNFVLNTLSQERAKKLLFTQFGKNGAKQAA
DFLNRYKGDAKAAYGAWLRNYIDGHINIYQYVVDKSKTSGINLSTDETIKL---------------------
----------------------------------------------------------------------LK
GQTISTPAPK-------------------ISTTTNNQQVTNVS-----------------------------
-----------------TIEKSESKIRINTSQA---------------------------------------
PTNNVGGD---------------------------N------KWHNPLAD-CKLRTAGL-------------
```

-------ANAKGATFGKVRNNGTKNHQGVD-----LQANPGTK--IYAVCG--GVIAFAGATGGAYGKVIVL
KVDIND-----------------------LPEKQKKYAQTKLTKNKYVYFFYAHL------SVIDVDKGDPV
DTGEVIGKTGATGNANKMTTISKGAHLHFEARSAPLLGVGLDGRFDPIPFINANLPY---------------
----------------------------

>KAB05_18
-----------------------------MSKIYIVKSGDT-----LWGISKKHHISVKELARINSLSGRMI
H-----------------------------------------------------------------------
---------------------NLRIG-QKIYLQN------------------------------DVNNTNNF
E----TQLKIILMDLSFKPILKATIQLEFDGKKIIRNTKNSIFEDINIQDH-SKGLKVFFKNLN-GT-----
-FDL---------------------IADHKVLPLGRKVLKLT----SRKMKVEGSHYAKDGILNETVNQIMS
NLKKVGKP---------------------------------------------------IVESISTTSNKDE
LKNK--------QPPLKIPEEKIEQKRTDNGNSTHI-------------------------I--AAQFTEDN
FLLKPVNNKYRAYIVNAAKRHGFTPHSLAAVIEAEAAKIKKTGEWNTNSKANSSTAAGLTQFLDETWLAMCK
D---KSSLVGQYVM-------NNPKLTIQQKLNLRF---------------NAEMAIDAAAAYAISNFKSSG
LPYQKLT--EPSSIAKFAY----LLHH---------EGATGGKNFVLNTLSQERAKKLLFTQFGKNGAKQAA
DFLNRYKGDAKAAYGAWLRNYIDGHINIYQYVVDKSKTSGINLSTDETIKL---------------------
----------------------------------------------------------------------LK
GQTISTPAPK-------------------ISTTTNNQQVTNVS-----------------------------
-----------------TIEKSESKIRINTSQA---------------------------------------
PTNNVGGD---------------------------N------KWHNPLAD-CKLRTAGL-------------
-------ANAKGATFGKVRNNGTKNHQGVD-----LQANPGTK--IYAVCG--GVIAFAGATGGAYGKVIVL
KVDIND-----------------------LPEKQKKYAQTKLTKNKYVYFFYAHL------SVIDVDKGDPV
DTGEVIGKTGATGNANKMTTISKGAHLHFEARSAPLLGVGLDGRFDPIPFINANLPY---------------
----------------------------

>KAB06_18
-----------------------------MSKIYIVKSGDT-----LWGISKKHHISVKELARINSLSGRMI
H-----------------------------------------------------------------------
---------------------NLRIG-QKIYLQN------------------------------DVNNTNNF
E----TQLKIILMDLSFKPILKATIQLEFDGKKIIRNTKNSIFEDINIQDH-SKGLKVFFKNLN-GT-----
-FDL---------------------IADHKVLPLGRKVLKLT----SRKMKVEGSHYAKDGILNETVNQIMS
NLKKVGKP---------------------------------------------------IVESISTTSNKDE
LKNK--------QPPLKIPEEKIEQKRTDNGNSTHI-------------------------I--AAQFTEDN
FLLKPVNNKYRAYIVNAAKRHGFTPHSLAAVIEAEAAKIKKTGEWNTNSKANSSTAAGLTQFLDETWLAMCK

D---KSSLVGQYVM-------NNPKLTIQQKLNLRF---------------NAEMAIDAAAAYAISNFKSSG
LPYQKLT--EPSSIAKFAY----LLHH---------EGATGGKNFVLNTLSQERAKKLLFTQFGKNGAKQAA
DFLNRYKGDAKAAYGAWLRNYIDGHINIYQYVVDKSKTSGINLSTDETIKL---------------------
----------------------------------------------------------------------LK
GQTISTPAPK-------------------ISTTTNNQQVTNVS-----------------------------
-----------------TIEKSESKIRINTSQA---------------------------------------
PTNNVGGD---------------------------N------KWHNPLAD-CKLRTAGL-------------
-------ANAKGATFGKVRNNGTKNHQGVD-----LQANPGTK--IYAVCG--GVIAFAGATGGAYGKVIVL
KVDIND-----------------------LPEKQKKYAQTKLTKNKYVYFFYAHL------SVIDVDKGDPV
DTGEVIGKTGATGNANKMTTISKGAHLHFEARSAPLLGVGLDGRFDPIPFINANLPY---------------
----------------------------

>KAB07_18

-----------------------------MSKIYIVKSGDT-----LWGISKKHHISVKELARINSLSGRMI
H-----------------------------------------------------------------------
---------------------NLRIG-QKIYLQN------------------------------DVNNTNNF
E----TQLKIILMDLSFKPILKATIQLEFDGKKIIRNTKNSIFEDINIQDH-SKGLKVFFKNLN-GT-----
-FDL---------------------IADHKVLPLGRKVLKLT----SRKMKVEGSHYAKDGILNETVNQIMS
NLKKVGKP---------------------------------------------------IVESISTTSNKDE
LKNK--------QPPLKIPEEKIEQKRTDNGNSTHI-------------------------I--AAQFTEDN
FLLKPVNNKYRAYIVNAAKRHGFTPHSLAAVIEAEAAKIKKTGEWNTNSKANSSTAAGLTQFLDETWLAMCK
D---KSSLVGQYVM-------NNPKLTIQQKLNLRF---------------NAEMAIDAAAAYAISNFKSSG
LPYQKLT--EPSSIAKFAY----LLHH---------EGATGGKNFVLNTLSQERAKKLLFTQFGKNGAKQAA
DFLNRYKGDAKAAYGAWLRNYIDGHINIYQYVVDKSKTSGINLSTDETIKL---------------------
----------------------------------------------------------------------LK
GQTISTPAPK-------------------ISTTTNNQQVTNVS-----------------------------
-----------------TIEKSESKIRINTSQA---------------------------------------
PTNNVGGD---------------------------N------KWHNPLAD-CKLRTAGL-------------
-------ANAKGATFGKVRNNGTKNHQGVD-----LQANPGTK--IYAVCG--GVIAFAGATGGAYGKVIVL
KVDIND-----------------------LPEKQKKYAQTKLTKNKYVYFFYAHL------SVIDVDKGDPV
DTGEVIGKTGATGNANKMTTISKGAHLHFEARSAPLLGVGLDGRFDPIPFINANLPY---------------
----------------------------

>KAB08_18

-----------------------------MSKIYIVKSGDT-----LWGISKKHHISVKELARINSLSGRMI

```
H-----------------------------------------------------------------------
---------------------NLRIG-QKIYLQN------------------------------DVNNTNNF
E----TQLKIILMDLSFKPILKATIQLEFDGKKIIRNTKNSIFEDINIQDH-SKGLKVFFKNLN-GT-----
-FDL---------------------IADHKVLPLGRKVLKLT----SRKMKVEGSHYAKDGILNETVNQIMS
NLKKVGKP---------------------------------------------------IVESISTTSNKDE
LKNK--------QPPLKIPEEKIEQKRTDNGNSTHI-------------------------I--AAQFTEDN
FLLKPVNNKYRAYIVNAAKRHGFTPHSLAAVIEAEAAKIKKTGEWNTNSKANSSTAAGLTQFLDETWLAMCK
D---KSSLVGQYVM-------NNPKLTIQQKLNLRF---------------NAEMAIDAAAAYAISNFKSSG
LPYQKLT--EPSSIAKFAY----LLHH---------EGATGGKNFVLNTLSQERAKKLLFTQFGKNGAKQAA
DFLNRYKGDAKAAYGAWLRNYIDGHINIYQYVVDKSKTSGINLSTDETIKL---------------------
----------------------------------------------------------------------LK
GQTISTPAPK-------------------ISTTTNNQQVTNVS-----------------------------
-----------------TIEKSESKIRINTSQA---------------------------------------
PTNNVGGD---------------------------N------KWHNPLAD-CKLRTAGL-------------
-------ANAKGATFGKVRNNGTKNHQGVD-----LQANPGTK--IYAVCG--GVIAFAGATGGAYGKVIVL
KVDIND-----------------------LPEKQKKYAQTKLTKNKYVYFFYAHL------SVIDVDKGDPV
DTGEVIGKTGATGNANKMTTISKGAHLHFEARSAPLLGVGLDGRFDPIPFINANLPY---------------
----------------------------
>SAA14_18
-----------------------------MSKIYIVKSGDT-----LWGISKKHHISVKELARINSLSGRMI
H-----------------------------------------------------------------------
---------------------NLRIG-QKIYLQN------------------------------DVNNTNNF
E----TQLKIILMDLSFKPILKATIQLEFDGKKIIRNTKNSIFEDINIQDH-SKGLKVFFKNLN-GT-----
-FDL---------------------IADHKVLPLGRKVLKLT----SRKMKVEGSHYAKDGILNETVNQIMS
NLKKVGKP---------------------------------------------------IVESISTTSNKDE
LKNK--------QPPLKIPEEKIEQKRTDNGNSTHI-------------------------I--AAQFTEDN
FLLKPVNNKYRAYIVNAAKRHGFTPHSLAAVIEAEAAKIKKTGEWNTNSKANSSTAAGLTQFLDETWLAMCK
D---KSSLVGQYVM-------NNPKLTIQQKLNLRF---------------NAEMAIDAAAAYAISNFKSSG
LPYQKLT--EPSSIAKFAY----LLHH---------EGATGGKNFVLNTLSQERAKKLLFTQFGKNGAKQAA
DFLNRYKGDAKAAYGAWLRNYIDGHINIYQYVVDKSKTSGINLSTDETIKL---------------------
----------------------------------------------------------------------LK
GQTISTPAPK-------------------ISTTTNNQQVTNVS-----------------------------
-----------------TIEKSESKIRINTSQA---------------------------------------
```

```
PTNNVGGD---------------------------N------KWHNPLAD-CKLRTAGL-------------
-------ANAKGATFGKVRNNGTKNHQGVD-----LQANPGTK--IYAVCG--GVIAFAGATGGAYGKVIVL
KVDIND-----------------------LPEKQKKYAQTKLTKNKYVYFFYAHL------SVIDVDKGDPV
DTGEVIGKTGATGNANKMTTISKGAHLHFEARSAPLLGVGLDGRFDPIPFINANLPY---------------
----------------------------
>SSA12_18
-----------------------------MSKIYIVKSGDT-----LWGISKKHHISVKELARINSLSGRMI
H-----------------------------------------------------------------------
---------------------NLRIG-QKIYLQN------------------------------DVNNTNNF
E----TQLKIILMDLSFKPILKATIQLEFDGKKIIRNTKNSIFEDINIQDH-SKGLKVFFKNLN-GT-----
-FDL---------------------IADHKVLPLGRKVLKLT----SRKMKVEGSHYAKDGILNETVNQIMS
NLKKVGKP-------------------------------------------IVESISTTSNKDE
LKNK--------QPPLKIPEEKIEQKRTDNGNSTHI-------------------------I--AAQFTEDN
FLLKPVNNKYRAYIVNAAKRHGFTPHSLAAVIEAEAAKIKKTGEWNTNSKANSSTAAGLTQFLDETWLAMCK
D---KSSLVGQYVM-------NNPKLTIQQKLNLRF---------------NAEMAIDAAAAYAISNFKSSG
LPYQKLT--EPSSIAKFAY----LLHH---------EGATGGKNFVLNTLSQERAKKLLFTQFGKNGAKQAA
DFLNRYKGDAKAAYGAWLRNYIDGHINIYQYVVDKSKTSGINLSTDETIKL---------------------
----------------------------------------------------------------------LK
GQTISTPAPK-------------------ISTTTNNQQVTNVS-----------------------------
-----------------TIEKSESKIRINTSQA---------------------------------------
PTNNVGGD---------------------------N------KWHNPLAD-CKLRTAGL-------------
-------ANAKGATFGKVRNNGTKNHQGVD-----LQANPGTK--IYAVCG--GVIAFAGATGGAYGKVIVL
KVDIND-----------------------LPEKQKKYAQTKLTKNKYVYFFYAHL------SVIDVDKGDPV
DTGEVIGKTGATGNANKMTTISKGAHLHFEARSAPLLGVGLDGRFDPIPFINANLPY---------------
----------------------------
>SSMA17_18
-----------------------------MSKIYIVKSGDT-----LWGISKKHHISVKELARINSLSGRMI
H-----------------------------------------------------------------------
---------------------NLRIG-QKIYLQN------------------------------DVNNTNNF
E----TQLKIILMDLSFKPILKATIQLEFDGKKIIRNTKNSIFEDINIQDH-SKGLKVFFKNLN-GT-----
-FDL---------------------IADHKVLPLGRKVLKLT----SRKMKVEGSHYAKDGILNETVNQIMS
NLKKVGKP-------------------------------------------IVESISTTSNKDE
LKNK--------QPPLKIPEEKIEQKRTDNGNSTHI-------------------------I--AAQFTEDN
```

```
FLLKPVNNKYRAYIVNAAKRHGFTPHSLAAVIEAEAAKIKKTGEWNTNSKANSSTAAGLTQFLDETWLAMCK
D---KSSLVGQYVM-------NNPKLTIQQKLNLRF---------------NAEMAIDAAAAYAISNFKSSG
LPYQKLT--EPSSIAKFAY----LLHH---------EGATGGKNFVLNTLSQERAKKLLFTQFGKNGAKQAA
DFLNRYKGDAKAAYGAWLRNYIDGHINIYQYVVDKSKTSGINLSTDETIKL---------------------
----------------------------------------------------------------------LK
GQTISTPAPK-------------------ISTTTNNQQVTNVS-----------------------------
-----------------TIEKSESKIRINTSQA---------------------------------------
PTNNVGGD---------------------------N------KWHNPLAD-CKLRTAGL-------------
-------ANAKGATFGKVRNNGTKNHQGVD-----LQANPGTK--IYAVCG--GVIAFAGATGGAYGKVIVL
KVDIND-----------------------LPEKQKKYAQTKLTKNKYVYFFYAHL------SVIDVDKGDPV
DTGEVIGKTGATGNANKMTTISKGAHLHFEARSAPLLGVGLDGRFDPIPFINANLPY---------------
----------------------------
>XH386_18
-----------------------------MSKIYIVKSGDT-----LWGISKKHHISVKELARINSLSGRMI
H-----------------------------------------------------------------------
---------------------NLRIG-QKIYLQN------------------------------DVNNTNNF
E----TQLKIILMDLSFKPILKATIQLEFDGKKIIRNTKNSIFEDINIQDH-SKGLKVFFKNLN-GT-----
-FDL---------------------IADHKVLPLGRKVLKLT----SRKMKVEGSHYAKDGILNETVNQIMS
NLKKVGKP---------------------------------------------------IVESISTTSNKDE
LKNK--------QPPLKIPEEKIEQKRTDNGNSTHI-------------------------I--AAQFTEDN
FLLKPVNNKYRAYIVNAAKRHGFTPHSLAAVIEAEAAKIKKTGEWNTNSKANSSTAAGLTQFLDETWLAMCK
D---KSSLVGQYVM-------NNPKLTIQQKLNLRF---------------NAEMAIDAAAAYAISNFKSSG
LPYQKLT--EPSSIAKFAY----LLHH---------EGATGGKNFVLNTLSQERAKKLLFTQFGKNGAKQAA
DFLNRYKGDAKAAYGAWLRNYIDGHINIYQYVVDKSKTSGINLSTDETIKL---------------------
----------------------------------------------------------------------LK
GQTISTPAPK-------------------ISTTTNNQQVTNVS-----------------------------
-----------------TIEKSESKIRINTSQA---------------------------------------
PTNNVGGD---------------------------N------KWHNPLAD-CKLRTAGL-------------
-------ANAKGATFGKVRNNGTKNHQGVD-----LQANPGTK--IYAVCG--GVIAFAGATGGAYGKVIVL
KVDIND-----------------------LPEKQKKYAQTKLTKNKYVYFFYAHL------SVIDVDKGDPV
DTGEVIGKTGATGNANKMTTISKGAHLHFEARSAPLLGVGLDGRFDPIPFINANLPY---------------
----------------------------
>XH856_18
```

```
-----------------------------MSKIYIVKSGDT-----LWGISKKHHISVKELARINSLSGRMI
H-----------------------------------------------------------------------
--------------------NLRIG-QKIYLQN------------------------------DVNNTNNF
E----TQLKIILMDLSFKPILKATIQLEFDGKKIIRNTKNSIFEDINIQDH-SKGLKVFFKNLN-GT-----
-FDL---------------------IADHKVLPLGRKVLKLT----SRKMKVEGSHYAKDGILNETVNQIMS
NLKKVGKP-------------------------------------------------IVESISTTSNKDE
LKNK--------QPPLKIPEEKIEQKRTDNGNSTHI-------------------------I--AAQFTEDN
FLLKPVNNKYRAYIVNAAKRHGFTPHSLAAVIEAEAAKIKKTGEWNTNSKANSSTAAGLTQFLDETWLAMCK
D---KSSLVGQYVM-------NNPKLTIQQKLNLRF---------------NAEMAIDAAAAYAISNFKSSG
LPYQKLT--EPSSIAKFAY----LLHH---------EGATGGKNFVLNTLSQERAKKLLFTQFGKNGAKQAA
DFLNRYKGDAKAAYGAWLRNYIDGHINIYQYVVDKSKTSGINLSTDETIKL---------------------
----------------------------------------------------------------------LK
GQTISTPAPK-------------------ISTTTNNQQVTNVS-----------------------------
-----------------TIEKSESKIRINTSQA---------------------------------------
PTNNVGGD---------------------------N------KWHNPLAD-CKLRTAGL-------------
-------ANAKGATFGKVRNNGTKNHQGVD-----LQANPGTK--IYAVCG--GVIAFAGATGGAYGKVIVL
KVDIND-----------------------LPEKQKKYAQTKLTKNKYVYFFYAHL------SVIDVDKGDPV
DTGEVIGKTGATGNANKMTTISKGAHLHFEARSAPLLGVGLDGRFDPIPFINANLPY---------------
---------------------------
>3207_4
-----------------------------MSNIHIVKKGDT-----LWGISNKYHIKLNELIEINGLYGRKK
N-----------------------------------------------------------------------
--------------------LLKIG-QQIYLKK--------------------------------DNVKKY
D----TTLVIKIYDLKWEPITHGKLLLEYDDKCHLATSNDKGVIEDIQIEDALKGIKISFYTLK-NK-----
-FEL---------------------IAHHKTLPLGKKILKLS----SRAMMIKGNTYKVEGIPRTNTKTIEK
ELKVNSKKVQHSSGASGASGASGASGASGASGASGASGASGASGASGASGASGASGASGASGASGASGASGA
SGASG-------ASGASGASGASETRIEGGLPAVTV-----------------------------APVYSEEN
LYLHPDNEKYRKIIIDAAKKYNLAPQALAAKINAEAGTKKGSQEWNPQAQASTSSAVGLTQFLSGTWYEICT
VSTYKDTLLQQYVI-------KKKLISNFKENMALKLISNTDKAKLKALGTDPTFSVDSAAAYARANINIVK
KIYSKIDSLEPGDLAKVGY----VAHHD--------GPTGFGKIVEATNDPSWSKLQGQVCPKNNPNCDTYL
NYKERFSNGRDAYRWWLCTEYTDSKINVNRFTLSPKDGKKFKNARTMEEIIVF-------------------
----------------------------------------------------------------------LG
GKPLVRPSRDFEKKGTTDNSKLNDNINIVITSLVNNKVQANIS-----------------------------
```

-----------------YFVKSKYGLKPHSTNVNGHEIIHAKKGDKIEILFNDQVIASINAINDKEEFKINF
PSKNITNA------------STEKQSSNSEQKLNFN------RWRNPLNGICQIRRFGY-NSLPLKANASSY
NEKNLEQATKIASRFNRTSYRASGIHQGVD-----LEADNGVN--IYPVCV--GTIAKVIPAYPGYGKTIIL
ECDVND--------------------------LPSSKKVLAKNLDTIYFIYAHLN------SINVSEGDIIT
TLDTVLGKTGNSGNAGSMTRIEDGSHLHFEVRSEIMKSMGKSGMSYRLDPFPWLDNCMTTENGVKVNR----
----------------------------
>ATCC17978-mff_4
-----------------------------MSNIHIVKKGDT-----LWGISNKYHIKLNELIEINGLYGRKK
N-----------------------------------------------------------------------
---------------------LLKIG-QQIYLKK--------------------------------DNVKKY
D----TTLVIKIYDLKWEPITHGKLLLEYDDKCHLATSNDKGVIEDIQIEDALKGIKISFYTLK-NK-----
-FEL---------------------IAHHKTLPLGKKILKLS----SRAMMIKGNTYKVEGIPRTNTKTIEK
ELKVNSKKVQHSSGASGASGASGASGASGASGASGASGASG-------------------------------
------------ASGASGASGASETRIEGGLPAVTV----------------------------APVYSEEN
LYLHPDNEKYRKIIIDAAKKYNLAPQALAAKINAEAGTKKGSQEWNPQAQASTSSAVGLTQFLSGTWYEICT
VSTYKDTLLQQYVI-------KKKLISNFKENMALKLISNTDKAKLKALGTDPTFSVDSAAAYARANINIVK
KIYSKIDSLEPGDLAKVGY----VAHHD--------GPTGFGKIVEATNDPSWSKLQGQVCPKNNPNCDTYL
NYKERFSNGRDAYRWWLCTEYTDSKINVNRFTLSPKDGKKFKNARTMEEIIVF-------------------
----------------------------------------------------------------------LG
GKPLVRPSRDFEKKGTTDNSKLNDNINIVITSLVNNKVQANIS-----------------------------
-----------------YFVKSKYGLKPHSTNVNGHEIIHAKKGDKIEILFNDQVIASINAINDKEEFKINF
PSKNITNA------------STEKQSSNSEQKLNFN------RWRNPLNGICQIRRFGY-NSLPLKANASSY
NEKNLEQATKIASRFNRTSYRASGIHQGVD-----LEADNGVN--IYPVCV--GTIAKVIPAYPGYGKTIIL
ECDVND--------------------------LPSSKKVLAKNLDTIYFIYAHLN------SINVSEGDSIT
TLDTVLGKTGNSGNAGSMTRIEDGSHLHFEVRSEIMKSMGKSGMSYRLDPFPWLDNCMTTENGVKVNR----
----------------------------
>6200_15
--------------------------------NSFNYGEMF-----GIPASAQSG-----------------
------------------------------------------------------------------------
---------------------LAYQG-QRNYECY------------------------------AETGELCK
I--------------KVPPLFDYVAC--------------------------SGGGLGI------GV-----
--------------------------GFVKNQWTGEYYISGS------------------------------
------------------------------------------------------------------------

```
------------KDSLLIPVAKSVAQNKQFSAKDL-----------------------------AGASCVGG
NIHN-IPSYTKTTMTMGEITNEFVSGAS--------------------------------------------
----VTVGGGAYGA----------------------------------------------VANVVVPLVSKS
SP----------------------------------------------------------------------
------------------------------------------------------------------------
----------------------------------------------------------------------VK
GTWASELGVG--------------------------------------------------------------
---------------TPGFNVGVSGTVSVDTIL---------------------------------------
---------------------------------------------DAVKPSKK-------------------
------------------------------------------------------------------------
------------------------------------------------------------------------
------------------------------------------------------------------------
----------------------------
>A1_15
--------------------------------NSFNYGEMF-----GIPASAQSG-----------------
------------------------------------------------------------------------
---------------------LAYQG-QRNYECY------------------------------AETGELCK
I--------------KVPPLFDYVAC--------------------------SGGGLGI------GV-----
--------------------------GFVKNQWTGEYYISGS------------------------------
------------------------------------------------------------------------
------------KDSLLIPVAKSVAQNKQFSAKDL-----------------------------AGASCVGG
NIHN-IPSYTKTTMTMGEITNEFVSGAS--------------------------------------------
----VTVGGGAYGA----------------------------------------------VANVVVPLVSKS
SP----------------------------------------------------------------------
------------------------------------------------------------------------
----------------------------------------------------------------------VK
GTWASELGVG--------------------------------------------------------------
---------------TPGFNVGVSGTVSVDTIL---------------------------------------
---------------------------------------------DAVKPSKK-------------------
------------------------------------------------------------------------
------------------------------------------------------------------------
------------------------------------------------------------------------
----------------------------
```

```
>A388_15
--------------------------------NSFNYGEMF-----GIPASAQSG-----------------
------------------------------------------------------------------------
---------------------LAYQG-QRNYECY------------------------------AETGELCK
I--------------KVPPLFDYVAC--------------------------SGGGLGI------GV-----
--------------------------GFVKNQWTGEYYISGS------------------------------
------------------------------------------------------------------------
------------KDSLLIPVAKSVAQNKQFSAKDL-----------------------------AGASCVGG
NIHN-IPSYTKTTMTMGEITNEFVSGAS--------------------------------------------
----VTVGGGAYGA----------------------------------------------VANVVVPLVSKS
SP----------------------------------------------------------------------
------------------------------------------------------------------------
----------------------------------------------------------------------VK
GTWASELGVG--------------------------------------------------------------
---------------TPGFNVGVSGTVSVDTIL---------------------------------------
---------------------------------------------DAVKPSKK-------------------
------------------------------------------------------------------------
------------------------------------------------------------------------
------------------------------------------------------------------------
----------------------------
>A85_15
--------------------------------NSFNYGEMF-----GIPASAQSG-----------------
------------------------------------------------------------------------
---------------------LAYQG-QRNYECY------------------------------AETGELCK
I--------------KVPPLFDYVAC--------------------------SGGGLGI------GV-----
--------------------------GFVKNQWTGEYYISGS------------------------------
------------------------------------------------------------------------
------------KDSLLIPVAKSVAQNKQFSAKDL-----------------------------AGASCVGG
NIHN-IPSYTKTTMTMGEITNEFVSGAS--------------------------------------------
----VTVGGGAYGA----------------------------------------------VANVVVPLVSKS
SP----------------------------------------------------------------------
------------------------------------------------------------------------
----------------------------------------------------------------------VK
```

```
GTWASELGVG--------------------------------------------------------------
---------------TPGFNVGVSGTVSVDTIL---------------------------------------
---------------------------------------------DAVKPSKK-------------------
------------------------------------------------------------------------
------------------------------------------------------------------------
------------------------------------------------------------------------
----------------------------
>AB0057_15
--------------------------------NSFNYGEMF-----GIPASAQSG-----------------
------------------------------------------------------------------------
---------------------LAYQG-QRNYECY------------------------------AETGELCK
I--------------KVPPLFDYVAC--------------------------SGGGLGI------GV-----
--------------------------GFVKNQWTGEYYISGS------------------------------
------------------------------------------------------------------------
------------KDSLLIPVAKSVAQNKQFSAKDL-----------------------------AGASCVGG
NIHN-IPSYTKTTMTMGEITNEFVSGAS--------------------------------------------
----VTVGGGAYGA----------------------------------------------VANVVVPLVSKS
SP----------------------------------------------------------------------
------------------------------------------------------------------------
----------------------------------------------------------------------VK
GTWASELGVG--------------------------------------------------------------
---------------TPGFNVGVSGTVSVDTIL---------------------------------------
---------------------------------------------DAVKPSKK-------------------
------------------------------------------------------------------------
------------------------------------------------------------------------
------------------------------------------------------------------------
----------------------------
>AB030_15
--------------------------------NSFNYGEMF-----GIPASAQSG-----------------
------------------------------------------------------------------------
---------------------LAYQG-QRNYECY------------------------------AETGELCK
I--------------KVPPLFDYVAC--------------------------SGGGLGI------GV-----
--------------------------GFVKNQWTGEYYISGS------------------------------
```

------------------------------------------------------------------------
------------KDSLLIPVAKSVAQNKQFSAKDL-----------------------------AGASCVGG
NIHN-IPSYTKTTMTMGEITNEFVSGAS--------------------------------------------
----VTVGGGAYGA----------------------------------------------VANVVVPLVSKS
SP----------------------------------------------------------------------
------------------------------------------------------------------------
----------------------------------------------------------------------VK
GTWASELGVG--------------------------------------------------------------
---------------TPGFNVGVSGTVSVDTIL---------------------------------------
---------------------------------------------DAVKPSKK-------------------
------------------------------------------------------------------------
------------------------------------------------------------------------
------------------------------------------------------------------------
----------------------------
>AB307-0294_15
--------------------------------NSFNYGEMF-----GIPASAQSG-----------------
------------------------------------------------------------------------
---------------------LAYQG-QRNYECY------------------------------AETGELCK
I--------------KVPPLFDYVAC--------------------------SGGGLGI------GV-----
--------------------------GFVKNQWTGEYYISGS------------------------------
------------------------------------------------------------------------
------------KDSLLIPVAKSVAQNKQFSAKDL-----------------------------AGASCVGG
NIHN-IPSYTKTTMTMGEITNEFVSGAS--------------------------------------------
----VTVGGGAYGA----------------------------------------------VANVVVPLVSKS
SP----------------------------------------------------------------------
------------------------------------------------------------------------
----------------------------------------------------------------------VK
GTWASELGVG--------------------------------------------------------------
---------------TPGFNVGVSGTVSVDTIL---------------------------------------
---------------------------------------------DAVKPSKK-------------------
------------------------------------------------------------------------
------------------------------------------------------------------------
------------------------------------------------------------------------

```
----------------------------
>AB5075-UW_15
--------------------------------NSFNYGEMF-----GIPASAQSG-----------------
------------------------------------------------------------------------
---------------------LAYQG-QRNYECY------------------------------AETGELCK
I--------------KVPPLFDYVAC--------------------------SGGGLGI------GV-----
--------------------------GFVKNQWTGEYYISGS------------------------------
------------------------------------------------------------------------
------------KDSLLIPVAKSVAQNKQFSAKDL-----------------------------AGASCVGG
NIHN-IPSYTKTTMTMGEITNEFVSGAS--------------------------------------------
----VTVGGGAYGA----------------------------------------------VANVVVPLVSKS
SP----------------------------------------------------------------------
------------------------------------------------------------------------
----------------------------------------------------------------------VK
GTWASELGVG--------------------------------------------------------------
---------------TPGFNVGVSGTVSVDTIL---------------------------------------
---------------------------------------------DAVKPSKK-------------------
------------------------------------------------------------------------
------------------------------------------------------------------------
------------------------------------------------------------------------
----------------------------
>AbH12O-A2_15
--------------------------------NSFNYGEMF-----GIPASAQSG-----------------
------------------------------------------------------------------------
---------------------LAYQG-QRNYECY------------------------------AETGELCK
I--------------KVPPLFDYVAC--------------------------SGGGLGI------GV-----
--------------------------GFVKNQWTGEYYISGS------------------------------
------------------------------------------------------------------------
------------KDSLLIPVAKSVAQNKQFSAKDL-----------------------------AGASCVGG
NIHN-IPSYTKTTMTMGEITNEFVSGAS--------------------------------------------
----VTVGGGAYGA----------------------------------------------VANVVVPLVSKS
SP----------------------------------------------------------------------
------------------------------------------------------------------------
```

----------------------------------------------------------------------VK
GTWASELGVG--------------------------------------------------------------
---------------TPGFNVGVSGTVSVDTIL---------------------------------------
---------------------------------------------DAVKPSKK-------------------
------------------------------------------------------------------------
------------------------------------------------------------------------
------------------------------------------------------------------------
----------------------------
>AF-401_15
--------------------------------NSFNYGEMF-----GIPASAQSG-----------------
------------------------------------------------------------------------
---------------------LAYQG-QRNYECY------------------------------AETGELCK
I--------------KVPPLFDYVAC--------------------------SGGGLGI------GV-----
--------------------------GFVKNQWTGEYYISGS------------------------------
------------------------------------------------------------------------
------------KDSLLIPVAKSVAQNKQFSAKDL-----------------------------AGASCVGG
NIHN-IPSYTKTTMTMGEITNEFVSGAS--------------------------------------------
----VTVGGGAYGA----------------------------------------------VANVVVPLVSKS
SP----------------------------------------------------------------------
------------------------------------------------------------------------
----------------------------------------------------------------------VK
GTWASELGVG--------------------------------------------------------------
---------------TPGFNVGVSGTVSVDTIL---------------------------------------
---------------------------------------------DAVKPSKK-------------------
------------------------------------------------------------------------
------------------------------------------------------------------------
------------------------------------------------------------------------
----------------------------
>AR_0063_15
--------------------------------NSFNYGEMF-----GIPASAQSG-----------------
------------------------------------------------------------------------
---------------------LAYQG-QRNYECY------------------------------AETGELCK
I--------------KVPPLFDYVAC--------------------------SGGGLGI------GV-----

--------------------------GFVKNQWTGEYYISGS------------------------------
------------------------------------------------------------------------
------------KDSLLIPVAKSVAQNKQFSAKDL-----------------------------AGASCVGG
NIHN-IPSYTKTTMTMGEITNEFVSGAS--------------------------------------------
----VTVGGGAYGA----------------------------------------------VANVVVPLVSKS
SP----------------------------------------------------------------------
------------------------------------------------------------------------
----------------------------------------------------------------------VK
GTWASELGVG--------------------------------------------------------------
---------------TPGFNVGVSGTVSVDTIL---------------------------------------
---------------------------------------------DAVKPSKK-------------------
------------------------------------------------------------------------
------------------------------------------------------------------------
------------------------------------------------------------------------
----------------------------
>AR_0078_15
--------------------------------NSFNYGEMF-----GIPASAQSG-----------------
------------------------------------------------------------------------
---------------------LAYQG-QRNYECY------------------------------AETGELCK
I--------------KVPPLFDYVAC--------------------------SGGGLGI------GV-----
--------------------------GFVKNQWTGEYYISGS------------------------------
------------------------------------------------------------------------
------------KDSLLIPVAKSVAQNKQFSAKDL-----------------------------AGASCVGG
NIHN-IPSYTKTTMTMGEITNEFVSGAS--------------------------------------------
----VTVGGGAYGA----------------------------------------------VANVVVPLVSKS
SP----------------------------------------------------------------------
------------------------------------------------------------------------
----------------------------------------------------------------------VK
GTWASELGVG--------------------------------------------------------------
---------------TPGFNVGVSGTVSVDTIL---------------------------------------
---------------------------------------------DAVKPSKK-------------------
------------------------------------------------------------------------
------------------------------------------------------------------------

```
------------------------------------------------------------------------
----------------------------
>AR_0083_15
--------------------------------NSFNYGEMF-----GIPASAQSG-----------------
------------------------------------------------------------------------
---------------------LAYQG-QRNYECY------------------------------AETGELCK
I--------------KVPPLFDYVAC--------------------------SGGGLGI------GV-----
--------------------------GFVKNQWTGEYYISGS------------------------------
------------------------------------------------------------------------
------------KDSLLIPVAKSVAQNKQFSAKDL-----------------------------AGASCVGG
NIHN-IPSYTKTTMTMGEITNEFVSGAS--------------------------------------------
----VTVGGGAYGA----------------------------------------------VANVVVPLVSKS
SP----------------------------------------------------------------------
------------------------------------------------------------------------
----------------------------------------------------------------------VK
GTWASELGVG--------------------------------------------------------------
---------------TPGFNVGVSGTVSVDTIL---------------------------------------
---------------------------------------------DAVKPSKK-------------------
------------------------------------------------------------------------
------------------------------------------------------------------------
------------------------------------------------------------------------
----------------------------
>AR_0101_15
--------------------------------NSFNYGEMF-----GIPASAQSG-----------------
------------------------------------------------------------------------
---------------------LAYQG-QRNYECY------------------------------AETGELCK
I--------------KVPPLFDYVAC--------------------------SGGGLGI------GV-----
--------------------------GFVKNQWTGEYYISGS------------------------------
------------------------------------------------------------------------
------------KDSLLIPVAKSVAQNKQFSAKDL-----------------------------AGASCVGG
NIHN-IPSYTKTTMTMGEITNEFVSGAS--------------------------------------------
----VTVGGGAYGA----------------------------------------------VANVVVPLVSKS
SP----------------------------------------------------------------------
```

------------------------------------------------------------------------
----------------------------------------------------------------------VK
GTWASELGVG--------------------------------------------------------------
---------------TPGFNVGVSGTVSVDTIL---------------------------------------
---------------------------------------------DAVKPSKK-------------------
------------------------------------------------------------------------
------------------------------------------------------------------------
------------------------------------------------------------------------
----------------------------
>D36_15
--------------------------------NSFNYGEMF-----GIPASAQSG-----------------
------------------------------------------------------------------------
---------------------LAYQG-QRNYECY------------------------------AETGELCK
I--------------KVPPLFDYVAC--------------------------SGGGLGI------GV-----
--------------------------GFVKNQWTGEYYISGS------------------------------
------------------------------------------------------------------------
------------KDSLLIPVAKSVAQNKQFSAKDL-----------------------------AGASCVGG
NIHN-IPSYTKTTMTMGEITNEFVSGAS--------------------------------------------
----VTVGGGAYGA----------------------------------------------VANVVVPLVSKS
SP----------------------------------------------------------------------
------------------------------------------------------------------------
----------------------------------------------------------------------VK
GTWASELGVG--------------------------------------------------------------
---------------TPGFNVGVSGTVSVDTIL---------------------------------------
---------------------------------------------DAVKPSKK-------------------
------------------------------------------------------------------------
------------------------------------------------------------------------
------------------------------------------------------------------------
----------------------------
>IOMTU433_15
--------------------------------NSFNYGEMF-----GIPASAQSG-----------------
------------------------------------------------------------------------
---------------------LAYQG-QRNYECY------------------------------AETGELCK

```
I--------------KVPPLFDYVAC--------------------------SGGGLGI------GV-----
--------------------------GFVKNQWTGEYYISGS------------------------------
------------------------------------------------------------------------
------------KDSLLIPVAKSVAQNKQFSAKDL-----------------------------AGASCVGG
NIHN-IPSYTKTTMTMGEITNEFVSGAS--------------------------------------------
----VTVGGGAYGA----------------------------------------------VANVVVPLVSKS
SP----------------------------------------------------------------------
------------------------------------------------------------------------
----------------------------------------------------------------------VK
GTWASELGVG--------------------------------------------------------------
---------------TPGFNVGVSGTVSVDTIL---------------------------------------
---------------------------------------------DAVKPSKK-------------------
------------------------------------------------------------------------
------------------------------------------------------------------------
------------------------------------------------------------------------
----------------------------
>USA15_15
--------------------------------NSFNYGEMF-----GIPASAQSG-----------------
------------------------------------------------------------------------
---------------------LAYQG-QRNYECY------------------------------AETGELCK
I--------------KVPPLFDYVAC--------------------------SGGGLGI------GV-----
--------------------------GFVKNQWTGEYYISGS------------------------------
------------------------------------------------------------------------
------------KDSLLIPVAKSVAQNKQFSAKDL-----------------------------AGASCVGG
NIHN-IPSYTKTTMTMGEITNEFVSGAS--------------------------------------------
----VTVGGGAYGA----------------------------------------------VANVVVPLVSKS
SP----------------------------------------------------------------------
------------------------------------------------------------------------
----------------------------------------------------------------------VK
GTWASELGVG--------------------------------------------------------------
---------------TPGFNVGVSGTVSVDTIL---------------------------------------
---------------------------------------------DAVKPSKK-------------------
------------------------------------------------------------------------
```

------------------------------------------------------------------------
------------------------------------------------------------------------
----------------------------
>WCHAB005078_15
--------------------------------NSFNYGEMF-----GIPASAQSG-----------------
------------------------------------------------------------------------
---------------------LAYQG-QRNYECY------------------------------AETGELCK
I--------------KVPPLFDYVAC--------------------------SGGGLGI------GV-----
--------------------------GFVKNQWTGEYYISGS------------------------------
------------------------------------------------------------------------
------------KDSLLIPVAKSVAQNKQFSAKDL-----------------------------AGASCVGG
NIHN-IPSYTKTTMTMGEITNEFVSGAS--------------------------------------------
----VTVGGGAYGA----------------------------------------------VANVVVPLVSKS
SP----------------------------------------------------------------------
------------------------------------------------------------------------
----------------------------------------------------------------------VK
GTWASELGVG--------------------------------------------------------------
---------------TPGFNVGVSGTVSVDTIL---------------------------------------
---------------------------------------------DAVKPSKK-------------------
------------------------------------------------------------------------
------------------------------------------------------------------------
------------------------------------------------------------------------
----------------------------
>SDF_15
--------------------------------NSFNYGEMF-----GIPASAQSG-----------------
------------------------------------------------------------------------
---------------------LAYQG-QRNYECY------------------------------AETGELCK
I--------------KVPPLFDYVAC--------------------------SGGGLGI------GV-----
--------------------------GFVKNQWTGEYYISGS------------------------------
------------------------------------------------------------------------
------------KDSLLIPVAKSVAQNKQFSAKDL-----------------------------AGASCVGG
NIHN-IPSYTKTTMAMGEITNEFVSGAS--------------------------------------------
----VTVGGGAYGA----------------------------------------------VANVVVPLVSKS

```
SP----------------------------------------------------------------------
------------------------------------------------------------------------
----------------------------------------------------------------------VK
GTWASELGVG--------------------------------------------------------------
---------------TPGFNVGVSGTVSVDTIL---------------------------------------
---------------------------------------------DAVKPSKK-------------------
------------------------------------------------------------------------
------------------------------------------------------------------------
------------------------------------------------------------------------
----------------------------
>AB031_26
----------------------QPVGGKKSGLGIGFLFPNT-----MAGFAKNPEDARNLR-----------
------------------------------------------------------------------------
---------------------ALGAIRNNNLAAA------------------------------SVAGYGPY
VVAGTAAAGSAAAPVVGRVAAPFI----------------------------GTGAQLEGAGLTAGT-----
---------------------VGKAAVTSAGIGGGMDVISQG----VKCKCLKDINLVR-------------
------------------------------------------------------------------------
------------TGGVAAVSAVTSGWGATTSAAA------------------------------GFGQVGWK
SIALNAPNMGQFL-----------------------------------------------------------
----KNNASGMVIF----------------------------------------------------------
------------------------------------------------------------------------
------------------------------------------------------------------------
----------------------------------------------------------------------AN
EQMIGQAG----------------------------------------------------------------
-----------------SRAVKAATEKDKSSKK---------------------------------------
------------------------------------------------------------------------
------------------------------------------------------------------------
------------------------------------------------------------------------
------------------------------------------------------------------------
----------------------------
>ZW85-1_20
MDKKMSENQKMILGKSKSNNLFNEAIAQQKQKQSQIPVMGK-----TKAVGDMRNPTVAKNINKKIEESEIA
K-----------------------SAKQSTAANMQNSSAGQAQKECSNFCRKKGFNILPLRYTVVKDNPPAL
```

PPTLGKNVKDIQLSHLKYAVEMINTG-YIYRLVK------------------------------RTSGALEW
A----GYKVTPNGHLSYFPVGKEAPK--------------------------SVPEFACKGAGH-SFNSSVI
AVESNPKDEAKIAYIIHTHVPMSKAKISEYEKNADKFVTEGK----WQKILINGGSAQEHCIAASQFKTTVY
NLNSFGKN----------------------------------------------RIDATLNKFKDEPKSLA
CMAL--------YDPVGITRKLNTLRNSEFKKVMDF------------------------------LAKKDGDF
TNEHRLHSSQCIDSIKICLENKLLNERS--QFSAQATKSIVLMGLWNELPKNLTDADDINYELEKIYKRKSK
AEQERLLKEAQEKQRANKGIYTEDMGKLSRGEGEQIWVKKYKPNLDMVAKSNFDKKINALIQECLAGARAIG
DDHIKWLLSKAVINALHAYDDQVQVPFGNIFYVHAMDMINGMSGVPSGQALLETWLGVEKIAKENIYMRAIC
YNQESLKNKYNAAAPSIIDISWDDGQNAAKTAIAAFVAADKAWQEWAADKTNNGYM----------------
----------------------------------------------------------------------LH
GQKFSLLKTF-------------YWMSELTQSAVKWSMKYTPGSEFAKKLGRLTFYTFAHSGALVEHVAKNS
LYYLIPVQTLTGRVKLTPEMVQNNWKKYSSSAT------------------------------------NP
SKAAKQID-----------------------------------RIIANSSPSAGLRVSGVVAIFELINLGTQI
-------SKFNIDPSVDNTLSYMSGMMATTAAILEVGSSSMEKFSFAERAAYIAKCGAYFAIAGAIVSFFID
LKGLNKAFEESNGYAFALNLLKAASSFGTLAVGFSAFLNLPIMRNSTLYSMVERYAVGRALLGIKVVRVGAM
LSLAGIALVALEIYLKNYVLDNAMQDWCQKCAFKLKKENGEDAFKDVKEEEKEFNNAVVSV-----------
----------------------------

>D1279779_12
----MSENQKMILGKSKSNNLLNEIIAQQKQKQSQIPVIGK-----TKAVGDMRNPTVAKNINKKIEESEIA
K-----------------------SAKQSTAANMQNSSAGQAQKECSNFCRKKGFNILPLRYTVVKDNPPAL
PATLGKNVKDIQLSHLKYAVEMIDTG-YIYRLVK------------------------------RTSGALEW
A----GYKVTPNGHLSYFPVGKEAPK--------------------------SVPEFACKGAGH-SFNSSVI
AVESNPKDEAKIAYIIHTHVPMSKAKISEYEKNADKFVTEGK----WQKILINGGSAQEHCIAASQFKTTVY
NLNSFGKN----------------------------------------------RIDATLNKFKDEPKSLA
CMAL--------YDPVGITRKLNDSRNSQFGKLTNY------------------------------LAKNVEKI
SNEHRLQSSQLVDSIKIVVENKLINQQLSARSQVADLSAKIEYAKDKIPKFGSMDQREYLSKIQEIWDRAPT
AERDKFLLQRNKKF-------AEGEQKLKEKIITQS----------------------KAEAKITWETKYAP
KI-------NWDTKKAFDK----EVKK---------LINEGRTLAQTYASDHIKWLKSKHLLNAFYVYDQTI
LKEYGALFHVHALAVMDGMTGCDEGQALMEKWLGVEEISDDNLYMRAVTYNQKNLIDQYNAKSSEITSLTWD
QTQSSLKSLIAAFVSADQ---------------------------------------------------LW
EQWLADPNNKINELKWYNPAKSLLWISEITRSVAKWSLQYNINPLIAKRLSRVSFIAYAHSSLLTDKVPKWS
LLYNVDFKMTNASTQLNVTEIERRWKNAGQNTA---------------------------------------
AQKIKEILSKSPASVAIRISSIVALFELINFGVQSNKFIGERNWENGFQLISGLLASSA-AVLDIAGGGMDA

LSFQEKANKIRLYGARLAVSAAIISFCIDAFAVYKEKEGDKDKILMAFLFTKATVSLVMVGVGFGLLLNLPI
LQNSWV---------------VSSANKIAFTRILLRANFIRIAAYCNWIGIGFLV------IEYLLKTYVLD
DDLQKWCEKSIFGKDSSKFKLFKDEEEAFSKAIMSI------------------------------------
----------------------------
>R2090_12
----MSENQKMILGKSKSNNLLNEIIAQQKQKQSQIPVIGK-----TKAVGDMRNPTVAKNINKKIEESEIA
K-----------------------SAKQSTAANMQNSSAGQAQKECSNFCRKKGFNILPLRYTVVKDNPPAL
PATLGKNVKDIQLSHLKYAVEMIDTG-YIYRLVK------------------------------RTSGALEW
A----GYKVTPNGHLSYFPVGKEAPK--------------------------SVPEFACKGAGH-SFNSSVI
AVESNPKDEAKIAYIIHTHVPMSKAKISEYEKNADKFVTEGK----WQKILINGGSAQEHCIAASQFKTTVY
NLNSFGKN-----------------------------------------------RIDATLNKFKDEPKSLA
CMAL--------YDPVGITRKLNDSRNSQFGKLTNY----------------------------LAKNVEKI
SNEHRLQSSQLVDSIKIVVENKLINQQLSARSQVADLSAKIEYAKDKIPKFGSMDQREYLSKIQEIWDRAPT
AERDKFLLQRNKKF-------AEGEQKLKEKIITQS----------------------KAEAKITWETKYAP
KI-------NWDTKKAFDK----EVKK---------LINEGRTLAQTYASDHIKWLKSKHLLNAFYVYDQTI
LKEYGALFHVHALAVMDGMTGCDEGQALMEKWLGVEEISDDNLYMRAVTYNQKNLIDQYNAKSSEITSLTWD
QTQSSLKSLIAAFVSADQ----------------------------------------------------LW
EQWLADPNNKINELKWYNPAKSLLWISEITRSVAKWSLQYNINPLIAKRLSRVSFIAYAHSSLLTDKVPKWS
LLYNVDFKMTNASTQLNVTEIERRWKNAGQNTA---------------------------------------
AQKIKEILSKSPASVAIRISSIVALFELINFGVQSNKFIGERNWENGFQLISGLLASSA-AVLDIAGGGMDA
LSFQEKANKIRLYGARLAVSAAIISFCIDAFAVYKEKEGDKDKILMAFLFTKATVSLVMVGVGFGLLLNLPI
LQNSWV---------------VSSANKIAFTRILLRANFIRIAAYCNWIGIGFLV------IEYLLKTYVLD
DDLQKWCEKSIFGKDSSKFKLFKDEEEAFSKAIMSI------------------------------------
----------------------------
>Ab04-mff_12
----MSENQKMILGKSKSNNLFNEAIAQQKQKQSQIPVMGK-----TKAVGDMRNPTVAKNINKKIEESEIA
K-----------------------SAKQSTAANMQNSSAGQAQKECSNFCRKKGFNILPLRYTVVKDNPPAL
PPTLGKNVKDIQLSHLKYAVEMINTG-YIYRLVK------------------------------RTSGALEW
A----GYKVTPNGHLSYFPVGKEAPK--------------------------SVPEFACKGAGH-SFNSSVI
AVESNPKDEAKIAYIIHTHVPMSKAKISEYEKNADKFVTEGK----WQKILINGGSAQEHCIAASQFKTTVY
NLNSFGKN-----------------------------------------------RIDATLNKFKDEPKSLA
CMAL--------YDPVGITRKLNDSRNSQFGKLTNY----------------------------LAKNVEKI
SNEHRLQSSQLVDSIKIVVENKLINQQLSARSQIADLSAKIEYAKDKIPKFGSMDQREYLSKIQEIWDRAPT

AERDKFLLQRNKKF-------AEGEQKLKEKIITQS----------------------KAEAKITWETKYAP
KI-------NWDTKKAFDK----EVKK---------LINEGRTLAQTYASDHMKWLKSKHLLNAFYVYDQTI
LKEYGALFHVHALAVMDGMTGCDEGQALMEKWLGVEEISDDNLYMRAVTYNQKNLIDQYNAKSSEITSLTWD
QTQSSLKSLIAAFVSADQ----------------------------------------------------LW
EQWLADPNNKINELKWYNPAKSLLWISEITRSVAKWSLQYNINPLIAKRLSRVSFIAYAHSSLLTDRVPKWS
LLYNVDFKMTNASTQLNVTEIERRWKNAGQNTA---------------------------------------
AQKIKEILSKSPASVAIRISSIVALFELINFGVQSNKFIGERNWENGFQLISGLLASSA-AVLDIAGGGMDA
LSFQEKANKIRLYGARLAVSAAIISFCIDAFAVYKEKEGDKDKILMAFLFTKATVSLVMVGVGFGLLLNLPI
LQNSWV---------------VSSANKIAFTRILLRANFIRIAAYCNWIGIGFLV------IEYLLKTYVLD
DDLQKWCEKSIFGKDSSKFKLFKDEEEAFSKAIMSI------------------------------------
----------------------------

>AR_0088_12
----MSENQKMILGKSKSNNLFNEAIAQQKQKQSQIPVMGK-----TKAVGDMRNPTVAKNINKKIEESEIA
K-----------------------SAKQSTAANMQNSSAGQAQKECSNFCRKKGFNILPLRYTVVKDNPPAL
PPTLGKNVKDIQLSHLKYAVEMINTG-YIYRLVK------------------------------RTSGALEW
A----GYKVTPNGHLSYFPVGKEAPK--------------------------SVPEFACKGAGH-SFNSSVI
AVESNPKDEAKIAYIIHTHVPMSKAKISEYEKNADKFVTEGK----WQKILINGGSAQEHCIAASQFKTTVY
NLNSFGKN-----------------------------------------------RIDATLNKFKDEPKSLA
CMAL--------YDPVGITRKLNDSRNSQFGKLTNY----------------------------LAKNVEKI
SNEHRLQSSQLVDSIKIVVENKLINQQLSARSQVADLSAKIEYAKDKIPKFGSMDQREYLSKIQEIWDRAPT
AERDKFLLQRNKKF-------AEGEQKLKEKIITQS----------------------KAEAKITWETKYAP
KI-------NWDTKKAFDK----EVKK---------LINEGRTLAQTYASDHIKWLKSKHLLNAFYVYDQTI
LKEYGALFHVHALAVMDGMTGCDEGQALMEKWLGVEEISDDNLYMRAVTYNQKNLIDQYNAKSSEITSLTWD
QTQSSLKSLIAAFVSADQ----------------------------------------------------LW
EQWLADPNNKINELKWYNPAKSLLWISEITRSVAKWSLQYNINPLIAKRLSRVSFIAYAHSSLLTDRVPKWS
LLYNVDFKMTNASTQLNVTEIERRWKNAGQNTA---------------------------------------
AQKIKEILSKSPASVAIRISSIVALFELINFGVQSNKFIGERNWENGFQLISGLLASSA-AVLDIAGGGMDA
LSFQEKANKIRLYGARLAVSAAIISFCIDAFAVYKEKEGDKDKILMAFLFTKATVSLVMVGVGFGLLLNLPI
LQNSWV---------------VSSANKIAFTRILLRANFIRIAAYCNWIGIGFLV------IEYLLKTYVLD
DDLQKWCEKSIFGKDSSKFKLFKDEEEAFSKAIMSI------------------------------------
----------------------------

>CIP70.10_12
----MSENQKMILGKSKSNNLFNEAIAQQKQKQSQIPVMGK-----TKAVGDMRNPTVAKNINKKIEESEIA

K-----------------------SAKQSTAANMQNSSAGQAQKECSNFCRKKGFNILPLRYTVVKDNPPAL
PPTLGKNVKDIQLSHLKYAVEMINTG-YIYRLVK------------------------------RTSGALEW
A----GYKVTPNGHLSYFPVGKEAPK--------------------------SVPEFACKGAGH-SFNSSVI
AVESNPKDEAKIAYIIHTHVPMSKAKISEYEKNADKFVTEGK----WQKILINGGSAQEHCIAASQFKTTVY
NLNSFGKN-----------------------------------------------RIDATLNKFKDEPKSLA
CMAL--------YDPVGITRKLNDSRNSQFGKLTNY----------------------------LAKNVEKI
SNEHRLQSSQLVDSIKIVVENKLINQQLSARSQVADLSAKIEYAKDKIPKFGSMDQREYLSKIQEIWDRAPT
AERDKFLLQRNKKF-------AEGEQKLKEKIITQS----------------------KAEAKITWETKYAP
KI-------NWDTKKAFDK----EVKK---------LINEGRTLAQTYASDHIKWLKSKHLLNAFYVYDQTI
LKEYGALFHVHALAVMDGMTGCDEGQALMEKWLGVEEISDDNLYMRAVTYNQKNLIDQYNAKSSEITSLTWD
QTQSSLKSLIAAFVSADQ----------------------------------------------------LW
EQWLADPNNKINELKWYNPAKSLLWISEITRSVAKWSLQYNINPLIAKRLSRVSFIAYAHSSLLTDRVPKWS
LLYNVDFKMTNASTQLNVTEIERRWKNAGQNTA---------------------------------------
AQKIKEILSKSPASVAIRISSIVALFELINFGVQSNKFIGERNWENGFQLISGLLASSA-AVLDIAGGGMDA
LSFQEKANKIRLYGARLAVSAAIISFCIDAFAVYKEKEGDKDKILMAFLFTKATVSLVMVGVGFGLLLNLPI
LQNSWV---------------VSSANKIAFTRILLRANFIRIAAYCNWIGIGFLV------IEYLLKTYVLD
DDLQKWCEKSIFGKDSSKFKLFKDEEEAFSKAIMSI------------------------------------
----------------------------
>HWBA8_12
----MSENQKMILGKSKSNNLFNEAIAQQKQKQSQIPVMGK-----TKAVGDMRNPTVAKNINKKIEESEIA
K-----------------------SAKQSTAANMQNSSAGQAQKECSNFCRKKGFNILPLRYTVVKDNPPAL
PPTLGKNVKDIQLSHLKYAVEMINTG-YIYRLVK------------------------------RTSGALEW
A----GYKVTPNGHLSYFPVGKEAPK--------------------------SVPEFACKGAGH-SFNSSVI
AVESNPKDEAKIAYIIHTHVPMSKAKISEYEKNADKFVTEGK----WQKILINGGSAQEHCIAASQFKTTVY
NLNSFGKN-----------------------------------------------RIDATLNKFKDEPKSLA
CMAL--------YDPVGITRKLNDSRNSQFGKLTNY----------------------------LAKNVEKI
SNEHRLQSSQLVDSIKIVVENKLINQQLSARSQVADLSAKIEYAKDKIPKFGSMDQREYLSKIQEIWDRAPT
AERDKFLLQRNKKF-------AEGEQKLKEKIITQS----------------------KAEAKITWETKYAP
KI-------NWDTKKAFDK----EVKK---------LINEGRTLAQTYASDHIKWLKSKHLLNAFYVYDQTI
LKEYGALFHVHALAVMDGMTGCDEGQALMEKWLGVEEISDDNLYMRAVTYNQKNLIDQYNAKSSEITSLTWD
QTQSSLKSLIAAFVSADQ----------------------------------------------------LW
EQWLADPNNKINELKWYNPAKSLLWISEITRSVAKWSLQYNINPLIAKRLSRVSFIAYAHSSLLTDRVPKWS
LLYNVDFKMTNASTQLNVTEIERRWKNAGQNTA---------------------------------------

AQKIKEILSKSPASVAIRISSIVALFELINFGVQSNKFIGERNWENGFQLISGLLASSA-AVLDIAGGGMDA
LSFQEKANKIRLYGARLAVSAAIISFCIDAFAVYKEKEGDKDKILMAFLFTKATVSLVMVGVGFGLLLNLPI
LQNSWV---------------VSSANKIAFTRILLRANFIRIAAYCNWIGIGFLV------IEYLLKTYVLD
DDLQKWCEKSIFGKDSSKFKLFKDEEEAFSKAIMSI------------------------------------
----------------------------
>R2091_12
----MSENQKMILGKSKSNNLFNEAIAQQKQKQSQIPVMGK-----TKAVGDMRNPTVAKNINKKIEESEIA
K-----------------------SAKQSTAANMQNSSAGQAQKECSNFCRKKGFNILPLRYTVVKDNPPAL
PPTLGKNVKDIQLSHLKYAVEMINTG-YIYRLVK------------------------------RTSGALEW
A----GYKVTPNGHLSYFPVGKEAPK--------------------------SVPEFACKGAGH-SFNSSVI
AVESNPKDEAKIAYIIHTHVPMSKAKISEYEKNADKFVTEGK----WQKILINGGSAQEHCIAASQFKTTVY
NLNSFGKN-----------------------------------------------RIDATLNKFKDEPKSLA
CMAL--------YDPVGITRKLNDSRNSQFGKLTNY----------------------------LAKNVEKI
SNEHRLQSSQLVDSIKIVVENKLINQQLSARSQVADLSAKIEYAKDKIPKFGSMDQREYLSKIQEIWDRAPT
AERDKFLLQRNKKF-------AEGEQKLKEKIITQS----------------------KAEAKITWETKYAP
KI-------NWDTKKAFDK----EVKK---------LINEGRTLAQTYASDHIKWLKSKHLLNAFYVYDQTI
LKEYGALFHVHALAVMDGMTGCDEGQALMEKWLGVEEISDDNLYMRAVTYNQKNLIDQYNAKSSEITSLTWD
QTQSSLKSLIAAFVSADQ----------------------------------------------------LW
EQWLADPNNKINELKWYNPAKSLLWISEITRSVAKWSLQYNINPLIAKRLSRVSFIAYAHSSLLTDRVPKWS
LLYNVDFKMTNASTQLNVTEIERRWKNAGQNTA---------------------------------------
AQKIKEILSKSPASVAIRISSIVALFELINFGVQSNKFIGERNWENGFQLISGLLASSA-AVLDIAGGGMDA
LSFQEKANKIRLYGARLAVSAAIISFCIDAFAVYKEKEGDKDKILMAFLFTKATVSLVMVGVGFGLLLNLPI
LQNSWV---------------VSSANKIAFTRILLRANFIRIAAYCNWIGIGFLV------IEYLLKTYVLD
DDLQKWCEKSIFGKDSSKFKLFKDEEEAFSKAIMSI------------------------------------
----------------------------
>LAC-4_12
----MSENQKMILGKSKSNNLFNEAIAQQKQKQSQIPVMGK-----TKAVGDMRNPTVAKNINKKIEESEIA
K-----------------------SAKQSTAANMQNSSAGQAQKECSNFCRKKGFNILPLRYTVVKDNPPAL
PPTLGKNVKDIQLSHLKYAVEMINTG-YIYRLVK------------------------------RTSGALEW
A----GYKVTPNGHLSYFPVGKEAPK--------------------------SVPEFACKGAGH-SFNSSVI
AVESNPKDEAKIAYIIHTHVPMSKAKISEYEKNADKFVTEGK----WQKILINGGSAQEHCIAASQFKTTVY
NLNSFGKN-----------------------------------------------RIDATLNKFKDEPKSLA
CMAL--------YDPVGITRKLNDSRNSQFGKLTNY----------------------------LAKNVEKI

SNEHRLQSSQLVDSIKIVVENKLINQQLSARSQIADLSAKIEYAKDKIPKFGSMDQREYLSKIQEIWDRAPT
AERDKFLLQRNKKF-------AEGEQKLKEKIITQS----------------------KAEAKITWETKYAP
KI-------NWDTKKAFDK----EVKK---------LINEGRTLAQTYASDHIKWLKSKHLLNAFYVYDQTI
LKEYGALFHVHALAVMDGMTGCDEGQALMEKWLGVEEISDDNLYMRAVTYNQKNLIDQYNAKSSEITSLTWD
QTQSSLKSLIAAFVSADQ------------------------------------------------------LW
EQWLADPNNKINELKWYNPAKSLLWISEITRSVAKWSLQYNINPLIAKRLSRVSFIAYAHSSLLTDRVPKWS
LLYNVDFKMTNASTQLNVTEIERRWKNAGQNTA---------------------------------------
AQKIKEILSKSPASVAIRISSIVALFELINFGVQSNKFIGERNWENGFQLISGLLASSA-AVLDIAGGGMDA
LSFQEKANKIRLYGARLAVSAAIISFCIDAFAVYKEKEGDKDKILMAFLFTKATVSLVMVGVGFGLLLNLPI
LQNSWV---------------VSSANKIAFTRILLRANFIRIAAYCNWIGIGFLV------IEYLLKTYVLD
DDLQKWCEKSIFGKDSSKFKLFKDEEEAFSKAIMSI------------------------------------
----------------------------
>WKA02_12
----MSENQKMILGKSKSNNLFNEAIAQQKQKQSQIPVMGK-----TKAVGDMRNPTVAKNINKKIEESEIA
K-----------------------SAKQSTAANMQNSSAGQAQKECSNFCRKKGFNILPLRYTVVKDNPPAL
PPTLGKNVKDIQLSHLKYAVEMINTG-YIYRLVK------------------------------RTSGALEW
A----GYKVTPNGHLSYFPVGKEAPK---------------------------SVPEFACKGAGH-SFNSSVI
AVESNPKDEAKIAYIIHTHVPMSKAKISEYEKNADKFVTEGK----WQKILINGGSAQEHCIAASQFKTTVY
NLNSFGKN------------------------------------------------RIDATLNKFKDEPKSLA
CMAL--------YDPVGITRKLNDSRNSQFGKLTNY------------------------------LAKNVEKI
SNEHRLQSSQLVDSIKIVVENKLINQQLSARSQIADLSAKIEYAKDKIPKFGSMDQREYLSKIQEIWDRAPT
AERDKFLLQRNKKF-------AEGEQKLKEKIITQS----------------------KAEAKITWETKYAP
KI-------NWDTKKAFDK----EVKK---------LINEGRTLAQTYASDHIKWLKSKHLLNAFYVYDQTI
LKEYGALFHVHALAVMDGMTGCDEGQALMEKWLGVEEISDDNLYMRAVTYNQKNLIDQYNAKSSEITSLTWD
QTQSSLKSLIAAFVSADQ------------------------------------------------------LW
EQWLADPNNKINELKWYNPAKSLLWISEITRSVAKWSLQYNINPLIAKRLSRVSFIAYAHSSLLTDRVPKWS
LLYNVDFKMTNASTQLNVTEIERRWKNAGQNTA---------------------------------------
AQKIKEILSKSPASVAIRISSIVALFELINFGVQSNKFIGERNWENGFQLISGLLASSA-AVLDIAGGGMDA
LSFQEKANKIRLYGARLAVSAAIISFCIDAFAVYKEKEGDKDKILMAFLFTKATVSLVMVGVGFGLLLNLPI
LQNSWV---------------VSSANKIAFTRILLRANFIRIAAYCNWIGIGFLV------IEYLLKTYVLD
DDLQKWCEKSIFGKDSSKFKLFKDEEEAFSKAIMSI------------------------------------

**Supplementary file S4b**

```
>Ab04-mff_13
------------------------------------------------------------------------
-----MSNSLDWNSKTNISEHS-------CKDAHCDCLTF---WEIYQQEAIKRLSNLQKANNYK-------
------------------------------------------------------------------------
------------------------------------------------------------------------
------------------------------------------------------------------------
---------LEP-GYEARARRIASVYAKIFLEIELGGNKNLLGRYYWMGLGAFASKTVA-------------
------------------------------------------------------------------------
-----------------------------------TIFKHWGSLLGYN------------VGVQEAINLFAR
GN-----------LWLFMDIVPWHLAWSASS--ES--FKS--------------------------------
------------------------------------------------------------------------
---------CKGTRDTSTFKFVKPA---------------------------------L---MNLPWSSCLP
SIKN------LKVTKEINDAFTLLPAIEKAFLNGRDEKSKYRKAS---------------------QNLFDH
LMAIAVQEQHNI--LQVVVWENCSVKFGARMQR----WFIGMPDA---------------------------
------------------------------------------------------------------------
-----------------TLVLSSDYSVDAVKKNWFGNYTGSKADQLVELKEDVYI-----------------
-------------APLKGTIAEDYDSRMKWIGKAAEKYHRLMLDE---------------------------
------------------------------------------------------------------------
-------------------------------------KGRPFLQQELKTISKWGNSKADFKIHSSSNEGKV-
------------------------------------------------------------------------
------------------------------------------------------------------------
---------------------------
>LAC-4_13
------------------------------------------------------------------------
-----MSNSLDWNSKTNISEHS-------CKDAHCDCLTF---WEIYQQEAIKRLSNLQKANNYK-------
------------------------------------------------------------------------
------------------------------------------------------------------------
------------------------------------------------------------------------
---------LEP-GYEARARRIASVYAKIFLEIELGGNKNLLGRYYWMGLGAFASKTVA-------------
------------------------------------------------------------------------
-----------------------------------TIFKHWGSLLGYN------------VGVQEAINLFAR
GN-----------LWLFMDIVPWHLAWSASS--ES--FKS--------------------------------
------------------------------------------------------------------------
```

---------CKGTRDTSTFKFVKPA---------------------------------L---MNLPWSSCLP
SIKN------LKVTKEINDAFTLLPAIEKAFLNGRDEKSKYRKAS---------------------QNLFDH
LMAIAVQEQHNI--LQVVVWENCSVKFGARMQR----WFIGMPDA---------------------------
------------------------------------------------------------------------
-----------------TLVLSSDYSVDAVKKNWFGNYTGSKADQLVELKEDVYI-----------------
-------------APLKGTIAEDYDSRMKWIGKAAEKYHRLMLDE---------------------------
------------------------------------------------------------------------
-------------------------------------KGRPFLQQELKTISKWGNSKADFKIHSSSNEGKV-
------------------------------------------------------------------------
------------------------------------------------------------------------
---------------------------
>WKA02_13
------------------------------------------------------------------------
-----MSNSLDWNSKTNISEHS-------CKDAHCDCLTF---WEIYQQEAIKRLSNLQKANNYK-------
------------------------------------------------------------------------
------------------------------------------------------------------------
------------------------------------------------------------------------
---------LEP-GYEARARRIASVYAKIFLEIELGGNKNLLGRYYWMGLGAFASKTVA-------------
------------------------------------------------------------------------
-----------------------------------TIFKHWGSLLGYN------------VGVQEAINLFAR
GN-----------LWLFMDIAPWHLAWSASS--ES--FKS--------------------------------
------------------------------------------------------------------------
---------CKGTRDTSTFKFIKPA---------------------------------L---MNLPWSSCLP
SIKN------LKVTKEINDAFTLLPAIEKAFLNGRDEKSKYRKAS---------------------QNLFDH
LMAIAVQEQHNI--LQVVVWENCSVKFGAWMQR----WFIGMPDA---------------------------
------------------------------------------------------------------------
-----------------TLVLSSDYSVDAVKKNWFGNYTGSKADQLVELKEDVYI-----------------
-------------APLKGTIAEDYDSRMKWIGKAAEKYHRLMLDE---------------------------
------------------------------------------------------------------------
-------------------------------------KGRPFLQQELKTISKWGNSKADFKIHSSSNEGKV-
------------------------------------------------------------------------
------------------------------------------------------------------------
---------------------------

```
>BJAB0715_13
------------------------------------------------------------------------
-----MSNSLDWNSKTNISEHS-------CKDAHCDCLTF---WEIYQQEAIKRLSNLQKANNYK-------
------------------------------------------------------------------------
------------------------------------------------------------------------
------------------------------------------------------------------------
---------LEP-GYEARARRIASVYAKIFLEIELGGNKNLLGRYYWMGLGAFASKTVA-------------
------------------------------------------------------------------------
-----------------------------------TIFKHWGSLLGYN------------VGVQEAINLFAR
GN-----------LWLFMDIAPWHLAWSASS--ES--FKS--------------------------------
------------------------------------------------------------------------
---------CKGTRDTSTFKFVKPA---------------------------------L---MNLPWSSCLP
SIKN------LKVTKEINDAFTLLPAIEKAFLNGRDEKSKYRKAS---------------------QNLFDH
LMAIAVQEQHNI--LQVVVWENCSVKFGAWMQR----WFIGMPDA---------------------------
------------------------------------------------------------------------
-----------------TLVLSSDYSVDAVKKNWFGNYTGSKADQLVELKEDVYI-----------------
-------------APLKGTIAEDYDSRMKWIGKAAEKYHRLMLDE---------------------------
------------------------------------------------------------------------
-------------------------------------KGRPFLQQELKTISKWGNSKADFKIHSSSNEGKV-
------------------------------------------------------------------------
------------------------------------------------------------------------
---------------------------
>D1279779_13
------------------------------------------------------------------------
-----MSNSLDWNSKTNISEHS-------CKDAHCDCLTF---WEIYQQEAIKRLSNLQKANNYK-------
------------------------------------------------------------------------
------------------------------------------------------------------------
------------------------------------------------------------------------
---------LEP-GYEARARRIASVYAKIFLEIELGGNKNLLGRYYWMGLGAFASKTVA-------------
------------------------------------------------------------------------
-----------------------------------TIFKHWGSLLGYN------------VGVQEAINLFAR
GN-----------LWLFMDIAPWHLAWSASS--ES--FKS--------------------------------
------------------------------------------------------------------------
```

```
---------CKGTRDTSTFKFVKPA---------------------------------L---MNLPWSSCLP
SIKN------LKVTKEINDAFTLLPAIEKAFLNGRDEKSKYRKAS---------------------QNLFDH
LMAIAVQEQHNI--LQVVVWENCSVKFGAWMQR----WFIGMPDA---------------------------
------------------------------------------------------------------------
-----------------TLVLSSDYSVDAVKKNWFGNYTGSKADQLVELKEDVYI-----------------
-------------APLKGTIAEDYDSRMKWIGKAAEKYHRLMLDE---------------------------
------------------------------------------------------------------------
-------------------------------------KGRPFLQQELKTISKWGNSKADFKIHSSSNEGKV-
------------------------------------------------------------------------
------------------------------------------------------------------------
---------------------------
>KAB02_13
------------------------------------------------------------------------
-----MSNSLDWNSKTNISEHS-------CKDAHCDCLTF---WEIYQQEAIKRLSNLQKANNYK-------
------------------------------------------------------------------------
------------------------------------------------------------------------
------------------------------------------------------------------------
---------LEP-GYEARARRIASVYAKIFLEIELGGNKNLLGRYYWMGLGAFASKTVA-------------
------------------------------------------------------------------------
-----------------------------------TIFKHWGSLLGYN------------VGVQEAINLFAR
GN-----------LWLFMDIAPWHLAWSASS--ES--FKS--------------------------------
------------------------------------------------------------------------
---------CKGTRDTSTFKFVKPA---------------------------------L---MNLPWSSCLP
SIKN------LKVTKEINDAFTLLPAIEKAFLNGRDEKSKYRKAS---------------------QNLFDH
LMAIAVQEQHNI--LQVVVWENCSVKFGAWMQR----WFIGMPDA---------------------------
------------------------------------------------------------------------
-----------------TLVLSSDYSVDAVKKNWFGNYTGSKADQLVELKEDVYI-----------------
-------------APLKGTIAEDYDSRMKWIGKAAEKYHRLMLDE---------------------------
------------------------------------------------------------------------
-------------------------------------KGRPFLQQELKTISKWGNSKADFKIHSSSNEGKV-
------------------------------------------------------------------------
------------------------------------------------------------------------
---------------------------
```

```
>KAB05_13
------------------------------------------------------------------------
-----MSNSLDWNSKTNISEHS-------CKDAHCDCLTF---WEIYQQEAIKRLSNLQKANNYK-------
------------------------------------------------------------------------
------------------------------------------------------------------------
------------------------------------------------------------------------
---------LEP-GYEARARRIASVYAKIFLEIELGGNKNLLGRYYWMGLGAFASKTVA-------------
------------------------------------------------------------------------
-----------------------------------TIFKHWGSLLGYN------------VGVQEAINLFAR
GN-----------LWLFMDIAPWHLAWSASS--ES--FKS--------------------------------
------------------------------------------------------------------------
---------CKGTRDTSTFKFVKPA---------------------------------L---MNLPWSSCLP
SIKN------LKVTKEINDAFTLLPAIEKAFLNGRDEKSKYRKAS---------------------QNLFDH
LMAIAVQEQHNI--LQVVVWENCSVKFGAWMQR----WFIGMPDA---------------------------
------------------------------------------------------------------------
-----------------TLVLSSDYSVDAVKKNWFGNYTGSKADQLVELKEDVYI-----------------
-------------APLKGTIAEDYDSRMKWIGKAAEKYHRLMLDE---------------------------
------------------------------------------------------------------------
-------------------------------------KGRPFLQQELKTISKWGNSKADFKIHSSSNEGKV-
------------------------------------------------------------------------
------------------------------------------------------------------------
---------------------------
>MDR-TJ_13
------------------------------------------------------------------------
-----MSNSLDWNSKTNISEHS-------CKDAHCDCLTF---WEIYQQEAIKRLSNLQKANNYK-------
------------------------------------------------------------------------
------------------------------------------------------------------------
------------------------------------------------------------------------
---------LEP-GYEARARRIASVYAKIFLEIELGGNKNLLGRYYWMGLGAFASKTVA-------------
------------------------------------------------------------------------
-----------------------------------TIFKHWGSLLGYN------------VGVQEAINLFAR
GN-----------LWLFMDIAPWHLAWSASS--ES--FKS--------------------------------
------------------------------------------------------------------------
```

```
---------CKGTRDTSTFKFVKPA---------------------------------L---MNLPWSSCLP
SIKN------LKVTKEINDAFTLLPAIEKAFLNGRDEKSKYRKAS---------------------QNLFDH
LMAIAVQEQHNI--LQVVVWENCSVKFGAWMQR----WFIGMPDA---------------------------
------------------------------------------------------------------------
-----------------TLVLSSDYSVDAVKKNWFGNYTGSKADQLVELKEDVYI-----------------
-------------APLKGTIAEDYDSRMKWIGKAAEKYHRLMLDE---------------------------
------------------------------------------------------------------------
-------------------------------------KGRPFLQQELKTISKWGNSKADFKIHSSSNEGKV-
------------------------------------------------------------------------
------------------------------------------------------------------------
---------------------------
>R2090_13
------------------------------------------------------------------------
-----MSNSLDWNSKTNISEHS-------CKDAHCDCLTF---WEIYQQEAIKRLSNLQKANNYK-------
------------------------------------------------------------------------
------------------------------------------------------------------------
------------------------------------------------------------------------
---------LEP-GYEARARRIASVYAKIFLEIELGGNKNLLGRYYWMGLGAFASKTVA-------------
------------------------------------------------------------------------
-----------------------------------TIFKHWGSLLGYN------------VGVQEAINLFAR
GN-----------LWLFMDIAPWHLAWSASS--ES--FKS--------------------------------
------------------------------------------------------------------------
---------CKGTRDTSTFKFVKPA---------------------------------L---MNLPWSSCLP
SIKN------LKVTKEINDAFTLLPAIEKAFLNGRDEKSKYRKAS---------------------QNLFDH
LMAIAVQEQHNI--LQVVVWENCSVKFGAWMQR----WFIGMPDA---------------------------
------------------------------------------------------------------------
-----------------TLVLSSDYSVDAVKKNWFGNYTGSKADQLVELKEDVYI-----------------
-------------APLKGTIAEDYDSRMKWIGKAAEKYHRLMLDE---------------------------
------------------------------------------------------------------------
-------------------------------------KGRPFLQQELKTISKWGNSKADFKIHSSSNEGKV-
------------------------------------------------------------------------
------------------------------------------------------------------------
---------------------------
```

```
>XH856_13
------------------------------------------------------------------------
-----MSNSLDWNSKTNISEHS-------CKDAHCDCLTF---WEIYQQEAIKRLSNLQKANNYK-------
------------------------------------------------------------------------
------------------------------------------------------------------------
------------------------------------------------------------------------
---------LEP-GYEARARRIASVYAKIFLEIELGGNKNLLGRYYWMGLGAFASKTVA-------------
------------------------------------------------------------------------
-----------------------------------TIFKHWGSLLGYN------------VGVQEAINLFAR
GN-----------LWLFMDIAPWHLAWSASS--ES--FKS--------------------------------
------------------------------------------------------------------------
---------CKGTRDTSTFKFVKPA---------------------------------L---MNLPWSSCLP
SIKN------LKVTKEINDAFTLLPAIEKAFLNGRDEKSKYRKAS---------------------QNLFDH
LMAIAVQEQHNI--LQVVVWENCSVKFGAWMQR----WFIGMPDA---------------------------
------------------------------------------------------------------------
-----------------TLVLSSDYSVDAVKKNWFGNYTGSKADQLVELKEDVYI-----------------
-------------APLKGTIAEDYDSRMKWIGKAAEKYHRLMLDE---------------------------
------------------------------------------------------------------------
-------------------------------------KGRPFLQQELKTISKWGNSKADFKIHSSSNEGKV-
------------------------------------------------------------------------
------------------------------------------------------------------------
---------------------------
>XH858_13
------------------------------------------------------------------------
-----MSNSLDWNSKTNISEHS-------CKDAHCDCLTF---WEIYQQEAIKRLSNLQKANNYK-------
------------------------------------------------------------------------
------------------------------------------------------------------------
------------------------------------------------------------------------
---------LEP-GYEARARRIASVYAKIFLEIELGGNKNLLGRYYWMGLGAFASKTVA-------------
------------------------------------------------------------------------
-----------------------------------TIFKHWGSLLGYN------------VGVQEAINLFAR
GN-----------LWLFMDIAPWHLAWSASS--ES--FKS--------------------------------
------------------------------------------------------------------------
```

---------CKGTRDTSTFKFVKPA---------------------------------L---MNLPWSSCLP
SIKN------LKVTKEINDAFTLLPAIEKAFLNGRDEKSKYRKAS---------------------QNLFDH
LMAIAVQEQHNI--LQVVVWENCSVKFGAWMQR----WFIGMPDA---------------------------
------------------------------------------------------------------------
-----------------TLVLSSDYSVDAVKKNWFGNYTGSKADQLVELKEDVYI-----------------
-------------APLKGTIAEDYDSRMKWIGKAAEKYHRLMLDE---------------------------
------------------------------------------------------------------------
-------------------------------------KGRPFLQQELKTISKWGNSKADFKIHSSSNEGKV-
------------------------------------------------------------------------
------------------------------------------------------------------------
---------------------------
>KAB06_13
------------------------------------------------------------------------
-----MSNSLDWNSKTNISEHS-------CKDAHCDCLTF---WEIYQQEAIKRLSNLQKANNYK-------
------------------------------------------------------------------------
------------------------------------------------------------------------
------------------------------------------------------------------------
---------LEP-GYEARARRIASVYAKIFLEIELGGNKNLLGRYYWMGLGAFASKTVA-------------
------------------------------------------------------------------------
-----------------------------------TIFKHWGSLLGYN------------VGVQEAINLFAR
GN-----------LWLFMDIAPWHLAWSASS--ES--FKS--------------------------------
------------------------------------------------------------------------
---------CKGTRDTSTFKFVKPA---------------------------------L---MNLPWSSCLP
SIKN------LKVTKEINDAFTLLPAIEKAFLNGRDEKSKYRKAS---------------------QNLFDH
LMAIAVQEQHNI--LQVVVWE-CSVKFGAWMQR----WFIGMPDA---------------------------
------------------------------------------------------------------------
-----------------TLVLSSDYSVDAVKKNWFGNYTGSKADQLVELKEDVYI-----------------
-------------APLKGTIAEDYDSRMKWIGKAAEKYHRLMLDE---------------------------
------------------------------------------------------------------------
-------------------------------------KGRPFLQQELKTISKWGNSKADFKIHSSSNEGKV-
------------------------------------------------------------------------
------------------------------------------------------------------------
---------------------------

```
>15A34_23
------------------------------------------------------------------MNTELK
HKLYNITFTLHDLTHKPIPNLY-------YEIKNGKDLV----KKGNTNAQGEITLKYVGGNTLT-------
----------------LFVRKDIDNTLKEIGKVHTPSKNIKVKLISPKMKFDVTLLPHQKQGKYWRGTYKVK
SGDTLSKIAKEHHTTISALLALNP------------------------------------------------
------------------------NIKSPHEIYQNENIDIVEGNADAIIKLDENMTVKVPPHKKTGTVSIDR
KSKSTSITPQTDTTNKTAEPKVGQTKLQPPVTKKEQSAASDSKSIPQQKTSTATTQKI--------------
------------------------------------------------------------------------
-----------------------------------EVQQEHNEDKKPV------------AVASLSGCVCKD
YD-----------LIWGKTTKFVNCEFRKKV--IQ--ICK--------------------------------
------------------------------------------------------------------------
-------DMWPNDTKNMANYLMACM---------------------------------H---LETGGSFDPA
QPNG---------------------LGYYGLIQFGPDVRKDLNNI--------------------SVEKLTK
MSGAQQLDLVKQ--HFTRSDRHKLMKSLTDMYL----YINYPNALLSGKNKPNDILYEGGS-----------
------------------------------------------------------------------------
----ERSAYHANPAFMKEKGEYQNIFAYRKDKNGNFKLDKNKKKIPIRGFEDGKT-----------------
-------------YIWEVTQEINKHLTTGLDSKNKENNFACSIKPVQPIQDICPNCQTKHVDLSASVEWISQ
FTQPRPNVACARTCVLILKNSKLSSSAGSPTGLFQVALENSNHSTIEATSNFQNG-----------------
-------------------------------------LNYLDKELNVGHPVMIGVDHKLGYGINEGTTDHFI
VVVGRGCENGKVYYRFYDVGTRHKEKGTSNSNKLYIINGFLRGKTAYNGSTYTMTQVRRN------------
------------------------------------------------------------------------
---------------------------
>ab736_9
---------------------------------------------------------MNKLVTITTKFYDKS
GNRIINLNVQSRYKGSLKANSQKTDKLGLFVFQASPNRTV---EILAKPPNQKDYTVFKTINSSM-------
------------------NSSEIHPIKVQLPKTIDEYKQVKQSKSTKGIVSTFFKIFDMNGKVMKNFPIQSR
PKGKGNSPDKYTNDEGIVEV----------------------------------------------------
------------------------RSSPNRDIEVLVLTSNDTFVLKSSINSANGSSQPVLIKLDEPYEKFKS
ASTIKILDRDGS-DYIVEKTNVKMLVVENGKKQLFSISNGKLSLQSMIGQK---------------------
------------------------------------------------------------------------
-----------------------------------LEFTVYKPDGKPL----------KTQTYMATRVKNNL
IE-----------FHLDVDITKGSTAQNDPE--INKKVKV--------------------------------
---------------------------------------------------------------------DIL
```

```
ITMEQMKKMWPKALATKMQPILDE--------------------------------------LNSDLLGYKL
DTRL-------------------RQAHFMAQVRQEVGSSFSLREQ----------------------VEYMG
PTALKQIGYYRT--HPKQAEIDGYKRGQGPANG--EVIANRMYDD---------------------------
------------------------------------------------------------------------
-------------NYRSAKYKLGNTSPGDGWKYLGRGLKQLTGKNNYQDLTNMYS-----------------
-------------TLWPGEKVDFVKNPELIEQPKYAVRSAIRFWLKFK------------------------
------------------------------------------------------------------------
-------------------------------------LYEVADRGANGEQVDAITKVINEATNSYADRRAHF
VQARKIFI----------------------------------------------------------------
------------------------------------------------------------------------
---------------------------
>ATCC19606_9
---------------------------------------------------------MNKLVTITTKFYDKS
GNRIINLNVQSRYKGSLKANSQKTDKLGLFVFQASPNRTV---EILAKPPNQKDYTVFKTINSSM-------
------------------NSSEIHPIKVQLPKTIDEYKQVKQSKSTKGIVSTFFKIFDMNGKVMKNFPIQSR
PKGKGNSPDKYTNDEGIVEV----------------------------------------------------
------------------------RSSPNRDIEVLVLTSNDTFVLKSSINSANGSSQPVLIKLDEPYEKFKS
ASTIKILDRDGS-DYIVEKTNVKMLVVENGKKQLFSISNGKLSLQSMIGQK---------------------
------------------------------------------------------------------------
-----------------------------------LEFTVYKPDGKPL----------KTQTYMATRVKNNL
IE-----------FHLDVDITKGSTAQNDPE--INKKVKV--------------------------------
---------------------------------------------------------------------DIL
ITMEQMKKMWPKALATKMQPILDE--------------------------------------LNSDLLGYKL
DTRL-------------------RQAHFMAQVRQEVGSSFSLREQ----------------------VEYMG
PTALKQIGYYRT--HPKQAEIDGYKRGQGPANG--EVIANRMYDD---------------------------
------------------------------------------------------------------------
-------------NYRSAKYKLGNTSPGDGWKYLGRGLKQLTGKNNYQDLTNMYS-----------------
-------------TLWPGEKVDFVKNPELIEQPKYAVRSAIRFWLKFK------------------------
------------------------------------------------------------------------
-------------------------------------LYEVADRGANGEQVDAITKVINEATNSYADRRAHF
VQARKIFI----------------------------------------------------------------
------------------------------------------------------------------------
---------------------------
```

```
>Ab04-mff_11
---------------------------------------------------MSYSEDFKIFVATINGEAENC
SETSWKVIAHTMRNRVGFANWKSWSTIIQIVTKTGYDAYT---QKNSPYKRAKKALDSGDISPKL-------
------------MSLIKAVEPIFNGTEPDFTGGVVYYYSPKAQAQLHKSDPSNYPSLVPDFVVSKSNPTQQV
QIPGTEKDDMRWYKVLTSIFDVSFVDNAGNPLVGVTVDVVYNDKKPVPLFKDLI------------------
------------------------TDQKGKIKKVRVCNYMGARFKVNGVLVKDKNNKEISLLGDGKNYASVI
VVNNGKGGIKSK-TDIHNQESIPGQKQESVKIDSKAEVNNEKKAEVSDNNTKQKNVNFSIKVIDSENKAIPN
FSYFLKYKNAEKKHSVGANGIEN-------------------------------------------------
-----------------------------------NLVALSGEEITVL------------ISGLDSKQEIIR
FT-----------AQEGMGEKTIKLNLHTFN--ILFRHKD--------------------------------
---------------------------------------TKKPITNLNLIQKYRNQIKQKKTDGNGKITVSA
MPGFELNYKLRDERNLLTIKVDKNK---------------------------------SLRVIDVDSSAIEQ
ASKN--IKIGTKVVEIAQSKQPVPSSKQKHPVETHDSTPKRDEKVKISTDGHPKTLVNDNGETEFIVYTYDQ
KTNQLFSGGNYS--IEYKGNKKRHSSGIHGIGK--KIHKGEIGQKIKITASSGGKEFIAFD-----------
------------------------------------------------------------------------
--GNLSRGMKAFELKIDRSVIPSVSDVIISFKGVNEQSRQAIVSQKTKNVLAYLAKEA--------------
-------------NMDKLYITSTIRTPRAQAEAMYIKATKYAKPGEDVKKVKAECIAKGLGKEATIQKMVEK
IVEFQNKGVRVSKHCVSEEQYRKNNIID--------------------------------------------
-------------------------------------LGTNSNGFGTGNTLNSVGKRFKAVCEKALKDGIIS
GFISADVAGEGAMHIEIIQ-----------------------------------------------------
------------------------------------------------------------------------
---------------------------
>BJAB0715_11
---------------------------------------------------MSYSEDFKIFVATINGEAENC
SETSWKVIAHTMRNRVGFANWKSWSTIIQIVTKTGYDAYT---QKNSPYKRAKKALDSGDISPKL-------
------------MSLIKAVEPIFNGTEPDFTGGVVYYYSPKAQAQLHKSDPSNYPSLVPDFVVSKSNPTQQV
QIPGTEKDDMRWYKVLTSIFDVSFVDNAGNPLVGVTVDVVYNDKKPVPLFKDLI------------------
------------------------TDQKGKIKKVRVCNYMGARFKVNGVLVKDKNNKEISLLGDGKNYASVI
VVNNGKGGIKSK-TDIHNQESIPGQKQESVKIDSKAEVNNEKKAEVSDNNTKQKNVNFSIKVIDSENKAIPN
FSYFLKYKNAEKKHSVGANGIEN-------------------------------------------------
-----------------------------------NLVALSGEEITVL------------ISGLDSKQEIIR
FT-----------AQEGMGEKTIKLNLHTFN--ILFRHKD--------------------------------
---------------------------------------TKKPITNLNLIQKYRNQIKQKKTDGNGKITVSA
```

MPGFELNYKLRDERNLLTIKVDKNK---------------------------------SLRVIDVDSSAIEQ
ASKN--IKIGTKVVEIAQSKQPVPSSKQKHPVETHDSTPKRDEKVKISTDGHPKTLVNDNGETEFIVYTYDQ
KTNQLFSGGNYS--IEYKGNKKRHSSGIHGIGK--KIHKGEIGQKIKITASSGGKEFIAFD-----------
------------------------------------------------------------------------
--GNLSRGMKAFELKIDRSVIPSVSDVIISFKGVNEQSRQAIVSQKTKNVLAYLAKEA--------------
-------------NMDKLYITSTIRTPRAQAEAMYIKATKYAKPGEDVKKVKAECIAKGLGKEATIQKMVEK
IVEFQNKGVRVSKHCVSEEQYRKNNIID--------------------------------------------
-------------------------------------LGTNSNGFGTGNTLNSVGKRFKAVCEKALKDGIIS
GFISADVAGEGAMHIEIIQ-----------------------------------------------------
------------------------------------------------------------------------
---------------------------

>LAC-4_11
---------------------------------------------------MSYSEDFKIFVATINGEAENC
SETSWKVIAHTMRNRVGFANWKSWSTIIQIVTKTGYDAYT---QKNSPYKRAKKALDSGDISPKL-------
------------MSLIKAVEPIFNGTEPDFTGGVVYYYSPKAQAQLHKSDPSNYPSLVPDFVVSKSNPTQQV
QIPGTEKDDMRWYKVLTSIFDVSFVDNAGNPLVGVTVDVVYNDKKPVPLFKDLI------------------
------------------------TDQKGKIKKVRVCNYMGARFKVNGVLVKDKNNKEISLLGDGKNYASVI
VVNNGKGGIKSK-TDIHNQESIPGQKQESVKIDSKAEVNNEKKAEVSDNNTKQKNVNFSIKVIDSENKAIPN
FSYFLKYKNAEKKHSVGANGIEN-------------------------------------------------
-----------------------------------NLVALSGEEITVL------------ISGLDSKQEIIR
FT-----------AQEGMGEKTIKLNLHTFN--ILFRHKD--------------------------------
---------------------------------------TKKPITNLNLIQKYRNQIKQKKTDGNGKITVSA
MPGFELNYKLRDERNLLTIKVDKNK---------------------------------SLRVIDVDSSAIEQ
ASKN--IKIGTKVVEIAQSKQPVPSSKQKHPVETHDSTPKRDEKVKISTDGHPKTLVNDNGETEFIVYTYDQ
KTNQLFSGGNYS--IEYKGNKKRHSSGIHGIGK--KIHKGEIGQKIKITASSGGKEFIAFD-----------
------------------------------------------------------------------------
--GNLSRGMKAFELKIDRSVIPSVSDVIISFKGVNEQSRQAIVSQKTKNVLAYLAKEA--------------
-------------NMDKLYITSTIRTPRAQAEAMYIKATKYAKPGEDVKKVKAECIAKGLGKEATIQKMVEK
IVEFQNKGVRVSKHCVSEEQYRKNNIID--------------------------------------------
-------------------------------------LGTNSNGFGTGNTLNSVGKRFKAVCEKALKDGIIS
GFISADVAGEGAMHIEIIQ-----------------------------------------------------
------------------------------------------------------------------------
---------------------------

>WKA02_11
---------------------------------------------------MSYSEDFKIFVATINGEAENC
SETSWKVIAHTMRNRVGFANWKSWSTIIQIVTKTGYDAYT---QKNSPYKRAKKALDSGDISPKL-------
------------MSLIKAVEPIFNGTEPDFTGGVVYYYSPKAQAQLHKSDPSNYPSLVPDFVVSKSNPTQQV
QIPGTEKDDMRWYKVLTSIFDVSFVDNAGNPLVGVTVDVVYNDKKPVPLFKDLI------------------
------------------------TDQKGKIKKVRVCNYMGARFKVNGVLVKDKNNKEISLLGDGKNYASVI
VVNNGKGGIKSK-TDIHNQESIPGQKQESVKIDSKAEVNNEKKAEVSDNNTKQKNVNFSIKVIDSENKAIPN
FSYFLKYKNAEKKHSVGANGIEN-------------------------------------------------
-----------------------------------NLVALSGEEITVL------------ISGLDSKQEIIR
FT-----------AQEGMGEKTIKLNLHTFN--ILFRHKD--------------------------------
---------------------------------------TKKPITNLNLIQKYRNQIKQKKTDGNGKITVSA
MPGFELNYKLRDERNLLTIKVDKNK---------------------------------SLRVIDVDSSAIEQ
ASKN--IKIGTKVVEIAQSKQPVPSSKQKHPVETHDSTPKRDEKVKISTDGHPKTLVNDNGETEFIVYTYDQ
KTNQLFSGGNYS--IEYKGNKKRHSSGIHGIGK--KIHKGEIGQKIKITASSGGKEFIAFD-----------
------------------------------------------------------------------------
--GNLSRGMKAFELKIDRSVIPSVSDVIISFKGVNEQSRQAIVSQKTKNVLAYLAKEA--------------
-------------NMDKLYITSTIRTPRAQAEAMYIKATKYAKPGEDVKKVKAECIAKGLGKEATIQKMVEK
IVEFQNKGVRVSKHCVSEEQYRKNNIID--------------------------------------------
-------------------------------------LGTNSNGFGTGNTLNSVGKRFKAVCEKALKDGIIS
GFISADVAGEGAMHIEIIQ-----------------------------------------------------
------------------------------------------------------------------------
---------------------------
>XH858_11
---------------------------------------------------MSYSEDFKIFVATINGEAENC
SETSWKVIAHTMRNRVGFANWKSWSTIIQIVTKTGYDAYT---QKNSPYKRAKKALDSGDISPKL-------
------------MSLIKAVEPIFNGTEPDFTGGVVYYYSPKAQAQLHKSDPSNYPSLVPDFVVSKSNPTQQV
QIPGTEKDDMRWYKVLTSIFDVSFVDNAGNPLVGVTVDVVYNDKKPVPLFKDLI------------------
------------------------TDQKGKIKKVRVCNYMGARFKVNGVLVKDKNNKEISLLGDGKNYASVI
VVNNGKGGIKSK-TDIHNQESIPGQKQESVKIDSKAEVNNEKKAEVSDNNTKQKNVNFSIKVIDSENKAIPN
FSYFLKYKNAEKKHSVGANGIEN-------------------------------------------------
-----------------------------------NLVALSGEEITVL------------ISGLDSKQEIIR
FT-----------AQEGMGEKTIKLNLHTFN--ILFRHKD--------------------------------
---------------------------------------TKKPITNLNLIQKYRNQIKQKKTDGNGKITVSA

```
MPGFELNYKLRDERNLLTIKVDKNK---------------------------------SLRVIDVDSSAIEQ
ASKN--IKIGTKVVEIAQSKQPVPSSKQKHPVETHDSTPKRDEKVKISTDGHPKTLVNDNGETEFIVYTYDQ
KTNQLFSGGNYS--IEYKGNKKRHSSGIHGIGK--KIHKGEIGQKIKITASSGGKEFIAFD-----------
------------------------------------------------------------------------
--GNLSRGMKAFELKIDRSVIPSVSDVIISFKGVNEQSRQAIVSQKTKNVLAYLAKEA--------------
-------------NMDKLYITSTIRTPRAQAEAMYIKATKYAKPGEDVKKVKAECIAKGLGKEATIQKMVEK
IVEFQNKGVRVSKHCVSEEQYRKNNIID--------------------------------------------
-------------------------------------LGTNSNGFGTGNTLNSVGKRFKAVCEKALKDGIIS
GFISADVAGEGAMHIEIIQ-----------------------------------------------------
------------------------------------------------------------------------
---------------------------
>AR_0088_32
----------------------------------------------------------------MSTAVAKS
PAKTIRVTQKGEQSAADWIKGV-------LDGSGSSPAQI---IVAVVIGCIPFVGQGVDVGNVI-------
------------VSIVKIAENPNNKDNWFDLVFNLIAFVPVAGDGLKIVFKQLRSGKAMGAILDAIPSKTMR
GNVEKWFRNLNWNAYTKELQTTSNKIIDGLI-----------------------------------------
------------------------DVFDSWMTRAVLGQARLKTLVVQLRKMKTVANRQIEMVMKDLQLAHKK
ALATPYPNTTAK-APIHKTGSKPLNRTSAPHTQQKGQVLKNTSGNTAKTGSKNTSTKRTSKK----------
------------------------------------------------------------------------
-----------------------------------RSNKELGSGGEHI------------TDYYFVKRKKSR
TK-----------INNNGVLYEY-HDTGHDG--IDHAWYS--------------------------------
------------------------------------------------------------------------
-------NSIGHKYRITDSKATNLA---------------------------------SHRKIMTPKAAMDA
LSMG-----------------LDVYVKSSKEKKTKGALGKTVSDG---------------------AQMSHL
WIANKIGSAKIT--SSHKVKLLEQIEAWKRVEF--KPASERVFKN---------------------------
------------------------------------------------------------------------
--------------------------GKGQTVAVKCPYDRSLVTITGNQFDHHSQC----------------
-------------KGLDEPKCTRSVTSHAITLEFVLPNEMLRE-----------------------------
------------------------------------------------------------------------
------------------------------------------------------------------------
------------------------------------------------------------------------
------------------------------------------------------------------------
---------------------------
```

>Tfe1
------------------------------------------------------------------------
MHWLNFKRYKSDVAKQAVPP---------HLNAAEFARHY---ADKPQTDTEEYLSLSGEMCWDA-------
------------------------------------------------------------------------
------------------------------------------------------------------------
------------------------------------------------------------------------
-----VVLCAHR-SGALSKAKYKQLWQT--------------------------------------------
------------------------------------------------------------------------
-----------------------------------VFDKQYKHFVSPD------------DTEIRTMADMLR
AP-----------QGCFIGIFSLRDAAAPRL--LH-------------------------------------
------------------------------------------------------------------------
----------------------------------------------------------A---MIGTGAGFAA
GNKN----------------------LCIGVGGAVGWENLNLARD---------------------------
------------------------------------------------------------------------
------------------------------------------------------------------------
------------------------------------------------------------------------
-------------LRWQPEGGFLRQGDNEVLRIFYRPFPA--------------------------------
------------------------------------------------------------------------
------------------------------------------------------------------------
------------------------------------------------------------------------
------------------------------------------------------------------------
---------------------------
>AB031_25
------------------------------------------------------MKKTIKFEKDSKSFETNL
AGFIEAVSFKESSSNLLLPLTH-------PINMEAASITN---EMGFIGLFQWGEEALYDLGYYL-------
---------GDQGISYADIKNTDKNSSLRISWKNQFNSNNWVGKWSGKRNINSKKDFLKHPEIQYEIIKEWI
KYLCNQLRNNNFNEYFGTTIQNIEITESGAIAGMHLVGIGGLGAFLGIPKFHGKKQTDGNGTHIKKYIENFG
GFDLEQ------------------CCNRKIYVTLKDNLGLELKDKEVIIVSQNKGKYVSGETKVKVKSDENG
NLPVIVRSPNTE-IKICADGKESNTIIQKANEKQKAILSDFKVSSHPATLE--KNSTPQPKP----QTNKTP
QEVRNEQTQPSTAPEKEKDSSSKDVDF---------------------------------------------
-----------------------------------NIQIVEGDSGKAI----------SNMNFFITYKGNIK
KH-----------TADGHGIKQNIIAEIGQD--IEVSVQG--------------------------------
------------------------------TGHKQVIHHFTANAALKNKTVKVSLPVHSFNISVTQDNKPVP

```
NTLFSIFYRGREISKRTDSRGMLNV---------------------------------R---MLTGFVFGFG
IKGK----SLILSRVEKNTITKAFTVNGSAVHASKAYEANDAKQK---------------------QNENLN
KHKKEKQEEAAK--KQADQAKTQKNSKNEVKQS--NTYTENGGKPLTTVSNQASVTSDTTN-----------
------------------------------------------------------------------YIIYPN
GTIDRINKDATGFAAYYYVANNQPILIGKSRIYTADRWLKKGKKGSGISH----------------------
-------------LVSIREIFGDSGSKPNNKWEKKIGNLNISIHNDTQQQHRYWLSTVAFAAYIGAVCKFGE
KVRFSGFSDRNGSPGGSSSHLNGEVGDIGYIRVDRNNSVGVNFEMADYDHDASLR-----------------
-------------------------------------FVNILISFGWGKTKNMLSEHYPAHLVNKYKLNQGY
ILPRCSQWTNPRHNNHLHLQGLSVTFNKDE------------------------------------------
------------------------------------------------------------------------
---------------------------
>RhsB
------------------------------------------------------------------------
------------------------------------------------AGCAMGEILADADKWSL-------
------------------------------------------------------------------------
------------------------------------------------------------------------
------------------------------------------------------------------------
----------------AKIGDRQKGMIKDKLS----------------------------------------
------------------------------------------------------------------------
-----------------------------------TVKERSKALNTKM------------REHFNANEQKII
SE-----------WEKQTGMNWPTLSSGSRA-----------------------------------------
------------------------------------------------------------------------
---------------------------------------------------------------TPHHVIPIK
NGGS-----------------------------------------------------------------NEW
WNIIPVQHPHTG--TIHGTGSALRTHLPYQKDGG--------------------------------------
------------------------------------------------------------------------
------------------------------------------------------------------------
-------------KLWNLLGY---------------------------------------------------
------------------------------------------------------------------------
------------------------------------------------------------------------
------------------------------------------------------------------------
------------------------------------------------------------------------
---------------------------
```

```
>ZW85-1_19
----------------------------------------------------------------VTDFLPSK
QDLLNALATISSPINKAIKKGQ-------ETARDVADWLW---VVIQGDFAEEQTTAQTVTGTVI-------
--------------SMIPFVDQICDVRDICANCIKIKEDSNNPWPWIGLILTLIGLFPVLGSLFKGIFKVIL
APVRRFMLRPATKALKFTGGNIYKLAEPAIES----------------------------------------
------------------------GITEMNKFLARPAVKRTIKNSNITNIYKAIAKKIREVKAGTNKQALLS
VFDILIDHLKESVKFIEKYGSKAIGVKAKQMLQSVLDIRNLANQKLGEFLK---------------------
------------------------------------------------------------------------
-----------------------------------PVQDFLEKLAVRI----------EKEGDQTFKATTNV
KN-----------IHNFQRIGQNAELKSVLN--NKPVWVD--------------------------------
------------------------------------------------------------------------
-------IVAKEKFKALSESPL----------------------------------------IPKNYPDISE
MSKN--------------------RALRDAFKTFHDLEPVHLSEG----------------EILYRVLDPKS
ADNSICWMRASE--YKKLKSKADWRRYFAVFAS----WNNNGEVV---------------------------
------------------------------------------------------------------------
-----KYRVPKGGMNVWEGPAASQTFKNSAGKVEKVNSKGEIFVLEGGGIQIVLDPN---------------
-------------DLVRSNVSKREATPWGYDSGLIGEAKTSM------------------------------
------------------------------------------------------------------------
-------------------------------------VGVPRLEQNWYGDKK--------------------
------------------------------------------------------------------------
------------------------------------------------------------------------
---------------------------
>Tfe2
------------------------------------------------------------------------
-MPLEVDGIIRGDRGSEPSRWQ------HASTKPLITLTW---HHTIPWNCLRNVWNGLVAGQHW-------
------------------------------------------------------------------------
------------------------------------------------------------------------
------------------------------------------------------------------------
-------------NALDEFMNLIGVPNRAEVITQIKNKNLQDRDGLHTL-----------------------
------------------------------------------------------------------------
-----------------------------------VTWQGWNIVEGPG------------NEYRAQGDDPGE
-------------NFDGWSGKGMSTNQQATL--QQ-------------------------------------
------------------------------------------------------------------------
```

```
--------------------------------------------------------------VNVLYQVMAP
LGSR-------------------------ALDAARQAPNISAEEA-----------------------SVLQ
RTIKQTRPTLRG--KEPIRWQEDMWHQVQPGKE--AKHFARWDTK---------------------------
------------------------------------------------------------------------
------------------------------------------------------------------------
-------------PVWRKRLHSDLAQAG--------------------------------------------
------------------------------------------------------------------------
------------------------------------------------------------------------
------------------------------------------------------------------------
------------------------------------------------------------------------
---------------------------
>Tke9
---------------MDSLSETLELCRRITSGYRPALRNSPVADCTRRFELLPLRYAAVGGNPAQRARLPKL
PSYLSPFQEVGELTHSSYAIRPLREGFLYLLIKRHSAPAYEWHSQYRVAPNGSLLYISADAPWEP-------
-------------APLTGNLDDIIRSFGWTITLYDLDDIQELRPLFSPSPLTPRLLDNYRLLEDEYRNSLPA
IDIARFIQPSDAPPQPHVLKHDQLNWVADFKAQDDADLQALLDLQPFNNDQIVSPHASRLALAPPVNQTKPR
GAAIVIEDAIGITQELNAWRNAAVDDVKTKWLERTVEPGVDNERKLLVAQSFLEIEKLYPQMVAEEIVKREV
MAEKVRNQLHLH-PEIYLFSERARQQADAHDERLKPHMAQFERGVRSKVQARQDAGEFRKRFDQKYGHLVDR
QAMHDQLDGFEQAMQHAQQAAEDRANDHTRWV----------------------------------------
-----------------------------------VSERLLQALDRYD------------NADLINGLAFAE
QT-----------GQCVIGMEL--SEGGTKV--LDHWWRS--------------------------------
---------------------------------------DVADRGNLAIRGITYNQEDLREELAALLEAAKA
EPPSASSFALPEALARQAHAAANAFGR-----------------------------------INTLYEQMQA
QSST-------------ASIGLYAWYVALGRQVLRTATPNSMDRA------------LHQGLRLTLFASVHE
TAVDIRLSEAAR--SGQPINPQRSAGQVSRYLD--QAWAEGLMQARNSDFYKVRVSGLVCL-----------
--------------------------------------------------------------------LEGM
LMAFKARELPDSDARVKTELLAAAMTTAAAGFEVGASYVDQVVARYGANSVTGRGAAAALGRL---------
-------------KLWGASLAVTGGFVLAWWDFADAAQQLIYRQQNLTKHSTKKPGTLALAYASRALATITI
SLAEAGTAIAIAKPLFDHLSQNAKTKLVRMMTTSMGALANKLGTQAARLL----------------------
-------------------------------------LARLVLGAFWIGVILTLIIYILEDDALEKWCKRSC
FRTHKNIKPYEEQEELSALHTAIGEVL---------------------------------------------
------------------------------------------------------------------------
---------------------------
```

```
>Tse2
------------------------------------------------------------------------
---------------MSYDYEK---------------------TSLTLYRAVFKANYDGDVGRYL-------
------------------------------------------------------------------------
------------------------------------------------------------------------
------------------------------------------------------------------------
---------HPD-KELAEAAEVAPLLH---------------------------------------------
------------------------------------------------------------------------
-----------------------------------PTFDSPNTPGVPA-----------RAPDIVAGRDGLY
AP----------------------DTGGTSV-----------------------------------------
------------------------------------------------------------------------
--------------------------------------------------------------FDRAGVLRRA
DGDF--------------------------VIPDGTDIPPDLKVK---------------------------
------QDSYNK--RLQATHYTIMPAKPMYREV---------------------------------------
------------------------------------------------------------------------
------------------------------------------------------------------------
-------------LMGQLDNFVRNAIRRQWEKARGL------------------------------------
------------------------------------------------------------------------
------------------------------------------------------------------------
------------------------------------------------------------------------
------------------------------------------------------------------------
---------------------------
>AF-401_30
----------------------------------------------------------------------MN
DAAIALESFAKQTCGRACVM---------PPIQPVVLDVY---KQSFTAFVGGAADKYQFLGRPL-------
------------------------------------------------------------------------
------------------------------------------------------------------------
------------------------------------------------------------------------
----------DI-DFPDDIGAGPTGIMKSCMRKFKEASSSQITKAKFEYYG--YEEAY--------------
------------------------------------------------------------------------
-----------------------------------STDSVVKRYRGKL------------LLNLLTDIKNIL
KE------------EPNTQINLVGHSLGGWN--VA-------------------------------------
------------------------------------------------------------------------
```

------------------------------------------------------------GLSEELSKAK
ICTV---------------HTLITIDPVGIRLSKSGVGND------------------------RARIYY
LEPDPVAKKWIN--IFSQPLKNYRDDYIAILGG--RWNDDDTAKA-------------------------
----------------------------------------------------------------------
----------------------------------------------------------------------
-------------NFNSVSTYHHGEASEMFFEKKFLENKM------------------------------
----------------------------------------------------------------------
------------------------------------SASDLLVSELKKVIK-------------------
----------------------------------------------------------------------
----------------------------------------------------------------------
--------------------------
>3207_1
---------------------------------------------------------------MDNVIDA
NKDIKKQATELLKKAHGKVSNV------PPVIPVKLTTY---THSLTIFIGGAADKYVFTPG--------
----------------------------------------------------------------------
----------------------------------------------------------------------
----------------------------------------------------------------------
-------------DVPVGWGDFRPIGPTYFVGNYVNNYFATIKGGSKN----------------------
----------------------------------------------------------------------
----------------------------------AFVKYYGYEEAYV---------SDTRELTVNTNKDK
K------------LNAFNDVKNFLKTYPQTQ--VN-----------------------------------
----------------------------------------------------------------------
--------IVGHSLGGWNAAGLAEI-----------------------------------LHKNNICKVN
VLVT-------------------------IDPVGEILSKIGLGS--------------------RTSIYY
SRPKPVFNLWIS--ISCDPKSYEWNNDLIADLG--GQWSSYPSSN-------------------------
----------------------------------------------------------------------
----------------------------SSYYYVTRYSHADFR---------------------------
-------------LMMQEKIVNNLSVQDILSKELSRIK--------------------------------
----------------------------------------------------------------------
----------------------------------------------------------------------
----------------------------------------------------------------------
----------------------------------------------------------------------
--------------------------

```
>AR_0088_1
-----------------------------------------------------------------MDNVIDA
NKDIKKQATELLKKAHGKVSNV-------PPVIPVKLTTY---THSLTIFIGGAADKYVFTPG---------
------------------------------------------------------------------------
------------------------------------------------------------------------
------------------------------------------------------------------------
-------------DVPVGWGDFRPIGPTYFVGNYVNNYFATIKGGSKN------------------------
------------------------------------------------------------------------
-----------------------------------AFVKYYGYEEAYV----------SDTRELTVNTNKDK
K------------LNAFNDVKNFLKTYPQTQ--VN-------------------------------------
------------------------------------------------------------------------
--------IVGHSLGGWNAAGLAEI-------------------------------------LHKNNICKVN
VLVT--------------------------IDPVGEILSKIGLGS---------------------RTSIYY
SRPKPVFNLWIS--ISCDPKSYEWNNDLIADLG--GQWSSYPSSN---------------------------
------------------------------------------------------------------------
-----------------------------SSYYYVTRYSHADFR----------------------------
-------------LMMQEKIVNNLSVQDILSKELSRIK----------------------------------
------------------------------------------------------------------------
------------------------------------------------------------------------
------------------------------------------------------------------------
------------------------------------------------------------------------
---------------------------
>HWAB8_1
-----------------------------------------------------------------MDNVIDA
NKDIKKQATELLKKAHGKVSNV-------PPVIPVKLTTY---THSLTIFIGGAADKYVFTPG---------
------------------------------------------------------------------------
------------------------------------------------------------------------
------------------------------------------------------------------------
-------------DVPVGWGDFRPIGPTYFVGNYVNNYFATIKGGSKN------------------------
------------------------------------------------------------------------
-----------------------------------AFVKYYGYEEAYV----------SDTRELTVNTNKDK
K------------LNAFNDVKNFLKTYPQTQ--VN-------------------------------------
------------------------------------------------------------------------
```

--------IVGHSLGGWNAAGLAEI-------------------------------------LHKNNICKVN
VLVT--------------------------IDPVGEILSKIGLGS---------------------RTSIYY
SRPKPVFNLWIS--ISCDPKSYEWNNDLIADLG--GQWSSYPSSN---------------------------
------------------------------------------------------------------------
-----------------------------SSYYYVTRYSHADFR----------------------------
-------------LMMQEKIVNNLSVQDILSKELSRIK----------------------------------
------------------------------------------------------------------------
------------------------------------------------------------------------
------------------------------------------------------------------------
------------------------------------------------------------------------
---------------------------
>AB042_1
-----------------------------------------------------------------MDNVIDA
NKDIKKQATELLKKAHGKVSNV-------PPVIPVKLTTY---THSLTIFIGGAADKYVFTPG---------
------------------------------------------------------------------------
------------------------------------------------------------------------
------------------------------------------------------------------------
-------------DVPVGWGDFRPIGPTYFVGNYVNNYFATIKGGSKN------------------------
------------------------------------------------------------------------
-----------------------------------AFVKYYGYEEAYV----------SDTRELTVNTNKDK
K------------LNAFNDVKNFLKTYPQTQ--VN-------------------------------------
------------------------------------------------------------------------
--------IVGHSLGGWNAAGLAEI-------------------------------------LHKNNICKVN
VLIT--------------------------IDPVGEILSKIGLGS---------------------RTSIYY
SRPKPVFNLWIS--ISCDPKSYEWNNDLIADLG--GQWSSYPSSN---------------------------
------------------------------------------------------------------------
-----------------------------SSYYYVTRYSHADFR----------------------------
-------------LMMQEKIVNNLSVQDILSKELSRIK----------------------------------
------------------------------------------------------------------------
------------------------------------------------------------------------
------------------------------------------------------------------------
------------------------------------------------------------------------
---------------------------

```
>ATCC17978-mff_1
-----------------------------------------------------------------MDNVIDA
NKDIKKQATELLKKAHGKVSNV-------PPVIPVKLTTY---THSLTIFIGGAADKYVFTPG---------
------------------------------------------------------------------------
------------------------------------------------------------------------
------------------------------------------------------------------------
-------------DVPVGWGDFRPIGPTYFVGNYVNNYFATIKGGSKN------------------------
------------------------------------------------------------------------
-----------------------------------AFVKYYGYEEAYV----------SDTRELTVNTNKDK
K------------LNAFNDVKNFLKTYPQTQ--VN-------------------------------------
------------------------------------------------------------------------
--------IVGHSLGGWNAAGLAEI-------------------------------------LHKNNICKVN
VLIT--------------------------IDPVGEILSKIGLGS---------------------RTSIYY
SRPKPVFNLWIS--ISCDPKSYEWNNDLIADLG--GQWSSYPSSN---------------------------
------------------------------------------------------------------------
-----------------------------SSYYYVTRYSHADFR----------------------------
-------------LMMQEKIVNNLSVQDILSKELSRIK----------------------------------
------------------------------------------------------------------------
------------------------------------------------------------------------
------------------------------------------------------------------------
------------------------------------------------------------------------
---------------------------
>ab736_7
--------------------------------------------------------------MSTSETYLND
EEIFELVRDIPLDTSIIYGEED-------QEFGICPYITF---YIYHQDNEVDEVANKIIDLYEE-------
------------------------------------------------------------------------
------------------------------------------------------------------------
---------------------------------FENEIIDKPFKLRYRDTGVWKNANKWRPSRQVMLDEMHE
SYKKYFVYFIGA-TTGDSGGQSARWALQAIIRDNGLRYTSLKISFGDKWFRENKKKWYAFVE----------
------------------------------------------------------------------------
-----------------------------------NCLIKLNPIQAYS------------GYEIGSPQSFNC
VS-----------PEFETVERIFSDYFYGLD--ID-------------------------------------
------------------------------------------------------------------HPSNMS
```

```
FSHDDPSGLIYTPSLAAGIRTPTWCFL-----------------------------------LSPYWIEKLG
LSEE-----------QIRLKLNDPRIEITKLPDPADPEKYSLWIR---------------------LGELSL
YPIEEGVPDLLV--MANELIKPIRCNDLKLTTL--DAWDDDPNPR---------------------------
------------------------------------------------------------------------
-------------------FDIDNSPQWIARFDEDSHWPEGKRVNKIHAVLLEQ------------------
-------------NAIKVLGGEICPKTGEWYSPANNMKKRYFAEGE--------------------------
------------------------------------------------------------------------
-------------------------------------IMPEIKDNPWGETIWYLEVTNKTE-----------
------------------------------------------------------------------------
------------------------------------------------------------------------
---------------------------
>ACICU_7
--------------------------------------------------------------MSTSETYLND
EEIFELVRDIPLDTSIIYGEED-------QEFGICPYITF---YIYHQDNEVDEVANKIIDLYEE-------
------------------------------------------------------------------------
------------------------------------------------------------------------
---------------------------------FENEIIDKPFKLRYRDTGVWKNANKWRPSRQVMLDEMHE
SYKKYFVYFIGA-TTGDSGGQSARWALQAIIRDNGLRYTSLKISFGDKWFRENKKKWYAFVE----------
------------------------------------------------------------------------
-----------------------------------NCLIKLNPIQAYS------------GYEIGSPQSFNC
VS-----------PEFETVERIFSDYFYGLD--ID-------------------------------------
------------------------------------------------------------------HPSNMS
FSHDDPSGLIYTPSLAAGIRTPTWCFL-----------------------------------LSPYWIEKLG
LSEE-----------QIRLKLNDPRIEITKLPDPADPEKYSLWIR---------------------LGELSL
YPIEEGVPDLLV--MANELIKPIRCNDLKLTTL--DAWDDDPNPR---------------------------
------------------------------------------------------------------------
-------------------FDIDNSPQWIARFDEDSHWPEGKRVNKIHAVLLEQ------------------
-------------NAIKVLGGEICPKTGEWYSPANNMKKRYFAEGE--------------------------
------------------------------------------------------------------------
-------------------------------------IMPEIKDNPWGETIWYLEVTNKTE-----------
------------------------------------------------------------------------
------------------------------------------------------------------------
---------------------------
```

```
>ATCC19606_7
--------------------------------------------------------------MSTSETYLND
EEIFELVRDIPLDTSIIYGEED-------QEFGICPYITF---YIYHQDNEVDEVANKIIDLYEE-------
------------------------------------------------------------------------
------------------------------------------------------------------------
---------------------------------FENEIIDKPFKLRYRDTGVWKNANKWRPSRQVMLDEMHE
SYKKYFVYFIGA-TTGDSGGQSARWALQAIIRDNGLRYTSLKISFGDKWFRENKKKWYAFVE----------
------------------------------------------------------------------------
-----------------------------------NCLIKLNPIQAYS------------GYEIGSPQSFNC
VS-----------PEFETVERIFSDYFYGLD--ID-------------------------------------
------------------------------------------------------------------HPSNMS
FSHDDPSGLIYTPSLAAGIRTPTWCFL-----------------------------------LSPYWIEKLG
LSEE-----------QIRLKLNDPRIEITKLPDPADPEKYSLWIR---------------------LGELSL
YPIEEGVPDLLV--MANELIKPIRCNDLKLTTL--DAWDDDPNPR---------------------------
------------------------------------------------------------------------
-------------------FDIDNSPQWIARFDEDSHWPEGKRVNKIHAVLLEQ------------------
-------------NAIKVLGGEICPKTGEWYSPANNMKKRYFAEGE--------------------------
------------------------------------------------------------------------
-------------------------------------IMPEIKDNPWGETIWYLEVTNKTE-----------
------------------------------------------------------------------------
------------------------------------------------------------------------
---------------------------
>ATCC17978-mff_2
---------------------------------------------------MPDPQNQQFWSKIAERPTEIQ
QQFWYGAEKLAKYLNDNPAMYK--GYMTYDTSKPDPNLSW---WEKILFYSGQAHYNEAVTHYNT-------
-----------------SKVIADQGSWLWGMLKGDFNKDPSMSQIIVGGLISLIPVADQVCDIRDLIANFIT
LSDEKARTKDNYMALALTSVGIIP------------------------------------------------
------------------------EVGSAIKTIVKSSRAKDVSKVKLFKFMEYFEEALTKLKIKCPWGKAPE
AWLRSRPWKGIA-TQAFNTLKNNIDRILSVINKCLIRFNGALKVALAKFQATLNHVLNTIKKYIDQLCDEVD
K-----------------------------------------------------------------------
-----------------------------------AIGRLLPQQPLAM----------AHAGGGGKNAPTGR
YE-----------ANVSSGKKTEASHRQKKE--TPPPPRK--------------------------------
------------------------------------------------------------------MKPKHVV
```

EPCLEPKKGLRNSWGKKTGKSQAE--------------------------------------LDAEFDRQLK
RQEE-----------------GLNRLTVEEYQQNRQLYEKYKRAG---------------------TGTQQQ
RIREDMQRQLEE--SEYKKLKTEQPQLSKGQIE--RMAENNAKKT---------------------------
------------------------------------------------------------------------
------LEGLDVLHNPDMQLGGFDVKYDPKKPPTLDDFGHSGVNRSIGSQMAAKK-----------------
-------------RLANMDAAAAQAKAKGMGKDAMDVELKRCK-----------------------------
------------------------------------------------------------------------
------------------------------------------------------------------------
------------------------------------------------------------------------
------------------------------------------------------------------------
---------------------------
>CIP70.10_2
---------------------------------------------------MPDPQNQQFWSKIAERPTEIQ
QQFWYGAEKLAKYLNDNPAMYK--GYMTYDTSKPDPNLSW---WEKILFYSGQAHYNEAVTHYNT-------
-----------------SKVIADQGSWLWGMLKGDFNKDPSMSQIIVGGLISLIPVADQVCDIRDLIANFIT
LSDEKARTKDNYMALALTSVGIIP------------------------------------------------
------------------------EVGSAIKTIVKSSRAKDVSKVKLFKFMEYFEEALTKLKIKCPWGKAPE
AWLRSRPWKGIA-TQAFNTLKNNIDRILSVINKCLIRFNGALKVALAKFQATLNHVLNTIKKYIDQLCDEVD
K-----------------------------------------------------------------------
-----------------------------------AIGRLLPQQPLAM----------AHAGGGGKNAPTGR
YE-----------ANVSSGKKTEASHRQKKE--TPPPPRK--------------------------------
-----------------------------------------------------------------MKPKHVV
EPCLEPKKGLRNSWGKKTGKSQAE--------------------------------------LDAEFDRQLK
RQEE-----------------GLNRLTVEEYQQNRQLYEKYKRAG---------------------TGTQQQ
RIREDMQRQLEE--SEYKKLKTEQPQLSKGQIE--RMAENNAKKT---------------------------
------------------------------------------------------------------------
------LEGLDVLHNPDMQLGGFDVKYDPKKPPTLDDFGHSGVNRSIGSQMAAKK-----------------
-------------RLANMDAAAAQAKAKGMGKDAMDVELKRCK-----------------------------
------------------------------------------------------------------------
------------------------------------------------------------------------
------------------------------------------------------------------------
------------------------------------------------------------------------
---------------------------

```
>R2091_2
---------------------------------------------------MPDPQNQQFWSKIAERPTEIQ
QQFWYGAEKLAKYLNDNPAMYK--GYMTYDTSKPDPNLSW---WEKILFYSGQAHYNEAVTHYNT-------
-----------------SKVIADQGSWLWGMLKGDFNKDPSMSQIIVGGLISLIPVADQVCDIRDLIANFIT
LSDEKARTKDNYMALALTSVGIIP------------------------------------------------
------------------------EVGSAIKTIVKSSRAKDVSKVKLFKFMEYFEEALTKLKIKCPWGKAPE
AWLRSRPWKGIA-TQAFNTLKNNIDRILSVINKCLIRFNGALKVALAKFQATLNHVLNTIKKYIDQLCDEVD
K-----------------------------------------------------------------------
-----------------------------------AIGRLLPQQPLAM----------AHAGGGGKNAPTGR
YE-----------ANVSSGKKTEASHRQKKE--TPPPPRK--------------------------------
-----------------------------------------------------------------MKPKHVV
EPCLEPKKGLRNSWGKKTGKSQAE--------------------------------------LDAEFDRQLK
RQEE-----------------GLNRLTVEEYQQNRQLYEKYKRAG---------------------TGTQQQ
RIREDMQRQLEE--SEYKKLKTEQPQLSKGQIE--RMAENNAKKT---------------------------
------------------------------------------------------------------------
------LEGLDVLHNPDMQLGGFDVKYDPKKPPTLDDFGHSGVNRSIGSQMAAKK-----------------
-------------RLANMDAAAAQAKAKGMGKDAMDVELKRCK-----------------------------
------------------------------------------------------------------------
------------------------------------------------------------------------
------------------------------------------------------------------------
------------------------------------------------------------------------
---------------------------
>TseF
------------------------------------------------------------------------
-------------------------------MAASGKLDL---VRGDSHLAGNLLDGSLEGPLRI-------
------------------------------------------------------------------------
------------------------------------------------------------------------
------------------------------------------------------------------------
---------DEH-DRPQASLGYRQGQLHGASTLFHPNGKVSAQLAF--------------------------
------------------------------------------------------------------------
-----------------------------------VDGKLHGPASFHA------------AEGWLQRKAHYR
NG-----------LLHGEAFNYFANGQVAER--EH--YRD--------------------------------
------------------------------------------------------------------------
```

```
--------------------------------------------------------------GVRDGVYQRF
HGNG-------------------------QLAFDGRYLNGQLLDG---------------------------
--AQPFADDGRP--LDSEGKPMARWRW----------WWTRLAEP---------------------------
------------------------------------------------------------------------
------------------------------------------------------------------------
------------------------------------------------------------------------
------------------------------------------------------------------------
------------------------------------------------------------------------
------------------------------------------------------------------------
------------------------------------------------------------------------
---------------------------
>CIP70.10_31
---------------------------------------------------MVDLNKDDVALITQAIAIGFS
PAGVALDVADLARASYNLYQEK-------SDDNYFEVILC---VIGFIPGPGDGLKAGLRIVNRK-------
-------------------PEILFELIRFIMTHCKIYGNPEQWLSEIISDTKIRELIRSG------------
------------------------------------------------------------------------
------------------------KREALNASNKNIGNRWAKYWINQSIEITFNFLEASISSLVQLLARKVL
HWKTKIPKSSAD-KRHVGGGKGHQNNSAHGTTYDGKTGQNGNGHG---------------------------
------------------------------------------------------------------------
-----------------------------------SKGKTLSKVDISK----------VLSNLEVGGVGEHM
AD-----------YWVANKLAIPAVHDSGQQ--LT-------------------------------------
------------------------------------------------------------------------
-------PKLQRPMTKLHIGIHDQG-------------------------------------IDAIWKSDKK
NIGI-------------------MNTKSYAIIEAKASLSMGKGTG--------------PGSLLNDLDKQQD
TRERHIERASAK--KEKRAEKKLIPKKKFLINQ--QMSDEWVKKR---------------------------
------------------------------------------------------------------------
-----------------------LRATSLRNVEIAGFSRHLLYFNYAHPETVEHL-----------------
-------------NVLAISIQDNQPVDHSEHLNEHTPSNFWGAAK---------------------------
------------------------------------------------------------------------
-------------------------------------IDEALNNRVTRANIKNGHTK---------------
------------------------------------------------------------------------
------------------------------------------------------------------------
---------------------------
```

```
>R2091_31
---------------------------------------------------MVDLNKDDVALITQAIAIGFS
PAGVALDVADLARASYNLYQEK-------SDDNYFEVILC---VIGFIPGPGDGLKAGLRIVNRK-------
-------------------PEILFELIRFIMTHCKIYGNPEQWLSEIISDTKIRELIRSG------------
------------------------------------------------------------------------
------------------------KREALNASNKNIGNRWAKYWINQSIEITFNFLEASISSLVQLLARKVL
HWKTKIPKSSAD-KRHVGGGKGHQNNSAHGTTYDGKTGQNGNGHG---------------------------
------------------------------------------------------------------------
-----------------------------------SKGKTLSKVDISK----------VLSNLEVGGVGEHM
AD-----------YWVANKLAIPAVHDSGQQ--LT-------------------------------------
------------------------------------------------------------------------
-------PKLQRPMTKLHIGIHDQG-------------------------------------IDAIWKSDKK
NIGI-------------------MNTKSYAIIEAKASLSMGKGTG--------------PGSLLNDLDKQQD
TRERHIERASAK--KEKRAEKKLIPKKKFLINQ--QMSDEWVKKR---------------------------
------------------------------------------------------------------------
-----------------------LRATSLRNVEIAGFSRHLLYFNYAHPETVEHL-----------------
-------------NVLAISIQDNQPVDHSEHLNEHTPSNFWGAAK---------------------------
------------------------------------------------------------------------
-------------------------------------IDEALNNRVTRANIKNGHTK---------------
------------------------------------------------------------------------
------------------------------------------------------------------------
---------------------------
>Tse5
------------------------------------------------------------------------
----------------------------IIPLVVIGAFAA---RAAIGAALGAGIELGMQTGKQV-------
------------------------------------------------------------------------
------------------------------------------------------------------------
------------------------------------------------------------------------
------------------------------------------------------------------------
------------------------------------------------------------------------
--------------------------------------------LGQM------------KDNWDSDRDLTD
IK-----------------------------------WKC--------------------------------
------------------------------------------------------------------------
```

--------------------------------------------------------------IDINWKHVGA
SAAI------------------------------GTVAPGMLSTG---------------------KTVVQS
AKAIRTLSGQAA--NTANRAAKLAARKAAHADT----IKKAVATQ---------------------------
------------------------------------------------------------------------
------------------------------------------------------------------------
-------------AAWQTGKQIVKCPLKDEEEECPPQ-----------------------------------
------------------------------------------------------------------------
------------------------------------------------------------------------
------------------------------------------------------------------------
------------------------------------------------------------------------
---------------------------

>VasX
MSNPNQAAKTGQTNDAQNPASACPFKQPLIGIIPVRYAFDVYDDQGQALHPLPKADRQWKGQFSIKQRSYTL
RQLRDGWLYVYDETAKTLHEYE-------VVGCKLTKIDW---SDDEANKPTHERGSKGESKSCL-------
---------------LYPAQHTLSIGYAHQRWTWRVCEHMRSNTSSRHAVMRKVSLKQFESNGTHPHAHFAQ
YLEDYVADIGTPAEQDIFKDTCTPSLPVEKSEEAVKGTEFKFVADKAVVSSSDYLQDLPEQNCGLFVALNDP
LADVSDLFVTFTTQVAKRTKAI--GDETQQHKMQMAELTRTLGRIRLEEKEIPDFVKQDPIRILELERAITE
YCATAKLAEIES-HHLASEGHSPSGNYALMQQQAEQKLAELKTLYRFEPTSAQMRKWRKKDNSFIDEVRWAD
LDNFLVEHYTELKGLDEQIKQHYAQFMSAFNQLGLDPLLFGMDNQDEVQQAYLLALTSQFLVVVTQVNHDEK
SL---------------------------------EILKKDLSFDSPK----------NLMALASTGFSLQA
NQAINNHIQGFSTAFLSTSNPSDMVAFATAI--AN--WDT--------------------------------
------------------------------------------------------FTGDERIQEKAWFKRWIE
PAQSSFGALQKAVANQAKESWQAVM---------------------------------E---LLFPYQNQPK
GGTP-------------SLLANLRLLLVESLVREEAVLQHNPKYAAELKQFETKLNAILQEMNDALELKPGN
VSPKNHQIATAQ--SAQRKLGQLLSSELPMMLT--LKNQAAMNTFQQSVNEKLSALSKNVK-----------
-------------------------------------------------------------TSSASVSQKLG
GLGGLLFALNLWNTMTVLENIRYKVAQYPSWNPFKNPALGEAIYATGNTIVVAGAISAGRAWV---------
-------------TIAEQGLLDRTLKNALNTTKVLGTKDALKTFAKSIALVATVGMIASALETWESWGKFND
SSKTDLERFGYLLKAGATGAQGIIFYIQFFTLLGSGIGGPSIAAISAGWMLAGFAV----------------
-------------------------------------IGIVYLIGVILTNVFKRSELEIWLSKSTWGKESAH
WPVGKELTELEHLLHRPSLRLSQVTQRKAAQWMDSGSLQWQLELTLPDYLKGQTIGLQITRLPAQPAYYQPQ
REAVTPILINEQQGKWSIEDNQPVYRITLGGSEKDTVGVCVALPLRWGKELSLKFYASGTRAGELDLQSAEA
NDIATRNLVVGKG--------------

```
>15A5_28
------------------------------------------------------------------------
--MGNNSTTSTTTNTANPPTQK-------VPLKPEAELHL---LVGSAYGEGDRESPYGHTAVYI-------
------------------------------------------------------------------------
------------------------------------------------------------------------
------------------------------------------------------------------------
-------KLKGK-DYIYDFGRYGKTKPESFGIFTLEGANSPRGE----------------------------
------------------------------------------------------------------------
-----------------------------------GILRIWSSFDAYI------------ADENSQGKGTSK
TR-----------TTHAYGYRIF-DSQAMLV--LN--YYN--------------------------------
------------------------------------------------------------------------
-------NLLKTATLRRNEKYYKSY---------------------------------A---LSQDYFALAP
NCTT------------------------QSLEATKKAIPSMAKSG------------------------HMF
VNAEKVLPTTVK--LAFKASKYEMPKYLFLPDN----LNDYLMKS---------------------------
------------------------------------------------------------------------
------------------------------------------------------------------------
-------------PDVKVDIKNTYHLKK--------------------------------------------
------------------------------------------------------------------------
------------------------------------------------------------------------
------------------------------------------------------------------------
------------------------------------------------------------------------
---------------------------
>Ab4568_28
------------------------------------------------------------------------
--MGNNSTTSTTTNTANPPTQK-------VPLKPEAELHL---LVGSAYGEGDRESPYGHTAVYI-------
------------------------------------------------------------------------
------------------------------------------------------------------------
------------------------------------------------------------------------
-------KLKGK-DYIYDFGRYGKTKPESFGIFTLEGANSPRGE----------------------------
------------------------------------------------------------------------
-----------------------------------GILRIWSSFDAYI------------ADENSQGKGTSK
TR-----------TTHAYGYRIF-DSQAMLV--LN--YYN--------------------------------
------------------------------------------------------------------------
```

-------NLLKTATLRRNEKYYKSY---------------------------------A---LSQDYFALAP
NCTT------------------------QSLEATKKAIPSMAKSG------------------------HMF
VNAEKVLPTTVK--LAFKASKYEMPKYLFLPDN----LNDYLMKS---------------------------
------------------------------------------------------------------------
------------------------------------------------------------------------
-------------PDVKVDIKNTYHLKK--------------------------------------------
------------------------------------------------------------------------
------------------------------------------------------------------------
------------------------------------------------------------------------
------------------------------------------------------------------------
---------------------------
>Ab4977_28
------------------------------------------------------------------------
--MGNNSTTSTTTNTANPPTQK-------VPLKPEAELHL---LVGSAYGEGDRESPYGHTAVYI-------
------------------------------------------------------------------------
------------------------------------------------------------------------
------------------------------------------------------------------------
-------KLKGK-DYIYDFGRYGKTKPESFGIFTLEGANSPRGE----------------------------
------------------------------------------------------------------------
-----------------------------------GILRIWSSFDAYI------------ADENSQGKGTSK
TR-----------TTHAYGYRIF-DSQAMLV--LN--YYN--------------------------------
------------------------------------------------------------------------
-------NLLKTATLRRNEKYYKSY---------------------------------A---LSQDYFALAP
NCTT------------------------QSLEATKKAIPSMAKSG------------------------HMF
VNAEKVLPTTVK--LAFKASKYEMPKYLFLPDN----LNDYLMKS---------------------------
------------------------------------------------------------------------
------------------------------------------------------------------------
-------------PDVKVDIKNTYHLKK--------------------------------------------
------------------------------------------------------------------------
------------------------------------------------------------------------
------------------------------------------------------------------------
------------------------------------------------------------------------
---------------------------

>JBA13_28
------------------------------------------------------------------------
--MGNNSTTSTTTNTANPPTQK-------VPLKPEAELHL---LVGSAYGEGDRESPYGHTAVYI-------
------------------------------------------------------------------------
------------------------------------------------------------------------
------------------------------------------------------------------------
-------KLKGK-DYIYDFGRYGKTKPESFGIFTLEGANSPRGE----------------------------
------------------------------------------------------------------------
-----------------------------------GILRIWSSFDAYI------------ADENSQGKGTSK
TR-----------TTHAYGYRIF-DSQAMLV--LN--YYN--------------------------------
------------------------------------------------------------------------
-------NLLKTATLRRNEKYYKSY---------------------------------A---LSQDYFALAP
NCTT------------------------QSLEATKKAIPSMAKSG------------------------HMF
VNAEKVLPTTVK--LAFKASKYEMPKYLFLPDN----LNDYLMKS---------------------------
------------------------------------------------------------------------
------------------------------------------------------------------------
-------------PDVKVDIKNTYHLKK--------------------------------------------
------------------------------------------------------------------------
------------------------------------------------------------------------
------------------------------------------------------------------------
------------------------------------------------------------------------
---------------------------
>KAB04_28
------------------------------------------------------------------------
--MGNNSTTSTTTNTANPPTQK-------VPLKPEAELHL---LVGSAYGEGDRESPYGHTAVYI-------
------------------------------------------------------------------------
------------------------------------------------------------------------
------------------------------------------------------------------------
-------KLKGK-DYIYDFGRYGKTKPESFGIFTLEGANSPRGE----------------------------
------------------------------------------------------------------------
-----------------------------------GILRIWSSFDAYI------------ADENSQGKGTSK
TR-----------TTHAYGYRIF-DSQAMLV--LN--YYN--------------------------------
------------------------------------------------------------------------

```
-------NLLKTATLRRNEKYYKSY---------------------------------A---LSQDYFALAP
NCTT------------------------QSLEATKKAIPSMAKSG------------------------HMF
VNAEKVLPTTVK--LAFKASKYEMPKYLFLPDN----LNDYLMKS---------------------------
------------------------------------------------------------------------
------------------------------------------------------------------------
-------------PDVKVDIKNTYHLKK--------------------------------------------
------------------------------------------------------------------------
------------------------------------------------------------------------
------------------------------------------------------------------------
------------------------------------------------------------------------
---------------------------
>KAB07_28
------------------------------------------------------------------------
--MGNNSTTSTTTNTANPPTQK-------VPLKPEAELHL---LVGSAYGEGDRESPYGHTAVYI-------
------------------------------------------------------------------------
------------------------------------------------------------------------
------------------------------------------------------------------------
-------KLKGK-DYIYDFGRYGKTKPESFGIFTLEGANSPRGE----------------------------
------------------------------------------------------------------------
-----------------------------------GILRIWSSFDAYI------------ADENSQGKGTSK
TR-----------TTHAYGYRIF-DSQAMLV--LN--YYN--------------------------------
------------------------------------------------------------------------
-------NLLKTATLRRNEKYYKSY---------------------------------A---LSQDYFALAP
NCTT------------------------QSLEATKKAIPSMAKSG------------------------HMF
VNAEKVLPTTVK--LAFKASKYEMPKYLFLPDN----LNDYLMKS---------------------------
------------------------------------------------------------------------
------------------------------------------------------------------------
-------------PDVKVDIKNTYHLKK--------------------------------------------
------------------------------------------------------------------------
------------------------------------------------------------------------
------------------------------------------------------------------------
------------------------------------------------------------------------
---------------------------
```

```
>KAB08_28
------------------------------------------------------------------------
--MGNNSTTSTTTNTANPPTQK-------VPLKPEAELHL---LVGSAYGEGDRESPYGHTAVYI-------
------------------------------------------------------------------------
------------------------------------------------------------------------
------------------------------------------------------------------------
-------KLKGK-DYIYDFGRYGKTKPESFGIFTLEGANSPRGE----------------------------
------------------------------------------------------------------------
-----------------------------------GILRIWSSFDAYI------------ADENSQGKGTSK
TR-----------TTHAYGYRIF-DSQAMLV--LN--YYN--------------------------------
------------------------------------------------------------------------
-------NLLKTATLRRNEKYYKSY---------------------------------A---LSQDYFALAP
NCTT------------------------QSLEATKKAIPSMAKSG------------------------HMF
VNAEKVLPTTVK--LAFKASKYEMPKYLFLPDN----LNDYLMKS---------------------------
------------------------------------------------------------------------
------------------------------------------------------------------------
-------------PDVKVDIKNTYHLKK--------------------------------------------
------------------------------------------------------------------------
------------------------------------------------------------------------
------------------------------------------------------------------------
------------------------------------------------------------------------
---------------------------
>KBN10P02143_28
------------------------------------------------------------------------
--MGNNSTTSTTTNTANPPTQK-------VPLKPEAELHL---LVGSAYGEGDRESPYGHTAVYI-------
------------------------------------------------------------------------
------------------------------------------------------------------------
------------------------------------------------------------------------
-------KLKGK-DYIYDFGRYGKTKPESFGIFTLEGANSPRGE----------------------------
------------------------------------------------------------------------
-----------------------------------GILRIWSSFDAYI------------ADENSQGKGTSK
TR-----------TTHAYGYRIF-DSQAMLV--LN--YYN--------------------------------
------------------------------------------------------------------------
```

```
-------NLLKTATLRRNEKYYKSY-------------------------------A---LSQDYFALAP
NCTT-----------------------QSLEATKKAIPSMAKSG-----------------------HMF
VNAEKVLPTTVK--LAFKASKYEMPKYLFLPDN----LNDYLMKS-------------------------
----------------------------------------------------------------------
----------------------------------------------------------------------
-------------PDVKVDIKNTYHLKK------------------------------------------
----------------------------------------------------------------------
----------------------------------------------------------------------
----------------------------------------------------------------------
----------------------------------------------------------------------
--------------------------
>SAA14_28
----------------------------------------------------------------------
--MGNNSTTSTTTNTANPPTQK-------VPLKPEAELHL---LVGSAYGEGDRESPYGHTAVYI-------
----------------------------------------------------------------------
----------------------------------------------------------------------
----------------------------------------------------------------------
-------KLKGK-DYIYDFGRYGKTKPESFGIFTLEGANSPRGE--------------------------
----------------------------------------------------------------------
----------------------------------GILRIWSSFDAYI-----------ADENSQGKGTSK
TR-----------TTHAYGYRIF-DSQAMLV--LN--YYN------------------------------
----------------------------------------------------------------------
-------NLLKTATLRRNEKYYKSY-------------------------------A---LSQDYFALAP
NCTT-----------------------QSLEATKKAIPSMAKSG-----------------------HMF
VNAEKVLPTTVK--LAFKASKYEMPKYLFLPDN----LNDYLMKS-------------------------
----------------------------------------------------------------------
----------------------------------------------------------------------
-------------PDVKVDIKNTYHLKK------------------------------------------
----------------------------------------------------------------------
----------------------------------------------------------------------
----------------------------------------------------------------------
----------------------------------------------------------------------
--------------------------
```

>SMC_Paed_Ab_BL01_28
------------------------------------------------------------------------
--MGNNSTTSTTTNTANPPTQK-------VPLKPEAELHL---LVGSAYGEGDRESPYGHTAVYI-------
------------------------------------------------------------------------
------------------------------------------------------------------------
------------------------------------------------------------------------
-------KLKGK-DYIYDFGRYGKTKPESFGIFTLEGANSPRGE----------------------------
------------------------------------------------------------------------
-----------------------------------GILRIWSSFDAYI------------ADENSQGKGTSK
TR-----------TTHAYGYRIF-DSQAMLV--LN--YYN--------------------------------
------------------------------------------------------------------------
-------NLLKTATLRRNEKYYKSY---------------------------------A---LSQDYFALAP
NCTT------------------------QSLEATKKAIPSMAKSG------------------------HMF
VNAEKVLPTTVK--LAFKASKYEMPKYLFLPDN----LNDYLMKS---------------------------
------------------------------------------------------------------------
------------------------------------------------------------------------
-------------PDVKVDIKNTYHLKK--------------------------------------------
------------------------------------------------------------------------
------------------------------------------------------------------------
------------------------------------------------------------------------
------------------------------------------------------------------------
---------------------------
>SSA12_28
------------------------------------------------------------------------
--MGNNSTTSTTTNTANPPTQK-------VPLKPEAELHL---LVGSAYGEGDRESPYGHTAVYI-------
------------------------------------------------------------------------
------------------------------------------------------------------------
------------------------------------------------------------------------
-------KLKGK-DYIYDFGRYGKTKPESFGIFTLEGANSPRGE----------------------------
------------------------------------------------------------------------
-----------------------------------GILRIWSSFDAYI------------ADENSQGKGTSK
TR-----------TTHAYGYRIF-DSQAMLV--LN--YYN--------------------------------
------------------------------------------------------------------------

-------NLLKTATLRRNEKYYKSY---------------------------------A---LSQDYFALAP
NCTT------------------------QSLEATKKAIPSMAKSG------------------------HMF
VNAEKVLPTTVK--LAFKASKYEMPKYLFLPDN----LNDYLMKS---------------------------
------------------------------------------------------------------------
------------------------------------------------------------------------
-------------PDVKVDIKNTYHLKK--------------------------------------------
------------------------------------------------------------------------
------------------------------------------------------------------------
------------------------------------------------------------------------
------------------------------------------------------------------------
---------------------------
>SSMA17_28
------------------------------------------------------------------------
--MGNNSTTSTTTNTANPPTQK-------VPLKPEAELHL---LVGSAYGEGDRESPYGHTAVYI-------
------------------------------------------------------------------------
------------------------------------------------------------------------
------------------------------------------------------------------------
-------KLKGK-DYIYDFGRYGKTKPESFGIFTLEGANSPRGE----------------------------
------------------------------------------------------------------------
-----------------------------------GILRIWSSFDAYI------------ADENSQGKGTSK
TR-----------TTHAYGYRIF-DSQAMLV--LN--YYN--------------------------------
------------------------------------------------------------------------
-------NLLKTATLRRNEKYYKSY---------------------------------A---LSQDYFALAP
NCTT------------------------QSLEATKKAIPSMAKSG------------------------HMF
VNAEKVLPTTVK--LAFKASKYEMPKYLFLPDN----LNDYLMKS---------------------------
------------------------------------------------------------------------
------------------------------------------------------------------------
-------------PDVKVDIKNTYHLKK--------------------------------------------
------------------------------------------------------------------------
------------------------------------------------------------------------
------------------------------------------------------------------------
------------------------------------------------------------------------
---------------------------

```
>YU-R612_28
------------------------------------------------------------------------
--MGNNSTTSTTTNTANPPTQK-------VPLKPEAELHL---LVGSAYGEGDRESPYGHTAVYI-------
------------------------------------------------------------------------
------------------------------------------------------------------------
------------------------------------------------------------------------
-------KLKGK-DYIYDFGRYGKTKPESFGIFTLEGANSPRGE----------------------------
------------------------------------------------------------------------
-----------------------------------GILRIWSSFDAYI------------ADENSQGKGTSK
TR-----------TTHAYGYRIF-DSQAMLV--LN--YYN--------------------------------
------------------------------------------------------------------------
-------NLLKTATLRRNEKYYKSY---------------------------------A---LSQDYFALAP
NCTT------------------------QSLEATKKAIPSMAKSG------------------------HMF
VNAEKVLPTTVK--LAFKASKYEMPKYLFLPDN----LNDYLMKS---------------------------
------------------------------------------------------------------------
------------------------------------------------------------------------
-------------PDVKVDIKNTYHLKK--------------------------------------------
------------------------------------------------------------------------
------------------------------------------------------------------------
------------------------------------------------------------------------
------------------------------------------------------------------------
---------------------------
>RhsA
------------------------------------------------------------------------
---------------AKCPTLA----------HGANGEIL---SAKATVSKAELRTGSGTNQSSR-------
------------------------------------------------------------------------
------------------------------------------------------------------------
------------------------------------------------------------------------
-------------DYARSLGN---------------------------------------------------
------------------------------------------------------------------------
-----------------------------------QTDDAGHILGNVL------------GGQGGKGNVFPQ
LP-----------AINRGQYRDFEKVVKDYI--GQ--HGS--------------------------------
------------------------------------------------------------------------
```

```
--------------------------------------------------------------VDIEWAFKYG
NGGT--------------------------------------------------------------RPTEIY
YDVYQNGQKVFG------------------------------------------------------------
------------------------------------------------------------------------
------------------------------------------------------------------------
-------------RIFNN------------------------------------------------------
------------------------------------------------------------------------
------------------------------------------------------------------------
------------------------------------------------------------------------
------------------------------------------------------------------------
---------------------------
>Tke4
------------------------------------------------------------------------
--------------------------------CPENVKEY-------------DITPYRPSNSPL-------
------------------------------------------------------------------------
------------------------------------------------------------------------
------------------------------------------------------------------------
-------------ENHH-------------------------------------------------------
------------------------------------------------------------------------
-----------------------------------GILDIWAAQNVPS-----------YGGKYPKGSPTIV
LS-----------SKNHAATKAVYREWLREK--TG-------------------------------------
------------------------------------------------------------------------
----------------------------------------------------------KPVGGKVDWSQISN
REVM--------------------------------------------------------------QLSERM
FDVAGVPATSRT--SYYRALNRYLHTGTF-------------------------------------------
------------------------------------------------------------------------
------------------------------------------------------------------------
------------------------------------------------------------------------
------------------------------------------------------------------------
------------------------------------------------------------------------
------------------------------------------------------------------------
------------------------------------------------------------------------
---------------------------
```

```
>A1_16
------------------------------------------------------------------------
-----------------------------------CSTTL---NRNLGGVKGDHLQAHHIIPEEI-------
------------------------------------------------------------------------
------------------------------------------------------------------------
------------------------------------------------------------------------
-------WAKRK-DFLDDIGI---------------------------------------------------
------------------------------------------------------------------------
-----------------------------------GGNRDKAENGVLM-------------PDSEAKAKQMK
RQ-----------LYHCGSHPIYSAGINQKL--GQ-------------------------------------
------------------------------------------------------------------------
--------------------------------------------------------------IQREFESKKI
TASQ--------------------------------------------------------------------
----------AR--DKVANLQSSMRLVLITPGT---------------------------------------
------------------------------------------------------------------------
------------------------------------------------------------------------
-------------KPIRLS-----------------------------------------------------
------------------------------------------------------------------------
------------------------------------------------------------------------
------------------------------------------------------------------------
------------------------------------------------------------------------
---------------------------
>A388_16
------------------------------------------------------------------------
-----------------------------------CSTTL---NRNLGGVKGDHLQAHHIIPEEI-------
------------------------------------------------------------------------
------------------------------------------------------------------------
------------------------------------------------------------------------
-------WAKRK-DFLDDIGI---------------------------------------------------
------------------------------------------------------------------------
-----------------------------------GGNRDKAENGVLM-------------PDSEAKAKQMK
RQ-----------LYHCGSHPIYSAGINQKL--GQ-------------------------------------
------------------------------------------------------------------------
```

```
--------------------------------------------------------------IQREFESKKI
TASQ--------------------------------------------------------------------
----------AR--DKVANLQSSMRLVLITPGT---------------------------------------
------------------------------------------------------------------------
------------------------------------------------------------------------
-------------KPIRLS-----------------------------------------------------
------------------------------------------------------------------------
------------------------------------------------------------------------
------------------------------------------------------------------------
------------------------------------------------------------------------
---------------------------
>A85_16
------------------------------------------------------------------------
-----------------------------------CSTTL---NRNLGGVKGDHLQAHHIIPEEI-------
------------------------------------------------------------------------
------------------------------------------------------------------------
------------------------------------------------------------------------
-------WAKRK-DFLDDIGI---------------------------------------------------
------------------------------------------------------------------------
-----------------------------------GGNRDKAENGVLM-------------PDSEAKAKQMK
RQ-----------LYHCGSHPIYSAGINQKL--GQ-------------------------------------
------------------------------------------------------------------------
--------------------------------------------------------------IQREFESKKI
TASQ--------------------------------------------------------------------
----------AR--DKVANLQSSMRLVLITPGT---------------------------------------
------------------------------------------------------------------------
------------------------------------------------------------------------
-------------KPIRLS-----------------------------------------------------
------------------------------------------------------------------------
------------------------------------------------------------------------
------------------------------------------------------------------------
------------------------------------------------------------------------
---------------------------
```

```
>AB0057_16
------------------------------------------------------------------------
-----------------------------------CSTTL---NRNLGGVKGDHLQAHHIIPEEI-------
------------------------------------------------------------------------
------------------------------------------------------------------------
------------------------------------------------------------------------
-------WAKRK-DFLDDIGI---------------------------------------------------
------------------------------------------------------------------------
-----------------------------------GGNRDKAENGVLM-------------PDSEAKAKQMK
RQ-----------LYHCGSHPIYSAGINQKL--GQ-------------------------------------
------------------------------------------------------------------------
--------------------------------------------------------------IQREFESKKI
TASQ--------------------------------------------------------------------
----------AR--DKVANLQSSMRLVLITPGT---------------------------------------
------------------------------------------------------------------------
------------------------------------------------------------------------
-------------KPIRLS-----------------------------------------------------
------------------------------------------------------------------------
------------------------------------------------------------------------
------------------------------------------------------------------------
------------------------------------------------------------------------
---------------------------
>AB307-0294_16
------------------------------------------------------------------------
-----------------------------------CSTTL---NRNLGGVKGDHLQAHHIIPEEI-------
------------------------------------------------------------------------
------------------------------------------------------------------------
------------------------------------------------------------------------
-------WAKRK-DFLDDIGI---------------------------------------------------
------------------------------------------------------------------------
-----------------------------------GGNRDKAENGVLM-------------PDSEAKAKQMK
RQ-----------LYHCGSHPIYSAGINQKL--GQ-------------------------------------
------------------------------------------------------------------------
```

```
--------------------------------------------------------------IQREFESKKI
TASQ--------------------------------------------------------------------
----------AR--DKVANLQSSMRLVLITPGT---------------------------------------
------------------------------------------------------------------------
------------------------------------------------------------------------
-------------KPIRLS-----------------------------------------------------
------------------------------------------------------------------------
------------------------------------------------------------------------
------------------------------------------------------------------------
------------------------------------------------------------------------
---------------------------
>AB5075-UW_16
------------------------------------------------------------------------
-----------------------------------CSTTL---NRNLGGVKGDHLQAHHIIPEEI-------
------------------------------------------------------------------------
------------------------------------------------------------------------
------------------------------------------------------------------------
-------WAKRK-DFLDDIGI---------------------------------------------------
------------------------------------------------------------------------
-----------------------------------GGNRDKAENGVLM-------------PDSEAKAKQMK
RQ-----------LYHCGSHPIYSAGINQKL--GQ-------------------------------------
------------------------------------------------------------------------
--------------------------------------------------------------IQREFESKKI
TASQ--------------------------------------------------------------------
----------AR--DKVANLQSSMRLVLITPGT---------------------------------------
------------------------------------------------------------------------
------------------------------------------------------------------------
-------------KPIRLS-----------------------------------------------------
------------------------------------------------------------------------
------------------------------------------------------------------------
------------------------------------------------------------------------
------------------------------------------------------------------------
---------------------------
```

```
>AR_0083_16
------------------------------------------------------------------------
-----------------------------------CSTTL---NRNLGGVKGDHLQAHHIIPEEI-------
------------------------------------------------------------------------
------------------------------------------------------------------------
------------------------------------------------------------------------
-------WAKRK-DFLDDIGI---------------------------------------------------
------------------------------------------------------------------------
-----------------------------------GGNRDKAENGVLM-------------PDSEAKAKQMK
RQ-----------LYHCGSHPIYSAGINQKL--GQ-------------------------------------
------------------------------------------------------------------------
--------------------------------------------------------------IQREFESKKI
TASQ--------------------------------------------------------------------
----------AR--DKVANLQSSMRLVLITPGT---------------------------------------
------------------------------------------------------------------------
------------------------------------------------------------------------
-------------KPIRLS-----------------------------------------------------
------------------------------------------------------------------------
------------------------------------------------------------------------
------------------------------------------------------------------------
------------------------------------------------------------------------
---------------------------
>D36_16
------------------------------------------------------------------------
-----------------------------------CSTTL---NRNLGGVKGDHLQAHHIIPEEI-------
------------------------------------------------------------------------
------------------------------------------------------------------------
------------------------------------------------------------------------
-------WAKRK-DFLDDIGI---------------------------------------------------
------------------------------------------------------------------------
-----------------------------------GGNRDKAENGVLM-------------PDSEAKAKQMK
RQ-----------LYHCGSHPIYSAGINQKL--GQ-------------------------------------
------------------------------------------------------------------------
```

```
--------------------------------------------------------------IQREFESKKI
TASQ--------------------------------------------------------------------
----------AR--DKVANLQSSMRLVLITPGT---------------------------------------
------------------------------------------------------------------------
------------------------------------------------------------------------
-------------KPIRLS-----------------------------------------------------
------------------------------------------------------------------------
------------------------------------------------------------------------
------------------------------------------------------------------------
------------------------------------------------------------------------
---------------------------
>USA15_16
------------------------------------------------------------------------
-----------------------------------CSTTL---NRNLGGVKGDHLQAHHIIPEEI-------
------------------------------------------------------------------------
------------------------------------------------------------------------
------------------------------------------------------------------------
-------WAKRK-DFLDDIGI---------------------------------------------------
------------------------------------------------------------------------
-----------------------------------GGNRDKAENGVLM-------------PDSEAKAKQMK
RQ-----------LYHCGSHPIYSAGINQKL--GQ-------------------------------------
------------------------------------------------------------------------
--------------------------------------------------------------IQREFESKKI
TASQ--------------------------------------------------------------------
----------AR--DKVANLQSSMRLVLITPGT---------------------------------------
------------------------------------------------------------------------
------------------------------------------------------------------------
-------------KPIRLS-----------------------------------------------------
------------------------------------------------------------------------
------------------------------------------------------------------------
------------------------------------------------------------------------
------------------------------------------------------------------------
---------------------------
```

>WCHAB005078_16
------------------------------------------------------------------------
-----------------------------------CSTTL---NRNLGGVKGDHLQAHHIIPEEI-------
------------------------------------------------------------------------
------------------------------------------------------------------------
------------------------------------------------------------------------
-------WAKRK-DFLDDIGI---------------------------------------------------
------------------------------------------------------------------------
-----------------------------------GGNRDKAENGVLM-------------PDSEAKAKQMK
RQ-----------LYHCGSHPIYSAGINQKL--GQ-------------------------------------
------------------------------------------------------------------------
--------------------------------------------------------------IQREFESKKI
TASQ--------------------------------------------------------------------
----------AR--DKVANLQSSMRLVLITPGT---------------------------------------
------------------------------------------------------------------------
------------------------------------------------------------------------
-------------KPIRLS-----------------------------------------------------
------------------------------------------------------------------------
------------------------------------------------------------------------
------------------------------------------------------------------------
------------------------------------------------------------------------
---------------------------
>AB030_24
--------------------------------------------------MKNGKSINQKSYPLTRYGFICA
VSRMESSSDLSLPLTAPVNIRA-------SQNKNSRGYIG---FFQFGEAALIDLGYYKHWNDNS-------
---------------DKTKANDWTGSWVGHNGVNSLSDFLKSPAKQIQIIGEWIDLLCKRLRNRSFNEYYGK
IINGIEITESGAIAGAHLVGEGGLGSFLGISGFKGNYKEVDGNNVHISRYIEMFNHYDLESCCSRKIYII--
------------------------LKNQIGQIAKNKKVTIESEYSGKFKQSKFIVSAESDEQGLLPVIIRHP
GSKIIIKVDGKQ-SAVITQASDKKQSIELSVSEDIKIQSVLQKPAEPEPKQEKVVVVEQKTVDLKKDEVKTN
APKDIKFDIAIIESDTNRKI----------------------------------------------------
-----------------------------------TNMRFFIRYKGKI------------KEHTSDSSGIKT
GIIAEEGENLDILVNGSKGYQKLKTISITKG--MENSCIS--------------------------------
-----------------------------------------------------------------------V

```
PLGLVSVKLKIHDSKGKILKNNKFF-------------------------------------VSYRGREIEK
ITDS-----------NGFIQLKMLNAFVYKLLLSNKKPILTLRND---------------PSISVINVNLNS
AATTIQMPRVASNPPSKTVPAKTKPAKETSSKR-EESFVDYLIDFIPSLGKTENVHTEKNG-----------
------------------------------------------------------------------------
----NPLTKQYGSDVVFTIKTINKETGKEENLAYSIKYNGAKRTHYTGQDGIGLKKHRGEEGKKIEIVVDVD
SKEQVLYTAILKNPMPVIEIRMDKPKDDDSYLFPLKARTNSYKNGFARFGSNRSGGKRKHAGCDLYAPTGTE
IRAMADGKVRVVKGFYSGTDVIEIVHKKHIIRYGEVLA----------------------------------
-------------------------------------GKSLVKVGDDVKRGQTIGYVGKLTVQVPSMMLHLE
MYSNPKDTSPLTVRGNNAYQRRSDLIDPTTFLDNSTL-----------------------------------
------------------------------------------------------------------------
---------------------------
>AbH12O-A2_24
--------------------------------------------------MKNGKSINQKSYPLTRYGFICA
VSRMESSSDLSLPLTAPVNIRA-------SQNKNSRGYIG---FFQFGEAALIDLGYYKHWNDNS-------
---------------DKTKANDWTGSWVGHNGVNSLSDFLKSPAKQIQIIGEWIDLLCKRLRNRSFNEYYGK
IINGIEITESGAIAGAHLVGEGGLGSFLGISGFKGNYKEVDGNNVHISRYIEMFNHYDLESCCSRKIYII--
------------------------LKNQIGQIAKNKKVTIESEYSGKFKQSKFIVSAESDEQGLLPVIIRHP
GSKIIIKVDGKQ-SAVITQASDKKQSIELSVSEDIKIQSVLQKPAEPEPKQEKVVVVEQKTVDLKKDEVKTN
APKDIKFDIAIIESDTNRKI----------------------------------------------------
-----------------------------------TNMRFFIRYKGKI------------KEHTSDSSGIKT
GIIAEEGENLDILVNGSKGYQKLKTISITKG--MENSCIS--------------------------------
-----------------------------------------------------------------------V
PLGLVSVKLKIHDSKGKILKNNKFF-------------------------------------VSYRGREIEK
ITDS-----------NGFIQLKMLNAFVYKLLLSNKKPILTLRND---------------PSISVINVNLNS
AATTIQMPRVASNPPSKTVPAKTKPAKETSSKR-EESFVDYLIDFIPSLGKTENVHTEKNG-----------
------------------------------------------------------------------------
----NPLTKQYGSDVVFTIKTINKETGKEENLAYSIKYNGAKRTHYTGQDGIGLKKHRGEEGKKIEIVVDVD
SKEQVLYTAILKNPMPVIEIRMDKPKDDDSYLFPLKARTNSYKNGFARFGSNRSGGKRKHAGCDLYAPTGTE
IRAMADGKVRVVKGFYSGTDVIEIVHKKHIIRYGEVLA----------------------------------
-------------------------------------GKSLVKVGDDVKRGQTIGYVGKLTVQVPSMMLHLE
MYSNPKDTSPLTVRGNNAYQRRSDLIDPTTFLDNSTL-----------------------------------
------------------------------------------------------------------------
---------------------------
```

```
>AF-401_24
--------------------------------------------------MKNGKSINQKSYPLTRYGFICA
VSRMESSSDLSLPLTAPVNIRA-------SQNKNSRGYIG---FFQFGEAALIDLGYYKHWNDNS-------
---------------DKTKANDWTGSWVGHNGVNSLSDFLKSPAKQIQIIGEWIDLLCKRLRNRSFNEYYGK
IINGIEITESGAIAGAHLVGEGGLGSFLGISGFKGNYKEVDGNNVHISRYIEMFNHYDLESCCSRKIYII--
------------------------LKNQIGQIAKNKKVTIESEYSGKFKQSKFIVSAESDEQGLLPVIIRHP
GSKIIIKVDGKQ-SAVITQASDKKQSIELSVSEDIKIQSVLQKPAEPEPKQEKVVVVEQKTVDLKKDEVKTN
APKDIKFDIAIIESDTNRKI----------------------------------------------------
-----------------------------------TNMRFFIRYKGKI------------KEHTSDSSGIKT
GIIAEEGENLDILVNGSKGYQKLKTISITKG--MENSCIS--------------------------------
-----------------------------------------------------------------------V
PLGLVSVKLKIHDSKGKILKNNKFF-------------------------------------VSYRGREIEK
ITDS-----------NGFIQLKMLNAFVYKLLLSNKKPILTLRND---------------PSISVINVNLNS
AATTIQMPRVASNPPSKTVPAKTKPAKETSSKR-EESFVDYLIDFIPSLGKTENVHTEKNG-----------
------------------------------------------------------------------------
----NPLTKQYGSDVVFTIKTINKETGKEENLAYSIKYNGAKRTHYTGQDGIGLKKHRGEEGKKIEIVVDVD
SKEQVLYTAILKNPMPVIEIRMDKPKDDDSYLFPLKARTNSYKNGFARFGSNRSGGKRKHAGCDLYAPTGTE
IRAMADGKVRVVKGFYSGTDVIEIVHKKHIIRYGEVLA----------------------------------
-------------------------------------GKSLVKVGDDVKRGQTIGYVGKLTVQVPSMMLHLE
MYSNPKDTSPLTVRGNNAYQRRSDLIDPTTFLDNSTL-----------------------------------
------------------------------------------------------------------------
---------------------------
>AR_0101_24
--------------------------------------------------MKNGKSINQKSYPLTRYGFICA
VSRMESSSDLSLPLTAPVNIRA-------SQNKNSRGYIG---FFQFGEAALIDLGYYKHWNDNS-------
---------------DKTKANDWTGSWVGHNGVNSLSDFLKSPAKQIQIIGEWIDLLCKRLRNRSFNEYYGK
IINGIEITESGAIAGAHLVGEGGLGSFLGISGFKGNYKEVDGNNVHISRYIEMFNHYDLESCCSRKIYII--
------------------------LKNQIGQIAKNKKVTIESEYSGKFKQSKFIVSAESDEQGLLPVIIRHP
GSKIIIKVDGKQ-SAVITQASDKKQSIELSVSEDIKIQSVLQKPAEPEPKQEKVVVVEQKTVDLKKDEVKTN
APKDIKFDIAIIESDTNRKI----------------------------------------------------
-----------------------------------TNMRFFIRYKGKI------------KEHTSDSSGIKT
GIIAEEGENLDILVNGSKGYQKLKTISITKG--MENSCIS--------------------------------
-----------------------------------------------------------------------V
```

```
PLGLVSVKLKIHDSKGKILKNNKFF-------------------------------------VSYRGREIEK
ITDS-----------NGFIQLKMLNAFVYKLLLSNKKPILTLRND---------------PSISVINVNLNS
AATTIQMPRVASNPPSKTVPAKTKPAKETSSKR-EESFVDYLIDFIPSLGKTENVHTEKNG-----------
------------------------------------------------------------------------
----NPLTKQYGSDVVFTIKTINKETGKEENLAYSIKYNGAKRTHYTGQDGIGLKKHRGEEGKKIEIVVDVD
SKEQVLYTAILKNPMPVIEIRMDKPKDDDSYLFPLKARTNSYKNGFARFGSNRSGGKRKHAGCDLYAPTGTE
IRAMADGKVRVVKGFYSGTDVIEIVHKKHIIRYGEVLA----------------------------------
-------------------------------------GKSLVKVGDDVKRGQTIGYVGKLTVQVPSMMLHLE
MYSNPKDTSPLTVRGNNAYQRRSDLIDPTTFLDNSTL-----------------------------------
------------------------------------------------------------------------
---------------------------
>AR_0063_24
--------------------------------------------------MKNGKSINQKSYPLTRYGFICA
VSRMESSSDLSLPLTAPVNIRA-------SQNKNSRGYIG---FFQFGEAALIDLGYYKHWNDNS-------
---------------DKTKANDWTGSWVGHNGVNSLSDFLKSPAKQIQIIGEWIDLLCKRLRNRSFNEYYGK
IINGIEITESGAIAGAHLVGEGGLGSFLGISGFKGNYKEVDGNNVHISRYIEMFNHYDLESCCSRKIYIILL
------------------------LKNQIGQIAKNKKVTIESEYSGKFKQSKFIVSAESDEQGLLPVIIRHP
GSKIIIKVDGKQ-SAVITQASDKKQSIELSVSEDIKIQSVLQKPAEPEPKQEKVVVVEQKTVDLKKDEVKTN
APKDIKFDIAIIESDTNRKI----------------------------------------------------
-----------------------------------TNMRFFIRYKGKI------------KEHTSDSSGIKT
GIIAEEGENLDILVNGSKGYQKLKTISITKG--MENSCIS--------------------------------
-----------------------------------------------------------------------V
PLGLVSVKLKIHDSKGKILKNNKFF-------------------------------------VSYRGREIEK
ITDS-----------NGFIQLKMLNAFVYKLLLSNKKPILTLRND---------------PSISVINVNLNS
AATTIQMPRVASNPPSKTVPAKTKPAKETSSKR-EESFVDYLIDFIPSLGKTENVHTEKNG-----------
------------------------------------------------------------------------
----NPLTKQYGSDVVFTIKTINKETGKEENLAYSIKYNGAKRTHYTGQDGIGLKKHRGEEGKKIEIVVDVD
SKEQVLYTAILKNPMPVIEIRMDKPKDDDSYLFPLKARTNSYKNGFARFGSNRSGGKRKHAGCDLYAPTGTE
IRAMADGKVRVVKGFYSGTDVIEIVHKKHIIRYGEVLA----------------------------------
-------------------------------------GKSLVKVGDDVKRGQTIGYVGKLTVQVPSMMLHLE
MYSNPKDTSPLTVRGNNAYQRRSDLIDPTTFLDNSTL-----------------------------------
------------------------------------------------------------------------
---------------------------
```

>ZW85-1_20
---------------------------------------MDKKMSENQKMILGKSKSNNLFNEAIAQQKQKQ
SQIPVMGKTKAVGDMRNPTVAK-------NINKKIEESEI---AKSAKQSTAANMQNSSAGQAQK-------
---------------ECSNFCRKKGFNILPLRYTVVKDNPPALPPTLGKNVKDIQLSHLKYAVEMINTGYIY
RLVKRTSGALEWAGYKVTPNGHLSYFPVGKEA------------PKSVPEFACKGAGHSFNSSVIAVES---
------------------------NPKDEAKIAYIIHTHVPMSKAKISEYEKNADKFVTEGKWQKILINGGS
AQEHCIAASQFK-TTVYNLNSFGKNRIDATLNKFKDEPKSLACMALYDPVG--ITRKLNTLR----NSEFKK
VMDFLAKKDGDFTNEHRLHSSQCIDSIKICLENKLLNERSQFSAQATKSI----------------------
-----------------------------------VLMGLWNELPKNL----------TDADDINYELEKIY
KR-----------KSKAEQERLLKEAQEKQR--ANKGIYT--------------------------------
---------EDMGKLSRGEGEQIWVKKYKPNLDMVAKSNFDKKINALIQECLAGARAIGDDHIKWLLSKAVI
NALHAYDDQVQVPFGNIFYVHAMDM----------------------INGMSGVPSGQA---LLETWLGVEK
IAKE---NIYMRAICYNQESLKNKYNAAAPSIIDISWDDGQNAAK---------------------TAIAAF
VAADKAWQEWAA--DKTNNGYMLHGQKFSLLKT--FYWMSELTQSAVKWSMKYTPGSEFAK-----------
-------------------------------------------------------------KLGRLTFYTFA
HSGALVEHVAKNSLYYLIPVQTLTGRVKLTPEMVQNNWKKYSSSATNPSKAAKQIDRIIANSSPSAGLRVSG
VVAIFE-------LINLGTQISKFNIDPSVDNTLSYMSGMMATTAAILEVGSSSMEKFSFAERAAYIAKCGA
YFAIAGAIVSFFIDLKGLNKAFEESNGYAFALNLLKAASSFGTLA-VG------------------------
-------------------------------------FSAFLNLPIMRNSTLYSMVERYAVGRALLGIKVVR
VGAMLSLAGIALVALEIYLKNYVLDNAMQDWCQKCAFKLKKENGEDAFKDVKEEEKEFNNAVVSV-------
------------------------------------------------------------------------
---------------------------
>D1279779_12
-------------------------------------------MSENQKMILGKSKSNNLLNEIIAQQKQKQ
SQIPVIGKTKAVGDMRNPTVAK-------NINKKIEESEI---AKSAKQSTAANMQNSSAGQAQK-------
---------------ECSNFCRKKGFNILPLRYTVVKDNPPALPATLGKNVKDIQLSHLKYAVEMIDTGYIY
RLVKRTSGALEWAGYKVTPNGHLSYFPVGKEA------------PKSVPEFACKGAGHSFNSSVIAVES---
------------------------NPKDEAKIAYIIHTHVPMSKAKISEYEKNADKFVTEGKWQKILINGGS
AQEHCIAASQFK-TTVYNLNSFGKNRIDATLNKFKDEPKSLACMALYDPVG--ITRKLNDSR----NSQFGK
LTNYLAKNVEKISNEHRLQSSQLVDSIKIVVENKLINQQLSARSQVADLSAKIEYAKDKIPKFGSMDQREYL
S----------------------------------KIQEIWDRAPTAE------------RDKFLLQRNKKF
AE-----------GEQKLKEKIITQSKAEAK--IT--WET--------------------------------
--------------------------KYAPKINWDTKKAFDKEVKKLINEGRTLAQTYASDHIKWLKSKHLL

NAFYVYDQTILKEYGALFHVHALAV---------MDGMTGCDEGQ-------------A---LMEKWLGVEE
ISDD---NLYMRAVTYNQKNLIDQYNAKSSEITSLTWDQTQSSLK---------------------SLIAAF
VSADQLWEQWLA--DPNNKINELKWYNPAKSLL----WISEITRSVAKWSLQYNINPLIAK-----------
-------------------------------------------------------------RLSRVSFIAYA
HSSLLTDKVPKWSLLYNVDFKMTNASTQLNVTEIERRWKNAGQNTAAQKIKEILSKSPASVAIRISSIVALF
E------------LINFGVQSNKFIGERNWENGFQLISGLLASSAAVLDIAGGGMDALSFQEKANKIRLYGA
RLAVSAAIISFCIDAFAVYKEKEGDKDKILMAFLFTKATVSLVMVGVG------------------------
-------------------------------------FGLLLNLPILQNSWVVSSANKIAFTRILLRANFIR
IAAYCNWIGIGFLVIEYLLKTYVLDDDLQKWCEKSIFGKDSSKFKLFKDEEEAFSKAIMSI-----------
------------------------------------------------------------------------
---------------------------

>R2090_12

-------------------------------------------MSENQKMILGKSKSNNLLNEIIAQQKQKQ
SQIPVIGKTKAVGDMRNPTVAK-------NINKKIEESEI---AKSAKQSTAANMQNSSAGQAQK-------
---------------ECSNFCRKKGFNILPLRYTVVKDNPPALPATLGKNVKDIQLSHLKYAVEMIDTGYIY
RLVKRTSGALEWAGYKVTPNGHLSYFPVGKEA------------PKSVPEFACKGAGHSFNSSVIAVES---
------------------------NPKDEAKIAYIIHTHVPMSKAKISEYEKNADKFVTEGKWQKILINGGS
AQEHCIAASQFK-TTVYNLNSFGKNRIDATLNKFKDEPKSLACMALYDPVG--ITRKLNDSR----NSQFGK
LTNYLAKNVEKISNEHRLQSSQLVDSIKIVVENKLINQQLSARSQVADLSAKIEYAKDKIPKFGSMDQREYL
S----------------------------------KIQEIWDRAPTAE------------RDKFLLQRNKKF
AE-----------GEQKLKEKIITQSKAEAK--IT--WET--------------------------------
--------------------------KYAPKINWDTKKAFDKEVKKLINEGRTLAQTYASDHIKWLKSKHLL
NAFYVYDQTILKEYGALFHVHALAV---------MDGMTGCDEGQ-------------A---LMEKWLGVEE
ISDD---NLYMRAVTYNQKNLIDQYNAKSSEITSLTWDQTQSSLK---------------------SLIAAF
VSADQLWEQWLA--DPNNKINELKWYNPAKSLL----WISEITRSVAKWSLQYNINPLIAK-----------
-------------------------------------------------------------RLSRVSFIAYA
HSSLLTDKVPKWSLLYNVDFKMTNASTQLNVTEIERRWKNAGQNTAAQKIKEILSKSPASVAIRISSIVALF
E------------LINFGVQSNKFIGERNWENGFQLISGLLASSAAVLDIAGGGMDALSFQEKANKIRLYGA
RLAVSAAIISFCIDAFAVYKEKEGDKDKILMAFLFTKATVSLVMVGVG------------------------
-------------------------------------FGLLLNLPILQNSWVVSSANKIAFTRILLRANFIR
IAAYCNWIGIGFLVIEYLLKTYVLDDDLQKWCEKSIFGKDSSKFKLFKDEEEAFSKAIMSI-----------
------------------------------------------------------------------------
---------------------------

>Ab04-mff_12
-------------------------------------------MSENQKMILGKSKSNNLFNEAIAQQKQKQ
SQIPVMGKTKAVGDMRNPTVAK-------NINKKIEESEI---AKSAKQSTAANMQNSSAGQAQK-------
---------------ECSNFCRKKGFNILPLRYTVVKDNPPALPPTLGKNVKDIQLSHLKYAVEMINTGYIY
RLVKRTSGALEWAGYKVTPNGHLSYFPVGKEA------------PKSVPEFACKGAGHSFNSSVIAVES---
------------------------NPKDEAKIAYIIHTHVPMSKAKISEYEKNADKFVTEGKWQKILINGGS
AQEHCIAASQFK-TTVYNLNSFGKNRIDATLNKFKDEPKSLACMALYDPVG--ITRKLNDSR----NSQFGK
LTNYLAKNVEKISNEHRLQSSQLVDSIKIVVENKLINQQLSARSQIADLSAKIEYAKDKIPKFGSMDQREYL
S----------------------------------KIQEIWDRAPTAE------------RDKFLLQRNKKF
AE-----------GEQKLKEKIITQSKAEAK--IT--WET--------------------------------
--------------------------KYAPKINWDTKKAFDKEVKKLINEGRTLAQTYASDHMKWLKSKHLL
NAFYVYDQTILKEYGALFHVHALAV---------MDGMTGCDEGQ-------------A---LMEKWLGVEE
ISDD---NLYMRAVTYNQKNLIDQYNAKSSEITSLTWDQTQSSLK---------------------SLIAAF
VSADQLWEQWLA--DPNNKINELKWYNPAKSLL----WISEITRSVAKWSLQYNINPLIAK-----------
-------------------------------------------------------------RLSRVSFIAYA
HSSLLTDRVPKWSLLYNVDFKMTNASTQLNVTEIERRWKNAGQNTAAQKIKEILSKSPASVAIRISSIVALF
E------------LINFGVQSNKFIGERNWENGFQLISGLLASSAAVLDIAGGGMDALSFQEKANKIRLYGA
RLAVSAAIISFCIDAFAVYKEKEGDKDKILMAFLFTKATVSLVMVGVG------------------------
-------------------------------------FGLLLNLPILQNSWVVSSANKIAFTRILLRANFIR
IAAYCNWIGIGFLVIEYLLKTYVLDDDLQKWCEKSIFGKDSSKFKLFKDEEEAFSKAIMSI-----------
------------------------------------------------------------------------
---------------------------
>AR_0088_12
-------------------------------------------MSENQKMILGKSKSNNLFNEAIAQQKQKQ
SQIPVMGKTKAVGDMRNPTVAK-------NINKKIEESEI---AKSAKQSTAANMQNSSAGQAQK-------
---------------ECSNFCRKKGFNILPLRYTVVKDNPPALPPTLGKNVKDIQLSHLKYAVEMINTGYIY
RLVKRTSGALEWAGYKVTPNGHLSYFPVGKEA------------PKSVPEFACKGAGHSFNSSVIAVES---
------------------------NPKDEAKIAYIIHTHVPMSKAKISEYEKNADKFVTEGKWQKILINGGS
AQEHCIAASQFK-TTVYNLNSFGKNRIDATLNKFKDEPKSLACMALYDPVG--ITRKLNDSR----NSQFGK
LTNYLAKNVEKISNEHRLQSSQLVDSIKIVVENKLINQQLSARSQVADLSAKIEYAKDKIPKFGSMDQREYL
S----------------------------------KIQEIWDRAPTAE------------RDKFLLQRNKKF
AE-----------GEQKLKEKIITQSKAEAK--IT--WET--------------------------------
--------------------------KYAPKINWDTKKAFDKEVKKLINEGRTLAQTYASDHIKWLKSKHLL

NAFYVYDQTILKEYGALFHVHALAV---------MDGMTGCDEGQ-------------A---LMEKWLGVEE
ISDD---NLYMRAVTYNQKNLIDQYNAKSSEITSLTWDQTQSSLK---------------------SLIAAF
VSADQLWEQWLA--DPNNKINELKWYNPAKSLL----WISEITRSVAKWSLQYNINPLIAK-----------
-------------------------------------------------------------RLSRVSFIAYA
HSSLLTDRVPKWSLLYNVDFKMTNASTQLNVTEIERRWKNAGQNTAAQKIKEILSKSPASVAIRISSIVALF
E------------LINFGVQSNKFIGERNWENGFQLISGLLASSAAVLDIAGGGMDALSFQEKANKIRLYGA
RLAVSAAIISFCIDAFAVYKEKEGDKDILMAFLFTKATVSLVMVGVG-------------------------
-------------------------------------FGLLLNLPILQNSWVVSSANKIAFTRILLRANFIR
IAAYCNWIGIGFLVIEYLLKTYVLDDDLQKWCEKSIFGKDSSKFKLFKDEEEAFSKAIMSI-----------
------------------------------------------------------------------------
---------------------------

>CIP70.10_12
-------------------------------------------MSENQKMILGKSKSNNLFNEAIAQQKQKQ
SQIPVMGKTKAVGDMRNPTVAK-------NINKKIEESEI---AKSAKQSTAANMQNSSAGQAQK-------
---------------ECSNFCRKKGFNILPLRYTVVKDNPPALPPTLGKNVKDIQLSHLKYAVEMINTGYIY
RLVKRTSGALEWAGYKVTPNGHLSYFPVGKEA------------PKSVPEFACKGAGHSFNSSVIAVES---
------------------------NPKDEAKIAYIIHTHVPMSKAKISEYEKNADKFVTEGKWQKILINGGS
AQEHCIAASQFK-TTVYNLNSFGKNRIDATLNKFKDEPKSLACMALYDPVG--ITRKLNDSR----NSQFGK
LTNYLAKNVEKISNEHRLQSSQLVDSIKIVVENKLINQQLSARSQVADLSAKIEYAKDKIPKFGSMDQREYL
S----------------------------------KIQEIWDRAPTAE------------RDKFLLQRNKKF
AE-----------GEQKLKEKIITQSKAEAK--IT--WET--------------------------------
--------------------------KYAPKINWDTKKAFDKEVKKLINEGRTLAQTYASDHIKWLKSKHLL
NAFYVYDQTILKEYGALFHVHALAV---------MDGMTGCDEGQ-------------A---LMEKWLGVEE
ISDD---NLYMRAVTYNQKNLIDQYNAKSSEITSLTWDQTQSSLK---------------------SLIAAF
VSADQLWEQWLA--DPNNKINELKWYNPAKSLL----WISEITRSVAKWSLQYNINPLIAK-----------
-------------------------------------------------------------RLSRVSFIAYA
HSSLLTDRVPKWSLLYNVDFKMTNASTQLNVTEIERRWKNAGQNTAAQKIKEILSKSPASVAIRISSIVALF
E------------LINFGVQSNKFIGERNWENGFQLISGLLASSAAVLDIAGGGMDALSFQEKANKIRLYGA
RLAVSAAIISFCIDAFAVYKEKEGDKDILMAFLFTKATVSLVMVGVG-------------------------
-------------------------------------FGLLLNLPILQNSWVVSSANKIAFTRILLRANFIR
IAAYCNWIGIGFLVIEYLLKTYVLDDDLQKWCEKSIFGKDSSKFKLFKDEEEAFSKAIMSI-----------
------------------------------------------------------------------------
---------------------------

```
>HWBA8_12
-------------------------------------------MSENQKMILGKSKSNNLFNEAIAQQKQKQ
SQIPVMGKTKAVGDMRNPTVAK-------NINKKIEESEI---AKSAKQSTAANMQNSSAGQAQK-------
---------------ECSNFCRKKGFNILPLRYTVVKDNPPALPPTLGKNVKDIQLSHLKYAVEMINTGYIY
RLVKRTSGALEWAGYKVTPNGHLSYFPVGKEA------------PKSVPEFACKGAGHSFNSSVIAVES---
------------------------NPKDEAKIAYIIHTHVPMSKAKISEYEKNADKFVTEGKWQKILINGGS
AQEHCIAASQFK-TTVYNLNSFGKNRIDATLNKFKDEPKSLACMALYDPVG--ITRKLNDSR----NSQFGK
LTNYLAKNVEKISNEHRLQSSQLVDSIKIVVENKLINQQLSARSQVADLSAKIEYAKDKIPKFGSMDQREYL
S----------------------------------KIQEIWDRAPTAE------------RDKFLLQRNKKF
AE-----------GEQKLKEKIITQSKAEAK--IT--WET--------------------------------
--------------------------KYAPKINWDTKKAFDKEVKKLINEGRTLAQTYASDHIKWLKSKHLL
NAFYVYDQTILKEYGALFHVHALAV---------MDGMTGCDEGQ-------------A---LMEKWLGVEE
ISDD---NLYMRAVTYNQKNLIDQYNAKSSEITSLTWDQTQSSLK---------------------SLIAAF
VSADQLWEQWLA--DPNNKINELKWYNPAKSLL----WISEITRSVAKWSLQYNINPLIAK-----------
-------------------------------------------------------------RLSRVSFIAYA
HSSLLTDRVPKWSLLYNVDFKMTNASTQLNVTEIERRWKNAGQNTAAQKIKEILSKSPASVAIRISSIVALF
E------------LINFGVQSNKFIGERNWENGFQLISGLLASSAAVLDIAGGGMDALSFQEKANKIRLYGA
RLAVSAAIISFCIDAFAVYKEKEGDKDKILMAFLFTKATVSLVMVGVG------------------------
-------------------------------------FGLLLNLPILQNSWVVSSANKIAFTRILLRANFIR
IAAYCNWIGIGFLVIEYLLKTYVLDDDLQKWCEKSIFGKDSSKFKLFKDEEEAFSKAIMSI-----------
------------------------------------------------------------------------
---------------------------
>R2091_12
-------------------------------------------MSENQKMILGKSKSNNLFNEAIAQQKQKQ
SQIPVMGKTKAVGDMRNPTVAK-------NINKKIEESEI---AKSAKQSTAANMQNSSAGQAQK-------
---------------ECSNFCRKKGFNILPLRYTVVKDNPPALPPTLGKNVKDIQLSHLKYAVEMINTGYIY
RLVKRTSGALEWAGYKVTPNGHLSYFPVGKEA------------PKSVPEFACKGAGHSFNSSVIAVES---
------------------------NPKDEAKIAYIIHTHVPMSKAKISEYEKNADKFVTEGKWQKILINGGS
AQEHCIAASQFK-TTVYNLNSFGKNRIDATLNKFKDEPKSLACMALYDPVG--ITRKLNDSR----NSQFGK
LTNYLAKNVEKISNEHRLQSSQLVDSIKIVVENKLINQQLSARSQVADLSAKIEYAKDKIPKFGSMDQREYL
S----------------------------------KIQEIWDRAPTAE------------RDKFLLQRNKKF
AE-----------GEQKLKEKIITQSKAEAK--IT--WET--------------------------------
--------------------------KYAPKINWDTKKAFDKEVKKLINEGRTLAQTYASDHIKWLKSKHLL
```

NAFYVYDQTILKEYGALFHVHALAV---------MDGMTGCDEGQ-------------A---LMEKWLGVEE
ISDD---NLYMRAVTYNQKNLIDQYNAKSSEITSLTWDQTQSSLK---------------------SLIAAF
VSADQLWEQWLA--DPNNKINELKWYNPAKSLL----WISEITRSVAKWSLQYNINPLIAK-----------
-------------------------------------------------------------RLSRVSFIAYA
HSSLLTDRVPKWSLLYNVDFKMTNASTQLNVTEIERRWKNAGQNTAAQKIKEILSKSPASVAIRISSIVALF
E------------LINFGVQSNKFIGERNWENGFQLISGLLASSAAVLDIAGGGMDALSFQEKANKIRLYGA
RLAVSAAIISFCIDAFAVYKEKEGDKDKILMAFLFTKATVSLVMVGVG------------------------
-------------------------------------FGLLLNLPILQNSWVVSSANKIAFTRILLRANFIR
IAAYCNWIGIGFLVIEYLLKTYVLDDDLQKWCEKSIFGKDSSKFKLFKDEEEAFSKAIMSI-----------
------------------------------------------------------------------------
---------------------------

>LAC-4_12

-------------------------------------------MSENQKMILGKSKSNNLFNEAIAQQKQKQ
SQIPVMGKTKAVGDMRNPTVAK-------NINKKIEESEI---AKSAKQSTAANMQNSSAGQAQK-------
---------------ECSNFCRKKGFNILPLRYTVVKDNPPALPPTLGKNVKDIQLSHLKYAVEMINTGYIY
RLVKRTSGALEWAGYKVTPNGHLSYFPVGKEA------------PKSVPEFACKGAGHSFNSSVIAVES---
------------------------NPKDEAKIAYIIHTHVPMSKAKISEYEKNADKFVTEGKWQKILINGGS
AQEHCIAASQFK-TTVYNLNSFGKNRIDATLNKFKDEPKSLACMALYDPVG--ITRKLNDSR----NSQFGK
LTNYLAKNVEKISNEHRLQSSQLVDSIKIVVENKLINQQLSARSQIADLSAKIEYAKDKIPKFGSMDQREYL
S----------------------------------KIQEIWDRAPTAE------------RDKFLLQRNKKF
AE-----------GEQKLKEKIITQSKAEAK--IT--WET--------------------------------
--------------------------KYAPKINWDTKKAFDKEVKKLINEGRTLAQTYASDHIKWLKSKHLL
NAFYVYDQTILKEYGALFHVHALAV---------MDGMTGCDEGQ-------------A---LMEKWLGVEE
ISDD---NLYMRAVTYNQKNLIDQYNAKSSEITSLTWDQTQSSLK---------------------SLIAAF
VSADQLWEQWLA--DPNNKINELKWYNPAKSLL----WISEITRSVAKWSLQYNINPLIAK-----------
-------------------------------------------------------------RLSRVSFIAYA
HSSLLTDRVPKWSLLYNVDFKMTNASTQLNVTEIERRWKNAGQNTAAQKIKEILSKSPASVAIRISSIVALF
E------------LINFGVQSNKFIGERNWENGFQLISGLLASSAAVLDIAGGGMDALSFQEKANKIRLYGA
RLAVSAAIISFCIDAFAVYKEKEGDKDKILMAFLFTKATVSLVMVGVG------------------------
-------------------------------------FGLLLNLPILQNSWVVSSANKIAFTRILLRANFIR
IAAYCNWIGIGFLVIEYLLKTYVLDDDLQKWCEKSIFGKDSSKFKLFKDEEEAFSKAIMSI-----------
------------------------------------------------------------------------
---------------------------

```
>WKA02_12
-------------------------------------------MSENQKMILGKSKSNNLFNEAIAQQKQKQ
SQIPVMGKTKAVGDMRNPTVAK-------NINKKIEESEI---AKSAKQSTAANMQNSSAGQAQK-------
---------------ECSNFCRKKGFNILPLRYTVVKDNPPALPPTLGKNVKDIQLSHLKYAVEMINTGYIY
RLVKRTSGALEWAGYKVTPNGHLSYFPVGKEA------------PKSVPEFACKGAGHSFNSSVIAVES---
------------------------NPKDEAKIAYIIHTHVPMSKAKISEYEKNADKFVTEGKWQKILINGGS
AQEHCIAASQFK-TTVYNLNSFGKNRIDATLNKFKDEPKSLACMALYDPVG--ITRKLNDSR----NSQFGK
LTNYLAKNVEKISNEHRLQSSQLVDSIKIVVENKLINQQLSARSQIADLSAKIEYAKDKIPKFGSMDQREYL
S----------------------------------KIQEIWDRAPTAE------------RDKFLLQRNKKF
AE-----------GEQKLKEKIITQSKAEAK--IT--WET--------------------------------
--------------------------KYAPKINWDTKKAFDKEVKKLINEGRTLAQTYASDHIKWLKSKHLL
NAFYVYDQTILKEYGALFHVHALAV---------MDGMTGCDEGQ-------------A---LMEKWLGVEE
ISDD---NLYMRAVTYNQKNLIDQYNAKSSEITSLTWDQTQSSLK---------------------SLIAAF
VSADQLWEQWLA--DPNNKINELKWYNPAKSLL----WISEITRSVAKWSLQYNINPLIAK-----------
-------------------------------------------------------------RLSRVSFIAYA
HSSLLTDRVPKWSLLYNVDFKMTNASTQLNVTEIERRWKNAGQNTAAQKIKEILSKSPASVAIRISSIVALF
E------------LINFGVQSNKFIGERNWENGFQLISGLLASSAAVLDIAGGGMDALSFQEKANKIRLYGA
RLAVSAAIISFCIDAFAVYKEKEGDKDKILMAFLFTKATVSLVMVGVG------------------------
-------------------------------------FGLLLNLPILQNSWVVSSANKIAFTRILLRANFIR
IAAYCNWIGIGFLVIEYLLKTYVLDDDLQKWCEKSIFGKDSSKFKLFKDEEEAFSKAIMSI-----------
------------------------------------------------------------------------
---------------------------
>ab736_10
----------------------------------------------------------------MTQAIQNE
TRSVRDMLIAAKVLAKDGKATE-------SPCKTCRVPVW---VSFFFDGTGNNKDADAATLNQS-------
------------------------------------------------------------------------
------------------------------------------------------------------------
------------------------NVVALFEAHKQDSKNGIEKFYYEGLGTQFRFDKYSVVDSGKITAAARS
LQGRKIDITDAE-WRKQGYSESGKGVQGALGLGVALGIKQRLQKAIFELVD--YLDKIYTQK----GITEIN
ISAFGFSRGA--------------------------------------------------------------
-----------------------------------TEARIFMNWLQHA------------PNVTTQGTGSGK
KL-----------FYRGKPLKAKFLGIFDTV--ES--IGN--------------------------------
------------------------------------------------------------------------
```

```
AAQNKNPELYRTRIEDYIEHSMHLV-------------------------------ASL---EMRQSFPLTP
TGKP----------------TANTVKGLIHDQKVYPGVHSNVGGG---------------------YMPMEQ
ARILGLSRITLH--AMYNRACAYGLKFFTLNEL----NAAKQRKI--------VFTRFYAF-----------
------------------------------------------------------------------------
------------------------DSKWQQDLNNFMAYVKGGTSFEQQMQGQIALY----------------
-------------HQWIREGGYARFIHRKTRERIGRKEKITAITKLNDGLFEN-------------------
------------------------------------------------------------------------
-------------------------------------IRQALNVYVPEGARPYDVIKGRDRKSTLPKEVIYY
FENYVCDSVGGFIAEASDFQAILNDGKAPNYFIPRGIVRPT-------------------------------
------------------------------------------------------------------------
---------------------------
>ATCC19606_10
----------------------------------------------------------------MTQAIQNE
TRSVRDMLIAAKVLAKDGKATE-------SPCKTCRVPVW---VSFFFDGTGNNKDADAATLNQS-------
------------------------------------------------------------------------
------------------------------------------------------------------------
------------------------NVVALFEAHKQDSKNGIEKFYYEGLGTQFRFDKYSVVDSGKITAAARS
LQGRKIDITDAE-WRKQGYSESGKGVQGALGLGVALGIKQRLQKAIFELVD--YLDKIYTQK----GITEIN
ISAFGFSRGA--------------------------------------------------------------
-----------------------------------TEARIFMNWLQHA------------PNVTTQGTGSGK
KL-----------FYRGKPLKAKFLGIFDTV--ES--IGN--------------------------------
------------------------------------------------------------------------
AAQNKNPELYRTRIEDYIEHSMHLV-------------------------------ASL---EMRQSFPLTP
TGKP----------------TANTVKGLIHDQKVYPGVHSNVGGG---------------------YMPMEQ
ARILGLSRITLH--AMYNRACAYGLKFFTLNEL----NAAKQRKI--------VFTRFYAF-----------
------------------------------------------------------------------------
------------------------DSKWQQDLNNFMAYVKGGTSFEQQMQGQIALY----------------
-------------HQWIREGGYARFIHRKTRERIGRKEKITAITKLNDGLFEN-------------------
------------------------------------------------------------------------
-------------------------------------IRQALNVYVPEGARPYDVIKGRDRKSTLPKEVIYY
FENYVCDSVGGFIAEASDFQAILNDGKAPNYFIPRGIVRPT-------------------------------
------------------------------------------------------------------------
---------------------------
```

```
>Hcp-ET1
---------------------------------------------------------------------MSN
IVYLTVTGEQQGSISAGCGTSESTGNRWQSGHEDEIFTFS---LLNNINNTGLGSQFHGITFCKL-------
-----------------------------------IDKSTPLFINSI-------------------------
------------------------------------------------------------------------
------------------------NNNEQLFMGFDFYRINRFGRLEKYYYIQLRGAFLSAIHHQIIENQLDT
ETITISYEFILCQHLIANTEFSYLALPENYNRLFLPNSKNQTNNRFKTLNSK--------------------
------------------------------------------------------------------------
-----------------------------------AIGRLLAAGGVYN----------GNIEGFRDTAEKLG
GD-----------AIKGYDQILNEKTAGIAI--AT-------------------------------------
------------------------------------------------------------------------
-----------ASILLTKRSNVDTY-------------------------------------TEINSYLGKL
RGQQ-----------------KLLDGIDIIEIIYIKRPSKDLANL---------------------RKEFNK
TVRKNFLIKLAK--TSETSGRFNAEDLLRMRKG--NVPLNYNVHH---------------------------
------------------------------------------------------------------------
------------------------------------------------------------------------
-------------KLSLDDGGTNDFENLVLIENEPYHKVFTNMQSRIA------------------------
------------------------------------------------------------------------
-------------------------------------KGILVGESKITPWAIPSGSIYPPMKNIMDHTK---
------------------------------------------------------------------------
------------------------------------------------------------------------
---------------------------
>Tle1Ec
--------------------------------------------------------------------MTKY
QGYDVTDATHKTSIHNDWKVVV-------AKKKPARGVTL---TIGIFFDGTGNNRENTASRLMK-------
---------------FNECSAARQGVNQKDAQSCEDFLKEINKNSISNGSYRGYYSNIHWLNILYHPDQVLK
KDQTSAQIKTYISGIGTAAGEADSVIGMGLG-----------------------------------------
------------------------TSILDIFEGVVTKTDEAMERITQALSEFMGFNLSPDFCIAKIQFDVFG
FSRGAAAARHFANRVMEQDPAIARAIAKGLRGDFYDGKPSGEVRFLGLFDTVAAIG----------------
------------------------------------------------------------------------
-----------------------------------GISNFFDINGRSN------------PGVKLELRPSVA
KK-----------VFQITAMNEYRYNFSLNS--IKGMWPE--------------------------------
------------------------------------------------------------------------
```

```
-------LALPGAHSDIGGGYNPVG---------------------------------SPLQENESLFLSCP
EFEI-----------VSDDTREMDTRVYRKAEQVRKMLMTLPALK-----------------HILPHGKLTT
KIRSIGVNNSNQ--RRAGVIQKQVGAAVFFERM--AVPNDWANVCLRVMLDAAQEAGVLFE-----------
------------------------------------------------------------------------
PIRQTNTELQLPSELIFLADKAIAQGKAVRLGQEPQAFTEEELYIIGKYTHCS-------------------
-------------ANWNIESDGNLWVDPTTGEIFIHRFGP--------------------------------
------------------------------------------------------------------------
-------------------------------------KGNKAFVFPNKPNDRWIRSVWYMDDQQRLNDNAVK
NTKVMMSGV---------------------------------------------------------------
------------------------------------------------------------------------
---------------------------
>PldB
----------------------------------------------------------MSDLYKPQTLKLYA
QQAGSVRLTLDWFANKAFYPPR-----AGVHIKPLINGQA---AFDAVHAAMEAARHSIDIITWG-------
-------------FDPAMRFKRPDGPRIGELLQTKGREGVQARVLVWSNQLARLKENTIPGAGVGGSGGTWV
GSGVASGSAVDNEVLRLEQRR---------------------------------------------------
------------------------QHNLNLIARQQEALERSERLHREGRLPSFDPRGAAHARTRIAELEAEN
AEIQRTLDSSGAQGYGGKRGSGGTRQDPWGQIFTRDWFKAVRGGGLQNVEFRTRDFEQTARPVMNGEQVRLV
NGRLQSLIHLLRADGIDDLGIGQLLVLTQFASH---------------------------------------
-----------------------------------HQKMVLVDYGSPQ------------AIGFVMGHNMHR
NY-----------WDTSAHLFDDRAAGRDPG--FG-PWQD--------------------------------
------------------------------------------------------------------ISMQVQ
GPVLADLSRNFSEAWDLETPWYKRW------------------------------FSTPSLTAERDALPLPK
IATP------ASNSVAQICRTQPQDDERSILEHYLKALGNATDYV---------------------YMENQY
FRYAGFAERLRK--TAQVRKARGVPGDLYLFVV--TNTPDSSDASKTTYDMMKGLGQEQLM-----------
--------------------------------------------------------------PQVQRDLAHD
LREKREQLKQVRENLHPDPYVRRGQENNIERLERKIEALEEKGVTPEVEQRLGDLGA---------------
-------------QEIPGLAKNTGEDDKPYQLVEVPGLKVVVATLATSDPAPGSPPPARLSAEAEAALGAPP
LKARYKHIYVHSKLLLVDDLYTLLSSANINVRSMHGDSE---------------------------------
-------------------------------------LGVAQPNPDLARAMREELWELHAQKVATTTEKNFK
LWNQKMDANWRQQRKDEPLVANLLRFWDVVTPYSPGLTVD--------------------------------
------------------------------------------------------------------------
---------------------------
```

```
>6200_21
------------------------------------------------------------------------
-------NFKSVGKIKEERARR-------QTSSSAPYIKH---TMAKCKTQAQNISKKEAQYWDM-------
------------------------------------------------------------------------
------------------------------------------------------------------------
------------------------------------------------------------------------
------------------------SKCES-------------------------------------------
------------------------------------------------------------------------
-----------------------------------NVAQVTAFRNRLE------------KEGLNKGYKVDL
GD------------NKGSHYYIY-DAGRNIG--FN---EG--------------------------------
------------------------------------------------------------------------
--------------------------------------------------------------KETQYMRIEV
SSTN-----------------------------EFHGHPISKQRY---------------------------
------------------------------------------------------------------------
------------------------------------------------------------------------
------------------------------------------------------------------------
-------------ESWQSKT----------------------------------------------------
------------------------------------------------------------------------
------------------------------------------------------------------------
------------------------------------------------------------------------
------------------------------------------------------------------------
---------------------------
>Tle1Bt
------------------------------------MNFRFAPAERPDIGVVTKEEKDAILRRLHDDDGMSC
CKTLHIGIFFDGTRNNAERDKSGHKHSNVARLRDAFPQDR---YHKSIYVAGVGTPFSSEIGDYGIGLQAVA
GASAGWAGEGRINWALLQIHNAVHECAFRVGLSTALGVDDKNLVKLMSLDMNFKGIDLGGNAPQPGSTGDIK
SRSSPGIGALKLIAAEQYGAELTWDKDTNW------------------------------------------
------------------------SQLKDDLDSSKWAAAVRAWDGRRRKILGDRRAQLKARVGDMLVKGKPR
IQRIRLYVFGFS-RGAAEARTFSNWLVDALESDFSLCGVPVSYDFLGIFDTVASVGIAQSAAATLFDGHGGW
ARKELMAVPHYVRRCVHMVAAHEPRGSFPLDLIDCSLEGREEIVYPGVHSD---------------------
-----------------------------------VGGGYGPAEQGRG------------RGDADKLSQVPL
VD-----------MYRAARIAGVPLDIQGPG--ITSEAAD--------------------------------
-------------------------------------------------VFKISAGLKQAFTAYVKASEGYY
```

```
YAKEHGTAGLMRAHYGLYLRWRRMR--------------------------------LKDMSLQPSFKAAQA
NCPQ--------------DAMDIDSANKELRAEWEDLLEIEKEGG-------------------PSVAHYAK
KFGAKVARDNPK--IVASVSAVLLPGVIVFSTR--PEVIYGVRKAGDRVTELVRAQLQEKW-----------
------------------------------------------------------------------------
---------EQWQQVRSDWNMGPPEAPISALYDNYMHDSRAWFKPLGDDD----------------------
-------------DVWNYKQIQELKSKQASFEREHAAWRKRAETGAPGPWQIAQAMSAGASGLGPIAMQPEP
EPRSPLTAQQADLLKRYDAAMQSAKQARAAKDPNAPTDSAVLTDPKVTGG----------------------
-------------------------------------LALQTSGREFYFLWGFLRWRTVFVNGVRWDQPRVP
TVQEEMEGMRMQMQRQVDMKGIGVLFQ---------------------------------------------
------------------------------------------------------------------------
---------------------------
>Tle3
------------------------------------------------------------------MNDRVR
SRTIVSAQSITLPKGGDVHLVP-------PPPKPCVTIVV----HGVNDLAGCYERIERGLCQGL-------
-------------NERLDMPPTLPGGQANPGYLTPAGYSLPADDEGKAENPDVVYYRRKFASGAGGAAVRSV
VVPFYWGFREEEQYINKTAAHGEWLDRNGNRLDKSGTKEGGQFVNAT-------------------------
------------------------TNLPDMWGQGFNGKLFGFISLDWFGGTMTHPLFSAAGRKYMVLAAMRL
AMLIKIIRKRYPDDTINVVGHSQGTLLTLLAHAFLKDDGVAPADGVIMLNSPYGLFEPLNEKLQGWSSQQTR
EARLATLKGILEFICGRRHPVPALSS----------------------------------------------
-----------------------------------VALRNCQGYGAIG------------GPGWVGGQGCQT
TI---------------DGERLSFDERDNRG--SVYLYFT--------------------------------
------------------------------------------------------------------PQDQTV
GLANVQGIGWRGIAEQVKGLPGRTG-------------------------------------LPQGFHQRIF
TVRK-------RNGEKEKIGGHAPPHVYPLLLAGEKTWEDTGLGG---------------------KDRFGR
ANFDQGDSVLLT--APRLPLPTEARFDFDGAVT--APGENSASGV---------------------------
------------------------------------------------------------------------
----------------------YQVRDTLDPIDAAIGVSNGGWKEKDSGHAVA-------------------
-------------QQVDAALAYRYGRDARSVERALNEGKELAQQTHVFSARELGTGMVLVTRAETPYEARLR
LQTAEGHLEPLSFHSAIPNNPEHNRRVLAYDLAIGAGDSVDDVVF---------------------------
-------------------------------------YQYLCRVADWRLDWKASDKGIFSQGDASVDLPDEE
VRALYRAEESKNSQLIDATVAYRKSGEFPVVVGNRLPSLVGTQTILDRYHEQAVRFGGTI------------
------------------------------------------------------------------------
---------------------------
```

```
>15A5_18
-------------------------------------MSKIYIVKSGDTLWGISKKHHISVKELARINSLSG
RMIHNLRIGQKIYLQNDVNNTNNFETQLKIILMDLSFKPI---LKATIQLEFDGKKIIRNTKNSI-------
----------------FEDINIQDHSKGLKVFFKNLNGTFDLIADHKVLPLGRKVLKLTSRKMKVEGSHYAK
DGILNETVNQIMSNLKKVGKPIVESISTTSNKDELKNKQPPLKIPEEKIEQK--------------------
------------------------RTDNGNSTHIIAAQFTEDNFLLKPVNNKYRAYIVNAAKRHGFTPHSLA
AVIEAEAAKIKK-TGEWNTNSKANSSTAAGLTQFLDETWLAMCKDKSSLVG--QYVMNNPKL----TIQQKL
NLRFNAEMAIDAAAAYAISNFKSSGLPYQK------------------------------------------
-----------------------------------LTEPSSIAKFAYL----------LHHEGATGGKNFVL
NT-----------LSQERAKKLLFTQFGKNG--AK--QAA--------------------------------
------------------------------------------------------------------------
----DFLNRYKGDAKAAYGAWLRNY---------------------------------IDGHINIYQYVVDK
SKTS---------------GINLSTDETIKLLKGQTISTPAPKIS--------------------TTTNNQQ
VTNVSTIEKSES--KIRINTSQAPTNNVGGDNK----WHNPLADCKLRTAGLANAKGATFG-----------
------------------------------------------------------------------------
-----KVRNNGTKNHQGVDLQANPGTKIYAVCGGVIAFAGATGGAYGKV-----------------------
-------------IVLKVDINDLPEKQKKYAQTKLTKNKYVYFFYAHLSVIDVD------------------
------------------------------------------------------------------------
-------------------------------------KGDPVDTGEVIGKTGATGNANKMTTISKGAHLHFE
ARSAPLLGVGLDGRFDPIPFINANLPY---------------------------------------------
------------------------------------------------------------------------
---------------------------
>3027STDY5784958_18
-------------------------------------MSKIYIVKSGDTLWGISKKHHISVKELARINSLSG
RMIHNLRIGQKIYLQNDVNNTNNFETQLKIILMDLSFKPI---LKATIQLEFDGKKIIRNTKNSI-------
----------------FEDINIQDHSKGLKVFFKNLNGTFDLIADHKVLPLGRKVLKLTSRKMKVEGSHYAK
DGILNETVNQIMSNLKKVGKPIVESISTTSNKDELKNKQPPLKIPEEKIEQK--------------------
------------------------RTDNGNSTHIIAAQFTEDNFLLKPVNNKYRAYIVNAAKRHGFTPHSLA
AVIEAEAAKIKK-TGEWNTNSKANSSTAAGLTQFLDETWLAMCKDKSSLVG--QYVMNNPKL----TIQQKL
NLRFNAEMAIDAAAAYAISNFKSSGLPYQK------------------------------------------
-----------------------------------LTEPSSIAKFAYL----------LHHEGATGGKNFVL
NT-----------LSQERAKKLLFTQFGKNG--AK--QAA--------------------------------
------------------------------------------------------------------------
```

```
----DFLNRYKGDAKAAYGAWLRNY---------------------------------IDGHINIYQYVVDK
SKTS---------------GINLSTDETIKLLKGQTISTPAPKIS--------------------TTTNNQQ
VTNVSTIEKSES--KIRINTSQAPTNNVGGDNK----WHNPLADCKLRTAGLANAKGATFG-----------
------------------------------------------------------------------------
-----KVRNNGTKNHQGVDLQANPGTKIYAVCGGVIAFAGATGGAYGKV-----------------------
-------------IVLKVDINDLPEKQKKYAQTKLTKNKYVYFFYAHLSVIDVD------------------
------------------------------------------------------------------------
-------------------------------------KGDPVDTGEVIGKTGATGNANKMTTISKGAHLHFE
ARSAPLLGVGLDGRFDPIPFINANLPY---------------------------------------------
------------------------------------------------------------------------
---------------------------
>AB07_18
-------------------------------------MSKIYIVKSGDTLWGISKKHHISVKELARINSLSG
RMIHNLRIGQKIYLQNDVNNTNNFETQLKIILMDLSFKPI---LKATIQLEFDGKKIIRNTKNSI-------
----------------FEDINIQDHSKGLKVFFKNLNGTFDLIADHKVLPLGRKVLKLTSRKMKVEGSHYAK
DGILNETVNQIMSNLKKVGKPIVESISTTSNKDELKNKQPPLKIPEEKIEQK--------------------
------------------------RTDNGNSTHIIAAQFTEDNFLLKPVNNKYRAYIVNAAKRHGFTPHSLA
AVIEAEAAKIKK-TGEWNTNSKANSSTAAGLTQFLDETWLAMCKDKSSLVG--QYVMNNPKL----TIQQKL
NLRFNAEMAIDAAAAYAISNFKSSGLPYQK------------------------------------------
-----------------------------------LTEPSSIAKFAYL----------LHHEGATGGKNFVL
NT-----------LSQERAKKLLFTQFGKNG--AK--QAA--------------------------------
------------------------------------------------------------------------
----DFLNRYKGDAKAAYGAWLRNY---------------------------------IDGHINIYQYVVDK
SKTS---------------GINLSTDETIKLLKGQTISTPAPKIS--------------------TTTNNQQ
VTNVSTIEKSES--KIRINTSQAPTNNVGGDNK----WHNPLADCKLRTAGLANAKGATFG-----------
------------------------------------------------------------------------
-----KVRNNGTKNHQGVDLQANPGTKIYAVCGGVIAFAGATGGAYGKV-----------------------
-------------IVLKVDINDLPEKQKKYAQTKLTKNKYVYFFYAHLSVIDVD------------------
------------------------------------------------------------------------
-------------------------------------KGDPVDTGEVIGKTGATGNANKMTTISKGAHLHFE
ARSAPLLGVGLDGRFDPIPFINANLPY---------------------------------------------
------------------------------------------------------------------------
---------------------------
```

```
>Ab4568_18
-------------------------------------MSKIYIVKSGDTLWGISKKHHISVKELARINSLSG
RMIHNLRIGQKIYLQNDVNNTNNFETQLKIILMDLSFKPI---LKATIQLEFDGKKIIRNTKNSI-------
----------------FEDINIQDHSKGLKVFFKNLNGTFDLIADHKVLPLGRKVLKLTSRKMKVEGSHYAK
DGILNETVNQIMSNLKKVGKPIVESISTTSNKDELKNKQPPLKIPEEKIEQK--------------------
------------------------RTDNGNSTHIIAAQFTEDNFLLKPVNNKYRAYIVNAAKRHGFTPHSLA
AVIEAEAAKIKK-TGEWNTNSKANSSTAAGLTQFLDETWLAMCKDKSSLVG--QYVMNNPKL----TIQQKL
NLRFNAEMAIDAAAAYAISNFKSSGLPYQK------------------------------------------
-----------------------------------LTEPSSIAKFAYL----------LHHEGATGGKNFVL
NT-----------LSQERAKKLLFTQFGKNG--AK--QAA--------------------------------
------------------------------------------------------------------------
----DFLNRYKGDAKAAYGAWLRNY---------------------------------IDGHINIYQYVVDK
SKTS---------------GINLSTDETIKLLKGQTISTPAPKIS--------------------TTTNNQQ
VTNVSTIEKSES--KIRINTSQAPTNNVGGDNK----WHNPLADCKLRTAGLANAKGATFG-----------
------------------------------------------------------------------------
-----KVRNNGTKNHQGVDLQANPGTKIYAVCGGVIAFAGATGGAYGKV-----------------------
-------------IVLKVDINDLPEKQKKYAQTKLTKNKYVYFFYAHLSVIDVD------------------
------------------------------------------------------------------------
-------------------------------------KGDPVDTGEVIGKTGATGNANKMTTISKGAHLHFE
ARSAPLLGVGLDGRFDPIPFINANLPY---------------------------------------------
------------------------------------------------------------------------
---------------------------
>Ab4977_18
-------------------------------------MSKIYIVKSGDTLWGISKKHHISVKELARINSLSG
RMIHNLRIGQKIYLQNDVNNTNNFETQLKIILMDLSFKPI---LKATIQLEFDGKKIIRNTKNSI-------
----------------FEDINIQDHSKGLKVFFKNLNGTFDLIADHKVLPLGRKVLKLTSRKMKVEGSHYAK
DGILNETVNQIMSNLKKVGKPIVESISTTSNKDELKNKQPPLKIPEEKIEQK--------------------
------------------------RTDNGNSTHIIAAQFTEDNFLLKPVNNKYRAYIVNAAKRHGFTPHSLA
AVIEAEAAKIKK-TGEWNTNSKANSSTAAGLTQFLDETWLAMCKDKSSLVG--QYVMNNPKL----TIQQKL
NLRFNAEMAIDAAAAYAISNFKSSGLPYQK------------------------------------------
-----------------------------------LTEPSSIAKFAYL----------LHHEGATGGKNFVL
NT-----------LSQERAKKLLFTQFGKNG--AK--QAA--------------------------------
------------------------------------------------------------------------
```

```
----DFLNRYKGDAKAAYGAWLRNY---------------------------------IDGHINIYQYVVDK
SKTS---------------GINLSTDETIKLLKGQTISTPAPKIS--------------------TTTNNQQ
VTNVSTIEKSES--KIRINTSQAPTNNVGGDNK----WHNPLADCKLRTAGLANAKGATFG-----------
------------------------------------------------------------------------
-----KVRNNGTKNHQGVDLQANPGTKIYAVCGGVIAFAGATGGAYGKV-----------------------
-------------IVLKVDINDLPEKQKKYAQTKLTKNKYVYFFYAHLSVIDVD------------------
------------------------------------------------------------------------
-------------------------------------KGDPVDTGEVIGKTGATGNANKMTTISKGAHLHFE
ARSAPLLGVGLDGRFDPIPFINANLPY---------------------------------------------
------------------------------------------------------------------------
---------------------------
>AC29_18
-------------------------------------MSKIYIVKSGDTLWGISKKHHISVKELARINSLSG
RMIHNLRIGQKIYLQNDVNNTNNFETQLKIILMDLSFKPI---LKATIQLEFDGKKIIRNTKNSI-------
----------------FEDINIQDHSKGLKVFFKNLNGTFDLIADHKVLPLGRKVLKLTSRKMKVEGSHYAK
DGILNETVNQIMSNLKKVGKPIVESISTTSNKDELKNKQPPLKIPEEKIEQK--------------------
------------------------RTDNGNSTHIIAAQFTEDNFLLKPVNNKYRAYIVNAAKRHGFTPHSLA
AVIEAEAAKIKK-TGEWNTNSKANSSTAAGLTQFLDETWLAMCKDKSSLVG--QYVMNNPKL----TIQQKL
NLRFNAEMAIDAAAAYAISNFKSSGLPYQK------------------------------------------
-----------------------------------LTEPSSIAKFAYL----------LHHEGATGGKNFVL
NT-----------LSQERAKKLLFTQFGKNG--AK--QAA--------------------------------
------------------------------------------------------------------------
----DFLNRYKGDAKAAYGAWLRNY---------------------------------IDGHINIYQYVVDK
SKTS---------------GINLSTDETIKLLKGQTISTPAPKIS--------------------TTTNNQQ
VTNVSTIEKSES--KIRINTSQAPTNNVGGDNK----WHNPLADCKLRTAGLANAKGATFG-----------
------------------------------------------------------------------------
-----KVRNNGTKNHQGVDLQANPGTKIYAVCGGVIAFAGATGGAYGKV-----------------------
-------------IVLKVDINDLPEKQKKYAQTKLTKNKYVYFFYAHLSVIDVD------------------
------------------------------------------------------------------------
-------------------------------------KGDPVDTGEVIGKTGATGNANKMTTISKGAHLHFE
ARSAPLLGVGLDGRFDPIPFINANLPY---------------------------------------------
------------------------------------------------------------------------
---------------------------
```

```
>AC30_18
-------------------------------------MSKIYIVKSGDTLWGISKKHHISVKELARINSLSG
RMIHNLRIGQKIYLQNDVNNTNNFETQLKIILMDLSFKPI---LKATIQLEFDGKKIIRNTKNSI-------
----------------FEDINIQDHSKGLKVFFKNLNGTFDLIADHKVLPLGRKVLKLTSRKMKVEGSHYAK
DGILNETVNQIMSNLKKVGKPIVESISTTSNKDELKNKQPPLKIPEEKIEQK--------------------
------------------------RTDNGNSTHIIAAQFTEDNFLLKPVNNKYRAYIVNAAKRHGFTPHSLA
AVIEAEAAKIKK-TGEWNTNSKANSSTAAGLTQFLDETWLAMCKDKSSLVG--QYVMNNPKL----TIQQKL
NLRFNAEMAIDAAAAYAISNFKSSGLPYQK------------------------------------------
-----------------------------------LTEPSSIAKFAYL----------LHHEGATGGKNFVL
NT-----------LSQERAKKLLFTQFGKNG--AK--QAA--------------------------------
------------------------------------------------------------------------
----DFLNRYKGDAKAAYGAWLRNY---------------------------------IDGHINIYQYVVDK
SKTS---------------GINLSTDETIKLLKGQTISTPAPKIS--------------------TTTNNQQ
VTNVSTIEKSES--KIRINTSQAPTNNVGGDNK----WHNPLADCKLRTAGLANAKGATFG-----------
------------------------------------------------------------------------
-----KVRNNGTKNHQGVDLQANPGTKIYAVCGGVIAFAGATGGAYGKV-----------------------
-------------IVLKVDINDLPEKQKKYAQTKLTKNKYVYFFYAHLSVIDVD------------------
------------------------------------------------------------------------
-------------------------------------KGDPVDTGEVIGKTGATGNANKMTTISKGAHLHFE
ARSAPLLGVGLDGRFDPIPFINANLPY---------------------------------------------
------------------------------------------------------------------------
---------------------------
>AF-673_18
-------------------------------------MSKIYIVKSGDTLWGISKKHHISVKELARINSLSG
RMIHNLRIGQKIYLQNDVNNTNNFETQLKIILMDLSFKPI---LKATIQLEFDGKKIIRNTKNSI-------
----------------FEDINIQDHSKGLKVFFKNLNGTFDLIADHKVLPLGRKVLKLTSRKMKVEGSHYAK
DGILNETVNQIMSNLKKVGKPIVESISTTSNKDELKNKQPPLKIPEEKIEQK--------------------
------------------------RTDNGNSTHIIAAQFTEDNFLLKPVNNKYRAYIVNAAKRHGFTPHSLA
AVIEAEAAKIKK-TGEWNTNSKANSSTAAGLTQFLDETWLAMCKDKSSLVG--QYVMNNPKL----TIQQKL
NLRFNAEMAIDAAAAYAISNFKSSGLPYQK------------------------------------------
-----------------------------------LTEPSSIAKFAYL----------LHHEGATGGKNFVL
NT-----------LSQERAKKLLFTQFGKNG--AK--QAA--------------------------------
------------------------------------------------------------------------
```

```
----DFLNRYKGDAKAAYGAWLRNY---------------------------------IDGHINIYQYVVDK
SKTS---------------GINLSTDETIKLLKGQTISTPAPKIS--------------------TTTNNQQ
VTNVSTIEKSES--KIRINTSQAPTNNVGGDNK----WHNPLADCKLRTAGLANAKGATFG-----------
------------------------------------------------------------------------
-----KVRNNGTKNHQGVDLQANPGTKIYAVCGGVIAFAGATGGAYGKV-----------------------
-------------IVLKVDINDLPEKQKKYAQTKLTKNKYVYFFYAHLSVIDVD------------------
------------------------------------------------------------------------
-------------------------------------KGDPVDTGEVIGKTGATGNANKMTTISKGAHLHFE
ARSAPLLGVGLDGRFDPIPFINANLPY---------------------------------------------
------------------------------------------------------------------------
---------------------------
>AR_0102_18
-------------------------------------MSKIYIVKSGDTLWGISKKHHISVKELARINSLSG
RMIHNLRIGQKIYLQNDVNNTNNFETQLKIILMDLSFKPI---LKATIQLEFDGKKIIRNTKNSI-------
----------------FEDINIQDHSKGLKVFFKNLNGTFDLIADHKVLPLGRKVLKLTSRKMKVEGSHYAK
DGILNETVNQIMSNLKKVGKPIVESISTTSNKDELKNKQPPLKIPEEKIEQK--------------------
------------------------RTDNGNSTHIIAAQFTEDNFLLKPVNNKYRAYIVNAAKRHGFTPHSLA
AVIEAEAAKIKK-TGEWNTNSKANSSTAAGLTQFLDETWLAMCKDKSSLVG--QYVMNNPKL----TIQQKL
NLRFNAEMAIDAAAAYAISNFKSSGLPYQK------------------------------------------
-----------------------------------LTEPSSIAKFAYL----------LHHEGATGGKNFVL
NT-----------LSQERAKKLLFTQFGKNG--AK--QAA--------------------------------
------------------------------------------------------------------------
----DFLNRYKGDAKAAYGAWLRNY---------------------------------IDGHINIYQYVVDK
SKTS---------------GINLSTDETIKLLKGQTISTPAPKIS--------------------TTTNNQQ
VTNVSTIEKSES--KIRINTSQAPTNNVGGDNK----WHNPLADCKLRTAGLANAKGATFG-----------
------------------------------------------------------------------------
-----KVRNNGTKNHQGVDLQANPGTKIYAVCGGVIAFAGATGGAYGKV-----------------------
-------------IVLKVDINDLPEKQKKYAQTKLTKNKYVYFFYAHLSVIDVD------------------
------------------------------------------------------------------------
-------------------------------------KGDPVDTGEVIGKTGATGNANKMTTISKGAHLHFE
ARSAPLLGVGLDGRFDPIPFINANLPY---------------------------------------------
------------------------------------------------------------------------
---------------------------
```

```
>AYP-A2_18
-------------------------------------MSKIYIVKSGDTLWGISKKHHISVKELARINSLSG
RMIHNLRIGQKIYLQNDVNNTNNFETQLKIILMDLSFKPI---LKATIQLEFDGKKIIRNTKNSI-------
----------------FEDINIQDHSKGLKVFFKNLNGTFDLIADHKVLPLGRKVLKLTSRKMKVEGSHYAK
DGILNETVNQIMSNLKKVGKPIVESISTTSNKDELKNKQPPLKIPEEKIEQK--------------------
------------------------RTDNGNSTHIIAAQFTEDNFLLKPVNNKYRAYIVNAAKRHGFTPHSLA
AVIEAEAAKIKK-TGEWNTNSKANSSTAAGLTQFLDETWLAMCKDKSSLVG--QYVMNNPKL----TIQQKL
NLRFNAEMAIDAAAAYAISNFKSSGLPYQK------------------------------------------
-----------------------------------LTEPSSIAKFAYL----------LHHEGATGGKNFVL
NT-----------LSQERAKKLLFTQFGKNG--AK--QAA--------------------------------
------------------------------------------------------------------------
----DFLNRYKGDAKAAYGAWLRNY---------------------------------IDGHINIYQYVVDK
SKTS---------------GINLSTDETIKLLKGQTISTPAPKIS--------------------TTTNNQQ
VTNVSTIEKSES--KIRINTSQAPTNNVGGDNK----WHNPLADCKLRTAGLANAKGATFG-----------
------------------------------------------------------------------------
-----KVRNNGTKNHQGVDLQANPGTKIYAVCGGVIAFAGATGGAYGKV-----------------------
-------------IVLKVDINDLPEKQKKYAQTKLTKNKYVYFFYAHLSVIDVD------------------
------------------------------------------------------------------------
-------------------------------------KGDPVDTGEVIGKTGATGNANKMTTISKGAHLHFE
ARSAPLLGVGLDGRFDPIPFINANLPY---------------------------------------------
------------------------------------------------------------------------
---------------------------
>CBA7_18
-------------------------------------MSKIYIVKSGDTLWGISKKHHISVKELARINSLSG
RMIHNLRIGQKIYLQNDVNNTNNFETQLKIILMDLSFKPI---LKATIQLEFDGKKIIRNTKNSI-------
----------------FEDINIQDHSKGLKVFFKNLNGTFDLIADHKVLPLGRKVLKLTSRKMKVEGSHYAK
DGILNETVNQIMSNLKKVGKPIVESISTTSNKDELKNKQPPLKIPEEKIEQK--------------------
------------------------RTDNGNSTHIIAAQFTEDNFLLKPVNNKYRAYIVNAAKRHGFTPHSLA
AVIEAEAAKIKK-TGEWNTNSKANSSTAAGLTQFLDETWLAMCKDKSSLVG--QYVMNNPKL----TIQQKL
NLRFNAEMAIDAAAAYAISNFKSSGLPYQK------------------------------------------
-----------------------------------LTEPSSIAKFAYL----------LHHEGATGGKNFVL
NT-----------LSQERAKKLLFTQFGKNG--AK--QAA--------------------------------
------------------------------------------------------------------------
```

```
----DFLNRYKGDAKAAYGAWLRNY---------------------------------IDGHINIYQYVVDK
SKTS---------------GINLSTDETIKLLKGQTISTPAPKIS--------------------TTTNNQQ
VTNVSTIEKSES--KIRINTSQAPTNNVGGDNK----WHNPLADCKLRTAGLANAKGATFG-----------
------------------------------------------------------------------------
-----KVRNNGTKNHQGVDLQANPGTKIYAVCGGVIAFAGATGGAYGKV-----------------------
-------------IVLKVDINDLPEKQKKYAQTKLTKNKYVYFFYAHLSVIDVD------------------
------------------------------------------------------------------------
-------------------------------------KGDPVDTGEVIGKTGATGNANKMTTISKGAHLHFE
ARSAPLLGVGLDGRFDPIPFINANLPY---------------------------------------------
------------------------------------------------------------------------
---------------------------
>CMC-CR-MDR-Ab4_18
-------------------------------------MSKIYIVKSGDTLWGISKKHHISVKELARINSLSG
RMIHNLRIGQKIYLQNDVNNTNNFETQLKIILMDLSFKPI---LKATIQLEFDGKKIIRNTKNSI-------
----------------FEDINIQDHSKGLKVFFKNLNGTFDLIADHKVLPLGRKVLKLTSRKMKVEGSHYAK
DGILNETVNQIMSNLKKVGKPIVESISTTSNKDELKNKQPPLKIPEEKIEQK--------------------
------------------------RTDNGNSTHIIAAQFTEDNFLLKPVNNKYRAYIVNAAKRHGFTPHSLA
AVIEAEAAKIKK-TGEWNTNSKANSSTAAGLTQFLDETWLAMCKDKSSLVG--QYVMNNPKL----TIQQKL
NLRFNAEMAIDAAAAYAISNFKSSGLPYQK------------------------------------------
-----------------------------------LTEPSSIAKFAYL----------LHHEGATGGKNFVL
NT-----------LSQERAKKLLFTQFGKNG--AK--QAA--------------------------------
------------------------------------------------------------------------
----DFLNRYKGDAKAAYGAWLRNY---------------------------------IDGHINIYQYVVDK
SKTS---------------GINLSTDETIKLLKGQTISTPAPKIS--------------------TTTNNQQ
VTNVSTIEKSES--KIRINTSQAPTNNVGGDNK----WHNPLADCKLRTAGLANAKGATFG-----------
------------------------------------------------------------------------
-----KVRNNGTKNHQGVDLQANPGTKIYAVCGGVIAFAGATGGAYGKV-----------------------
-------------IVLKVDINDLPEKQKKYAQTKLTKNKYVYFFYAHLSVIDVD------------------
------------------------------------------------------------------------
-------------------------------------KGDPVDTGEVIGKTGATGNANKMTTISKGAHLHFE
ARSAPLLGVGLDGRFDPIPFINANLPY---------------------------------------------
------------------------------------------------------------------------
---------------------------
```

```
>CMC-CR-MDR-Ab66_18
-------------------------------------MSKIYIVKSGDTLWGISKKHHISVKELARINSLSG
RMIHNLRIGQKIYLQNDVNNTNNFETQLKIILMDLSFKPI---LKATIQLEFDGKKIIRNTKNSI-------
----------------FEDINIQDHSKGLKVFFKNLNGTFDLIADHKVLPLGRKVLKLTSRKMKVEGSHYAK
DGILNETVNQIMSNLKKVGKPIVESISTTSNKDELKNKQPPLKIPEEKIEQK--------------------
------------------------RTDNGNSTHIIAAQFTEDNFLLKPVNNKYRAYIVNAAKRHGFTPHSLA
AVIEAEAAKIKK-TGEWNTNSKANSSTAAGLTQFLDETWLAMCKDKSSLVG--QYVMNNPKL----TIQQKL
NLRFNAEMAIDAAAAYAISNFKSSGLPYQK------------------------------------------
-----------------------------------LTEPSSIAKFAYL----------LHHEGATGGKNFVL
NT-----------LSQERAKKLLFTQFGKNG--AK--QAA--------------------------------
------------------------------------------------------------------------
----DFLNRYKGDAKAAYGAWLRNY---------------------------------IDGHINIYQYVVDK
SKTS---------------GINLSTDETIKLLKGQTISTPAPKIS--------------------TTTNNQQ
VTNVSTIEKSES--KIRINTSQAPTNNVGGDNK----WHNPLADCKLRTAGLANAKGATFG-----------
------------------------------------------------------------------------
-----KVRNNGTKNHQGVDLQANPGTKIYAVCGGVIAFAGATGGAYGKV-----------------------
-------------IVLKVDINDLPEKQKKYAQTKLTKNKYVYFFYAHLSVIDVD------------------
------------------------------------------------------------------------
-------------------------------------KGDPVDTGEVIGKTGATGNANKMTTISKGAHLHFE
ARSAPLLGVGLDGRFDPIPFINANLPY---------------------------------------------
------------------------------------------------------------------------
---------------------------
>HRAB-85_18
-------------------------------------MSKIYIVKSGDTLWGISKKHHISVKELARINSLSG
RMIHNLRIGQKIYLQNDVNNTNNFETQLKIILMDLSFKPI---LKATIQLEFDGKKIIRNTKNSI-------
----------------FEDINIQDHSKGLKVFFKNLNGTFDLIADHKVLPLGRKVLKLTSRKMKVEGSHYAK
DGILNETVNQIMSNLKKVGKPIVESISTTSNKDELKNKQPPLKIPEEKIEQK--------------------
------------------------RTDNGNSTHIIAAQFTEDNFLLKPVNNKYRAYIVNAAKRHGFTPHSLA
AVIEAEAAKIKK-TGEWNTNSKANSSTAAGLTQFLDETWLAMCKDKSSLVG--QYVMNNPKL----TIQQKL
NLRFNAEMAIDAAAAYAISNFKSSGLPYQK------------------------------------------
-----------------------------------LTEPSSIAKFAYL----------LHHEGATGGKNFVL
NT-----------LSQERAKKLLFTQFGKNG--AK--QAA--------------------------------
------------------------------------------------------------------------
```

```
----DFLNRYKGDAKAAYGAWLRNY---------------------------------IDGHINIYQYVVDK
SKTS---------------GINLSTDETIKLLKGQTISTPAPKIS--------------------TTTNNQQ
VTNVSTIEKSES--KIRINTSQAPTNNVGGDNK----WHNPLADCKLRTAGLANAKGATFG-----------
------------------------------------------------------------------------
-----KVRNNGTKNHQGVDLQANPGTKIYAVCGGVIAFAGATGGAYGKV-----------------------
-------------IVLKVDINDLPEKQKKYAQTKLTKNKYVYFFYAHLSVIDVD------------------
------------------------------------------------------------------------
-------------------------------------KGDPVDTGEVIGKTGATGNANKMTTISKGAHLHFE
ARSAPLLGVGLDGRFDPIPFINANLPY---------------------------------------------
------------------------------------------------------------------------
---------------------------
>JBA13_18
-------------------------------------MSKIYIVKSGDTLWGISKKHHISVKELARINSLSG
RMIHNLRIGQKIYLQNDVNNTNNFETQLKIILMDLSFKPI---LKATIQLEFDGKKIIRNTKNSI-------
----------------FEDINIQDHSKGLKVFFKNLNGTFDLIADHKVLPLGRKVLKLTSRKMKVEGSHYAK
DGILNETVNQIMSNLKKVGKPIVESISTTSNKDELKNKQPPLKIPEEKIEQK--------------------
------------------------RTDNGNSTHIIAAQFTEDNFLLKPVNNKYRAYIVNAAKRHGFTPHSLA
AVIEAEAAKIKK-TGEWNTNSKANSSTAAGLTQFLDETWLAMCKDKSSLVG--QYVMNNPKL----TIQQKL
NLRFNAEMAIDAAAAYAISNFKSSGLPYQK------------------------------------------
-----------------------------------LTEPSSIAKFAYL----------LHHEGATGGKNFVL
NT-----------LSQERAKKLLFTQFGKNG--AK--QAA--------------------------------
------------------------------------------------------------------------
----DFLNRYKGDAKAAYGAWLRNY---------------------------------IDGHINIYQYVVDK
SKTS---------------GINLSTDETIKLLKGQTISTPAPKIS--------------------TTTNNQQ
VTNVSTIEKSES--KIRINTSQAPTNNVGGDNK----WHNPLADCKLRTAGLANAKGATFG-----------
------------------------------------------------------------------------
-----KVRNNGTKNHQGVDLQANPGTKIYAVCGGVIAFAGATGGAYGKV-----------------------
-------------IVLKVDINDLPEKQKKYAQTKLTKNKYVYFFYAHLSVIDVD------------------
------------------------------------------------------------------------
-------------------------------------KGDPVDTGEVIGKTGATGNANKMTTISKGAHLHFE
ARSAPLLGVGLDGRFDPIPFINANLPY---------------------------------------------
------------------------------------------------------------------------
---------------------------
```

>KAB02_18
-------------------------------------MSKIYIVKSGDTLWGISKKHHISVKELARINSLSG
RMIHNLRIGQKIYLQNDVNNTNNFETQLKIILMDLSFKPI---LKATIQLEFDGKKIIRNTKNSI-------
----------------FEDINIQDHSKGLKVFFKNLNGTFDLIADHKVLPLGRKVLKLTSRKMKVEGSHYAK
DGILNETVNQIMSNLKKVGKPIVESISTTSNKDELKNKQPPLKIPEEKIEQK--------------------
------------------------RTDNGNSTHIIAAQFTEDNFLLKPVNNKYRAYIVNAAKRHGFTPHSLA
AVIEAEAAKIKK-TGEWNTNSKANSSTAAGLTQFLDETWLAMCKDKSSLVG--QYVMNNPKL----TIQQKL
NLRFNAEMAIDAAAAYAISNFKSSGLPYQK------------------------------------------
-----------------------------------LTEPSSIAKFAYL----------LHHEGATGGKNFVL
NT-----------LSQERAKKLLFTQFGKNG--AK--QAA--------------------------------
------------------------------------------------------------------------
----DFLNRYKGDAKAAYGAWLRNY---------------------------------IDGHINIYQYVVDK
SKTS---------------GINLSTDETIKLLKGQTISTPAPKIS--------------------TTTNNQQ
VTNVSTIEKSES--KIRINTSQAPTNNVGGDNK----WHNPLADCKLRTAGLANAKGATFG-----------
------------------------------------------------------------------------
-----KVRNNGTKNHQGVDLQANPGTKIYAVCGGVIAFAGATGGAYGKV-----------------------
-------------IVLKVDINDLPEKQKKYAQTKLTKNKYVYFFYAHLSVIDVD------------------
------------------------------------------------------------------------
-------------------------------------KGDPVDTGEVIGKTGATGNANKMTTISKGAHLHFE
ARSAPLLGVGLDGRFDPIPFINANLPY---------------------------------------------
------------------------------------------------------------------------
---------------------------
>KAB04_18
-------------------------------------MSKIYIVKSGDTLWGISKKHHISVKELARINSLSG
RMIHNLRIGQKIYLQNDVNNTNNFETQLKIILMDLSFKPI---LKATIQLEFDGKKIIRNTKNSI-------
----------------FEDINIQDHSKGLKVFFKNLNGTFDLIADHKVLPLGRKVLKLTSRKMKVEGSHYAK
DGILNETVNQIMSNLKKVGKPIVESISTTSNKDELKNKQPPLKIPEEKIEQK--------------------
------------------------RTDNGNSTHIIAAQFTEDNFLLKPVNNKYRAYIVNAAKRHGFTPHSLA
AVIEAEAAKIKK-TGEWNTNSKANSSTAAGLTQFLDETWLAMCKDKSSLVG--QYVMNNPKL----TIQQKL
NLRFNAEMAIDAAAAYAISNFKSSGLPYQK------------------------------------------
-----------------------------------LTEPSSIAKFAYL----------LHHEGATGGKNFVL
NT-----------LSQERAKKLLFTQFGKNG--AK--QAA--------------------------------
------------------------------------------------------------------------

```
----DFLNRYKGDAKAAYGAWLRNY---------------------------------IDGHINIYQYVVDK
SKTS---------------GINLSTDETIKLLKGQTISTPAPKIS--------------------TTTNNQQ
VTNVSTIEKSES--KIRINTSQAPTNNVGGDNK----WHNPLADCKLRTAGLANAKGATFG-----------
------------------------------------------------------------------------
-----KVRNNGTKNHQGVDLQANPGTKIYAVCGGVIAFAGATGGAYGKV-----------------------
-------------IVLKVDINDLPEKQKKYAQTKLTKNKYVYFFYAHLSVIDVD------------------
------------------------------------------------------------------------
-------------------------------------KGDPVDTGEVIGKTGATGNANKMTTISKGAHLHFE
ARSAPLLGVGLDGRFDPIPFINANLPY---------------------------------------------
------------------------------------------------------------------------
---------------------------
>KAB05_18
-------------------------------------MSKIYIVKSGDTLWGISKKHHISVKELARINSLSG
RMIHNLRIGQKIYLQNDVNNTNNFETQLKIILMDLSFKPI---LKATIQLEFDGKKIIRNTKNSI-------
----------------FEDINIQDHSKGLKVFFKNLNGTFDLIADHKVLPLGRKVLKLTSRKMKVEGSHYAK
DGILNETVNQIMSNLKKVGKPIVESISTTSNKDELKNKQPPLKIPEEKIEQK--------------------
------------------------RTDNGNSTHIIAAQFTEDNFLLKPVNNKYRAYIVNAAKRHGFTPHSLA
AVIEAEAAKIKK-TGEWNTNSKANSSTAAGLTQFLDETWLAMCKDKSSLVG--QYVMNNPKL----TIQQKL
NLRFNAEMAIDAAAAYAISNFKSSGLPYQK------------------------------------------
-----------------------------------LTEPSSIAKFAYL----------LHHEGATGGKNFVL
NT-----------LSQERAKKLLFTQFGKNG--AK--QAA--------------------------------
------------------------------------------------------------------------
----DFLNRYKGDAKAAYGAWLRNY---------------------------------IDGHINIYQYVVDK
SKTS---------------GINLSTDETIKLLKGQTISTPAPKIS--------------------TTTNNQQ
VTNVSTIEKSES--KIRINTSQAPTNNVGGDNK----WHNPLADCKLRTAGLANAKGATFG-----------
------------------------------------------------------------------------
-----KVRNNGTKNHQGVDLQANPGTKIYAVCGGVIAFAGATGGAYGKV-----------------------
-------------IVLKVDINDLPEKQKKYAQTKLTKNKYVYFFYAHLSVIDVD------------------
------------------------------------------------------------------------
-------------------------------------KGDPVDTGEVIGKTGATGNANKMTTISKGAHLHFE
ARSAPLLGVGLDGRFDPIPFINANLPY---------------------------------------------
------------------------------------------------------------------------
---------------------------
```

```
>KAB06_18
-------------------------------------MSKIYIVKSGDTLWGISKKHHISVKELARINSLSG
RMIHNLRIGQKIYLQNDVNNTNNFETQLKIILMDLSFKPI---LKATIQLEFDGKKIIRNTKNSI-------
----------------FEDINIQDHSKGLKVFFKNLNGTFDLIADHKVLPLGRKVLKLTSRKMKVEGSHYAK
DGILNETVNQIMSNLKKVGKPIVESISTTSNKDELKNKQPPLKIPEEKIEQK--------------------
------------------------RTDNGNSTHIIAAQFTEDNFLLKPVNNKYRAYIVNAAKRHGFTPHSLA
AVIEAEAAKIKK-TGEWNTNSKANSSTAAGLTQFLDETWLAMCKDKSSLVG--QYVMNNPKL----TIQQKL
NLRFNAEMAIDAAAAYAISNFKSSGLPYQK------------------------------------------
-----------------------------------LTEPSSIAKFAYL----------LHHEGATGGKNFVL
NT-----------LSQERAKKLLFTQFGKNG--AK--QAA--------------------------------
------------------------------------------------------------------------
----DFLNRYKGDAKAAYGAWLRNY---------------------------------IDGHINIYQYVVDK
SKTS---------------GINLSTDETIKLLKGQTISTPAPKIS--------------------TTTNNQQ
VTNVSTIEKSES--KIRINTSQAPTNNVGGDNK----WHNPLADCKLRTAGLANAKGATFG-----------
------------------------------------------------------------------------
-----KVRNNGTKNHQGVDLQANPGTKIYAVCGGVIAFAGATGGAYGKV-----------------------
-------------IVLKVDINDLPEKQKKYAQTKLTKNKYVYFFYAHLSVIDVD------------------
------------------------------------------------------------------------
-------------------------------------KGDPVDTGEVIGKTGATGNANKMTTISKGAHLHFE
ARSAPLLGVGLDGRFDPIPFINANLPY---------------------------------------------
------------------------------------------------------------------------
---------------------------
>KAB07_18
-------------------------------------MSKIYIVKSGDTLWGISKKHHISVKELARINSLSG
RMIHNLRIGQKIYLQNDVNNTNNFETQLKIILMDLSFKPI---LKATIQLEFDGKKIIRNTKNSI-------
----------------FEDINIQDHSKGLKVFFKNLNGTFDLIADHKVLPLGRKVLKLTSRKMKVEGSHYAK
DGILNETVNQIMSNLKKVGKPIVESISTTSNKDELKNKQPPLKIPEEKIEQK--------------------
------------------------RTDNGNSTHIIAAQFTEDNFLLKPVNNKYRAYIVNAAKRHGFTPHSLA
AVIEAEAAKIKK-TGEWNTNSKANSSTAAGLTQFLDETWLAMCKDKSSLVG--QYVMNNPKL----TIQQKL
NLRFNAEMAIDAAAAYAISNFKSSGLPYQK------------------------------------------
-----------------------------------LTEPSSIAKFAYL----------LHHEGATGGKNFVL
NT-----------LSQERAKKLLFTQFGKNG--AK--QAA--------------------------------
------------------------------------------------------------------------
```

----DFLNRYKGDAKAAYGAWLRNY---------------------------------IDGHINIYQYVVDK
SKTS---------------GINLSTDETIKLLKGQTISTPAPKIS--------------------TTTNNQQ
VTNVSTIEKSES--KIRINTSQAPTNNVGGDNK----WHNPLADCKLRTAGLANAKGATFG-----------
------------------------------------------------------------------------
-----KVRNNGTKNHQGVDLQANPGTKIYAVCGGVIAFAGATGGAYGKV-----------------------
-------------IVLKVDINDLPEKQKKYAQTKLTKNKYVYFFYAHLSVIDVD------------------
------------------------------------------------------------------------
-------------------------------------KGDPVDTGEVIGKTGATGNANKMTTISKGAHLHFE
ARSAPLLGVGLDGRFDPIPFINANLPY---------------------------------------------
------------------------------------------------------------------------
---------------------------
>KAB08_18
-------------------------------------MSKIYIVKSGDTLWGISKKHHISVKELARINSLSG
RMIHNLRIGQKIYLQNDVNNTNNFETQLKIILMDLSFKPI---LKATIQLEFDGKKIIRNTKNSI-------
----------------FEDINIQDHSKGLKVFFKNLNGTFDLIADHKVLPLGRKVLKLTSRKMKVEGSHYAK
DGILNETVNQIMSNLKKVGKPIVESISTTSNKDELKNKQPPLKIPEEKIEQK--------------------
------------------------RTDNGNSTHIIAAQFTEDNFLLKPVNNKYRAYIVNAAKRHGFTPHSLA
AVIEAEAAKIKK-TGEWNTNSKANSSTAAGLTQFLDETWLAMCKDKSSLVG--QYVMNNPKL----TIQQKL
NLRFNAEMAIDAAAAYAISNFKSSGLPYQK------------------------------------------
-----------------------------------LTEPSSIAKFAYL----------LHHEGATGGKNFVL
NT-----------LSQERAKKLLFTQFGKNG--AK--QAA--------------------------------
------------------------------------------------------------------------
----DFLNRYKGDAKAAYGAWLRNY---------------------------------IDGHINIYQYVVDK
SKTS---------------GINLSTDETIKLLKGQTISTPAPKIS--------------------TTTNNQQ
VTNVSTIEKSES--KIRINTSQAPTNNVGGDNK----WHNPLADCKLRTAGLANAKGATFG-----------
------------------------------------------------------------------------
-----KVRNNGTKNHQGVDLQANPGTKIYAVCGGVIAFAGATGGAYGKV-----------------------
-------------IVLKVDINDLPEKQKKYAQTKLTKNKYVYFFYAHLSVIDVD------------------
------------------------------------------------------------------------
-------------------------------------KGDPVDTGEVIGKTGATGNANKMTTISKGAHLHFE
ARSAPLLGVGLDGRFDPIPFINANLPY---------------------------------------------
------------------------------------------------------------------------
---------------------------

```
>SAA14_18
-------------------------------------MSKIYIVKSGDTLWGISKKHHISVKELARINSLSG
RMIHNLRIGQKIYLQNDVNNTNNFETQLKIILMDLSFKPI---LKATIQLEFDGKKIIRNTKNSI-------
----------------FEDINIQDHSKGLKVFFKNLNGTFDLIADHKVLPLGRKVLKLTSRKMKVEGSHYAK
DGILNETVNQIMSNLKKVGKPIVESISTTSNKDELKNKQPPLKIPEEKIEQK--------------------
------------------------RTDNGNSTHIIAAQFTEDNFLLKPVNNKYRAYIVNAAKRHGFTPHSLA
AVIEAEAAKIKK-TGEWNTNSKANSSTAAGLTQFLDETWLAMCKDKSSLVG--QYVMNNPKL----TIQQKL
NLRFNAEMAIDAAAAYAISNFKSSGLPYQK------------------------------------------
-----------------------------------LTEPSSIAKFAYL----------LHHEGATGGKNFVL
NT-----------LSQERAKKLLFTQFGKNG--AK--QAA--------------------------------
------------------------------------------------------------------------
----DFLNRYKGDAKAAYGAWLRNY---------------------------------IDGHINIYQYVVDK
SKTS---------------GINLSTDETIKLLKGQTISTPAPKIS--------------------TTTNNQQ
VTNVSTIEKSES--KIRINTSQAPTNNVGGDNK----WHNPLADCKLRTAGLANAKGATFG-----------
------------------------------------------------------------------------
-----KVRNNGTKNHQGVDLQANPGTKIYAVCGGVIAFAGATGGAYGKV-----------------------
-------------IVLKVDINDLPEKQKKYAQTKLTKNKYVYFFYAHLSVIDVD------------------
------------------------------------------------------------------------
-------------------------------------KGDPVDTGEVIGKTGATGNANKMTTISKGAHLHFE
ARSAPLLGVGLDGRFDPIPFINANLPY---------------------------------------------
------------------------------------------------------------------------
---------------------------
>SSA12_18
-------------------------------------MSKIYIVKSGDTLWGISKKHHISVKELARINSLSG
RMIHNLRIGQKIYLQNDVNNTNNFETQLKIILMDLSFKPI---LKATIQLEFDGKKIIRNTKNSI-------
----------------FEDINIQDHSKGLKVFFKNLNGTFDLIADHKVLPLGRKVLKLTSRKMKVEGSHYAK
DGILNETVNQIMSNLKKVGKPIVESISTTSNKDELKNKQPPLKIPEEKIEQK--------------------
------------------------RTDNGNSTHIIAAQFTEDNFLLKPVNNKYRAYIVNAAKRHGFTPHSLA
AVIEAEAAKIKK-TGEWNTNSKANSSTAAGLTQFLDETWLAMCKDKSSLVG--QYVMNNPKL----TIQQKL
NLRFNAEMAIDAAAAYAISNFKSSGLPYQK------------------------------------------
-----------------------------------LTEPSSIAKFAYL----------LHHEGATGGKNFVL
NT-----------LSQERAKKLLFTQFGKNG--AK--QAA--------------------------------
------------------------------------------------------------------------
```

```
----DFLNRYKGDAKAAYGAWLRNY---------------------------------IDGHINIYQYVVDK
SKTS---------------GINLSTDETIKLLKGQTISTPAPKIS--------------------TTTNNQQ
VTNVSTIEKSES--KIRINTSQAPTNNVGGDNK----WHNPLADCKLRTAGLANAKGATFG-----------
------------------------------------------------------------------------
-----KVRNNGTKNHQGVDLQANPGTKIYAVCGGVIAFAGATGGAYGKV-----------------------
-------------IVLKVDINDLPEKQKKYAQTKLTKNKYVYFFYAHLSVIDVD------------------
------------------------------------------------------------------------
-------------------------------------KGDPVDTGEVIGKTGATGNANKMTTISKGAHLHFE
ARSAPLLGVGLDGRFDPIPFINANLPY---------------------------------------------
------------------------------------------------------------------------
---------------------------
>SSMA17_18
-------------------------------------MSKIYIVKSGDTLWGISKKHHISVKELARINSLSG
RMIHNLRIGQKIYLQNDVNNTNNFETQLKIILMDLSFKPI---LKATIQLEFDGKKIIRNTKNSI-------
----------------FEDINIQDHSKGLKVFFKNLNGTFDLIADHKVLPLGRKVLKLTSRKMKVEGSHYAK
DGILNETVNQIMSNLKKVGKPIVESISTTSNKDELKNKQPPLKIPEEKIEQK--------------------
------------------------RTDNGNSTHIIAAQFTEDNFLLKPVNNKYRAYIVNAAKRHGFTPHSLA
AVIEAEAAKIKK-TGEWNTNSKANSSTAAGLTQFLDETWLAMCKDKSSLVG--QYVMNNPKL----TIQQKL
NLRFNAEMAIDAAAAYAISNFKSSGLPYQK------------------------------------------
-----------------------------------LTEPSSIAKFAYL----------LHHEGATGGKNFVL
NT-----------LSQERAKKLLFTQFGKNG--AK--QAA--------------------------------
------------------------------------------------------------------------
----DFLNRYKGDAKAAYGAWLRNY---------------------------------IDGHINIYQYVVDK
SKTS---------------GINLSTDETIKLLKGQTISTPAPKIS--------------------TTTNNQQ
VTNVSTIEKSES--KIRINTSQAPTNNVGGDNK----WHNPLADCKLRTAGLANAKGATFG-----------
------------------------------------------------------------------------
-----KVRNNGTKNHQGVDLQANPGTKIYAVCGGVIAFAGATGGAYGKV-----------------------
-------------IVLKVDINDLPEKQKKYAQTKLTKNKYVYFFYAHLSVIDVD------------------
------------------------------------------------------------------------
-------------------------------------KGDPVDTGEVIGKTGATGNANKMTTISKGAHLHFE
ARSAPLLGVGLDGRFDPIPFINANLPY---------------------------------------------
------------------------------------------------------------------------
---------------------------
```

>XH386_18
-------------------------------------MSKIYIVKSGDTLWGISKKHHISVKELARINSLSG
RMIHNLRIGQKIYLQNDVNNTNNFETQLKIILMDLSFKPI---LKATIQLEFDGKKIIRNTKNSI-------
----------------FEDINIQDHSKGLKVFFKNLNGTFDLIADHKVLPLGRKVLKLTSRKMKVEGSHYAK
DGILNETVNQIMSNLKKVGKPIVESISTTSNKDELKNKQPPLKIPEEKIEQK--------------------
------------------------RTDNGNSTHIIAAQFTEDNFLLKPVNNKYRAYIVNAAKRHGFTPHSLA
AVIEAEAAKIKK-TGEWNTNSKANSSTAAGLTQFLDETWLAMCKDKSSLVG--QYVMNNPKL----TIQQKL
NLRFNAEMAIDAAAAYAISNFKSSGLPYQK------------------------------------------
-----------------------------------LTEPSSIAKFAYL----------LHHEGATGGKNFVL
NT-----------LSQERAKKLLFTQFGKNG--AK--QAA--------------------------------
------------------------------------------------------------------------
----DFLNRYKGDAKAAYGAWLRNY---------------------------------IDGHINIYQYVVDK
SKTS---------------GINLSTDETIKLLKGQTISTPAPKIS--------------------TTTNNQQ
VTNVSTIEKSES--KIRINTSQAPTNNVGGDNK----WHNPLADCKLRTAGLANAKGATFG-----------
------------------------------------------------------------------------
-----KVRNNGTKNHQGVDLQANPGTKIYAVCGGVIAFAGATGGAYGKV-----------------------
-------------IVLKVDINDLPEKQKKYAQTKLTKNKYVYFFYAHLSVIDVD------------------
------------------------------------------------------------------------
-------------------------------------KGDPVDTGEVIGKTGATGNANKMTTISKGAHLHFE
ARSAPLLGVGLDGRFDPIPFINANLPY---------------------------------------------
------------------------------------------------------------------------
---------------------------
>XH856_18
-------------------------------------MSKIYIVKSGDTLWGISKKHHISVKELARINSLSG
RMIHNLRIGQKIYLQNDVNNTNNFETQLKIILMDLSFKPI---LKATIQLEFDGKKIIRNTKNSI-------
----------------FEDINIQDHSKGLKVFFKNLNGTFDLIADHKVLPLGRKVLKLTSRKMKVEGSHYAK
DGILNETVNQIMSNLKKVGKPIVESISTTSNKDELKNKQPPLKIPEEKIEQK--------------------
------------------------RTDNGNSTHIIAAQFTEDNFLLKPVNNKYRAYIVNAAKRHGFTPHSLA
AVIEAEAAKIKK-TGEWNTNSKANSSTAAGLTQFLDETWLAMCKDKSSLVG--QYVMNNPKL----TIQQKL
NLRFNAEMAIDAAAAYAISNFKSSGLPYQK------------------------------------------
-----------------------------------LTEPSSIAKFAYL----------LHHEGATGGKNFVL
NT-----------LSQERAKKLLFTQFGKNG--AK--QAA--------------------------------
------------------------------------------------------------------------

----DFLNRYKGDAKAAYGAWLRNY---------------------------------IDGHINIYQYVVDK
SKTS---------------GINLSTDETIKLLKGQTISTPAPKIS--------------------TTTNNQQ
VTNVSTIEKSES--KIRINTSQAPTNNVGGDNK----WHNPLADCKLRTAGLANAKGATFG-----------
------------------------------------------------------------------------
-----KVRNNGTKNHQGVDLQANPGTKIYAVCGGVIAFAGATGGAYGKV-----------------------
-------------IVLKVDINDLPEKQKKYAQTKLTKNKYVYFFYAHLSVIDVD------------------
------------------------------------------------------------------------
-------------------------------------KGDPVDTGEVIGKTGATGNANKMTTISKGAHLHFE
ARSAPLLGVGLDGRFDPIPFINANLPY---------------------------------------------
------------------------------------------------------------------------
---------------------------
>3207_4
------------------------------------MSNIHIVKKGDTLWGISNKYHIKLNELIEINGLYGR
KKNLLKIGQQIYLKKDNVKKYDTTLVIKIYDLKWEPITHG---KLLLEYDDKCHLATSNDKGVIE-------
------------------DIQIEDALKGIKISFYTLKNKFELIAHHKTLPLGKKILKLSSRAMMIKGNTYKV
EGIPRTNTKTIEKELKVNSKKVQHSSGASGASGASGASGASGASGASGASGASGASGASGASGASGASGASG
ASGASGASGASGASGASGASGASGASGAS-ETRIEGGLPAVTVAPVYSEENLYLHPDNEKYRKIIIDAAKKY
NLAPQALAAKINAEAGTKKGSQEWNPQAQASTSSAVGLTQFLSGTWYEICTVSTYKDTLLQQYVIKKKLISN
FKENMALKLISNTDKAKLKALGTDPTFSVDSAAAYARANIN-------------------------------
-----------------------------------IVKKIYSKIDSLE------------PGDLAKVGYVAH
HD-----------GPTGFGKIVEATNDPSWS--KLQGQVC--------------------------------
----------------------------------------------------------------------PK
NNPNCDTYLNYKERFSNGRDAYRWW-------------------------------------LCTEYTDSKI
NVNR----------------FTLSPKDGKKFKNARTMEEIIVFLG--------------------GKPLVRP
SRDFEKKGTTDN--SKLNDNINIVITSLVNNKV--QANISYFVKSKYGLKPHSTNVNGHEI-----------
---------------------------------------------------------------------IHA
KKGDKIEILFNDQVIASINAINDKEEFKINFPSKNITNASTEKQSSNSEQKLNF------------------
-------------NRWRNPLNGICQIRRFGYNSLPLKANASSYNEKNLEQATKIASRFNRTSYRASGIHQGV
DLEADNGVNIYPVCVGTIAKVIPAYPGYGKTIILECDV----------------------------------
-------------------------------------NDLPSSKKVLAKNLDTIYFIYAHLNSINVSEGDII
TTLDTVLGKTGNSGNAGSMTRIEDGSHLHFEVRSEIMKSMGKSGMSYRLDPFPWLDNCMTTENGVKVNR---
------------------------------------------------------------------------
---------------------------

```
>ATCC17978-mff_4
------------------------------------MSNIHIVKKGDTLWGISNKYHIKLNELIEINGLYGR
KKNLLKIGQQIYLKKDNVKKYDTTLVIKIYDLKWEPITHG---KLLLEYDDKCHLATSNDKGVIE-------
------------------DIQIEDALKGIKISFYTLKNKFELIAHHKTLPLGKKILKLSSRAMMIKGNTYKV
EGIPRTNTKTIEKELKVNSKKVQHSSGASGASGASGASGASGASGASGASGASGASGASGASGAS-------
------------------------------ETRIEGGLPAVTVAPVYSEENLYLHPDNEKYRKIIIDAAKKY
NLAPQALAAKINAEAGTKKGSQEWNPQAQASTSSAVGLTQFLSGTWYEICTVSTYKDTLLQQYVIKKKLISN
FKENMALKLISNTDKAKLKALGTDPTFSVDSAAAYARANIN-------------------------------
-----------------------------------IVKKIYSKIDSLE------------PGDLAKVGYVAH
HD-----------GPTGFGKIVEATNDPSWS--KLQGQVC--------------------------------
----------------------------------------------------------------------PK
NNPNCDTYLNYKERFSNGRDAYRWW-------------------------------------LCTEYTDSKI
NVNR----------------FTLSPKDGKKFKNARTMEEIIVFLG--------------------GKPLVRP
SRDFEKKGTTDN--SKLNDNINIVITSLVNNKV--QANISYFVKSKYGLKPHSTNVNGHEI-----------
---------------------------------------------------------------------IHA
KKGDKIEILFNDQVIASINAINDKEEFKINFPSKNITNASTEKQSSNSEQKLNF------------------
-------------NRWRNPLNGICQIRRFGYNSLPLKANASSYNEKNLEQATKIASRFNRTSYRASGIHQGV
DLEADNGVNIYPVCVGTIAKVIPAYPGYGKTIILECDV----------------------------------
-------------------------------------NDLPSSKKVLAKNLDTIYFIYAHLNSINVSEGDSI
TTLDTVLGKTGNSGNAGSMTRIEDGSHLHFEVRSEIMKSMGKSGMSYRLDPFPWLDNCMTTENGVKVNR---
------------------------------------------------------------------------
---------------------------
>Tde1
-------------------------------------------------------------------MSATT
TVAGAGGAIAGGWLGRVIGGLA-------GSLAGPGGTAVGA-WIGGQVGAMAGRAAASAIASYM-------
------------------------------------------------------------------------
------------------------------------------------------------------------
----------------------------------------------------------EDANVDADAKTKES
EQAKPCVDCGEI-DCFSPPEGATPKQVEEFRRQLKEQQDEINRM----------------------------
------------------------------------------------------------------------
-----------------------------------EPDDLVRNIDKYR------------QQGRPTDDAANR
RQ-----------SREDYRTDRTRELEEKYL--SK--GRN--------------------------------
------------------------------------------------------------------------
```

-------DYKEQAANDVAEEMKK---------------------------------------LAATHTLDLV
AGGD-------------------------GSISGLGDKSINSSLG----------------------SQWKG
RRSEQLRSHAKK--AAEQKKKMNAKLEECKPEG--GNDNSPDAET---------------------------
------------------------------------------------------------------------
------------------------------------------------------------------------
-------------PDNGTGKGDGNPDVPVS------------------------------------------
------------------------------------------------------------------------
------------------------------------------------------------------------
------------------------------------------------------------------------
------------------------------------------------------------------------
---------------------------
>TseH
----------------------------------------------------------MTDTVSSTPVADTT
PYEIFLSGNDDFLIPVVFPDYL-------ISVADEQSFEL---WGVKIKTPAVKAPYLGHAGVIL-------
------------------------------------------------------------------------
------------------------------------------------------------------------
------------------------------------------------------------------------
-----INGETGV-TRYYEYGRYKNPKSDIPGNVRKV------------------------------------
------------------------------------------------------------------------
-----------------------------------GVSNVTIKSGLIT------------ESSLLKVLKEVS
LR-----------SGQEGRISGVVLRGKFFS--EA-------------------------------------
------------------------------------------------------------------------
-------DSWLRGKMDLNNSP-----------------------------------------DKIPYDLDSH
NCMT---------------------------------------------------------------FVIDL
ADAMGLDPAWKP--PVVVPSAYIEQF----------------------------------------------
------------------------------------------------------------------------
------------------------------------------------------------------------
-------------QLSEIDLDYDYKTNKLTVSE---------------------------------------
------------------------------------------------------------------------
------------------------------------------------------------------------
------------------------------------------------------------------------
------------------------------------------------------------------------
---------------------------

```
>KatN
---------------------------------------------------------------------MFR
HVKKLQYTVRVSEPNPGLANLLLEQFGGPQGELAAACRYF---TQGLSDEDAGRRDMLMDIATEE-------
------------------------------------------------------------------------
------------------------------------------------------------------------
---------------------------------------------------------LSHLEIIGSLVGMLN
KGAKGALAEGTE-NEAELYRSLTQNGNDSHITSLLYGGGPALTNSAGVPWTAAYVD----------------
------------------------------------------------------------------------
-----------------------------------TIGEVTADLRSNI--------------AAEARAKIIY
ER-----------LINVTDDPGVKDTLAFLM--TR-------------------------------------
------------------------------------------------------------------------
----------EAAHMLSFEKALHSI-------------------------------------RNTFPPGKLP
PLEK-------------------YKNVYYNMSEGEDVRGSWNSDE-------------------NFDYVSDP
VSAVDGGDGKAS--INLSTKQEAMIKAMATRLK---------------------------------------
------------------------------------------------------------------------
------------------------------------------------------------------------
-------------SHEDINPVTGAELAEGEPQTKINSKN---------------------------------
------------------------------------------------------------------------
------------------------------------------------------------------------
------------------------------------------------------------------------
------------------------------------------------------------------------
---------------------------
>Tge2
------------------------------------------------------------------------
-------------------------------MGPGLLYNH---AAKTAVNGIDRSTEGTNVSARL-------
------------------------------------------------------------------------
------------------------------------------------------------------------
------------------------------------------------------------------------
-----CNNISAV-TFVSKYKAVCQPIAD--------------------------------------------
------------------------------------------------------------------------
-----------------------------------QLDLPVENLLGLA----------AQESQYGTGRIARE
LN-----------NYFSMHAPAPLQIGAEAP--LG--NAS--------------------------------
------------------------------------------------------------------------
```

```
--------------------------------------------------------------IKVAKFDSFQ
KSAQ----------------------------SFASSFGTAVRGQ---------------------RDPMAF
AQALVRSGYNTG--NAKTGGRDGFARYLAD------------------------------------------
------------------------------------------------------------------------
------------------------------------------------------------------------
-------------IIIAVRGRMAC------------------------------------------------
------------------------------------------------------------------------
------------------------------------------------------------------------
------------------------------------------------------------------------
------------------------------------------------------------------------
---------------------------
>Tke7
--------------------------------------------------------------------MSEA
RSFINPHAQSYESLKADTPMNANQRAKFDVLNAHVVNTVV---FPGELIIVGDPSTPSCTAHEAF-------
------------------------------------------------------------------------
------------------------------------------------------------------------
--------------------------LMGKALGIHLDIELNGGGFDGFLLENFELLQSLLARASIGAGTASD
AWSKHLEAIK---KTLEEIEQLHRDYLLKGTLKARDEFYSKRTALFLKLE----------------------
------------------------------------------------------------------------
-----------------------------------AQLDSMATYGVGL----------RNGGKIKHALDIST
KR-----------FLHGGEIKGYAEKISGVA--KAANWIK--------------------------------
------------------------------------------------------------------------
----------KGTYLGMALD------------------------------------------VASTELSIRN
ACVL----------------GREEECRKAEYVERTSLVASLGLGG---------------------MGGHVG
GLLGPIACVAVG--IPTGGTATFACAVLGGAAG---------------------------------------
------------------------------------------------------------------------
------------------------------------------------------------------------
-------------GIAGGEFGEVLGESVGEILYEAVR-----------------------------------
------------------------------------------------------------------------
------------------------------------------------------------------------
------------------------------------------------------------------------
------------------------------------------------------------------------
---------------------------
```

>15A34_5
------------------------------------------------------------------------
---------------------------------ASTNSIG---SYGSVKSASDTYEKYATSKSLK-------
------------------------------------------------------------------------
------------------------------------------------------------------------
------------------------------------------------------------------------
-------------DVAERYAAGKSVG----------------------------------------------
------------------------------------------------------------------------
-----------------------------------GSATAGVGVGIYA----------NYKGNGESTLT-ME
RK-----------VGLGYEYGVFERNYHYGE--RQ-------------------------------------
------------------------------------------------------------------------
--------------------------------------------------------------VNRGYIEGCA
ATGE---------------------SKYCAGSHTDKISPYIYKDE-------------------------YK
SKSKARGITISQ--GKEMVGNPNGPSIIHGPG----------------------------------------
------------------------------------------------------------------------
------------------------------------------------------------------------
-------------HSWDLGAHGFPSTY---------------------------------------------
------------------------------------------------------------------------
------------------------------------------------------------------------
------------------------------------------------------------------------
------------------------------------------------------------------------
---------------------------
>15A5_5
------------------------------------------------------------------------
---------------------------------ASTNSIG---SYGSVKSASDTYEKYATSKSLK-------
------------------------------------------------------------------------
------------------------------------------------------------------------
------------------------------------------------------------------------
-------------DVAERYAAGKSVG----------------------------------------------
------------------------------------------------------------------------
-----------------------------------GSATAGVGVGIYA----------NYKGNGESTLT-ME
RK-----------VGLGYEYGVFERNYHYGE--RQ-------------------------------------
------------------------------------------------------------------------

--------------------------------------------------------------VNRGYIEGCA
ATGE---------------------SKYCAGSHTDKISPYIYKDE-------------------------YK
SKSKARGITISQ--GKEMVGNPNGPSIIHGPG----------------------------------------
------------------------------------------------------------------------
------------------------------------------------------------------------
-------------HSWDLGAHGFPSTY---------------------------------------------
------------------------------------------------------------------------
------------------------------------------------------------------------
------------------------------------------------------------------------
------------------------------------------------------------------------
---------------------------
>3027STDY5784958_5
------------------------------------------------------------------------
---------------------------------ASTNSIG---SYGSVKSASDTYEKYATSKSLK-------
------------------------------------------------------------------------
------------------------------------------------------------------------
------------------------------------------------------------------------
-------------DVAERYAAGKSVG----------------------------------------------
------------------------------------------------------------------------
-----------------------------------GSATAGVGVGIYA----------NYKGNGESTLT-ME
RK-----------VGLGYEYGVFERNYHYGE--RQ-------------------------------------
------------------------------------------------------------------------
--------------------------------------------------------------VNRGYIEGCA
ATGE---------------------SKYCAGSHTDKISPYIYKDE-------------------------YK
SKSKARGITISQ--GKEMVGNPNGPSIIHGPG----------------------------------------
------------------------------------------------------------------------
------------------------------------------------------------------------
-------------HSWDLGAHGFPSTY---------------------------------------------
------------------------------------------------------------------------
------------------------------------------------------------------------
------------------------------------------------------------------------
------------------------------------------------------------------------
---------------------------

```
>Ab4653_5
------------------------------------------------------------------------
---------------------------------ASTNSIG---SYGSVKSASDTYEKYATSKSLK-------
------------------------------------------------------------------------
------------------------------------------------------------------------
------------------------------------------------------------------------
-------------DVAERYAAGKSVG----------------------------------------------
------------------------------------------------------------------------
-----------------------------------GSATAGVGVGIYA----------NYKGNGESTLT-ME
RK-----------VGLGYEYGVFERNYHYGE--RQ-------------------------------------
------------------------------------------------------------------------
--------------------------------------------------------------VNRGYIEGCA
ATGE---------------------SKYCAGSHTDKISPYIYKDE-------------------------YK
SKSKARGITISQ--GKEMVGNPNGPSIIHGPG----------------------------------------
------------------------------------------------------------------------
------------------------------------------------------------------------
-------------HSWDLGAHGFPSTY---------------------------------------------
------------------------------------------------------------------------
------------------------------------------------------------------------
------------------------------------------------------------------------
------------------------------------------------------------------------
---------------------------
>Ab4977_5
------------------------------------------------------------------------
---------------------------------ASTNSIG---SYGSVKSASDTYEKYATSKSLK-------
------------------------------------------------------------------------
------------------------------------------------------------------------
------------------------------------------------------------------------
-------------DVAERYAAGKSVG----------------------------------------------
------------------------------------------------------------------------
-----------------------------------GSATAGVGVGIYA----------NYKGNGESTLT-ME
RK-----------VGLGYEYGVFERNYHYGE--RQ-------------------------------------
------------------------------------------------------------------------
```

```
--------------------------------------------------------------VNRGYIEGCA
ATGE---------------------SKYCAGSHTDKISPYIYKDE-------------------------YK
SKSKARGITISQ--GKEMVGNPNGPSIIHGPG----------------------------------------
------------------------------------------------------------------------
------------------------------------------------------------------------
-------------HSWDLGAHGFPSTY---------------------------------------------
------------------------------------------------------------------------
------------------------------------------------------------------------
------------------------------------------------------------------------
------------------------------------------------------------------------
---------------------------
>ACICU_5
------------------------------------------------------------------------
---------------------------------ASTNSIG---SYGSVKSASDTYEKYATSKSLK-------
------------------------------------------------------------------------
------------------------------------------------------------------------
------------------------------------------------------------------------
-------------DVAERYAAGKSVG----------------------------------------------
------------------------------------------------------------------------
-----------------------------------GSATAGVGVGIYA----------NYKGNGESTLT-ME
RK-----------VGLGYEYGVFERNYHYGE--RQ-------------------------------------
------------------------------------------------------------------------
--------------------------------------------------------------VNRGYIEGCA
ATGE---------------------SKYCAGSHTDKISPYIYKDE-------------------------YK
SKSKARGITISQ--GKEMVGNPNGPSIIHGPG----------------------------------------
------------------------------------------------------------------------
------------------------------------------------------------------------
-------------HSWDLGAHGFPSTY---------------------------------------------
------------------------------------------------------------------------
------------------------------------------------------------------------
------------------------------------------------------------------------
------------------------------------------------------------------------
---------------------------
```

```
>AF-673_5
------------------------------------------------------------------------
---------------------------------ASTNSIG---SYGSVKSASDTYEKYATSKSLK-------
------------------------------------------------------------------------
------------------------------------------------------------------------
------------------------------------------------------------------------
-------------DVAERYAAGKSVG----------------------------------------------
------------------------------------------------------------------------
-----------------------------------GSATAGVGVGIYA----------NYKGNGESTLT-ME
RK-----------VGLGYEYGVFERNYHYGE--RQ-------------------------------------
------------------------------------------------------------------------
--------------------------------------------------------------VNRGYIEGCA
ATGE---------------------SKYCAGSHTDKISPYIYKDE-------------------------YK
SKSKARGITISQ--GKEMVGNPNGPSIIHGPG----------------------------------------
------------------------------------------------------------------------
------------------------------------------------------------------------
-------------HSWDLGAHGFPSTY---------------------------------------------
------------------------------------------------------------------------
------------------------------------------------------------------------
------------------------------------------------------------------------
------------------------------------------------------------------------
---------------------------
>AR_0056_5
------------------------------------------------------------------------
---------------------------------ASTNSIG---SYGSVKSASDTYEKYATSKSLK-------
------------------------------------------------------------------------
------------------------------------------------------------------------
------------------------------------------------------------------------
-------------DVAERYAAGKSVG----------------------------------------------
------------------------------------------------------------------------
-----------------------------------GSATAGVGVGIYA----------NYKGNGESTLT-ME
RK-----------VGLGYEYGVFERNYHYGE--RQ-------------------------------------
------------------------------------------------------------------------
```

------------------------------------------------------------VNRGYIEGCA
ATGE--------------------SKYCAGSHTDKISPYIYKDE------------------------YK
SKSKARGITISQ--GKEMVGNPNGPSIIHGPG--------------------------------------
----------------------------------------------------------------------
----------------------------------------------------------------------
-------------HSWDLGAHGFPSTY-------------------------------------------
----------------------------------------------------------------------
----------------------------------------------------------------------
----------------------------------------------------------------------
----------------------------------------------------------------------
--------------------------
>AR_0102_5
----------------------------------------------------------------------
--------------------------------ASTNSIG---SYGSVKSASDTYEKYATSKSLK------
----------------------------------------------------------------------
----------------------------------------------------------------------
----------------------------------------------------------------------
-------------DVAERYAAGKSVG--------------------------------------------
----------------------------------------------------------------------
----------------------------------GSATAGVGVGIYA---------NYKGNGESTLT-ME
RK-----------VGLGYEYGVFERNYHYGE--RQ-----------------------------------
----------------------------------------------------------------------
------------------------------------------------------------VNRGYIEGCA
ATGE--------------------SKYCAGSHTDKISPYIYKDE------------------------YK
SKSKARGITISQ--GKEMVGNPNGPSIIHGPG--------------------------------------
----------------------------------------------------------------------
----------------------------------------------------------------------
-------------HSWDLGAHGFPSTY-------------------------------------------
----------------------------------------------------------------------
----------------------------------------------------------------------
----------------------------------------------------------------------
----------------------------------------------------------------------
--------------------------

```
>BJAB07104_5
------------------------------------------------------------------------
---------------------------------ASTNSIG---SYGSVKSASDTYEKYATSKSLK-------
------------------------------------------------------------------------
------------------------------------------------------------------------
------------------------------------------------------------------------
-------------DVAERYAAGKSVG----------------------------------------------
------------------------------------------------------------------------
-----------------------------------GSATAGVGVGIYA----------NYKGNGESTLT-ME
RK-----------VGLGYEYGVFERNYHYGE--RQ-------------------------------------
------------------------------------------------------------------------
--------------------------------------------------------------VNRGYIEGCA
ATGE---------------------SKYCAGSHTDKISPYIYKDE-------------------------YK
SKSKARGITISQ--GKEMVGNPNGPSIIHGPG----------------------------------------
------------------------------------------------------------------------
------------------------------------------------------------------------
-------------HSWDLGAHGFPSTY---------------------------------------------
------------------------------------------------------------------------
------------------------------------------------------------------------
------------------------------------------------------------------------
------------------------------------------------------------------------
---------------------------
>BJAB0868_5
------------------------------------------------------------------------
---------------------------------ASTNSIG---SYGSVKSASDTYEKYATSKSLK-------
------------------------------------------------------------------------
------------------------------------------------------------------------
------------------------------------------------------------------------
-------------DVAERYAAGKSVG----------------------------------------------
------------------------------------------------------------------------
-----------------------------------GSATAGVGVGIYA----------NYKGNGESTLT-ME
RK-----------VGLGYEYGVFERNYHYGE--RQ-------------------------------------
------------------------------------------------------------------------
```

--------------------------------------------------------------VNRGYIEGCA
ATGE---------------------SKYCAGSHTDKISPYIYKDE-------------------------YK
SKSKARGITISQ--GKEMVGNPNGPSIIHGPG----------------------------------------
------------------------------------------------------------------------
------------------------------------------------------------------------
-------------HSWDLGAHGFPSTY---------------------------------------------
------------------------------------------------------------------------
------------------------------------------------------------------------
------------------------------------------------------------------------
------------------------------------------------------------------------
---------------------------
>CMC-CR-MDR-Ab4_5
------------------------------------------------------------------------
---------------------------------ASTNSIG---SYGSVKSASDTYEKYATSKSLK-------
------------------------------------------------------------------------
------------------------------------------------------------------------
------------------------------------------------------------------------
-------------DVAERYAAGKSVG----------------------------------------------
------------------------------------------------------------------------
-----------------------------------GSATAGVGVGIYA----------NYKGNGESTLT-ME
RK-----------VGLGYEYGVFERNYHYGE--RQ-------------------------------------
------------------------------------------------------------------------
--------------------------------------------------------------VNRGYIEGCA
ATGE---------------------SKYCAGSHTDKISPYIYKDE-------------------------YK
SKSKARGITISQ--GKEMVGNPNGPSIIHGPG----------------------------------------
------------------------------------------------------------------------
------------------------------------------------------------------------
-------------HSWDLGAHGFPSTY---------------------------------------------
------------------------------------------------------------------------
------------------------------------------------------------------------
------------------------------------------------------------------------
------------------------------------------------------------------------
---------------------------

```
>CMC-CR-MDR-Ab66_5
------------------------------------------------------------------------
---------------------------------ASTNSIG---SYGSVKSASDTYEKYATSKSLK-------
------------------------------------------------------------------------
------------------------------------------------------------------------
------------------------------------------------------------------------
-------------DVAERYAAGKSVG----------------------------------------------
------------------------------------------------------------------------
-----------------------------------GSATAGVGVGIYA----------NYKGNGESTLT-ME
RK-----------VGLGYEYGVFERNYHYGE--RQ-------------------------------------
------------------------------------------------------------------------
--------------------------------------------------------------VNRGYIEGCA
ATGE---------------------SKYCAGSHTDKISPYIYKDE-------------------------YK
SKSKARGITISQ--GKEMVGNPNGPSIIHGPG----------------------------------------
------------------------------------------------------------------------
------------------------------------------------------------------------
-------------HSWDLGAHGFPSTY---------------------------------------------
------------------------------------------------------------------------
------------------------------------------------------------------------
------------------------------------------------------------------------
------------------------------------------------------------------------
---------------------------
>CMC-MDR-Ab59_5
------------------------------------------------------------------------
---------------------------------ASTNSIG---SYGSVKSASDTYEKYATSKSLK-------
------------------------------------------------------------------------
------------------------------------------------------------------------
------------------------------------------------------------------------
-------------DVAERYAAGKSVG----------------------------------------------
------------------------------------------------------------------------
-----------------------------------GSATAGVGVGIYA----------NYKGNGESTLT-ME
RK-----------VGLGYEYGVFERNYHYGE--RQ-------------------------------------
------------------------------------------------------------------------
```

--------------------------------------------------------------VNRGYIEGCA
ATGE---------------------SKYCAGSHTDKISPYIYKDE-------------------------YK
SKSKARGITISQ--GKEMVGNPNGPSIIHGPG----------------------------------------
------------------------------------------------------------------------
------------------------------------------------------------------------
-------------HSWDLGAHGFPSTY---------------------------------------------
------------------------------------------------------------------------
------------------------------------------------------------------------
------------------------------------------------------------------------
------------------------------------------------------------------------
---------------------------
>HRAB-85_5
------------------------------------------------------------------------
---------------------------------ASTNSIG---SYGSVKSASDTYEKYATSKSLK-------
------------------------------------------------------------------------
------------------------------------------------------------------------
------------------------------------------------------------------------
-------------DVAERYAAGKSVG----------------------------------------------
------------------------------------------------------------------------
-----------------------------------GSATAGVGVGIYA----------NYKGNGESTLT-ME
RK-----------VGLGYEYGVFERNYHYGE--RQ-------------------------------------
------------------------------------------------------------------------
--------------------------------------------------------------VNRGYIEGCA
ATGE---------------------SKYCAGSHTDKISPYIYKDE-------------------------YK
SKSKARGITISQ--GKEMVGNPNGPSIIHGPG----------------------------------------
------------------------------------------------------------------------
------------------------------------------------------------------------
-------------HSWDLGAHGFPSTY---------------------------------------------
------------------------------------------------------------------------
------------------------------------------------------------------------
------------------------------------------------------------------------
------------------------------------------------------------------------
---------------------------

```
>KBN10P02143_5
------------------------------------------------------------------------
---------------------------------ASTNSIG---SYGSVKSASDTYEKYATSKSLK-------
------------------------------------------------------------------------
------------------------------------------------------------------------
------------------------------------------------------------------------
-------------DVAERYAAGKSVG----------------------------------------------
------------------------------------------------------------------------
-----------------------------------GSATAGVGVGIYA----------NYKGNGESTLT-ME
RK-----------VGLGYEYGVFERNYHYGE--RQ-------------------------------------
------------------------------------------------------------------------
--------------------------------------------------------------VNRGYIEGCA
ATGE---------------------SKYCAGSHTDKISPYIYKDE-------------------------YK
SKSKARGITISQ--GKEMVGNPNGPSIIHGPG----------------------------------------
------------------------------------------------------------------------
------------------------------------------------------------------------
-------------HSWDLGAHGFPSTY---------------------------------------------
------------------------------------------------------------------------
------------------------------------------------------------------------
------------------------------------------------------------------------
------------------------------------------------------------------------
---------------------------
>MDR-TJ_5
------------------------------------------------------------------------
---------------------------------ASTNSIG---SYGSVKSASDTYEKYATSKSLK-------
------------------------------------------------------------------------
------------------------------------------------------------------------
------------------------------------------------------------------------
-------------DVAERYAAGKSVG----------------------------------------------
------------------------------------------------------------------------
-----------------------------------GSATAGVGVGIYA----------NYKGNGESTLT-ME
RK-----------VGLGYEYGVFERNYHYGE--RQ-------------------------------------
------------------------------------------------------------------------
```

--------------------------------------------------------------VNRGYIEGCA
ATGE---------------------SKYCAGSHTDKISPYIYKDE-------------------------YK
SKSKARGITISQ--GKEMVGNPNGPSIIHGPG----------------------------------------
------------------------------------------------------------------------
------------------------------------------------------------------------
-------------HSWDLGAHGFPSTY---------------------------------------------
------------------------------------------------------------------------
------------------------------------------------------------------------
------------------------------------------------------------------------
------------------------------------------------------------------------
---------------------------
>MDR-ZJ06_5
------------------------------------------------------------------------
---------------------------------ASTNSIG---SYGSVKSASDTYEKYATSKSLK-------
------------------------------------------------------------------------
------------------------------------------------------------------------
------------------------------------------------------------------------
-------------DVAERYAAGKSVG----------------------------------------------
------------------------------------------------------------------------
-----------------------------------GSATAGVGVGIYA----------NYKGNGESTLT-ME
RK-----------VGLGYEYGVFERNYHYGE--RQ-------------------------------------
------------------------------------------------------------------------
--------------------------------------------------------------VNRGYIEGCA
ATGE---------------------SKYCAGSHTDKISPYIYKDE-------------------------YK
SKSKARGITISQ--GKEMVGNPNGPSIIHGPG----------------------------------------
------------------------------------------------------------------------
------------------------------------------------------------------------
-------------HSWDLGAHGFPSTY---------------------------------------------
------------------------------------------------------------------------
------------------------------------------------------------------------
------------------------------------------------------------------------
------------------------------------------------------------------------
---------------------------

```
>NCGM237_5
------------------------------------------------------------------------
---------------------------------ASTNSIG---SYGSVKSASDTYEKYATSKSLK-------
------------------------------------------------------------------------
------------------------------------------------------------------------
------------------------------------------------------------------------
-------------DVAERYAAGKSVG----------------------------------------------
------------------------------------------------------------------------
-----------------------------------GSATAGVGVGIYA----------NYKGNGESTLT-ME
RK-----------VGLGYEYGVFERNYHYGE--RQ-------------------------------------
------------------------------------------------------------------------
--------------------------------------------------------------VNRGYIEGCA
ATGE---------------------SKYCAGSHTDKISPYIYKDE-------------------------YK
SKSKARGITISQ--GKEMVGNPNGPSIIHGPG----------------------------------------
------------------------------------------------------------------------
------------------------------------------------------------------------
-------------HSWDLGAHGFPSTY---------------------------------------------
------------------------------------------------------------------------
------------------------------------------------------------------------
------------------------------------------------------------------------
------------------------------------------------------------------------
---------------------------
>SMC_Paed_Ab_BL01_5
------------------------------------------------------------------------
---------------------------------ASTNSIG---SYGSVKSASDTYEKYATSKSLK-------
------------------------------------------------------------------------
------------------------------------------------------------------------
------------------------------------------------------------------------
-------------DVAERYAAGKSVG----------------------------------------------
------------------------------------------------------------------------
-----------------------------------GSATAGVGVGIYA----------NYKGNGESTLT-ME
RK-----------VGLGYEYGVFERNYHYGE--RQ-------------------------------------
------------------------------------------------------------------------
```

```
--------------------------------------------------------------VNRGYIEGCA
ATGE---------------------SKYCAGSHTDKISPYIYKDE-------------------------YK
SKSKARGITISQ--GKEMVGNPNGPSIIHGPG----------------------------------------
------------------------------------------------------------------------
------------------------------------------------------------------------
-------------HSWDLGAHGFPSTY---------------------------------------------
------------------------------------------------------------------------
------------------------------------------------------------------------
------------------------------------------------------------------------
------------------------------------------------------------------------
---------------------------
>SSA6_5
------------------------------------------------------------------------
---------------------------------ASTNSIG---SYGSVKSASDTYEKYATSKSLK-------
------------------------------------------------------------------------
------------------------------------------------------------------------
------------------------------------------------------------------------
-------------DVAERYAAGKSVG----------------------------------------------
------------------------------------------------------------------------
-----------------------------------GSATAGVGVGIYA----------NYKGNGESTLT-ME
RK-----------VGLGYEYGVFERNYHYGE--RQ-------------------------------------
------------------------------------------------------------------------
--------------------------------------------------------------VNRGYIEGCA
ATGE---------------------SKYCAGSHTDKISPYIYKDE-------------------------YK
SKSKARGITISQ--GKEMVGNPNGPSIIHGPG----------------------------------------
------------------------------------------------------------------------
------------------------------------------------------------------------
-------------HSWDLGAHGFPSTY---------------------------------------------
------------------------------------------------------------------------
------------------------------------------------------------------------
------------------------------------------------------------------------
------------------------------------------------------------------------
---------------------------
```

```
>TCDC-AB0715_5
------------------------------------------------------------------------
---------------------------------ASTNSIG---SYGSVKSASDTYEKYATSKSLK-------
------------------------------------------------------------------------
------------------------------------------------------------------------
------------------------------------------------------------------------
-------------DVAERYAAGKSVG----------------------------------------------
------------------------------------------------------------------------
-----------------------------------GSATAGVGVGIYA----------NYKGNGESTLT-ME
RK-----------VGLGYEYGVFERNYHYGE--RQ-------------------------------------
------------------------------------------------------------------------
--------------------------------------------------------------VNRGYIEGCA
ATGE---------------------SKYCAGSHTDKISPYIYKDE-------------------------YK
SKSKARGITISQ--GKEMVGNPNGPSIIHGPG----------------------------------------
------------------------------------------------------------------------
------------------------------------------------------------------------
-------------HSWDLGAHGFPSTY---------------------------------------------
------------------------------------------------------------------------
------------------------------------------------------------------------
------------------------------------------------------------------------
------------------------------------------------------------------------
---------------------------
>TYTH-1_5
------------------------------------------------------------------------
---------------------------------ASTNSIG---SYGSVKSASDTYEKYATSKSLK-------
------------------------------------------------------------------------
------------------------------------------------------------------------
------------------------------------------------------------------------
-------------DVAERYAAGKSVG----------------------------------------------
------------------------------------------------------------------------
-----------------------------------GSATAGVGVGIYA----------NYKGNGESTLT-ME
RK-----------VGLGYEYGVFERNYHYGE--RQ-------------------------------------
------------------------------------------------------------------------
```

--------------------------------------------------------------VNRGYIEGCA
ATGE---------------------SKYCAGSHTDKISPYIYKDE-------------------------YK
SKSKARGITISQ--GKEMVGNPNGPSIIHGPG----------------------------------------
------------------------------------------------------------------------
------------------------------------------------------------------------
-------------HSWDLGAHGFPSTY---------------------------------------------
------------------------------------------------------------------------
------------------------------------------------------------------------
------------------------------------------------------------------------
------------------------------------------------------------------------
---------------------------
>USA2_5
------------------------------------------------------------------------
---------------------------------ASTNSIG---SYGSVKSASDTYEKYATSKSLK-------
------------------------------------------------------------------------
------------------------------------------------------------------------
------------------------------------------------------------------------
-------------DVAERYAAGKSVG----------------------------------------------
------------------------------------------------------------------------
-----------------------------------GSATAGVGVGIYA----------NYKGNGESTLT-ME
RK-----------VGLGYEYGVFERNYHYGE--RQ-------------------------------------
------------------------------------------------------------------------
--------------------------------------------------------------VNRGYIEGCA
ATGE---------------------SKYCAGSHTDKISPYIYKDE-------------------------YK
SKSKARGITISQ--GKEMVGNPNGPSIIHGPG----------------------------------------
------------------------------------------------------------------------
------------------------------------------------------------------------
-------------HSWDLGAHGFPSTY---------------------------------------------
------------------------------------------------------------------------
------------------------------------------------------------------------
------------------------------------------------------------------------
------------------------------------------------------------------------
---------------------------

>WCHAB005133_5
------------------------------------------------------------------------
---------------------------------ASTNSIG---SYGSVKSASDTYEKYATSKSLK-------
------------------------------------------------------------------------
------------------------------------------------------------------------
------------------------------------------------------------------------
-------------DVAERYAAGKSVG----------------------------------------------
------------------------------------------------------------------------
-----------------------------------GSATAGVGVGIYA----------NYKGNGESTLT-ME
RK-----------VGLGYEYGVFERNYHYGE--RQ-------------------------------------
------------------------------------------------------------------------
--------------------------------------------------------------VNRGYIEGCA
ATGE---------------------SKYCAGSHTDKISPYIYKDE-------------------------YK
SKSKARGITISQ--GKEMVGNPNGPSIIHGPG----------------------------------------
------------------------------------------------------------------------
------------------------------------------------------------------------
-------------HSWDLGAHGFPSTY---------------------------------------------
------------------------------------------------------------------------
------------------------------------------------------------------------
------------------------------------------------------------------------
------------------------------------------------------------------------
---------------------------
>XDR-BJ83_5
------------------------------------------------------------------------
---------------------------------ASTNSIG---SYGSVKSASDTYEKYATSKSLK-------
------------------------------------------------------------------------
------------------------------------------------------------------------
------------------------------------------------------------------------
-------------DVAERYAAGKSVG----------------------------------------------
------------------------------------------------------------------------
-----------------------------------GSATAGVGVGIYA----------NYKGNGESTLT-ME
RK-----------VGLGYEYGVFERNYHYGE--RQ-------------------------------------
------------------------------------------------------------------------

--------------------------------------------------------------VNRGYIEGCA
ATGE---------------------SKYCAGSHTDKISPYIYKDE-------------------------YK
SKSKARGITISQ--GKEMVGNPNGPSIIHGPG----------------------------------------
------------------------------------------------------------------------
------------------------------------------------------------------------
-------------HSWDLGAHGFPSTY---------------------------------------------
------------------------------------------------------------------------
------------------------------------------------------------------------
------------------------------------------------------------------------
------------------------------------------------------------------------
---------------------------
>XH386_5
------------------------------------------------------------------------
---------------------------------ASTNSIG---SYGSVKSASDTYEKYATSKSLK-------
------------------------------------------------------------------------
------------------------------------------------------------------------
------------------------------------------------------------------------
-------------DVAERYAAGKSVG----------------------------------------------
------------------------------------------------------------------------
-----------------------------------GSATAGVGVGIYA----------NYKGNGESTLT-ME
RK-----------VGLGYEYGVFERNYHYGE--RQ-------------------------------------
------------------------------------------------------------------------
--------------------------------------------------------------VNRGYIEGCA
ATGE---------------------SKYCAGSHTDKISPYIYKDE-------------------------YK
SKSKARGITISQ--GKEMVGNPNGPSIIHGPG----------------------------------------
------------------------------------------------------------------------
------------------------------------------------------------------------
-------------HSWDLGAHGFPSTY---------------------------------------------
------------------------------------------------------------------------
------------------------------------------------------------------------
------------------------------------------------------------------------
------------------------------------------------------------------------
---------------------------

```
>XH856_5
------------------------------------------------------------------------
---------------------------------ASTNSIG---SYGSVKSASDTYEKYATSKSLK-------
------------------------------------------------------------------------
------------------------------------------------------------------------
------------------------------------------------------------------------
-------------DVAERYAAGKSVG----------------------------------------------
------------------------------------------------------------------------
-----------------------------------GSATAGVGVGIYA----------NYKGNGESTLT-ME
RK-----------VGLGYEYGVFERNYHYGE--RQ-------------------------------------
------------------------------------------------------------------------
--------------------------------------------------------------VNRGYIEGCA
ATGE---------------------SKYCAGSHTDKISPYIYKDE-------------------------YK
SKSKARGITISQ--GKEMVGNPNGPSIIHGPG----------------------------------------
------------------------------------------------------------------------
------------------------------------------------------------------------
-------------HSWDLGAHGFPSTY---------------------------------------------
------------------------------------------------------------------------
------------------------------------------------------------------------
------------------------------------------------------------------------
------------------------------------------------------------------------
---------------------------
>YU-R612_5
------------------------------------------------------------------------
---------------------------------ASTNSIG---SYGSVKSASDTYEKYATSKSLK-------
------------------------------------------------------------------------
------------------------------------------------------------------------
------------------------------------------------------------------------
-------------DVAERYAAGKSVG----------------------------------------------
------------------------------------------------------------------------
-----------------------------------GSATAGVGVGIYA----------NYKGNGESTLT-ME
RK-----------VGLGYEYGVFERNYHYGE--RQ-------------------------------------
------------------------------------------------------------------------
```

```
--------------------------------------------------------------VNRGYIEGCA
ATGE---------------------SKYCAGSHTDKISPYIYKDE-------------------------YK
SKSKARGITISQ--GKEMVGNPNGPSIIHGPG----------------------------------------
------------------------------------------------------------------------
------------------------------------------------------------------------
-------------HSWDLGAHGFPSTY---------------------------------------------
------------------------------------------------------------------------
------------------------------------------------------------------------
------------------------------------------------------------------------
------------------------------------------------------------------------
---------------------------
>Ab4568_5
------------------------------------------------------------------------
---------------------------------ASTNSIG---SYGSVKSASDTYEKYATSKSLK-------
------------------------------------------------------------------------
------------------------------------------------------------------------
------------------------------------------------------------------------
-------------DVAERYAAGKSVG----------------------------------------------
------------------------------------------------------------------------
-----------------------------------GSATAGVGVGIYA----------NYKGNGESTLT-ME
RK-----------VGLGYEYGVFERNYHYGE--RQ-------------------------------------
------------------------------------------------------------------------
--------------------------------------------------------------VNRGYIEGCA
ATGE---------------------SKYCAGSHTDKISPYIYKDE-------------------------YK
SKSKARGITISQ--GKEMVGNPNGPSIIHGPG----------------------------------------
------------------------------------------------------------------------
------------------------------------------------------------------------
-------------HSWDLGAHGFPST----------------------------------------------
------------------------------------------------------------------------
------------------------------------------------------------------------
------------------------------------------------------------------------
------------------------------------------------------------------------
---------------------------
```

```
>Tke10
------------------------------------------------------------------------
-------------------------------MQRPSALRS---WPGPAAADDCNRAGCCLAGIAC-------
------------------------------------------------------------------------
------------------------------------------------------------------------
------------------------RLHLSRWPAMSSTADWLDQAVEKVADGFGWLRDLVLGEFAEERPLSVV
IADMLLSLVPGV-VIVLSARDLAAVCLRLGKRYAADGSQATPQPPEWQEWV--LLVACLMTL----IAP---
------------------------------------------------------------------------
-----------------------------------IIGAVVGAVGTPV------------GSMAGALVGQEA
AA-----------FLRGLCLLLIRESQVILQ--TVVAFLG--------------------------------
------------------------------------------------------------------------
--KFTRGSVEFWLHQVRFASYEKDL-------------------------------------LAYLNLFLTK
VLAA------------TAKLRSYLGNWPFNQAASHLLVRLQAMEA---------------------QFYAVQ
VHALREIPHALM--QLDARLAKVLEQASDLPAQ----VAQAGVHAQKPEPMAMSAGRVTSG-----------
------------------------------------------------------------------------
------IGMRPRYLERSEGALGPPVPTSPTIAAHGANVHVFDHATATRAQK---------------------
-------------GIYGEVVSDQYMLGKGHENLLSAARGPRSLEMKPTGRGLDGVYRNAHPPPPFIITETKY
RTGGTFSPGSLPVTKGSKGFP---------------------------------------------------
-------------------------------------SARQMSDAWIEPRLDLDLGKKQAEEIAFSGYERWL
MIVDESGVVTKIVKLDEHAKFVGNVIH---------------------------------------------
------------------------------------------------------------------------
---------------------------
>Tke6
------------------------------------------------------------MRVSLDGLGQAV
DGDVTTTGAICIATGEGYLDEGRMVLRMGDPTTPCPLCGL---EGKVVEGVWHFISDGQPVAMDG-------
------------------------------------------------------------------------
------------------------------------------------------------------------
-----------------------------------------------------ALVDCGCPEGSNRVVAPLG
EMPPPRLPVAVR-TSLTPAGPGSSSALAGASPAMVGGLPGQMQPGFYVVPR--SMSFP--------------
------------------------------------------------------------------------
-----------------------------------QILLELASHDSTL------------PISYLQRLNPTF
EQ-----------GFKAGEIFVIGDPDNGYA--CT-------------------------------------
------------------------------------------------------------------------
```

```
--------QEEGQLMAAAERARRSL---------------------------------AVLDFAESDFMMEY
QAEI----------------AGLLSDASLSMGVGKDMMDHGLRQL---------------------RSTLGN
IERLHQQEFLRH--GHLNSPSFFAERRLLLEQL--DGQLKATFLNKHLNLGAYERLKKSLN-----------
------------------------------------------------------------------------
--ISTKSLVHHWSKAGGPGQIPGYATYLDKVAKLSKYLRYGGHVGIGLGGTS--------------------
-------------SYLKVQEACRAGETEGCKKIRLSEAGA--------------------------------
------------------------------------------------------------------------
-------------------------------------FAGGLAGGIVGGKIAGITALAVCGVFSAGTAGFGA
PVCGIALVGGGAFAGSIAGAEGGEQMGELIYESHYD------------------------------------
------------------------------------------------------------------------
---------------------------
>Tke2
------------------------------------------------------------------------
------------------------------------------------------------ECWST-------
------------------------------------------------------------------------
------------------------------------------------------------------------
------------------------------------------------------------------------
------------------------------------------------------------------------
------------------------------------------------------------------------
-----------------------------------ARKNYWKAEAKAP------------TRAYSPTNLVRM
AE----------------------GKAPKMT--VE-------------------------------------
------------------------------------------------------------------------
--------VISRKTDKISIREYALE-------------------------------------LHHNDIPQRV
GGAG--------------------VHDSSNLLALTPWEHEAVDQF---------------------------
------------------------------------------------------------------------
------------------------------------------------------------------------
------------------------------------------------------------------------
-------------RHVGSDLIRVIKGVDVW------------------------------------------
------------------------------------------------------------------------
------------------------------------------------------------------------
------------------------------------------------------------------------
------------------------------------------------------------------------
---------------------------
```

```
>TecA
------------------------------------------------------------------------
-----------------------------MQLTQLGGHVA---QSGIAERQKHAQALMFGMANID-------
------------------------------------------------------------------------
------------------------------------------------------------------------
------------------------------------------------------------------------
-------------EYVSRGVCYDAA-----------------------------------------------
------------------------------------------------------------------------
-----------------------------------AYVRYLLRADALI------------APDALLDT----
-------------AGQSWRTRFNFETGDQWD--GR-------------------------------------
------------------------------------------------------------------------
--------------------------------------------------------------------ASIP
AGTA------------------------VGFARGGNVFHAAIAVG------------------------GTR
IRAINGGLLGAG-------------------------WMNPVDLA---------------------------
------------------------------------------------------------------------
------------------------------------------------------------------------
-------------RALQPDPAGGFTYDRTTIRVHLSRL----------------------------------
------------------------------------------------------------------------
------------------------------------------------------------------------
------------------------------------------------------------------------
------------------------------------------------------------------------
---------------------------
>Tse1
------------------------------------------------------------------------
-----------------MDSLD-------QCIVNACKNSW---DKSYLAGTPNKDNCSGFVQSVA-------
------------------------------------------------------------------------
------------------------------------------------------------------------
------------------------------------------------------------------------
-------------------AELGVPMPRGNANAMVD------------------------------------
------------------------------------------------------------------------
-----------------------------------GLEQSWTKLASGA----------EAAQKAAQGFLVIA
GL-----------KGRTYGHVAVVISGPLYR--QK-------------------------------------
------------------------------------------------------------------------
```

```
--------------------------------------------------------------YPMCWCGSIA
GAVG----------------------QSQGLKSVGQVWNRTDRDR---------------------------
------------------------------------------------------------------------
------------------------------------------------------------------------
------------------------------------------------------------------------
-------------LNYYVYSLASCSLPRAS------------------------------------------
------------------------------------------------------------------------
------------------------------------------------------------------------
------------------------------------------------------------------------
------------------------------------------------------------------------
---------------------------
>Tse3
-----------------------------------------------------------------MTATSDL
IESLISYSWDDWQVTRQEARRV------IAAIRNDNVPDA---TIAALDKSGSLIKLFQRVGPPE-------
------------------------------------------------------------------------
------------------------------------------------------------------------
-----------------------------------LARSLIASIAGRTTMQRYQARNALIRSLINNPLGTQT
DNWIYFPTITFF-DICADLADAAGRLGFAAAGATGVASQAIQGPFS--------------------------
------------------------------------------------------------------------
-----------------------------------GVGATGVNPTDLP------------SIAFGDQLKLLN
KD-----------PATVTKYSNPLGDLGAYL--SQ--LSP--------------------------------
------------------------------------------------------------------------
-------QDKLNQAQTLVGQPISTL-------------------------------------FPDAYPGNPP
SRAK-------------------------VMSAAARKYDLTPQLI---------------------GAIILA
EQRDQTRDEDAK--DYQAAVSIKSANTSIGLGQ--VVVSTAIKYE---------------------------
------------------------------------------------------------------------
---------------LFTDLLGQPVRRGLSRKAVATLLASDEFNIFATARYIRYVA----------------
-------------NLASQQDLRKLPKTRGAFPSIDLRAYAGNPRNWPRDNVRA-------------------
------------------------------------------------------------------------
-------------------------------------LASEYTSRPWDDNLSPGWPMFVDDAYATFLDPGMR
FP----------------------------------------------------------------------
------------------------------------------------------------------------
---------------------------
```

```
>Tse6
--------------------------------------------------------------MDAQAAARLG
DEIAHGFGVAAMVAGAVAGALIGAAVVAATAATGGLAAVI---LAGSIAAGGLSMFQIVKGLTTI-------
----------------FELPEPTTGVLIRGSFNVYVNSRNAMRAGDDVSA----------------------
------------------------------------------------------------------------
------------------------TCSGLPLNHPLWPFPVLIAEGSATVYINGKPAARLQSKMVCGAHIKTG
SQNTFIGGPTERVAFVLDLEEWLHTGLEALGLAALAGGLLLAAMAGVAALV---------------------
------------------------------------------------------------------------
-----------------------------------GVVAIGGLMMGGM----------ALLGDLGDRLGPGY
RD-----------LFQGVAGMALLGFGPKLA--GR-------------------------------------
------------------------------------------------------------------------
----------RPAAVTSETAQRRAY-------------------------------------LNNKFGRSGN
LDHD------------------------INYRGNRETAAKFFKSK-----------------------DIDP
ADAESYMNGLDF--NHPVRVETLAPGKNLWQYQSPGAPQGNWYTL---------------------------
------------------------------------------------------------------------
-------SPRVQPTELGINPMGTNRAANTIEPKVLNSYRTTQKVEVLRSTAAPTD-----------------
-------------DFWSVKGQSYPAKGGAQQLFSNEKGSFGLLPREGS------------------------
------------------------------------------------------------------------
------------------------------------------------------------------------
------------------------------------------------------------------------
------------------------------------------------------------------------
---------------------------
>TseM
------------------------------------------------------------------------
-----------------------------MRRRQRASRAG---VVARRATPGDAQANSGRAPAR--------
------------------------------------------------------------------------
------------------------------------------------------------------------
------------------------------------------------------------------------
------------------------------------------------------------------------
------------------------------------------------------------------------
------------------------------------------PFQSFV----------------SNGAMLMN
KQ-----------IVAGIVAGLVSMSSHAQ------------------------------------------
------------------------------------------------------------------------
```

```
--------------------------------------------------------------LGQLFQSVKE
QVTQ----------------------------AATSQVNQGVRSA---------------------TDEAVQ
ATSSRTRKAINS--VRSPSSAAAATSTSPSAAE---------------------------------------
------------------------------------------------------------------------
------------------------------------------------------------------------
-------------ETNDATLSEARK-----------------------------------------------
------------------------------------------------------------------------
------------------------------------------------------------------------
------------------------------------------------------------------------
------------------------------------------------------------------------
---------------------------
>VgrG-5
--------------------------------MSSSHRHYADTALADVAALTDAASRADAAPLANARRFTFA
STAYDAATFDVVDIDGRDAISQPYRFEITLVSRSVRIDFA---KMLSCEATLAILPPFGEAGTTR----YAG
VLAEFEQKERFRDFTVYRAALVPRLWRLSLYKASDVYLNEQTIPDIVKRVLRAASFGKRNFRMRHRGVYRKR
SFVCQYDESHLDFVSRWMEKEGLYYYFEHDGRREKLEIVDDRRDQPGPADDLALRYLPATCLD---------
------------------------AGIESDRVQAFACRATPLPREVVLRDFNHRKAELSLEVREHVAHDGVG
ERVSSDEHFHTKDEGRRYAKLRAEALVCEGRRFAGESTAAGLRAGRFFALSGHYRKDFDGRYLVTAVTHRGS
QAHLLFPDLDAPFGATPGEPVYRAEFEAIAANLQYRPPRTTPKPRAA-------------------------
-----------------------------------GVVSAIVDGEGSG------------KRAELDEHGQYK
VR-----------FPFAHTAHPTNKASARIR--MATPYAG--------------------------------
-----------------------------DDRGMHLPLLKRTEVKIAFDGGDPDRPVIVGAVPNSSHRSVVT
RSNPDAHRILTEHNQLYMKDGSGAA---------------------------------TWLHAPNNHIGIGA
VGPG-----DGLALLTSGNKFDFSLGNAYSFSGGLKCSVSMGGNT---------------------DIYVGV
RNSLDVSANFLT--TLQGNLRWMLPGSRSFEIN--DSASTLLQTLHKQSATGAIRLSAGQD-----------
-----------------ASALLQKQLDKLKGTVRKFMIVSGLANAGAAATAAGLIKGGGALADLPWAGFGVS
AAQFAGATGFSTALMATSRTLLSKIAKLQEALPLVADLSLDKQGIALAAKNLTHATRMSLTVDGVSWSTHAK
GPGAAGAAMSVGKGRWGVEAAEHAHVHANDTLLFAVPADPTSKFDLKELIGLRRDLDECVKGIADLEADISE
NEVLSTDQNTFGVGALVPTPPSPANAVAAVAIKAKEAKLVELNAKRKLVA----------------------
-------------------------------------TKIDNLQQKLAKHAKNLSAARMSASDAEVGFKGNR
LVATAEGVTLAHAQGKAKLDVREAKIGVEAGKSSLELDESKLAAGCGGASLKLGSDGAIDVRATNVKLNGSA
SLKLDGQLIQLG------------------------------------------------------------
---------------------------
```

>AB030_22
------------------------------------------------------------------------
------------------NSKK---------------------TPAQLTADRARFELGKTSGAAA-------
------------------------------------------------------------------------
------------------------------------------------------------------------
------------------------------------------------------------------------
-------------DLQVGNV----------------------------------------------------
------------------------------------------------------------------------
-----------------------------------NITGISGRVGKNV------------HPQLKKALRDVP
KE----------------------------------------------------------------------
------------------------------------------------------------------------
--------------------------------------------------------------EQAPWHGACA
EVDA---------------------------INKALKKGMNIEGA---------------------TIDVVN
INSNDKRHGTHK--PACSSCSNVLKQFGVKSNE---------------------------------------
------------------------------------------------------------------------
------------------------------------------------------------------------
-------------K----------------------------------------------------------
------------------------------------------------------------------------
------------------------------------------------------------------------
------------------------------------------------------------------------
------------------------------------------------------------------------
---------------------------
>AbH12O-A2_22
------------------------------------------------------------------------
------------------NSKK---------------------TPAQLTADRARFELGKTSGAAA-------
------------------------------------------------------------------------
------------------------------------------------------------------------
------------------------------------------------------------------------
-------------DLQVGNV----------------------------------------------------
------------------------------------------------------------------------
-----------------------------------NITGISGRVGKNV------------HPQLKKALRDVP
KE----------------------------------------------------------------------
------------------------------------------------------------------------

--------------------------------------------------------------EQAPWHGACA
EVDA---------------------------INKALKKGMNIEGA---------------------TIDVVN
INSNDKRHGTHK--PACSSCSNVLKQFGVKSNE---------------------------------------
------------------------------------------------------------------------
------------------------------------------------------------------------
-------------K----------------------------------------------------------
------------------------------------------------------------------------
------------------------------------------------------------------------
------------------------------------------------------------------------
------------------------------------------------------------------------
---------------------------
>AF-401_22
------------------------------------------------------------------------
------------------NSKK---------------------TPAQLTADRARFELGKTSGAAA-------
------------------------------------------------------------------------
------------------------------------------------------------------------
------------------------------------------------------------------------
-------------DLQVGNV----------------------------------------------------
------------------------------------------------------------------------
-----------------------------------NITGISGRVGKNV------------HPQLKKALRDVP
KE----------------------------------------------------------------------
------------------------------------------------------------------------
--------------------------------------------------------------EQAPWHGACA
EVDA---------------------------INKALKKGMNIEGA---------------------TIDVVN
INSNDKRHGTHK--PACSSCSNVLKQFGVKSNE---------------------------------------
------------------------------------------------------------------------
------------------------------------------------------------------------
-------------K----------------------------------------------------------
------------------------------------------------------------------------
------------------------------------------------------------------------
------------------------------------------------------------------------
------------------------------------------------------------------------
---------------------------

>AR_0063_22
------------------------------------------------------------------------
------------------NSKK---------------------TPAQLTADRARFELGKTSGAAA-------
------------------------------------------------------------------------
------------------------------------------------------------------------
------------------------------------------------------------------------
-------------DLQVGNV----------------------------------------------------
------------------------------------------------------------------------
-----------------------------------NITGISGRVGKNV------------HPQLKKALRDVP
KE----------------------------------------------------------------------
------------------------------------------------------------------------
--------------------------------------------------------------EQAPWHGACA
EVDA---------------------------INKALKKGMNIEGA---------------------TIDVVN
INSNDKRHGTHK--PACSSCSNVLKQFGVKSNE---------------------------------------
------------------------------------------------------------------------
------------------------------------------------------------------------
-------------K----------------------------------------------------------
------------------------------------------------------------------------
------------------------------------------------------------------------
------------------------------------------------------------------------
------------------------------------------------------------------------
---------------------------
>AR_0101_22
------------------------------------------------------------------------
------------------NSKK---------------------TPAQLTADRARFELGKTSGAAA-------
------------------------------------------------------------------------
------------------------------------------------------------------------
------------------------------------------------------------------------
-------------DLQVGNV----------------------------------------------------
------------------------------------------------------------------------
-----------------------------------NITGISGRVGKNV------------HPQLKKALRDVP
KE----------------------------------------------------------------------
------------------------------------------------------------------------

```
--------------------------------------------------------------EQAPWHGACA
EVDA---------------------------INKALKKGMNIEGA---------------------TIDVVN
INSNDKRHGTHK--PACSSCSNVLKQFGVKSNE---------------------------------------
------------------------------------------------------------------------
------------------------------------------------------------------------
-------------K----------------------------------------------------------
------------------------------------------------------------------------
------------------------------------------------------------------------
------------------------------------------------------------------------
------------------------------------------------------------------------
---------------------------
>IOMTU433_22
------------------------------------------------------------------------
------------------NSKK---------------------TPAQLTADRARFELGKTSGAAA-------
------------------------------------------------------------------------
------------------------------------------------------------------------
------------------------------------------------------------------------
-------------DLQVGNV----------------------------------------------------
------------------------------------------------------------------------
-----------------------------------NITGISGRVGKNV------------HPQLKKALRDVP
KE----------------------------------------------------------------------
------------------------------------------------------------------------
--------------------------------------------------------------EQAPWHGACA
EVDA---------------------------INKALKKGMNIEGA---------------------TIDVVN
INSNDKRHGTHK--PACSSCSNVLKQFGVKSNE---------------------------------------
------------------------------------------------------------------------
------------------------------------------------------------------------
-------------K----------------------------------------------------------
------------------------------------------------------------------------
------------------------------------------------------------------------
------------------------------------------------------------------------
------------------------------------------------------------------------
---------------------------
```

```
>Tse4
------------------------------------------------------------------------
--------------MSQLPIPL-----------PLNESRW---RYVTASGGGMTVAFLAGSGGSI-------
------------------------------------------------------------------------
------------------------------------------------------------------------
------------------------------------------------------------------------
-------------TLLSPEGENVSFRYGGVGGGIGLG-----------------------------------
------------------------------------------------------------------------
-----------------------------------MRLPRFGKVNLNV------------KGKSVGGAGALE
-------------ALPSTGTVLVADGLVGRD--LS-------------------------------------
------------------------------------------------------------------------
---------------------------------------------------------------RGDFTGPCM
YVEL-----------------------GAGVIAGGSGTAILFGLD---------------------PKLLAA
VALASASPLTAA--LGASMSRQLLQSSRGAL-----------------------------------------
------------------------------------------------------------------------
------------------------------------------------------------------------
-------------LMAGVNVGAQFGGGAAAYLGALF------------------------------------
------------------------------------------------------------------------
------------------------------------------------------------------------
------------------------------------------------------------------------
------------------------------------------------------------------------
---------------------------
>VgrG2b
-------------------------------------------MRQRDLKFTFVVGEGKLAFDVVEFELEEA
LCEPFRLNLKLASDKNAIDFKQ-------VLDQPGTFTLWQDGRPARYVHGIVSHFTQGSSGFRR-------
---------------TRYELLLEPQLARLELCCNWRIFQEKSVPEILQALLKEHRVLDYEQRIYHEHLPREY
CVQAGDSDHYLHDRLAFEEGLVYYFRFDEHRHTLVCSDRLYVQERIAGGPVLF-------------------
------------------------SAQPEGDNPQPVLHSFRYSENVRTARQTQRDYSFKRPTYDQEHHLAGE
ALEHQGSSYERY-DYPGRYKRSGAGRPFTESRLRGHRRDARVASVSGDDPRLIPGHAFALEGHPRADFNAWW
RPVRVVHRGTQYAGQEEESADAPLGVSYDLRAELVPEDVEWRPAPLPRPRIDGPQ----------IATVVGP
AGEEIHCDEWGRVKVQFPWDREGRHDEFSTCWIRVAQNWAGADWGHMA------------IPRIGQEVIVDY
LDGDCDQPIVTGRTYRATNRPPYALPDHKILSTIKSKEYK--------------------------------
------------------------------------------------------------------------
```

-GSRANELRIDDTTAQISAALMSDH---------------------------------GASALHLGYLTHPR
PEGG-------KPRGEGFELRTDEHGAVRAAKGLLLSTEEQLRAG---------------------AGHLDR
GVVVQVLEAALE--LARELGDYAGEHQGVGHDAAPQQTLQEAVRDLGHGANDESGKSNGGKPAIALSGPAGI
AAATPASLTLAAGEHVDSVARQNQQVTAGQKVVINAGSDIGLFAQGGELRQITHQGPMLLQAQKNDIRLEAK
QSVEVSASQQHVLVTAKEHITLMCGGAYLTLKGGNIELGMPGNFVVKAAKHSHVGAASLEAEL---------
-------------PQFEVGETQRRFVLKQLDGQTAMPNVPYTITMANGEVIEGVTDAEGATQLLQKDAMNIA
KVDMKHTKSPASAVAGIAAAVGAAVAVGKLLGGPDAEAGRALSE----------------------------
-------------------------------------GEISLAKGVFGDSIDYSTVRLRDEDYVPWQGKDYV
MAPNGHIYFGEELRGVADWSLESLQRQGLFIHEMTHVWQHQHGVNVLLVGAYQQARQFLLGDQYAYRLEPGK
TLKDYNIEQQGDIVRDYFLEKNEFGEASANSRFAGVLKNFPTGY----------------------------
---------------------------
>AB031_26
-------------------------------------------------------------------QPVGG
KKSGLGIGFLFPNTMAGFAKNP----------EDARNLRA---LGAIRNNNLAAASVAGYGPYVV-------
------------------------------------------------------------------------
------------------------------------------------------------------------
------------------------------------------------------------------------
-------------AGTAAAGSAAAPVVGRVAAPFIGTGAQLEGAGLTAG-----------------------
------------------------------------------------------------------------
-----------------------------------TVGKAAVTSAGIG----------GGMDVISQGVKCKC
LK----------------DINLVRTGGVAAV--SA-------------------------------------
------------------------------------------------------------------------
--------------------------------------------------------------VTSGWGATTS
AAAG--------------------------------FGQVGWKSI--------------------------A
LNAPNMGQFLKN--NASGMVIFANEQMIGQAGS---------------------------------------
------------------------------------------------------------------------
------------------------------------------------------------------------
-------------RAVKAATEKDKSSKK--------------------------------------------
------------------------------------------------------------------------
------------------------------------------------------------------------
------------------------------------------------------------------------
------------------------------------------------------------------------
---------------------------

```
>VgrG1
-----------------------------------------MADSTGLQFTVKVGALPESTFVVAEFALDEG
LNRPFNLRLELASAQPDLDFGA-------VLDQPCELLVW---YNGELQRRVCGVVSDFAQGDSG-------
------------FRRTRYQLLVQPALWRLSLRQNSRIFQAQKPDEILSILLQEHGITDYAF--ALKNEHAKR
EYCVQYRETDLDFVNRLAAEEGMFYFHEFEAGKHRIVFADDAAALTAGPELFFNLGNRS-------------
------------------------LEQGPYVRQFHYREAVRPSDVELKDYSFKTPAYGLSRKKVGVELTHQR
DTYQHFDFPGRY-KEDPTGKAFAQHRLDALRNDAVAGSGKSNSAALLPGQT--FSLTEHPNG----SLNTDW
QVVHIRHTGLQPQALEEEGGSGPTVYHNEFSVVKASTTWRARIGSPEAPHKPMVDGP-------QIVTVVGP
DGEEIYCDEHGRVKLQFPWDRYGSSNDQSSCWVRVSQGWAGGQYGMMA------------IPRIGHEVIVSF
LEGDPDQPIITGRTFHATNRPPYELPANKTR--TVLRTET--------------------------------
-------------------------HQGEGFNELRFEDQAGKEEIYIHGQKDLNVLIENDAAWHIKHDEHRD
IDNERVTRIKANDHLTVEGEKRDQI--------------------------------KADYSLTVDASLHQK
LGQSLLMEAGSEVHHKAGMKIVMEAGAELTLKVGGSFVKLDASGV---------------------TLSGGS
IKMNSGGSPGSG--SGWGGKAPIQPGNVEVPTP---PPATLPAPAIHKSMESMAPLAKPCP-----------
---------------------------------------------------------------AAPPPVPPP
PAGGPPAPPPAPPQLAGGPMMPPPPPPVAATGEGKKSAKKWATVEISKADEALRYYSAQGY-----------
-------------TLLNNYLRDRPYKQREAIDTLLSRSYLNDEPTSASEFDQAMKAYVADVEAGLAKLPASP
DLDFVYRGLALDKPELAALKDQFTGVGNIIVEPGFMSTSPDKAWVNDTL-----------------------
-------------------------------------LRIRLPAGHGGRLLGEAAHFKGEAEMLFPTQTRLR
VDRVVSSMSGDFDSLLNTIPTSDNASDNRSRIKRLIEVSVL-------------------------------
------------------------------------------------------------------------
---------------------------
>VgrG-1
-----------------------------------MATLAYSIEVEGLEDETLVVRGFHGQESLSNSVFLGQ
ACYGFRYEVQLASRVSNLTAEQ-------MVDKRAELKLY---RNSQLVQRVHGIVRAFSQGDIG-------
------------HHHTFYQLTLVPALERLSLRHNSRIFQKQTVPEILSILLQEMGINDYAF--ALKRDGVQR
EFCVQYRESDIDFLHRLAAEEGLVYSFVHEA-------------GKHTLYFSDASDSLSKLPEPIPYNALVG
G-----------------------AIDTPYIHGLTYRTQAEVSEVQLKDYSFKKPAYSFLQTVQGTELDYQQ
TRYQHFDAPGRY-KDDVNGAAFSQIRLDYLRRHAHTATGQSNEPLLRAGYK--FDLQEHLDP----AMNRDW
VVVSINHQGEQPQALQEDGGSGATTYSNQFSLIPGHLHWRAEPQPKPQVDGPM------------IATVVGP
EGEEIFCDEHGRVKIHFPWDRYSNGNEQSSCWVRVSQGWAGSQYGFIA------------IPRIGHEVIVEF
LNGDPDQPIITGRTYHATNTPPYTLPEHKTK--TVLRTET--------------------------------
-------------------------HQGEGFNELSFEDQAGKEQIYLHAQKDFDGLIENDHTTVIRHDHHLT
```

VENDQFTQIKHNQHLTVEWESREAV------------------------------TGEQVLSIEGSLHVKTG
KVWV--NEAGTEIHVKAGQKVVIEAGSEITVKAGGSFVKVDPAGV---------------------HLSGAL
VNLNSGGSAGSG--SGFGGAMPALPGGLEPAVA--LAPPQTISYQALLQAEQANVPAVKVCPLAAQEATPAV
NSITPPPPPPIAPPMAPPQPIMNPQPTANAQPNLGRSTKATPDFPTHFPKSSIGIENELAGLVVAMPANSAQ
KFGYVKSAQGDALFMLTKDMNQGSYQRPPSLQDGKNYQNWQTHTVELVSYPCEMDDKAAVETRKQAMLWLAT
HFTTHIDQSNHQ-PLAPIQSEDGRFVIEITNAKHVIAAGNGISAESQGQTITMTPSGQQATVGVAAKGFGTS
ATPELRLLESAPWYQKSLKSQFASLTSAENLDDKELAANVFAYLTSIYLKTAELA-----------------
-------------------------------------KKFGIYINEWDPMSEQITPNANGLTDPKVKNAWEI
LPRTKPSKIVEILSKSDAKAVMKHIKPQLQSRYSESLSKNVFQYFQDGGEVAGHGINNATVGDKHSPELAIL
FEFRTVPNELQSYLPKTESTTKSEVKLLDQFDPMKRKTVIQQVESLVQNSGDAFDKWYQSYRDSMNQPPVKN
AKKIASANQKAQWVKEHNPQEWQRIIA
>VgrG-3
-----------------------------------MARLQFQLKVDGLEDESLVVRGFEGQESLSDSVWRCE
PCYGFRYQVDLASALSNLTAEQ-------FVDQTAHLTIL---RDGQVVQQINGIVRQLSKGDTG-------
------------HRHTFYSLTLVPALERLSLRSNSRIFQQQSVPEIISILLQEMGIEDYAF--ALKRECAQR
EFCVQYRETDLQFLHRIAAEEGLVYSHLHEA-------------QKHTLLFTDSSDSQPKLAKPVPYNALAG
G-----------------------EINLPYVVDLQFKTTAQVSHTELKDYSFKKPAYGFTQRTQGKDIAYQQ
PNYEHFDAPGRY-KDDANGKAFSQIRLEYLRRDALLADAKSDEPLLLAGVR--FDLQDHLDH----AMNRDW
LVVQANHQGTQPQALQEEGGSGATTYSNQLKLIPAHITWRARPCAKPQVDGPM------------IATVVGP
QGEEIYCDNFGRVKVHFPWDRYSSSNEKSSCWVRVAQEWAGSQYGSMA------------IPRVGHE---VI
VS-----------FLNGDPDQPI-ITGRTYH--AT--NTAPYALPDHKTKTVLRTETHQGQG----------
------------------------------YNELSFEDQAGSEQILLHAQKDWDALIEHDHTEVIRHDQHLT
VDNDRFTRIQRNQHLTVEGEVRSKI--ALDSSHEVGASLQHKVGQRI-----------A---VEAGKEISLK
SGAK----------------IVVEAGAELTLKAGGSFVKVDAGGV---------------------HLVGPA
INLNAGGSAGSG--SAYGGQLAAAPRMLAQAKP----VAELVQPDIAASMQSGAARVIDVA-----------
----------------------------------------------------------------SLPTMMPS
SANNTANDEPVAEEKTPERILKSDLLKPSDELEKLAKRQASAYRQGNHSDEVKLLQEALIKLGF--------
-------------DLGKAGADGDFGSKTKTAIEQFQKSYQPSHQTHPSYSIGAVDGIVGKGTLLALDEALMD
GWVYENNIYQIWPLGKTSEKYESAGRGPGVISTGNGDYGGASYGCYQMSSNLGVVQKYIQSSKFKEFFSGLN
PATKEFNVVWQDIASRYPQEFREEQHQFIKRTHYDIQIGHLRGKGLLFEHNRAAVHDLIWSTSVQFGGRTNL
IFNALNGQNMESMTDKDIIILVQDYKLVNTERLFKSSPSWWSDLKKRAVSEKKALLELEIDGLEVDIK----
------------------------------------------------------------------------
---------------------------

```
>AR_0078_29
----------------------------------------------------------------------MN
KKSLVTIQILDLFGSPISKAQY-------EVKNQRTGQVI---AAGTTNSSGCIVEISRDKGTAL-------
---------------DVYIKSMFKGSMVKVQSFVMSKDRMVVKITSPKVLLNLKTLTNQGNNGQYKRKTHIV
KKGDTLFEIAQKNHTTVRVLERLNKIDDPNKISIGQVIKLPVHIPASG------------------------
------------------------NHSHQDKPKHKHSAQSTNKPTASSTKTTPAPAAKRPANPASQTKQENG
LPEKAHQLYEDT-KKALNEAASSVSKILTIDDRSQDGGTPKANTTNICKTNPQCISSGKSELIREVNIRLAG
FGGALPTDEFTELTATC-------------------------------------------------------
-----------------------------------IRQFQRDYMGVPD------------TGKICGSVLVAL
DQ-----------FYDEYPISSFMGKAACNC--GKCSGFG--------------------------------
-----------------------------------------------------------NGKMGVQSGINTA
NEYPGIHRSLIWILKSLNFYLK---NEF--KDKK----------------------------INVAYIESGY
RCIE------------NNKKHERTSVNHMGLALDIHFHKNGVRTR--------------------ELSDMEF
IRKNIMAKKMGASEQRIRDKIYLEPKVFRSGEAGATSWVHFDITM---------------------------
------------------------------------------------------------------------
-----FLAKYFNNNMFKTKVSDLNGVKLVNLAASSRILSCGGIVALPKSQNNITDEL---------------
-------------VLSNEDIIDIMKVTETEVIKFKTEKYFLEQAAGVVDTIMNRTKSGVWGNSVRKVVNADR
QFSKITGPKSLDPYGSVENMPMSHVSRKVRNFVNSYLL----------------------------------
-------------------------------------ERANGKKSIIGENLNYANKYYSDEKNRKAWVDKFH
NEAVKNGMILGTGKAIHAHGTVRELRDKMPKPFKIVLPKDFKGI----------------------------
------------------------------------------------------------------------
---------------------------
>AR_0088_14
----------------------------------------------------------------------MN
KKSLVTIQILDLFGSPISKAQY-------VVKNQRTGKVI---AAGPTNSSGCIVEISQDKGTTL-------
---------------DVYIKSMFKGSMVKVQSFVMSKDRMVVKITSPKVLLDLKTLTNQGNNGQYKRKTHIV
KKGDTLFEIAQKNHTTVRALERLNKIDDPNKISVGQVIKLPVHIPASG------------------------
------------------------NHSHQDKPKHKHSAQSTNKPTASSAKTTPAPAAKRPANPASQTKQENG
LPEKAHQLYEDT-KKALNEAASSVSKILTVDDRSQDGGTPKANTTNICKTNPQCISSGKSELIREVNIRLAG
FGGALPTDEFTELTATC-------------------------------------------------------
-----------------------------------IKQFQRDYMGVPD------------TGKICGSVLAAL
DR-----------FRDEYGIASYFESMKCPC--GECSGFG--------------------------------
----------------------------------------------nRSRSGNFRFEMYNKTTRQYVEVYRDA
```

KEYNGMHRSLIWALKAMLFYFKKMPNSAGYKIVKISSGYRC---------------------IDNNWKNRRP
TTNH---MGCALDIVVHNKNNQVVSMSELENQVRIKWFGKYLNTS-----------------LGWSPDKFGL
ERISDGAKTWVHLDVREFNKKYKIDKLFSTSKD--GLNGDYLINLFKADEKANKILGCSGI-----------
------------------------------------------------------------------------
---VVSPNKNPNLNDSSLEDLIRQLGSVISHGEGNYESYNTGTKNVKGDKVGYSFKNPGKGTVTS-------
-------------KRIQQIIDNAEAKDGNDKNRLFATGKYQTTYYTLQEGIKQGYFTGSEVYDANMQEQVFR
EFLISKRTKLSQFVRKGIGSLRDAQYDAAQEWASVAVP----------------------------------
-------------------------------------KGLPLKNGKISDGNKSYYEKPGQNSSSPESTKMVL
EILEKIHRFHKDGK----------------------------------------------------------
------------------------------------------------------------------------
---------------------------

>CIP70.10_14

----------------------------------------------------------------------MN
KKSLVTIQILDLFGSPISKAQY-------VVKNQRTGKVI---AAGPTNSSGCIVEISQDKGTTL-------
---------------DVYIKSMFKGSMVKVQSFVMSKDRMVVKITSPKVLLDLKTLTNQGNNGQYKRKTHIV
KKGDTLFEIAQKNHTTVRALERLNKIDDPNKISVGQVIKLPVHIPASG------------------------
------------------------NHSHQDKPKHKHSAQSTNKPTASSAKTTPAPAAKRPANPASQTKQENG
LPEKAHQLYEDT-KKALNEAASSVSKILTVDDRSQDGGTPKANTTNICKTNPQCISSGKSELIREVNIRLAG
FGGALPTDEFTELTATC-------------------------------------------------------
-----------------------------------IKQFQRDYMGVPD------------TGKICGSVLAAL
DR-----------FRDEYGIASYFESMKCPC--GECSGFG--------------------------------
----------------------------------------------RSRSGNFRFEMYNKTTRQYVEVYRDA
KEYNGMHRSLIWALKAMLFYFKKMPNSAGYKIVKISSGYRC---------------------IDNNWKNRRP
TTNH---MGCALDIVVHNKNNQVVSMSELENQVRIKWFGKYLNTS-----------------LGWSPDKFGL
ERISDGAKTWVHLDVREFNKKYKIDKLFSTSKD--GLNGDYLINLFKADEKANKILGCSGI-----------
------------------------------------------------------------------------
---VVSPNKNPNLNDSSLEDLIRQLGSVISHGEGNYESYNTGTKNVKGDKVGYSFKNPGKGTVTS-------
-------------KRIQQIIDNAEAKDGNDKNRLFATGKYQTTYYTLQEGIKQGYFTGSEVYDANMQEQVFR
EFLISKRTKLSQFVRKGIGSLRDAQYDAAQEWASVAVP----------------------------------
-------------------------------------KGLPLKNGKISDGNKSYYEKPGQNSSSPESTKMVL
EILEKIHRFHKDGK----------------------------------------------------------
------------------------------------------------------------------------
---------------------------

```
>HWBA8_14
----------------------------------------------------------------------MN
KKSLVTIQILDLFGSPISKAQY-------VVKNQRTGKVI---AAGPTNSSGCIVEISQDKGTTL-------
---------------DVYIKSMFKGSMVKVQSFVMSKDRMVVKITSPKVLLDLKTLTNQGNNGQYKRKTHIV
KKGDTLFEIAQKNHTTVRALERLNKIDDPNKISVGQVIKLPVHIPASG------------------------
------------------------NHSHQDKPKHKHSAQSTNKPTASSAKTTPAPAAKRPANPASQTKQENG
LPEKAHQLYEDT-KKALNEAASSVSKILTVDDRSQDGGTPKANTTNICKTNPQCISSGKSELIREVNIRLAG
FGGALPTDEFTELTATC-------------------------------------------------------
-----------------------------------IKQFQRDYMGVPD------------TGKICGSVLAAL
DR-----------FRDEYGIASYFESMKCPC--GECSGFG--------------------------------
----------------------------------------------RSRSGNFRFEMYNKTTRQYVEVYRDA
KEYNGMHRSLIWALKAMLFYFKKMPNSAGYKIVKISSGYRC---------------------IDNNWKNRRP
TTNH---MGCALDIVVHNKNNQVVSMSELENQVRIKWFGKYLNTS-----------------LGWSPDKFGL
ERISDGAKTWVHLDVREFNKKYKIDKLFSTSKD--GLNGDYLINLFKADEKANKILGCSGI-----------
------------------------------------------------------------------------
---VVSPNKNPNLNDSSLEDLIRQLGSVISHGEGNYESYNTGTKNVKGDKVGYSFKNPGKGTVTS-------
-------------KRIQQIIDNAEAKDGNDKNRLFATGKYQTTYYTLQEGIKQGYFTGSEVYDANMQEQVFR
EFLISKRTKLSQFVRKGIGSLRDAQYDAAQEWASVAVP----------------------------------
-------------------------------------KGLPLKNGKISDGNKSYYEKPGQNSSSPESTKMVL
EILEKIHRFHKDGK----------------------------------------------------------
------------------------------------------------------------------------
---------------------------
>R2091_14
----------------------------------------------------------------------MN
KKSLVTIQILDLFGSPISKAQY-------VVKNQRTGKVI---AAGPTNSSGCIVEISQDKGTTL-------
---------------DVYIKSMFKGSMVKVQSFVMSKDRMVVKITSPKVLLDLKTLTNQGNNGQYKRKTHIV
KKGDTLFEIAQKNHTTVRALERLNKIDDPNKISVGQVIKLPVHIPASG------------------------
------------------------NHSHQDKPKHKHSAQSTNKPTASSAKTTPAPAAKRPANPASQTKQENG
LPEKAHQLYEDT-KKALNEAASSVSKILTVDDRSQDGGTPKANTTNICKTNPQCISSGKSELIREVNIRLAG
FGGALPTDEFTELTATC-------------------------------------------------------
-----------------------------------IKQFQRDYMGVPD------------TGKICGSVLAAL
DR-----------FRDEYGIASYFESMKCPC--GECSGFG--------------------------------
----------------------------------------------RSRSGNFRFEMYNKTTRQYVEVYRDA
```

```
KEYNGMHRSLIWALKAMLFYFKKMPNSAGYKIVKISSGYRC---------------------IDNNWKNRRP
TTNH---MGCALDIVVHNKNNQVVSMSELENQVRIKWFGKYLNTS-----------------LGWSPDKFGL
ERISDGAKTWVHLDVREFNKKYKIDKLFSTSKD--GLNGDYLINLFKADEKANKILGCSGI-----------
------------------------------------------------------------------------
---VVSPNKNPNLNDSSLEDLIRQLGSVISHGEGNYESYNTGTKNVKGDKVGYSFKNPGKGTVTS-------
-------------KRIQQIIDNAEAKDGNDKNRLFATGKYQTTYYTLQEGIKQGYFTGSEVYDANMQEQVFR
EFLISKRTKLSQFVRKGIGSLRDAQYDAAQEWASVAVP----------------------------------
-------------------------------------KGLPLKNGKISDGNKSYYEKPGQNSSSPESTKMVL
EILEKIHRFHKDGK----------------------------------------------------------
------------------------------------------------------------------------
---------------------------
>TYTH-1_14
----------------------------------------------------------------------MN
KKSLVTIQILDLFGSPISKAQY-------EVKNQRTGQVI---AAGTTNSSGCIVEISRDKGTAL-------
---------------DVYIKSMFKGSMVKVQSFVMSKDRMVVKITSPKVLLNLKTLTNQGNNGQYKRKTHIV
KKGDTLFEIAQKNHTTVRALERLNKIDDPNKISIGQVIKLPVHIPASG------------------------
------------------------NHSHQDKSKHKHSAQPVNKPTASSTKTTPAAAAKRPANPTSQTKQENG
LPEKVHQLYEDT-KKALNEAASSASKILTVDDRSQDGGTPKANTTNICKTNPQCISSGKSELIREVNIRLAG
FGGALPTDEFTELTATC-------------------------------------------------------
-----------------------------------IKQFQRDYMGVPD------------TGKICGSVLAAL
DR-----------FRDEYGIASYFESMKCPC--GECSGFG--------------------------------
----------------------------------------------RSRSGNFRFEMYNKTTRQYVEVYRDA
KEYNGMHRSLIWALKAMLFYFKKMPNSAGYKIVKISSGYRC---------------------IDNNWKNRRP
TTNH---MGCALDIVVHNKNNQVVSMSELENQVRIKWFGKYLNTS-----------------LGWSPDKFGL
ERISDGAKTWVHLDVREFNKKYKIDKLFSTSKD--GLNGDYLINLFKADEKANKILGCSGI-----------
------------------------------------------------------------------------
---VVSPNKNPNLNDSSLEDLIRQLGSVISHGEGNYESYNTGTKNVKGDKVGYSFKNPGKGTVTS-------
-------------KRIQQIIDNAEAKDGNDKNRLFATGKYQTTYYTLQEGIKQGYFTGSEVYDANMQEQVFR
EFLISKRTKLSQFVRKGIGSLRDAQYDAAQEWASVAVP----------------------------------
-------------------------------------KGLPLKNGKISDGNKSYYEKPGQNSSSPESTKMVL
EILEKIHRFHKDGK----------------------------------------------------------
------------------------------------------------------------------------
---------------------------
```

>6200_15
------------------------------------------------------------------------
---------------NSFNYGE---------------------MFGIPASAQSGLAYQGQRNY---------
------------------------------------------------------------------------
------------------------------------------------------------------------
------------------------------------------------------------------------
-------------ECYAETGELCKI-----------------------------------------------
------------------------------------------------------------------------
-----------------------------------KVPPLFDYVACSG-------------GGLGIGVGFVK
NQ-----------WTGEYYISGSKDSLLIPV--AK--SVA--------------------------------
------------------------------------------------------------------------
--------------------------------------------------------------QNKQFSAKDL
AGAS-------------------CVGGNIHNIPSYTKTTMTMGEI---------------------TNEFVS
GASVTVGGGAYG--AVANVVVPLVSKSSPVKGT----WASELGVGT--------------------------
------------------------------------------------------------------------
------------------------------------------------------------------------
-------------PGFNVGVSGTVSVDTILDAVKPSKK----------------------------------
------------------------------------------------------------------------
------------------------------------------------------------------------
------------------------------------------------------------------------
------------------------------------------------------------------------
---------------------------
>A1_15
------------------------------------------------------------------------
---------------NSFNYGE---------------------MFGIPASAQSGLAYQGQRNY---------
------------------------------------------------------------------------
------------------------------------------------------------------------
------------------------------------------------------------------------
-------------ECYAETGELCKI-----------------------------------------------
------------------------------------------------------------------------
-----------------------------------KVPPLFDYVACSG-------------GGLGIGVGFVK
NQ-----------WTGEYYISGSKDSLLIPV--AK--SVA--------------------------------
------------------------------------------------------------------------

```
--------------------------------------------------------------QNKQFSAKDL
AGAS-------------------CVGGNIHNIPSYTKTTMTMGEI---------------------TNEFVS
GASVTVGGGAYG--AVANVVVPLVSKSSPVKGT----WASELGVGT--------------------------
------------------------------------------------------------------------
------------------------------------------------------------------------
-------------PGFNVGVSGTVSVDTILDAVKPSKK----------------------------------
------------------------------------------------------------------------
------------------------------------------------------------------------
------------------------------------------------------------------------
------------------------------------------------------------------------
---------------------------
>A388_15
------------------------------------------------------------------------
---------------NSFNYGE---------------------MFGIPASAQSGLAYQGQRNY---------
------------------------------------------------------------------------
------------------------------------------------------------------------
------------------------------------------------------------------------
-------------ECYAETGELCKI-----------------------------------------------
------------------------------------------------------------------------
-----------------------------------KVPPLFDYVACSG-------------GGLGIGVGFVK
NQ-----------WTGEYYISGSKDSLLIPV--AK--SVA--------------------------------
------------------------------------------------------------------------
--------------------------------------------------------------QNKQFSAKDL
AGAS-------------------CVGGNIHNIPSYTKTTMTMGEI---------------------TNEFVS
GASVTVGGGAYG--AVANVVVPLVSKSSPVKGT----WASELGVGT--------------------------
------------------------------------------------------------------------
------------------------------------------------------------------------
-------------PGFNVGVSGTVSVDTILDAVKPSKK----------------------------------
------------------------------------------------------------------------
------------------------------------------------------------------------
------------------------------------------------------------------------
------------------------------------------------------------------------
---------------------------
```

>A85_15
------------------------------------------------------------------------
---------------NSFNYGE---------------------MFGIPASAQSGLAYQGQRNY---------
------------------------------------------------------------------------
------------------------------------------------------------------------
------------------------------------------------------------------------
-------------ECYAETGELCKI-----------------------------------------------
------------------------------------------------------------------------
-----------------------------------KVPPLFDYVACSG-------------GGLGIGVGFVK
NQ-----------WTGEYYISGSKDSLLIPV--AK--SVA--------------------------------
------------------------------------------------------------------------
--------------------------------------------------------------QNKQFSAKDL
AGAS-------------------CVGGNIHNIPSYTKTTMTMGEI---------------------TNEFVS
GASVTVGGGAYG--AVANVVVPLVSKSSPVKGT----WASELGVGT--------------------------
------------------------------------------------------------------------
------------------------------------------------------------------------
-------------PGFNVGVSGTVSVDTILDAVKPSKK----------------------------------
------------------------------------------------------------------------
------------------------------------------------------------------------
------------------------------------------------------------------------
------------------------------------------------------------------------
---------------------------
>AB0057_15
------------------------------------------------------------------------
---------------NSFNYGE---------------------MFGIPASAQSGLAYQGQRNY---------
------------------------------------------------------------------------
------------------------------------------------------------------------
------------------------------------------------------------------------
-------------ECYAETGELCKI-----------------------------------------------
------------------------------------------------------------------------
-----------------------------------KVPPLFDYVACSG-------------GGLGIGVGFVK
NQ-----------WTGEYYISGSKDSLLIPV--AK--SVA--------------------------------
------------------------------------------------------------------------

```
--------------------------------------------------------------QNKQFSAKDL
AGAS-------------------CVGGNIHNIPSYTKTTMTMGEI---------------------TNEFVS
GASVTVGGGAYG--AVANVVVPLVSKSSPVKGT----WASELGVGT--------------------------
------------------------------------------------------------------------
------------------------------------------------------------------------
-------------PGFNVGVSGTVSVDTILDAVKPSKK----------------------------------
------------------------------------------------------------------------
------------------------------------------------------------------------
------------------------------------------------------------------------
------------------------------------------------------------------------
---------------------------
>AB030_15
------------------------------------------------------------------------
---------------NSFNYGE---------------------MFGIPASAQSGLAYQGQRNY---------
------------------------------------------------------------------------
------------------------------------------------------------------------
------------------------------------------------------------------------
-------------ECYAETGELCKI-----------------------------------------------
------------------------------------------------------------------------
-----------------------------------KVPPLFDYVACSG-------------GGLGIGVGFVK
NQ-----------WTGEYYISGSKDSLLIPV--AK--SVA--------------------------------
------------------------------------------------------------------------
--------------------------------------------------------------QNKQFSAKDL
AGAS-------------------CVGGNIHNIPSYTKTTMTMGEI---------------------TNEFVS
GASVTVGGGAYG--AVANVVVPLVSKSSPVKGT----WASELGVGT--------------------------
------------------------------------------------------------------------
------------------------------------------------------------------------
-------------PGFNVGVSGTVSVDTILDAVKPSKK----------------------------------
------------------------------------------------------------------------
------------------------------------------------------------------------
------------------------------------------------------------------------
------------------------------------------------------------------------
---------------------------
```

```
>AB307-0294_15
------------------------------------------------------------------------
---------------NSFNYGE---------------------MFGIPASAQSGLAYQGQRNY---------
------------------------------------------------------------------------
------------------------------------------------------------------------
------------------------------------------------------------------------
-------------ECYAETGELCKI-----------------------------------------------
------------------------------------------------------------------------
-----------------------------------KVPPLFDYVACSG-------------GGLGIGVGFVK
NQ-----------WTGEYYISGSKDSLLIPV--AK--SVA--------------------------------
------------------------------------------------------------------------
--------------------------------------------------------------QNKQFSAKDL
AGAS-------------------CVGGNIHNIPSYTKTTMTMGEI---------------------TNEFVS
GASVTVGGGAYG--AVANVVVPLVSKSSPVKGT----WASELGVGT--------------------------
------------------------------------------------------------------------
------------------------------------------------------------------------
-------------PGFNVGVSGTVSVDTILDAVKPSKK----------------------------------
------------------------------------------------------------------------
------------------------------------------------------------------------
------------------------------------------------------------------------
------------------------------------------------------------------------
---------------------------
>AB5075-UW_15
------------------------------------------------------------------------
---------------NSFNYGE---------------------MFGIPASAQSGLAYQGQRNY---------
------------------------------------------------------------------------
------------------------------------------------------------------------
------------------------------------------------------------------------
-------------ECYAETGELCKI-----------------------------------------------
------------------------------------------------------------------------
-----------------------------------KVPPLFDYVACSG-------------GGLGIGVGFVK
NQ-----------WTGEYYISGSKDSLLIPV--AK--SVA--------------------------------
------------------------------------------------------------------------
```

------------------------------------------------------------QNKQFSAKDL
AGAS------------------CVGGNIHNIPSYTKTTMTMGEI--------------------TNEFVS
GASVTVGGGAYG--AVANVVVPLVSKSSPVKGT----WASELGVGT------------------------
----------------------------------------------------------------------
----------------------------------------------------------------------
-------------PGFNVGVSGTVSVDTILDAVKPSKK--------------------------------
----------------------------------------------------------------------
----------------------------------------------------------------------
----------------------------------------------------------------------
----------------------------------------------------------------------
--------------------------
>AbH12O-A2_15
----------------------------------------------------------------------
---------------NSFNYGE--------------------MFGIPASAQSGLAYQGQRNY--------
----------------------------------------------------------------------
----------------------------------------------------------------------
----------------------------------------------------------------------
-------------ECYAETGELCKI---------------------------------------------
----------------------------------------------------------------------
----------------------------------KVPPLFDYVACSG------------GGLGIGVGFVK
NQ-----------WTGEYYISGSKDSLLIPV--AK--SVA------------------------------
----------------------------------------------------------------------
------------------------------------------------------------QNKQFSAKDL
AGAS------------------CVGGNIHNIPSYTKTTMTMGEI--------------------TNEFVS
GASVTVGGGAYG--AVANVVVPLVSKSSPVKGT----WASELGVGT------------------------
----------------------------------------------------------------------
----------------------------------------------------------------------
-------------PGFNVGVSGTVSVDTILDAVKPSKK--------------------------------
----------------------------------------------------------------------
----------------------------------------------------------------------
----------------------------------------------------------------------
----------------------------------------------------------------------
--------------------------

```
>AF-401_15
------------------------------------------------------------------------
---------------NSFNYGE---------------------MFGIPASAQSGLAYQGQRNY---------
------------------------------------------------------------------------
------------------------------------------------------------------------
------------------------------------------------------------------------
-------------ECYAETGELCKI-----------------------------------------------
------------------------------------------------------------------------
-----------------------------------KVPPLFDYVACSG-------------GGLGIGVGFVK
NQ-----------WTGEYYISGSKDSLLIPV--AK--SVA--------------------------------
------------------------------------------------------------------------
--------------------------------------------------------------QNKQFSAKDL
AGAS-------------------CVGGNIHNIPSYTKTTMTMGEI---------------------TNEFVS
GASVTVGGGAYG--AVANVVVPLVSKSSPVKGT----WASELGVGT--------------------------
------------------------------------------------------------------------
------------------------------------------------------------------------
-------------PGFNVGVSGTVSVDTILDAVKPSKK----------------------------------
------------------------------------------------------------------------
------------------------------------------------------------------------
------------------------------------------------------------------------
------------------------------------------------------------------------
---------------------------
>AR_0063_15
------------------------------------------------------------------------
---------------NSFNYGE---------------------MFGIPASAQSGLAYQGQRNY---------
------------------------------------------------------------------------
------------------------------------------------------------------------
------------------------------------------------------------------------
-------------ECYAETGELCKI-----------------------------------------------
------------------------------------------------------------------------
-----------------------------------KVPPLFDYVACSG-------------GGLGIGVGFVK
NQ-----------WTGEYYISGSKDSLLIPV--AK--SVA--------------------------------
------------------------------------------------------------------------
```

```
--------------------------------------------------------------QNKQFSAKDL
AGAS-------------------CVGGNIHNIPSYTKTTMTMGEI---------------------TNEFVS
GASVTVGGGAYG--AVANVVVPLVSKSSPVKGT----WASELGVGT--------------------------
------------------------------------------------------------------------
------------------------------------------------------------------------
-------------PGFNVGVSGTVSVDTILDAVKPSKK----------------------------------
------------------------------------------------------------------------
------------------------------------------------------------------------
------------------------------------------------------------------------
------------------------------------------------------------------------
---------------------------
>AR_0078_15
------------------------------------------------------------------------
---------------NSFNYGE---------------------MFGIPASAQSGLAYQGQRNY---------
------------------------------------------------------------------------
------------------------------------------------------------------------
------------------------------------------------------------------------
-------------ECYAETGELCKI-----------------------------------------------
------------------------------------------------------------------------
-----------------------------------KVPPLFDYVACSG-------------GGLGIGVGFVK
NQ-----------WTGEYYISGSKDSLLIPV--AK--SVA--------------------------------
------------------------------------------------------------------------
--------------------------------------------------------------QNKQFSAKDL
AGAS-------------------CVGGNIHNIPSYTKTTMTMGEI---------------------TNEFVS
GASVTVGGGAYG--AVANVVVPLVSKSSPVKGT----WASELGVGT--------------------------
------------------------------------------------------------------------
------------------------------------------------------------------------
-------------PGFNVGVSGTVSVDTILDAVKPSKK----------------------------------
------------------------------------------------------------------------
------------------------------------------------------------------------
------------------------------------------------------------------------
------------------------------------------------------------------------
---------------------------
```

```
>AR_0083_15
------------------------------------------------------------------------
---------------NSFNYGE---------------------MFGIPASAQSGLAYQGQRNY---------
------------------------------------------------------------------------
------------------------------------------------------------------------
------------------------------------------------------------------------
-------------ECYAETGELCKI-----------------------------------------------
------------------------------------------------------------------------
-----------------------------------KVPPLFDYVACSG-------------GGLGIGVGFVK
NQ-----------WTGEYYISGSKDSLLIPV--AK--SVA--------------------------------
------------------------------------------------------------------------
--------------------------------------------------------------QNKQFSAKDL
AGAS-------------------CVGGNIHNIPSYTKTTMTMGEI---------------------TNEFVS
GASVTVGGGAYG--AVANVVVPLVSKSSPVKGT----WASELGVGT--------------------------
------------------------------------------------------------------------
------------------------------------------------------------------------
-------------PGFNVGVSGTVSVDTILDAVKPSKK----------------------------------
------------------------------------------------------------------------
------------------------------------------------------------------------
------------------------------------------------------------------------
------------------------------------------------------------------------
---------------------------
>AR_0101_15
------------------------------------------------------------------------
---------------NSFNYGE---------------------MFGIPASAQSGLAYQGQRNY---------
------------------------------------------------------------------------
------------------------------------------------------------------------
------------------------------------------------------------------------
-------------ECYAETGELCKI-----------------------------------------------
------------------------------------------------------------------------
-----------------------------------KVPPLFDYVACSG-------------GGLGIGVGFVK
NQ-----------WTGEYYISGSKDSLLIPV--AK--SVA--------------------------------
------------------------------------------------------------------------
```

```
--------------------------------------------------------------QNKQFSAKDL
AGAS-------------------CVGGNIHNIPSYTKTTMTMGEI---------------------TNEFVS
GASVTVGGGAYG--AVANVVVPLVSKSSPVKGT----WASELGVGT--------------------------
------------------------------------------------------------------------
------------------------------------------------------------------------
-------------PGFNVGVSGTVSVDTILDAVKPSKK----------------------------------
------------------------------------------------------------------------
------------------------------------------------------------------------
------------------------------------------------------------------------
------------------------------------------------------------------------
---------------------------
>D36_15
------------------------------------------------------------------------
---------------NSFNYGE---------------------MFGIPASAQSGLAYQGQRNY---------
------------------------------------------------------------------------
------------------------------------------------------------------------
------------------------------------------------------------------------
-------------ECYAETGELCKI-----------------------------------------------
------------------------------------------------------------------------
-----------------------------------KVPPLFDYVACSG-------------GGLGIGVGFVK
NQ-----------WTGEYYISGSKDSLLIPV--AK--SVA--------------------------------
------------------------------------------------------------------------
--------------------------------------------------------------QNKQFSAKDL
AGAS-------------------CVGGNIHNIPSYTKTTMTMGEI---------------------TNEFVS
GASVTVGGGAYG--AVANVVVPLVSKSSPVKGT----WASELGVGT--------------------------
------------------------------------------------------------------------
------------------------------------------------------------------------
-------------PGFNVGVSGTVSVDTILDAVKPSKK----------------------------------
------------------------------------------------------------------------
------------------------------------------------------------------------
------------------------------------------------------------------------
------------------------------------------------------------------------
---------------------------
```

>IOMTU433_15
------------------------------------------------------------------------
---------------NSFNYGE---------------------MFGIPASAQSGLAYQGQRNY---------
------------------------------------------------------------------------
------------------------------------------------------------------------
------------------------------------------------------------------------
-------------ECYAETGELCKI-----------------------------------------------
------------------------------------------------------------------------
-----------------------------------KVPPLFDYVACSG-------------GGLGIGVGFVK
NQ-----------WTGEYYISGSKDSLLIPV--AK--SVA--------------------------------
------------------------------------------------------------------------
--------------------------------------------------------------QNKQFSAKDL
AGAS-------------------CVGGNIHNIPSYTKTTMTMGEI---------------------TNEFVS
GASVTVGGGAYG--AVANVVVPLVSKSSPVKGT----WASELGVGT--------------------------
------------------------------------------------------------------------
------------------------------------------------------------------------
-------------PGFNVGVSGTVSVDTILDAVKPSKK----------------------------------
------------------------------------------------------------------------
------------------------------------------------------------------------
------------------------------------------------------------------------
------------------------------------------------------------------------
---------------------------
>USA15_15
------------------------------------------------------------------------
---------------NSFNYGE---------------------MFGIPASAQSGLAYQGQRNY---------
------------------------------------------------------------------------
------------------------------------------------------------------------
------------------------------------------------------------------------
-------------ECYAETGELCKI-----------------------------------------------
------------------------------------------------------------------------
-----------------------------------KVPPLFDYVACSG-------------GGLGIGVGFVK
NQ-----------WTGEYYISGSKDSLLIPV--AK--SVA--------------------------------
------------------------------------------------------------------------

--------------------------------------------------------------QNKQFSAKDL
AGAS-------------------CVGGNIHNIPSYTKTTMTMGEI---------------------TNEFVS
GASVTVGGGAYG--AVANVVVPLVSKSSPVKGT----WASELGVGT--------------------------
------------------------------------------------------------------------
------------------------------------------------------------------------
-------------PGFNVGVSGTVSVDTILDAVKPSKK----------------------------------
------------------------------------------------------------------------
------------------------------------------------------------------------
------------------------------------------------------------------------
------------------------------------------------------------------------
---------------------------
>WCHAB005078_15
------------------------------------------------------------------------
---------------NSFNYGE---------------------MFGIPASAQSGLAYQGQRNY---------
------------------------------------------------------------------------
------------------------------------------------------------------------
------------------------------------------------------------------------
-------------ECYAETGELCKI-----------------------------------------------
------------------------------------------------------------------------
-----------------------------------KVPPLFDYVACSG-------------GGLGIGVGFVK
NQ-----------WTGEYYISGSKDSLLIPV--AK--SVA--------------------------------
------------------------------------------------------------------------
--------------------------------------------------------------QNKQFSAKDL
AGAS-------------------CVGGNIHNIPSYTKTTMTMGEI---------------------TNEFVS
GASVTVGGGAYG--AVANVVVPLVSKSSPVKGT----WASELGVGT--------------------------
------------------------------------------------------------------------
------------------------------------------------------------------------
-------------PGFNVGVSGTVSVDTILDAVKPSKK----------------------------------
------------------------------------------------------------------------
------------------------------------------------------------------------
------------------------------------------------------------------------
------------------------------------------------------------------------
---------------------------

```
>SDF_15
------------------------------------------------------------------------
---------------NSFNYGE---------------------MFGIPASAQSGLAYQGQRNY---------
------------------------------------------------------------------------
------------------------------------------------------------------------
------------------------------------------------------------------------
-------------ECYAETGELCKI-----------------------------------------------
------------------------------------------------------------------------
-----------------------------------KVPPLFDYVACSG-------------GGLGIGVGFVK
NQ-----------WTGEYYISGSKDSLLIPV--AK--SVA--------------------------------
------------------------------------------------------------------------
--------------------------------------------------------------QNKQFSAKDL
AGAS-------------------CVGGNIHNIPSYTKTTMAMGEI---------------------TNEFVS
GASVTVGGGAYG--AVANVVVPLVSKSSPVKGT----WASELGVGT--------------------------
------------------------------------------------------------------------
------------------------------------------------------------------------
-------------PGFNVGVSGTVSVDTILDAVKPSKK----------------------------------
------------------------------------------------------------------------
------------------------------------------------------------------------
------------------------------------------------------------------------
------------------------------------------------------------------------
---------------------------
>Tle5
MLQKKPYNGLHEKELNQINQQDGSPCVAISAPGCFIKGSNLFSEKRAGNRVRFFTTGRDYFSDLASALDSAS
SSIFITGWQVNYDVLLDGRRSLWQCLRQALERSPALKVYV---MPWLSPSGSLGTYDFETMLAVF-------
----QLNAGLEGGARAFCTPAIQQSDMQGLGVAFSHHQKSVVIDNRIGYVGGIDLAYGRRDDNDFSLDASGR
RGNDAYNPGLPHLGWMAEDEHVSSMGLMMATLFDLSRPLASLTLHAPTLRLSPFPHIAASDEPLLSIPLAPS
RARALNGAAY--------------LSDLFRSPMLPSLQWLGRAYNSSKEGLDEGFERLDALRRQMVASSIRA
IANLIADNLDAL-PIEPELERRLRAWLEELRTAALNLPEALRIKSLLLINQWMSETELGQVLTLISGKGFED
IPQNLSGKAGELAGSLFWTLHRLMQARAGGHQQPYRYLDEAPQPLASPDNARLAADQPRMPWQDVHCRIEGP
SVYDLAR----------------------------NFIDRWNGQQAYL------------AKTPALQDTALV
RSALEAVMKWLNSLAAAAGLENYLDEKRNLR--LELDPPTPCWINAPEQLPQEPEVRRGGMTVQVLRSAAAR
MLEQEQAGRLGAGVNLPLQVGVSTEGVQSNCKDAMLLAISGAQQFIYIENQFFQSEFGKEGEVFKDLPLSGP
```

MASLRDVGSLRRDFVVRIRLEEALE---------------------------------QRDLWLLDWAEVEK
IAQE-PGTEARQFLKSMLAMWGVNAQGWLTHKLGEAQHGLLNEIG-------EALARRIERAIQREHPFHVY
LVLPVHPEGALN--VPNIMHQVHLTQQSLVFGE--QSLVKRIQRQMALKALEGKSDPAQAR-----------
------------------------------------------------------EIIERKDARGRPVYEQQD
WSRYLTLLNLRTWAVLGGRVVTEQIYVHSKLLIADDRVAILGSANINDRSLQGERDSELAVMVRDSEPLTVR
LDGKNDAIVGKAIHQLRVNLWKKHFGLSQGPGGFVKPASELSAYLSIPAAQEAWEAIQTLAKENTRAYERTF
NFIPQNISQTQLQLTPEPPKGFEDGFPASIWPTWAYRKPGELRA----------------------------
-------------------------------------GGQLMEPMPYQEIFWRSSNLTSVKTFPPPNGVSGF
ITALPTSWTRGERNDSGLNLSILAHQDSRSLPTQVAMNGDSSAQGKHRT-----------------------
------------------------------------------------------------------------
---------------------------
>6200_17
----------------------------------------------------------------------MN
KKGLIQFRFVELFTGNNIPNLY-------HVIKNEKGTTI---ASGMTNSNGLTVMISRDVGDTL-------
---------------YVYLKNIITGDLKEKVRHTVIYKKEIVRVISSKILLDNIILAKS-------------
------------------------------------------------------------------------
------------------------TNKPGNYRYLTHKVKKGENLSSISHRFNCKTSEIVHLNKLKNPDHIDV
GQIIKIPYKGES-SDSKKSPDNKQSKHENNKSSSTQKVNSHENNNKNTSDK--PQKRIESKD----------
------------------------------------------------------------------------
-----------------------------------ITDEYTKESGKPM------------KVATNATSPCIC
KQ-----------YNLAWGSKVS-CEFRKRV--IK--IAQ--------------------------------
------------------------------------------------------------------------
-------NLWPNDSENMASQLMAVM---------------------------------H---LESAGTFSPK
IGTF-----------ISKKLTDDAKGGYVGLIQFGKFASIDLKVK---------------------RSDLAK
MSAVQQLDYVEK--YYKLNSAHTKIKNLTGLYL----WVNYPKNV--------KENRLEDE-----------
------------------------------------------------------------------------
---DIVYAAPKDAEVTSKKFLESPYHQNPSFMKENEEYKRDGKKVIRQGFKNGST-----------------
-------------KVWEVEQEIKKHLTEGIKSQNLEKNYNCAYI-------------------NQTEIKNTQ
KINLEKFAEILRKRAKEKSQHQCAKYVRIALEAGGADT----------------------------------
-------------------------------------SGHPVAASDWGPTLKKIGYKEIPEEFNKPQLGDIY
IITKTDKHQYGHIAGYDGSQWISDFKQKGHRIYSDHVNYRYFRM----------------------------
------------------------------------------------------------------------
---------------------------

```
>ZW85-1_17
----------------------------------------------------------------------MN
KKGLIQFRFVELFTGNNIPNLY-------HVIKNEKGTTI---ASGMTNSNGLTVMISRDVGDTL-------
---------------YVVYLKNIITGDLKEKVRHTVIYKKEIVRVISSKILLDNIILAKS------------
------------------------------------------------------------------------
------------------------TNKPGNYRYLTHKVKKGENLSSISHRFNCKTSEIVHLNKLKNPDHIDV
GQIIKIPYKGES-SDSKKSPDNKQSKHENNKSSSTQKVNSHENNNKNTSDK--PQKRIESKD----------
------------------------------------------------------------------------
-----------------------------------ITDEYTKESGKPM------------KVATNATSPCIC
KQ-----------YNLAWGSKVS-CEFRKRV--IK--IAQ--------------------------------
------------------------------------------------------------------------
-------NLWPNDSENMASQLMAVM---------------------------------H---LESAGTFSPK
IGTF-----------ISKKLTDDAKGGYVGLIQFGKFASIDLKVK---------------------RSDLAK
MSAVQQLDYVEK--YYKLNSAHTKIKNLTGLYL----WVNYPKNV--------KENRLEDE-----------
------------------------------------------------------------------------
---DIVYAAPKDAEVTSKKFLESPYHQNPSFMKENEEYKRDGKKVIRQGFKNGST-----------------
-------------KVWEVEQEIKKHLTEGIKSQNLEKNYNCAYI-------------------NQTEIKNTQ
KINLEKFAEILRKRAKEKSQHQCAKYVRIALEAGGADT----------------------------------
-------------------------------------SGHPVAASDWGPTLKKIGYKEIPEEFNKPQLGDIY
IITKTDKHQYGHIAGYDGSQWISDFKQKGHRIYSDHVNYRYFRM----------------------------
------------------------------------------------------------------------
---------------------------
>A1_17
----------------------------------------------------------------------MN
KKGLIQFRFVELFTGNNIPNLY-------HVIKNEKGTTI---ASGMTNSNGLTVMISRDVGDTL-------
---------------YVVYLKNIITGDLKEKVRHTVIYKKEIVRVTSSKILLDNIILAKS------------
------------------------------------------------------------------------
------------------------TNKPGNYRYLTHKVKKGENLSLISHRFNCKISEIVHLNKLKNPDHIDV
GQIIKIPYKGGS-SDSKKSPDNKQSKHENNKSSSTQKVNSHENNNKNTSDK--PQKRIESKD----------
------------------------------------------------------------------------
-----------------------------------ITDEYTKESGKPM------------KVATNATSPCIC
KQ-----------YNLAWGSKVS-CEFRKRV--IK--IAQ--------------------------------
------------------------------------------------------------------------
```

-------NLWPNDSENMASQLMAVM---------------------------------H---LESAGTFSPK
IGTF-----------ISKKLTDDAKGGYVGLIQFGKFASIDLKVK---------------------RSDLAK
MSAVQQLDYVEK--YYKLNSAHTKIKNLTGLYL----WVNYPKNV--------KENRLEDE-----------
------------------------------------------------------------------------
---DIVYAAPKDAEVTSKKFLESPYHQNPSFMKENEEYKRDGKKVIRQGFKNGST-----------------
-------------KVWEVEQEIKKHLTEGIKSQNLEKNYNCAYI-------------------NHTEIKNTQ
KINLEKFAEILRKRAKEKSQHQCAKYVRIALEAGGADT----------------------------------
-------------------------------------SGHPVAASDWGPTLKKIGYKEIPEEFNKPQLGDIY
IITKTDKHQYGHIAGYDGSQWISDFKQKGHRIYSDHVNYRYFRM----------------------------
------------------------------------------------------------------------
---------------------------

>A388_17

----------------------------------------------------------------------MN
KKGLIQFRFVELFTGNNIPNLY-------HVIKNEKGTTI---ASGMTNSNGLTVMISRDVGDTL-------
---------------YVYLKNIITGDLKEKVRHTVIYKKEIVRVTSSKILLDNIILAKS-------------
------------------------------------------------------------------------
------------------------TNKPGNYRYLTHKVKKGENLSLISHRFNCKISEIVHLNKLKNPDHIDV
GQIIKIPYKGGS-SDSKKSPDNKQSKHENNKSSSTQKVNSHENNNKNTSDK--PQKRIESKD----------
------------------------------------------------------------------------
-----------------------------------ITDEYTKESGKPM------------KVATNATSPCIC
KQ-----------YNLAWGSKVS-CEFRKRV--IK--IAQ--------------------------------
------------------------------------------------------------------------
-------NLWPNDSENMASQLMAVM---------------------------------H---LESAGTFSPK
IGTF-----------ISKKLTDDAKGGYVGLIQFGKFASIDLKVK---------------------RSDLAK
MSAVQQLDYVEK--YYKLNSAHTKIKNLTGLYL----WVNYPKNV--------KENRLEDE-----------
------------------------------------------------------------------------
---DIVYAAPKDAEVTSKKFLESPYHQNPSFMKENEEYKRDGKKVIRQGFKNGST-----------------
-------------KVWEVEQEIKKHLTEGIKSQNLEKNYNCAYI-------------------NHTEIKNTQ
KINLEKFAEILRKRAKEKSQHQCAKYVRIALEAGGADT----------------------------------
-------------------------------------SGHPVAASDWGPTLKKIGYKEIPEEFNKPQLGDIY
IITKTDKHQYGHIAGYDGSQWISDFKQKGHRIYSDHVNYRYFRM----------------------------
------------------------------------------------------------------------
---------------------------

```
>A85_17
----------------------------------------------------------------------MN
KKGLIQFRFVELFTGNNIPNLY-------HVIKNEKGTTI---ASGMTNSNGLTVMISRDVGDTL-------
---------------YVVYLKNIITGDLKEKVRHTVIYKKEIVRVTSSKILLDNIILAKS------------
------------------------------------------------------------------------
------------------------TNKPGNYRYLTHKVKKGENLSLISHRFNCKISEIVHLNKLKNPDHIDV
GQIIKIPYKGGS-SDSKKSPDNKQSKHENNKSSSTQKVNSHENNNKNTSDK--PQKRIESKD----------
------------------------------------------------------------------------
-----------------------------------ITDEYTKESGKPM------------KVATNATSPCIC
KQ-----------YNLAWGSKVS-CEFRKRV--IK--IAQ--------------------------------
------------------------------------------------------------------------
-------NLWPNDSENMASQLMAVM---------------------------------H---LESAGTFSPK
IGTF-----------ISKKLTDDAKGGYVGLIQFGKFASIDLKVK---------------------RSDLAK
MSAVQQLDYVEK--YYKLNSAHTKIKNLTGLYL----WVNYPKNV--------KENRLEDE-----------
------------------------------------------------------------------------
---DIVYAAPKDAEVTSKKFLESPYHQNPSFMKENEEYKRDGKKVIRQGFKNGST-----------------
-------------KVWEVEQEIKKHLTEGIKSQNLEKNYNCAYI-------------------NHTEIKNTQ
KINLEKFAEILRKRAKEKSQHQCAKYVRIALEAGGADT----------------------------------
-------------------------------------SGHPVAASDWGPTLKKIGYKEIPEEFNKPQLGDIY
IITKTDKHQYGHIAGYDGSQWISDFKQKGHRIYSDHVNYRYFRM----------------------------
------------------------------------------------------------------------
---------------------------
>AB307-0294_17
----------------------------------------------------------------------MN
KKGLIQFRFVELFTGNNIPNLY-------HVIKNEKGTTI---ASGMTNSNGLTVMISRDVGDTL-------
---------------YVVYLKNIITGDLKEKVRHTVIYKKEIVRVTSSKILLDNIILAKS------------
------------------------------------------------------------------------
------------------------TNKPGNYRYLTHKVKKGENLSLISHRFNCKISEIVHLNKLKNPDHIDV
GQIIKIPYKGGS-SDSKKSPDNKQSKHENNKSSSTQKVNSHENNNKNTSDK--PQKRIESKD----------
------------------------------------------------------------------------
-----------------------------------ITDEYTKESGKPM------------KVATNATSPCIC
KQ-----------YNLAWGSKVS-CEFRKRV--IK--IAQ--------------------------------
------------------------------------------------------------------------
```

-------NLWPNDSENMASQLMAVM---------------------------------H---LESAGTFSPK
IGTF-----------ISKKLTDDAKGGYVGLIQFGKFASIDLKVK---------------------RSDLAK
MSAVQQLDYVEK--YYKLNSAHTKIKNLTGLYL----WVNYPKNV--------KENRLEDE-----------
------------------------------------------------------------------------
---DIVYAAPKDAEVTSKKFLESPYHQNPSFMKENEEYKRDGKKVIRQGFKNGST-----------------
-------------KVWEVEQEIKKHLTEGIKSQNLEKNYNCAYI-------------------NHTEIKNTQ
KINLEKFAEILRKRAKEKSQHQCAKYVRIALEAGGADT----------------------------------
-------------------------------------SGHPVAASDWGPTLKKIGYKEIPEEFNKPQLGDIY
IITKTDKHQYGHIAGYDGSQWISDFKQKGHRIYSDHVNYRYFRM----------------------------
------------------------------------------------------------------------
---------------------------
>AB5075-UW_17
----------------------------------------------------------------------MN
KKGLIQFRFVELFTGNNIPNLY-------HVIKNEKGTTI---ASGMTNSNGLTVMISRDVGDTL-------
---------------YVYLKNIITGDLKEKVRHTVIYKKEIVRVTSSKILLDNIILAKS-------------
------------------------------------------------------------------------
------------------------TNKPGNYRYLTHKVKKGENLSLISHRFNCKISEIVHLNKLKNPDHIDV
GQIIKIPYKGGS-SDSKKSPDNKQSKHENNKSSSTQKVNSHENNNKNTSDK--PQKRIESKD----------
------------------------------------------------------------------------
-----------------------------------ITDEYTKESGKPM------------KVATNATSPCIC
KQ-----------YNLAWGSKVS-CEFRKRV--IK--IAQ--------------------------------
------------------------------------------------------------------------
-------NLWPNDSENMASQLMAVM---------------------------------H---LESAGTFSPK
IGTF-----------ISKKLTDDAKGGYVGLIQFGKFASIDLKVK---------------------RSDLAK
MSAVQQLDYVEK--YYKLNSAHTKIKNLTGLYL----WVNYPKNV--------KENRLEDE-----------
------------------------------------------------------------------------
---DIVYAAPKDAEVTSKKFLESPYHQNPSFMKENEEYKRDGKKVIRQGFKNGST-----------------
-------------KVWEVEQEIKKHLTEGIKSQNLEKNYNCAYI-------------------NHTEIKNTQ
KINLEKFAEILRKRAKEKSQHQCAKYVRIALEAGGADT----------------------------------
-------------------------------------SGHPVAASDWGPTLKKIGYKEIPEEFNKPQLGDIY
IITKTDKHQYGHIAGYDGSQWISDFKQKGHRIYSDHVNYRYFRM----------------------------
------------------------------------------------------------------------
---------------------------

```
>AR_0083_17
----------------------------------------------------------------------MN
KKGLIQFRFVELFTGNNIPNLY-------HVIKNEKGTTI---ASGMTNSNGLTVMISRDVGDTL-------
---------------YVVYLKNIITGDLKEKVRHTVIYKKEIVRVTSSKILLDNIILAKS------------
------------------------------------------------------------------------
------------------------TNKPGNYRYLTHKVKKGENLSLISHRFNCKISEIVHLNKLKNPDHIDV
GQIIKIPYKGGS-SDSKKSPDNKQSKHENNKSSSTQKVNSHENNNKNTSDK--PQKRIESKD----------
------------------------------------------------------------------------
-----------------------------------ITDEYTKESGKPM------------KVATNATSPCIC
KQ-----------YNLAWGSKVS-CEFRKRV--IK--IAQ--------------------------------
------------------------------------------------------------------------
-------NLWPNDSENMASQLMAVM---------------------------------H---LESAGTFSPK
IGTF-----------ISKKLTDDAKGGYVGLIQFGKFASIDLKVK---------------------RSDLAK
MSAVQQLDYVEK--YYKLNSAHTKIKNLTGLYL----WVNYPKNV--------KENRLEDE-----------
------------------------------------------------------------------------
---DIVYAAPKDAEVTSKKFLESPYHQNPSFMKENEEYKRDGKKVIRQGFKNGST-----------------
-------------KVWEVEQEIKKHLTEGIKSQNLEKNYNCAYI-------------------NHTEIKNTQ
KINLEKFAEILRKRAKEKSQHQCAKYVRIALEAGGADT----------------------------------
-------------------------------------SGHPVAASDWGPTLKKIGYKEIPEEFNKPQLGDIY
IITKTDKHQYGHIAGYDGSQWISDFKQKGHRIYSDHVNYRYFRM----------------------------
------------------------------------------------------------------------
---------------------------
>AYE_17
----------------------------------------------------------------------MN
KKGLIQFRFVELFTGNNIPNLY-------HVIKNEKGTTI---ASGMTNSNGLTVMISRDVGDTL-------
---------------YVVYLKNIITGDLKEKVRHTVIYKKEIVRVTSSKILLDNIILAKS------------
------------------------------------------------------------------------
------------------------TNKPGNYRYLTHKVKKGENLSLISHRFNCKISEIVHLNKLKNPDHIDV
GQIIKIPYKGGS-SDSKKSPDNKQSKHENNKSSSTQKVNSHENNNKNTSDK--PQKRIESKD----------
------------------------------------------------------------------------
-----------------------------------ITDEYTKESGKPM------------KVATNATSPCIC
KQ-----------YNLAWGSKVS-CEFRKRV--IK--IAQ--------------------------------
------------------------------------------------------------------------
```

```
-------NLWPNDSENMASQLMAVM---------------------------------H---LESAGTFSPK
IGTF-----------ISKKLTDDAKGGYVGLIQFGKFASIDLKVK---------------------RSDLAK
MSAVQQLDYVEK--YYKLNSAHTKIKNLTGLYL----WVNYPKNV--------KENRLEDE-----------
------------------------------------------------------------------------
---DIVYAAPKDAEVTSKKFLESPYHQNPSFMKENEEYKRDGKKVIRQGFKNGST-----------------
-------------KVWEVEQEIKKHLTEGIKSQNLEKNYNCAYI-------------------NHTEIKNTQ
KINLEKFAEILRKRAKEKSQHQCAKYVRIALEAGGADT----------------------------------
-------------------------------------SGHPVAASDWGPTLKKIGYKEIPEEFNKPQLGDIY
IITKTDKHQYGHIAGYDGSQWISDFKQKGHRIYSDHVNYRYFRM----------------------------
------------------------------------------------------------------------
---------------------------
>D36_17
----------------------------------------------------------------------MN
KKGLIQFRFVELFTGNNIPNLY-------HVIKNEKGTTI---ASGMTNSNGLTVMISRDVGDTL-------
---------------YVYLKNIITGDLKEKVRHTVIYKKEIVRVTSSKILLDNIILAKS-------------
------------------------------------------------------------------------
------------------------TNKPGNYRYLTHKVKKGENLSLISHRFNCKISEIVHLNKLKNPDHIDV
GQIIKIPYKGGS-SDSKKSPDNKQSKHENNKSSSTQKVNSHENNNKNTSDK--PQKRIESKD----------
------------------------------------------------------------------------
-----------------------------------ITDEYTKESGKPM------------KVATNATSPCIC
KQ-----------YNLAWGSKVS-CEFRKRV--IK--IAQ--------------------------------
------------------------------------------------------------------------
-------NLWPNDSENMASQLMAVM---------------------------------H---LESAGTFSPK
IGTF-----------ISKKLTDDAKGGYVGLIQFGKFASIDLKVK---------------------RSDLAK
MSAVQQLDYVEK--YYKLNSAHTKIKNLTGLYL----WVNYPKNV--------KENRLEDE-----------
------------------------------------------------------------------------
---DIVYAAPKDAEVTSKKFLESPYHQNPSFMKENEEYKRDGKKVIRQGFKNGST-----------------
-------------KVWEVEQEIKKHLTEGIKSQNLEKNYNCAYI-------------------NHTEIKNTQ
KINLEKFAEILRKRAKEKSQHQCAKYVRIALEAGGADT----------------------------------
-------------------------------------SGHPVAASDWGPTLKKIGYKEIPEEFNKPQLGDIY
IITKTDKHQYGHIAGYDGSQWISDFKQKGHRIYSDHVNYRYFRM----------------------------
------------------------------------------------------------------------
---------------------------
```

```
>WCHAB005078_17
----------------------------------------------------------------------MN
KKGLIQFRFVELFTGNNIPNLY-------HVIKNEKGTTI---ASGMTNSNGLTVMISRDVGDTL-------
---------------YVVYLKNIITGDLKEKVRHTVIYKKEIVRVTSSKILLDNIILAKS------------
------------------------------------------------------------------------
------------------------TNKPGNYRYLTHKVKKGENLSLISHRFNCKISEIVHLNKLKNPDHIDV
GQIIKIPYKGGS-SDSKKSPDNKQSKHENNKSSSTQKVNSHENNNKNTSDK--PQKRIESKD----------
------------------------------------------------------------------------
-----------------------------------ITDEYTKESGKPM------------KVATNATSPCIC
KQ-----------YNLAWGSKVS-CEFRKRV--IK--IAQ--------------------------------
------------------------------------------------------------------------
-------NLWPNDSENMASQLMAVM---------------------------------H---LESAGTFSPK
IGTF-----------ISKKLTDDAKGGYVGLIQFGKFASIDLKVK---------------------RSDLAK
MSAVQQLDYVEK--YYKLNSAHTKIKNLTGLYL----WVNYPKNV--------KENRLEDE-----------
------------------------------------------------------------------------
---DIVYAAPKDAEVTSKKFLESPYHQNPSFMKENEEYKRDGKKVIRQGFKNGST-----------------
-------------KVWEVEQEIKKHLTEGIKSQNLEKNYNCAYI-------------------NHTEIKNTQ
KINLEKFAEILRKRAKEKSQHQCAKYVRIALEAGGADT----------------------------------
-------------------------------------SGHPVAASDWGPTLKKIGYKEIPEEFNKPQLGDIY
IITKTDKHQYGHIAGYDGSQWISDFKQKGHRIYSDHVNYRYFRM----------------------------
------------------------------------------------------------------------
---------------------------
>TseT
-----------------------------------------------------------------------M
SGIGALFRPLPRMGPRLPFELPAPPRPHLPMPAPRPLPRP---APGALPRPVPRPMPVPQTLPRT-------
------------------------------------------------------------------------
------------------------------------------------------------------------
------------------------------------------------------------------------
-----------------ETGTLSRDRTREAERDCSKSQGRDK------------------------------
------------------------------------------------------------------------
-----------------------------------DCVECPPSRGEMA----------IANNGKGHSMSDLS
AR-----------YQQWVTNFPFPHEWFWSG--TW--WDG--------------------------------
------------------------------------------------------------------------
```

--FDEPRCTLLEAKANYAFLFVPLL---------------------------------G---VPRPWARAKV
KSDL-------------------LQKAEVHSDKARPTPPVFVEWH--------------------------F
LQRIVYEYCAAE--YLRMGLANLKAFWNPMPGT--DEHDDYQETR---------------------------
------------------------------------------------------------------------
------------------------------------------------------------------------
-------------AKEQEEMKRFCEENPGYCA----------------------------------------
------------------------------------------------------------------------
------------------------------------------------------------------------
------------------------------------------------------------------------
------------------------------------------------------------------------
---------------------------
>AB031_27
------------------------------------------------------------------------
------------------------------NNNPLVGIDL---SNKSRSEIENILSNSSEVSYKG-------
------------------------------------------------------------------------
------------------------------------------------------------------------
------------------------------------------------------------------------
-------------------GSPDKK-----------------------------------------------
------------------------------------------------------------------------
-----------------------------------FMQWKWNDNGQTA------------------------
-------------VRLDPPDKVT-------------------------------------------------
------------------------------------------------------------------------
--------------------------------------------------------------LYDHAHLYDK
NGNP--------------------------LDKDGNIVPRESPDA---------------------------
------------------------------------------------------------------------
------------------------------------------------------------------------
------------------------------------------------------------------------
-------------HIPIGDCK---------------------------------------------------
------------------------------------------------------------------------
------------------------------------------------------------------------
------------------------------------------------------------------------
------------------------------------------------------------------------
---------------------------

```
>A1296_8
----------------------------------------MYIVKKGDTLTSIANKNGCTVEQIIKLNSIKN
KNLIFVGQKLKLKN-----------------------------------TPTPIQNLGGKVGIAI-------
---------------VNKKGEPISNFKLKIQVGNNIYSNQLTNARGVVTLPRQKSGENIIIQGWSKTNNSYK
LISNRFITENRNLGIGISLDTIKFETKTDNHK------------KPINNNVNNNGQST--------------
------------------------TTNGSNSQNNPIARTSVEGCSNCTKITENELKQIFTQANSNDIKKIID
VYTNFSEKFGMN-NCLSKAHFFAQVLEEVGKKIEVKDGESLNYHSDILFLS--YTKIKRKEYIIEKGKRVEK
MTHGGPFSAFRGNRQ---------------------------------------------------------
-----------------------------------LCDKYGRNDNHPADQVMIANIAYANRNGNGDIQSGDG
WK-----------YRGRGIIQ---ITGKDKYDKIN-------------------------------------
------------------------------------------------------------------------
-------KAIKDNYPNVGISIDANN-------------------------------------INNIYEGTLA
SMAY---------------------WKSFGLSKLATQKQVDIRTQ---------------------LEVVDS
LIDIINRDTASR--SDRKKNFEYITAKVFKLNE----CKNSIVEANLLSKSTPTPTPTPTPTPTPTPTPTP-
------------------------------------------------------------------------
---------------TPTPTLSNVLKEIKQLVDRNIPYSQTGARGAGSNKNATSV-----------------
-------------ITANDLKGLDCSETVAIYLLKLGVTDKFYSI-------------------HTGVMLTEN
DFRKAIRSNKIEYVVGSKDLNFIPQIGDIFVWRNGGGHCGIVYDVDRQNDT---------------------
-------------------------------------VTILEAIGDVGSADENFNINNGGEKKVGCTRTAVY
RRSGKALAQHRGWVGYFRPIISGKKI----------------------------------------------
------------------------------------------------------------------------
---------------------------
>1656-2_8
----------------------------------------MYIVKKGDTLTSIANKNGCTVEQIIKLNSIKN
KNLIFVGQKLKLKNTPTPTPTP-----TPTPTPTPTPTPTPTPTPTPTPTPTPIQNLGGKVGIAI-------
---------------VNKKGEPISNFKLKIQVGNNIYSNQLTNARGVVTLPRQKSGENIIIQGWSKTNNSYK
LISNRFITENRNLGIGISLDTIKFETKTDNHK------------KPINNNVNNNGQST--------------
------------------------TTNGSNSQNNPIARTSVEGCSNCTKITENELKQIFTQANSNDIKKIID
VYTNFSEKFGMN-NCLSKAHFFAQVLEEVGKKIEVKDGESLNYHSDILFLS--YTKIKRKEYIIEKGKRVEK
MTHGGPFSAFRGNRQ---------------------------------------------------------
-----------------------------------LCDKYGRNDNHPADQVMIANIAYANRNGNGDIQSGDG
WK-----------YRGRGIIQ---ITGKDKYDKIN-------------------------------------
------------------------------------------------------------------------
```

-------KAIKDNYPNVGISIDANN-------------------------------------INNIYEGTLA
SMAY---------------------WKSFGLSKLATQKQVDIRTQ---------------------LEVVDS
LIDIINRDTASR--SDRKKNFEYITAKVFKLNE----CKNSIVEANLLSKSTPTPTPTPTP-----------
------------------------------------------------------------------------
---------------TPTPTLSNVLKEIKQLVDRNIPYSQTGARGAGSNKNATSV-----------------
-------------ITANDLKGLDCSETVAIYLLKLGVTDKFYSI-------------------HTGVMLTEN
DFRKAIRSNKIEYVVGSKDLNFIPQIGDIFVWRNGGGHCGIVYDVDRQNDT---------------------
-------------------------------------VTILEAIGDVGSADENFNINNGGEKKVGCTRTAVY
RRSGKALAQHRGWVGYFRPIISGKKI----------------------------------------------
------------------------------------------------------------------------
---------------------------
>ACICU_8
----------------------------------------MYIVKKGDTLTSIANKNGCTVEQIIKLNSIKN
KNLIFVGQKLKLKNTPTPTPTP-------------------------TPTPTPIQNLGGKVGIAI-------
---------------VNKKGEPISNFKLKIQVGNNIYSNQLTNARGVVTLPRQKSGENIIIQGWSKTNNSYK
LISNRFITENRNLGIGISLDTIKFETKTDNHK------------KPINNNVNNNGQST--------------
------------------------TTNGSNSQNNPIARTSVEGCSNCTKITENELKQIFTQANSNDIKKIID
VYTNFSEKFGMN-NCLSKAHFFAQVLEEVGKKIEVKDGESLNYHSDILFLS--YTKIKRKEYIIEKGKRVEK
MTHGGPFSAFRGNRQ---------------------------------------------------------
-----------------------------------LCDKYGRNDNHPADQVMIANIAYANRNGNGDIQSGDG
WK-----------YRGRGIIQ---ITGKDKYDKIN-------------------------------------
------------------------------------------------------------------------
-------KAIKDNYPNVGISIDANN-------------------------------------INNIYEGTLA
SMAY---------------------WKSFGLSKLATQKQVDIRTQ---------------------LEVVDS
LIDIINRDTASR--SDRKKNFEYITAKVFKLNE----CKNSIVEANLLSKSTPTPTPTP-------------
------------------------------------------------------------------------
---------------TPTPTLSNVLKEIKQLVDRNIPYSQTGARGAGSNKNATSV-----------------
-------------ITANDLKGLDCSETVAIYLLKLGVTDKFYSI-------------------HTGVMLTEN
DFRKAIRSNKIEYVVGSKDLNFIPQIGDIFVWRNGGGHCGIVYDVDRQNDT---------------------
-------------------------------------VTILEAIGDVGSADENFNINNGGEKKVGCTRTAVY
RRSGKALAQHRGWVGYFRPIISGKKI----------------------------------------------
------------------------------------------------------------------------
---------------------------

>AR_0056_8
----------------------------------------MYIVKKGDTLTSIANKNGCTVEQIIKLNSIKN
KNLIFVGQKLKLKNTPTPTPTP---------------------------TPTPIQNLGGKVGIAI-------
---------------VNKKGEPISNFKLKIQVGNNIYSNQLTNARGVVTLPRQKSGENIIIQGWSKTNNSYK
LISNRFITENRNLGIGISLDTIKFETKTDNHK------------KPINNNVNNNGQST--------------
------------------------TTNGSNSQNNPIARTSVEGCSNCTKITENELKQIFTQANSNDIKKIID
VYTNFSEKFGMN-NCLSKAHFFAQVLEEVGKKIEVKDGESLNYHSDILFLS--YTKIKRKEYIIEKGKRVEK
MTHGGPFSAFRGNRQ---------------------------------------------------------
-----------------------------------LCDKYGRNDNHPADQVMIANIAYANRNGNGDIQSGDG
WK-----------YRGRGIIQ---ITGKDKYDKIN-------------------------------------
------------------------------------------------------------------------
-------KAIKDNYPNVGISIDANN-------------------------------------INNIYEGTLA
SMAY---------------------WKSFGLSKLATQKQVDIRTQ---------------------LEVVDS
LIDIINRDTASR--SDRKKNFEYITAKVFKLNE----CKNSIVEANLLSKSTPTPTPTPTPTPTP-------
------------------------------------------------------------------------
---------------TPTPTLSNVLKEIKQLVDRNIPYSQTGARGAGSNKNATSV-----------------
-------------ITANDLKGLDCSETVAIYLLKLGVTDKFYSI-------------------HTGVMLTEN
DFRKAIRSNKIEYVVGSKDLNFIPQIGDIFVWRNGGGHCGIVYDVDRQNDT---------------------
-------------------------------------VTILEAIGDVGSADENFNINNGGEKKVGCTRTAVY
RRSGKALAQHRGWVGYFRPIISGKKI----------------------------------------------
------------------------------------------------------------------------
---------------------------
>D1279779_8
----------------------------------------MYIVKKGDTLTSIANKNGCTVEQIIKLNSIKN
KNLIFVGQKLKLKNTPTPTPTP---------------------TPTPTPTPTPIQNLGGKVGIAI-------
---------------VNKKGEPISNFKLKIQVGNNIYSNQLTNARGVVTLPRQKSGENIIIQGWSKTNNSYK
LISNRFITENRNLGIGISLDTIKFETKTDNHK------------KPINNNVNNNGQST--------------
------------------------TTNGSNSQNNPIARTSVEGCSNCTKITENELKQIFTQANSNDIKKIID
VYTNFSEKFGMN-NCLSKAHFFAQVLEEVGKKIEVKDGESLNYHSDILFLS--YTKIKRKEYIIEKGKRVEK
MTHGGPFSAFRGNRQ---------------------------------------------------------
-----------------------------------LCDKYGRNDNHPADQVMIANIAYANRNGNGDIQSGDG
WK-----------YRGRGIIQ---ITGKDKYDKIN-------------------------------------
------------------------------------------------------------------------

-------KAIKDNYPNVGISIDANN-------------------------------------INNIYEGTLA
SMAY---------------------WKSFGLSKLATQKQVDIRTQ---------------------LEVVDS
LIDIINRDTASR--SDRKKNFEYITAKVFKLNE----CKNSIVEANLLSKSTP-------------------
------------------------------------------------------------------------
---------------TPTPTLSNVLKEIKQLVDRNIPYSQTGARGAGSNKNATSV-----------------
-------------ITANDLKGLDCSETVAIYLLKLGVTDKFYSI-------------------HTGVMLTEN
DFRKAIRSNKIEYVVGSKDLNFIPQIGDIFVWRNGGGHCGIVYDVDRQNDT---------------------
-------------------------------------VTILEAIGDVGSADENFNINNGGEKKVGCTRTAVY
RRSGKALAQHRGWVGYFRPIISGKKI----------------------------------------------
------------------------------------------------------------------------
---------------------------
>DU202_8
----------------------------------------MYIVKKGDTLTSIANKNGCTVEQIIKLNSIKN
KNLIFVGQKLKLKNTPTPTPTP-----TPTPTPTPTPTPTPTPTPTPTPTPTPIQNLGGKVGIAI-------
---------------VNKKGEPISNFKLKIQVGNNIYSNQLTNARGVVTLPRQKSGENIIIQGWSKTNNSYK
LISNRFITENRNLGIGISLDTIKFETKTDNHK------------KPINNNVNNNGQST--------------
------------------------TTNGSNSQNNPIARTSVEGCSNCTKITENELKQIFTQANSNDIKKIID
VYTNFSEKFGMN-NCLSKAHFFAQVLEEVGKKIEVKDGESLNYHSDILFLS--YTKIKRKEYIIEKGKRVEK
MTHGGPFSAFRGNRQ---------------------------------------------------------
-----------------------------------LCDKYGRNDNHPADQVMIANIAYANRNGNGDIQSGDG
WK-----------YRGRGIIQ---ITGKDKYDKIN-------------------------------------
------------------------------------------------------------------------
-------KAIKDNYPNVGISIDANN-------------------------------------INNIYEGTLA
SMAY---------------------WKSFGLSKLATQKQVDIRTQ---------------------LEVVDS
LIDIINRDTASR--SDRKKNFEYITAKVFKLNE----CKNSIVEANLLSKSTPTPTPTPTPTPTP-------
------------------------------------------------------------------------
---------------TPTPTLSNVLKEIKQLVDRNIPYSQTGARGAGSNKNATSV-----------------
-------------ITANDLKGLDCSETVAIYLLKLGVTDKFYSI-------------------HTGVMLTEN
DFRKAIRSNKIEYVVGSKDLNFIPQIGDIFVWRNGGGHCGIVYDVDRQNDT---------------------
-------------------------------------VTILEAIGDVGSADENFNINNGGEKKVGCTRTAVY
RRSGKALAQHRGWVGYFRPIISGKKI----------------------------------------------
------------------------------------------------------------------------
---------------------------

```
>KAB03_8
----------------------------------------MYIVKKGDTLTSIANKNGCTVEQIIKLNSIKN
KNLIFVGQKLKLKNTPTPTPTP--------------TPTPTPTPTPTP-TPTPIQNLGGKVGIAI-------
---------------VNKKGEPISNFKLKIQVGNNIYSNQLTNARGVVTLPRQKSGENIIIQGWSKTNNSYK
LISNRFITENRNLGIGISLDTIKFETKTDNHK------------KPINNNVNNNGQST--------------
------------------------TTNGSNSQNNPIARTSVEGCSNCTKITENELKQIFTQANSNDIKKIID
VYTNFSEKFGMN-NCLSKAHFFAQVLEEVGKKIEVKDGESLNYHSDILFLS--YTKIKRKEYIIEKGKRVEK
MTHGGPFSAFRGNRQ---------------------------------------------------------
-----------------------------------LCDKYGRNDNHPADQVMIANIAYANRNGNGDIQSGDG
WK-----------YRGRGIIQ---ITGKDKYDKIN-------------------------------------
------------------------------------------------------------------------
-------KAIKDNYPNVGISIDANN-------------------------------------INNIYEGTLA
SMAY---------------------WKSFGLSKLATQKQVDIRTQ---------------------LEVVDS
LIDIINRDTASR--SDRKKNFEYITAKVFKLNE----CKNSIVEANLLSKSTPTPTPTPTPTPTP-------
------------------------------------------------------------------------
---------------TPTPTLSNVLKEIKQLVDRNIPYSQTGARGAGSNKNATSV-----------------
-------------ITANDLKGLDCSETVAIYLLKLGVTDKFYSI-------------------HTGVMLTEN
DFRKAIRSNKIEYVVGSKDLNFIPQIGDIFVWRNGGGHCGIVYDVDRQNDT---------------------
-------------------------------------VTILEAIGDVGSADENFNINNGGEKKVGCTRTAVY
RRSGKALAQHRGWVGYFRPIISGKKI----------------------------------------------
------------------------------------------------------------------------
---------------------------
>NCGM237_8
----------------------------------------MYIVKKGDTLTSIANKNGCTVEQIIKLNSIKN
KNLIFVGQKLKLKNTPTPTPTP---------------TPTPTPTPTPTPTPTPIQNLGGKVGIAI-------
---------------VNKKGEPISNFKLKIQVGNNIYSNQLTNARGVVTLPRQKSGENIIIQGWSKTNNSYK
LISNRFITENRNLGIGISLDTIKFETKTDNHK------------KPINNNVNNNGQST--------------
------------------------TTNGSNSQNNPIARTSVEGCSNCTKITENELKQIFTQANSNDIKKIID
VYTNFSEKFGMN-NCLSKAHFFAQVLEEVGKKIEVKDGESLNYHSDILFLS--YTKIKRKEYIIEKGKRVEK
MTHGGPFSAFRGNRQ---------------------------------------------------------
-----------------------------------LCDKYGRNDNHPADQVMIANIAYANRNGNGDIQSGDG
WK-----------YRGRGIIQ---ITGKDKYDKIN-------------------------------------
------------------------------------------------------------------------
```

```
-------KAIKDNYPNVGISIDANN-------------------------------------INNIYEGTLA
SMAY---------------------WKSFGLSKLATQKQVDIRTQ---------------------LEVVDS
LIDIINRDTASR--SDRKKNFEYITAKVFKLNE----CKNSIVEANLLSKSTPTPTP---------------
------------------------------------------------------------------------
---------------TPTPTLSNVLKEIKQLVDRNIPYSQTGARGAGSNKNATSV-----------------
-------------ITANDLKGLDCSETVAIYLLKLGVTDKFYSI-------------------HTGVMLTEN
DFRKAIRSNKIEYVVGSKDLNFIPQIGDIFVWRNGGGHCGIVYDVDRQNDT---------------------
-------------------------------------VTILEAIGDVGSADENFNINNGGEKKVGCTRTAVY
RRSGKALAQHRGWVGYFRPIISGKKI----------------------------------------------
------------------------------------------------------------------------
---------------------------
>R2090_8
----------------------------------------MYIVKKGDTLTSIANKNGCTVEQIIKLNSIKN
KNLIFVGQKLKLKNTPTPTPTP-------------------TPTPTPTPTPTPIQNLGGKVGIAI-------
---------------VNKKGEPISNFKLKIQVGNNIYSNQLTNARGVVTLPRQKSGENIIIQGWSKTNNSYK
LISNRFITENRNLGIGISLDTIKFETKTDNHK------------KPINNNVNNNGQST--------------
------------------------TTNGSNSQNNPIARTSVEGCSNCTKITENELKQIFTQANSNDIKKIID
VYTNFSEKFGMN-NCLSKAHFFAQVLEEVGKKIEVKDGESLNYHSDILFLS--YTKIKRKEYIIEKGKRVEK
MTHGGPFSAFRGNRQ---------------------------------------------------------
-----------------------------------LCDKYGRNDNHPADQVMIANIAYANRNGNGDIQSGDG
WK-----------YRGRGIIQ---ITGKDKYDKIN-------------------------------------
------------------------------------------------------------------------
-------KAIKDNYPNVGISIDANN-------------------------------------INNIYEGTLA
SMAY---------------------WKSFGLSKLATQKQVDIRTQ---------------------LEVVDS
LIDIINRDTASR--SDRKKNFEYITAKVFKLNE----CKNSIVEANLLSKSTPTPTPTPTP-----------
------------------------------------------------------------------------
---------------TPTPTLSNVLKEIKQLVDRNIPYSQTGARGAGSNKNATSV-----------------
-------------ITANDLKGLDCSETVAIYLLKLGVTDKFYSI-------------------HTGVMLTEN
DFRKAIRSNKIEYVVGSKDLNFIPQIGDIFVWRNGGGHCGIVYDVDRQNDT---------------------
-------------------------------------VTILEAIGDVGSADENFNINNGGEKKVGCTRTAVY
RRSGKALAQHRGWVGYFRPIISGKKI----------------------------------------------
------------------------------------------------------------------------
---------------------------
```

```
>AbPK1_8
----------------------------------------MYIVKKGDTLTSIANKNGCTVEQIIKLNSIKN
KNLIFVGQKLKLKNTPTPTPTP-------------------TPTPTPTPTPTPIQNLGGKVGIAI-------
---------------VNKKGEPISNFKLKIQVGNNIYSNQLTNARGVVTLPRQKSGENIIIQGWSKTNNSYK
LISNRFITENRNLGIGISLDTIKFEAKTDNHK------------KPINNNVNNNGQST--------------
------------------------TTNGSNSQNNPIARTSVEGCSNCTKITENELKQIFTQANSNDIKKIID
VYTNFSEKFGMN-NCLSKAHFFAQVLEEVGKKIEVKDGESLNYHSDILFLS--YTKIKRKEYIIEKGKRVEK
MTHGGPFSAFRGNRQ---------------------------------------------------------
-----------------------------------LCDKYGRNDNHPADQVMIANIAYANRNGNGDIQSGDG
WK-----------YRGRGIIQ---ITGKDKYDKIN-------------------------------------
------------------------------------------------------------------------
-------KAIKDNYPNVGISIDANN-------------------------------------INNIYEGTLA
SMAY---------------------WKSFGLSKLATQKQVDIRTQ---------------------LEVVDS
LIDIINRDTASR--SDRKKNFEYITAKVFKLNE----CKNSIVEANLLSKSTPTP-----------------
------------------------------------------------------------------------
---------------TPTPTLSNVLKEIKQLVDRNIPYSQTGARGAGSNKNATSV-----------------
-------------ITANDLKGLDCSETVAIYLLKLGVTDKFYSI-------------------HTGVMLTEN
DFRKAIRSNKIEYVVGSKDLNFIPQIGDIFVWRNGGGHCGIVYDVDRQNDT---------------------
-------------------------------------VTILEAIGDVGSADENFNINNGGEKKVGCTRTAVY
RRSGKALAQHRGWVGYFRPIISGKKI----------------------------------------------
------------------------------------------------------------------------
---------------------------
>Tde2
------------------------------------------------------------------MSIPRD
NYIGEPQYPDTWTTKTPREGLRDIDEARIVSLAPDVCLTPV--GSSVVPIPYPVVDFCGHDKNYT-------
---------------PSVRFTGKKAMVMRSCTTHVHGDAPGVRKGVKSGTVESVCEPIGHADQVRAEGSHVI
RHLDRFHMNSKNTE----------------------------------------------------------
------------------------GEAIFVRSTQTYNSPKDDDPVRGSLRAIEVADASEEQNRSSDAAPSGG
AALGFLVTPGFA-PLVQGAGGAAAGGASTAAGAATSGAGGAAAAAGGV------------------------
------------------------------------------------------------------------
-----------------------------------SLGTILAGIGIFA------------AGVLIPTNKMNF
SD---------TVPQDDFEQKLLLDAQRRMN--ELPFWDS--------------------------------
------------------------------------------------------------------------
```

-----GADIRTETFAKIQEHRKKNK---------------------------------PTQDPEPTPVPVPP
PGSN---------VRIDEDENRRCRLLIICFMPTKSTIDIDEFKR---------------------------
-QMELQEQGLNN--MSPQQMLANQAKYLANPAG--MRALSEPLQA---------------------------
------------------------------------------------------------------------
-----------------------KARQEYRNDPRIQKKYVDQYGPIQGPIKLGEY-----------------
-------------LDSAAALHNPDMIAGGKYNSVVDQTLPIENR----------------------------
------------------------------------------------------------------------
-------------------------------------IGGLSENSSMGSQWINPNRNGHTRASRLTEHAKRQ
AANNCPSVQVDLRLCPSNPSRPGEPLTGT-------------------------------------------
------------------------------------------------------------------------
---------------------------
>Tle4
----------------------------------------MSSEPLEPNQDVIIPRSRDSLGRPVYKAQLTR
TDNQSEKVALIRQTAPLPVIFIPGIMGTNLRNKADKSEVW---RPPNGLWPMDDLFASIGALWTW-------
------------------------AWRGPKARQELLKAEQVEVDDQGTIDVGQSGLSEEAARLRGWGKVMRS
AYNPVMGLMERRLDNIVSRRELQAWWNDEA------------------------------------------
------------------------LSPPGDQGEEQGKVGPIDEEELLRASRYQFDVWCAGYNWLQSNRQSAL
DVRDYIENTVLP-FYQKECGLDPEQMRRMKVILVTHSMGGLVARALTQLHG--YERVLGVVH----GVQPAT
GSSTIYHHMRCGYEGIAQV-----------------------------------------------------
-----------------------------------VLGRNAGEVTAIV------------ANSAGALELAPS
AE-----------YREGRPWLFLCDAQGQVL--KD--IDG--------------------------------
------------------------------------------------------------------------
---------KPRAYPQNQDPYEEIY-------------------------------------KNTTWYGLVP
EQNS---------------QYLDMSDKKEGLRVGPRDNFEDLIDS--------------------IANFHGE
LSAAGYHSETYA--HYGADDSRHSWRDLIWKGD--PTPLETPGAT---------------------------
------------------------------------------------------------------------
----LNDDENGTYNSWFRRGLPTIVQGPLETGNPLDASGSGGDETVPTDSGQA-------------------
-------------PALAGVKASFRHGSKGKGQANTKRGYEHQ------------------------------
------------------------------------------------------------------------
-------------------------------------ESYNDARAQWAALYGVIKITQLADWHPNDKGGT--
------------------------------------------------------------------------
------------------------------------------------------------------------
---------------------------

>Rhs2
------------------------------------------------------------------------
--------------------------------SNCSTLDR---IIGDANKVASRGGAITAKQAQI-------
------------------------------------------------------------------------
------------------------------------------------------------------------
------------------------------------------------------------------------
------------------------------------------------------------------------
------------------------------------------------------------------------
-----------------------------------LRDNLPVVQRRSV------------------------
-------------FQNQMARKEFVRDQHYLM--SQ--WEA--------------------------------
------------------------------------------------------------------------
----------------------------------------------------------N---TGRTWPTGAT
PHHI----------------------IPLESGGANKWWNLMPTHG---------------------------
TLPNHSLPGVPG--PHAAGG----------------------------------------------------
------------------------------------------------------------------------
------------------------------------------------------------------------
-------------VLRTTVQQSRKALPPGTITDLRL------------------------------------
------------------------------------------------------------------------
------------------------------------------------------------------------
------------------------------------------------------------------------
------------------------------------------------------------------------
---------------------------
>Tle2
-----------------------------------------------------------------------M
DSFNYCVQCNPEENWLELEFRS-------ENDEPIDGLLV---TITNQSAPSNTYTQTTSSGKVL-------
---------------FGKIAAGEWRASVSQASLLTEVEKYASRKEGQESPVKKRAAAELDAADKDTKQYRFT
TIGDFWDEAPKDEFLQKQHKGIDVNASAEKAG----------------------------------------
------------------------FRLSHNQTYVFEIKALRSYMPVIIDTDEFNLVNSYTFALLSKLAYATN
DFNRDDGKTIDNQGAISTVISQLKRKERPTYSGDLQAKWLLEEIPYSKALSAQYYAEDDVGSEGYIIFNDEL
AIIGVRGTEPYFQSKKPPVDNTKFKIIKAASGMAAV------------------------------------
-----------------------------------IADKIESATDSPG-----------MKDLIITDLDAAQ
IAPEEFGGTYVHRGFYQYTMALLSLMEKDLG--LH-------------------------------------
---------------------------------------------------------------------KIK

KFYCCGHSLGGAGALLISALIKDSY---------------------------------HPPVLRLYTYGMPR
VGTR-------------------SFVERYQNILHYRHVNNHDLVP---------------------QIPTVW
MNTDVSEGFHVL--DVFKSRVDLMRKMLTDDDD-----DNYQHHGHLSQLLTYNSNNQVLL-----------
------------------------------------------------------------------------
-----TPKQTQVTMLDLANLATNDSVAMVDGLSDASIVEHGMEQYIPNLFEQLT------------------
-------------ALSDESLMVHYQRAISALEQEIATLQQSYLTVKQAWIES--------------------
------------------------------------------------------------------------
-------------------------------------IGNGTPTMNIGRLMSEMHSINKLIENRNKIRGELR
QIVSDPQRMPATKFLISQQTLPDEIKVQIR------------------------------------------
------------------------------------------------------------------------
---------------------------
>EvpP
------------------------------------------------------------------------
----------------------------MSILNSELDWSH---VGSISTGPGTVVSDAFNISYGL-------
------------------------------------------------------------------------
------------------------------------------------------------------------
------------------------------------------------------------------------
--------PTKE-LLPAGTALYKFNGFSSLARPPITGDT---------------------------------
------------------------------------------------------------------------
-----------------------------------PLSPWWSPVQPFR------------HDGGLQQRMLVA
KL-----------------NGVSMREWGRLT--SV-------------------------------------
------------------------------------------------------------------------
--------------------------------------------------------------IKENWSSLDH
LLEI------------------------VLKVPVYAWFGGFKGMS-----------------------RIDN
GMPSKRNITLEQ--KGRGSNLPGGATQFYIPNL---------------------------------------
------------------------------------------------------------------------
------------------------------------------------------------------------
-------------TVGHISSHNFSALK---------------------------------------------
------------------------------------------------------------------------
------------------------------------------------------------------------
------------------------------------------------------------------------
------------------------------------------------------------------------
---------------------------

```
>15A34_6
------------------------------------------------------------------------
--------------------------------APCSLVRY---KPDKVTPQAGSRQDAIDRAWSL-------
------------------------------------------------------------------------
------------------------------------------------------------------------
------------------------------------------------------------------------
----------EK-QLIQTTGTG--------------------------------------------------
------------------------------------------------------------------------
-----------------------------------TRDWSKAELDTIL------------RTPSGSGKGHLS
S------------VMSNLGYTGHHINSVKNN--GA-------------------------------------
------------------------------------------------------------------------
--------------------------------------------------------------LGESWKGDPR
NIVF--------------------------------------------------------------LENPKH
PNSSPMPNAYNE--HFHSKQGHRGSTTNVSRGR---------------------------------------
------------------------------------------------------------------------
------------------------------------------------------------------------
-------------LIDRQAMINQFNKGCSI------------------------------------------
------------------------------------------------------------------------
------------------------------------------------------------------------
------------------------------------------------------------------------
------------------------------------------------------------------------
---------------------------
>15A5_6
------------------------------------------------------------------------
--------------------------------APCSLVRY---KPDKVTPQAGSRQDAIDRAWSL-------
------------------------------------------------------------------------
------------------------------------------------------------------------
------------------------------------------------------------------------
----------EK-QLIQTTGTG--------------------------------------------------
------------------------------------------------------------------------
-----------------------------------TRDWSKAELDTIL------------RTPSGSGKGHLS
S------------VMSNLGYTGHHINSVKNN--GA-------------------------------------
------------------------------------------------------------------------
```

--------------------------------------------------------------LGESWKGDPR
NIVF--------------------------------------------------------------LENPKH
PNSSPMPNAYNE--HFHSKQGHRGSTTNVSRGR---------------------------------------
------------------------------------------------------------------------
------------------------------------------------------------------------
-------------LIDRQAMINQFNKGCSI------------------------------------------
------------------------------------------------------------------------
------------------------------------------------------------------------
------------------------------------------------------------------------
------------------------------------------------------------------------
---------------------------
>1656-2_6
------------------------------------------------------------------------
--------------------------------APCSLVRY---KPDKVTPQAGSRQDAIDRAWSL-------
------------------------------------------------------------------------
------------------------------------------------------------------------
------------------------------------------------------------------------
----------EK-QLIQTTGTG--------------------------------------------------
------------------------------------------------------------------------
-----------------------------------TRDWSKAELDTIL------------RTPSGSGKGHLS
S------------VMSNLGYTGHHINSVKNN--GA-------------------------------------
------------------------------------------------------------------------
--------------------------------------------------------------LGESWKGDPR
NIVF--------------------------------------------------------------LENPKH
PNSSPMPNAYNE--HFHSKQGHRGSTTNVSRGR---------------------------------------
------------------------------------------------------------------------
------------------------------------------------------------------------
-------------LIDRQAMINQFNKGCSI------------------------------------------
------------------------------------------------------------------------
------------------------------------------------------------------------
------------------------------------------------------------------------
------------------------------------------------------------------------
---------------------------

```
>3027STDY5784958_6
------------------------------------------------------------------------
--------------------------------APCSLVRY---KPDKVTPQAGSRQDAIDRAWSL-------
------------------------------------------------------------------------
------------------------------------------------------------------------
------------------------------------------------------------------------
----------EK-QLIQTTGTG--------------------------------------------------
------------------------------------------------------------------------
-----------------------------------TRDWSKAELDTIL------------RTPSGSGKGHLS
S------------VMSNLGYTGHHINSVKNN--GA-------------------------------------
------------------------------------------------------------------------
--------------------------------------------------------------LGESWKGDPR
NIVF--------------------------------------------------------------LENPKH
PNSSPMPNAYNE--HFHSKQGHRGSTTNVSRGR---------------------------------------
------------------------------------------------------------------------
------------------------------------------------------------------------
-------------LIDRQAMINQFNKGCSI------------------------------------------
------------------------------------------------------------------------
------------------------------------------------------------------------
------------------------------------------------------------------------
------------------------------------------------------------------------
---------------------------
>AB07_6
------------------------------------------------------------------------
--------------------------------APCSLVRY---KPDKVTPQAGSRQDAIDRAWSL-------
------------------------------------------------------------------------
------------------------------------------------------------------------
------------------------------------------------------------------------
----------EK-QLIQTTGTG--------------------------------------------------
------------------------------------------------------------------------
-----------------------------------TRDWSKAELDTIL------------RTPSGSGKGHLS
S------------VMSNLGYTGHHINSVKNN--GA-------------------------------------
------------------------------------------------------------------------
```

------------------------------------------------------------LGESWKGDPR
NIVF------------------------------------------------------------LENPKH
PNSSPMPNAYNE--HFHSKQGHRGSTTNVSRGR-------------------------------------
----------------------------------------------------------------------
----------------------------------------------------------------------
-------------LIDRQAMINQFNKGCSI----------------------------------------
----------------------------------------------------------------------
----------------------------------------------------------------------
----------------------------------------------------------------------
----------------------------------------------------------------------
--------------------------
>AB34299_6
----------------------------------------------------------------------
-------------------------------APCSLVRY---KPDKVTPQAGSRQDAIDRAWSL------
----------------------------------------------------------------------
----------------------------------------------------------------------
----------------------------------------------------------------------
----------EK-QLIQTTGTG------------------------------------------------
----------------------------------------------------------------------
----------------------------------TRDWSKAELDTIL-----------RTPSGSGKGHLS
S------------VMSNLGYTGHHINSVKNN--GA-----------------------------------
----------------------------------------------------------------------
------------------------------------------------------------LGESWKGDPR
NIVF------------------------------------------------------------LENPKH
PNSSPMPNAYNE--HFHSKQGHRGSTTNVSRGR-------------------------------------
----------------------------------------------------------------------
----------------------------------------------------------------------
-------------LIDRQAMINQFNKGCSI----------------------------------------
----------------------------------------------------------------------
----------------------------------------------------------------------
----------------------------------------------------------------------
----------------------------------------------------------------------
--------------------------

```
>Ab4568_6
------------------------------------------------------------------------
--------------------------------APCSLVRY---KPDKVTPQAGSRQDAIDRAWSL-------
------------------------------------------------------------------------
------------------------------------------------------------------------
------------------------------------------------------------------------
----------EK-QLIQTTGTG--------------------------------------------------
------------------------------------------------------------------------
-----------------------------------TRDWSKAELDTIL------------RTPSGSGKGHLS
S------------VMSNLGYTGHHINSVKNN--GA-------------------------------------
------------------------------------------------------------------------
--------------------------------------------------------------LGESWKGDPR
NIVF--------------------------------------------------------------LENPKH
PNSSPMPNAYNE--HFHSKQGHRGSTTNVSRGR---------------------------------------
------------------------------------------------------------------------
------------------------------------------------------------------------
-------------LIDRQAMINQFNKGCSI------------------------------------------
------------------------------------------------------------------------
------------------------------------------------------------------------
------------------------------------------------------------------------
------------------------------------------------------------------------
---------------------------
>Ab4653_6
------------------------------------------------------------------------
--------------------------------APCSLVRY---KPDKVTPQAGSRQDAIDRAWSL-------
------------------------------------------------------------------------
------------------------------------------------------------------------
------------------------------------------------------------------------
----------EK-QLIQTTGTG--------------------------------------------------
------------------------------------------------------------------------
-----------------------------------TRDWSKAELDTIL------------RTPSGSGKGHLS
S------------VMSNLGYTGHHINSVKNN--GA-------------------------------------
------------------------------------------------------------------------
```

--------------------------------------------------------------LGESWKGDPR
NIVF--------------------------------------------------------------LENPKH
PNSSPMPNAYNE--HFHSKQGHRGSTTNVSRGR---------------------------------------
------------------------------------------------------------------------
------------------------------------------------------------------------
-------------LIDRQAMINQFNKGCSI------------------------------------------
------------------------------------------------------------------------
------------------------------------------------------------------------
------------------------------------------------------------------------
------------------------------------------------------------------------
---------------------------
>Ab4977_6
------------------------------------------------------------------------
--------------------------------APCSLVRY---KPDKVTPQAGSRQDAIDRAWSL-------
------------------------------------------------------------------------
------------------------------------------------------------------------
------------------------------------------------------------------------
----------EK-QLIQTTGTG--------------------------------------------------
------------------------------------------------------------------------
-----------------------------------TRDWSKAELDTIL------------RTPSGSGKGHLS
S------------VMSNLGYTGHHINSVKNN--GA-------------------------------------
------------------------------------------------------------------------
--------------------------------------------------------------LGESWKGDPR
NIVF--------------------------------------------------------------LENPKH
PNSSPMPNAYNE--HFHSKQGHRGSTTNVSRGR---------------------------------------
------------------------------------------------------------------------
------------------------------------------------------------------------
-------------LIDRQAMINQFNKGCSI------------------------------------------
------------------------------------------------------------------------
------------------------------------------------------------------------
------------------------------------------------------------------------
------------------------------------------------------------------------
---------------------------

```
>AbPK1_6
------------------------------------------------------------------------
--------------------------------APCSLVRY---KPDKVTPQAGSRQDAIDRAWSL-------
------------------------------------------------------------------------
------------------------------------------------------------------------
------------------------------------------------------------------------
----------EK-QLIQTTGTG--------------------------------------------------
------------------------------------------------------------------------
-----------------------------------TRDWSKAELDTIL------------RTPSGSGKGHLS
S------------VMSNLGYTGHHINSVKNN--GA-------------------------------------
------------------------------------------------------------------------
--------------------------------------------------------------LGESWKGDPR
NIVF--------------------------------------------------------------LENPKH
PNSSPMPNAYNE--HFHSKQGHRGSTTNVSRGR---------------------------------------
------------------------------------------------------------------------
------------------------------------------------------------------------
-------------LIDRQAMINQFNKGCSI------------------------------------------
------------------------------------------------------------------------
------------------------------------------------------------------------
------------------------------------------------------------------------
------------------------------------------------------------------------
---------------------------
>AC29_6
------------------------------------------------------------------------
--------------------------------APCSLVRY---KPDKVTPQAGSRQDAIDRAWSL-------
------------------------------------------------------------------------
------------------------------------------------------------------------
------------------------------------------------------------------------
----------EK-QLIQTTGTG--------------------------------------------------
------------------------------------------------------------------------
-----------------------------------TRDWSKAELDTIL------------RTPSGSGKGHLS
S------------VMSNLGYTGHHINSVKNN--GA-------------------------------------
------------------------------------------------------------------------
```

```
--------------------------------------------------------------LGESWKGDPR
NIVF--------------------------------------------------------------LENPKH
PNSSPMPNAYNE--HFHSKQGHRGSTTNVSRGR---------------------------------------
------------------------------------------------------------------------
------------------------------------------------------------------------
-------------LIDRQAMINQFNKGCSI------------------------------------------
------------------------------------------------------------------------
------------------------------------------------------------------------
------------------------------------------------------------------------
------------------------------------------------------------------------
---------------------------
>AC30_6
------------------------------------------------------------------------
--------------------------------APCSLVRY---KPDKVTPQAGSRQDAIDRAWSL-------
------------------------------------------------------------------------
------------------------------------------------------------------------
------------------------------------------------------------------------
----------EK-QLIQTTGTG--------------------------------------------------
------------------------------------------------------------------------
-----------------------------------TRDWSKAELDTIL------------RTPSGSGKGHLS
S------------VMSNLGYTGHHINSVKNN--GA-------------------------------------
------------------------------------------------------------------------
--------------------------------------------------------------LGESWKGDPR
NIVF--------------------------------------------------------------LENPKH
PNSSPMPNAYNE--HFHSKQGHRGSTTNVSRGR---------------------------------------
------------------------------------------------------------------------
------------------------------------------------------------------------
-------------LIDRQAMINQFNKGCSI------------------------------------------
------------------------------------------------------------------------
------------------------------------------------------------------------
------------------------------------------------------------------------
------------------------------------------------------------------------
---------------------------
```

```
>ACICU_6
------------------------------------------------------------------------
--------------------------------APCSLVRY---KPDKVTPQAGSRQDAIDRAWSL-------
------------------------------------------------------------------------
------------------------------------------------------------------------
------------------------------------------------------------------------
----------EK-QLIQTTGTG--------------------------------------------------
------------------------------------------------------------------------
-----------------------------------TRDWSKAELDTIL------------RTPSGSGKGHLS
S------------VMSNLGYTGHHINSVKNN--GA-------------------------------------
------------------------------------------------------------------------
--------------------------------------------------------------LGESWKGDPR
NIVF--------------------------------------------------------------LENPKH
PNSSPMPNAYNE--HFHSKQGHRGSTTNVSRGR---------------------------------------
------------------------------------------------------------------------
------------------------------------------------------------------------
-------------LIDRQAMINQFNKGCSI------------------------------------------
------------------------------------------------------------------------
------------------------------------------------------------------------
------------------------------------------------------------------------
------------------------------------------------------------------------
---------------------------
>AF-673_6
------------------------------------------------------------------------
--------------------------------APCSLVRY---KPDKVTPQAGSRQDAIDRAWSL-------
------------------------------------------------------------------------
------------------------------------------------------------------------
------------------------------------------------------------------------
----------EK-QLIQTTGTG--------------------------------------------------
------------------------------------------------------------------------
-----------------------------------TRDWSKAELDTIL------------RTPSGSGKGHLS
S------------VMSNLGYTGHHINSVKNN--GA-------------------------------------
------------------------------------------------------------------------
```

```
------------------------------------------------------------LGESWKGDPR
NIVF------------------------------------------------------------LENPKH
PNSSPMPNAYNE--HFHSKQGHRGSTTNVSRGR-------------------------------------
----------------------------------------------------------------------
----------------------------------------------------------------------
-------------LIDRQAMINQFNKGCSI----------------------------------------
----------------------------------------------------------------------
----------------------------------------------------------------------
----------------------------------------------------------------------
----------------------------------------------------------------------
--------------------------
>AR_0056_6
----------------------------------------------------------------------
-------------------------------APCSLVRY---KPDKVTPQAGSRQDAIDRAWSL------
----------------------------------------------------------------------
----------------------------------------------------------------------
----------------------------------------------------------------------
----------EK-QLIQTTGTG------------------------------------------------
----------------------------------------------------------------------
----------------------------------TRDWSKAELDTIL-----------RTPSGSGKGHLS
S------------VMSNLGYTGHHINSVKNN--GA-----------------------------------
----------------------------------------------------------------------
------------------------------------------------------------LGESWKGDPR
NIVF------------------------------------------------------------LENPKH
PNSSPMPNAYNE--HFHSKQGHRGSTTNVSRGR-------------------------------------
----------------------------------------------------------------------
----------------------------------------------------------------------
-------------LIDRQAMINQFNKGCSI----------------------------------------
----------------------------------------------------------------------
----------------------------------------------------------------------
----------------------------------------------------------------------
----------------------------------------------------------------------
--------------------------
```

>AYP-A2_6
------------------------------------------------------------------------
--------------------------------APCSLVRY---KPDKVTPQAGSRQDAIDRAWSL-------
------------------------------------------------------------------------
------------------------------------------------------------------------
------------------------------------------------------------------------
----------EK-QLIQTTGTG--------------------------------------------------
------------------------------------------------------------------------
-----------------------------------TRDWSKAELDTIL------------RTPSGSGKGHLS
S------------VMSNLGYTGHHINSVKNN--GA-------------------------------------
------------------------------------------------------------------------
--------------------------------------------------------------LGESWKGDPR
NIVF--------------------------------------------------------------LENPKH
PNSSPMPNAYNE--HFHSKQGHRGSTTNVSRGR---------------------------------------
------------------------------------------------------------------------
------------------------------------------------------------------------
-------------LIDRQAMINQFNKGCSI------------------------------------------
------------------------------------------------------------------------
------------------------------------------------------------------------
------------------------------------------------------------------------
------------------------------------------------------------------------
---------------------------
>BJAB07104_6
------------------------------------------------------------------------
--------------------------------APCSLVRY---KPDKVTPQAGSRQDAIDRAWSL-------
------------------------------------------------------------------------
------------------------------------------------------------------------
------------------------------------------------------------------------
----------EK-QLIQTTGTG--------------------------------------------------
------------------------------------------------------------------------
-----------------------------------TRDWSKAELDTIL------------RTPSGSGKGHLS
S------------VMSNLGYTGHHINSVKNN--GA-------------------------------------
------------------------------------------------------------------------

--------------------------------------------------------------LGESWKGDPR
NIVF--------------------------------------------------------------LENPKH
PNSSPMPNAYNE--HFHSKQGHRGSTTNVSRGR---------------------------------------
------------------------------------------------------------------------
------------------------------------------------------------------------
-------------LIDRQAMINQFNKGCSI------------------------------------------
------------------------------------------------------------------------
------------------------------------------------------------------------
------------------------------------------------------------------------
------------------------------------------------------------------------
---------------------------
>BJAB0868_6
------------------------------------------------------------------------
--------------------------------APCSLVRY---KPDKVTPQAGSRQDAIDRAWSL-------
------------------------------------------------------------------------
------------------------------------------------------------------------
------------------------------------------------------------------------
----------EK-QLIQTTGTG--------------------------------------------------
------------------------------------------------------------------------
-----------------------------------TRDWSKAELDTIL------------RTPSGSGKGHLS
S------------VMSNLGYTGHHINSVKNN--GA-------------------------------------
------------------------------------------------------------------------
--------------------------------------------------------------LGESWKGDPR
NIVF--------------------------------------------------------------LENPKH
PNSSPMPNAYNE--HFHSKQGHRGSTTNVSRGR---------------------------------------
------------------------------------------------------------------------
------------------------------------------------------------------------
-------------LIDRQAMINQFNKGCSI------------------------------------------
------------------------------------------------------------------------
------------------------------------------------------------------------
------------------------------------------------------------------------
------------------------------------------------------------------------
---------------------------

```
>CBA7_6
------------------------------------------------------------------------
--------------------------------APCSLVRY---KPDKVTPQAGSRQDAIDRAWSL-------
------------------------------------------------------------------------
------------------------------------------------------------------------
------------------------------------------------------------------------
----------EK-QLIQTTGTG--------------------------------------------------
------------------------------------------------------------------------
-----------------------------------TRDWSKAELDTIL------------RTPSGSGKGHLS
S------------VMSNLGYTGHHINSVKNN--GA-------------------------------------
------------------------------------------------------------------------
--------------------------------------------------------------LGESWKGDPR
NIVF--------------------------------------------------------------LENPKH
PNSSPMPNAYNE--HFHSKQGHRGSTTNVSRGR---------------------------------------
------------------------------------------------------------------------
------------------------------------------------------------------------
-------------LIDRQAMINQFNKGCSI------------------------------------------
------------------------------------------------------------------------
------------------------------------------------------------------------
------------------------------------------------------------------------
------------------------------------------------------------------------
---------------------------
>CMC-CR-MDR-Ab4_6
------------------------------------------------------------------------
--------------------------------APCSLVRY---KPDKVTPQAGSRQDAIDRAWSL-------
------------------------------------------------------------------------
------------------------------------------------------------------------
------------------------------------------------------------------------
----------EK-QLIQTTGTG--------------------------------------------------
------------------------------------------------------------------------
-----------------------------------TRDWSKAELDTIL------------RTPSGSGKGHLS
S------------VMSNLGYTGHHINSVKNN--GA-------------------------------------
------------------------------------------------------------------------
```

--------------------------------------------------------------LGESWKGDPR
NIVF--------------------------------------------------------------LENPKH
PNSSPMPNAYNE--HFHSKQGHRGSTTNVSRGR---------------------------------------
------------------------------------------------------------------------
------------------------------------------------------------------------
-------------LIDRQAMINQFNKGCSI------------------------------------------
------------------------------------------------------------------------
------------------------------------------------------------------------
------------------------------------------------------------------------
------------------------------------------------------------------------
---------------------------
>CMC-CR-MDR-Ab66_6
------------------------------------------------------------------------
--------------------------------APCSLVRY---KPDKVTPQAGSRQDAIDRAWSL-------
------------------------------------------------------------------------
------------------------------------------------------------------------
------------------------------------------------------------------------
----------EK-QLIQTTGTG--------------------------------------------------
------------------------------------------------------------------------
-----------------------------------TRDWSKAELDTIL------------RTPSGSGKGHLS
S------------VMSNLGYTGHHINSVKNN--GA-------------------------------------
------------------------------------------------------------------------
--------------------------------------------------------------LGESWKGDPR
NIVF--------------------------------------------------------------LENPKH
PNSSPMPNAYNE--HFHSKQGHRGSTTNVSRGR---------------------------------------
------------------------------------------------------------------------
------------------------------------------------------------------------
-------------LIDRQAMINQFNKGCSI------------------------------------------
------------------------------------------------------------------------
------------------------------------------------------------------------
------------------------------------------------------------------------
------------------------------------------------------------------------
---------------------------

```
>CMC-MDR-Ab59_6
------------------------------------------------------------------------
--------------------------------APCSLVRY---KPDKVTPQAGSRQDAIDRAWSL-------
------------------------------------------------------------------------
------------------------------------------------------------------------
------------------------------------------------------------------------
----------EK-QLIQTTGTG--------------------------------------------------
------------------------------------------------------------------------
-----------------------------------TRDWSKAELDTIL------------RTPSGSGKGHLS
S------------VMSNLGYTGHHINSVKNN--GA-------------------------------------
------------------------------------------------------------------------
--------------------------------------------------------------LGESWKGDPR
NIVF--------------------------------------------------------------LENPKH
PNSSPMPNAYNE--HFHSKQGHRGSTTNVSRGR---------------------------------------
------------------------------------------------------------------------
------------------------------------------------------------------------
-------------LIDRQAMINQFNKGCSI------------------------------------------
------------------------------------------------------------------------
------------------------------------------------------------------------
------------------------------------------------------------------------
------------------------------------------------------------------------
---------------------------
>DU202_6
------------------------------------------------------------------------
--------------------------------APCSLVRY---KPDKVTPQAGSRQDAIDRAWSL-------
------------------------------------------------------------------------
------------------------------------------------------------------------
------------------------------------------------------------------------
----------EK-QLIQTTGTG--------------------------------------------------
------------------------------------------------------------------------
-----------------------------------TRDWSKAELDTIL------------RTPSGSGKGHLS
S------------VMSNLGYTGHHINSVKNN--GA-------------------------------------
------------------------------------------------------------------------
```

--------------------------------------------------------------LGESWKGDPR
NIVF--------------------------------------------------------------LENPKH
PNSSPMPNAYNE--HFHSKQGHRGSTTNVSRGR---------------------------------------
------------------------------------------------------------------------
------------------------------------------------------------------------
-------------LIDRQAMINQFNKGCSI------------------------------------------
------------------------------------------------------------------------
------------------------------------------------------------------------
------------------------------------------------------------------------
------------------------------------------------------------------------
---------------------------
>HRAB-85_6
------------------------------------------------------------------------
--------------------------------APCSLVRY---KPDKVTPQAGSRQDAIDRAWSL-------
------------------------------------------------------------------------
------------------------------------------------------------------------
------------------------------------------------------------------------
----------EK-QLIQTTGTG--------------------------------------------------
------------------------------------------------------------------------
-----------------------------------TRDWSKAELDTIL------------RTPSGSGKGHLS
S------------VMSNLGYTGHHINSVKNN--GA-------------------------------------
------------------------------------------------------------------------
--------------------------------------------------------------LGESWKGDPR
NIVF--------------------------------------------------------------LENPKH
PNSSPMPNAYNE--HFHSKQGHRGSTTNVSRGR---------------------------------------
------------------------------------------------------------------------
------------------------------------------------------------------------
-------------LIDRQAMINQFNKGCSI------------------------------------------
------------------------------------------------------------------------
------------------------------------------------------------------------
------------------------------------------------------------------------
------------------------------------------------------------------------
---------------------------

```
>JBA13_6
------------------------------------------------------------------------
--------------------------------APCSLVRY---KPDKVTPQAGSRQDAIDRAWSL-------
------------------------------------------------------------------------
------------------------------------------------------------------------
------------------------------------------------------------------------
----------EK-QLIQTTGTG--------------------------------------------------
------------------------------------------------------------------------
-----------------------------------TRDWSKAELDTIL------------RTPSGSGKGHLS
S------------VMSNLGYTGHHINSVKNN--GA-------------------------------------
------------------------------------------------------------------------
--------------------------------------------------------------LGESWKGDPR
NIVF--------------------------------------------------------------LENPKH
PNSSPMPNAYNE--HFHSKQGHRGSTTNVSRGR---------------------------------------
------------------------------------------------------------------------
------------------------------------------------------------------------
-------------LIDRQAMINQFNKGCSI------------------------------------------
------------------------------------------------------------------------
------------------------------------------------------------------------
------------------------------------------------------------------------
------------------------------------------------------------------------
---------------------------
>KAB01_6
------------------------------------------------------------------------
--------------------------------APCSLVRY---KPDKVTPQAGSRQDAIDRAWSL-------
------------------------------------------------------------------------
------------------------------------------------------------------------
------------------------------------------------------------------------
----------EK-QLIQTTGTG--------------------------------------------------
------------------------------------------------------------------------
-----------------------------------TRDWSKAELDTIL------------RTPSGSGKGHLS
S------------VMSNLGYTGHHINSVKNN--GA-------------------------------------
------------------------------------------------------------------------
```

```
--------------------------------------------------------------LGESWKGDPR
NIVF--------------------------------------------------------------LENPKH
PNSSPMPNAYNE--HFHSKQGHRGSTTNVSRGR---------------------------------------
------------------------------------------------------------------------
------------------------------------------------------------------------
-------------LIDRQAMINQFNKGCSI------------------------------------------
------------------------------------------------------------------------
------------------------------------------------------------------------
------------------------------------------------------------------------
------------------------------------------------------------------------
---------------------------
>KAB02_6
------------------------------------------------------------------------
--------------------------------APCSLVRY---KPDKVTPQAGSRQDAIDRAWSL-------
------------------------------------------------------------------------
------------------------------------------------------------------------
------------------------------------------------------------------------
----------EK-QLIQTTGTG--------------------------------------------------
------------------------------------------------------------------------
-----------------------------------TRDWSKAELDTIL------------RTPSGSGKGHLS
S------------VMSNLGYTGHHINSVKNN--GA-------------------------------------
------------------------------------------------------------------------
--------------------------------------------------------------LGESWKGDPR
NIVF--------------------------------------------------------------LENPKH
PNSSPMPNAYNE--HFHSKQGHRGSTTNVSRGR---------------------------------------
------------------------------------------------------------------------
------------------------------------------------------------------------
-------------LIDRQAMINQFNKGCSI------------------------------------------
------------------------------------------------------------------------
------------------------------------------------------------------------
------------------------------------------------------------------------
------------------------------------------------------------------------
---------------------------
```

>KAB03_6
------------------------------------------------------------------------
--------------------------------APCSLVRY---KPDKVTPQAGSRQDAIDRAWSL-------
------------------------------------------------------------------------
------------------------------------------------------------------------
------------------------------------------------------------------------
----------EK-QLIQTTGTG--------------------------------------------------
------------------------------------------------------------------------
-----------------------------------TRDWSKAELDTIL------------RTPSGSGKGHLS
S------------VMSNLGYTGHHINSVKNN--GA-------------------------------------
------------------------------------------------------------------------
--------------------------------------------------------------LGESWKGDPR
NIVF--------------------------------------------------------------LENPKH
PNSSPMPNAYNE--HFHSKQGHRGSTTNVSRGR---------------------------------------
------------------------------------------------------------------------
------------------------------------------------------------------------
-------------LIDRQAMINQFNKGCSI------------------------------------------
------------------------------------------------------------------------
------------------------------------------------------------------------
------------------------------------------------------------------------
------------------------------------------------------------------------
---------------------------
>KAB04_6
------------------------------------------------------------------------
--------------------------------APCSLVRY---KPDKVTPQAGSRQDAIDRAWSL-------
------------------------------------------------------------------------
------------------------------------------------------------------------
------------------------------------------------------------------------
----------EK-QLIQTTGTG--------------------------------------------------
------------------------------------------------------------------------
-----------------------------------TRDWSKAELDTIL------------RTPSGSGKGHLS
S------------VMSNLGYTGHHINSVKNN--GA-------------------------------------
------------------------------------------------------------------------

--------------------------------------------------------------LGESWKGDPR
NIVF--------------------------------------------------------------LENPKH
PNSSPMPNAYNE--HFHSKQGHRGSTTNVSRGR---------------------------------------
------------------------------------------------------------------------
------------------------------------------------------------------------
-------------LIDRQAMINQFNKGCSI------------------------------------------
------------------------------------------------------------------------
------------------------------------------------------------------------
------------------------------------------------------------------------
------------------------------------------------------------------------
---------------------------
>KAB05_6
------------------------------------------------------------------------
--------------------------------APCSLVRY---KPDKVTPQAGSRQDAIDRAWSL-------
------------------------------------------------------------------------
------------------------------------------------------------------------
------------------------------------------------------------------------
----------EK-QLIQTTGTG--------------------------------------------------
------------------------------------------------------------------------
-----------------------------------TRDWSKAELDTIL------------RTPSGSGKGHLS
S------------VMSNLGYTGHHINSVKNN--GA-------------------------------------
------------------------------------------------------------------------
--------------------------------------------------------------LGESWKGDPR
NIVF--------------------------------------------------------------LENPKH
PNSSPMPNAYNE--HFHSKQGHRGSTTNVSRGR---------------------------------------
------------------------------------------------------------------------
------------------------------------------------------------------------
-------------LIDRQAMINQFNKGCSI------------------------------------------
------------------------------------------------------------------------
------------------------------------------------------------------------
------------------------------------------------------------------------
------------------------------------------------------------------------
---------------------------

```
>KAB06_6
------------------------------------------------------------------------
--------------------------------APCSLVRY---KPDKVTPQAGSRQDAIDRAWSL-------
------------------------------------------------------------------------
------------------------------------------------------------------------
------------------------------------------------------------------------
----------EK-QLIQTTGTG--------------------------------------------------
------------------------------------------------------------------------
-----------------------------------TRDWSKAELDTIL------------RTPSGSGKGHLS
S------------VMSNLGYTGHHINSVKNN--GA-------------------------------------
------------------------------------------------------------------------
--------------------------------------------------------------LGESWKGDPR
NIVF--------------------------------------------------------------LENPKH
PNSSPMPNAYNE--HFHSKQGHRGSTTNVSRGR---------------------------------------
------------------------------------------------------------------------
------------------------------------------------------------------------
-------------LIDRQAMINQFNKGCSI------------------------------------------
------------------------------------------------------------------------
------------------------------------------------------------------------
------------------------------------------------------------------------
------------------------------------------------------------------------
---------------------------
>KAB07_6
------------------------------------------------------------------------
--------------------------------APCSLVRY---KPDKVTPQAGSRQDAIDRAWSL-------
------------------------------------------------------------------------
------------------------------------------------------------------------
------------------------------------------------------------------------
----------EK-QLIQTTGTG--------------------------------------------------
------------------------------------------------------------------------
-----------------------------------TRDWSKAELDTIL------------RTPSGSGKGHLS
S------------VMSNLGYTGHHINSVKNN--GA-------------------------------------
------------------------------------------------------------------------
```

```
------------------------------------------------------------LGESWKGDPR
NIVF------------------------------------------------------------LENPKH
PNSSPMPNAYNE--HFHSKQGHRGSTTNVSRGR-------------------------------------
----------------------------------------------------------------------
----------------------------------------------------------------------
-------------LIDRQAMINQFNKGCSI----------------------------------------
----------------------------------------------------------------------
----------------------------------------------------------------------
----------------------------------------------------------------------
----------------------------------------------------------------------
--------------------------
>KAB08_6
----------------------------------------------------------------------
-------------------------------APCSLVRY---KPDKVTPQAGSRQDAIDRAWSL------
----------------------------------------------------------------------
----------------------------------------------------------------------
----------------------------------------------------------------------
----------EK-QLIQTTGTG------------------------------------------------
----------------------------------------------------------------------
----------------------------------TRDWSKAELDTIL-----------RTPSGSGKGHLS
S------------VMSNLGYTGHHINSVKNN--GA-----------------------------------
----------------------------------------------------------------------
------------------------------------------------------------LGESWKGDPR
NIVF------------------------------------------------------------LENPKH
PNSSPMPNAYNE--HFHSKQGHRGSTTNVSRGR-------------------------------------
----------------------------------------------------------------------
----------------------------------------------------------------------
-------------LIDRQAMINQFNKGCSI----------------------------------------
----------------------------------------------------------------------
----------------------------------------------------------------------
----------------------------------------------------------------------
----------------------------------------------------------------------
--------------------------
```

```
>KBN10P02143_6
------------------------------------------------------------------------
--------------------------------APCSLVRY---KPDKVTPQAGSRQDAIDRAWSL-------
------------------------------------------------------------------------
------------------------------------------------------------------------
------------------------------------------------------------------------
----------EK-QLIQTTGTG--------------------------------------------------
------------------------------------------------------------------------
-----------------------------------TRDWSKAELDTIL------------RTPSGSGKGHLS
S------------VMSNLGYTGHHINSVKNN--GA-------------------------------------
------------------------------------------------------------------------
--------------------------------------------------------------LGESWKGDPR
NIVF--------------------------------------------------------------LENPKH
PNSSPMPNAYNE--HFHSKQGHRGSTTNVSRGR---------------------------------------
------------------------------------------------------------------------
------------------------------------------------------------------------
-------------LIDRQAMINQFNKGCSI------------------------------------------
------------------------------------------------------------------------
------------------------------------------------------------------------
------------------------------------------------------------------------
------------------------------------------------------------------------
---------------------------
>MDR-TJ_6
------------------------------------------------------------------------
--------------------------------APCSLVRY---KPDKVTPQAGSRQDAIDRAWSL-------
------------------------------------------------------------------------
------------------------------------------------------------------------
------------------------------------------------------------------------
----------EK-QLIQTTGTG--------------------------------------------------
------------------------------------------------------------------------
-----------------------------------TRDWSKAELDTIL------------RTPSGSGKGHLS
S------------VMSNLGYTGHHINSVKNN--GA-------------------------------------
------------------------------------------------------------------------
```

```
--------------------------------------------------------------LGESWKGDPR
NIVF--------------------------------------------------------------LENPKH
PNSSPMPNAYNE--HFHSKQGHRGSTTNVSRGR---------------------------------------
------------------------------------------------------------------------
------------------------------------------------------------------------
-------------LIDRQAMINQFNKGCSI------------------------------------------
------------------------------------------------------------------------
------------------------------------------------------------------------
------------------------------------------------------------------------
------------------------------------------------------------------------
---------------------------
>MDR-ZJ06_6
------------------------------------------------------------------------
--------------------------------APCSLVRY---KPDKVTPQAGSRQDAIDRAWSL-------
------------------------------------------------------------------------
------------------------------------------------------------------------
------------------------------------------------------------------------
----------EK-QLIQTTGTG--------------------------------------------------
------------------------------------------------------------------------
-----------------------------------TRDWSKAELDTIL------------RTPSGSGKGHLS
S------------VMSNLGYTGHHINSVKNN--GA-------------------------------------
------------------------------------------------------------------------
--------------------------------------------------------------LGESWKGDPR
NIVF--------------------------------------------------------------LENPKH
PNSSPMPNAYNE--HFHSKQGHRGSTTNVSRGR---------------------------------------
------------------------------------------------------------------------
------------------------------------------------------------------------
-------------LIDRQAMINQFNKGCSI------------------------------------------
------------------------------------------------------------------------
------------------------------------------------------------------------
------------------------------------------------------------------------
------------------------------------------------------------------------
---------------------------
```

```
>NCGM237_6
------------------------------------------------------------------------
--------------------------------APCSLVRY---KPDKVTPQAGSRQDAIDRAWSL-------
------------------------------------------------------------------------
------------------------------------------------------------------------
------------------------------------------------------------------------
----------EK-QLIQTTGTG--------------------------------------------------
------------------------------------------------------------------------
-----------------------------------TRDWSKAELDTIL------------RTPSGSGKGHLS
S------------VMSNLGYTGHHINSVKNN--GA-------------------------------------
------------------------------------------------------------------------
--------------------------------------------------------------LGESWKGDPR
NIVF--------------------------------------------------------------LENPKH
PNSSPMPNAYNE--HFHSKQGHRGSTTNVSRGR---------------------------------------
------------------------------------------------------------------------
------------------------------------------------------------------------
-------------LIDRQAMINQFNKGCSI------------------------------------------
------------------------------------------------------------------------
------------------------------------------------------------------------
------------------------------------------------------------------------
------------------------------------------------------------------------
---------------------------
>SAA14_6
------------------------------------------------------------------------
--------------------------------APCSLVRY---KPDKVTPQAGSRQDAIDRAWSL-------
------------------------------------------------------------------------
------------------------------------------------------------------------
------------------------------------------------------------------------
----------EK-QLIQTTGTG--------------------------------------------------
------------------------------------------------------------------------
-----------------------------------TRDWSKAELDTIL------------RTPSGSGKGHLS
S------------VMSNLGYTGHHINSVKNN--GA-------------------------------------
------------------------------------------------------------------------
```

```
--------------------------------------------------------------LGESWKGDPR
NIVF--------------------------------------------------------------LENPKH
PNSSPMPNAYNE--HFHSKQGHRGSTTNVSRGR---------------------------------------
------------------------------------------------------------------------
------------------------------------------------------------------------
-------------LIDRQAMINQFNKGCSI------------------------------------------
------------------------------------------------------------------------
------------------------------------------------------------------------
------------------------------------------------------------------------
------------------------------------------------------------------------
---------------------------
>SMC_Paed_Ab_BL01_6
------------------------------------------------------------------------
--------------------------------APCSLVRY---KPDKVTPQAGSRQDAIDRAWSL-------
------------------------------------------------------------------------
------------------------------------------------------------------------
------------------------------------------------------------------------
----------EK-QLIQTTGTG--------------------------------------------------
------------------------------------------------------------------------
-----------------------------------TRDWSKAELDTIL------------RTPSGSGKGHLS
S------------VMSNLGYTGHHINSVKNN--GA-------------------------------------
------------------------------------------------------------------------
--------------------------------------------------------------LGESWKGDPR
NIVF--------------------------------------------------------------LENPKH
PNSSPMPNAYNE--HFHSKQGHRGSTTNVSRGR---------------------------------------
------------------------------------------------------------------------
------------------------------------------------------------------------
-------------LIDRQAMINQFNKGCSI------------------------------------------
------------------------------------------------------------------------
------------------------------------------------------------------------
------------------------------------------------------------------------
------------------------------------------------------------------------
---------------------------
```

```
>SSA12_6
------------------------------------------------------------------------
--------------------------------APCSLVRY---KPDKVTPQAGSRQDAIDRAWSL-------
------------------------------------------------------------------------
------------------------------------------------------------------------
------------------------------------------------------------------------
----------EK-QLIQTTGTG--------------------------------------------------
------------------------------------------------------------------------
-----------------------------------TRDWSKAELDTIL------------RTPSGSGKGHLS
S------------VMSNLGYTGHHINSVKNN--GA-------------------------------------
------------------------------------------------------------------------
--------------------------------------------------------------LGESWKGDPR
NIVF--------------------------------------------------------------LENPKH
PNSSPMPNAYNE--HFHSKQGHRGSTTNVSRGR---------------------------------------
------------------------------------------------------------------------
------------------------------------------------------------------------
-------------LIDRQAMINQFNKGCSI------------------------------------------
------------------------------------------------------------------------
------------------------------------------------------------------------
------------------------------------------------------------------------
------------------------------------------------------------------------
---------------------------
>SSA6_6
------------------------------------------------------------------------
--------------------------------APCSLVRY---KPDKVTPQAGSRQDAIDRAWSL-------
------------------------------------------------------------------------
------------------------------------------------------------------------
------------------------------------------------------------------------
----------EK-QLIQTTGTG--------------------------------------------------
------------------------------------------------------------------------
-----------------------------------TRDWSKAELDTIL------------RTPSGSGKGHLS
S------------VMSNLGYTGHHINSVKNN--GA-------------------------------------
------------------------------------------------------------------------
```

--------------------------------------------------------------LGESWKGDPR
NIVF--------------------------------------------------------------LENPKH
PNSSPMPNAYNE--HFHSKQGHRGSTTNVSRGR---------------------------------------
------------------------------------------------------------------------
------------------------------------------------------------------------
-------------LIDRQAMINQFNKGCSI------------------------------------------
------------------------------------------------------------------------
------------------------------------------------------------------------
------------------------------------------------------------------------
------------------------------------------------------------------------
---------------------------
>TCDC-AB0715_6
------------------------------------------------------------------------
--------------------------------APCSLVRY---KPDKVTPQAGSRQDAIDRAWSL-------
------------------------------------------------------------------------
------------------------------------------------------------------------
------------------------------------------------------------------------
----------EK-QLIQTTGTG--------------------------------------------------
------------------------------------------------------------------------
-----------------------------------TRDWSKAELDTIL------------RTPSGSGKGHLS
S------------VMSNLGYTGHHINSVKNN--GA-------------------------------------
------------------------------------------------------------------------
--------------------------------------------------------------LGESWKGDPR
NIVF--------------------------------------------------------------LENPKH
PNSSPMPNAYNE--HFHSKQGHRGSTTNVSRGR---------------------------------------
------------------------------------------------------------------------
------------------------------------------------------------------------
-------------LIDRQAMINQFNKGCSI------------------------------------------
------------------------------------------------------------------------
------------------------------------------------------------------------
------------------------------------------------------------------------
------------------------------------------------------------------------
---------------------------

>TYTH-1_6
------------------------------------------------------------------------
--------------------------------APCSLVRY---KPDKVTPQAGSRQDAIDRAWSL-------
------------------------------------------------------------------------
------------------------------------------------------------------------
------------------------------------------------------------------------
----------EK-QLIQTTGTG--------------------------------------------------
------------------------------------------------------------------------
-----------------------------------TRDWSKAELDTIL------------RTPSGSGKGHLS
S------------VMSNLGYTGHHINSVKNN--GA-------------------------------------
------------------------------------------------------------------------
--------------------------------------------------------------LGESWKGDPR
NIVF--------------------------------------------------------------LENPKH
PNSSPMPNAYNE--HFHSKQGHRGSTTNVSRGR---------------------------------------
------------------------------------------------------------------------
------------------------------------------------------------------------
-------------LIDRQAMINQFNKGCSI------------------------------------------
------------------------------------------------------------------------
------------------------------------------------------------------------
------------------------------------------------------------------------
------------------------------------------------------------------------
---------------------------
>USA2_6
------------------------------------------------------------------------
--------------------------------APCSLVRY---KPDKVTPQAGSRQDAIDRAWSL-------
------------------------------------------------------------------------
------------------------------------------------------------------------
------------------------------------------------------------------------
----------EK-QLIQTTGTG--------------------------------------------------
------------------------------------------------------------------------
-----------------------------------TRDWSKAELDTIL------------RTPSGSGKGHLS
S------------VMSNLGYTGHHINSVKNN--GA-------------------------------------
------------------------------------------------------------------------

--------------------------------------------------------------LGESWKGDPR
NIVF--------------------------------------------------------------LENPKH
PNSSPMPNAYNE--HFHSKQGHRGSTTNVSRGR---------------------------------------
------------------------------------------------------------------------
------------------------------------------------------------------------
-------------LIDRQAMINQFNKGCSI------------------------------------------
------------------------------------------------------------------------
------------------------------------------------------------------------
------------------------------------------------------------------------
------------------------------------------------------------------------
---------------------------
>WCHAB005133_6
------------------------------------------------------------------------
--------------------------------APCSLVRY---KPDKVTPQAGSRQDAIDRAWSL-------
------------------------------------------------------------------------
------------------------------------------------------------------------
------------------------------------------------------------------------
----------EK-QLIQTTGTG--------------------------------------------------
------------------------------------------------------------------------
-----------------------------------TRDWSKAELDTIL------------RTPSGSGKGHLS
S------------VMSNLGYTGHHINSVKNN--GA-------------------------------------
------------------------------------------------------------------------
--------------------------------------------------------------LGESWKGDPR
NIVF--------------------------------------------------------------LENPKH
PNSSPMPNAYNE--HFHSKQGHRGSTTNVSRGR---------------------------------------
------------------------------------------------------------------------
------------------------------------------------------------------------
-------------LIDRQAMINQFNKGCSI------------------------------------------
------------------------------------------------------------------------
------------------------------------------------------------------------
------------------------------------------------------------------------
------------------------------------------------------------------------
---------------------------

```
>XDR-BJ83_6
------------------------------------------------------------------------
--------------------------------APCSLVRY---KPDKVTPQAGSRQDAIDRAWSL-------
------------------------------------------------------------------------
------------------------------------------------------------------------
------------------------------------------------------------------------
----------EK-QLIQTTGTG--------------------------------------------------
------------------------------------------------------------------------
-----------------------------------TRDWSKAELDTIL------------RTPSGSGKGHLS
S------------VMSNLGYTGHHINSVKNN--GA-------------------------------------
------------------------------------------------------------------------
--------------------------------------------------------------LGESWKGDPR
NIVF--------------------------------------------------------------LENPKH
PNSSPMPNAYNE--HFHSKQGHRGSTTNVSRGR---------------------------------------
------------------------------------------------------------------------
------------------------------------------------------------------------
-------------LIDRQAMINQFNKGCSI------------------------------------------
------------------------------------------------------------------------
------------------------------------------------------------------------
------------------------------------------------------------------------
------------------------------------------------------------------------
---------------------------
>XH386_6
------------------------------------------------------------------------
--------------------------------APCSLVRY---KPDKVTPQAGSRQDAIDRAWSL-------
------------------------------------------------------------------------
------------------------------------------------------------------------
------------------------------------------------------------------------
----------EK-QLIQTTGTG--------------------------------------------------
------------------------------------------------------------------------
-----------------------------------TRDWSKAELDTIL------------RTPSGSGKGHLS
S------------VMSNLGYTGHHINSVKNN--GA-------------------------------------
------------------------------------------------------------------------
```

--------------------------------------------------------------LGESWKGDPR
NIVF--------------------------------------------------------------LENPKH
PNSSPMPNAYNE--HFHSKQGHRGSTTNVSRGR---------------------------------------
------------------------------------------------------------------------
------------------------------------------------------------------------
-------------LIDRQAMINQFNKGCSI------------------------------------------
------------------------------------------------------------------------
------------------------------------------------------------------------
------------------------------------------------------------------------
------------------------------------------------------------------------
---------------------------
>XH856_6
------------------------------------------------------------------------
--------------------------------APCSLVRY---KPDKVTPQAGSRQDAIDRAWSL-------
------------------------------------------------------------------------
------------------------------------------------------------------------
------------------------------------------------------------------------
----------EK-QLIQTTGTG--------------------------------------------------
------------------------------------------------------------------------
-----------------------------------TRDWSKAELDTIL------------RTPSGSGKGHLS
S------------VMSNLGYTGHHINSVKNN--GA-------------------------------------
------------------------------------------------------------------------
--------------------------------------------------------------LGESWKGDPR
NIVF--------------------------------------------------------------LENPKH
PNSSPMPNAYNE--HFHSKQGHRGSTTNVSRGR---------------------------------------
------------------------------------------------------------------------
------------------------------------------------------------------------
-------------LIDRQAMINQFNKGCSI------------------------------------------
------------------------------------------------------------------------
------------------------------------------------------------------------
------------------------------------------------------------------------
------------------------------------------------------------------------
---------------------------

```
>XH857_6
------------------------------------------------------------------------
--------------------------------APCSLVRY---KPDKVTPQAGSRQDAIDRAWSL-------
------------------------------------------------------------------------
------------------------------------------------------------------------
------------------------------------------------------------------------
----------EK-QLIQTTGTG--------------------------------------------------
------------------------------------------------------------------------
-----------------------------------TRDWSKAELDTIL------------RTPSGSGKGHLS
S------------VMSNLGYTGHHINSVKNN--GA-------------------------------------
------------------------------------------------------------------------
--------------------------------------------------------------LGESWKGDPR
NIVF--------------------------------------------------------------LENPKH
PNSSPMPNAYNE--HFHSKQGHRGSTTNVSRGR---------------------------------------
------------------------------------------------------------------------
------------------------------------------------------------------------
-------------LIDRQAMINQFNKGCSI------------------------------------------
------------------------------------------------------------------------
------------------------------------------------------------------------
------------------------------------------------------------------------
------------------------------------------------------------------------
---------------------------
>XH859_6
------------------------------------------------------------------------
--------------------------------APCSLVRY---KPDKVTPQAGSRQDAIDRAWSL-------
------------------------------------------------------------------------
------------------------------------------------------------------------
------------------------------------------------------------------------
----------EK-QLIQTTGTG--------------------------------------------------
------------------------------------------------------------------------
-----------------------------------TRDWSKAELDTIL------------RTPSGSGKGHLS
S------------VMSNLGYTGHHINSVKNN--GA-------------------------------------
------------------------------------------------------------------------
```

--------------------------------------------------------------LGESWKGDPR
NIVF--------------------------------------------------------------LENPKH
PNSSPMPNAYNE--HFHSKQGHRGSTTNVSRGR---------------------------------------
------------------------------------------------------------------------
------------------------------------------------------------------------
-------------LIDRQAMINQFNKGCSI------------------------------------------
------------------------------------------------------------------------
------------------------------------------------------------------------
------------------------------------------------------------------------
------------------------------------------------------------------------
---------------------------
>XH860_6
------------------------------------------------------------------------
--------------------------------APCSLVRY---KPDKVTPQAGSRQDAIDRAWSL-------
------------------------------------------------------------------------
------------------------------------------------------------------------
------------------------------------------------------------------------
----------EK-QLIQTTGTG--------------------------------------------------
------------------------------------------------------------------------
-----------------------------------TRDWSKAELDTIL------------RTPSGSGKGHLS
S------------VMSNLGYTGHHINSVKNN--GA-------------------------------------
------------------------------------------------------------------------
--------------------------------------------------------------LGESWKGDPR
NIVF--------------------------------------------------------------LENPKH
PNSSPMPNAYNE--HFHSKQGHRGSTTNVSRGR---------------------------------------
------------------------------------------------------------------------
------------------------------------------------------------------------
-------------LIDRQAMINQFNKGCSI------------------------------------------
------------------------------------------------------------------------
------------------------------------------------------------------------
------------------------------------------------------------------------
------------------------------------------------------------------------
---------------------------

>YU-R612_6
------------------------------------------------------------------------
--------------------------------APCSLVRY---KPDKVTPQAGSRQDAIDRAWSL-------
------------------------------------------------------------------------
------------------------------------------------------------------------
------------------------------------------------------------------------
----------EK-QLIQTTGTG--------------------------------------------------
------------------------------------------------------------------------
-----------------------------------TRDWSKAELDTIL------------RTPSGSGKGHLS
S------------VMSNLGYTGHHINSVKNN--GA-------------------------------------
------------------------------------------------------------------------
--------------------------------------------------------------LGESWKGDPR
NIVF--------------------------------------------------------------LENPKH
PNSSPMPNAYNE--HFHSKQGHRGSTTNVSRGR---------------------------------------
------------------------------------------------------------------------
------------------------------------------------------------------------
-------------LIDRQAMINQFNKGCSI------------------------------------------
------------------------------------------------------------------------
------------------------------------------------------------------------
------------------------------------------------------------------------
------------------------------------------------------------------------
---------------------------
>Rhs1
------------------------------------------------------------------------
-------KPRCAATKANDHNQA---------------------AFGRQW-QGRGIYKGRDSWSNI-------
------------------------------------------------------------------------
------------------------------------------------------------------------
------------------------------------------------------------------------
-------MLKEG-DIVYGGAPGQSGFYFNKATLDAAGG----------------------------------
------------------------------------------------------------------------
-----------------------------------SRAKLWESLQVL-------------------------
-------------PHEKFGYRSKIQAYRVKR--ET-------------------------------------
------------------------------------------------------------------------

```
--------------------------------------------------------------IAGTGKAISQ
DPTR--------------------------------------------------------------------
--------------FGEGGGTQFFLSNYKTVLE---------------------------------------
------------------------------------------------------------------------
------------------------------------------------------------------------
-------------PIDKPFEIGL-------------------------------------------------
------------------------------------------------------------------------
------------------------------------------------------------------------
------------------------------------------------------------------------
------------------------------------------------------------------------
---------------------------
>AB042_3
---------------------------------MNPYTIFEYTIVFYNQRNKELPNVKYSIIFFCEDGTKKT
YEGATNPKGQTKPIPLNQNGKL-------HIFVEGHETIF---SPKKTIKPILAEGSNIVEVNDIRLKSNTS
FLTKAQYELMQKKTSKDLEELRKNAKAVKQKNFFNLSKIRPTFPLDLAEELNERINMSYEEYRKKNTHIVKK
TKYLKFKNYALYRFVDSAGNGIPIVDYQIFAQGQSRPLINARPGPPDKKGYTQLVYTHLKSRVTYTLGSARK
NSEWYEPITCVDK-----------QTIYQIIFPTSIAVTQPDINHKENMEARQKPPIVINPTTNEVLILPSA
VYAEFDKKTKILSDAVREVHKSNANLIKAIQSRDLDEIKELEKRLNINQEK--AIEKINGEFKQTADLREVW
VVETTGKTNEKISKYNLKRRYLKVTEYEELKAKRRNPQTEVDVTANQYTVQQPAQ-----------------
-----------------------------------IRSSFEKLSQQLL----------TVKGNAGSDEKAVY
NLIGGLGGEIAEEYKNSRDVTVTQEAQWMRM--VAGASGE--------------------------------
--------------------------------------------------------------GSISAAPKGV
SIKTSGDMSAKWTLFEGVKEWRKFYP------------------------------------CETGWKLEYD
NYDLGTIRFLIGAEISGFSGANLGIAGNLSVDISHQGAAQVIKAV----------VRPPERSMSQMVDRNKK
PMFQPAQGSLKI--IGNNNQENAQNQGNISINAFAGVQIQGLLKGAVEWFKPKGDGSGEGE-----------
--------------------------------------FVAIASAAAGGGVSAGVGAQGQFQIGYDQTSGNF
KILVAAHLCWGMGAKGVASFVVGTEHLLSYLGFIKSQVAHAGFKTLLYINEAAFLLAAQVLAYCIGE-----
-------------NHPVISDINRIAASYGDWIRRLDIDQGRYKTAQNINSSSGKKELLYATPETKGILLYAV
THWTDRTAPIFDMNVKFSDMKIEFFPTRKTAVINI-------------------------------------
-------------------------------------FKTCISTEEWENTIQHIHPRGNKLTQTQLGKVEGD
IIRFLNYGNDEKYAEDIIRCLNSGIEYKGTQINAWLQDYLKYRKGAKKIAGSLNYMLVKNQDDNRFKQLEIQ
QGVWGDSEEITLIASNLNVLSPFDQDDTDKYETYNV------------------------------------
---------------------------
```

>ATCC17978-mff_3
---------------------------------MNPYTIFEYTIVFYNQRNKELPNVKYSIIFFCEDGTKKT
YEGATNPKGQTKPIPLNQNGKL-------HIFVEGHETIF---SPKKTIKPILAEGSNIVEVNDIRLKSNTS
FLTKAQYELMQKKTSKDLEELRKNAKAVKQKNFFNLSKIRPTFPLDLAEELNERINMSYEEYRKKNTHIVKK
TKYLKFKNYALYRFVDSAGNGIPIVDYQIFAQGQSRPLINARPGPPDKKGYTQLVYTHLKSRVTYTLGSARK
NSEWYEPITCVDK-----------QTIYQIIFPTSIAVTQPDINHKENMEARQKPPIVINPTTNEVLILPSA
VYAEFDKKTKILSDAVREVHKSNANLIKAIQSRDLDEIKELEKRLNINQEK--AIEKINGEFKQTADLREVW
VVETTGKTNEKISKYNLKRRYLKVTEYEELKAKRRNPQTEVDVTANQYTVQQPAQ-----------------
-----------------------------------IRSSFEKLSQQLL----------TVKGNAGSDEKAVY
NLIGGLGGEIAEEYKNSRDVTVTQEAQWMRM--VAGASGE--------------------------------
--------------------------------------------------------------GSISAAPKGV
SIKTSGDMSAKWTLFEGVKEWRKFYP------------------------------------CETGWKLEYD
NYDLGTIRFLIGAEISGFSGANLGIAGNLSVDISHQGAAQVIKAV----------VRPPERSMSQMVDRNKK
PMFQPAQGSLKI--IGNNNQENAQNQGNISINAFAGVQIQGLLKGAVEWFKPKGDGSGEGE-----------
--------------------------------------FVAIASAAAGGGVSAGVGAQGQFQIGYDQTSGNF
KILVAAHLCWGMGAKGVASFVVGTEHLLSYLGFIKSQVAHAGFKTLLYINEAAFLLAAQVLAYCIGE-----
-------------NHPVISDINRIAASYGDWIRRLDIDQGRYKTAQNINSSSGKKELLYATPETKGILLYAV
THWTDRTAPIFDMNVKFSDMKIEFFPTRKTAVINI-------------------------------------
-------------------------------------FKTCISTEEWENTIQHIHPRGNKLTQTQLGKVEGD
IIRFLNYGNDEKYAEDIIRCLNSGIEYKGTQINAWLQDYLKYRKGAKKIAGSLNYMLVKNQDDNRFKQLEIQ
QGVWGDSEEITLIASNLNVLSPFDQDDTDKYETYNV------------------------------------
---------------------------
>YezP
------------------------------------------------------------------------
------------------------------MLDPKNIQNV-----LKSSTDLGQQSQSDLQKPFV-------
------------------------------------------------------------------------
------------------------------------------------------------------------
------------------------------------------------------------------------
-------------KGMQNMEMSGADAARSIMQSVNQ------------------------------------
------------------------------------------------------------------------
-----------------------------------SCETMMSDMNKIL--------------GGLQDNVDAH
QK-----------MQSEERPKDFERAMSDLR--ST-------------------------------------
------------------------------------------------------------------------

--------------------------------------------------------------LSNGFPEEMK
SMFD--------------------------------------------------------------------
------------------------------------------------------------------------
------------------------------------------------------------------------
------------------------------------------------------------------------
-------------KLPKM------------------------------------------------------
------------------------------------------------------------------------
------------------------------------------------------------------------
------------------------------------------------------------------------
------------------------------------------------------------------------
---------------------------

**Supplementary file S4c**

>AR_0078_15
MAKENTQQKEIKRQPAVELAVFNLNSVTDVADLQMIA--------SQVQLYLQVCGNTTLEQ-IKSKANITT
VANIFALTGSVLDLMLYATDKKTGDAAVQRGALLAANLIGLFS----EPNNEAHARMALRPMFGLMAECLYR
ENGKIK----------------------ETDIKRLGLHLNAMIAGDLENFLKETQAKLSSLLISATTLGVTI
LQSMATPATGI----NAGITTAAGASAEKRDPKLKFTNWAVPLIDLLGKPSQANLTPKIQPNITSRLQQEAT
QAIAALSQTLQQQ---------------------------ANAGQKYTLAWLLQETLKAIQALENKG--NAS
VPINQTGEYERHTKGDTLEFVSLQADAL-NAPPC------------EGADSQSGKSISYSIGAERV---QHA
DFYLP-KIGFSFIRQYNSQMDEFDQSMVGARWMMPFSNMIQQN----------------AQGYLFIDSKGRK
HQLPVSIIFETYEVP-YEGWIIKPLKN---------GELI-----LDFGGEWRS----HFQSFDGGKNYYL-
-VKKMNETSQEEILLEYLLLDHI-----AYLKVINFKLKQAEY-ELKFAF--------NEQVKIIAVFLDDK
A------EPL-------ARYEYDTQGNLIKAIDQNG-HTRTYEYNQFHQLTRYTDRTGRGQNIRYEST----
-----------------------EAKAKAIEEWADDGSFHTKLKWHPRLRQVAVYDAYDVPTYYYF---DLD
GFTYRTRLADGRESWYSRDGKKRITRQIDFDGRETQQEYNDQDQLVKIVQPNGGIFRFAY-NKQGNLVEIKD
PEGSIWKREYDENRNVSKEINPLGHITQYKYNN---DNQLVEVIDAKGGVKKIQYNELGQMISYTDCSGK-S
STWEYDEDGALTAEQTANNKVVQYFYS-------------TKGRDKGQL-------------QSIIYPDGLK
EYFEHDEEGRLLKHTDTKGLVTEYKYNQVGLLEQRIDANRHSVAYQWDKQG-RIQKLINQNQAEYLFGYNPY
GYLIREQAFDGEEKHYSYNE-NGRLFQIRRPNI-------------------LTQFDYYADGQIASKSFTHL
HT----GQKQTEQFDYNLNSQLSRAS------------NEVSQIDLYRNALGQLVREHQHYKIPELKPLTAV
LHYEYDELGNLIKTIRP-DGH---------TLNHLVY--GSGHIYAIGLNNQEVVSFQRDDLHRETTRLLAN
G------LMQTKQYNDVGLLSSQFIQPEQETQDYLQYQA----------------HRKYHYDKNYLLSQV--
--------------------------------EDSRLGKLNYQYDPIGRLI------AAQSLHKTESFNFDP

AGNLIDS------ESVLSP-------------------------AQIKNNLIKSYKGKHYQYDVQGNVTEII
QAGKNL-----KLTWDNQNRLIR-------SDNNGLVTEYGYDVFGRRLYKKTAK-------------ELTL
FGWDGDLM--IWESFKSAQTNYTKHYIYEPDS-FVPLLQAGYKDFIQLIETPDYQEYQTKPYSIYKDPVWNR
NLGKERTALEQFTFYHCDQVGTPQTMTNIRGECVWEILQDT-WGAVSQIKA----LNQDNPFEQNNLRFQGQ
YYDRETELHYNRYRYYEPHSARYVSKDPIGLEGGMNTSSYVSD-PNQWIDPKGL
>A1_15
MAKENTQQKEIKRQPAVELAVFNLNSVTDVADLQMIA--------SQVQLYLQVCGNTTLEQ-IKSKANITT
VANIFALTGSVLDLMLYATDKKTGDAAVQRGALLAANLIGLFS----EPNNEAHARMALRPMFGLMAECLYR
ENGKIK----------------------ETDIKRLGLHLNAMIAGDLENFLKETQAKLSSLLISATTLGVTI
LQSMATPATGI----NAGITTAAGASAEKRDPKLKFTNWAVPLIDLLGKPSQANLTPKIQPNITSRLQQEAT
QAIAALSQTLQQQ---------------------------ANAGQKYTLAWLLQETLKAIQALENKG--NAS
VPINQTGEYERHTKGDTLEFVSLQADAL-NAPPC------------EGADSQSGKSISYSIGAERV---QHA
DFYLP-KIGFSFIRQYNSQMDEFDQSMVGARWMMPFSNMIQQN----------------AQGYLFIDSKGRK
HQLPVSIIFETYEVP-YEGWIIKPLKN---------GELI-----LDFGGEWRS----HFQSFDGGKNYYL-
-VKKMNETSQEEILLEYLLLDHI-----AYLKVINFKLKQAEY-ELKFAF--------NEQVKIIAVFLDDK
A------EPL-------ARYEYDTQGNLIKAIDQNG-HTRTYEYNQFHQLTRYTDRTGRGQNIRYEST----
------------------------EAKAKAIEEWADDGSFHTKLKWHPRLRQVAVYDAYDVPTYYYF---DLD
GFTYRTRLADGRESWYSRDGKKRITRQIDFDGRETQQEYNDQDQLVKIVQPNGGIIRFAY-NKQGNLVEIKD
PEGSIWKREYDENRNVSKEINPLGHITQYKYNN---DNQLVEVIDAKGGVKKIQYNELGQMISYTDCSGK-S
STWEYDEDGALTAEQTANNKVVQYFYS-------------TKGRDKGQL-------------QSIIYPDGLK
EYFEHDEEGRLLKHTDTKGLVTEYKYNQVGLLEQRIDANRHSVAYQWDKQG-RIQKLINQNQAEYLFGYNPY
GYLIREQAFDGEEKHYSYNE-NGRLFQIRRPNI-------------------LTQFDYYADGQIASKSFTHL
HT----GQKQTEQFDYNLNSQLSRAS------------NEVSQIDLYRNALGQLVREHQHYKIPELKPLTAV
LHYEYDELGNLIKTIRP-DGH---------TLNHLVY--GSGHIYAIGLNNQEVVSFQRDDLHRETTRLLAN
G------LMQTKQYNDVGLLSSQFIQPEQETQDYLQYQA----------------HRKYHYDKNYLLSQV--
----------------------------------EDSRLGKLNYQYDPIGRLI------AAQSLHKTESFNFDP
AGNLIDS------ESVLSP-------------------------AQIKNNLIKSYKGKHYQYDVQGNVTEII
QAGKNL-----KLTWDNQNRLIR-------SDNNGLVTEYGYDVFGRRLYKKTAK-------------ELTL
FGWDGDLM--IWESFKSAQTNYTKHYIYEPDS-FVPLLQAGYKDFIQLIETPDYQEYQTKPYSIYKDPVWNR
NLGKERTALEQFTFYHCDQVGTPQTMTNIRGECVWEILQDT-WGAVSQIKA----LNQDNPFEQNNLRFQGQ
YYDRETELHYNRYRYYEPHSARYVSKDPIGLEGGMNTSSYVSD-PNQWIDPKGL
>A388_15
MAKENTQQKEIKRQPAVELAVFNLNSVTDVADLQMIA--------SQVQLYLQVCGNTTLEQ-IKSKANITT

VANIFALTGSVLDLMLYATDKKTGDAAVQRGALLAANLIGLFS----EPNNEAHARMALRPMFGLMAECLYR
ENGKIK----------------------ETDIKRLGLHLNAMIAGDLENFLKETQAKLSSLLISATTLGVTI
LQSMATPATGI----NAGITTAAGASAEKRDPKLKFTNWAVPLIDLLGKPSQANLTPKIQPNITSRLQQEAT
QAIAALSQTLQQQ---------------------------ANAGQKYTLAWLLQETLKAIQALENKG--NAS
VPINQTGEYERHTKGDTLEFVSLQADAL-NAPPC------------EGADSQSGKSISYSIGAERV---QHA
DFYLP-KIGFSFIRQYNSQMDEFDQSMVGARWMMPFSNMIQQN----------------AQGYLFIDSKGRK
HQLPVSIIFETYEVP-YEGWIIKPLKN---------GELI-----LDFGGEWRS----HFQSFDGGKNYYL-
-VKKMNETSQEEILLEYLLLDHI-----AYLKVINFKLKQAEY-ELKFAF--------NEQVKIIAVFLDDK
A------EPL-------ARYEYDTQGNLIKAIDQNG-HTRTYEYNQFHQLTRYTDRTGRGQNIRYEST----
-----------------------EAKAKAIEEWADDGSFHTKLKWHPRLRQVAVYDAYDVPTYYYF---DLD
GFTYRTRLADGRESWYSRDGKKRITRQIDFDGRETQQEYNDQDQLVKIVQPNGGIIRFAY-NKQGNLVEIKD
PEGSIWKREYDENRNVSKEINPLGHITQYKYNN---DNQLVEVIDAKGGVKKIQYNELGQMISYTDCSGK-S
STWEYDEDGALTAEQTANNKVVQYFYS-------------TKGRDKGQL-------------QSIIYPDGLK
EYFEHDEEGRLLKHTDTKGLVTEYKYNQVGLLEQRIDANRHSVAYQWDKQG-RIQKLINQNQAEYLFGYNPY
GYLIREQAFDGEEKHYSYNE-NGRLFQIRRPNI-------------------LTQFDYYADGQIASKSFTHL
HT----GQKQTEQFDYNLNSQLSRAS------------NEVSQIDLYRNALGQLVREHQHYKIPELKPLTAV
LHYEYDELGNLIKTIRP-DGH---------TLNHLVY--GSGHIYAIGLNNQEVVSFQRDDLHRETTRLLAN
G------LMQTKQYNDVGLLSSQFIQPEQETQDYLQYQA----------------HRKYHYDKNYLLSQV--
--------------------------------EDSRLGKLNYQYDPIGRLI------AAQSLHKTESFNFDP
AGNLIDS------ESVLSP-------------------------AQIKNNLIKSYKGKHYQYDVQGNVTEII
QAGKNL-----KLTWDNQNRLIR-------SDNNGLVTEYGYDVFGRRLYKKTAK-------------ELTL
FGWDGDLM--IWESFKSAQTNYTKHYIYEPDS-FVPLLQAGYKDFIQLIETPDYQEYQTKPYSIYKDPVWNR
NLGKERTALEQFTFYHCDQVGTPQTMTNIRGECVWEILQDT-WGAVSQIKA----LNQDNPFEQNNLRFQGQ
YYDRETELHYNRYRYYEPHSARYVSKDPIGLEGGMNTSSYVSD-PNQWIDPKGL
>A85_15
MAKENTQQKEIKRQPAVELAVFNLNSVTDVADLQMIA--------SQVQLYLQVCGNTTLEQ-IKSKANITT
VANIFALTGSVLDLMLYATDKKTGDAAVQRGALLAANLIGLFS----EPNNEAHARMALRPMFGLMAECLYR
ENGKIK----------------------ETDIKRLGLHLNAMIAGDLENFLKETQAKLSSLLISATTLGVTI
LQSMATPATGI----NAGITTAAGASAEKRDPKLKFTNWAVPLIDLLGKPSQANLTPKIQPNITSRLQQEAT
QAIAALSQTLQQQ---------------------------ANAGQKYTLAWLLQETLKAIQALENKG--NAS
VPINQTGEYERHTKGDTLEFVSLQADAL-NAPPC------------EGADSQSGKSISYSIGAERV---QHA
DFYLP-KIGFSFIRQYNSQMDEFDQSMVGARWMMPFSNMIQQN----------------AQGYLFIDSKGRK
HQLPVSIIFETYEVP-YEGWIIKPLKN---------GELI-----LDFGGEWRS----HFQSFDGGKNYYL-

-VKKMNETSQEEILLEYLLLDHI-----AYLKVINFKLKQAEY-ELKFAF--------NEQVKIIAVFLDDK
A------EPL-------ARYEYDTQGNLIKAIDQNG-HTRTYEYNQFHQLTRYTDRTGRGQNIRYEST----
-----------------------EAKAKAIEEWADDGSFHTKLKWHPRLRQVAVYDAYDVPTYYYF---DLD
GFTYRTRLADGRESWYSRDGKKRITRQIDFDGRETQQEYNDQDQLVKIVQPNGGIIRFAY-NKQGNLVEIKD
PEGSIWKREYDENRNVSKEINPLGHITQYKYNN---DNQLVEVIDAKGGVKKIQYNELGQMISYTDCSGK-S
STWEYDEDGALTAEQTANNKVVQYFYS-------------TKGRDKGQL-------------QSIIYPDGLK
EYFEHDEEGRLLKHTDTKGLVTEYKYNQVGLLEQRIDANRHSVAYQWDKQG-RIQKLINQNQAEYLFGYNPY
GYLIREQAFDGEEKHYSYNE-NGRLFQIRRPNI-------------------LTQFDYYADGQIASKSFTHL
HT----GQKQTEQFDYNLNSQLSRAS------------NEVSQIDLYRNALGQLVREHQHYKIPELKPLTAV
LHYEYDELGNLIKTIRP-DGH---------TLNHLVY--GSGHIYAIGLNNQEVVSFQRDDLHRETTRLLAN
G------LMQTKQYNDVGLLSSQFIQPEQETQDYLQYQA----------------HRKYHYDKNYLLSQV--
--------------------------------EDSRLGKLNYQYDPIGRLI------AAQSLHKTESFNFDP
AGNLIDS------ESVLSP-------------------------AQIKNNLIKSYKGKHYQYDVQGNVTEII
QAGKNL-----KLTWDNQNRLIR-------SDNNGLVTEYGYDVFGRRLYKKTAK-------------ELTL
FGWDGDLM--IWESFKSAQTNYTKHYIYEPDS-FVPLLQAGYKDFIQLIETPDYQEYQTKPYSIYKDPVWNR
NLGKERTALEQFTFYHCDQVGTPQTMTNIRGECVWEILQDT-WGAVSQIKA----LNQDNPFEQNNLRFQGQ
YYDRETELHYNRYRYYEPHSARYVSKDPIGLEGGMNTSSYVSD-PNQWIDPKGL
>AB0057_15
MAKENTQQKEIKRQPAVELAVFNLNSVTDVADLQMIA--------SQVQLYLQVCGNTTLEQ-IKSKANITT
VANIFALTGSVLDLMLYATDKKTGDAAVQRGALLAANLIGLFS----EPNNEAHARMALRPMFGLMAECLYR
ENGKIK----------------------ETDIKRLGLHLNAMIAGDLENFLKETQAKLSSLLISATTLGVTI
LQSMATPATGI----NAGITTAAGASAEKRDPKLKFTNWAVPLIDLLGKPSQANLTPKIQPNITSRLQQEAT
QAIAALSQTLQQQ---------------------------ANAGQKYTLAWLLQETLKAIQALENKG--NAS
VPINQTGEYERHTKGDTLEFVSLQADAL-NAPPC------------EGADSQSGKSISYSIGAERV---QHA
DFYLP-KIGFSFIRQYNSQMDEFDQSMVGARWMMPFSNMIQQN----------------AQGYLFIDSKGRK
HQLPVSIIFETYEVP-YEGWIIKPLKN---------GELI-----LDFGGEWRS----HFQSFDGGKNYYL-
-VKKMNETSQEEILLEYLLLDHI-----AYLKVINFKLKQAEY-ELKFAF--------NEQVKIIAVFLDDK
A------EPL-------ARYEYDTQGNLIKAIDQNG-HTRTYEYNQFHQLTRYTDRTGRGQNIRYEST----
-----------------------EAKAKAIEEWADDGSFHTKLKWHPRLRQVAVYDAYDVPTYYYF---DLD
GFTYRTRLADGRESWYSRDGKKRITRQIDFDGRETQQEYNDQDQLVKIVQPNGGIIRFAY-NKQGNLVEIKD
PEGSIWKREYDENRNVSKEINPLGHITQYKYNN---DNQLVEVIDAKGGVKKIQYNELGQMISYTDCSGK-S
STWEYDEDGALTAEQTANNKVVQYFYS-------------TKGRDKGQL-------------QSIIYPDGLK
EYFEHDEEGRLLKHTDTKGLVTEYKYNQVGLLEQRIDANRHSVAYQWDKQG-RIQKLINQNQAEYLFGYNPY

GYLIREQAFDGEEKHYSYNE-NGRLFQIRRPNI-------------------LTQFDYYADGQIASKSFTHL
HT----GQKQTEQFDYNLNSQLSRAS------------NEVSQIDLYRNALGQLVREHQHYKIPELKPLTAV
LHYEYDELGNLIKTIRP-DGH---------TLNHLVY--GSGHIYAIGLNNQEVVSFQRDDLHRETTRLLAN
G------LMQTKQYNDVGLLSSQFIQPEQETQDYLQYQA----------------HRKYHYDKNYLLSQV--
--------------------------------EDSRLGKLNYQYDPIGRLI------AAQSLHKTESFNFDP
AGNLIDS------ESVLSP-------------------------AQIKNNLIKSYKGKHYQYDVQGNVTEII
QAGKNL-----KLTWDNQNRLIR-------SDNNGLVTEYGYDVFGRRLYKKTAK-------------ELTL
FGWDGDLM--IWESFKSAQTNYTKHYIYEPDS-FVPLLQAGYKDFIQLIETPDYQEYQTKPYSIYKDPVWNR
NLGKERTALEQFTFYHCDQVGTPQTMTNIRGECVWEILQDT-WGAVSQIKA----LNQDNPFEQNNLRFQGQ
YYDRETELHYNRYRYYEPHSARYVSKDPIGLEGGMNTSSYVSD-PNQWIDPKGL
>AB307-0294_15
MAKENTQQKEIKRQPAVELAVFNLNSVTDVADLQMIA--------SQVQLYLQVCGNTTLEQ-IKSKANITT
VANIFALTGSVLDLMLYATDKKTGDAAVQRGALLAANLIGLFS----EPNNEAHARMALRPMFGLMAECLYR
ENGKIK----------------------ETDIKRLGLHLNAMIAGDLENFLKETQAKLSSLLISATTLGVTI
LQSMATPATGI----NAGITTAAGASAEKRDPKLKFTNWAVPLIDLLGKPSQANLTPKIQPNITSRLQQEAT
QAIAALSQTLQQQ---------------------------ANAGQKYTLAWLLQETLKAIQALENKG--NAS
VPINQTGEYERHTKGDTLEFVSLQADAL-NAPPC------------EGADSQSGKSISYSIGAERV---QHA
DFYLP-KIGFSFIRQYNSQMDEFDQSMVGARWMMPFSNMIQQN----------------AQGYLFIDSKGRK
HQLPVSIIFETYEVP-YEGWIIKPLKN---------GELI-----LDFGGEWRS----HFQSFDGGKNYYL-
-VKKMNETSQEEILLEYLLLDHI-----AYLKVINFKLKQAEY-ELKFAF--------NEQVKIIAVFLDDK
A------EPL-------ARYEYDTQGNLIKAIDQNG-HTRTYEYNQFHQLTRYTDRTGRGQNIRYEST----
-----------------------EAKAKAIEEWADDGSFHTKLKWHPRLRQVAVYDAYDVPTYYYF---DLD
GFTYRTRLADGRESWYSRDGKKRITRQIDFDGRETQQEYNDQDQLVKIVQPNGGIIRFAY-NKQGNLVEIKD
PEGSIWKREYDENRNVSKEINPLGHITQYKYNN---DNQLVEVIDAKGGVKKIQYNELGQMISYTDCSGK-S
STWEYDEDGALTAEQTANNKVVQYFYS-------------TKGRDKGQL-------------QSIIYPDGLK
EYFEHDEEGRLLKHTDTKGLVTEYKYNQVGLLEQRIDANRHSVAYQWDKQG-RIQKLINQNQAEYLFGYNPY
GYLIREQAFDGEEKHYSYNE-NGRLFQIRRPNI-------------------LTQFDYYADGQIASKSFTHL
HT----GQKQTEQFDYNLNSQLSRAS------------NEVSQIDLYRNALGQLVREHQHYKIPELKPLTAV
LHYEYDELGNLIKTIRP-DGH---------TLNHLVY--GSGHIYAIGLNNQEVVSFQRDDLHRETTRLLAN
G------LMQTKQYNDVGLLSSQFIQPEQETQDYLQYQA----------------HRKYHYDKNYLLSQV--
--------------------------------EDSRLGKLNYQYDPIGRLI------AAQSLHKTESFNFDP
AGNLIDS------ESVLSP-------------------------AQIKNNLIKSYKGKHYQYDVQGNVTEII
QAGKNL-----KLTWDNQNRLIR-------SDNNGLVTEYGYDVFGRRLYKKTAK-------------ELTL

FGWDGDLM--IWESFKSAQTNYTKHYIYEPDS-FVPLLQAGYKDFIQLIETPDYQEYQTKPYSIYKDPVWNR
NLGKERTALEQFTFYHCDQVGTPQTMTNIRGECVWEILQDT-WGAVSQIKA----LNQDNPFEQNNLRFQGQ
YYDRETELHYNRYRYYEPHSARYVSKDPIGLEGGMNTSSYVSD-PNQWIDPKGL
>AR_0083_15
MAKENTQQKEIKRQPAVELAVFNLNSVTDVADLQMIA--------SQVQLYLQVCGNTTLEQ-IKSKANITT
VANIFALTGSVLDLMLYATDKKTGDAAVQRGALLAANLIGLFS----EPNNEAHARMALRPMFGLMAECLYR
ENGKIK----------------------ETDIKRLGLHLNAMIAGDLENFLKETQAKLSSLLISATTLGVTI
LQSMATPATGI----NAGITTAAGASAEKRDPKLKFTNWAVPLIDLLGKPSQANLTPKIQPNITSRLQQEAT
QAIAALSQTLQQQ---------------------------ANAGQKYTLAWLLQETLKAIQALENKG--NAS
VPINQTGEYERHTKGDTLEFVSLQADAL-NAPPC------------EGADSQSGKSISYSIGAERV---QHA
DFYLP-KIGFSFIRQYNSQMDEFDQSMVGARWMMPFSNMIQQN----------------AQGYLFIDSKGRK
HQLPVSIIFETYEVP-YEGWIIKPLKN---------GELI-----LDFGGEWRS----HFQSFDGGKNYYL-
-VKKMNETSQEEILLEYLLLDHI-----AYLKVINFKLKQAEY-ELKFAF--------NEQVKIIAVFLDDK
A------EPL-------ARYEYDTQGNLIKAIDQNG-HTRTYEYNQFHQLTRYTDRTGRGQNIRYEST----
-----------------------EAKAKAIEEWADDGSFHTKLKWHPRLRQVAVYDAYDVPTYYYF---DLD
GFTYRTRLADGRESWYSRDGKKRITRQIDFDGRETQQEYNDQDQLVKIVQPNGGIIRFAY-NKQGNLVEIKD
PEGSIWKREYDENRNVSKEINPLGHITQYKYNN---DNQLVEVIDAKGGVKKIQYNELGQMISYTDCSGK-S
STWEYDEDGALTAEQTANNKVVQYFYS-------------TKGRDKGQL-------------QSIIYPDGLK
EYFEHDEEGRLLKHTDTKGLVTEYKYNQVGLLEQRIDANRHSVAYQWDKQG-RIQKLINQNQAEYLFGYNPY
GYLIREQAFDGEEKHYSYNE-NGRLFQIRRPNI--------------------LTQFDYYADGQIASKSFTHL
HT----GQKQTEQFDYNLNSQLSRAS------------NEVSQIDLYRNALGQLVREHQHYKIPELKPLTAV
LHYEYDELGNLIKTIRP-DGH---------TLNHLVY--GSGHIYAIGLNNQEVVSFQRDDLHRETTRLLAN
G------LMQTKQYNDVGLLSSQFIQPEQETQDYLQYQA----------------HRKYHYDKNYLLSQV--
--------------------------------EDSRLGKLNYQYDPIGRLI------AAQSLHKTESFNFDP
AGNLIDS------ESVLSP-------------------------AQIKNNLIKSYKGKHYQYDVQGNVTEII
QAGKNL-----KLTWDNQNRLIR-------SDNNGLVTEYGYDVFGRRLYKKTAK-------------ELTL
FGWDGDLM--IWESFKSAQTNYTKHYIYEPDS-FVPLLQAGYKDFIQLIETPDYQEYQTKPYSIYKDPVWNR
NLGKERTALEQFTFYHCDQVGTPQTMTNIRGECVWEILQDT-WGAVSQIKA----LNQDNPFEQNNLRFQGQ
YYDRETELHYNRYRYYEPHSARYVSKDPIGLEGGMNTSSYVSD-PNQWIDPKGL
>D36_15
MAKENTQQKEIKRQPAVELAVFNLNSVTDVADLQMIA--------SQVQLYLQVCGNTTLEQ-IKSKANITT
VANIFALTGSVLDLMLYATDKKTGDAAVQRGALLAANLIGLFS----EPNNEAHARMALRPMFGLMAECLYR
ENGKIK----------------------ETDIKRLGLHLNAMIAGDLENFLKETQAKLSSLLISATTLGVTI

LQSMATPATGI----NAGITTAAGASAEKRDPKLKFTNWAVPLIDLLGKPSQANLTPKIQPNITSRLQQEAT
QAIAALSQTLQQQ---------------------------ANAGQKYTLAWLLQETLKAIQALENKG--NAS
VPINQTGEYERHTKGDTLEFVSLQADAL-NAPPC------------EGADSQSGKSISYSIGAERV---QHA
DFYLP-KIGFSFIRQYNSQMDEFDQSMVGARWMMPFSNMIQQN----------------AQGYLFIDSKGRK
HQLPVSIIFETYEVP-YEGWIIKPLKN---------GELI-----LDFGGEWRS----HFQSFDGGKNYYL-
-VKKMNETSQEEILLEYLLLDHI-----AYLKVINFKLKQAEY-ELKFAF--------NEQVKIIAVFLDDK
A------EPL-------ARYEYDTQGNLIKAIDQNG-HTRTYEYNQFHQLTRYTDRTGRGQNIRYEST----
-----------------------EAKAKAIEEWADDGSFHTKLKWHPRLRQVAVYDAYDVPTYYYF---DLD
GFTYRTRLADGRESWYSRDGKKRITRQIDFDGRETQQEYNDQDQLVKIVQPNGGIIRFAY-NKQGNLVEIKD
PEGSIWKREYDENRNVSKEINPLGHITQYKYNN---DNQLVEVIDAKGGVKKIQYNELGQMISYTDCSGK-S
STWEYDEDGALTAEQTANNKVVQYFYS-------------TKGRDKGQL-------------QSIIYPDGLK
EYFEHDEEGRLLKHTDTKGLVTEYKYNQVGLLEQRIDANRHSVAYQWDKQG-RIQKLINQNQAEYLFGYNPY
GYLIREQAFDGEEKHYSYNE-NGRLFQIRRPNI-------------------LTQFDYYADGQIASKSFTHL
HT----GQKQTEQFDYNLNSQLSRAS------------NEVSQIDLYRNALGQLVREHQHYKIPELKPLTAV
LHYEYDELGNLIKTIRP-DGH---------TLNHLVY--GSGHIYAIGLNNQEVVSFQRDDLHRETTRLLAN
G------LMQTKQYNDVGLLSSQFIQPEQETQDYLQYQA----------------HRKYHYDKNYLLSQV--
--------------------------------EDSRLGKLNYQYDPIGRLI------AAQSLHKTESFNFDP
AGNLIDS------ESVLSP-------------------------AQIKNNLIKSYKGKHYQYDVQGNVTEII
QAGKNL-----KLTWDNQNRLIR-------SDNNGLVTEYGYDVFGRRLYKKTAK-------------ELTL
FGWDGDLM--IWESFKSAQTNYTKHYIYEPDS-FVPLLQAGYKDFIQLIETPDYQEYQTKPYSIYKDPVWNR
NLGKERTALEQFTFYHCDQVGTPQTMTNIRGECVWEILQDT-WGAVSQIKA----LNQDNPFEQNNLRFQGQ
YYDRETELHYNRYRYYEPHSARYVSKDPIGLEGGMNTSSYVSD-PNQWIDPKGL
>USA15_15
MAKENTQQKEIKRQPAVELAVFNLNSVTDVADLQMIA--------SQVQLYLQVCGNTTLEQ-IKSKANITT
VANIFALTGSVLDLMLYATDKKTGDAAVQRGALLAANLIGLFS----EPNNEAHARMALRPMFGLMAECLYR
ENGKIK----------------------ETDIKRLGLHLNAMIAGDLENFLKETQAKLSSLLISATTLGVTI
LQSMATPATGI----NAGITTAAGASAEKRDPKLKFTNWAVPLIDLLGKPSQANLTPKIQPNITSRLQQEAT
QAIAALSQTLQQQ---------------------------ANAGQKYTLAWLLQETLKAIQALENKG--NAS
VPINQTGEYERHTKGDTLEFVSLQADAL-NAPPC------------EGADSQSGKSISYSIGAERV---QHA
DFYLP-KIGFSFIRQYNSQMDEFDQSMVGARWMMPFSNMIQQN----------------AQGYLFIDSKGRK
HQLPVSIIFETYEVP-YEGWIIKPLKN---------GELI-----LDFGGEWRS----HFQSFDGGKNYYL-
-VKKMNETSQEEILLEYLLLDHI-----AYLKVINFKLKQAEY-ELKFAF--------NEQVKIIAVFLDDK
A------EPL-------ARYEYDTQGNLIKAIDQNG-HTRTYEYNQFHQLTRYTDRTGRGQNIRYEST----

-----------------------EAKAKAIEEWADDGSFHTKLKWHPRLRQVAVYDAYDVPTYYYF---DLD
GFTYRTRLADGRESWYSRDGKKRITRQIDFDGRETQQEYNDQDQLVKIVQPNGGIIRFAY-NKQGNLVEIKD
PEGSIWKREYDENRNVSKEINPLGHITQYKYNN---DNQLVEVIDAKGGVKKIQYNELGQMISYTDCSGK-S
STWEYDEDGALTAEQTANNKVVQYFYS-------------TKGRDKGQL-------------QSIIYPDGLK
EYFEHDEEGRLLKHTDTKGLVTEYKYNQVGLLEQRIDANRHSVAYQWDKQG-RIQKLINQNQAEYLFGYNPY
GYLIREQAFDGEEKHYSYNE-NGRLFQIRRPNI-------------------LTQFDYYADGQIASKSFTHL
HT----GQKQTEQFDYNLNSQLSRAS------------NEVSQIDLYRNALGQLVREHQHYKIPELKPLTAV
LHYEYDELGNLIKTIRP-DGH---------TLNHLVY--GSGHIYAIGLNNQEVVSFQRDDLHRETTRLLAN
G------LMQTKQYNDVGLLSSQFIQPEQETQDYLQYQA----------------HRKYHYDKNYLLSQV--
--------------------------------EDSRLGKLNYQYDPIGRLI------AAQSLHKTESFNFDP
AGNLIDS------ESVLSP-------------------------AQIKNNLIKSYKGKHYQYDVQGNVTEII
QAGKNL-----KLTWDNQNRLIR-------SDNNGLVTEYGYDVFGRRLYKKTAK-------------ELTL
FGWDGDLM--IWESFKSAQTNYTKHYIYEPDS-FVPLLQAGYKDFIQLIETPDYQEYQTKPYSIYKDPVWNR
NLGKERTALEQFTFYHCDQVGTPQTMTNIRGECVWEILQDT-WGAVSQIKA----LNQDNPFEQNNLRFQGQ
YYDRETELHYNRYRYYEPHSARYVSKDPIGLEGGMNTSSYVSD-PNQWIDPKGL
>WCHAB005078_15
MAKENTQQKEIKRQPAVELAVFNLNSVTDVADLQMIA--------SQVQLYLQVCGNTTLEQ-IKSKANITT
VANIFALTGSVLDLMLYATDKKTGDAAVQRGALLAANLIGLFS----EPNNEAHARMALRPMFGLMAECLYR
ENGKIK----------------------ETDIKRLGLHLNAMIAGDLENFLKETQAKLSSLLISATTLGVTI
LQSMATPATGI----NAGITTAAGASAEKRDPKLKFTNWAVPLIDLLGKPSQANLTPKIQPNITSRLQQEAT
QAIAALSQTLQQQ---------------------------ANAGQKYTLAWLLQETLKAIQALENKG--NAS
VPINQTGEYERHTKGDTLEFVSLQADAL-NAPPC------------EGADSQSGKSISYSIGAERV---QHA
DFYLP-KIGFSFIRQYNSQMDEFDQSMVGARWMMPFSNMIQQN----------------AQGYLFIDSKGRK
HQLPVSIIFETYEVP-YEGWIIKPLKN---------GELI-----LDFGGEWRS----HFQSFDGGKNYYL-
-VKKMNETSQEEILLEYLLLDHI-----AYLKVINFKLKQAEY-ELKFAF--------NEQVKIIAVFLDDK
A------EPL-------ARYEYDTQGNLIKAIDQNG-HTRTYEYNQFHQLTRYTDRTGRGQNIRYEST----
-----------------------EAKAKAIEEWADDGSFHTKLKWHPRLRQVAVYDAYDVPTYYYF---DLD
GFTYRTRLADGRESWYSRDGKKRITRQIDFDGRETQQEYNDQDQLVKIVQPNGGIIRFAY-NKQGNLVEIKD
PEGSIWKREYDENRNVSKEINPLGHITQYKYNN---DNQLVEVIDAKGGVKKIQYNELGQMISYTDCSGK-S
STWEYDEDGALTAEQTANNKVVQYFYS-------------TKGRDKGQL-------------QSIIYPDGLK
EYFEHDEEGRLLKHTDTKGLVTEYKYNQVGLLEQRIDANRHSVAYQWDKQG-RIQKLINQNQAEYLFGYNPY
GYLIREQAFDGEEKHYSYNE-NGRLFQIRRPNI-------------------LTQFDYYADGQIASKSFTHL
HT----GQKQTEQFDYNLNSQLSRAS------------NEVSQIDLYRNALGQLVREHQHYKIPELKPLTAV

LHYEYDELGNLIKTIRP-DGH---------TLNHLVY--GSGHIYAIGLNNQEVVSFQRDDLHRETTRLLAN
G------LMQTKQYNDVGLLSSQFIQPEQETQDYLQYQA----------------HRKYHYDKNYLLSQV--
--------------------------------EDSRLGKLNYQYDPIGRLI------AAQSLHKTESFNFDP
AGNLIDS------ESVLSP-------------------------AQIKNNLIKSYKGKHYQYDVQGNVTEII
QAGKNL-----KLTWDNQNRLIR-------SDNNGLVTEYGYDVFGRRLYKKTAK-------------ELTL
FGWDGDLM--IWESFKSAQTNYTKHYIYEPDS-FVPLLQAGYKDFIQLIETPDYQEYQTKPYSIYKDPVWNR
NLGKERTALEQFTFYHCDQVGTPQTMTNIRGECVWEILQDT-WGAVSQIKA----LNQDNPFEQNNLRFQGQ
YYDRETELHYNRYRYYEPHSARYVSKDPIGLEGGMNTSSYVSD-PNQWIDPKGL
>AB5075-UW_15
MAKENTQQKEIKRQPAVELAVFNLNSVTDVADLQMIA--------SQVQLYLQVCGNTTLEQ-IKSKANITT
VANIFALTGSVLDLMLYATDKKTGDAAVQRGALLAANLIGLFS----EPNNEAHARMALRPMFGLMAECLYR
ENGKIK----------------------ETDIKRLGLHLNAMIAGDLENFLKETQAKLSSLLISATTLGVTI
LQSMATPATGI----NAGITTAAGASAEKRDPKLKFTNWAVPLIDLLGKPSQANLTPKIQPNITSRLQQEAT
QAIAALSQTLQQQ---------------------------ANAGQKYTLAWLLQETLKAIQALENKG--NAS
VPINQTGEYERHTKGDTLEFVSLQADAL-NAPPC------------EGADSQSGKSISYSIGAERV---QHA
DFYLP-KIGFSFIREYNSQMDEFDQSMVGARWMMPFSNMIQQN----------------AQGYLFIDSKGRK
HQLPVSIIFETYEVP-YEGWIIKPLKN---------GELI-----LDFGGEWRS----HFQSFDGGKNYYL-
-VKKMNETSQEEILLEYLLLDHI-----AYLKVINFKLKQAEY-ELKFAF--------NEQVKIIAVFLDDK
A------EPL-------ARYEYDTQGNLIKAIDQNG-HTRTYEYNQFHQLTRYTDRTGRGQNIRYEST----
-----------------------EAKAKAIEEWADDGSFHTKLKWHPRLRQVAVYDAYDVPTYYYF---DLD
GFTYRTRLADGRESWYSRDGKKRITRQIDFDGRETQQEYNDQDQLVKIVQPNGGIIRFAY-NKQGNLVEIKD
PEGSIWKREYDENRNVSKEINPLGHITQYKYNN---DNQLVEVIDAKGGVKKIQYNELGQMISYTDCSGK-S
STWEYDEDGALTAEQTANNKVVQYFYS-------------TKGRDKGQL-------------QSIIYPDGLK
EYFEHDEEGRLLKHTDTKGLVTEYKYNQVGLLEQRIDANRHSVAYQWDKQG-RIQKLINQNQAEYLFGYNPY
GYLIREQAFDGEEKHYSYNE-NGRLFQIRRPNI-------------------LTQFDYYADGQIASKSFTHL
HT----GQKQTEQFDYNLNSQLSRAS------------NEVSQIDLYRNALGQLVREHQHYKIPELKPLTAV
LHYEYDELGNLIKTIRP-DGH---------TLNHLVY--GSGHIYAIGLNNQEVVSFQRDDLHRETTRLLAN
G------LMQTKQYNDVGLLSSQFIQPEQETQDYLQYQA----------------HRKYHYDKNYLLSQV--
--------------------------------EDSRLGKLNYQYDPIGRLI------AAQSLHKTESFNFDP
AGNLIDS------ESVLSP-------------------------AQIKNNLIKSYKGKHYQYDVQGNVTEII
QAGKNL-----KLTWDNQNRLIR-------SDNNGLVTEYGYDVFGRRLYKKTAK-------------ELTL
FGWDGDLM--IWESFKSAQTNYTKHYIYEPDS-FVPLLQAGYKDFIQLIETPDYQEYQTKPYSIYKDPVWNR
NLGKERTALEQFTFYHCDQVGTPQTMTNIRGECVWEILQDT-WGAVSQIKA----LNQDNPFEQNNLRFQGQ

YYDRETELHYNRYRYYEPHSARYVSKDPIGLEGGMNTSSYVSD-PNQWIDPKGL
>AB031_26
MAKENTQQKEIKKQPAVELAVFNLNSVTDVADLQMIA--------SQVQLYLQVCGNTTLEQ-IKSKANITT
VANIFALTGSVLDLMLYATDKKTGDAAVQRGALLAANLIGLFS----EPSNEAHARMALRPMFGLMAECLYR
ENGKIK----------------------ETDIKRLGLHLNAMIAGDLENFLKETQAKLSSLLTSAATLGVTI
LQSMATPATGI----NAGITTAAGASAEKRDPKLKFTNWAVPLIDLLGKPSQANLTPKIQPNITSRLQQEAT
QAIAALSQTLQQQ---------------------------ANAGQKYTLAWLLQETLKAIQALENKG--NAS
VPINQTGEYERHTKGDTLEFVSLQADAL-NAPPC------------EGADSQSGKSISYSIGAERV---QHA
DFYLP-KIGFSFIRQYNSQMDEFEQSMVGARWMMPFSNMIQQT----------------AQGYLFIDSKGRK
HQLPASITLETYEVP-YEGITIQPLKN---------GDLI-----LNFGGDWSF----QFHQFSVGQPYQL-
-IQQFNEETKEKIDLSYLIFEKV-----AYLQHVDFQLEHAKH-QLKFAF--------SEQIKIIAVFLDDN
A------EPL-------ARYDYDTQGNLIKAIDQNG-HTRTYEYNDFHQLTRYIDRTGRGQNIRYEST----
-----------------------DAKAKAIEEWADDGSFHTKLKWHPRLRQVAVYDAYDVPTYYYF---DLN
GFTYRTRLADGRESWYSRDGKKRITRQIDFDGRETQQEYNDQDQLVKIVQPNGGIIRFAY-NEQGNLVEIKD
PEGSIWKREYDENRNVSKEINPLGHITQYKYNN---DNQLVEVIDAKGGVKKIQYNELGQMISYTDCSGK-S
SIWEYDEDGVLTAEQTANNKVVQYFYS-------------TKGRDKGQL-------------QSIIYPDGLK
EYFEHDEEGRLLKHTDTKGLVTEYKYNQVGLLEQRIDANRHSIAYQWDKQG-RIQKLINQNQAEYLFGYNPY
GYLIREQAFDGEEKHYSYNE-NGRLFKIRQPNI-------------------VTEFDYYADGQIASKSFTHL
HT----GQKQTEQFDYNLNSQLSRAS------------NEVSQIDFYRNALGQLIREHQHYKIPELKPLTAV
LHYEYDELGNLIKTIRP-DGH---------TLNHLVY--GSGHIYAIGLNNQEVASFQRDDLHRETTRLLAN
G------LIQTKQYNDVGLLSSQLIQPEQETQDYLQYQA----------------HRHYQYDQNYLLSQV--
---------------------------------EDSRLGKLNYQYDPIGRLI------AAQSLHKTESFNFDP
AGNLIDS------DSVLSP-------------------------AQIKNNLIKSYKGKHYQYDAQGNVTEII
QAGKNL-----KLTWDNQNRLIR-------SDNNGLVTEYGYDVFGRRLYKKTTN-------------ELTL
FGWDGDLM--IWESVKSAQMSYTKHYIYEPNS-FVPLLQAGYKDFIQLIETPDYQEYQTKPYSIYKDPVWNS
TTRKKRADLEQITFYHCDQVGTPQTMTNTRGECVWEILQDT-WGTALEIKV----VNQDNPFEQNNLRFQGQ
YYDSETELHYNRYRYYEPHSARYMSKDPIGLEGGLNTSAYVNN-PTHWIDPMGL
>AB030_15
MAKENTQQKEIKKQPTVELAVFNLNSVTDVADLQMIA--------SQVQLYLQVCGNTTLEQ-IKSKANITT
VANIFALTGSVLDLMLYATDKKTGDAAVQRGALLAANLIGLFS----EPNNEAHARMALRPMFGLMAECLYR
ENGKIK----------------------ETDIKRLGLHLNAMIAGDLENFLKETQAKLSSLLTSATTLGVTI
LQSMATPATGI----NAGITTAAGASAEKRDPKLKFTNWAVPLIDLLGKPSQANLTPKIQPNITSRLQQEAT
QAIAALSQTLQQQ---------------------------ANAGQKYTLAWLLQETLKAIQALENKG--NAS

VPVNQTGEYERHTKGDTLEFVSLQADAL-NAPPC------------EGADSQSGKSISYSIGAERV---QHA
DFYLP-KIGFSFTRQYNSQMDEFDQSMVGARWMMPFSNMIQQN----------------AQGYLFIDSKGRK
HQLPASITFETYEVP-YEGITIQPLKN---------GDLV-----LNFGGDWTF----QFHQFSVGQPYQL-
-IQQFNEKTQEKIDLSYLIFEKV-----AYLQYVVFQLEHAKH-QLKFAF--------NEQVKIMAVFLDDK
A------EPL-------ARYEYDIQGNLIKAIDQNG-YTRTYEYNNFHQLTRYTDRTGRGQNIRYEST----
-----------------------DAKAKAVEEWADDGSFHTKLKWHPRLRQVAVYDAYDVPTHYYF---DLN
GFTYRTRLADGRESWYSRDGQKRITRQIDFEGRETQQEYNDQDQLVKIVQPNGGIIHFAY-NEQGNLVEIKD
PEGSIWKREYDENGNLSKEINPLGHITQYKYNN---DNQLVEVIDAKGGVKKIQYNELGQMISYTDCSGK-S
STWEYDEDGALTAQQTANNKVVQYFYS-------------TKGRDKGQL-------------QSIIYPDGLK
EYFEHDEEGRLLKHIDTKGLVTEYKYNQVGLLEQRIDANRHSVTYQWDKQG-RIQKLINQNQAEYLFGYNPY
GYLILEQAFDGEEKHYSYNE-NGRLFQIRQPNI-------------------LTEFDYYADGQIASKSFTHL
HT----GQKQTEQFDYNLNSQLSRAS------------NEVSQIDFYRNALGQLVREHQHYKIPELKSLTAV
LHYEYDELGNLIKTIRP-DGH---------TLNHLVY--GSGHIYAIGLNNQEVASFQRDDLHRETTRLLAN
G------LIQTKQYNDVGLLSSQLIQPEQETQDYLQYQA----------------HRHYQYDQNYLLSQV--
--------------------------------EDSRLGRLNYQYDPIGRLI------AAQSLHKTESFNFDP
AGNLIDS------DSVLSP-------------------------AQIKNNLIKSYKGKHYQYDAQGNVTEII
QAGKNL-----KLTWDNQNRLIR-------SDNNGLVTEYGYDVFGRRLYKKTTN-------------ELTL
FGWDGDLM--IWESVKSAQMSYTKHYIYEPNS-FVPLLQAGYKDFIQLIETPDYQEYQTKPYSIYKDPVWNS
TTRKKRADLEQITFYHCDQVGTPQTMTDIRGECVWEILQDT-WGAVSQIKA----LNQDNPFEQNNLRFQGQ
YYDQETELHYNRYRYYEPHSARYVSKDPIGLEGGMNTSSYVSD-PNQWIDPKGL
>AbH12O-A2_15
MAKENTQQKEIKKQPTVELAVFNLNSVTDVADLQMIA--------SQVQLYLQVCGNTTLEQ-IKSKANITT
VANIFALTGSVLDLMLYATDKKTGDAAVQRGALLAANLIGLFS----EPNNEAHARMALRPMFGLMAECLYR
ENGKIK----------------------ETDIKRLGLHLNAMIAGDLENFLKETQAKLSSLLTSATTLGVTI
LQSMATPATGI----NAGITTAAGASAEKRDPKLKFTNWAVPLIDLLGKPSQANLTPKIQPNITSRLQQEAT
QAIAALSQTLQQQ---------------------------ANAGQKYTLAWLLQETLKAIQALENKG--NAS
VPVNQTGEYERHTKGDTLEFVSLQADAL-NAPPC------------EGADSQSGKSISYSIGAERV---QHA
DFYL-PKIGFSFTRQYNSQMDEFDQSMVGARWMMPFSNMIQQN----------------AQGYLFIDSKGRK
HQLPASITFETYEVP-YEGITIQPLKN---------GDLV-----LNFGGDWTF----QFHQFSVGQPYQL-
-IQQFNEKTQEKIDLSYLIFEKV-----AYLQYVVFQLEHAKH-QLKFAF--------NEQVKIMAVFLDDK
A------EPL-------ARYEYDIQGNLIKAIDQNG-YTRTYEYNNFHQLTRYTDRTGRGQNIRYEST----
-----------------------DAKAKAVEEWADDGSFHTKLKWHPRLRQVAVYDAYDVPTHYYF---DLN
GFTYRTRLADGRESWYSRDGQKRITRQIDFEGRETQQEYNDQDQLVKIVQPNGGIIHFAY-NEQGNLVEIKD

```
PEGSIWKREYDENGNLSKEINPLGHITQYKYNN---DNQLVEVIDAKGGVKKIQYNELGQMISYTDCSGK-S
STWEYDEDGALTAQQTANNKVVQYFYS-------------TKGRDKGQL-------------QSIIYPDGLK
EYFEHDEEGRLLKHIDTKGLVTEYKYNQVGLLEQRIDANRHSVTYQWDKQG-RIQKLINQNQAEYLFGYNPY
GYLILEQAFDGEEKHYSYNE-NGRLFQIRQPNI-------------------LTEFDYYADGQIASKSFTHL
HT----GQKQTEQFDYNLNSQLSRAS------------NEVSQIDFYRNALGQLVREHQHYKIPELKSLTAV
LHYEYDELGNLIKTIRP-DGH---------TLNHLVY--GSGHIYAIGLNNQEVASFQRDDLHRETTRLLAN
G------LIQTKQYNDVGLLSSQLIQPEQETQDYLQYQA----------------HRHYQYDQNYLLSQV--
--------------------------------EDSRLGRLNYQYDPIGRLI------AAQSLHKTESFNFDP
AGNLIDS------DSVLSP-------------------------AQIKNNLIKSYKGKHYQYDAQGNVTEII
QAGKNL-----KLTWDNQNRLIR-------SDNNGLVTEYGYDVFGRRLYKKTTN-------------ELTL
FGWDGDLM--IWESVKSAQMSYTKHYIYEPNS-FVPLLQAGYKDFIQLIETPDYQEYQTKPYSIYKDPVWNS
TTRKKRADLEQITFYHCDQVGTPQTMTDIRGECVWEILQDT-WGAVSQIKA----LNQDNPFEQNNLRFQGQ
YYDQETELHYNRYRYYEPHSARYVSKDPIGLEGGMNTSSYVSD-PNQWIDPKGL
>AF-401_15
MAKENTQQKEIKKQPTVELAVFNLNSVTDVADLQMIA--------SQVQLYLQVCGNTTLEQ-IKSKANITT
VANIFALTGSVLDLMLYATDKKTGDAAVQRGALLAANLIGLFS----EPNNEAHARMALRPMFGLMAECLYR
ENGKIK----------------------ETDIKRLGLHLNAMIAGDLENFLKETQAKLSSLLTSATTLGVTI
LQSMATPATGI----NAGITTAAGASAEKRDPKLKFTNWAVPLIDLLGKPSQANLTPKIQPNITSRLQQEAT
QAIAALSQTLQQQ---------------------------ANAGQKYTLAWLLQETLKAIQALENKG--NAS
VPVNQTGEYERHTKGDTLEFVSLQADAL-NAPPC------------EGADSQSGKSISYSIGAERV---QHA
DFYLP-KIGFSFTRQYNSQMDEFDQSMVGARWMMPFSNMIQQN----------------AQGYLFIDSKGRK
HQLPASITFETYEVP-YEGITIQPLKN---------GDLV-----LNFGGDWTF----QFHQFSVGQPYQL-
-IQQFNEKTQEKIDLSYLIFEKV-----AYLQYVVFQLEHAKH-QLKFAF--------NEQVKIMAVFLDDK
A------EPL-------ARYEYDIQGNLIKAIDQNG-YTRTYEYNNFHQLTRYTDRTGRGQNIRYEST----
-----------------------DAKAKAVEEWADDGSFHTKLKWHPRLRQVAVYDAYDVPTHYYF---DLN
GFTYRTRLADGRESWYSRDGQKRITRQIDFEGRETQQEYNDQDQLVKIVQPNGGIIHFAY-NEQGNLVEIKD
PEGSIWKREYDENGNLSKEINPLGHITQYKYNN---DNQLVEVIDAKGGVKKIQYNELGQMISYTDCSGK-S
STWEYDEDGALTAQQTANNKVVQYFYS-------------TKGRDKGQL-------------QSIIYPDGLK
EYFEHDEEGRLLKHIDTKGLVTEYKYNQVGLLEQRIDANRHSVTYQWDKQG-RIQKLINQNQAEYLFGYNPY
GYLILEQAFDGEEKHYSYNE-NGRLFQIRQPNI-------------------LTEFDYYADGQIASKSFTHL
HT----GQKQTEQFDYNLNSQLSRAS------------NEVSQIDFYRNALGQLVREHQHYKIPELKSLTAV
LHYEYDELGNLIKTIRP-DGH---------TLNHLVY--GSGHIYAIGLNNQEVASFQRDDLHRETTRLLAN
G------LIQTKQYNDVGLLSSQLIQPEQETQDYLQYQA----------------HRHYQYDQNYLLSQV--
```

--------------------------------EDSRLGRLNYQYDPIGRLI------AAQSLHKTESFNFDP
AGNLIDS------DSVLSP-------------------------AQIKNNLIKSYKGKHYQYDAQGNVTEII
QAGKNL-----KLTWDNQNRLIR-------SDNNGLVTEYGYDVFGRRLYKKTTN-------------ELTL
FGWDGDLM--IWESVKSAQMSYTKHYIYEPNS-FVPLLQAGYKDFIQLIETPDYQEYQTKPYSIYKDPVWNS
TTRKKRADLEQITFYHCDQVGTPQTMTDIRGECVWEILQDT-WGAVSQIKA----LNQDNPFEQNNLRFQGQ
YYDQETELHYNRYRYYEPHSARYVSKDPIGLEGGMNTSSYVSD-PNQWIDPKGL
>AR_0063_15
MAKENTQQKEIKKQPTVELAVFNLNSVTDVADLQMIA--------SQVQLYLQVCGNTTLEQ-IKSKANITT
VANIFALTGSVLDLMLYATDKKTGDAAVQRGALLAANLIGLFS----EPNNEAHARMALRPMFGLMAECLYR
ENGKIK----------------------ETDIKRLGLHLNAMIAGDLENFLKETQAKLSSLLTSATTLGVTI
LQSMATPATGI----NAGITTAAGASAEKRDPKLKFTNWAVPLIDLLGKPSQANLTPKIQPNITSRLQQEAT
QAIAALSQTLQQQ---------------------------ANAGQKYTLAWLLQETLKAIQALENKG--NAS
VPVNQTGEYERHTKGDTLEFVSLQADAL-NAPPC------------EGADSQSGKSISYSIGAERV---QHA
DFYLP-KIGFSFTRQYNSQMDEFDQSMVGARWMMPFSNMIQQN----------------AQGYLFIDSKGRK
HQLPASITFETYEVP-YEGITIQPLKN---------GDLV-----LNFGGDWTF----QFHQFSVGQPYQL-
-IQQFNEKTQEKIDLSYLIFEKV-----AYLQYVVFQLEHAKH-QLKFAF--------NEQVKIMAVFLDDK
A------EPL-------ARYEYDIQGNLIKAIDQNG-YTRTYEYNNFHQLTRYTDRTGRGQNIRYEST----
-----------------------DAKAKAVEEWADDGSFHTKLKWHPRLRQVAVYDAYDVPTHYYF---DLN
GFTYRTRLADGRESWYSRDGQKRITRQIDFEGRETQQEYNDQDQLVKIVQPNGGIIHFAY-NEQGNLVEIKD
PEGSIWKREYDENGNLSKEINPLGHITQYKYNN---DNQLVEVIDAKGGVKKIQYNELGQMISYTDCSGK-S
STWEYDEDGALTAQQTANNKVVQYFYS-------------TKGRDKGQL-------------QSIIYPDGLK
EYFEHDEEGRLLKHIDTKGLVTEYKYNQVGLLEQRIDANRHSVTYQWDKQG-RIQKLINQNQAEYLFGYNPY
GYLILEQAFDGEEKHYSYNE-NGRLFQIRQPNI-------------------LTEFDYYADGQIASKSFTHL
HT----GQKQTEQFDYNLNSQLSRAS------------NEVSQIDFYRNALGQLVREHQHYKIPELKSLTAV
LHYEYDELGNLIKTIRP-DGH---------TLNHLVY--GSGHIYAIGLNNQEVASFQRDDLHRETTRLLAN
G------LIQTKQYNDVGLLSSQLIQPEQETQDYLQYQA----------------HRHYQYDQNYLLSQV--
--------------------------------EDSRLGRLNYQYDPIGRLI------AAQSLHKTESFNFDP
AGNLIDS------DSVLSP-------------------------AQIKNNLIKSYKGKHYQYDAQGNVTEII
QAGKNL-----KLTWDNQNRLIR-------SDNNGLVTEYGYDVFGRRLYKKTTN-------------ELTL
FGWDGDLM--IWESVKSAQMSYTKHYIYEPNS-FVPLLQAGYKDFIQLIETPDYQEYQTKPYSIYKDPVWNS
TTRKKRADLEQITFYHCDQVGTPQTMTDIRGECVWEILQDT-WGAVSQIKA----LNQDNPFEQNNLRFQGQ
YYDQETELHYNRYRYYEPHSARYVSKDPIGLEGGMNTSSYVSD-PNQWIDPKGL
>AR_0101_15

MAKENTQQKEIKKQPTVELAVFNLNSVTDVADLQMIA--------SQVQLYLQVCGNTTLEQ-IKSKANITT
VANIFALTGSVLDLMLYATDKKTGDAAVQRGALLAANLIGLFS----EPNNEAHARMALRPMFGLMAECLYR
ENGKIK----------------------ETDIKRLGLHLNAMIAGDLENFLKETQAKLSSLLTSATTLGVTI
LQSMATPATGI----NAGITTAAGASAEKRDPKLKFTNWAVPLIDLLGKPSQANLTPKIQPNITSRLQQEAT
QAIAALSQTLQQQ---------------------------ANAGQKYTLAWLLQETLKAIQALENKG--NAS
VPVNQTGEYERHTKGDTLEFVSLQADAL-NAPPC------------EGADSQSGKSISYSIGAERV---QHA
DFYLP-KIGFSFTRQYNSQMDEFDQSMVGARWMMPFSNMIQQN----------------AQGYLFIDSKGRK
HQLPASITFETYEVP-YEGITIQPLKN---------GDLV-----LNFGGDWTF----QFHQFSVGQPYQL-
-IQQFNEKTQEKIDLSYLIFEKV-----AYLQYVVFQLEHAKH-QLKFAF--------NEQVKIMAVFLDDK
A------EPL-------ARYEYDIQGNLIKAIDQNG-YTRTYEYNNFHQLTRYTDRTGRGQNIRYEST----
-----------------------DAKAKAVEEWADDGSFHTKLKWHPRLRQVAVYDAYDVPTHYYF---DLN
GFTYRTRLADGRESWYSRDGQKRITRQIDFEGRETQQEYNDQDQLVKIVQPNGGIIHFAY-NEQGNLVEIKD
PEGSIWKREYDENGNLSKEINPLGHITQYKYNN---DNQLVEVIDAKGGVKKIQYNELGQMISYTDCSGK-S
STWEYDEDGALTAQQTANNKVVQYFYS-------------TKGRDKGQL-------------QSIIYPDGLK
EYFEHDEEGRLLKHIDTKGLVTEYKYNQVGLLEQRIDANRHSVTYQWDKQG-RIQKLINQNQAEYLFGYNPY
GYLILEQAFDGEEKHYSYNE-NGRLFQIRQPNI-------------------LTEFDYYADGQIASKSFTHL
HT----GQKQTEQFDYNLNSQLSRAS------------NEVSQIDFYRNALGQLVREHQHYKIPELKSLTAV
LHYEYDELGNLIKTIRP-DGH---------TLNHLVY--GSGHIYAIGLNNQEVASFQRDDLHRETTRLLAN
G------LIQTKQYNDVGLLSSQLIQPEQETQDYLQYQA----------------HRHYQYDQNYLLSQV--
---------------------------------EDSRLGRLNYQYDPIGRLI------AAQSLHKTESFNFDP
AGNLIDS------DSVLSP-------------------------AQIKNNLIKSYKGKHYQYDAQGNVTEII
QAGKNL-----KLTWDNQNRLIR-------SDNNGLVTEYGYDVFGRRLYKKTTN-------------ELTL
FGWDGDLM--IWESVKSAQMSYTKHYIYEPNS-FVPLLQAGYKDFIQLIETPDYQEYQTKPYSIYKDPVWNS
TTRKKRADLEQITFYHCDQVGTPQTMTDIRGECVWEILQDT-WGAVSQIKA----LNQDNPFEQNNLRFQGQ
YYDQETELHYNRYRYYEPHSARYVSKDPIGLEGGMNTSSYVSD-PNQWIDPKGL
>15A34_5
MAKENTQQKEIKKQPAVELAVFNLNSVTDVADLQMVA--------SQVQRYLQVCGNTSLEQ-IKSKANITT
VANIFALTGSVLDLMLYATDKKTGDAAVQRGALLAANLIGLFS----EPNNEAHARMALRPMFGLMAECLYR
ENGKIK----------------------ETDIKRLGLHLNAMIAGDLENFLKETQAKLSSLLTSATTLGVTI
LQSMATPATGI----NAGITTAAGASAEKRDPKLKFTNWAVPLIDLLGKPSQANLTPKIQPNITSRLQQEAT
QAIAALSQTLQQQ---------------------------ANAGQKYTLAWLLQETLKAIQALENKG--NAS
VPVNQTGEYERHTKGDTLEFVSLQADAL-NAPPC------------EGADSQSGKSISYSIGAERV---QHA
DFYLP-KIGFSFTRQYNSQMDEFDQSMVGARWMMPFSNMIQQN----------------AKGYLFIDSKGRK

HQLPASITFETYEVP-YEGITIQPLKN---------GDLV-----LNFGGDWTF----QFHQFSVGQPYQL-
-IQQFNEKTQEKIDLSYLIFEKV-----AYLQYVVFQLEHAKH-QLKFAF--------NEQVKIMAVFLDDK
A------EPL-------ARYEYDIQGNLIKAIDQNG-YTRTYEYNNFHQLTRYTDRTGRGQNIRYEST----
-----------------------DAKAKAVEEWADDGSFHTKLKWHPRLRQVAVYNAYDVPTYYYF---DLN
GFTYRTRLADGRESWYSRDGKKRITRQIDFEGRETQQEYNDQDQLVKIVQPNGGVIRFAY-DEQDNLVEIKD
PEGSIWKREYDENGNLNKEINPLGHITQYKYNN---DNQLVEVIDAKGGVKKIQYNELGQMISYTDCSGK-S
STWEYDEDGVLTAVQTANNKVVQYFYS-------------IKGRDKGQL-------------QSIIYPDGLK
EYFEHDEEGRLLKHTDTKGLVTEYKYNQVGLLEQRIDANRHSVAYQWDKQG-RIQKLINQNQAEYLFGYNPY
GYLIREQAFDGEEKHYSYNE-NGRIFQIRQPNI-------------------LTQFDYYADGQIASKSFTHL
HT----GQKQTEQFDYNLNSQLSRAS------------NEVSQIDFYRNALGQLVREHQHYKIPELKPLTAV
LHYEYDELGNLIKTIRP-DGH---------TLNHLVY--GSGHIYAIGLNNQEVVSFQRDDLHRETTRLLAN
G------LMQTKQYNDVGLLSSQFIQPEQETQDYLQYQA----------------HRKYHYDKNYLLSQV--
--------------------------------EDSRLGKLNYQYDPIGRLI------AAQSLHKTESFNFDP
AGNLIDS------DSVLSP-------------------------AQIKNNLIKSYKGKHYQYDVQGNVTEII
QAGKNL-----KLTWDNQNRLIR-------SDNNGLVTEYGYDVFGRRLYKKTAK-------------ELTL
FGWDGDLM--IWESFKSAQTNYTKHYIYEPDS-FVPLLQTGYKDFIQLIETPDYQEYQTKPYSIYKDPVWNS
TTRKKRADLEQITFYHCDQVGTPQTMTNIRGECVWEILQDT-WGAVSQIKA----LNQDNPFEQNNLRFQGQ
YYDQETELHYNRYRYYEPHSARYVSKDPIGLSGGINTSAYVSD-PTQWIDPRGL
>SSA6_5
MAKENTQQKEIKKQPAVELAVFNLNSVTDVADLQMVA--------SQVQRYLQVCGNTSLEQ-IKSKANITT
VANIFALTGSVLDLMLYATDKKTGDAAVQRGALLAANLIGLFS----EPNNEAHARMALRPMFGLMAECLYR
ENGKIK----------------------ETDIKRLGLHLNAMIAGDLENFLKETQAKLSSLLTSATTLGVTI
LQSMATPATGI----NAGITTAAGASAEKRDPKLKFTNWAVPLIDLLGKPSQANLTPKIQPNITSRLQQEAT
QAIAALSQTLQQQ---------------------------ANAGQKYTLAWLLQETLKAIQALENKG--NAS
VPVNQTGEYERHTKGDTLEFVSLQADAL-NAPPC------------EGADSQSGKSISYSIGAERV---QHA
DFYLP-KIGFSFTRQYNSQMDEFDQSMVGARWMMPFSNMIQQN----------------AKGYLFIDSKGRK
HQLPASITFETYEVP-YEGITIQPLKN---------GDLV-----LNFGGDWTF----QFHQFSVGQPYQL-
-IQQFNEKTQEKIDLSYLIFEKV-----AYLQYVVFQLEHAKH-QLKFAF--------NEQVKIMAVFLDDK
A------EPL-------ARYEYDIQGNLIKAIDQNG-YTRTYEYNNFHQLTRYTDRTGRGQNIRYEST----
-----------------------DAKAKAVEEWADDGSFHTKLKWHPRLRQVAVYNAYDVPTYYYF---DLN
GFTYRTRLADGRESWYSRDGKKRITRQIDFEGRETQQEYNDQDQLVKIVQPNGGVIRFAY-DEQDNLVEIKD
PEGSIWKREYDENGNLNKEINPLGHITQYKYNN---DNQLVEVIDAKGGVKKIQYNELGQMISYTDCSGK-S
STWEYDEDGVLTAVQTANNKVVQYFYS-------------IKGRDKGQL-------------QSIIYPDGLK

EYFEHDEEGRLLKHTDTKGLVTEYKYNQVGLLEQRIDANRHSVAYQWDKQG-RIQKLINQNQAEYLFGYNPY
GYLIREQAFDGEEKHYSYNE-NGRIFQIRQPNI-------------------LTQFDYYADGQIASKSFTHL
HT----GQKQTEQFDYNLNSQLSRAS------------NEVSQIDFYRNALGQLVREHQHYKIPELKPLTAV
LHYEYDELGNLIKTIRP-DGH---------TLNHLVY--GSGHIYAIGLNNQEVVSFQRDDLHRETTRLLAN
G------LMQTKQYNDVGLLSSQFIQPEQETQDYLQYQA----------------HRKYHYDKNYLLSQV--
--------------------------------EDSRLGKLNYQYDPIGRLI------AAQSLHKTESFNFDP
AGNLIDS------DSVLSP-------------------------AQIKNNLIKSYKGKHYQYDVQGNVTEII
QAGKNL-----KLTWDNQNRLIR-------SDNNGLVTEYGYDVFGRRLYKKTAK-------------ELTL
FGWDGDLM--IWESFKSAQTNYTKHYIYEPDS-FVPLLQTGYKDFIQLIETPDYQEYQTKPYSIYKDPVWNS
TTRKKRADLEQITFYHCDQVGTPQTMTNIRGECVWEILQDT-WGAVSQIKA----LNQDNPFEQNNLRFQGQ
YYDQETELHYNRYRYYEPHSARYVSKDPIGLSGGINTSAYVSD-PTQWIDPRGL
>USA2_5
MAKENTQQKEIKKQPAVELAVFNLNSVTDVADLQMVA--------SQVQRYLQVCGNTSLEQ-IKSKANITT
VANIFALTGSVLDLMLYATDKKTGDAAVQRGALLAANLIGLFS----EPNNEAHARMALRPMFGLMAECLYR
ENGKIK----------------------ETDIKRLGLHLNAMIAGDLENFLKETQAKLSSLLTSATTLGVTI
LQSMATPATGI----NAGITTAAGASAEKRDPKLKFTNWAVPLIDLLGKPSQANLTPKIQPNITSRLQQEAT
QAIAALSQTLQQQ---------------------------ANAGQKYTLAWLLQETLKAIQALENKG--NAS
VPVNQTGEYERHTKGDTLEFVSLQADAL-NAPPC------------EGADSQSGKSISYSIGAERV---QHA
DFYLP-KIGFSFTRQYNSQMDEFDQSMVGARWMMPFSNMIQQN----------------AKGYLFIDSKGRK
HQLPASITFETYEVP-YEGITIQPLKN---------GDLV-----LNFGGDWTF----QFHQFSVGQPYQL-
-IQQFNEKTQEKIDLSYLIFEKV-----AYLQYVVFQLEHAKH-QLKFAF--------NEQVKIMAVFLDDK
A------EPL-------ARYEYDIQGNLIKAIDQNG-YTRTYEYNNFHQLTRYTDRTGRGQNIRYEST----
-----------------------DAKAKAVEEWADDGSFHTKLKWHPRLRQVAVYNAYDVPTYYYF---DLN
GFTYRTRLADGRESWYSRDGKKRITRQIDFEGRETQQEYNDQDQLVKIVQPNGGVIRFAY-DEQDNLVEIKD
PEGSIWKREYDENGNLNKEINPLGHITQYKYNN---DNQLVEVIDAKGGVKKIQYNELGQMISYTDCSGK-S
STWEYDEDGVLTAVQTANNKVVQYFYS-------------IKGRDKGQL-------------QSIIYPDGLK
EYFEHDEEGRLLKHTDTKGLVTEYKYNQVGLLEQRIDANRHSVAYQWDKQG-RIQKLINQNQAEYLFGYNPY
GYLIREQAFDGEEKHYSYNE-NGRIFQIRQPNI-------------------LTQFDYYADGQIASKSFTHL
HT----GQKQTEQFDYNLNSQLSRAS------------NEVSQIDFYRNALGQLVREHQHYKIPELKPLTAV
LHYEYDELGNLIKTIRP-DGH---------TLNHLVY--GSGHIYAIGLNNQEVVSFQRDDLHRETTRLLAN
G------LMQTKQYNDVGLLSSQFIQPEQETQDYLQYQA----------------HRKYHYDKNYLLSQV--
--------------------------------EDSRLGKLNYQYDPIGRLI------AAQSLHKTESFNFDP
AGNLIDS------DSVLSP-------------------------AQIKNNLIKSYKGKHYQYDVQGNVTEII

QAGKNL-----KLTWDNQNRLIR-------SDNNGLVTEYGYDVFGRRLYKKTAK-------------ELTL
FGWDGDLM--IWESFKSAQTNYTKHYIYEPDS-FVPLLQTGYKDFIQLIETPDYQEYQTKPYSIYKDPVWNS
TTRKKRADLEQITFYHCDQVGTPQTMTNIRGECVWEILQDT-WGAVSQIKA----LNQDNPFEQNNLRFQGQ
YYDQETELHYNRYRYYEPHSARYVSKDPIGLSGGINTSAYVSD-PTQWIDPRGL
>AF-673_5
MAKENTQQKEIKKQPAVELAVFNLNSVTDVADLQMVA--------SQVQRYLQVCGNTSLEQ-IKSKANITT
VANIFALTGSVLDLMLYATDKKTGDAAVQRGALLAANLIGLFS----EPNNEAHARMALRPMFGLMAECLYR
ENGKIK----------------------ETDIKRLGLHLNAMIAGDLENFLKETQAKLSSLLTSATTLGVTI
LQSMATPATGI----NAGITTAAGASAEKRDPKLKFTNWAVPLIDLLGKPSQANLTPKIQPNITSRLQQEAT
QAIAALSQTLQQQ---------------------------ANAGQKYTLAWLLQETLKAIQALENKG--NAS
VPVNQTGEYERHTKGDTLEFVSLQADAL-NAPPC------------EGADSQSGKSISYSIGAERV---QHA
DFYLP-KIGFSFTRQYNSQMDEFDQSMVGARWMMPFSNMIQQN----------------AKGYLFIDSKGRK
HQLPASITFETYEVP-YEGITIQPLKN---------GDLV-----LNFGGDWTF----QFHQFSVGQPYQL-
-IQQFNEKTQEKIDLSYLIFEKV-----AYLQYVVFQLEHAKH-QLKFAF--------NEQVKIMAVFLDDK
A------EPL-------ARYEYDIQGNLIKAIDQNG-YTRTYEYNNFHQLTRYTDRTGRGQNIRYEST----
-----------------------DAKAKAVEEWADDGSFHTKLKWHPRLRQVAVYNAYDVPTYYYF---DLN
GFTYRTRLADGRESWYSRDGKKRITRQIDFEGRETQQEYNDQDQLVKIVQPNGGVIRFAY-DEQDNLVEIKD
PEGSIWKREYDENGNLNKEINPLGHITQYKYNN---DNQLVEVIDAKGGVKKIQYNELGQMISYTDCSGK-S
STWEYDEDGVLTAEQTANNKVVQYFYS-------------IKGRDKGQL-------------QSIIYPDGLK
EYFEHDEEGRLLKHTDTKGLVTEYKYNQVGLLEQRIDANRHSVAYQWDKQG-RIQKLINQNQAEYLFGYNPY
GYLIREQAFDGEEKHYSYNE-NGRIFQILQPNI-------------------LTQFDYYADGQIASKSFTHL
HT----GQKQTEQFDYNLNSQLSRAS------------NEVSQIDFYRNALGQLVREHQHYKIPELKPLTAV
LHYEYDELGNLIKTIRP-DGH---------TLNHLVY--GSGHIYAIGLNNQEVVSFQRDDLHRETTRLLAN
G------LMQTKQYNDVGLLSSQFIQPEQETQDYLQYQA----------------HRKYHYDKNYLLSQV--
--------------------------------EDSRLGKLNYQYDPIGRLI------AAQSLHKTESFNFDP
AGNLIDS------DSVLSP-------------------------AQIKNNLIKSYKGKHYQYDVQGNVTEII
QAGKNL-----KLTWDNQNRLIR-------SDNNGLVTEYGYDVFGRRLYKKTAK-------------ELTL
FGWDGDLM--IWESFKSAQTNYTKHYIYEPDS-FVPLLQTGYKDFIQLIETPDYQEYQTKPYSIYKDPVWNS
TTRKKRADLEQITFYHCDQVGTPQTMTNIRGECVWEILQDT-WGAVSQIKA----LNQDNPFEQNNLRFQGQ
YYDQETELHYNRYRYYEPHSARYVSKDPIGLSGGINTSAYVSD-PTQWIDPRGL
>CMC-CR-MDR-Ab4_5
MAKENTQQKEIKKQPAVELAVFNLNSVTDVADLQMVA--------SQVQRYLQVCGNTSLEQ-IKSKANITT
VANIFALTGSVLDLMLYATDKKTGDAAVQRGALLAANLIGLFS----EPNNEAHARMALRPMFGLMAECLYR

ENGKIK----------------------ETDIKRLGLHLNAMIAGDLENFLKETQAKLSSLLTSATTLGVTI
LQSMATPATGI----NAGITTAAGASAEKRDPKLKFTNWAVPLIDLLGKPSQANLTPKIQPNITSRLQQEAT
QAIAALSQTLQQQ---------------------------ANAGQKYTLAWLLQETLKAIQALENKG--NAS
VPVNQTGEYERHTKGDTLEFVSLQADAL-NAPPC------------EGADSQSGKSISYSIGAERV---QHA
DFYLP-KIGFSFTRQYNSQMDEFDQSMVGARWMMPFSNMIQQN----------------AKGYLFIDSKGRK
HQLPASITFETYEVP-YEGITIQPLKN---------GDLV-----LNFGGDWTF----QFHQFSVGQPYQL-
-IQQFNEKTQEKIDLSYLIFEKV-----AYLQYVVFQLEHAKH-QLKFAF--------NEQVKIMAVFLDDK
A------EPL-------ARYEYDIQGNLIKAIDQNG-YTRTYEYNNFHQLTRYTDRTGRGQNIRYEST----
-----------------------DAKAKAVEEWADDGSFHTKLKWHPRLRQVAVYNAYDVPTYYYF---DLN
GFTYRTRLADGRESWYSRDGKKRITRQIDFEGRETQQEYNDQDQLVKIVQPNGGVIRFAY-DEQDNLVEIKD
PEGSIWKREYDENGNLNKEINPLGHITQYKYNN---DNQLVEVIDAKGGVKKIQYNELGQMISYTDCSGK-S
STWEYDEDGVLTAEQTANNKVVQYFYS-------------IKGRDKGQL-------------QSIIYPDGLK
EYFEHDEEGRLLKHTDTKGLVTEYKYNQVGLLEQRIDANRHSVAYQWDKQG-RIQKLINQNQAEYLFGYNPY
GYLIREQAFDGEEKHYSYNE-NGRIFQILQPNI-------------------LTQFDYYADGQIASKSFTHL
HT----GQKQTEQFDYNLNSQLSRAS------------NEVSQIDFYRNALGQLVREHQHYKIPELKPLTAV
LHYEYDELGNLIKTIRP-DGH---------TLNHLVY--GSGHIYAIGLNNQEVVSFQRDDLHRETTRLLAN
G------LMQTKQYNDVGLLSSQFIQPEQETQDYLQYQA----------------HRKYHYDKNYLLSQV--
--------------------------------EDSRLGKLNYQYDPIGRLI------AAQSLHKTESFNFDP
AGNLIDS------DSVLSP-------------------------AQIKNNLIKSYKGKHYQYDVQGNVTEII
QAGKNL-----KLTWDNQNRLIR-------SDNNGLVTEYGYDVFGRRLYKKTAK-------------ELTL
FGWDGDLM--IWESFKSAQTNYTKHYIYEPDS-FVPLLQTGYKDFIQLIETPDYQEYQTKPYSIYKDPVWNS
TTRKKRADLEQITFYHCDQVGTPQTMTNIRGECVWEILQDT-WGAVSQIKA----LNQDNPFEQNNLRFQGQ
YYDQETELHYNRYRYYEPHSARYVSKDPIGLSGGINTSAYVSD-PTQWIDPRGL
>CMC-CR-MDR-Ab66_5
MAKENTQQKEIKKQPAVELAVFNLNSVTDVADLQMVA--------SQVQRYLQVCGNTSLEQ-IKSKANITT
VANIFALTGSVLDLMLYATDKKTGDAAVQRGALLAANLIGLFS----EPNNEAHARMALRPMFGLMAECLYR
ENGKIK----------------------ETDIKRLGLHLNAMIAGDLENFLKETQAKLSSLLTSATTLGVTI
LQSMATPATGI----NAGITTAAGASAEKRDPKLKFTNWAVPLIDLLGKPSQANLTPKIQPNITSRLQQEAT
QAIAALSQTLQQQ---------------------------ANAGQKYTLAWLLQETLKAIQALENKG--NAS
VPVNQTGEYERHTKGDTLEFVSLQADAL-NAPPC------------EGADSQSGKSISYSIGAERV---QHA
DFYLP-KIGFSFTRQYNSQMDEFDQSMVGARWMMPFSNMIQQN----------------AKGYLFIDSKGRK
HQLPASITFETYEVP-YEGITIQPLKN---------GDLV-----LNFGGDWTF----QFHQFSVGQPYQL-
-IQQFNEKTQEKIDLSYLIFEKV-----AYLQYVVFQLEHAKH-QLKFAF--------NEQVKIMAVFLDDK

A------EPL-------ARYEYDIQGNLIKAIDQNG-YTRTYEYNNFHQLTRYTDRTGRGQNIRYEST----
-----------------------DAKAKAVEEWADDGSFHTKLKWHPRLRQVAVYNAYDVPTYYYF---DLN
GFTYRTRLADGRESWYSRDGKKRITRQIDFEGRETQQEYNDQDQLVKIVQPNGGVIRFAY-DEQDNLVEIKD
PEGSIWKREYDENGNLNKEINPLGHITQYKYNN---DNQLVEVIDAKGGVKKIQYNELGQMISYTDCSGK-S
STWEYDEDGVLTAEQTANNKVVQYFYS-------------IKGRDKGQL-------------QSIIYPDGLK
EYFEHDEEGRLLKHTDTKGLVTEYKYNQVGLLEQRIDANRHSVAYQWDKQG-RIQKLINQNQAEYLFGYNPY
GYLIREQAFDGEEKHYSYNE-NGRIFQILQPNI-------------------LTQFDYYADGQIASKSFTHL
HT----GQKQTEQFDYNLNSQLSRAS------------NEVSQIDFYRNALGQLVREHQHYKIPELKPLTAV
LHYEYDELGNLIKTIRP-DGH---------TLNHLVY--GSGHIYAIGLNNQEVVSFQRDDLHRETTRLLAN
G------LMQTKQYNDVGLLSSQFIQPEQETQDYLQYQA----------------HRKYHYDKNYLLSQV--
--------------------------------EDSRLGKLNYQYDPIGRLI------AAQSLHKTESFNFDP
AGNLIDS------DSVLSP-------------------------AQIKNNLIKSYKGKHYQYDVQGNVTEII
QAGKNL-----KLTWDNQNRLIR-------SDNNGLVTEYGYDVFGRRLYKKTAK-------------ELTL
FGWDGDLM--IWESFKSAQTNYTKHYIYEPDS-FVPLLQTGYKDFIQLIETPDYQEYQTKPYSIYKDPVWNS
TTRKKRADLEQITFYHCDQVGTPQTMTNIRGECVWEILQDT-WGAVSQIKA----LNQDNPFEQNNLRFQGQ
YYDQETELHYNRYRYYEPHSARYVSKDPIGLSGGINTSAYVSD-PTQWIDPRGL
>CMC-MDR-Ab59_5
MAKENTQQKEIKKQPAVELAVFNLNSVTDVADLQMVA--------SQVQRYLQVCGNTSLEQ-IKSKANITT
VANIFALTGSVLDLMLYATDKKTGDAAVQRGALLAANLIGLFS----EPNNEAHARMALRPMFGLMAECLYR
ENGKIK----------------------ETDIKRLGLHLNAMIAGDLENFLKETQAKLSSLLTSATTLGVTI
LQSMATPATGI----NAGITTAAGASAEKRDPKLKFTNWAVPLIDLLGKPSQANLTPKIQPNITSRLQQEAT
QAIAALSQTLQQQ---------------------------ANAGQKYTLAWLLQETLKAIQALENKG--NAS
VPVNQTGEYERHTKGDTLEFVSLQADAL-NAPPC------------EGADSQSGKSISYSIGAERV---QHA
DFYLP-KIGFSFTRQYNSQMDEFDQSMVGARWMMPFSNMIQQN----------------AKGYLFIDSKGRK
HQLPASITFETYEVP-YEGITIQPLKN---------GDLV-----LNFGGDWTF----QFHQFSVGQPYQL-
-IQQFNEKTQEKIDLSYLIFEKV-----AYLQYVVFQLEHAKH-QLKFAF--------NEQVKIMAVFLDDK
A------EPL-------ARYEYDIQGNLIKAIDQNG-YTRTYEYNNFHQLTRYTDRTGRGQNIRYEST----
-----------------------DAKAKAVEEWADDGSFHTKLKWHPRLRQVAVYNAYDVPTYYYF---DLN
GFTYRTRLADGRESWYSRDGKKRITRQIDFEGRETQQEYNDQDQLVKIVQPNGGVIRFAY-DEQDNLVEIKD
PEGSIWKREYDENGNLNKEINPLGHITQYKYNN---DNQLVEVIDAKGGVKKIQYNELGQMISYTDCSGK-S
STWEYDEDGVLTAEQTANNKVVQYFYS-------------IKGRDKGQL-------------QSIIYPDGLK
EYFEHDEEGRLLKHTDTKGLVTEYKYNQVGLLEQRIDANRHSVAYQWDKQG-RIQKLINQNQAEYLFGYNPY
GYLIREQAFDGEEKHYSYNE-NGRIFQILQPNI-------------------LTQFDYYADGQIASKSFTHL

```
HT----GQKQTEQFDYNLNSQLSRAS------------NEVSQIDFYRNALGQLVREHQHYKIPELKPLTAV
LHYEYDELGNLIKTIRP-DGH---------TLNHLVY--GSGHIYAIGLNNQEVVSFQRDDLHRETTRLLAN
G------LMQTKQYNDVGLLSSQFIQPEQETQDYLQYQA----------------HRKYHYDKNYLLSQV--
--------------------------------EDSRLGKLNYQYDPIGRLI------AAQSLHKTESFNFDP
AGNLIDS------DSVLSP-------------------------AQIKNNLIKSYKGKHYQYDVQGNVTEII
QAGKNL-----KLTWDNQNRLIR-------SDNNGLVTEYGYDVFGRRLYKKTAK-------------ELTL
FGWDGDLM--IWESFKSAQTNYTKHYIYEPDS-FVPLLQTGYKDFIQLIETPDYQEYQTKPYSIYKDPVWNS
TTRKKRADLEQITFYHCDQVGTPQTMTNIRGECVWEILQDT-WGAVSQIKA----LNQDNPFEQNNLRFQGQ
YYDQETELHYNRYRYYEPHSARYVSKDPIGLSGGINTSAYVSD-PTQWIDPRGL
>BJAB0868_5
MAKENTQQKEIKKQPAVELAVFNLNSVTDVADLQMVA--------SQVQRYLQVCGNTSLEQ-IKSKANITT
VANIFALTGSVLDLMLYATDKKTGDAAVQRGALLAANLIGLFS----EPNNEAHARMALRPMFGLMAECLYR
ENGKIK----------------------ETDIKRLGLHLNAMIAGDLENFLKETQAKLSSLLTSATTLGVTI
LQSMATPATGI----NAGITTAAGASAEKRDPKLKFTNWAVPLIDLLGKPSQANLTPKIQPNITSRLQQEAT
QAIAALSQTLQQQ---------------------------ANAGQKYTLAWLLQETLKAIQALENKG--NAS
VPVNQTGEYERHTKGDTLEFVSLQADAL-NAPPC------------EGADSQSGKSISYSIGAERV---QHA
DFYLP-KIGFSFTRQYNSQMDEFDQSMVGARWMMPFSNMIQQN----------------AKGYLFIDSKGRK
HQLPASITFETYEVP-YEGITIQPLKN---------GDLV-----LNFGGDWTF----QFHQFSVGQPYQL-
-IQQFNEKTQEKIDLSYLIFEKV-----AYLQYVVFQLEHAKH-QLKFAF--------NEQVKIMAGFLDDK
A------EPL-------ARYEYDIQGNLIKAIDQNG-YTRTYEYNNFHQLTRYTDRTGRGQNIRYEST----
-----------------------DAKAKAVEEWADDGSFHTKLKWHPRLRQVAVYNAYDVPTYYYF---DLN
GFTYRTRLADGRESWYSRDGKKRITRQIDFEGRETQQEYNDQDQLVKIVQPNGGVIRFAY-DEQDNLVEIKD
PEGSIWKREYDENGNLNKEINPLGHITQYKYNN---DNQLVEVIDAKGGVKKIQYNELGQMISYTDCSGK-S
STWEYDEDGVLTAEQTANNKVVQYFYS-------------IKGRDKGQL-------------QSIIYPDGLK
EYFEHDEEGRLLKHTDTKGLVTEYKYNQVGLLEQRIDANRHSVAYQWDKQG-RIQKLINQNQAEYLFGYNPY
GYLIREQAFDGEEKHYSYNE-NGRIFQIRQPNI-------------------LTQFDYYADGQIASKSFTHL
HT----GQKQTEQFDYNLNSQLSRAS------------NEVSQIDFYRNALGQLVREHQHYKIPELKPLTAV
LHYEYDELGNLIKTIRP-DGH---------TLNHLVY--GSGHIYAIGLNNQEVVSFQRDDLHRETTRLLAN
G------LMQTKQYNDVGLLSSQFIQPEQETQDYLQYQA----------------HRKYHYDKNYLLSQV--
--------------------------------EDSRLGKLNYQYDPIGRLI------AAQSLHKTESFNFDP
AGNLIDS------DSVLSP-------------------------AQIKNNLIKSYKGKHYQYDVQGNVTEII
QAGKNL-----KLTWDNQNRLIR-------SDNNGLVTEYGYDVFGRRLYKKTAK-------------ELTL
FGWDGDLM--IWESFKSAQTNYTKHYIYEPDS-FVPLLQTGYKDFIQLIETPDYQEYQTKPYSIYKDPVWNS
```

TTRKKRADLEQITFYHCDQVGTPQTMTNIRGECVWEILQDT-WGAVSQIKA----LNQDNPFEQNNLRFQGQ
YYDQETELHYNRYRYYEPHSARYVSKDPIGLSGGINTSAYVSD-PTQWIDPRGL
>15A5_5
MAKENTQQKEIKKQPAVELAVFNLNSVTDVADLQMVA--------SQVQRYLQVCGNTSLEQ-IKSKANITT
VANIFALTGSVLDLMLYATDKKTGDAAVQRGALLAANLIGLFS----EPNNEAHARMALRPMFGLMAECLYR
ENGKIK----------------------ETDIKRLGLHLNAMIAGDLENFLKETQAKLSSLLTSATTLGVTI
LQSMATPATGI----NAGITTAAGASAEKRDPKLKFTNWAVPLIDLLGKPSQANLTPKIQPNITSRLQQEAT
QAIAALSQTLQQQ--------------------------ANAGQKYTLAWLLQETLKAIQALENKG--NAS
VPVNQTGEYERHTKGDTLEFVSLQADAL-NAPPC------------EGADSQSGKSISYSIGAERV---QHA
DFYLP-KIGFSFTRQYNSQMDEFDQSMVGARWMMPFSNMIQQN----------------AKGYLFIDSKGRK
HQLPASITFETYEVP-YEGITIQPLKN---------GDLV-----LNFGGDWTF----QFHQFSVGQPYQL-
-IQQFNEKTQEKIDLSYLIFEKV-----AYLQYVVFQLEHAKH-QLKFAF--------NEQVKIMAVFLDDK
A------EPL-------ARYEYDIQGNLIKAIDQNG-YTRTYEYNNFHQLTRYTDRTGRGQNIRYEST----
------------------------DAKAKAVEEWADDGSFHTKLKWHPRLRQVAVYNAYDVPTYYYF---DLN
GFTYRTRLADGRESWYSRDGKKRITRQIDFEGRETQQEYNDQDQLVKIVQPNGGVIRFAY-DEQDNLVEIKD
PEGSIWKREYDENGNLNKEINPLGHITQYKYNN---DNQLVEVIDAKGGVKKIQYNELGQMISYTDCSGK-S
STWEYDEDGVLTAEQTANNKVVQYFYS-------------IKGRDKGQL-------------QSIIYPDGLK
EYFEHDEEGRLLKHTDTKGLVTEYKYNQVGLLEQRIDANRHSVAYQWDKQG-RIQKLINQNQAEYLFGYNPY
GYLIREQAFDGEEKHYSYNE-NGRIFQIRQPNI-------------------LTQFDYYADGQIASKSFTHL
HT----GQKQTEQFDYNLNSQLSRAS------------NEVSQIDFYRNALGQLVREHQHYKIPELKPLTAV
LHYEYDELGNLIKTIRP-DGH---------TLNHLVY--GSGHIYAIGLNNQEVVSFQRDDLHRETTRLLAN
G------LMQTKQYNDVGLLSSQFIQPEQETQDYLQYQA----------------HRKYHYDKNYLLSQV--
---------------------------------EDSRLGKLNYQYDPIGRLI------AAQSLHKTESFNFDP
AGNLIDS------DSVLSP-------------------------AQIKNNLIKSYKGKHYQYDVQGNVTEII
QAGKNL-----KLTWDNQNRLIR-------SDNNGLVTEYGYDVFGRRLYKKTAK-------------ELTL
FGWDGDLM--IWESFKSAQTNYTKHYIYEPDS-FVPLLQTGYKDFIQLIETPDYQEYQTKPYSIYKDPVWNS
TTRKKRADLEQITFYHCDQVGTPQTMTNIRGECVWEILQDT-WGAVSQIKA----LNQDNPFEQNNLRFQGQ
YYDQETELHYNRYRYYEPHSARYVSKDPIGLSGGINTSAYVSD-PTQWIDPRGL
>3027STDY5784958_5
MAKENTQQKEIKKQPAVELAVFNLNSVTDVADLQMVA--------SQVQRYLQVCGNTSLEQ-IKSKANITT
VANIFALTGSVLDLMLYATDKKTGDAAVQRGALLAANLIGLFS----EPNNEAHARMALRPMFGLMAECLYR
ENGKIK----------------------ETDIKRLGLHLNAMIAGDLENFLKETQAKLSSLLTSATTLGVTI
LQSMATPATGI----NAGITTAAGASAEKRDPKLKFTNWAVPLIDLLGKPSQANLTPKIQPNITSRLQQEAT

QAIAALSQTLQQQ---------------------------ANAGQKYTLAWLLQETLKAIQALENKG--NAS
VPVNQTGEYERHTKGDTLEFVSLQADAL-NAPPC------------EGADSQSGKSISYSIGAERV---QHA
DFYLP-KIGFSFTRQYNSQMDEFDQSMVGARWMMPFSNMIQQN----------------AKGYLFIDSKGRK
HQLPASITFETYEVP-YEGITIQPLKN---------GDLV-----LNFGGDWTF----QFHQFSVGQPYQL-
-IQQFNEKTQEKIDLSYLIFEKV-----AYLQYVVFQLEHAKH-QLKFAF--------NEQVKIMAVFLDDK
A------EPL-------ARYEYDIQGNLIKAIDQNG-YTRTYEYNNFHQLTRYTDRTGRGQNIRYEST----
-----------------------DAKAKAVEEWADDGSFHTKLKWHPRLRQVAVYNAYDVPTYYYF---DLN
GFTYRTRLADGRESWYSRDGKKRITRQIDFEGRETQQEYNDQDQLVKIVQPNGGVIRFAY-DEQDNLVEIKD
PEGSIWKREYDENGNLNKEINPLGHITQYKYNN---DNQLVEVIDAKGGVKKIQYNELGQMISYTDCSGK-S
STWEYDEDGVLTAEQTANNKVVQYFYS-------------IKGRDKGQL-------------QSIIYPDGLK
EYFEHDEEGRLLKHTDTKGLVTEYKYNQVGLLEQRIDANRHSVAYQWDKQG-RIQKLINQNQAEYLFGYNPY
GYLIREQAFDGEEKHYSYNE-NGRIFQIRQPNI-------------------LTQFDYYADGQIASKSFTHL
HT----GQKQTEQFDYNLNSQLSRAS------------NEVSQIDFYRNALGQLVREHQHYKIPELKPLTAV
LHYEYDELGNLIKTIRP-DGH---------TLNHLVY--GSGHIYAIGLNNQEVVSFQRDDLHRETTRLLAN
G------LMQTKQYNDVGLLSSQFIQPEQETQDYLQYQA----------------HRKYHYDKNYLLSQV--
--------------------------------EDSRLGKLNYQYDPIGRLI------AAQSLHKTESFNFDP
AGNLIDS------DSVLSP-------------------------AQIKNNLIKSYKGKHYQYDVQGNVTEII
QAGKNL-----KLTWDNQNRLIR-------SDNNGLVTEYGYDVFGRRLYKKTAK-------------ELTL
FGWDGDLM--IWESFKSAQTNYTKHYIYEPDS-FVPLLQTGYKDFIQLIETPDYQEYQTKPYSIYKDPVWNS
TTRKKRADLEQITFYHCDQVGTPQTMTNIRGECVWEILQDT-WGAVSQIKA----LNQDNPFEQNNLRFQGQ
YYDQETELHYNRYRYYEPHSARYVSKDPIGLSGGINTSAYVSD-PTQWIDPRGL
>Ab4568_5
MAKENTQQKEIKKQPAVELAVFNLNSVTDVADLQMVA--------SQVQRYLQVCGNTSLEQ-IKSKANITT
VANIFALTGSVLDLMLYATDKKTGDAAVQRGALLAANLIGLFS----EPNNEAHARMALRPMFGLMAECLYR
ENGKIK----------------------ETDIKRLGLHLNAMIAGDLENFLKETQAKLSSLLTSATTLGVTI
LQSMATPATGI----NAGITTAAGASAEKRDPKLKFTNWAVPLIDLLGKPSQANLTPKIQPNITSRLQQEAT
QAIAALSQTLQQQ---------------------------ANAGQKYTLAWLLQETLKAIQALENKG--NAS
VPVNQTGEYERHTKGDTLEFVSLQADAL-NAPPC------------EGADSQSGKSISYSIGAERV---QHA
DFYLP-KIGFSFTRQYNSQMDEFDQSMVGARWMMPFSNMIQQN----------------AKGYLFIDSKGRK
HQLPASITFETYEVP-YEGITIQPLKN---------GDLV-----LNFGGDWTF----QFHQFSVGQPYQL-
-IQQFNEKTQEKIDLSYLIFEKV-----AYLQYVVFQLEHAKH-QLKFAF--------NEQVKIMAVFLDDK
A------EPL-------ARYEYDIQGNLIKAIDQNG-YTRTYEYNNFHQLTRYTDRTGRGQNIRYEST----
-----------------------DAKAKAVEEWADDGSFHTKLKWHPRLRQVAVYNAYDVPTYYYF---DLN

GFTYRTRLADGRESWYSRDGKKRITRQIDFEGRETQQEYNDQDQLVKIVQPNGGVIRFAY-DEQDNLVEIKD
PEGSIWKREYDENGNLNKEINPLGHITQYKYNN---DNQLVEVIDAKGGVKKIQYNELGQMISYTDCSGK-S
STWEYDEDGVLTAEQTANNKVVQYFYS-------------IKGRDKGQL-------------QSIIYPDGLK
EYFEHDEEGRLLKHTDTKGLVTEYKYNQVGLLEQRIDANRHSVAYQWDKQG-RIQKLINQNQAEYLFGYNPY
GYLIREQAFDGEEKHYSYNE-NGRIFQIRQPNI-------------------LTQFDYYADGQIASKSFTHL
HT----GQKQTEQFDYNLNSQLSRAS------------NEVSQIDFYRNALGQLVREHQHYKIPELKPLTAV
LHYEYDELGNLIKTIRP-DGH---------TLNHLVY--GSGHIYAIGLNNQEVVSFQRDDLHRETTRLLAN
G------LMQTKQYNDVGLLSSQFIQPEQETQDYLQYQA----------------HRKYHYDKNYLLSQV--
--------------------------------EDSRLGKLNYQYDPIGRLI------AAQSLHKTESFNFDP
AGNLIDS------DSVLSP------------------------AQIKNNLIKSYKGKHYQYDVQGNVTEII
QAGKNL-----KLTWDNQNRLIR-------SDNNGLVTEYGYDVFGRRLYKKTAK-------------ELTL
FGWDGDLM--IWESFKSAQTNYTKHYIYEPDS-FVPLLQTGYKDFIQLIETPDYQEYQTKPYSIYKDPVWNS
TTRKKRADLEQITFYHCDQVGTPQTMTNIRGECVWEILQDT-WGAVSQIKA----LNQDNPFEQNNLRFQGQ
YYDQETELHYNRYRYYEPHSARYVSKDPIGLSGGINTSAYVSD-PTQWIDPRGL
>Ab4653_5
MAKENTQQKEIKKQPAVELAVFNLNSVTDVADLQMVA--------SQVQRYLQVCGNTSLEQ-IKSKANITT
VANIFALTGSVLDLMLYATDKKTGDAAVQRGALLAANLIGLFS----EPNNEAHARMALRPMFGLMAECLYR
ENGKIK----------------------ETDIKRLGLHLNAMIAGDLENFLKETQAKLSSLLTSATTLGVTI
LQSMATPATGI----NAGITTAAGASAEKRDPKLKFTNWAVPLIDLLGKPSQANLTPKIQPNITSRLQQEAT
QAIAALSQTLQQQ---------------------------ANAGQKYTLAWLLQETLKAIQALENKG--NAS
VPVNQTGEYERHTKGDTLEFVSLQADAL-NAPPC------------EGADSQSGKSISYSIGAERV---QHA
DFYLP-KIGFSFTRQYNSQMDEFDQSMVGARWMMPFSNMIQQN----------------AKGYLFIDSKGRK
HQLPASITFETYEVP-YEGITIQPLKN---------GDLV-----LNFGGDWTF----QFHQFSVGQPYQL-
-IQQFNEKTQEKIDLSYLIFEKV-----AYLQYVVFQLEHAKH-QLKFAF--------NEQVKIMAVFLDDK
A------EPL-------ARYEYDIQGNLIKAIDQNG-YTRTYEYNNFHQLTRYTDRTGRGQNIRYEST----
-----------------------DAKAKAVEEWADDGSFHTKLKWHPRLRQVAVYNAYDVPTYYYF---DLN
GFTYRTRLADGRESWYSRDGKKRITRQIDFEGRETQQEYNDQDQLVKIVQPNGGVIRFAY-DEQDNLVEIKD
PEGSIWKREYDENGNLNKEINPLGHITQYKYNN---DNQLVEVIDAKGGVKKIQYNELGQMISYTDCSGK-S
STWEYDEDGVLTAEQTANNKVVQYFYS-------------IKGRDKGQL-------------QSIIYPDGLK
EYFEHDEEGRLLKHTDTKGLVTEYKYNQVGLLEQRIDANRHSVAYQWDKQG-RIQKLINQNQAEYLFGYNPY
GYLIREQAFDGEEKHYSYNE-NGRIFQIRQPNI-------------------LTQFDYYADGQIASKSFTHL
HT----GQKQTEQFDYNLNSQLSRAS------------NEVSQIDFYRNALGQLVREHQHYKIPELKPLTAV
LHYEYDELGNLIKTIRP-DGH---------TLNHLVY--GSGHIYAIGLNNQEVVSFQRDDLHRETTRLLAN

G------LMQTKQYNDVGLLSSQFIQPEQETQDYLQYQA----------------HRKYHYDKNYLLSQV--
--------------------------------EDSRLGKLNYQYDPIGRLI------AAQSLHKTESFNFDP
AGNLIDS------DSVLSP-------------------------AQIKNNLIKSYKGKHYQYDVQGNVTEII
QAGKNL-----KLTWDNQNRLIR-------SDNNGLVTEYGYDVFGRRLYKKTAK-------------ELTL
FGWDGDLM--IWESFKSAQTNYTKHYIYEPDS-FVPLLQTGYKDFIQLIETPDYQEYQTKPYSIYKDPVWNS
TTRKKRADLEQITFYHCDQVGTPQTMTNIRGECVWEILQDT-WGAVSQIKA----LNQDNPFEQNNLRFQGQ
YYDQETELHYNRYRYYEPHSARYVSKDPIGLSGGINTSAYVSD-PTQWIDPRGL
>Ab4977_5
MAKENTQQKEIKKQPAVELAVFNLNSVTDVADLQMVA--------SQVQRYLQVCGNTSLEQ-IKSKANITT
VANIFALTGSVLDLMLYATDKKTGDAAVQRGALLAANLIGLFS----EPNNEAHARMALRPMFGLMAECLYR
ENGKIK----------------------ETDIKRLGLHLNAMIAGDLENFLKETQAKLSSLLTSATTLGVTI
LQSMATPATGI----NAGITTAAGASAEKRDPKLKFTNWAVPLIDLLGKPSQANLTPKIQPNITSRLQQEAT
QAIAALSQTLQQQ---------------------------ANAGQKYTLAWLLQETLKAIQALENKG--NAS
VPVNQTGEYERHTKGDTLEFVSLQADAL-NAPPC------------EGADSQSGKSISYSIGAERV---QHA
DFYLP-KIGFSFTRQYNSQMDEFDQSMVGARWMMPFSNMIQQN----------------AKGYLFIDSKGRK
HQLPASITFETYEVP-YEGITIQPLKN---------GDLV-----LNFGGDWTF----QFHQFSVGQPYQL-
-IQQFNEKTQEKIDLSYLIFEKV-----AYLQYVVFQLEHAKH-QLKFAF--------NEQVKIMAVFLDDK
A------EPL-------ARYEYDIQGNLIKAIDQNG-YTRTYEYNNFHQLTRYTDRTGRGQNIRYEST----
-----------------------DAKAKAVEEWADDGSFHTKLKWHPRLRQVAVYNAYDVPTYYYF---DLN
GFTYRTRLADGRESWYSRDGKKRITRQIDFEGRETQQEYNDQDQLVKIVQPNGGVIRFAY-DEQDNLVEIKD
PEGSIWKREYDENGNLNKEINPLGHITQYKYNN---DNQLVEVIDAKGGVKKIQYNELGQMISYTDCSGK-S
STWEYDEDGVLTAEQTANNKVVQYFYS-------------IKGRDKGQL-------------QSIIYPDGLK
EYFEHDEEGRLLKHTDTKGLVTEYKYNQVGLLEQRIDANRHSVAYQWDKQG-RIQKLINQNQAEYLFGYNPY
GYLIREQAFDGEEKHYSYNE-NGRIFQIRQPNI-------------------LTQFDYYADGQIASKSFTHL
HT----GQKQTEQFDYNLNSQLSRAS------------NEVSQIDFYRNALGQLVREHQHYKIPELKPLTAV
LHYEYDELGNLIKTIRP-DGH---------TLNHLVY--GSGHIYAIGLNNQEVVSFQRDDLHRETTRLLAN
G------LMQTKQYNDVGLLSSQFIQPEQETQDYLQYQA----------------HRKYHYDKNYLLSQV--
--------------------------------EDSRLGKLNYQYDPIGRLI------AAQSLHKTESFNFDP
AGNLIDS------DSVLSP-------------------------AQIKNNLIKSYKGKHYQYDVQGNVTEII
QAGKNL-----KLTWDNQNRLIR-------SDNNGLVTEYGYDVFGRRLYKKTAK-------------ELTL
FGWDGDLM--IWESFKSAQTNYTKHYIYEPDS-FVPLLQTGYKDFIQLIETPDYQEYQTKPYSIYKDPVWNS
TTRKKRADLEQITFYHCDQVGTPQTMTNIRGECVWEILQDT-WGAVSQIKA----LNQDNPFEQNNLRFQGQ
YYDQETELHYNRYRYYEPHSARYVSKDPIGLSGGINTSAYVSD-PTQWIDPRGL

>ACICU_5
MAKENTQQKEIKKQPAVELAVFNLNSVTDVADLQMVA--------SQVQRYLQVCGNTSLEQ-IKSKANITT
VANIFALTGSVLDLMLYATDKKTGDAAVQRGALLAANLIGLFS----EPNNEAHARMALRPMFGLMAECLYR
ENGKIK----------------------ETDIKRLGLHLNAMIAGDLENFLKETQAKLSSLLTSATTLGVTI
LQSMATPATGI----NAGITTAAGASAEKRDPKLKFTNWAVPLIDLLGKPSQANLTPKIQPNITSRLQQEAT
QAIAALSQTLQQQ---------------------------ANAGQKYTLAWLLQETLKAIQALENKG--NAS
VPVNQTGEYERHTKGDTLEFVSLQADAL-NAPPC------------EGADSQSGKSISYSIGAERV---QHA
DFYLP-KIGFSFTRQYNSQMDEFDQSMVGARWMMPFSNMIQQN----------------AKGYLFIDSKGRK
HQLPASITFETYEVP-YEGITIQPLKN---------GDLV-----LNFGGDWTF----QFHQFSVGQPYQL-
-IQQFNEKTQEKIDLSYLIFEKV-----AYLQYVVFQLEHAKH-QLKFAF--------NEQVKIMAVFLDDK
A------EPL-------ARYEYDIQGNLIKAIDQNG-YTRTYEYNNFHQLTRYTDRTGRGQNIRYEST----
-----------------------DAKAKAVEEWADDGSFHTKLKWHPRLRQVAVYNAYDVPTYYYF---DLN
GFTYRTRLADGRESWYSRDGKKRITRQIDFEGRETQQEYNDQDQLVKIVQPNGGVIRFAY-DEQDNLVEIKD
PEGSIWKREYDENGNLNKEINPLGHITQYKYNN---DNQLVEVIDAKGGVKKIQYNELGQMISYTDCSGK-S
STWEYDEDGVLTAEQTANNKVVQYFYS-------------IKGRDKGQL-------------QSIIYPDGLK
EYFEHDEEGRLLKHTDTKGLVTEYKYNQVGLLEQRIDANRHSVAYQWDKQG-RIQKLINQNQAEYLFGYNPY
GYLIREQAFDGEEKHYSYNE-NGRIFQIRQPNI-------------------LTQFDYYADGQIASKSFTHL
HT----GQKQTEQFDYNLNSQLSRAS------------NEVSQIDFYRNALGQLVREHQHYKIPELKPLTAV
LHYEYDELGNLIKTIRP-DGH---------TLNHLVY--GSGHIYAIGLNNQEVVSFQRDDLHRETTRLLAN
G------LMQTKQYNDVGLLSSQFIQPEQETQDYLQYQA----------------HRKYHYDKNYLLSQV--
--------------------------------EDSRLGKLNYQYDPIGRLI------AAQSLHKTESFNFDP
AGNLIDS------DSVLSP-------------------------AQIKNNLIKSYKGKHYQYDVQGNVTEII
QAGKNL-----KLTWDNQNRLIR-------SDNNGLVTEYGYDVFGRRLYKKTAK-------------ELTL
FGWDGDLM--IWESFKSAQTNYTKHYIYEPDS-FVPLLQTGYKDFIQLIETPDYQEYQTKPYSIYKDPVWNS
TTRKKRADLEQITFYHCDQVGTPQTMTNIRGECVWEILQDT-WGAVSQIKA----LNQDNPFEQNNLRFQGQ
YYDQETELHYNRYRYYEPHSARYVSKDPIGLSGGINTSAYVSD-PTQWIDPRGL
>AR_0056_5
MAKENTQQKEIKKQPAVELAVFNLNSVTDVADLQMVA--------SQVQRYLQVCGNTSLEQ-IKSKANITT
VANIFALTGSVLDLMLYATDKKTGDAAVQRGALLAANLIGLFS----EPNNEAHARMALRPMFGLMAECLYR
ENGKIK----------------------ETDIKRLGLHLNAMIAGDLENFLKETQAKLSSLLTSATTLGVTI
LQSMATPATGI----NAGITTAAGASAEKRDPKLKFTNWAVPLIDLLGKPSQANLTPKIQPNITSRLQQEAT
QAIAALSQTLQQQ---------------------------ANAGQKYTLAWLLQETLKAIQALENKG--NAS
VPVNQTGEYERHTKGDTLEFVSLQADAL-NAPPC------------EGADSQSGKSISYSIGAERV---QHA

DFYLP-KIGFSFTRQYNSQMDEFDQSMVGARWMMPFSNMIQQN----------------AKGYLFIDSKGRK
HQLPASITFETYEVP-YEGITIQPLKN---------GDLV-----LNFGGDWTF----QFHQFSVGQPYQL-
-IQQFNEKTQEKIDLSYLIFEKV-----AYLQYVVFQLEHAKH-QLKFAF--------NEQVKIMAVFLDDK
A------EPL-------ARYEYDIQGNLIKAIDQNG-YTRTYEYNNFHQLTRYTDRTGRGQNIRYEST----
-----------------------DAKAKAVEEWADDGSFHTKLKWHPRLRQVAVYNAYDVPTYYYF---DLN
GFTYRTRLADGRESWYSRDGKKRITRQIDFEGRETQQEYNDQDQLVKIVQPNGGVIRFAY-DEQDNLVEIKD
PEGSIWKREYDENGNLNKEINPLGHITQYKYNN---DNQLVEVIDAKGGVKKIQYNELGQMISYTDCSGK-S
STWEYDEDGVLTAEQTANNKVVQYFYS-------------IKGRDKGQL-------------QSIIYPDGLK
EYFEHDEEGRLLKHTDTKGLVTEYKYNQVGLLEQRIDANRHSVAYQWDKQG-RIQKLINQNQAEYLFGYNPY
GYLIREQAFDGEEKHYSYNE-NGRIFQIRQPNI-------------------LTQFDYYADGQIASKSFTHL
HT----GQKQTEQFDYNLNSQLSRAS------------NEVSQIDFYRNALGQLVREHQHYKIPELKPLTAV
LHYEYDELGNLIKTIRP-DGH---------TLNHLVY--GSGHIYAIGLNNQEVVSFQRDDLHRETTRLLAN
G------LMQTKQYNDVGLLSSQFIQPEQETQDYLQYQA----------------HRKYHYDKNYLLSQV--
--------------------------------EDSRLGKLNYQYDPIGRLI------AAQSLHKTESFNFDP
AGNLIDS------DSVLSP-------------------------AQIKNNLIKSYKGKHYQYDVQGNVTEII
QAGKNL-----KLTWDNQNRLIR-------SDNNGLVTEYGYDVFGRRLYKKTAK-------------ELTL
FGWDGDLM--IWESFKSAQTNYTKHYIYEPDS-FVPLLQTGYKDFIQLIETPDYQEYQTKPYSIYKDPVWNS
TTRKKRADLEQITFYHCDQVGTPQTMTNIRGECVWEILQDT-WGAVSQIKA----LNQDNPFEQNNLRFQGQ
YYDQETELHYNRYRYYEPHSARYVSKDPIGLSGGINTSAYVSD-PTQWIDPRGL
>AR_0102_5
MAKENTQQKEIKKQPAVELAVFNLNSVTDVADLQMVA--------SQVQRYLQVCGNTSLEQ-IKSKANITT
VANIFALTGSVLDLMLYATDKKTGDAAVQRGALLAANLIGLFS----EPNNEAHARMALRPMFGLMAECLYR
ENGKIK----------------------ETDIKRLGLHLNAMIAGDLENFLKETQAKLSSLLTSATTLGVTI
LQSMATPATGI----NAGITTAAGASAEKRDPKLKFTNWAVPLIDLLGKPSQANLTPKIQPNITSRLQQEAT
QAIAALSQTLQQQ---------------------------ANAGQKYTLAWLLQETLKAIQALENKG--NAS
VPVNQTGEYERHTKGDTLEFVSLQADAL-NAPPC------------EGADSQSGKSISYSIGAERV---QHA
DFYLP-KIGFSFTRQYNSQMDEFDQSMVGARWMMPFSNMIQQN----------------AKGYLFIDSKGRK
HQLPASITFETYEVP-YEGITIQPLKN---------GDLV-----LNFGGDWTF----QFHQFSVGQPYQL-
-IQQFNEKTQEKIDLSYLIFEKV-----AYLQYVVFQLEHAKH-QLKFAF--------NEQVKIMAVFLDDK
A------EPL-------ARYEYDIQGNLIKAIDQNG-YTRTYEYNNFHQLTRYTDRTGRGQNIRYEST----
-----------------------DAKAKAVEEWADDGSFHTKLKWHPRLRQVAVYNAYDVPTYYYF---DLN
GFTYRTRLADGRESWYSRDGKKRITRQIDFEGRETQQEYNDQDQLVKIVQPNGGVIRFAY-DEQDNLVEIKD
PEGSIWKREYDENGNLNKEINPLGHITQYKYNN---DNQLVEVIDAKGGVKKIQYNELGQMISYTDCSGK-S

STWEYDEDGVLTAEQTANNKVVQYFYS-------------IKGRDKGQL-------------QSIIYPDGLK
EYFEHDEEGRLLKHTDTKGLVTEYKYNQVGLLEQRIDANRHSVAYQWDKQG-RIQKLINQNQAEYLFGYNPY
GYLIREQAFDGEEKHYSYNE-NGRIFQIRQPNI-------------------LTQFDYYADGQIASKSFTHL
HT----GQKQTEQFDYNLNSQLSRAS------------NEVSQIDFYRNALGQLVREHQHYKIPELKPLTAV
LHYEYDELGNLIKTIRP-DGH---------TLNHLVY--GSGHIYAIGLNNQEVVSFQRDDLHRETTRLLAN
G------LMQTKQYNDVGLLSSQFIQPEQETQDYLQYQA----------------HRKYHYDKNYLLSQV--
--------------------------------EDSRLGKLNYQYDPIGRLI------AAQSLHKTESFNFDP
AGNLIDS------DSVLSP-------------------------AQIKNNLIKSYKGKHYQYDVQGNVTEII
QAGKNL-----KLTWDNQNRLIR-------SDNNGLVTEYGYDVFGRRLYKKTAK-------------ELTL
FGWDGDLM--IWESFKSAQTNYTKHYIYEPDS-FVPLLQTGYKDFIQLIETPDYQEYQTKPYSIYKDPVWNS
TTRKKRADLEQITFYHCDQVGTPQTMTNIRGECVWEILQDT-WGAVSQIKA----LNQDNPFEQNNLRFQGQ
YYDQETELHYNRYRYYEPHSARYVSKDPIGLSGGINTSAYVSD-PTQWIDPRGL
>BJAB07104_5
MAKENTQQKEIKKQPAVELAVFNLNSVTDVADLQMVA--------SQVQRYLQVCGNTSLEQ-IKSKANITT
VANIFALTGSVLDLMLYATDKKTGDAAVQRGALLAANLIGLFS----EPNNEAHARMALRPMFGLMAECLYR
ENGKIK----------------------ETDIKRLGLHLNAMIAGDLENFLKETQAKLSSLLTSATTLGVTI
LQSMATPATGI----NAGITTAAGASAEKRDPKLKFTNWAVPLIDLLGKPSQANLTPKIQPNITSRLQQEAT
QAIAALSQTLQQQ---------------------------ANAGQKYTLAWLLQETLKAIQALENKG--NAS
VPVNQTGEYERHTKGDTLEFVSLQADAL-NAPPC------------EGADSQSGKSISYSIGAERV---QHA
DFYLP-KIGFSFTRQYNSQMDEFDQSMVGARWMMPFSNMIQQN----------------AKGYLFIDSKGRK
HQLPASITFETYEVP-YEGITIQPLKN---------GDLV-----LNFGGDWTF----QFHQFSVGQPYQL-
-IQQFNEKTQEKIDLSYLIFEKV-----AYLQYVVFQLEHAKH-QLKFAF--------NEQVKIMAVFLDDK
A------EPL-------ARYEYDIQGNLIKAIDQNG-YTRTYEYNNFHQLTRYTDRTGRGQNIRYEST----
-----------------------DAKAKAVEEWADDGSFHTKLKWHPRLRQVAVYNAYDVPTYYYF---DLN
GFTYRTRLADGRESWYSRDGKKRITRQIDFEGRETQQEYNDQDQLVKIVQPNGGVIRFAY-DEQDNLVEIKD
PEGSIWKREYDENGNLNKEINPLGHITQYKYNN---DNQLVEVIDAKGGVKKIQYNELGQMISYTDCSGK-S
STWEYDEDGVLTAEQTANNKVVQYFYS-------------IKGRDKGQL-------------QSIIYPDGLK
EYFEHDEEGRLLKHTDTKGLVTEYKYNQVGLLEQRIDANRHSVAYQWDKQG-RIQKLINQNQAEYLFGYNPY
GYLIREQAFDGEEKHYSYNE-NGRIFQIRQPNI-------------------LTQFDYYADGQIASKSFTHL
HT----GQKQTEQFDYNLNSQLSRAS------------NEVSQIDFYRNALGQLVREHQHYKIPELKPLTAV
LHYEYDELGNLIKTIRP-DGH---------TLNHLVY--GSGHIYAIGLNNQEVVSFQRDDLHRETTRLLAN
G------LMQTKQYNDVGLLSSQFIQPEQETQDYLQYQA----------------HRKYHYDKNYLLSQV--
--------------------------------EDSRLGKLNYQYDPIGRLI------AAQSLHKTESFNFDP

AGNLIDS------DSVLSP-------------------------AQIKNNLIKSYKGKHYQYDVQGNVTEII
QAGKNL-----KLTWDNQNRLIR-------SDNNGLVTEYGYDVFGRRLYKKTAK-------------ELTL
FGWDGDLM--IWESFKSAQTNYTKHYIYEPDS-FVPLLQTGYKDFIQLIETPDYQEYQTKPYSIYKDPVWNS
TTRKKRADLEQITFYHCDQVGTPQTMTNIRGECVWEILQDT-WGAVSQIKA----LNQDNPFEQNNLRFQGQ
YYDQETELHYNRYRYYEPHSARYVSKDPIGLSGGINTSAYVSD-PTQWIDPRGL
>HRAB-85_5
MAKENTQQKEIKKQPAVELAVFNLNSVTDVADLQMVA--------SQVQRYLQVCGNTSLEQ-IKSKANITT
VANIFALTGSVLDLMLYATDKKTGDAAVQRGALLAANLIGLFS----EPNNEAHARMALRPMFGLMAECLYR
ENGKIK----------------------ETDIKRLGLHLNAMIAGDLENFLKETQAKLSSLLTSATTLGVTI
LQSMATPATGI----NAGITTAAGASAEKRDPKLKFTNWAVPLIDLLGKPSQANLTPKIQPNITSRLQQEAT
QAIAALSQTLQQQ---------------------------ANAGQKYTLAWLLQETLKAIQALENKG--NAS
VPVNQTGEYERHTKGDTLEFVSLQADAL-NAPPC------------EGADSQSGKSISYSIGAERV---QHA
DFYLP-KIGFSFTRQYNSQMDEFDQSMVGARWMMPFSNMIQQN----------------AKGYLFIDSKGRK
HQLPASITFETYEVP-YEGITIQPLKN---------GDLV-----LNFGGDWTF----QFHQFSVGQPYQL-
-IQQFNEKTQEKIDLSYLIFEKV-----AYLQYVVFQLEHAKH-QLKFAF--------NEQVKIMAVFLDDK
A------EPL-------ARYEYDIQGNLIKAIDQNG-YTRTYEYNNFHQLTRYTDRTGRGQNIRYEST----
-----------------------DAKAKAVEEWADDGSFHTKLKWHPRLRQVAVYNAYDVPTYYYF---DLN
GFTYRTRLADGRESWYSRDGKKRITRQIDFEGRETQQEYNDQDQLVKIVQPNGGVIRFAY-DEQDNLVEIKD
PEGSIWKREYDENGNLNKEINPLGHITQYKYNN---DNQLVEVIDAKGGVKKIQYNELGQMISYTDCSGK-S
STWEYDEDGVLTAEQTANNKVVQYFYS-------------IKGRDKGQL-------------QSIIYPDGLK
EYFEHDEEGRLLKHTDTKGLVTEYKYNQVGLLEQRIDANRHSVAYQWDKQG-RIQKLINQNQAEYLFGYNPY
GYLIREQAFDGEEKHYSYNE-NGRIFQIRQPNI-------------------LTQFDYYADGQIASKSFTHL
HT----GQKQTEQFDYNLNSQLSRAS------------NEVSQIDFYRNALGQLVREHQHYKIPELKPLTAV
LHYEYDELGNLIKTIRP-DGH---------TLNHLVY--GSGHIYAIGLNNQEVVSFQRDDLHRETTRLLAN
G------LMQTKQYNDVGLLSSQFIQPEQETQDYLQYQA----------------HRKYHYDKNYLLSQV--
---------------------------------EDSRLGKLNYQYDPIGRLI------AAQSLHKTESFNFDP
AGNLIDS------DSVLSP-------------------------AQIKNNLIKSYKGKHYQYDVQGNVTEII
QAGKNL-----KLTWDNQNRLIR-------SDNNGLVTEYGYDVFGRRLYKKTAK-------------ELTL
FGWDGDLM--IWESFKSAQTNYTKHYIYEPDS-FVPLLQTGYKDFIQLIETPDYQEYQTKPYSIYKDPVWNS
TTRKKRADLEQITFYHCDQVGTPQTMTNIRGECVWEILQDT-WGAVSQIKA----LNQDNPFEQNNLRFQGQ
YYDQETELHYNRYRYYEPHSARYVSKDPIGLSGGINTSAYVSD-PTQWIDPRGL
>KBN10P02143_5
MAKENTQQKEIKKQPAVELAVFNLNSVTDVADLQMVA--------SQVQRYLQVCGNTSLEQ-IKSKANITT

VANIFALTGSVLDLMLYATDKKTGDAAVQRGALLAANLIGLFS----EPNNEAHARMALRPMFGLMAECLYR
ENGKIK----------------------ETDIKRLGLHLNAMIAGDLENFLKETQAKLSSLLTSATTLGVTI
LQSMATPATGI----NAGITTAAGASAEKRDPKLKFTNWAVPLIDLLGKPSQANLTPKIQPNITSRLQQEAT
QAIAALSQTLQQQ---------------------------ANAGQKYTLAWLLQETLKAIQALENKG--NAS
VPVNQTGEYERHTKGDTLEFVSLQADAL-NAPPC------------EGADSQSGKSISYSIGAERV---QHA
DFYLP-KIGFSFTRQYNSQMDEFDQSMVGARWMMPFSNMIQQN----------------AKGYLFIDSKGRK
HQLPASITFETYEVP-YEGITIQPLKN---------GDLV-----LNFGGDWTF----QFHQFSVGQPYQL-
-IQQFNEKTQEKIDLSYLIFEKV-----AYLQYVVFQLEHAKH-QLKFAF--------NEQVKIMAVFLDDK
A------EPL-------ARYEYDIQGNLIKAIDQNG-YTRTYEYNNFHQLTRYTDRTGRGQNIRYEST----
-----------------------DAKAKAVEEWADDGSFHTKLKWHPRLRQVAVYNAYDVPTYYYF---DLN
GFTYRTRLADGRESWYSRDGKKRITRQIDFEGRETQQEYNDQDQLVKIVQPNGGVIRFAY-DEQDNLVEIKD
PEGSIWKREYDENGNLNKEINPLGHITQYKYNN---DNQLVEVIDAKGGVKKIQYNELGQMISYTDCSGK-S
STWEYDEDGVLTAEQTANNKVVQYFYS-------------IKGRDKGQL-------------QSIIYPDGLK
EYFEHDEEGRLLKHTDTKGLVTEYKYNQVGLLEQRIDANRHSVAYQWDKQG-RIQKLINQNQAEYLFGYNPY
GYLIREQAFDGEEKHYSYNE-NGRIFQIRQPNI-------------------LTQFDYYADGQIASKSFTHL
HT----GQKQTEQFDYNLNSQLSRAS------------NEVSQIDFYRNALGQLVREHQHYKIPELKPLTAV
LHYEYDELGNLIKTIRP-DGH---------TLNHLVY--GSGHIYAIGLNNQEVVSFQRDDLHRETTRLLAN
G------LMQTKQYNDVGLLSSQFIQPEQETQDYLQYQA----------------HRKYHYDKNYLLSQV--
--------------------------------EDSRLGKLNYQYDPIGRLI------AAQSLHKTESFNFDP
AGNLIDS------DSVLSP-------------------------AQIKNNLIKSYKGKHYQYDVQGNVTEII
QAGKNL-----KLTWDNQNRLIR-------SDNNGLVTEYGYDVFGRRLYKKTAK-------------ELTL
FGWDGDLM--IWESFKSAQTNYTKHYIYEPDS-FVPLLQTGYKDFIQLIETPDYQEYQTKPYSIYKDPVWNS
TTRKKRADLEQITFYHCDQVGTPQTMTNIRGECVWEILQDT-WGAVSQIKA----LNQDNPFEQNNLRFQGQ
YYDQETELHYNRYRYYEPHSARYVSKDPIGLSGGINTSAYVSD-PTQWIDPRGL
>MDR-TJ_5
MAKENTQQKEIKKQPAVELAVFNLNSVTDVADLQMVA--------SQVQRYLQVCGNTSLEQ-IKSKANITT
VANIFALTGSVLDLMLYATDKKTGDAAVQRGALLAANLIGLFS----EPNNEAHARMALRPMFGLMAECLYR
ENGKIK----------------------ETDIKRLGLHLNAMIAGDLENFLKETQAKLSSLLTSATTLGVTI
LQSMATPATGI----NAGITTAAGASAEKRDPKLKFTNWAVPLIDLLGKPSQANLTPKIQPNITSRLQQEAT
QAIAALSQTLQQQ---------------------------ANAGQKYTLAWLLQETLKAIQALENKG--NAS
VPVNQTGEYERHTKGDTLEFVSLQADAL-NAPPC------------EGADSQSGKSISYSIGAERV---QHA
DFYLP-KIGFSFTRQYNSQMDEFDQSMVGARWMMPFSNMIQQN----------------AKGYLFIDSKGRK
HQLPASITFETYEVP-YEGITIQPLKN---------GDLV-----LNFGGDWTF----QFHQFSVGQPYQL-

```
-IQQFNEKTQEKIDLSYLIFEKV-----AYLQYVVFQLEHAKH-QLKFAF--------NEQVKIMAVFLDDK
A------EPL-------ARYEYDIQGNLIKAIDQNG-YTRTYEYNNFHQLTRYTDRTGRGQNIRYEST----
-----------------------DAKAKAVEEWADDGSFHTKLKWHPRLRQVAVYNAYDVPTYYYF---DLN
GFTYRTRLADGRESWYSRDGKKRITRQIDFEGRETQQEYNDQDQLVKIVQPNGGVIRFAY-DEQDNLVEIKD
PEGSIWKREYDENGNLNKEINPLGHITQYKYNN---DNQLVEVIDAKGGVKKIQYNELGQMISYTDCSGK-S
STWEYDEDGVLTAEQTANNKVVQYFYS-------------IKGRDKGQL-------------QSIIYPDGLK
EYFEHDEEGRLLKHTDTKGLVTEYKYNQVGLLEQRIDANRHSVAYQWDKQG-RIQKLINQNQAEYLFGYNPY
GYLIREQAFDGEEKHYSYNE-NGRIFQIRQPNI-------------------LTQFDYYADGQIASKSFTHL
HT----GQKQTEQFDYNLNSQLSRAS------------NEVSQIDFYRNALGQLVREHQHYKIPELKPLTAV
LHYEYDELGNLIKTIRP-DGH---------TLNHLVY--GSGHIYAIGLNNQEVVSFQRDDLHRETTRLLAN
G------LMQTKQYNDVGLLSSQFIQPEQETQDYLQYQA----------------HRKYHYDKNYLLSQV--
--------------------------------EDSRLGKLNYQYDPIGRLI------AAQSLHKTESFNFDP
AGNLIDS------DSVLSP-------------------------AQIKNNLIKSYKGKHYQYDVQGNVTEII
QAGKNL-----KLTWDNQNRLIR-------SDNNGLVTEYGYDVFGRRLYKKTAK-------------ELTL
FGWDGDLM--IWESFKSAQTNYTKHYIYEPDS-FVPLLQTGYKDFIQLIETPDYQEYQTKPYSIYKDPVWNS
TTRKKRADLEQITFYHCDQVGTPQTMTNIRGECVWEILQDT-WGAVSQIKA----LNQDNPFEQNNLRFQGQ
YYDQETELHYNRYRYYEPHSARYVSKDPIGLSGGINTSAYVSD-PTQWIDPRGL
>MDR-ZJ06_5
MAKENTQQKEIKKQPAVELAVFNLNSVTDVADLQMVA--------SQVQRYLQVCGNTSLEQ-IKSKANITT
VANIFALTGSVLDLMLYATDKKTGDAAVQRGALLAANLIGLFS----EPNNEAHARMALRPMFGLMAECLYR
ENGKIK----------------------ETDIKRLGLHLNAMIAGDLENFLKETQAKLSSLLTSATTLGVTI
LQSMATPATGI----NAGITTAAGASAEKRDPKLKFTNWAVPLIDLLGKPSQANLTPKIQPNITSRLQQEAT
QAIAALSQTLQQQ---------------------------ANAGQKYTLAWLLQETLKAIQALENKG--NAS
VPVNQTGEYERHTKGDTLEFVSLQADAL-NAPPC------------EGADSQSGKSISYSIGAERV---QHA
DFYLP-KIGFSFTRQYNSQMDEFDQSMVGARWMMPFSNMIQQN----------------AKGYLFIDSKGRK
HQLPASITFETYEVP-YEGITIQPLKN---------GDLV-----LNFGGDWTF----QFHQFSVGQPYQL-
-IQQFNEKTQEKIDLSYLIFEKV-----AYLQYVVFQLEHAKH-QLKFAF--------NEQVKIMAVFLDDK
A------EPL-------ARYEYDIQGNLIKAIDQNG-YTRTYEYNNFHQLTRYTDRTGRGQNIRYEST----
-----------------------DAKAKAVEEWADDGSFHTKLKWHPRLRQVAVYNAYDVPTYYYF---DLN
GFTYRTRLADGRESWYSRDGKKRITRQIDFEGRETQQEYNDQDQLVKIVQPNGGVIRFAY-DEQDNLVEIKD
PEGSIWKREYDENGNLNKEINPLGHITQYKYNN---DNQLVEVIDAKGGVKKIQYNELGQMISYTDCSGK-S
STWEYDEDGVLTAEQTANNKVVQYFYS-------------IKGRDKGQL-------------QSIIYPDGLK
EYFEHDEEGRLLKHTDTKGLVTEYKYNQVGLLEQRIDANRHSVAYQWDKQG-RIQKLINQNQAEYLFGYNPY
```

GYLIREQAFDGEEKHYSYNE-NGRIFQIRQPNI-------------------LTQFDYYADGQIASKSFTHL
HT----GQKQTEQFDYNLNSQLSRAS------------NEVSQIDFYRNALGQLVREHQHYKIPELKPLTAV
LHYEYDELGNLIKTIRP-DGH---------TLNHLVY--GSGHIYAIGLNNQEVVSFQRDDLHRETTRLLAN
G------LMQTKQYNDVGLLSSQFIQPEQETQDYLQYQA----------------HRKYHYDKNYLLSQV--
--------------------------------EDSRLGKLNYQYDPIGRLI------AAQSLHKTESFNFDP
AGNLIDS------DSVLSP-------------------------AQIKNNLIKSYKGKHYQYDVQGNVTEII
QAGKNL-----KLTWDNQNRLIR-------SDNNGLVTEYGYDVFGRRLYKKTAK-------------ELTL
FGWDGDLM--IWESFKSAQTNYTKHYIYEPDS-FVPLLQTGYKDFIQLIETPDYQEYQTKPYSIYKDPVWNS
TTRKKRADLEQITFYHCDQVGTPQTMTNIRGECVWEILQDT-WGAVSQIKA----LNQDNPFEQNNLRFQGQ
YYDQETELHYNRYRYYEPHSARYVSKDPIGLSGGINTSAYVSD-PTQWIDPRGL
>NCGM237_5
MAKENTQQKEIKKQPAVELAVFNLNSVTDVADLQMVA--------SQVQRYLQVCGNTSLEQ-IKSKANITT
VANIFALTGSVLDLMLYATDKKTGDAAVQRGALLAANLIGLFS----EPNNEAHARMALRPMFGLMAECLYR
ENGKIK----------------------ETDIKRLGLHLNAMIAGDLENFLKETQAKLSSLLTSATTLGVTI
LQSMATPATGI----NAGITTAAGASAEKRDPKLKFTNWAVPLIDLLGKPSQANLTPKIQPNITSRLQQEAT
QAIAALSQTLQQQ---------------------------ANAGQKYTLAWLLQETLKAIQALENKG--NAS
VPVNQTGEYERHTKGDTLEFVSLQADAL-NAPPC------------EGADSQSGKSISYSIGAERV---QHA
DFYLP-KIGFSFTRQYNSQMDEFDQSMVGARWMMPFSNMIQQN----------------AKGYLFIDSKGRK
HQLPASITFETYEVP-YEGITIQPLKN---------GDLV-----LNFGGDWTF----QFHQFSVGQPYQL-
-IQQFNEKTQEKIDLSYLIFEKV-----AYLQYVVFQLEHAKH-QLKFAF--------NEQVKIMAVFLDDK
A------EPL-------ARYEYDIQGNLIKAIDQNG-YTRTYEYNNFHQLTRYTDRTGRGQNIRYEST----
-----------------------DAKAKAVEEWADDGSFHTKLKWHPRLRQVAVYNAYDVPTYYYF---DLN
GFTYRTRLADGRESWYSRDGKKRITRQIDFEGRETQQEYNDQDQLVKIVQPNGGVIRFAY-DEQDNLVEIKD
PEGSIWKREYDENGNLNKEINPLGHITQYKYNN---DNQLVEVIDAKGGVKKIQYNELGQMISYTDCSGK-S
STWEYDEDGVLTAEQTANNKVVQYFYS-------------IKGRDKGQL-------------QSIIYPDGLK
EYFEHDEEGRLLKHTDTKGLVTEYKYNQVGLLEQRIDANRHSVAYQWDKQG-RIQKLINQNQAEYLFGYNPY
GYLIREQAFDGEEKHYSYNE-NGRIFQIRQPNI-------------------LTQFDYYADGQIASKSFTHL
HT----GQKQTEQFDYNLNSQLSRAS------------NEVSQIDFYRNALGQLVREHQHYKIPELKPLTAV
LHYEYDELGNLIKTIRP-DGH---------TLNHLVY--GSGHIYAIGLNNQEVVSFQRDDLHRETTRLLAN
G------LMQTKQYNDVGLLSSQFIQPEQETQDYLQYQA----------------HRKYHYDKNYLLSQV--
--------------------------------EDSRLGKLNYQYDPIGRLI------AAQSLHKTESFNFDP
AGNLIDS------DSVLSP-------------------------AQIKNNLIKSYKGKHYQYDVQGNVTEII
QAGKNL-----KLTWDNQNRLIR-------SDNNGLVTEYGYDVFGRRLYKKTAK-------------ELTL

FGWDGDLM--IWESFKSAQTNYTKHYIYEPDS-FVPLLQTGYKDFIQLIETPDYQEYQTKPYSIYKDPVWNS
TTRKKRADLEQITFYHCDQVGTPQTMTNIRGECVWEILQDT-WGAVSQIKA----LNQDNPFEQNNLRFQGQ
YYDQETELHYNRYRYYEPHSARYVSKDPIGLSGGINTSAYVSD-PTQWIDPRGL
>SMC_Paed_Ab_BL01_5
MAKENTQQKEIKKQPAVELAVFNLNSVTDVADLQMVA--------SQVQRYLQVCGNTSLEQ-IKSKANITT
VANIFALTGSVLDLMLYATDKKTGDAAVQRGALLAANLIGLFS----EPNNEAHARMALRPMFGLMAECLYR
ENGKIK----------------------ETDIKRLGLHLNAMIAGDLENFLKETQAKLSSLLTSATTLGVTI
LQSMATPATGI----NAGITTAAGASAEKRDPKLKFTNWAVPLIDLLGKPSQANLTPKIQPNITSRLQQEAT
QAIAALSQTLQQQ---------------------------ANAGQKYTLAWLLQETLKAIQALENKG--NAS
VPVNQTGEYERHTKGDTLEFVSLQADAL-NAPPC------------EGADSQSGKSISYSIGAERV---QHA
DFYLP-KIGFSFTRQYNSQMDEFDQSMVGARWMMPFSNMIQQN----------------AKGYLFIDSKGRK
HQLPASITFETYEVP-YEGITIQPLKN---------GDLV-----LNFGGDWTF----QFHQFSVGQPYQL-
-IQQFNEKTQEKIDLSYLIFEKV-----AYLQYVVFQLEHAKH-QLKFAF--------NEQVKIMAVFLDDK
A------EPL-------ARYEYDIQGNLIKAIDQNG-YTRTYEYNNFHQLTRYTDRTGRGQNIRYEST----
-----------------------DAKAKAVEEWADDGSFHTKLKWHPRLRQVAVYNAYDVPTYYYF---DLN
GFTYRTRLADGRESWYSRDGKKRITRQIDFEGRETQQEYNDQDQLVKIVQPNGGVIRFAY-DEQDNLVEIKD
PEGSIWKREYDENGNLNKEINPLGHITQYKYNN---DNQLVEVIDAKGGVKKIQYNELGQMISYTDCSGK-S
STWEYDEDGVLTAEQTANNKVVQYFYS-------------IKGRDKGQL-------------QSIIYPDGLK
EYFEHDEEGRLLKHTDTKGLVTEYKYNQVGLLEQRIDANRHSVAYQWDKQG-RIQKLINQNQAEYLFGYNPY
GYLIREQAFDGEEKHYSYNE-NGRIFQIRQPNI-------------------LTQFDYYADGQIASKSFTHL
HT----GQKQTEQFDYNLNSQLSRAS------------NEVSQIDFYRNALGQLVREHQHYKIPELKPLTAV
LHYEYDELGNLIKTIRP-DGH---------TLNHLVY--GSGHIYAIGLNNQEVVSFQRDDLHRETTRLLAN
G------LMQTKQYNDVGLLSSQFIQPEQETQDYLQYQA----------------HRKYHYDKNYLLSQV--
--------------------------------EDSRLGKLNYQYDPIGRLI------AAQSLHKTESFNFDP
AGNLIDS------DSVLSP-------------------------AQIKNNLIKSYKGKHYQYDVQGNVTEII
QAGKNL-----KLTWDNQNRLIR-------SDNNGLVTEYGYDVFGRRLYKKTAK-------------ELTL
FGWDGDLM--IWESFKSAQTNYTKHYIYEPDS-FVPLLQTGYKDFIQLIETPDYQEYQTKPYSIYKDPVWNS
TTRKKRADLEQITFYHCDQVGTPQTMTNIRGECVWEILQDT-WGAVSQIKA----LNQDNPFEQNNLRFQGQ
YYDQETELHYNRYRYYEPHSARYVSKDPIGLSGGINTSAYVSD-PTQWIDPRGL
>TCDC-0715_5
MAKENTQQKEIKKQPAVELAVFNLNSVTDVADLQMVA--------SQVQRYLQVCGNTSLEQ-IKSKANITT
VANIFALTGSVLDLMLYATDKKTGDAAVQRGALLAANLIGLFS----EPNNEAHARMALRPMFGLMAECLYR
ENGKIK----------------------ETDIKRLGLHLNAMIAGDLENFLKETQAKLSSLLTSATTLGVTI

LQSMATPATGI----NAGITTAAGASAEKRDPKLKFTNWAVPLIDLLGKPSQANLTPKIQPNITSRLQQEAT
QAIAALSQTLQQQ---------------------------ANAGQKYTLAWLLQETLKAIQALENKG--NAS
VPVNQTGEYERHTKGDTLEFVSLQADAL-NAPPC------------EGADSQSGKSISYSIGAERV---QHA
DFYLP-KIGFSFTRQYNSQMDEFDQSMVGARWMMPFSNMIQQN----------------AKGYLFIDSKGRK
HQLPASITFETYEVP-YEGITIQPLKN---------GDLV-----LNFGGDWTF----QFHQFSVGQPYQL-
-IQQFNEKTQEKIDLSYLIFEKV-----AYLQYVVFQLEHAKH-QLKFAF--------NEQVKIMAVFLDDK
A------EPL-------ARYEYDIQGNLIKAIDQNG-YTRTYEYNNFHQLTRYTDRTGRGQNIRYEST----
-----------------------DAKAKAVEEWADDGSFHTKLKWHPRLRQVAVYNAYDVPTYYYF---DLN
GFTYRTRLADGRESWYSRDGKKRITRQIDFEGRETQQEYNDQDQLVKIVQPNGGVIRFAY-DEQDNLVEIKD
PEGSIWKREYDENGNLNKEINPLGHITQYKYNN---DNQLVEVIDAKGGVKKIQYNELGQMISYTDCSGK-S
STWEYDEDGVLTAEQTANNKVVQYFYS-------------IKGRDKGQL-------------QSIIYPDGLK
EYFEHDEEGRLLKHTDTKGLVTEYKYNQVGLLEQRIDANRHSVAYQWDKQG-RIQKLINQNQAEYLFGYNPY
GYLIREQAFDGEEKHYSYNE-NGRIFQIRQPNI-------------------LTQFDYYADGQIASKSFTHL
HT----GQKQTEQFDYNLNSQLSRAS------------NEVSQIDFYRNALGQLVREHQHYKIPELKPLTAV
LHYEYDELGNLIKTIRP-DGH---------TLNHLVY--GSGHIYAIGLNNQEVVSFQRDDLHRETTRLLAN
G------LMQTKQYNDVGLLSSQFIQPEQETQDYLQYQA----------------HRKYHYDKNYLLSQV--
--------------------------------EDSRLGKLNYQYDPIGRLI------AAQSLHKTESFNFDP
AGNLIDS------DSVLSP-------------------------AQIKNNLIKSYKGKHYQYDVQGNVTEII
QAGKNL-----KLTWDNQNRLIR-------SDNNGLVTEYGYDVFGRRLYKKTAK-------------ELTL
FGWDGDLM--IWESFKSAQTNYTKHYIYEPDS-FVPLLQTGYKDFIQLIETPDYQEYQTKPYSIYKDPVWNS
TTRKKRADLEQITFYHCDQVGTPQTMTNIRGECVWEILQDT-WGAVSQIKA----LNQDNPFEQNNLRFQGQ
YYDQETELHYNRYRYYEPHSARYVSKDPIGLSGGINTSAYVSD-PTQWIDPRGL
>TYTH-1_5
MAKENTQQKEIKKQPAVELAVFNLNSVTDVADLQMVA--------SQVQRYLQVCGNTSLEQ-IKSKANITT
VANIFALTGSVLDLMLYATDKKTGDAAVQRGALLAANLIGLFS----EPNNEAHARMALRPMFGLMAECLYR
ENGKIK----------------------ETDIKRLGLHLNAMIAGDLENFLKETQAKLSSLLTSATTLGVTI
LQSMATPATGI----NAGITTAAGASAEKRDPKLKFTNWAVPLIDLLGKPSQANLTPKIQPNITSRLQQEAT
QAIAALSQTLQQQ---------------------------ANAGQKYTLAWLLQETLKAIQALENKG--NAS
VPVNQTGEYERHTKGDTLEFVSLQADAL-NAPPC------------EGADSQSGKSISYSIGAERV---QHA
DFYLP-KIGFSFTRQYNSQMDEFDQSMVGARWMMPFSNMIQQN----------------AKGYLFIDSKGRK
HQLPASITFETYEVP-YEGITIQPLKN---------GDLV-----LNFGGDWTF----QFHQFSVGQPYQL-
-IQQFNEKTQEKIDLSYLIFEKV-----AYLQYVVFQLEHAKH-QLKFAF--------NEQVKIMAVFLDDK
A------EPL-------ARYEYDIQGNLIKAIDQNG-YTRTYEYNNFHQLTRYTDRTGRGQNIRYEST----

-----------------------DAKAKAVEEWADDGSFHTKLKWHPRLRQVAVYNAYDVPTYYYF---DLN
GFTYRTRLADGRESWYSRDGKKRITRQIDFEGRETQQEYNDQDLVKIVQPNGGVIRFAY-DEQDNLVEIKD
PEGSIWKREYDENGNLNKEINPLGHITQYKYNN---DNQLVEVIDAKGGVKKIQYNELGQMISYTDCSGK-S
STWEYDEDGVLTAEQTANNKVVQYFYS-------------IKGRDKGQL-------------QSIIYPDGLK
EYFEHDEEGRLLKHTDTKGLVTEYKYNQVGLLEQRIDANRHSVAYQWDKQG-RIQKLINQNQAEYLFGYNPY
GYLIREQAFDGEEKHYSYNE-NGRIFQIRQPNI-------------------LTQFDYYADGQIASKSFTHL
HT----GQKQTEQFDYNLNSQLSRAS------------NEVSQIDFYRNALGQLVREHQHYKIPELKPLTAV
LHYEYDELGNLIKTIRP-DGH---------TLNHLVY--GSGHIYAIGLNNQEVVSFQRDDLHRETTRLLAN
G------LMQTKQYNDVGLLSSQFIQPEQETQDYLQYQA----------------HRKYHYDKNYLLSQV--
--------------------------------EDSRLGKLNYQYDPIGRLI------AAQSLHKTESFNFDP
AGNLIDS------DSVLSP-------------------------AQIKNNLIKSYKGKHYQYDVQGNVTEII
QAGKNL-----KLTWDNQNRLIR-------SDNNGLVTEYGYDVFGRRLYKKTAK-------------ELTL
FGWDGDLM--IWESFKSAQTNYTKHYIYEPDS-FVPLLQTGYKDFIQLIETPDYQEYQTKPYSIYKDPVWNS
TTRKKRADLEQITFYHCDQVGTPQTMTNIRGECVWEILQDT-WGAVSQIKA----LNQDNPFEQNNLRFQGQ
YYDQETELHYNRYRYYEPHSARYVSKDPIGLSGGINTSAYVSD-PTQWIDPRGL
>WCHAB005133_5
MAKENTQQKEIKKQPAVELAVFNLNSVTDVADLQMVA--------SQVQRYLQVCGNTSLEQ-IKSKANITT
VANIFALTGSVLDLMLYATDKKTGDAAVQRGALLAANLIGLFS----EPNNEAHARMALRPMFGLMAECLYR
ENGKIK----------------------ETDIKRLGLHLNAMIAGDLENFLKETQAKLSSLLTSATTLGVTI
LQSMATPATGI----NAGITTAAGASAEKRDPKLKFTNWAVPLIDLLGKPSQANLTPKIQPNITSRLQQEAT
QAIAALSQTLQQQ---------------------------ANAGQKYTLAWLLQETLKAIQALENKG--NAS
VPVNQTGEYERHTKGDTLEFVSLQADAL-NAPPC------------EGADSQSGKSISYSIGAERV---QHA
DFYLP-KIGFSFTRQYNSQMDEFDQSMVGARWMMPFSNMIQQN----------------AKGYLFIDSKGRK
HQLPASITFETYEVP-YEGITIQPLKN---------GDLV-----LNFGGDWTF----QFHQFSVGQPYQL-
-IQQFNEKTQEKIDLSYLIFEKV-----AYLQYVVFQLEHAKH-QLKFAF--------NEQVKIMAVFLDDK
A------EPL-------ARYEYDIQGNLIKAIDQNG-YTRTYEYNNFHQLTRYTDRTGRGQNIRYEST----
-----------------------DAKAKAVEEWADDGSFHTKLKWHPRLRQVAVYNAYDVPTYYYF---DLN
GFTYRTRLADGRESWYSRDGKKRITRQIDFEGRETQQEYNDQDLVKIVQPNGGVIRFAY-DEQDNLVEIKD
PEGSIWKREYDENGNLNKEINPLGHITQYKYNN---DNQLVEVIDAKGGVKKIQYNELGQMISYTDCSGK-S
STWEYDEDGVLTAEQTANNKVVQYFYS-------------IKGRDKGQL-------------QSIIYPDGLK
EYFEHDEEGRLLKHTDTKGLVTEYKYNQVGLLEQRIDANRHSVAYQWDKQG-RIQKLINQNQAEYLFGYNPY
GYLIREQAFDGEEKHYSYNE-NGRIFQIRQPNI-------------------LTQFDYYADGQIASKSFTHL
HT----GQKQTEQFDYNLNSQLSRAS------------NEVSQIDFYRNALGQLVREHQHYKIPELKPLTAV

LHYEYDELGNLIKTIRP-DGH---------TLNHLVY--GSGHIYAIGLNNQEVVSFQRDDLHRETTRLLAN
G------LMQTKQYNDVGLLSSQFIQPEQETQDYLQYQA----------------HRKYHYDKNYLLSQV--
--------------------------------EDSRLGKLNYQYDPIGRLI------AAQSLHKTESFNFDP
AGNLIDS------DSVLSP-------------------------AQIKNNLIKSYKGKHYQYDVQGNVTEII
QAGKNL-----KLTWDNQNRLIR-------SDNNGLVTEYGYDVFGRRLYKKTAK-------------ELTL
FGWDGDLM--IWESFKSAQTNYTKHYIYEPDS-FVPLLQTGYKDFIQLIETPDYQEYQTKPYSIYKDPVWNS
TTRKKRADLEQITFYHCDQVGTPQTMTNIRGECVWEILQDT-WGAVSQIKA----LNQDNPFEQNNLRFQGQ
YYDQETELHYNRYRYYEPHSARYVSKDPIGLSGGINTSAYVSD-PTQWIDPRGL
>XDR-BJ83_5
MAKENTQQKEIKKQPAVELAVFNLNSVTDVADLQMVA--------SQVQRYLQVCGNTSLEQ-IKSKANITT
VANIFALTGSVLDLMLYATDKKTGDAAVQRGALLAANLIGLFS----EPNNEAHARMALRPMFGLMAECLYR
ENGKIK----------------------ETDIKRLGLHLNAMIAGDLENFLKETQAKLSSLLTSATTLGVTI
LQSMATPATGI----NAGITTAAGASAEKRDPKLKFTNWAVPLIDLLGKPSQANLTPKIQPNITSRLQQEAT
QAIAALSQTLQQQ---------------------------ANAGQKYTLAWLLQETLKAIQALENKG--NAS
VPVNQTGEYERHTKGDTLEFVSLQADAL-NAPPC------------EGADSQSGKSISYSIGAERV---QHA
DFYLP-KIGFSFTRQYNSQMDEFDQSMVGARWMMPFSNMIQQN----------------AKGYLFIDSKGRK
HQLPASITFETYEVP-YEGITIQPLKN---------GDLV-----LNFGGDWTF----QFHQFSVGQPYQL-
-IQQFNEKTQEKIDLSYLIFEKV-----AYLQYVVFQLEHAKH-QLKFAF--------NEQVKIMAVFLDDK
A------EPL-------ARYEYDIQGNLIKAIDQNG-YTRTYEYNNFHQLTRYTDRTGRGQNIRYEST----
-----------------------DAKAKAVEEWADDGSFHTKLKWHPRLRQVAVYNAYDVPTYYYF---DLN
GFTYRTRLADGRESWYSRDGKKRITRQIDFEGRETQQEYNDQDQLVKIVQPNGGVIRFAY-DEQDNLVEIKD
PEGSIWKREYDENGNLNKEINPLGHITQYKYNN---DNQLVEVIDAKGGVKKIQYNELGQMISYTDCSGK-S
STWEYDEDGVLTAEQTANNKVVQYFYS-------------IKGRDKGQL-------------QSIIYPDGLK
EYFEHDEEGRLLKHTDTKGLVTEYKYNQVGLLEQRIDANRHSVAYQWDKQG-RIQKLINQNQAEYLFGYNPY
GYLIREQAFDGEEKHYSYNE-NGRIFQIRQPNI--------------------LTQFDYYADGQIASKSFTHL
HT----GQKQTEQFDYNLNSQLSRAS------------NEVSQIDFYRNALGQLVREHQHYKIPELKPLTAV
LHYEYDELGNLIKTIRP-DGH---------TLNHLVY--GSGHIYAIGLNNQEVVSFQRDDLHRETTRLLAN
G------LMQTKQYNDVGLLSSQFIQPEQETQDYLQYQA----------------HRKYHYDKNYLLSQV--
--------------------------------EDSRLGKLNYQYDPIGRLI------AAQSLHKTESFNFDP
AGNLIDS------DSVLSP-------------------------AQIKNNLIKSYKGKHYQYDVQGNVTEII
QAGKNL-----KLTWDNQNRLIR-------SDNNGLVTEYGYDVFGRRLYKKTAK-------------ELTL
FGWDGDLM--IWESFKSAQTNYTKHYIYEPDS-FVPLLQTGYKDFIQLIETPDYQEYQTKPYSIYKDPVWNS
TTRKKRADLEQITFYHCDQVGTPQTMTNIRGECVWEILQDT-WGAVSQIKA----LNQDNPFEQNNLRFQGQ

YYDQETELHYNRYRYYEPHSARYVSKDPIGLSGGINTSAYVSD-PTQWIDPRGL
>XH386_5
MAKENTQQKEIKKQPAVELAVFNLNSVTDVADLQMVA--------SQVQRYLQVCGNTSLEQ-IKSKANITT
VANIFALTGSVLDLMLYATDKKTGDAAVQRGALLAANLIGLFS----EPNNEAHARMALRPMFGLMAECLYR
ENGKIK----------------------ETDIKRLGLHLNAMIAGDLENFLKETQAKLSSLLTSATTLGVTI
LQSMATPATGI----NAGITTAAGASAEKRDPKLKFTNWAVPLIDLLGKPSQANLTPKIQPNITSRLQQEAT
QAIAALSQTLQQQ---------------------------ANAGQKYTLAWLLQETLKAIQALENKG--NAS
VPVNQTGEYERHTKGDTLEFVSLQADAL-NAPPC------------EGADSQSGKSISYSIGAERV---QHA
DFYLP-KIGFSFTRQYNSQMDEFDQSMVGARWMMPFSNMIQQN----------------AKGYLFIDSKGRK
HQLPASITFETYEVP-YEGITIQPLKN---------GDLV-----LNFGGDWTF----QFHQFSVGQPYQL-
-IQQFNEKTQEKIDLSYLIFEKV-----AYLQYVVFQLEHAKH-QLKFAF--------NEQVKIMAVFLDDK
A------EPL-------ARYEYDIQGNLIKAIDQNG-YTRTYEYNNFHQLTRYTDRTGRGQNIRYEST----
-----------------------DAKAKAVEEWADDGSFHTKLKWHPRLRQVAVYNAYDVPTYYYF---DLN
GFTYRTRLADGRESWYSRDGKKRITRQIDFEGRETQQEYNDQDQLVKIVQPNGGVIRFAY-DEQDNLVEIKD
PEGSIWKREYDENGNLNKEINPLGHITQYKYNN---DNQLVEVIDAKGGVKKIQYNELGQMISYTDCSGK-S
STWEYDEDGVLTAEQTANNKVVQYFYS-------------IKGRDKGQL-------------QSIIYPDGLK
EYFEHDEEGRLLKHTDTKGLVTEYKYNQVGLLEQRIDANRHSVAYQWDKQG-RIQKLINQNQAEYLFGYNPY
GYLIREQAFDGEEKHYSYNE-NGRIFQIRQPNI-------------------LTQFDYYADGQIASKSFTHL
HT----GQKQTEQFDYNLNSQLSRAS------------NEVSQIDFYRNALGQLVREHQHYKIPELKPLTAV
LHYEYDELGNLIKTIRP-DGH---------TLNHLVY--GSGHIYAIGLNNQEVVSFQRDDLHRETTRLLAN
G------LMQTKQYNDVGLLSSQFIQPEQETQDYLQYQA----------------HRKYHYDKNYLLSQV--
--------------------------------EDSRLGKLNYQYDPIGRLI------AAQSLHKTESFNFDP
AGNLIDS------DSVLSP-------------------------AQIKNNLIKSYKGKHYQYDVQGNVTEII
QAGKNL-----KLTWDNQNRLIR-------SDNNGLVTEYGYDVFGRRLYKKTAK-------------ELTL
FGWDGDLM--IWESFKSAQTNYTKHYIYEPDS-FVPLLQTGYKDFIQLIETPDYQEYQTKPYSIYKDPVWNS
TTRKKRADLEQITFYHCDQVGTPQTMTNIRGECVWEILQDT-WGAVSQIKA----LNQDNPFEQNNLRFQGQ
YYDQETELHYNRYRYYEPHSARYVSKDPIGLSGGINTSAYVSD-PTQWIDPRGL
>XH856_5
MAKENTQQKEIKKQPAVELAVFNLNSVTDVADLQMVA--------SQVQRYLQVCGNTSLEQ-IKSKANITT
VANIFALTGSVLDLMLYATDKKTGDAAVQRGALLAANLIGLFS----EPNNEAHARMALRPMFGLMAECLYR
ENGKIK----------------------ETDIKRLGLHLNAMIAGDLENFLKETQAKLSSLLTSATTLGVTI
LQSMATPATGI----NAGITTAAGASAEKRDPKLKFTNWAVPLIDLLGKPSQANLTPKIQPNITSRLQQEAT
QAIAALSQTLQQQ---------------------------ANAGQKYTLAWLLQETLKAIQALENKG--NAS

VPVNQTGEYERHTKGDTLEFVSLQADAL-NAPPC------------EGADSQSGKSISYSIGAERV---QHA
DFYLP-KIGFSFTRQYNSQMDEFDQSMVGARWMMPFSNMIQQN----------------AKGYLFIDSKGRK
HQLPASITFETYEVP-YEGITIQPLKN---------GDLV-----LNFGGDWTF----QFHQFSVGQPYQL-
-IQQFNEKTQEKIDLSYLIFEKV-----AYLQYVVFQLEHAKH-QLKFAF--------NEQVKIMAVFLDDK
A------EPL-------ARYEYDIQGNLIKAIDQNG-YTRTYEYNNFHQLTRYTDRTGRGQNIRYEST----
-----------------------DAKAKAVEEWADDGSFHTKLKWHPRLRQVAVYNAYDVPTYYYF---DLN
GFTYRTRLADGRESWYSRDGKKRITRQIDFEGRETQQEYNDQDQLVKIVQPNGGVIRFAY-DEQDNLVEIKD
PEGSIWKREYDENGNLNKEINPLGHITQYKYNN---DNQLVEVIDAKGGVKKIQYNELGQMISYTDCSGK-S
STWEYDEDGVLTAEQTANNKVVQYFYS-------------IKGRDKGQL-------------QSIIYPDGLK
EYFEHDEEGRLLKHTDTKGLVTEYKYNQVGLLEQRIDANRHSVAYQWDKQG-RIQKLINQNQAEYLFGYNPY
GYLIREQAFDGEEKHYSYNE-NGRIFQIRQPNI-------------------LTQFDYYADGQIASKSFTHL
HT----GQKQTEQFDYNLNSQLSRAS------------NEVSQIDFYRNALGQLVREHQHYKIPELKPLTAV
LHYEYDELGNLIKTIRP-DGH---------TLNHLVY--GSGHIYAIGLNNQEVVSFQRDDLHRETTRLLAN
G------LMQTKQYNDVGLLSSQFIQPEQETQDYLQYQA----------------HRKYHYDKNYLLSQV--
--------------------------------EDSRLGKLNYQYDPIGRLI------AAQSLHKTESFNFDP
AGNLIDS------DSVLSP------------------------AQIKNNLIKSYKGKHYQYDVQGNVTEII
QAGKNL-----KLTWDNQNRLIR-------SDNNGLVTEYGYDVFGRRLYKKTAK-------------ELTL
FGWDGDLM--IWESFKSAQTNYTKHYIYEPDS-FVPLLQTGYKDFIQLIETPDYQEYQTKPYSIYKDPVWNS
TTRKKRADLEQITFYHCDQVGTPQTMTNIRGECVWEILQDT-WGAVSQIKA----LNQDNPFEQNNLRFQGQ
YYDQETELHYNRYRYYEPHSARYVSKDPIGLSGGINTSAYVSD-PTQWIDPRGL
>YU-R612_5
MAKENTQQKEIKKQPAVELAVFNLNSVTDVADLQMVA--------SQVQRYLQVCGNTSLEQ-IKSKANITT
VANIFALTGSVLDLMLYATDKKTGDAAVQRGALLAANLIGLFS----EPNNEAHARMALRPMFGLMAECLYR
ENGKIK----------------------ETDIKRLGLHLNAMIAGDLENFLKETQAKLSSLLTSATTLGVTI
LQSMATPATGI----NAGITTAAGASAEKRDPKLKFTNWAVPLIDLLGKPSQANLTPKIQPNITSRLQQEAT
QAIAALSQTLQQQ--------------------------ANAGQKYTLAWLLQETLKAIQALENKG--NAS
VPVNQTGEYERHTKGDTLEFVSLQADAL-NAPPC------------EGADSQSGKSISYSIGAERV---QHA
DFYLP-KIGFSFTRQYNSQMDEFDQSMVGARWMMPFSNMIQQN----------------AKGYLFIDSKGRK
HQLPASITFETYEVP-YEGITIQPLKN---------GDLV-----LNFGGDWTF----QFHQFSVGQPYQL-
-IQQFNEKTQEKIDLSYLIFEKV-----AYLQYVVFQLEHAKH-QLKFAF--------NEQVKIMAVFLDDK
A------EPL-------ARYEYDIQGNLIKAIDQNG-YTRTYEYNNFHQLTRYTDRTGRGQNIRYEST----
-----------------------DAKAKAVEEWADDGSFHTKLKWHPRLRQVAVYNAYDVPTYYYF---DLN
GFTYRTRLADGRESWYSRDGKKRITRQIDFEGRETQQEYNDQDQLVKIVQPNGGVIRFAY-DEQDNLVEIKD

PEGSIWKREYDENGNLNKEINPLGHITQYKYNN---DNQLVEVIDAKGGVKKIQYNELGQMISYTDCSGK-S
STWEYDEDGVLTAEQTANNKVVQYFYS-------------IKGRDKGQL-------------QSIIYPDGLK
EYFEHDEEGRLLKHTDTKGLVTEYKYNQVGLLEQRIDANRHSVAYQWDKQG-RIQKLINQNQAEYLFGYNPY
GYLIREQAFDGEEKHYSYNE-NGRIFQIRQPNI-------------------LTQFDYYADGQIASKSFTHL
HT----GQKQTEQFDYNLNSQLSRAS------------NEVSQIDFYRNALGQLVREHQHYKIPELKPLTAV
LHYEYDELGNLIKTIRP-DGH---------TLNHLVY--GSGHIYAIGLNNQEVVSFQRDDLHRETTRLLAN
G------LMQTKQYNDVGLLSSQFIQPEQETQDYLQYQA----------------HRKYHYDKNYLLSQV--
--------------------------------EDSRLGKLNYQYDPIGRLI------AAQSLHKTESFNFDP
AGNLIDS------DSVLSP-------------------------AQIKNNLIKSYKGKHYQYDVQGNVTEII
QAGKNL-----KLTWDNQNRLIR-------SDNNGLVTEYGYDVFGRRLYKKTAK-------------ELTL
FGWDGDLM--IWESFKSAQTNYTKHYIYEPDS-FVPLLQTGYKDFIQLIETPDYQEYQTKPYSIYKDPVWNS
TTRKKRADLEQITFYHCDQVGTPQTMTNIRGECVWEILQDT-WGAVSQIKA----LNQDNPFEQNNLRFQGQ
YYDQETELHYNRYRYYEPHSARYVSKDPIGLSGGINTSAYVSD-PTQWIDPRGL
>SDF_15
MAKENTQQKEIKRQPAVELAVFNLNSVTDVADLQMIA--------SQVQLYLQVCGSTTLEQ-IKSKANITT
VANIFALTGSVLDLMLYATDKKTGDAAVQRGALLAANLIGLFS----EPNNEAHARMALRPMFGLMAECLYR
ENGKIK----------------------ETDIKRLGLHFNAMIAGDLENFLKETQAKLSSLLISATTLGVTI
LQSMATPATGI----NAGITTAAGASAEKRDPKLKFTNWAVPLIDLLGKPSQANLTPKIQPNITSRLQQEAT
QAIAALSQTLQQ----------------------------ANAGQKYTLAWLLQETLKAIQALENKG--NAS
VPVNQTGEYERHTKGDTLEFVSLQADAL-NAPPC------------EGADSQSGKSISYSIGAERV---QHA
DFYLP-KIGFSFIRQYNSQMDEFDQSMVGARWMMPFSNMIQRT----------------AQGYLFIDSKGRK
HQLPASIIFETYEVP-YEGIIVQPLKN---------GDLV-----LNFGGDWTF----QFHQFSVGQPYQL-
-IQQFNEKTQEKIDLSYLIFEKV-----AYLQCVNFQLEHAKH-QLKFAF--------NEQVKIMAVFLDDN
A------EPL-------AHYDYDTQGNLIKAIDQNG-HTRTYEYNYFHQLTRYTDRTGRGQNIRYEST----
-----------------------DAKAKAIEEWADDGSFHTKLKWHPRLRQVAVYDAYDVPTYYYF---DLN
GFTYRTRLADGRESWYSRDGKKRITRQIDFEGRETQQEYNDQDQLVKIVQPNGGIIRFEY-NEQGNLVEIKD
PEGSIWKREYDENRNVSKEINPLGHITQYKYNN---DNQLVEVIDAKGGVKKIQYNELGQMISYTDCSGK-S
SIWEYDEDGVLTAEQTAKNKVVQYFYS-------------TKGRDKGQL-------------QSIIYPDGLK
EYFEHDEEGRLLKHIDTKGLVTEYKYNQVGLLEQRIDANRHSVTYQWDKQG-RIQKLINQNQAEYLFGYNPY
GYLIREQAFDGEEKHYSYNE-NGRLFKIRQPNT-------------------LTEFDYYADGQIASKSFTHL
HT----GQKQTEQFDYNLNSQLSRAS------------NEVSQIDFYRNALGQLVREHQHYKIPELKPLTAV
LHYEYDELGNLIKTIRP-DGH---------TLNHLVY--GSGHIYAIGLNNQEVVSFQRDDLHRETTRLLAN
G------LMQTKQYNDVGLLSSQFIQPEQETQDYLQYQA----------------HRKYHYDKNYLLSQV--

--------------------------------EDSRLGKLNYQYDPIGRLI------AAQSLHKTESFNFDP
AGNLIDS------DSVLSP-------------------------AQIKNNLIKSYKGKHYQYDVQGNVTEII
QAGKNL-----KLTWDNQNRLIR-------SDNNGLVTEYGYDVFGRRLYKKTTN-------------ELTL
FGWDGDLM--IWESFKSAQTNYTKHYIYEPDS-FVPLLQTGYKDLIQLIETPDYQEYQTKPYSIYKDPVWNS
TTRKKRADLEQITFYHCDQVGIPQTMTNIRGECVWEILQDT-WGAVSQIKA----LNQDNPFEQNNLRFQGQ
YYDQETELHYNRYRYYEPHSARYVSKDPIGLEGGMNTSSYVSD-PNQWIDPKGL
>6200_15
MAKENTQQKEIKRQPAVELAVFNLNSVTDVADLQMIA--------SQVQLYLQVCGNTTLEQ-IKSKANITT
VANIFALTGSVLDLMLYATDKKTGDAAVQRGALLAANLIGLFS----EPNNEAHARMALRPMFGLMAECLYR
ENGKIK----------------------ETDIKRLGLHLNAMIAGDLENFLKETQAKLSSLLISATTLGVTI
LQSMATPATGI----NAGISTAAGASAEKRDPKLKFTNWAVPLIDLLGKPSQANLTPKIQPNITSRLQQEAT
QAIAALSQTLQQQ--------------------------ANAGQKYTLAWLLQEALKAIQALENKG--NAS
VPVNQTGEYERHTKGDTLEFVSLQADAL-NAPPC------------EGADSQSGKSISYSIGAERV---QHA
DFYLP-KIGFSFIRQYNSQMDEFDQSMVGARWMMPFSNMIQRT----------------AQGYLFIDSKGRK
HQLPASITFETYEVP-YEGIIVQPLKN---------GDLV-----LNFGGDWTF----QFHQFSVGQPYQL-
-IQQFNEKTQEKIDLSYLIFEKV-----AYLQCVNFQLEHAKH-QLKFAF--------NEQVKIMAVFLDDN
A------EPL-------ARYDYDTQGNLIKAIDQNG-HTRTYEYNYFHQLTRYTDRTGRGQNIRYEST----
-----------------------DAKAKAIEEWADDGSFHTKLKWHPRLRQVAVYDAYDVPTYYYF---DLN
GFTYRTRLADGRESWYSRDGKKRITRQIDFEGRETQQEYNDQDQLVKIVQPNGGVIRFAY-DEQDNLVEIKD
PEGSIWKREYDENGNLNKEINPLGHITQYKYNN---DNQLVEVIDAKGGVKKIQYNELGQMISYTDCSGK-S
STWEYDEDGVLTAEQTANNKVVQYFYS-------------TKGRDKGQL-------------QSIIYPDGLK
EYFEHDEEGRLLKHTDTKGLVTEYKYNQVGLLEQRIDANRHSVAYQWDKQG-RIQKLINQNQAEYLFGYNPY
GYLIREQAFDGEEKHYSYNE-NGRIFQIRQPNI-------------------LTQFDYYADGQIASKSFTHL
HT----GQKQTEQFDYNLNSQLSRAS------------NEVSQIDFYRNALGQLVREHQHYKIPELKPLTAV
LHYEYDELGNLIKTIRP-DGH---------TLNHLVY--GSGHIYAIGLNNQEVVSFQRDDLHRETTRLLAN
G------LIQTKQYNDVGLLSSQLIQPEQETQDYLQYQA----------------HRHYQYDQNYLLSQV--
--------------------------------EDSRLGRLNYQYDPIGRLI------AAQSLHKTESFNFDP
AGNLIDS------DSVLSP-------------------------AQIKNNLIKSYKGKHYQYDAQGNVTEII
QAGKNL-----KLTWDNQNRLIR-------SDNNGLVTEYGYDVFGRRLYKKTTN-------------ELTL
FGWDGDLM--IWESVKSAQMSYTKHYIYEPDS-FVPLLQAGYKDFIQLIETPDYQEYQTKPYSIYKDPVWNR
NLGKERTALEQFTFYHCDQVGTPQTMTNIRGECVWEILQDT-WGAVSQIKA----LNQDNPFEQNNLRFQGQ
YYDQETELHYNRYRYYEPHSARYVSKDPIGLEGGMNTSSYVSD-PNQWIDPKGL
>IOMTU433_15

MAKENTQQKEIKRQPAVELAVFNLNSVTDVADLQMIA--------SQVQLYLQVCGNTTLEQ-IKSKANITT
VANIFALTGSVLDLMLYATDKKTGDAAVQRGALLAANLIGLFS----EPNNEAHARMALRPMFGLMAECLYR
ENGKIK----------------------ETDIKRLGLHLNAMIAGDLENFLKETQAKLSSLLISATTLGVTI
LQSMATPATGI----NAGITTAAGASAEKRDPKLKFTNWAVPLIDLLGKPSQANLTPKIQPNITSRLQQEAT
QAIAALGQALQQQ---------------------------ANAGQKYTLAWLLQETLKAIQALENKG--NAS
VPVNQTGEYERHTKGDTLEFVSLQADAL-NAPPC------------EGADSQSGKSISYSIGAERV---QHA
DFYLP-KIGFSFIRQYNSQMDEFDQSMVGARWMMPFSNMIQRT----------------AQGYLFIDSKGRK
HQLPASIIFETYEVP-YEGIIVQPLKN---------GDLV-----LNFGGDWTF----QFHQFSVGQPYQL-
-IQQFNEKTQEKIDLSYLIFEKV-----AYLQCVNFQLEHAKH-QLKFAF--------NEQVKIMAVFLDDN
A------EPL-------ARYDYDTQGNLIKAIDQNG-HTRTYEYNYFHQLTRYTDRTGRGQNIRYEST----
-----------------------DAKAKAIEEWADDGSFHTKLKWHPRLRQVAVYDAYDVPTYYYF---DLN
GFTYRTRLADGRESWYSRDGKKRITRQIDFEGRETQQEYNDQDQLVKIVQPNGGIIRFEY-NEQGNLVEIKD
PEGSIWKREYDENGNLNKEINPLGHITQYKYNN---DNQLVEVIDAKGGVKKIQYNELGQMISYTDCSGK-S
STWEYDEDGVLTAEQTANNKVVQYFYS-------------TKGRDKGQL-------------QSIIYPDGLK
EYFEHDEEGRLLKHTDTKGLVTEYKYNQVGLLEQRIDANRHSVAYQWDKQG-RIQKLINQNQAEYLFGYNPY
GYLIREQAFDGEEKHYSYNE-NGRIFQIRQPNI-------------------LTQFDYYADGQIASKSFTHL
HT----GQKQTEQFDYNLNSQLSRAS------------NEVSQIDFYRNALGQLVREHQHYKIPELKPLTAV
LHYEYDELGNLIKTIRP-DGH---------TLNHLVY--GSGHIYAIGLNNQEVVSFQRDDLHRETTRLLAN
G------LMQTKQYNDVGLLSSQFIQPEQETQDYLQYQA----------------HRKYHYDKNYLLSQV--
--------------------------------EDSRLGKLNYQYDPIGRLI------AAQSLHKTESFNFDP
AGNLIDS------DSVLSP-------------------------AQIKNNLIKSYKGKHYQYDVQGNVTEII
QAGKNL-----KLTWDNQNRLIR-------SDNNGLVTEYGYDVFGRRLYKKTAK-------------ELTL
FGWDGDLM--IWESFKSAQTNYTKHYIYEPDS-FVPLLQAGYKDFIQLIETPDYQEYQTKPYSIYKDPVWNR
NLGKERTALEQFTFYHCDQVGTPQTMTNIRGECVWEILQDT-WGAVSQIKA----LNQDNPFEQNNLRFQGQ
YYDQETELHYNRYRYYEPHSARYVSKDPIGLEGGMNTSSYVSD-PNQWIDPKGL
>AB030_22
MPTAPKTISQAKAKPSVRTNTGQVAVVPL--NKLYAADVKAGMNKIDSWLKTNTKGYVDLNTIKSTLGTLPV
IGNAIALVDAIYDVIDISKK---PSPAFTDWLNLGLDVIGIIP----APPTMATMRMSLRPTLNLVRQKAKG
TIS-------------------------DTILVILSDHLNERIAGELDQFAKNAQPLVNQMLQNCGTKITAI
SNDFANGIQKV----LNGKVYSSAGNLKNAQK--QLNKVNLNNLQRNPKATISNAVDGLVNIWKAGVKE-QI
NTVAKGVSAVVPASATQPIQNAITSIRVFGRKAPSYLMALADPAKAMSIAWLLNVLLKAVQKFKLKGTKTAN
IKPTQVTEKRRQKPNGELEKANVQAKSKGNPSETVCALNLRGGKSKPKSTSGTKRNITFALGTECL---THQ
DAYVSSILSFVLIRKYASNLYQLDHGEFGARWITPFTTRIVPQSEYIEETEERAGYLKHLSGFEFIGADARP

VKLPNLKVGESFHNK-TEDFYYSVISE---------KVQM-----ISYQKEEKH--------LFEKYQDGY-
RLSAIEYKNGLTLA---VRYDHHFEGL-SFISDIVIKEKQKQLIHVAFQV--------NASGRIEDVWLVEN
G---QLARPL-------ASYNYNEKGDLVEAITENG-ASYHYQY-DHHLLTRYTDLTGRGMNLEYDGV----
-----------------------EPTSKAYHEWADDGSSDTILQWDENIRLTYVTDAYGAETWYYY---DIE
GYTYRIIYPDGLEEWFFRDDFGRVTKHVDTDAAVTIYDYDEHGNIVSMTQPDGSLLYYAY-DENRQLIGMVD
AEGGRWFKEYDAAGNITKETDPLKRETQYSYNG---LGLLTSIKDPKGGTKTLSYDDQGNLISYIDCSGK-E
TKWTYDKKGRVVAIENALKQKVEYFYSDLTIEQREPIIKGLPLNAFGQL-------------EKIKHADGTE
EHFIHDAEGRLLAHIDQKQNITRYEYDAAGLIASRTDALNHKLKYEWDKLN-RLKRLINENGASYQFFYDVA
SRLVKEIDFDGKETIYNYDETDGNLVSSVEVASMHGQDLRDRIAPKDR----IQKFLFDSMGRLEQRTAGYG
YVEHDLEQQLVEEFAYDSNGNLIQAK------------NAESNLQWYYDAVGNVIREHHQ---DYKTKKTGV
WKHSYDEINDRIKTVRP-DGQ---------KIDWLTY--GSGHVHSLIVNGQDVVSFERDDLHREIARHYAN
G------ISQEQHYDTMGRLTQQNIVNGHE-FGYPSNEQVSQTQNNAIQETQQLIQRLYQYDKTGQLTGI--
--------------------------------NDTRRGNINYKYDPVGRLL------EASSKLGKETFSFDP
ASNILDS------YHSQKV-----------QSHSQKLDETVYGYNRLVNNVVKEYLDQQYQYDVYGQLVCQK
STKGNL-----YLEWDACGRLIK-------SRNAEYTAEYRYDALGRRIQKRSKH-------HHTSGEHNII
YGWDGNTL--AYES----NEQITKHYIYEKDS-FVPLAQAVYAEEIELHQTPDWAD---KPYSLQRDPLWKV
TKTAK--DFKDFWFYHCDHLGTPQEMTDHTGAVIWKAEYKA-WGECKAEKAKSNFFEDSEIISN-NIRFQGQ
YFDQETGLHYNRYRYYSPYVGRFISKDPIGLLGGDNVYAYAPN-PVEWVDPLGL
>AbH12O-A2_22
MPTAPKTISQAKAKPSVRTNTGQVAVVPL--NKLYAADVKAGMNKIDSWLKTNTKGYVDLNTIKSTLGTLPV
IGNAIALVDAIYDVIDISKK---PSPAFTDWLNLGLDVIGIIP----APPTMATMRMSLRPTLNLVRQKAKG
TIS-------------------------DTILVILSDHLNERIAGELDQFAKNAQPLVNQMLQNCGTKITAI
SNDFANGIQKV----LNGKVYSSAGNLKNAQK--QLNKVNLNNLQRNPKATISNAVDGLVNIWKAGVKE-QI
NTVAKGVSAVVPASATQPIQNAITSIRVFGRKAPSYLMALADPAKAMSIAWLLNVLLKAVQKFKLKGTKTAN
IKPTQVTEKRRQKPNGELEKANVQAKSKGNPSETVCALNLRGGKSKPKSTSGTKRNITFALGTECL---THQ
DAYVSSILSFVLIRKYASNLYQLDHGEFGARWITPFTTRIVPQSEYIEETEERAGYLKHLSGFEFIGADARP
VKLPNLKVGESFHNK-TEDFYYSVISE---------KVQM-----ISYQKEEKH--------LFEKYQDGY-
RLSAIEYKNGLTLA---VRYDHHFEGL-SFISDIVIKEKQKQLIHVAFQV--------NASGRIEDVWLVEN
G---QLARPL-------ASYNYNEKGDLVEAITENG-ASYHYQY-DHHLLTRYTDLTGRGMNLEYDGV----
-----------------------EPTSKAYHEWADDGSSDTILQWDENIRLTYVTDAYGAETWYYY---DIE
GYTYRIIYPDGLEEWFFRDDFGRVTKHVDTDAAVTIYDYDEHGNIVSMTQPDGSLLYYAY-DENRQLIGMVD
AEGGRWFKEYDAAGNITKETDPLKRETQYSYNG---LGLLTSIKDPKGGTKTLSYDDQGNLISYIDCSGK-E
TKWTYDKKGRVVAIENALKQKVEYFYSDLTIEQREPIIKGLPLNAFGQL-------------EKIKHADGTE

EHFIHDAEGRLLAHIDQKQNITRYEYDAAGLIASRTDALNHKLKYEWDKLN-RLKRLINENGASYQFFYDVA
SRLVKEIDFDGKETIYNYDETDGNLVSSVEVASMHGQDLRDRIAPKDR----IQKFLFDSMGRLEQRTAGYG
YVEHDLEQQLVEEFAYDSNGNLIQAK------------NAESNLQWYYDAVGNVIREHHQ---DYKTKKTGV
WKHSYDEINDRIKTVRP-DGQ---------KIDWLTY--GSGHVHSLIVNGQDVVSFERDDLHREIARHYAN
G------ISQEQHYDTMGRLTQQNIVNGHE-FGYPSNEQVSQTQNNAIQETQQLIQRLYQYDKTGQLTGI--
--------------------------------NDTRRGNINYKYDPVGRLL------EASSKLGKETFSFDP
ASNILDS------YHSQKV-----------QSHSQKLDETVYGYNRLVNNVVKEYLDQQYQYDVYGQLVCQK
STKGNL-----YLEWDACGRLIK-------SRNAEYTAEYRYDALGRRIQKRSKH-------HHTSGEHNII
YGWDGNTL--AYES----NEQITKHYIYEKDS-FVPLAQAVYAEEIELHQTPDWAD---KPYSLQRDPLWKV
TKTAK--DFKDFWFYHCDHLGTPQEMTDHTGAVIWKAEYKA-WGECKAEKAKSNFFEDSEIISN-NIRFQGQ
YFDQETGLHYNRYRYYSPYVGRFISKDPIGLLGGDNVYAYAPN-PVEWVDPLGL
>AF-401_22
MPTAPKTISQAKAKPSVRTNTGQVAVVPL--NKLYAADVKAGMNKIDSWLKTNTKGYVDLNTIKSTLGTLPV
IGNAIALVDAIYDVIDISKK---PSPAFTDWLNLGLDVIGIIP----APPTMATMRMSLRPTLNLVRQKAKG
TIS-------------------------DTILVILSDHLNERIAGELDQFAKNAQPLVNQMLQNCGTKITAI
SNDFANGIQKV----LNGKVYSSAGNLKNAQK--QLNKVNLNNLQRNPKATISNAVDGLVNIWKAGVKE-QI
NTVAKGVSAVVPASATQPIQNAITSIRVFGRKAPSYLMALADPAKAMSIAWLLNVLLKAVQKFKLKGTKTAN
IKPTQVTEKRRQKPNGELEKANVQAKSKGNPSETVCALNLRGGKSKPKSTSGTKRNITFALGTECL---THQ
DAYVSSILSFVLIRKYASNLYQLDHGEFGARWITPFTTRIVPQSEYIEETEERAGYLKHLSGFEFIGADARP
VKLPNLKVGESFHNK-TEDFYYSVISE---------KVQM-----ISYQKEEKH--------LFEKYQDGY-
RLSAIEYKNGLTLA---VRYDHHFEGL-SFISDIVIKEKQKQLIHVAFQV--------NASGRIEDVWLVEN
G---QLARPL-------ASYNYNEKGDLVEAITENG-ASYHYQY-DHHLLTRYTDLTGRGMNLEYDGV----
-----------------------EPTSKAYHEWADDGSSDTILQWDENIRLTYVTDAYGAETWYYY---DIE
GYTYRIIYPDGLEEWFFRDDFGRVTKHVDTDAAVTIYDYDEHGNIVSMTQPDGSLLYYAY-DENRQLIGMVD
AEGGRWFKEYDAAGNITKETDPLKRETQYSYNG---LGLLTSIKDPKGGTKTLSYDDQGNLISYIDCSGK-E
TKWTYDKKGRVVAIENALKQKVEYFYSDLTIEQREPIIKGLPLNAFGQL-------------EKIKHADGTE
EHFIHDAEGRLLAHIDQKQNITRYEYDAAGLIASRTDALNHKLKYEWDKLN-RLKRLINENGASYQFFYDVA
SRLVKEIDFDGKETIYNYDETDGNLVSSVEVASMHGQDLRDRIAPKDR----IQKFLFDSMGRLEQRTAGYG
YVEHDLEQQLVEEFAYDSNGNLIQAK------------NAESNLQWYYDAVGNVIREHHQ---DYKTKKTGV
WKHSYDEINDRIKTVRP-DGQ---------KIDWLTY--GSGHVHSLIVNGQDVVSFERDDLHREIARHYAN
G------ISQEQHYDTMGRLTQQNIVNGHE-FGYPSNEQVSQTQNNAIQETQQLIQRLYQYDKTGQLTGI--
--------------------------------NDTRRGNINYKYDPVGRLL------EASSKLGKETFSFDP
ASNILDS------YHSQKV-----------QSHSQKLDETVYGYNRLVNNVVKEYLDQQYQYDVYGQLVCQK

STKGNL-----YLEWDACGRLIK-------SRNAEYTAEYRYDALGRRIQKRSKH-------HHTSGEHNII
YGWDGNTL--AYES----NEQITKHYIYEKDS-FVPLAQAVYAEEIELHQTPDWAD---KPYSLQRDPLWKV
TKTAK--DFKDFWFYHCDHLGTPQEMTDHTGAVIWKAEYKA-WGECKAEKAKSNFFEDSEIISN-NIRFQGQ
YFDQETGLHYNRYRYYSPYVGRFISKDPIGLLGGDNVYAYAPN-PVEWVDPLGL
>AR_0063_22
MPTAPKTISQAKAKPSVRTNTGQVAVVPL--NKLYAADVKAGMNKIDSWLKTNTKGYVDLNTIKSTLGTLPV
IGNAIALVDAIYDVIDISKK---PSPAFTDWLNLGLDVIGIIP----APPTMATMRMSLRPTLNLVRQKAKG
TIS-------------------------DTILVILSDHLNERIAGELDQFAKNAQPLVNQMLQNCGTKITAI
SNDFANGIQKV----LNGKVYSSAGNLKNAQK--QLNKVNLNNLQRNPKATISNAVDGLVNIWKAGVKE-QI
NTVAKGVSAVVPASATQPIQNAITSIRVFGRKAPSYLMALADPAKAMSIAWLLNVLLKAVQKFKLKGTKTAN
IKPTQVTEKRRQKPNGELEKANVQAKSKGNPSETVCALNLRGGKSKPKSTSGTKRNITFALGTECL---THQ
DAYVSSILSFVLIRKYASNLYQLDHGEFGARWITPFTTRIVPQSEYIEETEERAGYLKHLSGFEFIGADARP
VKLPNLKVGESFHNK-TEDFYYSVISE---------KVQM-----ISYQKEEKH--------LFEKYQDGY-
RLSAIEYKNGLTLA---VRYDHHFEGL-SFISDIVIKEKQKQLIHVAFQV--------NASGRIEDVWLVEN
G---QLARPL-------ASYNYNEKGDLVEAITENG-ASYHYQY-DHHLLTRYTDLTGRGMNLEYDGV----
-----------------------EPTSKAYHEWADDGSSDTILQWDENIRLTYVTDAYGAETWYYY---DIE
GYTYRIIYPDGLEEWFFRDDFGRVTKHVDTDAAVTIYDYDEHGNIVSMTQPDGSLLYYAY-DENRQLIGMVD
AEGGRWFKEYDAAGNITKETDPLKRETQYSYNG---LGLLTSIKDPKGGTKTLSYDDQGNLISYIDCSGK-E
TKWTYDKKGRVVAIENALKQKVEYFYSDLTIEQREPIIKGLPLNAFGQL-------------EKIKHADGTE
EHFIHDAEGRLLAHIDQKQNITRYEYDAAGLIASRTDALNHKLKYEWDKLN-RLKRLINENGASYQFFYDVA
SRLVKEIDFDGKETIYNYDETDGNLVSSVEVASMHGQDLRDRIAPKDR----IQKFLFDSMGRLEQRTAGYG
YVEHDLEQQLVEEFAYDSNGNLIQAK------------NAESNLQWYYDAVGNVIREHHQ---DYKTKKTGV
WKHSYDEINDRIKTVRP-DGQ---------KIDWLTY--GSGHVHSLIVNGQDVVSFERDDLHREIARHYAN
G------ISQEQHYDTMGRLTQQNIVNGHE-FGYPSNEQVSQTQNNAIQETQQLIQRLYQYDKTGQLTGI--
--------------------------------NDTRRGNINYKYDPVGRLL------EASSKLGKETFSFDP
ASNILDS------YHSQKV-----------QSHSQKLDETVYGYNRLVNNVVKEYLDQQYQYDVYGQLVCQK
STKGNL-----YLEWDACGRLIK-------SRNAEYTAEYRYDALGRRIQKRSKH-------HHTSGEHNII
YGWDGNTL--AYES----NEQITKHYIYEKDS-FVPLAQAVYAEEIELHQTPDWAD---KPYSLQRDPLWKV
TKTAK--DFKDFWFYHCDHLGTPQEMTDHTGAVIWKAEYKA-WGECKAEKAKSNFFEDSEIISN-NIRFQGQ
YFDQETGLHYNRYRYYSPYVGRFISKDPIGLLGGDNVYAYAPN-PVEWVDPLGL
>AR_0101_22
MPTAPKTISQAKAKPSVRTNTGQVAVVPL--NKLYAADVKAGMNKIDSWLKTNTKGYVDLNTIKSTLGTLPV
IGNAIALVDAIYDVIDISKK---PSPAFTDWLNLGLDVIGIIP----APPTMATMRMSLRPTLNLVRQKAKG

TIS-------------------------DTILVILSDHLNERIAGELDQFAKNAQPLVNQMLQNCGTKITAI
SNDFANGIQKV----LNGKVYSSAGNLKNAQK--QLNKVNLNNLQRNPKATISNAVDGLVNIWKAGVKE-QI
NTVAKGVSAVVPASATQPIQNAITSIRVFGRKAPSYLMALADPAKAMSIAWLLNVLLKAVQKFKLKGTKTAN
IKPTQVTEKRRQKPNGELEKANVQAKSKGNPSETVCALNLRGGKSKPKSTSGTKRNITFALGTECL---THQ
DAYVSSILSFVLIRKYASNLYQLDHGEFGARWITPFTTRIVPQSEYIEETEERAGYLKHLSGFEFIGADARP
VKLPNLKVGESFHNK-TEDFYYSVISE---------KVQM-----ISYQKEEKH--------LFEKYQDGY-
RLSAIEYKNGLTLA---VRYDHHFEGL-SFISDIVIKEKQKQLIHVAFQV--------NASGRIEDVWLVEN
G---QLARPL-------ASYNYNEKGDLVEAITENG-ASYHYQY-DHHLLTRYTDLTGRGMNLEYDGV----
-----------------------EPTSKAYHEWADDGSSDTILQWDENIRLTYVTDAYGAETWYYY---DIE
GYTYRIIYPDGLEEWFFRDDFGRVTKHVDTDAAVTIYDYDEHGNIVSMTQPDGSLLYYAY-DENRQLIGMVD
AEGGRWFKEYDAAGNITKETDPLKRETQYSYNG---LGLLTSIKDPKGGTKTLSYDDQGNLISYIDCSGK-E
TKWTYDKKGRVVAIENALKQKVEYFYSDLTIEQREPIIKGLPLNAFGQL-------------EKIKHADGTE
EHFIHDAEGRLLAHIDQKQNITRYEYDAAGLIASRTDALNHKLKYEWDKLN-RLKRLINENGASYQFFYDVA
SRLVKEIDFDGKETIYNYDETDGNLVSSVEVASMHGQDLRDRIAPKDR----IQKFLFDSMGRLEQRTAGYG
YVEHDLEQQLVEEFAYDSNGNLIQAK------------NAESNLQWYYDAVGNVIREHHQ---DYKTKKTGV
WKHSYDEINDRIKTVRP-DGQ---------KIDWLTY--GSGHVHSLIVNGQDVVSFERDDLHREIARHYAN
G------ISQEQHYDTMGRLTQQNIVNGHE-FGYPSNEQVSQTQNNAIQETQQLIQRLYQYDKTGQLTGI--
--------------------------------NDTRRGNINYKYDPVGRLL------EASSKLGKETFSFDP
ASNILDS------YHSQKV-----------QSHSQKLDETVYGYNRLVNNVVKEYLDQQYQYDVYGQLVCQK
STKGNL-----YLEWDACGRLIK-------SRNAEYTAEYRYDALGRRIQKRSKH-------HHTSGEHNII
YGWDGNTL--AYES----NEQITKHYIYEKDS-FVPLAQAVYAEEIELHQTPDWAD---KPYSLQRDPLWKV
TKTAK--DFKDFWFYHCDHLGTPQEMTDHTGAVIWKAEYKA-WGECKAEKAKSNFFEDSEIISN-NIRFQGQ
YFDQETGLHYNRYRYYSPYVGRFISKDPIGLLGGDNVYAYAPN-PVEWVDPLGL
>IOMTU433_22
MPTAPKTISQAKAKPSVRTNTGQVAVVPL--NKLYAADVKAGMNKIDSWLKTNTKGYVDLNTIKSTLGTLPV
IGNAIALVDAIYDVIDISKK---PSPAFTDWLNLGLDVIGIIP----APPTMATMRMSLRPTLNLVRQKAKG
TIS-------------------------DTILVILSDHLNERIAGELDQFAKNAQPLVNQMLQNCGTKITAI
SNDFANGIQKV----LNGKVYSSAGNLKNAQK--QLNKVNLNNLQRNPKATISNAVDGLVNIWKAGVKE-QI
NTVAKGVSAVVPASATQPIQNAITSIRVFGRKAPSYLMALADPAKAMSIAWLLNVLLKAVQKFKLKGTKTAN
IKPTQVTEKRRQKPNGELEKANVQAKSKGNPSETVCALNLRGGKSKPKSISGTKRNITFALGTECL---THQ
DAYVSSILSFVLTRKYASNLYQLDHGEFGARWITPFTTRIVPQSEYIEETEERAGYLKHLSGFEFIGADARP
VKLPNLKVGESFHNK-TEDFYYSVISE---------KVQM-----ISYQKEEKH--------LFEKYQDGY-
RLSAIEYKNGLTLA---VRYDHHFEGL-SFISDIVIKEKQKQLIHVAFQV--------NASGRIEDVWLVEN

G---QLARPL-------ASYNYNEKGDLVEAITENG-ASYHYQY-DHHLLTRYTDLTGRGMNLEYDGV----
-----------------------EPTSKAYHEWADDGSSDTTLQWDENIRLTYVTDAYGAETWYYY---DIE
GYTYRIIYPDGLEEWFFRDDFGRVTKHVDTDAAVTIYDYDKHGNIVSMTQPDGSLLHYAY-DENRQLIGMVD
AEEGRWFKEYDAAGNITKETDPLKRETQYSYNG---LGLLTSIKDPKGGTKTLSYDDQGNLISYIDCSGK-E
TKWTYDKKGRVVAIENALKQKVEYFYSDLTIEQREPVIKGLPLNAFGQL-------------EKIKHADGTE
EHFIHDAEGRLLAHVDPKQNITRYEYDAAGLIASRTDALNHKLKYEWDKLN-RLKRLINENGASYQFFYDVA
SRLVKEIDFDGKETIYNYDETDGNLVSSVEVASMHGQDLRDRIAPKDR----IQKFLFDSMGRLEQRTAGYG
YVEHDLEQQLVEEFAYDSNGNLIQAK------------NAESNLQWYYDAVGNVILEHHQ---DYKTKKTGV
WKHSYDEINDRIKTVRP-DGQ---------KIDWLTY--GSGHVHSLIVNGQDVVSFERDDLHREIARHYAN
G------ISQEQHYDTMGRLTQQNIVNGHE-FGYPSNEQVSQTQNNAIQETQQLIQRLYQYDKTGQLTGI--
--------------------------------NDTRRGNINYKYDPVGRLL------EASSKLGKETFSFDP
ASNILDS------YHSQKA-----------QSHSQKLDETGYGYNRLVNNVVKEYLDQQYQYDVYGQLVCQK
STKGNL-----YLEWDACGRLIK-------SRNAEYTAEYRYDALGRRIQKRSKH-------HHTGDEHNII
YGWDGNTL--AYES----NEQITKHYIYEKDS-FVPLAQAVYAEEIELHQTPDWAD---KPYSLQRDPLWKV
TKTPK--DFKEFWFYHCDHLGTPQEMTDHTGAVIWKAEYKA-WGECKAEKAKSNFFEDSEIISN-NIRFQGQ
YFDQETGLHYNRYRYYSPYVGRFISKDPIGLLGGDNVYAYAPN-PVEWVDPLGL
>AB031_27
MPTAPKTISQAKAKPSVRTNTGQVAVVPL--NKLYAADVKAGMNKIDSWLKTNTKGYVDLNTIKSTLGTLPV
IGNAIALVDAIYDVIDISKK---PSPAFTDWLNLGLDVIGIIP----APPTMATMRMSLRPTLNLVRQKAKG
TIS-------------------------DTILVILSDHLNERIAGELDQFAKNAQPLVNQMLQNCGTKITAI
SNDFANGIQKV----LNGKVYSSAGNLKNAQK--QLNKVNLNNLQRNPKATISNAVDGLVNIWKAGVKE-QI
NTVAKGVSAVVPASATQPIQNAITSIRVFGRKAPSYLMALADPAKAMSIAWLLNVLLKAVQKFKLKGTKTAN
IKPTQVTEKRRQKPNGELEKANVQAKSKGNPSETVCALNLRGGKSKPKSTSGTKRNITFALGTECL---THQ
DAYVSSILSFVLTRKYASNLYQLDHGEFGARWITPFTTRIVPQSEYIEETEERAGYLKHLSGFEFIGADARP
VKLPNLKVGESFHNK-TEDFYYSVISE---------KVQM-----ISYQKEEKH--------LFEKYQDGY-
RLSAIEYKNGLTLA---VRYDHHFEGL-SFISDIIIKEKQKQLIHVAFQV--------NASGRIEDVWLVEN
G---QLARPL-------ASYNYNEKGDLVEAITENG-ASYHYQY-DHHLLTRYTDLTGRGMNLEYDGV----
-----------------------EPTSKAYHEWADDGSSDTTLQWDENIRLTYVTDAYGAETWYYY---DIE
GYTYRIIYPDGLEEWFFRDDFGRVTKHVDTDAAVTIYDYDEHGNIVSMTQPDGSLLHYAY-DENRQLIGMVD
AEGGRWFKEYDAAGNITKETDPLKRETQYSYNG---LGLLTSIKDPKGGTKTLSYDDQGNLISYIDCSGK-E
TKWTYDKKGRVVAIENALKQKVEYFYSDLTIEQREPVIKGLPLNAFGQL-------------EKIKHADGTE
EHFIHDAEGRLLAHIDPKQNITRYEYDAAGLIASRTDALNHKLKYEWDKLN-RLKRLINENGASYQFFYDVA
SRLVKEIDFDGKETIYNYDETDGNLVSSVEVASMHGQDLRDRIAPKDR----IQKFLFDSMGRLEQRTAGYG

```
YVEHDLEQQLVEEFAYDSNGNLIQAK------------NAESNLQWYYDAVGNVIREHHQ---DYKTKKTGV
WKHSYDEINDRIKTVRP-DGQ---------KIDWLTY--GSGHVHSLIVNGQDVVSFERDDLHREIARHYAN
G------ISQEQHYDTMGRLTQQNIVNGHE-FGYPSNEQVS--QNNAIQETQQLIQRLYQYDKTGQLTGI--
--------------------------------KDTRRGNINYKYDPVGRLL------EASSKLGKETFSFDP
ASNILDS------YHSQKV-----------QSHSQKLEETGFGYNRLVNNVVKEYLDQQYQYDVYGQLVCQK
STKGNL-----YLEWDACGRLIK-------SRNAEYTAEYRYDALGRRIQKRSKH-------HHTGDEHNII
YGWDGNTL--AYES----NEQITKHYIYEKDS-FVPLAQAVYAEEIELHQTPDWAD---KPYSLQCDPLWKV
TKTAK--DFKDFWFYHCDHLGTPQEMTDHTGAVIWKAEYKA-WGECKAERAKSNFFENSEIISN-NIRFQGQ
YFDEETGLHYNRYRYYSPYVGRFISKDPIGLLGGFNGYVYAPN-PIGWVDPYGL
>A1_16
---MSQN-SVVAPLNTFSPKDLTAKKAE-----------------VDKWFREYTNGVVTVDRLETICRSVPV
LGSAFAIGDIIIDIISMINKGGLDKVEIFDWLNLGIDVIGLVP----MGPVGPSVRSAARPALFYVKNESEK
IIKAQAKKLGKKTLTSQEVKKALSTGFKDSASVFLTTIIAENVAGTLENFAKKGQSLLNQILKEVGNWIVLL
TKTIDDGFKKLVSGSLNGLPNLKRAGQQSLGV--------IKGIFELDGTRIVNNAKYATENVAKTVGK---
-GYVNLANLAVSDEARAKVLALGAKIRSIGQVAQAKVNGLSDPNTLWTIGWLFSIMGMVAAKHRQKR---AQ
IKAKETTKANASHPSTATDKSNKQAHAENNANQC------------KNCMGGTGGSITFAMGTEFF---THV
DAQLGGIIQDSISRTYVSNLYQMDDAIFGARWVTPFTTKISRKFKYTSKKKDHKDY---LNGLEYICLDGRA
IDLPDLKKGQSIYDP-IEQYTYTVLSD---------QLHL-----IAYGEDEKR--------YYEKYGEDY-
RLSYIERKNGFKVA---LRYDHVSTDNKTILSDILFKQDDNLLAHLALQL--------TPQGLVSDIWTIKN
G---QLDRVL-------ASYDYDQQGDLVQATNEFA-ASYYYQY-THHLITRYTDLTHRGMNLKWDGI----
-----------------------LPTSKAIEEWADNASRASKLEWDKNIRKTTVLDVEGNSTEHYY---DID
GYTYRIVYPDNFEEWFFRDDAKNITLHIAKDGSKTSYTYDERGNVLTTTQDDGATSYFEY-DEKNQLTGMVD
AEQGRWFKQYDGSGNLIKEIDPLKHETAYVYNA---MGLVTSITDAKGGSKSLKYDDQGNLISYTDCSGK-E
TKWQYDERGRVISIENALNQKVEYFYTELTLENREPIIKGLPLNAFGQL-------------EKIKHADGTE
EHFIHDAEGRLLAHVDPKQNITRYEYDEAGLILSRTDALNHKLKYKWDRLG-RLTRLINENGASYQFFYDVA
SRLVKEIDFDGKETVYHYDEKSGQLATSIEVASAYGQDLKDRAAPKDR----IQQFIFDSMGRLEQRTAGYG
HYGLELEEKQTEEFAYDYMGRIIQAK------------NAQSNLQWFYDAAGNLVQEHQQ---DYKINKTAV
WKHQYDEINDRIKTTRP-DGQ---------VIDWLTY--GSGHVQSLIVNGQDFVSFERDDLHREIARHYAN
G------VSQEQQYDLAGRLKSQMMLSEHE-NGYQNQYK---RHNNALEQTSQLVQRLYQYDKTGELTAI--
--------------------------------RDTRRGNIAYKYDPVGRLL------EASSKLGKETFSFDP
ASNILDS------YHSQKV-----------QSHSQKLDETSYGYNRLVNNVVKEYLDQQYQYDAYGQLIRQK
TSQGDL-----NLEWDVYGRMVK-------SRNSQYTAEYRYDALGRRIQKWSKH-------HHTGQEQNII
YGWDGDTL--AYES----TEELTKHYIYEKDS-FVPMLQAVYLSPIELHQTPDWSD---RPYNIHRDPLWKT
```

EKEGK--EFDDVWFYHCDHLGTPQEMTDHTGAIIWKAEYKA-WGECKAEEAKSNFFENSEIISN-NIRFQGQ
YFDEETGLHYNRYRYYSPYVGRFVSKDPIGLLGGNNVYVYAKN-PITWIDSKGL
>A388_16
---MSQN-SVVAPLNTFSPKDLTAKKAE-----------------VDKWFREYTNGVVTVDRLETICRSVPV
LGSAFAIGDIIIDIISMINKGGLDKVEIFDWLNLGIDVIGLVP----MGPVGPSVRSAARPALFYVKNESEK
IIKAQAKKLGKKTLTSQEVKKALSTGFKDSASVFLTTIIAENVAGTLENFAKKGQSLLNQILKEVGNWIVLL
TKTIDDGFKKLVSGSLNGLPNLKRAGQQSLGV--------IKGIFELDGTRIVNNAKYATENVAKTVGK---
-GYVNLANLAVSDEARAKVLALGAKIRSIGQVAQAKVNGLSDPNTLWTIGWLFSIMGMVAAKHRQKR---AQ
IKAKETTKANASHPSTATDKSNKQAHAENNANQC------------KNCMGGTGGSITFAMGTEFF---THV
DAQLGGIIQDSISRTYVSNLYQMDDAIFGARWVTPFTTKISRKFKYTSKKKDHKDY---LNGLEYICLDGRA
IDLPDLKKGQSIYDP-IEQYTYTVLSD---------QLHL-----IAYGEDEKR--------YYEKYGEDY-
RLSYIERKNGFKVA---LRYDHVSTDNKTILSDILFKQDDNLLAHLALQL--------TPQGLVSDIWTIKN
G---QLDRVL-------ASYDYDQQGDLVQATNEFA-ASYYYQY-THHLITRYTDLTHRGMNLKWDGI----
-----------------------LPTSKAIEEWADNASRASKLEWDKNIRKTTVLDVEGNSTEHYY---DID
GYTYRIVYPDNFEEWFFRDDAKNITLHIAKDGSKTSYTYDERGNVLTTTQDDGATSYFEY-DEKNQLTGMVD
AEQGRWFKQYDGSGNLIKEIDPLKHETAYVYNA---MGLVTSITDAKGGSKSLKYDDQGNLISYTDCSGK-E
TKWQYDERGRVISIENALNQKVEYFYTELTLENREPIIKGLPLNAFGQL-------------EKIKHADGTE
EHFIHDAEGRLLAHVDPKQNITRYEYDEAGLILSRTDALNHKLKYKWDRLG-RLTRLINENGASYQFFYDVA
SRLVKEIDFDGKETVYHYDEKSGQLATSIEVASAYGQDLKDRAAPKDR----IQQFIFDSMGRLEQRTAGYG
HYGLELEEKQTEEFAYDYMGRIIQAK------------NAQSNLQWFYDAAGNLVQEHQQ---DYKINKTAV
WKHQYDEINDRIKTTRP-DGQ---------VIDWLTY--GSGHVQSLIVNGQDFVSFERDDLHREIARHYAN
G------VSQEQQYDLAGRLKSQMMLSEHE-NGYQNQYK---RHNNALEQTSQLVQRLYQYDKTGELTAI--
--------------------------------RDTRRGNIAYKYDPVGRLL------EASSKLGKETFSFDP
ASNILDS------YHSQKV-----------QSHSQKLDETSYGYNRLVNNVVKEYLDQQYQYDAYGQLIRQK
TSQGDL-----NLEWDVYGRMVK-------SRNSQYTAEYRYDALGRRIQKWSKH-------HHTGQEQNII
YGWDGDTL--AYES----TEELTKHYIYEKDS-FVPMLQAVYLSPIELHQTPDWSD---RPYNIHRDPLWKT
EKEGK--EFDDVWFYHCDHLGTPQEMTDHTGAIIWKAEYKA-WGECKAEEAKSNFFENSEIISN-NIRFQGQ
YFDEETGLHYNRYRYYSPYVGRFVSKDPIGLLGGNNVYVYAKN-PITWIDSKGL
>AB0057_16
---MSQN-SVVAPLNTFSPKDLTAKKAE-----------------VDKWFREYTNGVVTVDRLETICRSVPV
LGSAFAIGDIIIDIISMINKGGLDKVEIFDWLNLGIDVIGLVP----MGPVGPSVRSAARPALFYVKNESEK
IIKAQAKKLGKKTLTSQEVKKALSTGFKDSASVFLTTIIAENVAGTLENFAKKGQSLLNQILKEVGNWIVLL
TKTIDDGFKKLVSGSLNGLPNLKRAGQQSLGV--------IKGIFELDGTRIVNNAKYATENVAKTVGK---

-GYVNLANLAVSDEARAKVLALGAKIRSIGQVAQAKVNGLSDPNTLWTIGWLFSIMGMVAAKHRQKR---AQ
IKAKETTKANASHPSTATDKSNKQAHAENNANQC------------KNCMGGTGGSITFAMGTEFF---THV
DAQLGGIIQDSISRTYVSNLYQMDDAIFGARWVTPFTTKISRKFKYTSKKKDHKDY---LNGLEYICLDGRA
IDLPDLKKGQSIYDP-IEQYTYTVLSD---------QLHL-----IAYGEDEKR--------YYEKYGEDY-
RLSYIERKNGFKVA---LRYDHVSTDNKTILSDILFKQDDNLLAHLALQL--------TPQGLVSDIWTIKN
G---QLDRVL-------ASYDYDQQGDLVQATNEFA-ASYYYQY-THHLITRYTDLTHRGMNLKWDGI----
-----------------------LPTSKAIEEWADNASRASKLEWDKNIRKTTVLDVEGNSTEHYY---DID
GYTYRIVYPDNFEEWFFRDDAKNITLHIAKDGSKTSYTYDERGNVLTTTQDDGATSYFEY-DEKNQLTGMVD
AEQGRWFKQYDGSGNLIKEIDPLKHETAYVYNA---MGLVTSITDAKGGSKSLKYDDQGNLISYTDCSGK-E
TKWQYDERGRVISIENALNQKVEYFYTELTLENREPIIKGLPLNAFGQL-------------EKIKHADGTE
EHFIHDAEGRLLAHVDPKQNITRYEYDEAGLILSRTDALNHKLKYKWDRLG-RLTRLINENGASYQFFYDVA
SRLVKEIDFDGKETVYHYDEKSGQLATSIEVASAYGQDLKDRAAPKDR----IQQFIFDSMGRLEQRTAGYG
HYGLELEEKQTEEFAYDYMGRIIQAK------------NAQSNLQWFYDAAGNLVQEHQQ---DYKINKTAV
WKHQYDEINDRIKTTRP-DGQ---------VIDWLTY--GSGHVQSLIVNGQDFVSFERDDLHREIARHYAN
G------VSQEQQYDLAGRLKSQMMLSEHE-NGYQNQYK---RHNNALEQTSQLVQRLYQYDKTGELTAI--
--------------------------------RDTRRGNIAYKYDPVGRLL------EASSKLGKETFSFDP
ASNILDS------YHSQKV-----------QSHSQKLDETSYGYNRLVNNVVKEYLDQQYQYDAYGQLIRQK
TSQGDL-----NLEWDVYGRMVK-------SRNSQYTAEYRYDALGRRIQKWSKH-------HHTGQEQNII
YGWDGDTL--AYES----TEELTKHYIYEKDS-FVPMLQAVYLSPIELHQTPDWSD---RPYNIHRDPLWKT
EKEGK--EFDDVWFYHCDHLGTPQEMTDHTGAIIWKAEYKA-WGECKAEEAKSNFFENSEIISN-NIRFQGQ
YFDEETGLHYNRYRYYSPYVGRFVSKDPIGLLGGNNVYVYAKN-PITWIDSKGL
>AB307-0294_16
---MSQN-SVVAPLNTFSPKDLTAKKAE-----------------VDKWFREYTNGVVTVDRLETICRSVPV
LGSAFAIGDIIIDIISMINKGGLDKVEIFDWLNLGIDVIGLVP----MGPVGPSVRSAARPALFYVKNESEK
IIKAQAKKLGKKTLTSQEVKKALSTGFKDSASVFLTTIIAENVAGTLENFAKKGQSLLNQILKEVGNWIVLL
TKTIDDGFKKLVSGSLNGLPNLKRAGQQSLGV--------IKGIFELDGTRIVNNAKYATENVAKTVGK---
-GYVNLANLAVSDEARAKVLALGAKIRSIGQVAQAKVNGLSDPNTLWTIGWLFSIMGMVAAKHRQKR---AQ
IKAKETTKANASHPSTATDKSNKQAHAENNANQC------------KNCMGGTGGSITFAMGTEFF---THV
DAQLGGIIQDSISRTYVSNLYQMDDAIFGARWVTPFTTKISRKFKYTSKKKDHKDY---LNGLEYICLDGRA
IDLPDLKKGQSIYDP-IEQYTYTVLSD---------QLHL-----IAYGEDEKR--------YYEKYGEDY-
RLSYIERKNGFKVA---LRYDHVSTDNKTILSDILFKQDDNLLAHLALQL--------TPQGLVSDIWTIKN
G---QLDRVL-------ASYDYDQQGDLVQATNEFA-ASYYYQY-THHLITRYTDLTHRGMNLKWDGI----
-----------------------LPTSKAIEEWADNASRASKLEWDKNIRKTTVLDVEGNSTEHYY---DID

GYTYRIVYPDNFEEWFFRDDAKNITLHIAKDGSKTSYTYDERGNVLTTTQDDGATSYFEY-DEKNQLTGMVD
AEQGRWFKQYDGSGNLIKEIDPLKHETAYVYNA---MGLVTSITDAKGGSKSLKYDDQGNLISYTDCSGK-E
TKWQYDERGRVISIENALNQKVEYFYTELTLENREPIIKGLPLNAFGQL-------------EKIKHADGTE
EHFIHDAEGRLLAHVDPKQNITRYEYDEAGLILSRTDALNHKLKYKWDRLG-RLTRLINENGASYQFFYDVA
SRLVKEIDFDGKETVYHYDEKSGQLATSIEVASAYGQDLKDRAAPKDR----IQQFIFDSMGRLEQRTAGYG
HYGLELEEKQTEEFAYDYMGRIIQAK------------NAQSNLQWFYDAAGNLVQEHQQ---DYKINKTAV
WKHQYDEINDRIKTTRP-DGQ---------VIDWLTY--GSGHVQSLIVNGQDFVSFERDDLHREIARHYAN
G------VSQEQQYDLAGRLKSQMMLSEHE-NGYQNQYK---RHNNALEQTSQLVQRLYQYDKTGELTAI--
--------------------------------RDTRRGNIAYKYDPVGRLL------EASSKLGKETFSFDP
ASNILDS------YHSQKV-----------QSHSQKLDETSYGYNRLVNNVVKEYLDQQYQYDAYGQLIRQK
TSQGDL-----NLEWDVYGRMVK-------SRNSQYTAEYRYDALGRRIQKWSKH-------HHTGQEQNII
YGWDGDTL--AYES----TEELTKHYIYEKDS-FVPMLQAVYLSPIELHQTPDWSD---RPYNIHRDPLWKT
EKEGK--EFDDVWFYHCDHLGTPQEMTDHTGAIIWKAEYKA-WGECKAEEAKSNFFENSEIISN-NIRFQGQ
YFDEETGLHYNRYRYYSPYVGRFVSKDPIGLLGGNNVYVYAKN-PITWIDSKGL
>AB5075-UW_16
---MSQN-SVVAPLNTFSPKDLTAKKAE-----------------VDKWFREYTNGVVTVDRLETICRSVPV
LGSAFAIGDIIIDIISMINKGGLDKVEIFDWLNLGIDVIGLVP----MGPVGPSVRSAARPALFYVKNESEK
IIKAQAKKLGKKTLTSQEVKKALSTGFKDSASVFLTTIIAENVAGTLENFAKKGQSLLNQILKEVGNWIVLL
TKTIDDGFKKLVSGSLNGLPNLKRAGQQSLGV--------IKGIFELDGTRIVNNAKYATENVAKTVGK---
-GYVNLANLAVSDEARAKVLALGAKIRSIGQVAQAKVNGLSDPNTLWTIGWLFSIMGMVAAKHRQKR---AQ
IKAKETTKANASHPSTATDKSNKQAHAENNANQC------------KNCMGGTGGSITFAMGTEFF---THV
DAQLGGIIQDSISRTYVSNLYQMDDAIFGARWVTPFTTKISRKFKYTSKKKDHKDY---LNGLEYICLDGRA
IDLPDLKKGQSIYDP-IEQYTYTVLSD---------QLHL-----IAYGEDEKR--------YYEKYGEDY-
RLSYIERKNGFKVA---LRYDHVSTDNKTILSDILFKQDDNLLAHLALQL--------TPQGLVSDIWTIKN
G---QLDRVL-------ASYDYDQQGDLVQATNEFA-ASYYYQY-THHLITRYTDLTHRGMNLKWDGI----
-----------------------LPTSKAIEEWADNASRASKLEWDKNIRKTTVLDVEGNSTEHYY---DID
GYTYRIVYPDNFEEWFFRDDAKNITLHIAKDGSKTSYTYDERGNVLTTTQDDGATSYFEY-DEKNQLTGMVD
AEQGRWFKQYDGSGNLIKEIDPLKHETAYVYNA---MGLVTSITDAKGGSKSLKYDDQGNLISYTDCSGK-E
TKWQYDERGRVISIENALNQKVEYFYTELTLENREPIIKGLPLNAFGQL-------------EKIKHADGTE
EHFIHDAEGRLLAHVDPKQNITRYEYDEAGLILSRTDALNHKLKYKWDRLG-RLTRLINENGASYQFFYDVA
SRLVKEIDFDGKETVYHYDEKSGQLATSIEVASAYGQDLKDRAAPKDR----IQQFIFDSMGRLEQRTAGYG
HYGLELEEKQTEEFAYDYMGRIIQAK------------NAQSNLQWFYDAAGNLVQEHQQ---DYKINKTAV
WKHQYDEINDRIKTTRP-DGQ---------VIDWLTY--GSGHVQSLIVNGQDFVSFERDDLHREIARHYAN

G------VSQEQQYDLAGRLKSQMMLSEHE-NGYQNQYK---RHNNALEQTSQLVQRLYQYDKTGELTAI--
---------------------------------RDTRRGNIAYKYDPVGRLL------EASSKLGKETFSFDP
ASNILDS------YHSQKV-----------QSHSQKLDETSYGYNRLVNNVVKEYLDQQYQYDAYGQLIRQK
TSQGDL-----NLEWDVYGRMVK-------SRNSQYTAEYRYDALGRRIQKWSKH-------HHTGQEQNII
YGWDGDTL--AYES----TEELTKHYIYEKDS-FVPMLQAVYLSPIELHQTPDWSD---RPYNIHRDPLWKT
EKEGK--EFDDVWFYHCDHLGTPQEMTDHTGAIIWKAEYKA-WGECKAEEAKSNFFENSEIISN-NIRFQGQ
YFDEETGLHYNRYRYYSPYVGRFVSKDPIGLLGGNNVYVYAKN-PITWIDSKGL
>AR_0083_16
---MSQN-SVVAPLNTFSPKDLTAKKAE-----------------VDKWFREYTNGVVTVDRLETICRSVPV
LGSAFAIGDIIIDIISMINKGGLDKVEIFDWLNLGIDVIGLVP----MGPVGPSVRSAARPALFYVKNESEK
IIKAQAKKLGKKTLTSQEVKKALSTGFKDSASVFLTTIIAENVAGTLENFAKKGQSLLNQILKEVGNWIVLL
TKTIDDGFKKLVSGSLNGLPNLKRAGQQSLGV--------IKGIFELDGTRIVNNAKYATENVAKTVGK---
-GYVNLANLAVSDEARAKVLALGAKIRSIGQVAQAKVNGLSDPNTLWTIGWLFSIMGMVAAKHRQKR---AQ
IKAKETTKANASHPSTATDKSNKQAHAENNANQC------------KNCMGGTGGSITFAMGTEFF---THV
DAQLGGIIQDSISRTYVSNLYQMDDAIFGARWVTPFTTKISRKFKYTSKKKDHKDY---LNGLEYICLDGRA
IDLPDLKKGQSIYDP-IEQYTYTVLSD---------QLHL-----IAYGEDEKR--------YYEKYGEDY-
RLSYIERKNGFKVA---LRYDHVSTDNKTILSDILFKQDDNLLAHLALQL--------TPQGLVSDIWTIKN
G---QLDRVL-------ASYDYDQQGDLVQATNEFA-ASYYYQY-THHLITRYTDLTHRGMNLKWDGI----
-----------------------LPTSKAIEEWADNASRASKLEWDKNIRKTTVLDVEGNSTEHYY---DID
GYTYRIVYPDNFEEWFFRDDAKNITLHIAKDGSKTSYTYDERGNVLTTTQDDGATSYFEY-DEKNQLTGMVD
AEQGRWFKQYDGSGNLIKEIDPLKHETAYVYNA---MGLVTSITDAKGGSKSLKYDDQGNLISYTDCSGK-E
TKWQYDERGRVISIENALNQKVEYFYTELTLENREPIIKGLPLNAFGQL-------------EKIKHADGTE
EHFIHDAEGRLLAHVDPKQNITRYEYDEAGLILSRTDALNHKLKYKWDRLG-RLTRLINENGASYQFFYDVA
SRLVKEIDFDGKETVYHYDEKSGQLATSIEVASAYGQDLKDRAAPKDR----IQQFIFDSMGRLEQRTAGYG
HYGLELEEKQTEEFAYDYMGRIIQAK------------NAQSNLQWFYDAAGNLVQEHQQ---DYKINKTAV
WKHQYDEINDRIKTTRP-DGQ---------VIDWLTY--GSGHVQSLIVNGQDFVSFERDDLHREIARHYAN
G------VSQEQQYDLAGRLKSQMMLSEHE-NGYQNQYK---RHNNALEQTSQLVQRLYQYDKTGELTAI--
---------------------------------RDTRRGNIAYKYDPVGRLL------EASSKLGKETFSFDP
ASNILDS------YHSQKV-----------QSHSQKLDETSYGYNRLVNNVVKEYLDQQYQYDAYGQLIRQK
TSQGDL-----NLEWDVYGRMVK-------SRNSQYTAEYRYDALGRRIQKWSKH-------HHTGQEQNII
YGWDGDTL--AYES----TEELTKHYIYEKDS-FVPMLQAVYLSPIELHQTPDWSD---RPYNIHRDPLWKT
EKEGK--EFDDVWFYHCDHLGTPQEMTDHTGAIIWKAEYKA-WGECKAEEAKSNFFENSEIISN-NIRFQGQ
YFDEETGLHYNRYRYYSPYVGRFVSKDPIGLLGGNNVYVYAKN-PITWIDSKGL

>D36_16
---MSQN-SVVAPLNTFSPKDLTAKKAE-----------------VDKWFREYTNGVVTVDRLETICRSVPV
LGSAFAIGDIIIDIISMINKGGLDKVEIFDWLNLGIDVIGLVP----MGPVGPSVRSAARPALFYVKNESEK
IIKAQAKKLGKKTLTSQEVKKALSTGFKDSASVFLTTIIAENVAGTLENFAKKGQSLLNQILKEVGNWIVLL
TKTIDDGFKKLVSGSLNGLPNLKRAGQQSLGV--------IKGIFELDGTRIVNNAKYATENVAKTVGK---
-GYVNLANLAVSDEARAKVLALGAKIRSIGQVAQAKVNGLSDPNTLWTIGWLFSIMGMVAAKHRQKR---AQ
IKAKETTKANASHPSTATDKSNKQAHAENNANQC------------KNCMGGTGGSITFAMGTEFF---THV
DAQLGGIIQDSISRTYVSNLYQMDDAIFGARWVTPFTTKISRKFKYTSKKKDHKDY---LNGLEYICLDGRA
IDLPDLKKGQSIYDP-IEQYTYTVLSD---------QLHL-----IAYGEDEKR--------YYEKYGEDY-
RLSYIERKNGFKVA---LRYDHVSTDNKTILSDILFKQDDNLLAHLALQL--------TPQGLVSDIWTIKN
G---QLDRVL-------ASYDYDQQGDLVQATNEFA-ASYYYQY-THHLITRYTDLTHRGMNLKWDGI----
-----------------------LPTSKAIEEWADNASRASKLEWDKNIRKTTVLDVEGNSTEHYY---DID
GYTYRIVYPDNFEEWFFRDDAKNITLHIAKDGSKTSYTYDERGNVLTTTQDDGATSYFEY-DEKNQLTGMVD
AEQGRWFKQYDGSGNLIKEIDPLKHETAYVYNA---MGLVTSITDAKGGSKSLKYDDQGNLISYTDCSGK-E
TKWQYDERGRVISIENALNQKVEYFYTELTLENREPIIKGLPLNAFGQL-------------EKIKHADGTE
EHFIHDAEGRLLAHVDPKQNITRYEYDEAGLILSRTDALNHKLKYKWDRLG-RLTRLINENGASYQFFYDVA
SRLVKEIDFDGKETVYHYDEKSGQLATSIEVASAYGQDLKDRAAPKDR----IQQFIFDSMGRLEQRTAGYG
HYGLELEEKQTEEFAYDYMGRIIQAK------------NAQSNLQWFYDAAGNLVQEHQQ---DYKINKTAV
WKHQYDEINDRIKTTRP-DGQ---------VIDWLTY--GSGHVQSLIVNGQDFVSFERDDLHREIARHYAN
G------VSQEQQYDLAGRLKSQMMLSEHE-NGYQNQYK---RHNNALEQTSQLVQRLYQYDKTGELTAI--
--------------------------------RDTRRGNIAYKYDPVGRLL------EASSKLGKETFSFDP
ASNILDS------YHSQKV-----------QSHSQKLDETSYGYNRLVNNVVKEYLDQQYQYDAYGQLIRQK
TSQGDL-----NLEWDVYGRMVK-------SRNSQYTAEYRYDALGRRIQKWSKH-------HHTGQEQNII
YGWDGDTL--AYES----TEELTKHYIYEKDS-FVPMLQAVYLSPIELHQTPDWSD---RPYNIHRDPLWKT
EKEGK--EFDDVWFYHCDHLGTPQEMTDHTGAIIWKAEYKA-WGECKAEEAKSNFFENSEIISN-NIRFQGQ
YFDEETGLHYNRYRYYSPYVGRFVSKDPIGLLGGNNVYVYAKN-PITWIDSKGL
>USA15_16
---MSQN-SVVAPLNTFSPKDLTAKKAE-----------------VDKWFREYTNGVVTVDRLETICRSVPV
LGSAFAIGDIIIDIISMINKGGLDKVEIFDWLNLGIDVIGLVP----MGPVGPSVRSAARPALFYVKNESEK
IIKAQAKKLGKKTLTSQEVKKALSTGFKDSASVFLTTIIAENVAGTLENFAKKGQSLLNQILKEVGNWIVLL
TKTIDDGFKKLVSGSLNGLPNLKRAGQQSLGV--------IKGIFELDGTRIVNNAKYATENVAKTVGK---
-GYVNLANLAVSDEARAKVLALGAKIRSIGQVAQAKVNGLSDPNTLWTIGWLFSIMGMVAAKHRQKR---AQ
IKAKETTKANASHPSTATDKSNKQAHAENNANQC------------KNCMGGTGGSITFAMGTEFF---THV

DAQLGGIIQDSISRTYVSNLYQMDDAIFGARWVTPFTTKISRKFKYTSKKKDHKDY---LNGLEYICLDGRA
IDLPDLKKGQSIYDP-IEQYTYTVLSD---------QLHL-----IAYGEDEKR--------YYEKYGEDY-
RLSYIERKNGFKVA---LRYDHVSTDNKTILSDILFKQDDNLLAHLALQL--------TPQGLVSDIWTIKN
G---QLDRVL-------ASYDYDQQGDLVQATNEFA-ASYYYQY-THHLITRYTDLTHRGMNLKWDGI----
-----------------------LPTSKAIEEWADNASRASKLEWDKNIRKTTVLDVEGNSTEHYY---DID
GYTYRIVYPDNFEEWFFRDDAKNITLHIAKDGSKTSYTYDERGNVLTTTQDDGATSYFEY-DEKNQLTGMVD
AEQGRWFKQYDGSGNLIKEIDPLKHETAYVYNA---MGLVTSITDAKGGSKSLKYDDQGNLISYTDCSGK-E
TKWQYDERGRVISIENALNQKVEYFYTELTLENREPIIKGLPLNAFGQL-------------EKIKHADGTE
EHFIHDAEGRLLAHVDPKQNITRYEYDEAGLILSRTDALNHKLKYKWDRLG-RLTRLINENGASYQFFYDVA
SRLVKEIDFDGKETVYHYDEKSGQLATSIEVASAYGQDLKDRAAPKDR----IQQFIFDSMGRLEQRTAGYG
HYGLELEEKQTEEFAYDYMGRIIQAK------------NAQSNLQWFYDAAGNLVQEHQQ---DYKINKTAV
WKHQYDEINDRIKTTRP-DGQ---------VIDWLTY--GSGHVQSLIVNGQDFVSFERDDLHREIARHYAN
G------VSQEQQYDLAGRLKSQMMLSEHE-NGYQNQYK---RHNNALEQTSQLVQRLYQYDKTGELTAI--
--------------------------------RDTRRGNIAYKYDPVGRLL------EASSKLGKETFSFDP
ASNILDS------YHSQKV-----------QSHSQKLDETSYGYNRLVNNVVKEYLDQQYQYDAYGQLIRQK
TSQGDL-----NLEWDVYGRMVK-------SRNSQYTAEYRYDALGRRIQKWSKH-------HHTGQEQNII
YGWDGDTL--AYES----TEELTKHYIYEKDS-FVPMLQAVYLSPIELHQTPDWSD---RPYNIHRDPLWKT
EKEGK--EFDDVWFYHCDHLGTPQEMTDHTGAIIWKAEYKA-WGECKAEEAKSNFFENSEIISN-NIRFQGQ
YFDEETGLHYNRYRYYSPYVGRFVSKDPIGLLGGNNVYVYAKN-PITWIDSKGL
>WCHAB005078_16
---MSQN-SVVAPLNTFSPKDLTAKKAE-----------------VDKWFREYTNGVVTVDRLETICRSVPV
LGSAFAIGDIIIDIISMINKGGLDKVEIFDWLNLGIDVIGLVP----MGPVGPSVRSAARPALFYVKNESEK
IIKAQAKKLGKKTLTSQEVKKALSTGFKDSASVFLTTIIAENVAGTLENFAKKGQSLLNQILKEVGNWIVLL
TKTIDDGFKKLVSGSLNGLPNLKRAGQQSLGV--------IKGIFELDGTRIVNNAKYATENVAKTVGK---
-GYVNLANLAVSDEARAKVLALGAKIRSIGQVAQAKVNGLSDPNTLWTIGWLFSIMGMVAAKHRQKR---AQ
IKAKETTKANASHPSTATDKSNKQAHAENNANQC------------KNCMGGTGGSITFAMGTEFF---THV
DAQLGGIIQDSISRTYVSNLYQMDDAIFGARWVTPFTTKISRKFKYTSKKKDHKDY---LNGLEYICLDGRA
IDLPDLKKGQSIYDP-IEQYTYTVLSD---------QLHL-----IAYGEDEKR--------YYEKYGEDY-
RLSYIERKNGFKVA---LRYDHVSTDNKTILSDILFKQDDNLLAHLALQL--------TPQGLVSDIWTIKN
G---QLDRVL-------ASYDYDQQGDLVQATNEFA-ASYYYQY-THHLITRYTDLTHRGMNLKWDGI----
-----------------------LPTSKAIEEWADNASRASKLEWDKNIRKTTVLDVEGNSTEHYY---DID
GYTYRIVYPDNFEEWFFRDDAKNITLHIAKDGSKTSYTYDERGNVLTTTQDDGATSYFEY-DEKNQLTGMVD
AEQGRWFKQYDGSGNLIKEIDPLKHETAYVYNA---MGLVTSITDAKGGSKSLKYDDQGNLISYTDCSGK-E

TKWQYDERGRVISIENALNQKVEYFYTELTLENREPIIKGLPLNAFGQL-------------EKIKHADGTE
EHFIHDAEGRLLAHVDPKQNITRYEYDEAGLILSRTDALNHKLKYKWDRLG-RLTRLINENGASYQFFYDVA
SRLVKEIDFDGKETVYHYDEKSGQLATSIEVASAYGQDLKDRAAPKDR----IQQFIFDSMGRLEQRTAGYG
HYGLELEEKQTEEFAYDYMGRIIQAK------------NAQSNLQWFYDAAGNLVQEHQQ---DYKINKTAV
WKHQYDEINDRIKTTRP-DGQ---------VIDWLTY--GSGHVQSLIVNGQDFVSFERDDLHREIARHYAN
G------VSQEQQYDLAGRLKSQMMLSEHE-NGYQNQYK---RHNNALEQTSQLVQRLYQYDKTGELTAI--
--------------------------------RDTRRGNIAYKYDPVGRLL------EASSKLGKETFSFDP
ASNILDS------YHSQKV-----------QSHSQKLDETSYGYNRLVNNVVKEYLDQQYQYDAYGQLIRQK
TSQGDL-----NLEWDVYGRMVK-------SRNSQYTAEYRYDALGRRIQKWSKH-------HHTGQEQNII
YGWDGDTL--AYES----TEELTKHYIYEKDS-FVPMLQAVYLSPIELHQTPDWSD---RPYNIHRDPLWKT
EKEGK--EFDDVWFYHCDHLGTPQEMTDHTGAIIWKAEYKA-WGECKAEEAKSNFFENSEIISN-NIRFQGQ
YFDEETGLHYNRYRYYSPYVGRFVSKDPIGLLGGNNVYVYAKN-PITWIDSKGL
>A85_16
---MSQN-SVVAPLNTFSPKDLTAKKAE-----------------VDKWFREYTNGVVTVDRLETICRSVPV
LGSAFAIGDIIIDIISMINKGGLDKVEIFDWLNLGIDVIGLVP----MGPVGPSVRSAARPALFYVKNESEI
IIKAQAKKLGKKTLTSQEVKKALSTGFKDSASVFLTTIIAENVAGTLENFAKKGQSLLNQILKEVGNWIVLL
TKTIDDGFKKLVSGSLNGLPNLKRAGQQSLGV--------IKGIFELDGTRIVNNAKYATENVAKTVGK---
-GYVNLANLAVSDEARAKVLALGAKIRSIGQVAQAKVNGLSDPNTLWTIGWLFSIMGMVAAKHRQKR---AQ
IKAKETTKANASHPSTATDKSNKQAHAENNANQC------------KNCMGGTGGSITFAMGTEFF---THV
DAQLGGIIQDSISRTYVSNLYQMDDAIFGARWVTPFTTKISRKFKYTSKKKDHKDY---LNGLEYICLDGRA
IDLPDLKKGQSIYDP-IEQYTYTVLSD---------QLHL-----IAYGEDEKR--------YYEKYGEDY-
RLSYIERKNGFKVA---LRYDHVSTDNKTILSDILFKQDDNLLAHLALQL--------TPQGLVSDIWTIKN
G---QLDRVL-------ASYDYDQQGDLVQATNEFA-ASYYYQY-THHLITRYTDLTHRGMNLKWDGI----
-----------------------LPTSKAIEEWADNASRASKLEWDKNIRKTTVLDVEGNSTEHYY---DID
GYTYRIVYPDNFEEWFFRDDAKNITLHIAKDGSKTSYTYDERGNVLTTTQDDGATSYFEY-DEKNQLTGMVD
AEQGRWFKQYDGSGNLIKEIDPLKHETAYVYNA---MGLVTSITDAKGGSKSLKYDDQGNLISYTDCSGK-E
TKWQYDERGRVISIENALNQKVEYFYTELTLENREPIIKGLPLNAFGQL-------------EKIKHADGTE
EHFIHDAEGRLLAHVDPKQNITRYEYDEAGLILSRTDALNHKLKYKWDRLG-RLTRLINENGASYQFFYDVA
SRLVKEIDFDGKETVYHYDEKSGQLATSIEVASAYGQDLKDRAAPKDR----IQQFIFDSMGRLEQRTAGYG
HYGLELEEKQTEEFAYDYMGRIIQAK------------NAQSNLQWFYDAAGNLVQEHQQ---DYKINKTAV
WKHQYDEINDRIKTTRP-DGQ---------VIDWLTY--GSGHVQSLIVNGQDFVSFERDDLHREIARHYAN
G------VSQEQQYDLAGRLKSQMMLSEHE-NGYQNQYK---RHNNALEQTSQLVQRLYQYDKTGELTAI--
--------------------------------RDTRRGNIAYKYDPVGRLL------EASSKLGKETFSFDP

ASNILDS------YHSQKV-----------QSHSQKLDETSYGYNRLVNNVVKEYLDQQYQYDAYGQLIRQK
TSQGDL-----NLEWDVYGRMVK-------SRNSQYTAEYRYDALGRRIQKWSKH-------HHTGQEQNII
YGWDGDTL--AYES----TEELTKHYIYEKDS-FVPMLQAVYLSPIELHQTPDWSD---RPYNIHRDPLWKT
EKEGK--EFDDVWFYHCDHLGTPQEMTDHTGAIIWKAEYKA-WGECKAEEAKSNFFENSEIISN-NIRFQGQ
YFDEETGLHYNRYRYYSPYVGRFVSKDPIGLLGGNNVYVYAKN-PITWIDSKGL
>15A34_6
---MSGKTEVVKPLTEVAIKDVSHSKQK-----------------IDSWLQSHTHQIVTLDLVASVMGTVPI
IGNVMAAIDVV-SDICYIYDKGFQNADIMDWTTMGIDLIGVAP-----IPGTTAIRTSARPALFLVRQEVKR
VGKAALG---------------------EAVIVVISNHLNDQVAGDIEKFCKTGLNKLNSILATCGATVQNA
TTQINGGIVRV---VTKGQIFTNAGSNASRAQ--QQAAGVIKGIKSGDLTRAGKNLVYMTENWGKALIKTGA
NSNAKVASSLVPAHLKQPILNVGATINKIGITANTKIKGLGNTAQPQTIGWLFDVLRMSASKFRKKK--SAQ
VKPTQTTQVKHDKKNNKLEKSNDEHAATHNANAC------------KNCKGGTTHSITFALGTEFL---NHI
DAQIHPLLIEQFSRTYVSNLYQYDQSIFGARWITPFTTKIVRCSSYNVHHPKEPIE---QDGWEYIGSDGRP
VRLPELKVGQSYYDE-VETFTYTVISP---------EIRA-----ISYGVSETR--------FYQKYKNEF-
RLASIERQNGFSIG---LRYDHELENSDSYLSDIIFKQQQQILAHVALQI--------NDAQKVVAAWLVEE
G---QLIRQL-------SAYQYNEQDDLIKATNEFA-ASYEYQY-QAHLLTRYTDLTHRGMNLKWDGV----
-----------------------LPSSKAIEEWADDYSSATKLDWDENVRVTSVMDIDGNVTEYYY---GIT
GYTYRIIYPDNLQEWFFRDQAKNITSHIATDGTETSYTYDERGNVLSMVQADGSTFYYEY-DEKDNLIGFVD
AEQGRWFKEYDASGNIIKEIDPLKRETVYAYNA---MGLVTSITDAKGGKKTLKYDDRGNLISYTDCSGK-E
TKWHYDDRGRVKFIENALKQKVEYFYTELTTELRQPIAKGLPLNAFGQL-------------EKIKHADGAE
EHFIHDAEGRLLVHIDPKGQQTRYEYDAAGLITKRTDPLNQTLKYQWDRLS-RLKRLINENGASYEFVYDAA
GRLVKEIDFDGKETVYAYDEYNGRLTTSIEVASTYGQDFKDRAAPKDR----IQQFIFDSMGRLEQRTAGYG
YQGQELEEKQTEEFSYDGLGNLVQAK------------NAETNLQWFYDAAGNLIKEHHQ---DVVTQKTAV
WKHVYDELNNRTTTIRP-DGQ---------KIDWLSY--GSGHVYGMALNGEDVVSFERDDLHRETLRHYAN
G------LSQQQSYDEVGRLTQQLMLSGHD-KGYQAQTQ-----NNAIQHTNQLIERLYHYDKTGELTLI--
--------------------------------KDTRRGAIHYKYDPVGRLL------EATSKLGKETFNFDP
ASNILER------YNSTKE-----------QSSQHISDEKGYGYNRLVNNIVQEYLDQQYQYDAFGQLIRQK
SSQGDL-----NLEWDVFGRLVR-------SRNNQYTAEYRYDALGRRIQKRSKH-------HHTGQEQNIS
YGWDGDTL--AYES----SADLTKHYFYEKDS-FVPLLQAAYHHPIELHQTQDWSD---KTYSIYKDPLWNT
VKQSQ--GFDDVWFYHCDHLGTPQEMSDQTGAIIWKAEYKA-WGECKLEQTNSDFFEKSEIISN-NIRFQGQ
YFDEETGLHYNRYRYYSPYVGRFISKDPIGLLGGFNVYAYTAN-PVQWVDPYGL
>15A5_6
---MSGKTEVVKPLTEVAIKDVSHSKQK-----------------IDSWLQSHTHQIVTLDLVASVMGTVPI

IGNVMAAIDVV-SDICYIYDKGFQNADIMDWTTMGIDLIGVAP-----IPGTTAIRTSARPALFLVRQEVKR
VGKAALG---------------------EAVIVVISNHLNDQVAGDIEKFCKTGLNKLNSILATCGATVQNA
TTQINGGIVRV---VTKGQIFTNAGSNASRAQ--QQAAGVIKGIKSGDLTRAGKNLVYMTENWGKALIKTGA
NSNAKVASSLVPAHLKQPILNVGATINKIGITANTKIKGLGNTAQPQTIGWLFDVLRMSASKFRKKK--SAQ
VKPTQTTQVKHDKKNNKLEKSNDEHAATHNANAC------------KNCKGGTTHSITFALGTEFL---NHI
DAQIHPLLIEQFSRTYVSNLYQYDQSIFGARWITPFTTKIVRCSSYNVHHPKEPIE---QDGWEYIGSDGRP
VRLPELKVGQSYYDE-VETFTYTVISP---------EIRA-----ISYGVSETR--------FYQKYKNEF-
RLASIERQNGFSIG---LRYDHELENSDSYLSDIIFKQQQQILAHVALQI--------NDAQKVVAAWLVEE
G---QLIRQL-------SAYQYNEQDDLIKATNEFA-ASYEYQY-QAHLLTRYTDLTHRGMNLKWDGV----
-----------------------LPSSKAIEEWADDYSSATKLDWDENVRVTSVMDIDGNVTEYYY---GIT
GYTYRIIYPDNLQEWFFRDQAKNITSHIATDGTETSYTYDERGNVLSMVQADGSTFYYEY-DEKDNLIGFVD
AEQGRWFKEYDASGNIIKEIDPLKRETVYAYNA---MGLVTSITDAKGGKKTLKYDDRGNLISYTDCSGK-E
TKWHYDDRGRVKFIENALKQKVEYFYTELTTELRQPIAKGLPLNAFGQL-------------EKIKHADGAE
EHFIHDAEGRLLVHIDPKGQQTRYEYDAAGLITKRTDPLNQTLKYQWDRLS-RLKRLINENGASYEFVYDAA
GRLVKEIDFDGKETVYAYDEYNGRLTTSIEVASTYGQDFKDRAAPKDR----IQQFIFDSMGRLEQRTAGYG
YQGQELEEKQTEEFSYDGLGNLVQAK------------NAETNLQWFYDAAGNLIKEHHQ---DVVTQKTAV
WKHVYDELNNRTTTIRP-DGQ---------KIDWLSY--GSGHVYGMALNGEDVVSFERDDLHRETLRHYAN
G------LSQQQSYDEVGRLTQQLMLSGHD-KGYQAQTQ-----NNAIQHTNQLIERLYHYDKTGELTLI--
--------------------------------KDTRRGAIHYKYDPVGRLL------EATSKLGKETFNFDP
ASNILER------YNSTKE-----------QSSQHISDEKGYGYNRLVNNIVQEYLDQQYQYDAFGQLIRQK
SSQGDL-----NLEWDVFGRLVR-------SRNNQYTAEYRYDALGRRIQKRSKH-------HHTGQEQNIS
YGWDGDTL--AYES----SADLTKHYFYEKDS-FVPLLQAAYHHPIELHQTQDWSD---KTYSIYKDPLWNT
VKQSQ--GFDDVWFYHCDHLGTPQEMSDQTGAIIWKAEYKA-WGECKLEQTNSDFFEKSEIISN-NIRFQGQ
YFDEETGLHYNRYRYYSPYVGRFISKDPIGLLGGFNVYAYTAN-PVQWVDPYGL
>1656-2_6
---MSGKTEVVKPLTEVAIKDVSHSKQK-----------------IDSWLQSHTHQIVTLDLVASVMGTVPI
IGNVMAAIDVV-SDICYIYDKGFQNADIMDWTTMGIDLIGVAP-----IPGTTAIRTSARPALFLVRQEVKR
VGKAALG---------------------EAVIVVISNHLNDQVAGDIEKFCKTGLNKLNSILATCGATVQNA
TTQINGGIVRV---VTKGQIFTNAGSNASRAQ--QQAAGVIKGIKSGDLTRAGKNLVYMTENWGKALIKTGA
NSNAKVASSLVPAHLKQPILNVGATINKIGITANTKIKGLGNTAQPQTIGWLFDVLRMSASKFRKKK--SAQ
VKPTQTTQVKHDKKNNKLEKSNDEHAATHNANAC------------KNCKGGTTHSITFALGTEFL---NHI
DAQIHPLLIEQFSRTYVSNLYQYDQSIFGARWITPFTTKIVRCSSYNVHHPKEPIE---QDGWEYIGSDGRP
VRLPELKVGQSYYDE-VETFTYTVISP---------EIRA-----ISYGVSETR--------FYQKYKNEF-

RLASIERQNGFSIG---LRYDHELENSDSYLSDIIFKQQQQILAHVALQI--------NDAQKVVAAWLVEE
G---QLIRQL-------SAYQYNEQDDLIKATNEFA-ASYEYQY-QAHLLTRYTDLTHRGMNLKWDGV----
-----------------------LPSSKAIEEWADDYSSATKLDWDENVRVTSVMDIDGNVTEYYY---GIT
GYTYRIIYPDNLQEWFFRDQAKNITSHIATDGTETSYTYDERGNVLSMVQADGSTFYYEY-DEKDNLIGFVD
AEQGRWFKEYDASGNIIKEIDPLKRETVYAYNA---MGLVTSITDAKGGKKTLKYDDRGNLISYTDCSGK-E
TKWHYDDRGRVKFIENALKQKVEYFYTELTTELRQPIAKGLPLNAFGQL-------------EKIKHADGAE
EHFIHDAEGRLLVHIDPKGQQTRYEYDAAGLITKRTDPLNQTLKYQWDRLS-RLKRLINENGASYEFVYDAA
GRLVKEIDFDGKETVYAYDEYNGRLTTSIEVASTYGQDFKDRAAPKDR----IQQFIFDSMGRLEQRTAGYG
YQGQELEEKQTEEFSYDGLGNLVQAK------------NAETNLQWFYDAAGNLIKEHHQ---DVVTQKTAV
WKHVYDELNNRTTTIRP-DGQ---------KIDWLSY--GSGHVYGMALNGEDVVSFERDDLHRETLRHYAN
G------LSQQQSYDEVGRLTQQLMLSGHD-KGYQAQTQ-----NNAIQHTNQLIERLYHYDKTGELTLI--
--------------------------------KDTRRGAIHYKYDPVGRLL------EATSKLGKETFNFDP
ASNILER------YNSTKE-----------QSSQHISDEKGYGYNRLVNNIVQEYLDQQYQYDAFGQLIRQK
SSQGDL-----NLEWDVFGRLVR-------SRNNQYTAEYRYDALGRRIQKRSKH-------HHTGQEQNIS
YGWDGDTL--AYES----SADLTKHYFYEKDS-FVPLLQAAYHHPIELHQTQDWSD---KTYSIYKDPLWNT
VKQSQ--GFDDVWFYHCDHLGTPQEMSDQTGAIIWKAEYKA-WGECKLEQTNSDFFEKSEIISN-NIRFQGQ
YFDEETGLHYNRYRYYSPYVGRFISKDPIGLLGGFNVYAYTAN-PVQWVDPYGL
>3027STDY5784958_6
---MSGKTEVVKPLTEVAIKDVSHSKQK-----------------IDSWLQSHTHQIVTLDLVASVMGTVPI
IGNVMAAIDVV-SDICYIYDKGFQNADIMDWTTMGIDLIGVAP-----IPGTTAIRTSARPALFLVRQEVKR
VGKAALG---------------------EAVIVVISNHLNDQVAGDIEKFCKTGLNKLNSILATCGATVQNA
TTQINGGIVRV---VTKGQIFTNAGSNASRAQ--QQAAGVIKGIKSGDLTRAGKNLVYMTENWGKALIKTGA
NSNAKVASSLVPAHLKQPILNVGATINKIGITANTKIKGLGNTAQPQTIGWLFDVLRMSASKFRKKK--SAQ
VKPTQTTQVKHDKKNNKLEKSNDEHAATHNANAC------------KNCKGGTTHSITFALGTEFL---NHI
DAQIHPLLIEQFSRTYVSNLYQYDQSIFGARWITPFTTKIVRCSSYNVHHPKEPIE---QDGWEYIGSDGRP
VRLPELKVGQSYYDE-VETFTYTVISP---------EIRA-----ISYGVSETR--------FYQKYKNEF-
RLASIERQNGFSIG---LRYDHELENSDSYLSDIIFKQQQQILAHVALQI--------NDAQKVVAAWLVEE
G---QLIRQL-------SAYQYNEQDDLIKATNEFA-ASYEYQY-QAHLLTRYTDLTHRGMNLKWDGV----
-----------------------LPSSKAIEEWADDYSSATKLDWDENVRVTSVMDIDGNVTEYYY---GIT
GYTYRIIYPDNLQEWFFRDQAKNITSHIATDGTETSYTYDERGNVLSMVQADGSTFYYEY-DEKDNLIGFVD
AEQGRWFKEYDASGNIIKEIDPLKRETVYAYNA---MGLVTSITDAKGGKKTLKYDDRGNLISYTDCSGK-E
TKWHYDDRGRVKFIENALKQKVEYFYTELTTELRQPIAKGLPLNAFGQL-------------EKIKHADGAE
EHFIHDAEGRLLVHIDPKGQQTRYEYDAAGLITKRTDPLNQTLKYQWDRLS-RLKRLINENGASYEFVYDAA

GRLVKEIDFDGKETVYAYDEYNGRLTTSIEVASTYGQDFKDRAAPKDR----IQQFIFDSMGRLEQRTAGYG
YQGQELEEKQTEEFSYDGLGNLVQAK------------NAETNLQWFYDAAGNLIKEHHQ---DVVTQKTAV
WKHVYDELNNRTTTIRP-DGQ---------KIDWLSY--GSGHVYGMALNGEDVVSFERDDLHRETLRHYAN
G------LSQQQSYDEVGRLTQQLMLSGHD-KGYQAQTQ-----NNAIQHTNQLIERLYHYDKTGELTLI--
--------------------------------KDTRRGAIHYKYDPVGRLL------EATSKLGKETFNFDP
ASNILER------YNSTKE-----------QSSQHISDEKGYGYNRLVNNIVQEYLDQQYQYDAFGQLIRQK
SSQGDL-----NLEWDVFGRLVR-------SRNNQYTAEYRYDALGRRIQKRSKH-------HHTGQEQNIS
YGWDGDTL--AYES----SADLTKHYFYEKDS-FVPLLQAAYHHPIELHQTQDWSD---KTYSIYKDPLWNT
VKQSQ--GFDDVWFYHCDHLGTPQEMSDQTGAIIWKAEYKA-WGECKLEQTNSDFFEKSEIISN-NIRFQGQ
YFDEETGLHYNRYRYYSPYVGRFISKDPIGLLGGFNVYAYTAN-PVQWVDPYGL
>AB07_6
---MSGKTEVVKPLTEVAIKDVSHSKQK-----------------IDSWLQSHTHQIVTLDLVASVMGTVPI
IGNVMAAIDVV-SDICYIYDKGFQNADIMDWTTMGIDLIGVAP-----IPGTTAIRTSARPALFLVRQEVKR
VGKAALG---------------------EAVIVVISNHLNDQVAGDIEKFCKTGLNKLNSILATCGATVQNA
TTQINGGIVRV---VTKGQIFTNAGSNASRAQ--QQAAGVIKGIKSGDLTRAGKNLVYMTENWGKALIKTGA
NSNAKVASSLVPAHLKQPILNVGATINKIGITANTKIKGLGNTAQPQTIGWLFDVLRMSASKFRKKK--SAQ
VKPTQTTQVKHDKKNNKLEKSNDEHAATHNANAC------------KNCKGGTTHSITFALGTEFL---NHI
DAQIHPLLIEQFSRTYVSNLYQYDQSIFGARWITPFTTKIVRCSSYNVHHPKEPIE---QDGWEYIGSDGRP
VRLPELKVGQSYYDE-VETFTYTVISP---------EIRA-----ISYGVSETR--------FYQKYKNEF-
RLASIERQNGFSIG---LRYDHELENSDSYLSDIIFKQQQQILAHVALQI--------NDAQKVVAAWLVEE
G---QLIRQL-------SAYQYNEQDDLIKATNEFA-ASYEYQY-QAHLLTRYTDLTHRGMNLKWDGV----
-----------------------LPSSKAIEEWADDYSSATKLDWDENVRVTSVMDIDGNVTEYYY---GIT
GYTYRIIYPDNLQEWFFRDQAKNITSHIATDGTETSYTYDERGNVLSMVQADGSTFYYEY-DEKDNLIGFVD
AEQGRWFKEYDASGNIIKEIDPLKRETVYAYNA---MGLVTSITDAKGGKKTLKYDDRGNLISYTDCSGK-E
TKWHYDDRGRVKFIENALKQKVEYFYTELTTELRQPIAKGLPLNAFGQL-------------EKIKHADGAE
EHFIHDAEGRLLVHIDPKGQQTRYEYDAAGLITKRTDPLNQTLKYQWDRLS-RLKRLINENGASYEFVYDAA
GRLVKEIDFDGKETVYAYDEYNGRLTTSIEVASTYGQDFKDRAAPKDR----IQQFIFDSMGRLEQRTAGYG
YQGQELEEKQTEEFSYDGLGNLVQAK------------NAETNLQWFYDAAGNLIKEHHQ---DVVTQKTAV
WKHVYDELNNRTTTIRP-DGQ---------KIDWLSY--GSGHVYGMALNGEDVVSFERDDLHRETLRHYAN
G------LSQQQSYDEVGRLTQQLMLSGHD-KGYQAQTQ-----NNAIQHTNQLIERLYHYDKTGELTLI--
--------------------------------KDTRRGAIHYKYDPVGRLL------EATSKLGKETFNFDP
ASNILER------YNSTKE-----------QSSQHISDEKGYGYNRLVNNIVQEYLDQQYQYDAFGQLIRQK
SSQGDL-----NLEWDVFGRLVR-------SRNNQYTAEYRYDALGRRIQKRSKH-------HHTGQEQNIS

YGWDGDTL--AYES----SADLTKHYFYEKDS-FVPLLQAAYHHPIELHQTQDWSD---KTYSIYKDPLWNT
VKQSQ--GFDDVWFYHCDHLGTPQEMSDQTGAIIWKAEYKA-WGECKLEQTNSDFFEKSEIISN-NIRFQGQ
YFDEETGLHYNRYRYYSPYVGRFISKDPIGLLGGFNVYAYTAN-PVQWVDPYGL
>AB34299_6
---MSGKTEVVKPLTEVAIKDVSHSKQK-----------------IDSWLQSHTHQIVTLDLVASVMGTVPI
IGNVMAAIDVV-SDICYIYDKGFQNADIMDWTTMGIDLIGVAP-----IPGTTAIRTSARPALFLVRQEVKR
VGKAALG---------------------EAVIVVISNHLNDQVAGDIEKFCKTGLNKLNSILATCGATVQNA
TTQINGGIVRV---VTKGQIFTNAGSNASRAQ--QQAAGVIKGIKSGDLTRAGKNLVYMTENWGKALIKTGA
NSNAKVASSLVPAHLKQPILNVGATINKIGITANTKIKGLGNTAQPQTIGWLFDVLRMSASKFRKKK--SAQ
VKPTQTTQVKHDKKNNKLEKSNDEHAATHNANAC------------KNCKGGTTHSITFALGTEFL---NHI
DAQIHPLLIEQFSRTYVSNLYQYDQSIFGARWITPFTTKIVRCSSYNVHHPKEPIE---QDGWEYIGSDGRP
VRLPELKVGQSYYDE-VETFTYTVISP---------EIRA-----ISYGVSETR--------FYQKYKNEF-
RLASIERQNGFSIG---LRYDHELENSDSYLSDIIFKQQQQILAHVALQI--------NDAQKVVAAWLVEE
G---QLIRQL-------SAYQYNEQDDLIKATNEFA-ASYEYQY-QAHLLTRYTDLTHRGMNLKWDGV----
-----------------------LPSSKAIEEWADDYSSATKLDWDENVRVTSVMDIDGNVTEYYY---GIT
GYTYRIIYPDNLQEWFFRDQAKNITSHIATDGTETSYTYDERGNVLSMVQADGSTFYYEY-DEKDNLIGFVD
AEQGRWFKEYDASGNIIKEIDPLKRETVYAYNA---MGLVTSITDAKGGKKTLKYDDRGNLISYTDCSGK-E
TKWHYDDRGRVKFIENALKQKVEYFYTELTTELRQPIAKGLPLNAFGQL-------------EKIKHADGAE
EHFIHDAEGRLLVHIDPKGQQTRYEYDAAGLITKRTDPLNQTLKYQWDRLS-RLKRLINENGASYEFVYDAA
GRLVKEIDFDGKETVYAYDEYNGRLTTSIEVASTYGQDFKDRAAPKDR----IQQFIFDSMGRLEQRTAGYG
YQGQELEEKQTEEFSYDGLGNLVQAK------------NAETNLQWFYDAAGNLIKEHHQ---DVVTQKTAV
WKHVYDELNNRTTTIRP-DGQ---------KIDWLSY--GSGHVYGMALNGEDVVSFERDDLHRETLRHYAN
G------LSQQQSYDEVGRLTQQLMLSGHD-KGYQAQTQ-----NNAIQHTNQLIERLYHYDKTGELTLI--
--------------------------------KDTRRGAIHYKYDPVGRLL------EATSKLGKETFNFDP
ASNILER------YNSTKE-----------QSSQHISDEKGYGYNRLVNNIVQEYLDQQYQYDAFGQLIRQK
SSQGDL-----NLEWDVFGRLVR-------SRNNQYTAEYRYDALGRRIQKRSKH-------HHTGQEQNIS
YGWDGDTL--AYES----SADLTKHYFYEKDS-FVPLLQAAYHHPIELHQTQDWSD---KTYSIYKDPLWNT
VKQSQ--GFDDVWFYHCDHLGTPQEMSDQTGAIIWKAEYKA-WGECKLEQTNSDFFEKSEIISN-NIRFQGQ
YFDEETGLHYNRYRYYSPYVGRFISKDPIGLLGGFNVYAYTAN-PVQWVDPYGL
>Ab4568_6
---MSGKTEVVKPLTEVAIKDVSHSKQK-----------------IDSWLQSHTHQIVTLDLVASVMGTVPI
IGNVMAAIDVV-SDICYIYDKGFQNADIMDWTTMGIDLIGVAP-----IPGTTAIRTSARPALFLVRQEVKR
VGKAALG---------------------EAVIVVISNHLNDQVAGDIEKFCKTGLNKLNSILATCGATVQNA

TTQINGGIVRV---VTKGQIFTNAGSNASRAQ--QQAAGVIKGIKSGDLTRAGKNLVYMTENWGKALIKTGA
NSNAKVASSLVPAHLKQPILNVGATINKIGITANTKIKGLGNTAQPQTIGWLFDVLRMSASKFRKKK--SAQ
VKPTQTTQVKHDKKNNKLEKSNDEHAATHNANAC------------KNCKGGTTHSITFALGTEFL---NHI
DAQIHPLLIEQFSRTYVSNLYQYDQSIFGARWITPFTTKIVRCSSYNVHHPKEPIE---QDGWEYIGSDGRP
VRLPELKVGQSYYDE-VETFTYTVISP---------EIRA-----ISYGVSETR--------FYQKYKNEF-
RLASIERQNGFSIG---LRYDHELENSDSYLSDIIFKQQQQILAHVALQI--------NDAQKVVAAWLVEE
G---QLIRQL-------SAYQYNEQDDLIKATNEFA-ASYEYQY-QAHLLTRYTDLTHRGMNLKWDGV----
-----------------------LPSSKAIEEWADDYSSATKLDWDENVRVTSVMDIDGNVTEYYY---GIT
GYTYRIIYPDNLQEWFFRDQAKNITSHIATDGTETSYTYDERGNVLSMVQADGSTFYYEY-DEKDNLIGFVD
AEQGRWFKEYDASGNIIKEIDPLKRETVYAYNA---MGLVTSITDAKGGKKTLKYDDRGNLISYTDCSGK-E
TKWHYDDRGRVKFIENALKQKVEYFYTELTTELRQPIAKGLPLNAFGQL-------------EKIKHADGAE
EHFIHDAEGRLLVHIDPKGQQTRYEYDAAGLITKRTDPLNQTLKYQWDRLS-RLKRLINENGASYEFVYDAA
GRLVKEIDFDGKETVYAYDEYNGRLTTSIEVASTYGQDFKDRAAPKDR----IQQFIFDSMGRLEQRTAGYG
YQGQELEEKQTEEFSYDGLGNLVQAK------------NAETNLQWFYDAAGNLIKEHHQ---DVVTQKTAV
WKHVYDELNNRTTTIRP-DGQ---------KIDWLSY--GSGHVYGMALNGEDVVSFERDDLHRETLRHYAN
G------LSQQQSYDEVGRLTQQLMLSGHD-KGYQAQTQ-----NNAIQHTNQLIERLYHYDKTGELTLI--
---------------------------------KDTRRGAIHYKYDPVGRLL------EATSKLGKETFNFDP
ASNILER------YNSTKE-----------QSSQHISDEKGYGYNRLVNNIVQEYLDQQYQYDAFGQLIRQK
SSQGDL-----NLEWDVFGRLVR-------SRNNQYTAEYRYDALGRRIQKRSKH-------HHTGQEQNIS
YGWDGDTL--AYES----SADLTKHYFYEKDS-FVPLLQAAYHHPIELHQTQDWSD---KTYSIYKDPLWNT
VKQSQ--GFDDVWFYHCDHLGTPQEMSDQTGAIIWKAEYKA-WGECKLEQTNSDFFEKSEIISN-NIRFQGQ
YFDEETGLHYNRYRYYSPYVGRFISKDPIGLLGGFNVYAYTAN-PVQWVDPYGL
>Ab4653_6
---MSGKTEVVKPLTEVAIKDVSHSKQK-----------------IDSWLQSHTHQIVTLDLVASVMGTVPI
IGNVMAAIDVV-SDICYIYDKGFQNADIMDWTTMGIDLIGVAP-----IPGTTAIRTSARPALFLVRQEVKR
VGKAALG---------------------EAVIVVISNHLNDQVAGDIEKFCKTGLNKLNSILATCGATVQNA
TTQINGGIVRV---VTKGQIFTNAGSNASRAQ--QQAAGVIKGIKSGDLTRAGKNLVYMTENWGKALIKTGA
NSNAKVASSLVPAHLKQPILNVGATINKIGITANTKIKGLGNTAQPQTIGWLFDVLRMSASKFRKKK--SAQ
VKPTQTTQVKHDKKNNKLEKSNDEHAATHNANAC------------KNCKGGTTHSITFALGTEFL---NHI
DAQIHPLLIEQFSRTYVSNLYQYDQSIFGARWITPFTTKIVRCSSYNVHHPKEPIE---QDGWEYIGSDGRP
VRLPELKVGQSYYDE-VETFTYTVISP---------EIRA-----ISYGVSETR--------FYQKYKNEF-
RLASIERQNGFSIG---LRYDHELENSDSYLSDIIFKQQQQILAHVALQI--------NDAQKVVAAWLVEE
G---QLIRQL-------SAYQYNEQDDLIKATNEFA-ASYEYQY-QAHLLTRYTDLTHRGMNLKWDGV----

-----------------------LPSSKAIEEWADDYSSATKLDWDENVRVTSVMDIDGNVTEYYY---GIT
GYTYRIIYPDNLQEWFFRDQAKNITSHIATDGTETSYTYDERGNVLSMVQADGSTFYYEY-DEKDNLIGFVD
AEQGRWFKEYDASGNIIKEIDPLKRETVYAYNA---MGLVTSITDAKGGKKTLKYDDRGNLISYTDCSGK-E
TKWHYDDRGRVKFIENALKQKVEYFYTELTTELRQPIAKGLPLNAFGQL-------------EKIKHADGAE
EHFIHDAEGRLLVHIDPKGQQTRYEYDAAGLITKRTDPLNQTLKYQWDRLS-RLKRLINENGASYEFVYDAA
GRLVKEIDFDGKETVYAYDEYNGRLTTSIEVASTYGQDFKDRAAPKDR----IQQFIFDSMGRLEQRTAGYG
YQGQELEEKQTEEFSYDGLGNLVQAK------------NAETNLQWFYDAAGNLIKEHHQ---DVVTQKTAV
WKHVYDELNNRTTTIRP-DGQ---------KIDWLSY--GSGHVYGMALNGEDVVSFERDDLHRETLRHYAN
G------LSQQQSYDEVGRLTQQLMLSGHD-KGYQAQTQ-----NNAIQHTNQLIERLYHYDKTGELTLI--
--------------------------------KDTRRGAIHYKYDPVGRLL------EATSKLGKETFNFDP
ASNILER------YNSTKE-----------QSSQHISDEKGYGYNRLVNNIVQEYLDQQYQYDAFGQLIRQK
SSQGDL-----NLEWDVFGRLVR-------SRNNQYTAEYRYDALGRRIQKRSKH-------HHTGQEQNIS
YGWDGDTL--AYES----SADLTKHYFYEKDS-FVPLLQAAYHHPIELHQTQDWSD---KTYSIYKDPLWNT
VKQSQ--GFDDVWFYHCDHLGTPQEMSDQTGAIIWKAEYKA-WGECKLEQTNSDFFEKSEIISN-NIRFQGQ
YFDEETGLHYNRYRYYSPYVGRFISKDPIGLLGGFNVYAYTAN-PVQWVDPYGL
>Ab4977_6
---MSGKTEVVKPLTEVAIKDVSHSKQK-----------------IDSWLQSHTHQIVTLDLVASVMGTVPI
IGNVMAAIDVV-SDICYIYDKGFQNADIMDWTTMGIDLIGVAP-----IPGTTAIRTSARPALFLVRQEVKR
VGKAALG---------------------EAVIVVISNHLNDQVAGDIEKFCKTGLNKLNSILATCGATVQNA
TTQINGGIVRV---VTKGQIFTNAGSNASRAQ--QQAAGVIKGIKSGDLTRAGKNLVYMTENWGKALIKTGA
NSNAKVASSLVPAHLKQPILNVGATINKIGITANTKIKGLGNTAQPQTIGWLFDVLRMSASKFRKKK--SAQ
VKPTQTTQVKHDKKNNKLEKSNDEHAATHNANAC------------KNCKGGTTHSITFALGTEFL---NHI
DAQIHPLLIEQFSRTYVSNLYQYDQSIFGARWITPFTTKIVRCSSYNVHHPKEPIE---QDGWEYIGSDGRP
VRLPELKVGQSYYDE-VETFTYTVISP---------EIRA-----ISYGVSETR--------FYQKYKNEF-
RLASIERQNGFSIG---LRYDHELENSDSYLSDIIFKQQQQILAHVALQI--------NDAQKVVAAWLVEE
G---QLIRQL-------SAYQYNEQDDLIKATNEFA-ASYEYQY-QAHLLTRYTDLTHRGMNLKWDGV----
-----------------------LPSSKAIEEWADDYSSATKLDWDENVRVTSVMDIDGNVTEYYY---GIT
GYTYRIIYPDNLQEWFFRDQAKNITSHIATDGTETSYTYDERGNVLSMVQADGSTFYYEY-DEKDNLIGFVD
AEQGRWFKEYDASGNIIKEIDPLKRETVYAYNA---MGLVTSITDAKGGKKTLKYDDRGNLISYTDCSGK-E
TKWHYDDRGRVKFIENALKQKVEYFYTELTTELRQPIAKGLPLNAFGQL-------------EKIKHADGAE
EHFIHDAEGRLLVHIDPKGQQTRYEYDAAGLITKRTDPLNQTLKYQWDRLS-RLKRLINENGASYEFVYDAA
GRLVKEIDFDGKETVYAYDEYNGRLTTSIEVASTYGQDFKDRAAPKDR----IQQFIFDSMGRLEQRTAGYG
YQGQELEEKQTEEFSYDGLGNLVQAK------------NAETNLQWFYDAAGNLIKEHHQ---DVVTQKTAV

WKHVYDELNNRTTTIRP-DGQ---------KIDWLSY--GSGHVYGMALNGEDVVSFERDDLHRETLRHYAN
G------LSQQQSYDEVGRLTQQLMLSGHD-KGYQAQTQ-----NNAIQHTNQLIERLYHYDKTGELTLI--
--------------------------------KDTRRGAIHYKYDPVGRLL------EATSKLGKETFNFDP
ASNILER------YNSTKE-----------QSSQHISDEKGYGYNRLVNNIVQEYLDQQYQYDAFGQLIRQK
SSQGDL-----NLEWDVFGRLVR-------SRNNQYTAEYRYDALGRRIQKRSKH-------HHTGQEQNIS
YGWDGDTL--AYES----SADLTKHYFYEKDS-FVPLLQAAYHHPIELHQTQDWSD---KTYSIYKDPLWNT
VKQSQ--GFDDVWFYHCDHLGTPQEMSDQTGAIIWKAEYKA-WGECKLEQTNSDFFEKSEIISN-NIRFQGQ
YFDEETGLHYNRYRYYSPYVGRFISKDPIGLLGGFNVYAYTAN-PVQWVDPYGL
>AbPK1_6
---MSGKTEVVKPLTEVAIKDVSHSKQK-----------------IDSWLQSHTHQIVTLDLVASVMGTVPI
IGNVMAAIDVV-SDICYIYDKGFQNADIMDWTTMGIDLIGVAP-----IPGTTAIRTSARPALFLVRQEVKR
VGKAALG---------------------EAVIVVISNHLNDQVAGDIEKFCKTGLNKLNSILATCGATVQNA
TTQINGGIVRV---VTKGQIFTNAGSNASRAQ--QQAAGVIKGIKSGDLTRAGKNLVYMTENWGKALIKTGA
NSNAKVASSLVPAHLKQPILNVGATINKIGITANTKIKGLGNTAQPQTIGWLFDVLRMSASKFRKKK--SAQ
VKPTQTTQVKHDKKNNKLEKSNDEHAATHNANAC------------KNCKGGTTHSITFALGTEFL---NHI
DAQIHPLLIEQFSRTYVSNLYQYDQSIFGARWITPFTTKIVRCSSYNVHHPKEPIE---QDGWEYIGSDGRP
VRLPELKVGQSYYDE-VETFTYTVISP---------EIRA-----ISYGVSETR--------FYQKYKNEF-
RLASIERQNGFSIG---LRYDHELENSDSYLSDIIFKQQQQILAHVALQI--------NDAQKVVAAWLVEE
G---QLIRQL-------SAYQYNEQDDLIKATNEFA-ASYEYQY-QAHLLTRYTDLTHRGMNLKWDGV----
-----------------------LPSSKAIEEWADDYSSATKLDWDENVRVTSVMDIDGNVTEYYY---GIT
GYTYRIIYPDNLQEWFFRDQAKNITSHIATDGTETSYTYDERGNVLSMVQADGSTFYYEY-DEKDNLIGFVD
AEQGRWFKEYDASGNIIKEIDPLKRETVYAYNA---MGLVTSITDAKGGKKTLKYDDRGNLISYTDCSGK-E
TKWHYDDRGRVKFIENALKQKVEYFYTELTTELRQPIAKGLPLNAFGQL-------------EKIKHADGAE
EHFIHDAEGRLLVHIDPKGQQTRYEYDAAGLITKRTDPLNQTLKYQWDRLS-RLKRLINENGASYEFVYDAA
GRLVKEIDFDGKETVYAYDEYNGRLTTSIEVASTYGQDFKDRAAPKDR----IQQFIFDSMGRLEQRTAGYG
YQGQELEEKQTEEFSYDGLGNLVQAK------------NAETNLQWFYDAAGNLIKEHHQ---DVVTQKTAV
WKHVYDELNNRTTTIRP-DGQ---------KIDWLSY--GSGHVYGMALNGEDVVSFERDDLHRETLRHYAN
G------LSQQQSYDEVGRLTQQLMLSGHD-KGYQAQTQ-----NNAIQHTNQLIERLYHYDKTGELTLI--
--------------------------------KDTRRGAIHYKYDPVGRLL------EATSKLGKETFNFDP
ASNILER------YNSTKE-----------QSSQHISDEKGYGYNRLVNNIVQEYLDQQYQYDAFGQLIRQK
SSQGDL-----NLEWDVFGRLVR-------SRNNQYTAEYRYDALGRRIQKRSKH-------HHTGQEQNIS
YGWDGDTL--AYES----SADLTKHYFYEKDS-FVPLLQAAYHHPIELHQTQDWSD---KTYSIYKDPLWNT
VKQSQ--GFDDVWFYHCDHLGTPQEMSDQTGAIIWKAEYKA-WGECKLEQTNSDFFEKSEIISN-NIRFQGQ

YFDEETGLHYNRYRYYSPYVGRFISKDPIGLLGGFNVYAYTAN-PVQWVDPYGL
>AC29_6
---MSGKTEVVKPLTEVAIKDVSHSKQK-----------------IDSWLQSHTHQIVTLDLVASVMGTVPI
IGNVMAAIDVV-SDICYIYDKGFQNADIMDWTTMGIDLIGVAP-----IPGTTAIRTSARPALFLVRQEVKR
VGKAALG---------------------EAVIVVISNHLNDQVAGDIEKFCKTGLNKLNSILATCGATVQNA
TTQINGGIVRV---VTKGQIFTNAGSNASRAQ--QQAAGVIKGIKSGDLTRAGKNLVYMTENWGKALIKTGA
NSNAKVASSLVPAHLKQPILNVGATINKIGITANTKIKGLGNTAQPQTIGWLFDVLRMSASKFRKKK--SAQ
VKPTQTTQVKHDKKNNKLEKSNDEHAATHNANAC------------KNCKGGTTHSITFALGTEFL---NHI
DAQIHPLLIEQFSRTYVSNLYQYDQSIFGARWITPFTTKIVRCSSYNVHHPKEPIE---QDGWEYIGSDGRP
VRLPELKVGQSYYDE-VETFTYTVISP---------EIRA-----ISYGVSETR--------FYQKYKNEF-
RLASIERQNGFSIG---LRYDHELENSDSYLSDIIFKQQQQILAHVALQI--------NDAQKVVAAWLVEE
G---QLIRQL-------SAYQYNEQDDLIKATNEFA-ASYEYQY-QAHLLTRYTDLTHRGMNLKWDGV----
-----------------------LPSSKAIEEWADDYSSATKLDWDENVRVTSVMDIDGNVTEYYY---GIT
GYTYRIIYPDNLQEWFFRDQAKNITSHIATDGTETSYTYDERGNVLSMVQADGSTFYYEY-DEKDNLIGFVD
AEQGRWFKEYDASGNIIKEIDPLKRETVYAYNA---MGLVTSITDAKGGKKTLKYDDRGNLISYTDCSGK-E
TKWHYDDRGRVKFIENALKQKVEYFYTELTTELRQPIAKGLPLNAFGQL-------------EKIKHADGAE
EHFIHDAEGRLLVHIDPKGQQTRYEYDAAGLITKRTDPLNQTLKYQWDRLS-RLKRLINENGASYEFVYDAA
GRLVKEIDFDGKETVYAYDEYNGRLTTSIEVASTYGQDFKDRAAPKDR----IQQFIFDSMGRLEQRTAGYG
YQGQELEEKQTEEFSYDGLGNLVQAK------------NAETNLQWFYDAAGNLIKEHHQ---DVVTQKTAV
WKHVYDELNNRTTTIRP-DGQ---------KIDWLSY--GSGHVYGMALNGEDVVSFERDDLHRETLRHYAN
G------LSQQQSYDEVGRLTQQLMLSGHD-KGYQAQTQ-----NNAIQHTNQLIERLYHYDKTGELTLI--
--------------------------------KDTRRGAIHYKYDPVGRLL------EATSKLGKETFNFDP
ASNILER------YNSTKE-----------QSSQHISDEKGYGYNRLVNNIVQEYLDQQYQYDAFGQLIRQK
SSQGDL-----NLEWDVFGRLVR-------SRNNQYTAEYRYDALGRRIQKRSKH-------HHTGQEQNIS
YGWDGDTL--AYES----SADLTKHYFYEKDS-FVPLLQAAYHHPIELHQTQDWSD---KTYSIYKDPLWNT
VKQSQ--GFDDVWFYHCDHLGTPQEMSDQTGAIIWKAEYKA-WGECKLEQTNSDFFEKSEIISN-NIRFQGQ
YFDEETGLHYNRYRYYSPYVGRFISKDPIGLLGGFNVYAYTAN-PVQWVDPYGL
>AC30_6
---MSGKTEVVKPLTEVAIKDVSHSKQK-----------------IDSWLQSHTHQIVTLDLVASVMGTVPI
IGNVMAAIDVV-SDICYIYDKGFQNADIMDWTTMGIDLIGVAP-----IPGTTAIRTSARPALFLVRQEVKR
VGKAALG---------------------EAVIVVISNHLNDQVAGDIEKFCKTGLNKLNSILATCGATVQNA
TTQINGGIVRV---VTKGQIFTNAGSNASRAQ--QQAAGVIKGIKSGDLTRAGKNLVYMTENWGKALIKTGA
NSNAKVASSLVPAHLKQPILNVGATINKIGITANTKIKGLGNTAQPQTIGWLFDVLRMSASKFRKKK--SAQ

VKPTQTTQVKHDKKNNKLEKSNDEHAATHNANAC------------KNCKGGTTHSITFALGTEFL---NHI
DAQIHPLLIEQFSRTYVSNLYQYDQSIFGARWITPFTTKIVRCSSYNVHHPKEPIE---QDGWEYIGSDGRP
VRLPELKVGQSYYDE-VETFTYTVISP---------EIRA-----ISYGVSETR--------FYQKYKNEF-
RLASIERQNGFSIG---LRYDHELENSDSYLSDIIFKQQQQILAHVALQI--------NDAQKVVAAWLVEE
G---QLIRQL-------SAYQYNEQDDLIKATNEFA-ASYEYQY-QAHLLTRYTDLTHRGMNLKWDGV----
-----------------------LPSSKAIEEWADDYSSATKLDWDENVRVTSVMDIDGNVTEYYY---GIT
GYTYRIIYPDNLQEWFFRDQAKNITSHIATDGTETSYTYDERGNVLSMVQADGSTFYYEY-DEKDNLIGFVD
AEQGRWFKEYDASGNIIKEIDPLKRETVYAYNA---MGLVTSITDAKGGKKTLKYDDRGNLISYTDCSGK-E
TKWHYDDRGRVKFIENALKQKVEYFYTELTTELRQPIAKGLPLNAFGQL-------------EKIKHADGAE
EHFIHDAEGRLLVHIDPKGQQTRYEYDAAGLITKRTDPLNQTLKYQWDRLS-RLKRLINENGASYEFVYDAA
GRLVKEIDFDGKETVYAYDEYNGRLTTSIEVASTYGQDFKDRAAPKDR----IQQFIFDSMGRLEQRTAGYG
YQGQELEEKQTEEFSYDGLGNLVQAK------------NAETNLQWFYDAAGNLIKEHHQ---DVVTQKTAV
WKHVYDELNNRTTTIRP-DGQ---------KIDWLSY--GSGHVYGMALNGEDVVSFERDDLHRETLRHYAN
G------LSQQQSYDEVGRLTQQLMLSGHD-KGYQAQTQ-----NNAIQHTNQLIERLYHYDKTGELTLI--
--------------------------------KDTRRGAIHYKYDPVGRLL------EATSKLGKETFNFDP
ASNILER------YNSTKE-----------QSSQHISDEKGYGYNRLVNNIVQEYLDQQYQYDAFGQLIRQK
SSQGDL-----NLEWDVFGRLVR-------SRNNQYTAEYRYDALGRRIQKRSKH-------HHTGQEQNIS
YGWDGDTL--AYES----SADLTKHYFYEKDS-FVPLLQAAYHHPIELHQTQDWSD---KTYSIYKDPLWNT
VKQSQ--GFDDVWFYHCDHLGTPQEMSDQTGAIIWKAEYKA-WGECKLEQTNSDFFEKSEIISN-NIRFQGQ
YFDEETGLHYNRYRYYSPYVGRFISKDPIGLLGGFNVYAYTAN-PVQWVDPYGL
>ACICU_6
---MSGKTEVVKPLTEVAIKDVSHSKQK-----------------IDSWLQSHTHQIVTLDLVASVMGTVPI
IGNVMAAIDVV-SDICYIYDKGFQNADIMDWTTMGIDLIGVAP-----IPGTTAIRTSARPALFLVRQEVKR
VGKAALG---------------------EAVIVVISNHLNDQVAGDIEKFCKTGLNKLNSILATCGATVQNA
TTQINGGIVRV---VTKGQIFTNAGSNASRAQ--QQAAGVIKGIKSGDLTRAGKNLVYMTENWGKALIKTGA
NSNAKVASSLVPAHLKQPILNVGATINKIGITANTKIKGLGNTAQPQTIGWLFDVLRMSASKFRKKK--SAQ
VKPTQTTQVKHDKKNNKLEKSNDEHAATHNANAC------------KNCKGGTTHSITFALGTEFL---NHI
DAQIHPLLIEQFSRTYVSNLYQYDQSIFGARWITPFTTKIVRCSSYNVHHPKEPIE---QDGWEYIGSDGRP
VRLPELKVGQSYYDE-VETFTYTVISP---------EIRA-----ISYGVSETR--------FYQKYKNEF-
RLASIERQNGFSIG---LRYDHELENSDSYLSDIIFKQQQQILAHVALQI--------NDAQKVVAAWLVEE
G---QLIRQL-------SAYQYNEQDDLIKATNEFA-ASYEYQY-QAHLLTRYTDLTHRGMNLKWDGV----
-----------------------LPSSKAIEEWADDYSSATKLDWDENVRVTSVMDIDGNVTEYYY---GIT
GYTYRIIYPDNLQEWFFRDQAKNITSHIATDGTETSYTYDERGNVLSMVQADGSTFYYEY-DEKDNLIGFVD

```
AEQGRWFKEYDASGNIIKEIDPLKRETVYAYNA---MGLVTSITDAKGGKKTLKYDDRGNLISYTDCSGK-E
TKWHYDDRGRVKFIENALKQKVEYFYTELTTELRQPIAKGLPLNAFGQL-------------EKIKHADGAE
EHFIHDAEGRLLVHIDPKGQQTRYEYDAAGLITKRTDPLNQTLKYQWDRLS-RLKRLINENGASYEFVYDAA
GRLVKEIDFDGKETVYAYDEYNGRLTTSIEVASTYGQDFKDRAAPKDR----IQQFIFDSMGRLEQRTAGYG
YQGQELEEKQTEEFSYDGLGNLVQAK------------NAETNLQWFYDAAGNLIKEHHQ---DVVTQKTAV
WKHVYDELNNRTTTIRP-DGQ---------KIDWLSY--GSGHVYGMALNGEDVVSFERDDLHRETLRHYAN
G------LSQQQSYDEVGRLTQQLMLSGHD-KGYQAQTQ-----NNAIQHTNQLIERLYHYDKTGELTLI--
--------------------------------KDTRRGAIHYKYDPVGRLL------EATSKLGKETFNFDP
ASNILER------YNSTKE-----------QSSQHISDEKGYGYNRLVNNIVQEYLDQQYQYDAFGQLIRQK
SSQGDL-----NLEWDVFGRLVR-------SRNNQYTAEYRYDALGRRIQKRSKH-------HHTGQEQNIS
YGWDGDTL--AYES----SADLTKHYFYEKDS-FVPLLQAAYHHPIELHQTQDWSD---KTYSIYKDPLWNT
VKQSQ--GFDDVWFYHCDHLGTPQEMSDQTGAIIWKAEYKA-WGECKLEQTNSDFFEKSEIISN-NIRFQGQ
YFDEETGLHYNRYRYYSPYVGRFISKDPIGLLGGFNVYAYTAN-PVQWVDPYGL
>AF-673_6
---MSGKTEVVKPLTEVAIKDVSHSKQK-----------------IDSWLQSHTHQIVTLDLVASVMGTVPI
IGNVMAAIDVV-SDICYIYDKGFQNADIMDWTTMGIDLIGVAP-----IPGTTAIRTSARPALFLVRQEVKR
VGKAALG---------------------EAVIVVISNHLNDQVAGDIEKFCKTGLNKLNSILATCGATVQNA
TTQINGGIVRV---VTKGQIFTNAGSNASRAQ--QQAAGVIKGIKSGDLTRAGKNLVYMTENWGKALIKTGA
NSNAKVASSLVPAHLKQPILNVGATINKIGITANTKIKGLGNTAQPQTIGWLFDVLRMSASKFRKKK--SAQ
VKPTQTTQVKHDKKNNKLEKSNDEHAATHNANAC------------KNCKGGTTHSITFALGTEFL---NHI
DAQIHPLLIEQFSRTYVSNLYQYDQSIFGARWITPFTTKIVRCSSYNVHHPKEPIE---QDGWEYIGSDGRP
VRLPELKVGQSYYDE-VETFTYTVISP---------EIRA-----ISYGVSETR--------FYQKYKNEF-
RLASIERQNGFSIG---LRYDHELENSDSYLSDIIFKQQQQILAHVALQI--------NDAQKVVAAWLVEE
G---QLIRQL-------SAYQYNEQDDLIKATNEFA-ASYEYQY-QAHLLTRYTDLTHRGMNLKWDGV----
-----------------------LPSSKAIEEWADDYSSATKLDWDENVRVTSVMDIDGNVTEYYY---GIT
GYTYRIIYPDNLQEWFFRDQAKNITSHIATDGTETSYTYDERGNVLSMVQADGSTFYYEY-DEKDNLIGFVD
AEQGRWFKEYDASGNIIKEIDPLKRETVYAYNA---MGLVTSITDAKGGKKTLKYDDRGNLISYTDCSGK-E
TKWHYDDRGRVKFIENALKQKVEYFYTELTTELRQPIAKGLPLNAFGQL-------------EKIKHADGAE
EHFIHDAEGRLLVHIDPKGQQTRYEYDAAGLITKRTDPLNQTLKYQWDRLS-RLKRLINENGASYEFVYDAA
GRLVKEIDFDGKETVYAYDEYNGRLTTSIEVASTYGQDFKDRAAPKDR----IQQFIFDSMGRLEQRTAGYG
YQGQELEEKQTEEFSYDGLGNLVQAK------------NAETNLQWFYDAAGNLIKEHHQ---DVVTQKTAV
WKHVYDELNNRTTTIRP-DGQ---------KIDWLSY--GSGHVYGMALNGEDVVSFERDDLHRETLRHYAN
G------LSQQQSYDEVGRLTQQLMLSGHD-KGYQAQTQ-----NNAIQHTNQLIERLYHYDKTGELTLI--
```

--------------------------------KDTRRGAIHYKYDPVGRLL------EATSKLGKETFNFDP
ASNILER------YNSTKE-----------QSSQHISDEKGYGYNRLVNNIVQEYLDQQYQYDAFGQLIRQK
SSQGDL-----NLEWDVFGRLVR-------SRNNQYTAEYRYDALGRRIQKRSKH-------HHTGQEQNIS
YGWDGDTL--AYES----SADLTKHYFYEKDS-FVPLLQAAYHHPIELHQTQDWSD---KTYSIYKDPLWNT
VKQSQ--GFDDVWFYHCDHLGTPQEMSDQTGAIIWKAEYKA-WGECKLEQTNSDFFEKSEIISN-NIRFQGQ
YFDEETGLHYNRYRYYSPYVGRFISKDPIGLLGGFNVYAYTAN-PVQWVDPYGL
>AR_0056_6
---MSGKTEVVKPLTEVAIKDVSHSKQK-----------------IDSWLQSHTHQIVTLDLVASVMGTVPI
IGNVMAAIDVV-SDICYIYDKGFQNADIMDWTTMGIDLIGVAP-----IPGTTAIRTSARPALFLVRQEVKR
VGKAALG---------------------EAVIVVISNHLNDQVAGDIEKFCKTGLNKLNSILATCGATVQNA
TTQINGGIVRV---VTKGQIFTNAGSNASRAQ--QQAAGVIKGIKSGDLTRAGKNLVYMTENWGKALIKTGA
NSNAKVASSLVPAHLKQPILNVGATINKIGITANTKIKGLGNTAQPQTIGWLFDVLRMSASKFRKKK--SAQ
VKPTQTTQVKHDKKNNKLEKSNDEHAATHNANAC------------KNCKGGTTHSITFALGTEFL---NHI
DAQIHPLLIEQFSRTYVSNLYQYDQSIFGARWITPFTTKIVRCSSYNVHHPKEPIE---QDGWEYIGSDGRP
VRLPELKVGQSYYDE-VETFTYTVISP---------EIRA-----ISYGVSETR--------FYQKYKNEF-
RLASIERQNGFSIG---LRYDHELENSDSYLSDIIFKQQQQILAHVALQI--------NDAQKVVAAWLVEE
G---QLIRQL-------SAYQYNEQDDLIKATNEFA-ASYEYQY-QAHLLTRYTDLTHRGMNLKWDGV----
-----------------------LPSSKAIEEWADDYSSATKLDWDENVRVTSVMDIDGNVTEYYY---GIT
GYTYRIIYPDNLQEWFFRDQAKNITSHIATDGTETSYTYDERGNVLSMVQADGSTFYYEY-DEKDNLIGFVD
AEQGRWFKEYDASGNIIKEIDPLKRETVYAYNA---MGLVTSITDAKGGKKTLKYDDRGNLISYTDCSGK-E
TKWHYDDRGRVKFIENALKQKVEYFYTELTTELRQPIAKGLPLNAFGQL-------------EKIKHADGAE
EHFIHDAEGRLLVHIDPKGQQTRYEYDAAGLITKRTDPLNQTLKYQWDRLS-RLKRLINENGASYEFVYDAA
GRLVKEIDFDGKETVYAYDEYNGRLTTSIEVASTYGQDFKDRAAPKDR----IQQFIFDSMGRLEQRTAGYG
YQGQELEEKQTEEFSYDGLGNLVQAK------------NAETNLQWFYDAAGNLIKEHHQ---DVVTQKTAV
WKHVYDELNNRTTTIRP-DGQ---------KIDWLSY--GSGHVYGMALNGEDVVSFERDDLHRETLRHYAN
G------LSQQQSYDEVGRLTQQLMLSGHD-KGYQAQTQ-----NNAIQHTNQLIERLYHYDKTGELTLI--
--------------------------------KDTRRGAIHYKYDPVGRLL------EATSKLGKETFNFDP
ASNILER------YNSTKE-----------QSSQHISDEKGYGYNRLVNNIVQEYLDQQYQYDAFGQLIRQK
SSQGDL-----NLEWDVFGRLVR-------SRNNQYTAEYRYDALGRRIQKRSKH-------HHTGQEQNIS
YGWDGDTL--AYES----SADLTKHYFYEKDS-FVPLLQAAYHHPIELHQTQDWSD---KTYSIYKDPLWNT
VKQSQ--GFDDVWFYHCDHLGTPQEMSDQTGAIIWKAEYKA-WGECKLEQTNSDFFEKSEIISN-NIRFQGQ
YFDEETGLHYNRYRYYSPYVGRFISKDPIGLLGGFNVYAYTAN-PVQWVDPYGL
>AYP-A2_6

---MSGKTEVVKPLTEVAIKDVSHSKQK-----------------IDSWLQSHTHQIVTLDLVASVMGTVPI
IGNVMAAIDVV-SDICYIYDKGFQNADIMDWTTMGIDLIGVAP-----IPGTTAIRTSARPALFLVRQEVKR
VGKAALG---------------------EAVIVVISNHLNDQVAGDIEKFCKTGLNKLNSILATCGATVQNA
TTQINGGIVRV---VTKGQIFTNAGSNASRAQ--QQAAGVIKGIKSGDLTRAGKNLVYMTENWGKALIKTGA
NSNAKVASSLVPAHLKQPILNVGATINKIGITANTKIKGLGNTAQPQTIGWLFDVLRMSASKFRKKK--SAQ
VKPTQTTQVKHDKKNNKLEKSNDEHAATHNANAC------------KNCKGGTTHSITFALGTEFL---NHI
DAQIHPLLIEQFSRTYVSNLYQYDQSIFGARWITPFTTKIVRCSSYNVHHPKEPIE---QDGWEYIGSDGRP
VRLPELKVGQSYYDE-VETFTYTVISP---------EIRA-----ISYGVSETR--------FYQKYKNEF-
RLASIERQNGFSIG---LRYDHELENSDSYLSDIIFKQQQQILAHVALQI--------NDAQKVVAAWLVEE
G---QLIRQL-------SAYQYNEQDDLIKATNEFA-ASYEYQY-QAHLLTRYTDLTHRGMNLKWDGV----
------------------------LPSSKAIEEWADDYSSATKLDWDENVRVTSVMDIDGNVTEYYY---GIT
GYTYRIIYPDNLQEWFFRDQAKNITSHIATDGTETSYTYDERGNVLSMVQADGSTFYYEY-DEKDNLIGFVD
AEQGRWFKEYDASGNIIKEIDPLKRETVYAYNA---MGLVTSITDAKGGKKTLKYDDRGNLISYTDCSGK-E
TKWHYDDRGRVKFIENALKQKVEYFYTELTTELRQPIAKGLPLNAFGQL-------------EKIKHADGAE
EHFIHDAEGRLLVHIDPKGQQTRYEYDAAGLITKRTDPLNQTLKYQWDRLS-RLKRLINENGASYEFVYDAA
GRLVKEIDFDGKETVYAYDEYNGRLTTSIEVASTYGQDFKDRAAPKDR----IQQFIFDSMGRLEQRTAGYG
YQGQELEEKQTEEFSYDGLGNLVQAK------------NAETNLQWFYDAAGNLIKEHHQ---DVVTQKTAV
WKHVYDELNNRTTTIRP-DGQ---------KIDWLSY--GSGHVYGMALNGEDVVSFERDDLHRETLRHYAN
G------LSQQQSYDEVGRLTQQLMLSGHD-KGYQAQTQ-----NNAIQHTNQLIERLYHYDKTGELTLI--
----------------------------------KDTRRGAIHYKYDPVGRLL------EATSKLGKETFNFDP
ASNILER------YNSTKE-----------QSSQHISDEKGYGYNRLVNNIVQEYLDQQYQYDAFGQLIRQK
SSQGDL-----NLEWDVFGRLVR-------SRNNQYTAEYRYDALGRRIQKRSKH-------HHTGQEQNIS
YGWDGDTL--AYES----SADLTKHYFYEKDS-FVPLLQAAYHHPIELHQTQDWSD---KTYSIYKDPLWNT
VKQSQ--GFDDVWFYHCDHLGTPQEMSDQTGAIIWKAEYKA-WGECKLEQTNSDFFEKSEIISN-NIRFQGQ
YFDEETGLHYNRYRYYSPYVGRFISKDPIGLLGGFNVYAYTAN-PVQWVDPYGL
>BJAB07104_6
---MSGKTEVVKPLTEVAIKDVSHSKQK-----------------IDSWLQSHTHQIVTLDLVASVMGTVPI
IGNVMAAIDVV-SDICYIYDKGFQNADIMDWTTMGIDLIGVAP-----IPGTTAIRTSARPALFLVRQEVKR
VGKAALG---------------------EAVIVVISNHLNDQVAGDIEKFCKTGLNKLNSILATCGATVQNA
TTQINGGIVRV---VTKGQIFTNAGSNASRAQ--QQAAGVIKGIKSGDLTRAGKNLVYMTENWGKALIKTGA
NSNAKVASSLVPAHLKQPILNVGATINKIGITANTKIKGLGNTAQPQTIGWLFDVLRMSASKFRKKK--SAQ
VKPTQTTQVKHDKKNNKLEKSNDEHAATHNANAC------------KNCKGGTTHSITFALGTEFL---NHI
DAQIHPLLIEQFSRTYVSNLYQYDQSIFGARWITPFTTKIVRCSSYNVHHPKEPIE---QDGWEYIGSDGRP

VRLPELKVGQSYYDE-VETFTYTVISP---------EIRA-----ISYGVSETR--------FYQKYKNEF-
RLASIERQNGFSIG---LRYDHELENSDSYLSDIIFKQQQQILAHVALQI--------NDAQKVVAAWLVEE
G---QLIRQL-------SAYQYNEQDDLIKATNEFA-ASYEYQY-QAHLLTRYTDLTHRGMNLKWDGV----
-----------------------LPSSKAIEEWADDYSSATKLDWDENVRVTSVMDIDGNVTEYYY---GIT
GYTYRIIYPDNLQEWFFRDQAKNITSHIATDGTETSYTYDERGNVLSMVQADGSTFYYEY-DEKDNLIGFVD
AEQGRWFKEYDASGNIIKEIDPLKRETVYAYNA---MGLVTSITDAKGGKKTLKYDDRGNLISYTDCSGK-E
TKWHYDDRGRVKFIENALKQKVEYFYTELTTELRQPIAKGLPLNAFGQL-------------EKIKHADGAE
EHFIHDAEGRLLVHIDPKGQQTRYEYDAAGLITKRTDPLNQTLKYQWDRLS-RLKRLINENGASYEFVYDAA
GRLVKEIDFDGKETVYAYDEYNGRLTTSIEVASTYGQDFKDRAAPKDR----IQQFIFDSMGRLEQRTAGYG
YQGQELEEKQTEEFSYDGLGNLVQAK------------NAETNLQWFYDAAGNLIKEHHQ---DVVTQKTAV
WKHVYDELNNRTTTIRP-DGQ---------KIDWLSY--GSGHVYGMALNGEDVVSFERDDLHRETLRHYAN
G------LSQQQSYDEVGRLTQQLMLSGHD-KGYQAQTQ-----NNAIQHTNQLIERLYHYDKTGELTLI--
--------------------------------KDTRRGAIHYKYDPVGRLL------EATSKLGKETFNFDP
ASNILER------YNSTKE-----------QSSQHISDEKGYGYNRLVNNIVQEYLDQQYQYDAFGQLIRQK
SSQGDL-----NLEWDVFGRLVR-------SRNNQYTAEYRYDALGRRIQKRSKH-------HHTGQEQNIS
YGWDGDTL--AYES----SADLTKHYFYEKDS-FVPLLQAAYHHPIELHQTQDWSD---KTYSIYKDPLWNT
VKQSQ--GFDDVWFYHCDHLGTPQEMSDQTGAIIWKAEYKA-WGECKLEQTNSDFFEKSEIISN-NIRFQGQ
YFDEETGLHYNRYRYYSPYVGRFISKDPIGLLGGFNVYAYTAN-PVQWVDPYGL
>BJAB0868_6
---MSGKTEVVKPLTEVAIKDVSHSKQK-----------------IDSWLQSHTHQIVTLDLVASVMGTVPI
IGNVMAAIDVV-SDICYIYDKGFQNADIMDWTTMGIDLIGVAP-----IPGTTAIRTSARPALFLVRQEVKR
VGKAALG---------------------EAVIVVISNHLNDQVAGDIEKFCKTGLNKLNSILATCGATVQNA
TTQINGGIVRV---VTKGQIFTNAGSNASRAQ--QQAAGVIKGIKSGDLTRAGKNLVYMTENWGKALIKTGA
NSNAKVASSLVPAHLKQPILNVGATINKIGITANTKIKGLGNTAQPQTIGWLFDVLRMSASKFRKKK--SAQ
VKPTQTTQVKHDKKNNKLEKSNDEHAATHNANAC------------KNCKGGTTHSITFALGTEFL---NHI
DAQIHPLLIEQFSRTYVSNLYQYDQSIFGARWITPFTTKIVRCSSYNVHHPKEPIE---QDGWEYIGSDGRP
VRLPELKVGQSYYDE-VETFTYTVISP---------EIRA-----ISYGVSETR--------FYQKYKNEF-
RLASIERQNGFSIG---LRYDHELENSDSYLSDIIFKQQQQILAHVALQI--------NDAQKVVAAWLVEE
G---QLIRQL-------SAYQYNEQDDLIKATNEFA-ASYEYQY-QAHLLTRYTDLTHRGMNLKWDGV----
-----------------------LPSSKAIEEWADDYSSATKLDWDENVRVTSVMDIDGNVTEYYY---GIT
GYTYRIIYPDNLQEWFFRDQAKNITSHIATDGTETSYTYDERGNVLSMVQADGSTFYYEY-DEKDNLIGFVD
AEQGRWFKEYDASGNIIKEIDPLKRETVYAYNA---MGLVTSITDAKGGKKTLKYDDRGNLISYTDCSGK-E
TKWHYDDRGRVKFIENALKQKVEYFYTELTTELRQPIAKGLPLNAFGQL-------------EKIKHADGAE

EHFIHDAEGRLLVHIDPKGQQTRYEYDAAGLITKRTDPLNQTLKYQWDRLS-RLKRLINENGASYEFVYDAA
GRLVKEIDFDGKETVYAYDEYNGRLTTSIEVASTYGQDFKDRAAPKDR----IQQFIFDSMGRLEQRTAGYG
YQGQELEEKQTEEFSYDGLGNLVQAK------------NAETNLQWFYDAAGNLIKEHHQ---DVVTQKTAV
WKHVYDELNNRTTTIRP-DGQ---------KIDWLSY--GSGHVYGMALNGEDVVSFERDDLHRETLRHYAN
G------LSQQQSYDEVGRLTQQLMLSGHD-KGYQAQTQ-----NNAIQHTNQLIERLYHYDKTGELTLI--
--------------------------------KDTRRGAIHYKYDPVGRLL------EATSKLGKETFNFDP
ASNILER------YNSTKE-----------QSSQHISDEKGYGYNRLVNNIVQEYLDQQYQYDAFGQLIRQK
SSQGDL-----NLEWDVFGRLVR-------SRNNQYTAEYRYDALGRRIQKRSKH-------HHTGQEQNIS
YGWDGDTL--AYES----SADLTKHYFYEKDS-FVPLLQAAYHHPIELHQTQDWSD---KTYSIYKDPLWNT
VKQSQ--GFDDVWFYHCDHLGTPQEMSDQTGAIIWKAEYKA-WGECKLEQTNSDFFEKSEIISN-NIRFQGQ
YFDEETGLHYNRYRYYSPYVGRFISKDPIGLLGGFNVYAYTAN-PVQWVDPYGL
>CBA7_6
---MSGKTEVVKPLTEVAIKDVSHSKQK-----------------IDSWLQSHTHQIVTLDLVASVMGTVPI
IGNVMAAIDVV-SDICYIYDKGFQNADIMDWTTMGIDLIGVAP-----IPGTTAIRTSARPALFLVRQEVKR
VGKAALG---------------------EAVIVVISNHLNDQVAGDIEKFCKTGLNKLNSILATCGATVQNA
TTQINGGIVRV---VTKGQIFTNAGSNASRAQ--QQAAGVIKGIKSGDLTRAGKNLVYMTENWGKALIKTGA
NSNAKVASSLVPAHLKQPILNVGATINKIGITANTKIKGLGNTAQPQTIGWLFDVLRMSASKFRKKK--SAQ
VKPTQTTQVKHDKKNNKLEKSNDEHAATHNANAC------------KNCKGGTTHSITFALGTEFL---NHI
DAQIHPLLIEQFSRTYVSNLYQYDQSIFGARWITPFTTKIVRCSSYNVHHPKEPIE---QDGWEYIGSDGRP
VRLPELKVGQSYYDE-VETFTYTVISP---------EIRA-----ISYGVSETR--------FYQKYKNEF-
RLASIERQNGFSIG---LRYDHELENSDSYLSDIIFKQQQQILAHVALQI--------NDAQKVVAAWLVEE
G---QLIRQL-------SAYQYNEQDDLIKATNEFA-ASYEYQY-QAHLLTRYTDLTHRGMNLKWDGV----
-----------------------LPSSKAIEEWADDYSSATKLDWDENVRVTSVMDIDGNVTEYYY---GIT
GYTYRIIYPDNLQEWFFRDQAKNITSHIATDGTETSYTYDERGNVLSMVQADGSTFYYEY-DEKDNLIGFVD
AEQGRWFKEYDASGNIIKEIDPLKRETVYAYNA---MGLVTSITDAKGGKKTLKYDDRGNLISYTDCSGK-E
TKWHYDDRGRVKFIENALKQKVEYFYTELTTELRQPIAKGLPLNAFGQL-------------EKIKHADGAE
EHFIHDAEGRLLVHIDPKGQQTRYEYDAAGLITKRTDPLNQTLKYQWDRLS-RLKRLINENGASYEFVYDAA
GRLVKEIDFDGKETVYAYDEYNGRLTTSIEVASTYGQDFKDRAAPKDR----IQQFIFDSMGRLEQRTAGYG
YQGQELEEKQTEEFSYDGLGNLVQAK------------NAETNLQWFYDAAGNLIKEHHQ---DVVTQKTAV
WKHVYDELNNRTTTIRP-DGQ---------KIDWLSY--GSGHVYGMALNGEDVVSFERDDLHRETLRHYAN
G------LSQQQSYDEVGRLTQQLMLSGHD-KGYQAQTQ-----NNAIQHTNQLIERLYHYDKTGELTLI--
--------------------------------KDTRRGAIHYKYDPVGRLL------EATSKLGKETFNFDP
ASNILER------YNSTKE-----------QSSQHISDEKGYGYNRLVNNIVQEYLDQQYQYDAFGQLIRQK

SSQGDL-----NLEWDVFGRLVR-------SRNNQYTAEYRYDALGRRIQKRSKH-------HHTGQEQNIS
YGWDGDTL--AYES----SADLTKHYFYEKDS-FVPLLQAAYHHPIELHQTQDWSD---KTYSIYKDPLWNT
VKQSQ--GFDDVWFYHCDHLGTPQEMSDQTGAIIWKAEYKA-WGECKLEQTNSDFFEKSEIISN-NIRFQGQ
YFDEETGLHYNRYRYYSPYVGRFISKDPIGLLGGFNVYAYTAN-PVQWVDPYGL
>CMC-CR-MDR-Ab4_6
---MSGKTEVVKPLTEVAIKDVSHSKQK-----------------IDSWLQSHTHQIVTLDLVASVMGTVPI
IGNVMAAIDVV-SDICYIYDKGFQNADIMDWTTMGIDLIGVAP-----IPGTTAIRTSARPALFLVRQEVKR
VGKAALG---------------------EAVIVVISNHLNDQVAGDIEKFCKTGLNKLNSILATCGATVQNA
TTQINGGIVRV---VTKGQIFTNAGSNASRAQ--QQAAGVIKGIKSGDLTRAGKNLVYMTENWGKALIKTGA
NSNAKVASSLVPAHLKQPILNVGATINKIGITANTKIKGLGNTAQPQTIGWLFDVLRMSASKFRKKK--SAQ
VKPTQTTQVKHDKKNNKLEKSNDEHAATHNANAC------------KNCKGGTTHSITFALGTEFL---NHI
DAQIHPLLIEQFSRTYVSNLYQYDQSIFGARWITPFTTKIVRCSSYNVHHPKEPIE---QDGWEYIGSDGRP
VRLPELKVGQSYYDE-VETFTYTVISP---------EIRA-----ISYGVSETR--------FYQKYKNEF-
RLASIERQNGFSIG---LRYDHELENSDSYLSDIIFKQQQQILAHVALQI--------NDAQKVVAAWLVEE
G---QLIRQL-------SAYQYNEQDDLIKATNEFA-ASYEYQY-QAHLLTRYTDLTHRGMNLKWDGV----
-----------------------LPSSKAIEEWADDYSSATKLDWDENVRVTSVMDIDGNVTEYYY---GIT
GYTYRIIYPDNLQEWFFRDQAKNITSHIATDGTETSYTYDERGNVLSMVQADGSTFYYEY-DEKDNLIGFVD
AEQGRWFKEYDASGNIIKEIDPLKRETVYAYNA---MGLVTSITDAKGGKKTLKYDDRGNLISYTDCSGK-E
TKWHYDDRGRVKFIENALKQKVEYFYTELTTELRQPIAKGLPLNAFGQL-------------EKIKHADGAE
EHFIHDAEGRLLVHIDPKGQQTRYEYDAAGLITKRTDPLNQTLKYQWDRLS-RLKRLINENGASYEFVYDAA
GRLVKEIDFDGKETVYAYDEYNGRLTTSIEVASTYGQDFKDRAAPKDR----IQQFIFDSMGRLEQRTAGYG
YQGQELEEKQTEEFSYDGLGNLVQAK------------NAETNLQWFYDAAGNLIKEHHQ---DVVTQKTAV
WKHVYDELNNRTTTIRP-DGQ---------KIDWLSY--GSGHVYGMALNGEDVVSFERDDLHRETLRHYAN
G------LSQQQSYDEVGRLTQQLMLSGHD-KGYQAQTQ-----NNAIQHTNQLIERLYHYDKTGELTLI--
--------------------------------KDTRRGAIHYKYDPVGRLL------EATSKLGKETFNFDP
ASNILER------YNSTKE-----------QSSQHISDEKGYGYNRLVNNIVQEYLDQQYQYDAFGQLIRQK
SSQGDL-----NLEWDVFGRLVR-------SRNNQYTAEYRYDALGRRIQKRSKH-------HHTGQEQNIS
YGWDGDTL--AYES----SADLTKHYFYEKDS-FVPLLQAAYHHPIELHQTQDWSD---KTYSIYKDPLWNT
VKQSQ--GFDDVWFYHCDHLGTPQEMSDQTGAIIWKAEYKA-WGECKLEQTNSDFFEKSEIISN-NIRFQGQ
YFDEETGLHYNRYRYYSPYVGRFISKDPIGLLGGFNVYAYTAN-PVQWVDPYGL
>CMC-CR-MDR-Ab66_6
---MSGKTEVVKPLTEVAIKDVSHSKQK-----------------IDSWLQSHTHQIVTLDLVASVMGTVPI
IGNVMAAIDVV-SDICYIYDKGFQNADIMDWTTMGIDLIGVAP-----IPGTTAIRTSARPALFLVRQEVKR

VGKAALG---------------------EAVIVVISNHLNDQVAGDIEKFCKTGLNKLNSILATCGATVQNA
TTQINGGIVRV---VTKGQIFTNAGSNASRAQ--QQAAGVIKGIKSGDLTRAGKNLVYMTENWGKALIKTGA
NSNAKVASSLVPAHLKQPILNVGATINKIGITANTKIKGLGNTAQPQTIGWLFDVLRMSASKFRKKK--SAQ
VKPTQTTQVKHDKKNNKLEKSNDEHAATHNANAC------------KNCKGGTTHSITFALGTEFL---NHI
DAQIHPLLIEQFSRTYVSNLYQYDQSIFGARWITPFTTKIVRCSSYNVHHPKEPIE---QDGWEYIGSDGRP
VRLPELKVGQSYYDE-VETFTYTVISP---------EIRA-----ISYGVSETR--------FYQKYKNEF-
RLASIERQNGFSIG---LRYDHELENSDSYLSDIIFKQQQQILAHVALQI--------NDAQKVVAAWLVEE
G---QLIRQL-------SAYQYNEQDDLIKATNEFA-ASYEYQY-QAHLLTRYTDLTHRGMNLKWDGV----
-----------------------LPSSKAIEEWADDYSSATKLDWDENVRVTSVMDIDGNVTEYYY---GIT
GYTYRIIYPDNLQEWFFRDQAKNITSHIATDGTETSYTYDERGNVLSMVQADGSTFYYEY-DEKDNLIGFVD
AEQGRWFKEYDASGNIIKEIDPLKRETVYAYNA---MGLVTSITDAKGGKKTLKYDDRGNLISYTDCSGK-E
TKWHYDDRGRVKFIENALKQKVEYFYTELTTELRQPIAKGLPLNAFGQL-------------EKIKHADGAE
EHFIHDAEGRLLVHIDPKGQQTRYEYDAAGLITKRTDPLNQTLKYQWDRLS-RLKRLINENGASYEFVYDAA
GRLVKEIDFDGKETVYAYDEYNGRLTTSIEVASTYGQDFKDRAAPKDR----IQQFIFDSMGRLEQRTAGYG
YQGQELEEKQTEEFSYDGLGNLVQAK------------NAETNLQWFYDAAGNLIKEHHQ---DVVTQKTAV
WKHVYDELNNRTTTIRP-DGQ---------KIDWLSY--GSGHVYGMALNGEDVVSFERDDLHRETLRHYAN
G------LSQQQSYDEVGRLTQQLMLSGHD-KGYQAQTQ-----NNAIQHTNQLIERLYHYDKTGELTLI--
-------------------------------KDTRRGAIHYKYDPVGRLL------EATSKLGKETFNFDP
ASNILER------YNSTKE-----------QSSQHISDEKGYGYNRLVNNIVQEYLDQQYQYDAFGQLIRQK
SSQGDL-----NLEWDVFGRLVR-------SRNNQYTAEYRYDALGRRIQKRSKH-------HHTGQEQNIS
YGWDGDTL--AYES----SADLTKHYFYEKDS-FVPLLQAAYHHPIELHQTQDWSD---KTYSIYKDPLWNT
VKQSQ--GFDDVWFYHCDHLGTPQEMSDQTGAIIWKAEYKA-WGECKLEQTNSDFFEKSEIISN-NIRFQGQ
YFDEETGLHYNRYRYYSPYVGRFISKDPIGLLGGFNVYAYTAN-PVQWVDPYGL
>CMC-MDR-Ab59_6
---MSGKTEVVKPLTEVAIKDVSHSKQK-----------------IDSWLQSHTHQIVTLDLVASVMGTVPI
IGNVMAAIDVV-SDICYIYDKGFQNADIMDWTTMGIDLIGVAP-----IPGTTAIRTSARPALFLVRQEVKR
VGKAALG---------------------EAVIVVISNHLNDQVAGDIEKFCKTGLNKLNSILATCGATVQNA
TTQINGGIVRV---VTKGQIFTNAGSNASRAQ--QQAAGVIKGIKSGDLTRAGKNLVYMTENWGKALIKTGA
NSNAKVASSLVPAHLKQPILNVGATINKIGITANTKIKGLGNTAQPQTIGWLFDVLRMSASKFRKKK--SAQ
VKPTQTTQVKHDKKNNKLEKSNDEHAATHNANAC------------KNCKGGTTHSITFALGTEFL---NHI
DAQIHPLLIEQFSRTYVSNLYQYDQSIFGARWITPFTTKIVRCSSYNVHHPKEPIE---QDGWEYIGSDGRP
VRLPELKVGQSYYDE-VETFTYTVISP---------EIRA-----ISYGVSETR--------FYQKYKNEF-
RLASIERQNGFSIG---LRYDHELENSDSYLSDIIFKQQQQILAHVALQI--------NDAQKVVAAWLVEE

G---QLIRQL-------SAYQYNEQDDLIKATNEFA-ASYEYQY-QAHLLTRYTDLTHRGMNLKWDGV----
-----------------------LPSSKAIEEWADDYSSATKLDWDENVRVTSVMDIDGNVTEYYY---GIT
GYTYRIIYPDNLQEWFFRDQAKNITSHIATDGTETSYTYDERGNVLSMVQADGSTFYYEY-DEKDNLIGFVD
AEQGRWFKEYDASGNIIKEIDPLKRETVYAYNA---MGLVTSITDAKGGKKTLKYDDRGNLISYTDCSGK-E
TKWHYDDRGRVKFIENALKQKVEYFYTELTTELRQPIAKGLPLNAFGQL-------------EKIKHADGAE
EHFIHDAEGRLLVHIDPKGQQTRYEYDAAGLITKRTDPLNQTLKYQWDRLS-RLKRLINENGASYEFVYDAA
GRLVKEIDFDGKETVYAYDEYNGRLTTSIEVASTYGQDFKDRAAPKDR----IQQFIFDSMGRLEQRTAGYG
YQGQELEEKQTEEFSYDGLGNLVQAK------------NAETNLQWFYDAAGNLIKEHHQ---DVVTQKTAV
WKHVYDELNNRTTTIRP-DGQ---------KIDWLSY--GSGHVYGMALNGEDVVSFERDDLHRETLRHYAN
G------LSQQQSYDEVGRLTQQLMLSGHD-KGYQAQTQ-----NNAIQHTNQLIERLYHYDKTGELTLI--
--------------------------------KDTRRGAIHYKYDPVGRLL------EATSKLGKETFNFDP
ASNILER------YNSTKE-----------QSSQHISDEKGYGYNRLVNNIVQEYLDQQYQYDAFGQLIRQK
SSQGDL-----NLEWDVFGRLVR-------SRNNQYTAEYRYDALGRRIQKRSKH-------HHTGQEQNIS
YGWDGDTL--AYES----SADLTKHYFYEKDS-FVPLLQAAYHHPIELHQTQDWSD---KTYSIYKDPLWNT
VKQSQ--GFDDVWFYHCDHLGTPQEMSDQTGAIIWKAEYKA-WGECKLEQTNSDFFEKSEIISN-NIRFQGQ
YFDEETGLHYNRYRYYSPYVGRFISKDPIGLLGGFNVYAYTAN-PVQWVDPYGL
>DU202_6
---MSGKTEVVKPLTEVAIKDVSHSKQK-----------------IDSWLQSHTHQIVTLDLVASVMGTVPI
IGNVMAAIDVV-SDICYIYDKGFQNADIMDWTTMGIDLIGVAP-----IPGTTAIRTSARPALFLVRQEVKR
VGKAALG---------------------EAVIVVISNHLNDQVAGDIEKFCKTGLNKLNSILATCGATVQNA
TTQINGGIVRV---VTKGQIFTNAGSNASRAQ--QQAAGVIKGIKSGDLTRAGKNLVYMTENWGKALIKTGA
NSNAKVASSLVPAHLKQPILNVGATINKIGITANTKIKGLGNTAQPQTIGWLFDVLRMSASKFRKKK--SAQ
VKPTQTTQVKHDKKNNKLEKSNDEHAATHNANAC------------KNCKGGTTHSITFALGTEFL---NHI
DAQIHPLLIEQFSRTYVSNLYQYDQSIFGARWITPFTTKIVRCSSYNVHHPKEPIE---QDGWEYIGSDGRP
VRLPELKVGQSYYDE-VETFTYTVISP---------EIRA-----ISYGVSETR--------FYQKYKNEF-
RLASIERQNGFSIG---LRYDHELENSDSYLSDIIFKQQQQILAHVALQI--------NDAQKVVAAWLVEE
G---QLIRQL-------SAYQYNEQDDLIKATNEFA-ASYEYQY-QAHLLTRYTDLTHRGMNLKWDGV----
-----------------------LPSSKAIEEWADDYSSATKLDWDENVRVTSVMDIDGNVTEYYY---GIT
GYTYRIIYPDNLQEWFFRDQAKNITSHIATDGTETSYTYDERGNVLSMVQADGSTFYYEY-DEKDNLIGFVD
AEQGRWFKEYDASGNIIKEIDPLKRETVYAYNA---MGLVTSITDAKGGKKTLKYDDRGNLISYTDCSGK-E
TKWHYDDRGRVKFIENALKQKVEYFYTELTTELRQPIAKGLPLNAFGQL-------------EKIKHADGAE
EHFIHDAEGRLLVHIDPKGQQTRYEYDAAGLITKRTDPLNQTLKYQWDRLS-RLKRLINENGASYEFVYDAA
GRLVKEIDFDGKETVYAYDEYNGRLTTSIEVASTYGQDFKDRAAPKDR----IQQFIFDSMGRLEQRTAGYG

```
YQGQELEEKQTEEFSYDGLGNLVQAK------------NAETNLQWFYDAAGNLIKEHHQ---DVVTQKTAV
WKHVYDELNNRTTTIRP-DGQ---------KIDWLSY--GSGHVYGMALNGEDVVSFERDDLHRETLRHYAN
G------LSQQQSYDEVGRLTQQLMLSGHD-KGYQAQTQ-----NNAIQHTNQLIERLYHYDKTGELTLI--
--------------------------------KDTRRGAIHYKYDPVGRLL------EATSKLGKETFNFDP
ASNILER------YNSTKE-----------QSSQHISDEKGYGYNRLVNNIVQEYLDQQYQYDAFGQLIRQK
SSQGDL-----NLEWDVFGRLVR-------SRNNQYTAEYRYDALGRRIQKRSKH-------HHTGQEQNIS
YGWDGDTL--AYES----SADLTKHYFYEKDS-FVPLLQAAYHHPIELHQTQDWSD---KTYSIYKDPLWNT
VKQSQ--GFDDVWFYHCDHLGTPQEMSDQTGAIIWKAEYKA-WGECKLEQTNSDFFEKSEIISN-NIRFQGQ
YFDEETGLHYNRYRYYSPYVGRFISKDPIGLLGGFNVYAYTAN-PVQWVDPYGL
>HRAB-85_6
---MSGKTEVVKPLTEVAIKDVSHSKQK-----------------IDSWLQSHTHQIVTLDLVASVMGTVPI
IGNVMAAIDVV-SDICYIYDKGFQNADIMDWTTMGIDLIGVAP-----IPGTTAIRTSARPALFLVRQEVKR
VGKAALG---------------------EAVIVVISNHLNDQVAGDIEKFCKTGLNKLNSILATCGATVQNA
TTQINGGIVRV---VTKGQIFTNAGSNASRAQ--QQAAGVIKGIKSGDLTRAGKNLVYMTENWGKALIKTGA
NSNAKVASSLVPAHLKQPILNVGATINKIGITANTKIKGLGNTAQPQTIGWLFDVLRMSASKFRKKK--SAQ
VKPTQTTQVKHDKKNNKLEKSNDEHAATHNANAC------------KNCKGGTTHSITFALGTEFL---NHI
DAQIHPLLIEQFSRTYVSNLYQYDQSIFGARWITPFTTKIVRCSSYNVHHPKEPIE---QDGWEYIGSDGRP
VRLPELKVGQSYYDE-VETFTYTVISP---------EIRA-----ISYGVSETR--------FYQKYKNEF-
RLASIERQNGFSIG---LRYDHELENSDSYLSDIIFKQQQQILAHVALQI--------NDAQKVVAAWLVEE
G---QLIRQL-------SAYQYNEQDDLIKATNEFA-ASYEYQY-QAHLLTRYTDLTHRGMNLKWDGV----
-----------------------LPSSKAIEEWADDYSSATKLDWDENVRVTSVMDIDGNVTEYYY---GIT
GYTYRIIYPDNLQEWFFRDQAKNITSHIATDGTETSYTYDERGNVLSMVQADGSTFYYEY-DEKDNLIGFVD
AEQGRWFKEYDASGNIIKEIDPLKRETVYAYNA---MGLVTSITDAKGGKKTLKYDDRGNLISYTDCSGK-E
TKWHYDDRGRVKFIENALKQKVEYFYTELTTELRQPIAKGLPLNAFGQL-------------EKIKHADGAE
EHFIHDAEGRLLVHIDPKGQQTRYEYDAAGLITKRTDPLNQTLKYQWDRLS-RLKRLINENGASYEFVYDAA
GRLVKEIDFDGKETVYAYDEYNGRLTTSIEVASTYGQDFKDRAAPKDR----IQQFIFDSMGRLEQRTAGYG
YQGQELEEKQTEEFSYDGLGNLVQAK------------NAETNLQWFYDAAGNLIKEHHQ---DVVTQKTAV
WKHVYDELNNRTTTIRP-DGQ---------KIDWLSY--GSGHVYGMALNGEDVVSFERDDLHRETLRHYAN
G------LSQQQSYDEVGRLTQQLMLSGHD-KGYQAQTQ-----NNAIQHTNQLIERLYHYDKTGELTLI--
--------------------------------KDTRRGAIHYKYDPVGRLL------EATSKLGKETFNFDP
ASNILER------YNSTKE-----------QSSQHISDEKGYGYNRLVNNIVQEYLDQQYQYDAFGQLIRQK
SSQGDL-----NLEWDVFGRLVR-------SRNNQYTAEYRYDALGRRIQKRSKH-------HHTGQEQNIS
YGWDGDTL--AYES----SADLTKHYFYEKDS-FVPLLQAAYHHPIELHQTQDWSD---KTYSIYKDPLWNT
```

VKQSQ--GFDDVWFYHCDHLGTPQEMSDQTGAIIWKAEYKA-WGECKLEQTNSDFFEKSEIISN-NIRFQGQ
YFDEETGLHYNRYRYYSPYVGRFISKDPIGLLGGFNVYAYTAN-PVQWVDPYGL
>JBA13_6
---MSGKTEVVKPLTEVAIKDVSHSKQK-----------------IDSWLQSHTHQIVTLDLVASVMGTVPI
IGNVMAAIDVV-SDICYIYDKGFQNADIMDWTTMGIDLIGVAP-----IPGTTAIRTSARPALFLVRQEVKR
VGKAALG---------------------EAVIVVISNHLNDQVAGDIEKFCKTGLNKLNSILATCGATVQNA
TTQINGGIVRV---VTKGQIFTNAGSNASRAQ--QQAAGVIKGIKSGDLTRAGKNLVYMTENWGKALIKTGA
NSNAKVASSLVPAHLKQPILNVGATINKIGITANTKIKGLGNTAQPQTIGWLFDVLRMSASKFRKKK--SAQ
VKPTQTTQVKHDKKNNKLEKSNDEHAATHNANAC------------KNCKGGTTHSITFALGTEFL---NHI
DAQIHPLLIEQFSRTYVSNLYQYDQSIFGARWITPFTTKIVRCSSYNVHHPKEPIE---QDGWEYIGSDGRP
VRLPELKVGQSYYDE-VETFTYTVISP---------EIRA-----ISYGVSETR--------FYQKYKNEF-
RLASIERQNGFSIG---LRYDHELENSDSYLSDIIFKQQQQILAHVALQI--------NDAQKVVAAWLVEE
G---QLIRQL-------SAYQYNEQDDLIKATNEFA-ASYEYQY-QAHLLTRYTDLTHRGMNLKWDGV----
------------------------LPSSKAIEEWADDYSSATKLDWDENVRVTSVMDIDGNVTEYYY---GIT
GYTYRIIYPDNLQEWFFRDQAKNITSHIATDGTETSYTYDERGNVLSMVQADGSTFYYEY-DEKDNLIGFVD
AEQGRWFKEYDASGNIIKEIDPLKRETVYAYNA---MGLVTSITDAKGGKKTLKYDDRGNLISYTDCSGK-E
TKWHYDDRGRVKFIENALKQKVEYFYTELTTELRQPIAKGLPLNAFGQL-------------EKIKHADGAE
EHFIHDAEGRLLVHIDPKGQQTRYEYDAAGLITKRTDPLNQTLKYQWDRLS-RLKRLINENGASYEFVYDAA
GRLVKEIDFDGKETVYAYDEYNGRLTTSIEVASTYGQDFKDRAAPKDR----IQQFIFDSMGRLEQRTAGYG
YQGQELEEKQTEEFSYDGLGNLVQAK------------NAETNLQWFYDAAGNLIKEHHQ---DVVTQKTAV
WKHVYDELNNRTTTIRP-DGQ---------KIDWLSY--GSGHVYGMALNGEDVVSFERDDLHRETLRHYAN
G------LSQQQSYDEVGRLTQQLMLSGHD-KGYQAQTQ-----NNAIQHTNQLIERLYHYDKTGELTLI--
---------------------------------KDTRRGAIHYKYDPVGRLL------EATSKLGKETFNFDP
ASNILER------YNSTKE-----------QSSQHISDEKGYGYNRLVNNIVQEYLDQQYQYDAFGQLIRQK
SSQGDL-----NLEWDVFGRLVR-------SRNNQYTAEYRYDALGRRIQKRSKH-------HHTGQEQNIS
YGWDGDTL--AYES----SADLTKHYFYEKDS-FVPLLQAAYHHPIELHQTQDWSD---KTYSIYKDPLWNT
VKQSQ--GFDDVWFYHCDHLGTPQEMSDQTGAIIWKAEYKA-WGECKLEQTNSDFFEKSEIISN-NIRFQGQ
YFDEETGLHYNRYRYYSPYVGRFISKDPIGLLGGFNVYAYTAN-PVQWVDPYGL
>KAB01_6
---MSGKTEVVKPLTEVAIKDVSHSKQK-----------------IDSWLQSHTHQIVTLDLVASVMGTVPI
IGNVMAAIDVV-SDICYIYDKGFQNADIMDWTTMGIDLIGVAP-----IPGTTAIRTSARPALFLVRQEVKR
VGKAALG---------------------EAVIVVISNHLNDQVAGDIEKFCKTGLNKLNSILATCGATVQNA
TTQINGGIVRV---VTKGQIFTNAGSNASRAQ--QQAAGVIKGIKSGDLTRAGKNLVYMTENWGKALIKTGA

NSNAKVASSLVPAHLKQPILNVGATINKIGITANTKIKGLGNTAQPQTIGWLFDVLRMSASKFRKKK--SAQ
VKPTQTTQVKHDKKNNKLEKSNDEHAATHNANAC------------KNCKGGTTHSITFALGTEFL---NHI
DAQIHPLLIEQFSRTYVSNLYQYDQSIFGARWITPFTTKIVRCSSYNVHHPKEPIE---QDGWEYIGSDGRP
VRLPELKVGQSYYDE-VETFTYTVISP---------EIRA-----ISYGVSETR--------FYQKYKNEF-
RLASIERQNGFSIG---LRYDHELENSDSYLSDIIFKQQQQILAHVALQI--------NDAQKVVAAWLVEE
G---QLIRQL-------SAYQYNEQDDLIKATNEFA-ASYEYQY-QAHLLTRYTDLTHRGMNLKWDGV----
-----------------------LPSSKAIEEWADDYSSATKLDWDENVRVTSVMDIDGNVTEYYY---GIT
GYTYRIIYPDNLQEWFFRDQAKNITSHIATDGTETSYTYDERGNVLSMVQADGSTFYYEY-DEKDNLIGFVD
AEQGRWFKEYDASGNIIKEIDPLKRETVYAYNA---MGLVTSITDAKGGKKTLKYDDRGNLISYTDCSGK-E
TKWHYDDRGRVKFIENALKQKVEYFYTELTTELRQPIAKGLPLNAFGQL-------------EKIKHADGAE
EHFIHDAEGRLLVHIDPKGQQTRYEYDAAGLITKRTDPLNQTLKYQWDRLS-RLKRLINENGASYEFVYDAA
GRLVKEIDFDGKETVYAYDEYNGRLTTSIEVASTYGQDFKDRAAPKDR----IQQFIFDSMGRLEQRTAGYG
YQGQELEEKQTEEFSYDGLGNLVQAK------------NAETNLQWFYDAAGNLIKEHHQ---DVVTQKTAV
WKHVYDELNNRTTTIRP-DGQ---------KIDWLSY--GSGHVYGMALNGEDVVSFERDDLHRETLRHYAN
G------LSQQQSYDEVGRLTQQLMLSGHD-KGYQAQTQ-----NNAIQHTNQLIERLYHYDKTGELTLI--
--------------------------------KDTRRGAIHYKYDPVGRLL------EATSKLGKETFNFDP
ASNILER------YNSTKE-----------QSSQHISDEKGYGYNRLVNNIVQEYLDQQYQYDAFGQLIRQK
SSQGDL-----NLEWDVFGRLVR-------SRNNQYTAEYRYDALGRRIQKRSKH-------HHTGQEQNIS
YGWDGDTL--AYES----SADLTKHYFYEKDS-FVPLLQAAYHHPIELHQTQDWSD---KTYSIYKDPLWNT
VKQSQ--GFDDVWFYHCDHLGTPQEMSDQTGAIIWKAEYKA-WGECKLEQTNSDFFEKSEIISN-NIRFQGQ
YFDEETGLHYNRYRYYSPYVGRFISKDPIGLLGGFNVYAYTAN-PVQWVDPYGL
>KAB02_6
---MSGKTEVVKPLTEVAIKDVSHSKQK-----------------IDSWLQSHTHQIVTLDLVASVMGTVPI
IGNVMAAIDVV-SDICYIYDKGFQNADIMDWTTMGIDLIGVAP-----IPGTTAIRTSARPALFLVRQEVKR
VGKAALG---------------------EAVIVVISNHLNDQVAGDIEKFCKTGLNKLNSILATCGATVQNA
TTQINGGIVRV---VTKGQIFTNAGSNASRAQ--QQAAGVIKGIKSGDLTRAGKNLVYMTENWGKALIKTGA
NSNAKVASSLVPAHLKQPILNVGATINKIGITANTKIKGLGNTAQPQTIGWLFDVLRMSASKFRKKK--SAQ
VKPTQTTQVKHDKKNNKLEKSNDEHAATHNANAC------------KNCKGGTTHSITFALGTEFL---NHI
DAQIHPLLIEQFSRTYVSNLYQYDQSIFGARWITPFTTKIVRCSSYNVHHPKEPIE---QDGWEYIGSDGRP
VRLPELKVGQSYYDE-VETFTYTVISP---------EIRA-----ISYGVSETR--------FYQKYKNEF-
RLASIERQNGFSIG---LRYDHELENSDSYLSDIIFKQQQQILAHVALQI--------NDAQKVVAAWLVEE
G---QLIRQL-------SAYQYNEQDDLIKATNEFA-ASYEYQY-QAHLLTRYTDLTHRGMNLKWDGV----
-----------------------LPSSKAIEEWADDYSSATKLDWDENVRVTSVMDIDGNVTEYYY---GIT

GYTYRIIYPDNLQEWFFRDQAKNITSHIATDGTETSYTYDERGNVLSMVQADGSTFYYEY-DEKDNLIGFVD
AEQGRWFKEYDASGNIIKEIDPLKRETVYAYNA---MGLVTSITDAKGGKKTLKYDDRGNLISYTDCSGK-E
TKWHYDDRGRVKFIENALKQKVEYFYTELTTELRQPIAKGLPLNAFGQL-------------EKIKHADGAE
EHFIHDAEGRLLVHIDPKGQQTRYEYDAAGLITKRTDPLNQTLKYQWDRLS-RLKRLINENGASYEFVYDAA
GRLVKEIDFDGKETVYAYDEYNGRLTTSIEVASTYGQDFKDRAAPKDR----IQQFIFDSMGRLEQRTAGYG
YQGQELEEKQTEEFSYDGLGNLVQAK------------NAETNLQWFYDAAGNLIKEHHQ---DVVTQKTAV
WKHVYDELNNRTTTIRP-DGQ---------KIDWLSY--GSGHVYGMALNGEDVVSFERDDLHRETLRHYAN
G------LSQQQSYDEVGRLTQQLMLSGHD-KGYQAQTQ-----NNAIQHTNQLIERLYHYDKTGELTLI--
--------------------------------KDTRRGAIHYKYDPVGRLL------EATSKLGKETFNFDP
ASNILER------YNSTKE-----------QSSQHISDEKGYGYNRLVNNIVQEYLDQQYQYDAFGQLIRQK
SSQGDL-----NLEWDVFGRLVR-------SRNNQYTAEYRYDALGRRIQKRSKH-------HHTGQEQNIS
YGWDGDTL--AYES----SADLTKHYFYEKDS-FVPLLQAAYHHPIELHQTQDWSD---KTYSIYKDPLWNT
VKQSQ--GFDDVWFYHCDHLGTPQEMSDQTGAIIWKAEYKA-WGECKLEQTNSDFFEKSEIISN-NIRFQGQ
YFDEETGLHYNRYRYYSPYVGRFISKDPIGLLGGFNVYAYTAN-PVQWVDPYGL
>KAB03_6
---MSGKTEVVKPLTEVAIKDVSHSKQK-----------------IDSWLQSHTHQIVTLDLVASVMGTVPI
IGNVMAAIDVV-SDICYIYDKGFQNADIMDWTTMGIDLIGVAP-----IPGTTAIRTSARPALFLVRQEVKR
VGKAALG---------------------EAVIVVISNHLNDQVAGDIEKFCKTGLNKLNSILATCGATVQNA
TTQINGGIVRV---VTKGQIFTNAGSNASRAQ--QQAAGVIKGIKSGDLTRAGKNLVYMTENWGKALIKTGA
NSNAKVASSLVPAHLKQPILNVGATINKIGITANTKIKGLGNTAQPQTIGWLFDVLRMSASKFRKKK--SAQ
VKPTQTTQVKHDKKNNKLEKSNDEHAATHNANAC------------KNCKGGTTHSITFALGTEFL---NHI
DAQIHPLLIEQFSRTYVSNLYQYDQSIFGARWITPFTTKIVRCSSYNVHHPKEPIE---QDGWEYIGSDGRP
VRLPELKVGQSYYDE-VETFTYTVISP---------EIRA-----ISYGVSETR--------FYQKYKNEF-
RLASIERQNGFSIG---LRYDHELENSDSYLSDIIFKQQQQILAHVALQI--------NDAQKVVAAWLVEE
G---QLIRQL-------SAYQYNEQDDLIKATNEFA-ASYEYQY-QAHLLTRYTDLTHRGMNLKWDGV----
-----------------------LPSSKAIEEWADDYSSATKLDWDENVRVTSVMDIDGNVTEYYY---GIT
GYTYRIIYPDNLQEWFFRDQAKNITSHIATDGTETSYTYDERGNVLSMVQADGSTFYYEY-DEKDNLIGFVD
AEQGRWFKEYDASGNIIKEIDPLKRETVYAYNA---MGLVTSITDAKGGKKTLKYDDRGNLISYTDCSGK-E
TKWHYDDRGRVKFIENALKQKVEYFYTELTTELRQPIAKGLPLNAFGQL-------------EKIKHADGAE
EHFIHDAEGRLLVHIDPKGQQTRYEYDAAGLITKRTDPLNQTLKYQWDRLS-RLKRLINENGASYEFVYDAA
GRLVKEIDFDGKETVYAYDEYNGRLTTSIEVASTYGQDFKDRAAPKDR----IQQFIFDSMGRLEQRTAGYG
YQGQELEEKQTEEFSYDGLGNLVQAK------------NAETNLQWFYDAAGNLIKEHHQ---DVVTQKTAV
WKHVYDELNNRTTTIRP-DGQ---------KIDWLSY--GSGHVYGMALNGEDVVSFERDDLHRETLRHYAN

```
G------LSQQQSYDEVGRLTQQLMLSGHD-KGYQAQTQ-----NNAIQHTNQLIERLYHYDKTGELTLI--
--------------------------------KDTRRGAIHYKYDPVGRLL------EATSKLGKETFNFDP
ASNILER------YNSTKE-----------QSSQHISDEKGYGYNRLVNNIVQEYLDQQYQYDAFGQLIRQK
SSQGDL-----NLEWDVFGRLVR-------SRNNQYTAEYRYDALGRRIQKRSKH-------HHTGQEQNIS
YGWDGDTL--AYES----SADLTKHYFYEKDS-FVPLLQAAYHHPIELHQTQDWSD---KTYSIYKDPLWNT
VKQSQ--GFDDVWFYHCDHLGTPQEMSDQTGAIIWKAEYKA-WGECKLEQTNSDFFEKSEIISN-NIRFQGQ
YFDEETGLHYNRYRYYSPYVGRFISKDPIGLLGGFNVYAYTAN-PVQWVDPYGL
>KAB04_6
---MSGKTEVVKPLTEVAIKDVSHSKQK-----------------IDSWLQSHTHQIVTLDLVASVMGTVPI
IGNVMAAIDVV-SDICYIYDKGFQNADIMDWTTMGIDLIGVAP-----IPGTTAIRTSARPALFLVRQEVKR
VGKAALG---------------------EAVIVVISNHLNDQVAGDIEKFCKTGLNKLNSILATCGATVQNA
TTQINGGIVRV---VTKGQIFTNAGSNASRAQ--QQAAGVIKGIKSGDLTRAGKNLVYMTENWGKALIKTGA
NSNAKVASSLVPAHLKQPILNVGATINKIGITANTKIKGLGNTAQPQTIGWLFDVLRMSASKFRKKK--SAQ
VKPTQTTQVKHDKKNNKLEKSNDEHAATHNANAC------------KNCKGGTTHSITFALGTEFL---NHI
DAQIHPLLIEQFSRTYVSNLYQYDQSIFGARWITPFTTKIVRCSSYNVHHPKEPIE---QDGWEYIGSDGRP
VRLPELKVGQSYYDE-VETFTYTVISP---------EIRA-----ISYGVSETR--------FYQKYKNEF-
RLASIERQNGFSIG---LRYDHELENSDSYLSDIIFKQQQQILAHVALQI--------NDAQKVVAAWLVEE
G---QLIRQL-------SAYQYNEQDDLIKATNEFA-ASYEYQY-QAHLLTRYTDLTHRGMNLKWDGV----
-----------------------LPSSKAIEEWADDYSSATKLDWDENVRVTSVMDIDGNVTEYYY---GIT
GYTYRIIYPDNLQEWFFRDQAKNITSHIATDGTETSYTYDERGNVLSMVQADGSTFYYEY-DEKDNLIGFVD
AEQGRWFKEYDASGNIIKEIDPLKRETVYAYNA---MGLVTSITDAKGGKKTLKYDDRGNLISYTDCSGK-E
TKWHYDDRGRVKFIENALKQKVEYFYTELTTELRQPIAKGLPLNAFGQL-------------EKIKHADGAE
EHFIHDAEGRLLVHIDPKGQQTRYEYDAAGLITKRTDPLNQTLKYQWDRLS-RLKRLINENGASYEFVYDAA
GRLVKEIDFDGKETVYAYDEYNGRLTTSIEVASTYGQDFKDRAAPKDR----IQQFIFDSMGRLEQRTAGYG
YQGQELEEKQTEEFSYDGLGNLVQAK------------NAETNLQWFYDAAGNLIKEHHQ---DVVTQKTAV
WKHVYDELNNRTTTIRP-DGQ---------KIDWLSY--GSGHVYGMALNGEDVVSFERDDLHRETLRHYAN
G------LSQQQSYDEVGRLTQQLMLSGHD-KGYQAQTQ-----NNAIQHTNQLIERLYHYDKTGELTLI--
--------------------------------KDTRRGAIHYKYDPVGRLL------EATSKLGKETFNFDP
ASNILER------YNSTKE-----------QSSQHISDEKGYGYNRLVNNIVQEYLDQQYQYDAFGQLIRQK
SSQGDL-----NLEWDVFGRLVR-------SRNNQYTAEYRYDALGRRIQKRSKH-------HHTGQEQNIS
YGWDGDTL--AYES----SADLTKHYFYEKDS-FVPLLQAAYHHPIELHQTQDWSD---KTYSIYKDPLWNT
VKQSQ--GFDDVWFYHCDHLGTPQEMSDQTGAIIWKAEYKA-WGECKLEQTNSDFFEKSEIISN-NIRFQGQ
YFDEETGLHYNRYRYYSPYVGRFISKDPIGLLGGFNVYAYTAN-PVQWVDPYGL
```

>KAB05_6
---MSGKTEVVKPLTEVAIKDVSHSKQK-----------------IDSWLQSHTHQIVTLDLVASVMGTVPI
IGNVMAAIDVV-SDICYIYDKGFQNADIMDWTTMGIDLIGVAP-----IPGTTAIRTSARPALFLVRQEVKR
VGKAALG---------------------EAVIVVISNHLNDQVAGDIEKFCKTGLNKLNSILATCGATVQNA
TTQINGGIVRV---VTKGQIFTNAGSNASRAQ--QQAAGVIKGIKSGDLTRAGKNLVYMTENWGKALIKTGA
NSNAKVASSLVPAHLKQPILNVGATINKIGITANTKIKGLGNTAQPQTIGWLFDVLRMSASKFRKKK--SAQ
VKPTQTTQVKHDKKNNKLEKSNDEHAATHNANAC------------KNCKGGTTHSITFALGTEFL---NHI
DAQIHPLLIEQFSRTYVSNLYQYDQSIFGARWITPFTTKIVRCSSYNVHHPKEPIE---QDGWEYIGSDGRP
VRLPELKVGQSYYDE-VETFTYTVISP---------EIRA-----ISYGVSETR--------FYQKYKNEF-
RLASIERQNGFSIG---LRYDHELENSDSYLSDIIFKQQQQILAHVALQI--------NDAQKVVAAWLVEE
G---QLIRQL-------SAYQYNEQDDLIKATNEFA-ASYEYQY-QAHLLTRYTDLTHRGMNLKWDGV----
-----------------------LPSSKAIEEWADDYSSATKLDWDENVRVTSVMDIDGNVTEYYY---GIT
GYTYRIIYPDNLQEWFFRDQAKNITSHIATDGTETSYTYDERGNVLSMVQADGSTFYYEY-DEKDNLIGFVD
AEQGRWFKEYDASGNIIKEIDPLKRETVYAYNA---MGLVTSITDAKGGKKTLKYDDRGNLISYTDCSGK-E
TKWHYDDRGRVKFIENALKQKVEYFYTELTTELRQPIAKGLPLNAFGQL-------------EKIKHADGAE
EHFIHDAEGRLLVHIDPKGQQTRYEYDAAGLITKRTDPLNQTLKYQWDRLS-RLKRLINENGASYEFVYDAA
GRLVKEIDFDGKETVYAYDEYNGRLTTSIEVASTYGQDFKDRAAPKDR----IQQFIFDSMGRLEQRTAGYG
YQGQELEEKQTEEFSYDGLGNLVQAK------------NAETNLQWFYDAAGNLIKEHHQ---DVVTQKTAV
WKHVYDELNNRTTTIRP-DGQ---------KIDWLSY--GSGHVYGMALNGEDVVSFERDDLHRETLRHYAN
G------LSQQQSYDEVGRLTQQLMLSGHD-KGYQAQTQ-----NNAIQHTNQLIERLYHYDKTGELTLI--
--------------------------------KDTRRGAIHYKYDPVGRLL------EATSKLGKETFNFDP
ASNILER------YNSTKE-----------QSSQHISDEKGYGYNRLVNNIVQEYLDQQYQYDAFGQLIRQK
SSQGDL-----NLEWDVFGRLVR-------SRNNQYTAEYRYDALGRRIQKRSKH-------HHTGQEQNIS
YGWDGDTL--AYES----SADLTKHYFYEKDS-FVPLLQAAYHHPIELHQTQDWSD---KTYSIYKDPLWNT
VKQSQ--GFDDVWFYHCDHLGTPQEMSDQTGAIIWKAEYKA-WGECKLEQTNSDFFEKSEIISN-NIRFQGQ
YFDEETGLHYNRYRYYSPYVGRFISKDPIGLLGGFNVYAYTAN-PVQWVDPYGL
>KAB06_6
---MSGKTEVVKPLTEVAIKDVSHSKQK-----------------IDSWLQSHTHQIVTLDLVASVMGTVPI
IGNVMAAIDVV-SDICYIYDKGFQNADIMDWTTMGIDLIGVAP-----IPGTTAIRTSARPALFLVRQEVKR
VGKAALG---------------------EAVIVVISNHLNDQVAGDIEKFCKTGLNKLNSILATCGATVQNA
TTQINGGIVRV---VTKGQIFTNAGSNASRAQ--QQAAGVIKGIKSGDLTRAGKNLVYMTENWGKALIKTGA
NSNAKVASSLVPAHLKQPILNVGATINKIGITANTKIKGLGNTAQPQTIGWLFDVLRMSASKFRKKK--SAQ
VKPTQTTQVKHDKKNNKLEKSNDEHAATHNANAC------------KNCKGGTTHSITFALGTEFL---NHI

DAQIHPLLIEQFSRTYVSNLYQYDQSIFGARWITPFTTKIVRCSSYNVHHPKEPIE---QDGWEYIGSDGRP
VRLPELKVGQSYYDE-VETFTYTVISP---------EIRA-----ISYGVSETR--------FYQKYKNEF-
RLASIERQNGFSIG---LRYDHELENSDSYLSDIIFKQQQQILAHVALQI--------NDAQKVVAAWLVEE
G---QLIRQL-------SAYQYNEQDDLIKATNEFA-ASYEYQY-QAHLLTRYTDLTHRGMNLKWDGV----
-----------------------LPSSKAIEEWADDYSSATKLDWDENVRVTSVMDIDGNVTEYYY---GIT
GYTYRIIYPDNLQEWFFRDQAKNITSHIATDGTETSYTYDERGNVLSMVQADGSTFYYEY-DEKDNLIGFVD
AEQGRWFKEYDASGNIIKEIDPLKRETVYAYNA---MGLVTSITDAKGGKKTLKYDDRGNLISYTDCSGK-E
TKWHYDDRGRVKFIENALKQKVEYFYTELTTELRQPIAKGLPLNAFGQL-------------EKIKHADGAE
EHFIHDAEGRLLVHIDPKGQQTRYEYDAAGLITKRTDPLNQTLKYQWDRLS-RLKRLINENGASYEFVYDAA
GRLVKEIDFDGKETVYAYDEYNGRLTTSIEVASTYGQDFKDRAAPKDR----IQQFIFDSMGRLEQRTAGYG
YQGQELEEKQTEEFSYDGLGNLVQAK------------NAETNLQWFYDAAGNLIKEHHQ---DVVTQKTAV
WKHVYDELNNRTTTIRP-DGQ---------KIDWLSY--GSGHVYGMALNGEDVVSFERDDLHRETLRHYAN
G------LSQQQSYDEVGRLTQQLMLSGHD-KGYQAQTQ-----NNAIQHTNQLIERLYHYDKTGELTLI--
--------------------------------KDTRRGAIHYKYDPVGRLL------EATSKLGKETFNFDP
ASNILER------YNSTKE-----------QSSQHISDEKGYGYNRLVNNIVQEYLDQQYQYDAFGQLIRQK
SSQGDL-----NLEWDVFGRLVR-------SRNNQYTAEYRYDALGRRIQKRSKH-------HHTGQEQNIS
YGWDGDTL--AYES----SADLTKHYFYEKDS-FVPLLQAAYHHPIELHQTQDWSD---KTYSIYKDPLWNT
VKQSQ--GFDDVWFYHCDHLGTPQEMSDQTGAIIWKAEYKA-WGECKLEQTNSDFFEKSEIISN-NIRFQGQ
YFDEETGLHYNRYRYYSPYVGRFISKDPIGLLGGFNVYAYTAN-PVQWVDPYGL
>KAB07_6
---MSGKTEVVKPLTEVAIKDVSHSKQK-----------------IDSWLQSHTHQIVTLDLVASVMGTVPI
IGNVMAAIDVV-SDICYIYDKGFQNADIMDWTTMGIDLIGVAP-----IPGTTAIRTSARPALFLVRQEVKR
VGKAALG---------------------EAVIVVISNHLNDQVAGDIEKFCKTGLNKLNSILATCGATVQNA
TTQINGGIVRV---VTKGQIFTNAGSNASRAQ--QQAAGVIKGIKSGDLTRAGKNLVYMTENWGKALIKTGA
NSNAKVASSLVPAHLKQPILNVGATINKIGITANTKIKGLGNTAQPQTIGWLFDVLRMSASKFRKKK--SAQ
VKPTQTTQVKHDKKNNKLEKSNDEHAATHNANAC------------KNCKGGTTHSITFALGTEFL---NHI
DAQIHPLLIEQFSRTYVSNLYQYDQSIFGARWITPFTTKIVRCSSYNVHHPKEPIE---QDGWEYIGSDGRP
VRLPELKVGQSYYDE-VETFTYTVISP---------EIRA-----ISYGVSETR--------FYQKYKNEF-
RLASIERQNGFSIG---LRYDHELENSDSYLSDIIFKQQQQILAHVALQI--------NDAQKVVAAWLVEE
G---QLIRQL-------SAYQYNEQDDLIKATNEFA-ASYEYQY-QAHLLTRYTDLTHRGMNLKWDGV----
-----------------------LPSSKAIEEWADDYSSATKLDWDENVRVTSVMDIDGNVTEYYY---GIT
GYTYRIIYPDNLQEWFFRDQAKNITSHIATDGTETSYTYDERGNVLSMVQADGSTFYYEY-DEKDNLIGFVD
AEQGRWFKEYDASGNIIKEIDPLKRETVYAYNA---MGLVTSITDAKGGKKTLKYDDRGNLISYTDCSGK-E

TKWHYDDRGRVKFIENALKQKVEYFYTELTTELRQPIAKGLPLNAFGQL-------------EKIKHADGAE
EHFIHDAEGRLLVHIDPKGQQTRYEYDAAGLITKRTDPLNQTLKYQWDRLS-RLKRLINENGASYEFVYDAA
GRLVKEIDFDGKETVYAYDEYNGRLTTSIEVASTYGQDFKDRAAPKDR----IQQFIFDSMGRLEQRTAGYG
YQGQELEEKQTEEFSYDGLGNLVQAK------------NAETNLQWFYDAAGNLIKEHHQ---DVVTQKTAV
WKHVYDELNNRTTTIRP-DGQ---------KIDWLSY--GSGHVYGMALNGEDVVSFERDDLHRETLRHYAN
G------LSQQQSYDEVGRLTQQLMLSGHD-KGYQAQTQ-----NNAIQHTNQLIERLYHYDKTGELTLI--
--------------------------------KDTRRGAIHYKYDPVGRLL------EATSKLGKETFNFDP
ASNILER------YNSTKE-----------QSSQHISDEKGYGYNRLVNNIVQEYLDQQYQYDAFGQLIRQK
SSQGDL-----NLEWDVFGRLVR-------SRNNQYTAEYRYDALGRRIQKRSKH-------HHTGQEQNIS
YGWDGDTL--AYES----SADLTKHYFYEKDS-FVPLLQAAYHHPIELHQTQDWSD---KTYSIYKDPLWNT
VKQSQ--GFDDVWFYHCDHLGTPQEMSDQTGAIIWKAEYKA-WGECKLEQTNSDFFEKSEIISN-NIRFQGQ
YFDEETGLHYNRYRYYSPYVGRFISKDPIGLLGGFNVYAYTAN-PVQWVDPYGL
>KAB08_6
---MSGKTEVVKPLTEVAIKDVSHSKQK-----------------IDSWLQSHTHQIVTLDLVASVMGTVPI
IGNVMAAIDVV-SDICYIYDKGFQNADIMDWTTMGIDLIGVAP-----IPGTTAIRTSARPALFLVRQEVKR
VGKAALG---------------------EAVIVVISNHLNDQVAGDIEKFCKTGLNKLNSILATCGATVQNA
TTQINGGIVRV---VTKGQIFTNAGSNASRAQ--QQAAGVIKGIKSGDLTRAGKNLVYMTENWGKALIKTGA
NSNAKVASSLVPAHLKQPILNVGATINKIGITANTKIKGLGNTAQPQTIGWLFDVLRMSASKFRKKK--SAQ
VKPTQTTQVKHDKKNNKLEKSNDEHAATHNANAC------------KNCKGGTTHSITFALGTEFL---NHI
DAQIHPLLIEQFSRTYVSNLYQYDQSIFGARWITPFTTKIVRCSSYNVHHPKEPIE---QDGWEYIGSDGRP
VRLPELKVGQSYYDE-VETFTYTVISP---------EIRA-----ISYGVSETR--------FYQKYKNEF-
RLASIERQNGFSIG---LRYDHELENSDSYLSDIIFKQQQQILAHVALQI--------NDAQKVVAAWLVEE
G---QLIRQL-------SAYQYNEQDDLIKATNEFA-ASYEYQY-QAHLLTRYTDLTHRGMNLKWDGV----
-----------------------LPSSKAIEEWADDYSSATKLDWDENVRVTSVMDIDGNVTEYYY---GIT
GYTYRIIYPDNLQEWFFRDQAKNITSHIATDGTETSYTYDERGNVLSMVQADGSTFYYEY-DEKDNLIGFVD
AEQGRWFKEYDASGNIIKEIDPLKRETVYAYNA---MGLVTSITDAKGGKKTLKYDDRGNLISYTDCSGK-E
TKWHYDDRGRVKFIENALKQKVEYFYTELTTELRQPIAKGLPLNAFGQL-------------EKIKHADGAE
EHFIHDAEGRLLVHIDPKGQQTRYEYDAAGLITKRTDPLNQTLKYQWDRLS-RLKRLINENGASYEFVYDAA
GRLVKEIDFDGKETVYAYDEYNGRLTTSIEVASTYGQDFKDRAAPKDR----IQQFIFDSMGRLEQRTAGYG
YQGQELEEKQTEEFSYDGLGNLVQAK------------NAETNLQWFYDAAGNLIKEHHQ---DVVTQKTAV
WKHVYDELNNRTTTIRP-DGQ---------KIDWLSY--GSGHVYGMALNGEDVVSFERDDLHRETLRHYAN
G------LSQQQSYDEVGRLTQQLMLSGHD-KGYQAQTQ-----NNAIQHTNQLIERLYHYDKTGELTLI--
--------------------------------KDTRRGAIHYKYDPVGRLL------EATSKLGKETFNFDP

ASNILER------YNSTKE-----------QSSQHISDEKGYGYNRLVNNIVQEYLDQQYQYDAFGQLIRQK
SSQGDL-----NLEWDVFGRLVR-------SRNNQYTAEYRYDALGRRIQKRSKH-------HHTGQEQNIS
YGWDGDTL--AYES----SADLTKHYFYEKDS-FVPLLQAAYHHPIELHQTQDWSD---KTYSIYKDPLWNT
VKQSQ--GFDDVWFYHCDHLGTPQEMSDQTGAIIWKAEYKA-WGECKLEQTNSDFFEKSEIISN-NIRFQGQ
YFDEETGLHYNRYRYYSPYVGRFISKDPIGLLGGFNVYAYTAN-PVQWVDPYGL
>KBN10P02143_6
---MSGKTEVVKPLTEVAIKDVSHSKQK-----------------IDSWLQSHTHQIVTLDLVASVMGTVPI
IGNVMAAIDVV-SDICYIYDKGFQNADIMDWTTMGIDLIGVAP-----IPGTTAIRTSARPALFLVRQEVKR
VGKAALG---------------------EAVIVVISNHLNDQVAGDIEKFCKTGLNKLNSILATCGATVQNA
TTQINGGIVRV---VTKGQIFTNAGSNASRAQ--QQAAGVIKGIKSGDLTRAGKNLVYMTENWGKALIKTGA
NSNAKVASSLVPAHLKQPILNVGATINKIGITANTKIKGLGNTAQPQTIGWLFDVLRMSASKFRKKK--SAQ
VKPTQTTQVKHDKKNNKLEKSNDEHAATHNANAC------------KNCKGGTTHSITFALGTEFL---NHI
DAQIHPLLIEQFSRTYVSNLYQYDQSIFGARWITPFTTKIVRCSSYNVHHPKEPIE---QDGWEYIGSDGRP
VRLPELKVGQSYYDE-VETFTYTVISP---------EIRA-----ISYGVSETR--------FYQKYKNEF-
RLASIERQNGFSIG---LRYDHELENSDSYLSDIIFKQQQQILAHVALQI--------NDAQKVVAAWLVEE
G---QLIRQL-------SAYQYNEQDDLIKATNEFA-ASYEYQY-QAHLLTRYTDLTHRGMNLKWDGV----
-----------------------LPSSKAIEEWADDYSSATKLDWDENVRVTSVMDIDGNVTEYYY---GIT
GYTYRIIYPDNLQEWFFRDQAKNITSHIATDGTETSYTYDERGNVLSMVQADGSTFYYEY-DEKDNLIGFVD
AEQGRWFKEYDASGNIIKEIDPLKRETVYAYNA---MGLVTSITDAKGGKKTLKYDDRGNLISYTDCSGK-E
TKWHYDDRGRVKFIENALKQKVEYFYTELTTELRQPIAKGLPLNAFGQL-------------EKIKHADGAE
EHFIHDAEGRLLVHIDPKGQQTRYEYDAAGLITKRTDPLNQTLKYQWDRLS-RLKRLINENGASYEFVYDAA
GRLVKEIDFDGKETVYAYDEYNGRLTTSIEVASTYGQDFKDRAAPKDR----IQQFIFDSMGRLEQRTAGYG
YQGQELEEKQTEEFSYDGLGNLVQAK------------NAETNLQWFYDAAGNLIKEHHQ---DVVTQKTAV
WKHVYDELNNRTTTIRP-DGQ---------KIDWLSY--GSGHVYGMALNGEDVVSFERDDLHRETLRHYAN
G------LSQQQSYDEVGRLTQQLMLSGHD-KGYQAQTQ-----NNAIQHTNQLIERLYHYDKTGELTLI--
---------------------------------KDTRRGAIHYKYDPVGRLL------EATSKLGKETFNFDP
ASNILER------YNSTKE-----------QSSQHISDEKGYGYNRLVNNIVQEYLDQQYQYDAFGQLIRQK
SSQGDL-----NLEWDVFGRLVR-------SRNNQYTAEYRYDALGRRIQKRSKH-------HHTGQEQNIS
YGWDGDTL--AYES----SADLTKHYFYEKDS-FVPLLQAAYHHPIELHQTQDWSD---KTYSIYKDPLWNT
VKQSQ--GFDDVWFYHCDHLGTPQEMSDQTGAIIWKAEYKA-WGECKLEQTNSDFFEKSEIISN-NIRFQGQ
YFDEETGLHYNRYRYYSPYVGRFISKDPIGLLGGFNVYAYTAN-PVQWVDPYGL
>MDR-TJ_6
---MSGKTEVVKPLTEVAIKDVSHSKQK-----------------IDSWLQSHTHQIVTLDLVASVMGTVPI

IGNVMAAIDVV-SDICYIYDKGFQNADIMDWTTMGIDLIGVAP-----IPGTTAIRTSARPALFLVRQEVKR
VGKAALG---------------------EAVIVVISNHLNDQVAGDIEKFCKTGLNKLNSILATCGATVQNA
TTQINGGIVRV---VTKGQIFTNAGSNASRAQ--QQAAGVIKGIKSGDLTRAGKNLVYMTENWGKALIKTGA
NSNAKVASSLVPAHLKQPILNVGATINKIGITANTKIKGLGNTAQPQTIGWLFDVLRMSASKFRKKK--SAQ
VKPTQTTQVKHDKKNNKLEKSNDEHAATHNANAC------------KNCKGGTTHSITFALGTEFL---NHI
DAQIHPLLIEQFSRTYVSNLYQYDQSIFGARWITPFTTKIVRCSSYNVHHPKEPIE---QDGWEYIGSDGRP
VRLPELKVGQSYYDE-VETFTYTVISP---------EIRA-----ISYGVSETR--------FYQKYKNEF-
RLASIERQNGFSIG---LRYDHELENSDSYLSDIIFKQQQQILAHVALQI--------NDAQKVVAAWLVEE
G---QLIRQL-------SAYQYNEQDDLIKATNEFA-ASYEYQY-QAHLLTRYTDLTHRGMNLKWDGV----
-----------------------LPSSKAIEEWADDYSSATKLDWDENVRVTSVMDIDGNVTEYYY---GIT
GYTYRIIYPDNLQEWFFRDQAKNITSHIATDGTETSYTYDERGNVLSMVQADGSTFYYEY-DEKDNLIGFVD
AEQGRWFKEYDASGNIIKEIDPLKRETVYAYNA---MGLVTSITDAKGGKKTLKYDDRGNLISYTDCSGK-E
TKWHYDDRGRVKFIENALKQKVEYFYTELTTELRQPIAKGLPLNAFGQL-------------EKIKHADGAE
EHFIHDAEGRLLVHIDPKGQQTRYEYDAAGLITKRTDPLNQTLKYQWDRLS-RLKRLINENGASYEFVYDAA
GRLVKEIDFDGKETVYAYDEYNGRLTTSIEVASTYGQDFKDRAAPKDR----IQQFIFDSMGRLEQRTAGYG
YQGQELEEKQTEEFSYDGLGNLVQAK------------NAETNLQWFYDAAGNLIKEHHQ---DVVTQKTAV
WKHVYDELNNRTTTIRP-DGQ---------KIDWLSY--GSGHVYGMALNGEDVVSFERDDLHRETLRHYAN
G------LSQQQSYDEVGRLTQQLMLSGHD-KGYQAQTQ-----NNAIQHTNQLIERLYHYDKTGELTLI--
--------------------------------KDTRRGAIHYKYDPVGRLL------EATSKLGKETFNFDP
ASNILER------YNSTKE-----------QSSQHISDEKGYGYNRLVNNIVQEYLDQQYQYDAFGQLIRQK
SSQGDL-----NLEWDVFGRLVR-------SRNNQYTAEYRYDALGRRIQKRSKH-------HHTGQEQNIS
YGWDGDTL--AYES----SADLTKHYFYEKDS-FVPLLQAAYHHPIELHQTQDWSD---KTYSIYKDPLWNT
VKQSQ--GFDDVWFYHCDHLGTPQEMSDQTGAIIWKAEYKA-WGECKLEQTNSDFFEKSEIISN-NIRFQGQ
YFDEETGLHYNRYRYYSPYVGRFISKDPIGLLGGFNVYAYTAN-PVQWVDPYGL
>MDR-ZJ06_6
---MSGKTEVVKPLTEVAIKDVSHSKQK-----------------IDSWLQSHTHQIVTLDLVASVMGTVPI
IGNVMAAIDVV-SDICYIYDKGFQNADIMDWTTMGIDLIGVAP-----IPGTTAIRTSARPALFLVRQEVKR
VGKAALG---------------------EAVIVVISNHLNDQVAGDIEKFCKTGLNKLNSILATCGATVQNA
TTQINGGIVRV---VTKGQIFTNAGSNASRAQ--QQAAGVIKGIKSGDLTRAGKNLVYMTENWGKALIKTGA
NSNAKVASSLVPAHLKQPILNVGATINKIGITANTKIKGLGNTAQPQTIGWLFDVLRMSASKFRKKK--SAQ
VKPTQTTQVKHDKKNNKLEKSNDEHAATHNANAC------------KNCKGGTTHSITFALGTEFL---NHI
DAQIHPLLIEQFSRTYVSNLYQYDQSIFGARWITPFTTKIVRCSSYNVHHPKEPIE---QDGWEYIGSDGRP
VRLPELKVGQSYYDE-VETFTYTVISP---------EIRA-----ISYGVSETR--------FYQKYKNEF-

RLASIERQNGFSIG---LRYDHELENSDSYLSDIIFKQQQQILAHVALQI--------NDAQKVVAAWLVEE
G---QLIRQL-------SAYQYNEQDDLIKATNEFA-ASYEYQY-QAHLLTRYTDLTHRGMNLKWDGV----
-----------------------LPSSKAIEEWADDYSSATKLDWDENVRVTSVMDIDGNVTEYYY---GIT
GYTYRIIYPDNLQEWFFRDQAKNITSHIATDGTETSYTYDERGNVLSMVQADGSTFYYEY-DEKDNLIGFVD
AEQGRWFKEYDASGNIIKEIDPLKRETVYAYNA---MGLVTSITDAKGGKKTLKYDDRGNLISYTDCSGK-E
TKWHYDDRGRVKFIENALKQKVEYFYTELTTELRQPIAKGLPLNAFGQL-------------EKIKHADGAE
EHFIHDAEGRLLVHIDPKGQQTRYEYDAAGLITKRTDPLNQTLKYQWDRLS-RLKRLINENGASYEFVYDAA
GRLVKEIDFDGKETVYAYDEYNGRLTTSIEVASTYGQDFKDRAAPKDR----IQQFIFDSMGRLEQRTAGYG
YQGQELEEKQTEEFSYDGLGNLVQAK------------NAETNLQWFYDAAGNLIKEHHQ---DVVTQKTAV
WKHVYDELNNRTTTIRP-DGQ---------KIDWLSY--GSGHVYGMALNGEDVVSFERDDLHRETLRHYAN
G------LSQQQSYDEVGRLTQQLMLSGHD-KGYQAQTQ-----NNAIQHTNQLIERLYHYDKTGELTLI--
--------------------------------KDTRRGAIHYKYDPVGRLL------EATSKLGKETFNFDP
ASNILER------YNSTKE-----------QSSQHISDEKGYGYNRLVNNIVQEYLDQQYQYDAFGQLIRQK
SSQGDL-----NLEWDVFGRLVR-------SRNNQYTAEYRYDALGRRIQKRSKH-------HHTGQEQNIS
YGWDGDTL--AYES----SADLTKHYFYEKDS-FVPLLQAAYHHPIELHQTQDWSD---KTYSIYKDPLWNT
VKQSQ--GFDDVWFYHCDHLGTPQEMSDQTGAIIWKAEYKA-WGECKLEQTNSDFFEKSEIISN-NIRFQGQ
YFDEETGLHYNRYRYYSPYVGRFISKDPIGLLGGFNVYAYTAN-PVQWVDPYGL
>NCGM237_6
---MSGKTEVVKPLTEVAIKDVSHSKQK-----------------IDSWLQSHTHQIVTLDLVASVMGTVPI
IGNVMAAIDVV-SDICYIYDKGFQNADIMDWTTMGIDLIGVAP-----IPGTTAIRTSARPALFLVRQEVKR
VGKAALG---------------------EAVIVVISNHLNDQVAGDIEKFCKTGLNKLNSILATCGATVQNA
TTQINGGIVRV---VTKGQIFTNAGSNASRAQ--QQAAGVIKGIKSGDLTRAGKNLVYMTENWGKALIKTGA
NSNAKVASSLVPAHLKQPILNVGATINKIGITANTKIKGLGNTAQPQTIGWLFDVLRMSASKFRKKK--SAQ
VKPTQTTQVKHDKKNNKLEKSNDEHAATHNANAC------------KNCKGGTTHSITFALGTEFL---NHI
DAQIHPLLIEQFSRTYVSNLYQYDQSIFGARWITPFTTKIVRCSSYNVHHPKEPIE---QDGWEYIGSDGRP
VRLPELKVGQSYYDE-VETFTYTVISP---------EIRA-----ISYGVSETR--------FYQKYKNEF-
RLASIERQNGFSIG---LRYDHELENSDSYLSDIIFKQQQQILAHVALQI--------NDAQKVVAAWLVEE
G---QLIRQL-------SAYQYNEQDDLIKATNEFA-ASYEYQY-QAHLLTRYTDLTHRGMNLKWDGV----
-----------------------LPSSKAIEEWADDYSSATKLDWDENVRVTSVMDIDGNVTEYYY---GIT
GYTYRIIYPDNLQEWFFRDQAKNITSHIATDGTETSYTYDERGNVLSMVQADGSTFYYEY-DEKDNLIGFVD
AEQGRWFKEYDASGNIIKEIDPLKRETVYAYNA---MGLVTSITDAKGGKKTLKYDDRGNLISYTDCSGK-E
TKWHYDDRGRVKFIENALKQKVEYFYTELTTELRQPIAKGLPLNAFGQL-------------EKIKHADGAE
EHFIHDAEGRLLVHIDPKGQQTRYEYDAAGLITKRTDPLNQTLKYQWDRLS-RLKRLINENGASYEFVYDAA

GRLVKEIDFDGKETVYAYDEYNGRLTTSIEVASTYGQDFKDRAAPKDR----IQQFIFDSMGRLEQRTAGYG
YQGQELEEKQTEEFSYDGLGNLVQAK------------NAETNLQWFYDAAGNLIKEHHQ---DVVTQKTAV
WKHVYDELNNRTTTIRP-DGQ---------KIDWLSY--GSGHVYGMALNGEDVVSFERDDLHRETLRHYAN
G------LSQQQSYDEVGRLTQQLMLSGHD-KGYQAQTQ-----NNAIQHTNQLIERLYHYDKTGELTLI--
--------------------------------KDTRRGAIHYKYDPVGRLL------EATSKLGKETFNFDP
ASNILER------YNSTKE-----------QSSQHISDEKGYGYNRLVNNIVQEYLDQQYQYDAFGQLIRQK
SSQGDL-----NLEWDVFGRLVR-------SRNNQYTAEYRYDALGRRIQKRSKH-------HHTGQEQNIS
YGWDGDTL--AYES----SADLTKHYFYEKDS-FVPLLQAAYHHPIELHQTQDWSD---KTYSIYKDPLWNT
VKQSQ--GFDDVWFYHCDHLGTPQEMSDQTGAIIWKAEYKA-WGECKLEQTNSDFFEKSEIISN-NIRFQGQ
YFDEETGLHYNRYRYYSPYVGRFISKDPIGLLGGFNVYAYTAN-PVQWVDPYGL
>SAA14_6
---MSGKTEVVKPLTEVAIKDVSHSKQK-----------------IDSWLQSHTHQIVTLDLVASVMGTVPI
IGNVMAAIDVV-SDICYIYDKGFQNADIMDWTTMGIDLIGVAP-----IPGTTAIRTSARPALFLVRQEVKR
VGKAALG---------------------EAVIVVISNHLNDQVAGDIEKFCKTGLNKLNSILATCGATVQNA
TTQINGGIVRV---VTKGQIFTNAGSNASRAQ--QQAAGVIKGIKSGDLTRAGKNLVYMTENWGKALIKTGA
NSNAKVASSLVPAHLKQPILNVGATINKIGITANTKIKGLGNTAQPQTIGWLFDVLRMSASKFRKKK--SAQ
VKPTQTTQVKHDKKNNKLEKSNDEHAATHNANAC------------KNCKGGTTHSITFALGTEFL---NHI
DAQIHPLLIEQFSRTYVSNLYQYDQSIFGARWITPFTTKIVRCSSYNVHHPKEPIE---QDGWEYIGSDGRP
VRLPELKVGQSYYDE-VETFTYTVISP---------EIRA-----ISYGVSETR--------FYQKYKNEF-
RLASIERQNGFSIG---LRYDHELENSDSYLSDIIFKQQQQILAHVALQI--------NDAQKVVAAWLVEE
G---QLIRQL-------SAYQYNEQDDLIKATNEFA-ASYEYQY-QAHLLTRYTDLTHRGMNLKWDGV----
-----------------------LPSSKAIEEWADDYSSATKLDWDENVRVTSVMDIDGNVTEYYY---GIT
GYTYRIIYPDNLQEWFFRDQAKNITSHIATDGTETSYTYDERGNVLSMVQADGSTFYYEY-DEKDNLIGFVD
AEQGRWFKEYDASGNIIKEIDPLKRETVYAYNA---MGLVTSITDAKGGKKTLKYDDRGNLISYTDCSGK-E
TKWHYDDRGRVKFIENALKQKVEYFYTELTTELRQPIAKGLPLNAFGQL-------------EKIKHADGAE
EHFIHDAEGRLLVHIDPKGQQTRYEYDAAGLITKRTDPLNQTLKYQWDRLS-RLKRLINENGASYEFVYDAA
GRLVKEIDFDGKETVYAYDEYNGRLTTSIEVASTYGQDFKDRAAPKDR----IQQFIFDSMGRLEQRTAGYG
YQGQELEEKQTEEFSYDGLGNLVQAK------------NAETNLQWFYDAAGNLIKEHHQ---DVVTQKTAV
WKHVYDELNNRTTTIRP-DGQ---------KIDWLSY--GSGHVYGMALNGEDVVSFERDDLHRETLRHYAN
G------LSQQQSYDEVGRLTQQLMLSGHD-KGYQAQTQ-----NNAIQHTNQLIERLYHYDKTGELTLI--
--------------------------------KDTRRGAIHYKYDPVGRLL------EATSKLGKETFNFDP
ASNILER------YNSTKE-----------QSSQHISDEKGYGYNRLVNNIVQEYLDQQYQYDAFGQLIRQK
SSQGDL-----NLEWDVFGRLVR-------SRNNQYTAEYRYDALGRRIQKRSKH-------HHTGQEQNIS

```
YGWDGDTL--AYES----SADLTKHYFYEKDS-FVPLLQAAYHHPIELHQTQDWSD---KTYSIYKDPLWNT
VKQSQ--GFDDVWFYHCDHLGTPQEMSDQTGAIIWKAEYKA-WGECKLEQTNSDFFEKSEIISN-NIRFQGQ
YFDEETGLHYNRYRYYSPYVGRFISKDPIGLLGGFNVYAYTAN-PVQWVDPYGL
>SMC_Paed_Ab_BL01_6
---MSGKTEVVKPLTEVAIKDVSHSKQK-----------------IDSWLQSHTHQIVTLDLVASVMGTVPI
IGNVMAAIDVV-SDICYIYDKGFQNADIMDWTTMGIDLIGVAP-----IPGTTAIRTSARPALFLVRQEVKR
VGKAALG---------------------EAVIVVISNHLNDQVAGDIEKFCKTGLNKLNSILATCGATVQNA
TTQINGGIVRV---VTKGQIFTNAGSNASRAQ--QQAAGVIKGIKSGDLTRAGKNLVYMTENWGKALIKTGA
NSNAKVASSLVPAHLKQPILNVGATINKIGITANTKIKGLGNTAQPQTIGWLFDVLRMSASKFRKKK--SAQ
VKPTQTTQVKHDKKNNKLEKSNDEHAATHNANAC------------KNCKGGTTHSITFALGTEFL---NHI
DAQIHPLLIEQFSRTYVSNLYQYDQSIFGARWITPFTTKIVRCSSYNVHHPKEPIE---QDGWEYIGSDGRP
VRLPELKVGQSYYDE-VETFTYTVISP---------EIRA-----ISYGVSETR--------FYQKYKNEF-
RLASIERQNGFSIG---LRYDHELENSDSYLSDIIFKQQQQILAHVALQI--------NDAQKVVAAWLVEE
G---QLIRQL-------SAYQYNEQDDLIKATNEFA-ASYEYQY-QAHLLTRYTDLTHRGMNLKWDGV----
-----------------------LPSSKAIEEWADDYSSATKLDWDENVRVTSVMDIDGNVTEYYY---GIT
GYTYRIIYPDNLQEWFFRDQAKNITSHIATDGTETSYTYDERGNVLSMVQADGSTFYYEY-DEKDNLIGFVD
AEQGRWFKEYDASGNIIKEIDPLKRETVYAYNA---MGLVTSITDAKGGKKTLKYDDRGNLISYTDCSGK-E
TKWHYDDRGRVKFIENALKQKVEYFYTELTTELRQPIAKGLPLNAFGQL-------------EKIKHADGAE
EHFIHDAEGRLLVHIDPKGQQTRYEYDAAGLITKRTDPLNQTLKYQWDRLS-RLKRLINENGASYEFVYDAA
GRLVKEIDFDGKETVYAYDEYNGRLTTSIEVASTYGQDFKDRAAPKDR----IQQFIFDSMGRLEQRTAGYG
YQGQELEEKQTEEFSYDGLGNLVQAK------------NAETNLQWFYDAAGNLIKEHHQ---DVVTQKTAV
WKHVYDELNNRTTTIRP-DGQ---------KIDWLSY--GSGHVYGMALNGEDVVSFERDDLHRETLRHYAN
G------LSQQQSYDEVGRLTQQLMLSGHD-KGYQAQTQ-----NNAIQHTNQLIERLYHYDKTGELTLI--
--------------------------------KDTRRGAIHYKYDPVGRLL------EATSKLGKETFNFDP
ASNILER------YNSTKE-----------QSSQHISDEKGYGYNRLVNNIVQEYLDQQYQYDAFGQLIRQK
SSQGDL-----NLEWDVFGRLVR-------SRNNQYTAEYRYDALGRRIQKRSKH-------HHTGQEQNIS
YGWDGDTL--AYES----SADLTKHYFYEKDS-FVPLLQAAYHHPIELHQTQDWSD---KTYSIYKDPLWNT
VKQSQ--GFDDVWFYHCDHLGTPQEMSDQTGAIIWKAEYKA-WGECKLEQTNSDFFEKSEIISN-NIRFQGQ
YFDEETGLHYNRYRYYSPYVGRFISKDPIGLLGGFNVYAYTAN-PVQWVDPYGL
>SSA12_6
---MSGKTEVVKPLTEVAIKDVSHSKQK-----------------IDSWLQSHTHQIVTLDLVASVMGTVPI
IGNVMAAIDVV-SDICYIYDKGFQNADIMDWTTMGIDLIGVAP-----IPGTTAIRTSARPALFLVRQEVKR
VGKAALG---------------------EAVIVVISNHLNDQVAGDIEKFCKTGLNKLNSILATCGATVQNA
```

```
TTQINGGIVRV---VTKGQIFTNAGSNASRAQ--QQAAGVIKGIKSGDLTRAGKNLVYMTENWGKALIKTGA
NSNAKVASSLVPAHLKQPILNVGATINKIGITANTKIKGLGNTAQPQTIGWLFDVLRMSASKFRKKK--SAQ
VKPTQTTQVKHDKKNNKLEKSNDEHAATHNANAC------------KNCKGGTTHSITFALGTEFL---NHI
DAQIHPLLIEQFSRTYVSNLYQYDQSIFGARWITPFTTKIVRCSSYNVHHPKEPIE---QDGWEYIGSDGRP
VRLPELKVGQSYYDE-VETFTYTVISP---------EIRA-----ISYGVSETR--------FYQKYKNEF-
RLASIERQNGFSIG---LRYDHELENSDSYLSDIIFKQQQQILAHVALQI--------NDAQKVVAAWLVEE
G---QLIRQL-------SAYQYNEQDDLIKATNEFA-ASYEYQY-QAHLLTRYTDLTHRGMNLKWDGV----
-----------------------LPSSKAIEEWADDYSSATKLDWDENVRVTSVMDIDGNVTEYYY---GIT
GYTYRIIYPDNLQEWFFRDQAKNITSHIATDGTETSYTYDERGNVLSMVQADGSTFYYEY-DEKDNLIGFVD
AEQGRWFKEYDASGNIIKEIDPLKRETVYAYNA---MGLVTSITDAKGGKKTLKYDDRGNLISYTDCSGK-E
TKWHYDDRGRVKFIENALKQKVEYFYTELTTELRQPIAKGLPLNAFGQL-------------EKIKHADGAE
EHFIHDAEGRLLVHIDPKGQQTRYEYDAAGLITKRTDPLNQTLKYQWDRLS-RLKRLINENGASYEFVYDAA
GRLVKEIDFDGKETVYAYDEYNGRLTTSIEVASTYGQDFKDRAAPKDR----IQQFIFDSMGRLEQRTAGYG
YQGQELEEKQTEEFSYDGLGNLVQAK------------NAETNLQWFYDAAGNLIKEHHQ---DVVTQKTAV
WKHVYDELNNRTTTIRP-DGQ---------KIDWLSY--GSGHVYGMALNGEDVVSFERDDLHRETLRHYAN
G------LSQQQSYDEVGRLTQQLMLSGHD-KGYQAQTQ-----NNAIQHTNQLIERLYHYDKTGELTLI--
--------------------------------KDTRRGAIHYKYDPVGRLL------EATSKLGKETFNFDP
ASNILER------YNSTKE-----------QSSQHISDEKGYGYNRLVNNIVQEYLDQQYQYDAFGQLIRQK
SSQGDL-----NLEWDVFGRLVR-------SRNNQYTAEYRYDALGRRIQKRSKH-------HHTGQEQNIS
YGWDGDTL--AYES----SADLTKHYFYEKDS-FVPLLQAAYHHPIELHQTQDWSD---KTYSIYKDPLWNT
VKQSQ--GFDDVWFYHCDHLGTPQEMSDQTGAIIWKAEYKA-WGECKLEQTNSDFFEKSEIISN-NIRFQGQ
YFDEETGLHYNRYRYYSPYVGRFISKDPIGLLGGFNVYAYTAN-PVQWVDPYGL
>SSA6_6
---MSGKTEVVKPLTEVAIKDVSHSKQK-----------------IDSWLQSHTHQIVTLDLVASVMGTVPI
IGNVMAAIDVV-SDICYIYDKGFQNADIMDWTTMGIDLIGVAP-----IPGTTAIRTSARPALFLVRQEVKR
VGKAALG---------------------EAVIVVISNHLNDQVAGDIEKFCKTGLNKLNSILATCGATVQNA
TTQINGGIVRV---VTKGQIFTNAGSNASRAQ--QQAAGVIKGIKSGDLTRAGKNLVYMTENWGKALIKTGA
NSNAKVASSLVPAHLKQPILNVGATINKIGITANTKIKGLGNTAQPQTIGWLFDVLRMSASKFRKKK--SAQ
VKPTQTTQVKHDKKNNKLEKSNDEHAATHNANAC------------KNCKGGTTHSITFALGTEFL---NHI
DAQIHPLLIEQFSRTYVSNLYQYDQSIFGARWITPFTTKIVRCSSYNVHHPKEPIE---QDGWEYIGSDGRP
VRLPELKVGQSYYDE-VETFTYTVISP---------EIRA-----ISYGVSETR--------FYQKYKNEF-
RLASIERQNGFSIG---LRYDHELENSDSYLSDIIFKQQQQILAHVALQI--------NDAQKVVAAWLVEE
G---QLIRQL-------SAYQYNEQDDLIKATNEFA-ASYEYQY-QAHLLTRYTDLTHRGMNLKWDGV----
```

-----------------------LPSSKAIEEWADDYSSATKLDWDENVRVTSVMDIDGNVTEYYY---GIT
GYTYRIIYPDNLQEWFFRDQAKNITSHIATDGTETSYTYDERGNVLSMVQADGSTFYYEY-DEKDNLIGFVD
AEQGRWFKEYDASGNIIKEIDPLKRETVYAYNA---MGLVTSITDAKGGKKTLKYDDRGNLISYTDCSGK-E
TKWHYDDRGRVKFIENALKQKVEYFYTELTTELRQPIAKGLPLNAFGQL-------------EKIKHADGAE
EHFIHDAEGRLLVHIDPKGQQTRYEYDAAGLITKRTDPLNQTLKYQWDRLS-RLKRLINENGASYEFVYDAA
GRLVKEIDFDGKETVYAYDEYNGRLTTSIEVASTYGQDFKDRAAPKDR----IQQFIFDSMGRLEQRTAGYG
YQGQELEEKQTEEFSYDGLGNLVQAK------------NAETNLQWFYDAAGNLIKEHHQ---DVVTQKTAV
WKHVYDELNNRTTTIRP-DGQ---------KIDWLSY--GSGHVYGMALNGEDVVSFERDDLHRETLRHYAN
G------LSQQQSYDEVGRLTQQLMLSGHD-KGYQAQTQ-----NNAIQHTNQLIERLYHYDKTGELTLI--
--------------------------------KDTRRGAIHYKYDPVGRLL------EATSKLGKETFNFDP
ASNILER------YNSTKE-----------QSSQHISDEKGYGYNRLVNNIVQEYLDQQYQYDAFGQLIRQK
SSQGDL-----NLEWDVFGRLVR-------SRNNQYTAEYRYDALGRRIQKRSKH-------HHTGQEQNIS
YGWDGDTL--AYES----SADLTKHYFYEKDS-FVPLLQAAYHHPIELHQTQDWSD---KTYSIYKDPLWNT
VKQSQ--GFDDVWFYHCDHLGTPQEMSDQTGAIIWKAEYKA-WGECKLEQTNSDFFEKSEIISN-NIRFQGQ
YFDEETGLHYNRYRYYSPYVGRFISKDPIGLLGGFNVYAYTAN-PVQWVDPYGL
>TCDC-0715_6
---MSGKTEVVKPLTEVAIKDVSHSKQK-----------------IDSWLQSHTHQIVTLDLVASVMGTVPI
IGNVMAAIDVV-SDICYIYDKGFQNADIMDWTTMGIDLIGVAP-----IPGTTAIRTSARPALFLVRQEVKR
VGKAALG---------------------EAVIVVISNHLNDQVAGDIEKFCKTGLNKLNSILATCGATVQNA
TTQINGGIVRV---VTKGQIFTNAGSNASRAQ--QQAAGVIKGIKSGDLTRAGKNLVYMTENWGKALIKTGA
NSNAKVASSLVPAHLKQPILNVGATINKIGITANTKIKGLGNTAQPQTIGWLFDVLRMSASKFRKKK--SAQ
VKPTQTTQVKHDKKNNKLEKSNDEHAATHNANAC------------KNCKGGTTHSITFALGTEFL---NHI
DAQIHPLLIEQFSRTYVSNLYQYDQSIFGARWITPFTTKIVRCSSYNVHHPKEPIE---QDGWEYIGSDGRP
VRLPELKVGQSYYDE-VETFTYTVISP---------EIRA-----ISYGVSETR--------FYQKYKNEF-
RLASIERQNGFSIG---LRYDHELENSDSYLSDIIFKQQQQILAHVALQI--------NDAQKVVAAWLVEE
G---QLIRQL-------SAYQYNEQDDLIKATNEFA-ASYEYQY-QAHLLTRYTDLTHRGMNLKWDGV----
-----------------------LPSSKAIEEWADDYSSATKLDWDENVRVTSVMDIDGNVTEYYY---GIT
GYTYRIIYPDNLQEWFFRDQAKNITSHIATDGTETSYTYDERGNVLSMVQADGSTFYYEY-DEKDNLIGFVD
AEQGRWFKEYDASGNIIKEIDPLKRETVYAYNA---MGLVTSITDAKGGKKTLKYDDRGNLISYTDCSGK-E
TKWHYDDRGRVKFIENALKQKVEYFYTELTTELRQPIAKGLPLNAFGQL-------------EKIKHADGAE
EHFIHDAEGRLLVHIDPKGQQTRYEYDAAGLITKRTDPLNQTLKYQWDRLS-RLKRLINENGASYEFVYDAA
GRLVKEIDFDGKETVYAYDEYNGRLTTSIEVASTYGQDFKDRAAPKDR----IQQFIFDSMGRLEQRTAGYG
YQGQELEEKQTEEFSYDGLGNLVQAK------------NAETNLQWFYDAAGNLIKEHHQ---DVVTQKTAV

WKHVYDELNNRTTTIRP-DGQ---------KIDWLSY--GSGHVYGMALNGEDVVSFERDDLHRETLRHYAN
G------LSQQQSYDEVGRLTQQLMLSGHD-KGYQAQTQ-----NNAIQHTNQLIERLYHYDKTGELTLI--
--------------------------------KDTRRGAIHYKYDPVGRLL------EATSKLGKETFNFDP
ASNILER------YNSTKE-----------QSSQHISDEKGYGYNRLVNNIVQEYLDQQYQYDAFGQLIRQK
SSQGDL-----NLEWDVFGRLVR-------SRNNQYTAEYRYDALGRRIQKRSKH-------HHTGQEQNIS
YGWDGDTL--AYES----SADLTKHYFYEKDS-FVPLLQAAYHHPIELHQTQDWSD---KTYSIYKDPLWNT
VKQSQ--GFDDVWFYHCDHLGTPQEMSDQTGAIIWKAEYKA-WGECKLEQTNSDFFEKSEIISN-NIRFQGQ
YFDEETGLHYNRYRYYSPYVGRFISKDPIGLLGGFNVYAYTAN-PVQWVDPYGL
>TYTH-1_6
---MSGKTEVVKPLTEVAIKDVSHSKQK-----------------IDSWLQSHTHQIVTLDLVASVMGTVPI
IGNVMAAIDVV-SDICYIYDKGFQNADIMDWTTMGIDLIGVAP-----IPGTTAIRTSARPALFLVRQEVKR
VGKAALG---------------------EAVIVVISNHLNDQVAGDIEKFCKTGLNKLNSILATCGATVQNA
TTQINGGIVRV---VTKGQIFTNAGSNASRAQ--QQAAGVIKGIKSGDLTRAGKNLVYMTENWGKALIKTGA
NSNAKVASSLVPAHLKQPILNVGATINKIGITANTKIKGLGNTAQPQTIGWLFDVLRMSASKFRKKK--SAQ
VKPTQTTQVKHDKKNNKLEKSNDEHAATHNANAC------------KNCKGGTTHSITFALGTEFL---NHI
DAQIHPLLIEQFSRTYVSNLYQYDQSIFGARWITPFTTKIVRCSSYNVHHPKEPIE---QDGWEYIGSDGRP
VRLPELKVGQSYYDE-VETFTYTVISP---------EIRA-----ISYGVSETR--------FYQKYKNEF-
RLASIERQNGFSIG---LRYDHELENSDSYLSDIIFKQQQQILAHVALQI--------NDAQKVVAAWLVEE
G---QLIRQL-------SAYQYNEQDDLIKATNEFA-ASYEYQY-QAHLLTRYTDLTHRGMNLKWDGV----
-----------------------LPSSKAIEEWADDYSSATKLDWDENVRVTSVMDIDGNVTEYYY---GIT
GYTYRIIYPDNLQEWFFRDQAKNITSHIATDGTETSYTYDERGNVLSMVQADGSTFYYEY-DEKDNLIGFVD
AEQGRWFKEYDASGNIIKEIDPLKRETVYAYNA---MGLVTSITDAKGGKKTLKYDDRGNLISYTDCSGK-E
TKWHYDDRGRVKFIENALKQKVEYFYTELTTELRQPIAKGLPLNAFGQL-------------EKIKHADGAE
EHFIHDAEGRLLVHIDPKGQQTRYEYDAAGLITKRTDPLNQTLKYQWDRLS-RLKRLINENGASYEFVYDAA
GRLVKEIDFDGKETVYAYDEYNGRLTTSIEVASTYGQDFKDRAAPKDR----IQQFIFDSMGRLEQRTAGYG
YQGQELEEKQTEEFSYDGLGNLVQAK------------NAETNLQWFYDAAGNLIKEHHQ---DVVTQKTAV
WKHVYDELNNRTTTIRP-DGQ---------KIDWLSY--GSGHVYGMALNGEDVVSFERDDLHRETLRHYAN
G------LSQQQSYDEVGRLTQQLMLSGHD-KGYQAQTQ-----NNAIQHTNQLIERLYHYDKTGELTLI--
--------------------------------KDTRRGAIHYKYDPVGRLL------EATSKLGKETFNFDP
ASNILER------YNSTKE-----------QSSQHISDEKGYGYNRLVNNIVQEYLDQQYQYDAFGQLIRQK
SSQGDL-----NLEWDVFGRLVR-------SRNNQYTAEYRYDALGRRIQKRSKH-------HHTGQEQNIS
YGWDGDTL--AYES----SADLTKHYFYEKDS-FVPLLQAAYHHPIELHQTQDWSD---KTYSIYKDPLWNT
VKQSQ--GFDDVWFYHCDHLGTPQEMSDQTGAIIWKAEYKA-WGECKLEQTNSDFFEKSEIISN-NIRFQGQ

YFDEETGLHYNRYRYYSPYVGRFISKDPIGLLGGFNVYAYTAN-PVQWVDPYGL
>USA2_6
---MSGKTEVVKPLTEVAIKDVSHSKQK-----------------IDSWLQSHTHQIVTLDLVASVMGTVPI
IGNVMAAIDVV-SDICYIYDKGFQNADIMDWTTMGIDLIGVAP-----IPGTTAIRTSARPALFLVRQEVKR
VGKAALG---------------------EAVIVVISNHLNDQVAGDIEKFCKTGLNKLNSILATCGATVQNA
TTQINGGIVRV---VTKGQIFTNAGSNASRAQ--QQAAGVIKGIKSGDLTRAGKNLVYMTENWGKALIKTGA
NSNAKVASSLVPAHLKQPILNVGATINKIGITANTKIKGLGNTAQPQTIGWLFDVLRMSASKFRKKK--SAQ
VKPTQTTQVKHDKKNNKLEKSNDEHAATHNANAC------------KNCKGGTTHSITFALGTEFL---NHI
DAQIHPLLIEQFSRTYVSNLYQYDQSIFGARWITPFTTKIVRCSSYNVHHPKEPIE---QDGWEYIGSDGRP
VRLPELKVGQSYYDE-VETFTYTVISP---------EIRA-----ISYGVSETR--------FYQKYKNEF-
RLASIERQNGFSIG---LRYDHELENSDSYLSDIIFKQQQQILAHVALQI--------NDAQKVVAAWLVEE
G---QLIRQL-------SAYQYNEQDDLIKATNEFA-ASYEYQY-QAHLLTRYTDLTHRGMNLKWDGV----
-----------------------LPSSKAIEEWADDYSSATKLDWDENVRVTSVMDIDGNVTEYYY---GIT
GYTYRIIYPDNLQEWFFRDQAKNITSHIATDGTETSYTYDERGNVLSMVQADGSTFYYEY-DEKDNLIGFVD
AEQGRWFKEYDASGNIIKEIDPLKRETVYAYNA---MGLVTSITDAKGGKKTLKYDDRGNLISYTDCSGK-E
TKWHYDDRGRVKFIENALKQKVEYFYTELTTELRQPIAKGLPLNAFGQL-------------EKIKHADGAE
EHFIHDAEGRLLVHIDPKGQQTRYEYDAAGLITKRTDPLNQTLKYQWDRLS-RLKRLINENGASYEFVYDAA
GRLVKEIDFDGKETVYAYDEYNGRLTTSIEVASTYGQDFKDRAAPKDR----IQQFIFDSMGRLEQRTAGYG
YQGQELEEKQTEEFSYDGLGNLVQAK------------NAETNLQWFYDAAGNLIKEHHQ---DVVTQKTAV
WKHVYDELNNRTTTIRP-DGQ---------KIDWLSY--GSGHVYGMALNGEDVVSFERDDLHRETLRHYAN
G------LSQQQSYDEVGRLTQQLMLSGHD-KGYQAQTQ-----NNAIQHTNQLIERLYHYDKTGELTLI--
--------------------------------KDTRRGAIHYKYDPVGRLL------EATSKLGKETFNFDP
ASNILER------YNSTKE-----------QSSQHISDEKGYGYNRLVNNIVQEYLDQQYQYDAFGQLIRQK
SSQGDL-----NLEWDVFGRLVR-------SRNNQYTAEYRYDALGRRIQKRSKH-------HHTGQEQNIS
YGWDGDTL--AYES----SADLTKHYFYEKDS-FVPLLQAAYHHPIELHQTQDWSD---KTYSIYKDPLWNT
VKQSQ--GFDDVWFYHCDHLGTPQEMSDQTGAIIWKAEYKA-WGECKLEQTNSDFFEKSEIISN-NIRFQGQ
YFDEETGLHYNRYRYYSPYVGRFISKDPIGLLGGFNVYAYTAN-PVQWVDPYGL
>WCHAB005133_6
---MSGKTEVVKPLTEVAIKDVSHSKQK-----------------IDSWLQSHTHQIVTLDLVASVMGTVPI
IGNVMAAIDVV-SDICYIYDKGFQNADIMDWTTMGIDLIGVAP-----IPGTTAIRTSARPALFLVRQEVKR
VGKAALG---------------------EAVIVVISNHLNDQVAGDIEKFCKTGLNKLNSILATCGATVQNA
TTQINGGIVRV---VTKGQIFTNAGSNASRAQ--QQAAGVIKGIKSGDLTRAGKNLVYMTENWGKALIKTGA
NSNAKVASSLVPAHLKQPILNVGATINKIGITANTKIKGLGNTAQPQTIGWLFDVLRMSASKFRKKK--SAQ

VKPTQTTQVKHDKKNNKLEKSNDEHAATHNANAC------------KNCKGGTTHSITFALGTEFL---NHI
DAQIHPLLIEQFSRTYVSNLYQYDQSIFGARWITPFTTKIVRCSSYNVHHPKEPIE---QDGWEYIGSDGRP
VRLPELKVGQSYYDE-VETFTYTVISP---------EIRA-----ISYGVSETR--------FYQKYKNEF-
RLASIERQNGFSIG---LRYDHELENSDSYLSDIIFKQQQQILAHVALQI--------NDAQKVVAAWLVEE
G---QLIRQL-------SAYQYNEQDDLIKATNEFA-ASYEYQY-QAHLLTRYTDLTHRGMNLKWDGV----
-----------------------LPSSKAIEEWADDYSSATKLDWDENVRVTSVMDIDGNVTEYYY---GIT
GYTYRIIYPDNLQEWFFRDQAKNITSHIATDGTETSYTYDERGNVLSMVQADGSTFYYEY-DEKDNLIGFVD
AEQGRWFKEYDASGNIIKEIDPLKRETVYAYNA---MGLVTSITDAKGGKKTLKYDDRGNLISYTDCSGK-E
TKWHYDDRGRVKFIENALKQKVEYFYTELTTELRQPIAKGLPLNAFGQL-------------EKIKHADGAE
EHFIHDAEGRLLVHIDPKGQQTRYEYDAAGLITKRTDPLNQTLKYQWDRLS-RLKRLINENGASYEFVYDAA
GRLVKEIDFDGKETVYAYDEYNGRLTTSIEVASTYGQDFKDRAAPKDR----IQQFIFDSMGRLEQRTAGYG
YQGQELEEKQTEEFSYDGLGNLVQAK------------NAETNLQWFYDAAGNLIKEHHQ---DVVTQKTAV
WKHVYDELNNRTTTIRP-DGQ---------KIDWLSY--GSGHVYGMALNGEDVVSFERDDLHRETLRHYAN
G------LSQQQSYDEVGRLTQQLMLSGHD-KGYQAQTQ-----NNAIQHTNQLIERLYHYDKTGELTLI--
--------------------------------KDTRRGAIHYKYDPVGRLL------EATSKLGKETFNFDP
ASNILER------YNSTKE-----------QSSQHISDEKGYGYNRLVNNIVQEYLDQQYQYDAFGQLIRQK
SSQGDL-----NLEWDVFGRLVR-------SRNNQYTAEYRYDALGRRIQKRSKH-------HHTGQEQNIS
YGWDGDTL--AYES----SADLTKHYFYEKDS-FVPLLQAAYHHPIELHQTQDWSD---KTYSIYKDPLWNT
VKQSQ--GFDDVWFYHCDHLGTPQEMSDQTGAIIWKAEYKA-WGECKLEQTNSDFFEKSEIISN-NIRFQGQ
YFDEETGLHYNRYRYYSPYVGRFISKDPIGLLGGFNVYAYTAN-PVQWVDPYGL
>XDR-BJ83_6
---MSGKTEVVKPLTEVAIKDVSHSKQK-----------------IDSWLQSHTHQIVTLDLVASVMGTVPI
IGNVMAAIDVV-SDICYIYDKGFQNADIMDWTTMGIDLIGVAP-----IPGTTAIRTSARPALFLVRQEVKR
VGKAALG---------------------EAVIVVISNHLNDQVAGDIEKFCKTGLNKLNSILATCGATVQNA
TTQINGGIVRV---VTKGQIFTNAGSNASRAQ--QQAAGVIKGIKSGDLTRAGKNLVYMTENWGKALIKTGA
NSNAKVASSLVPAHLKQPILNVGATINKIGITANTKIKGLGNTAQPQTIGWLFDVLRMSASKFRKKK--SAQ
VKPTQTTQVKHDKKNNKLEKSNDEHAATHNANAC------------KNCKGGTTHSITFALGTEFL---NHI
DAQIHPLLIEQFSRTYVSNLYQYDQSIFGARWITPFTTKIVRCSSYNVHHPKEPIE---QDGWEYIGSDGRP
VRLPELKVGQSYYDE-VETFTYTVISP---------EIRA-----ISYGVSETR--------FYQKYKNEF-
RLASIERQNGFSIG---LRYDHELENSDSYLSDIIFKQQQQILAHVALQI--------NDAQKVVAAWLVEE
G---QLIRQL-------SAYQYNEQDDLIKATNEFA-ASYEYQY-QAHLLTRYTDLTHRGMNLKWDGV----
-----------------------LPSSKAIEEWADDYSSATKLDWDENVRVTSVMDIDGNVTEYYY---GIT
GYTYRIIYPDNLQEWFFRDQAKNITSHIATDGTETSYTYDERGNVLSMVQADGSTFYYEY-DEKDNLIGFVD

AEQGRWFKEYDASGNIIKEIDPLKRETVYAYNA---MGLVTSITDAKGGKKTLKYDDRGNLISYTDCSGK-E
TKWHYDDRGRVKFIENALKQKVEYFYTELTTELRQPIAKGLPLNAFGQL-------------EKIKHADGAE
EHFIHDAEGRLLVHIDPKGQQTRYEYDAAGLITKRTDPLNQTLKYQWDRLS-RLKRLINENGASYEFVYDAA
GRLVKEIDFDGKETVYAYDEYNGRLTTSIEVASTYGQDFKDRAAPKDR----IQQFIFDSMGRLEQRTAGYG
YQGQELEEKQTEEFSYDGLGNLVQAK------------NAETNLQWFYDAAGNLIKEHHQ---DVVTQKTAV
WKHVYDELNNRTTTIRP-DGQ---------KIDWLSY--GSGHVYGMALNGEDVVSFERDDLHRETLRHYAN
G------LSQQQSYDEVGRLTQQLMLSGHD-KGYQAQTQ-----NNAIQHTNQLIERLYHYDKTGELTLI--
--------------------------------KDTRRGAIHYKYDPVGRLL------EATSKLGKETFNFDP
ASNILER------YNSTKE-----------QSSQHISDEKGYGYNRLVNNIVQEYLDQQYQYDAFGQLIRQK
SSQGDL-----NLEWDVFGRLVR-------SRNNQYTAEYRYDALGRRIQKRSKH-------HHTGQEQNIS
YGWDGDTL--AYES----SADLTKHYFYEKDS-FVPLLQAAYHHPIELHQTQDWSD---KTYSIYKDPLWNT
VKQSQ--GFDDVWFYHCDHLGTPQEMSDQTGAIIWKAEYKA-WGECKLEQTNSDFFEKSEIISN-NIRFQGQ
YFDEETGLHYNRYRYYSPYVGRFISKDPIGLLGGFNVYAYTAN-PVQWVDPYGL
>XH386_6
---MSGKTEVVKPLTEVAIKDVSHSKQK-----------------IDSWLQSHTHQIVTLDLVASVMGTVPI
IGNVMAAIDVV-SDICYIYDKGFQNADIMDWTTMGIDLIGVAP-----IPGTTAIRTSARPALFLVRQEVKR
VGKAALG---------------------EAVIVVISNHLNDQVAGDIEKFCKTGLNKLNSILATCGATVQNA
TTQINGGIVRV---VTKGQIFTNAGSNASRAQ--QQAAGVIKGIKSGDLTRAGKNLVYMTENWGKALIKTGA
NSNAKVASSLVPAHLKQPILNVGATINKIGITANTKIKGLGNTAQPQTIGWLFDVLRMSASKFRKKK--SAQ
VKPTQTTQVKHDKKNNKLEKSNDEHAATHNANAC------------KNCKGGTTHSITFALGTEFL---NHI
DAQIHPLLIEQFSRTYVSNLYQYDQSIFGARWITPFTTKIVRCSSYNVHHPKEPIE---QDGWEYIGSDGRP
VRLPELKVGQSYYDE-VETFTYTVISP---------EIRA-----ISYGVSETR--------FYQKYKNEF-
RLASIERQNGFSIG---LRYDHELENSDSYLSDIIFKQQQQILAHVALQI--------NDAQKVVAAWLVEE
G---QLIRQL-------SAYQYNEQDDLIKATNEFA-ASYEYQY-QAHLLTRYTDLTHRGMNLKWDGV----
-----------------------LPSSKAIEEWADDYSSATKLDWDENVRVTSVMDIDGNVTEYYY---GIT
GYTYRIIYPDNLQEWFFRDQAKNITSHIATDGTETSYTYDERGNVLSMVQADGSTFYYEY-DEKDNLIGFVD
AEQGRWFKEYDASGNIIKEIDPLKRETVYAYNA---MGLVTSITDAKGGKKTLKYDDRGNLISYTDCSGK-E
TKWHYDDRGRVKFIENALKQKVEYFYTELTTELRQPIAKGLPLNAFGQL-------------EKIKHADGAE
EHFIHDAEGRLLVHIDPKGQQTRYEYDAAGLITKRTDPLNQTLKYQWDRLS-RLKRLINENGASYEFVYDAA
GRLVKEIDFDGKETVYAYDEYNGRLTTSIEVASTYGQDFKDRAAPKDR----IQQFIFDSMGRLEQRTAGYG
YQGQELEEKQTEEFSYDGLGNLVQAK------------NAETNLQWFYDAAGNLIKEHHQ---DVVTQKTAV
WKHVYDELNNRTTTIRP-DGQ---------KIDWLSY--GSGHVYGMALNGEDVVSFERDDLHRETLRHYAN
G------LSQQQSYDEVGRLTQQLMLSGHD-KGYQAQTQ-----NNAIQHTNQLIERLYHYDKTGELTLI--

--------------------------------KDTRRGAIHYKYDPVGRLL------EATSKLGKETFNFDP
ASNILER------YNSTKE-----------QSSQHISDEKGYGYNRLVNNIVQEYLDQQYQYDAFGQLIRQK
SSQGDL-----NLEWDVFGRLVR-------SRNNQYTAEYRYDALGRRIQKRSKH-------HHTGQEQNIS
YGWDGDTL--AYES----SADLTKHYFYEKDS-FVPLLQAAYHHPIELHQTQDWSD---KTYSIYKDPLWNT
VKQSQ--GFDDVWFYHCDHLGTPQEMSDQTGAIIWKAEYKA-WGECKLEQTNSDFFEKSEIISN-NIRFQGQ
YFDEETGLHYNRYRYYSPYVGRFISKDPIGLLGGFNVYAYTAN-PVQWVDPYGL
>XH856_6
---MSGKTEVVKPLTEVAIKDVSHSKQK-----------------IDSWLQSHTHQIVTLDLVASVMGTVPI
IGNVMAAIDVV-SDICYIYDKGFQNADIMDWTTMGIDLIGVAP-----IPGTTAIRTSARPALFLVRQEVKR
VGKAALG---------------------EAVIVVISNHLNDQVAGDIEKFCKTGLNKLNSILATCGATVQNA
TTQINGGIVRV---VTKGQIFTNAGSNASRAQ--QQAAGVIKGIKSGDLTRAGKNLVYMTENWGKALIKTGA
NSNAKVASSLVPAHLKQPILNVGATINKIGITANTKIKGLGNTAQPQTIGWLFDVLRMSASKFRKKK--SAQ
VKPTQTTQVKHDKKNNKLEKSNDEHAATHNANAC------------KNCKGGTTHSITFALGTEFL---NHI
DAQIHPLLIEQFSRTYVSNLYQYDQSIFGARWITPFTTKIVRCSSYNVHHPKEPIE---QDGWEYIGSDGRP
VRLPELKVGQSYYDE-VETFTYTVISP---------EIRA-----ISYGVSETR--------FYQKYKNEF-
RLASIERQNGFSIG---LRYDHELENSDSYLSDIIFKQQQQILAHVALQI--------NDAQKVVAAWLVEE
G---QLIRQL-------SAYQYNEQDDLIKATNEFA-ASYEYQY-QAHLLTRYTDLTHRGMNLKWDGV----
-----------------------LPSSKAIEEWADDYSSATKLDWDENVRVTSVMDIDGNVTEYYY---GIT
GYTYRIIYPDNLQEWFFRDQAKNITSHIATDGTETSYTYDERGNVLSMVQADGSTFYYEY-DEKDNLIGFVD
AEQGRWFKEYDASGNIIKEIDPLKRETVYAYNA---MGLVTSITDAKGGKKTLKYDDRGNLISYTDCSGK-E
TKWHYDDRGRVKFIENALKQKVEYFYTELTTELRQPIAKGLPLNAFGQL-------------EKIKHADGAE
EHFIHDAEGRLLVHIDPKGQQTRYEYDAAGLITKRTDPLNQTLKYQWDRLS-RLKRLINENGASYEFVYDAA
GRLVKEIDFDGKETVYAYDEYNGRLTTSIEVASTYGQDFKDRAAPKDR----IQQFIFDSMGRLEQRTAGYG
YQGQELEEKQTEEFSYDGLGNLVQAK------------NAETNLQWFYDAAGNLIKEHHQ---DVVTQKTAV
WKHVYDELNNRTTTIRP-DGQ---------KIDWLSY--GSGHVYGMALNGEDVVSFERDDLHRETLRHYAN
G------LSQQQSYDEVGRLTQQLMLSGHD-KGYQAQTQ-----NNAIQHTNQLIERLYHYDKTGELTLI--
--------------------------------KDTRRGAIHYKYDPVGRLL------EATSKLGKETFNFDP
ASNILER------YNSTKE-----------QSSQHISDEKGYGYNRLVNNIVQEYLDQQYQYDAFGQLIRQK
SSQGDL-----NLEWDVFGRLVR-------SRNNQYTAEYRYDALGRRIQKRSKH-------HHTGQEQNIS
YGWDGDTL--AYES----SADLTKHYFYEKDS-FVPLLQAAYHHPIELHQTQDWSD---KTYSIYKDPLWNT
VKQSQ--GFDDVWFYHCDHLGTPQEMSDQTGAIIWKAEYKA-WGECKLEQTNSDFFEKSEIISN-NIRFQGQ
YFDEETGLHYNRYRYYSPYVGRFISKDPIGLLGGFNVYAYTAN-PVQWVDPYGL
>XH857_6

---MSGKTEVVKPLTEVAIKDVSHSKQK-----------------IDSWLQSHTHQIVTLDLVASVMGTVPI
IGNVMAAIDVV-SDICYIYDKGFQNADIMDWTTMGIDLIGVAP-----IPGTTAIRTSARPALFLVRQEVKR
VGKAALG---------------------EAVIVVISNHLNDQVAGDIEKFCKTGLNKLNSILATCGATVQNA
TTQINGGIVRV---VTKGQIFTNAGSNASRAQ--QQAAGVIKGIKSGDLTRAGKNLVYMTENWGKALIKTGA
NSNAKVASSLVPAHLKQPILNVGATINKIGITANTKIKGLGNTAQPQTIGWLFDVLRMSASKFRKKK--SAQ
VKPTQTTQVKHDKKNNKLEKSNDEHAATHNANAC------------KNCKGGTTHSITFALGTEFL---NHI
DAQIHPLLIEQFSRTYVSNLYQYDQSIFGARWITPFTTKIVRCSSYNVHHPKEPIE---QDGWEYIGSDGRP
VRLPELKVGQSYYDE-VETFTYTVISP---------EIRA-----ISYGVSETR--------FYQKYKNEF-
RLASIERQNGFSIG---LRYDHELENSDSYLSDIIFKQQQILAHVALQI---------NDAQKVVAAWLVEE
G---QLIRQL-------SAYQYNEQDDLIKATNEFA-ASYEYQY-QAHLLTRYTDLTHRGMNLKWDGV----
-----------------------LPSSKAIEEWADDYSSATKLDWDENVRVTSVMDIDGNVTEYYY---GIT
GYTYRIIYPDNLQEWFFRDQAKNITSHIATDGTETSYTYDERGNVLSMVQADGSTFYYEY-DEKDNLIGFVD
AEQGRWFKEYDASGNIIKEIDPLKRETVYAYNA---MGLVTSITDAKGGKKTLKYDDRGNLISYTDCSGK-E
TKWHYDDRGRVKFIENALKQKVEYFYTELTTELRQPIAKGLPLNAFGQL-------------EKIKHADGAE
EHFIHDAEGRLLVHIDPKGQQTRYEYDAAGLITKRTDPLNQTLKYQWDRLS-RLKRLINENGASYEFVYDAA
GRLVKEIDFDGKETVYAYDEYNGRLTTSIEVASTYGQDFKDRAAPKDR----IQQFIFDSMGRLEQRTAGYG
YQGQELEEKQTEEFSYDGLGNLVQAK------------NAETNLQWFYDAAGNLIKEHHQ---DVVTQKTAV
WKHVYDELNNRTTTIRP-DGQ---------KIDWLSY--GSGHVYGMALNGEDVVSFERDDLHRETLRHYAN
G------LSQQQSYDEVGRLTQQLMLSGHD-KGYQAQTQ-----NNAIQHTNQLIERLYHYDKTGELTLI--
--------------------------------KDTRRGAIHYKYDPVGRLL------EATSKLGKETFNFDP
ASNILER------YNSTKE-----------QSSQHISDEKGYGYNRLVNNIVQEYLDQQYQYDAFGQLIRQK
SSQGDL-----NLEWDVFGRLVR-------SRNNQYTAEYRYDALGRRIQKRSKH-------HHTGQEQNIS
YGWDGDTL--AYES----SADLTKHYFYEKDS-FVPLLQAAYHHPIELHQTQDWSD---KTYSIYKDPLWNT
VKQSQ--GFDDVWFYHCDHLGTPQEMSDQTGAIIWKAEYKA-WGECKLEQTNSDFFEKSEIISN-NIRFQGQ
YFDEETGLHYNRYRYYSPYVGRFISKDPIGLLGGFNVYAYTAN-PVQWVDPYGL
>XH859_6
---MSGKTEVVKPLTEVAIKDVSHSKQK-----------------IDSWLQSHTHQIVTLDLVASVMGTVPI
IGNVMAAIDVV-SDICYIYDKGFQNADIMDWTTMGIDLIGVAP-----IPGTTAIRTSARPALFLVRQEVKR
VGKAALG---------------------EAVIVVISNHLNDQVAGDIEKFCKTGLNKLNSILATCGATVQNA
TTQINGGIVRV---VTKGQIFTNAGSNASRAQ--QQAAGVIKGIKSGDLTRAGKNLVYMTENWGKALIKTGA
NSNAKVASSLVPAHLKQPILNVGATINKIGITANTKIKGLGNTAQPQTIGWLFDVLRMSASKFRKKK--SAQ
VKPTQTTQVKHDKKNNKLEKSNDEHAATHNANAC------------KNCKGGTTHSITFALGTEFL---NHI
DAQIHPLLIEQFSRTYVSNLYQYDQSIFGARWITPFTTKIVRCSSYNVHHPKEPIE---QDGWEYIGSDGRP

VRLPELKVGQSYYDE-VETFTYTVISP---------EIRA-----ISYGVSETR--------FYQKYKNEF-
RLASIERQNGFSIG---LRYDHELENSDSYLSDIIFKQQQQILAHVALQI--------NDAQKVVAAWLVEE
G---QLIRQL-------SAYQYNEQDDLIKATNEFA-ASYEYQY-QAHLLTRYTDLTHRGMNLKWDGV----
-----------------------LPSSKAIEEWADDYSSATKLDWDENVRVTSVMDIDGNVTEYYY---GIT
GYTYRIIYPDNLQEWFFRDQAKNITSHIATDGTETSYTYDERGNVLSMVQADGSTFYYEY-DEKDNLIGFVD
AEQGRWFKEYDASGNIIKEIDPLKRETVYAYNA---MGLVTSITDAKGGKKTLKYDDRGNLISYTDCSGK-E
TKWHYDDRGRVKFIENALKQKVEYFYTELTTELRQPIAKGLPLNAFGQL-------------EKIKHADGAE
EHFIHDAEGRLLVHIDPKGQQTRYEYDAAGLITKRTDPLNQTLKYQWDRLS-RLKRLINENGASYEFVYDAA
GRLVKEIDFDGKETVYAYDEYNGRLTTSIEVASTYGQDFKDRAAPKDR----IQQFIFDSMGRLEQRTAGYG
YQGQELEEKQTEEFSYDGLGNLVQAK------------NAETNLQWFYDAAGNLIKEHHQ---DVVTQKTAV
WKHVYDELNNRTTTIRP-DGQ---------KIDWLSY--GSGHVYGMALNGEDVVSFERDDLHRETLRHYAN
G------LSQQQSYDEVGRLTQQLMLSGHD-KGYQAQTQ-----NNAIQHTNQLIERLYHYDKTGELTLI--
--------------------------------KDTRRGAIHYKYDPVGRLL------EATSKLGKETFNFDP
ASNILER------YNSTKE-----------QSSQHISDEKGYGYNRLVNNIVQEYLDQQYQYDAFGQLIRQK
SSQGDL-----NLEWDVFGRLVR-------SRNNQYTAEYRYDALGRRIQKRSKH-------HHTGQEQNIS
YGWDGDTL--AYES----SADLTKHYFYEKDS-FVPLLQAAYHHPIELHQTQDWSD---KTYSIYKDPLWNT
VKQSQ--GFDDVWFYHCDHLGTPQEMSDQTGAIIWKAEYKA-WGECKLEQTNSDFFEKSEIISN-NIRFQGQ
YFDEETGLHYNRYRYYSPYVGRFISKDPIGLLGGFNVYAYTAN-PVQWVDPYGL
>XH860_6
---MSGKTEVVKPLTEVAIKDVSHSKQK-----------------IDSWLQSHTHQIVTLDLVASVMGTVPI
IGNVMAAIDVV-SDICYIYDKGFQNADIMDWTTMGIDLIGVAP-----IPGTTAIRTSARPALFLVRQEVKR
VGKAALG---------------------EAVIVVISNHLNDQVAGDIEKFCKTGLNKLNSILATCGATVQNA
TTQINGGIVRV---VTKGQIFTNAGSNASRAQ--QQAAGVIKGIKSGDLTRAGKNLVYMTENWGKALIKTGA
NSNAKVASSLVPAHLKQPILNVGATINKIGITANTKIKGLGNTAQPQTIGWLFDVLRMSASKFRKKK--SAQ
VKPTQTTQVKHDKKNNKLEKSNDEHAATHNANAC------------KNCKGGTTHSITFALGTEFL---NHI
DAQIHPLLIEQFSRTYVSNLYQYDQSIFGARWITPFTTKIVRCSSYNVHHPKEPIE---QDGWEYIGSDGRP
VRLPELKVGQSYYDE-VETFTYTVISP---------EIRA-----ISYGVSETR--------FYQKYKNEF-
RLASIERQNGFSIG---LRYDHELENSDSYLSDIIFKQQQQILAHVALQI--------NDAQKVVAAWLVEE
G---QLIRQL-------SAYQYNEQDDLIKATNEFA-ASYEYQY-QAHLLTRYTDLTHRGMNLKWDGV----
-----------------------LPSSKAIEEWADDYSSATKLDWDENVRVTSVMDIDGNVTEYYY---GIT
GYTYRIIYPDNLQEWFFRDQAKNITSHIATDGTETSYTYDERGNVLSMVQADGSTFYYEY-DEKDNLIGFVD
AEQGRWFKEYDASGNIIKEIDPLKRETVYAYNA---MGLVTSITDAKGGKKTLKYDDRGNLISYTDCSGK-E
TKWHYDDRGRVKFIENALKQKVEYFYTELTTELRQPIAKGLPLNAFGQL-------------EKIKHADGAE

EHFIHDAEGRLLVHIDPKGQQTRYEYDAAGLITKRTDPLNQTLKYQWDRLS-RLKRLINENGASYEFVYDAA
GRLVKEIDFDGKETVYAYDEYNGRLTTSIEVASTYGQDFKDRAAPKDR----IQQFIFDSMGRLEQRTAGYG
YQGQELEEKQTEEFSYDGLGNLVQAK------------NAETNLQWFYDAAGNLIKEHHQ---DVVTQKTAV
WKHVYDELNNRTTTIRP-DGQ---------KIDWLSY--GSGHVYGMALNGEDVVSFERDDLHRETLRHYAN
G------LSQQQSYDEVGRLTQQLMLSGHD-KGYQAQTQ-----NNAIQHTNQLIERLYHYDKTGELTLI--
--------------------------------KDTRRGAIHYKYDPVGRLL------EATSKLGKETFNFDP
ASNILER------YNSTKE-----------QSSQHISDEKGYGYNRLVNNIVQEYLDQQYQYDAFGQLIRQK
SSQGDL-----NLEWDVFGRLVR-------SRNNQYTAEYRYDALGRRIQKRSKH-------HHTGQEQNIS
YGWDGDTL--AYES----SADLTKHYFYEKDS-FVPLLQAAYHHPIELHQTQDWSD---KTYSIYKDPLWNT
VKQSQ--GFDDVWFYHCDHLGTPQEMSDQTGAIIWKAEYKA-WGECKLEQTNSDFFEKSEIISN-NIRFQGQ
YFDEETGLHYNRYRYYSPYVGRFISKDPIGLLGGFNVYAYTAN-PVQWVDPYGL
>YU-R612_6
---MSGKTEVVKPLTEVAIKDVSHSKQK-----------------IDSWLQSHTHQIVTLDLVASVMGTVPI
IGNVMAAIDVV-SDICYIYDKGFQNADIMDWTTMGIDLIGVAP-----IPGTTAIRTSARPALFLVRQEVKR
VGKAALG---------------------EAVIVVISNHLNDQVAGDIEKFCKTGLNKLNSILATCGATVQNA
TTQINGGIVRV---VTKGQIFTNAGSNASRAQ--QQAAGVIKGIKSGDLTRAGKNLVYMTENWGKALIKTGA
NSNAKVASSLVPAHLKQPILNVGATINKIGITANTKIKGLGNTAQPQTIGWLFDVLRMSASKFRKKK--SAQ
VKPTQTTQVKHDKKNNKLEKSNDEHAATHNANAC------------KNCKGGTTHSITFALGTEFL---NHI
DAQIHPLLIEQFSRTYVSNLYQYDQSIFGARWITPFTTKIVRCSSYNVHHPKEPIE---QDGWEYIGSDGRP
VRLPELKVGQSYYDE-VETFTYTVISP---------EIRA-----ISYGVSETR--------FYQKYKNEF-
RLASIERQNGFSIG---LRYDHELENSDSYLSDIIFKQQQQILAHVALQI--------NDAQKVVAAWLVEE
G---QLIRQL-------SAYQYNEQDDLIKATNEFA-ASYEYQY-QAHLLTRYTDLTHRGMNLKWDGV----
-----------------------LPSSKAIEEWADDYSSATKLDWDENVRVTSVMDIDGNVTEYYY---GIT
GYTYRIIYPDNLQEWFFRDQAKNITSHIATDGTETSYTYDERGNVLSMVQADGSTFYYEY-DEKDNLIGFVD
AEQGRWFKEYDASGNIIKEIDPLKRETVYAYNA---MGLVTSITDAKGGKKTLKYDDRGNLISYTDCSGK-E
TKWHYDDRGRVKFIENALKQKVEYFYTELTTELRQPIAKGLPLNAFGQL-------------EKIKHADGAE
EHFIHDAEGRLLVHIDPKGQQTRYEYDAAGLITKRTDPLNQTLKYQWDRLS-RLKRLINENGASYEFVYDAA
GRLVKEIDFDGKETVYAYDEYNGRLTTSIEVASTYGQDFKDRAAPKDR----IQQFIFDSMGRLEQRTAGYG
YQGQELEEKQTEEFSYDGLGNLVQAK------------NAETNLQWFYDAAGNLIKEHHQ---DVVTQKTAV
WKHVYDELNNRTTTIRP-DGQ---------KIDWLSY--GSGHVYGMALNGEDVVSFERDDLHRETLRHYAN
G------LSQQQSYDEVGRLTQQLMLSGHD-KGYQAQTQ-----NNAIQHTNQLIERLYHYDKTGELTLI--
--------------------------------KDTRRGAIHYKYDPVGRLL------EATSKLGKETFNFDP
ASNILER------YNSTKE-----------QSSQHISDEKGYGYNRLVNNIVQEYLDQQYQYDAFGQLIRQK

```
SSQGDL-----NLEWDVFGRLVR-------SRNNQYTAEYRYDALGRRIQKRSKH-------HHTGQEQNIS
YGWDGDTL--AYES----SADLTKHYFYEKDS-FVPLLQAAYHHPIELHQTQDWSD---KTYSIYKDPLWNT
VKQSQ--GFDDVWFYHCDHLGTPQEMSDQTGAIIWKAEYKA-WGECKLEQTNSDFFEKSEIISN-NIRFQGQ
YFDEETGLHYNRYRYYSPYVGRFISKDPIGLLGGFNVYAYTAN-PVQWVDPYGL
>6200_21
---MSGKTEVVKPLTEVAIKDVSHSKQK-----------------IDSWLKSHTHQIVTLDLVASVMGTVPI
IGNVMAAIDVV-SDICYIYDKGFQNADIMDWTTMGIDLIGVAP-----IPGTTAIRTSARPALFLVRQEVKR
VGKAALG---------------------EAVIVVISNHLNDQVAGDIEKFCKTGLNKLNSILATCGATVQNA
TTQINGGIVRV---VTKGQIFTNAGSNASRAQ--QQAAGVIKGIKSGDLTRAGKNLVYMTENWGKALIKTGA
NSNAKVASSLVPAHLKQPILNVGATINKIGITANTKIKGLGNTAQPQTIGWLFDVLRMSASKFRKKK--SAQ
VKPTQTTQVKHDKKNNKLEKSNDEHAATHNANAC------------KNCKGGTTHSITFALGTEFL---NHT
DAQIHPLLIEQFSRTYVSNLYQYDQSIFGARWITPFTTKIVRCSSYNVHHPKEPIE---QDGWEYIGSDGRP
VRLPELKVGQSYYDE-VETFTYTVISP---------EIRA-----ISYGVSETR--------FYQKYKNEF-
RLASIERQNGFSIG---LRYDHELENSDSYLSDIIFKQQQQILAHVALQI--------NDAQKVVAVWLVEE
G---QLIRQL-------SAYQYNEQDDLIKATNEFA-ASYEYQY-QAHLLTRYTDLTHRGMNLKWDGV----
-----------------------LPSSKAIEEWADDYSSATKLDWDENVRVTSVMDIDGNVTEYYY---GIT
GYTYRIIYPDNLQEWFFRDQAKNITSHIATDGTETSYTYDERGNVLSMVQADGSTFYYEY-DEKDNLIGFVD
AEQGRWFKEYDASGNIIKEIDPLKRETVYAYNA---MGLVTSITDAKGGKKTLKYDDQGNLISYTDCSGK-E
TKWHYDDRGRVKFIENALKQKVEYFYTELTTELRQPIAKGLPLNAFGQL-------------EKIKHADGAE
EHFIHDAEGRLLVHIDPKGQQTRYEYDAAGLITKRIDPLNQTLKYQWDRLS-RLKRLTNENGASYEFVYDAA
GRLVKEIDFDGKETVYAYDEYNGRLTTSIEVASTYGQDFKDRAAPKDR----IQQFIFDSMGRLEQRTAGYG
YQGQELEEKQTEEFSYDGLGNLVQAK------------NAETNLQWFYDAAGNLIKEHHQ---DVVTQKTAV
WKHVYDELNNRTTTIRP-DGQ---------KIDWLSY--GSGHVYGMALNGEDIVSFERDDLHRETLRHYAN
G------LSQQQRYDEVGRLTQQLMLNGHD-KGYQAQTQ-----NNAIQHTNQLIERLYHYDKTGELTLI--
--------------------------------KDTRRGAIHYKYDPVGRLL------EATSKLGKETFNFDP
ASNILDR------YNSTKE-----------QSSQHTATEKGLGYNRLVNNIVQEYLDQQYQYDAFGQLIRQK
SSQGDL-----NLEWDVLGRLVR-------SRNNQYTADYRYDALGRRIQKRSKH-------HHTGQEQNII
YGWDGDTL--AYES----SADLTKHYFYEKDS-FVPLLQAAYHHSIELHQTQDWTE---KTYSIYKDPLWNT
VKQSQ--GFDDVWFYHCDHLGTPQEMSNKTGAIVWKAQYKA-WGECKVEQAKSDFFENSEIISN-NIRFQGQ
YFDGETGLHYNRYRYYSPYIGRFISKDPIGLLGGYNHYQYGPN-PISWVDPTGL
>RhsB
---MLNDILSRVARVGAMHAGNRPNPPADRPQPCQGKPPTSPGKTIKHKSFLGALAGAVAGALVAAAVAAAA
VFLVGVTGGLAVAAVGALAVFAAGDLISAVTNKVSAVVDSASPAFGPVASGSGNVFVEKQPVARA-------
```

```
----------------------------------------------------------TKDTVACTKHNSPQ
L--IAQGSESV---FVNDAPAARIDDKTVCGA--TLKEG-ASTVFFGSGQGTYLEIADEFSWW---EKA---
----------------------------------------LLIAVEFLVPPSRGMLKGLGKLFTRNG--LKS
VLKGAKAGALFIT--KVPGKMGCAARAFKANKGM--------ARFKEAAKAFKKDPVYLASGEVIE---SRT
DIELGQTLPLVFERTYRSAS-AHT-GLLGRGWHDSWSE-VATVTHDGL-----------NTHVVITLAQGYD
IDFTFHQDVQAVYCPHYPEFTLHRRGD---------GFS------LWHRDQQTW------RDFSVVQGERR-
LLSAIHDSHDNRIE---LVRDPK-----GYLRQLRHSDGVTLL--LVWQG--------EYLHQIQRI---DG
G------QKT-----LLAEYRQDEQGRLVEANATHA-YHLYYEYNTAHRLTRWHDNDQTWARYEY-------
-----------------------DAQGRCV-YTTCADGFLT-ARFDYLPDRVVMTDGLGQRSEFGF------
----------NDLHLMSWEQS--------PLGHITRYEYDEVGNLLREISPAGRVVEFTYLDDTGRVSTFTD
GSGHQWQYDYDDAQRLCGVTDPLGREWGWVYDA---EGNPERLTGPDASEVRFTWNRYGLLTQVSDAAGEVQ
ARLQYDHRQRLLSATDAESRTRQLRYD------RQDRVVQWQRADGARFRL-GYRRASWTLPEQLIRPDDKE
EQRQYDRHNNLLSYVDGNGALWRQTFGPFDLLTARTDAEGRTWRYEYDRESQQLIAVTAPDGSRWQWWLDAD
GRVIRERDMTGTETHYGYDE-DGLCIRVRN---------GEGD---------TRHFLYDARGLLLRETAPDD
TL----------HYRYDAAGRLTEVS------------SATAHVQLDYDLRDRVVREWHN---------GTL
LTRQYDDAARTVTRTLTWDGDADDTTGTLAPLTSLFHYTRTGELRQVQLPDGADLTLTHDAAGRESLRTGGS
G------FVQQREYDVMGWLTREQSGAQHD-GRLQPAQT-----------------REYRYDGAGNLTGV--
---------------------------------RHNRDAEGYRLDATGRVQEMLSGGAGKPVDTTARFHYTR
TG--LPQ------EAGRLT--------------------------EWQAGRLVQHDDTHYQYDRAGRLIRKQ
VVQPGYRPQVWQYRWDSRNQLRVV------DTPNGERWLYRYDPFGRRVGKRCDQ----------KAEE-TR
YLWDGDQIAEIRHY-RHGQLIQRRHWVYNG---WELVVQ------QRQHTGGDW------------------
----------ETDFVTSSQNGTPQALFTPDGTLRWQVPKATLWGQRQTEKS--------ESPDP-GLAFAGQ
LRDSESGLCYNRFRYYDPAGGCYVSPDPIGIAGGESNYGYVQN-PNTRVDPLGL
>RhsA
---MLNDILSRVARVGAMHAGNRPNPPDDRPQPCRGKPPTSPGKTIKHKSFLGALAGAVAGALVAAAVAAAA
VFLVGVTGGLAVAAVGALAVFAAGDLISAVTNKVSAMVDSASPAFGPVASGSGNVFVEKQPVARA-------
----------------------------------------------------------TKDTVACTKHNSPQ
L--IAQGSESV---FVNDAPAARIDDKTVCGA--TVKEG-ASTVFFGSGQGTYLDIADEFSWW---EKA---
----------------------------------------LLIAVEFLVPPSRGMLKGLGKLFIR-G--PKA
VLRGSRAGA-KWIAGRLADKSSCASKAFKASSGL--------TRAKAAVKAFLKDPVYIASGEVIE---SRT
DIELGQTLPLAFERTYRSAS-VHI-GLLGRGWHDSWSE-VATVTRDGL-----------NTHVVITLAQGYD
IDFTFHQDVQAVYCPHYPEFTLHRRGD---------GFS------LWHRDQQTW------RDFSVVQGERR-
LLSAIHDSHDNRIE---LVRDPK-----GYLRQVRHSDGVTLL--LVWQG--------EFLHQIQRI---DG
```

G------QKT-----LLAEYRQDEQGRLVEANATQA-YHLYYDYDAAHRLTRWHDNDQTWARYEY-------
-----------------------DAQGRCV-YTTCADGFLT-ARFDYLPDRVVMTDGLGQCSEFGF------
----------NDLFLMSWEKS--------PLGHVTRYEYDDYGNLLREISPAGRVVEFTYLDDTGRVSTFTD
ASGHQWQYDYDAAQRLCGVTDPLGREWGWMYDA---EGNPERLTGPDASEVRFTWNRYGLLTQVSDAAGEVQ
ARLQYDHRQRLLSATDAESRTRQLRYD------GQDRVVQWQRADGARF-RLGYRRASWTLPEQLIRPDDKE
EQRQYDRHNNLLSYVDGNGALWRQTFGPFDLLTARTDAEGRTWHYAYDKESQQLTTVIAPDGSHWQWWLDAD
GRVIERDMTGTETHYDYDE-DGLCIRVRN----------GEGD---------TRHFLYDARGLLLRETAPDD
TL----------HYRYDAVGRLTEVS------------SSTAHVQLEYDLRDRMVREWHN---------GTL
LTRQVDDAARTVTRTLTWDGDADDTINALAPLTSLFHYTRTGELRQVQLPDGADLTLTHDAAGRESHRTGGS
G------FVQQREYDVMGWLTREMSGAQHD-GHLLATQT-----------------REYRYDGAGNLTGV--
---------------------------------RHNRDAEGYRLDATGRVQEILSGGAGKPVDTTARFLYTR
TG--LPQ------EAGRLT--------------------------EWQAGRLVQHDDTHYQYDRAGRLIRKQ
VVQPGYRPQVWQYRWDSRNQLRVV------DTPNGERWLYRYDPFGRRVGKRCDQ----------KAEE-IR
YLWDGDQIAEIRHY-RHGQLIQRRHWVYNG---WELVVQ------QRQHTGGDW------------------
----------ETDFVTSSQNGTPQALFTPDGTLRWQAPKATLWGQRQAEKS--------ESPDP-GLAFAGQ
LRDSESGLCYNRFRYYDPAGGCYVSPDPIGIAGGESNYGYVSN-PMCWVDPFGL
>Tse5
------------------------------------------------------------------------
------------------------------------------------------------------------
------------------------------------------------------------------------
---------------MSGLPVSHVGEKVSGGV---ISTG-SPTVHVGSSAV----------------------
-------------------------------------------------GLADRVSACV-------------
--------------------------------PL--------V----------GKPVNPMLGSKLL--PEEV
DFALAAPDTFTFARGYLSSN-PRI-GRLGRGWWLPGESMHLELSEDA---------------CVLVDAQGRR
IGFPALAPGAQHYSG-SEELWLRRGGSSGGEAQAWRGRWAAVPAELQTQEGSVLVLSGHSYLHFQRCPDGIW
RLQASFGRAGYRTE---FRWSGR-----GLLTGVRDSAGRS-Y-ALVYQQACEPSEGDDGLRLFGVILASHD
GPPPDYIDPQSPGLDWLVRYQFSDSGDLIAVRDRLGQVVRVFAW-REHMLVAHGEPGGLEVRYEWDVH----
-----------------------APHGRVVKQIEAGGLTRT-FRYLRDA--TEVSDSLGRVERYEFAGEGGQ
RRWTALVRADGSRSEFDYDLFGRLVAMRDPLGRETRRRRDGQGRMLEEESPGKARYRKRVDEETGLLVELED
AMQRRWTFERDERGNATTVRGPAGST-RYAYEDPRLPDRPTRIVDPRGGERRLEWNRFGLLAALTDCSGQ-V
WRYDYDNEGRLVASSDPLGQLTRRRYD-----------------PLGQL-------------IGLELADGSA
LSYEYDALGRQTRIADAEGHATLFSWGHGDLLARVSDAGGGELSYLHDEAG-RLVALTNENGVQAQFRYDLL
DRLVEETGFDGRRQRYRYNA-ADELIARED---------ADGR---------ETTYAYDRDGRLASIRVPAT

EH----APALVERYRWLADGRLASAG------------GADCEVRYTYDEVGNLRLESQV----HADGWVYS
VEHSHDALGVRQTSRYG-DAP---------PVAWLTY--GPGHLHG-ALVGAVELAFERDALHREVRRDARR
DGQDDALFTQERQHAPLGRLQRSRLRLAGG----FDWQ------------------RGYRYDGLGQLVGI--
--------------------------------DDNQYPSVRYEYDLGGRLL-----ASRRAGAAASTYRYDA
AGNRLEGVGEHAREDARQAFAENELYRSGFSRSETRASQAGEGPARWAGNRVERIAGNRYRFDALGNLVERI
GADGERL----RLAYDGAQRLVHLTRD--YADGTRLEARYRYDALSRRIAKVVLR---------DGVEQQVR
FGWDGDRQ--CAEA----FARELRTTVHEPGG-FVPLLR------LEQACEPDPPELLQLRQAFAAEGQPLP
AQCVPALGEARIAFFHTDHLGTPLQLSDERGQLRWQGVPDD-WRAVAPERQ---------PGAQ-PIRFQGQ
YHDEESGLYYNRYRYYLPEAGRYASQDPLGLGGGPNPYAYALNAPTLAYDPTGL
>Tke4
---MAQTVHAARMGDAILHPSLAAEMISAVAEAVIYAAATAA---VAAAISLAVVGTVATGG----AAGVAI
AAVAGVAVGAV-STLSVGEDQTVGDAISSFCDSLGNSVDSPDP-YGKIESGSTNVLINSEPAARAAGITGPP
GGGAADTEQEEPSILENVGSMAMAAVPYLVPFVGLGMAIRDIFNPPVTTPANPGSEPAEGDKISCSRHPPLP
DTFIAQGSDKV---FINGQPAARSGDKTTCDATIDVNENVSPNVRIGGGTATVRDIRN--------------
----------------------------------------GKSKIAMFTGIIAGMLISR-RFGRIKG-----
----------------------CTL----------------------------GNPVAVATGSKLQEGPEDI
DFNLPGLLAIQWARRYDSRDMRSD-GLLGMGWSVPYEVELARVPHPQG-----------GTLWIYIDNDGNR
LELGRLSAGDAFISA-VDGLAFFQLED---------GQTV-----VEDINEGLY----RVFDTDPLNPKRS-
RLIRLGDRNLNCLD---LLYDEQ-----GRLKALYDKYGQTVV-QLHYAARHP-----QRVSEVSRVFLKNG
G------TPTVERSELLVSYRYTDNGQLHEVLDATDQRVRRFTYTPEGYLNSHQLASGAVREYEWARFAIPE
TRPTPKRLDGTPYRLPPLLEPQPDHEWRVIRHWGSDGEEYR-FEYNLEAGETLVTDNLGRRDHYYW---GPL
YEVYKHIDPQGN-CWLAEVIAGQLIKRIDPQGGEWRYSYDDIGRLIETRDPLGRSEHIKYLRHWALPIQVTD
TAGRTRQYGYDSHGNLLWQQDPLGRETQYQYDP---EGRVTQVTDALEKSKYLSWNTCGQLLSYRDCSNA-Q
TLYHYDAHGRLRESINARGEHTHFRYD-----------------ARGYL-------------VESERPDGRI
DRYEIDVAGQLTRYIDPAQKTLQFRYDPSGRLVERTDAMGYSVKFRYDAYG-RLLQLTNENDESYRFGWDEL
DRLVAQKDLDGSGRLYAYNV-LDEVIRLTHVPSPDEQPPLSDNAPPTRTTAIRHDFERDAIGRLVSKRTEDG
TT----------DYRYDAADNLLSIT-------FTDNKGEKQQLDYTYDANGQLLSETNS---------AGL
LQYRYDELGNLQTLVLP-DQR---------ELNHLYY--GSGHLHQINLNGRVISDFERDAVHDEVLRTQGK
-------LVTRTRYDTSGRLAGKAIHYRDAPAEVLPLLD-----------------KAYRYDASDNLIAEVL
TQTQRRGMSNAANDENANLEQIIGRFLDLPHTGKTYSGHNRYGYDLNEQLQTVHQSRPNWQATQVEDFKYDK
AGNLFDG--------PKLN-------------------------GLIKHNRVLVYQDKRYRYDRFGRLCEKR
IGSNWVQ----YFEYDAEQRLVCVEQY---RSGERERVVFAYDPLGRRISKEVYQ------RDYPEPRRRVL
FHWQGFRL--LQEV----QSGLASLYVYATVESYDPLAR------VDGKP----------------------

-------GSEAIHYFHTTLVGQPEQLTDSDGNTIWRSDHHG-WGKIINEWH-----SQQNGREQ-NLRNQGQ
YIDRETGLHFNIFRFYDPDIGRFTTTDPLGIEGGVNLYSYAPN-IVNYSDPLGL
>Rhs1
---MAE-IWAARKDDELIHTSALADIASAVAEVAVYAVATAAVSAAAAAAAPALAGVAVLGA--AATAASGC
VG-AGLIVGFATSLTGV------GEHISNGCDAVANWLFPPSV-SGVILTGSPNVFTNAKPAARAAGRLLTP
AEIKALPAPQPPQSFWDYG---------AELLAAGGNILSQLWQPGVAGPSQPSQ-PLEEDKISCDKHPLPQ
Y--LAEGSSKV---FINGQPAVRSNDRSTCEA--KVSTDVSKNVQIGGGTVVVRD-----------------
----------------------------------------IKSGKLPGIGLALTVLPLL--LGNPAK--LVK
NLPCMIGGGLAAAGVNALGEAIHAA----------------------------FNPVHAATGAKVLNDEEDL
DLLLPARFPLIWQRNYNSRN-GYQ-GLFGHGWRTPFEIWVDLNDRDG------------VIECCLHDETGRE
LRFPLPEVNQPSFSA-GEGLMFRRGEE---------GQLL-----IADTDGSVW----RLFVPLPQTPSRL-
RLVSLSDEYDNALL---LNYDER-----QRLSHIVDSAGTMTL-RLEYGDP-------RFDDRVTALRHND-
-------EPR-------VRYRYDGGGDLHQVIDAGGLVTREFHYNAQHLLTRHQQAGGPSFAYRWAMF----
------------------------DDWRVVEHRDSAGGGCT-IDYDLAAGLTTVTEYDGLRHQHRW---NAQ
RLIVEYTDERGEVWHYQWNDEDMLIATRDPLGHGWHFRYDEAGNLVEEEDPLGHIRLTGWLPQRALPISVKE
PNGATQHFYYDERHALIAEIDALGHTQRYLRDG---YGQVVQAVDANGGISHFAYDEHGQLLRARDCSGK-Q
TLYKYTAQGWLSEVTDAAGERVHYRYD-----------------AAGRL-------------TQLQRAEGWQ
ETLSWNAQGQLDRYQGADGRLTHYRYDAAGRLLATRNPLDGEVVRHYDARG-RLTALRNENAESYLFHWTAC
NLLQEEIGLDGVSTRYRYDE-CRRVVERRFAANTSAAF--------------SHYFSHDGAGQLVSKQTPDG
ET----------RYRYSPLGQLLHAASYRGNAWHDEPTTAQSEVSFDYDLLGRVTREQGA---------NGT
LRYDYDPMGNRTQTTLP-DGR---------TLRALYY--GSGHLLQLALDEITLSEFERDNLHRELSRTQGR
-------LTSRVHYDRLGRKDRREIFNDNR-QRPAPASS----------------SRQWSYDYQNNLTRE--
------------------------------EHNANPFSYQRYQYDATGRLL-----SRDGTRPDTERYRYDP
ASNLLDE-----------------------------------GQFRQRHNRVLHYQGIDYRYDVFGRTVEKR
KGHYRW-----QYLYDADHRLCEVVRYSQHRSEAQQHVHFHYDPLGRRTQKRVWQQSQDLRQPAGKCQT-TT
FLWEGFRL--LQET----RDGMPLTYVYADQGSYEPLAR------IDGH-----------------------
-------APAQVFYFHTAPNGEPESLTDSDGTLRWQSHSSA-WGRIKYEEN-----QQDLDYSQ-NLRLQGQ
YLDRETGLHYNLFRYYDPDIGRFTQHDPIGLAGGINLYQYAPN-PLGWVDPWGW
>Tke2
------MFEAARFGDEISHTSALGGFLIG----------------------------AALGI-----ALVAT
VAIATFTCGFGVALLAGLAAGIGGSLLTAAGEAIGSMFSSPS---GTITTASPNVFINSRKAARV-------
----------------------------------------------------------EKSIGACDKHPGPV
Q--IAEGSTNV---FINSVAAARKGDKLTCGA--TISGG-SDNVIIGGGTYRYLPVDDEVPEWLRT------

----------------------------------------TVDVLMAIAGAAGGIAQLI-KAGTQAG--MKA
VMPCALKFTAGFVAGEVASRYVVEPVAR---KAI---------------GGLVGNPVDLTTGRKLI--PDEI
DFSLPGLMPIEWSRFYASDL-TVD-SVLGRGWVLPWEQSLRRQ----------------GAFIYLTDNQGRE
VPFVTLQPGQRIYNP-HEQVYLVCTEG---------GHYI-----LQTLDNLFF-----YFGEVPDTNTEV-
PLQRIENVLGHFLH---FTRTPD-----GTLTDISATGGTRVH--LHYDNPL------GRLTDIKRVVNNEA
V------ETL-------TQYRYDEHGQLSAVINRNGDTVRSFSY-ADGLMVTHSNALGLGCHYRWQTL----
-----------------------GDKPRVVEHWTSDGEHYH-FRYDLDSRTSWATDVLGRELEVQY---NAD
HRVVASRDYGGERYAIELDEHGNMVGLTLPDGNRLQFKYDEFSRLLEETDPLGRKTQYEYHHLTTLVTQVSY
PDGSTWRARFDDKGNLLAEFDALGQMTEYLNSD---DGLPHTIIDATYKSKYLWWNTLAQVERYQDCSGK-S
TYYRYDERQHLVAVTDALNQTTTLARK-----------------PDGEV-------------LRISHPDGTT
ESFTYNVYGQVLSHTDGKGQTTRLMRTARGLPSSRQDAKGQRVRYEYDKAM-RLTALVNENNATYSFAYDAS
DRLSEEVRVDNLTRRFSYDV-GGHLTRLDEIGY-GENAERPER---------HTLFERDAIGRLIAKLNSDA
QQ----------RFTYDDGDRLLSIE-RQPTGIGKQLGITEEKLEYAYDLLGRLTKEISP---------DGT
LGYEYDPLSNLTTLTLP-DGR---------KVNHLYY--GSGHLHQLNLDGQVISDMERDDLHREVYRTQGK
-------LTSCFGYDAMGRKAWQFASTLPA-DKLSQVHNTGINTSLLVEHAYNPIHRRYQYDPAGELVRT--
--------------------------------LDKLRGEIKYEYEANGQLR----SRDTGSLIGSEEFRYDP
AANRLDF------NARQFD--------------------------KVKDNRIKQWRDQEYRYDPWGNLIEKR
SGHSKLQ----HFSYDCENRLVRAETL--VNGKLESQGEYRYDSLGRRIAKQAEI--------NGEVEQ-KR
FLWQGLRM--LREE----TPGQSILYLYEPGS-YAPLAR------VDQVEG---------------------
-------EGQKVYYFHTDQIGTPLELTDSEGEIVWQATYRS-WGTIETLSI--------KEVDQ-NLRFQGQ
YFDAETGLHYNTFRYYYSEVGRYVTQDPIGLDGGLNLYRYGQN-PAGWIDPWGW
>Rhs2
------MTEAARVGDTIGHSHALAGMIAG----------------TLVGGLIAAAGALAAGALFVAGLAASC
IGVGVLLIGAS-LAVGYLT----GEAATAARDGIADAGAGSLTPKGNIVTGSPNVFINGKPAALA-------
----------------------------------------------------------TNSQVACSDDGPSM
Q--MAQGSDKV---SINGQPASRVGDKTNCDA--QVMEG-SPNVFIGGGTVTTLPIKPEVPDWLYK------
----------------------------------------VSDLTLLFAGLVGGVGGAAGKLGALGK--LLG
KLPGI----------NKLARIACRAGTLMTATAA---------------VGIIARPVDIVSGQKFLDGEDDL
DFVLPSRLPVAWQRYWRSGN-PGD-SVLGRGWNLFWESSLQPY----------------QDGLVWRAPSGDF
VAFPMVPRGHKTYCE-AEKCWLMHNDD---------GSWQ-----LFDVGEQIF-------HYPPLAGEQPS
RLSMITDAIGNATS---LFYDDE-----GLLSELVDSAGQRLM--CRYAQ--------GRLREV-ALQTAEG
E------RTL-------ARYGYDEQGQLTTVSNRAGEVTRRFGW-RDGLMISHQDAAGLLNEYQWQEI----
-----------------------DGVPRVTAYRNSAGESLE-FGYDFAGGRRSAVRGDGKRAEWRL---DDD

DNVAQYTDFDQRRYGFIY-QRGELCSVLLPGGAQRQSEWDPYGRLLSETDPLGRVTRYQYSRNSGRLFAVAY
PDGSSEAQHWDTLGRPTRYVDALGNTTLYRYPDDE-ESLPASVIDALGGEVKLEWDARGQLTRYTDCSGS-V
TAYTYDALGQLTAQTDAEGHQTRYRWD-----------------NGGRL-------------HTLIHPDGGE
ERFNWNAHGQLAEHQDALGSLTRWQYNALGLPVSITDRINRTRRYHYSPQG-WLTRLENGNGGDYRFSYDAV
GRVVAEERPDDTRHYYRYGA-AGLLEEHREVGLPGGAGELTQR---------EQRFRFDEAGQLVWRSNASA
EW----------HYRFDAMGRLRELN-RLPTASGVALGIEPDSVQMRYDAAGRLLGEQGV---------NGE
LQYQWDALANLQALTLP-QGD---------RLQWLYY--GSGHASAIKFNQQVVSEFTRDRLHRETGRSQGA
-------LQQQRRYDAMGRRSWQSSAFGHD-KLTRPEDG--------------VLWRAYRYTGRGELAGV--
--------------------------------SDALRGEVHYGYDAEGRLL---QHREPNQGKPGARLVYDL
ADNLLGE------RSPQSD------------------IDAHLPLAPIADNRLTHWQKLFYRYDAWGNLISRR
NGLYEQ-----HYRYDADNRLVQAHGR---GPQGEFEAQYHYDALGRRSRKTVHY--------KGKTEQTTH
FLWQGYRL--LQEQ---RDDGSRRSWSYDPASPWSPLAA------LEQAGDS--------------------
-------RSADIYWYHTDLNSAPLEVTDAAGNLCWSGQYDT-FGKLQGQTVAGAAKRQGAQYQQ-PLRYAGQ
YQDDESGLHYNLFRYYEPEVGRFTTQDPIGLEGGLNLYAYGPN-PLTWIDPFGL

**Supplementary file S4d**
>ab736_7
--------------------MLPVVIGIIELAGYAITAYEL--------YRTAANAYDEVKNYQDNIK---K
AKEEIKKIMKNLDKE-----ITDKIDRQREKVLLTTLTTGDKQTN--STKSASGRPQVKSNVITA-----AI
KQKIPFRPIISQIC-EKADHLPMIQLRKQKGKKLKDVIPKSKVDIVAKLL----KMTAQELAGASVDEYIII
RLKQLAVNFMFEFMDELLKWRSPLKSEVCFGYDIKTRKYLAPQ----LDGQTRIKRVGSELNPFWPMPYKGK
GVIGADIIIPEYRGEPLTLTNIFALVEIKFQNDRIDEKQFNNYNDLKKQCADVKHAGKITGSEGFKLSLFRY
PEDASTTEAKDSKPATMNKKRKTN
>ATCC19606_7
--------------------MLPVVIGIIELAGYAITAYEL--------YRTAANAYDEVKNYQDNIK---K
AKEEIKKIMKNLDKE-----ITDKIDRQREKVLLTTLTTGDKQTN--STKSASGRPQVKSNVITA-----AI
KQKIPFRPIISQIC-EKADHLPMIQLRKQKGKKLKDVIPKSKVDIVAKLL----KMTAQELAGASVDEYIII
RLKQLAVNFMFEFMDELLKWRSPLKSEVCFGYDIKTRKYLAPQ----LDGQTRIKRVGSELNPFWPMPYKGK
GVIGADIIIPEYRGEPLTLTNIFALVEIKFQNDRIDEKQFNNYNDLKKQCADVKHAGKITGSEGFKLSLFRY
PEDASTTEAKDSKPATMNKKRKTN
>AB030_24
------------------------MKRSLTLLLSTFISIQA-------------------------------
--------------------FAE---------------ENACEKI--WDSFKNKNESIKIDKVNV-------

--PCKQIWED----------SNKAKLVKTIGLNPKDAQWM--------------STGECQVIEVNKNKYQVY
YAYLKN--------DHSDINIINDTNGNYFSYYRNLGRYDM------FEDKFYPTKKAN-------------
-KNGVELSCP----GLGNFEKMKPILSSYINNKKIDIKRF--------SFEDLNK-----------------
------------------------
>AbH12O-A2_24
------------------------MKRSLTLLLSTFISIQA-------------------------------
--------------------FAE---------------ENACEKI--WDSFKNKNESIKIDKVNV-------
--PCKQIWED----------SNKAKLVKTIGLNPKDAQWM--------------STGECQVIEVNKNKYQVY
YAYLKN--------DHSDINIINDTNGNYFSYYRNLGRYDM------FEDKFYPTKKAN-------------
-KNGVELSCP----GLGNFEKMKPILSSYINNKKIDIKRF--------SFEDLNK-----------------
------------------------
>AF-401_24
------------------------MKRSLTLLLSTFISIQA-------------------------------
--------------------FAE---------------ENACEKI--WDSFKNKNESIKIDKVNV-------
--PCKQIWED----------SNKAKLVKTIGLNPKDAQWM--------------STGECQVIEVNKNKYQVY
YAYLKN--------DHSDINIINDTNGNYFSYYRNLGRYDM------FEDKFYPTKKAN-------------
-KNGVELSCP----GLGNFEKMKPILSSYINNKKIDIKRF--------SFEDLNK-----------------
------------------------
>AR_0063_24
------------------------MKRSLTLLLSTFISIQA-------------------------------
--------------------FAE---------------ENACEKI--WDSFKNKNESIKIDKVNV-------
--PCKQIWED----------SNKAKLVKTIGLNPKDAQWM--------------STGECQVIEVNKNKYQVY
YAYLKN--------DHSDINIINDTNGNYFSYYRNLGRYDM------FEDKFYPTKKAN-------------
-KNGVELSCP----GLGNFEKMKPILSSYINNKKIDIKRF--------SFEDLNK-----------------
------------------------
>AR_0101_24
------------------------MKRSLTLLLSTFISIQA-------------------------------
--------------------FAE---------------ENACEKI--WDSFKNKNESIKIDKVNV-------
--PCKQIWED----------SNKAKLVKTIGLNPKDAQWM--------------STGECQVIEVNKNKYQVY
YAYLKN--------DHSDINIINDTNGNYFSYYRNLGRYDM------FEDKFYPTKKAN-------------
-KNGVELSCP----GLGNFEKMKPILSSYINNKKIDIKRF--------SFEDLNK-----------------
------------------------
>AB031_25

------------------------MKVLKIVIGTYIL---------------------------------L
NSISLYAES-----------------------------TNECMKY--WSEKSAASLKV---LGEV-------
--ECENIWEYG----KSNVTQGRLKLLNQLKLKPNNNQW---------MS-----TGECSKVSYNKNNYYIY
RAYYKE--------DRDEIWIAYNDKGSFSY----------------FRKVSSPKKEGE-------------
-NSKVSLSCA----KNGEYDEARSVLNTYLKNNTSVSMSYYKVGDWYKGD----------------------
------------------------
>Ab04-mff_12
MSLSVFNTTQYEDLKDITSKIEEQCIQILNEYGQIIFLLDTTLNDSIFNGVIDNLSNKNIAYIPLAHK-KGI
AKSNLFFICIDGKKV-----FEQVKEDLLQNLSRNKNITNDCYFVHGFGVAMTRSLEELRDQIKAKLVISDQ
GKKILFRWYDPRVM-IYLDEIFKTEYVGGLLDGFKEWHF---------LH----PKGLFSFKSYDESRSIL-
--NLKKVNLE-QSIELDLIEIANNVFQQLSEFEQVDLIKIHPVEIHQNLKEAYYDFGIQNHLDLVTYGLYSQ
IIHKNFIQHP----MVTNVLYDHCVMNKMSFTDAMDYVTKDLYEQ---IAKELNG-----------------
------------------------
>BJAB0715_12
MSLSVFNTTQYEDLKDITSKIEEQCIQILNEYGQIIFLLDTTLNDSIFNGVIDNLSNKNIAYIPLAHK-KGI
AKSNLFFICIDGKKV-----FEQVKEDLLQNLSRNKNITNDCYFVHGFGVAMTRSLEELRDQIKAKLVISDQ
GKKILFRWYDPRVM-IYLDEIFKTEYVGGLLDGFKEWHF---------LH----PKGLFSFKSYDESRSIL-
--NLKKVNLE-QSIELDLIEIANNVFQQLSEFEQVDLIKIHPVEIHQNLKEAYYDFGIQNHLDLVTYGLYSQ
IIHKNFIQHP----MVTNVLYDHCVMNKMSFTDAMDYVTKDLYEQ---IAKELNG-----------------
------------------------
>LAC-4_12
MSLSVFNTTQYEDLKDITSKIEEQCIQILNEYGQIIFLLDTTLNDSIFNGVIDNLSNKNIAYIPLAHK-KGI
AKSNLFFICIDGKKV-----FEQVKEDLLQNLSRNKNITNDCYFVHGFGVAMTRSLEELRDQIKAKLVISDQ
GKKILFRWYDPRVM-IYLDEIFKTEYVGGLLDGFKEWHF---------LH----PKGLFSFKSYDESRSIL-
--NLKKVNLE-QSIELDLIEIANNVFQQLSEFEQVDLIKIHPVEIHQNLKEAYYDFGIQNHLDLVTYGLYSQ
IIHKNFIQHP----MVTNVLYDHCVMNKMSFTDAMDYVTKDLYEQ---IAKELNG-----------------
------------------------
>WKA02_12
MSLSVFNTTQYEDLKDITSKIEEQCIQILNEYGQIIFLLDTTLNDSIFNGVIDNLSNKNIAYIPLAHK-KGI
AKSNLFFICIDGKKV-----FEQVKEDLLQNLSRNKNITNDCYFVHGFGVAMTRSLEELRDQIKAKLVISDQ
GKKILFRWYDPRVM-IYLDEIFKTEYVGGLLDGFKEWHF---------LH----PKGLFSFKSYDESRSIL-
--NLKKVNLE-QSIELDLIEIANNVFQQLSEFEQVDLIKIHPVEIHQNLKEAYYDFGIQNHLDLVTYGLYSQ
IIHKNFIQHP----MVTNVLYDHCVMNKMSFTDAMDYVTKDLYEQ---IAKELNG-----------------

------------------------
>XH858_12
MSLSVFNTTQYEDLKDITSKIEEQCIQILNEYGQIIFLLDTTLNDSIFNGVIDNLSNKNIAYIPLAHK-KGI
AKSNLFFICIDGKKV-----FEQVKEDLLQNLSRNKNITNDCYFVHGFGVAMTRSLEELRDQIKAKLVISDQ
GKKILFRWYDPRVM-IYLDEIFKTEYVGGLLDGFKEWHF---------LH----PKGLFSFKSYDESRSIL-
--NLKKVNLE-QSIELDLIEIANNVFQQLSEFEQVDLIKIHPVEIHQNLKEAYYDFGIQNHLDLVTYGLYSQ
IIHKNFIQHP----MVTNVLYDHCVMNKMSFTDAMDYVTKDLYEQ---IAKELNG-----------------
------------------------
>AR_0088_12
MSLSVFNTTQYEDLKDITSKIEEQCIQILNEYGQIIFLLDTTLNDSIFNGVIDNLSNKNIAYIPLAHK-KGI
AKSNLFFICIDGKKV-----FEQVKEDLLQNLSRNKNITNDCYFVHGFGVAMTRSLEELRDQIKAKLVISDQ
GKKILFRWYDPRVM-IYLDEIFKTEYVGGLLNGFKEWHF---------LH----PKGLFSFKSYDESRSIL-
--NLKKVNLE-QSIELDLIEIANNVFQQLSEFEQVDLIKIHPVEIHQNLKEAYYDFGIQNHLDLVTYGLYSQ
IIHKNFIQHP----MVTNVLYDHCVMNKMSFTDAMDYVTKDLYEQ---IAKELNG-----------------
------------------------
>CIP70.10_12
MSLSVFNTTQYEDLKDITSKIEEQCIQILNEYGQIIFLLDTTLNDSIFNGVIDNLSNKNIAYIPLAHK-KGI
AKSNLFFICIDGKKV-----FEQVKEDLLQNLSRNKNITNDCYFVHGFGVAMTRSLEELRDQIKAKLVISDQ
GKKILFRWYDPRVM-IYLDEIFKTEYVGGLLNGFKEWHF---------LH----PKGLFSFKSYDESRSIL-
--NLKKVNLE-QSIELDLIEIANNVFQQLSEFEQVDLIKIHPVEIHQNLKEAYYDFGIQNHLDLVTYGLYSQ
IIHKNFIQHP----MVTNVLYDHCVMNKMSFTDAMDYVTKDLYEQ---IAKELNG-----------------
------------------------
>HWBA8_12
MSLSVFNTTQYEDLKDITSKIEEQCIQILNEYGQIIFLLDTTLNDSIFNGVIDNLSNKNIAYIPLAHK-KGI
AKSNLFFICIDGKKV-----FEQVKEDLLQNLSRNKNITNDCYFVHGFGVAMTRSLEELRDQIKAKLVISDQ
GKKILFRWYDPRVM-IYLDEIFKTEYVGGLLNGFKEWHF---------LH----PKGLFSFKSYDESRSIL-
--NLKKVNLE-QSIELDLIEIANNVFQQLSEFEQVDLIKIHPVEIHQNLKEAYYDFGIQNHLDLVTYGLYSQ
IIHKNFIQHP----MVTNVLYDHCVMNKMSFTDAMDYVTKDLYEQ---IAKELNG-----------------
------------------------
>R2091_12
MSLSVFNTTQYEDLKDITSKIEEQCIQILNEYGQIIFLLDTTLNDSIFNGVIDNLSNKNIAYIPLAHK-KGI
AKSNLFFICIDGKKV-----FEQVKEDLLQNLSRNKNITNDCYFVHGFGVAMTRSLEELRDQIKAKLVISDQ
GKKILFRWYDPRVM-IYLDEIFKTEYVGGLLNGFKEWHF---------LH----PKGLFSFKSYDESRSIL-

--NLKKVNLE-QSIELDLIEIANNVFQQLSEFEQVDLIKIHPVEIHQNLKEAYYDFGIQNHLDLVTYGLYSQ
IIHKNFIQHP----MVTNVLYDHCVMNKMSFTDAMDYVTKDLYEQ---IAKELNG-----------------
------------------------
>D1279779_12
MSLSVFNTTQYEDLKDITSKIEEQCIQILNEYGQIIFLLDTTLNDSIFNGVIDNLSNKNIAYIPLAHK-KEI
AKSNLFFICIDDKKV-----FEQIKEDLLQNLSRNKNITNDCYFVHGFGVAKTRSLEELRDQIKTKLVISDQ
GKKILFRWYDPRVM-IYLDEIFKTEYVGGLLNGFKEWHF---------LH----PKGLFSFKSYDESQSIL-
--NLTKVNLE-QSIELDLIEIANNVFQQLNEFEQVDLIKIHPVEIHQNLKEAYYDFGIQNHLDLVTYGLYSQ
IIHKNFIQHP----MVTNVLYDHCVMNKMSFTDAMNYVTKDLYEQ---IAKELNG-----------------
------------------------
>R2090_12
MSLSVFNTTQYEDLKDITSKIEEQCIQILNEYGQIIFLLDTTLNDSIFNGVIDNLSNKNIAYIPLAHK-KEI
AKSNLFFICIDDKKV-----FEQIKEDLLQNLSRNKNITNDCYFVHGFGVAKTRSLEELRDQIKTKLVISDQ
GKKILFRWYDPRVM-IYLDEIFKTEYVGGLLNGFKEWHF---------LH----PKGLFSFKSYDESQSIL-
--NLTKVNLE-QSIELDLIEIANNVFQQLNEFEQVDLIKIHPVEIHQNLKEAYYDFGIQNHLDLVTYGLYSQ
IIHKNFIQHP----MVTNVLYDHCVMNKMSFTDAMNYVTKDLYEQ---IAKELNG-----------------
------------------------
>ZW85-1_20
MSLLVFNTTQYEDLKDITSKIEEQCIQILNEYGQIIFLLDTTLNDSIFNGVIDNLSNKNIAYIPLAHK-KEI
AKSNLFFICIDDKKV-----FEQIKEDLLQNLSRNKNITNDCYFVHGFGVAKTRSLEELRDQIKTKLVISDQ
GKKILFRWYDPRVM-IYLDEIFKTEYVGGLLNGFKEWHF---------LH----PKGLFSFKSYDESQSIL-
--NLTKVNLE-QSIELDLIEIANNVFQQLNEFEQVDLIKIHPVEIHQNLKEAYYDFGIQNHLDLVTYGLYSQ
IIHKNFIQHP----MVINVLYDHCVMNKMSFTDAMNYVTKDLYEQ---IAKELNG-----------------
------------------------
>Ab04-mff_11
----------------------MNMIEILQSKRSQFYLLDINS------LIILALAFCGVIISSTVYA---K
DIFNVELKCKGNDQE-----YCDIVK------------NSDNKTT--FILRNMRYPEVDKVNENIFHAYGSC
GSPCQYHFFI-----SKIQEDQTKEFIAL--DKKNN-----------------------CLIESDSKKNIIY
ARKLFN---------KNKKIIANLKNKEFNNVPIDSAIYNS------FQEKSYFDDKGQLH-----------
-LVAMLADVD----KNGNSLYFNKIIKKT--------------------CE---------------------
------------------------
>BJAB0715_11
----------------------MNMIEILQSKRSQFYLLDINS------LIILALAFCGVIISSTVYA---K

DIFNVELKCKGNDQE-----YCDIVK------------NSDNKTT--FILRNMRYPEVDKVNENIFHAYGSC
GSPCQYHFFI-----SKIQEDQTKEFIAL--DKKNN-----------------------CLIESDSKKNIIY
ARKLFN---------KNKKIIANLKNKEFNNVPIDSAIYNS------FQEKSYFDDKGQLH-----------
-LVAMLADVD----KNGNSLYFNKIIKKT--------------------CE---------------------
------------------------
>WKA02_11
----------------------MNMIEILQSKRSQFYLLDINS------LIILALAFCGVIISSTVYA---K
DIFNVELKCKGNDQE-----YCDIVK------------NSDNKTT--FILRNMRYPEVDKVNENIFHAYGSC
GSPCQYHFFI-----SKIQEDQTKEFIAL--DKKNN-----------------------CLIESDSKKNIIY
ARKLFN---------KNKKIIANLKNKEFNNVPIDSAIYNS------FQEKSYFDDKGQLH-----------
-LVAMLADVD----KNGNSLYFNKIIKKT--------------------CE---------------------
------------------------
>XH858_11
----------------------MNMIEILQSKRSQFYLLDINS------LIILALAFCGVIISSTVYA---K
DIFNVELKCKGNDQE-----YCDIVK------------NSDNKTT--FILRNMRYPEVDKVNENIFHAYGSC
GSPCQYHFFI-----SKIQEDQTKEFIAL--DKKNN-----------------------CLIESDSKKNIIY
ARKLFN---------KNKKIIANLKNKEFNNVPIDSAIYNS------FQEKSYFDDKGQLH-----------
-LVAMLADVD----KNGNSLYFNKIIKKT--------------------CE---------------------
------------------------
>ab736_9
-------------------------MSGANIVVPKISLPTIKTP----------------------------
--------------------F-----------------SNKINYN--WNINSEGKKELFIINKKN-------
--NILIRSHK-----NELDKNNNLSSLRFYTPEEVD----------------------FTAMIFNSD-YIQL
KQNIPD-----------SENIDEL-----------------------FEETLLYDEEND-------------
--------------DVYTEEEF--------------------------------------------------
------------------------
>ATCC19606_9
-------------------------MSGANIVVPKISLPTIKTP----------------------------
--------------------F-----------------SNKINYN--WNINSEGKKELFIINKKN-------
--NILIRSHK-----NELDKNNNLSSLRFYTPEEVD----------------------FTAMIFNSD-YIQL
KQNIPD-----------SENIDEL-----------------------FEETLLYDEEND-------------
--------------DVYTEEEF--------------------------------------------------
------------------------

>ab736_10
----------------------MEIFTQSADFLENIFAPTEEIDESFYYFLIDSSSFSGVDSFCKDFPFDGG
QKINLYLDFSENLQENGALLYSFTHEECIKNIELIKNIRDQGGLN--FFNSYFEIEQIKEHLEELMEIKQPN
GKSALFRFQDSFAFHATVSVLNELKWRKILSFKINYWIWQNIDNTFHRLDNFPTNRSILTTLSFSEDEFKDI
NINLKPMRLM-PLLKEYDENLKDLPFYQLYEIAINLLKEAKKQQLISFEDEILFSTLYH-------------
-KFGEFLLAE----GPFKKALNKTQMTAMSFQKTTDEIDLEELDDWYKRYQEVQV-----------------
------------------------
>ATCC19606_10
----------------------MEIFTQSADFLENIFAPTEEIDESFYYFLIDSSSFSGVDSFCKDFPFDGG
QKINLYLDFSENLQENGALLYSFTHEECIKNIELIKNIRDQGGLN--FFNSYFEIEQIKEHLEELMEIKQPN
GKSALFRFQDSFAFHATVSVLNELKWRKILSFKINYWIWQNIDNTFHRLDNFPTNRSILTTLSFSEDEFKDI
NINLKPMRLM-PLLKEYDENLKDLPFYQLYEIAINLLKEAKKQQLISFEDEILFSTLYH-------------
-KFGEFLLAE----GPFKKALNKTQMTAMSFQKTTDEIDLEELDDWYKRYQEVQV-----------------

**Supplementary file S4e**
>6200_21
---------MSIDNLEYFLD---KELLLPLKVP-----------SNWYISKNYL---------------YQV
SC-----NWLNQ------------------------------------------------------------
---------------------------LNDEDKFK------------MSEIFLYKNIFYAKLE---------
RIINNLTYSFVIDISIY-----PEIEEGLYTKFEYE--IGVGLYEISKKNKLIFMRNF--NFYN-IVEVCE-
-------FLNIILIDVCHSL------------------------------------GESI-----SEID---
IFKNVD---NFFEKNK--------------------------------------------------------
---------------------------
>KAB01_8
------------MRSKIFIS----VILFGLSAC------AAKADQNQLKDRELD----KLVACNGGNTEYTN
ID-----GYFTI----------------PDFGC---PYDGKNNKIGNA---MVYLIAPDHNNDYIQLNNMKN
IKGKYIYIVPFKFLNQD-----------KKSGSFY----------IAKDNYSYEVYFNEGGKW---------
VRKPDIIVSNNKVSVLS-----EINGFLNDRAKSFS--KKTEKKLKKYKMDINHDGVE--DFIE-VVGKDNK
P------SSIHILNGLDNSL-------IYKNDKIF--------NNSFNECNYSSFDNISI-----SKNN---
FTLEYS---TCADSSQIGYRYATFKTDKNKEPILIQDNYLVS------------------------PREETS
DNFKPKKVNCLTSSKITFNQFNGRCG-
>ACICU_8
------------MRSKIFIS----VILFGLSAC------AAKADQNQLKDRELD----KLVACNGGNTEYTN

ID-----GYFTI----------------PDFGC---PYDGKNNKIGNA---MVYLIAPDHNNDYIQLNNMKN
IKGKYIYIVPFKFLNQD-----------KKSGSFY----------IAKDNYSYEVYFNEGGKW---------
VRKPDIIVSNNKVSVLS-----EINGFLNDRAKSFS--KKTEKKLKKYKMDINHDGVE--DFIE-VVGKDNK
P------SSIHILNGLDNSL-------IYKNDKIF--------NNSFNECNYSSFDNISI-----SKNN---
FTLEYS---TCADSSQIGYRYATFKTDKNKEPILIQDNYLVS------------------------PREETS
DNFKPKKVNCLTSSKITFNQFNGRCG-
>A1296_8
------------MRSKIFIS----VILFGLSAC------AAKADQNQLKDRELD----KLVACNGGNTEYTN
ID-----GYFTI----------------PDFGC---PYDGKNNKIGNA---MVYLIAPDHNNDYIQLNNMKN
IKGKYIYIVPFKFLNQD-----------KKSGSFY----------IAKDNYSYEVYFNEGGKW---------
VRKPDIIVSNNKVSVLS-----EINGFLNDRAKSFS--KKTEKKLKKYKMDINHDGVE--DFIE-VVGKDNK
P------SSIHILNGLDNSL-------IYKNDKIF--------NNSFNECNYSSFDNISI-----SKNN---
FTLEYS---TCADSSQIGYRYATFKTDKNKEPILIQDNYLVS------------------------PREETS
DNFKPKKVNCLTSSKITFNQFNGRCG-
>AbPK1_8
------------MRSKIFIS----VILFGLSAC------AAKADQNQLKDRELD----KLVACNGGNTEYTN
ID-----GYFTI----------------PDFGC---PYDGKNNKIGNA---MVYLIAPDHNNDYIQLNNMKN
IKGKYIYIVPFKFLNQD-----------KKSGSFY----------IAKDNYSYEVYFNEGGKW---------
VRKPDIIVSNNKVSVLS-----EINGFLNDRAKSFS--KKTEKKLKKYKMDINHDGVE--DFIE-VVGKDNK
P------SSIHILNGLDNSL-------IYKNDKIF--------NNSFNECNYSSFDNISI-----SKNN---
FTLEYS---TCADSSQIGYRYATFKTDKNKEPILIQDNYLVS------------------------PREETS
DNFKPKKVNCLTSSKITFNQFNGRCG-
>AR_0056_8
------------MRSKIFIS----VILFGLSAC------AAKADQNQLKDRELD----KLVACNGGNTEYTN
ID-----GYFTI----------------PDFGC---PYDGKNNKIGNA---MVYLIAPDHNNDYIQLNNMKN
IKGKYIYIVPFKFLNQD-----------KKSGSFY----------IAKDNYSYEVYFNEGGKW---------
VRKPDIIVSNNKVSVLS-----EINGFLNDRAKSFS--KKTEKKLKKYKMDINHDGVE--DFIE-VVGKDNK
P------SSIHILNGLDNSL-------IYKNDKIF--------NNSFNECNYSSFDNISI-----SKNN---
FTLEYS---TCADSSQIGYRYATFKTDKNKEPILIQDNYLVS------------------------PREETS
DNFKPKKVNCLTSSKITFNQFNGRCG-
>KAB03_8
------------MRSKIFIS----VILFGLSAC------AAKADQNQLKDRELD----KLVACNGGNTEYTN
ID-----GYFTI----------------PDFGC---PYDGKNNKIGNA---MVYLIAPDHNNDYIQLNNMKN

IKGKYIYIVPFKFLNQD-----------KKSGSFY----------IAKDNYSYEVYFNEGGKW---------
VRKPDIIVSNNKVSVLS-----EINGFLNDRAKSFS--KKTEKKLKKYKMDINHDGVE--DFIE-VVGKDNK
P------SSIHILNGLDNSL-------IYKNDKIF--------NNSFNECNYSSFDNISI-----SKNN---
FTLEYS---TCADSSQIGYRYATFKTDKNKEPILIQDNYLVS------------------------PREETS
DNFKPKKVNCLTSSKITFNQFNGRCG-
>1656-2_8
------------MRSKIFIS----VILFGLSAC------AAKADQNQLKDRELD----KLVACNGGNTEYTN
ID-----GYFTI----------------PDFGC---PYDGKNNKIGNA---MVYLIAPDHNNDYIQLNNMKN
IKGKYIYIVPFKFLNQD-----------KKSGSFY----------IAKDNYSYEVYFNEGGKW---------
VRKPDIIVSNNKVSVLS-----EINGFLNDRAKSFS--KKTEKKLKKYKMDINHDGVE--DFIE-VVGKDNK
P------SSIHILNGLDNSL-------IYKNDKIF--------NNSFNECNYSSFDNISI-----SKNN---
FTLEYS---TCADSSQIGYRYATFKTDKNKEPILIQDNYLVS------------------------PREETS
DNFKPKKVNCLTSSKITFNQFNGRCG-
>DU202_8
------------MRSKIFIS----VILFGLSAC------AAKADQNQLKDRELD----KLVACNGGNTEYTN
ID-----GYFTI----------------PDFGC---PYDGKNNKIGNA---MVYLIAPDHNNDYIQLNNMKN
IKGKYIYIVPFKFLNQD-----------KKSGSFY----------IAKDNYSYEVYFNEGGKW---------
VRKPDIIVSNNKVSVLS-----EINGFLNDRAKSFS--KKTEKKLKKYKMDINHDGVE--DFIE-VVGKDNK
P------SSIHILNGLDNSL-------IYKNDKIF--------NNSFNECNYSSFDNISI-----SKNN---
FTLEYS---TCADSSQIGYRYATFKTDKNKEPILIQDNYLVS------------------------PREETS
DNFKPKKVNCLTSSKITFNQFNGRCG-
>R2090_8
------------MRSKIFIS----VILFGLSAC------AAKADQNQLKDRELD----KLVACNGGNTEYTN
ID-----GYFTI----------------PDFGC---PYDGKNNKIGNA---MVYLIAPDHNNDYIQLNNMKN
IKGKYIYIVPFKFLNQD-----------KKSGSFY----------IAKDNYSYEVYFNEGGKW---------
VRKPDIIVSNNKISVLS-----EINSFLNDRAKSFS--KKTEKKLKKYKMDINHDGVE--DFIE-VVGKDNK
P------SSIHILNGLDNSL-------IYKNDKIF--------NNSFNECDYSSFDNISI-----SKNN---
FTLEYS---TCADSSQIGYRYATFKTDKNKEPILIQDNYLVS------------------------PREETS
DNFKPKKVNCLTSSKITFNQFNGRCG-
>6200_15
----------MQTWNKAFFK---FMTIILMGTC--------CIACDLRKKNEVT----V----------IEN
KN-----IIIKK----------------FESDA----EYIFGNQ----------------------------
---------------------------ILGKKIFS--------------------SYNAERLI---------

FQVKDLEQNSDNFIDKI----------------------DGRLNKRGWNYKEKYKEAY--IYCD---RDMN-
-------QLELVPPIKIGTV--------------------MQSGEGQSLNQLVDYWNIGF-----IHSR---
HKRYVC---NMNS-----------------------------------------------------------
---------------------------
>WKA02_15
----------MQTWNKAFFK---FMTIILMGTC--------CIACDLRKKNEVT----V----------VEN
KN-----IIIKK----------------FESDA----EYIFGNQ----------------------------
---------------------------ILGKKIFS--------------------SYNAERLI---------
FQVKDLEQNSDNFIDKI----------------------DGRLNKRGWNYKEKYKEAY--IYCD---RDMN-
-------QLELVPPIKIGTV--------------------MQSGEGQSLNQLVDYWNIGF-----IHSR---
HKRYVC---NMNS-----------------------------------------------------------
---------------------------
>AR_0083_15
----------MQTWNKAFFK---FMTIILMGTC--------CIACDLRKKNEVT----V----------VEN
KN-----IIIKK----------------FESDA----EYIFGNQ----------------------------
---------------------------ILGKKIFS--------------------SYNAERLI---------
FQVKDLEQNSDNFIDKI----------------------DGRLNKRGWNYKEKYKEAY--IYCD---RDMN-
-------QLELVPPIKIGTV--------------------MQSGEGQSLNQLVDYWNIGF-----IHSR---
HKRYVC---NMNS-----------------------------------------------------------
---------------------------
>USA15_15
----------MQTWNKAFFK---FMTIILMGTC--------CIACDLRKKNEVT----V----------VEN
KN-----IIIKK----------------FESDA----EYIFGNQ----------------------------
---------------------------ILGKKIFS--------------------SYNAERLI---------
FQVKDLEQNSDNFIDKI----------------------DGRLNKRGWNYKEKYKEAY--IYCD---RDMN-
-------QLELVPPIKIGTV--------------------MQSGEGQSLNQLVDYWNIGF-----IHSR---
HKRYVC---NMNS-----------------------------------------------------------
---------------------------
>IOMTU433_15
----------MQTWNKAFFK---FMTIILMGTC--------CIACDLRKKNEVT----V----------VEN
KN-----IIIKK----------------FESDA----EYIFGNQ----------------------------
---------------------------ILGKKIFS--------------------SYNAERLI---------
FQVKDLEQNSDNFIDKI----------------------DGRLNKRGWNYKEKYKEAY--IYCD---RDMN-

-------QLELVPPIKIGTV--------------------MQSGEGQSLNQLVDYWNIGF-----IHSR---
HKRYVC---NMNS-----------------------------------------------------------
---------------------------
>A1_15
----------MQTWNKAFFK---FMTIILMGTC--------CIACDLRKKNEVT----V----------VEN
KN-----IIIKK----------------FESDA----EYIFGNQ----------------------------
---------------------------ILGKKIFS--------------------SYNAERLI---------
FQVKDLEQNSDNFIDKI----------------------DGRLNKRGWNYKEKYKEAY--IYCD---RDMN-
-------QLELVPPIKIGTV--------------------MQSGEGQSLNQLVDYWNIGF-----IHSR---
HKRYVC---NMNS-----------------------------------------------------------
---------------------------
>A388_15
----------MQTWNKAFFK---FMTIILMGTC--------CIACDLRKKNEVT----V----------VEN
KN-----IIIKK----------------FESDA----EYIFGNQ----------------------------
---------------------------ILGKKIFS--------------------SYNAERLI---------
FQVKDLEQNSDNFIDKI----------------------DGRLNKRGWNYKEKYKEAY--IYCD---RDMN-
-------QLELVPPIKIGTV--------------------MQSGEGQSLNQLVDYWNIGF-----IHSR---
HKRYVC---NMNS-----------------------------------------------------------
---------------------------
>A85_15
----------MQTWNKAFFK---FMTIILMGTC--------CIACDLRKKNEVT----V----------VEN
KN-----IIIKK----------------FESDA----EYIFGNQ----------------------------
---------------------------ILGKKIFS--------------------SYNAERLI---------
FQVKDLEQNSDNFIDKI----------------------DGRLNKRGWNYKEKYKEAY--IYCD---RDMN-
-------QLELVPPIKIGTV--------------------MQSGEGQSLNQLVDYWNIGF-----IHSR---
HKRYVC---NMNS-----------------------------------------------------------
---------------------------
>AB0057_15
----------MQTWNKAFFK---FMTIILMGTC--------CIACDLRKKNEVT----V----------VEN
KN-----IIIKK----------------FESDA----EYIFGNQ----------------------------
---------------------------ILGKKIFS--------------------SYNAERLI---------
FQVKDLEQNSDNFIDKI----------------------DGRLNKRGWNYKEKYKEAY--IYCD---RDMN-
-------QLELVPPIKIGTV--------------------MQSGEGQSLNQLVDYWNIGF-----IHSR---

HKRYVC---NMNS-----------------------------------------------------------
---------------------------
>AB307-0294_15
----------MQTWNKAFFK---FMTIILMGTC--------CIACDLRKKNEVT----V----------VEN
KN-----IIIKK----------------FESDA----EYIFGNQ----------------------------
---------------------------ILGKKIFS--------------------SYNAERLI---------
FQVKDLEQNSDNFIDKI----------------------DGRLNKRGWNYKEKYKEAY--IYCD---RDMN-
-------QLELVPPIKIGTV--------------------MQSGEGQSLNQLVDYWNIGF-----IHSR---
HKRYVC---NMNS-----------------------------------------------------------
---------------------------
>AB5075-UW_15
----------MQTWNKAFFK---FMTIILMGTC--------CIACDLRKKNEVT----V----------VEN
KN-----IIIKK----------------FESDA----EYIFGNQ----------------------------
---------------------------ILGKKIFS--------------------SYNAERLI---------
FQVKDLEQNSDNFIDKI----------------------DGRLNKRGWNYKEKYKEAY--IYCD---RDMN-
-------QLELVPPIKIGTV--------------------MQSGEGQSLNQLVDYWNIGF-----IHSR---
HKRYVC---NMNS-----------------------------------------------------------
---------------------------
>AR_0078_15
----------MQTWNKAFFK---FMTIILMGTC--------CIACDLRKKNEVT----V----------VEN
KN-----IIIKK----------------FESDA----EYIFGNQ----------------------------
---------------------------ILGKKIFS--------------------SYNAERLI---------
FQVKDLEQNSDNFIDKI----------------------DGRLNKRGWNYKEKYKEAY--IYCD---RDMN-
-------QLELVPPIKIGTV--------------------MQSGEGQSLNQLVDYWNIGF-----IHSR---
HKRYVC---NMNS-----------------------------------------------------------
---------------------------
>D36_15
----------MQTWNKAFFK---FMTIILMGTC--------CIACDLRKKNEVT----V----------VEN
KN-----IIIKK----------------FESDA----EYIFGNQ----------------------------
---------------------------ILGKKIFS--------------------SYNAERLI---------
FQVKDLEQNSDNFIDKI----------------------DGRLNKRGWNYKEKYKEAY--IYCD---RDMN-
-------QLELVPPIKIGTV--------------------MQSGEGQSLNQLVDYWNIGF-----IHSR---
HKRYVC---NMNS-----------------------------------------------------------

```
---------------------------
>SDF_15
----------MQTWNKAFFK---FMTIILMGTC--------CIACDLRKKNEVT----V----------VEN
KN-----IIIKK----------------FESDA----EYIFGNQ----------------------------
---------------------------ILGKKIFS--------------------SYNAERLI---------
FQVKDLEQNSDNFIDKI----------------------DGRLNKRGWNYKEKYKEAY--IYCD---RDMN-
-------QLELVPPIKIGTV--------------------MQSGEGQSLNQLVDYWNIGF-----IHSR---
HKRYVC---NMNS-----------------------------------------------------------
---------------------------
>WCHAB005078_15
----------MQTWNKAFFK---FMTIILMGTC--------CIACDLRKKNEVT----V----------VEN
KN-----IIIKK----------------FESDA----EYIFGNQ----------------------------
---------------------------ILGKKIFS--------------------SYNAERLI---------
FQVKDLEQNSDNFIDKI----------------------DGRLNKRGWNYKEKYKEAY--IYCD---RDMN-
-------QLELVPPIKIGTV--------------------MQSGEGQSLNQLVDYWNIGF-----IHSR---
HKRYVC---NMNS-----------------------------------------------------------
---------------------------
>AB030_15
----------MQTWNKAFFK---FMTIILMGTC--------CIACDLRKKNEVT----V----------VEN
KN-----IIIKK----------------FESDA----EYIFGNQ----------------------------
---------------------------ILGKKIFS--------------------SYNAERLI---------
FQVKDLEQNSDNFINKI----------------------DGRLNKRGWNYKEKYKEAY--IYCD---RDMN-
-------QLELVPPIKIGTV--------------------MQSGEGQSLNQLVDYWNIGF-----IHSR---
HKRYVC---NMNS-----------------------------------------------------------
---------------------------
>AbH12O-A2_15
----------MQTWNKAFFK---FMTIILMGTC--------CIACDLRKKNEVT----V----------VEN
KN-----IIIKK----------------FESDA----EYIFGNQ----------------------------
---------------------------ILGKKIFS--------------------SYNAERLI---------
FQVKDLEQNSDNFINKI----------------------DGRLNKRGWNYKEKYKEAY--IYCD---RDMN-
-------QLELVPPIKIGTV--------------------MQSGEGQSLNQLVDYWNIGF-----IHSR---
HKRYVC---NMNS-----------------------------------------------------------
---------------------------
```

```
>AF-401_15
----------MQTWNKAFFK---FMTIILMGTC--------CIACDLRKKNEVT----V----------VEN
KN-----IIIKK----------------FESDA----EYIFGNQ----------------------------
---------------------------ILGKKIFS--------------------SYNAERLI---------
FQVKDLEQNSDNFINKI----------------------DGRLNKRGWNYKEKYKEAY--IYCD---RDMN-
-------QLELVPPIKIGTV--------------------MQSGEGQSLNQLVDYWNIGF-----IHSR---
HKRYVC---NMNS-----------------------------------------------------------
---------------------------
>AR_0063_15
----------MQTWNKAFFK---FMTIILMGTC--------CIACDLRKKNEVT----V----------VEN
KN-----IIIKK----------------FESDA----EYIFGNQ----------------------------
---------------------------ILGKKIFS--------------------SYNAERLI---------
FQVKDLEQNSDNFINKI----------------------DGRLNKRGWNYKEKYKEAY--IYCD---RDMN-
-------QLELVPPIKIGTV--------------------MQSGEGQSLNQLVDYWNIGF-----IHSR---
HKRYVC---NMNS-----------------------------------------------------------
---------------------------
>AR_0101_15
----------MQTWNKAFFK---FMTIILMGTC--------CIACDLRKKNEVT----V----------VEN
KN-----IIIKK----------------FESDA----EYIFGNQ----------------------------
---------------------------ILGKKIFS--------------------SYNAERLI---------
FQVKDLEQNSDNFINKI----------------------DGRLNKRGWNYKEKYKEAY--IYCD---RDMN-
-------QLELVPPIKIGTV--------------------MQSGEGQSLNQLVDYWNIGF-----IHSR---
HKRYVC---NMNS-----------------------------------------------------------
---------------------------
>AB030_24
-------------MNKILIA----LSFLFLTAC-----------NDFNKLEKES------------------
NA-----SIEHQ------------------------------------------------------------
---------------------------NESNSVIK-------------------------------------
---SQLSNSNDSLIKET-----TVSQVKDKKIENSK--PQMDAQDKITCMGKTLNDWY--TFDE--------
-------KLEQPKCKKLNGY-----------------------KLKNYQCSVEKNVFDLN-----SDAI---
RITMKD---TRVFAFHSLEQCQEAVEMWEANGI---------------------------------------
---------------------------
>AbH12O-A2_24
```

-------------MNKILIA----LSFLFLTAC-----------NDFNKLEKES------------------
NA-----SIEHQ------------------------------------------------------------
---------------------------NESNSVIK-------------------------------------
---SQLSNSNDSLIKET-----TVSQVKDKKIENSK--PQMDAQDKITCMGKTLNDWY--TFDE--------
-------KLEQPKCKKLNGY-----------------------KLKNYQCSVEKNVFDLN-----SDAI---
RITMKD---TRVFAFHSLEQCQEAVEMWEANGI---------------------------------------
---------------------------
>AF-401_24
-------------MNKILIA----LSFLFLTAC-----------NDFNKLEKES------------------
NA-----SIEHQ------------------------------------------------------------
---------------------------NESNSVIK-------------------------------------
---SQLSNSNDSLIKET-----TVSQVKDKKIENSK--PQMDAQDKITCMGKTLNDWY--TFDE--------
-------KLEQPKCKKLNGY-----------------------KLKNYQCSVEKNVFDLN-----SDAI---
RITMKD---TRVFAFHSLEQCQEAVEMWEANGI---------------------------------------
---------------------------
>AR_0101_24
-------------MNKILIA----LSFLFLTAC-----------NDFNKLEKES------------------
NA-----SIEHQ------------------------------------------------------------
---------------------------NESNSVIK-------------------------------------
---SQLSNSNDSLIKET-----TVSQVKDKKIENSK--PQMDAQDKITCMGKTLNDWY--TFDE--------
-------KLEQPKCKKLNGY-----------------------KLKNYQCSVEKNVFDLN-----SDAI---
RITMKD---TRVFAFHSLEQCQEAVEMWEANGI---------------------------------------
---------------------------
>AR_0063_24
-------------MNKILIA----LSFLFLTAC-----------NDFNKLEKES------------------
NA-----SIEHQ------------------------------------------------------------
---------------------------NESNSVIK-------------------------------------
---SQLSNSNDSLIKET-----TVSQVKDKKIENSK--PQMDAQDKITCMGKTLNDWY--TFDE--------
-------KLEQPKCKKLNGY-----------------------KLKNYQCSVEKNVFDLN-----SDAI---
RITMKD---TRVFAFHSLEQCQEAVEMWEANGI---------------------------------------
---------------------------
>AB031_25
-----------MRFIFAFFI-------IFLSAC---------SHSNTIADKKSY---------------LDF

NS-----VNLKD--------------KSYIIGN---KYSDVN------------------------------
---------------------------FSSKGIAK---------------------DQIGSCT---------
-TFSDFNNSISYTLNNQ-----KLVEVLIGNENKSI--YTSKNIRNGMGVENIYKNYK--GYNI--------
-------KKMKS-------------------------------DGAGDSQDDYTYIVTDN-----LQKN---
NSLIFD---VVHGKIQGIHLGKKGFDLSDCE-----------------------------------------
---------------------------
>Ab04-mff_11
-------------MSRLF-----LLFVISLMYA--------CTTEADQPKSSNT----K----------LYF
SY-----RICEK----------------AGKCK---NFTPTKKMDNYLFKRLDPAYDEFYI-----------
---------------------------KDNNGNFT--------------LYYKNVYSEDVQVG---------
WAEAKADKNNSSELSLN-----IDKVNLALGGIDFI--KNGYATKQAESKNFKSGIYY--LADN--------
-------YTGAPYYQKQKIF--------FKK------------NAGDLVLDCNSYLNNPS-----VKNESYG
VFYGVC---SEGEKVYFKQVN---------------------------------------------------
---------------------------
>BJAB0715_11
-------------MSRLF-----LLFVISLMYA--------CTTEADQPKSSNT----K----------LYF
SY-----RICEK----------------AGKCK---NFTPTKKMDNYLFKRLDPAYDEFYI-----------
---------------------------KDNNGNFT--------------LYYKNVYSEDVQVG---------
WAEAKADKNNSSELSLN-----IDKVNLALGGIDFI--KNGYATKQAESKNFKSGIYY--LADN--------
-------YTGAPYYQKQKIF--------FKK------------NAGDLVLDCNSYLNNPS-----VKNESYG
VFYGVC---SEGEKVYFKQVN---------------------------------------------------
---------------------------
>WKA02_11
-------------MSRLF-----LLFVISLMYA--------CTTEADQPKSSNT----K----------LYF
SY-----RICEK----------------AGKCK---NFTPTKKMDNYLFKRLDPAYDEFYI-----------
---------------------------KDNNGNFT--------------LYYKNVYSEDVQVG---------
WAEAKADKNNSSELSLN-----IDKVNLALGGIDFI--KNGYATKQAESKNFKSGIYY--LADN--------
-------YTGAPYYQKQKIF--------FKK------------NAGDLVLDCNSYLNNPS-----VKNESYG
VFYGVC---SEGEKVYFKQVN---------------------------------------------------
---------------------------
>XH858_11
-------------MSRLF-----LLFVISLMYA--------CTTEADQPKSSNT----K----------LYF
SY-----RICEK----------------AGKCK---NFTPTKKMDNYLFKRLDPAYDEFYI-----------

---------------------------KDNNGNFT--------------LYYKNVYSEDVQVG---------
WAEAKADKNNSSELSLN-----IDKVNLALGGIDFI--KNGYATKQAESKNFKSGIYY--LADN--------
-------YTGAPYYQKQKIF--------FKK------------NAGDLVLDCNSYLNNPS-----VKNESYG
VFYGVC---SEGEKVYFKQVN---------------------------------------------------
---------------------------

>LAC-4_11
-------------MSRLF-----LLFVISLMYA--------CTTEADQPKSSNT----K----------LYF
SY-----RICEK----------------AGKCK---NFTPTKKMDNYLFKRLDPAYDEFYI-----------
---------------------------KDNNGNFT--------------LYYKNVYSEDVQVG---------
WAEAKADKNNSSELSLN-----IDKVNLALGGIDFI--KNGYATKQAESKNFKSGIYY--LADN--------
-------YTGAPYYQKQKIF--------FKK------------NAGDLVLDCNSYLNNPS-----VKNESYG
VFYGVC---SEGEKVYFKQVN---------------------------------------------------
---------------------------

>15A34_23
--------MMMKKLISLLVL---IIVAFPTVAR------------------------------------IPK
GD-----YIMID------------------------------------------------------------
----------------------------KKKGGFV-------------------------------------
RLCKSESTEELENIILT-----DKSLHLALPESTEY--LILKEKMNKNEHILEVRNPY--NIVY-TYKIKK-
-------INNSDVYKWDAYL-------------------------GNKRIEDLSFFSIAK-----NKAK---
NIISVG---ICK------------------------------------------------------------
---------------------------

>A1_17
--------------MKLLIL---EILCLSLLSG-----------CASAKEAEC----------------YNT
SK-----ALITD----------------LYNKF---P-----------------------------------
---------------------------INGDEVIE------------------------NKNI---------
KTLSNFFDKNLTNLLIK-----DQECSAR----------NNGVCNIDFNILIHSQDTP--NKYK--------
-----------ILQDTDNLV-----------------------TVKVEYHQYSEFINFVI-----NKES---
SCKKIGNIKYDDGSDLIKMLRK--------------------------------------------------
---------------------------

>A388_17
--------------MKLLIL---EILCLSLLSG-----------CASAKEAEC----------------YNT
SK-----ALITD----------------LYNKF---P-----------------------------------
---------------------------INGDEVIE------------------------NKNI---------

KTLSNFFDKNLTNLLIK-----DQECSAR----------NNGVCNIDFNILIHSQDTP--NKYK--------
-----------ILQDTDNLV-----------------------TVKVEYHQYSEFINFVI-----NKES---
SCKKIGNIKYDDGSDLIKMLRK--------------------------------------------------
---------------------------
>A85_17
--------------MKLLIL---EILCLSLLSG-----------CASAKEAEC----------------YNT
SK-----ALITD----------------LYNKF---P-----------------------------------
---------------------------INGDEVIE------------------------NKNI---------
KTLSNFFDKNLTNLLIK-----DQECSAR----------NNGVCNIDFNILIHSQDTP--NKYK--------
-----------ILQDTDNLV-----------------------TVKVEYHQYSEFINFVI-----NKES---
SCKKIGNIKYDDGSDLIKMLRK--------------------------------------------------
---------------------------
>AB307-0294_17
--------------MKLLIL---EILCLSLLSG-----------CASAKEAEC----------------YNT
SK-----ALITD----------------LYNKF---P-----------------------------------
---------------------------INGDEVIE------------------------NKNI---------
KTLSNFFDKNLTNLLIK-----DQECSAR----------NNGVCNIDFNILIHSQDTP--NKYK--------
-----------ILQDTDNLV-----------------------TVKVEYHQYSEFINFVI-----NKES---
SCKKIGNIKYDDGSDLIKMLRK--------------------------------------------------
---------------------------
>AB5075-UW_17
--------------MKLLIL---EILCLSLLSG-----------CASAKEAEC----------------YNT
SK-----ALITD----------------LYNKF---P-----------------------------------
---------------------------INGDEVIE------------------------NKNI---------
KTLSNFFDKNLTNLLIK-----DQECSAR----------NNGVCNIDFNILIHSQDTP--NKYK--------
-----------ILQDTDNLV-----------------------TVKVEYHQYSEFINFVI-----NKES---
SCKKIGNIKYDDGSDLIKMLRK--------------------------------------------------
---------------------------
>AR_0083_17
--------------MKLLIL---EILCLSLLSG-----------CASAKEAEC----------------YNT
SK-----ALITD----------------LYNKF---P-----------------------------------
---------------------------INGDEVIE------------------------NKNI---------
KTLSNFFDKNLTNLLIK-----DQECSAR----------NNGVCNIDFNILIHSQDTP--NKYK--------

-----------ILQDTDNLV-----------------------TVKVEYHQYSEFINFVI-----NKES---
SCKKIGNIKYDDGSDLIKMLRK--------------------------------------------------
---------------------------
>AYE_17
--------------MKLLIL---EILCLSLLSG-----------CASAKEAEC----------------YNT
SK-----ALITD----------------LYNKF---P-----------------------------------
---------------------------INGDEVIE------------------------NKNI---------
KTLSNFFDKNLTNLLIK-----DQECSAR----------NNGVCNIDFNILIHSQDTP--NKYK--------
-----------ILQDTDNLV-----------------------TVKVEYHQYSEFINFVI-----NKES---
SCKKIGNIKYDDGSDLIKMLRK--------------------------------------------------
---------------------------
>D36_17
--------------MKLLIL---EILCLSLLSG-----------CASAKEAEC----------------YNT
SK-----ALITD----------------LYNKF---P-----------------------------------
---------------------------INGDEVIE------------------------NKNI---------
KTLSNFFDKNLTNLLIK-----DQECSAR----------NNGVCNIDFNILIHSQDTP--NKYK--------
-----------ILQDTDNLV-----------------------TVKVEYHQYSEFINFVI-----NKES---
SCKKIGNIKYDDGSDLIKMLRK--------------------------------------------------
---------------------------
>WCHAB005078_17
--------------MKLLIL---EILCLSLLSG-----------CASAKEAEC----------------YNT
SK-----ALITD----------------LYNKF---P-----------------------------------
---------------------------INGDEVIE------------------------NKNI---------
KTLSNFFDKNLTNLLIK-----DQECSAR----------NNGVCNIDFNILIHSQDTP--NKYK--------
-----------ILQDTDNLV-----------------------TVKVEYHQYSEFINFVI-----NKES---
SCKKIGNIKYDDGSDLIKMLRK--------------------------------------------------
---------------------------
>6200_17
--------------MKLLIL---EILCLSLLSG-----------CASAKEAEC----------------YNT
SK-----ALITD----------------LYNKF---P-----------------------------------
---------------------------INGDEVIE------------------------NKNI---------
KTLSNFFDKNLTNLLIK-----DQECSAR----------NNGVCNIDFNILIHSQDTP--NKYK--------
-----------ILQDTDSLV-----------------------TVKVEYHQYSEFINFVI-----NKES---

SCKKIGNIKYDDGSDLIKMLRK--------------------------------------------------
---------------------------
>ZW85-1_17
--------------MKLLIL---EILCLSLLSG-----------CASAKEAEC----------------YNT
SK-----ALITD----------------LYNKF---P-----------------------------------
---------------------------INGDEVIE------------------------NKNI---------
KTLSNFFDKNLTNLLIK-----DQECSAR----------NNGVCNIDFNILIHSQDTP--NKYK--------
-----------ILQDTDSLV-----------------------TVKVEYHQYSEFINFVI-----NKES---
SCKKIGNIKYDDGSDLIKMLRK--------------------------------------------------
---------------------------
>ab736_9
-------------MKKYFTT--VLLIISSLIYS-----------QNIFANDQCN----I---------EETV
NL-----TIFKK------------------------------------------------------------
----------------------------NDYKFYN-------------------------------------
YMCDSVEGSYLKGYLGS-----QEKKIFVSDYSDFA--AKESPKLLAVSIYKSKKRKP--PILI--------
-------TLNSSYYCCTPQM-----------------------EGEMYQVNLYQISENKT-----LNLK---
NITNIL---GVNAEGFEGRAEGKVYYKYKNISEIKKWLDKNY------------------------------
---------------------------
>ATCC19606_9
-------------MKKYFTT--VLLIISSLIYS-----------QNIFANDQCN----I---------EETV
NL-----TIFKK------------------------------------------------------------
----------------------------NDYKFYN-------------------------------------
YMCDSVEGSYLKGYLGS-----QEKKIFVSDYSDFA--AKESPKLLAVSIYKSKKRKP--PILI--------
-------TLNSSYYCCTPQM-----------------------EGEMYQVNLYQISENKT-----LNLK---
NITNIL---GVNAEGFEGRAEGKVYYKYKNISEIKKWLDKNY------------------------------
---------------------------
>DU202_6
-----------MNFNDLLKK---IDECNEIIYG-----------ERFLINQDNS-----------------V
ND-----DLFKS----------------YLSCI---PENDFN------------------------------
---------------------------IDLGGNIN----------------DFLGKDSLENIQ---------
YMFEDIFDGESTTVFLT-----QHDVNVVYINHNSG---EIGACISGDELHIIAKNLF--DFFS-QINVIQ-
-------NLIIDLYDSNPRG-----------------------ENGGYKTGFLEELKKTL-----IKEN---
VVLDVD---YFLEFFYG-------------------------------------------------------

```
---------------------------
>TCDC-0715_6
-----------MNFNDLLKK---IDECNEIIYG-----------ERFLINQDNS-----------------V
ND-----DLFKS----------------YLSCI---PENDFN------------------------------
---------------------------IDLGGNIN----------------DFLGKDSLENIQ---------
YMFEDIFDGESTTVFLT-----QHDVNVVYINHNSG---EIGACISGDELHIIAKNLF--DFFS-QINVIQ-
-------NLIIDLYDSNPRG-----------------------ENGGYKTGFLEELKKTL-----IKEN---
VVLDVD---YFLEFFYG-------------------------------------------------------
---------------------------
>AF-673_6
-----------MNFNDLLKK---IDECNEIIYG-----------ERFLINQDNS-----------------V
ND-----DLFKS----------------YLSCI---PENDFN------------------------------
---------------------------IDLGGNIN----------------DFLGKDSLENIQ---------
YMFEDIFDGESTTVFLT-----QHDVNVVYINHNSG---EIGACISGDELHIIAKNLF--DFFS-QINVIQ-
-------NLIIDLYDSNPRG-----------------------ENGGYKTGFLEELKKTL-----IKEN---
VVLDVD---YFLEFFYG-------------------------------------------------------
---------------------------
>CMC-CR-MDR-Ab4_6
-----------MNFNDLLKK---IDECNEIIYG-----------ERFLINQDNS-----------------V
ND-----DLFKS----------------YLSCI---PENDFN------------------------------
---------------------------IDLGGNIN----------------DFLGKDSLENIQ---------
YMFEDIFDGESTTVFLT-----QHDVNVVYINHNSG---EIGACISGDELHIIAKNLF--DFFS-QINVIQ-
-------NLIIDLYDSNPRG-----------------------ENGGYKTGFLEELKKTL-----IKEN---
VVLDVD---YFLEFFYG-------------------------------------------------------
---------------------------
>CMC-CR-MDR-Ab66_6
-----------MNFNDLLKK---IDECNEIIYG-----------ERFLINQDNS-----------------V
ND-----DLFKS----------------YLSCI---PENDFN------------------------------
---------------------------IDLGGNIN----------------DFLGKDSLENIQ---------
YMFEDIFDGESTTVFLT-----QHDVNVVYINHNSG---EIGACISGDELHIIAKNLF--DFFS-QINVIQ-
-------NLIIDLYDSNPRG-----------------------ENGGYKTGFLEELKKTL-----IKEN---
VVLDVD---YFLEFFYG-------------------------------------------------------
---------------------------
```

>CMC-MDR-Ab59_6
-----------MNFNDLLKK---IDECNEIIYG-----------ERFLINQDNS-----------------V
ND-----DLFKS----------------YLSCI---PENDFN------------------------------
---------------------------IDLGGNIN----------------DFLGKDSLENIQ---------
YMFEDIFDGESTTVFLT-----QHDVNVVYINHNSG---EIGACISGDELHIIAKNLF--DFFS-QINVIQ-
-------NLIIDLYDSNPRG-----------------------ENGGYKTGFLEELKKTL-----IKEN---
VVLDVD---YFLEFFYG-------------------------------------------------------
---------------------------
>15A34_6
-----------MNFNDLLKK---IDECNEIIYG-----------ERFLINQDNS-----------------V
ND-----DLFKS----------------YLSCI---PENDFN------------------------------
---------------------------IDLGGNIN----------------DFLGKDSLENIQ---------
YMFEDIFDGESTTVFLT-----QHDVNVVYINHNSG---EIGACISGDELHIIAKNLF--DFFS-QINVIQ-
-------NLIIDLYDSNPRG-----------------------ENGGYKTGFLEELKKTL-----IKEN---
VVLDVD---YFLEFFYG-------------------------------------------------------
---------------------------
>15A5_6
-----------MNFNDLLKK---IDECNEIIYG-----------ERFLINQDNS-----------------V
ND-----DLFKS----------------YLSCI---PENDFN------------------------------
---------------------------IDLGGNIN----------------DFLGKDSLENIQ---------
YMFEDIFDGESTTVFLT-----QHDVNVVYINHNSG---EIGACISGDELHIIAKNLF--DFFS-QINVIQ-
-------NLIIDLYDSNPRG-----------------------ENGGYKTGFLEELKKTL-----IKEN---
VVLDVD---YFLEFFYG-------------------------------------------------------
---------------------------
>1656-2_6
-----------MNFNDLLKK---IDECNEIIYG-----------ERFLINQDNS-----------------V
ND-----DLFKS----------------YLSCI---PENDFN------------------------------
---------------------------IDLGGNIN----------------DFLGKDSLENIQ---------
YMFEDIFDGESTTVFLT-----QHDVNVVYINHNSG---EIGACISGDELHIIAKNLF--DFFS-QINVIQ-
-------NLIIDLYDSNPRG-----------------------ENGGYKTGFLEELKKTL-----IKEN---
VVLDVD---YFLEFFYG-------------------------------------------------------
---------------------------
>3027STDY5784958_6

-----------MNFNDLLKK---IDECNEIIYG-----------ERFLINQDNS-----------------V
ND-----DLFKS----------------YLSCI---PENDFN------------------------------
---------------------------IDLGGNIN----------------DFLGKDSLENIQ---------
YMFEDIFDGESTTVFLT-----QHDVNVVYINHNSG---EIGACISGDELHIIAKNLF--DFFS-QINVIQ-
-------NLIIDLYDSNPRG-----------------------ENGGYKTGFLEELKKTL-----IKEN---
VVLDVD---YFLEFFYG-------------------------------------------------------
---------------------------

>AB07_6
-----------MNFNDLLKK---IDECNEIIYG-----------ERFLINQDNS-----------------V
ND-----DLFKS----------------YLSCI---PENDFN------------------------------
---------------------------IDLGGNIN----------------DFLGKDSLENIQ---------
YMFEDIFDGESTTVFLT-----QHDVNVVYINHNSG---EIGACISGDELHIIAKNLF--DFFS-QINVIQ-
-------NLIIDLYDSNPRG-----------------------ENGGYKTGFLEELKKTL-----IKEN---
VVLDVD---YFLEFFYG-------------------------------------------------------
---------------------------

>AB34299_6
-----------MNFNDLLKK---IDECNEIIYG-----------ERFLINQDNS-----------------V
ND-----DLFKS----------------YLSCI---PENDFN------------------------------
---------------------------IDLGGNIN----------------DFLGKDSLENIQ---------
YMFEDIFDGESTTVFLT-----QHDVNVVYINHNSG---EIGACISGDELHIIAKNLF--DFFS-QINVIQ-
-------NLIIDLYDSNPRG-----------------------ENGGYKTGFLEELKKTL-----IKEN---
VVLDVD---YFLEFFYG-------------------------------------------------------
---------------------------

>Ab4568_6
-----------MNFNDLLKK---IDECNEIIYG-----------ERFLINQDNS-----------------V
ND-----DLFKS----------------YLSCI---PENDFN------------------------------
---------------------------IDLGGNIN----------------DFLGKDSLENIQ---------
YMFEDIFDGESTTVFLT-----QHDVNVVYINHNSG---EIGACISGDELHIIAKNLF--DFFS-QINVIQ-
-------NLIIDLYDSNPRG-----------------------ENGGYKTGFLEELKKTL-----IKEN---
VVLDVD---YFLEFFYG-------------------------------------------------------
---------------------------

>Ab4653_6
-----------MNFNDLLKK---IDECNEIIYG-----------ERFLINQDNS-----------------V

```
ND-----DLFKS----------------YLSCI---PENDFN------------------------------
---------------------------IDLGGNIN----------------DFLGKDSLENIQ---------
YMFEDIFDGESTTVFLT-----QHDVNVVYINHNSG---EIGACISGDELHIIAKNLF--DFFS-QINVIQ-
-------NLIIDLYDSNPRG-----------------------ENGGYKTGFLEELKKTL-----IKEN---
VVLDVD---YFLEFFYG-------------------------------------------------------
---------------------------
>Ab4977_6
-----------MNFNDLLKK---IDECNEIIYG-----------ERFLINQDNS-----------------V
ND-----DLFKS----------------YLSCI---PENDFN------------------------------
---------------------------IDLGGNIN----------------DFLGKDSLENIQ---------
YMFEDIFDGESTTVFLT-----QHDVNVVYINHNSG---EIGACISGDELHIIAKNLF--DFFS-QINVIQ-
-------NLIIDLYDSNPRG-----------------------ENGGYKTGFLEELKKTL-----IKEN---
VVLDVD---YFLEFFYG-------------------------------------------------------
---------------------------
>AbPK1_6
-----------MNFNDLLKK---IDECNEIIYG-----------ERFLINQDNS-----------------V
ND-----DLFKS----------------YLSCI---PENDFN------------------------------
---------------------------IDLGGNIN----------------DFLGKDSLENIQ---------
YMFEDIFDGESTTVFLT-----QHDVNVVYINHNSG---EIGACISGDELHIIAKNLF--DFFS-QINVIQ-
-------NLIIDLYDSNPRG-----------------------ENGGYKTGFLEELKKTL-----IKEN---
VVLDVD---YFLEFFYG-------------------------------------------------------
---------------------------
>AC29_6
-----------MNFNDLLKK---IDECNEIIYG-----------ERFLINQDNS-----------------V
ND-----DLFKS----------------YLSCI---PENDFN------------------------------
---------------------------IDLGGNIN----------------DFLGKDSLENIQ---------
YMFEDIFDGESTTVFLT-----QHDVNVVYINHNSG---EIGACISGDELHIIAKNLF--DFFS-QINVIQ-
-------NLIIDLYDSNPRG-----------------------ENGGYKTGFLEELKKTL-----IKEN---
VVLDVD---YFLEFFYG-------------------------------------------------------
---------------------------
>AC30_6
-----------MNFNDLLKK---IDECNEIIYG-----------ERFLINQDNS-----------------V
ND-----DLFKS----------------YLSCI---PENDFN------------------------------
```

---------------------------IDLGGNIN----------------DFLGKDSLENIQ---------
YMFEDIFDGESTTVFLT-----QHDVNVVYINHNSG---EIGACISGDELHIIAKNLF--DFFS-QINVIQ-
-------NLIIDLYDSNPRG-----------------------ENGGYKTGFLEELKKTL-----IKEN---
VVLDVD---YFLEFFYG-------------------------------------------------------
---------------------------
>ACICU_6
-----------MNFNDLLKK---IDECNEIIYG-----------ERFLINQDNS-----------------V
ND-----DLFKS----------------YLSCI---PENDFN------------------------------
---------------------------IDLGGNIN----------------DFLGKDSLENIQ---------
YMFEDIFDGESTTVFLT-----QHDVNVVYINHNSG---EIGACISGDELHIIAKNLF--DFFS-QINVIQ-
-------NLIIDLYDSNPRG-----------------------ENGGYKTGFLEELKKTL-----IKEN---
VVLDVD---YFLEFFYG-------------------------------------------------------
---------------------------
>AR_0056_6
-----------MNFNDLLKK---IDECNEIIYG-----------ERFLINQDNS-----------------V
ND-----DLFKS----------------YLSCI---PENDFN------------------------------
---------------------------IDLGGNIN----------------DFLGKDSLENIQ---------
YMFEDIFDGESTTVFLT-----QHDVNVVYINHNSG---EIGACISGDELHIIAKNLF--DFFS-QINVIQ-
-------NLIIDLYDSNPRG-----------------------ENGGYKTGFLEELKKTL-----IKEN---
VVLDVD---YFLEFFYG-------------------------------------------------------
---------------------------
>AYP-A2_6
-----------MNFNDLLKK---IDECNEIIYG-----------ERFLINQDNS-----------------V
ND-----DLFKS----------------YLSCI---PENDFN------------------------------
---------------------------IDLGGNIN----------------DFLGKDSLENIQ---------
YMFEDIFDGESTTVFLT-----QHDVNVVYINHNSG---EIGACISGDELHIIAKNLF--DFFS-QINVIQ-
-------NLIIDLYDSNPRG-----------------------ENGGYKTGFLEELKKTL-----IKEN---
VVLDVD---YFLEFFYG-------------------------------------------------------
---------------------------
>BJAB07104_6
-----------MNFNDLLKK---IDECNEIIYG-----------ERFLINQDNS-----------------V
ND-----DLFKS----------------YLSCI---PENDFN------------------------------
---------------------------IDLGGNIN----------------DFLGKDSLENIQ---------

YMFEDIFDGESTTVFLT-----QHDVNVVYINHNSG---EIGACISGDELHIIAKNLF--DFFS-QINVIQ-
-------NLIIDLYDSNPRG-----------------------ENGGYKTGFLEELKKTL-----IKEN---
VVLDVD---YFLEFFYG-------------------------------------------------------
---------------------------
>BJAB0868_6
-----------MNFNDLLKK---IDECNEIIYG-----------ERFLINQDNS-----------------V
ND-----DLFKS----------------YLSCI---PENDFN------------------------------
---------------------------IDLGGNIN----------------DFLGKDSLENIQ---------
YMFEDIFDGESTTVFLT-----QHDVNVVYINHNSG---EIGACISGDELHIIAKNLF--DFFS-QINVIQ-
-------NLIIDLYDSNPRG-----------------------ENGGYKTGFLEELKKTL-----IKEN---
VVLDVD---YFLEFFYG-------------------------------------------------------
---------------------------
>CBA7_6
-----------MNFNDLLKK---IDECNEIIYG-----------ERFLINQDNS-----------------V
ND-----DLFKS----------------YLSCI---PENDFN------------------------------
---------------------------IDLGGNIN----------------DFLGKDSLENIQ---------
YMFEDIFDGESTTVFLT-----QHDVNVVYINHNSG---EIGACISGDELHIIAKNLF--DFFS-QINVIQ-
-------NLIIDLYDSNPRG-----------------------ENGGYKTGFLEELKKTL-----IKEN---
VVLDVD---YFLEFFYG-------------------------------------------------------
---------------------------
>HRAB-85_6
-----------MNFNDLLKK---IDECNEIIYG-----------ERFLINQDNS-----------------V
ND-----DLFKS----------------YLSCI---PENDFN------------------------------
---------------------------IDLGGNIN----------------DFLGKDSLENIQ---------
YMFEDIFDGESTTVFLT-----QHDVNVVYINHNSG---EIGACISGDELHIIAKNLF--DFFS-QINVIQ-
-------NLIIDLYDSNPRG-----------------------ENGGYKTGFLEELKKTL-----IKEN---
VVLDVD---YFLEFFYG-------------------------------------------------------
---------------------------
>JBA13_6
-----------MNFNDLLKK---IDECNEIIYG-----------ERFLINQDNS-----------------V
ND-----DLFKS----------------YLSCI---PENDFN------------------------------
---------------------------IDLGGNIN----------------DFLGKDSLENIQ---------
YMFEDIFDGESTTVFLT-----QHDVNVVYINHNSG---EIGACISGDELHIIAKNLF--DFFS-QINVIQ-

-------NLIIDLYDSNPRG-----------------------ENGGYKTGFLEELKKTL-----IKEN---
VVLDVD---YFLEFFYG-------------------------------------------------------
---------------------------
>KAB01_6
-----------MNFNDLLKK---IDECNEIIYG-----------ERFLINQDNS-----------------V
ND-----DLFKS----------------YLSCI---PENDFN------------------------------
---------------------------IDLGGNIN----------------DFLGKDSLENIQ---------
YMFEDIFDGESTTVFLT-----QHDVNVVYINHNSG---EIGACISGDELHIIAKNLF--DFFS-QINVIQ-
-------NLIIDLYDSNPRG-----------------------ENGGYKTGFLEELKKTL-----IKEN---
VVLDVD---YFLEFFYG-------------------------------------------------------
---------------------------
>KAB02_6
-----------MNFNDLLKK---IDECNEIIYG-----------ERFLINQDNS-----------------V
ND-----DLFKS----------------YLSCI---PENDFN------------------------------
---------------------------IDLGGNIN----------------DFLGKDSLENIQ---------
YMFEDIFDGESTTVFLT-----QHDVNVVYINHNSG---EIGACISGDELHIIAKNLF--DFFS-QINVIQ-
-------NLIIDLYDSNPRG-----------------------ENGGYKTGFLEELKKTL-----IKEN---
VVLDVD---YFLEFFYG-------------------------------------------------------
---------------------------
>KAB03_6
-----------MNFNDLLKK---IDECNEIIYG-----------ERFLINQDNS-----------------V
ND-----DLFKS----------------YLSCI---PENDFN------------------------------
---------------------------IDLGGNIN----------------DFLGKDSLENIQ---------
YMFEDIFDGESTTVFLT-----QHDVNVVYINHNSG---EIGACISGDELHIIAKNLF--DFFS-QINVIQ-
-------NLIIDLYDSNPRG-----------------------ENGGYKTGFLEELKKTL-----IKEN---
VVLDVD---YFLEFFYG-------------------------------------------------------
---------------------------
>KAB04_6
-----------MNFNDLLKK---IDECNEIIYG-----------ERFLINQDNS-----------------V
ND-----DLFKS----------------YLSCI---PENDFN------------------------------
---------------------------IDLGGNIN----------------DFLGKDSLENIQ---------
YMFEDIFDGESTTVFLT-----QHDVNVVYINHNSG---EIGACISGDELHIIAKNLF--DFFS-QINVIQ-
-------NLIIDLYDSNPRG-----------------------ENGGYKTGFLEELKKTL-----IKEN---

```
VVLDVD---YFLEFFYG-------------------------------------------------------
---------------------------
>KAB05_6
-----------MNFNDLLKK---IDECNEIIYG-----------ERFLINQDNS-----------------V
ND-----DLFKS----------------YLSCI---PENDFN------------------------------
---------------------------IDLGGNIN----------------DFLGKDSLENIQ---------
YMFEDIFDGESTTVFLT-----QHDVNVVYINHNSG---EIGACISGDELHIIAKNLF--DFFS-QINVIQ-
-------NLIIDLYDSNPRG-----------------------ENGGYKTGFLEELKKTL-----IKEN---
VVLDVD---YFLEFFYG-------------------------------------------------------
---------------------------
>KAB06_6
-----------MNFNDLLKK---IDECNEIIYG-----------ERFLINQDNS-----------------V
ND-----DLFKS----------------YLSCI---PENDFN------------------------------
---------------------------IDLGGNIN----------------DFLGKDSLENIQ---------
YMFEDIFDGESTTVFLT-----QHDVNVVYINHNSG---EIGACISGDELHIIAKNLF--DFFS-QINVIQ-
-------NLIIDLYDSNPRG-----------------------ENGGYKTGFLEELKKTL-----IKEN---
VVLDVD---YFLEFFYG-------------------------------------------------------
---------------------------
>KAB07_6
-----------MNFNDLLKK---IDECNEIIYG-----------ERFLINQDNS-----------------V
ND-----DLFKS----------------YLSCI---PENDFN------------------------------
---------------------------IDLGGNIN----------------DFLGKDSLENIQ---------
YMFEDIFDGESTTVFLT-----QHDVNVVYINHNSG---EIGACISGDELHIIAKNLF--DFFS-QINVIQ-
-------NLIIDLYDSNPRG-----------------------ENGGYKTGFLEELKKTL-----IKEN---
VVLDVD---YFLEFFYG-------------------------------------------------------
---------------------------
>KAB08_6
-----------MNFNDLLKK---IDECNEIIYG-----------ERFLINQDNS-----------------V
ND-----DLFKS----------------YLSCI---PENDFN------------------------------
---------------------------IDLGGNIN----------------DFLGKDSLENIQ---------
YMFEDIFDGESTTVFLT-----QHDVNVVYINHNSG---EIGACISGDELHIIAKNLF--DFFS-QINVIQ-
-------NLIIDLYDSNPRG-----------------------ENGGYKTGFLEELKKTL-----IKEN---
VVLDVD---YFLEFFYG-------------------------------------------------------
```

---------------------------
>KBN10P02143_6
-----------MNFNDLLKK---IDECNEIIYG-----------ERFLINQDNS-----------------V
ND-----DLFKS----------------YLSCI---PENDFN------------------------------
---------------------------IDLGGNIN----------------DFLGKDSLENIQ---------
YMFEDIFDGESTTVFLT-----QHDVNVVYINHNSG---EIGACISGDELHIIAKNLF--DFFS-QINVIQ-
-------NLIIDLYDSNPRG-----------------------ENGGYKTGFLEELKKTL-----IKEN---
VVLDVD---YFLEFFYG-------------------------------------------------------
---------------------------
>MDR-TJ_6
-----------MNFNDLLKK---IDECNEIIYG-----------ERFLINQDNS-----------------V
ND-----DLFKS----------------YLSCI---PENDFN------------------------------
---------------------------IDLGGNIN----------------DFLGKDSLENIQ---------
YMFEDIFDGESTTVFLT-----QHDVNVVYINHNSG---EIGACISGDELHIIAKNLF--DFFS-QINVIQ-
-------NLIIDLYDSNPRG-----------------------ENGGYKTGFLEELKKTL-----IKEN---
VVLDVD---YFLEFFYG-------------------------------------------------------
---------------------------
>MDR-ZJ06_6
-----------MNFNDLLKK---IDECNEIIYG-----------ERFLINQDNS-----------------V
ND-----DLFKS----------------YLSCI---PENDFN------------------------------
---------------------------IDLGGNIN----------------DFLGKDSLENIQ---------
YMFEDIFDGESTTVFLT-----QHDVNVVYINHNSG---EIGACISGDELHIIAKNLF--DFFS-QINVIQ-
-------NLIIDLYDSNPRG-----------------------ENGGYKTGFLEELKKTL-----IKEN---
VVLDVD---YFLEFFYG-------------------------------------------------------
---------------------------
>NCGM237_6
-----------MNFNDLLKK---IDECNEIIYG-----------ERFLINQDNS-----------------V
ND-----DLFKS----------------YLSCI---PENDFN------------------------------
---------------------------IDLGGNIN----------------DFLGKDSLENIQ---------
YMFEDIFDGESTTVFLT-----QHDVNVVYINHNSG---EIGACISGDELHIIAKNLF--DFFS-QINVIQ-
-------NLIIDLYDSNPRG-----------------------ENGGYKTGFLEELKKTL-----IKEN---
VVLDVD---YFLEFFYG-------------------------------------------------------
---------------------------

```
>SAA14_6
-----------MNFNDLLKK---IDECNEIIYG-----------ERFLINQDNS-----------------V
ND-----DLFKS----------------YLSCI---PENDFN------------------------------
---------------------------IDLGGNIN----------------DFLGKDSLENIQ---------
YMFEDIFDGESTTVFLT-----QHDVNVVYINHNSG---EIGACISGDELHIIAKNLF--DFFS-QINVIQ-
-------NLIIDLYDSNPRG-----------------------ENGGYKTGFLEELKKTL-----IKEN---
VVLDVD---YFLEFFYG-------------------------------------------------------
---------------------------
>SMC_Paed_Ab_BL01_6
-----------MNFNDLLKK---IDECNEIIYG-----------ERFLINQDNS-----------------V
ND-----DLFKS----------------YLSCI---PENDFN------------------------------
---------------------------IDLGGNIN----------------DFLGKDSLENIQ---------
YMFEDIFDGESTTVFLT-----QHDVNVVYINHNSG---EIGACISGDELHIIAKNLF--DFFS-QINVIQ-
-------NLIIDLYDSNPRG-----------------------ENGGYKTGFLEELKKTL-----IKEN---
VVLDVD---YFLEFFYG-------------------------------------------------------
---------------------------
>SSA12_6
-----------MNFNDLLKK---IDECNEIIYG-----------ERFLINQDNS-----------------V
ND-----DLFKS----------------YLSCI---PENDFN------------------------------
---------------------------IDLGGNIN----------------DFLGKDSLENIQ---------
YMFEDIFDGESTTVFLT-----QHDVNVVYINHNSG---EIGACISGDELHIIAKNLF--DFFS-QINVIQ-
-------NLIIDLYDSNPRG-----------------------ENGGYKTGFLEELKKTL-----IKEN---
VVLDVD---YFLEFFYG-------------------------------------------------------
---------------------------
>SSA6_6
-----------MNFNDLLKK---IDECNEIIYG-----------ERFLINQDNS-----------------V
ND-----DLFKS----------------YLSCI---PENDFN------------------------------
---------------------------IDLGGNIN----------------DFLGKDSLENIQ---------
YMFEDIFDGESTTVFLT-----QHDVNVVYINHNSG---EIGACISGDELHIIAKNLF--DFFS-QINVIQ-
-------NLIIDLYDSNPRG-----------------------ENGGYKTGFLEELKKTL-----IKEN---
VVLDVD---YFLEFFYG-------------------------------------------------------
---------------------------
>TYTH-1_6
```

-----------MNFNDLLKK---IDECNEIIYG-----------ERFLINQDNS-----------------V
ND-----DLFKS----------------YLSCI---PENDFN------------------------------
---------------------------IDLGGNIN----------------DFLGKDSLENIQ---------
YMFEDIFDGESTTVFLT-----QHDVNVVYINHNSG---EIGACISGDELHIIAKNLF--DFFS-QINVIQ-
-------NLIIDLYDSNPRG-----------------------ENGGYKTGFLEELKKTL-----IKEN---
VVLDVD---YFLEFFYG-------------------------------------------------------
---------------------------
>USA2_6
-----------MNFNDLLKK---IDECNEIIYG-----------ERFLINQDNS-----------------V
ND-----DLFKS----------------YLSCI---PENDFN------------------------------
---------------------------IDLGGNIN----------------DFLGKDSLENIQ---------
YMFEDIFDGESTTVFLT-----QHDVNVVYINHNSG---EIGACISGDELHIIAKNLF--DFFS-QINVIQ-
-------NLIIDLYDSNPRG-----------------------ENGGYKTGFLEELKKTL-----IKEN---
VVLDVD---YFLEFFYG-------------------------------------------------------
---------------------------
>WCHAB005133_6
-----------MNFNDLLKK---IDECNEIIYG-----------ERFLINQDNS-----------------V
ND-----DLFKS----------------YLSCI---PENDFN------------------------------
---------------------------IDLGGNIN----------------DFLGKDSLENIQ---------
YMFEDIFDGESTTVFLT-----QHDVNVVYINHNSG---EIGACISGDELHIIAKNLF--DFFS-QINVIQ-
-------NLIIDLYDSNPRG-----------------------ENGGYKTGFLEELKKTL-----IKEN---
VVLDVD---YFLEFFYG-------------------------------------------------------
---------------------------
>XDR-BJ83_6
-----------MNFNDLLKK---IDECNEIIYG-----------ERFLINQDNS-----------------V
ND-----DLFKS----------------YLSCI---PENDFN------------------------------
---------------------------IDLGGNIN----------------DFLGKDSLENIQ---------
YMFEDIFDGESTTVFLT-----QHDVNVVYINHNSG---EIGACISGDELHIIAKNLF--DFFS-QINVIQ-
-------NLIIDLYDSNPRG-----------------------ENGGYKTGFLEELKKTL-----IKEN---
VVLDVD---YFLEFFYG-------------------------------------------------------
---------------------------
>XH386_6
-----------MNFNDLLKK---IDECNEIIYG-----------ERFLINQDNS-----------------V

```
ND-----DLFKS----------------YLSCI---PENDFN------------------------------
---------------------------IDLGGNIN----------------DFLGKDSLENIQ---------
YMFEDIFDGESTTVFLT-----QHDVNVVYINHNSG---EIGACISGDELHIIAKNLF--DFFS-QINVIQ-
-------NLIIDLYDSNPRG-----------------------ENGGYKTGFLEELKKTL-----IKEN---
VVLDVD---YFLEFFYG-------------------------------------------------------
---------------------------
>XH856_6
-----------MNFNDLLKK---IDECNEIIYG-----------ERFLINQDNS-----------------V
ND-----DLFKS----------------YLSCI---PENDFN------------------------------
---------------------------IDLGGNIN----------------DFLGKDSLENIQ---------
YMFEDIFDGESTTVFLT-----QHDVNVVYINHNSG---EIGACISGDELHIIAKNLF--DFFS-QINVIQ-
-------NLIIDLYDSNPRG-----------------------ENGGYKTGFLEELKKTL-----IKEN---
VVLDVD---YFLEFFYG-------------------------------------------------------
---------------------------
>XH857_6
-----------MNFNDLLKK---IDECNEIIYG-----------ERFLINQDNS-----------------V
ND-----DLFKS----------------YLSCI---PENDFN------------------------------
---------------------------IDLGGNIN----------------DFLGKDSLENIQ---------
YMFEDIFDGESTTVFLT-----QHDVNVVYINHNSG---EIGACISGDELHIIAKNLF--DFFS-QINVIQ-
-------NLIIDLYDSNPRG-----------------------ENGGYKTGFLEELKKTL-----IKEN---
VVLDVD---YFLEFFYG-------------------------------------------------------
---------------------------
>XH859_6
-----------MNFNDLLKK---IDECNEIIYG-----------ERFLINQDNS-----------------V
ND-----DLFKS----------------YLSCI---PENDFN------------------------------
---------------------------IDLGGNIN----------------DFLGKDSLENIQ---------
YMFEDIFDGESTTVFLT-----QHDVNVVYINHNSG---EIGACISGDELHIIAKNLF--DFFS-QINVIQ-
-------NLIIDLYDSNPRG-----------------------ENGGYKTGFLEELKKTL-----IKEN---
VVLDVD---YFLEFFYG-------------------------------------------------------
---------------------------
>XH860_6
-----------MNFNDLLKK---IDECNEIIYG-----------ERFLINQDNS-----------------V
ND-----DLFKS----------------YLSCI---PENDFN------------------------------
```

```
---------------------------IDLGGNIN----------------DFLGKDSLENIQ---------
YMFEDIFDGESTTVFLT-----QHDVNVVYINHNSG---EIGACISGDELHIIAKNLF--DFFS-QINVIQ-
-------NLIIDLYDSNPRG-----------------------ENGGYKTGFLEELKKTL-----IKEN---
VVLDVD---YFLEFFYG-------------------------------------------------------
---------------------------
>YU-R612_6
-----------MNFNDLLKK---IDECNEIIYG-----------ERFLINQDNS-----------------V
ND-----DLFKS----------------YLSCI---PENDFN------------------------------
---------------------------IDLGGNIN----------------DFLGKDSLENIQ---------
YMFEDIFDGESTTVFLT------QHDVNVVYINHNS--GEIGACISGDELHIIAKNLF--DFFS-QINVIQ-
-------NLIIDLYDSNPRG-----------------------ENGGYKTGFLEELKKTL-----IKEN---
VVLDVD---YFLEFFYG-------------------------------------------------------
---------------------------
>AB030_22
----------MRNNQE-------LDDLIDQLYD------------------------------------LEL
EQ-----AYFVY----------------IKFCY---KI----------------------------------
---------------------------IPLLSNIN--------------------------SE---------
YIIESIQKAKSFWFDSK-----SNIDEKEIINRRVK--IIKYMEEKDIDFNINSEPEL--KFSI--------
----------IPLWTKPPSE-----------------------DIGDSLDWAFTLLKVIN-----IPDD---
IVKTKL---YEALNDIP-------------------------------------------------------
---------------------------
>AbH12O-A2_22
----------MRNNQE-------LDDLIDQLYD------------------------------------LEL
EQ-----AYFVY----------------IKFCY---KI----------------------------------
---------------------------IPLLSNIN--------------------------SE---------
YIIESIQKAKSFWFDSK-----SNIDEKEIINRRVK--IIKYMEEKDIDFNINSEPEL--KFSI--------
----------IPLWTKPPSE-----------------------DIGDSLDWAFTLLKVIN-----IPDD---
IVKTKL---YEALNDIP-------------------------------------------------------
---------------------------
>AF-401_22
----------MRNNQE-------LDDLIDQLYD------------------------------------LEL
EQ-----AYFVY----------------IKFCY---KI----------------------------------
---------------------------IPLLSNIN--------------------------SE---------
```

```
YIIESIQKAKSFWFDSK-----SNIDEKEIINRRVK--IIKYMEEKDIDFNINSEPEL--KFSI--------
----------IPLWTKPPSE-----------------------DIGDSLDWAFTLLKVIN-----IPDD---
IVKTKL---YEALNDIP-------------------------------------------------------
---------------------------
>AR_0063_22
----------MRNNQE-------LDDLIDQLYD------------------------------------LEL
EQ-----AYFVY----------------IKFCY---KI----------------------------------
---------------------------IPLLSNIN--------------------------SE---------
YIIESIQKAKSFWFDSK-----SNIDEKEIINRRVK--IIKYMEEKDIDFNINSEPEL--KFSI--------
----------IPLWTKPPSE-----------------------DIGDSLDWAFTLLKVIN-----IPDD---
IVKTKL---YEALNDIP-------------------------------------------------------
---------------------------
>AR_0101_22
----------MRNNQE-------LDDLIDQLYD------------------------------------LEL
EQ-----AYFVY----------------IKFCY---KI----------------------------------
---------------------------IPLLSNIN--------------------------SE---------
YIIESIQKAKSFWFDSK-----SNIDEKEIINRRVK--IIKYMEEKDIDFNINSEPEL--KFSI--------
----------IPLWTKPPSE-----------------------DIGDSLDWAFTLLKVIN-----IPDD---
IVKTKL---YEALNDIP-------------------------------------------------------
---------------------------
>IOMTU433_22
----------MRNNQE-------LDDLIDQLYD------------------------------------LEL
EQ-----AYFVY----------------IKFCY---KI----------------------------------
---------------------------IPLLSNIN--------------------------SE---------
YIIESIQKAKSFWFDSK-----SNIDEKEIINRRVK--IIKYMEEKDIDFNINSEPEL--KFSI--------
----------IPLWTKPPSE-----------------------DIGDSLDWAFTLLKVIN-----IPDD---
IVKTKL---YEALNDIP-------------------------------------------------------
---------------------------
>ATCC17978-mff_2
---------MNQEDIDYFYE----------KYG-----------------QPID----K----------VEV
TE-----DIIKK----------------YRGKL---PESILE-----------QWHLFGFAGYLNGLYWITN
PDDYAEVIYDWLEETP-----------LPDDDVYH-----------VLARSAFGELLIWGERN---------
YGRYYIKTMEGILHDNG-----EQLESAEFYGSDF---FFLPKKNYLDYTDKNGNKLF--DRAVKKLGVLK-
```

---------ADEMYAFEPAL----------------------ALGGEESLQHLTKVNLP------VHMK---
LLKQVT---PLRLRTFEDLTAALYGTSYSVDDLTSGQDAESQYQESVQAGEVCPRTGYWTTPAQPNTRHYCK
KGEVLPEIKEQDWGEVYWYWDGEN---
>CIP70.10_2
---------MNQEDIDYFYE----------KYG-----------------QPID----K----------VEV
TE-----DIIKK----------------YRGKL---PESILE-----------QWHLFGFAGYLNGLYWITN
PDDYAEVIYDWLEETP-----------LPDDDVYH-----------VLARSAFGELLIWGERN---------
YGRYYIKTMEGILHDNG-----EQLESAEFYGSDFF--FLPKKNYLDYT-DKNGNKLF--DRAVKKLGVLK-
---------ADEMYAFEPAL----------------------ALGGEESLQHLTKVNLP------VHMK---
LLKQVT---PLRLRTFEDLTAALYGTSYSVDDLTSGQDAESQYQESVQAGEVCPRTGYWTTPAQPNTRHYCK
KGEVLPEIKEQDWGEVYWYWDGEN---
>R2091_2
---------MNQEDIDYFYE----------KYG-----------------QPID----K----------VEV
TE-----DIIKK----------------YRGKL---PESILE-----------QWHLFGFAGYLNGLYWITN
PDDYAEVIYDWLEETP-----------LPDDDVYH-----------VLARSAFGELLIWGERN---------
YGRYYIKTMEGILHDNG-----EQLESAEFYGSDFF--FLPKKNYLDYT-DKNGNKLF--DRAVKKLGVLK-
---------ADEMYAFEPAL----------------------ALGGEESLQHLTKVNLP------VHMK---
LLKQVT---PLRLRTFEDLTAALYGTSYSVDDLTSGQDAESQYQESVQAGEVCPRTGYWTTPAQPNTRHYCK
KGEVLPEIKEQDWGEVYWYWDGEN---
>CIP70.10_31
--------------VSNFTE---QRRQQFLTED------------YYLKTKEYNEEGIP----------LYL
ES-----DAFED---------------DQYNCG---EASRWTAITDIYTSLLYLTYTAGHSIQELIPVFEKV
IDGYEKQSEALALFHKSEKPSVITSQNETLNILSLAFLLHRNDLFLRIHSLVNGEGDSHTGED---------
EIINKFFKLNDSTHPTI-----NEGNLFSLSYAKLC--DVIDNVLVKSDKKHSVELLD--EYLS-NWYQM--
-------NRNEIWYNSHLDL-----------------------ENKSSYVGYWAFEAAAL-----VYLL---
DLDDSS---LYKHLFYPKDIVQWARNQTKIKALNDDVIQIKL-----KGGEKCTRTGYWQTPAQPNLRRYFV
QDEVLPTLIEMDWGEVYWYWDGEN---
>R2091_31
--------------VSNFTE---QRRQQFLTED------------YYLKTKEYNEEGIP----------LYL
ES-----DAFED---------------DQYNCG---EASRWTAITDIYTSLLYLTYTAGHSIQELIPVFEKV
IDGYEKQSEALALFHKSEKPSVITSQNETLNILSLAFLLHRNDLFLRIHSLVNGEGDSHTGED---------
EIINKFFKLNDSTHPTI-----NEGNLFSLSYAKLC--DVIDNVLVKSDKKHSVELLD--EYLS-NWYQM--
-------NRNEIWYNSHLDL-----------------------ENKSSYVGYWAFEAAAL-----VYLL---

DLDDSS---LYKHLFYPKDIVQWARNQTKIKALNDDVIQIKL-----KGGEKCTRTGYWQTPAQPNLRRYFV
QDEVLPTLIEMDWGEVYWYWDGEN---
>AR_0088_32
MKLEYQVNDQEIDWQKWFDQ----ENKDSDSYR-----------DSFLETEMYQ----R--RFNTFLNGIYS
VT-----DGFRK----------------YKTSA---KAGKMMSAGHQRLQDVLNLMSLQYSAGGDISFIKEL
YPYLLHWMEEYAETHAAYHQTEE----AGGRYVWHISLGTEDYWYIALRLICFGLLTGYADQM---------
ARIMPIIDYVEATPEGQ-----EKDGLIERLVAPFV--ADRGTPPDEARRHLPYRKLI--KVFNAAPEQRP-
-------ALMLQYLESWYEA-----------SRREPYYNQHPQADIDEGFLYYGYWSWEA-----AAVT---
WLLDIDDALYREHEFYPKDLVDFARTQSNVVPNEEQPERIKV-----KGGEACIKTGHWITPAKPDTRLYFT
QGTILPILSETDWGEVYWYWDGEN---
>AR_0088_14
--------MKNTSVSSTMLIVLSILGCSSNTSIAKGINKIDVKDCSIDYDEVDFCSKER----------LKD
YN-----NILKN----------------KVSNF---DKNKFLMNFKEKNYLYFAVIDLEKLKVFTFPASLSV
MSNIKPIEFSSDSNS------------FCLNGNFN--QYQNSYRAVKTCYTYNNGSFDFKSRV---------
ELDKDINNSNNVIKKLN-----LPISSDFFSKCREK--NSAKKCTQLENSNNRAYSFS--ELEKISPDFINV
LNDTKVDSLNVNTFRFLPQF------------------------KDSFYAIAEKYIDTDE-----SSSS---
EFYLIKIKPDLKVEKIGDYYSIDSSGVISYRDKNSKILKKNLR-----------------------------
---------------------------
>CIP70.10_14
--------MKNTSVSSTMLIVLSILGCSSNTSIAKGINKIDVKDCSIDYDEVDFCSKER----------LKD
YN-----NILKN----------------KVSNF---DKNKFLMNFKEKNYLYFAVIDLEKLKVFTFPASLSV
MSNIKPIEFSSDSNS------------FCLNGNFN--QYQNSYRAVKTCYTYNNGSFDFKSRV---------
ELDKDINNSNNVIKKLN-----LPISSDFFSKCREK--NSAKKCTQLENSNNRAYSFS--ELEKISPDFINV
LNDTKVDSLNVNTFRFLPQF------------------------KDSFYAIAEKYIDTDE-----SSSS---
EFYLIKIKPDLKVEKIGDYYSIDSSGVISYRDKNSKILKKNLR-----------------------------
---------------------------
>HWBA8_14
--------MKNTSVSSTMLIVLSILGCSSNTSIAKGINKIDVKDCSIDYDEVDFCSKER----------LKD
YN-----NILKN----------------KVSNF---DKNKFLMNFKEKNYLYFAVIDLEKLKVFTFPASLSV
MSNIKPIEFSSDSNS------------FCLNGNFN--QYQNSYRAVKTCYTYNNGSFDFKSRV---------
ELDKDINNSNNVIKKLN-----LPISSDFFSKCREK--NSAKKCTQLENSNNRAYSFS--ELEKISPDFINV
LNDTKVDSLNVNTFRFLPQF------------------------KDSFYAIAEKYIDTDE-----SSSS---
EFYLIKIKPDLKVEKIGDYYSIDSSGVISYRDKNSKILKKNLR-----------------------------

```
---------------------------
>R2091_14
--------MKNTSVSSTMLIVLSILGCSSNTSIAKGINKIDVKDCSIDYDEVDFCSKER----------LKD
YN-----NILKN----------------KVSNF---DKNKFLMNFKEKNYLYFAVIDLEKLKVFTFPASLSV
MSNIKPIEFSSDSNS------------FCLNGNFN--QYQNSYRAVKTCYTYNNGSFDFKSRV---------
ELDKDINNSNNVIKKLN-----LPISSDFFSKCREK--NSAKKCTQLENSNNRAYSFS--ELEKISPDFINV
LNDTKVDSLNVNTFRFLPQF------------------------KDSFYAIAEKYIDTDE-----SSSS---
EFYLIKIKPDLKVEKIGDYYSIDSSGVISYRDKNSKILKKNLR-----------------------------
---------------------------
>TYTH-1_14
--------MKNTSVSSTMLIVLSILGCSSNTSIAKGINKIDVKDCSIDYDEVDFCSKER----------LKD
YN-----NILKN----------------KVSNF---DKNKFLMNFKEKNYLYFAVIDLEKLKVFTFPASLSV
MSNIKPIEFSSDSNS------------FCLNGNFN--QYQNSYRAVKTCYTYNNGSFDFKSRV---------
ELDKDINNSNNVIKKLN-----LPISSDFFSKCREK--NSAKKCTQLENSNNRAYSFS--ELEKISPDFINV
LNDTKVDSLNVNTFRFLPQF------------------------KDSFYAIAEKYIDTDE-----SSSS---
EFYLIKIKPDLKVEKIGDYYSIDSSGVISYRDKNSKILKKNLR-----------------------------
---------------------------
>15A5_28
-----------MPKIHILER---PLKISSNGDN----------YSEYTILPAGT----V----------LYD
DS-----DSLNR------------------------------------------------------------
-------------------------------RVMV--------------------YFNLQGVD---------
FNFEEQDPEISKQPSEV-----RSIRSTELSDILK----TIPLTKKDIYLIIKYDDSI-NDSVR--------
-------SILFKKYKIDPSE----------------------------------------------------
---------YEK------------------------------------------------------------
---------------------------
>Ab4568_28
-----------MPKIHILER---PLKISSNGDN----------YSEYTILPAGT----V----------LYD
DS-----DSLNR------------------------------------------------------------
-------------------------------RVMV--------------------YFNLQGVD---------
FNFEEQDPEISKQPSEV-----RSIRSTELSDILK----TIPLTKKDIYLIIKYDDSI-NDSVR--------
-------SILFKKYKIDPSE----------------------------------------------------
---------YEK------------------------------------------------------------
---------------------------
```

>Ab4977_28
-----------MPKIHILER---PLKISSNGDN----------YSEYTILPAGT----V----------LYD
DS-----DSLNR------------------------------------------------------------
-------------------------------RVMV--------------------YFNLQGVD---------
FNFEEQDPEISKQPSEV-----RSIRSTELSDILK----TIPLTKKDIYLIIKYDDSI-NDSVR--------
-------SILFKKYKIDPSE----------------------------------------------------
---------YEK------------------------------------------------------------
---------------------------
>JBA13_28
-----------MPKIHILER---PLKISSNGDN----------YSEYTILPAGT----V----------LYD
DS-----DSLNR------------------------------------------------------------
-------------------------------RVMV--------------------YFNLQGVD---------
FNFEEQDPEISKQPSEV-----RSIRSTELSDILK----TIPLTKKDIYLIIKYDDSI-NDSVR--------
-------SILFKKYKIDPSE----------------------------------------------------
---------YEK------------------------------------------------------------
---------------------------
>KAB04_28
-----------MPKIHILER---PLKISSNGDN----------YSEYTILPAGT----V----------LYD
DS-----DSLNR------------------------------------------------------------
-------------------------------RVMV--------------------YFNLQGVD---------
FNFEEQDPEISKQPSEV-----RSIRSTELSDILK----TIPLTKKDIYLIIKYDDSI-NDSVR--------
-------SILFKKYKIDPSE----------------------------------------------------
---------YEK------------------------------------------------------------
---------------------------
>KAB07_28
-----------MPKIHILER---PLKISSNGDN----------YSEYTILPAGT----V----------LYD
DS-----DSLNR------------------------------------------------------------
-------------------------------RVMV--------------------YFNLQGVD---------
FNFEEQDPEISKQPSEV-----RSIRSTELSDILK----TIPLTKKDIYLIIKYDDSI-NDSVR--------
-------SILFKKYKIDPSE----------------------------------------------------
---------YEK------------------------------------------------------------
---------------------------
>KAB08_28

```
-----------MPKIHILER---PLKISSNGDN----------YSEYTILPAGT----V----------LYD
DS-----DSLNR------------------------------------------------------------
-------------------------------RVMV--------------------YFNLQGVD---------
FNFEEQDPEISKQPSEV-----RSIRSTELSDILK----TIPLTKKDIYLIIKYDDSI-NDSVR--------
-------SILFKKYKIDPSE----------------------------------------------------
---------YEK------------------------------------------------------------
---------------------------
>KBN10P02143_28
-----------MPKIHILER---PLKISSNGDN----------YSEYTILPAGT----V----------LYD
DS-----DSLNR------------------------------------------------------------
-------------------------------RVMV--------------------YFNLQGVD---------
FNFEEQDPEISKQPSEV-----RSIRSTELSDILK----TIPLTKKDIYLIIKYDDSI-NDSVR--------
-------SILFKKYKIDPSE----------------------------------------------------
---------YEK------------------------------------------------------------
---------------------------
>SAA14_28
-----------MPKIHILER---PLKISSNGDN----------YSEYTILPAGT----V----------LYD
DS-----DSLNR------------------------------------------------------------
-------------------------------RVMV--------------------YFNLQGVD---------
FNFEEQDPEISKQPSEV-----RSIRSTELSDILK----TIPLTKKDIYLIIKYDDSI-NDSVR--------
-------SILFKKYKIDPSE----------------------------------------------------
---------YEK------------------------------------------------------------
---------------------------
>SMC_Paed_Ab_BL01_28
-----------MPKIHILER---PLKISSNGDN----------YSEYTILPAGT----V----------LYD
DS-----DSLNR------------------------------------------------------------
-------------------------------RVMV--------------------YFNLQGVD---------
FNFEEQDPEISKQPSEV-----RSIRSTELSDILK----TIPLTKKDIYLIIKYDDSI-NDSVR--------
-------SILFKKYKIDPSE----------------------------------------------------
---------YEK------------------------------------------------------------
---------------------------
>SSA12_28
-----------MPKIHILER---PLKISSNGDN----------YSEYTILPAGT----V----------LYD
```

DS-----DSLNR------------------------------------------------------------
-------------------------------RVMV--------------------YFNLQGVD---------
FNFEEQDPEISKQPSEV-----RSIRSTELSDILK----TIPLTKKDIYLIIKYDDSI-NDSVR--------
-------SILFKKYKIDPSE----------------------------------------------------
---------YEK------------------------------------------------------------
---------------------------
>SSMA17_28
-----------MPKIHILER---PLKISSNGDN----------YSEYTILPAGT----V----------LYD
DS-----DSLNR------------------------------------------------------------
-------------------------------RVMV--------------------YFNLQGVD---------
FNFEEQDPEISKQPSEV-----RSIRSTELSDILK----TIPLTKKDIYLIIKYDDSI-NDSVR--------
-------SILFKKYKIDPSE----------------------------------------------------
---------YEK------------------------------------------------------------
---------------------------
>YU-R612_28
-----------MPKIHILER---PLKISSNGDN----------YSEYTILPAGT----V----------LYD
DS-----DSLNR------------------------------------------------------------
-------------------------------RVMV--------------------YFNLQGVD---------
FNFEEQDPEISKQPSEV-----RSIRSTELSDILK----TIPLTKKDIYLIIKYDDSI-NDSVR--------
-------SILFKKYKIDPSE----------------------------------------------------
---------YEK------------------------------------------------------------
---------------------------
>BJAB0715_12
--------------MKVIQG---EIQKLTIEDS------------KILPLKNIE----K----------QAV
GS-----AMLGA----------LASSSTLMTNA---PIMLMAARGIDAKTFTCEINGYKVIGQFTTVQFQEN
DPLVIV---ISDEQEQG--RHLAYSILDPRTGLLY----------------------MLYEMG---------
RSLKNSYKNIWQLVFIW-----SISASIIFTGLFFI--FDIFSGKEIFNFSYLIENLK--LFFL-----IL-
-------CVLLIFFSLFMNL-------VFRRSFK---------QAAELSEKIFSILGFKN-----VDEQ---
DFYNDF---IIKDGVHV----SVMEYRKNLKGPDP----------------------------------YPE
D---------------YFSKNTTSE--
>XH858_12
--------------MKVIQG---EIQKLTIEDS------------KILPLKNIE----K----------QAV
GS-----AMLGA----------LASSSTLMTNA---PIMLMAARGIDAKTFTCEINGYKVIGQFTTVQFQEN

DPLVIV---ISDEQEQG--RHLAYSILDPRTGLLY----------------------MLYEMG---------
RSLKNSYKNIWQLVFIW-----SISASIIFTGLFFI--FDIFSGKEIFNFSYLIENLK--LFFL-----IL-
-------CVLLIFFSLFMNL-------VFRRSFK---------QAAELSEKIFSILGFKN-----VDEQ---
DFYNDF---IIKDGVHV----SVMEYRKNLKGPDP----------------------------------YPE
D---------------YFSKNTTSE--
>Ab04-mff_12
--------------MKVIQG---EIQKLTIEDS------------KILPLKNIE----K-----------QAV
GS-----AMLGA----------LASSSTLMTNA---PIMLMAARGIDAKTFTCEINGYKVIGQFTTVQFQEN
DPLVIV---ISDEQEQG--RHLAYSILDPRTGLLY----------------------MLYEMG---------
RSLKNSYKNIWQLVFIW-----SISASIIFTGLFFI--FDIFSGKEIFNFSYLIENLK--LFFL-----IL-
-------CVLLIFFSLFMNL-------VFRRSFK---------QAAELSEKIFSILGFKN-----VDEQ---
DFYNDF---IIKDGVHV----SVMEYRKNLKGPDP----------------------------------YPE
D---------------YFSKNTTSE--
>AR_0088_12
--------------MKVIQG---EIQKLTIEDS------------KILPLKNIE----K-----------QAV
GS-----AMLGA----------LASSSTLMTNA---PIMLMAARGIDAKTFTCEINGYKVIGQFTTVQFQEN
DPLVIV---ISDEQEQG--RHLAYSILDPRTGLLY----------------------MLYEMG---------
RSLKNSYKNIWQLVFIW-----SISASIIFTGLFFI--FDIFSGKEIFNFSYLIENLK--LFFL-----IL-
-------CVLLIFFSLFMNL-------VFRRSFK---------QAAELSEKIFSILGFKN-----VDEQ---
DFYNDF---IIKDGVHV----SVMEYRKNLKGPDP----------------------------------YPE
D---------------YFSKNTTSE--
>CIP70.10_12
--------------MKVIQG---EIQKLTIEDS------------KILPLKNIE----K-----------QAV
GS-----AMLGA----------LASSSTLMTNA---PIMLMAARGIDAKTFTCEINGYKVIGQFTTVQFQEN
DPLVIV---ISDEQEQG--RHLAYSILDPRTGLLY----------------------MLYEMG---------
RSLKNSYKNIWQLVFIW-----SISASIIFTGLFFI--FDIFSGKEIFNFSYLIENLK--LFFL-----IL-
-------CVLLIFFSLFMNL-------VFRRSFK---------QAAELSEKIFSILGFKN-----VDEQ---
DFYNDF---IIKDGVHV----SVMEYRKNLKGPDP----------------------------------YPE
D---------------YFSKNTTSE--
>HWBA8_12
--------------MKVIQG---EIQKLTIEDS------------KILPLKNIE----K-----------QAV
GS-----AMLGA----------LASSSTLMTNA---PIMLMAARGIDAKTFTCEINGYKVIGQFTTVQFQEN
DPLVIV---ISDEQEQG--RHLAYSILDPRTGLLY----------------------MLYEMG---------

```
RSLKNSYKNIWQLVFIW-----SISASIIFTGLFFI--FDIFSGKEIFNFSYLIENLK--LFFL-----IL-
-------CVLLIFFSLFMNL-------VFRRSFK---------QAAELSEKIFSILGFKN-----VDEQ---
DFYNDF---IIKDGVHV----SVMEYRKNLKGPDP----------------------------------YPE
D---------------YFSKNTTSE--
>LAC-4_12
--------------MKVIQG---EIQKLTIEDS------------KILPLKNIE----K----------QAV
GS-----AMLGA----------LASSSTLMTNA---PIMLMAARGIDAKTFTCEINGYKVIGQFTTVQFQEN
DPLVIV---ISDEQEQG--RHLAYSILDPRTGLLY----------------------MLYEMG---------
RSLKNSYKNIWQLVFIW-----SISASIIFTGLFFI--FDIFSGKEIFNFSYLIENLK--LFFL-----IL-
-------CVLLIFFSLFMNL-------VFRRSFK---------QAAELSEKIFSILGFKN-----VDEQ---
DFYNDF---IIKDGVHV----SVMEYRKNLKGPDP----------------------------------YPE
D---------------YFSKNTTSE--
>R2091_12
--------------MKVIQG---EIQKLTIEDS------------KILPLKNIE----K----------QAV
GS-----AMLGA----------LASSSTLMTNA---PIMLMAARGIDAKTFTCEINGYKVIGQFTTVQFQEN
DPLVIV---ISDEQEQG--RHLAYSILDPRTGLLY----------------------MLYEMG---------
RSLKNSYKNIWQLVFIW-----SISASIIFTGLFFI--FDIFSGKEIFNFSYLIENLK--LFFL-----IL-
-------CVLLIFFSLFMNL-------VFRRSFK---------QAAELSEKIFSILGFKN-----VDEQ---
DFYNDF---IIKDGVHV----SVMEYRKNLKGPDP----------------------------------YPE
D---------------YFSKNTTSE--
>WKA02_12
--------------MKVIQG---EIQKLTIEDS------------KILPLKNIE----K----------QAV
GS-----AMLGA----------LASSSTLMTNA---PIMLMAARGIDAKTFTCEINGYKVIGQFTTVQFQEN
DPLVIV---ISDEQEQG--RHLAYSILDPRTGLLY----------------------MLYEMG---------
RSLKNSYKNIWQLVFIW-----SISASIIFTGLFFI--FDIFSGKEIFNFSYLIENLK--LFFL-----IL-
-------CVLLIFFSLFMNL-------VFRRSFK---------QAAELSEKIFSILGFKN-----VDEQ---
DFYNDF---IIKDGVHV----SVMEYRKNLKGPDP----------------------------------YPE
D---------------YFSKNTTSE--
>ZW85-1_20
----------LKLNLKLVKG---PIHSLEKAKT------------------------------------KIP
IN-----QNIGGIAAGTAFAGVLAESSSLLTSA---PILVMAGKAKDGITFTGEIEEHSLLGQFTTVRFKEN
DELIAVINDSPVDGRYQ-----VYAILDPKSGLLY----------------------MIYEMG---------
RSVKKGYKAIFMQTYYF-----T------LLSWAVI--SISLLLFYIFRFSYSWDSFL--RLIV-----WSF
```

GGVFGIAALMAFVNYFG-----------YRKSYE---------NFGILSEQIFEKLGFEY-----PKEQ---
DFYNDF---LTDDGVQI----SVMKYRKNLKGPDP----------------------------------YPE
D---------------YFSKNTTSE--
>TCDC-AB0715_18
--------MKVLNSFAIMIA---CSVSFNICYA--------KENSEWNDYLQDS---------------TQI
NSPIEYIDSYNK----------------YTVLN---PNLKIK------------------------------
---------------------------LVDFGNIN-----------KIKKDIKYSLKKYGVQI---------
LNRKEVYNIFEGKVPLK-----SNVDFTLLYNDKNIVAFRIRENSNIDYVKEPYKNFTANAYFY--------
-------NLINNKFIEVPVL-------NSDSEDKSKSTDILQGDQLTYDSKKGQYIYLAN-----IKSY---
KTGKTQ---SIKTIFNSNLKCISSTLGCETIGVLPATKAN--------------------------------
---------------------------
>Ab4653_18
--------MKVLNSFAIMIA---CSVSFNICYA--------KENSEWNDYLQDS---------------TQI
NS---PIEYIDS----------------YNKYTVLNPNLKIK------------------------------
---------------------------LVDFGNIN-----------KIKKDIKYSLKKYGVQI---------
LNRKEVYNIFEGKVPLK-----SNVDFTLLYNDKNIVAFRIRENSNIDYVKEPYKNFTANAYFY--------
-------NLINNKFIEVPVL-------NSDSEDKSKSTDILQGDQLTYDSKKGQYIYLAN-----IKSY---
KTGKTQ---SIKTIFNSNLKCISSTLGCETIGVLPATKAN--------------------------------
---------------------------
>BJAB07104_18
--------MKVLNSFAIMIA---CSVSFNICYA--------KENSEWNDYLQDS---------------TQI
NSPIEYIDSYNK----------------YTVLN---PNLKIK------------------------------
---------------------------LVDFGNIN-----------KIKKDIKYSLKKYGVQI---------
LNRKEVYNIFEGKVPLK-----SNVDFTLLYNDKNIVAFRIRENSNIDYVKEPYKNFTANAYFY--------
-------NLINNKFIEVPVL-------NSDSEDKSKSTDILQGDQLTYDSKKGQYIYLAN-----IKSY---
KTGKTQ---SIKTIFNSNLKCISSTLGCETIGVLPATKAN--------------------------------
---------------------------
>BJAB0868_18
--------MKVLNSFAIMIA---CSVSFNICYA--------KENSEWNDYLQDS---------------TQI
NSPIEYIDSYNK----------------YTVLN---PNLKIK------------------------------
---------------------------LVDFGNIN-----------KIKKDIKYSLKKYGVQI---------
LNRKEVYNIFEGKVPLK-----SNVDFTLLYNDKNIVAFRIRENSNIDYVKEPYKNFTANAYFY--------
-------NLINNKFIEVPVL-------NSDSEDKSKSTDILQGDQLTYDSKKGQYIYLAN-----IKSY---

KTGKTQ---SIKTIFNSNLKCISSTLGCETIGVLPATKAN--------------------------------
---------------------------
>CMC-MDR-Ab59_18
--------MKVLNSFAIMIA---CSVSFNICYA--------KENSEWNDYLQDS---------------TQI
NSPIEYIDSYNK----------------YTVLN---PNLKIK------------------------------
---------------------------LVDFGNIN-----------KIKKDIKYSLKKYGVQI---------
LNRKEVYNIFEGKVPLK-----SNVDFTLLYNDKNIVAFRIRENSNIDYVKEPYKNFTANAYFY--------
-------NLINNKFIEVPVL-------NSDSEDKSKSTDILQGDQLTYDSKKGQYIYLAN-----IKSY---
KTGKTQ---SIKTIFNSNLKCISSTLGCETIGVLPATKAN--------------------------------
---------------------------
>KBN10P02143_18
--------MKVLNSFAIMIA---CSVSFNICYA--------KENSEWNDYLQDS---------------TQI
NSPIEYIDSYNK----------------YTVLN---PNLKIK------------------------------
---------------------------LVDFGNIN-----------KIKKDIKYSLKKYGVQI---------
LNRKEVYNIFEGKVPLK-----SNVDFTLLYNDKNIVAFRIRENSNIDYVKEPYKNFTANAYFY--------
-------NLINNKFIEVPVL-------NSDSEDKSKSTDILQGDQLTYDSKKGQYIYLAN-----IKSY---
KTGKTQ---SIKTIFNSNLKCISSTLGCETIGVLPATKAN--------------------------------
---------------------------
>SMC_Paed_Ab_BL01_18
--------MKVLNSFAIMIA---CSVSFNICYA--------KENSEWNDYLQDS---------------TQI
NSPIEYIDSYNK----------------YTVLN---PNLKIK------------------------------
---------------------------LVDFGNIN-----------KIKKDIKYSLKKYGVQI---------
LNRKEVYNIFEGKVPLK-----SNVDFTLLYNDKNIVAFRIRENSNIDYVKEPYKNFTANAYFY--------
-------NLINNKFIEVPVL-------NSDSEDKSKSTDILQGDQLTYDSKKGQYIYLAN-----IKSY---
KTGKTQ---SIKTIFNSNLKCISSTLGCETIGVLPATKAN--------------------------------
---------------------------
>SSA6_18
--------MKVLNSFAIMIA---CSVSFNICYA--------KENSEWNDYLQDS---------------TQI
NSPIEYIDSYNK----------------YTVLN---PNLKIK------------------------------
---------------------------LVDFGNIN-----------KIKKDIKYSLKKYGVQI---------
LNRKEVYNIFEGKVPLK-----SNVDFTLLYNDKNIVAFRIRENSNIDYVKEPYKNFTANAYFY--------
-------NLINNKFIEVPVL-------NSDSEDKSKSTDILQGDQLTYDSKKGQYIYLAN-----IKSY---
KTGKTQ---SIKTIFNSNLKCISSTLGCETIGVLPATKAN--------------------------------

```
---------------------------
>USA2_18
--------MKVLNSFAIMIA---CSVSFNICYA--------KENSEWNDYLQDS---------------TQI
NSPIEYIDSYNK----------------YTVLN---PNLKIK------------------------------
---------------------------LVDFGNIN-----------KIKKDIKYSLKKYGVQI---------
LNRKEVYNIFEGKVPLK-----SNVDFTLLYNDKNIVAFRIRENSNIDYVKEPYKNFTANAYFY--------
-------NLINNKFIEVPVL-------NSDSEDKSKSTDILQGDQLTYDSKKGQYIYLAN-----IKSY---
KTGKTQ---SIKTIFNSNLKCISSTLGCETIGVLPATKAN--------------------------------
---------------------------
>WCHAB005133_18
--------MKVLNSFAIMIA---CSVSFNICYA--------KENSEWNDYLQDS---------------TQI
NSPIEYIDSYNK----------------YTVLN---PNLKIK------------------------------
---------------------------LVDFGNIN-----------KIKKDIKYSLKKYGVQI---------
LNRKEVYNIFEGKVPLK-----SNVDFTLLYNDKNIVAFRIRENSNIDYVKEPYKNFTANAYFY--------
-------NLINNKFIEVPVL-------NSDSEDKSKSTDILQGDQLTYDSKKGQYIYLAN-----IKSY---
KTGKTQ---SIKTIFNSNLKCISSTLGCETIGVLPATKAN--------------------------------
---------------------------
>XDR-BJ83_18
--------MKVLNSFAIMIA---CSVSFNICYA--------KENSEWNDYLQDS---------------TQI
NSPIEYIDSYNK----------------YTVLN---PNLKIK------------------------------
---------------------------LVDFGNIN-----------KIKKDIKYSLKKYGVQI---------
LNRKEVYNIFEGKVPLK-----SNVDFTLLYNDKNIVAFRIRENSNIDYVKEPYKNFTANAYFY--------
-------NLINNKFIEVPVL-------NSDSEDKSKSTDILQGDQLTYDSKKGQYIYLAN-----IKSY---
KTGKTQ---SIKTIFNSNLKCISSTLGCETIGVLPATKAN--------------------------------
---------------------------
>XH859_18
--------MKVLNSFAIMIA---CSVSFNICYA--------KENSEWNDYLQDS---------------TQI
NSPIEYIDSYNK----------------YTVLN---PNLKIK------------------------------
---------------------------LVDFGNIN-----------KIKKDIKYSLKKYGVQI---------
LNRKEVYNIFEGKVPLK-----SNVDFTLLYNDKNIVAFRIRENSNIDYVKEPYKNFTANAYFY--------
-------NLINNKFIEVPVL-------NSDSEDKSKSTDILQGDQLTYDSKKGQYIYLAN-----IKSY---
KTGKTQ---SIKTIFNSNLKCISSTLGCETIGVLPATKAN--------------------------------
---------------------------
```

```
>YU-R612_18
--------MKVLNSFAIMIA---CSVSFNICYA--------KENSEWNDYLQDS---------------TQI
NSPIEYIDSYNK----------------YTVLN---PNLKIK------------------------------
---------------------------LVDFGNIN-----------KIKKDIKYSLKKYGVQI---------
LNRKEVYNIFEGKVPLK-----SNVDFTLLYNDKNIVAFRIRENSNIDYVKEPYKNFTANAYFY--------
-------NLINNKFIEVPVL-------NSDSEDKSKSTDILQGDQLTYDSKKGQYIYLAN-----IKSY---
KTGKTQ---SIKTIFNSNLKCISSTLGCETIGVLPATKAN--------------------------------
---------------------------
>MDR-TJ_18
--------MKVLNSFAIMIA---CSVSFNICYA--------KENSEWNDYLQDS---------------TQI
NSPIEYIDSYNK----------------YTVLN---PNLKIK------------------------------
---------------------------LVDFGNIN-----------KIKKDIKYSLKKYGVQI---------
LNRKEVYNIFEGKVPLK-----SNVDFTLLYNDKNIVAFRIRENSNIDYVKEPYKNFTANAYFY--------
-------NLINNKFIEVPVL-------NSDSEDKSKSTDILQGDQLTYDSKKGQYIYLAN-----IKSY---
KTGKTQ---SIKTIFNSNLKCISSTLGCETIGVLPATKAN--------------------------------
---------------------------
>MDR-ZJ06_18
--------MKVLNSFAIMIA---CSVSFNICYA--------KENSEWNDYLQDS---------------TQI
NSPIEYIDSYNK----------------YTVLN---PNLKIK------------------------------
---------------------------LVDFGNIN-----------KIKKDIKYSLKKYGVQI---------
LNRKEVYNIFEGKVPLK-----SNVDFTLLYNDKNIVAFRIRENSNIDYVKEPYKNFTANAYFY--------
-------NLINNKFIEVPVL-------NSDSEDKSKSTDILQGDQLTYDSKKGQYIYLAN-----IKSY---
KTGKTQ---SIKTIFNSNLKCISSTLGCETIGVLPATKAN--------------------------------
---------------------------
>KAB05_18
--------MKVLNSFAIMIA---CSVSFNICYA--------KENSEWNDYLQDS---------------TQI
NSPIEYIDSYNK----------------YTVLN---PNLKIK------------------------------
---------------------------LVDFGNIN-----------KIKKDIKYSLKKYGVQI---------
LNRKEVYNIFEGKVPLK-----SNVDFTLLYNDKNIVAFRIRENSNIDYVKEPYKNFTANAYFY--------
-------NLINNKFIEVPVL-------NSDSEDKSKSTDILQGDQLTYDSKKGQYIYLAN-----IKSY---
KTGKTQ---SIKTIFNSNLKCISSTLGCETIGVLPATKAN--------------------------------
---------------------------
>SSMA17_18
```

--------MKVLNSFAIMIA---CSVSFNICYA--------KENSEWNDYLQDS---------------TQI
NSPIEYIDSYNK----------------YTVLN---PNLKIK------------------------------
---------------------------LVDFGNIN-----------KIKKDIKYSLKKYGVQI---------
LNRKEVYNIFEGKVPLK-----SNVDFTLLYNDKNIVAFRIRENSNIDYVKEPYKNFTANAYFY--------
-------NLINNKFIEVPVL-------NSDSEDKSKSTDILQGDQLTYDSKKGQYIYLAN-----IKSY---
KTGKTQ---SIKTIFNSNLKCISSTLGCETIGVLPATKAN--------------------------------
---------------------------
>15A5_18
--------MKVLNSFAIMIA---CSVSFNICYA--------KENSEWNDYLQDS---------------TQI
NSPIEYIDSYNK----------------YTVLN---PNLKIK------------------------------
---------------------------LVDFGNIN-----------KIKKDIKYSLKKYGVQI---------
LNRKEVYNIFEGKVPLK-----SNVDFTLLYNDKNIVAFRIRENSNIDYVKEPYKNFTANAYFY--------
-------NLINNKFIEVPVL-------NSDSEDKSKSTDILQGDQLTYDSKKGQYIYLAN-----IKSY---
KTGKTQ---SIKTIFNSNLKCISSTLGCETIGVLPATKAN--------------------------------
---------------------------
>3027STDY5784958_18
--------MKVLNSFAIMIA---CSVSFNICYA--------KENSEWNDYLQDS---------------TQI
NSPIEYIDSYNK----------------YTVLN---PNLKIK------------------------------
---------------------------LVDFGNIN-----------KIKKDIKYSLKKYGVQI---------
LNRKEVYNIFEGKVPLK-----SNVDFTLLYNDKNIVAFRIRENSNIDYVKEPYKNFTANAYFY--------
-------NLINNKFIEVPVL-------NSDSEDKSKSTDILQGDQLTYDSKKGQYIYLAN-----IKSY---
KTGKTQ---SIKTIFNSNLKCISSTLGCETIGVLPATKAN--------------------------------
---------------------------
>AB07_18
--------MKVLNSFAIMIA---CSVSFNICYA--------KENSEWNDYLQDS---------------TQI
NSPIEYIDSYNK----------------YTVLN---PNLKIK------------------------------
---------------------------LVDFGNIN-----------KIKKDIKYSLKKYGVQI---------
LNRKEVYNIFEGKVPLK-----SNVDFTLLYNDKNIVAFRIRENSNIDYVKEPYKNFTANAYFY--------
-------NLINNKFIEVPVL-------NSDSEDKSKSTDILQGDQLTYDSKKGQYIYLAN-----IKSY---
KTGKTQ---SIKTIFNSNLKCISSTLGCETIGVLPATKAN--------------------------------
---------------------------
>Ab4568_18
--------MKVLNSFAIMIA---CSVSFNICYA--------KENSEWNDYLQDS---------------TQI

NSPIEYIDSYNK----------------YTVLN---PNLKIK------------------------------
---------------------------LVDFGNIN-----------KIKKDIKYSLKKYGVQI---------
LNRKEVYNIFEGKVPLK-----SNVDFTLLYNDKNIVAFRIRENSNIDYVKEPYKNFTANAYFY--------
-------NLINNKFIEVPVL-------NSDSEDKSKSTDILQGDQLTYDSKKGQYIYLAN-----IKSY---
KTGKTQ---SIKTIFNSNLKCISSTLGCETIGVLPATKAN--------------------------------
---------------------------
>Ab4977_18
--------MKVLNSFAIMIA---CSVSFNICYA--------KENSEWNDYLQDS---------------TQI
NSPIEYIDSYNK----------------YTVLN---PNLKIK------------------------------
---------------------------LVDFGNIN-----------KIKKDIKYSLKKYGVQI---------
LNRKEVYNIFEGKVPLK-----SNVDFTLLYNDKNIVAFRIRENSNIDYVKEPYKNFTANAYFY--------
-------NLINNKFIEVPVL-------NSDSEDKSKSTDILQGDQLTYDSKKGQYIYLAN-----IKSY---
KTGKTQ---SIKTIFNSNLKCISSTLGCETIGVLPATKAN--------------------------------
---------------------------
>AC29_18
--------MKVLNSFAIMIA---CSVSFNICYA--------KENSEWNDYLQDS---------------TQI
NSPIEYIDSYNK----------------YTVLN---PNLKIK------------------------------
---------------------------LVDFGNIN-----------KIKKDIKYSLKKYGVQI---------
LNRKEVYNIFEGKVPLK-----SNVDFTLLYNDKNIVAFRIRENSNIDYVKEPYKNFTANAYFY--------
-------NLINNKFIEVPVL-------NSDSEDKSKSTDILQGDQLTYDSKKGQYIYLAN-----IKSY---
KTGKTQ---SIKTIFNSNLKCISSTLGCETIGVLPATKAN--------------------------------
---------------------------
>AC30_18
--------MKVLNSFAIMIA---CSVSFNICYA--------KENSEWNDYLQDS---------------TQI
NSPIEYIDSYNK----------------YTVLN---PNLKIK------------------------------
---------------------------LVDFGNIN-----------KIKKDIKYSLKKYGVQI---------
LNRKEVYNIFEGKVPLK-----SNVDFTLLYNDKNIVAFRIRENSNIDYVKEPYKNFTANAYFY--------
-------NLINNKFIEVPVL-------NSDSEDKSKSTDILQGDQLTYDSKKGQYIYLAN-----IKSY---
KTGKTQ---SIKTIFNSNLKCISSTLGCETIGVLPATKAN--------------------------------
---------------------------
>AF-673_18
--------MKVLNSFAIMIA---CSVSFNICYA--------KENSEWNDYLQDS---------------TQI
NSPIEYIDSYNK----------------YTVLN---PNLKIK------------------------------

---------------------------LVDFGNIN-----------KIKKDIKYSLKKYGVQI---------
LNRKEVYNIFEGKVPLK-----SNVDFTLLYNDKNIVAFRIRENSNIDYVKEPYKNFTANAYFY--------
-------NLINNKFIEVPVL-------NSDSEDKSKSTDILQGDQLTYDSKKGQYIYLAN-----IKSY---
KTGKTQ---SIKTIFNSNLKCISSTLGCETIGVLPATKAN--------------------------------
---------------------------
>AR_0102_18
--------MKVLNSFAIMIA---CSVSFNICYA--------KENSEWNDYLQDS---------------TQI
NSPIEYIDSYNK----------------YTVLN---PNLKIK------------------------------
---------------------------LVDFGNIN-----------KIKKDIKYSLKKYGVQI---------
LNRKEVYNIFEGKVPLK-----SNVDFTLLYNDKNIVAFRIRENSNIDYVKEPYKNFTANAYFY--------
-------NLINNKFIEVPVL-------NSDSEDKSKSTDILQGDQLTYDSKKGQYIYLAN-----IKSY---
KTGKTQ---SIKTIFNSNLKCISSTLGCETIGVLPATKAN--------------------------------
---------------------------
>AYP-A2_18
--------MKVLNSFAIMIA---CSVSFNICYA--------KENSEWNDYLQDS---------------TQI
NSPIEYIDSYNK----------------YTVLN---PNLKIK------------------------------
---------------------------LVDFGNIN-----------KIKKDIKYSLKKYGVQI---------
LNRKEVYNIFEGKVPLK-----SNVDFTLLYNDKNIVAFRIRENSNIDYVKEPYKNFTANAYFY--------
-------NLINNKFIEVPVL-------NSDSEDKSKSTDILQGDQLTYDSKKGQYIYLAN-----IKSY---
KTGKTQ---SIKTIFNSNLKCISSTLGCETIGVLPATKAN--------------------------------
---------------------------
>CBA7_18
--------MKVLNSFAIMIA---CSVSFNICYA--------KENSEWNDYLQDS---------------TQI
NSPIEYIDSYNK----------------YTVLN---PNLKIK------------------------------
---------------------------LVDFGNIN-----------KIKKDIKYSLKKYGVQI---------
LNRKEVYNIFEGKVPLK-----SNVDFTLLYNDKNIVAFRIRENSNIDYVKEPYKNFTANAYFY--------
-------NLINNKFIEVPVL-------NSDSEDKSKSTDILQGDQLTYDSKKGQYIYLAN-----IKSY---
KTGKTQ---SIKTIFNSNLKCISSTLGCETIGVLPATKAN--------------------------------
---------------------------
>CMC-CR-MDR-Ab4_18
--------MKVLNSFAIMIA---CSVSFNICYA--------KENSEWNDYLQDS---------------TQI
NSPIEYIDSYNK----------------YTVLN---PNLKIK------------------------------
---------------------------LVDFGNIN-----------KIKKDIKYSLKKYGVQI---------

LNRKEVYNIFEGKVPLK-----SNVDFTLLYNDKNIVAFRIRENSNIDYVKEPYKNFTANAYFY--------
-------NLINNKFIEVPVL-------NSDSEDKSKSTDILQGDQLTYDSKKGQYIYLAN-----IKSY---
KTGKTQ---SIKTIFNSNLKCISSTLGCETIGVLPATKAN--------------------------------
---------------------------
>CMC-CR-MDR-Ab66_18
--------MKVLNSFAIMIA---CSVSFNICYA--------KENSEWNDYLQDS---------------TQI
NSPIEYIDSYNK----------------YTVLN---PNLKIK------------------------------
---------------------------LVDFGNIN-----------KIKKDIKYSLKKYGVQI---------
LNRKEVYNIFEGKVPLK-----SNVDFTLLYNDKNIVAFRIRENSNIDYVKEPYKNFTANAYFY--------
-------NLINNKFIEVPVL-------NSDSEDKSKSTDILQGDQLTYDSKKGQYIYLAN-----IKSY---
KTGKTQ---SIKTIFNSNLKCISSTLGCETIGVLPATKAN--------------------------------
---------------------------
>HRAB-85_18
--------MKVLNSFAIMIA---CSVSFNICYA--------KENSEWNDYLQDS---------------TQI
NSPIEYIDSYNK----------------YTVLN---PNLKIK------------------------------
---------------------------LVDFGNIN-----------KIKKDIKYSLKKYGVQI---------
LNRKEVYNIFEGKVPLK-----SNVDFTLLYNDKNIVAFRIRENSNIDYVKEPYKNFTANAYFY--------
-------NLINNKFIEVPVL-------NSDSEDKSKSTDILQGDQLTYDSKKGQYIYLAN-----IKSY---
KTGKTQ---SIKTIFNSNLKCISSTLGCETIGVLPATKAN--------------------------------
---------------------------
>JBA13_18
--------MKVLNSFAIMIA---CSVSFNICYA--------KENSEWNDYLQDS---------------TQI
NSPIEYIDSYNK----------------YTVLN---PNLKIK------------------------------
---------------------------LVDFGNIN-----------KIKKDIKYSLKKYGVQI---------
LNRKEVYNIFEGKVPLK-----SNVDFTLLYNDKNIVAFRIRENSNIDYVKEPYKNFTANAYFY--------
-------NLINNKFIEVPVL-------NSDSEDKSKSTDILQGDQLTYDSKKGQYIYLAN-----IKSY---
KTGKTQ---SIKTIFNSNLKCISSTLGCETIGVLPATKAN--------------------------------
---------------------------
>KAB02_18
--------MKVLNSFAIMIA---CSVSFNICYA--------KENSEWNDYLQDS---------------TQI
NSPIEYIDSYNK----------------YTVLN---PNLKIK------------------------------
---------------------------LVDFGNIN-----------KIKKDIKYSLKKYGVQI---------
LNRKEVYNIFEGKVPLK-----SNVDFTLLYNDKNIVAFRIRENSNIDYVKEPYKNFTANAYFY--------

-------NLINNKFIEVPVL-------NSDSEDKSKSTDILQGDQLTYDSKKGQYIYLAN-----IKSY---
KTGKTQ---SIKTIFNSNLKCISSTLGCETIGVLPATKAN--------------------------------
---------------------------
>KAB04_18
--------MKVLNSFAIMIA---CSVSFNICYA--------KENSEWNDYLQDS---------------TQI
NSPIEYIDSYNK----------------YTVLN---PNLKIK------------------------------
---------------------------LVDFGNIN-----------KIKKDIKYSLKKYGVQI---------
LNRKEVYNIFEGKVPLK-----SNVDFTLLYNDKNIVAFRIRENSNIDYVKEPYKNFTANAYFY--------
-------NLINNKFIEVPVL-------NSDSEDKSKSTDILQGDQLTYDSKKGQYIYLAN-----IKSY---
KTGKTQ---SIKTIFNSNLKCISSTLGCETIGVLPATKAN--------------------------------
---------------------------
>KAB06_18
--------MKVLNSFAIMIA---CSVSFNICYA--------KENSEWNDYLQDS---------------TQI
NSPIEYIDSYNK----------------YTVLN---PNLKIK------------------------------
---------------------------LVDFGNIN-----------KIKKDIKYSLKKYGVQI---------
LNRKEVYNIFEGKVPLK-----SNVDFTLLYNDKNIVAFRIRENSNIDYVKEPYKNFTANAYFY--------
-------NLINNKFIEVPVL-------NSDSEDKSKSTDILQGDQLTYDSKKGQYIYLAN-----IKSY---
KTGKTQ---SIKTIFNSNLKCISSTLGCETIGVLPATKAN--------------------------------
---------------------------
>KAB07_18
--------MKVLNSFAIMIA---CSVSFNICYA--------KENSEWNDYLQDS---------------TQI
NSPIEYIDSYNK----------------YTVLN---PNLKIK------------------------------
---------------------------LVDFGNIN-----------KIKKDIKYSLKKYGVQI---------
LNRKEVYNIFEGKVPLK-----SNVDFTLLYNDKNIVAFRIRENSNIDYVKEPYKNFTANAYFY--------
-------NLINNKFIEVPVL-------NSDSEDKSKSTDILQGDQLTYDSKKGQYIYLAN-----IKSY---
KTGKTQ---SIKTIFNSNLKCISSTLGCETIGVLPATKAN--------------------------------
---------------------------
>KAB08_18
--------MKVLNSFAIMIA---CSVSFNICYA--------KENSEWNDYLQDS---------------TQI
NSPIEYIDSYNK----------------YTVLN---PNLKIK------------------------------
---------------------------LVDFGNIN-----------KIKKDIKYSLKKYGVQI---------
LNRKEVYNIFEGKVPLK-----SNVDFTLLYNDKNIVAFRIRENSNIDYVKEPYKNFTANAYFY--------
-------NLINNKFIEVPVL-------NSDSEDKSKSTDILQGDQLTYDSKKGQYIYLAN-----IKSY---

KTGKTQ---SIKTIFNSNLKCISSTLGCETIGVLPATKAN--------------------------------
---------------------------
>SSA12_18
--------MKVLNSFAIMIA---CSVSFNICYA--------KENSEWNDYLQDS---------------TQI
NSPIEYIDSYNK----------------YTVLN---PNLKIK------------------------------
---------------------------LVDFGNIN-----------KIKKDIKYSLKKYGVQI---------
LNRKEVYNIFEGKVPLK-----SNVDFTLLYNDKNIVAFRIRENSNIDYVKEPYKNFTANAYFY--------
-------NLINNKFIEVPVL-------NSDSEDKSKSTDILQGDQLTYDSKKGQYIYLAN-----IKSY---
KTGKTQ---SIKTIFNSNLKCISSTLGCETIGVLPATKAN--------------------------------
---------------------------
>XH386_18
--------MKVLNSFAIMIA---CSVSFNICYA--------KENSEWNDYLQDS---------------TQI
NSPIEYIDSYNK----------------YTVLN---PNLKIK------------------------------
---------------------------LVDFGNIN-----------KIKKDIKYSLKKYGVQI---------
LNRKEVYNIFEGKVPLK-----SNVDFTLLYNDKNIVAFRIRENSNIDYVKEPYKNFTANAYFY--------
-------NLINNKFIEVPVL-------NSDSEDKSKSTDILQGDQLTYDSKKGQYIYLAN-----IKSY---
KTGKTQ---SIKTIFNSNLKCISSTLGCETIGVLPATKAN--------------------------------
---------------------------
>XH856_18
--------MKVLNSFAIMIA---CSVSFNICYA--------KENSEWNDYLQDS---------------TQI
NSPIEYIDSYNK----------------YTVLN---PNLKIK------------------------------
---------------------------LVDFGNIN-----------KIKKDIKYSLKKYGVQI---------
LNRKEVYNIFEGKVPLK-----SNVDFTLLYNDKNIVAFRIRENSNIDYVKEPYKNFTANAYFY--------
-------NLINNKFIEVPVL-------NSDSEDKSKSTDILQGDQLTYDSKKGQYIYLAN-----IKSY---
KTGKTQ---SIKTIFNSNLKCISSTLGCETIGVLPATKAN--------------------------------
---------------------------
>AR_0078_29
----------MFGKYVFYTGWLLATLSLSVSAC----------ADNSKVNCNTN----N----------VYG
DL-----KCLEK----------------ENKKL---INQLDNKKNKERKDYNQWIKEIKNKCEGKISYAS--
---------------------------GEGAGLIK----------NQCYREEYLSRLSFINTG-------KV
KKSDTNIHGLELTYLPY-----NSNDHLKCIMKKEV--NSCSKVNLVKSSNLIKYYPF--ISPA-YGDSIVL
PETDDSKQIIVSPFEDNED------------------------EGPQLEISIINAFGVVEQKTISANKN---
VIIDKNYNLIYKEGNKEKILKLK-------------------------------------------------

```
---------------------------
>AF-401_30
----------------IF-----LLSSLFLIGC---------------------------------------
-S-----ESFSN------------------------------------------------------------
------------------------------------------------------------------------
FTIDNVVKSNEKLNFSI-----NFKNNEVLDKVK-----ESSSKKIVCKNTANNNQML--DAYIDKIEN---
-------NKIDVSVEFCANN-----------------------DNRSCEPIDISSVKKIN-----LECQ---
AIFSAMIGNVSKSEKFPIIWEKK-------------------------------------------------
---------------------------
>AYE_16
--------------MKYFVL---SATQSSLCPI---------------SLVDDA----V----------LYM
NK-----EQASQ----------------NIECG------------------SLPWYSYKTSKNCD-------
---------------------------LPIEGVLI-----------LKSKKIEISIRNINENI---------
YVVDEIFKNIFGKYINS-----DFIRVEVLSE-------SLEKINNKNYYIFRFNQIF--DFKN-----VL-
-------DLNKSTYRLDNDF---------------------------LVLEKAEFLSDLD-----IDIL---
KIDNMD---SAQDTFFISEFVKKEIEKNKYQGINIFELTLAQ----------------WRDSDDFSFMFLSE
D---------------EVNKFVWPI--
>A1_16
--------------MKYFVL---SATQSSLCPI---------------SLVDDA----V----------LYM
NK-----EQASQ----------------NIECG------------------SLPWYSYKTSKNCD-------
---------------------------LPIEGVLI-----------LKSKKIEISIRNINENI---------
YVVDEIFKNIFGKYINS-----DFIRVEVLSE-------SLEKINNKNYYIFRFNQIF--DFKN-----VL-
-------DLNKSTYRLDNDF---------------------------LVLEKAEFLSDLD-----IDIL---
KIDNMD---SAQDTFFISEFVKKEIEKNKYQGINIFELTLAQ----------------WRDSDDFSFMFLSE
D---------------EVNKFVWPI--
>A388_16
--------------MKYFVL---SATQSSLCPI---------------SLVDDA----V----------LYM
NK-----EQASQ----------------NIECG------------------SLPWYSYKTSKNCD-------
---------------------------LPIEGVLI-----------LKSKKIEISIRNINENI---------
YVVDEIFKNIFGKYINS-----DFIRVEVLSE-------SLEKINNKNYYIFRFNQIF--DFKN-----VL-
-------DLNKSTYRLDNDF---------------------------LVLEKAEFLSDLD-----IDIL---
KIDNMD---SAQDTFFISEFVKKEIEKNKYQGINIFELTLAQ----------------WRDSDDFSFMFLSE
D---------------EVNKFVWPI--
```

```
>A85_16
--------------MKYFVL---SATQSSLCPI---------------SLVDDA----V----------LYM
NK-----EQASQ----------------NIECG------------------SLPWYSYKTSKNCD-------
---------------------------LPIEGVLI-----------LKSKKIEISIRNINENI---------
YVVDEIFKNIFGKYINS-----DFIRVEVLSE-------SLEKINNKNYYIFRFNQIF--DFKN-----VL-
-------DLNKSTYRLDNDF---------------------------LVLEKAEFLSDLD-----IDIL---
KIDNMD---SAQDTFFISEFVKKEIEKNKYQGINIFELTLAQ----------------WRDSDDFSFMFLSE
D---------------EVNKFVWPI--
>AB307-0294_16
--------------MKYFVL---SATQSSLCPI---------------SLVDDA----V----------LYM
NK-----EQASQ----------------NIECG------------------SLPWYSYKTSKNCD-------
---------------------------LPIEGVLI-----------LKSKKIEISIRNINENI---------
YVVDEIFKNIFGKYINS-----DFIRVEVLSE-------SLEKINNKNYYIFRFNQIF--DFKN-----VL-
-------DLNKSTYRLDNDF---------------------------LVLEKAEFLSDLD-----IDIL---
KIDNMD---SAQDTFFISEFVKKEIEKNKYQGINIFELTLAQ----------------WRDSDDFSFMFLSE
D---------------EVNKFVWPI--
>AB5075-UW_16
--------------MKYFVL---SATQSSLCPI---------------SLVDDA----V----------LYM
NK-----EQASQ----------------NIECG------------------SLPWYSYKTSKNCD-------
---------------------------LPIEGVLI-----------LKSKKIEISIRNINENI---------
YVVDEIFKNIFGKYINS-----DFIRVEVLSE-------SLEKINNKNYYIFRFNQIF--DFKN-----VL-
-------DLNKSTYRLDNDF---------------------------LVLEKAEFLSDLD-----IDIL---
KIDNMD---SAQDTFFISEFVKKEIEKNKYQGINIFELTLAQ----------------WRDSDDFSFMFLSE
D---------------EVNKFVWPI--
>AR_0083_16
--------------MKYFVL---SATQSSLCPI---------------SLVDDA----V----------LYM
NK-----EQASQ----------------NIECG------------------SLPWYSYKTSKNCD-------
---------------------------LPIEGVLI-----------LKSKKIEISIRNINENI---------
YVVDEIFKNIFGKYINS-----DFIRVEVLSE-------SLEKINNKNYYIFRFNQIF--DFKN-----VL-
-------DLNKSTYRLDNDF---------------------------LVLEKAEFLSDLD-----IDIL---
KIDNMD---SAQDTFFISEFVKKEIEKNKYQGINIFELTLAQ----------------WRDSDDFSFMFLSE
D---------------EVNKFVWPI--
>D36_16
```

--------------MKYFVL---SATQSSLCPI---------------SLVDDA----V----------LYM
NK-----EQASQ----------------NIECG------------------SLPWYSYKTSKNCD-------
---------------------------LPIEGVLI-----------LKSKKIEISIRNINENI---------
YVVDEIFKNIFGKYINS-----DFIRVEVLSE-------SLEKINNKNYYIFRFNQIF--DFKN-----VL-
-------DLNKSTYRLDNDF---------------------------LVLEKAEFLSDLD-----IDIL---
KIDNMD---SAQDTFFISEFVKKEIEKNKYQGINIFELTLAQ----------------WRDSDDFSFMFLSE
D---------------EVNKFVWPI--
>USA15_16
--------------MKYFVL---SATQSSLCPI---------------SLVDDA----V----------LYM
NK-----EQASQ----------------NIECG------------------SLPWYSYKTSKNCD-------
---------------------------LPIEGVLI-----------LKSKKIEISIRNINENI---------
YVVDEIFKNIFGKYINS-----DFIRVEVLSE-------SLEKINNKNYYIFRFNQIF--DFKN-----VL-
-------DLNKSTYRLDNDF---------------------------LVLEKAEFLSDLD-----IDIL---
KIDNMD---SAQDTFFISEFVKKEIEKNKYQGINIFELTLAQ----------------WRDSDDFSFMFLSE
D---------------EVNKFVWPI--
>WCHAB005078_16
--------------MKYFVL---SATQSSLCPI---------------SLVDDA----V----------LYM
NK-----EQASQ----------------NIECG------------------SLPWYSYKTSKNCD-------
---------------------------LPIEGVLI-----------LKSKKIEISIRNINENI---------
YVVDEIFKNIFGKYINS-----DFIRVEVLSE-------SLEKINNKNYYIFRFNQIF--DFKN-----VL-
-------DLNKSTYRLDNDF---------------------------LVLEKAEFLSDLD-----IDIL---
KIDNMD---SAQDTFFISEFVKKEIEKNKYQGINIFELTLAQ----------------WRDSDDFSFMFLSE
D---------------EVNKFVWPI--
>3207_1
--------------MKYFLL---TLLSITLSAC-----------NPFINNEKAS-----------------N
NN-----AEIKS---------------LTYSRL---------------------------------------
---------------------------DGMSGDIF--------------------KFNLETND---------
-DLNKIYQENNYKYSHF-----KCDNIKNYF-------VTGAISVEGEKLKKGKYTSS--GYFKVCEDESM-
-------NVCIDKNQLEKLL----------------------TSNMSCRVVFGGLLQSSKV----VADN---
ILISKE---AIRKSNFQ-------------------------------------------------------
---------------------------
>AB042_1
--------------MKYFLL---TLLSITLSAC-----------NPFINNEKAS-----------------N

```
NN-----AEIKS---------------LTYSRL---------------------------------------
---------------------------DGMSGDIF--------------------KFNLETND---------
-DLNKIYQENNYKYSHF-----KCDNIKNYFV-------TGAISVEGEKLKKGKYTSS--GYFKVCEDESM-
-------NVCIDKNQLEKLL----------------------TSNMSCRVVFGGLLQSSKV----VADN---
ILISKE---AIRKSNFQ-------------------------------------------------------
---------------------------
>AR_0088_1
--------------MKYFLL---TLLSITLSAC-----------NPFINNEKAS-----------------N
NN-----AEIKS---------------LTYSRL---------------------------------------
---------------------------DGMSGDIF--------------------KFNLETND---------
-DLNKIYQENNYKYSHF-----KCDNIKNYFV-------TGAISVEGEKLKKGKYTSS--GYFKVCEDESM-
-------NVCIDKNQLEKLL----------------------TSNMSCRVVFGGLLQSSKV----VADN---
ILISKE---AIRKSNFQ-------------------------------------------------------
---------------------------
>ATCC17978-mff_1
--------------MKYFLL---TLLSITLSAC-----------NPFINNEKAS-----------------N
NN-----AEIKS---------------LTYSRL---------------------------------------
---------------------------DGMSGDIF--------------------KFNLETND---------
-DLNKIYQENNYKYSHF-----KCDNIKNYFV-------TGAISVEGEKLKKGKYTSS--GYFKVCEDESM-
-------NVCIDKNQLEKLL----------------------TSNMSCRVVFGGLLQSSKV----VADN---
ILISKE---AIRKSNFQ-------------------------------------------------------
---------------------------
>HWAB8_1
--------------MKYFLL---TLLSITLSAC-----------NPFINNEKAS-----------------N
NN-----AEIKS---------------LTYSRL---------------------------------------
---------------------------DGMSGDIF--------------------KFNLETND---------
-DLNKIYQENNYKYSHF-----KCDNIKNYFV-------TGAISVEGEKLKKGKYTSS--GYFKVCEDESM-
-------NVCIDKNQLEKLL----------------------TSNMSCRVVFGGLLQSSKV----VADN---
ILISKE---AIRKSNFQ-------------------------------------------------------
---------------------------
>ATCC17978-mff_4
----------MIKYKLCLAL---LLGASTVANA------------EFNIENNPN----QPVYIKLGYQNVLN
SS-----FIYTD----------VSKNVDLKKNI---VTVPYGEVTVLLNPVLNNNNEIKGKDYYFINYQPKI
```

LLGKIQYKLPFVISDVGAIGTSI-IYNDNNISIFS--------IESYANPVSNNNSKKFKSDI---------
YIFNKKNLGIYNPSFLS-----INVKLKSLNDASMK--FTKLSSIKYNKNDKSYQVVY--DFQK-VTEDFT-
-------STGKVNYEKFPIQ---------------------------YSFSLIPDKNKPF-----LIKD---
IIEKRK---GDKNIISKIENNDMYSYSLK-------------------------------------------
---------------------------
>3207_4
----------MIKYKLCLAL---LLGASTVANA------------EFNIENNPN----QPVYIKLGYQNVLN
SS-----FIYTD----------VSKNVDLKKNI---VTVPYGEVTVLLNPVLNNNNEIKGKDYYFINYQPKI
LLGKIQYKLPFVISDVGAIGTSI-IYNDNNISIFS--------IESYANPVSNNNSKKFKSDI---------
YIFNKKNLGIYNPSFLS-----INVKLKSLNDASMK--FTKLSSIKYNKNDKSYQVVY--DFQK-VTEDFT-
-------STGKVNYEKFPIQ---------------------------YSFSLIPDKNKPF-----LIKD---
IIEKRK---GDKNIISKIENNDMYSYSLK-------------------------------------------
---------------------------
>AB031_26
-------------MLTMLVE---VIMSVFI------------------------------------------
-------ANFKA------------------------------------------------------------
----------------------------SEHPIIN-------------------------------------
IIVRGLLVAVIGFFIAL-----------------------FSSIVEGWDFSIGFALIF--SLAI--------
-------GMLVSVFLLVVEI-------------------------------FFNY----------IDKK---
------------------------------------------------------------------------
---------------------------
>AB031_27
--------------MKLEQI---ETADMDVKSI------------------------------------KIY
GD-----KFY--------------------------------------------------------------
---------------------------ILFSAVYD-------------------------------------
TCLKQYIKNVELVFSNW----------------------SSFKIEKYDGNIEEYKQLD--LLEI--------
-------NQSKDLFTLIQII-----------------------HVSEDVVELEGFNDDGE-----WNKV---
VFFNPS---YSLNIIDI-------------------------------------------------------
---------------------------
>ab736_10
----------MKNIFKLVLS---AALLPLMVAC-----QAQTVDLTFLVYNYST------------------
-------EGLGE------------------------------------------------------------
---------------------------IQVNGKGS------------------TIADAAERLG---------

-SIGEAGTACCVHLVEN-----SDTADVSFYT-------DRGDGYKQYHIKVPVENLK--DTPR---------
-------SYAVLHYFPNNTG------------------------VIEVSMRRPSFRKDLF-------DK---
ALGNKA---KNIQLDSPTMWRTISENQAARMQFPD-------------------------------------
---------------------------
>ATCC19606_10
----------MKNIFKLVLS---AALLPLMVAC-----QAQTVDLTFLVYNYST------------------
-------EGLGE------------------------------------------------------------
---------------------------IQVNGKGS------------------TIADAAERLG---------
-SIGEAGTACCVHLVEN-----SDTADVSFYT-------DRGDGYKQYHIKVPVENLK--DTPR---------
-------SYAVLHYFPNNTG------------------------VIEVSMRRPSFRKDLF-------DK---
ALGNKA---KNIQLDSPTMWRTISENQAARMQFPD-------------------------------------
---------------------------
>ZW85-1_19
-----------MSQFEIRPQ---EIYLLERYSSPAYFEELVIAFKNMLDAAENALELFM----------QDL
PY-----DYRDR----------------HISEQ---PDVVWGEHVLPNFRSTMQSLHYGYKQLLDGDLSA--
---------------------------LQFAGNVV--------------------TDFRNQRV---------
DYFPDWMDEANLAVFDE-----EQMKASRLAS-------NIKATVMGNWVTGNLSHRY--NSQS-RGELNL-
-------PLSLPIYRLNLNV--------------TVKTGETVPEDGIYIPSIQDASAQLL-----LKGH---
DAIEALVGLSRSGLQYAREESTTWLLVKRIADEGGSVETIQAENLKGYAGQSCQQSGHWWSPANQSQTRYFE
KGEIFPEIPNNAWGETIWYLEVTNKAE
>ATCC19606_7
-------------------M---ITTCLFLVAC-----SKPASNNRMNQNQDVI----A----------LHF
GE-----QGMQD----------------FSKNS----TTPVDRQPAGMNFLSLDWTPPKLGRVRVFSEKSNL
EIENVISVLGTQVARRSNDGIQI----MDINASLH-------------PNEYTTSQEAYTAYT---------
KLVHQINDKQWKQYFLP-----FSARIDKQDNLKHM--TETMGEVIDPTYILNFKEWQ--DVLS-KTN----
-------RLVFNLYNGDVEL-------GISLRRTYKD------DKKEQYMVRYSFENFKY-----AGRN---
AISDSD---KMNSEQLKQAFETEVANNKKARKTEENNMKKEG-------------------------YHIDE
S---------------YIDPDIWPYVQ
>ab736_7
-------------------M---ITTCLFLVAC-----SKPASNNRMNQNQDVI----A----------LHF
GE-----QGMQD----------------FSKNS----TTPVDRQPAGMNFLSLDWTPPKLGRVRVFSEKSNL
EIENVISVLGTQVARRSNDGIQI----MDINASLH-------------PNEYTTSQEAYTAYT---------
KLVHQINDKQWKQYFLP-----FSARIDKQDNLKHM--TETMGEVIDPTYILNFKEWQ--DVLS-KTN----

-------RLVFNLYNGDVEL-------GISLRRTYKD------DKKEQYMVRYSFENFKY-----AGRN---
AISDSD---KMNSEQLKQAFETEVANNKKARKTEENNMKKEG-------------------------YHIDE
S----------------YIDPDIWPYVQ
>ACICU_7
-------------------M---ITTCLFLVAC-----SKPASNNRMNQNQDVI----A----------LHF
GE-----QGMQD----------------FSKNS----TTPVDRQPAGMNFLSLDWTPPKLGRVRVFSEKSNL
EIENVISVLGTQVARRSNDGIQI----MDINASLH-------------PNEYTTSQEAYTAYT---------
KLVHQINDKQWKQYFLP-----FSARIDKQDNLKHM--TETMGEVIDPTYILNFKEWQ--DVLS-KTN----
-------RLVFNLYNGDVEL-------GISLRRTYKD------DKKEQYMVRYSFENFKY-----AGRN---
AISDSD---KMNSEQLKQAFETEVANNKKARKTEENNMKKEG-------------------------YHIDE
S----------------YIDPDIWPYVQ
>KAB06_13
----------MKNYLSLFIC---IITCIFLTSC-KPANNTTQGNKSMNQNQDVI----A----------LHF
GE-----QGMQD----------------FSKNS----TTPVDRQPAGMNFLSLDWTPPKLGRVRVFSEKSNL
EIENVISVLGTQVARRSNDGIQI----MDINASLH-------------PNEYTTSQEAYTA-T---------
KLVHQINDKQWKQYFLP-----FSARIDKQDNLKHM--TETMGEVIDPTYILNFKEWQ--DVLS-KTN----
-------RLVFNLYNGDVEL-------GISLRRTYKD------DKKEQYMVRYSFENFKY-----AGRN---
AISDSD---KMNSEQLKQAFETEVANNKKARKTEENNMKKEG-------------------------YHIDE
S----------------YIDPDIWPYVQ
>LAC-4_13
----------MKNYLSLFIC---IITCIFLTSC-KPANNTTQGNKSMNQNQDVI----A----------LHF
GE-----QGMQD----------------FSKNS----TTPVDRQPAGMNFLSLDWTPPKLGRVRVFSEKSNL
EIENVISVLGTQVARRSNDGIQI----MDINASLH-------------PNEYTTSQEAYTAYT---------
KLVHQINDKQWKQYFLP-----FSARIDKQDNLKHM--TETMGEVIDPTYILNFKEWQ--DVLS-KTN----
-------RLVFNLYNGDVEL-------GISLRRTYKD------DKKEQYMVRYSFENFKY-----AGRN---
AISDSD---KMNSEQLKQAFETEVANNKKARKTEENNMKKEG-------------------------YHIDE
S----------------YIDPDIWPYVQ
>Ab04-mff_13
----------MKNYLSLFIC---IITCIFLTSC-KPANNTTQGNKSMNQNQDVI----A----------LHF
GE-----QGMQD----------------FSKNS----TTPVDRQPAGMNFLSLDWTPPKLGRVRVFSEKSNL
EIENVISVLGTQVARRSNDGIQI----MDINASLH-------------PNEYTTSQEAYTAYT---------
KLVHQINDKQWKQYFLP-----FSARIDKQDNLKHM--TETMGEVIDPTYILNFKEWQ--DVLS-KTN----
-------RLVFNLYNGDVEL-------GISLRRTYKD------DKKEQYMVRYSFENFKY-----AGRN---

AISDSD---KMNSEQLKQAFETEVANNKKARKTEENNMKKEG-------------------------YHIDE
S----------------YIDPDIWPYVQ
>BJAB0715_13
----------MKNYLSLFIC---IITCIFLTSC-KPANNTTQGNKSMNQNQDVI----A----------LHF
GE-----QGMQD----------------FSKNS----TTPVDRQPAGMNFLSLDWTPPKLGRVRVFSEKSNL
EIENVISVLGTQVARRSNDGIQI----MDINASLH-------------PNEYTTSQEAYTAYT---------
KLVHQINDKQWKQYFLP-----FSARIDKQDNLKHM--TETMGEVIDPTYILNFKEWQ--DVLS-KTN----
-------RLVFNLYNGDVEL-------GISLRRTYKD------DKKEQYMVRYSFENFKY-----AGRN---
AISDSD---KMNSEQLKQAFETEVANNKKARKTEENNMKKEG-------------------------YHIDE
S----------------YIDPDIWPYVQ
>D1279779_13
----------MKNYLSLFIC---IITCIFLTSC-KPANNTTQGNKSMNQNQDVI----A----------LHF
GE-----QGMQD----------------FSKNS----TTPVDRQPAGMNFLSLDWTPPKLGRVRVFSEKSNL
EIENVISVLGTQVARRSNDGIQI----MDINASLH-------------PNEYTTSQEAYTAYT---------
KLVHQINDKQWKQYFLP-----FSARIDKQDNLKHM--TETMGEVIDPTYILNFKEWQ--DVLS-KTN----
-------RLVFNLYNGDVEL-------GISLRRTYKD------DKKEQYMVRYSFENFKY-----AGRN---
AISDSD---KMNSEQLKQAFETEVANNKKARKTEENNMKKEG-------------------------YHIDE
S----------------YIDPDIWPYVQ
>KAB02_13
----------MKNYLSLFIC---IITCIFLTSC-KPANNTTQGNKSMNQNQDVI----A----------LHF
GE-----QGMQD----------------FSKNS----TTPVDRQPAGMNFLSLDWTPPKLGRVRVFSEKSNL
EIENVISVLGTQVARRSNDGIQI----MDINASLH-------------PNEYTTSQEAYTAYT---------
KLVHQINDKQWKQYFLP-----FSARIDKQDNLKHM--TETMGEVIDPTYILNFKEWQ--DVLS-KTN----
-------RLVFNLYNGDVEL-------GISLRRTYKD------DKKEQYMVRYSFENFKY-----AGRN---
AISDSD---KMNSEQLKQAFETEVANNKKARKTEENNMKKEG-------------------------YHIDE
S----------------YIDPDIWPYVQ
>KAB05_13
----------MKNYLSLFIC---IITCIFLTSC-KPANNTTQGNKSMNQNQDVI----A----------LHF
GE-----QGMQD----------------FSKNS----TTPVDRQPAGMNFLSLDWTPPKLGRVRVFSEKSNL
EIENVISVLGTQVARRSNDGIQI----MDINASLH-------------PNEYTTSQEAYTAYT---------
KLVHQINDKQWKQYFLP-----FSARIDKQDNLKHM--TETMGEVIDPTYILNFKEWQ--DVLS-KTN----
-------RLVFNLYNGDVEL-------GISLRRTYKD------DKKEQYMVRYSFENFKY-----AGRN---
AISDSD---KMNSEQLKQAFETEVANNKKARKTEENNMKKEG-------------------------YHIDE

S---------------YIDPDIWPYVQ
>MDR-TJ_13
----------MKNYLSLFIC---IITCIFLTSC-KPANNTTQGNKSMNQNQDVI----A----------LHF
GE-----QGMQD----------------FSKNS----TTPVDRQPAGMNFLSLDWTPPKLGRVRVFSEKSNL
EIENVISVLGTQVARRSNDGIQI----MDINASLH-------------PNEYTTSQEAYTAYT---------
KLVHQINDKQWKQYFLP-----FSARIDKQDNLKHM--TETMGEVIDPTYILNFKEWQ--DVLS-KTN----
-------RLVFNLYNGDVEL-------GISLRRTYKD------DKKEQYMVRYSFENFKY-----AGRN---
AISDSD---KMNSEQLKQAFETEVANNKKARKTEENNMKKEG-------------------------YHIDE
S---------------YIDPDIWPYVQ
>R2090_13
----------MKNYLSLFIC---IITCIFLTSC-KPANNTTQGNKSMNQNQDVI----A----------LHF
GE-----QGMQD----------------FSKNS----TTPVDRQPAGMNFLSLDWTPPKLGRVRVFSEKSNL
EIENVISVLGTQVARRSNDGIQI----MDINASLH-------------PNEYTTSQEAYTAYT---------
KLVHQINDKQWKQYFLP-----FSARIDKQDNLKHM--TETMGEVIDPTYILNFKEWQ--DVLS-KTN----
-------RLVFNLYNGDVEL-------GISLRRTYKD------DKKEQYMVRYSFENFKY-----AGRN---
AISDSD---KMNSEQLKQAFETEVANNKKARKTEENNMKKEG-------------------------YHIDE
S---------------YIDPDIWPYVQ
>WKA02_13
----------MKNYLSLFIC---IITCIFLTSC-KPANNTTQGNKSMNQNQDVI----A----------LHF
GE-----QGMQD----------------FSKNS----TTPVDRQPAGMNFLSLDWTPPKLGRVRVFSEKSNL
EIENVISVLGTQVARRSNDGIQI----MDINASLH-------------PNEYTTSQEAYTAYT---------
KLVHQINDKQWKQYFLP-----FSARIDKQDNLKHM--TETMGEVIDPTYILNFKEWQ--DVLS-KTN----
-------RLVFNLYNGDVEL-------GISLRRTYKD------DKKEQYMVRYSFENFKY-----AGRN---
AISDSD---KMNSEQLKQAFETEVANNKKARKTEENNMKKEG-------------------------YHIDE
S---------------YIDPDIWPYVQ
>XH856_13
----------MKNYLSLFIC---IITCIFLTSC-KPANNTTQGNKSMNQNQDVI----A----------LHF
GE-----QGMQD----------------FSKNS----TTPVDRQPAGMNFLSLDWTPPKLGRVRVFSEKSNL
EIENVISVLGTQVARRSNDGIQI----MDINASLH-------------PNEYTTSQEAYTAYT---------
KLVHQINDKQWKQYFLP-----FSARIDKQDNLKHM--TETMGEVIDPTYILNFKEWQ--DVLS-KTN----
-------RLVFNLYNGDVEL-------GISLRRTYKD------DKKEQYMVRYSFENFKY-----AGRN---
AISDSD---KMNSEQLKQAFETEVANNKKARKTEENNMKKEG-------------------------YHIDE
S---------------YIDPDIWPYVQ

>XH858_13
----------MKNYLSLFIC---IITCIFLTSC-KPANNTTQGNKSMNQNQDVI----A----------LHF
GE-----QGMQD----------------FSKNS----TTPVDRQPAGMNFLSLDWTPPKLGRVRVFSEKSNL
EIENVISVLGTQVARRSNDGIQI----MDINASLH-------------PNEYTTSQEAYTAYT---------
KLVHQINDKQWKQYFLP-----FSARIDKQDNLKHM--TETMGEVIDPTYILNFKEWQ--DVLS-KTN----
-------RLVFNLYNGDVEL-------GISLRRTYKD------DKKEQYMVRYSFENFKY-----AGRN---
AISDSD---KMNSEQLKQAFETEVANNKKARKTEENNMKKEG-------------------------YHIDE
S---------------YIDPDIWPYVQ

**Supplementary file S4f**
>SDF_16
-MLNSIHQVLDSLGISPQKRAIHVQFMSPILNAQVFLQRIDGVHALNDGLKAELLCLSTNATIQLKSFIGVQ
AAVDIVTERGELTRVTGIITHAQQGQSDGSLTLYKLTLEDPTALWKYRRNSRVFMNKSVVEIWEILFKEWQT
KNPLFAASLSLDLSGLT-QTYDVRPFVMQHNESDWNFLTRLLRSENISWLIDEAQHIVPSTDTSIQAQKLRL
IDANSQYQPLDRKTIRYHRSSAVEQYDSMTRLTAERSLQPNVMHIQRWQAEILDQEEGIGSVQSKHQHSEQY
DNATLGLEQAWNYAPAWIGDLKGEDGVTKSGNQQVERLNQNLNNYYEAQAKRFIAQTTVRDAYVGYYFELNE
HPEIDQ-HESTDRSFLIISKSFFNQNNLPKDLNDQINGLLAQSNWAIQNPE----NSDERQANQLILQRRHI
PTTPAYNPQIHSPVTHPQRAKVVGPEGEEIYVDEWGRIKVRFLFTRSADHSHDGGAGTNNNDTDSAWIDVLT
PWAGEGYGARFLPRIGEIVVIDFFNGDIDRPFVMGRIHEAQRQPTKFDNKGKLPDTKKLSGFRSKEVSGGGF
GQLRFDDTPGQISTQLQSSHGASQLNLGKLSHPKDKAESEDRGEGFELRTDQWGALRAGQGLLVSTHKQDNA
KGDHLDANEVKQQIEGGLNNAKALSEVAKNQKTDEIESL---------------------------------
----------------------------------------EQLKDFASQIQQ--------------------
-------------------------------------------------------------------QIAKF
EKALLLLSSPDGIALSSSEDIHISADAQINQIAGDSINISTQKNVIAHAQNRLSLFAAQSGLKAVAAQGKVE
IQAQSDALDVLANKGITISSTEDCIEISSPKEILITGASSQITLNGSGISPKTGGKFQVNAGQHVFQGGASA
SVKSSLPP------PPKRAQGVLELLHDYSHGAFVKGAGYTVT--DNLGKVVNGKLDDKGFARVSGLAT-GS
VKVLFDPDPRNPWDEASDFKRKVEWPNQNDVEGSI---GIESGSKGLTETLKNDFKKLSQLASNVKSVQQKI
ESVKKIKDQGAKALLPMAMEQVLGGSDGSKLSQ--------------------SFVSQNSFMPSSQHINPLR
RLAQASSDKTAITKIQSPFETFV
>A1_16
-MLNSIHQVLDSLGISPQKRAIHVQFTSSILNAQVFLQRIDGVHALNDGLKAELLCLSTNATIQLKSFIGVQ
AAVDIVTERGELTRVTGIITHAQQGQSDGSLTLYKLTLEDPTALWKYRRNSRVFMNKSVVEIWEILFKEWQT
KNPLFAASLSLDLSGLT-QTYDVRPFVMQHNESDWNFLTRLLRSENISWLIDEAQHIVPSTETPIQAQKLRL

IDANSQYQPLDRKTIRYHRSSAVEQYDSMTRLTAERSLQPNVMHIQRWQAEILDQEEGIGSVQSKHQHSEHY
DNATLGLEQAWNYSPAWIGDLKGEDGVTKSGNQQVERLNQNLNNYYEAQAKRFIAQTTVRDAYVGYYFELNE
HPEIDQ-HESTDRSFLIISKSFFNQNNLPKDLNDRINGLLAQSNWAIQNPE----NSDERQANQLILQRRHI
PTTPAYSPQIHSPVTHPQRAKVVGPEGEEIYVDEWGRIKVRFLFTRSDDHSHDGGAGTNNNDTDSAWIDVLT
PWAGEGYGVRFLPRIGEIVVIDFFNGDIDRPFVMGRIHEAQRQPTKFDNKGKLPDTKKLSGIRSKEVSGGGF
GQLRFDDTPGQISTQLQSSHGASQLNMGKLSHPKDKAESEDRGEGFELRTDQWGALRAGQGLLVSTHKQDNA
KGEHLDAEVAKKQLEGSQTNSKALSDIAKNQKTDEIESI---------------------------------
----------------------------------------EQLKDFASQIQQ--------------------
-------------------------------------------------------------------QIAKF
EKALLLLSSPDGIALSSSEDIHISADAQINQIAGDSINISTQKNVIAHAQNRLSLFAAQSGLKAVAAQGKVE
IQAQSDALDVLANKGITISSTEDCIEISSPKEIVITGASSQITLNGSGIFPKTGGKFQVNAGQHVFQGGASA
SVKSSLPP------PPKRAQGVLELLHDYSHGAFVKGAGYTVT--DNLGKVVNGKLDDKGFARVSGLAT-GS
VKVLFDPDPRNPWDEASDFKRKVEWPNQNDVEGSI---GIESGSKGLTETLKNDFKKLSQLASNVKSVQQKI
ESVKKIKDQGAKALLPMAMEQVLGGSDGSKLSQ--------------------SFVSQNSFMPSSQQSNPLR
RLAQASSDKTAITKIQSPFETFV
>A388_16
-MLNSIHQVLDSLGISPQKRAIHVQFTSSILNAQVFLQRIDGVHALNDGLKAELLCLSTNATIQLKSFIGVQ
AAVDIVTERGELTRVTGIITHAQQGQSDGSLTLYKLTLEDPTALWKYRRNSRVFMNKSVVEIWEILFKEWQT
KNPLFAASLSLDLSGLT-QTYDVRPFVMQHNESDWNFLTRLLRSENISWLIDEAQHIVPSTETPIQAQKLRL
IDANSQYQPLDRKTIRYHRSSAVEQYDSMTRLTAERSLQPNVMHIQRWQAEILDQEEGIGSVQSKHQHSEHY
DNATLGLEQAWNYSPAWIGDLKGEDGVTKSGNQQVERLNQNLNNYYEAQAKRFIAQTTVRDAYVGYYFELNE
HPEIDQ-HESTDRSFLIISKSFFNQNNLPKDLNDRINGLLAQSNWAIQNPE----NSDERQANQLILQRRHI
PTTPAYSPQIHSPVTHPQRAKVVGPEGEEIYVDEWGRIKVRFLFTRSDDHSHDGGAGTNNNDTDSAWIDVLT
PWAGEGYGVRFLPRIGEIVVIDFFNGDIDRPFVMGRIHEAQRQPTKFDNKGKLPDTKKLSGIRSKEVSGGGF
GQLRFDDTPGQISTQLQSSHGASQLNMGKLSHPKDKAESEDRGEGFELRTDQWGALRAGQGLLVSTHKQDNA
KGEHLDAEVAKKQLEGSQTNSKALSDIAKNQKTDEIESI---------------------------------
----------------------------------------EQLKDFASQIQQ--------------------
-------------------------------------------------------------------QIAKF
EKALLLLSSPDGIALSSSEDIHISADAQINQIAGDSINISTQKNVIAHAQNRLSLFAAQSGLKAVAAQGKVE
IQAQSDALDVLANKGITISSTEDCIEISSPKEIVITGASSQITLNGSGIFPKTGGKFQVNAGQHVFQGGASA
SVKSSLPP------PPKRAQGVLELLHDYSHGAFVKGAGYTVT--DNLGKVVNGKLDDKGFARVSGLAT-GS
VKVLFDPDPRNPWDEASDFKRKVEWPNQNDVEGSI---GIESGSKGLTETLKNDFKKLSQLASNVKSVQQKI
ESVKKIKDQGAKALLPMAMEQVLGGSDGSKLSQ--------------------SFVSQNSFMPSSQQSNPLR

RLAQASSDKTAITKIQSPFETFV
>A85_16
-MLNSIHQVLDSLGISPQKRAIHVQFTSSILNAQVFLQRIDGVHALNDGLKAELLCLSTNATIQLKSFIGVQ
AAVDIVTERGELTRVTGIITHAQQGQSDGSLTLYKLTLEDPTALWKYRRNSRVFMNKSVVEIWEILFKEWQT
KNPLFAASLSLDLSGLT-QTYDVRPFVMQHNESDWNFLTRLLRSENISWLIDEAQHIVPSTETPIQAQKLRL
IDANSQYQPLDRKTIRYHRSSAVEQYDSMTRLTAERSLQPNVMHIQRWQAEILDQEEGIGSVQSKHQHSEHY
DNATLGLEQAWNYSPAWIGDLKGEDGVTKSGNQQVERLNQNLNNYYEAQAKRFIAQTTVRDAYVGYYFELNE
HPEIDQ-HESTDRSFLIISKSFFNQNNLPKDLNDRINGLLAQSNWAIQNPE----NSDERQANQLILQRRHI
PTTPAYSPQIHSPVTHPQRAKVVGPEGEEIYVDEWGRIKVRFLFTRSDDHSHDGGAGTNNNDTDSAWIDVLT
PWAGEGYGVRFLPRIGEIVVIDFFNGDIDRPFVMGRIHEAQRQPTKFDNKGKLPDTKKLSGIRSKEVSGGGF
GQLRFDDTPGQISTQLQSSHGASQLNMGKLSHPKDKAESEDRGEGFELRTDQWGALRAGQGLLVSTHKQDNA
KGEHLDAEVAKKQLEGSQTNSKALSDIAKNQKTDEIESI---------------------------------
----------------------------------------EQLKDFASQIQQ--------------------
-----------------------------------------------------------------QIAKF
EKALLLLSSPDGIALSSSEDIHISADAQINQIAGDSINISTQKNVIAHAQNRLSLFAAQSGLKAVAAQGKVE
IQAQSDALDVLANKGITISSTEDCIEISSPKEIVITGASSQITLNGSGIFPKTGGKFQVNAGQHVFQGGASA
SVKSSLPP------PPKRAQGVLELLHDYSHGAFVKGAGYTVT--DNLGKVVNGKLDDKGFARVSGLAT-GS
VKVLFDPDPRNPWDEASDFKRKVEWPNQNDVEGSI---GIESGSKGLTETLKNDFKKLSQLASNVKSVQQKI
ESVKKIKDQGAKALLPMAMEQVLGGSDGSKLSQ--------------------SFVSQNSFMPSSQQSNPLR
RLAQASSDKTAITKIQSPFETFV
>AB0057_16
-MLNSIHQVLDSLGISPQKRAIHVQFTSSILNAQVFLQRIDGVHALNDGLKAELLCLSTNATIQLKSFIGVQ
AAVDIVTERGELTRVTGIITHAQQGQSDGSLTLYKLTLEDPTALWKYRRNSRVFMNKSVVEIWEILFKEWQT
KNPLFAASLSLDLSGLT-QTYDVRPFVMQHNESDWNFLTRLLRSENISWLIDEAQHIVPSTETPIQAQKLRL
IDANSQYQPLDRKTIRYHRSSAVEQYDSMTRLTAERSLQPNVMHIQRWQAEILDQEEGIGSVQSKHQHSEHY
DNATLGLEQAWNYSPAWIGDLKGEDGVTKSGNQQVERLNQNLNNYYEAQAKRFIAQTTVRDAYVGYYFELNE
HPEIDQ-HESTDRSFLIISKSFFNQNNLPKDLNDRINGLLAQSNWAIQNPE----NSDERQANQLILQRRHI
PTTPAYSPQIHSPVTHPQRAKVVGPEGEEIYVDEWGRIKVRFLFTRSDDHSHDGGAGTNNNDTDSAWIDVLT
PWAGEGYGVRFLPRIGEIVVIDFFNGDIDRPFVMGRIHEAQRQPTKFDNKGKLPDTKKLSGIRSKEVSGGGF
GQLRFDDTPGQISTQLQSSHGASQLNMGKLSHPKDKAESEDRGEGFELRTDQWGALRAGQGLLVSTHKQDNA
KGEHLDAEVAKKQLEGSQTNSKALSDIAKNQKTDEIESI---------------------------------
----------------------------------------EQLKDFASQIQQ--------------------
-----------------------------------------------------------------QIAKF

EKALLLLSSPDGIALSSSEDIHISADAQINQIAGDSINISTQKNVIAHAQNRLSLFAAQSGLKAVAAQGKVE
IQAQSDALDVLANKGITISSTEDCIEISSPKEIVITGASSQITLNGSGIFPKTGGKFQVNAGQHVFQGGASA
SVKSSLPP------PPKRAQGVLELLHDYSHGAFVKGAGYTVT--DNLGKVVNGKLDDKGFARVSGLAT-GS
VKVLFDPDPRNPWDEASDFKRKVEWPNQNDVEGSI---GIESGSKGLTETLKNDFKKLSQLASNVKSVQQKI
ESVKKIKDQGAKALLPMAMEQVLGGSDGSKLSQ--------------------SFVSQNSFMPSSQQSNPLR
RLAQASSDKTAITKIQSPFETFV
>AB307-0294_16
-MLNSIHQVLDSLGISPQKRAIHVQFTSSILNAQVFLQRIDGVHALNDGLKAELLCLSTNATIQLKSFIGVQ
AAVDIVTERGELTRVTGIITHAQQGQSDGSLTLYKLTLEDPTALWKYRRNSRVFMNKSVVEIWEILFKEWQT
KNPLFAASLSLDLSGLT-QTYDVRPFVMQHNESDWNFLTRLLRSENISWLIDEAQHIVPSTETPIQAQKLRL
IDANSQYQPLDRKTIRYHRSSAVEQYDSMTRLTAERSLQPNVMHIQRWQAEILDQEEGIGSVQSKHQHSEHY
DNATLGLEQAWNYSPAWIGDLKGEDGVTKSGNQQVERLNQNLNNYYEAQAKRFIAQTTVRDAYVGYYFELNE
HPEIDQ-HESTDRSFLIISKSFFNQNNLPKDLNDRINGLLAQSNWAIQNPE----NSDERQANQLILQRRHI
PTTPAYSPQIHSPVTHPQRAKVVGPEGEEIYVDEWGRIKVRFLFTRSDDHSHDGGAGTNNNDTDSAWIDVLT
PWAGEGYGVRFLPRIGEIVVIDFFNGDIDRPFVMGRIHEAQRQPTKFDNKGKLPDTKKLSGIRSKEVSGGGF
GQLRFDDTPGQISTQLQSSHGASQLNMGKLSHPKDKAESEDRGEGFELRTDQWGALRAGQGLLVSTHKQDNA
KGEHLDAEVAKKQLEGSQTNSKALSDIAKNQKTDEIESI---------------------------------
----------------------------------------EQLKDFASQIQQ--------------------
-------------------------------------------------------------------QIAKF
EKALLLLSSPDGIALSSSEDIHISADAQINQIAGDSINISTQKNVIAHAQNRLSLFAAQSGLKAVAAQGKVE
IQAQSDALDVLANKGITISSTEDCIEISSPKEIVITGASSQITLNGSGIFPKTGGKFQVNAGQHVFQGGASA
SVKSSLPP------PPKRAQGVLELLHDYSHGAFVKGAGYTVT--DNLGKVVNGKLDDKGFARVSGLAT-GS
VKVLFDPDPRNPWDEASDFKRKVEWPNQNDVEGSI---GIESGSKGLTETLKNDFKKLSQLASNVKSVQQKI
ESVKKIKDQGAKALLPMAMEQVLGGSDGSKLSQ--------------------SFVSQNSFMPSSQQSNPLR
RLAQASSDKTAITKIQSPFETFV
>AB5075-UW_16
-MLNSIHQVLDSLGISPQKRAIHVQFTSSILNAQVFLQRIDGVHALNDGLKAELLCLSTNATIQLKSFIGVQ
AAVDIVTERGELTRVTGIITHAQQGQSDGSLTLYKLTLEDPTALWKYRRNSRVFMNKSVVEIWEILFKEWQT
KNPLFAASLSLDLSGLT-QTYDVRPFVMQHNESDWNFLTRLLRSENISWLIDEAQHIVPSTETPIQAQKLRL
IDANSQYQPLDRKTIRYHRSSAVEQYDSMTRLTAERSLQPNVMHIQRWQAEILDQEEGIGSVQSKHQHSEHY
DNATLGLEQAWNYSPAWIGDLKGEDGVTKSGNQQVERLNQNLNNYYEAQAKRFIAQTTVRDAYVGYYFELNE
HPEIDQ-HESTDRSFLIISKSFFNQNNLPKDLNDRINGLLAQSNWAIQNPE----NSDERQANQLILQRRHI
PTTPAYSPQIHSPVTHPQRAKVVGPEGEEIYVDEWGRIKVRFLFTRSDDHSHDGGAGTNNNDTDSAWIDVLT

PWAGEGYGVRFLPRIGEIVVIDFFNGDIDRPFVMGRIHEAQRQPTKFDNKGKLPDTKKLSGIRSKEVSGGGF
GQLRFDDTPGQISTQLQSSHGASQLNMGKLSHPKDKAESEDRGEGFELRTDQWGALRAGQGLLVSTHKQDNA
KGEHLDAEVAKKQLEGSQTNSKALSDIAKNQKTDEIESI----------------------------------
----------------------------------------EQLKDFASQIQQ--------------------
-------------------------------------------------------------------QIAKF
EKALLLLSSPDGIALSSSEDIHISADAQINQIAGDSINISTQKNVIAHAQNRLSLFAAQSGLKAVAAQGKVE
IQAQSDALDVLANKGITISSTEDCIEISSPKEIVITGASSQITLNGSGIFPKTGGKFQVNAGQHVFQGGASA
SVKSSLPP------PPKRAQGVLELLHDYSHGAFVKGAGYTVT--DNLGKVVNGKLDDKGFARVSGLAT-GS
VKVLFDPDPRNPWDEASDFKRKVEWPNQNDVEGSI---GIESGSKGLTETLKNDFKKLSQLASNVKSVQQKI
ESVKKIKDQGAKALLPMAMEQVLGGSDGSKLSQ--------------------SFVSQNSFMPSSQQSNPLR
RLAQASSDKTAITKIQSPFETFV
>AR_0083_16
-MLNSIHQVLDSLGISPQKRAIHVQFTSSILNAQVFLQRIDGVHALNDGLKAELLCLSTNATIQLKSFIGVQ
AAVDIVTERGELTRVTGIITHAQQGQSDGSLTLYKLTLEDPTALWKYRRNSRVFMNKSVVEIWEILFKEWQT
KNPLFAASLSLDLSGLT-QTYDVRPFVMQHNESDWNFLTRLLRSENISWLIDEAQHIVPSTETPIQAQKLRL
IDANSQYQPLDRKTIRYHRSSAVEQYDSMTRLTAERSLQPNVMHIQRWQAEILDQEEGIGSVQSKHQHSEHY
DNATLGLEQAWNYSPAWIGDLKGEDGVTKSGNQQVERLNQNLNNYYEAQAKRFIAQTTVRDAYVGYYFELNE
HPEIDQ-HESTDRSFLIISKSFFNQNNLPKDLNDRINGLLAQSNWAIQNPE----NSDERQANQLILQRRHI
PTTPAYSPQIHSPVTHPQRAKVVGPEGEEIYVDEWGRIKVRFLFTRSDDHSHDGGAGTNNNDTDSAWIDVLT
PWAGEGYGVRFLPRIGEIVVIDFFNGDIDRPFVMGRIHEAQRQPTKFDNKGKLPDTKKLSGIRSKEVSGGGF
GQLRFDDTPGQISTQLQSSHGASQLNMGKLSHPKDKAESEDRGEGFELRTDQWGALRAGQGLLVSTHKQDNA
KGEHLDAEVAKKQLEGSQTNSKALSDIAKNQKTDEIESI----------------------------------
----------------------------------------EQLKDFASQIQQ--------------------
-------------------------------------------------------------------QIAKF
EKALLLLSSPDGIALSSSEDIHISADAQINQIAGDSINISTQKNVIAHAQNRLSLFAAQSGLKAVAAQGKVE
IQAQSDALDVLANKGITISSTEDCIEISSPKEIVITGASSQITLNGSGIFPKTGGKFQVNAGQHVFQGGASA
SVKSSLPP------PPKRAQGVLELLHDYSHGAFVKGAGYTVT--DNLGKVVNGKLDDKGFARVSGLAT-GS
VKVLFDPDPRNPWDEASDFKRKVEWPNQNDVEGSI---GIESGSKGLTETLKNDFKKLSQLASNVKSVQQKI
ESVKKIKDQGAKALLPMAMEQVLGGSDGSKLSQ--------------------SFVSQNSFMPSSQQSNPLR
RLAQASSDKTAITKIQSPFETFV
>D36_16
-MLNSIHQVLDSLGISPQKRAIHVQFTSSILNAQVFLQRIDGVHALNDGLKAELLCLSTNATIQLKSFIGVQ
AAVDIVTERGELTRVTGIITHAQQGQSDGSLTLYKLTLEDPTALWKYRRNSRVFMNKSVVEIWEILFKEWQT

KNPLFAASLSLDLSGLT-QTYDVRPFVMQHNESDWNFLTRLLRSENISWLIDEAQHIVPSTETPIQAQKLRL
IDANSQYQPLDRKTIRYHRSSAVEQYDSMTRLTAERSLQPNVMHIQRWQAEILDQEEGIGSVQSKHQHSEHY
DNATLGLEQAWNYSPAWIGDLKGEDGVTKSGNQQVERLNQNLNNYYEAQAKRFIAQTTVRDAYVGYYFELNE
HPEIDQ-HESTDRSFLIISKSFFNQNNLPKDLNDRINGLLAQSNWAIQNPE----NSDERQANQLILQRRHI
PTTPAYSPQIHSPVTHPQRAKVVGPEGEEIYVDEWGRIKVRFLFTRSDDHSHDGGAGTNNNDTDSAWIDVLT
PWAGEGYGVRFLPRIGEIVVIDFFNGDIDRPFVMGRIHEAQRQPTKFDNKGKLPDTKKLSGIRSKEVSGGGF
GQLRFDDTPGQISTQLQSSHGASQLNMGKLSHPKDKAESEDRGEGFELRTDQWGALRAGQGLLVSTHKQDNA
KGEHLDAEVAKKQLEGSQTNSKALSDIAKNQKTDEIESI---------------------------------
----------------------------------------EQLKDFASQIQQ--------------------
---------------------------------------------------------------------QIAKF
EKALLLLSSPDGIALSSSEDIHISADAQINQIAGDSINISTQKNVIAHAQNRLSLFAAQSGLKAVAAQGKVE
IQAQSDALDVLANKGITISSTEDCIEISSPKEIVITGASSQITLNGSGIFPKTGGKFQVNAGQHVFQGGASA
SVKSSLPP------PPKRAQGVLELLHDYSHGAFVKGAGYTVT--DNLGKVVNGKLDDKGFARVSGLAT-GS
VKVLFDPDPRNPWDEASDFKRKVEWPNQNDVEGSI---GIESGSKGLTETLKNDFKKLSQLASNVKSVQQKI
ESVKKIKDQGAKALLPMAMEQVLGGSDGSKLSQ--------------------SFVSQNSFMPSSQQSNPLR
RLAQASSDKTAITKIQSPFETFV
>USA15_16
-MLNSIHQVLDSLGISPQKRAIHVQFTSSILNAQVFLQRIDGVHALNDGLKAELLCLSTNATIQLKSFIGVQ
AAVDIVTERGELTRVTGIITHAQQGQSDGSLTLYKLTLEDPTALWKYRRNSRVFMNKSVVEIWEILFKEWQT
KNPLFAASLSLDLSGLT-QTYDVRPFVMQHNESDWNFLTRLLRSENISWLIDEAQHIVPSTETPIQAQKLRL
IDANSQYQPLDRKTIRYHRSSAVEQYDSMTRLTAERSLQPNVMHIQRWQAEILDQEEGIGSVQSKHQHSEHY
DNATLGLEQAWNYSPAWIGDLKGEDGVTKSGNQQVERLNQNLNNYYEAQAKRFIAQTTVRDAYVGYYFELNE
HPEIDQ-HESTDRSFLIISKSFFNQNNLPKDLNDRINGLLAQSNWAIQNPE----NSDERQANQLILQRRHI
PTTPAYSPQIHSPVTHPQRAKVVGPEGEEIYVDEWGRIKVRFLFTRSDDHSHDGGAGTNNNDTDSAWIDVLT
PWAGEGYGVRFLPRIGEIVVIDFFNGDIDRPFVMGRIHEAQRQPTKFDNKGKLPDTKKLSGIRSKEVSGGGF
GQLRFDDTPGQISTQLQSSHGASQLNMGKLSHPKDKAESEDRGEGFELRTDQWGALRAGQGLLVSTHKQDNA
KGEHLDAEVAKKQLEGSQTNSKALSDIAKNQKTDEIESI---------------------------------
----------------------------------------EQLKDFASQIQQ--------------------
---------------------------------------------------------------------QIAKF
EKALLLLSSPDGIALSSSEDIHISADAQINQIAGDSINISTQKNVIAHAQNRLSLFAAQSGLKAVAAQGKVE
IQAQSDALDVLANKGITISSTEDCIEISSPKEIVITGASSQITLNGSGIFPKTGGKFQVNAGQHVFQGGASA
SVKSSLPP------PPKRAQGVLELLHDYSHGAFVKGAGYTVT--DNLGKVVNGKLDDKGFARVSGLAT-GS
VKVLFDPDPRNPWDEASDFKRKVEWPNQNDVEGSI---GIESGSKGLTETLKNDFKKLSQLASNVKSVQQKI

ESVKKIKDQGAKALLPMAMEQVLGGSDGSKLSQ--------------------SFVSQNSFMPSSQQSNPLR
RLAQASSDKTAITKIQSPFETFV
>WCHAB005078_16
-MLNSIHQVLDSLGISPQKRAIHVQFTSSILNAQVFLQRIDGVHALNDGLKAELLCLSTNATIQLKSFIGVQ
AAVDIVTERGELTRVTGIITHAQQGQSDGSLTLYKLTLEDPTALWKYRRNSRVFMNKSVVEIWEILFKEWQT
KNPLFAASLSLDLSGLT-QTYDVRPFVMQHNESDWNFLTRLLRSENISWLIDEAQHIVPSTETPIQAQKLRL
IDANSQYQPLDRKTIRYHRSSAVEQYDSMTRLTAERSLQPNVMHIQRWQAEILDQEEGIGSVQSKHQHSEHY
DNATLGLEQAWNYSPAWIGDLKGEDGVTKSGNQQVERLNQNLNNYYEAQAKRFIAQTTVRDAYVGYYFELNE
HPEIDQ-HESTDRSFLIISKSFFNQNNLPKDLNDRINGLLAQSNWAIQNPE----NSDERQANQLILQRRHI
PTTPAYSPQIHSPVTHPQRAKVVGPEGEEIYVDEWGRIKVRFLFTRSDDHSHDGGAGTNNNDTDSAWIDVLT
PWAGEGYGVRFLPRIGEIVVIDFFNGDIDRPFVMGRIHEAQRQPTKFDNKGKLPDTKKLSGIRSKEVSGGGF
GQLRFDDTPGQISTQLQSSHGASQLNMGKLSHPKDKAESEDRGEGFELRTDQWGALRAGQGLLVSTHKQDNA
KGEHLDAEVAKKQLEGSQTNSKALSDIAKNQKTDEIESI-----------------------------------
----------------------------------------EQLKDFASQIQQ--------------------
-----------------------------------------------------------------QIAKF
EKALLLLSSPDGIALSSSEDIHISADAQINQIAGDSINISTQKNVIAHAQNRLSLFAAQSGLKAVAAQGKVE
IQAQSDALDVLANKGITISSTEDCIEISSPKEIVITGASSQITLNGSGIFPKTGGKFQVNAGQHVFQGGASA
SVKSSLPP------PPKRAQGVLELLHDYSHGAFVKGAGYTVT--DNLGKVVNGKLDDKGFARVSGLAT-GS
VKVLFDPDPRNPWDEASDFKRKVEWPNQNDVEGSI---GIESGSKGLTETLKNDFKKLSQLASNVKSVQQKI
ESVKKIKDQGAKALLPMAMEQVLGGSDGSKLSQ--------------------SFVSQNSFMPSSQQSNPLR
RLAQASSDKTAITKIQSPFETFV
>AYE_16
-MLNSIHQVLDSLGISPQKRAIHVQFTSSILNAQVFLQRIDGVHALNDGLKAELLCLSTNATIQLKSFIGVQ
AAVDIVTERGELTRVTGIITHAQQGQSDGSLTLYKLTLEDPTALWKYRRNSRVFMNKSVVEIWEILFKEWQT
KNPLFAASLSLDLSGLT-QTYDVRPFVMQHNESDWNFLTRLLRSENISWLIDEAQHIVPSTETPIQAQKLRL
IDANSQYQPLDRKTIRYHRSSAVEQYDSMTRLTAERSLQPNVMHIQRWQAEILDQEEGIGSVQSKHQHSEHY
DNATLGLEQAWNYSPAWIGDLKGEDGVTKSGNQQVERLNQNLNNYYEAQAKRFIAQTTVRDAYVGYYFELNE
HPEIDQ-HESTDRSFLIISKSFFNQNNLPKDLNDRINGLLAQSNWAIQNPE----NSDERQANQLILQRRHI
PTTPAYSPQIHSPVTHPQRAKVVGPEGEEIYVDEWGRIKVRFLFTRSDDHSHDGGAGTNNNDTDSAWIDVLT
PWAGEGYGVRFLPRIGEIVVIDFFNGDIDRPFVMGRIHEAQRQPTKFDNKGKLPDTKKLSGIRSKDVSGGGF
GQLRFDDTPGQISTQLQSSHGASQLNMGKLSHPKDKAESEDRGEGFELRTDQWGALRAGQGLLVSTHKQDNA
KGEHLDAEVAKKQLEGSQTNSKALSDIAKNQKTDEIESI-----------------------------------
----------------------------------------EQLKDFASQIQQ--------------------

```
-------------------------------------------------------------------QIAKF
EKALLLLSSPDGIALSSSEDIHISADAQINQIAGDSINISTQKNVIAHAQNRLSLFAAQSGLKAVAAQGKVE
IQAQSDALDVLANKGITISSTEDCIEISSPKEIVITGASSQITLNGSGIFPKTGGKFQVNAGQHVFQGGASA
SVKSSLPP------PPKRAQGVLELLHDYSHGAFVKGAGYTVT--DNLGKVVNGKLDDKGFARVSGLAT-GS
VKVLFDPDPRNPWDEASDFKRKVEWPNQNDVEGSI---GIESGSKGLTETLKNDFKKLSQLASNVKSVQQKI
ESVKKIKDQGAKALLPMAMEQVLGGSDGSKLSQ--------------------SFVSQNSFMPSSQQSNPLR
RLAQASSDKTAITKIQSPFETFV
>6200_21
-MLNSIHQVLDSLGISPQKRAIHVQFTSPILNAQVFLQRIDGVHALNDGLKAELLCLSTNATIQLKSFIGVQ
AAVDIVTERGELTRVTGIITHAQQGQSDGSLTLYKLTLEDPTALWKYRRNSRVFMNKSVVEIWEILFKEWQT
KNPLFAASLSLDLSGLT-QTYEVRPFVMQHNESDWNFLTRLLRSENISWLIDEAQHIVPSTDTPIQAQKLRL
IDANSQYQPLDRKTIRYHRSSAVEQYDSMTRLTAERSLQPNVMHIQRWQAEILDQEEGIGSVQSKHQHSEQY
DNATLGLEQAWNYAPAWIGDLKGEDGVTKSGNQQVERLNQNLHNYYEAQAKRFIAQTTVRDAYVGYYFELNE
HPEIDQ-HESTDRSFLIISKSFFNQNNLPKDLNDQINGLLAQSNWAIQNPE----NSDERQANQLILQRRHI
PTTPAYNPQIHSPVTHPQRAKVVGPEGEEIYVDEWGRIKVRFLFTRSDDHSHDGGAGTNNNDTDSAWIDVLT
PWAGEGYGARFLPRIGEIVVIDFFNGDIDRPFVMGRIHEAQRHPTKFDNKGKLPDTKKLSGIRSKEVSGGGF
GQLRFDDTPGQISTQLQSSHGASQLNLGKLSHPKDKAESEDRGEGFELRTDQWGALRAGQGLLVSTHKQDNA
KGDHLDAEVAKKQLEGSQTNSKALSDIAKNQKTDEIESI---------------------------------
----------------------------------------EQLKDFASQIQQ--------------------
-------------------------------------------------------------------QIAKF
EKALLLLSSPDGIALSSSEDIHISADAQINQIAGDSINISTQKNVIAHAQNRLSLFAAQSGLKAVAAQGKVE
IQAQSDALDVLANKGITISSTEDCIEISSPKEIVITGASSQITLNGSGIFPKTGGKFQVNAGQHVFQGGASA
SVQSSLPP------PPKRVQGVLELFHEYAHGEFVKGGSYRVV--DNFGKEVTGKLDDKGFAKVSGLAT-GA
VKVFFESDHRDPWDTASDFKRPVEWPNKNDADSEQSDSLIAQMSK----TAQSKLGELSKQLTNPTNIMKNI
QTAQSIKSEGAKALMPMLKTQAQGLVTDQVKSF---------LPISEAGQKIGNSIELTSIQKMNDFNKSGS
IDGNSLVNNTLHQYLQSPFKKNS
>1656-2_6
-MLNSIHQVLDTLGISPQKRAIHVQFTSPILNDQVFLQRIDGVHALNDGLKAELLCLSTNATIQLKSFIGVQ
AAVDIVTERGELTRVTGIITHAQQGQSDGSLTLYKLTLEDPTALWKYRRNSRVFMNKSVVEIWEILFKEWQT
KNPLFAASLSLDLSGLT-QTYDVRPFVMQHNESDWNFLTRLLRSENISWLIDEAQHIVPSTDTSIQAQKLRL
IDANSQYQPLDRKTIRYHRSSAVEQYDSMTRLTAERSLQPNVMHIQRWQAEILDQEEGIGSVQSKHQHSEHY
DNATLGLEQAWNYSPAWIGDLKGEDGVTKSGNQQVERLNQNLHNYYEAQAKRFIAQTTVRDAYVGYYFELNE
HPEIDQ-HESSDKSFLIVSKNFFNQNNLPKDLNDQINGLLAQSNWAIQNPE----NSDERQANQLILQRRHI
```

PTTPAYNPQIHSPVTHPQRAKVVGPEGEEIYVDEWGRIKVRFLFTRSDDHSHDGGAGTNNNDTDSAWIDVLT
PWAGEGYGARFLPRIGEIVVIDFFNGDIDRPFVMGRIHEAQRQPTKFDNKGKLPDTKKLSGIRSKEVSGGGF
GQLRFDDTPGQISTQLQSSHGASQLNLGKLSHPKDKAESEDRGEGFELRTDQWGALRAGQGLLVSTHKQDNA
KGDHLDAEVAKKQLEGSQTNSKALSDIAKNQKTDEIESI-----------------------------------
----------------------------------------EQLKDFASQIQQ--------------------
----------------------------------------------------------------QIAKF
EKALLLLSSPDGIALSSSEDIHISADAQINQIAGDSINISTQKNVIAHAQNRLSLFAAQSGLKAVAAQGKVE
IQAQSDALDVLANKGITISSTEDCIEISSPKEIVITGASSQITLNGSGIFPKTGGKFQVNAGQHVFQGGASA
SVQSSLPP------PPKRVQGVLELFHEYAHGEFVKGGSYRVV--DNFGKEVTGKLDDKGFAKVSGLAT-GA
VKVFFESDHRDPWDTASDFKRPVEWPNKNDADSEQSDSLIAQMSK----TAQSKLGELSKQLTNPTNIMKNI
QTAQSIKSEGAKALMPMLKTQAQGLVTDQVKSF---------LPISEAGQKIGNNIELTSIQKMNDFNKSGS
IDGNSLVNNTLHQYLQSPFKKNS
>15A34_6
-MLNSIHQVLDSLGISPQKRAIHVQFTSPILNDQVFLQRIDGVHALNDGLKAELLCLSTNATIQLKSFIGVQ
AAVDIVTERGELTRVTGIITHAQQGQSDGSLTLYKLTLEDPTALWKYRRNSRVFMNKSVVEIWEILFKEWQT
KNPLFAASLSLDLSGLT-QTYDVRPFVMQHNESDWNFLTRLLRSENISWLIDEAQHIVPSTDTSIQAQKLRL
IDANSQYQPLDRKTIRYHRSSAVEQYDSMTRLTAERSLQPNVMHIQRWQAEILDQEEGIGSVQSKHQHSEHY
DNATLGLEQAWNYSPAWIGDLKGEDGVTKSGNQQVERLNQNLHNYYEAQAKRFIAQTTVRDAYVGYYFELNE
HPEIDQ-HESSDKSFLIVSKNFFNQNNLPKDLNDQINGLLAQSNWAIQNPE----NSDERQANQLILQRRHI
PTTPAYNPQIHSPVTHPQRAKVVGPEGEEIYVDEWGRIKVRFLFTRSDDHSHDGGAGTNNNDTDSAWIDVLT
PWAGEGYGARFLPRIGEIVVIDFFNGDIDRPFVMGRIHEAQRQPTKFDNKGKLPDTKKLSGIRSKEVSGGGF
GQLRFDDTPGQISTQLQSSHGASQLNLGKLSHPKDKAESEDRGEGFELRTDQWGALRAGQGLLVSTHKQDNA
KGDHLDAEVAKKQLEGSQTNSKALSDIAKNQKTDEIESI-----------------------------------
----------------------------------------EQLKDFASQIQQ--------------------
----------------------------------------------------------------QIAKF
EKALLLLSSPDGIALSSSEDIHISADAQINQIAGDSINISTQKNVIAHAQNRLSLFAAQSGLKAVAAQGKVE
IQAQSDALDVLANKGITISSTEDCIEISSPKEIVITGASSQITLNGSGIFPKTGGKFQVNAGQHVFQGGASA
SVQSSLPP------PPKRVQGVLELFHEYAHGEFVKGGSYRVV--DNFGKEVTGKLDDKGFAKVSGLAT-GA
VKVFFESDHRDPWDTASDFKRPVEWPNKNDADSEQSDSLIAQMSK----TAQSKLGELSKQLTNPTNIMKNI
QTAQSIKSEGAKALMPMLKTQAQGLVTDQVKSF---------LPISEAGQKIGNNIELTSIQKMNDFNKSGS
IDGNSLVNNTLHQYLQSPFKKNS
>15A5_6
-MLNSIHQVLDSLGISPQKRAIHVQFTSPILNDQVFLQRIDGVHALNDGLKAELLCLSTNATIQLKSFIGVQ

AAVDIVTERGELTRVTGIITHAQQGQSDGSLTLYKLTLEDPTALWKYRRNSRVFMNKSVVEIWEILFKEWQT
KNPLFAASLSLDLSGLT-QTYDVRPFVMQHNESDWNFLTRLLRSENISWLIDEAQHIVPSTDTSIQAQKLRL
IDANSQYQPLDRKTIRYHRSSAVEQYDSMTRLTAERSLQPNVMHIQRWQAEILDQEEGIGSVQSKHQHSEHY
DNATLGLEQAWNYSPAWIGDLKGEDGVTKSGNQQVERLNQNLHNYYEAQAKRFIAQTTVRDAYVGYYFELNE
HPEIDQ-HESSDKSFLIVSKNFFNQNNLPKDLNDQINGLLAQSNWAIQNPE----NSDERQANQLILQRRHI
PTTPAYNPQIHSPVTHPQRAKVVGPEGEEIYVDEWGRIKVRFLFTRSDDHSHDGGAGTNNNDTDSAWIDVLT
PWAGEGYGARFLPRIGEIVVIDFFNGDIDRPFVMGRIHEAQRQPTKFDNKGKLPDTKKLSGIRSKEVSGGGF
GQLRFDDTPGQISTQLQSSHGASQLNLGKLSHPKDKAESEDRGEGFELRTDQWGALRAGQGLLVSTHKQDNA
KGDHLDAEVAKKQLEGSQTNSKALSDIAKNQKTDEIESI-----------------------------------
---------------------------------------EQLKDFASQIQQ--------------------
-----------------------------------------------------------------QIAKF
EKALLLLSSPDGIALSSSEDIHISADAQINQIAGDSINISTQKNVIAHAQNRLSLFAAQSGLKAVAAQGKVE
IQAQSDALDVLANKGITISSTEDCIEISSPKEIVITGASSQITLNGSGIFPKTGGKFQVNAGQHVFQGGASA
SVQSSLPP------PPKRVQGVLELFHEYAHGEFVKGGSYRVV--DNFGKEVTGKLDDKGFAKVSGLAT-GA
VKVFFESDHRDPWDTASDFKRPVEWPNKNDADSEQSDSLIAQMSK----TAQSKLGELSKQLTNPTNIMKNI
QTAQSIKSEGAKALMPMLKTQAQGLVTDQVKSF---------LPISEAGQKIGNNIELTSIQKMNDFNKSGS
IDGNSLVNNTLHQYLQSPFKKNS
>3027STDY5784958_6
-MLNSIHQVLDSLGISPQKRAIHVQFTSPILNDQVFLQRIDGVHALNDGLKAELLCLSTNATIQLKSFIGVQ
AAVDIVTERGELTRVTGIITHAQQGQSDGSLTLYKLTLEDPTALWKYRRNSRVFMNKSVVEIWEILFKEWQT
KNPLFAASLSLDLSGLT-QTYDVRPFVMQHNESDWNFLTRLLRSENISWLIDEAQHIVPSTDTSIQAQKLRL
IDANSQYQPLDRKTIRYHRSSAVEQYDSMTRLTAERSLQPNVMHIQRWQAEILDQEEGIGSVQSKHQHSEHY
DNATLGLEQAWNYSPAWIGDLKGEDGVTKSGNQQVERLNQNLHNYYEAQAKRFIAQTTVRDAYVGYYFELNE
HPEIDQ-HESSDKSFLIVSKNFFNQNNLPKDLNDQINGLLAQSNWAIQNPE----NSDERQANQLILQRRHI
PTTPAYNPQIHSPVTHPQRAKVVGPEGEEIYVDEWGRIKVRFLFTRSDDHSHDGGAGTNNNDTDSAWIDVLT
PWAGEGYGARFLPRIGEIVVIDFFNGDIDRPFVMGRIHEAQRQPTKFDNKGKLPDTKKLSGIRSKEVSGGGF
GQLRFDDTPGQISTQLQSSHGASQLNLGKLSHPKDKAESEDRGEGFELRTDQWGALRAGQGLLVSTHKQDNA
KGDHLDAEVAKKQLEGSQTNSKALSDIAKNQKTDEIESI-----------------------------------
---------------------------------------EQLKDFASQIQQ--------------------
-----------------------------------------------------------------QIAKF
EKALLLLSSPDGIALSSSEDIHISADAQINQIAGDSINISTQKNVIAHAQNRLSLFAAQSGLKAVAAQGKVE
IQAQSDALDVLANKGITISSTEDCIEISSPKEIVITGASSQITLNGSGIFPKTGGKFQVNAGQHVFQGGASA
SVQSSLPP------PPKRVQGVLELFHEYAHGEFVKGGSYRVV--DNFGKEVTGKLDDKGFAKVSGLAT-GA

VKVFFESDHRDPWDTASDFKRPVEWPNKNDADSEQSDSLIAQMSK----TAQSKLGELSKQLTNPTNIMKNI
QTAQSIKSEGAKALMPMLKTQAQGLVTDQVKSF---------LPISEAGQKIGNNIELTSIQKMNDFNKSGS
IDGNSLVNNTLHQYLQSPFKKNS
>AB07_6
-MLNSIHQVLDSLGISPQKRAIHVQFTSPILNDQVFLQRIDGVHALNDGLKAELLCLSTNATIQLKSFIGVQ
AAVDIVTERGELTRVTGIITHAQQGQSDGSLTLYKLTLEDPTALWKYRRNSRVFMNKSVVEIWEILFKEWQT
KNPLFAASLSLDLSGLT-QTYDVRPFVMQHNESDWNFLTRLLRSENISWLIDEAQHIVPSTDTSIQAQKLRL
IDANSQYQPLDRKTIRYHRSSAVEQYDSMTRLTAERSLQPNVMHIQRWQAEILDQEEGIGSVQSKHQHSEHY
DNATLGLEQAWNYSPAWIGDLKGEDGVTKSGNQQVERLNQNLHNYYEAQAKRFIAQTTVRDAYVGYYFELNE
HPEIDQ-HESSDKSFLIVSKNFFNQNNLPKDLNDQINGLLAQSNWAIQNPE----NSDERQANQLILQRRHI
PTTPAYNPQIHSPVTHPQRAKVVGPEGEEIYVDEWGRIKVRFLFTRSDDHSHDGGAGTNNNDTDSAWIDVLT
PWAGEGYGARFLPRIGEIVVIDFFNGDIDRPFVMGRIHEAQRQPTKFDNKGKLPDTKKLSGIRSKEVSGGGF
GQLRFDDTPGQISTQLQSSHGASQLNLGKLSHPKDKAESEDRGEGFELRTDQWGALRAGQGLLVSTHKQDNA
KGDHLDAEVAKKQLEGSQTNSKALSDIAKNQKTDEIESI---------------------------------
----------------------------------------EQLKDFASQIQQ--------------------
-------------------------------------------------------------------QIAKF
EKALLLLSSPDGIALSSSEDIHISADAQINQIAGDSINISTQKNVIAHAQNRLSLFAAQSGLKAVAAQGKVE
IQAQSDALDVLANKGITISSTEDCIEISSPKEIVITGASSQITLNGSGIFPKTGGKFQVNAGQHVFQGGASA
SVQSSLPP------PPKRVQGVLELFHEYAHGEFVKGGSYRVV--DNFGKEVTGKLDDKGFAKVSGLAT-GA
VKVFFESDHRDPWDTASDFKRPVEWPNKNDADSEQSDSLIAQMSK----TAQSKLGELSKQLTNPTNIMKNI
QTAQSIKSEGAKALMPMLKTQAQGLVTDQVKSF---------LPISEAGQKIGNNIELTSIQKMNDFNKSGS
IDGNSLVNNTLHQYLQSPFKKNS
>AB34299_6
-MLNSIHQVLDSLGISPQKRAIHVQFTSPILNDQVFLQRIDGVHALNDGLKAELLCLSTNATIQLKSFIGVQ
AAVDIVTERGELTRVTGIITHAQQGQSDGSLTLYKLTLEDPTALWKYRRNSRVFMNKSVVEIWEILFKEWQT
KNPLFAASLSLDLSGLT-QTYDVRPFVMQHNESDWNFLTRLLRSENISWLIDEAQHIVPSTDTSIQAQKLRL
IDANSQYQPLDRKTIRYHRSSAVEQYDSMTRLTAERSLQPNVMHIQRWQAEILDQEEGIGSVQSKHQHSEHY
DNATLGLEQAWNYSPAWIGDLKGEDGVTKSGNQQVERLNQNLHNYYEAQAKRFIAQTTVRDAYVGYYFELNE
HPEIDQ-HESSDKSFLIVSKNFFNQNNLPKDLNDQINGLLAQSNWAIQNPE----NSDERQANQLILQRRHI
PTTPAYNPQIHSPVTHPQRAKVVGPEGEEIYVDEWGRIKVRFLFTRSDDHSHDGGAGTNNNDTDSAWIDVLT
PWAGEGYGARFLPRIGEIVVIDFFNGDIDRPFVMGRIHEAQRQPTKFDNKGKLPDTKKLSGIRSKEVSGGGF
GQLRFDDTPGQISTQLQSSHGASQLNLGKLSHPKDKAESEDRGEGFELRTDQWGALRAGQGLLVSTHKQDNA
KGDHLDAEVAKKQLEGSQTNSKALSDIAKNQKTDEIESI---------------------------------

----------------------------------------EQLKDFASQIQQ--------------------
-------------------------------------------------------------------QIAKF
EKALLLLSSPDGIALSSSEDIHISADAQINQIAGDSINISTQKNVIAHAQNRLSLFAAQSGLKAVAAQGKVE
IQAQSDALDVLANKGITISSTEDCIEISSPKEIVITGASSQITLNGSGIFPKTGGKFQVNAGQHVFQGGASA
SVQSSLPP------PPKRVQGVLELFHEYAHGEFVKGGSYRVV--DNFGKEVTGKLDDKGFAKVSGLAT-GA
VKVFFESDHRDPWDTASDFKRPVEWPNKNDADSEQSDSLIAQMSK----TAQSKLGELSKQLTNPTNIMKNI
QTAQSIKSEGAKALMPMLKTQAQGLVTDQVKSF---------LPISEAGQKIGNNIELTSIQKMNDFNKSGS
IDGNSLVNNTLHQYLQSPFKKNS
>Ab4568_6
-MLNSIHQVLDSLGISPQKRAIHVQFTSPILNDQVFLQRIDGVHALNDGLKAELLCLSTNATIQLKSFIGVQ
AAVDIVTERGELTRVTGIITHAQQGQSDGSLTLYKLTLEDPTALWKYRRNSRVFMNKSVVEIWEILFKEWQT
KNPLFAASLSLDLSGLT-QTYDVRPFVMQHNESDWNFLTRLLRSENISWLIDEAQHIVPSTDTSIQAQKLRL
IDANSQYQPLDRKTIRYHRSSAVEQYDSMTRLTAERSLQPNVMHIQRWQAEILDQEEGIGSVQSKHQHSEHY
DNATLGLEQAWNYSPAWIGDLKGEDGVTKSGNQQVERLNQNLHNYYEAQAKRFIAQTTVRDAYVGYYFELNE
HPEIDQ-HESSDKSFLIVSKNFFNQNNLPKDLNDQINGLLAQSNWAIQNPE----NSDERQANQLILQRRHI
PTTPAYNPQIHSPVTHPQRAKVVGPEGEEIYVDEWGRIKVRFLFTRSDDHSHDGGAGTNNNDTDSAWIDVLT
PWAGEGYGARFLPRIGEIVVIDFFNGDIDRPFVMGRIHEAQRQPTKFDNKGKLPDTKKLSGIRSKEVSGGGF
GQLRFDDTPGQISTQLQSSHGASQLNLGKLSHPKDKAESEDRGEGFELRTDQWGALRAGQGLLVSTHKQDNA
KGDHLDAEVAKKQLEGSQTNSKALSDIAKNQKTDEIESI---------------------------------
----------------------------------------EQLKDFASQIQQ--------------------
-------------------------------------------------------------------QIAKF
EKALLLLSSPDGIALSSSEDIHISADAQINQIAGDSINISTQKNVIAHAQNRLSLFAAQSGLKAVAAQGKVE
IQAQSDALDVLANKGITISSTEDCIEISSPKEIVITGASSQITLNGSGIFPKTGGKFQVNAGQHVFQGGASA
SVQSSLPP------PPKRVQGVLELFHEYAHGEFVKGGSYRVV--DNFGKEVTGKLDDKGFAKVSGLAT-GA
VKVFFESDHRDPWDTASDFKRPVEWPNKNDADSEQSDSLIAQMSK----TAQSKLGELSKQLTNPTNIMKNI
QTAQSIKSEGAKALMPMLKTQAQGLVTDQVKSF---------LPISEAGQKIGNNIELTSIQKMNDFNKSGS
IDGNSLVNNTLHQYLQSPFKKNS
>Ab4653_6
-MLNSIHQVLDSLGISPQKRAIHVQFTSPILNDQVFLQRIDGVHALNDGLKAELLCLSTNATIQLKSFIGVQ
AAVDIVTERGELTRVTGIITHAQQGQSDGSLTLYKLTLEDPTALWKYRRNSRVFMNKSVVEIWEILFKEWQT
KNPLFAASLSLDLSGLT-QTYDVRPFVMQHNESDWNFLTRLLRSENISWLIDEAQHIVPSTDTSIQAQKLRL
IDANSQYQPLDRKTIRYHRSSAVEQYDSMTRLTAERSLQPNVMHIQRWQAEILDQEEGIGSVQSKHQHSEHY
DNATLGLEQAWNYSPAWIGDLKGEDGVTKSGNQQVERLNQNLHNYYEAQAKRFIAQTTVRDAYVGYYFELNE

HPEIDQ-HESSDKSFLIVSKNFFNQNNLPKDLNDQINGLLAQSNWAIQNPE----NSDERQANQLILQRRHI
PTTPAYNPQIHSPVTHPQRAKVVGPEGEEIYVDEWGRIKVRFLFTRSDDHSHDGGAGTNNNDTDSAWIDVLT
PWAGEGYGARFLPRIGEIVVIDFFNGDIDRPFVMGRIHEAQRQPTKFDNKGKLPDTKKLSGIRSKEVSGGGF
GQLRFDDTPGQISTQLQSSHGASQLNLGKLSHPKDKAESEDRGEGFELRTDQWGALRAGQGLLVSTHKQDNA
KGDHLDAEVAKKQLEGSQTNSKALSDIAKNQKTDEIESI---------------------------------
----------------------------------------EQLKDFASQIQQ--------------------
-------------------------------------------------------------------QIAKF
EKALLLLSSPDGIALSSSEDIHISADAQINQIAGDSINISTQKNVIAHAQNRLSLFAAQSGLKAVAAQGKVE
IQAQSDALDVLANKGITISSTEDCIEISSPKEIVITGASSQITLNGSGIFPKTGGKFQVNAGQHVFQGGASA
SVQSSLPP------PPKRVQGVLELFHEYAHGEFVKGGSYRVV--DNFGKEVTGKLDDKGFAKVSGLAT-GA
VKVFFESDHRDPWDTASDFKRPVEWPNKNDADSEQSDSLIAQMSK----TAQSKLGELSKQLTNPTNIMKNI
QTAQSIKSEGAKALMPMLKTQAQGLVTDQVKSF---------LPISEAGQKIGNNIELTSIQKMNDFNKSGS
IDGNSLVNNTLHQYLQSPFKKNS
>Ab4977_6
-MLNSIHQVLDSLGISPQKRAIHVQFTSPILNDQVFLQRIDGVHALNDGLKAELLCLSTNATIQLKSFIGVQ
AAVDIVTERGELTRVTGIITHAQQGQSDGSLTLYKLTLEDPTALWKYRRNSRVFMNKSVVEIWEILFKEWQT
KNPLFAASLSLDLSGLT-QTYDVRPFVMQHNESDWNFLTRLLRSENISWLIDEAQHIVPSTDTSIQAQKLRL
IDANSQYQPLDRKTIRYHRSSAVEQYDSMTRLTAERSLQPNVMHIQRWQAEILDQEEGIGSVQSKHQHSEHY
DNATLGLEQAWNYSPAWIGDLKGEDGVTKSGNQQVERLNQNLHNYYEAQAKRFIAQTTVRDAYVGYYFELNE
HPEIDQ-HESSDKSFLIVSKNFFNQNNLPKDLNDQINGLLAQSNWAIQNPE----NSDERQANQLILQRRHI
PTTPAYNPQIHSPVTHPQRAKVVGPEGEEIYVDEWGRIKVRFLFTRSDDHSHDGGAGTNNNDTDSAWIDVLT
PWAGEGYGARFLPRIGEIVVIDFFNGDIDRPFVMGRIHEAQRQPTKFDNKGKLPDTKKLSGIRSKEVSGGGF
GQLRFDDTPGQISTQLQSSHGASQLNLGKLSHPKDKAESEDRGEGFELRTDQWGALRAGQGLLVSTHKQDNA
KGDHLDAEVAKKQLEGSQTNSKALSDIAKNQKTDEIESI---------------------------------
----------------------------------------EQLKDFASQIQQ--------------------
-------------------------------------------------------------------QIAKF
EKALLLLSSPDGIALSSSEDIHISADAQINQIAGDSINISTQKNVIAHAQNRLSLFAAQSGLKAVAAQGKVE
IQAQSDALDVLANKGITISSTEDCIEISSPKEIVITGASSQITLNGSGIFPKTGGKFQVNAGQHVFQGGASA
SVQSSLPP------PPKRVQGVLELFHEYAHGEFVKGGSYRVV--DNFGKEVTGKLDDKGFAKVSGLAT-GA
VKVFFESDHRDPWDTASDFKRPVEWPNKNDADSEQSDSLIAQMSK----TAQSKLGELSKQLTNPTNIMKNI
QTAQSIKSEGAKALMPMLKTQAQGLVTDQVKSF---------LPISEAGQKIGNNIELTSIQKMNDFNKSGS
IDGNSLVNNTLHQYLQSPFKKNS
>AbPK1_6

-MLNSIHQVLDSLGISPQKRAIHVQFTSPILNDQVFLQRIDGVHALNDGLKAELLCLSTNATIQLKSFIGVQ
AAVDIVTERGELTRVTGIITHAQQGQSDGSLTLYKLTLEDPTALWKYRRNSRVFMNKSVVEIWEILFKEWQT
KNPLFAASLSLDLSGLT-QTYDVRPFVMQHNESDWNFLTRLLRSENISWLIDEAQHIVPSTDTSIQAQKLRL
IDANSQYQPLDRKTIRYHRSSAVEQYDSMTRLTAERSLQPNVMHIQRWQAEILDQEEGIGSVQSKHQHSEHY
DNATLGLEQAWNYSPAWIGDLKGEDGVTKSGNQQVERLNQNLHNYYEAQAKRFIAQTTVRDAYVGYYFELNE
HPEIDQ-HESSDKSFLIVSKNFFNQNNLPKDLNDQINGLLAQSNWAIQNPE----NSDERQANQLILQRRHI
PTTPAYNPQIHSPVTHPQRAKVVGPEGEEIYVDEWGRIKVRFLFTRSDDHSHDGGAGTNNNDTDSAWIDVLT
PWAGEGYGARFLPRIGEIVVIDFFNGDIDRPFVMGRIHEAQRQPTKFDNKGKLPDTKKLSGIRSKEVSGGGF
GQLRFDDTPGQISTQLQSSHGASQLNLGKLSHPKDKAESEDRGEGFELRTDQWGALRAGQGLLVSTHKQDNA
KGDHLDAEVAKKQLEGSQTNSKALSDIAKNQKTDEIESI---------------------------------
---------------------------------------EQLKDFASQIQQ--------------------
---------------------------------------------------------------------QIAKF
EKALLLLSSPDGIALSSSEDIHISADAQINQIAGDSINISTQKNVIAHAQNRLSLFAAQSGLKAVAAQGKVE
IQAQSDALDVLANKGITISSTEDCIEISSPKEIVITGASSQITLNGSGIFPKTGGKFQVNAGQHVFQGGASA
SVQSSLPP------PPKRVQGVLELFHEYAHGEFVKGGSYRVV--DNFGKEVTGKLDDKGFAKVSGLAT-GA
VKVFFESDHRDPWDTASDFKRPVEWPNKNDADSEQSDSLIAQMSK----TAQSKLGELSKQLTNPTNIMKNI
QTAQSIKSEGAKALMPMLKTQAQGLVTDQVKSF---------LPISEAGQKIGNNIELTSIQKMNDFNKSGS
IDGNSLVNNTLHQYLQSPFKKNS
>AC29_6
-MLNSIHQVLDSLGISPQKRAIHVQFTSPILNDQVFLQRIDGVHALNDGLKAELLCLSTNATIQLKSFIGVQ
AAVDIVTERGELTRVTGIITHAQQGQSDGSLTLYKLTLEDPTALWKYRRNSRVFMNKSVVEIWEILFKEWQT
KNPLFAASLSLDLSGLT-QTYDVRPFVMQHNESDWNFLTRLLRSENISWLIDEAQHIVPSTDTSIQAQKLRL
IDANSQYQPLDRKTIRYHRSSAVEQYDSMTRLTAERSLQPNVMHIQRWQAEILDQEEGIGSVQSKHQHSEHY
DNATLGLEQAWNYSPAWIGDLKGEDGVTKSGNQQVERLNQNLHNYYEAQAKRFIAQTTVRDAYVGYYFELNE
HPEIDQ-HESSDKSFLIVSKNFFNQNNLPKDLNDQINGLLAQSNWAIQNPE----NSDERQANQLILQRRHI
PTTPAYNPQIHSPVTHPQRAKVVGPEGEEIYVDEWGRIKVRFLFTRSDDHSHDGGAGTNNNDTDSAWIDVLT
PWAGEGYGARFLPRIGEIVVIDFFNGDIDRPFVMGRIHEAQRQPTKFDNKGKLPDTKKLSGIRSKEVSGGGF
GQLRFDDTPGQISTQLQSSHGASQLNLGKLSHPKDKAESEDRGEGFELRTDQWGALRAGQGLLVSTHKQDNA
KGDHLDAEVAKKQLEGSQTNSKALSDIAKNQKTDEIESI---------------------------------
---------------------------------------EQLKDFASQIQQ--------------------
---------------------------------------------------------------------QIAKF
EKALLLLSSPDGIALSSSEDIHISADAQINQIAGDSINISTQKNVIAHAQNRLSLFAAQSGLKAVAAQGKVE
IQAQSDALDVLANKGITISSTEDCIEISSPKEIVITGASSQITLNGSGIFPKTGGKFQVNAGQHVFQGGASA

SVQSSLPP------PPKRVQGVLELFHEYAHGEFVKGGSYRVV--DNFGKEVTGKLDDKGFAKVSGLAT-GA
VKVFFESDHRDPWDTASDFKRPVEWPNKNDADSEQSDSLIAQMSK----TAQSKLGELSKQLTNPTNIMKNI
QTAQSIKSEGAKALMPMLKTQAQGLVTDQVKSF---------LPISEAGQKIGNNIELTSIQKMNDFNKSGS
IDGNSLVNNTLHQYLQSPFKKNS
>AC30_6
-MLNSIHQVLDSLGISPQKRAIHVQFTSPILNDQVFLQRIDGVHALNDGLKAELLCLSTNATIQLKSFIGVQ
AAVDIVTERGELTRVTGIITHAQQGQSDGSLTLYKLTLEDPTALWKYRRNSRVFMNKSVVEIWEILFKEWQT
KNPLFAASLSLDLSGLT-QTYDVRPFVMQHNESDWNFLTRLLRSENISWLIDEAQHIVPSTDTSIQAQKLRL
IDANSQYQPLDRKTIRYHRSSAVEQYDSMTRLTAERSLQPNVMHIQRWQAEILDQEEGIGSVQSKHQHSEHY
DNATLGLEQAWNYSPAWIGDLKGEDGVTKSGNQQVERLNQNLHNYYEAQAKRFIAQTTVRDAYVGYYFELNE
HPEIDQ-HESSDKSFLIVSKNFFNQNNLPKDLNDQINGLLAQSNWAIQNPE----NSDERQANQLILQRRHI
PTTPAYNPQIHSPVTHPQRAKVVGPEGEEIYVDEWGRIKVRFLFTRSDDHSHDGGAGTNNNDTDSAWIDVLT
PWAGEGYGARFLPRIGEIVVIDFFNGDIDRPFVMGRIHEAQRQPTKFDNKGKLPDTKKLSGIRSKEVSGGGF
GQLRFDDTPGQISTQLQSSHGASQLNLGKLSHPKDKAESEDRGEGFELRTDQWGALRAGQGLLVSTHKQDNA
KGDHLDAEVAKKQLEGSQTNSKALSDIAKNQKTDEIESI----------------------------------
----------------------------------------EQLKDFASQIQQ--------------------
--------------------------------------------------------------------QIAKF
EKALLLLSSPDGIALSSSEDIHISADAQINQIAGDSINISTQKNVIAHAQNRLSLFAAQSGLKAVAAQGKVE
IQAQSDALDVLANKGITISSTEDCIEISSPKEIVITGASSQITLNGSGIFPKTGGKFQVNAGQHVFQGGASA
SVQSSLPP------PPKRVQGVLELFHEYAHGEFVKGGSYRVV--DNFGKEVTGKLDDKGFAKVSGLAT-GA
VKVFFESDHRDPWDTASDFKRPVEWPNKNDADSEQSDSLIAQMSK----TAQSKLGELSKQLTNPTNIMKNI
QTAQSIKSEGAKALMPMLKTQAQGLVTDQVKSF---------LPISEAGQKIGNNIELTSIQKMNDFNKSGS
IDGNSLVNNTLHQYLQSPFKKNS
>ACICU_6
-MLNSIHQVLDSLGISPQKRAIHVQFTSPILNDQVFLQRIDGVHALNDGLKAELLCLSTNATIQLKSFIGVQ
AAVDIVTERGELTRVTGIITHAQQGQSDGSLTLYKLTLEDPTALWKYRRNSRVFMNKSVVEIWEILFKEWQT
KNPLFAASLSLDLSGLT-QTYDVRPFVMQHNESDWNFLTRLLRSENISWLIDEAQHIVPSTDTSIQAQKLRL
IDANSQYQPLDRKTIRYHRSSAVEQYDSMTRLTAERSLQPNVMHIQRWQAEILDQEEGIGSVQSKHQHSEHY
DNATLGLEQAWNYSPAWIGDLKGEDGVTKSGNQQVERLNQNLHNYYEAQAKRFIAQTTVRDAYVGYYFELNE
HPEIDQ-HESSDKSFLIVSKNFFNQNNLPKDLNDQINGLLAQSNWAIQNPE----NSDERQANQLILQRRHI
PTTPAYNPQIHSPVTHPQRAKVVGPEGEEIYVDEWGRIKVRFLFTRSDDHSHDGGAGTNNNDTDSAWIDVLT
PWAGEGYGARFLPRIGEIVVIDFFNGDIDRPFVMGRIHEAQRQPTKFDNKGKLPDTKKLSGIRSKEVSGGGF
GQLRFDDTPGQISTQLQSSHGASQLNLGKLSHPKDKAESEDRGEGFELRTDQWGALRAGQGLLVSTHKQDNA

KGDHLDAEVAKKQLEGSQTNSKALSDIAKNQKTDEIESI---------------------------------
----------------------------------------EQLKDFASQIQQ--------------------
-------------------------------------------------------------------QIAKF
EKALLLLSSPDGIALSSSEDIHISADAQINQIAGDSINISTQKNVIAHAQNRLSLFAAQSGLKAVAAQGKVE
IQAQSDALDVLANKGITISSTEDCIEISSPKEIVITGASSQITLNGSGIFPKTGGKFQVNAGQHVFQGGASA
SVQSSLPP------PPKRVQGVLELFHEYAHGEFVKGGSYRVV--DNFGKEVTGKLDDKGFAKVSGLAT-GA
VKVFFESDHRDPWDTASDFKRPVEWPNKNDADSEQSDSLIAQMSK----TAQSKLGELSKQLTNPTNIMKNI
QTAQSIKSEGAKALMPMLKTQAQGLVTDQVKSF---------LPISEAGQKIGNNIELTSIQKMNDFNKSGS
IDGNSLVNNTLHQYLQSPFKKNS
>AR_0056_6
-MLNSIHQVLDSLGISPQKRAIHVQFTSPILNDQVFLQRIDGVHALNDGLKAELLCLSTNATIQLKSFIGVQ
AAVDIVTERGELTRVTGIITHAQQGQSDGSLTLYKLTLEDPTALWKYRRNSRVFMNKSVVEIWEILFKEWQT
KNPLFAASLSLDLSGLT-QTYDVRPFVMQHNESDWNFLTRLLRSENISWLIDEAQHIVPSTDTSIQAQKLRL
IDANSQYQPLDRKTIRYHRSSAVEQYDSMTRLTAERSLQPNVMHIQRWQAEILDQEEGIGSVQSKHQHSEHY
DNATLGLEQAWNYSPAWIGDLKGEDGVTKSGNQQVERLNQNLHNYYEAQAKRFIAQTTVRDAYVGYYFELNE
HPEIDQ-HESSDKSFLIVSKNFFNQNNLPKDLNDQINGLLAQSNWAIQNPE----NSDERQANQLILQRRHI
PTTPAYNPQIHSPVTHPQRAKVVGPEGEEIYVDEWGRIKVRFLFTRSDDHSHDGGAGTNNNDTDSAWIDVLT
PWAGEGYGARFLPRIGEIVVIDFFNGDIDRPFVMGRIHEAQRQPTKFDNKGKLPDTKKLSGIRSKEVSGGGF
GQLRFDDTPGQISTQLQSSHGASQLNLGKLSHPKDKAESEDRGEGFELRTDQWGALRAGQGLLVSTHKQDNA
KGDHLDAEVAKKQLEGSQTNSKALSDIAKNQKTDEIESI---------------------------------
----------------------------------------EQLKDFASQIQQ--------------------
-------------------------------------------------------------------QIAKF
EKALLLLSSPDGIALSSSEDIHISADAQINQIAGDSINISTQKNVIAHAQNRLSLFAAQSGLKAVAAQGKVE
IQAQSDALDVLANKGITISSTEDCIEISSPKEIVITGASSQITLNGSGIFPKTGGKFQVNAGQHVFQGGASA
SVQSSLPP------PPKRVQGVLELFHEYAHGEFVKGGSYRVV--DNFGKEVTGKLDDKGFAKVSGLAT-GA
VKVFFESDHRDPWDTASDFKRPVEWPNKNDADSEQSDSLIAQMSK----TAQSKLGELSKQLTNPTNIMKNI
QTAQSIKSEGAKALMPMLKTQAQGLVTDQVKSF---------LPISEAGQKIGNNIELTSIQKMNDFNKSGS
IDGNSLVNNTLHQYLQSPFKKNS
>AR_0102_6
-MLNSIHQVLDSLGISPQKRAIHVQFTSPILNDQVFLQRIDGVHALNDGLKAELLCLSTNATIQLKSFIGVQ
AAVDIVTERGELTRVTGIITHAQQGQSDGSLTLYKLTLEDPTALWKYRRNSRVFMNKSVVEIWEILFKEWQT
KNPLFAASLSLDLSGLT-QTYDVRPFVMQHNESDWNFLTRLLRSENISWLIDEAQHIVPSTDTSIQAQKLRL
IDANSQYQPLDRKTIRYHRSSAVEQYDSMTRLTAERSLQPNVMHIQRWQAEILDQEEGIGSVQSKHQHSEHY

DNATLGLEQAWNYSPAWIGDLKGEDGVTKSGNQQVERLNQNLHNYYEAQAKRFIAQTTVRDAYVGYYFELNE
HPEIDQ-HESSDKSFLIVSKNFFNQNNLPKDLNDQINGLLAQSNWAIQNPE----NSDERQANQLILQRRHI
PTTPAYNPQIHSPVTHPQRAKVVGPEGEEIYVDEWGRIKVRFLFTRSDDHSHDGGAGTNNNDTDSAWIDVLT
PWAGEGYGARFLPRIGEIVVIDFFNGDIDRPFVMGRIHEAQRQPTKFDNKGKLPDTKKLSGIRSKEVSGGGF
GQLRFDDTPGQISTQLQSSHGASQLNLGKLSHPKDKAESEDRGEGFELRTDQWGALRAGQGLLVSTHKQDNA
KGDHLDAEVAKKQLEGSQTNSKALSDIAKNQKTDEIESI-----------------------------------
---------------------------------------EQLKDFASQIQQ--------------------
-----------------------------------------------------------------QIAKF
EKALLLLSSPDGIALSSSEDIHISADAQINQIAGDSINISTQKNVIAHAQNRLSLFAAQSGLKAVAAQGKVE
IQAQSDALDVLANKGITISSTEDCIEISSPKEIVITGASSQITLNGSGIFPKTGGKFQVNAGQHVFQGGASA
SVQSSLPP------PPKRVQGVLELFHEYAHGEFVKGGSYRVV--DNFGKEVTGKLDDKGFAKVSGLAT-GA
VKVFFESDHRDPWDTASDFKRPVEWPNKNDADSEQSDSLIAQMSK----TAQSKLGELSKQLTNPTNIMKNI
QTAQSIKSEGAKALMPMLKTQAQGLVTDQVKSF---------LPISEAGQKIGNNIELTSIQKMNDFNKSGS
IDGNSLVNNTLHQYLQSPFKKNS
>AYP-A2_6
-MLNSIHQVLDSLGISPQKRAIHVQFTSPILNDQVFLQRIDGVHALNDGLKAELLCLSTNATIQLKSFIGVQ
AAVDIVTERGELTRVTGIITHAQQGQSDGSLTLYKLTLEDPTALWKYRRNSRVFMNKSVVEIWEILFKEWQT
KNPLFAASLSLDLSGLT-QTYDVRPFVMQHNESDWNFLTRLLRSENISWLIDEAQHIVPSTDTSIQAQKLRL
IDANSQYQPLDRKTIRYHRSSAVEQYDSMTRLTAERSLQPNVMHIQRWQAEILDQEEGIGSVQSKHQHSEHY
DNATLGLEQAWNYSPAWIGDLKGEDGVTKSGNQQVERLNQNLHNYYEAQAKRFIAQTTVRDAYVGYYFELNE
HPEIDQ-HESSDKSFLIVSKNFFNQNNLPKDLNDQINGLLAQSNWAIQNPE----NSDERQANQLILQRRHI
PTTPAYNPQIHSPVTHPQRAKVVGPEGEEIYVDEWGRIKVRFLFTRSDDHSHDGGAGTNNNDTDSAWIDVLT
PWAGEGYGARFLPRIGEIVVIDFFNGDIDRPFVMGRIHEAQRQPTKFDNKGKLPDTKKLSGIRSKEVSGGGF
GQLRFDDTPGQISTQLQSSHGASQLNLGKLSHPKDKAESEDRGEGFELRTDQWGALRAGQGLLVSTHKQDNA
KGDHLDAEVAKKQLEGSQTNSKALSDIAKNQKTDEIESI-----------------------------------
---------------------------------------EQLKDFASQIQQ--------------------
-----------------------------------------------------------------QIAKF
EKALLLLSSPDGIALSSSEDIHISADAQINQIAGDSINISTQKNVIAHAQNRLSLFAAQSGLKAVAAQGKVE
IQAQSDALDVLANKGITISSTEDCIEISSPKEIVITGASSQITLNGSGIFPKTGGKFQVNAGQHVFQGGASA
SVQSSLPP------PPKRVQGVLELFHEYAHGEFVKGGSYRVV--DNFGKEVTGKLDDKGFAKVSGLAT-GA
VKVFFESDHRDPWDTASDFKRPVEWPNKNDADSEQSDSLIAQMSK----TAQSKLGELSKQLTNPTNIMKNI
QTAQSIKSEGAKALMPMLKTQAQGLVTDQVKSF---------LPISEAGQKIGNNIELTSIQKMNDFNKSGS
IDGNSLVNNTLHQYLQSPFKKNS

>BJAB07104_6
-MLNSIHQVLDSLGISPQKRAIHVQFTSPILNDQVFLQRIDGVHALNDGLKAELLCLSTNATIQLKSFIGVQ
AAVDIVTERGELTRVTGIITHAQQGQSDGSLTLYKLTLEDPTALWKYRRNSRVFMNKSVVEIWEILFKEWQT
KNPLFAASLSLDLSGLT-QTYDVRPFVMQHNESDWNFLTRLLRSENISWLIDEAQHIVPSTDTSIQAQKLRL
IDANSQYQPLDRKTIRYHRSSAVEQYDSMTRLTAERSLQPNVMHIQRWQAEILDQEEGIGSVQSKHQHSEHY
DNATLGLEQAWNYSPAWIGDLKGEDGVTKSGNQQVERLNQNLHNYYEAQAKRFIAQTTVRDAYVGYYFELNE
HPEIDQ-HESSDKSFLIVSKNFFNQNNLPKDLNDQINGLLAQSNWAIQNPE----NSDERQANQLILQRRHI
PTTPAYNPQIHSPVTHPQRAKVVGPEGEEIYVDEWGRIKVRFLFTRSDDHSHDGGAGTNNNDTDSAWIDVLT
PWAGEGYGARFLPRIGEIVVIDFFNGDIDRPFVMGRIHEAQRQPTKFDNKGKLPDTKKLSGIRSKEVSGGGF
GQLRFDDTPGQISTQLQSSHGASQLNLGKLSHPKDKAESEDRGEGFELRTDQWGALRAGQGLLVSTHKQDNA
KGDHLDAEVAKKQLEGSQTNSKALSDIAKNQKTDEIESI---------------------------------
----------------------------------------EQLKDFASQIQQ--------------------
-------------------------------------------------------------------QIAKF
EKALLLLSSPDGIALSSSEDIHISADAQINQIAGDSINISTQKNVIAHAQNRLSLFAAQSGLKAVAAQGKVE
IQAQSDALDVLANKGITISSTEDCIEISSPKEIVITGASSQITLNGSGIFPKTGGKFQVNAGQHVFQGGASA
SVQSSLPP------PPKRVQGVLELFHEYAHGEFVKGGSYRVV--DNFGKEVTGKLDDKGFAKVSGLAT-GA
VKVFFESDHRDPWDTASDFKRPVEWPNKNDADSEQSDSLIAQMSK----TAQSKLGELSKQLTNPTNIMKNI
QTAQSIKSEGAKALMPMLKTQAQGLVTDQVKSF---------LPISEAGQKIGNNIELTSIQKMNDFNKSGS
IDGNSLVNNTLHQYLQSPFKKNS
>BJAB0868_6
-MLNSIHQVLDSLGISPQKRAIHVQFTSPILNDQVFLQRIDGVHALNDGLKAELLCLSTNATIQLKSFIGVQ
AAVDIVTERGELTRVTGIITHAQQGQSDGSLTLYKLTLEDPTALWKYRRNSRVFMNKSVVEIWEILFKEWQT
KNPLFAASLSLDLSGLT-QTYDVRPFVMQHNESDWNFLTRLLRSENISWLIDEAQHIVPSTDTSIQAQKLRL
IDANSQYQPLDRKTIRYHRSSAVEQYDSMTRLTAERSLQPNVMHIQRWQAEILDQEEGIGSVQSKHQHSEHY
DNATLGLEQAWNYSPAWIGDLKGEDGVTKSGNQQVERLNQNLHNYYEAQAKRFIAQTTVRDAYVGYYFELNE
HPEIDQ-HESSDKSFLIVSKNFFNQNNLPKDLNDQINGLLAQSNWAIQNPE----NSDERQANQLILQRRHI
PTTPAYNPQIHSPVTHPQRAKVVGPEGEEIYVDEWGRIKVRFLFTRSDDHSHDGGAGTNNNDTDSAWIDVLT
PWAGEGYGARFLPRIGEIVVIDFFNGDIDRPFVMGRIHEAQRQPTKFDNKGKLPDTKKLSGIRSKEVSGGGF
GQLRFDDTPGQISTQLQSSHGASQLNLGKLSHPKDKAESEDRGEGFELRTDQWGALRAGQGLLVSTHKQDNA
KGDHLDAEVAKKQLEGSQTNSKALSDIAKNQKTDEIESI---------------------------------
----------------------------------------EQLKDFASQIQQ--------------------
-------------------------------------------------------------------QIAKF
EKALLLLSSPDGIALSSSEDIHISADAQINQIAGDSINISTQKNVIAHAQNRLSLFAAQSGLKAVAAQGKVE

IQAQSDALDVLANKGITISSTEDCIEISSPKEIVITGASSQITLNGSGIFPKTGGKFQVNAGQHVFQGGASA
SVQSSLPP------PPKRVQGVLELFHEYAHGEFVKGGSYRVV--DNFGKEVTGKLDDKGFAKVSGLAT-GA
VKVFFESDHRDPWDTASDFKRPVEWPNKNDADSEQSDSLIAQMSK----TAQSKLGELSKQLTNPTNIMKNI
QTAQSIKSEGAKALMPMLKTQAQGLVTDQVKSF---------LPISEAGQKIGNNIELTSIQKMNDFNKSGS
IDGNSLVNNTLHQYLQSPFKKNS
>CBA7_6
-MLNSIHQVLDSLGISPQKRAIHVQFTSPILNDQVFLQRIDGVHALNDGLKAELLCLSTNATIQLKSFIGVQ
AAVDIVTERGELTRVTGIITHAQQGQSDGSLTLYKLTLEDPTALWKYRRNSRVFMNKSVVEIWEILFKEWQT
KNPLFAASLSLDLSGLT-QTYDVRPFVMQHNESDWNFLTRLLRSENISWLIDEAQHIVPSTDTSIQAQKLRL
IDANSQYQPLDRKTIRYHRSSAVEQYDSMTRLTAERSLQPNVMHIQRWQAEILDQEEGIGSVQSKHQHSEHY
DNATLGLEQAWNYSPAWIGDLKGEDGVTKSGNQQVERLNQNLHNYYEAQAKRFIAQTTVRDAYVGYYFELNE
HPEIDQ-HESSDKSFLIVSKNFFNQNNLPKDLNDQINGLLAQSNWAIQNPE----NSDERQANQLILQRRHI
PTTPAYNPQIHSPVTHPQRAKVVGPEGEEIYVDEWGRIKVRFLFTRSDDHSHDGGAGTNNNDTDSAWIDVLT
PWAGEGYGARFLPRIGEIVVIDFFNGDIDRPFVMGRIHEAQRQPTKFDNKGKLPDTKKLSGIRSKEVSGGGF
GQLRFDDTPGQISTQLQSSHGASQLNLGKLSHPKDKAESEDRGEGFELRTDQWGALRAGQGLLVSTHKQDNA
KGDHLDAEVAKKQLEGSQTNSKALSDIAKNQKTDEIESI---------------------------------
----------------------------------------EQLKDFASQIQQ--------------------
--------------------------------------------------------------------QIAKF
EKALLLLSSPDGIALSSSEDIHISADAQINQIAGDSINISTQKNVIAHAQNRLSLFAAQSGLKAVAAQGKVE
IQAQSDALDVLANKGITISSTEDCIEISSPKEIVITGASSQITLNGSGIFPKTGGKFQVNAGQHVFQGGASA
SVQSSLPP------PPKRVQGVLELFHEYAHGEFVKGGSYRVV--DNFGKEVTGKLDDKGFAKVSGLAT-GA
VKVFFESDHRDPWDTASDFKRPVEWPNKNDADSEQSDSLIAQMSK----TAQSKLGELSKQLTNPTNIMKNI
QTAQSIKSEGAKALMPMLKTQAQGLVTDQVKSF---------LPISEAGQKIGNNIELTSIQKMNDFNKSGS
IDGNSLVNNTLHQYLQSPFKKNS
>HRAB-85_6
-MLNSIHQVLDSLGISPQKRAIHVQFTSPILNDQVFLQRIDGVHALNDGLKAELLCLSTNATIQLKSFIGVQ
AAVDIVTERGELTRVTGIITHAQQGQSDGSLTLYKLTLEDPTALWKYRRNSRVFMNKSVVEIWEILFKEWQT
KNPLFAASLSLDLSGLT-QTYDVRPFVMQHNESDWNFLTRLLRSENISWLIDEAQHIVPSTDTSIQAQKLRL
IDANSQYQPLDRKTIRYHRSSAVEQYDSMTRLTAERSLQPNVMHIQRWQAEILDQEEGIGSVQSKHQHSEHY
DNATLGLEQAWNYSPAWIGDLKGEDGVTKSGNQQVERLNQNLHNYYEAQAKRFIAQTTVRDAYVGYYFELNE
HPEIDQ-HESSDKSFLIVSKNFFNQNNLPKDLNDQINGLLAQSNWAIQNPE----NSDERQANQLILQRRHI
PTTPAYNPQIHSPVTHPQRAKVVGPEGEEIYVDEWGRIKVRFLFTRSDDHSHDGGAGTNNNDTDSAWIDVLT
PWAGEGYGARFLPRIGEIVVIDFFNGDIDRPFVMGRIHEAQRQPTKFDNKGKLPDTKKLSGIRSKEVSGGGF

GQLRFDDTPGQISTQLQSSHGASQLNLGKLSHPKDKAESEDRGEGFELRTDQWGALRAGQGLLVSTHKQDNA
KGDHLDAEVAKKQLEGSQTNSKALSDIAKNQKTDEIESI---------------------------------
----------------------------------------EQLKDFASQIQQ--------------------
-------------------------------------------------------------------QIAKF
EKALLLLLSSPDGIALSSSEDIHISADAQINQIAGDSINISTQKNVIAHAQNRLSLFAAQSGLKAVAAQGKVE
IQAQSDALDVLANKGITISSTEDCIEISSPKEIVITGASSQITLNGSGIFPKTGGKFQVNAGQHVFQGGASA
SVQSSLPP------PPKRVQGVLELFHEYAHGEFVKGGSYRVV--DNFGKEVTGKLDDKGFAKVSGLAT-GA
VKVFFESDHRDPWDTASDFKRPVEWPNKNDADSEQSDSLIAQMSK----TAQSKLGELSKQLTNPTNIMKNI
QTAQSIKSEGAKALMPMLKTQAQGLVTDQVKSF---------LPISEAGQKIGNNIELTSIQKMNDFNKSGS
IDGNSLVNNTLHQYLQSPFKKNS
>JBA13_6
-MLNSIHQVLDSLGISPQKRAIHVQFTSPILNDQVFLQRIDGVHALNDGLKAELLCLSTNATIQLKSFIGVQ
AAVDIVTERGELTRVTGIITHAQQGQSDGSLTLYKLTLEDPTALWKYRRNSRVFMNKSVVEIWEILFKEWQT
KNPLFAASLSLDLSGLT-QTYDVRPFVMQHNESDWNFLTRLLRSENISWLIDEAQHIVPSTDTSIQAQKLRL
IDANSQYQPLDRKTIRYHRSSAVEQYDSMTRLTAERSLQPNVMHIQRWQAEILDQEEGIGSVQSKHQHSEHY
DNATLGLEQAWNYSPAWIGDLKGEDGVTKSGNQQVERLNQNLHNYYEAQAKRFIAQTTVRDAYVGYYFELNE
HPEIDQ-HESSDKSFLIVSKNFFNQNNLPKDLNDQINGLLAQSNWAIQNPE----NSDERQANQLILQRRHI
PTTPAYNPQIHSPVTHPQRAKVVGPEGEEIYVDEWGRIKVRFLFTRSDDHSHDGGAGTNNNDTDSAWIDVLT
PWAGEGYGARFLPRIGEIVVIDFFNGDIDRPFVMGRIHEAQRQPTKFDNKGKLPDTKKLSGIRSKEVSGGGF
GQLRFDDTPGQISTQLQSSHGASQLNLGKLSHPKDKAESEDRGEGFELRTDQWGALRAGQGLLVSTHKQDNA
KGDHLDAEVAKKQLEGSQTNSKALSDIAKNQKTDEIESI---------------------------------
----------------------------------------EQLKDFASQIQQ--------------------
-------------------------------------------------------------------QIAKF
EKALLLLLSSPDGIALSSSEDIHISADAQINQIAGDSINISTQKNVIAHAQNRLSLFAAQSGLKAVAAQGKVE
IQAQSDALDVLANKGITISSTEDCIEISSPKEIVITGASSQITLNGSGIFPKTGGKFQVNAGQHVFQGGASA
SVQSSLPP------PPKRVQGVLELFHEYAHGEFVKGGSYRVV--DNFGKEVTGKLDDKGFAKVSGLAT-GA
VKVFFESDHRDPWDTASDFKRPVEWPNKNDADSEQSDSLIAQMSK----TAQSKLGELSKQLTNPTNIMKNI
QTAQSIKSEGAKALMPMLKTQAQGLVTDQVKSF---------LPISEAGQKIGNNIELTSIQKMNDFNKSGS
IDGNSLVNNTLHQYLQSPFKKNS
>KAB01_6
-MLNSIHQVLDSLGISPQKRAIHVQFTSPILNDQVFLQRIDGVHALNDGLKAELLCLSTNATIQLKSFIGVQ
AAVDIVTERGELTRVTGIITHAQQGQSDGSLTLYKLTLEDPTALWKYRRNSRVFMNKSVVEIWEILFKEWQT
KNPLFAASLSLDLSGLT-QTYDVRPFVMQHNESDWNFLTRLLRSENISWLIDEAQHIVPSTDTSIQAQKLRL

IDANSQYQPLDRKTIRYHRSSAVEQYDSMTRLTAERSLQPNVMHIQRWQAEILDQEEGIGSVQSKHQHSEHY
DNATLGLEQAWNYSPAWIGDLKGEDGVTKSGNQQVERLNQNLHNYYEAQAKRFIAQTTVRDAYVGYYFELNE
HPEIDQ-HESSDKSFLIVSKNFFNQNNLPKDLNDQINGLLAQSNWAIQNPE----NSDERQANQLILQRRHI
PTTPAYNPQIHSPVTHPQRAKVVGPEGEEIYVDEWGRIKVRFLFTRSDDHSHDGGAGTNNNDTDSAWIDVLT
PWAGEGYGARFLPRIGEIVVIDFFNGDIDRPFVMGRIHEAQRQPTKFDNKGKLPDTKKLSGIRSKEVSGGGF
GQLRFDDTPGQISTQLQSSHGASQLNLGKLSHPKDKAESEDRGEGFELRTDQWGALRAGQGLLVSTHKQDNA
KGDHLDAEVAKKQLEGSQTNSKALSDIAKNQKTDEIESI-----------------------------------
----------------------------------------EQLKDFASQIQQ--------------------
-------------------------------------------------------------------QIAKF
EKALLLLSSPDGIALSSSEDIHISADAQINQIAGDSINISTQKNVIAHAQNRLSLFAAQSGLKAVAAQGKVE
IQAQSDALDVLANKGITISSTEDCIEISSPKEIVITGASSQITLNGSGIFPKTGGKFQVNAGQHVFQGGASA
SVQSSLPP------PPKRVQGVLELFHEYAHGEFVKGGSYRVV--DNFGKEVTGKLDDKGFAKVSGLAT-GA
VKVFFESDHRDPWDTASDFKRPVEWPNKNDADSEQSDSLIAQMSK----TAQSKLGELSKQLTNPTNIMKNI
QTAQSIKSEGAKALMPMLKTQAQGLVTDQVKSF---------LPISEAGQKIGNNIELTSIQKMNDFNKSGS
IDGNSLVNNTLHQYLQSPFKKNS
>KAB02_6
-MLNSIHQVLDSLGISPQKRAIHVQFTSPILNDQVFLQRIDGVHALNDGLKAELLCLSTNATIQLKSFIGVQ
AAVDIVTERGELTRVTGIITHAQQGQSDGSLTLYKLTLEDPTALWKYRRNSRVFMNKSVVEIWEILFKEWQT
KNPLFAASLSLDLSGLT-QTYDVRPFVMQHNESDWNFLTRLLRSENISWLIDEAQHIVPSTDTSIQAQKLRL
IDANSQYQPLDRKTIRYHRSSAVEQYDSMTRLTAERSLQPNVMHIQRWQAEILDQEEGIGSVQSKHQHSEHY
DNATLGLEQAWNYSPAWIGDLKGEDGVTKSGNQQVERLNQNLHNYYEAQAKRFIAQTTVRDAYVGYYFELNE
HPEIDQ-HESSDKSFLIVSKNFFNQNNLPKDLNDQINGLLAQSNWAIQNPE----NSDERQANQLILQRRHI
PTTPAYNPQIHSPVTHPQRAKVVGPEGEEIYVDEWGRIKVRFLFTRSDDHSHDGGAGTNNNDTDSAWIDVLT
PWAGEGYGARFLPRIGEIVVIDFFNGDIDRPFVMGRIHEAQRQPTKFDNKGKLPDTKKLSGIRSKEVSGGGF
GQLRFDDTPGQISTQLQSSHGASQLNLGKLSHPKDKAESEDRGEGFELRTDQWGALRAGQGLLVSTHKQDNA
KGDHLDAEVAKKQLEGSQTNSKALSDIAKNQKTDEIESI-----------------------------------
----------------------------------------EQLKDFASQIQQ--------------------
-------------------------------------------------------------------QIAKF
EKALLLLSSPDGIALSSSEDIHISADAQINQIAGDSINISTQKNVIAHAQNRLSLFAAQSGLKAVAAQGKVE
IQAQSDALDVLANKGITISSTEDCIEISSPKEIVITGASSQITLNGSGIFPKTGGKFQVNAGQHVFQGGASA
SVQSSLPP------PPKRVQGVLELFHEYAHGEFVKGGSYRVV--DNFGKEVTGKLDDKGFAKVSGLAT-GA
VKVFFESDHRDPWDTASDFKRPVEWPNKNDADSEQSDSLIAQMSK----TAQSKLGELSKQLTNPTNIMKNI
QTAQSIKSEGAKALMPMLKTQAQGLVTDQVKSF---------LPISEAGQKIGNNIELTSIQKMNDFNKSGS

IDGNSLVNNTLHQYLQSPFKKNS
>KAB03_6
-MLNSIHQVLDSLGISPQKRAIHVQFTSPILNDQVFLQRIDGVHALNDGLKAELLCLSTNATIQLKSFIGVQ
AAVDIVTERGELTRVTGIITHAQQGQSDGSLTLYKLTLEDPTALWKYRRNSRVFMNKSVVEIWEILFKEWQT
KNPLFAASLSLDLSGLT-QTYDVRPFVMQHNESDWNFLTRLLRSENISWLIDEAQHIVPSTDTSIQAQKLRL
IDANSQYQPLDRKTIRYHRSSAVEQYDSMTRLTAERSLQPNVMHIQRWQAEILDQEEGIGSVQSKHQHSEHY
DNATLGLEQAWNYSPAWIGDLKGEDGVTKSGNQQVERLNQNLHNYYEAQAKRFIAQTTVRDAYVGYYFELNE
HPEIDQ-HESSDKSFLIVSKNFFNQNNLPKDLNDQINGLLAQSNWAIQNPE----NSDERQANQLILQRRHI
PTTPAYNPQIHSPVTHPQRAKVVGPEGEEIYVDEWGRIKVRFLFTRSDDHSHDGGAGTNNNDTDSAWIDVLT
PWAGEGYGARFLPRIGEIVVIDFFNGDIDRPFVMGRIHEAQRQPTKFDNKGKLPDTKKLSGIRSKEVSGGGF
GQLRFDDTPGQISTQLQSSHGASQLNLGKLSHPKDKAESEDRGEGFELRTDQWGALRAGQGLLVSTHKQDNA
KGDHLDAEVAKKQLEGSQTNSKALSDIAKNQKTDEIESI-----------------------------------
----------------------------------------EQLKDFASQIQQ--------------------
---------------------------------------------------------------------QIAKF
EKALLLLSSPDGIALSSSEDIHISADAQINQIAGDSINISTQKNVIAHAQNRLSLFAAQSGLKAVAAQGKVE
IQAQSDALDVLANKGITISSTEDCIEISSPKEIVITGASSQITLNGSGIFPKTGGKFQVNAGQHVFQGGASA
SVQSSLPP------PPKRVQGVLELFHEYAHGEFVKGGSYRVV--DNFGKEVTGKLDDKGFAKVSGLAT-GA
VKVFFESDHRDPWDTASDFKRPVEWPNKNDADSEQSDSLIAQMSK----TAQSKLGELSKQLTNPTNIMKNI
QTAQSIKSEGAKALMPMLKTQAQGLVTDQVKSF---------LPISEAGQKIGNNIELTSIQKMNDFNKSGS
IDGNSLVNNTLHQYLQSPFKKNS
>KAB04_6
-MLNSIHQVLDSLGISPQKRAIHVQFTSPILNDQVFLQRIDGVHALNDGLKAELLCLSTNATIQLKSFIGVQ
AAVDIVTERGELTRVTGIITHAQQGQSDGSLTLYKLTLEDPTALWKYRRNSRVFMNKSVVEIWEILFKEWQT
KNPLFAASLSLDLSGLT-QTYDVRPFVMQHNESDWNFLTRLLRSENISWLIDEAQHIVPSTDTSIQAQKLRL
IDANSQYQPLDRKTIRYHRSSAVEQYDSMTRLTAERSLQPNVMHIQRWQAEILDQEEGIGSVQSKHQHSEHY
DNATLGLEQAWNYSPAWIGDLKGEDGVTKSGNQQVERLNQNLHNYYEAQAKRFIAQTTVRDAYVGYYFELNE
HPEIDQ-HESSDKSFLIVSKNFFNQNNLPKDLNDQINGLLAQSNWAIQNPE----NSDERQANQLILQRRHI
PTTPAYNPQIHSPVTHPQRAKVVGPEGEEIYVDEWGRIKVRFLFTRSDDHSHDGGAGTNNNDTDSAWIDVLT
PWAGEGYGARFLPRIGEIVVIDFFNGDIDRPFVMGRIHEAQRQPTKFDNKGKLPDTKKLSGIRSKEVSGGGF
GQLRFDDTPGQISTQLQSSHGASQLNLGKLSHPKDKAESEDRGEGFELRTDQWGALRAGQGLLVSTHKQDNA
KGDHLDAEVAKKQLEGSQTNSKALSDIAKNQKTDEIESI-----------------------------------
----------------------------------------EQLKDFASQIQQ--------------------
---------------------------------------------------------------------QIAKF

```
EKALLLLSSPDGIALSSSEDIHISADAQINQIAGDSINISTQKNVIAHAQNRLSLFAAQSGLKAVAAQGKVE
IQAQSDALDVLANKGITISSTEDCIEISSPKEIVITGASSQITLNGSGIFPKTGGKFQVNAGQHVFQGGASA
SVQSSLPP------PPKRVQGVLELFHEYAHGEFVKGGSYRVV--DNFGKEVTGKLDDKGFAKVSGLAT-GA
VKVFFESDHRDPWDTASDFKRPVEWPNKNDADSEQSDSLIAQMSK----TAQSKLGELSKQLTNPTNIMKNI
QTAQSIKSEGAKALMPMLKTQAQGLVTDQVKSF---------LPISEAGQKIGNNIELTSIQKMNDFNKSGS
IDGNSLVNNTLHQYLQSPFKKNS
>KAB05_6
-MLNSIHQVLDSLGISPQKRAIHVQFTSPILNDQVFLQRIDGVHALNDGLKAELLCLSTNATIQLKSFIGVQ
AAVDIVTERGELTRVTGIITHAQQGQSDGSLTLYKLTLEDPTALWKYRRNSRVFMNKSVVEIWEILFKEWQT
KNPLFAASLSLDLSGLT-QTYDVRPFVMQHNESDWNFLTRLLRSENISWLIDEAQHIVPSTDTSIQAQKLRL
IDANSQYQPLDRKTIRYHRSSAVEQYDSMTRLTAERSLQPNVMHIQRWQAEILDQEEGIGSVQSKHQHSEHY
DNATLGLEQAWNYSPAWIGDLKGEDGVTKSGNQQVERLNQNLHNYYEAQAKRFIAQTTVRDAYVGYYFELNE
HPEIDQ-HESSDKSFLIVSKNFFNQNNLPKDLNDQINGLLAQSNWAIQNPE----NSDERQANQLILQRRHI
PTTPAYNPQIHSPVTHPQRAKVVGPEGEEIYVDEWGRIKVRFLFTRSDDHSHDGGAGTNNNDTDSAWIDVLT
PWAGEGYGARFLPRIGEIVVIDFFNGDIDRPFVMGRIHEAQRQPTKFDNKGKLPDTKKLSGIRSKEVSGGGF
GQLRFDDTPGQISTQLQSSHGASQLNLGKLSHPKDKAESEDRGEGFELRTDQWGALRAGQGLLVSTHKQDNA
KGDHLDAEVAKKQLEGSQTNSKALSDIAKNQKTDEIESI---------------------------------
----------------------------------------EQLKDFASQIQQ--------------------
-------------------------------------------------------------------QIAKF
EKALLLLSSPDGIALSSSEDIHISADAQINQIAGDSINISTQKNVIAHAQNRLSLFAAQSGLKAVAAQGKVE
IQAQSDALDVLANKGITISSTEDCIEISSPKEIVITGASSQITLNGSGIFPKTGGKFQVNAGQHVFQGGASA
SVQSSLPP------PPKRVQGVLELFHEYAHGEFVKGGSYRVV--DNFGKEVTGKLDDKGFAKVSGLAT-GA
VKVFFESDHRDPWDTASDFKRPVEWPNKNDADSEQSDSLIAQMSK----TAQSKLGELSKQLTNPTNIMKNI
QTAQSIKSEGAKALMPMLKTQAQGLVTDQVKSF---------LPISEAGQKIGNNIELTSIQKMNDFNKSGS
IDGNSLVNNTLHQYLQSPFKKNS
>KAB06_6
-MLNSIHQVLDSLGISPQKRAIHVQFTSPILNDQVFLQRIDGVHALNDGLKAELLCLSTNATIQLKSFIGVQ
AAVDIVTERGELTRVTGIITHAQQGQSDGSLTLYKLTLEDPTALWKYRRNSRVFMNKSVVEIWEILFKEWQT
KNPLFAASLSLDLSGLT-QTYDVRPFVMQHNESDWNFLTRLLRSENISWLIDEAQHIVPSTDTSIQAQKLRL
IDANSQYQPLDRKTIRYHRSSAVEQYDSMTRLTAERSLQPNVMHIQRWQAEILDQEEGIGSVQSKHQHSEHY
DNATLGLEQAWNYSPAWIGDLKGEDGVTKSGNQQVERLNQNLHNYYEAQAKRFIAQTTVRDAYVGYYFELNE
HPEIDQ-HESSDKSFLIVSKNFFNQNNLPKDLNDQINGLLAQSNWAIQNPE----NSDERQANQLILQRRHI
PTTPAYNPQIHSPVTHPQRAKVVGPEGEEIYVDEWGRIKVRFLFTRSDDHSHDGGAGTNNNDTDSAWIDVLT
```

PWAGEGYGARFLPRIGEIVVIDFFNGDIDRPFVMGRIHEAQRQPTKFDNKGKLPDTKKLSGIRSKEVSGGGF
GQLRFDDTPGQISTQLQSSHGASQLNLGKLSHPKDKAESEDRGEGFELRTDQWGALRAGQGLLVSTHKQDNA
KGDHLDAEVAKKQLEGSQTNSKALSDIAKNQKTDEIESI---------------------------------
----------------------------------------EQLKDFASQIQQ--------------------
-------------------------------------------------------------------QIAKF
EKALLLLSSPDGIALSSSEDIHISADAQINQIAGDSINISTQKNVIAHAQNRLSLFAAQSGLKAVAAQGKVE
IQAQSDALDVLANKGITISSTEDCIEISSPKEIVITGASSQITLNGSGIFPKTGGKFQVNAGQHVFQGGASA
SVQSSLPP------PPKRVQGVLELFHEYAHGEFVKGGSYRVV--DNFGKEVTGKLDDKGFAKVSGLAT-GA
VKVFFESDHRDPWDTASDFKRPVEWPNKNDADSEQSDSLIAQMSK----TAQSKLGELSKQLTNPTNIMKNI
QTAQSIKSEGAKALMPMLKTQAQGLVTDQVKSF---------LPISEAGQKIGNNIELTSIQKMNDFNKSGS
IDGNSLVNNTLHQYLQSPFKKNS
>KAB07_6
-MLNSIHQVLDSLGISPQKRAIHVQFTSPILNDQVFLQRIDGVHALNDGLKAELLCLSTNATIQLKSFIGVQ
AAVDIVTERGELTRVTGIITHAQQGQSDGSLTLYKLTLEDPTALWKYRRNSRVFMNKSVVEIWEILFKEWQT
KNPLFAASLSLDLSGLT-QTYDVRPFVMQHNESDWNFLTRLLRSENISWLIDEAQHIVPSTDTSIQAQKLRL
IDANSQYQPLDRKTIRYHRSSAVEQYDSMTRLTAERSLQPNVMHIQRWQAEILDQEEGIGSVQSKHQHSEHY
DNATLGLEQAWNYSPAWIGDLKGEDGVTKSGNQQVERLNQNLHNYYEAQAKRFIAQTTVRDAYVGYYFELNE
HPEIDQ-HESSDKSFLIVSKNFFNQNNLPKDLNDQINGLLAQSNWAIQNPE----NSDERQANQLILQRRHI
PTTPAYNPQIHSPVTHPQRAKVVGPEGEEIYVDEWGRIKVRFLFTRSDDHSHDGGAGTNNNDTDSAWIDVLT
PWAGEGYGARFLPRIGEIVVIDFFNGDIDRPFVMGRIHEAQRQPTKFDNKGKLPDTKKLSGIRSKEVSGGGF
GQLRFDDTPGQISTQLQSSHGASQLNLGKLSHPKDKAESEDRGEGFELRTDQWGALRAGQGLLVSTHKQDNA
KGDHLDAEVAKKQLEGSQTNSKALSDIAKNQKTDEIESI---------------------------------
----------------------------------------EQLKDFASQIQQ--------------------
-------------------------------------------------------------------QIAKF
EKALLLLSSPDGIALSSSEDIHISADAQINQIAGDSINISTQKNVIAHAQNRLSLFAAQSGLKAVAAQGKVE
IQAQSDALDVLANKGITISSTEDCIEISSPKEIVITGASSQITLNGSGIFPKTGGKFQVNAGQHVFQGGASA
SVQSSLPP------PPKRVQGVLELFHEYAHGEFVKGGSYRVV--DNFGKEVTGKLDDKGFAKVSGLAT-GA
VKVFFESDHRDPWDTASDFKRPVEWPNKNDADSEQSDSLIAQMSK----TAQSKLGELSKQLTNPTNIMKNI
QTAQSIKSEGAKALMPMLKTQAQGLVTDQVKSF---------LPISEAGQKIGNNIELTSIQKMNDFNKSGS
IDGNSLVNNTLHQYLQSPFKKNS
>KAB08_6
-MLNSIHQVLDSLGISPQKRAIHVQFTSPILNDQVFLQRIDGVHALNDGLKAELLCLSTNATIQLKSFIGVQ
AAVDIVTERGELTRVTGIITHAQQGQSDGSLTLYKLTLEDPTALWKYRRNSRVFMNKSVVEIWEILFKEWQT

KNPLFAASLSLDLSGLT-QTYDVRPFVMQHNESDWNFLTRLLRSENISWLIDEAQHIVPSTDTSIQAQKLRL
IDANSQYQPLDRKTIRYHRSSAVEQYDSMTRLTAERSLQPNVMHIQRWQAEILDQEEGIGSVQSKHQHSEHY
DNATLGLEQAWNYSPAWIGDLKGEDGVTKSGNQQVERLNQNLHNYYEAQAKRFIAQTTVRDAYVGYYFELNE
HPEIDQ-HESSDKSFLIVSKNFFNQNNLPKDLNDQINGLLAQSNWAIQNPE----NSDERQANQLILQRRHI
PTTPAYNPQIHSPVTHPQRAKVVGPEGEEIYVDEWGRIKVRFLFTRSDDHSHDGGAGTNNNDTDSAWIDVLT
PWAGEGYGARFLPRIGEIVVIDFFNGDIDRPFVMGRIHEAQRQPTKFDNKGKLPDTKKLSGIRSKEVSGGGF
GQLRFDDTPGQISTQLQSSHGASQLNLGKLSHPKDKAESEDRGEGFELRTDQWGALRAGQGLLVSTHKQDNA
KGDHLDAEVAKKQLEGSQTNSKALSDIAKNQKTDEIESI----------------------------------
----------------------------------------EQLKDFASQIQQ--------------------
---------------------------------------------------------------------QIAKF
EKALLLLSSPDGIALSSSEDIHISADAQINQIAGDSINISTQKNVIAHAQNRLSLFAAQSGLKAVAAQGKVE
IQAQSDALDVLANKGITISSTEDCIEISSPKEIVITGASSQITLNGSGIFPKTGGKFQVNAGQHVFQGGASA
SVQSSLPP------PPKRVQGVLELFHEYAHGEFVKGGSYRVV--DNFGKEVTGKLDDKGFAKVSGLAT-GA
VKVFFESDHRDPWDTASDFKRPVEWPNKNDADSEQSDSLIAQMSK----TAQSKLGELSKQLTNPTNIMKNI
QTAQSIKSEGAKALMPMLKTQAQGLVTDQVKSF---------LPISEAGQKIGNNIELTSIQKMNDFNKSGS
IDGNSLVNNTLHQYLQSPFKKNS
>KBN10P02143_6
-MLNSIHQVLDSLGISPQKRAIHVQFTSPILNDQVFLQRIDGVHALNDGLKAELLCLSTNATIQLKSFIGVQ
AAVDIVTERGELTRVTGIITHAQQGQSDGSLTLYKLTLEDPTALWKYRRNSRVFMNKSVVEIWEILFKEWQT
KNPLFAASLSLDLSGLT-QTYDVRPFVMQHNESDWNFLTRLLRSENISWLIDEAQHIVPSTDTSIQAQKLRL
IDANSQYQPLDRKTIRYHRSSAVEQYDSMTRLTAERSLQPNVMHIQRWQAEILDQEEGIGSVQSKHQHSEHY
DNATLGLEQAWNYSPAWIGDLKGEDGVTKSGNQQVERLNQNLHNYYEAQAKRFIAQTTVRDAYVGYYFELNE
HPEIDQ-HESSDKSFLIVSKNFFNQNNLPKDLNDQINGLLAQSNWAIQNPE----NSDERQANQLILQRRHI
PTTPAYNPQIHSPVTHPQRAKVVGPEGEEIYVDEWGRIKVRFLFTRSDDHSHDGGAGTNNNDTDSAWIDVLT
PWAGEGYGARFLPRIGEIVVIDFFNGDIDRPFVMGRIHEAQRQPTKFDNKGKLPDTKKLSGIRSKEVSGGGF
GQLRFDDTPGQISTQLQSSHGASQLNLGKLSHPKDKAESEDRGEGFELRTDQWGALRAGQGLLVSTHKQDNA
KGDHLDAEVAKKQLEGSQTNSKALSDIAKNQKTDEIESI----------------------------------
----------------------------------------EQLKDFASQIQQ--------------------
---------------------------------------------------------------------QIAKF
EKALLLLSSPDGIALSSSEDIHISADAQINQIAGDSINISTQKNVIAHAQNRLSLFAAQSGLKAVAAQGKVE
IQAQSDALDVLANKGITISSTEDCIEISSPKEIVITGASSQITLNGSGIFPKTGGKFQVNAGQHVFQGGASA
SVQSSLPP------PPKRVQGVLELFHEYAHGEFVKGGSYRVV--DNFGKEVTGKLDDKGFAKVSGLAT-GA
VKVFFESDHRDPWDTASDFKRPVEWPNKNDADSEQSDSLIAQMSK----TAQSKLGELSKQLTNPTNIMKNI

QTAQSIKSEGAKALMPMLKTQAQGLVTDQVKSF---------LPISEAGQKIGNNIELTSIQKMNDFNKSGS
IDGNSLVNNTLHQYLQSPFKKNS
>MDR-TJ_6
-MLNSIHQVLDSLGISPQKRAIHVQFTSPILNDQVFLQRIDGVHALNDGLKAELLCLSTNATIQLKSFIGVQ
AAVDIVTERGELTRVTGIITHAQQGQSDGSLTLYKLTLEDPTALWKYRRNSRVFMNKSVVEIWEILFKEWQT
KNPLFAASLSLDLSGLT-QTYDVRPFVMQHNESDWNFLTRLLRSENISWLIDEAQHIVPSTDTSIQAQKLRL
IDANSQYQPLDRKTIRYHRSSAVEQYDSMTRLTAERSLQPNVMHIQRWQAEILDQEEGIGSVQSKHQHSEHY
DNATLGLEQAWNYSPAWIGDLKGEDGVTKSGNQQVERLNQNLHNYYEAQAKRFIAQTTVRDAYVGYYFELNE
HPEIDQ-HESSDKSFLIVSKNFFNQNNLPKDLNDQINGLLAQSNWAIQNPE----NSDERQANQLILQRRHI
PTTPAYNPQIHSPVTHPQRAKVVGPEGEEIYVDEWGRIKVRFLFTRSDDHSHDGGAGTNNNDTDSAWIDVLT
PWAGEGYGARFLPRIGEIVVIDFFNGDIDRPFVMGRIHEAQRQPTKFDNKGKLPDTKKLSGIRSKEVSGGGF
GQLRFDDTPGQISTQLQSSHGASQLNLGKLSHPKDKAESEDRGEGFELRTDQWGALRAGQGLLVSTHKQDNA
KGDHLDAEVAKKQLEGSQTNSKALSDIAKNQKTDEIESI-----------------------------------
---------------------------------------EQLKDFASQIQQ--------------------
-----------------------------------------------------------------QIAKF
EKALLLLSSPDGIALSSSEDIHISADAQINQIAGDSINISTQKNVIAHAQNRLSLFAAQSGLKAVAAQGKVE
IQAQSDALDVLANKGITISSTEDCIEISSPKEIVITGASSQITLNGSGIFPKTGGKFQVNAGQHVFQGGASA
SVQSSLPP------PPKRVQGVLELFHEYAHGEFVKGGSYRVV--DNFGKEVTGKLDDKGFAKVSGLAT-GA
VKVFFESDHRDPWDTASDFKRPVEWPNKNDADSEQSDSLIAQMSK----TAQSKLGELSKQLTNPTNIMKNI
QTAQSIKSEGAKALMPMLKTQAQGLVTDQVKSF---------LPISEAGQKIGNNIELTSIQKMNDFNKSGS
IDGNSLVNNTLHQYLQSPFKKNS
>MDR-ZJ06_6
-MLNSIHQVLDSLGISPQKRAIHVQFTSPILNDQVFLQRIDGVHALNDGLKAELLCLSTNATIQLKSFIGVQ
AAVDIVTERGELTRVTGIITHAQQGQSDGSLTLYKLTLEDPTALWKYRRNSRVFMNKSVVEIWEILFKEWQT
KNPLFAASLSLDLSGLT-QTYDVRPFVMQHNESDWNFLTRLLRSENISWLIDEAQHIVPSTDTSIQAQKLRL
IDANSQYQPLDRKTIRYHRSSAVEQYDSMTRLTAERSLQPNVMHIQRWQAEILDQEEGIGSVQSKHQHSEHY
DNATLGLEQAWNYSPAWIGDLKGEDGVTKSGNQQVERLNQNLHNYYEAQAKRFIAQTTVRDAYVGYYFELNE
HPEIDQ-HESSDKSFLIVSKNFFNQNNLPKDLNDQINGLLAQSNWAIQNPE----NSDERQANQLILQRRHI
PTTPAYNPQIHSPVTHPQRAKVVGPEGEEIYVDEWGRIKVRFLFTRSDDHSHDGGAGTNNNDTDSAWIDVLT
PWAGEGYGARFLPRIGEIVVIDFFNGDIDRPFVMGRIHEAQRQPTKFDNKGKLPDTKKLSGIRSKEVSGGGF
GQLRFDDTPGQISTQLQSSHGASQLNLGKLSHPKDKAESEDRGEGFELRTDQWGALRAGQGLLVSTHKQDNA
KGDHLDAEVAKKQLEGSQTNSKALSDIAKNQKTDEIESI-----------------------------------
---------------------------------------EQLKDFASQIQQ--------------------

-----------------------------------------------------------------QIAKF
EKALLLLSSPDGIALSSSEDIHISADAQINQIAGDSINISTQKNVIAHAQNRLSLFAAQSGLKAVAAQGKVE
IQAQSDALDVLANKGITISSTEDCIEISSPKEIVITGASSQITLNGSGIFPKTGGKFQVNAGQHVFQGGASA
SVQSSLPP------PPKRVQGVLELFHEYAHGEFVKGGSYRVV--DNFGKEVTGKLDDKGFAKVSGLAT-GA
VKVFFESDHRDPWDTASDFKRPVEWPNKNDADSEQSDSLIAQMSK----TAQSKLGELSKQLTNPTNIMKNI
QTAQSIKSEGAKALMPMLKTQAQGLVTDQVKSF---------LPISEAGQKIGNNIELTSIQKMNDFNKSGS
IDGNSLVNNTLHQYLQSPFKKNS
>NCGM237_6
-MLNSIHQVLDSLGISPQKRAIHVQFTSPILNDQVFLQRIDGVHALNDGLKAELLCLSTNATIQLKSFIGVQ
AAVDIVTERGELTRVTGIITHAQQGQSDGSLTLYKLTLEDPTALWKYRRNSRVFMNKSVVEIWEILFKEWQT
KNPLFAASLSLDLSGLT-QTYDVRPFVMQHNESDWNFLTRLLRSENISWLIDEAQHIVPSTDTSIQAQKLRL
IDANSQYQPLDRKTIRYHRSSAVEQYDSMTRLTAERSLQPNVMHIQRWQAEILDQEEGIGSVQSKHQHSEHY
DNATLGLEQAWNYSPAWIGDLKGEDGVTKSGNQQVERLNQNLHNYYEAQAKRFIAQTTVRDAYVGYYFELNE
HPEIDQ-HESSDKSFLIVSKNFFNQNNLPKDLNDQINGLLAQSNWAIQNPE----NSDERQANQLILQRRHI
PTTPAYNPQIHSPVTHPQRAKVVGPEGEEIYVDEWGRIKVRFLFTRSDDHSHDGGAGTNNNDTDSAWIDVLT
PWAGEGYGARFLPRIGEIVVIDFFNGDIDRPFVMGRIHEAQRQPTKFDNKGKLPDTKKLSGIRSKEVSGGGF
GQLRFDDTPGQISTQLQSSHGASQLNLGKLSHPKDKAESEDRGEGFELRTDQWGALRAGQGLLVSTHKQDNA
KGDHLDAEVAKKQLEGSQTNSKALSDIAKNQKTDEIESI-------------------------------
---------------------------------------EQLKDFASQIQQ-------------------
-----------------------------------------------------------------QIAKF
EKALLLLSSPDGIALSSSEDIHISADAQINQIAGDSINISTQKNVIAHAQNRLSLFAAQSGLKAVAAQGKVE
IQAQSDALDVLANKGITISSTEDCIEISSPKEIVITGASSQITLNGSGIFPKTGGKFQVNAGQHVFQGGASA
SVQSSLPP------PPKRVQGVLELFHEYAHGEFVKGGSYRVV--DNFGKEVTGKLDDKGFAKVSGLAT-GA
VKVFFESDHRDPWDTASDFKRPVEWPNKNDADSEQSDSLIAQMSK----TAQSKLGELSKQLTNPTNIMKNI
QTAQSIKSEGAKALMPMLKTQAQGLVTDQVKSF---------LPISEAGQKIGNNIELTSIQKMNDFNKSGS
IDGNSLVNNTLHQYLQSPFKKNS
>SAA14_6
-MLNSIHQVLDSLGISPQKRAIHVQFTSPILNDQVFLQRIDGVHALNDGLKAELLCLSTNATIQLKSFIGVQ
AAVDIVTERGELTRVTGIITHAQQGQSDGSLTLYKLTLEDPTALWKYRRNSRVFMNKSVVEIWEILFKEWQT
KNPLFAASLSLDLSGLT-QTYDVRPFVMQHNESDWNFLTRLLRSENISWLIDEAQHIVPSTDTSIQAQKLRL
IDANSQYQPLDRKTIRYHRSSAVEQYDSMTRLTAERSLQPNVMHIQRWQAEILDQEEGIGSVQSKHQHSEHY
DNATLGLEQAWNYSPAWIGDLKGEDGVTKSGNQQVERLNQNLHNYYEAQAKRFIAQTTVRDAYVGYYFELNE
HPEIDQ-HESSDKSFLIVSKNFFNQNNLPKDLNDQINGLLAQSNWAIQNPE----NSDERQANQLILQRRHI

PTTPAYNPQIHSPVTHPQRAKVVGPEGEEIYVDEWGRIKVRFLFTRSDDHSHDGGAGTNNNDTDSAWIDVLT
PWAGEGYGARFLPRIGEIVVIDFFNGDIDRPFVMGRIHEAQRQPTKFDNKGKLPDTKKLSGIRSKEVSGGGF
GQLRFDDTPGQISTQLQSSHGASQLNLGKLSHPKDKAESEDRGEGFELRTDQWGALRAGQGLLVSTHKQDNA
KGDHLDAEVAKKQLEGSQTNSKALSDIAKNQKTDEIESI---------------------------------
----------------------------------------EQLKDFASQIQQ--------------------
-------------------------------------------------------------------QIAKF
EKALLLLSSPDGIALSSSEDIHISADAQINQIAGDSINISTQKNVIAHAQNRLSLFAAQSGLKAVAAQGKVE
IQAQSDALDVLANKGITISSTEDCIEISSPKEIVITGASSQITLNGSGIFPKTGGKFQVNAGQHVFQGGASA
SVQSSLPP------PPKRVQGVLELFHEYAHGEFVKGGSYRVV--DNFGKEVTGKLDDKGFAKVSGLAT-GA
VKVFFESDHRDPWDTASDFKRPVEWPNKNDADSEQSDSLIAQMSK----TAQSKLGELSKQLTNPTNIMKNI
QTAQSIKSEGAKALMPMLKTQAQGLVTDQVKSF---------LPISEAGQKIGNNIELTSIQKMNDFNKSGS
IDGNSLVNNTLHQYLQSPFKKNS
>SMC_Paed_Ab_BL01_6
-MLNSIHQVLDSLGISPQKRAIHVQFTSPILNDQVFLQRIDGVHALNDGLKAELLCLSTNATIQLKSFIGVQ
AAVDIVTERGELTRVTGIITHAQQGQSDGSLTLYKLTLEDPTALWKYRRNSRVFMNKSVVEIWEILFKEWQT
KNPLFAASLSLDLSGLT-QTYDVRPFVMQHNESDWNFLTRLLRSENISWLIDEAQHIVPSTDTSIQAQKLRL
IDANSQYQPLDRKTIRYHRSSAVEQYDSMTRLTAERSLQPNVMHIQRWQAEILDQEEGIGSVQSKHQHSEHY
DNATLGLEQAWNYSPAWIGDLKGEDGVTKSGNQQVERLNQNLHNYYEAQAKRFIAQTTVRDAYVGYYFELNE
HPEIDQ-HESSDKSFLIVSKNFFNQNNLPKDLNDQINGLLAQSNWAIQNPE----NSDERQANQLILQRRHI
PTTPAYNPQIHSPVTHPQRAKVVGPEGEEIYVDEWGRIKVRFLFTRSDDHSHDGGAGTNNNDTDSAWIDVLT
PWAGEGYGARFLPRIGEIVVIDFFNGDIDRPFVMGRIHEAQRQPTKFDNKGKLPDTKKLSGIRSKEVSGGGF
GQLRFDDTPGQISTQLQSSHGASQLNLGKLSHPKDKAESEDRGEGFELRTDQWGALRAGQGLLVSTHKQDNA
KGDHLDAEVAKKQLEGSQTNSKALSDIAKNQKTDEIESI---------------------------------
----------------------------------------EQLKDFASQIQQ--------------------
-------------------------------------------------------------------QIAKF
EKALLLLSSPDGIALSSSEDIHISADAQINQIAGDSINISTQKNVIAHAQNRLSLFAAQSGLKAVAAQGKVE
IQAQSDALDVLANKGITISSTEDCIEISSPKEIVITGASSQITLNGSGIFPKTGGKFQVNAGQHVFQGGASA
SVQSSLPP------PPKRVQGVLELFHEYAHGEFVKGGSYRVV--DNFGKEVTGKLDDKGFAKVSGLAT-GA
VKVFFESDHRDPWDTASDFKRPVEWPNKNDADSEQSDSLIAQMSK----TAQSKLGELSKQLTNPTNIMKNI
QTAQSIKSEGAKALMPMLKTQAQGLVTDQVKSF---------LPISEAGQKIGNNIELTSIQKMNDFNKSGS
IDGNSLVNNTLHQYLQSPFKKNS
>SSA12_6
-MLNSIHQVLDSLGISPQKRAIHVQFTSPILNDQVFLQRIDGVHALNDGLKAELLCLSTNATIQLKSFIGVQ

AAVDIVTERGELTRVTGIITHAQQGQSDGSLTLYKLTLEDPTALWKYRRNSRVFMNKSVVEIWEILFKEWQT
KNPLFAASLSLDLSGLT-QTYDVRPFVMQHNESDWNFLTRLLRSENISWLIDEAQHIVPSTDTSIQAQKLRL
IDANSQYQPLDRKTIRYHRSSAVEQYDSMTRLTAERSLQPNVMHIQRWQAEILDQEEGIGSVQSKHQHSEHY
DNATLGLEQAWNYSPAWIGDLKGEDGVTKSGNQQVERLNQNLHNYYEAQAKRFIAQTTVRDAYVGYYFELNE
HPEIDQ-HESSDKSFLIVSKNFFNQNNLPKDLNDQINGLLAQSNWAIQNPE----NSDERQANQLILQRRHI
PTTPAYNPQIHSPVTHPQRAKVVGPEGEEIYVDEWGRIKVRFLFTRSDDHSHDGGAGTNNNDTDSAWIDVLT
PWAGEGYGARFLPRIGEIVVIDFFNGDIDRPFVMGRIHEAQRQPTKFDNKGKLPDTKKLSGIRSKEVSGGGF
GQLRFDDTPGQISTQLQSSHGASQLNLGKLSHPKDKAESEDRGEGFELRTDQWGALRAGQGLLVSTHKQDNA
KGDHLDAEVAKKQLEGSQTNSKALSDIAKNQKTDEIESI---------------------------------
----------------------------------------EQLKDFASQIQQ--------------------
-------------------------------------------------------------------QIAKF
EKALLLLSSPDGIALSSSEDIHISADAQINQIAGDSINISTQKNVIAHAQNRLSLFAAQSGLKAVAAQGKVE
IQAQSDALDVLANKGITISSTEDCIEISSPKEIVITGASSQITLNGSGIFPKTGGKFQVNAGQHVFQGGASA
SVQSSLPP------PPKRVQGVLELFHEYAHGEFVKGGSYRVV--DNFGKEVTGKLDDKGFAKVSGLAT-GA
VKVFFESDHRDPWDTASDFKRPVEWPNKNDADSEQSDSLIAQMSK----TAQSKLGELSKQLTNPTNIMKNI
QTAQSIKSEGAKALMPMLKTQAQGLVTDQVKSF---------LPISEAGQKIGNNIELTSIQKMNDFNKSGS
IDGNSLVNNTLHQYLQSPFKKNS
>SSA6_6
-MLNSIHQVLDSLGISPQKRAIHVQFTSPILNDQVFLQRIDGVHALNDGLKAELLCLSTNATIQLKSFIGVQ
AAVDIVTERGELTRVTGIITHAQQGQSDGSLTLYKLTLEDPTALWKYRRNSRVFMNKSVVEIWEILFKEWQT
KNPLFAASLSLDLSGLT-QTYDVRPFVMQHNESDWNFLTRLLRSENISWLIDEAQHIVPSTDTSIQAQKLRL
IDANSQYQPLDRKTIRYHRSSAVEQYDSMTRLTAERSLQPNVMHIQRWQAEILDQEEGIGSVQSKHQHSEHY
DNATLGLEQAWNYSPAWIGDLKGEDGVTKSGNQQVERLNQNLHNYYEAQAKRFIAQTTVRDAYVGYYFELNE
HPEIDQ-HESSDKSFLIVSKNFFNQNNLPKDLNDQINGLLAQSNWAIQNPE----NSDERQANQLILQRRHI
PTTPAYNPQIHSPVTHPQRAKVVGPEGEEIYVDEWGRIKVRFLFTRSDDHSHDGGAGTNNNDTDSAWIDVLT
PWAGEGYGARFLPRIGEIVVIDFFNGDIDRPFVMGRIHEAQRQPTKFDNKGKLPDTKKLSGIRSKEVSGGGF
GQLRFDDTPGQISTQLQSSHGASQLNLGKLSHPKDKAESEDRGEGFELRTDQWGALRAGQGLLVSTHKQDNA
KGDHLDAEVAKKQLEGSQTNSKALSDIAKNQKTDEIESI---------------------------------
----------------------------------------EQLKDFASQIQQ--------------------
-------------------------------------------------------------------QIAKF
EKALLLLSSPDGIALSSSEDIHISADAQINQIAGDSINISTQKNVIAHAQNRLSLFAAQSGLKAVAAQGKVE
IQAQSDALDVLANKGITISSTEDCIEISSPKEIVITGASSQITLNGSGIFPKTGGKFQVNAGQHVFQGGASA
SVQSSLPP------PPKRVQGVLELFHEYAHGEFVKGGSYRVV--DNFGKEVTGKLDDKGFAKVSGLAT-GA

VKVFFESDHRDPWDTASDFKRPVEWPNKNDADSEQSDSLIAQMSK----TAQSKLGELSKQLTNPTNIMKNI
QTAQSIKSEGAKALMPMLKTQAQGLVTDQVKSF---------LPISEAGQKIGNNIELTSIQKMNDFNKSGS
IDGNSLVNNTLHQYLQSPFKKNS
>TYTH-1_6
-MLNSIHQVLDSLGISPQKRAIHVQFTSPILNDQVFLQRIDGVHALNDGLKAELLCLSTNATIQLKSFIGVQ
AAVDIVTERGELTRVTGIITHAQQGQSDGSLTLYKLTLEDPTALWKYRRNSRVFMNKSVVEIWEILFKEWQT
KNPLFAASLSLDLSGLT-QTYDVRPFVMQHNESDWNFLTRLLRSENISWLIDEAQHIVPSTDTSIQAQKLRL
IDANSQYQPLDRKTIRYHRSSAVEQYDSMTRLTAERSLQPNVMHIQRWQAEILDQEEGIGSVQSKHQHSEHY
DNATLGLEQAWNYSPAWIGDLKGEDGVTKSGNQQVERLNQNLHNYYEAQAKRFIAQTTVRDAYVGYYFELNE
HPEIDQ-HESSDKSFLIVSKNFFNQNNLPKDLNDQINGLLAQSNWAIQNPE----NSDERQANQLILQRRHI
PTTPAYNPQIHSPVTHPQRAKVVGPEGEEIYVDEWGRIKVRFLFTRSDDHSHDGGAGTNNNDTDSAWIDVLT
PWAGEGYGARFLPRIGEIVVIDFFNGDIDRPFVMGRIHEAQRQPTKFDNKGKLPDTKKLSGIRSKEVSGGGF
GQLRFDDTPGQISTQLQSSHGASQLNLGKLSHPKDKAESEDRGEGFELRTDQWGALRAGQGLLVSTHKQDNA
KGDHLDAEVAKKQLEGSQTNSKALSDIAKNQKTDEIESI---------------------------------
----------------------------------------EQLKDFASQIQQ--------------------
-------------------------------------------------------------------QIAKF
EKALLLLSSPDGIALSSSEDIHISADAQINQIAGDSINISTQKNVIAHAQNRLSLFAAQSGLKAVAAQGKVE
IQAQSDALDVLANKGITISSTEDCIEISSPKEIVITGASSQITLNGSGIFPKTGGKFQVNAGQHVFQGGASA
SVQSSLPP------PPKRVQGVLELFHEYAHGEFVKGGSYRVV--DNFGKEVTGKLDDKGFAKVSGLAT-GA
VKVFFESDHRDPWDTASDFKRPVEWPNKNDADSEQSDSLIAQMSK----TAQSKLGELSKQLTNPTNIMKNI
QTAQSIKSEGAKALMPMLKTQAQGLVTDQVKSF---------LPISEAGQKIGNNIELTSIQKMNDFNKSGS
IDGNSLVNNTLHQYLQSPFKKNS
>USA2_6
-MLNSIHQVLDSLGISPQKRAIHVQFTSPILNDQVFLQRIDGVHALNDGLKAELLCLSTNATIQLKSFIGVQ
AAVDIVTERGELTRVTGIITHAQQGQSDGSLTLYKLTLEDPTALWKYRRNSRVFMNKSVVEIWEILFKEWQT
KNPLFAASLSLDLSGLT-QTYDVRPFVMQHNESDWNFLTRLLRSENISWLIDEAQHIVPSTDTSIQAQKLRL
IDANSQYQPLDRKTIRYHRSSAVEQYDSMTRLTAERSLQPNVMHIQRWQAEILDQEEGIGSVQSKHQHSEHY
DNATLGLEQAWNYSPAWIGDLKGEDGVTKSGNQQVERLNQNLHNYYEAQAKRFIAQTTVRDAYVGYYFELNE
HPEIDQ-HESSDKSFLIVSKNFFNQNNLPKDLNDQINGLLAQSNWAIQNPE----NSDERQANQLILQRRHI
PTTPAYNPQIHSPVTHPQRAKVVGPEGEEIYVDEWGRIKVRFLFTRSDDHSHDGGAGTNNNDTDSAWIDVLT
PWAGEGYGARFLPRIGEIVVIDFFNGDIDRPFVMGRIHEAQRQPTKFDNKGKLPDTKKLSGIRSKEVSGGGF
GQLRFDDTPGQISTQLQSSHGASQLNLGKLSHPKDKAESEDRGEGFELRTDQWGALRAGQGLLVSTHKQDNA
KGDHLDAEVAKKQLEGSQTNSKALSDIAKNQKTDEIESI---------------------------------

----------------------------------------EQLKDFASQIQQ--------------------
-------------------------------------------------------------------QIAKF
EKALLLLSSPDGIALSSSEDIHISADAQINQIAGDSINISTQKNVIAHAQNRLSLFAAQSGLKAVAAQGKVE
IQAQSDALDVLANKGITISSTEDCIEISSPKEIVITGASSQITLNGSGIFPKTGGKFQVNAGQHVFQGGASA
SVQSSLPP------PPKRVQGVLELFHEYAHGEFVKGGSYRVV--DNFGKEVTGKLDDKGFAKVSGLAT-GA
VKVFFESDHRDPWDTASDFKRPVEWPNKNDADSEQSDSLIAQMSK----TAQSKLGELSKQLTNPTNIMKNI
QTAQSIKSEGAKALMPMLKTQAQGLVTDQVKSF---------LPISEAGQKIGNNIELTSIQKMNDFNKSGS
IDGNSLVNNTLHQYLQSPFKKNS
>WCHAB005133_6
-MLNSIHQVLDSLGISPQKRAIHVQFTSPILNDQVFLQRIDGVHALNDGLKAELLCLSTNATIQLKSFIGVQ
AAVDIVTERGELTRVTGIITHAQQGQSDGSLTLYKLTLEDPTALWKYRRNSRVFMNKSVVEIWEILFKEWQT
KNPLFAASLSLDLSGLT-QTYDVRPFVMQHNESDWNFLTRLLRSENISWLIDEAQHIVPSTDTSIQAQKLRL
IDANSQYQPLDRKTIRYHRSSAVEQYDSMTRLTAERSLQPNVMHIQRWQAEILDQEEGIGSVQSKHQHSEHY
DNATLGLEQAWNYSPAWIGDLKGEDGVTKSGNQQVERLNQNLHNYYEAQAKRFIAQTTVRDAYVGYYFELNE
HPEIDQ-HESSDKSFLIVSKNFFNQNNLPKDLNDQINGLLAQSNWAIQNPE----NSDERQANQLILQRRHI
PTTPAYNPQIHSPVTHPQRAKVVGPEGEEIYVDEWGRIKVRFLFTRSDDHSHDGGAGTNNNDTDSAWIDVLT
PWAGEGYGARFLPRIGEIVVIDFFNGDIDRPFVMGRIHEAQRQPTKFDNKGKLPDTKKLSGIRSKEVSGGGF
GQLRFDDTPGQISTQLQSSHGASQLNLGKLSHPKDKAESEDRGEGFELRTDQWGALRAGQGLLVSTHKQDNA
KGDHLDAEVAKKQLEGSQTNSKALSDIAKNQKTDEIESI---------------------------------
----------------------------------------EQLKDFASQIQQ--------------------
-------------------------------------------------------------------QIAKF
EKALLLLSSPDGIALSSSEDIHISADAQINQIAGDSINISTQKNVIAHAQNRLSLFAAQSGLKAVAAQGKVE
IQAQSDALDVLANKGITISSTEDCIEISSPKEIVITGASSQITLNGSGIFPKTGGKFQVNAGQHVFQGGASA
SVQSSLPP------PPKRVQGVLELFHEYAHGEFVKGGSYRVV--DNFGKEVTGKLDDKGFAKVSGLAT-GA
VKVFFESDHRDPWDTASDFKRPVEWPNKNDADSEQSDSLIAQMSK----TAQSKLGELSKQLTNPTNIMKNI
QTAQSIKSEGAKALMPMLKTQAQGLVTDQVKSF---------LPISEAGQKIGNNIELTSIQKMNDFNKSGS
IDGNSLVNNTLHQYLQSPFKKNS
>XDR-BJ83_6
-MLNSIHQVLDSLGISPQKRAIHVQFTSPILNDQVFLQRIDGVHALNDGLKAELLCLSTNATIQLKSFIGVQ
AAVDIVTERGELTRVTGIITHAQQGQSDGSLTLYKLTLEDPTALWKYRRNSRVFMNKSVVEIWEILFKEWQT
KNPLFAASLSLDLSGLT-QTYDVRPFVMQHNESDWNFLTRLLRSENISWLIDEAQHIVPSTDTSIQAQKLRL
IDANSQYQPLDRKTIRYHRSSAVEQYDSMTRLTAERSLQPNVMHIQRWQAEILDQEEGIGSVQSKHQHSEHY
DNATLGLEQAWNYSPAWIGDLKGEDGVTKSGNQQVERLNQNLHNYYEAQAKRFIAQTTVRDAYVGYYFELNE

HPEIDQ-HESSDKSFLIVSKNFFNQNNLPKDLNDQINGLLAQSNWAIQNPE----NSDERQANQLILQRRHI
PTTPAYNPQIHSPVTHPQRAKVVGPEGEEIYVDEWGRIKVRFLFTRSDDHSHDGGAGTNNNDTDSAWIDVLT
PWAGEGYGARFLPRIGEIVVIDFFNGDIDRPFVMGRIHEAQRQPTKFDNKGKLPDTKKLSGIRSKEVSGGGF
GQLRFDDTPGQISTQLQSSHGASQLNLGKLSHPKDKAESEDRGEGFELRTDQWGALRAGQGLLVSTHKQDNA
KGDHLDAEVAKKQLEGSQTNSKALSDIAKNQKTDEIESI-----------------------------------
----------------------------------------EQLKDFASQIQQ--------------------
---------------------------------------------------------------QIAKF
EKALLLLSSPDGIALSSSEDIHISADAQINQIAGDSINISTQKNVIAHAQNRLSLFAAQSGLKAVAAQGKVE
IQAQSDALDVLANKGITISSTEDCIEISSPKEIVITGASSQITLNGSGIFPKTGGKFQVNAGQHVFQGGASA
SVQSSLPP------PPKRVQGVLELFHEYAHGEFVKGGSYRVV--DNFGKEVTGKLDDKGFAKVSGLAT-GA
VKVFFESDHRDPWDTASDFKRPVEWPNKNDADSEQSDSLIAQMSK----TAQSKLGELSKQLTNPTNIMKNI
QTAQSIKSEGAKALMPMLKTQAQGLVTDQVKSF---------LPISEAGQKIGNNIELTSIQKMNDFNKSGS
IDGNSLVNNTLHQYLQSPFKKNS
>XH386_6
-MLNSIHQVLDSLGISPQKRAIHVQFTSPILNDQVFLQRIDGVHALNDGLKAELLCLSTNATIQLKSFIGVQ
AAVDIVTERGELTRVTGIITHAQQGQSDGSLTLYKLTLEDPTALWKYRRNSRVFMNKSVVEIWEILFKEWQT
KNPLFAASLSLDLSGLT-QTYDVRPFVMQHNESDWNFLTRLLRSENISWLIDEAQHIVPSTDTSIQAQKLRL
IDANSQYQPLDRKTIRYHRSSAVEQYDSMTRLTAERSLQPNVMHIQRWQAEILDQEEGIGSVQSKHQHSEHY
DNATLGLEQAWNYSPAWIGDLKGEDGVTKSGNQQVERLNQNLHNYYEAQAKRFIAQTTVRDAYVGYYFELNE
HPEIDQ-HESSDKSFLIVSKNFFNQNNLPKDLNDQINGLLAQSNWAIQNPE----NSDERQANQLILQRRHI
PTTPAYNPQIHSPVTHPQRAKVVGPEGEEIYVDEWGRIKVRFLFTRSDDHSHDGGAGTNNNDTDSAWIDVLT
PWAGEGYGARFLPRIGEIVVIDFFNGDIDRPFVMGRIHEAQRQPTKFDNKGKLPDTKKLSGIRSKEVSGGGF
GQLRFDDTPGQISTQLQSSHGASQLNLGKLSHPKDKAESEDRGEGFELRTDQWGALRAGQGLLVSTHKQDNA
KGDHLDAEVAKKQLEGSQTNSKALSDIAKNQKTDEIESI-----------------------------------
----------------------------------------EQLKDFASQIQQ--------------------
---------------------------------------------------------------QIAKF
EKALLLLSSPDGIALSSSEDIHISADAQINQIAGDSINISTQKNVIAHAQNRLSLFAAQSGLKAVAAQGKVE
IQAQSDALDVLANKGITISSTEDCIEISSPKEIVITGASSQITLNGSGIFPKTGGKFQVNAGQHVFQGGASA
SVQSSLPP------PPKRVQGVLELFHEYAHGEFVKGGSYRVV--DNFGKEVTGKLDDKGFAKVSGLAT-GA
VKVFFESDHRDPWDTASDFKRPVEWPNKNDADSEQSDSLIAQMSK----TAQSKLGELSKQLTNPTNIMKNI
QTAQSIKSEGAKALMPMLKTQAQGLVTDQVKSF---------LPISEAGQKIGNNIELTSIQKMNDFNKSGS
IDGNSLVNNTLHQYLQSPFKKNS
>XH856_6

-MLNSIHQVLDSLGISPQKRAIHVQFTSPILNDQVFLQRIDGVHALNDGLKAELLCLSTNATIQLKSFIGVQ
AAVDIVTERGELTRVTGIITHAQQGQSDGSLTLYKLTLEDPTALWKYRRNSRVFMNKSVVEIWEILFKEWQT
KNPLFAASLSLDLSGLT-QTYDVRPFVMQHNESDWNFLTRLLRSENISWLIDEAQHIVPSTDTSIQAQKLRL
IDANSQYQPLDRKTIRYHRSSAVEQYDSMTRLTAERSLQPNVMHIQRWQAEILDQEEGIGSVQSKHQHSEHY
DNATLGLEQAWNYSPAWIGDLKGEDGVTKSGNQQVERLNQNLHNYYEAQAKRFIAQTTVRDAYVGYYFELNE
HPEIDQ-HESSDKSFLIVSKNFFNQNNLPKDLNDQINGLLAQSNWAIQNPE----NSDERQANQLILQRRHI
PTTPAYNPQIHSPVTHPQRAKVVGPEGEEIYVDEWGRIKVRFLFTRSDDHSHDGGAGTNNNDTDSAWIDVLT
PWAGEGYGARFLPRIGEIVVIDFFNGDIDRPFVMGRIHEAQRQPTKFDNKGKLPDTKKLSGIRSKEVSGGGF
GQLRFDDTPGQISTQLQSSHGASQLNLGKLSHPKDKAESEDRGEGFELRTDQWGALRAGQGLLVSTHKQDNA
KGDHLDAEVAKKQLEGSQTNSKALSDIAKNQKTDEIESI---------------------------------
----------------------------------------EQLKDFASQIQQ--------------------
-------------------------------------------------------------------QIAKF
EKALLLLSSPDGIALSSSEDIHISADAQINQIAGDSINISTQKNVIAHAQNRLSLFAAQSGLKAVAAQGKVE
IQAQSDALDVLANKGITISSTEDCIEISSPKEIVITGASSQITLNGSGIFPKTGGKFQVNAGQHVFQGGASA
SVQSSLPP------PPKRVQGVLELFHEYAHGEFVKGGSYRVV--DNFGKEVTGKLDDKGFAKVSGLAT-GA
VKVFFESDHRDPWDTASDFKRPVEWPNKNDADSEQSDSLIAQMSK----TAQSKLGELSKQLTNPTNIMKNI
QTAQSIKSEGAKALMPMLKTQAQGLVTDQVKSF---------LPISEAGQKIGNNIELTSIQKMNDFNKSGS
IDGNSLVNNTLHQYLQSPFKKNS
>XH857_6
-MLNSIHQVLDSLGISPQKRAIHVQFTSPILNDQVFLQRIDGVHALNDGLKAELLCLSTNATIQLKSFIGVQ
AAVDIVTERGELTRVTGIITHAQQGQSDGSLTLYKLTLEDPTALWKYRRNSRVFMNKSVVEIWEILFKEWQT
KNPLFAASLSLDLSGLT-QTYDVRPFVMQHNESDWNFLTRLLRSENISWLIDEAQHIVPSTDTSIQAQKLRL
IDANSQYQPLDRKTIRYHRSSAVEQYDSMTRLTAERSLQPNVMHIQRWQAEILDQEEGIGSVQSKHQHSEHY
DNATLGLEQAWNYSPAWIGDLKGEDGVTKSGNQQVERLNQNLHNYYEAQAKRFIAQTTVRDAYVGYYFELNE
HPEIDQ-HESSDKSFLIVSKNFFNQNNLPKDLNDQINGLLAQSNWAIQNPE----NSDERQANQLILQRRHI
PTTPAYNPQIHSPVTHPQRAKVVGPEGEEIYVDEWGRIKVRFLFTRSDDHSHDGGAGTNNNDTDSAWIDVLT
PWAGEGYGARFLPRIGEIVVIDFFNGDIDRPFVMGRIHEAQRQPTKFDNKGKLPDTKKLSGIRSKEVSGGGF
GQLRFDDTPGQISTQLQSSHGASQLNLGKLSHPKDKAESEDRGEGFELRTDQWGALRAGQGLLVSTHKQDNA
KGDHLDAEVAKKQLEGSQTNSKALSDIAKNQKTDEIESI---------------------------------
----------------------------------------EQLKDFASQIQQ--------------------
-------------------------------------------------------------------QIAKF
EKALLLLSSPDGIALSSSEDIHISADAQINQIAGDSINISTQKNVIAHAQNRLSLFAAQSGLKAVAAQGKVE
IQAQSDALDVLANKGITISSTEDCIEISSPKEIVITGASSQITLNGSGIFPKTGGKFQVNAGQHVFQGGASA

SVQSSLPP------PPKRVQGVLELFHEYAHGEFVKGGSYRVV--DNFGKEVTGKLDDKGFAKVSGLAT-GA
VKVFFESDHRDPWDTASDFKRPVEWPNKNDADSEQSDSLIAQMSK----TAQSKLGELSKQLTNPTNIMKNI
QTAQSIKSEGAKALMPMLKTQAQGLVTDQVKSF---------LPISEAGQKIGNNIELTSIQKMNDFNKSGS
IDGNSLVNNTLHQYLQSPFKKNS
>XH859_6
-MLNSIHQVLDSLGISPQKRAIHVQFTSPILNDQVFLQRIDGVHALNDGLKAELLCLSTNATIQLKSFIGVQ
AAVDIVTERGELTRVTGIITHAQQGQSDGSLTLYKLTLEDPTALWKYRRNSRVFMNKSVVEIWEILFKEWQT
KNPLFAASLSLDLSGLT-QTYDVRPFVMQHNESDWNFLTRLLRSENISWLIDEAQHIVPSTDTSIQAQKLRL
IDANSQYQPLDRKTIRYHRSSAVEQYDSMTRLTAERSLQPNVMHIQRWQAEILDQEEGIGSVQSKHQHSEHY
DNATLGLEQAWNYSPAWIGDLKGEDGVTKSGNQQVERLNQNLHNYYEAQAKRFIAQTTVRDAYVGYYFELNE
HPEIDQ-HESSDKSFLIVSKNFFNQNNLPKDLNDQINGLLAQSNWAIQNPE----NSDERQANQLILQRRHI
PTTPAYNPQIHSPVTHPQRAKVVGPEGEEIYVDEWGRIKVRFLFTRSDDHSHDGGAGTNNNDTDSAWIDVLT
PWAGEGYGARFLPRIGEIVVIDFFNGDIDRPFVMGRIHEAQRQPTKFDNKGKLPDTKKLSGIRSKEVSGGGF
GQLRFDDTPGQISTQLQSSHGASQLNLGKLSHPKDKAESEDRGEGFELRTDQWGALRAGQGLLVSTHKQDNA
KGDHLDAEVAKKQLEGSQTNSKALSDIAKNQKTDEIESI-------------------------------
---------------------------------------EQLKDFASQIQQ-------------------
-----------------------------------------------------------------QIAKF
EKALLLLSSPDGIALSSSEDIHISADAQINQIAGDSINISTQKNVIAHAQNRLSLFAAQSGLKAVAAQGKVE
IQAQSDALDVLANKGITISSTEDCIEISSPKEIVITGASSQITLNGSGIFPKTGGKFQVNAGQHVFQGGASA
SVQSSLPP------PPKRVQGVLELFHEYAHGEFVKGGSYRVV--DNFGKEVTGKLDDKGFAKVSGLAT-GA
VKVFFESDHRDPWDTASDFKRPVEWPNKNDADSEQSDSLIAQMSK----TAQSKLGELSKQLTNPTNIMKNI
QTAQSIKSEGAKALMPMLKTQAQGLVTDQVKSF---------LPISEAGQKIGNNIELTSIQKMNDFNKSGS
IDGNSLVNNTLHQYLQSPFKKNS
>XH860_6
-MLNSIHQVLDSLGISPQKRAIHVQFTSPILNDQVFLQRIDGVHALNDGLKAELLCLSTNATIQLKSFIGVQ
AAVDIVTERGELTRVTGIITHAQQGQSDGSLTLYKLTLEDPTALWKYRRNSRVFMNKSVVEIWEILFKEWQT
KNPLFAASLSLDLSGLT-QTYDVRPFVMQHNESDWNFLTRLLRSENISWLIDEAQHIVPSTDTSIQAQKLRL
IDANSQYQPLDRKTIRYHRSSAVEQYDSMTRLTAERSLQPNVMHIQRWQAEILDQEEGIGSVQSKHQHSEHY
DNATLGLEQAWNYSPAWIGDLKGEDGVTKSGNQQVERLNQNLHNYYEAQAKRFIAQTTVRDAYVGYYFELNE
HPEIDQ-HESSDKSFLIVSKNFFNQNNLPKDLNDQINGLLAQSNWAIQNPE----NSDERQANQLILQRRHI
PTTPAYNPQIHSPVTHPQRAKVVGPEGEEIYVDEWGRIKVRFLFTRSDDHSHDGGAGTNNNDTDSAWIDVLT
PWAGEGYGARFLPRIGEIVVIDFFNGDIDRPFVMGRIHEAQRQPTKFDNKGKLPDTKKLSGIRSKEVSGGGF
GQLRFDDTPGQISTQLQSSHGASQLNLGKLSHPKDKAESEDRGEGFELRTDQWGALRAGQGLLVSTHKQDNA

KGDHLDAEVAKKQLEGSQTNSKALSDIAKNQKTDEIESI---------------------------------
----------------------------------------EQLKDFASQIQQ--------------------
-------------------------------------------------------------------QIAKF
EKALLLLSSPDGIALSSSEDIHISADAQINQIAGDSINISTQKNVIAHAQNRLSLFAAQSGLKAVAAQGKVE
IQAQSDALDVLANKGITISSTEDCIEISSPKEIVITGASSQITLNGSGIFPKTGGKFQVNAGQHVFQGGASA
SVQSSLPP------PPKRVQGVLELFHEYAHGEFVKGGSYRVV--DNFGKEVTGKLDDKGFAKVSGLAT-GA
VKVFFESDHRDPWDTASDFKRPVEWPNKNDADSEQSDSLIAQMSK----TAQSKLGELSKQLTNPTNIMKNI
QTAQSIKSEGAKALMPMLKTQAQGLVTDQVKSF---------LPISEAGQKIGNNIELTSIQKMNDFNKSGS
IDGNSLVNNTLHQYLQSPFKKNS
>YU-R612_6
-MLNSIHQVLDSLGISPQKRAIHVQFTSPILNDQVFLQRIDGVHALNDGLKAELLCLSTNATIQLKSFIGVQ
AAVDIVTERGELTRVTGIITHAQQGQSDGSLTLYKLTLEDPTALWKYRRNSRVFMNKSVVEIWEILFKEWQT
KNPLFAASLSLDLSGLT-QTYDVRPFVMQHNESDWNFLTRLLRSENISWLIDEAQHIVPSTDTSIQAQKLRL
IDANSQYQPLDRKTIRYHRSSAVEQYDSMTRLTAERSLQPNVMHIQRWQAEILDQEEGIGSVQSKHQHSEHY
DNATLGLEQAWNYSPAWIGDLKGEDGVTKSGNQQVERLNQNLHNYYEAQAKRFIAQTTVRDAYVGYYFELNE
HPEIDQ-HESSDKSFLIVSKNFFNQNNLPKDLNDQINGLLAQSNWAIQNPE----NSDERQANQLILQRRHI
PTTPAYNPQIHSPVTHPQRAKVVGPEGEEIYVDEWGRIKVRFLFTRSDDHSHDGGAGTNNNDTDSAWIDVLT
PWAGEGYGARFLPRIGEIVVIDFFNGDIDRPFVMGRIHEAQRQPTKFDNKGKLPDTKKLSGIRSKEVSGGGF
GQLRFDDTPGQISTQLQSSHGASQLNLGKLSHPKDKAESEDRGEGFELRTDQWGALRAGQGLLVSTHKQDNA
KGDHLDAEVAKKQLEGSQTNSKALSDIAKNQKTDEIESI---------------------------------
----------------------------------------EQLKDFASQIQQ--------------------
-------------------------------------------------------------------QIAKF
EKALLLLSSPDGIALSSSEDIHISADAQINQIAGDSINISTQKNVIAHAQNRLSLFAAQSGLKAVAAQGKVE
IQAQSDALDVLANKGITISSTEDCIEISSPKEIVITGASSQITLNGSGIFPKTGGKFQVNAGQHVFQGGASA
SVQSSLPP------PPKRVQGVLELFHEYAHGEFVKGGSYRVV--DNFGKEVTGKLDDKGFAKVSGLAT-GA
VKVFFESDHRDPWDTASDFKRPVEWPNKNDADSEQSDSLIAQMSK----TAQSKLGELSKQLTNPTNIMKNI
QTAQSIKSEGAKALMPMLKTQAQGLVTDQVKSF---------LPISEAGQKIGNNIELTSIQKMNDFNKSGS
IDGNSLVNNTLHQYLQSPFKKNS
>AF-673_6
-MLNSIHQVLDSLGISPQKRAIHVQFTSPILNDQVFLQRIDGVHALNDGLKAELLCLSTNATIQLKSFIGVQ
AAVDIVTERGELTRVTGIITHAQQGQSDGSLTLYKLTLEDPTALWKYRRNSRVFMNKSVVEIWEILFKEWQT
KNPLFAASLSLDLSGLT-QTYDVRPFVMQHNESDWNFLTRLLRSENISWLIDEAQHIVPSTDTSIQAQKLRL
IDANSQYQPLDRKTIRYHRSSAVEQYDSMTRLTAERSLQPNVMHIQRWQAEILDQEEGIGSVQSKHQHSEHY

DNATLGLEQAWNYSPAWIGDLKGEDGVTKSGNQQVERLNQNLHNYYEAQAKRFIAQTTVRDAYVGYYFELNE
HPEIDQ-HESSDKSFLIVSKNFFNQNNLPKDLNDQINGLLAQSNWAIQNPE----NSDERQANQLILQRRHI
PTTPAYNPQIHSPVTHPQRAKVVGPEGEEIYVDEWGRIKVRFLFTRSDDHSHDGGAGTNNNDTDSAWIDVLT
PWAGEGYGARFLPRIGEIVVIDFFNGDIDRPFVMGRIHEAQRQPTKFDNKGKLPDTKKLSGIRSKEVSGGGF
GQLRFDDTPGQISTQLQSSHGASQLNLGKLSHPKDKAESEDRGEGFELRTDQWGALRAGQGLLVSTHKQDNA
KGDHLDAEVAKKQLEGSQTNSKALSDIAKNQKTDEIESI-----------------------------------
---------------------------------------EQLKDFASQIQQ--------------------
-----------------------------------------------------------------QIAKF
EKALLLLSSPDGIALSSSEDIHISADAQINQIAGDSINISTQKNVIAHAQNRLSLFAAQSGLKAVAAQGKVE
IQAQSDALDVLANKGITISSTEDCIEISSPKEIVITGASSQITLNGSGIFPKTGGKFQVNAGQHVFQGGASA
SVQSSLPP------PPKR---VLELFHEYAHGEFVKGGSYRVV--DNFGKEVTGKLDDKGFAKVSGLAT-GA
VKVFFESDHRDPWDTASDFKRPVEWPNKNDADSEQSDSLIAQMSK----TAQSKLGELSKQLTNPTNIMKNI
QTAQSIKSEGAKALMPMLKTQAQGLVTDQVKSF---------LPISEAGQKIGNNIELTSIQKMNDFNKSGS
IDGNSLVNNTLHQYLQSPFKKNS
>CMC-CR-MDR-Ab4_6
-MLNSIHQVLDSLGISPQKRAIHVQFTSPILNDQVFLQRIDGVHALNDGLKAELLCLSTNATIQLKSFIGVQ
AAVDIVTERGELTRVTGIITHAQQGQSDGSLTLYKLTLEDPTALWKYRRNSRVFMNKSVVEIWEILFKEWQT
KNPLFAASLSLDLSGLT-QTYDVRPFVMQHNESDWNFLTRLLRSENISWLIDEAQHIVPSTDTSIQAQKLRL
IDANSQYQPLDRKTIRYHRSSAVEQYDSMTRLTAERSLQPNVMHIQRWQAEILDQEEGIGSVQSKHQHSEHY
DNATLGLEQAWNYSPAWIGDLKGEDGVTKSGNQQVERLNQNLHNYYEAQAKRFIAQTTVRDAYVGYYFELNE
HPEIDQ-HESSDKSFLIVSKNFFNQNNLPKDLNDQINGLLAQSNWAIQNPE----NSDERQANQLILQRRHI
PTTPAYNPQIHSPVTHPQRAKVVGPEGEEIYVDEWGRIKVRFLFTRSDDHSHDGGAGTNNNDTDSAWIDVLT
PWAGEGYGARFLPRIGEIVVIDFFNGDIDRPFVMGRIHEAQRQPTKFDNKGKLPDTKKLSGIRSKEVSGGGF
GQLRFDDTPGQISTQLQSSHGASQLNLGKLSHPKDKAESEDRGEGFELRTDQWGALRAGQGLLVSTHKQDNA
KGDHLDAEVAKKQLEGSQTNSKALSDIAKNQKTDEIESI-----------------------------------
---------------------------------------EQLKDFASQIQQ--------------------
-----------------------------------------------------------------QIAKF
EKALLLLSSPDGIALSSSEDIHISADAQINQIAGDSINISTQKNVIAHAQNRLSLFAAQSGLKAVAAQGKVE
IQAQSDALDVLANKGITISSTEDCIEISSPKEIVITGASSQITLNGSGIFPKTGGKFQVNAGQHVFQGGASA
SVQSSLPP------PPKR---VLELFHEYAHGEFVKGGSYRVV--DNFGKEVTGKLDDKGFAKVSGLAT-GA
VKVFFESDHRDPWDTASDFKRPVEWPNKNDADSEQSDSLIAQMSK----TAQSKLGELSKQLTNPTNIMKNI
QTAQSIKSEGAKALMPMLKTQAQGLVTDQVKSF---------LPISEAGQKIGNNIELTSIQKMNDFNKSGS
IDGNSLVNNTLHQYLQSPFKKNS

>CMC-CR-MDR-Ab66_6
-MLNSIHQVLDSLGISPQKRAIHVQFTSPILNDQVFLQRIDGVHALNDGLKAELLCLSTNATIQLKSFIGVQ
AAVDIVTERGELTRVTGIITHAQQGQSDGSLTLYKLTLEDPTALWKYRRNSRVFMNKSVVEIWEILFKEWQT
KNPLFAASLSLDLSGLT-QTYDVRPFVMQHNESDWNFLTRLLRSENISWLIDEAQHIVPSTDTSIQAQKLRL
IDANSQYQPLDRKTIRYHRSSAVEQYDSMTRLTAERSLQPNVMHIQRWQAEILDQEEGIGSVQSKHQHSEHY
DNATLGLEQAWNYSPAWIGDLKGEDGVTKSGNQQVERLNQNLHNYYEAQAKRFIAQTTVRDAYVGYYFELNE
HPEIDQ-HESSDKSFLIVSKNFFNQNNLPKDLNDQINGLLAQSNWAIQNPE----NSDERQANQLILQRRHI
PTTPAYNPQIHSPVTHPQRAKVVGPEGEEIYVDEWGRIKVRFLFTRSDDHSHDGGAGTNNNDTDSAWIDVLT
PWAGEGYGARFLPRIGEIVVIDFFNGDIDRPFVMGRIHEAQRQPTKFDNKGKLPDTKKLSGIRSKEVSGGGF
GQLRFDDTPGQISTQLQSSHGASQLNLGKLSHPKDKAESEDRGEGFELRTDQWGALRAGQGLLVSTHKQDNA
KGDHLDAEVAKKQLEGSQTNSKALSDIAKNQKTDEIESI----------------------------------
----------------------------------------EQLKDFASQIQQ--------------------
-----------------------------------------------------------------QIAKF
EKALLLLSSPDGIALSSSEDIHISADAQINQIAGDSINISTQKNVIAHAQNRLSLFAAQSGLKAVAAQGKVE
IQAQSDALDVLANKGITISSTEDCIEISSPKEIVITGASSQITLNGSGIFPKTGGKFQVNAGQHVFQGGASA
SVQSSLPP------PPKR---VLELFHEYAHGEFVKGGSYRVV--DNFGKEVTGKLDDKGFAKVSGLAT-GA
VKVFFESDHRDPWDTASDFKRPVEWPNKNDADSEQSDSLIAQMSK----TAQSKLGELSKQLTNPTNIMKNI
QTAQSIKSEGAKALMPMLKTQAQGLVTDQVKSF---------LPISEAGQKIGNNIELTSIQKMNDFNKSGS
IDGNSLVNNTLHQYLQSPFKKNS
>CMC-MDR-Ab59_6
-MLNSIHQVLDSLGISPQKRAIHVQFTSPILNDQVFLQRIDGVHALNDGLKAELLCLSTNATIQLKSFIGVQ
AAVDIVTERGELTRVTGIITHAQQGQSDGSLTLYKLTLEDPTALWKYRRNSRVFMNKSVVEIWEILFKEWQT
KNPLFAASLSLDLSGLT-QTYDVRPFVMQHNESDWNFLTRLLRSENISWLIDEAQHIVPSTDTSIQAQKLRL
IDANSQYQPLDRKTIRYHRSSAVEQYDSMTRLTAERSLQPNVMHIQRWQAEILDQEEGIGSVQSKHQHSEHY
DNATLGLEQAWNYSPAWIGDLKGEDGVTKSGNQQVERLNQNLHNYYEAQAKRFIAQTTVRDAYVGYYFELNE
HPEIDQ-HESSDKSFLIVSKNFFNQNNLPKDLNDQINGLLAQSNWAIQNPE----NSDERQANQLILQRRHI
PTTPAYNPQIHSPVTHPQRAKVVGPEGEEIYVDEWGRIKVRFLFTRSDDHSHDGGAGTNNNDTDSAWIDVLT
PWAGEGYGARFLPRIGEIVVIDFFNGDIDRPFVMGRIHEAQRQPTKFDNKGKLPDTKKLSGIRSKEVSGGGF
GQLRFDDTPGQISTQLQSSHGASQLNLGKLSHPKDKAESEDRGEGFELRTDQWGALRAGQGLLVSTHKQDNA
KGDHLDAEVAKKQLEGSQTNSKALSDIAKNQKTDEIESI----------------------------------
----------------------------------------EQLKDFASQIQQ--------------------
-----------------------------------------------------------------QIAKF
EKALLLLSSPDGIALSSSEDIHISADAQINQIAGDSINISTQKNVIAHAQNRLSLFAAQSGLKAVAAQGKVE

IQAQSDALDVLANKGITISSTEDCIEISSPKEIVITGASSQITLNGSGIFPKTGGKFQVNAGQHVFQGGASA
SVQSSLPP------PPKR---VLELFHEYAHGEFVKGGSYRVV--DNFGKEVTGKLDDKGFAKVSGLAT-GA
VKVFFESDHRDPWDTASDFKRPVEWPNKNDADSEQSDSLIAQMSK----TAQSKLGELSKQLTNPTNIMKNI
QTAQSIKSEGAKALMPMLKTQAQGLVTDQVKSF---------LPISEAGQKIGNNIELTSIQKMNDFNKSGS
IDGNSLVNNTLHQYLQSPFKKNS
>IOMTU433_22
-MLNSIHQVLDSLGISPQKRAIHVQFTSPILNDQVFLQRIDGVHALNDGLKAELLCLSTNATIQLKSFIGVQ
AAVDIVTERGELTRVTGIITHAQQGQSDGSLTLYKLTLEDPTALWKYRRNSRVFMNKSVFEIWEILFKEWQT
KNPLFAASLSLDLSGLT-QTYDVRPFVMQHNESDWNFLTRLLRSENISWLIDEAQHIVPSTDTPIQAQKLRL
IDANSQYQPLDRKTIRYHRSSAVEQYDSMTRLTAERSLQPNVMHIQRWQAEILDQEEGIGSVQSKHQHSEQY
DNATLGLEQAWNYAPAWIGDLKGEDGVTKSGNQQVERLNQNLNNYYEAQAKRFIAQTTVRDAYVGYYFELNE
HPEIDQ-HESTDRSFLIISKSFFNQNNLPKDLNDQINGLLAQSNWSIQNPE----NSDERQANQLILQRRHI
PTTPAYNPQIHSPVTHPQRAKVVGPEGEEIYVDEWGRIKVRFLFTRSDDHSHDGGAGTNNNDTDSAWVDVLT
PWAGEGYGARFLPRIGEIVVIDFFNGDIDRPFVMGRIHEAQRHPTKFDNKGKLPDTKKLSGIRSKEVSGSGF
GQLRFDDTPGQISTQLQSSHGASQLNLGKLSHPKDKAESEDRGEGFELRTDQWGALRAGQGLLVSTHKQDNA
KGEHLDAEVAKKQLEGSQTNSKALSDIAKNQKTDEIESL-------------------------------
---------------------------------------EQLKDFASQIQQ-------------------
-----------------------------------------------------------------QIAKF
EKALLLLSSPDGIALSSSEDIHISADAQINQIAGDSINISTQKNVIAHAQNRLSLFAAQSGLKAVAAQGKVE
IQAQSDALDVLANKGITISSTEDCIEISSPKEIVITGASSQITLNGSGIFPKTGGKFQVNAGQHVFQGGASA
SVKSSLPP------PPKRGQGVLELFHEYAHGEFVKQGGYTVT--DSLGKQFKGQLDDKGFVRVSGLAI-GS
AKIVFEEDKRNPWDEASDFKRPPMWPSDNDTSAQTLMGKADSVLKGAVTEAKKALG---QTFTNPSSIMRTI
DTAKQLKENGASALLPMVQQGVESHLTSQLTNFTSKVLSQSSIGQGWMHRKTNPVAPVTSGLNVNPLRAQDS
KNTETALNPMSFKFK--------
>3207_22
-MLNSIHQVLDSLGISPQKRAIHVQFTSPILNDQVFLQRIDGVHALNDGLKAELLCLSTNATIQLKSFIGVQ
AAVDIVTERGELTRVTGIITHAQQGQSDGSLTLYKLTLEDPTALWKYRRNSRVFMNKSVVEIWEILFKEWQT
KNPLFAASLSLDLSGLT-QTYEVRPFVMQHNESDWNFLTRLLRSENISWLIDEAQHIVPSTDTPIQAQKLRL
IDANSQYQPLDRKTIRYHRSSAVEQYDSMTRLTAERSLQPNVMHIQRWQAEILDQEEGIGSVQSKHQHSEHY
DNATLGLEQAWNYAPAWIGDLKGEDGVTKSGNQQVERLNQNLHNYYEAQAKRFIAQTTVRDAYVGYYFELNE
HPEIDQ-HESSDKSFLIVSKNFFNQNNLPKDLNDQINGLLAQSNWAIQNPE----NSDERQANQLILQRRHI
PTTPAYNPQIHSPVTHPQRAKVVGPEGEEIYVDEWGRIKVRFLFTRSDDHSHDGGAGTNNNDTDSAWIDVLT
PWAGEGYGARFLPRIGEIVVIDFFNGDIDRPFVMGRIHEAQRHPTKFDNKGKLPDTKKLSGIRSKEVSGGGF

GQLRFDDTPGQISTQLQSSHGASQLNLGKLSHPKDKAESEDRGEGFELRTDQWGALRAGQGLLVSTHKQDNA
KGDHLDAEVAKKQLEGSQTNSKALSDIAKNQKTDEIESI------------------------------------
---------------------------------------EQLKDFASQIQQ--------------------
-----------------------------------------------------------------QIAKF
EKALLLLSSPDGIALSSSEDIHISADAQINQIAGDSINISTQKNVIAHAQNRLSLFAAQSGLKAVAAQGKVE
IQAQSDALDVLANKGITISSTEDCIEISSPKEIVITGASSQITLNGSGIFPKTGGKFQVNAGQHVFQGGASA
SVQSSLPP------PPKRGQGVLELFHEYAHGEFVKQGGYTVT--DSLGKQFKGQLDDKGFVRVSGLAT-GS
AKIVFEEDKRNPWDEASDFKRPPMWPSDNDTSAQTLMGKADSVLKGAVTEAKKALG---QTFTNPSSIMRTI
DTAKQLKENGASALLPMVQQGVESHLTSQLTNFTSKVLSQSSTGQGWMHGKTNPVAPVTSGLNVNPLRAQDS
KNTETALNPMSFKFK--------
>AB030_22
-MLNSIHQVLDSLGISPQKRAIHVQFTSPILNDQVFLQRIDGVHALNDGLKAELLCLSTNATIQLKSFIGVQ
AAVDIVTERGELTRVTGIITHAQQGQSDGSLTLYKLTLEDPTALWKYRRNSRVFMNKSVVEIWEILFKEWQT
KNPLFAASLSLDLSGLT-QTYEVRPFVMQHNESDWNFLTRLLRSENISWLIDEAQHIVPSTDTPIQAQKLRL
IDANSQYQPLDRKTIRYHRSSAVEQYDSMTRLTAERSLQPNVMHIQRWQAEILDQEEGIGSVQSKHQHSEHY
DNATLGLEQAWNYAPAWIGDLKGEDGVTKSGNQQVERLNQNLHNYYEAQAKRFIAQTTVRDAYVGYYFELNE
HPEIDQ-HESSDKSFLIVSKNFFNQNNLPKDLNDQINGLLAQSNWAIQNPE----NSDERQANQLILQRRHI
PTTPAYNPQIHSPVTHPQRAKVVGPEGEEIYVDEWGRIKVRFLFTRSDDHSHDGGAGTNNNDTDSAWIDVLT
PWAGEGYGARFLPRIGEIVVIDFFNGDIDRPFVMGRIHEAQRHPTKFDNKGKLPDTKKLSGIRSKEVSGGGF
GQLRFDDTPGQISTQLQSSHGASQLNLGKLSHPKDKAESEDRGEGFELRTDQWGALRAGQGLLVSTHKQDNA
KGDHLDAEVAKKQLEGSQTNSKALSDIAKNQKTDEIESI------------------------------------
---------------------------------------EQLKDFASQIQQ--------------------
-----------------------------------------------------------------QIAKF
EKALLLLSSPDGIALSSSEDIHISADAQINQIAGDSINISTQKNVIAHAQNRLSLFAAQSGLKAVAAQGKVE
IQAQSDALDVLANKGITISSTEDCIEISSPKEIVITGASSQITLNGSGIFPKTGGKFQVNAGQHVFQGGASA
SVQSSLPP------PPKRGQGVLELFHEYAHGEFVKQGGYTVT--DSLGKQFKGQLDDKGFVRVSGLAT-GS
AKIVFEEDKRNPWDEASDFKRPPMWPSDNDTSAQTLMGKADSVLKGAVTEAKKALG---QTFTNPSSIMRTI
DTAKQLKENGASALLPMVQQGVESHLTSQLTNFTSKVLSQSSTGQGWMHGKTNPVAPVTSGLNVNPLRAQDS
KNTETALNPMSFKFK--------
>AbH12O-A2_22
-MLNSIHQVLDSLGISPQKRAIHVQFTSPILNDQVFLQRIDGVHALNDGLKAELLCLSTNATIQLKSFIGVQ
AAVDIVTERGELTRVTGIITHAQQGQSDGSLTLYKLTLEDPTALWKYRRNSRVFMNKSVVEIWEILFKEWQT
KNPLFAASLSLDLSGLT-QTYEVRPFVMQHNESDWNFLTRLLRSENISWLIDEAQHIVPSTDTPIQAQKLRL

IDANSQYQPLDRKTIRYHRSSAVEQYDSMTRLTAERSLQPNVMHIQRWQAEILDQEEGIGSVQSKHQHSEHY
DNATLGLEQAWNYAPAWIGDLKGEDGVTKSGNQQVERLNQNLHNYYEAQAKRFIAQTTVRDAYVGYYFELNE
HPEIDQ-HESSDKSFLIVSKNFFNQNNLPKDLNDQINGLLAQSNWAIQNPE----NSDERQANQLILQRRHI
PTTPAYNPQIHSPVTHPQRAKVVGPEGEEIYVDEWGRIKVRFLFTRSDDHSHDGGAGTNNNDTDSAWIDVLT
PWAGEGYGARFLPRIGEIVVIDFFNGDIDRPFVMGRIHEAQRHPTKFDNKGKLPDTKKLSGIRSKEVSGGGF
GQLRFDDTPGQISTQLQSSHGASQLNLGKLSHPKDKAESEDRGEGFELRTDQWGALRAGQGLLVSTHKQDNA
KGDHLDAEVAKKQLEGSQTNSKALSDIAKNQKTDEIESI---------------------------------
----------------------------------------EQLKDFASQIQQ--------------------
-------------------------------------------------------------------QIAKF
EKALLLLSSPDGIALSSSEDIHISADAQINQIAGDSINISTQKNVIAHAQNRLSLFAAQSGLKAVAAQGKVE
IQAQSDALDVLANKGITISSTEDCIEISSPKEIVITGASSQITLNGSGIFPKTGGKFQVNAGQHVFQGGASA
SVQSSLPP------PPKRGQGVLELFHEYAHGEFVKQGGYTVT--DSLGKQFKGQLDDKGFVRVSGLAT-GS
AKIVFEEDKRNPWDEASDFKRPPMWPSDNDTSAQTLMGKADSVLKGAVTEAKKALG---QTFTNPSSIMRTI
DTAKQLKENGASALLPMVQQGVESHLTSQLTNFTSKVLSQSSTGQGWMHGKTNPVAPVTSGLNVNPLRAQDS
KNTETALNPMSFKFK--------
>AF-401_22
-MLNSIHQVLDSLGISPQKRAIHVQFTSPILNDQVFLQRIDGVHALNDGLKAELLCLSTNATIQLKSFIGVQ
AAVDIVTERGELTRVTGIITHAQQGQSDGSLTLYKLTLEDPTALWKYRRNSRVFMNKSVVEIWEILFKEWQT
KNPLFAASLSLDLSGLT-QTYEVRPFVMQHNESDWNFLTRLLRSENISWLIDEAQHIVPSTDTPIQAQKLRL
IDANSQYQPLDRKTIRYHRSSAVEQYDSMTRLTAERSLQPNVMHIQRWQAEILDQEEGIGSVQSKHQHSEHY
DNATLGLEQAWNYAPAWIGDLKGEDGVTKSGNQQVERLNQNLHNYYEAQAKRFIAQTTVRDAYVGYYFELNE
HPEIDQ-HESSDKSFLIVSKNFFNQNNLPKDLNDQINGLLAQSNWAIQNPE----NSDERQANQLILQRRHI
PTTPAYNPQIHSPVTHPQRAKVVGPEGEEIYVDEWGRIKVRFLFTRSDDHSHDGGAGTNNNDTDSAWIDVLT
PWAGEGYGARFLPRIGEIVVIDFFNGDIDRPFVMGRIHEAQRHPTKFDNKGKLPDTKKLSGIRSKEVSGGGF
GQLRFDDTPGQISTQLQSSHGASQLNLGKLSHPKDKAESEDRGEGFELRTDQWGALRAGQGLLVSTHKQDNA
KGDHLDAEVAKKQLEGSQTNSKALSDIAKNQKTDEIESI---------------------------------
----------------------------------------EQLKDFASQIQQ--------------------
-------------------------------------------------------------------QIAKF
EKALLLLSSPDGIALSSSEDIHISADAQINQIAGDSINISTQKNVIAHAQNRLSLFAAQSGLKAVAAQGKVE
IQAQSDALDVLANKGITISSTEDCIEISSPKEIVITGASSQITLNGSGIFPKTGGKFQVNAGQHVFQGGASA
SVQSSLPP------PPKRGQGVLELFHEYAHGEFVKQGGYTVT--DSLGKQFKGQLDDKGFVRVSGLAT-GS
AKIVFEEDKRNPWDEASDFKRPPMWPSDNDTSAQTLMGKADSVLKGAVTEAKKALG---QTFTNPSSIMRTI
DTAKQLKENGASALLPMVQQGVESHLTSQLTNFTSKVLSQSSTGQGWMHGKTNPVAPVTSGLNVNPLRAQDS

KNTETALNPMSFKFK--------
>AR_0063_22
-MLNSIHQVLDSLGISPQKRAIHVQFTSPILNDQVFLQRIDGVHALNDGLKAELLCLSTNATIQLKSFIGVQ
AAVDIVTERGELTRVTGIITHAQQGQSDGSLTLYKLTLEDPTALWKYRRNSRVFMNKSVVEIWEILFKEWQT
KNPLFAASLSLDLSGLT-QTYEVRPFVMQHNESDWNFLTRLLRSENISWLIDEAQHIVPSTDTPIQAQKLRL
IDANSQYQPLDRKTIRYHRSSAVEQYDSMTRLTAERSLQPNVMHIQRWQAEILDQEEGIGSVQSKHQHSEHY
DNATLGLEQAWNYAPAWIGDLKGEDGVTKSGNQQVERLNQNLHNYYEAQAKRFIAQTTVRDAYVGYYFELNE
HPEIDQ-HESSDKSFLIVSKNFFNQNNLPKDLNDQINGLLAQSNWAIQNPE----NSDERQANQLILQRRHI
PTTPAYNPQIHSPVTHPQRAKVVGPEGEEIYVDEWGRIKVRFLFTRSDDHSHDGGAGTNNNDTDSAWIDVLT
PWAGEGYGARFLPRIGEIVVIDFFNGDIDRPFVMGRIHEAQRHPTKFDNKGKLPDTKKLSGIRSKEVSGGGF
GQLRFDDTPGQISTQLQSSHGASQLNLGKLSHPKDKAESEDRGEGFELRTDQWGALRAGQGLLVSTHKQDNA
KGDHLDAEVAKKQLEGSQTNSKALSDIAKNQKTDEIESI----------------------------------
---------------------------------------EQLKDFASQIQQ--------------------
-----------------------------------------------------------------QIAKF
EKALLLLSSPDGIALSSSEDIHISADAQINQIAGDSINISTQKNVIAHAQNRLSLFAAQSGLKAVAAQGKVE
IQAQSDALDVLANKGITISSTEDCIEISSPKEIVITGASSQITLNGSGIFPKTGGKFQVNAGQHVFQGGASA
SVQSSLPP------PPKRGQGVLELFHEYAHGEFVKQGGYTVT--DSLGKQFKGQLDDKGFVRVSGLAT-GS
AKIVFEEDKRNPWDEASDFKRPPMWPSDNDTSAQTLMGKADSVLKGAVTEAKKALG---QTFTNPSSIMRTI
DTAKQLKENGASALLPMVQQGVESHLTSQLTNFTSKVLSQSSTGQGWMHGKTNPVAPVTSGLNVNPLRAQDS
KNTETALNPMSFKFK--------
>AR_0101_22
-MLNSIHQVLDSLGISPQKRAIHVQFTSPILNDQVFLQRIDGVHALNDGLKAELLCLSTNATIQLKSFIGVQ
AAVDIVTERGELTRVTGIITHAQQGQSDGSLTLYKLTLEDPTALWKYRRNSRVFMNKSVVEIWEILFKEWQT
KNPLFAASLSLDLSGLT-QTYEVRPFVMQHNESDWNFLTRLLRSENISWLIDEAQHIVPSTDTPIQAQKLRL
IDANSQYQPLDRKTIRYHRSSAVEQYDSMTRLTAERSLQPNVMHIQRWQAEILDQEEGIGSVQSKHQHSEHY
DNATLGLEQAWNYAPAWIGDLKGEDGVTKSGNQQVERLNQNLHNYYEAQAKRFIAQTTVRDAYVGYYFELNE
HPEIDQ-HESSDKSFLIVSKNFFNQNNLPKDLNDQINGLLAQSNWAIQNPE----NSDERQANQLILQRRHI
PTTPAYNPQIHSPVTHPQRAKVVGPEGEEIYVDEWGRIKVRFLFTRSDDHSHDGGAGTNNNDTDSAWIDVLT
PWAGEGYGARFLPRIGEIVVIDFFNGDIDRPFVMGRIHEAQRHPTKFDNKGKLPDTKKLSGIRSKEVSGGGF
GQLRFDDTPGQISTQLQSSHGASQLNLGKLSHPKDKAESEDRGEGFELRTDQWGALRAGQGLLVSTHKQDNA
KGDHLDAEVAKKQLEGSQTNSKALSDIAKNQKTDEIESI----------------------------------
---------------------------------------EQLKDFASQIQQ--------------------
-----------------------------------------------------------------QIAKF

EKALLLLSSPDGIALSSSEDIHISADAQINQIAGDSINISTQKNVIAHAQNRLSLFAAQSGLKAVAAQGKVE
IQAQSDALDVLANKGITISSTEDCIEISSPKEIVITGASSQITLNGSGIFPKTGGKFQVNAGQHVFQGGASA
SVQSSLPP------PPKRGQGVLELFHEYAHGEFVKQGGYTVT--DSLGKQFKGQLDDKGFVRVSGLAT-GS
AKIVFEEDKRNPWDEASDFKRPPMWPSDNDTSAQTLMGKADSVLKGAVTEAKKALG---QTFTNPSSIMRTI
DTAKQLKENGASALLPMVQQGVESHLTSQLTNFTSKVLSQSSTGQGWMHGKTNPVAPVTSGLNVNPLRAQDS
KNTETALNPMSFKFK--------
>AB031_27
-MLNSIHQVLDSLGISPQKRAIHVQFTSPILNAQVFLQRIDGVHALNDGLKAELLCLSTNATIQLKSFIGVQ
AAVDIVTERGELTRVTGIITHAQQGQSDGSLTLYKLTLEDPTALWKYRRNSRVFMNKSVVEIWEILFKEWQS
KNPLFAASLSLDLSGLT-QTYDVRPFVMQHNESDWNFLTRLLRSENISWLIDEAQHTVPSTDTSIQAQKLRL
IDANSQYQPLDRKTIRYHRSSAVEQYDSMTRLTAERSLQPNVMHIQRWQAEILDQEEGIGSVQSKHQHSEHY
DNATLGLEQAWNYSPAWIGDLKGEDGVTKSGNQQVERLNQNLHNYYEAQAKRFIAQTTVRDAYVGYYFELNE
HPEIDQ-HESTDRSFLIISKSFFNQNNLPKDLNDQINGLLAQSNWAIQNPE----NSDERQANQLILQRRHI
PITPAYNPQIHSPVTHPQRAKVVGPEGEEIYVDEWGRIKVRFLFTRSDDHSHDGGAGTNNNDTDSAWIDVLT
PWAGEGYGARFLPRIGEIVVIDFFNGDIDRPFVMGRIHEAQRHPTKFDNKGKLPDTKKLSGIRSKEVSGSGF
GQLRFDDTPGQISTQLQSSHGASQLNLGKLSHPKDKAESEDRGEGFELRTDQWGALRAGQGLLVSTHKQDYA
KGDHLDAEVAKKQLEGSQTNSKALSDIAKNQKTDEIESI----------------------------------
----------------------------------------EQLKDFASQIQQ--------------------
-------------------------------------------------------------------QIAKF
EKALLLLSSPDGIALSSSEDIHISADAQINQIAGDSINISTQKNVIAHAQNRLSLFAAQSGLKAVAAQGKIE
IQAQSDALDVLANKGITISSTEDCIEISSPKEIVITGASSQITLNGSGIFPKTGGKFQVNAGQHVFQGGASA
SVQSSLPP------PPKRGQGVLELFHEYAHGEFVKQGGYTVT--DSLGKQFKGQLDDKGFVRVSGLAT-GS
AKIVFEEDKRNPWDEASDFKRPPMWPSDNDTSAQTLMGKADSVLKGAVTEAKKALG---QTFTNPSSIMRTI
DTAKQLKENGASALLPMVQQGVESHLTGQLTNFTSKVLSQSSTGQGWMHGKTNPVAPVTSGLNVNPLRAQDS
KNTETALNPMSFKFK--------
>VgrG2Abay
-MSFHLFGALERLGLSAQQRALHLTLSDVSLNSQVYLQRIQGQQHLNQGSSAELICFSSYAHLPLKQFIGCQ
AAVDQVTDTGQLFRSSGIITAATQGQSDGALTLYTLTLQDPTALWHKRRNSRVFMNKSVRDISEILFKEWQN
KSPLFAASLTLDTSGLS-RDYDIRPFSMQANETDYDYLTRLWRSEGINWLIDEAQLIVPSITSPIQPQILRL
IDDNTAYQPLPRTSLRYQRSSATEPFDSITQFTATRSLQPTSVYLQRWQADALQQQEGSGSVQSTQHHSQHY
DNASLNLEDAWQISPAWTQDLNGEDQVTASSNTQIEQLNQQINAYHHLQSKQFTATGSVRDAQVGYWFQLDG
HPELDQ-HSNEERQLLIISKYYHNQNNLPKELAQQIQHLLPQ------QPN----SKEQRHSTELTLVRRNI
KTVPEYHPQQHQPQTHPQRAKVVGPEGEQVHVDEWGRIKVRFLFTRSEDHSHDGGAGSNDNDTDSAWVDVLT

PWAGEGYGARFLPRIGEIVVIDYFDGDIDRPFVLGRIHEAERSPTKFDIKGQLPHTRHLSGIRSEEIQGSGY
NQLRFDDTTGQISSQLHSSHGATQLNLGNLSHPKEQLESEGRGEGFELRTDQWGAVRAGEGLHLTTHKQEQA
QGQHLDAKEASQQLESSLNNAKALSEVAKNQQTDPLEVL------------------------------------
------------------------------------------DNLKQFLKQIEQ---------------------
-----------------------------------------------------------------QDQNKAAAF
KQALMILSAPSSIAMSSNQDIHLSADGQLSQHAGESLSFSTQKSLIAHASDKISLFAAQQGARLYAGKGKIE
LQAQGDGADLIARKDIQIISTEDVINISAQKRILITAKTSQVEINEQGIILRTGGKAEFKAGQHVFMGGQNI
NETILDLP-------------------------ELKDCEY-----KMKDAASTGKSSVRIK------------
--------------------------------------------------------------------------
--------------------------------------------------------------------------
------------------------
>VgrG3Abay
-MSFHLFGALERLGLSAQQRALHLTLSDVSLNSQVYLQRIQGQQQLNQGSSAELICFSSHAHLPLKQFIGCQ
AAVDQVTDTGQLFRSSGIITAATQGQSDGALTLYTLTLQDPTALWHKRRNSRVFMNKSVRDISEILFKEWQN
KSPLFAASLTLDTSGLS-RDYDIRPFSMQANETDYNYLTRLWRSEGINWLIDEAQLIVPSTTSSIQPQILRL
IDDNTAYQPLPRASLRYQRSSATEPFDSITQFTATRSLQPTSVYLQRWQADALQQQEGSGSVQSTQHHSQHY
DNASLNLEDAWQISPAWTQDLNGEDQVTASSNTQIEQINQQINAYHHLQSKQFTATGSVRDAQVGYWFQLDG
HPELDQ-HSNEERQLLIISKYYHNQNNLPKELAQQIQHLLPQ------QPN----SKEQRHSTQLTLVRRNI
KTVPEYHPQQ--PQTHPQHAKVVGPEGETVHVDEWGRIKVRFLFTRSEDHSHDGGAGSNDNDTDSAWVDVLT
PWAGEGYGARFLPRIGEIVVIDYFDGNIDRPFVLGRIHEAERSPTKFDIKGQLPHTRHLSGIRSEEIQGSGY
NQLRFDDTTGQISSQLHSSHGATQLNLGNLSHPKEQLESEGRGEGFELRTDQWGAIRAGEGLHLTTHKQEQA
QGQHLDAQEASQQLESSLNNAKALSEVAKNQQTDPLEVL------------------------------------
------------------------------------------DNLKQFLEQIEQ---------------------
-----------------------------------------------------------------QDQNKAAAF
KQALMILSAPSSIAMSSNQDIHLSADGQLSQHAGDSLNISTQKSLIAHASDKISLFAAQQGARLYAGKGKIE
LQAQGDGADLIARKDIQIISTEEAVYITSPKEINLTVNGSQLKLNDSGVFSTTGGKFEVKAGQHIFEGGEKV
TVSKAAEN----------KPCYLTFEIVDLDGNPAKNIEYIAF--RADGTHQEGRTDVSGKTQTFQTEG---
------------------SEQISIHISDKNASKYKLITKG-----------------------------------
--------------------------------------------------------------------------
------------------------
>AB042_3
MMSHKIFQILDSLGLVAQNRVLHVQFSNASLNNQVFLQRIEGEHTLNQGSVAELLCLSTNAHIALKQFIGCQ
VAVDQVTDTGQFFRTTGIITEASQGQSDGSLTIYNLTLKDPTALWHKRRNSRVFMNKSVRDISEILFKEWQG

KSPLFASSITLDTSGLT-KDYDVRPFVMQSNESDYDFLTRLWRSEGINWLVDESQLFVADPNASIQPQVLRL
IDDNQNYQALERRSIRYQRSSATEQFDTITQVKAERRLQPTSVHVQRWQADALQQEEGSGSVQGTQKHSEHY
DNASLNLEDAWHVSPAWMQDLNGKDQATASGNSQIEQLNQHINAYHHLSSKQFTVAGNVRDAQVGYWFELND
HPELDQ-HDSADKEFLILSKHYYNQNNLPKELQQQLERLLPQGKLKAAQLD--SQNPEQRHFAELNVVRRNI
KAVPEYNPLEHRPAAHPQRARVVGLEGESIHVDQWGRIKVRFLFTRADDHSHDGGAGSNDNDTDSAWVDVLT
PWAGAGYGARFLPRVGEIVVIDFFDGNVDRPFVVGRIHEAERQPTQFDQKGQLPDTKKLSGIRSEEVDGKGF
NQLRFDDTTGQISAQLQSSHAASQLNLGNLSHPKDKAESDGRGEGFELRTDQWGAVRAGSGLLISTHKQDQA
QGVHLDASEAKQQIEGGLNNAKALSEVAKNQQTDPLDML---------------------------------
----------------------------------------ENIQTFLEVLKQ--------------------
---------------------------------------------------------------EDPKKAAEF
QSAVMLLASPKSIAVSSNEDIHLSANGQLTQSAGDSINMSTQKNIVNHASQKISLFAAQEGARLFAGKGKVE
IQAQGDGLDVIARKGIQITSTEDTVFITSPTEINLTANGSQVKLNGSGIFPVTGGKLEVKAGQHLFMGGEQV
T-----PP-----KKQENKPCYLTFEITDLDGKPAKNVEYIAF--RMDGSRQKGQTNAQGLTQRFETDG---
-----------------SEQISIHICDENASKYKLAAKG---------------------------------
------------------------------------------------------------------------
-----------------------

>ATCC17978-mff_3
MMSHKIFQILDSLGLVAQNRVLHVQFSNASLNNQVFLQRIEGEHTLNQGSVAELLCLSTNAHIALKQFIGCQ
VAVDQVTDTGQFFRTTGIITEASQGQSDGSLTIYNLTLKDPTALWHKRRNSRVFMNKSVRDISEILFKEWQG
KSPLFASSITLDTSGLT-KDYDVRPFVMQSNESDYDFLTRLWRSEGINWLVDESQLFVADPNASIQPQVLRL
IDDNQNYQALERRSIRYQRSSATEQFDTITQVKAERRLQPTSVHVQRWQADALQQEEGSGSVQGTQKHSEHY
DNASLNLEDAWHVSPAWMQDLNGKDQATASGNSQIEQLNQHINAYHHLSSKQFTVAGNVRDAQVGYWFELND
HPELDQ-HDSADKEFLILSKHYYNQNNLPKELQQQLERLLPQGKLKAAQLD--SQNPEQRHFAELNVVRRNI
KAVPEYNPLEHRPAAHPQRARVVGLEGESIHVDQWGRIKVRFLFTRADDHSHDGGAGSNDNDTDSAWVDVLT
PWAGAGYGARFLPRVGEIVVIDFFDGNVDRPFVVGRIHEAERQPTQFDQKGQLPDTKKLSGIRSEEVDGKGF
NQLRFDDTTGQISAQLQSSHAASQLNLGNLSHPKDKAESDGRGEGFELRTDQWGAVRAGSGLLISTHKQDQA
QGVHLDASEAKQQIEGGLNNAKALSEVAKNQQTDPLDML---------------------------------
----------------------------------------ENIQTFLEVLKQ--------------------
---------------------------------------------------------------EDPKKAAEF
QSAVMLLASPKSIAVSSNEDIHLSANGQLTQSAGDSINMSTQKNIVNHASQKISLFAAQEGARLFAGKGKVE
IQAQGDGLDVIARKGIQITSTEDTVFITSPTEINLTANGSQVKLNGSGIFPVTGGKLEVKAGQHLFMGGEQV
T-----PP-----KKQENKPCYLTFEITDLDGKPAKNVEYIAF--RMDGSRQKGQTNAQGLTQRFETDG---
-----------------SEQISIHICDENASKYKLAAKG---------------------------------

----------------------------------------------------------------------
----------------------
>ZW85-1_20
MMSHKIFQILDSLGLVAQNRVLHVQFSNASLNNQVFLQRIEGEHTLNQGSVAELLCLSTNAHIALKQFIGCQ
VAVDQVTDTGQFFRTTGIITEASQGQSDGSLTIYNLTLKDPTALWHKRRNSRVFMNKSVRD-TRILFKEWQG
KSPLSAASLTLDTSGLT-KDYDVRPFVMQSNESDYDFLTRLWRSEGINWLVDESQLFVADPNASIQPQVLRL
IDDNQNYQALERRSIRYQRSSATEQFDTITQVKAERRLQPTSVHVQRWQADALQQEEGSGSVQGTQKHSEHY
DNASLNLEDAWHVSPAWMQDLNGEDQATASGNSQIEQLNQHINAYHHLSSKQFTVAGNVRDAQVGYWFELND
HPELDQ-HDSADKEFLILNKHYYNQNNLPKELQQQLERLLPQGKLKAAQLD--SQNPEQRHFAELNVVRRNI
KAVPEYNPLEHRPAAHPQRARVVGLEGESIHVDQWGRIKVRFLFTRADDHSHDGGAGSNDNDTDSAWVDVLT
PWAGAGYGARFLPRVGEIVVIDFFDGNIDRPFVVGRIHEAERHPTQFDQKGQLPDTKKLSGIRSEEVDGKGF
NQLRFDDTTGQISAQLQSSHAASQLNLGNLSHPKDKAESDGRGEGFELRTDQWGAVRAGSGLLVSTHKQDQA
QGVHLDASEAKQQIEGGLNNAKALSEVAKNQQTDPLDML-------------------------------
---------------------------------------ENIQTFLEVLKQ-------------------
-------------------------------------------------------------EDPKKAAEF
QSAVMLLASPKSIAVSSNEDIHLSANGQLTQSAGDSINMSTQKNIVNHASQKISLFAAQEGARLFAGKGKVE
IQAQGDGLDVIARKGVQITSTEDTVYITSPTEINLTANGSQVKLNDSGIFPVTGGKLEVKAGQHLFRGGSSI
NPPALDLP------------------------------------DCSAKQTQAAQNGSAKVDLS------
----------------------------------------------------------------------
----------------------------------------------------------------------
----------------------
>XH858_12
MMSHKIFQILDSLGLVAQNRVLHVQFSNASLNNQVFLQRIEGEHTLNQGSVAELLCLSTNAHIALKQFIGCQ
VAVDQVTDTGQFFRTTGIITEASQGQSDGSLTIYNLTLKDPTALWHKRRNSRVFMNKSVRDISEILFKEWQE
KSPLFASSITFDTSGLS-KDYDVRPFVMQSNESDYDFLTRLWRSEGINWLVDESRLFVADPNASIQSQVLRL
IDDNQNYQALERRSIRYQRSSATEQFDTITQVKAERHLQPTSVHVQRWQADALQQEEGSGSVQGTQKHSEHY
DNASLNLEDAWHVSPAWMQDLNGEDQATASGNSQIEQLNQHINAYHHLSSKQFTVAGNVRDAQVGYWFELND
HPELDQ-HDSADKEFLILSKHYYNQNNLPKELQQQLERLLPQGKLKAAQLD--SQNPEQRHFAELNVVRRNI
KAVPEYNPLEHRPAAHPQRARVVGLEGESIHVDQWGRIKVRFLFTRVDDHSHDGGAGSNDNDTDSAWVDVLT
PWAGAGYGARFLPRVGEIVVIDFFDGNIDRPFVVGRIHEAERHPTQFDQKGQLPDTKKLSGIRSEEVDGKGF
NQLRFDDTTGQISAQLQSSHAASQLNLGNLSHPKDKAESDGRGEGFELRTDQWGAVRAGSGLLVSTHKQDQA
QGVHLDASEAKQQIEGGLNNAKALSEVAKNQQTDPLDML-------------------------------
---------------------------------------ENIQTFLEVLKQ-------------------

---------------------------------------------------------------EDPKKAAEF
QSAVMLLASPKSIAVSSNEDIHLSANGQLTQSAGDSINMSTQKNIVNHASQKISLFAAQEGARLFAGKGKVE
IQAQGDGLDVIARKGVQITSTEDTVYITSPTEINLTANGSQVKLNGSGIFPVTGGKLEVKAGQHLFMGGSSI
NPPALDLP-------------------------------------DCSAKQTQAAQNGSAKVDLS-------
------------------------------------------------------------------------
------------------------------------------------------------------------
-----------------------
>D1279779_12
MMSHKIFQILDSLGLVAQNRVLHVQFSNASLNNQVFLQRIEGEHTLNQGSVAELLCLSTNAHISLKQFIGCQ
VAVDQVTDTGQFFRTTGIITEASQGQSDGSLTIYNLTLKDPTALWHKRRNSRVFMNKSVRDISEILFKEWQG
KSPLFASSITLDRSGLT-KDYDVRPFVMQSNESDYDFLTRLWRSEGINWLVDESQLFVADPNASIQPQVLRL
IDDNQNYQALERRSIRYQRSSATEQFDTITQVKAERRLQPTSVHVQRWQADALQQEEGSGSVQGTQKHSEHY
DNASLNLEDAWHVSPAWMQDLNGEDQATASGNSQIEQLNQHINAYHHLSSKQFTVAGNVRDAQVGYWFELNE
HPELDQ-HDSADKEFLILSKHYYNQNNLPKELQQQLERLLPQGKLKAAQLD--SQNPEQRHFAELNVVRRNI
KAVPEYNPLEHRPAAHPQRARVVGLEGESIHVDQWGRIKVRFLFTRADDHSHDGGAGSNDNDTDSAWIDVLT
PWAGAGYGARFLPRVGEIVVIDFFDGNIDRPFVVGRIHEAERHPTQFDQKGQLPDTKKLSGIRSEEVDGKGF
NQLRFDDTTGQISAQLQSSHAASQLNLGNLSHPKDKAESDGRGEGFELRTDQWGAVRAGSGLLVSTHKQDQA
QGVHLDASEAKQQIEGGLNNAKALSEVAKNQQTDPLDML---------------------------------
----------------------------------------ENIQTFLEVLKQ--------------------
---------------------------------------------------------------EDPKKAAEF
QSAVMLLASPKSIAVSSNEDIHLSANGQLTQSAGDSINMSTQKNIVNHASQKISLFAAQEGARLFAGKGKVE
IQAQGDGLDVIARKGIQITSTEDTVFITSPTEINLTANGSQVKLNGSGIFPVTGGKLEVKAGQHLFMGGSSI
NPPALDLP-------------------------------------DCSAKQTQAAQNGSAKVDLS-------
------------------------------------------------------------------------
------------------------------------------------------------------------
-----------------------
>R2090_12
MMSHKIFQILDSLGLVAQNRVLHVQFSNASLNNQVFLQRIEGEHTLNQGSVAELLCLSTNAHISLKQFIGCQ
VAVDQVTDTGQFFRTTGIITEASQGQSDGSLTIYNLTLKDPTALWHKRRNSRVFMNKSVRDISEILFKEWQG
KSPLFASSITLDRSGLT-KDYDVRPFVMQSNESDYDFLTRLWRSEGINWLVDESQLFVADPNASIQPQVLRL
IDDNQNYQALERRSIRYQRSSATEQFDTITQVKAERRLQPTSVHVQRWQADALQQEEGSGSVQGTQKHSEHY
DNASLNLEDAWHVSPAWMQDLNGEDQATASGNSQIEQLNQHINAYHHLSSKQFTVAGNVRDAQVGYWFELNE
HPELDQ-HDSADKEFLILSKHYYNQNNLPKELQQQLERLLPQGKLKAAQLD--SQNPEQRHFAELNVVRRNI

KAVPEYNPLEHRPAAHPQRARVVGLEGESIHVDQWGRIKVRFLFTRADDHSHDGGAGSNDNDTDSAWIDVLT
PWAGAGYGARFLPRVGEIVVIDFFDGNIDRPFVVGRIHEAERHPTQFDQKGQLPDTKKLSGIRSEEVDGKGF
NQLRFDDTTGQISAQLQSSHAASQLNLGNLSHPKDKAESDGRGEGFELRTDQWGAVRAGSGLLVSTHKQDQA
QGVHLDASEAKQQIEGGLNNAKALSEVAKNQQTDPLDML-----------------------------------
---------------------------------------ENIQTFLEVLKQ--------------------
--------------------------------------------------------------EDPKKAAEF
QSAVMLLASPKSIAVSSNEDIHLSANGQLTQSAGDSINMSTQKNIVNHASQKISLFAAQEGARLFAGKGKVE
IQAQGDGLDVIARKGIQITSTEDTVFITSPTEINLTANGSQVKLNGSGIFPVTGGKLEVKAGQHLFMGGSSI
NPPALDLP------------------------------------DCSAKQTQAAQNGSAKVDLS-------
------------------------------------------------------------------------
------------------------------------------------------------------------
----------------------
>AR_0088_12
MMSHKIFQILDSLGLVAQNRVLHVQFSNASLNNQVFLQRIEGEHTLNQGSVAELLCLSTNAHIALKQFIGCQ
VAVDQVTDTGQFFRTTGIITEASQGQSDGSLTIYNLTLKDPTALWHKRRNSRVFMNKSVRDISEILFKEWQE
KSPLFASSITLDTSGLT-KDYDVRPFVMQSNESDYDFLTRLWRSEGINWLVDESQLFVADPNASIQPQVLRL
IDDNQNYQALERRSIRYQRSSATEQFDTITQVKAERRLQPTSVHVQRWQADALQQEEGSGSVQGTQKHSEHY
DNASLNLEDAWHVSPAWMQDLNGEDQATASGNSQIEQLNQHINAYHHLSSKQFTVAGNVRDAQVGYWFELNE
HPELDQ-HDSADKEFLILSKHYYNQNNLPKELQQQLECLLPQGKLKAAQLD--SQNPEQRHFAELNVVRRNI
KAVPEYNPLEHRPAAHPQRARVVGLEGESIHVDQWGRIKVRFLFTRADDHSHDGGAGSNDNDTDSAWIDVLT
PWAGAGYGARFLPRVGEIVVIDFFDGNIDRPFVVGRIHEAERHPTQFDQKGQLPDTKKLSGIRSEEVDGKGF
NQLRFDDTTGQISAQLQSSHAASQLNLGNLSHPKDKAESDGRGEGFELRTDQWGAVRAGSGLLISTHKQDQA
QGVHLDASEAKQQIEGGLNNAKALSEVAKNQQTDPLDML-----------------------------------
---------------------------------------ENIQTFLEVLKQ--------------------
--------------------------------------------------------------EDPKKAAEF
QSAVMLLASPKSIAVSSNEDIHLSANGQLTQSAGDSINMSTQKNIVNHASQKISLFAAQEGARLFAGKGKVE
IQAQGDGLDVIARKGVQITSTEDTVYITSPTEINLTANGSQVKLNGSGIFPVTGGKLEVKAGQHLFMGGSSI
NPPALDLP------------------------------------DCSAKQTQAAQNGSAKVDLS-------
------------------------------------------------------------------------
------------------------------------------------------------------------
----------------------
>CIP70.10_12
MMSHKIFQILDSLGLVAQNRVLHVQFSNASLNNQVFLQRIEGEHTLNQGSVAELLCLSTNAHIALKQFIGCQ

VAVDQVTDTGQFFRTTGIITEASQGQSDGSLTIYNLTLKDPTALWHKRRNSRVFMNKSVRDISEILFKEWQE
KSPLFASSITLDTSGLT-KDYDVRPFVMQSNESDYDFLTRLWRSEGINWLVDESQLFVADPNASIQPQVLRL
IDDNQNYQALERRSIRYQRSSATEQFDTITQVKAERRLQPTSVHVQRWQADALQQEEGSGSVQGTQKHSEHY
DNASLNLEDAWHVSPAWMQDLNGEDQATASGNSQIEQLNQHINAYHHLSSKQFTVAGNVRDAQVGYWFELNE
HPELDQ-HDSADKEFLILSKHYYNQNNLPKELQQQLECLLPQGKLKAAQLD--SQNPEQRHFAELNVVRRNI
KAVPEYNPLEHRPAAHPQRARVVGLEGESIHVDQWGRIKVRFLFTRADDHSHDGGAGSNDNDTDSAWIDVLT
PWAGAGYGARFLPRVGEIVVIDFFDGNIDRPFVVGRIHEAERHPTQFDQKGQLPDTKKLSGIRSEEVDGKGF
NQLRFDDTTGQISAQLQSSHAASQLNLGNLSHPKDKAESDGRGEGFELRTDQWGAVRAGSGLLISTHKQDQA
QGVHLDASEAKQQIEGGLNNAKALSEVAKNQQTDPLDML---------------------------------
----------------------------------------ENIQTFLEVLKQ--------------------
---------------------------------------------------------------EDPKKAAEF
QSAVMLLASPKSIAVSSNEDIHLSANGQLTQSAGDSINMSTQKNIVNHASQKISLFAAQEGARLFAGKGKVE
IQAQGDGLDVIARKGVQITSTEDTVYITSPTEINLTANGSQVKLNGSGIFPVTGGKLEVKAGQHLFMGGSSI
NPPALDLP-------------------------------------DCSAKQTQAAQNGSAKVDLS-------
------------------------------------------------------------------------
------------------------------------------------------------------------
-----------------------
>HWBA8_12
MMSHKIFQILDSLGLVAQNRVLHVQFSNASLNNQVFLQRIEGEHTLNQGSVAELLCLSTNAHIALKQFIGCQ
VAVDQVTDTGQFFRTTGIITEASQGQSDGSLTIYNLTLKDPTALWHKRRNSRVFMNKSVRDISEILFKEWQE
KSPLFASSITLDTSGLT-KDYDVRPFVMQSNESDYDFLTRLWRSEGINWLVDESQLFVADPNASIQPQVLRL
IDDNQNYQALERRSIRYQRSSATEQFDTITQVKAERRLQPTSVHVQRWQADALQQEEGSGSVQGTQKHSEHY
DNASLNLEDAWHVSPAWMQDLNGEDQATASGNSQIEQLNQHINAYHHLSSKQFTVAGNVRDAQVGYWFELNE
HPELDQ-HDSADKEFLILSKHYYNQNNLPKELQQQLECLLPQGKLKAAQLD--SQNPEQRHFAELNVVRRNI
KAVPEYNPLEHRPAAHPQRARVVGLEGESIHVDQWGRIKVRFLFTRADDHSHDGGAGSNDNDTDSAWIDVLT
PWAGAGYGARFLPRVGEIVVIDFFDGNIDRPFVVGRIHEAERHPTQFDQKGQLPDTKKLSGIRSEEVDGKGF
NQLRFDDTTGQISAQLQSSHAASQLNLGNLSHPKDKAESDGRGEGFELRTDQWGAVRAGSGLLISTHKQDQA
QGVHLDASEAKQQIEGGLNNAKALSEVAKNQQTDPLDML---------------------------------
----------------------------------------ENIQTFLEVLKQ--------------------
---------------------------------------------------------------EDPKKAAEF
QSAVMLLASPKSIAVSSNEDIHLSANGQLTQSAGDSINMSTQKNIVNHASQKISLFAAQEGARLFAGKGKVE
IQAQGDGLDVIARKGVQITSTEDTVYITSPTEINLTANGSQVKLNGSGIFPVTGGKLEVKAGQHLFMGGSSI
NPPALDLP-------------------------------------DCSAKQTQAAQNGSAKVDLS-------

------------------------------------------------------------------------
------------------------------------------------------------------------
-----------------------
>R2091_12
MMSHKIFQILDSLGLVAQNRVLHVQFSNASLNNQVFLQRIEGEHTLNQGSVAELLCLSTNAHIALKQFIGCQ
VAVDQVTDTGQFFRTTGIITEASQGQSDGSLTIYNLTLKDPTALWHKRRNSRVFMNKSVRDISEILFKEWQE
KSPLFASSITLDTSGLT-KDYDVRPFVMQSNESDYDFLTRLWRSEGINWLVDESQLFVADPNASIQPQVLRL
IDDNQNYQALERRSIRYQRSSATEQFDTITQVKAERRLQPTSVHVQRWQADALQQEEGSGSVQGTQKHSEHY
DNASLNLEDAWHVSPAWMQDLNGEDQATASGNSQIEQLNQHINAYHHLSSKQFTVAGNVRDAQVGYWFELNE
HPELDQ-HDSADKEFLILSKHYYNQNNLPKELQQQLECLLPQGKLKAAQLD--SQNPEQRHFAELNVVRRNI
KAVPEYNPLEHRPAAHPQRARVVGLEGESIHVDQWGRIKVRFLFTRADDHSHDGGAGSNDNDTDSAWIDVLT
PWAGAGYGARFLPRVGEIVVIDFFDGNIDRPFVVGRIHEAERHPTQFDQKGQLPDTKKLSGIRSEEVDGKGF
NQLRFDDTTGQISAQLQSSHAASQLNLGNLSHPKDKAESDGRGEGFELRTDQWGAVRAGSGLLISTHKQDQA
QGVHLDASEAKQQIEGGLNNAKALSEVAKNQQTDPLDML---------------------------------
----------------------------------------ENIQTFLEVLKQ--------------------
-----------------------------------------------------------------EDPKKAAEF
QSAVMLLASPKSIAVSSNEDIHLSANGQLTQSAGDSINMSTQKNIVNHASQKISLFAAQEGARLFAGKGKVE
IQAQGDGLDVIARKGVQITSTEDTVYITSPTEINLTANGSQVKLNGSGIFPVTGGKLEVKAGQHLFMGGSSI
NPPALDLP-------------------------------------DCSAKQTQAAQNGSAKVDLS-------
------------------------------------------------------------------------
------------------------------------------------------------------------
-----------------------
>BJAB0715_12
MMSHKIFQILDSLGLVAQNRVLHVQFSNASLNNQVFLQRIEGEHTLNQGSGAELLCLSTNAHIALKQFIGCQ
VAVDQVTDTGQFFRTTGIITEASQGQSDGSLTIYNLTLKDPTALWHKRRNSRVFMNKSVRDISEILFKEWQG
KSPLFASSITLDTSGLT-KDYDVRPFVMQSNESDYDFLTRLWRSEGINWLVDESQLFVADPNASIQPQVLRL
IDDNQNYQALERRSIRYQRSSATEQFDTITQVKAERHLQPTSVHVQRWQADALQQEEGSGSVQGTQKHSEHY
DNASLNLEDAWHVSPAWMQDLNGEDQATASGNSQIEQLNQHINAYHHLSSKQFTVAGNVRDAQVGYWFELNE
HPELDQ-HDSADKEFLILSKHYYNQNNLPKELQQQLERLLPQGKLKAAQLD--SQNPEQRHFAELNVVRRNI
KAVPEYNPLEHRPAAHPQRARVVGLEGESIHVDQWGRIKVRFLFTRVDDHSHDGGAGSNDNDTDSAWVDVLT
PWAGAGYGARFLPRVGEIVVIDFFDGNIDRPFVVGRIHEAERHPTQFDQKGQLPDTKKLSGIRSEEVDGKGF
NQLRFDDTTGQISAQLQSSHAASQLNLGNLSHPKDKAESDGRGEGFELRTDQWGAVRAGSGLLVSTHKQDQA
QGVHLDASEAKQQIEGGLNNAKALSEVAKNQQTDPLDML---------------------------------

----------------------------------------ENIQTFLEVLKQ--------------------
---------------------------------------------------------------EDPKKAAEF
QSAVMLLASPKSIAVSSNEDIHLSANGQLTQSAGDSINMSTQKNIVNHASQKISLFAAQEGARLFAGKGKVE
IQAQGDGLDVIARKGVQITSTEDTVYITSPTEINLTANGSQVKLNGSGIFPVTGGKLEVKAGQHLFMGGSSI
NPPALDLP-------------------------------------DCSAKQTQAAQNGSAKVDLS-------
------------------------------------------------------------------------
------------------------------------------------------------------------
-----------------------
>Ab04-mff_12
MMSHKIFQILDSLGLVAQNRVLHVQFSNASLNNQVFLQRIEGEHTLNQGSVAELLCLSTNAHIALKQFIGCQ
VAVDQVTDTGQFFRTTGIITEASQGQSDGSLTIYNLTLKDPTALWHKRRNSRVFMNKSVRDISEILFKEWQG
KSPLFASSITLDTSGLT-KDYDVRPFVMQSNESDYDFLTRLWRSEGINWLVDESQLFVADPNASIQPQVLRL
IDDNQNYQALERRSIRYQRSSATEQFDTITQVKAERRLQPTSVHVQRWQADALQQEEGSGSVQGTQKHSEHY
DNASLNLEDAWHVSPAWMQDLNGEDQATASGNSQIEQLNQHINAYHHLSSKQFTVAGNVRDAQVGYWFELNE
HPELDQ-HDSADKEFLILSKHYYNQNNLPKELQQQLERLLPQGKLKAAQLD--SQNPEQRHFAELNVVRRNI
KAVPEYNPLEHRPAAHPQRARVVGLEGESIHVDQWGRIKVRFLFTRADDHSHDGGAGSNDNDTDSAWVDVLT
PWAGAGYGARFLPRVGEIVVIDFFDGNIDRPFVVGRIHEAERHPTQFDQKGQLPDTKKLSGIRSEEVDGKGF
NQLRFDDTTGQISAQLQSSHAASQLNLGNLSHPKDKAESDGRGEGFELRTDQWGAVRAGSGLLVSTHKQDQA
QGVHLDASEAKQQIEGGLNNAKALSEVAKNQQTDPLDML---------------------------------
----------------------------------------ENIQTFLEVLKQ--------------------
---------------------------------------------------------------EDPKKAAEF
QSAVMLLASPKSIAVSSNEDIHLSANGQLTQSAGDSINMSTQKNIVNHASQKISLFAAQEGARLFAGKGKVE
IQAQGDGLDVIARKGVQITSTEDTVYITSPTEINLTANGSQVKLNGSGIFPVTGGKLEVKAGQHLFMGGSSI
NPPALDLP-------------------------------------DCSAKQTQAAQNGSAKVDLS-------
------------------------------------------------------------------------
------------------------------------------------------------------------
-----------------------
>LAC-4_12
MMSHKIFQILDSLGLVAQNRVLHVQFSNASLNNQVFLQRIEGEHTLNQGSVAELLCLSTNAHIALKQFIGCQ
VAVDQVTDTGQFFRTTGIITEASQGQSDGSLTIYNLTLKDPTALWHKRRNSRVFMNKSVRDISEILFKEWQG
KSPLFASSITLDTSGLT-KDYDVRPFVMQSNESDYDFLTRLWRSEGINWLVDESQLFVADPNASIQPQVLRL
IDDNQNYQALERRSIRYQRSSATEQFDTITQVKAERRLQPTSVHVQRWQADALQQEEGSGSVQGTQKHSEHY
DNASLNLEDAWHVSPAWMQDLNGEDQATASGNSQIEQLNQHINAYHHLSSKQFTVAGNVRDAQVGYWFELNE

HPELDQ-HDSADKEFLILSKHYYNQNNLPKELQQQLERLLPQGKLKAAQLD--SQNPEQRHFAELNVVRRNI
KAVPEYNPLEHRPAAHPQRARVVGLEGESIHVDQWGRIKVRFLFTRADDHSHDGGAGSNDNDTDSAWVDVLT
PWAGAGYGARFLPRVGEIVVIDFFDGNIDRPFVVGRIHEAERHPTQFDQKGQLPDTKKLSGIRSEEVDGKGF
NQLRFDDTTGQISAQLQSSHAASQLNLGNLSHPKDKAESDGRGEGFELRTDQWGAVRAGSGLLVSTHKQDQA
QGVHLDASEAKQQIEGGLNNAKALSEVAKNQQTDPLDML-------------------------------
---------------------------------------ENIQTFLEVLKQ-------------------
-------------------------------------------------------------EDPKKAAEF
QSAVMLLASPKSIAVSSNEDIHLSANGQLTQSAGDSINMSTQKNIVNHASQKISLFAAQEGARLFAGKGKVE
IQAQGDGLDVIARKGVQITSTEDTVYITSPTEINLTANGSQVKLNGSGIFPVTGGKLEVKAGQHLFMGGSSI
NPPALDLP-----------------------------------DCSAKQTQAAQNGSAKVDLS-------
----------------------------------------------------------------------
----------------------------------------------------------------------
----------------------
>WKA02_12
MMSHKIFQILDSLGLVAQNRVLHVQFSNASLNNQVFLQRIEGEHTLNQGSVAELLCLSTNAHIALKQFIGCQ
VAVDQVTDTGQFFRTTGIITEASQGQSDGSLTIYNLTLKDPTALWHKRRNSRVFMNKSVRDISEILFKEWQG
KSPLFASSITLDTSGLT-KDYDVRPFVMQSNESDYDFLTRLWRSEGINWLVDESQLFVADPNASIQPQVLRL
IDDNQNYQALERRSIRYQRSSATEQFDTITQVKAERRLQPTSVHVQRWQADALQQEEGSGSVQGTQKHSEHY
DNASLNLEDAWHVSPAWMQDLNGEDQATASGNSQIEQLNQHINAYHHLSSKQFTVAGNVRDAQVGYWFELNE
HPELDQ-HDSADKEFLILSKHYYNQNNLPKELQQQLERLLPQGKLKAAQLD--SQNPEQRHFAELNVVRRNI
KAVPEYNPLEHRPAAHPQRARVVGLEGESIHVDQWGRIKVRFLFTRADDHSHDGGAGSNDNDTDSAWVDVLT
PWAGAGYGARFLPRVGEIVVIDFFDGNIDRPFVVGRIHEAERHPTQFDQKGQLPDTKKLSGIRSEEVDGKGF
NQLRFDDTTGQISAQLQSSHAASQLNLGNLSHPKDKAESDGRGEGFELRTDQWGAVRAGSGLLVSTHKQDQA
QGVHLDASEAKQQIEGGLNNAKALSEVAKNQQTDPLDML-------------------------------
---------------------------------------ENIQTFLEVLKQ-------------------
-------------------------------------------------------------EDPKKAAEF
QSAVMLLASPKSIAVSSNEDIHLSANGQLTQSAGDSINMSTQKNIVNHASQKISLFAAQEGARLFAGKGKVE
IQAQGDGLDVIARKGVQITSTEDTVYITSPTEINLTANGSQVKLNGSGIFPVTGGKLEVKAGQHLFMGGSSI
NPPALDLP-----------------------------------DCSAKQTQAAQNGSAKVDLS-------
----------------------------------------------------------------------
----------------------------------------------------------------------
----------------------
>CIP70.10_31

-MQMSVSSILERLGLVSQNRAVHIQFSNQSLNQQVFLQRIEGEHTLNQGSVAELLCLSTNAHIALKQFIGCQ
VAVDQVTDTGQFFRTTGIITEASQGQSDGSLTIYNLTLKDPTALWHKRRNSRVFMNKSVRDISEILFKEWQG
KSPLFASSITLDTSGLT-KDYDVRPFVMQSNESDYDFLTRLWRSEGINWLVDESQLFVAEPNASIQPQVLRL
IDDNQNYQALEHRSIRYQRSSATEQFDTITQVKAERRLQPTSVHVQRWQADALQQEEGSGSVQGTQKHSEHY
DNASLNLEDAWHVSPAWMQDLNGEDQATASGNSQIEQLNQHINAYHHLSSKQFTVAGNVRDAQVGYWFELND
HPELDQ-HDSADKEFLILSKHYYNQNNLPIELQQQLERLLPQGKLKAAQLD--SQNPEQRHFAELNIVRRNI
KAVPEYNPLEHRPAAHPQRARVVGLEGESIHVDQWGRIKVRFLFTRTDDHSHDGGAGSNDNDTDSAWIDVLT
PWAGAGYGARFLPRVGEIVVIDFFDGNIDRPFVVGRIHEAERHPTQFDQKGQLPDTKKLSGIRSEEVDGKGF
NQLRFDDTTGQISAQLQSSHAASQLNLGNLSHPKDKAESDGRGEGFELRTDQWGAVRAGSGLLVSTHKQDQA
QGVHLDASEAKQQIEGGLNNAKALSEVAKNQQTDPLEML---------------------------------
----------------------------------------ENLKTFIEQIEE--------------------
-----------------------------------------------------------KDQDKAAAF
KQALMILTAPNSIALASNEDIHLSADGQLNQTAGDSINLSTQKNLIAHAQNKISLFAAQQGARLYAGKGKVE
IQAQGDGADLIARKAVQVISTEDKIEATAAKEIVLTAGGSQVKITGSGIFMTTSGKFEVKAGQHLFQSGEKV
PYNLPPLG---------ELKSFLELNYHWPTLEPMKGAPYLVV--FESGQEFKGNLDDKGFAKIPNVPTNES
YTVWYGEDQRKVEIEVLEESILENFTDQDEIELDELLKNWDVEN----------------------------
------------------------------------------------------------------------
-----------------------

>R2091_31
-MQMSVSSILERLGLVSQNRAVHIQFSNQSLNQQVFLQRIEGEHTLNQGSVAELLCLSTNAHIALKQFIGCQ
VAVDQVTDTGQFFRTTGIITEASQGQSDGSLTIYNLTLKDPTALWHKRRNSRVFMNKSVRDISEILFKEWQG
KSPLFASSITLDTSGLT-KDYDVRPFVMQSNESDYDFLTRLWRSEGINWLVDESQLFVAEPNASIQPQVLRL
IDDNQNYQALEHRSIRYQRSSATEQFDTITQVKAERRLQPTSVHVQRWQADALQQEEGSGSVQGTQKHSEHY
DNASLNLEDAWHVSPAWMQDLNGEDQATASGNSQIEQLNQHINAYHHLSSKQFTVAGNVRDAQVGYWFELND
HPELDQ-HDSADKEFLILSKHYYNQNNLPIELQQQLERLLPQGKLKAAQLD--SQNPEQRHFAELNIVRRNI
KAVPEYNPLEHRPAAHPQRARVVGLEGESIHVDQWGRIKVRFLFTRTDDHSHDGGAGSNDNDTDSAWIDVLT
PWAGAGYGARFLPRVGEIVVIDFFDGNIDRPFVVGRIHEAERHPTQFDQKGQLPDTKKLSGIRSEEVDGKGF
NQLRFDDTTGQISAQLQSSHAASQLNLGNLSHPKDKAESDGRGEGFELRTDQWGAVRAGSGLLVSTHKQDQA
QGVHLDASEAKQQIEGGLNNAKALSEVAKNQQTDPLEML---------------------------------
----------------------------------------ENLKTFIEQIEE--------------------
-----------------------------------------------------------KDQDKAAAF
KQALMILTAPNSIALASNEDIHLSADGQLNQTAGDSINLSTQKNLIAHAQNKISLFAAQQGARLYAGKGKVE
IQAQGDGADLIARKAVQVISTEDKIEATAAKEIVLTAGGSQVKITGSGIFMTTSGKFEVKAGQHLFQSGEKV

PYNLPPLG---------ELKSFLELNYHWPTLEPMKGAPYLVV--FESGQEFKGNLDDKGFAKIPNVPTNES
YTVWYGEDQRKVEIEVLEESILENFTDQDEIELDELLKNWDVEN----------------------------
------------------------------------------------------------------------
-----------------------
>ab736_7
-MQMSVSSILERLGLVSQNRAVHIQFSNQSLNQQVFLQRIEGEHTLNQGSVAELLCLSTNAHIALKQFIGCQ
VAVDQVTDTGQFFRTTGIITEASQGQSDGSLTIYNLTLKDPTALWHKRRNSRVFMNKSVRDISEILFKEWQG
KSPLFASSITLDTSGLT-KDYDVRPFVMQSNESDYDFLTRLWRSEGINWLVDESQLFVADPNASIQPQVLRL
IDDNQNYQALERRSIRYQRSSATEQFDTITQVKAERRLQPTSVHVQRWQADALQQEEGSGSVQGTQKHSEHY
DNASLNLEDAWHVSPAWMQDLNGEDQATASGNSQIEQLNQHINAYHHLSSKQFTVAGNVRDAQVGYWFELND
HPELDQ-HDSADKEFLILSKHYYNQNNLPKELQQQLERLLPQGKLKAAQLD--SQNPEQRHFAELNIVRRNI
KAVPEYNPLEHRPAAHPQRARVVGLEGESIHVDQWGRIKVRFLFTRADDHSHDGGAGSNDNDTDSAWVDVLT
PWAGAGYGARFLPRVGEIVVIDFFDGNIDRPFVVGRIHEAERHPTQFDQKGQLPDTKKLSGIRSEEVDGKGF
NQLRFDDTTGQISAQLQSSHAASQLNLGNLSYPKDKAESDGRGEGFELRTDQWGAVRAGSGLLISTHKQDQA
QGVHLDANEAKQQIEGGLNNAKALSEVAKNQQTDPLEML---------------------------------
----------------------------------------ENLKTFIEQIEE--------------------
---------------------------------------------------------------KDQDKAAAF
KQALMILTAPNSIALASNEDIHLSADGQLNQTAGDSINLSTQKNLIAHAQNKISLFAAQQGARLYAGKGKVE
IQAQGDGADLIARKAVQVISTEDKIEATAAKEIVLTAGGSQVKITGSGIFMTTSGKFEVKAGQHVFMGGGSA
NANVPALP----KLEFAQSPYSAQYQLFKADGRNFQGYKYFIHD-SKNNLIKQGITDNQGFTEQVVTES-KE
RIIGYKSVMRESERITENWEAKLEQVANKMSSVGK-------------------------------------
------------------------------------------------------------------------
-----------------------
>ACICU_7
-MQMSVSSILERLGLVSQNRAVHIQFSNQSLNQQVFLQRIEGEHTLNQGSVAELLCLSTNAHIALKQFIGCQ
VAVDQVTDTGQFFRTTGIITEASQGQSDGSLTIYNLTLKDPTALWHKRRNSRVFMNKSVRDISEILFKEWQG
KSPLFASSITLDTSGLT-KDYDVRPFVMQSNESDYDFLTRLWRSEGINWLVDESQLFVADPNASIQPQVLRL
IDDNQNYQALERRSIRYQRSSATEQFDTITQVKAERRLQPTSVHVQRWQADALQQEEGSGSVQGTQKHSEHY
DNASLNLEDAWHVSPAWMQDLNGEDQATASGNSQIEQLNQHINAYHHLSSKQFTVAGNVRDAQVGYWFELND
HPELDQ-HDSADKEFLILSKHYYNQNNLPKELQQQLERLLPQGKLKAAQLD--SQNPEQRHFAELNIVRRNI
KAVPEYNPLEHRPAAHPQRARVVGLEGESIHVDQWGRIKVRFLFTRADDHSHDGGAGSNDNDTDSAWVDVLT
PWAGAGYGARFLPRVGEIVVIDFFDGNIDRPFVVGRIHEAERHPTQFDQKGQLPDTKKLSGIRSEEVDGKGF
NQLRFDDTTGQISAQLQSSHAASQLNLGNLSYPKDKAESDGRGEGFELRTDQWGAVRAGSGLLISTHKQDQA

QGVHLDANEAKQQIEGGLNNAKALSEVAKNQQTDPLEML---------------------------------
----------------------------------------ENLKTFIEQIEE--------------------
---------------------------------------------------------------KDQDKAAAF
KQALMILTAPNSIALASNEDIHLSADGQLNQTAGDSINLSTQKNLIAHAQNKISLFAAQQGARLYAGKGKVE
IQAQGDGADLIARKAVQVISTEDKIEATAAKEIVLTAGGSQVKITGSGIFMTTSGKFEVKAGQHVFMGGGSA
NANVPALP----KLEFAQSPYSAQYQLFKADGRNFQGYKYFIHD-SKNNLIKQGITDNQGFTEQVVTES-KE
RIIGYKSVMRESERITENWEAKLEQVANKMSSVGK-------------------------------------
------------------------------------------------------------------------
-----------------------

>ATCC19606_7
---MSVSSILERLGLVSQNRAVHIQFSNQSLNQQVFLQRIEGEHTLNQGSVAELLCLSTNAHIALKQFIGCQ
VAVDQVTDTGQFFRTTGIITEASQGQSDGSLTIYNLTLKDPTALWHKRRNSRVFMNKSVRDISEILFKEWQG
KSPLFASSITLDTSGLT-KDYDVRPFVMQSNESDYDFLTRLWRSEGINWLVDESQLFVADPNASIQPQVLRL
IDDNQNYQALERRSIRYQRSSATEQFDTITQVKAERRLQPTSVHVQRWQADALQQEEGSGSVQGTQKHSEHY
DNASLNLEDAWHVSPAWMQDLNGEDQATASGNSQIEQLNQHINAYHHLSSKQFTVAGNVRDAQVGYWFELND
HPELDQ-HDSADKEFLILSKHYYNQNNLPKELQQQLERLLPQGKLKAAQLD--SQNPEQRHFAELNIVRRNI
KAVPEYNPLEHRPAAHPQRARVVGLEGESIHVDQWGRIKVRFLFTRADDHSHDGGAGSNDNDTDSAWVDVLT
PWAGAGYGARFLPRVGEIVVIDFFDGNIDRPFVVGRIHEAERHPTQFDQKGQLPDTKKLSGIRSEEVDGKGF
NQLRFDDTTGQISAQLQSSHAASQLNLGNLSYPKDKAESDGRGEGFELRTDQWGAVRAGSGLLISTHKQDQA
QGVHLDANEAKQQIEGGLNNAKALSEVAKNQQTDPLEML---------------------------------
----------------------------------------ENLKTFIEQIEE--------------------
---------------------------------------------------------------KDQDKAAAF
KQALMILTAPNSIALASNEDIHLSADGQLNQTAGDSINLSTQKNLIAHAQNKISLFAAQQGARLYAGKGKVE
IQAQGDGADLIARKAVQVISTEDKIEATAAKEIVLTAGGSQVKITGSGIFMTTSGKFEVKAGQHVFMGGGSA
NANVPALP----KLEFAQSPYSAQYQLFKADGRNFQGYKYFIHD-SKNNLIKQGITDNQGFTEQVVTES-KE
RIIGYKSVMRESERITENWEAKLEQVANKMSSVGK-------------------------------------
------------------------------------------------------------------------
-----------------------

>ZW85-1_19
-MQMSVSSILERLGLVSQNRAVHIQFSNQSLNQQVFLQRIEGEHTLNQGSVAELLCLSTNAHIALKQFIGCQ
VAVDQVTDTGQFFRTTGIITEASQGQSDGSLTIYNLTLKDPTALWHKRRNSRVFMNKSVRDISEILFKEWQG
KSPLFAASLTLDTSGLT-KDYDVRPFVMQSNESDYDFLTRLWRSESINWLVDESQLFVADPNASIQPQVLRL
IDDNQNYQALERRSIRYQRSSATEQFDTITQVKAERRLQPTSVHVQRWQADALQQEEGSGSVQGTQKHSEHY

DNASLNLEDAWHVSPAWMQDLNGEDQATASGNSQIEQLNQHINAYHHLSSKQFTVAGNVRDAQVGYWFELND
HPELDQ-HDSADKEFLILNKHYYNQNNLPKELQQQLERLLPQGKLKAAQLD--SQNPEQRHFAELNVVRRNI
KAVPEYNPLEHRPAAHPQRARVVGLEGESIHVDQWGRIKVRFLFTRADDHSHDGGAGSNDNDTDSAWVDVLT
PWAGAGYGARFLPRVGEIVVIDFFDGNIDRPFVVGRIHEAERHPTQFDQKGQLPDTKKLSGIRSEEVDGKGF
NQLRFDDTTGQISAQLQSSHAASQLNLGNLSHPKDKAESDGRGEGFELRTDQWGAVRAGSGLLVSTHKQDQA
QGVHLDASEAKQQIEGGLNNAKALSEVAKNQQTDPLEML----------------------------------
---------------------------------------ENLKTFIEQIEE--------------------
--------------------------------------------------------------KDQDKAAAF
KQALMILTAPNSIALASNEDIHLSADGQLNQTAGDSINLSTQKNLIAHAQNKISLFAAQQGARLYAGKGKVE
IQAQGDGADLIARKAVQVISTEDKIEATAAKEIVLTAGGSQVKITGSGIFMTTSGKFEVKAGQHLFMGGGSA
NANVPALP-------EFNKTNWIALEHLDVDNQPFANLGYKIF--FENNQVIEGKLDEQGKAHHENVPD-KA
IRVEYQELPAKPDTPWDTYDAVLKELNSFEEGDIRD------------------------------------
------------------------------------------------------------------------
----------------------

>15A5_28
-MQMSVSSILERLGLVSQNRAVHIQFSNQSLNQQVFLQRIEGEHTLNQGSVAELLCLSTNAHIALKQFIGCQ
VAVDQVTDTGQFFRTTGIITEASQGQSDGSLTIYNLTLKDPTALWHKRRNSRVFMNKSVRDISEILFKEWQE
KSPLFASSITLDTSGLT-KDYDVRPFVMQSNESDYDFLTRLWRSEGINWLVDESQLFVADPNASIQPQVLRL
IDDNQNYQALERRSIRYQRSSATEQFDTITQVKAERRLQPTSVHVQRWQADALQQEEGSGSVQGTQKHSEHY
DNASLNLEDAWHVSPAWMQDLNGEDQATASGNSQIEQLNQHINAYHHLSSKQFTVAGNVRDAQVGYWFELND
HPELDQ-HDSADKEFLILSKHYYNQNNLPKELQQQLERLLPQGKLKAAQLD--SQNPEQRHFAELNIVRRNI
KAVPEYNPLEHRPAAHPQRARVVGLEGESIHVDQWGRIKVRFLFTRTDDHSHDGGAGSNDNDTDSAWVDVLT
PWAGAGYGARFLPRVGEIVVIDFFDGNIDRPFVVGRIHEAERHPTQFDQKGQLPDTKKLSGIRSEEVDGKGF
NQLLFDDTTGQISAQLQSSHAASQLNLGNLSHPKDKAESDGRGEGFELRTDQWGAVRAGSGLLVSTHKQDQA
QGVHLDASEAKQQIEGGLNNAKALSEVAKNQQTDPLEML----------------------------------
---------------------------------------ENLKTFIEQIEE--------------------
--------------------------------------------------------------KDQDKAAAF
KQALMILTAPNSIALASNEDIHLSADGQLNQTAGDSINLSTQKNLIAHAQNKISLFAAQQGARLYAGKGKVE
IQAQGDGADLIARKAVQVISTEDKIEATAAKEIVLTAGGSQVKITGSGIFMTTSGKFEVKAGQHLFMGGGSV
NANVPALP----DLRIKTEHRNFQAIDSSKN-EPLEGIPYSILN-KRTGIKFYGFTNEKGMTKTIKSAE---
-----------------NDELVVNWFEQEDLVNGE-------------------------------------
------------------------------------------------------------------------
----------------------

>Ab4568_28
-MQMSVSSILERLGLVSQNRAVHIQFSNQSLNQQVFLQRIEGEHTLNQGSVAELLCLSTNAHIALKQFIGCQ
VAVDQVTDTGQFFRTTGIITEASQGQSDGSLTIYNLTLKDPTALWHKRRNSRVFMNKSVRDISEILFKEWQE
KSPLFASSITLDTSGLT-KDYDVRPFVMQSNESDYDFLTRLWRSEGINWLVDESQLFVADPNASIQPQVLRL
IDDNQNYQALERRSIRYQRSSATEQFDTITQVKAERRLQPTSVHVQRWQADALQQEEGSGSVQGTQKHSEHY
DNASLNLEDAWHVSPAWMQDLNGEDQATASGNSQIEQLNQHINAYHHLSSKQFTVAGNVRDAQVGYWFELND
HPELDQ-HDSADKEFLILSKHYYNQNNLPKELQQQLERLLPQGKLKAAQLD--SQNPEQRHFAELNIVRRNI
KAVPEYNPLEHRPAAHPQRARVVGLEGESIHVDQWGRIKVRFLFTRTDDHSHDGGAGSNDNDTDSAWVDVLT
PWAGAGYGARFLPRVGEIVVIDFFDGNIDRPFVVGRIHEAERHPTQFDQKGQLPDTKKLSGIRSEEVDGKGF
NQLLFDDTTGQISAQLQSSHAASQLNLGNLSHPKDKAESDGRGEGFELRTDQWGAVRAGSGLLVSTHKQDQA
QGVHLDASEAKQQIEGGLNNAKALSEVAKNQQTDPLEML---------------------------------
----------------------------------------ENLKTFIEQIEE--------------------
---------------------------------------------------------------KDQDKAAAF
KQALMILTAPNSIALASNEDIHLSADGQLNQTAGDSINLSTQKNLIAHAQNKISLFAAQQGARLYAGKGKVE
IQAQGDGADLIARKAVQVISTEDKIEATAAKEIVLTAGGSQVKITGSGIFMTTSGKFEVKAGQHLFMGGGSV
NANVPALP----DLRIKTEHRNFQAIDSSKN-EPLEGIPYSILN-KRTGIKFYGFTNEKGMTKTIKSAE---
-----------------NDELVVNWFEQEDLVNGE-------------------------------------
------------------------------------------------------------------------
-----------------------
>Ab4977_28
-MQMSVSSILERLGLVSQNRAVHIQFSNQSLNQQVFLQRIEGEHTLNQGSVAELLCLSTNAHIALKQFIGCQ
VAVDQVTDTGQFFRTTGIITEASQGQSDGSLTIYNLTLKDPTALWHKRRNSRVFMNKSVRDISEILFKEWQE
KSPLFASSITLDTSGLT-KDYDVRPFVMQSNESDYDFLTRLWRSEGINWLVDESQLFVADPNASIQPQVLRL
IDDNQNYQALERRSIRYQRSSATEQFDTITQVKAERRLQPTSVHVQRWQADALQQEEGSGSVQGTQKHSEHY
DNASLNLEDAWHVSPAWMQDLNGEDQATASGNSQIEQLNQHINAYHHLSSKQFTVAGNVRDAQVGYWFELND
HPELDQ-HDSADKEFLILSKHYYNQNNLPKELQQQLERLLPQGKLKAAQLD--SQNPEQRHFAELNIVRRNI
KAVPEYNPLEHRPAAHPQRARVVGLEGESIHVDQWGRIKVRFLFTRTDDHSHDGGAGSNDNDTDSAWVDVLT
PWAGAGYGARFLPRVGEIVVIDFFDGNIDRPFVVGRIHEAERHPTQFDQKGQLPDTKKLSGIRSEEVDGKGF
NQLLFDDTTGQISAQLQSSHAASQLNLGNLSHPKDKAESDGRGEGFELRTDQWGAVRAGSGLLVSTHKQDQA
QGVHLDASEAKQQIEGGLNNAKALSEVAKNQQTDPLEML---------------------------------
----------------------------------------ENLKTFIEQIEE--------------------
---------------------------------------------------------------KDQDKAAAF
KQALMILTAPNSIALASNEDIHLSADGQLNQTAGDSINLSTQKNLIAHAQNKISLFAAQQGARLYAGKGKVE

IQAQGDGADLIARKAVQVISTEDKIEATAAKEIVLTAGGSQVKITGSGIFMTTSGKFEVKAGQHLFMGGGSV
NANVPALP----DLRIKTEHRNFQAIDSSKN-EPLEGIPYSILN-KRTGIKFYGFTNEKGMTKTIKSAE---
------------------NDELVVNWFEQEDLVNGE----------------------------------------
------------------------------------------------------------------------
-----------------------

>JBA13_28

-MQMSVSSILERLGLVSQNRAVHIQFSNQSLNQQVFLQRIEGEHTLNQGSVAELLCLSTNAHIALKQFIGCQ
VAVDQVTDTGQFFRTTGIITEASQGQSDGSLTIYNLTLKDPTALWHKRRNSRVFMNKSVRDISEILFKEWQE
KSPLFASSITLDTSGLT-KDYDVRPFVMQSNESDYDFLTRLWRSEGINWLVDESQLFVADPNASIQPQVLRL
IDDNQNYQALERRSIRYQRSSATEQFDTITQVKAERRLQPTSVHVQRWQADALQQEEGSGSVQGTQKHSEHY
DNASLNLEDAWHVSPAWMQDLNGEDQATASGNSQIEQLNQHINAYHHLSSKQFTVAGNVRDAQVGYWFELND
HPELDQ-HDSADKEFLILSKHYYNQNNLPKELQQQLERLLPQGKLKAAQLD--SQNPEQRHFAELNIVRRNI
KAVPEYNPLEHRPAAHPQRARVVGLEGESIHVDQWGRIKVRFLFTRTDDHSHDGGAGSNDNDTDSAWVDVLT
PWAGAGYGARFLPRVGEIVVIDFFDGNIDRPFVVGRIHEAERHPTQFDQKGQLPDTKKLSGIRSEEVDGKGF
NQLLFDDTTGQISAQLQSSHAASQLNLGNLSHPKDKAESDGRGEGFELRTDQWGAVRAGSGLLVSTHKQDQA
QGVHLDASEAKQQIEGGLNNAKALSEVAKNQQTDPLEML---------------------------------
----------------------------------------ENLKTFIEQIEE--------------------
----------------------------------------------------------------KDQDKAAAF
KQALMILTAPNSIALASNEDIHLSADGQLNQTAGDSINLSTQKNLIAHAQNKISLFAAQQGARLYAGKGKVE
IQAQGDGADLIARKAVQVISTEDKIEATAAKEIVLTAGGSQVKITGSGIFMTTSGKFEVKAGQHLFMGGGSV
NANVPALP----DLRIKTEHRNFQAIDSSKN-EPLEGIPYSILN-KRTGIKFYGFTNEKGMTKTIKSAE---
------------------NDELVVNWFEQEDLVNGE----------------------------------------
------------------------------------------------------------------------
-----------------------

>KAB04_28

-MQMSVSSILERLGLVSQNRAVHIQFSNQSLNQQVFLQRIEGEHTLNQGSVAELLCLSTNAHIALKQFIGCQ
VAVDQVTDTGQFFRTTGIITEASQGQSDGSLTIYNLTLKDPTALWHKRRNSRVFMNKSVRDISEILFKEWQE
KSPLFASSITLDTSGLT-KDYDVRPFVMQSNESDYDFLTRLWRSEGINWLVDESQLFVADPNASIQPQVLRL
IDDNQNYQALERRSIRYQRSSATEQFDTITQVKAERRLQPTSVHVQRWQADALQQEEGSGSVQGTQKHSEHY
DNASLNLEDAWHVSPAWMQDLNGEDQATASGNSQIEQLNQHINAYHHLSSKQFTVAGNVRDAQVGYWFELND
HPELDQ-HDSADKEFLILSKHYYNQNNLPKELQQQLERLLPQGKLKAAQLD--SQNPEQRHFAELNIVRRNI
KAVPEYNPLEHRPAAHPQRARVVGLEGESIHVDQWGRIKVRFLFTRTDDHSHDGGAGSNDNDTDSAWVDVLT
PWAGAGYGARFLPRVGEIVVIDFFDGNIDRPFVVGRIHEAERHPTQFDQKGQLPDTKKLSGIRSEEVDGKGF

NQLLFDDTTGQISAQLQSSHAASQLNLGNLSHPKDKAESDGRGEGFELRTDQWGAVRAGSGLLVSTHKQDQA
QGVHLDASEAKQQIEGGLNNAKALSEVAKNQQTDPLEML---------------------------------
----------------------------------------ENLKTFIEQIEE--------------------
---------------------------------------------------------------KDQDKAAAF
KQALMILTAPNSIALASNEDIHLSADGQLNQTAGDSINLSTQKNLIAHAQNKISLFAAQQGARLYAGKGKVE
IQAQGDGADLIARKAVQVISTEDKIEATAAKEIVLTAGGSQVKITGSGIFMTTSGKFEVKAGQHLFMGGGSV
NANVPALP----DLRIKTEHRNFQAIDSSKN-EPLEGIPYSILN-KRTGIKFYGFTNEKGMTKTIKSAE---
-----------------NDELVVNWFEQEDLVNGE-------------------------------------
------------------------------------------------------------------------
-----------------------
>KAB07_28
-MQMSVSSILERLGLVSQNRAVHIQFSNQSLNQQVFLQRIEGEHTLNQGSVAELLCLSTNAHIALKQFIGCQ
VAVDQVTDTGQFFRTTGIITEASQGQSDGSLTIYNLTLKDPTALWHKRRNSRVFMNKSVRDISEILFKEWQE
KSPLFASSITLDTSGLT-KDYDVRPFVMQSNESDYDFLTRLWRSEGINWLVDESQLFVADPNASIQPQVLRL
IDDNQNYQALERRSIRYQRSSATEQFDTITQVKAERRLQPTSVHVQRWQADALQQEEGSGSVQGTQKHSEHY
DNASLNLEDAWHVSPAWMQDLNGEDQATASGNSQIEQLNQHINAYHHLSSKQFTVAGNVRDAQVGYWFELND
HPELDQ-HDSADKEFLILSKHYYNQNNLPKELQQQLERLLPQGKLKAAQLD--SQNPEQRHFAELNIVRRNI
KAVPEYNPLEHRPAAHPQRARVVGLEGESIHVDQWGRIKVRFLFTRTDDHSHDGGAGSNDNDTDSAWVDVLT
PWAGAGYGARFLPRVGEIVVIDFFDGNIDRPFVVGRIHEAERHPTQFDQKGQLPDTKKLSGIRSEEVDGKGF
NQLLFDDTTGQISAQLQSSHAASQLNLGNLSHPKDKAESDGRGEGFELRTDQWGAVRAGSGLLVSTHKQDQA
QGVHLDASEAKQQIEGGLNNAKALSEVAKNQQTDPLEML---------------------------------
----------------------------------------ENLKTFIEQIEE--------------------
---------------------------------------------------------------KDQDKAAAF
KQALMILTAPNSIALASNEDIHLSADGQLNQTAGDSINLSTQKNLIAHAQNKISLFAAQQGARLYAGKGKVE
IQAQGDGADLIARKAVQVISTEDKIEATAAKEIVLTAGGSQVKITGSGIFMTTSGKFEVKAGQHLFMGGGSV
NANVPALP----DLRIKTEHRNFQAIDSSKN-EPLEGIPYSILN-KRTGIKFYGFTNEKGMTKTIKSAE---
-----------------NDELVVNWFEQEDLVNGE-------------------------------------
------------------------------------------------------------------------
-----------------------
>KAB08_28
-MQMSVSSILERLGLVSQNRAVHIQFSNQSLNQQVFLQRIEGEHTLNQGSVAELLCLSTNAHIALKQFIGCQ
VAVDQVTDTGQFFRTTGIITEASQGQSDGSLTIYNLTLKDPTALWHKRRNSRVFMNKSVRDISEILFKEWQE
KSPLFASSITLDTSGLT-KDYDVRPFVMQSNESDYDFLTRLWRSEGINWLVDESQLFVADPNASIQPQVLRL

IDDNQNYQALERRSIRYQRSSATEQFDTITQVKAERRLQPTSVHVQRWQADALQQEEGSGSVQGTQKHSEHY
DNASLNLEDAWHVSPAWMQDLNGEDQATASGNSQIEQLNQHINAYHHLSSKQFTVAGNVRDAQVGYWFELND
HPELDQ-HDSADKEFLILSKHYYNQNNLPKELQQQLERLLPQGKLKAAQLD--SQNPEQRHFAELNIVRRNI
KAVPEYNPLEHRPAAHPQRARVVGLEGESIHVDQWGRIKVRFLFTRTDDHSHDGGAGSNDNDTDSAWVDVLT
PWAGAGYGARFLPRVGEIVVIDFFDGNIDRPFVVGRIHEAERHPTQFDQKGQLPDTKKLSGIRSEEVDGKGF
NQLLFDDTTGQISAQLQSSHAASQLNLGNLSHPKDKAESDGRGEGFELRTDQWGAVRAGSGLLVSTHKQDQA
QGVHLDASEAKQQIEGGLNNAKALSEVAKNQQTDPLEML---------------------------------
----------------------------------------ENLKTFIEQIEE--------------------
---------------------------------------------------------------KDQDKAAAF
KQALMILTAPNSIALASNEDIHLSADGQLNQTAGDSINLSTQKNLIAHAQNKISLFAAQQGARLYAGKGKVE
IQAQGDGADLIARKAVQVISTEDKIEATAAKEIVLTAGGSQVKITGSGIFMTTSGKFEVKAGQHLFMGGGSV
NANVPALP----DLRIKTEHRNFQAIDSSKN-EPLEGIPYSILN-KRTGIKFYGFTNEKGMTKTIKSAE---
-----------------NDELVVNWFEQEDLVNGE-------------------------------------
------------------------------------------------------------------------
-----------------------

>KBN10P02143_28
-MQMSVSSILERLGLVSQNRAVHIQFSNQSLNQQVFLQRIEGEHTLNQGSVAELLCLSTNAHIALKQFIGCQ
VAVDQVTDTGQFFRTTGIITEASQGQSDGSLTIYNLTLKDPTALWHKRRNSRVFMNKSVRDISEILFKEWQE
KSPLFASSITLDTSGLT-KDYDVRPFVMQSNESDYDFLTRLWRSEGINWLVDESQLFVADPNASIQPQVLRL
IDDNQNYQALERRSIRYQRSSATEQFDTITQVKAERRLQPTSVHVQRWQADALQQEEGSGSVQGTQKHSEHY
DNASLNLEDAWHVSPAWMQDLNGEDQATASGNSQIEQLNQHINAYHHLSSKQFTVAGNVRDAQVGYWFELND
HPELDQ-HDSADKEFLILSKHYYNQNNLPKELQQQLERLLPQGKLKAAQLD--SQNPEQRHFAELNIVRRNI
KAVPEYNPLEHRPAAHPQRARVVGLEGESIHVDQWGRIKVRFLFTRTDDHSHDGGAGSNDNDTDSAWVDVLT
PWAGAGYGARFLPRVGEIVVIDFFDGNIDRPFVVGRIHEAERHPTQFDQKGQLPDTKKLSGIRSEEVDGKGF
NQLLFDDTTGQISAQLQSSHAASQLNLGNLSHPKDKAESDGRGEGFELRTDQWGAVRAGSGLLVSTHKQDQA
QGVHLDASEAKQQIEGGLNNAKALSEVAKNQQTDPLEML---------------------------------
----------------------------------------ENLKTFIEQIEE--------------------
---------------------------------------------------------------KDQDKAAAF
KQALMILTAPNSIALASNEDIHLSADGQLNQTAGDSINLSTQKNLIAHAQNKISLFAAQQGARLYAGKGKVE
IQAQGDGADLIARKAVQVISTEDKIEATAAKEIVLTAGGSQVKITGSGIFMTTSGKFEVKAGQHLFMGGGSV
NANVPALP----DLRIKTEHRNFQAIDSSKN-EPLEGIPYSILN-KRTGIKFYGFTNEKGMTKTIKSAE---
-----------------NDELVVNWFEQEDLVNGE-------------------------------------
------------------------------------------------------------------------

```
-----------------------
>SAA14_28
-MQMSVSSILERLGLVSQNRAVHIQFSNQSLNQQVFLQRIEGEHTLNQGSVAELLCLSTNAHIALKQFIGCQ
VAVDQVTDTGQFFRTTGIITEASQGQSDGSLTIYNLTLKDPTALWHKRRNSRVFMNKSVRDISEILFKEWQE
KSPLFASSITLDTSGLT-KDYDVRPFVMQSNESDYDFLTRLWRSEGINWLVDESQLFVADPNASIQPQVLRL
IDDNQNYQALERRSIRYQRSSATEQFDTITQVKAERRLQPTSVHVQRWQADALQQEEGSGSVQGTQKHSEHY
DNASLNLEDAWHVSPAWMQDLNGEDQATASGNSQIEQLNQHINAYHHLSSKQFTVAGNVRDAQVGYWFELND
HPELDQ-HDSADKEFLILSKHYYNQNNLPKELQQQLERLLPQGKLKAAQLD--SQNPEQRHFAELNIVRRNI
KAVPEYNPLEHRPAAHPQRARVVGLEGESIHVDQWGRIKVRFLFTRTDDHSHDGGAGSNDNDTDSAWVDVLT
PWAGAGYGARFLPRVGEIVVIDFFDGNIDRPFVVGRIHEAERHPTQFDQKGQLPDTKKLSGIRSEEVDGKGF
NQLLFDDTTGQISAQLQSSHAASQLNLGNLSHPKDKAESDGRGEGFELRTDQWGAVRAGSGLLVSTHKQDQA
QGVHLDASEAKQQIEGGLNNAKALSEVAKNQQTDPLEML---------------------------------
----------------------------------------ENLKTFIEQIEE--------------------
---------------------------------------------------------------KDQDKAAAF
KQALMILTAPNSIALASNEDIHLSADGQLNQTAGDSINLSTQKNLIAHAQNKISLFAAQQGARLYAGKGKVE
IQAQGDGADLIARKAVQVISTEDKIEATAAKEIVLTAGGSQVKITGSGIFMTTSGKFEVKAGQHLFMGGGSV
NANVPALP----DLRIKTEHRNFQAIDSSKN-EPLEGIPYSILN-KRTGIKFYGFTNEKGMTKTIKSAE---
-----------------NDELVVNWFEQEDLVNGE-------------------------------------
------------------------------------------------------------------------
-----------------------
>SMC_Paed_Ab_BL01_28
-MQMSVSSILERLGLVSQNRAVHIQFSNQSLNQQVFLQRIEGEHTLNQGSVAELLCLSTNAHIALKQFIGCQ
VAVDQVTDTGQFFRTTGIITEASQGQSDGSLTIYNLTLKDPTALWHKRRNSRVFMNKSVRDISEILFKEWQE
KSPLFASSITLDTSGLT-KDYDVRPFVMQSNESDYDFLTRLWRSEGINWLVDESQLFVADPNASIQPQVLRL
IDDNQNYQALERRSIRYQRSSATEQFDTITQVKAERRLQPTSVHVQRWQADALQQEEGSGSVQGTQKHSEHY
DNASLNLEDAWHVSPAWMQDLNGEDQATASGNSQIEQLNQHINAYHHLSSKQFTVAGNVRDAQVGYWFELND
HPELDQ-HDSADKEFLILSKHYYNQNNLPKELQQQLERLLPQGKLKAAQLD--SQNPEQRHFAELNIVRRNI
KAVPEYNPLEHRPAAHPQRARVVGLEGESIHVDQWGRIKVRFLFTRTDDHSHDGGAGSNDNDTDSAWVDVLT
PWAGAGYGARFLPRVGEIVVIDFFDGNIDRPFVVGRIHEAERHPTQFDQKGQLPDTKKLSGIRSEEVDGKGF
NQLLFDDTTGQISAQLQSSHAASQLNLGNLSHPKDKAESDGRGEGFELRTDQWGAVRAGSGLLVSTHKQDQA
QGVHLDASEAKQQIEGGLNNAKALSEVAKNQQTDPLEML---------------------------------
----------------------------------------ENLKTFIEQIEE--------------------
---------------------------------------------------------------KDQDKAAAF
```

KQALMILTAPNSIALASNEDIHLSADGQLNQTAGDSINLSTQKNLIAHAQNKISLFAAQQGARLYAGKGKVE
IQAQGDGADLIARKAVQVISTEDKIEATAAKEIVLTAGGSQVKITGSGIFMTTSGKFEVKAGQHLFMGGGSV
NANVPALP----DLRIKTEHRNFQAIDSSKN-EPLEGIPYSILN-KRTGIKFYGFTNEKGMTKTIKSAE---
-----------------NDELVVNWFEQEDLVNGE-------------------------------------
------------------------------------------------------------------------
-----------------------
>SSA12_28
-MQMSVSSILERLGLVSQNRAVHIQFSNQSLNQQVFLQRIEGEHTLNQGSVAELLCLSTNAHIALKQFIGCQ
VAVDQVTDTGQFFRTTGIITEASQGQSDGSLTIYNLTLKDPTALWHKRRNSRVFMNKSVRDISEILFKEWQE
KSPLFASSITLDTSGLT-KDYDVRPFVMQSNESDYDFLTRLWRSEGINWLVDESQLFVADPNASIQPQVLRL
IDDNQNYQALERRSIRYQRSSATEQFDTITQVKAERRLQPTSVHVQRWQADALQQEEGSGSVQGTQKHSEHY
DNASLNLEDAWHVSPAWMQDLNGEDQATASGNSQIEQLNQHINAYHHLSSKQFTVAGNVRDAQVGYWFELND
HPELDQ-HDSADKEFLILSKHYYNQNNLPKELQQQLERLLPQGKLKAAQLD--SQNPEQRHFAELNIVRRNI
KAVPEYNPLEHRPAAHPQRARVVGLEGESIHVDQWGRIKVRFLFTRTDDHSHDGGAGSNDNDTDSAWVDVLT
PWAGAGYGARFLPRVGEIVVIDFFDGNIDRPFVVGRIHEAERHPTQFDQKGQLPDTKKLSGIRSEEVDGKGF
NQLLFDDTTGQISAQLQSSHAASQLNLGNLSHPKDKAESDGRGEGFELRTDQWGAVRAGSGLLVSTHKQDQA
QGVHLDASEAKQQIEGGLNNAKALSEVAKNQQTDPLEML---------------------------------
----------------------------------------ENLKTFIEQIEE--------------------
----------------------------------------------------------------KDQDKAAAF
KQALMILTAPNSIALASNEDIHLSADGQLNQTAGDSINLSTQKNLIAHAQNKISLFAAQQGARLYAGKGKVE
IQAQGDGADLIARKAVQVISTEDKIEATAAKEIVLTAGGSQVKITGSGIFMTTSGKFEVKAGQHLFMGGGSV
NANVPALP----DLRIKTEHRNFQAIDSSKN-EPLEGIPYSILN-KRTGIKFYGFTNEKGMTKTIKSAE---
-----------------NDELVVNWFEQEDLVNGE-------------------------------------
------------------------------------------------------------------------
-----------------------
>SSMA17_28
-MQMSVSSILERLGLVSQNRAVHIQFSNQSLNQQVFLQRIEGEHTLNQGSVAELLCLSTNAHIALKQFIGCQ
VAVDQVTDTGQFFRTTGIITEASQGQSDGSLTIYNLTLKDPTALWHKRRNSRVFMNKSVRDISEILFKEWQE
KSPLFASSITLDTSGLT-KDYDVRPFVMQSNESDYDFLTRLWRSEGINWLVDESQLFVADPNASIQPQVLRL
IDDNQNYQALERRSIRYQRSSATEQFDTITQVKAERRLQPTSVHVQRWQADALQQEEGSGSVQGTQKHSEHY
DNASLNLEDAWHVSPAWMQDLNGEDQATASGNSQIEQLNQHINAYHHLSSKQFTVAGNVRDAQVGYWFELND
HPELDQ-HDSADKEFLILSKHYYNQNNLPKELQQQLERLLPQGKLKAAQLD--SQNPEQRHFAELNIVRRNI
KAVPEYNPLEHRPAAHPQRARVVGLEGESIHVDQWGRIKVRFLFTRTDDHSHDGGAGSNDNDTDSAWVDVLT

PWAGAGYGARFLPRVGEIVVIDFFDGNIDRPFVVGRIHEAERHPTQFDQKGQLPDTKKLSGIRSEEVDGKGF
NQLLFDDTTGQISAQLQSSHAASQLNLGNLSHPKDKAESDGRGEGFELRTDQWGAVRAGSGLLVSTHKQDQA
QGVHLDASEAKQQIEGGLNNAKALSEVAKNQQTDPLEML------------------------------------
----------------------------------------ENLKTFIEQIEE--------------------
---------------------------------------------------------------KDQDKAAAF
KQALMILTAPNSIALASNEDIHLSADGQLNQTAGDSINLSTQKNLIAHAQNKISLFAAQQGARLYAGKGKVE
IQAQGDGADLIARKAVQVISTEDKIEATAAKEIVLTAGGSQVKITGSGIFMTTSGKFEVKAGQHLFMGGGSV
NANVPALP----DLRIKTEHRNFQAIDSSKN-EPLEGIPYSILN-KRTGIKFYGFTNEKGMTKTIKSAE---
-----------------NDELVVNWFEQEDLVNGE-------------------------------------
------------------------------------------------------------------------
-----------------------

>YU-R612_28
-MQMSVSSILERLGLVSQNRAVHIQFSNQSLNQQVFLQRIEGEHTLNQGSVAELLCLSTNAHIALKQFIGCQ
VAVDQVTDTGQFFRTTGIITEASQGQSDGSLTIYNLTLKDPTALWHKRRNSRVFMNKSVRDISEILFKEWQE
KSPLFASSITLDTSGLT-KDYDVRPFVMQSNESDYDFLTRLWRSEGINWLVDESQLFVADPNASIQPQVLRL
IDDNQNYQALERRSIRYQRSSATEQFDTITQVKAERRLQPTSVHVQRWQADALQQEEGSGSVQGTQKHSEHY
DNASLNLEDAWHVSPAWMQDLNGEDQATASGNSQIEQLNQHINAYHHLSSKQFTVAGNVRDAQVGYWFELND
HPELDQ-HDSADKEFLILSKHYYNQNNLPKELQQQLERLLPQGKLKAAQLD--SQNPEQRHFAELNIVRRNI
KAVPEYNPLEHRPAAHPQRARVVGLEGESIHVDQWGRIKVRFLFTRTDDHSHDGGAGSNDNDTDSAWVDVLT
PWAGAGYGARFLPRVGEIVVIDFFDGNIDRPFVVGRIHEAERHPTQFDQKGQLPDTKKLSGIRSEEVDGKGF
NQLLFDDTTGQISAQLQSSHAASQLNLGNLSHPKDKAESDGRGEGFELRTDQWGAVRAGSGLLVSTHKQDQA
QGVHLDASEAKQQIEGGLNNAKALSEVAKNQQTDPLEML------------------------------------
----------------------------------------ENLKTFIEQIEE--------------------
---------------------------------------------------------------KDQDKAAAF
KQALMILTAPNSIALASNEDIHLSADGQLNQTAGDSINLSTQKNLIAHAQNKISLFAAQQGARLYAGKGKVE
IQAQGDGADLIARKAVQVISTEDKIEATAAKEIVLTAGGSQVKITGSGIFMTTSGKFEVKAGQHLFMGGGSV
NANVPALP----DLRIKTEHRNFQAIDSSKN-EPLEGIPYSILN-KRTGIKFYGFTNEKGMTKTIKSAE---
-----------------NDELVVNWFEQEDLVNGE-------------------------------------
------------------------------------------------------------------------
-----------------------

>BJAB0715_13
-MQMSVSSILERLGLVSQNRAVHIQFSNQSLNQQVFLQRIEGEHTLNQGSGAELLCLSTNAHIALKQFIGCQ
VAVDQVTDTGQFFRTTGIITEASQGQSDGSLTIYNLTLKDPTALWHKRRNSRVFMNKSVRDISEILFKEWQE

KSPLFASSITFDTSGLS-KDYDVRPFVMQSNESDYDFLTRLWRSEGINWLVDESRLFVADPNASIQSQVLRL
IDDNQNYQALERRSIRYQRSSATEQFDTITQVKAERHLQPTSVHVQRWQADALQQEEGSGSVQGTQKHSEHY
DNASLNLEDAWHVSPAWMQDLNGEDQATASGNSQIEQLNQHINAYHHLSSKQFTVAGNVRDAQVGYWFELNE
HPELDQ-HDSADKEFLILSKHYYNQNNLPKELQQQLERLLPQGKLKAAQLD--SQNPEQRHFAELNVVRRNI
KAVPEYNPLEHRPAAHPQRARVVGLEGESIHVDQWGRIKVRFLFTRVDDHSHDGGAGSNDNDTDSAWVDVLT
PWAGAGYGARFLPRVGEIVVIDFFDGNIDRPFVVGRIHEAERHPTQFDQKGQLPDTKKLSGIRSEEVDGKGF
NQLRFDDTTGQISAQLQSSHAASQLNLGNLSHPKDKAESDGRGEGFELRTDQWGAVRAGSGLLVSTHKQDQA
QGVHLDASEAKQQIEGGLNNAKALSEVAKNQQTDPLEML------------------------------------
----------------------------------------ENLKTFIEQIEE--------------------
------------------------------------------------------------------NDQDKAAAF
KQALMILTAPNSIALASNEDIHLSADGQLNQTAGDSINLSTQKNLIAHAQNKISLFAAQQGARLYAGKGKVE
IQAQGDGADLIARKAVQVISTEDKIEATAAKEIVLTAGGSQVKITGSGIFMTTSGKFEVKAGQHVFMGGAEV
GMNLQGLP--------AYEAYNERFKMLLPSGEPLSFIDYKI---SSEGKEIFANSDKKGQTKEIHSPK---
---EQELKLDLLWLDLETVDETEVWVDPAQGSKNV-------------------------------------
------------------------------------------------------------------------
-----------------------
>D1279779_13
-MQMSVSSILERLGLVSQNRAVHIQFSNQSLNQQVFLQRIEGEHTLNQGSVAELLCLSTNAHIALKQFIGCQ
VAVDQVTDTGQFFRTTGIITEASQGQSDGSLTIYNLTLKDPTALWHKRRNSRVFMNKSVRDISEILFKEWQG
KSPLFASSITLDTSGLT-KDYDVRPFVMQSNESDYDFLTRLWRSEGINWLVDESQLFVADPNASIQPQVLRL
IDDNQNYQALERRSIRYQRSSATEQFDTITQVKAERRLQPTSVHVQRWQADALQQEEGSGSVQGTQKHSEHY
DNASLNLEDAWHVSPAWMQDLNGEDQATASGNSQIEQLNQHINAYHHLSSKQFTVAGNVRDAQVGYWFELND
HPELDQ-HDSADKEFLILSKHYYNQNNLPKELQQQLERLLPQGKLKAAQLD--SQNPEQRHFAELNVVRRNI
KAVPEYNPLEHRPAAHPQRARVVGLEGESIHVDQWGRIKVRFLFTRADDHSHDGGAGSNDNDTDSAWVDVLT
PWAGAGYGARFLPRVGEIVVIDFFDGNIDRPFVVGRIHEAERHPTQFDQKGQLPDTKKLSGIRSEEVDGKGF
NQLRFDDTTGQISAQLQSSHAASQLNLGNLSHPKDKAESDGRGEGFELRTDQWGAVRAGSGLLISTHKQDQA
QGVHLDASEAKQQIEGGLNNAKALSEVAKNQQTDPLEML------------------------------------
----------------------------------------ENLKTFIEQIEE--------------------
------------------------------------------------------------------KDQDKAAAF
KQALMILTAPNSIALASNEDIHLSADGQLNQTAGDSINLSTQKNLIAHAQNKISLFAAQQGARLYAGKGKVE
IQAQGDGADLIARKAVQVISTEDKIEATAAKEIVLTAGGSQVKITGSGIFMTTSGKFEVKAGQHLFMGGAEV
GMNLQGLP--------AYEAYNERFKMLLPSGEPLSFIDYKI---SSEGKEIFANSDKKGQTKEIHSPK---
---EQELKLDLLWLDLETVDETEVWVDPAQGSKNV-------------------------------------

------------------------------------------------------------------------
-----------------------
>MDR-TJ_13
-MQMSVSSILERLGLVSQNRAVHIQFSNQSLNQQVFLQRIEGEHTLNQGSVAELLCLSTNAHIALKQFIGCQ
VAVDQVTDTGQFFRTTGIITEASQGQSDGSLTIYNLTLKDPTALWHKRRNSRVFMNKSVRDISEILFKEWQG
KSPLFASSITLDTSGLT-KDYDVRPFVMQSNESDYDFLTRLWRSEGINWLVDESQLFVADPNASIQPQVLRL
IDDNQNYQALERRSIRYQRSSATEQFDTITQVKAERRLQPTSVHVQRWQADALQQEEGSGSVQGTQKHSEHY
DNASLNLEDAWHVSPAWMQDLNGEDQATASGNSQIEQLNQHINAYHHLSSKQFTVAGNVRDAQVGYWFELND
HPELDQ-HDSADKEFLILSKHYYNQNNLPKELQQQLERLLPQGKLKAAQLD--SQNPEQRHFAELNVVRRNI
KAVPEYNPLEHRPAAHPQRARVVGLEGESIHVDQWGRIKVRFLFTRADDHSHDGGAGSNDNDTDSAWVDVLT
PWAGAGYGARFLPRVGEIVVIDFFDGNIDRPFVVGRIHEAERHPTQFDQKGQLPDTKKLSGIRSEEVDGKGF
NQLRFDDTTGQISAQLQSSHAASQLNLGNLSHPKDKAESDGRGEGFELRTDQWGAVRAGSGLLISTHKQDQA
QGVHLDASEAKQQIEGGLNNAKALSEVAKNQQTDPLEML---------------------------------
----------------------------------------ENLKTFIEQIEE--------------------
---------------------------------------------------------------KDQDKAAAF
KQALMILTAPNSIALASNEDIHLSADGQLNQTAGDSINLSTQKNLIAHAQNKISLFAAQQGARLYAGKGKVE
IQAQGDGADLIARKAVQVISTEDKIEATAAKEIVLTAGGSQVKITGSGIFMTTSGKFEVKAGQHLFMGGAEV
GMNLQGLP--------AYEAYNERFKMLLPSGEPLSFIDYKI---SSEGKEIFANSDKKGQTKEIHSPK---
---EQELKLDLLWLDLETVDETEVWVDPAQGSKNV-------------------------------------
------------------------------------------------------------------------
-----------------------
>R2090_13
-MQMSVSSILERLGLVSQNRAVHIQFSNQSLNQQVFLQRIEGEHTLNQGSVAELLCLSTNAHIALKQFIGCQ
VAVDQVTDTGQFFRTTGIITEASQGQSDGSLTIYNLTLKDPTALWHKRRNSRVFMNKSVRDISEILFKEWQG
KSPLFASSITLDTSGLT-KDYDVRPFVMQSNESDYDFLTRLWRSEGINWLVDESQLFVADPNASIQPQVLRL
IDDNQNYQALERRSIRYQRSSATEQFDTITQVKAERRLQPTSVHVQRWQADALQQEEGSGSVQGTQKHSEHY
DNASLNLEDAWHVSPAWMQDLNGEDQATASGNSQIEQLNQHINAYHHLSSKQFTVAGNVRDAQVGYWFELND
HPELDQ-HDSADKEFLILSKHYYNQNNLPKELQQQLERLLPQGKLKAAQLD--SQNPEQRHFAELNVVRRNI
KAVPEYNPLEHRPAAHPQRARVVGLEGESIHVDQWGRIKVRFLFTRADDHSHDGGAGSNDNDTDSAWVDVLT
PWAGAGYGARFLPRVGEIVVIDFFDGNIDRPFVVGRIHEAERHPTQFDQKGQLPDTKKLSGIRSEEVDGKGF
NQLRFDDTTGQISAQLQSSHAASQLNLGNLSHPKDKAESDGRGEGFELRTDQWGAVRAGSGLLISTHKQDQA
QGVHLDASEAKQQIEGGLNNAKALSEVAKNQQTDPLEML---------------------------------
----------------------------------------ENLKTFIEQIEE--------------------

---------------------------------------------------------------KDQDKAAAF
KQALMILTAPNSIALASNEDIHLSADGQLNQTAGDSINLSTQKNLIAHAQNKISLFAAQQGARLYAGKGKVE
IQAQGDGADLIARKAVQVISTEDKIEATAAKEIVLTAGGSQVKITGSGIFMTTSGKFEVKAGQHLFMGGAEV
GMNLQGLP--------AYEAYNERFKMLLPSGEPLSFIDYKI---SSEGKEIFANSDKKGQTKEIHSPK---
---EQELKLDLLWLDLETVDETEVWVDPAQGSKNV-------------------------------------
------------------------------------------------------------------------
-----------------------
>XH858_13
-MQMSVSSILERLGLVSQNRAVHIQFSNQSLNQQVFLQRIEGEHTLNQGSGAELLCLSTNAHIALKQFIGCQ
VAVDQVTDTGQFFRTTGIITEASQGQSDGSLTIYNLTLKDPTALWHKRRNSRVFMNKSVRDISEILFKEWQG
KSPLFASSITLDTSGLT-KDYDVRPFVMQSNESDYDFLTRLWRSEGINWLVDESQLFVADPNASIQPQVLRL
IDDNQNYQALERRSIRYQRSSATEQFDTITQVKAERRLQPTSVHVQRWQADALQQEEGSGSVQGTQKHSEHY
DNASLNLEDAWHVSPAWMQDLNGEDQATASGNSQIEQLNQHINAYHHLSSKQFTVAGNVRDAQVGYWFELNE
HPELDQ-HDSADKEFLILSKHYYNQNNLPKELQQQLERLLPQGKLKAAQLD--SQNPEQRHFAELNVVRRNI
KAVPEYNPLEHRPAAHPQRARVVGLEGESIHVDQWGRIKVRFLFTRADDHSHDGGAGSNDNDTDSAWVDVLT
PWAGAGYGARFLPRVGEIVVIDFFDGNIDRPFVVGRIHEAERHPTQFDQKGQLPDTKKLSGIRSEEVDGKGF
NQLRFDDTTGQISAQLQSSHAASQLNLGNLSHPKDKAESDGRGEGFELRTDQWGAVRAGSGLLVSTHKQDQA
QGVHLDASEAKQQIEGGLNNAKALSEVAKNQQTDPLEML---------------------------------
----------------------------------------ENLKTFIEQIEE--------------------
---------------------------------------------------------------NDQDKAAAF
KQALMILTAPNSIALASNEDIHLSADGQLNQTAGDSINLSTQKNLIAHAQNKISLFAAQQGARLYAGKGKVE
IQAQGDGADLIARKAVQVISTEDKIEATAAKEIVLTAGGSQVKITGSGIFMTTSGKFEVKAGQHVFMGGAEV
GMNLQGLP--------AYEAYNERFKMLLPSGEPLSFIDYKI---SSEGKEIFANSDKKGQTKEIHSPK---
---EQELKLDLLWLDLETVDETEVWVDPAQGSKNV-------------------------------------
------------------------------------------------------------------------
-----------------------
>Ab04-mff_13
-MQMSVSSILERLGLVSQNRAVHIQFSNQSLNQQVFLQRIEGEHTLNQGSVAELLCLSTNAHIALKQFIGCQ
VAVDQVTDTGQFFRTTGIITEASQGQSDGSLTIYNLTLKDPTALWHKRRNSRVFMNKSVRDISEILFKEWQG
KSPLFASSITLDTSGLT-KDYDVRPFVMQSNESDYDFLTRLWRSEGINWLVDESQLFVADPNASIQPQVLRL
IDDNQNYQALERRSIRYQRSSATEQFDTITQVKAERRLQPTSVHVQRWQADALQQEEGSGSVQGTQKHSEHY
DNASLNLEDAWHVSPAWMQDLNGEDQATASGNSQIEQLNQHINAYHHLSSKQFTVAGNVRDAQVGYWFELNE
HPELDQ-HDSADKEFLILSKHYYNQNNLPKELQQQLERLLPQGKLKAAQLD--SQNPEQRHFAELNVVRRNI

KAVPEYNPLEHRPAAHPQRARVVGLEGESIHVDQWGRIKVRFLFTRADDHSHDGGAGSNDNDTDSAWVDVLT
PWAGAGYGARFLPRVGEIVVIDFFDGNIDRPFVVGRIHEAERHPTQFDQKGQLPDTKKLSGIRSEEVDGKGF
NQLRFDDTTGQISAQLQSSHAASQLNLGNLSHPKDKAESDGRGEGFELRTDQWGAVRAGSGLLVSTHKQDQA
QGVHLDASEAKQQIEGGLNNAKALSEVAKNQQTDPLEML---------------------------------
----------------------------------------ENLKTFIEQIEE--------------------
---------------------------------------------------------------NDQDKAAAF
KQALMILTAPNSIALASNEDIHLSADGQLNQTAGDSINLSTQKNLIAHAQNKISLFAAQQGARLYAGKGKVE
IQAQGDGADLIARKAVQVISTEDKIEATAAKEIVLTAGGSQVKITGSGIFMTTSGKFEVKAGQHVFMGGAEV
GMNLQGLP--------AYEAYNERFKMLLPSGEPLSFIDYKI---SSEGKEIFANSDKKGQTKEIHSPK---
---EQELKLDLLWLDLETVDETEVWVDPAQGSKNV-------------------------------------
------------------------------------------------------------------------
-----------------------
>KAB02_13
-MQMSVSSILERLGLVSQNRAVHIQFSNQSLNQQVFLQRIEGEHTLNQGSVAELLCLSTNAHIALKQFIGCQ
VAVDQVTDTGQFFRTTGIITEASQGQSDGSLTIYNLTLKDPTALWHKRRNSRVFMNKSVRDISEILFKEWQG
KSPLFASSITLDTSGLT-KDYDVRPFVMQSNESDYDFLTRLWRSEGINWLVDESQLFVADPNASIQPQVLRL
IDDNQNYQALERRSIRYQRSSATEQFDTITQVKAERRLQPTSVHVQRWQADALQQEEGSGSVQGTQKHSEHY
DNASLNLEDAWHVSPAWMQDLNGEDQATASGNSQIEQLNQHINAYHHLSSKQFTVAGNVRDAQVGYWFELNE
HPELDQ-HDSADKEFLILSKHYYNQNNLPKELQQQLERLLPQGKLKAAQLD--SQNPEQRHFAELNVVRRNI
KAVPEYNPLEHRPAAHPQRARVVGLEGESIHVDQWGRIKVRFLFTRADDHSHDGGAGSNDNDTDSAWVDVLT
PWAGAGYGARFLPRVGEIVVIDFFDGNIDRPFVVGRIHEAERHPTQFDQKGQLPDTKKLSGIRSEEVDGKGF
NQLRFDDTTGQISAQLQSSHAASQLNLGNLSHPKDKAESDGRGEGFELRTDQWGAVRAGSGLLVSTHKQDQA
QGVHLDASEAKQQIEGGLNNAKALSEVAKNQQTDPLEML---------------------------------
----------------------------------------ENLKTFIEQIEE--------------------
---------------------------------------------------------------NDQDKAAAF
KQALMILTAPNSIALASNEDIHLSADGQLNQTAGDSINLSTQKNLIAHAQNKISLFAAQQGARLYAGKGKVE
IQAQGDGADLIARKAVQVISTEDKIEATAAKEIVLTAGGSQVKITGSGIFMTTSGKFEVKAGQHVFMGGAEV
GMNLQGLP--------AYEAYNERFKMLLPSGEPLSFIDYKI---SSEGKEIFANSDKKGQTKEIHSPK---
---EQELKLDLLWLDLETVDETEVWVDPAQGSKNV-------------------------------------
------------------------------------------------------------------------
-----------------------
>KAB05_13
-MQMSVSSILERLGLVSQNRAVHIQFSNQSLNQQVFLQRIEGEHTLNQGSVAELLCLSTNAHIALKQFIGCQ

VAVDQVTDTGQFFRTTGIITEASQGQSDGSLTIYNLTLKDPTALWHKRRNSRVFMNKSVRDISEILFKEWQG
KSPLFASSITLDTSGLT-KDYDVRPFVMQSNESDYDFLTRLWRSEGINWLVDESQLFVADPNASIQPQVLRL
IDDNQNYQALERRSIRYQRSSATEQFDTITQVKAERRLQPTSVHVQRWQADALQQEEGSGSVQGTQKHSEHY
DNASLNLEDAWHVSPAWMQDLNGEDQATASGNSQIEQLNQHINAYHHLSSKQFTVAGNVRDAQVGYWFELNE
HPELDQ-HDSADKEFLILSKHYYNQNNLPKELQQQLERLLPQGKLKAAQLD--SQNPEQRHFAELNVVRRNI
KAVPEYNPLEHRPAAHPQRARVVGLEGESIHVDQWGRIKVRFLFTRADDHSHDGGAGSNDNDTDSAWVDVLT
PWAGAGYGARFLPRVGEIVVIDFFDGNIDRPFVVGRIHEAERHPTQFDQKGQLPDTKKLSGIRSEEVDGKGF
NQLRFDDTTGQISAQLQSSHAASQLNLGNLSHPKDKAESDGRGEGFELRTDQWGAVRAGSGLLVSTHKQDQA
QGVHLDASEAKQQIEGGLNNAKALSEVAKNQQTDPLEML---------------------------------
----------------------------------------ENLKTFIEQIEE--------------------
---------------------------------------------------------------NDQDKAAAF
KQALMILTAPNSIALASNEDIHLSADGQLNQTAGDSINLSTQKNLIAHAQNKISLFAAQQGARLYAGKGKVE
IQAQGDGADLIARKAVQVISTEDKIEATAAKEIVLTAGGSQVKITGSGIFMTTSGKFEVKAGQHVFMGGAEV
GMNLQGLP--------AYEAYNERFKMLLPSGEPLSFIDYKI---SSEGKEIFANSDKKGQTKEIHSPK---
---EQELKLDLLWLDLETVDETEVWVDPAQGSKNV-------------------------------------
------------------------------------------------------------------------
-----------------------

>KAB06_13
-MQMSVSSILERLGLVSQNRAVHIQFSNQSLNQQVFLQRIEGEHTLNQGSVAELLCLSTNAHIALKQFIGCQ
VAVDQVTDTGQFFRTTGIITEASQGQSDGSLTIYNLTLKDPTALWHKRRNSRVFMNKSVRDISEILFKEWQG
KSPLFASSITLDTSGLT-KDYDVRPFVMQSNESDYDFLTRLWRSEGINWLVDESQLFVADPNASIQPQVLRL
IDDNQNYQALERRSIRYQRSSATEQFDTITQVKAERRLQPTSVHVQRWQADALQQEEGSGSVQGTQKHSEHY
DNASLNLEDAWHVSPAWMQDLNGEDQATASGNSQIEQLNQHINAYHHLSSKQFTVAGNVRDAQVGYWFELNE
HPELDQ-HDSADKEFLILSKHYYNQNNLPKELQQQLERLLPQGKLKAAQLD--SQNPEQRHFAELNVVRRNI
KAVPEYNPLEHRPAAHPQRARVVGLEGESIHVDQWGRIKVRFLFTRADDHSHDGGAGSNDNDTDSAWVDVLT
PWAGAGYGARFLPRVGEIVVIDFFDGNIDRPFVVGRIHEAERHPTQFDQKGQLPDTKKLSGIRSEEVDGKGF
NQLRFDDTTGQISAQLQSSHAASQLNLGNLSHPKDKAESDGRGEGFELRTDQWGAVRAGSGLLVSTHKQDQA
QGVHLDASEAKQQIEGGLNNAKALSEVAKNQQTDPLEML---------------------------------
----------------------------------------ENLKTFIEQIEE--------------------
---------------------------------------------------------------NDQDKAAAF
KQALMILTAPNSIALASNEDIHLSADGQLNQTAGDSINLSTQKNLIAHAQNKISLFAAQQGARLYAGKGKVE
IQAQGDGADLIARKAVQVISTEDKIEATAAKEIVLTAGGSQVKITGSGIFMTTSGKFEVKAGQHVFMGGAEV
GMNLQGLP--------AYEAYNERFKMLLPSGEPLSFIDYKI---SSEGKEIFANSDKKGQTKEIHSPK---

---EQELKLDLLWLDLETVDETEVWVDPAQGSKNV-------------------------------------
------------------------------------------------------------------------
-----------------------
>WKA02_13
-MQMSVSSILERLGLVSQNRAVHIQFSNQSLNQQVFLQRIEGEHTLNQGSVAELLCLSTNAHIALKQFIGCQ
VAVDQVTDTGQFFRTTGIITEASQGQSDGSLTIYNLTLKDPTALWHKRRNSRVFMNKSVRDISEILFKEWQG
KSPLFASSITLDTSGLT-KDYDVRPFVMQSNESDYDFLTRLWRSEGINWLVDESQLFVADPNASIQPQVLRL
IDDNQNYQALERRSIRYQRSSATEQFDTITQVKAERRLQPTSVHVQRWQADALQQEEGSGSVQGTQKHSEHY
DNASLNLEDAWHVSPAWMQDLNGEDQATASGNSQIEQLNQHINAYHHLSSKQFTVAGNVRDAQVGYWFELNE
HPELDQ-HDSADKEFLILSKHYYNQNNLPKELQQQLERLLPQGKLKAAQLD--SQNPEQRHFAELNVVRRNI
KAVPEYNPLEHRPAAHPQRARVVGLEGESIHVDQWGRIKVRFLFTRADDHSHDGGAGSNDNDTDSAWVDVLT
PWAGAGYGARFLPRVGEIVVIDFFDGNIDRPFVVGRIHEAERHPTQFDQKGQLPDTKKLSGIRSEEVDGKGF
NQLRFDDTTGQISAQLQSSHAASQLNLGNLSHPKDKAESDGRGEGFELRTDQWGAVRAGSGLLVSTHKQDQA
QGVHLDASEAKQQIEGGLNNAKALSEVAKNQQTDPLEML---------------------------------
----------------------------------------ENLKTFIEQIEE--------------------
---------------------------------------------------------------NDQDKAAAF
KQALMILTAPNSIALASNEDIHLSADGQLNQTAGDSINLSTQKNLIAHAQNKISLFAAQQGARLYAGKGKVE
IQAQGDGADLIARKAVQVISTEDKIEATAAKEIVLTAGGSQVKITGSGIFMTTSGKFEVKAGQHVFMGGAEV
GMNLQGLP--------AYEAYNERFKMLLPSGEPLSFIDYKI---SSEGKEIFANSDKKGQTKEIHSPK---
---EQELKLDLLWLDLETVDETEVWVDPAQGSKNV-------------------------------------
------------------------------------------------------------------------
-----------------------
>XH856_13
-MQMSVSSILERLGLVSQNRAVHIQFSNQSLNQQVFLQRIEGEHTLNQGSVAELLCLSTNAHIALKQFIGCQ
VAVDQVTDTGQFFRTTGIITEASQGQSDGSLTIYNLTLKDPTALWHKRRNSRVFMNKSVRDISEILFKEWQG
KSPLFASSITLDTSGLT-KDYDVRPFVMQSNESDYDFLTRLWRSEGINWLVDESQLFVADPNASIQPQVLRL
IDDNQNYQALERRSIRYQRSSATEQFDTITQVKAERRLQPTSVHVQRWQADALQQEEGSGSVQGTQKHSEHY
DNASLNLEDAWHVSPAWMQDLNGEDQATASGNSQIEQLNQHINAYHHLSSKQFTVAGNVRDAQVGYWFELNE
HPELDQ-HDSADKEFLILSKHYYNQNNLPKELQQQLERLLPQGKLKAAQLD--SQNPEQRHFAELNVVRRNI
KAVPEYNPLEHRPAAHPQRARVVGLEGESIHVDQWGRIKVRFLFTRADDHSHDGGAGSNDNDTDSAWVDVLT
PWAGAGYGARFLPRVGEIVVIDFFDGNIDRPFVVGRIHEAERHPTQFDQKGQLPDTKKLSGIRSEEVDGKGF
NQLRFDDTTGQISAQLQSSHAASQLNLGNLSHPKDKAESDGRGEGFELRTDQWGAVRAGSGLLVSTHKQDQA
QGVHLDASEAKQQIEGGLNNAKALSEVAKNQQTDPLEML---------------------------------

----------------------------------------ENLKTFIEQIEE--------------------
---------------------------------------------------------------NDQDKAAAF
KQALMILTAPNSIALASNEDIHLSADGQLNQTAGDSINLSTQKNLIAHAQNKISLFAAQQGARLYAGKGKVE
IQAQGDGADLIARKAVQVISTEDKIEATAAKEIVLTAGGSQVKITGSGIFMTTSGKFEVKAGQHVFMGGAEV
GMNLQGLP--------AYEAYNERFKMLLPSGEPLSFIDYKI---SSEGKEIFANSDKKGQTKEIHSPK---
---EQELKLDLLWLDLETVDETEVWVDPAQGSKNV-------------------------------------
------------------------------------------------------------------------
-----------------------
>AF-401_30
-MQMSVSSILERLGLVSQNRAVHIQFSNQSLNQQVFLQRIEGEHTLNQGSVAELLCLSTNAHIALKQFIGCQ
VAVDQVTDTGQFFRTTGIITEASQGQSDGSLTIYNLTLKDPTALWHKRRNSRVFMNKSVRDISEILFKEWQE
KSPLFASSITLDTSGLT-EDYDVRPFVMQSNESDYDFLTRLWRSEGINWLVVESQLFVAEPNASIQPQVLRL
IDDNQNYQALERRSIRYQRSSATEQFDTITQVKAERRLQPTSVHVQRWQADALQQEEGSGSVQGTQKHSEHY
DNASLNLEDAWHVSPAWMQDLNGEDQATASGNSQIEQLNQHINAYHHLSSKQFTVAGNVRDAQVGYWFELNE
HPELDQ-HDSADKEFLILSKHYYNQNNLPKELQQQLERLLPQGKLKAAQLD--SQNPEQRHFAELNVVRRNV
KAVPEYNPLEHRPAAYPQRARVVGLEGESIHVDQWGRIKVRFLFTRADDHSHDGGAGSNDNDTDSAWVDVLT
PWAGAGYGARFLPRVGEIVVIDFFDGNIDRPFVVGRIHEAERHPTQFDQKGQLPDTKKLSGIRSEEVDGKGF
NQLRFDDTTGQISAQLQSSHAASQLNLGNLSHPKDKAESDGRGEGFELRTDQWGAVRAGSGLLVSTHKQDQA
QGVHLDASEAKQQIEGGLNNAKALSEVAKNQQTDPLEML---------------------------------
----------------------------------------ENLKTFIEQIEE--------------------
---------------------------------------------------------------KDQDKAAAF
KQALMILTAPNSIALASNEDIHLSADGQLNQTAGDSINLSTQKNLIAHAQNKISLFAAQQGARLYAGKGKVE
IQAQGDGADLIARKAVQVISTEDKIEATAAKEIVLTAGGSQVKITGSGIFMTTSGKFEVKAGQHVFMGGGSV
NSSLPYLP------EQGKQKYGVWFDVMDKQGNKLKPGTDYIIFDENDKEIERGKLDRTGLVKLETEEP---
-----------------NKQYKIHVEI---------------------------------------------
------------------------------------------------------------------------
-----------------------
>3207_1
-MQMSVSSILERLGLVSQNRAVHIQFSNQSLNQQVFLQRIEGEHTLNQGSVAELLCLSTNAHIALKQFIGCQ
VAVDQVTDTGQFFRTTGIITEASQGQSDGSLTIYNLTLKDPTALWHKRRNSRVFMNKSVRDISEILFKEWQG
KSPLFASSITLDTSGLT-KDYDVRPFVMQSNESDYDFLTRLWRSEGINWLVDESQLFIVDPNASIQPQVLRL
IDDNQNYQALERRSIRYQRSSATEQFDTITQVKAERRLQPTSVHVQRWQADALQQEEGSGSVQGTQKHSEHY
DNASLNLEDAWHVSPAWMQDLNGEDQATASGNSQIEQLNQHINAYHHLSSKQFTVAGNVRDAQVGYWFELND

HPELDQ-HDSADKEFLILSKHYYNQNNLPKELQQQLERLLPQGKLKAAQLD--SQNPEQRHFAELNVVRRNI
KAVPEYNPLEHRPAAHPQRARVVGLEGESIHVDQWGRIKVRFLFTRADDHSHDGGAGSNDNDTDSAWVDVLT
PWAGAGYGARFLPRVGEIVVIDFFDGNVDRPFVVGRIHEAERHPTQFDQKGQLPDTKKLSGIRSEEVDGKGF
NQLRFDDTTGQISAQLQSSHAASQLNLGNLSHPKDKAESDGRGEGFELRTDQWGAVRAGSGLLVSTHKQDQA
QGVHLDASEAKQQIEGGLNNAKALSEVAKNQQTDPLEML---------------------------------
----------------------------------------ENLKTFIEQIEE--------------------
---------------------------------------------------------------KDQDKAAAF
KQALMILTAPNSIALASNEDIHLSADGQLNQTAGDSINLSTQKNLIAHAQNKISLFAAQQGARLYAGKGKVE
IQAQGDGADLIARKAVQVISTEDKIEATAAKEIVLTAGGSQVKITGSGIFMTTSGKFEVKAGQHLFMGGGSV
NSSLPYLP------EQGKQKYGVWFDVMDKQGNKLKPGTEYIIFDEHDKEIERGKLDRTGLVKLETEEP---
-----------------NKQYKIHVVN---------------------------------------------
------------------------------------------------------------------------
-----------------------
>AR_0088_1
-MQMSVSSILERLGLVSQNRAVHIQFSNQSLNQQVFLQRIEGEHTLNQGSVAELLCLSTNAHIALKQFIGCQ
VAVDQVTDTGQFFRTTGIITEASQGQSDGSLTIYNLTLKDPTALWHKRRNSRVFMNKSVRDISEILFKEWQG
KSPLFASSITLDTSGLT-KDYDVRPFVMQSNESDYDFLTRLWRSEGINWLVDESQLFIVDPNASIQPQVLRL
IDDNQNYQALERRSIRYQRSSATEQFDTITQVKAERRLQPTSVHVQRWQADALQQEEGSGSVQGTQKHSEHY
DNASLNLEDAWHVSPAWMQDLNGEDQATASGNSQIEQLNQHINAYHHLSSKQFTVAGNVRDAQVGYWFELND
HPELDQ-HDSADKEFLILSKHYYNQNNLPKELQQQLERLLPQGKLKAAQLD--SQNPEQRHFAELNVVRRNI
KAVPEYNPLEHRPAAHPQRARVVGLEGESIHVDQWGRIKVRFLFTRADDHSHDGGAGSNDNDTDSAWVDVLT
PWAGAGYGARFLPRVGEIVVIDFFDGNVDRPFVVGRIHEAERHPTQFDQKGQLPDTKKLSGIRSEEVDGKGF
NQLRFDDTTGQISAQLQSSHAASQLNLGNLSHPKDKAESDGRGEGFELRTDQWGAVRAGSGLLVSTHKQDQA
QGVHLDASEAKQQIEGGLNNAKALSEVAKNQQTDPLEML---------------------------------
----------------------------------------ENLKTFIEQIEE--------------------
---------------------------------------------------------------KDQDKAAAF
KQALMILTAPNSIALASNEDIHLSADGQLNQTAGDSINLSTQKNLIAHAQNKISLFAAQQGARLYAGKGKVE
IQAQGDGADLIARKAVQVISTEDKIEATAAKEIVLTAGGSQVKITGSGIFMTTSGKFEVKAGQHLFMGGGSV
NSSLPYLP------EQGKQKYGVWFDVMDKQGNKLKPGTEYIIFDEHDKEIERGKLDRTGLVKLETEEP---
-----------------NKQYKIHVVN---------------------------------------------
------------------------------------------------------------------------
-----------------------
>HWAB8_1

-MQMSVSSILERLGLVSQNRAVHIQFSNQSLNQQVFLQRIEGEHTLNQGSVAELLCLSTNAHIALKQFIGCQ
VAVDQVTDTGQFFRTTGIITEASQGQSDGSLTIYNLTLKDPTALWHKRRNSRVFMNKSVRDISEILFKEWQG
KSPLFASSITLDTSGLT-KDYDVRPFVMQSNESDYDFLTRLWRSEGINWLVDESQLFIVDPNASIQPQVLRL
IDDNQNYQALERRSIRYQRSSATEQFDTITQVKAERRLQPTSVHVQRWQADALQQEEGSGSVQGTQKHSEHY
DNASLNLEDAWHVSPAWMQDLNGEDQATASGNSQIEQLNQHINAYHHLSSKQFTVAGNVRDAQVGYWFELND
HPELDQ-HDSADKEFLILSKHYYNQNNLPKELQQQLERLLPQGKLKAAQLD--SQNPEQRHFAELNVVRRNI
KAVPEYNPLEHRPAAHPQRARVVGLEGESIHVDQWGRIKVRFLFTRADDHSHDGGAGSNDNDTDSAWVDVLT
PWAGAGYGARFLPRVGEIVVIDFFDGNVDRPFVVGRIHEAERHPTQFDQKGQLPDTKKLSGIRSEEVDGKGF
NQLRFDDTTGQISAQLQSSHAASQLNLGNLSHPKDKAESDGRGEGFELRTDQWGAVRAGSGLLVSTHKQDQA
QGVHLDASEAKQQIEGGLNNAKALSEVAKNQQTDPLEML---------------------------------
----------------------------------------ENLKTFIEQIEE--------------------
---------------------------------------------------------------KDQDKAAAF
KQALMILTAPNSIALASNEDIHLSADGQLNQTAGDSINLSTQKNLIAHAQNKISLFAAQQGARLYAGKGKVE
IQAQGDGADLIARKAVQVISTEDKIEATAAKEIVLTAGGSQVKITGSGIFMTTSGKFEVKAGQHLFMGGGSV
NSSLPYLP------EQGKQKYGVWFDVMDKQGNKLKPGTEYIIFDEHDKEIERGKLDRTGLVKLETEEP---
-----------------NKQYKIHVVN---------------------------------------------
------------------------------------------------------------------------
-----------------------

>AB042_1

-MQMSVSSILERLGLVSQNRAVHIQFSNQSLNQQVFLQRIEGEHTLNQGSVAELLCLSTNAHIALKQFIGCQ
VAVDQVTDTGQFFRTTGIITEASQGQSDGSLTIYNLTLKDPTALWHKRRNSRVFMNKSVRDISEILFKEWQG
KSPLFASSITLDTSGLT-KDYDVRPFVMQSNESDYDFLTRLWRSEGINWLVDESQLFIADPNASIQPQVLRL
IDDNQNYQALERRSIRYQRSSATEQFDTITQVKAERRLQPTSVHVQRWQADALQQEEGSGSVQGTQKHSEHY
DNASLNLEDAWHVSPAWMQDLNGEDQATASGNSQIEQLNQHINAYHHLSSKQFTVAGNVRDAQVGYWFELND
HPELDQ-HDSADKEFLILSKHYYNQNNLPKELQQQLERLLPQGKLKAAQLD--SQNPEQRHFAELNVVRRNI
KAVPEYNPLEHRPAAHPQRARVVGLEGESIHVDQWGRIKVRFLFTRADDHSHDGGAGSNDNDTDSAWVDVLT
PWAGAGYGARFLPRVGEIVVIDFFDGNIDRPFVVGRIHEAERHPTQFDQKGQLPDTKKLSGIRSEEVDGKGF
NQLRFDDTTGQISAQLQSSHAASQLNLGNLSHPKDKAESDGRGEGFELRTDQWGAVRAGSGLLVSTHKQDQA
QGVHLDASEAKQQIEGGLNNAKALSEVAKNQQTDPLEML---------------------------------
----------------------------------------ENLKTFIEQIEE--------------------
---------------------------------------------------------------KDQDKAAAF
KQALMILTAPNSIALASNEDIHLSADGQLNQTAGDSINLSTQKNLIAHAQNKISLFAAQQGARLYAGKGKVE
IQAQGDGADLIARKAVQVISTEDKIEATAAKEIVLTAGGSQVKITGSGIFMTTSGKFEVKAGQHLFMGGGSV

NSSLPYLP------EQGKQKYGVWFDVMDKQGNKLKPGTEYIIFDEHDKEIERGKLDRTGLVKLETEEP---
-----------------NKQYKIHVVN------------------------------------------
---------------------------------------------------------------------
----------------------
>ATCC17978-mff_1
-MQMSVSSILERLGLVSQNRAVHIQFSNQSLNQQVFLQRIEGEHTLNQGSVAELLCLSTNAHIALKQFIGCQ
VAVDQVTDTGQFFRTTGIITEASQGQSDGSLTIYNLTLKDPTALWHKRRNSRVFMNKSVRDISEILFKEWQG
KSPLFASSITLDTSGLT-KDYDVRPFVMQSNESDYDFLTRLWRSEGINWLVDESQLFIADPNASIQPQVLRL
IDDNQNYQALERRSIRYQRSSATEQFDTITQVKAERRLQPTSVHVQRWQADALQQEEGSGSVQGTQKHSEHY
DNASLNLEDAWHVSPAWMQDLNGEDQATASGNSQIEQLNQHINAYHHLSSKQFTVAGNVRDAQVGYWFELND
HPELDQ-HDSADKEFLILSKHYYNQNNLPKELQQQLERLLPQGKLKAAQLD--SQNPEQRHFAELNVVRRNI
KAVPEYNPLEHRPAAHPQRARVVGLEGESIHVDQWGRIKVRFLFTRADDHSHDGGAGSNDNDTDSAWVDVLT
PWAGAGYGARFLPRVGEIVVIDFFDGNIDRPFVVGRIHEAERHPTQFDQKGQLPDTKKLSGIRSEEVDGKGF
NQLRFDDTTGQISAQLQSSHAASQLNLGNLSHPKDKAESDGRGEGFELRTDQWGAVRAGSGLLVSTHKQDQA
QGVHLDASEAKQQIEGGLNNAKALSEVAKNQQTDPLEML----------------------------------
---------------------------------------ENLKTFIEQIEE--------------------
-------------------------------------------------------------KDQDKAAAF
KQALMILTAPNSIALASNEDIHLSADGQLNQTAGDSINLSTQKNLIAHAQNKISLFAAQQGARLYAGKGKVE
IQAQGDGADLIARKAVQVISTEDKIEATAAKEIVLTAGGSQVKITGSGIFMTTSGKFEVKAGQHLFMGGGSV
NSSLPYLP------EQGKQKYGVWFDVMDKQGNKLKPGTEYIIFDEHDKEIERGKLDRTGLVKLETEEP---
-----------------NKQYKIHVVN------------------------------------------
---------------------------------------------------------------------
----------------------
>AB5075-UW_15
-MFNNIFQILESFGFLSQHRSVYLQFSDASLNSQVFLQRIDGQHYLNQGMTAELICLSTNAHIPLKTFIGLQ
VAVDQVTDRGSFFRTTGIITGASQGQSDGALTLYKLTVSDPTYLWHKRRNSRVFINKSVKEISEILFQEWQG
KSPLFASSLTLDLSGLK-QTYDVRPFVMQLNESDYDFLTRLWRSEGISWLIDEAELTVASNMDNIQPQKLRL
IDDNNQYQALTRRVIRYHRSSATEQFDSMTSLMADRSLQPTSIFVQRWQPDVLQQTDGAGSVQSKHQHSTNY
DNQSLSLEEAWHFSPAWMQDLNGEDGATSASNQQIEKFNQNLSAYYDAQSKQFIAKTTVRDTQVGYWFELNE
HPEIDQ-HESTDKEFLIIGKNYYNQNNLPKDLNQQIQTLLQQSDWQASNTD-------ERQANQLILQRRYI
PTTPAYNPQTHSPVAHPQRAKVVGPEGEEIYVDEWGRIKVRFLFTRSDDHSHDGGAGTNNNDTDSAWIDVLT
PWAGEGYGARFLPRIGEIVVIDFFNGDIDRPFVMGRIHEAQRHPTKFDNKGKLPDTKKLSGIRSKEVSGSGF
GQLRFDDTPGQISTQLQSSHGASQLNLGKLSHPKDKAESEDRGEGFELRTDQWGALRAGQGLLVSTHKQDNA

KGDHLYAEVAKKQLEGSQTNSKALSDIAKNQKTDEIESI-----------------------------------
---------------------------------------EQLKDFASQIQQ--------------------
----------------------------------------------------------------------QIAKF
EKALLLLSSPDGIALSSSEDIHISADAQINQIAGDSINISTQKNVIAHAQNRLSLFAAQSGLKAVAAQGKVE
IQAQADALDVLSKLGITISSTDDKVIISSPKEVKITGGSSQITLNGSGIFPKTGGKFQVNAGQHLFMGGASA
NASAPELP------KAKPMQGALELLRSYGGDNFFKQNSYKVI--DSLGKQITGKLDGNGFAQVTGIAP-GP
AKVVFEKDNTSAWLQSSDFKRNYTWAEPVKSVQGLMKNALEAVGQNTMSQLQNNLLSTDKNSFKNLGKNTLD
NLAGQTVAQIKNQVTNTALNTVSKQLNLNLSAD----QMKSLGQMATNPSQSLEMLKEQGGDFLSDQMTAKL
FKTTNQESPIQQGDLDTFVRSKK
>A1_15
-MFNNIFQILESFGFLSQHRSVYLQFSDASLNSQVFLQRIDGQHYLNQGMTAELICLSTNAHIPLKTFIGLQ
VAVDQVTDRGSFFRTTGIITGASQGQSDGALTLYKLTVSDPTYLWHKRRNSRVFMNKSVKEISEILFQEWQG
KSPLFASSLTLDLSGLK-QTYDVRPFVMQLNESDYDFLTRLWRSEGISWLIDEAELTVASNMDNIQPQKLRL
IDDNNQYQALTRRVIRYHRSSATEQFDSMTSLMADRSLQPTSIFVQRWQPDVLQQTDGAGSVQSKHQHSTNY
DNQSLSLEEAWHFSPAWMQDLNGEDGATSASNQQIEKFNQNLSAYYDAQSKQFIAKTTVRDTQVGYWFELNE
HPEIDQ-HESTDKEFLIIGKNYYNQNNLPKDLNQQIQTLLQQSDWQASNTD-------ERQANQLILQRRYI
PTTPAYNPQTHSPVAHPQRAKVVGPEGEEIYVDEWGRIKVRFLFTRSDDHSHDGGAGTNNNDTDSAWIDVLT
PWAGEGYGARFLPRIGEIVVIDFFNGDIDRPFVMGRIHEAQRHPTKFDNKGKLPDTKKLSGIRSKEVSGSGF
GQLRFDDTPGQISTQLQSSHGASQLNLGKLSHPKDKAESEDRGEGFELRTDQWGALRAGQGLLVSTHKQDNA
KGDHLDAEVAKKQLEGSQTNSKALSDIAKNQKTDEIESI-----------------------------------
---------------------------------------EQLKDFASQIQQ--------------------
----------------------------------------------------------------------QIAKF
EKALLLLSSPDGIALSSSEDIHISADAQINQIAGDSINISTQKNVIAHAQNRLSLFAAQSGLKAVAAQGKVE
IQAQADALDVLSKLGITISSTDDKVIISSPKEVKITGGSSQITLNGSGIFPKTGGKFQVNAGQHLFMGGASA
NASAPELP------KAKPMQGALELLRSYGGDNFFKQNSYKVI--DSLGKQITGKLDGNGFAQVTGIAP-GP
AKVVFEKDNTSAWLQSSDFKRNYTWAEPVKSVQGLMKNALEAVGQNTMSQLQNNLLSTDKNSFKNLGKNTLD
NLAGQTVAQIKNQVTNTALNTVSKQLNLNLSAD----QMKSLGQMATNPSQSLEMLKEQGGDFLSDQMTAKL
FKTTNQESPIQQGDLDTFVRSKK
>A388_15
-MFNNIFQILESFGFLSQHRSVYLQFSDASLNSQVFLQRIDGQHYLNQGMTAELICLSTNAHIPLKTFIGLQ
VAVDQVTDRGSFFRTTGIITGASQGQSDGALTLYKLTVSDPTYLWHKRRNSRVFMNKSVKEISEILFQEWQG
KSPLFASSLTLDLSGLK-QTYDVRPFVMQLNESDYDFLTRLWRSEGISWLIDEAELTVASNMDNIQPQKLRL
IDDNNQYQALTRRVIRYHRSSATEQFDSMTSLMADRSLQPTSIFVQRWQPDVLQQTDGAGSVQSKHQHSTNY

DNQSLSLEEAWHFSPAWMQDLNGEDGATSASNQQIEKFNQNLSAYYDAQSKQFIAKTTVRDTQVGYWFELNE
HPEIDQ-HESTDKEFLIIGKNYYNQNNLPKDLNQQIQTLLQQSDWQASNTD-------ERQANQLILQRRYI
PTTPAYNPQTHSPVAHPQRAKVVGPEGEEIYVDEWGRIKVRFLFTRSDDHSHDGGAGTNNNDTDSAWIDVLT
PWAGEGYGARFLPRIGEIVVIDFFNGDIDRPFVMGRIHEAQRHPTKFDNKGKLPDTKKLSGIRSKEVSGSGF
GQLRFDDTPGQISTQLQSSHGASQLNLGKLSHPKDKAESEDRGEGFELRTDQWGALRAGQGLLVSTHKQDNA
KGDHLDAEVAKKQLEGSQTNSKALSDIAKNQKTDEIESI---------------------------------
----------------------------------------EQLKDFASQIQQ--------------------
-------------------------------------------------------------------QIAKF
EKALLLLSSPDGIALSSSEDIHISADAQINQIAGDSINISTQKNVIAHAQNRLSLFAAQSGLKAVAAQGKVE
IQAQADALDVLSKLGITISSTDDKVIISSPKEVKITGGSSQITLNGSGIFPKTGGKFQVNAGQHLFMGGASA
NASAPELP------KAKPMQGALELLRSYGGDNFFKQNSYKVI--DSLGKQITGKLDGNGFAQVTGIAP-GP
AKVVFEKDNTSAWLQSSDFKRNYTWAEPVKSVQGLMKNALEAVGQNTMSQLQNNLLSTDKNSFKNLGKNTLD
NLAGQTVAQIKNQVTNTALNTVSKQLNLNLSAD----QMKSLGQMATNPSQSLEMLKEQGGDFLSDQMTAKL
FKTTNQESPIQQGDLDTFVRSKK
>AB307-0294_15
-MFNNIFQILESFGFLSQHRSVYLQFSDASLNSQVFLQRIDGQHYLNQGMTAELICLSTNAHIPLKTFIGLQ
VAVDQVTDRGSFFRTTGIITGASQGQSDGALTLYKLTVSDPTYLWHKRRNSRVFMNKSVKEISEILFQEWQG
KSPLFASSLTLDLSGLK-QTYDVRPFVMQLNESDYDFLTRLWRSEGISWLIDEAELTVASNMDNIQPQKLRL
IDDNNQYQALTRRVIRYHRSSATEQFDSMTSLMADRSLQPTSIFVQRWQPDVLQQTDGAGSVQSKHQHSTNY
DNQSLSLEEAWHFSPAWMQDLNGEDGATSASNQQIEKFNQNLSAYYDAQSKQFIAKTTVRDTQVGYWFELNE
HPEIDQ-HESTDKEFLIIGKNYYNQNNLPKDLNQQIQTLLQQSDWQASNTD-------ERQANQLILQRRYI
PTTPAYNPQTHSPVAHPQRAKVVGPEGEEIYVDEWGRIKVRFLFTRSDDHSHDGGAGTNNNDTDSAWIDVLT
PWAGEGYGARFLPRIGEIVVIDFFNGDIDRPFVMGRIHEAQRHPTKFDNKGKLPDTKKLSGIRSKEVSGSGF
GQLRFDDTPGQISTQLQSSHGASQLNLGKLSHPKDKAESEDRGEGFELRTDQWGALRAGQGLLVSTHKQDNA
KGDHLDAEVAKKQLEGSQTNSKALSDIAKNQKTDEIESI---------------------------------
----------------------------------------EQLKDFASQIQQ--------------------
-------------------------------------------------------------------QIAKF
EKALLLLSSPDGIALSSSEDIHISADAQINQIAGDSINISTQKNVIAHAQNRLSLFAAQSGLKAVAAQGKVE
IQAQADALDVLSKLGITISSTDDKVIISSPKEVKITGGSSQITLNGSGIFPKTGGKFQVNAGQHLFMGGASA
NASAPELP------KAKPMQGALELLRSYGGDNFFKQNSYKVI--DSLGKQITGKLDGNGFAQVTGIAP-GP
AKVVFEKDNTSAWLQSSDFKRNYTWAEPVKSVQGLMKNALEAVGQNTMSQLQNNLLSTDKNSFKNLGKNTLD
NLAGQTVAQIKNQVTNTALNTVSKQLNLNLSAD----QMKSLGQMATNPSQSLEMLKEQGGDFLSDQMTAKL
FKTTNQESPIQQGDLDTFVRSKK

>AR_0078_15
-MFNNIFQILESFGFLSQHRSVYLQFSDASLNSQVFLQRIDGQHYLNQGMTAELICLSTNAHIPLKTFIGLQ
VAVDQVTDRGSFFRTTGIITGASQGQSDGALTLYKLTVSDPTYLWHKRRNSRVFMNKSVKEISEILFQEWQG
KSPLFASSLTLDLSGLK-QTYDVRPFVMQLNESDYDFLTRLWRSEGISWLIDEAELTVASNMDNIQPQKLRL
IDDNNQYQALTRRVIRYHRSSATEQFDSMTSLMADRSLQPTSIFVQRWQPDVLQQTDGAGSVQSKHQHSTNY
DNQSLSLEEAWHFSPAWMQDLNGEDGATSASNQQIEKFNQNLSAYYDAQSKQFIAKTTVRDTQVGYWFELNE
HPEIDQ-HESTDKEFLIIGKNYYNQNNLPKDLNQQIQTLLQQSDWQASNTD-------ERQANQLILQRRYI
PTTPAYNPQTHSPVAHPQRAKVVGPEGEEIYVDEWGRIKVRFLFTRSDDHSHDGGAGTNNNDTDSAWIDVLT
PWAGEGYGARFLPRIGEIVVIDFFNGDIDRPFVMGRIHEAQRHPTKFDNKGKLPDTKKLSGIRSKEVSGSGF
GQLRFDDTPGQISTQLQSSHGASQLNLGKLSHPKDKAESEDRGEGFELRTDQWGALRAGQGLLVSTHKQDNA
KGDHLDAEVAKKQLEGSQTNSKALSDIAKNQKTDEIESI---------------------------------
----------------------------------------EQLKDFASQIQQ--------------------
-------------------------------------------------------------------QIAKF
EKALLLLSSPDGIALSSSEDIHISADAQINQIAGDSINISTQKNVIAHAQNRLSLFAAQSGLKAVAAQGKVE
IQAQADALDVLSKLGITISSTDDKVIISSPKEVKITGGSSQITLNGSGIFPKTGGKFQVNAGQHLFMGGASA
NASAPELP------KAKPMQGALELLRSYGGDNFFKQNSYKVI--DSLGKQITGKLDGNGFAQVTGIAP-GP
AKVVFEKDNTSAWLQSSDFKRNYTWAEPVKSVQGLMKNALEAVGQNTMSQLQNNLLSTDKNSFKNLGKNTLD
NLAGQTVAQIKNQVTNTALNTVSKQLNLNLSAD----QMKSLGQMATNPSQSLEMLKEQGGDFLSDQMTAKL
FKTTNQESPIQQGDLDTFVRSKK
>D36_15
-MFNNIFQILESFGFLSQHRSVYLQFSDASLNSQVFLQRIDGQHYLNQGMTAELICLSTNAHIPLKTFIGLQ
VAVDQVTDRGSFFRTTGIITGASQGQSDGALTLYKLTVSDPTYLWHKRRNSRVFMNKSVKEISEILFQEWQG
KSPLFASSLTLDLSGLK-QTYDVRPFVMQLNESDYDFLTRLWRSEGISWLIDEAELTVASNMDNIQPQKLRL
IDDNNQYQALTRRVIRYHRSSATEQFDSMTSLMADRSLQPTSIFVQRWQPDVLQQTDGAGSVQSKHQHSTNY
DNQSLSLEEAWHFSPAWMQDLNGEDGATSASNQQIEKFNQNLSAYYDAQSKQFIAKTTVRDTQVGYWFELNE
HPEIDQ-HESTDKEFLIIGKNYYNQNNLPKDLNQQIQTLLQQSDWQASNTD-------ERQANQLILQRRYI
PTTPAYNPQTHSPVAHPQRAKVVGPEGEEIYVDEWGRIKVRFLFTRSDDHSHDGGAGTNNNDTDSAWIDVLT
PWAGEGYGARFLPRIGEIVVIDFFNGDIDRPFVMGRIHEAQRHPTKFDNKGKLPDTKKLSGIRSKEVSGSGF
GQLRFDDTPGQISTQLQSSHGASQLNLGKLSHPKDKAESEDRGEGFELRTDQWGALRAGQGLLVSTHKQDNA
KGDHLDAEVAKKQLEGSQTNSKALSDIAKNQKTDEIESI---------------------------------
----------------------------------------EQLKDFASQIQQ--------------------
-------------------------------------------------------------------QIAKF
EKALLLLSSPDGIALSSSEDIHISADAQINQIAGDSINISTQKNVIAHAQNRLSLFAAQSGLKAVAAQGKVE

IQAQADALDVLSKLGITISSTDDKVIISSPKEVKITGGSSQITLNGSGIFPKTGGKFQVNAGQHLFMGGASA
NASAPELP------KAKPMQGALELLRSYGGDNFFKQNSYKVI--DSLGKQITGKLDGNGFAQVTGIAP-GP
AKVVFEKDNTSAWLQSSDFKRNYTWAEPVKSVQGLMKNALEAVGQNTMSQLQNNLLSTDKNSFKNLGKNTLD
NLAGQTVAQIKNQVTNTALNTVSKQLNLNLSAD----QMKSLGQMATNPSQSLEMLKEQGGDFLSDQMTAKL
FKTTNQESPIQQGDLDTFVRSKK
>A85_15
-MFNNIFQILESFGFLSQHRSVYLQFSDASLNSQVFLQRIDGQHYLNQGMTAELICLSTNAHIPLKTFIGLQ
VAVDQVTDRGSFFRTTGIITGASQGQSDGALTLYKLTVSDPTYLWHKRRNSRVFMNKSVKEISEILFQEWQG
KSPLFASSLTLDLSGLK-QTYDVRPFVMQLNESDYDFLTRLWRSEGISWLIDEAELTVASNMDNIQPQKLRL
IDDNNQYQALTRRVIRYHRSSATEQFDSMTSLMADRSLQPTSIFVQRWQPDVLQQTDGAGSVQSKHQHSTNY
DNQSLSLEEAWHFSPAWMQDLNGEDGATSASNQQIEKFNQNLSAYYDAQSKQFIAKTTVRDTQVGYWFELNE
HPEIDQ-HESTDKEFLIIGKNYYNQNNLPKDLNQQIQTLLQQSDWQASNTD-------ERQANQLILQRRYI
PTTPAYNPQTHSPVAHPQRAKVVGPEGEEIYVDEWGRIKVRFLFTRSDDHSHDGGAGTNNNDTDSAWIDVLT
PWAGEGYGARFLPRIGEIVVIDFFNGDIDRPFVMGRIHEAQRHPTKFDNKGKLPDTKKLSGIRSKEVSGSGF
GQLRFDDTPGQISTQLQSSHGASQLNLGKLSHPKDKAESEDRGEGFELRTDQWGALRAGQGLLVSTHKQDNA
KGDHLYAEVAKKQLEGSQTNSKALSDIAKNQKTDEIESI---------------------------------
----------------------------------------EQLKDFASQIQQ--------------------
-------------------------------------------------------------------QIAKF
EKALLLLSSPDGIALSSSEDIHISADAQINQIAGDSINISTQKNVIAHAQNRLSLFAAQSGLKAVAAQGKVE
IQAQADALDVLSKLGITISSTDDKVIISSPKEVKITGGSSQITLNGSGIFPKTGGKFQVNAGQHLFMGGASA
NASAPELP------KAKPMQGALELLRSYGGDNFFKQNSYKVI--DSLGKQITGKLDGNGFAQVTGIAP-GP
AKVVFEKDNTSAWLQSSDFKRNYTWAEPVKSVQGLMKNALEAVGQNTMSQLQNNLLSTDKNSFKNLGKNTLD
NLAGQTVAQIKNQVTNTALNTVSKQLNLNLSAD----QMKSLGQMATNPSQSLEMLKEQGGDFLSDQMTAKL
FKTTNQESPIQQGDLDTFVRSKK
>AB0057_15
-MFNNIFQILESFGFLSQHRSVYLQFSDASLNSQVFLQRIDGQHYLNQGMTAELICLSTNAHIPLKTFIGLQ
VAVDQVTDRGSFFRTTGIITGASQGQSDGALTLYKLTVSDPTYLWHKRRNSRVFMNKSVKEISEILFQEWQG
KSPLFASSLTLDLSGLK-QTYDVRPFVMQLNESDYDFLTRLWRSEGISWLIDEAELTVASNMDNIQPQKLRL
IDDNNQYQALTRRVIRYHRSSATEQFDSMTSLMADRSLQPTSIFVQRWQPDVLQQTDGAGSVQSKHQHSTNY
DNQSLSLEEAWHFSPAWMQDLNGEDGATSASNQQIEKFNQNLSAYYDAQSKQFIAKTTVRDTQVGYWFELNE
HPEIDQ-HESTDKEFLIIGKNYYNQNNLPKDLNQQIQTLLQQSDWQASNTD-------ERQANQLILQRRYI
PTTPAYNPQTHSPVAHPQRAKVVGPEGEEIYVDEWGRIKVRFLFTRSDDHSHDGGAGTNNNDTDSAWIDVLT
PWAGEGYGARFLPRIGEIVVIDFFNGDIDRPFVMGRIHEAQRHPTKFDNKGKLPDTKKLSGIRSKEVSGSGF

GQLRFDDTPGQISTQLQSSHGASQLNLGKLSHPKDKAESEDRGEGFELRTDQWGALRAGQGLLVSTHKQDNA
KGDHLYAEVAKKQLEGSQTNSKALSDIAKNQKTDEIESI---------------------------------
----------------------------------------EQLKDFASQIQQ--------------------
-------------------------------------------------------------------QIAKF
EKALLLLSSPDGIALSSSEDIHISADAQINQIAGDSINISTQKNVIAHAQNRLSLFAAQSGLKAVAAQGKVE
IQAQADALDVLSKLGITISSTDDKVIISSPKEVKITGGSSQITLNGSGIFPKTGGKFQVNAGQHLFMGGASA
NASAPELP------KAKPMQGALELLRSYGGDNFFKQNSYKVI--DSLGKQITGKLDGNGFAQVTGIAP-GP
AKVVFEKDNTSAWLQSSDFKRNYTWAEPVKSVQGLMKNALEAVGQNTMSQLQNNLLSTDKNSFKNLGKNTLD
NLAGQTVAQIKNQVTNTALNTVSKQLNLNLSAD----QMKSLGQMATNPSQSLEMLKEQGGDFLSDQMTAKL
FKTTNQESPIQQGDLDTFVRSKK
>WCHAB005078_15
-MFNNIFQILESFGFLSQHRSVYLQFSDASLNSQVFLQRIDGQHYLNQGMTAELICLSTNAHIPLKTFIGLQ
VAVDQVTDRGSFFRTTGIITGASQGQSDGALTLYKLTVSDPTYLWHKRRNSRVFMNKSVKEISEILFQEWQG
KSPLFASSLTLDLSGLK-QTYDVRPFVMQLNESDYDFLTRLWRSEGISWLIDEAELTVASNMDNIQPQKLRL
IDDNNQYQALTRRVIRYHRSSATEQFDSMTSLMADRSLQPTSIFVQRWQPDVLQQTDGAGSVQSKHQHSTNY
DNQSLSLEEAWHFSPAWMQDLNGEDGATSASNQQIEKFNQNLSAYYDAQSKQFIAKTTVRDTQVGYWFELNE
HPEIDQ-HESTDKEFLIIGKNYYNQNNLPKDLNQQIQTLLQQSDWQASNTD-------ERQANQLILQRRYI
PTTPAYNPQTHSPVAHPQRAKVVGPEGEEIYVDEWGRIKVRFLFTRSDDHSHDGGAGTNNNDTDSAWIDVLT
PWAGEGYGARFLPRIGEIVVIDFFNGDIDRPFVMGRIHEAQRHPTKFDNKGKLPDTKKLSGIRSKEVSGSGF
GQLRFDDTPGQISTQLQSSHGASQLNLGKLSHPKDKAESEDRGEGFELRTDQWGALRAGQGLLVSTHKQDNA
KGDHLYAEVAKKQLEGSQTNSKALSDIAKNQKTDEIESI---------------------------------
----------------------------------------EQLKDFASQIQQ--------------------
-------------------------------------------------------------------QIAKF
EKALLLLSSPDGIALSSSEDIHISADAQINQIAGDSINISTQKNVIAHAQNRLSLFAAQSGLKAVAAQGKVE
IQAQADALDVLSKLGITISSTDDKVIISSPKEVKITGGSSQITLNGSGIFPKTGGKFQVNAGQHLFMGGASA
NASAPELP------KAKPMQGALELLRSYGGDNFFKQNSYKVI--DSLGKQITGKLDGNGFAQVTGIAP-GP
AKVVFEKDNTSAWLQSSDFKRNYTWAEPVKSVQGLMKNALEAVGQNTMSQLQNNLLSTDKNSFKNLGKNTLD
NLAGQTVAQIKNQVTNTALNTVSKQLNLNLSAD----QMKSLGQMATNPSQSLEMLKEQGGDFLSDQMTAKL
FKTTNQESPIQQGDLDTFVRSKK
>SDF_15
-MFNNIFQILESFGFLSQHRSVYLQFSDASLNSQVFLQRIDGQHYLNQGMTAELICLSTNAHIPLKTFIGVQ
VAVDQVTDRGSFFCTTGIITGASQGQSDGALTLYKLAISDPTYLWHKRRNSRVFMNKSVKEISEILFQEWQG
KSPLFASSLTLDLSGLK-QTYDVRPFVMQLNESDYEFLTRLWRSEGISWLIDEAELTVASNMDNIQPQKLRL

```
IDDNNQYQALTRRAIRYHRSSATEQFDSMTSLMADRSLQPTSIFVQRWQPDVLQQTDGAGSVQSKHQHSTNY
DNQSLSLEEAWHFSPAWMQDLNGEDGATSASNQQLEKFNQNLSAYYDAQSKQFIAKTTVRDTQVGYWFELNE
HPEIDQ-HESTDKEFLIIGKNYYNQNNLPKDLNQQIQTLLQQSDWQASNTD-------ERQANQLILQRRHI
PTTPAYNPQTHSPVAHPQRAKVVGPEGEEIYVDEWGRIKVRFLFTRSDDHSHDGGAGTNNNDTDSAWIDVLT
PWAGEGYGARFLPRIGEIVVINFFNGDIDRPFVMGRVHEAQRHPTKFDNKGKLPDTKKLSGIRSKEVSGSGF
GQLRFDDTPGQISTQLQSSHGASQLNLGKLSHPKDKAESEDRGEGFELRTDQWGALRAGQGLLVSTHKQDNA
KGDHLDAEVAKKQLEGSQTNSKALSDIAKNQKTDEIESL---------------------------------
----------------------------------------EQLKDFASQIQQ--------------------
--------------------------------------------------------------------QIAKF
EKALLLLSSPDGIALSSSEDIHISADAQINQIAGDSINISTQKNVIAHAQNRLSLFAAQSGLKAVAAQGKVE
IQAQADALDVLSKLGITISSTDDKVIISSPKEVKITGGSSQITLNGSGIFPKTGGKFQVNAGQHLFMGGASA
NASAPELP------KAKPMQGALELLRSYGGDNFFKQNSYKVI--DSLGKQITGKLDGNGFAQITGIAP-GP
AKVVFEKDNTSAWLQSSDFKRNYTWAEPVKSVQGLMKNALEAVGQNTMSQLQNNLLSTDKNSFKNLGKNTLD
NLAGQTVAQIKNQVTNTALNTVSKQLNLNLSAD----QMKSLGQMATNPSQSLEMLKEQGGDFLSDQMTAKL
FKTTNQESPIQQGDLDTFVRTKK
>WKA02_15
-MFNNIFQILESFGFLSQHRSVYLQFSDASLNSQVFLQRIDGQHYLNQGMTAELICLSTNAHIPLKTFIGVQ
VAVDQVTDRGSFFRTTGIITGVSQGQSDGALTLYKLAISDPTYLWHKRRNSRVFMNKSVKEISEILFQEWQG
KSPLFASSLTLDLSGLK-QTYDVRPFVMQLNESDYDFLTRLWRSEGISWLIDEAELTVASNMDNIQPQKLRL
IDDNNQYQALTRRAIRYHRSSATEQFDSMTSLMADRSLQPTSIFVQRWQPDVLQQTDGAGSVQSKHQHSTNY
DNQSLSLEEAWHFSPAWMQDLNGEDGATSASNQQLEKFNQNLSAYYDAQSKQFIAKTTVRDTQVGYWFELNE
HPEIDQ-HESTDKEFLIIGKNYYNQNNLPKDLNQQIQTLLQQSDWQASNTN-------ERQANQLILQRRYI
PTTPAYNPQTHSPVAHPQRAKVVGPEGEEIYVDEWGRIKVRFLFTRSDDHSHDGGAGTNNNDTDSAWIDVLT
PWAGEGYGARFLPRIGEIVVIDFFNGDIDRPFVMGRIHEAQRHPTKFDNKGKLPDTKKLSGIRSKEVSGSGF
GQLRFDDTPGQISTQLQSSHGASQLNLGKLSHPKDKAESEDRGEGFELRTDQWGALRAGQGLLVSTHKQDNA
KGDHLDAEVAKKQLEGSQTNSKALSDIAKNQKTDEIESI---------------------------------
----------------------------------------EQLKDFASQIQQ--------------------
--------------------------------------------------------------------QIAKF
EKALLLLSSPDGIALSSSEDIHISADAQINQIAGDSINISTQKNVIAHAQNRLSLFAAQSGLKAVAAQGKVE
IQAQSDALDVLSKLGITISSTDDKVIISSPKEVKITGGSSQITLNGSGIFPKTGGKFQVNAGQHLFMGGASA
NASAPELP------KAKPMQGALELLRSYGGDNFFKQNSYKVI--DSLGKQITGKLDGNGFAQVTGIAP-GP
AKVVFEKDNTSAWLQSSDFKRNYTWAEPVKSVQGLMKNALEAVGQNTMSQLQNNLLSTDKNSFKNLGKNTLD
NLAGQTVAQIKNQVTNTALNTVSKQLNLNLSAD----QMKSLGQMATNPSQSLEMLKEQGGDFLSDQMTAKL
```

FKTTNQESPIQQGDLDTFVRSKK
>AB031_26
-MFNNISQVLESFGFLSQHRSVYLQFSDASLNSQVFLQRIDGQHYLNQGMTAELICLSTNAHIPLKTFIGVQ
VAVDQVTDRGSFFRTTGIITGASQGQSDGALTLYKLAISDPTYLWHKRRNSRVFMNKSVKEISEILFQEWQG
KSPLFASSLTLDLSGLK-QTYDVRPFVMQLNESDYEFLTRLWRSEGISWLIDEAELTVASNMDNIQPQKLRL
IDDNNQYQALTRRAIRYHRSSATEQFDSMTSLMADRSLQPTSIFVQRWQPDVLQQTDGAGSVQSKHQHSTNY
DNQSLSLEEAWHFSPAWMQDLNGEDGATSASNQQLEKFNQNLSAYYDAQSKQFIAKTTVRDTQVGYWFELNE
HPEIDQ-HESTDKEFLIIGKNYYNQNNLPKDLNQQIQTLVQQSDWQASNTD-------ERQANQLILQRRYI
PTTPAYNPQTHSPVAHPQRAKVVGPEGEEIYVDEWGRIKVRFLFTRSDDHSHDGGAGTNNNDTDSAWIDVLT
PWAGEGYGARFLPRIGEIVVINFFNGDIDRPFVMGRVHEAQRHPTKFDNKGKLPDTKKLSGIRSKEVSGGGF
GQLRFDDTPGQISTQLQSSHGASQLNLGKLSHPKDKAESEDRGEGFELRTDQWGALRAGQGLLVSTHKQDNA
KGDHLDAEVAKKQLEGSQTNSKALSDIAKNQKTDEIESI------------------------------------
----------------------------------------EQLKDFASQIQQ--------------------
-------------------------------------------------------------------QIAKF
EKALLLLSSPDGIALSSSEDIHISADAQINQIAGDSINISTQKNVIAHAQNRLSLFAAQSGLKAVAAQGKVE
IQAQADALDVLSKLGITISSTDDKVIISSPKEVKITGGSSQITLNGSGIFPKTGGKFQVNAGQHLFMGGASA
NASAPELP------KAKPMQGALELLRSYGGDNFFKQNSYKVI--DSLGKQITGKLDGNGFAQVTGIAP-GP
AKVVFEKDNTSAWLQSSDFKRNYTWAEPVKSVQGLMKNALEAVGQNTMSQLQNNLLSTDKNSFKNLGKNTLA
NLAGQTVGQIKNQVTNTALNTVSKQLNLNLSAD----QMKSLGQMATNPSQSLEMLKEQGGDFLSDQMTAKL
FKTTNQESPIQQGALDTFVRSKK
>AB030_15
-MFNNIFQILESFGFLSQHRSVYLQFSDASLNSQVFLQRIDGQHYLNQGMTAELICLSTNAHIPLKTFIGVQ
VAVDQVTDRGSFFRTTGIITGASQGQSDGALTLYKLAISDPTYLWHKRRNSRVFMNKSVKEISEILFQEWQG
KSPLFASSLTLDLSGLK-QTYDVRPFVMQLNESDYDFLTRLWRSEGISWLIDEAELTVASNMDNIQPQKLRL
IDDNNQYQALTRRAIRYHRSSATEQFDSMTSLMADRSLQPTSIFVQRWQPDVLQQTDGAGSVQSKHQHSTNY
DNQSLSLEEAWHFSPAWMQDLNGEDGATSASNQQLEKFNQNLSAYYDAQSKQFIAKTTVRDTQVGYWFELNE
HPEIDQ-HESTDKEFLIIGKNYYNQNNLPKDLNQQIQTLVQQSDWQASNTD-------ERQANQLILQRRYI
PTTPAYNPQTHSPVAHPQRAKVVGPEGEEIYVDEWGRIKVRFLFTRSDDHSHDGGAGTNNNDTDSAWIDVLT
PWAGEGYGARFLPRIGEIVVIDFFNGDIDRPFVMGRIHEAQRQPTKFDNKGKLPDTKKLSGIRSKEVSGGGF
GQLRFDDTSGQISTQLQSSHGASQLNLGKLSHPKDKAESEDRGEGFELRTDQWGALRAGQGLLVSTHKQDNA
KGDHLDAEVAKKQLEGSQTNSKALSDIAKNQKTDEIESI------------------------------------
----------------------------------------EQLKDFASQIQQ--------------------
-------------------------------------------------------------------QIAKF

EKALLLLSSPDGIALSSSEDIHISADAQINQIAGDSINISTQKNVIAHAQNRLSLFAAQSGLKAVAAQGKVE
IQAQADALDVLSKLGITISSTDDKVIISSPKEVKITGGSSQITLNGSGIFPKTGGKFQVNAGQHLFMAGASA
NASAPELP------KAKPMQGALELLRSYGGDNFFKQNSYKVI--DSLGKQITGKLDGNGFAQVTGIAP-GP
AKVVFEKDNTSAWLQSSDFKRNYTWAEPVKSVQGLMKNALEAVGQNTMSQLQNNLLSTDKNSFKNLGKNTLA
NLAGQTVGQIKNQVTNTALNTVSKQLNLNLSAD----QMKSLGQMATNPSQSLEMLKEQG-D----------
--------------LDTFVRSKQ
>AbH12O-A2_15
-MFNNIFQILESFGFLSQHRSVYLQFSDASLNSQVFLQRIDGQHYLNQGMTAELICLSTNAHIPLKTFIGVQ
VAVDQVTDRGSFFRTTGIITGASQGQSDGALTLYKLAISDPTYLWHKRRNSRVFMNKSVKEISEILFQEWQG
KSPLFASSLTLDLSGLK-QTYDVRPFVMQLNESDYDFLTRLWRSEGISWLIDEAELTVASNMDNIQPQKLRL
IDDNNQYQALTRRAIRYHRSSATEQFDSMTSLMADRSLQPTSIFVQRWQPDVLQQTDGAGSVQSKHQHSTNY
DNQSLSLEEAWHFSPAWMQDLNGEDGATSASNQQLEKFNQNLSAYYDAQSKQFIAKTTVRDTQVGYWFELNE
HPEIDQ-HESTDKEFLIIGKNYYNQNNLPKDLNQQIQTLVQQSDWQASNTD-------ERQANQLILQRRYI
PTTPAYNPQTHSPVAHPQRAKVVGPEGEEIYVDEWGRIKVRFLFTRSDDHSHDGGAGTNNNDTDSAWIDVLT
PWAGEGYGARFLPRIGEIVVIDFFNGDIDRPFVMGRIHEAQRQPTKFDNKGKLPDTKKLSGIRSKEVSGGGF
GQLRFDDTSGQISTQLQSSHGASQLNLGKLSHPKDKAESEDRGEGFELRTDQWGALRAGQGLLVSTHKQDNA
KGDHLDAEVAKKQLEGSQTNSKALSDIAKNQKTDEIESI---------------------------------
----------------------------------------EQLKDFASQIQQ--------------------
-------------------------------------------------------------------QIAKF
EKALLLLSSPDGIALSSSEDIHISADAQINQIAGDSINISTQKNVIAHAQNRLSLFAAQSGLKAVAAQGKVE
IQAQADALDVLSKLGITISSTDDKVIISSPKEVKITGGSSQITLNGSGIFPKTGGKFQVNAGQHLFMAGASA
NASAPELP------KAKPMQGALELLRSYGGDNFFKQNSYKVI--DSLGKQITGKLDGNGFAQVTGIAP-GP
AKVVFEKDNTSAWLQSSDFKRNYTWAEPVKSVQGLMKNALEAVGQNTMSQLQNNLLSTDKNSFKNLGKNTLA
NLAGQTVGQIKNQVTNTALNTVSKQLNLNLSAD----QMKSLGQMATNPSQSLEMLKEQG-D----------
--------------LDTFVRSKQ
>AF-401_15
-MFNNIFQILESFGFLSQHRSVYLQFSDASLNSQVFLQRIDGQHYLNQGMTAELICLSTNAHIPLKTFIGVQ
VAVDQVTDRGSFFRTTGIITGASQGQSDGALTLYKLAISDPTYLWHKRRNSRVFMNKSVKEISEILFQEWQG
KSPLFASSLTLDLSGLK-QTYDVRPFVMQLNESDYDFLTRLWRSEGISWLIDEAELTVASNMDNIQPQKLRL
IDDNNQYQALTRRAIRYHRSSATEQFDSMTSLMADRSLQPTSIFVQRWQPDVLQQTDGAGSVQSKHQHSTNY
DNQSLSLEEAWHFSPAWMQDLNGEDGATSASNQQLEKFNQNLSAYYDAQSKQFIAKTTVRDTQVGYWFELNE
HPEIDQ-HESTDKEFLIIGKNYYNQNNLPKDLNQQIQTLVQQSDWQASNTD-------ERQANQLILQRRYI
PTTPAYNPQTHSPVAHPQRAKVVGPEGEEIYVDEWGRIKVRFLFTRSDDHSHDGGAGTNNNDTDSAWIDVLT

PWAGEGYGARFLPRIGEIVVIDFFNGDIDRPFVMGRIHEAQRQPTKFDNKGKLPDTKKLSGIRSKEVSGGGF
GQLRFDDTSGQISTQLQSSHGASQLNLGKLSHPKDKAESEDRGEGFELRTDQWGALRAGQGLLVSTHKQDNA
KGDHLDAEVAKKQLEGSQTNSKALSDIAKNQKTDEIESI---------------------------------
----------------------------------------EQLKDFASQIQQ--------------------
-------------------------------------------------------------------QIAKF
EKALLLLSSPDGIALSSSEDIHISADAQINQIAGDSINISTQKNVIAHAQNRLSLFAAQSGLKAVAAQGKVE
IQAQADALDVLSKLGITISSTDDKVIISSPKEVKITGGSSQITLNGSGIFPKTGGKFQVNAGQHLFMAGASA
NASAPELP------KAKPMQGALELLRSYGGDNFFKQNSYKVI--DSLGKQITGKLDGNGFAQVTGIAP-GP
AKVVFEKDNTSAWLQSSDFKRNYTWAEPVKSVQGLMKNALEAVGQNTMSQLQNNLLSTDKNSFKNLGKNTLA
NLAGQTVGQIKNQVTNTALNTVSKQLNLNLSAD----QMKSLGQMATNPSQSLEMLKEQG-D----------
--------------LDTFVRSKQ
>AR_0063_15
-MFNNIFQILESFGFLSQHRSVYLQFSDASLNSQVFLQRIDGQHYLNQGMTAELICLSTNAHIPLKTFIGVQ
VAVDQVTDRGSFFRTTGIITGASQGQSDGALTLYKLAISDPTYLWHKRRNSRVFMNKSVKEISEILFQEWQG
KSPLFASSLTLDLSGLK-QTYDVRPFVMQLNESDYDFLTRLWRSEGISWLIDEAELTVASNMDNIQPQKLRL
IDDNNQYQALTRRAIRYHRSSATEQFDSMTSLMADRSLQPTSIFVQRWQPDVLQQTDGAGSVQSKHQHSTNY
DNQSLSLEEAWHFSPAWMQDLNGEDGATSASNQQLEKFNQNLSAYYDAQSKQFIAKTTVRDTQVGYWFELNE
HPEIDQ-HESTDKEFLIIGKNYYNQNNLPKDLNQQIQTLVQQSDWQASNTD-------ERQANQLILQRRYI
PTTPAYNPQTHSPVAHPQRAKVVGPEGEEIYVDEWGRIKVRFLFTRSDDHSHDGGAGTNNNDTDSAWIDVLT
PWAGEGYGARFLPRIGEIVVIDFFNGDIDRPFVMGRIHEAQRQPTKFDNKGKLPDTKKLSGIRSKEVSGGGF
GQLRFDDTSGQISTQLQSSHGASQLNLGKLSHPKDKAESEDRGEGFELRTDQWGALRAGQGLLVSTHKQDNA
KGDHLDAEVAKKQLEGSQTNSKALSDIAKNQKTDEIESI---------------------------------
----------------------------------------EQLKDFASQIQQ--------------------
-------------------------------------------------------------------QIAKF
EKALLLLSSPDGIALSSSEDIHISADAQINQIAGDSINISTQKNVIAHAQNRLSLFAAQSGLKAVAAQGKVE
IQAQADALDVLSKLGITISSTDDKVIISSPKEVKITGGSSQITLNGSGIFPKTGGKFQVNAGQHLFMAGASA
NASAPELP------KAKPMQGALELLRSYGGDNFFKQNSYKVI--DSLGKQITGKLDGNGFAQVTGIAP-GP
AKVVFEKDNTSAWLQSSDFKRNYTWAEPVKSVQGLMKNALEAVGQNTMSQLQNNLLSTDKNSFKNLGKNTLA
NLAGQTVGQIKNQVTNTALNTVSKQLNLNLSAD----QMKSLGQMATNPSQSLEMLKEQG-D----------
--------------LDTFVRSKQ
>AR_0101_15
-MFNNIFQILESFGFLSQHRSVYLQFSDASLNSQVFLQRIDGQHYLNQGMTAELICLSTNAHIPLKTFIGVQ
VAVDQVTDRGSFFRTTGIITGASQGQSDGALTLYKLAISDPTYLWHKRRNSRVFMNKSVKEISEILFQEWQG

KSPLFASSLTLDLSGLK-QTYDVRPFVMQLNESDYDFLTRLWRSEGISWLIDEAELTVASNMDNIQPQKLRL
IDDNNQYQALTRRAIRYHRSSATEQFDSMTSLMADRSLQPTSIFVQRWQPDVLQQTDGAGSVQSKHQHSTNY
DNQSLSLEEAWHFSPAWMQDLNGEDGATSASNQQLEKFNQNLSAYYDAQSKQFIAKTTVRDTQVGYWFELNE
HPEIDQ-HESTDKEFLIIGKNYYNQNNLPKDLNQQIQTLVQQSDWQASNTD-------ERQANQLILQRRYI
PTTPAYNPQTHSPVAHPQRAKVVGPEGEEIYVDEWGRIKVRFLFTRSDDHSHDGGAGTNNNDTDSAWIDVLT
PWAGEGYGARFLPRIGEIVVIDFFNGDIDRPFVMGRIHEAQRQPTKFDNKGKLPDTKKLSGIRSKEVSGGGF
GQLRFDDTSGQISTQLQSSHGASQLNLGKLSHPKDKAESEDRGEGFELRTDQWGALRAGQGLLVSTHKQDNA
KGDHLDAEVAKKQLEGSQTNSKALSDIAKNQKTDEIESI---------------------------------
----------------------------------------EQLKDFASQIQQ--------------------
-------------------------------------------------------------------QIAKF
EKALLLLSSPDGIALSSSEDIHISADAQINQIAGDSINISTQKNVIAHAQNRLSLFAAQSGLKAVAAQGKVE
IQAQADALDVLSKLGITISSTDDKVIISSPKEVKITGGSSQITLNGSGIFPKTGGKFQVNAGQHLFMAGASA
NASAPELP------KAKPMQGALELLRSYGGDNFFKQNSYKVI--DSLGKQITGKLDGNGFAQVTGIAP-GP
AKVVFEKDNTSAWLQSSDFKRNYTWAEPVKSVQGLMKNALEAVGQNTMSQLQNNLLSTDKNSFKNLGKNTLA
NLAGQTVGQIKNQVTNTALNTVSKQLNLNLSAD----QMKSLGQMATNPSQSLEMLKEQG-D----------
--------------LDTFVRSKQ
>6200_15
-MFNNIFQILESFGFLSQHRSVYLQFSDASLNSQVFLQRIDGQHYLNQGMTAELICLSTNAHIPLKTFIGVQ
VAVDQVTDRGSFFRTTGIITGASQGQSDGALTLYKLAISDPTYLWHKRRNSRVFMNKSVKEISEILFQEWQG
KSPLFASSLTLDLSGLK-QTYDVRPFVMQLNESDYDFLTRLWRSEGISWLIDEAELTVASNMDNIQPQKLRL
IDDNNQYQALTRRAIRYHRSSATEQFDSMTSLMADRSLQPTSIFVQRWQPDVLQQTDGAGSVQSKHQHSTNY
DNQSLSLEEAWHFSPAWMQDLNGEDGATSASNQQLEKFNQNLSAYYDAQSKQFIAKTTVRDTQVGYWFELNE
HPEIDQ-HESTDKEFLIIGKNYYNQNNLPKDLNQQIQTLVQQSDWQASNTD-------ERQANQLILQRRYI
PTTPAYNPQTHSPVAHPQRAKVVGPEGEEIYVDEWGRIKVRFLFTRSDDHSHDGGAGTNNNDTDSAWIDVLT
PWAGEGYGARFLPRIGEIVVINFFNGDIDRPFVMGRVHEAQRYPTKFDNKGKLPDTKKLSGIRSKEVSGSGF
GQLRFDDTPGQISTQLQSSHGASQLNLGKLSHPKDKAESEDRGEGFELRTDQWGALRAGQGLLVSTHKQDNA
KGDHLDAEVAKKQLEGSQTNSKALSDIAKNQKTDEIESL---------------------------------
----------------------------------------EQLKDFASQIQQ--------------------
-------------------------------------------------------------------QIAKF
EKALLLLSSPDGIALSSSEDIHISADAQINQIAGDSINISTQKNVIAHAQNRLSLFAAQSGLKAVAAQGKVE
IQAQADALDVLSKLGITISSTDDKVIISSPKEVKITGGSSQITLNGSGIFPKTGGKFQVNAGQHLFMGGASA
NASAPELP------KAKPMQGALELLRSYGGDNFFKQNSYKVI--DSLGKQITGKLDGNGFAQVTGIAP-GP
AKVVFEKDNTSAWLQSSDFKRNYTWAEPVKSVQGLMKNALEAVGQNTMSQLQNNLLSTDKNSFKNLGKNTLD

```
NLAGQTVAQIKNQVTNTALNTVSKQLNLNLSAD----QMKSLGQMATNPSQSLEMLKEQGGDFLSDQMTAKL
FKTTNQESPIQQGNLDTFVRSKK
>TCDC-AB0715_5
-MFNNIFQILESFGFLSQHRSVYLQFSDASLNSQVFLQRIDGQHYLNQGMTAELICLSTNAHIPLKTFIGVQ
VAVDQVTDRGSFFRTTGIITGASQGQSDGALTLYKLAISDPTYLWHKRRNSRVFMNKSVKEISEILFQEWQG
KSPLFASSLTLDLSGLK-QTYDVRPFVMQLNESDYDFLTRLWRSEGISWLIDEAELTVASNMDNIQPQKLRL
IDDNNQYQALTRRAIRYHRSSATEQFDSMTSLMADRSLQPTSIFVQRWQPDVLQQTDGAGSVQSKHQHSTNY
DNQSLSLEEAWHFSPAWMQDLNGEDGATSASNQQLEKFNQNLSAYYDAQSKQFIAKTTVRDTQVGYWFELNE
HPEIDQ-HESTDKEFLIIGKNYYNQNNLPKDLNQQIQTLVQQSDWQASNTD-------ERQANQLILQRRYI
PTTPAYNPQTHSPVAHPQRAKVVGPEGEEIYVDEWGRIKVRFLFTRSDDHSHDGGAGTNNNDTDSAWIDVLT
PWAGEGYGARFLPRIGEIVVIDFFNGDIDRPFVMGRIHEAQRQPTKFDNKGKLPDTKKLSGIRSKEVSGGGF
GQLRFDDTPGQISTQLQSSHGASQLNLGKLSHPKDKAESEDRGEGFELRTDQWGALRAGQGLLVSTHKQDNA
KGDHLDAEVAKKQLEGSQTNSKALSDIAKNQKTDEIESI---------------------------------
---------------------------------------EQLKDFASQIQQ--------------------
---------------------------------------------------------------------QIAKF
EKALLLLSSPDGIALSSSEDIHISADAQINQIAGDSINISTQKNVIAHAQNRLSLFAAQSGLKAVAAQGKVE
IQAQADALDVLSKLGITISSTDDKVIISSPKEVKITGGSSQITLNGSGIFPKTGGKFQVNAGQHLFMGGASA
NASAPELP------KAKPMQGALELLRSYGGDNFFKQNSYKVI--DSLGKQITGKLDGNGFAQVTGIAP-GP
AKVVFEKDNTSAWLQSSDFKRNYTWAEPVKSVQGLMKNALEAVGQNTMSQLQNNLLSTDKNSFKNLGKNTLD
NLAGQTVAQIKNQVTNTALNTVSKQLNLNLSAD----QMKSLGQMATNPSQSLEMLKEQGGDFLSDQMTAKL
FKTTNQESPIQQGDLDTFVRSKK
>JBA13_0
-MFNNIFQILESFGFLSQHRSVYLQFSDASLNSQVFLQRIDGQHYLNQGMTAELICLSTNAHIPLKTFIGVQ
VAVDQVTDRGSFFRTTGIITGASQGQSDGALTLYKLAISDPTYLWHKRRNSRVFMNKSVKEISEILFQEWQG
KSPLFASSLTLDLSGLK-QTYDVRPFVMQLNESDYDFLTRLWRSEGISWLIDEAELTVASNMDNIQPQKLRL
IDDNNQYQALTRRAIRYHRSSATEQFDSMTSLMADRSLQPTSIFVQRWQPDVLQQTDGAGSVQSKHQHSTNY
DNQSLSLEEAWHFSPAWMQDLNGEDGATSASNQQLEKFNQNLSAYYDAQSKQFIAKTTVRDTQVGYWFELNE
HPEIDQ-HESTDKEFLIIGKNYYNQNNLPKDLNQQIQTLVQQSDWQASNTD-------ERQANQLILQRRYI
PTTPAYNPQTHSPVAHPQRAKVVGPEGEEIYVDEWGRIKVRFLFTRSDDHSHDGGAGTNNNDTDSAWIDVLT
PWAGEGYGARFLPRIGEIVVINFFNGDIDRPFVMGRVHEAQRHPTKFDNKGKLPDTKKLSGIRSKEVSGGGF
GQLRFDDTPGQISTQLQSSHGASQLNLGKLSHPKDKAESEDRGEGFELRTDQWGALRAGQGLLVSTHKQDNA
KGDHLDAEVAKKQLEGSQTNSKALSDIAKNQKTDEIESI---------------------------------
---------------------------------------EQLKDFASQIQQ--------------------
```

-------------------------------------------------------------------QIAKF
EKALLLLSSPDGIALSSSEDIHISADAQINQIAGDSINISTQKNVIAHAQNRLSLFAAQSGLKAVAAQGKVE
IQAQADALDVLSKLGITISSTDDKVIISSPKEVKITGGSSQITLNGSGIFPKTGGKFQVNAGQHLFMGGASA
NASAPELP------KAKPMQGALELLRSYGGDNFFKQNSYKVI--DSLGKQITGKLDGNGFAQVTGIAP-GP
AKVVFEKDNTSAWLQSSDFKRNYTWAEPVKSVQGLMKNALEAVGQNTMSQLQNNLLSTDKNSFKNLGKNTLD
NLAGQTVAQIKNQVTNTALNTVSKQLNLNLSAD----QMKSLGQMATNPSQSLEMLKEQGGDFLSDQMTAKL
FKTTNQESPIQQGDLDTFVRSKK
>KAB02_0
-MFNNIFQILESFGFLSQHRSVYLQFSDASLNSQVFLQRIDGQHYLNQGMTAELICLSTNAHIPLKTFIGVQ
VAVDQVTDRGSFFRTTGIITGASQGQSDGALTLYKLAISDPTYLWHKRRNSRVFMNKSVKEISEILFQEWQG
KSPLFASSLTLDLSGLK-QTYDVRPFVMQLNESDYDFLTRLWRSEGISWLIDEAELTVASNMDNIQPQKLRL
IDDNNQYQALTRRAIRYHRSSATEQFDSMTSLMADRSLQPTSIFVQRWQPDVLQQTDGAGSVQSKHQHSTNY
DNQSLSLEEAWHFSPAWMQDLNGEDGATSASNQQLEKFNQNLSAYYDAQSKQFIAKTTVRDTQVGYWFELNE
HPEIDQ-HESTDKEFLIIGKNYYNQNNLPKDLNQQIQTLVQQSDWQASNTD-------ERQANQLILQRRYI
PTTPAYNPQTHSPVAHPQRAKVVGPEGEEIYVDEWGRIKVRFLFTRSDDHSHDGGAGTNNNDTDSAWIDVLT
PWAGEGYGARFLPRIGEIVVINFFNGDIDRPFVMGRVHEAQRHPTKFDNKGKLPDTKKLSGIRSKEVSGGGF
GQLRFDDTPGQISTQLQSSHGASQLNLGKLSHPKDKAESEDRGEGFELRTDQWGALRAGQGLLVSTHKQDNA
KGDHLDAEVAKKQLEGSQTNSKALSDIAKNQKTDEIESI---------------------------------
----------------------------------------EQLKDFASQIQQ--------------------
-------------------------------------------------------------------QIAKF
EKALLLLSSPDGIALSSSEDIHISADAQINQIAGDSINISTQKNVIAHAQNRLSLFAAQSGLKAVAAQGKVE
IQAQADALDVLSKLGITISSTDDKVIISSPKEVKITGGSSQITLNGSGIFPKTGGKFQVNAGQHLFMGGASA
NASAPELP------KAKPMQGALELLRSYGGDNFFKQNSYKVI--DSLGKQITGKLDGNGFAQVTGIAP-GP
AKVVFEKDNTSAWLQSSDFKRNYTWAEPVKSVQGLMKNALEAVGQNTMSQLQNNLLSTDKNSFKNLGKNTLD
NLAGQTVAQIKNQVTNTALNTVSKQLNLNLSAD----QMKSLGQMATNPSQSLEMLKEQGGDFLSDQMTAKL
FKTTNQESPIQQGDLDTFVRSKK
>KAB04_0
-MFNNIFQILESFGFLSQHRSVYLQFSDASLNSQVFLQRIDGQHYLNQGMTAELICLSTNAHIPLKTFIGVQ
VAVDQVTDRGSFFRTTGIITGASQGQSDGALTLYKLAISDPTYLWHKRRNSRVFMNKSVKEISEILFQEWQG
KSPLFASSLTLDLSGLK-QTYDVRPFVMQLNESDYDFLTRLWRSEGISWLIDEAELTVASNMDNIQPQKLRL
IDDNNQYQALTRRAIRYHRSSATEQFDSMTSLMADRSLQPTSIFVQRWQPDVLQQTDGAGSVQSKHQHSTNY
DNQSLSLEEAWHFSPAWMQDLNGEDGATSASNQQLEKFNQNLSAYYDAQSKQFIAKTTVRDTQVGYWFELNE
HPEIDQ-HESTDKEFLIIGKNYYNQNNLPKDLNQQIQTLVQQSDWQASNTD-------ERQANQLILQRRYI

PTTPAYNPQTHSPVAHPQRAKVVGPEGEEIYVDEWGRIKVRFLFTRSDDHSHDGGAGTNNNDTDSAWIDVLT
PWAGEGYGARFLPRIGEIVVINFFNGDIDRPFVMGRVHEAQRHPTKFDNKGKLPDTKKLSGIRSKEVSGGGF
GQLRFDDTPGQISTQLQSSHGASQLNLGKLSHPKDKAESEDRGEGFELRTDQWGALRAGQGLLVSTHKQDNA
KGDHLDAEVAKKQLEGSQTNSKALSDIAKNQKTDEIESI---------------------------------
----------------------------------------EQLKDFASQIQQ--------------------
-------------------------------------------------------------------QIAKF
EKALLLLSSPDGIALSSSEDIHISADAQINQIAGDSINISTQKNVIAHAQNRLSLFAAQSGLKAVAAQGKVE
IQAQADALDVLSKLGITISSTDDKVIISSPKEVKITGGSSQITLNGSGIFPKTGGKFQVNAGQHLFMGGASA
NASAPELP------KAKPMQGALELLRSYGGDNFFKQNSYKVI--DSLGKQITGKLDGNGFAQVTGIAP-GP
AKVVFEKDNTSAWLQSSDFKRNYTWAEPVKSVQGLMKNALEAVGQNTMSQLQNNLLSTDKNSFKNLGKNTLD
NLAGQTVAQIKNQVTNTALNTVSKQLNLNLSAD----QMKSLGQMATNPSQSLEMLKEQGGDFLSDQMTAKL
FKTTNQESPIQQGDLDTFVRSKK
>KAB05_0
-MFNNIFQILESFGFLSQHRSVYLQFSDASLNSQVFLQRIDGQHYLNQGMTAELICLSTNAHIPLKTFIGVQ
VAVDQVTDRGSFFRTTGIITGASQGQSDGALTLYKLAISDPTYLWHKRRNSRVFMNKSVKEISEILFQEWQG
KSPLFASSLTLDLSGLK-QTYDVRPFVMQLNESDYDFLTRLWRSEGISWLIDEAELTVASNMDNIQPQKLRL
IDDNNQYQALTRRAIRYHRSSATEQFDSMTSLMADRSLQPTSIFVQRWQPDVLQQTDGAGSVQSKHQHSTNY
DNQSLSLEEAWHFSPAWMQDLNGEDGATSASNQQLEKFNQNLSAYYDAQSKQFIAKTTVRDTQVGYWFELNE
HPEIDQ-HESTDKEFLIIGKNYYNQNNLPKDLNQQIQTLVQQSDWQASNTD-------ERQANQLILQRRYI
PTTPAYNPQTHSPVAHPQRAKVVGPEGEEIYVDEWGRIKVRFLFTRSDDHSHDGGAGTNNNDTDSAWIDVLT
PWAGEGYGARFLPRIGEIVVINFFNGDIDRPFVMGRVHEAQRHPTKFDNKGKLPDTKKLSGIRSKEVSGGGF
GQLRFDDTPGQISTQLQSSHGASQLNLGKLSHPKDKAESEDRGEGFELRTDQWGALRAGQGLLVSTHKQDNA
KGDHLDAEVAKKQLEGSQTNSKALSDIAKNQKTDEIESI---------------------------------
----------------------------------------EQLKDFASQIQQ--------------------
-------------------------------------------------------------------QIAKF
EKALLLLSSPDGIALSSSEDIHISADAQINQIAGDSINISTQKNVIAHAQNRLSLFAAQSGLKAVAAQGKVE
IQAQADALDVLSKLGITISSTDDKVIISSPKEVKITGGSSQITLNGSGIFPKTGGKFQVNAGQHLFMGGASA
NASAPELP------KAKPMQGALELLRSYGGDNFFKQNSYKVI--DSLGKQITGKLDGNGFAQVTGIAP-GP
AKVVFEKDNTSAWLQSSDFKRNYTWAEPVKSVQGLMKNALEAVGQNTMSQLQNNLLSTDKNSFKNLGKNTLD
NLAGQTVAQIKNQVTNTALNTVSKQLNLNLSAD----QMKSLGQMATNPSQSLEMLKEQGGDFLSDQMTAKL
FKTTNQESPIQQGDLDTFVRSKK
>KAB06_0
-MFNNIFQILESFGFLSQHRSVYLQFSDASLNSQVFLQRIDGQHYLNQGMTAELICLSTNAHIPLKTFIGVQ

VAVDQVTDRGSFFRTTGIITGASQGQSDGALTLYKLAISDPTYLWHKRRNSRVFMNKSVKEISEILFQEWQG
KSPLFASSLTLDLSGLK-QTYDVRPFVMQLNESDYDFLTRLWRSEGISWLIDEAELTVASNMDNIQPQKLRL
IDDNNQYQALTRRAIRYHRSSATEQFDSMTSLMADRSLQPTSIFVQRWQPDVLQQTDGAGSVQSKHQHSTNY
DNQSLSLEEAWHFSPAWMQDLNGEDGATSASNQQLEKFNQNLSAYYDAQSKQFIAKTTVRDTQVGYWFELNE
HPEIDQ-HESTDKEFLIIGKNYYNQNNLPKDLNQQIQTLVQQSDWQASNTD-------ERQANQLILQRRYI
PTTPAYNPQTHSPVAHPQRAKVVGPEGEEIYVDEWGRIKVRFLFTRSDDHSHDGGAGTNNNDTDSAWIDVLT
PWAGEGYGARFLPRIGEIVVINFFNGDIDRPFVMGRVHEAQRHPTKFDNKGKLPDTKKLSGIRSKEVSGGGF
GQLRFDDTPGQISTQLQSSHGASQLNLGKLSHPKDKAESEDRGEGFELRTDQWGALRAGQGLLVSTHKQDNA
KGDHLDAEVAKKQLEGSQTNSKALSDIAKNQKTDEIESI---------------------------------
----------------------------------------EQLKDFASQIQQ--------------------
-------------------------------------------------------------------QIAKF
EKALLLLSSPDGIALSSSEDIHISADAQINQIAGDSINISTQKNVIAHAQNRLSLFAAQSGLKAVAAQGKVE
IQAQADALDVLSKLGITISSTDDKVIISSPKEVKITGGSSQITLNGSGIFPKTGGKFQVNAGQHLFMGGASA
NASAPELP------KAKPMQGALELLRSYGGDNFFKQNSYKVI--DSLGKQITGKLDGNGFAQVTGIAP-GP
AKVVFEKDNTSAWLQSSDFKRNYTWAEPVKSVQGLMKNALEAVGQNTMSQLQNNLLSTDKNSFKNLGKNTLD
NLAGQTVAQIKNQVTNTALNTVSKQLNLNLSAD----QMKSLGQMATNPSQSLEMLKEQGGDFLSDQMTAKL
FKTTNQESPIQQGDLDTFVRSKK
>KAB07_0
-MFNNIFQILESFGFLSQHRSVYLQFSDASLNSQVFLQRIDGQHYLNQGMTAELICLSTNAHIPLKTFIGVQ
VAVDQVTDRGSFFRTTGIITGASQGQSDGALTLYKLAISDPTYLWHKRRNSRVFMNKSVKEISEILFQEWQG
KSPLFASSLTLDLSGLK-QTYDVRPFVMQLNESDYDFLTRLWRSEGISWLIDEAELTVASNMDNIQPQKLRL
IDDNNQYQALTRRAIRYHRSSATEQFDSMTSLMADRSLQPTSIFVQRWQPDVLQQTDGAGSVQSKHQHSTNY
DNQSLSLEEAWHFSPAWMQDLNGEDGATSASNQQLEKFNQNLSAYYDAQSKQFIAKTTVRDTQVGYWFELNE
HPEIDQ-HESTDKEFLIIGKNYYNQNNLPKDLNQQIQTLVQQSDWQASNTD-------ERQANQLILQRRYI
PTTPAYNPQTHSPVAHPQRAKVVGPEGEEIYVDEWGRIKVRFLFTRSDDHSHDGGAGTNNNDTDSAWIDVLT
PWAGEGYGARFLPRIGEIVVINFFNGDIDRPFVMGRVHEAQRHPTKFDNKGKLPDTKKLSGIRSKEVSGGGF
GQLRFDDTPGQISTQLQSSHGASQLNLGKLSHPKDKAESEDRGEGFELRTDQWGALRAGQGLLVSTHKQDNA
KGDHLDAEVAKKQLEGSQTNSKALSDIAKNQKTDEIESI---------------------------------
----------------------------------------EQLKDFASQIQQ--------------------
-------------------------------------------------------------------QIAKF
EKALLLLSSPDGIALSSSEDIHISADAQINQIAGDSINISTQKNVIAHAQNRLSLFAAQSGLKAVAAQGKVE
IQAQADALDVLSKLGITISSTDDKVIISSPKEVKITGGSSQITLNGSGIFPKTGGKFQVNAGQHLFMGGASA
NASAPELP------KAKPMQGALELLRSYGGDNFFKQNSYKVI--DSLGKQITGKLDGNGFAQVTGIAP-GP

AKVVFEKDNTSAWLQSSDFKRNYTWAEPVKSVQGLMKNALEAVGQNTMSQLQNNLLSTDKNSFKNLGKNTLD
NLAGQTVAQIKNQVTNTALNTVSKQLNLNLSAD----QMKSLGQMATNPSQSLEMLKEQGGDFLSDQMTAKL
FKTTNQESPIQQGDLDTFVRSKK
>KAB08_0
-MFNNIFQILESFGFLSQHRSVYLQFSDASLNSQVFLQRIDGQHYLNQGMTAELICLSTNAHIPLKTFIGVQ
VAVDQVTDRGSFFRTTGIITGASQGQSDGALTLYKLAISDPTYLWHKRRNSRVFMNKSVKEISEILFQEWQG
KSPLFASSLTLDLSGLK-QTYDVRPFVMQLNESDYDFLTRLWRSEGISWLIDEAELTVASNMDNIQPQKLRL
IDDNNQYQALTRRAIRYHRSSATEQFDSMTSLMADRSLQPTSIFVQRWQPDVLQQTDGAGSVQSKHQHSTNY
DNQSLSLEEAWHFSPAWMQDLNGEDGATSASNQQLEKFNQNLSAYYDAQSKQFIAKTTVRDTQVGYWFELNE
HPEIDQ-HESTDKEFLIIGKNYYNQNNLPKDLNQQIQTLVQQSDWQASNTD-------ERQANQLILQRRYI
PTTPAYNPQTHSPVAHPQRAKVVGPEGEEIYVDEWGRIKVRFLFTRSDDHSHDGGAGTNNNDTDSAWIDVLT
PWAGEGYGARFLPRIGEIVVINFFNGDIDRPFVMGRVHEAQRHPTKFDNKGKLPDTKKLSGIRSKEVSGGGF
GQLRFDDTPGQISTQLQSSHGASQLNLGKLSHPKDKAESEDRGEGFELRTDQWGALRAGQGLLVSTHKQDNA
KGDHLDAEVAKKQLEGSQTNSKALSDIAKNQKTDEIESI---------------------------------
----------------------------------------EQLKDFASQIQQ--------------------
-------------------------------------------------------------------QIAKF
EKALLLLSSPDGIALSSSEDIHISADAQINQIAGDSINISTQKNVIAHAQNRLSLFAAQSGLKAVAAQGKVE
IQAQADALDVLSKLGITISSTDDKVIISSPKEVKITGGSSQITLNGSGIFPKTGGKFQVNAGQHLFMGGASA
NASAPELP------KAKPMQGALELLRSYGGDNFFKQNSYKVI--DSLGKQITGKLDGNGFAQVTGIAP-GP
AKVVFEKDNTSAWLQSSDFKRNYTWAEPVKSVQGLMKNALEAVGQNTMSQLQNNLLSTDKNSFKNLGKNTLD
NLAGQTVAQIKNQVTNTALNTVSKQLNLNLSAD----QMKSLGQMATNPSQSLEMLKEQGGDFLSDQMTAKL
FKTTNQESPIQQGDLDTFVRSKK
>SAA14_0
-MFNNIFQILESFGFLSQHRSVYLQFSDASLNSQVFLQRIDGQHYLNQGMTAELICLSTNAHIPLKTFIGVQ
VAVDQVTDRGSFFRTTGIITGASQGQSDGALTLYKLAISDPTYLWHKRRNSRVFMNKSVKEISEILFQEWQG
KSPLFASSLTLDLSGLK-QTYDVRPFVMQLNESDYDFLTRLWRSEGISWLIDEAELTVASNMDNIQPQKLRL
IDDNNQYQALTRRAIRYHRSSATEQFDSMTSLMADRSLQPTSIFVQRWQPDVLQQTDGAGSVQSKHQHSTNY
DNQSLSLEEAWHFSPAWMQDLNGEDGATSASNQQLEKFNQNLSAYYDAQSKQFIAKTTVRDTQVGYWFELNE
HPEIDQ-HESTDKEFLIIGKNYYNQNNLPKDLNQQIQTLVQQSDWQASNTD-------ERQANQLILQRRYI
PTTPAYNPQTHSPVAHPQRAKVVGPEGEEIYVDEWGRIKVRFLFTRSDDHSHDGGAGTNNNDTDSAWIDVLT
PWAGEGYGARFLPRIGEIVVINFFNGDIDRPFVMGRVHEAQRHPTKFDNKGKLPDTKKLSGIRSKEVSGGGF
GQLRFDDTPGQISTQLQSSHGASQLNLGKLSHPKDKAESEDRGEGFELRTDQWGALRAGQGLLVSTHKQDNA
KGDHLDAEVAKKQLEGSQTNSKALSDIAKNQKTDEIESI---------------------------------

----------------------------------------EQLKDFASQIQQ--------------------
-------------------------------------------------------------------QIAKF
EKALLLLSSPDGIALSSSEDIHISADAQINQIAGDSINISTQKNVIAHAQNRLSLFAAQSGLKAVAAQGKVE
IQAQADALDVLSKLGITISSTDDKVIISSPKEVKITGGSSQITLNGSGIFPKTGGKFQVNAGQHLFMGGASA
NASAPELP------KAKPMQGALELLRSYGGDNFFKQNSYKVI--DSLGKQITGKLDGNGFAQVTGIAP-GP
AKVVFEKDNTSAWLQSSDFKRNYTWAEPVKSVQGLMKNALEAVGQNTMSQLQNNLLSTDKNSFKNLGKNTLD
NLAGQTVAQIKNQVTNTALNTVSKQLNLNLSAD----QMKSLGQMATNPSQSLEMLKEQGGDFLSDQMTAKL
FKTTNQESPIQQGDLDTFVRSKK
>SSA12_0
-MFNNIFQILESFGFLSQHRSVYLQFSDASLNSQVFLQRIDGQHYLNQGMTAELICLSTNAHIPLKTFIGVQ
VAVDQVTDRGSFFRTTGIITGASQGQSDGALTLYKLAISDPTYLWHKRRNSRVFMNKSVKEISEILFQEWQG
KSPLFASSLTLDLSGLK-QTYDVRPFVMQLNESDYDFLTRLWRSEGISWLIDEAELTVASNMDNIQPQKLRL
IDDNNQYQALTRRAIRYHRSSATEQFDSMTSLMADRSLQPTSIFVQRWQPDVLQQTDGAGSVQSKHQHSTNY
DNQSLSLEEAWHFSPAWMQDLNGEDGATSASNQQLEKFNQNLSAYYDAQSKQFIAKTTVRDTQVGYWFELNE
HPEIDQ-HESTDKEFLIIGKNYYNQNNLPKDLNQQIQTLVQQSDWQASNTD-------ERQANQLILQRRYI
PTTPAYNPQTHSPVAHPQRAKVVGPEGEEIYVDEWGRIKVRFLFTRSDDHSHDGGAGTNNNDTDSAWIDVLT
PWAGEGYGARFLPRIGEIVVINFFNGDIDRPFVMGRVHEAQRHPTKFDNKGKLPDTKKLSGIRSKEVSGGGF
GQLRFDDTPGQISTQLQSSHGASQLNLGKLSHPKDKAESEDRGEGFELRTDQWGALRAGQGLLVSTHKQDNA
KGDHLDAEVAKKQLEGSQTNSKALSDIAKNQKTDEIESI---------------------------------
----------------------------------------EQLKDFASQIQQ--------------------
-------------------------------------------------------------------QIAKF
EKALLLLSSPDGIALSSSEDIHISADAQINQIAGDSINISTQKNVIAHAQNRLSLFAAQSGLKAVAAQGKVE
IQAQADALDVLSKLGITISSTDDKVIISSPKEVKITGGSSQITLNGSGIFPKTGGKFQVNAGQHLFMGGASA
NASAPELP------KAKPMQGALELLRSYGGDNFFKQNSYKVI--DSLGKQITGKLDGNGFAQVTGIAP-GP
AKVVFEKDNTSAWLQSSDFKRNYTWAEPVKSVQGLMKNALEAVGQNTMSQLQNNLLSTDKNSFKNLGKNTLD
NLAGQTVAQIKNQVTNTALNTVSKQLNLNLSAD----QMKSLGQMATNPSQSLEMLKEQGGDFLSDQMTAKL
FKTTNQESPIQQGDLDTFVRSKK
>SSMA17_0
-MFNNIFQILESFGFLSQHRSVYLQFSDASLNSQVFLQRIDGQHYLNQGMTAELICLSTNAHIPLKTFIGVQ
VAVDQVTDRGSFFRTTGIITGASQGQSDGALTLYKLAISDPTYLWHKRRNSRVFMNKSVKEISEILFQEWQG
KSPLFASSLTLDLSGLK-QTYDVRPFVMQLNESDYDFLTRLWRSEGISWLIDEAELTVASNMDNIQPQKLRL
IDDNNQYQALTRRAIRYHRSSATEQFDSMTSLMADRSLQPTSIFVQRWQPDVLQQTDGAGSVQSKHQHSTNY
DNQSLSLEEAWHFSPAWMQDLNGEDGATSASNQQLEKFNQNLSAYYDAQSKQFIAKTTVRDTQVGYWFELNE

HPEIDQ-HESTDKEFLIIGKNYYNQNNLPKDLNQQIQTLVQQSDWQASNTD-------ERQANQLILQRRYI
PTTPAYNPQTHSPVAHPQRAKVVGPEGEEIYVDEWGRIKVRFLFTRSDDHSHDGGAGTNNNDTDSAWIDVLT
PWAGEGYGARFLPRIGEIVVINFFNGDIDRPFVMGRVHEAQRHPTKFDNKGKLPDTKKLSGIRSKEVSGGGF
GQLRFDDTPGQISTQLQSSHGASQLNLGKLSHPKDKAESEDRGEGFELRTDQWGALRAGQGLLVSTHKQDNA
KGDHLDAEVAKKQLEGSQTNSKALSDIAKNQKTDEIESI---------------------------------
----------------------------------------EQLKDFASQIQQ--------------------
-------------------------------------------------------------------QIAKF
EKALLLLSSPDGIALSSSEDIHISADAQINQIAGDSINISTQKNVIAHAQNRLSLFAAQSGLKAVAAQGKVE
IQAQADALDVLSKLGITISSTDDKVIISSPKEVKITGGSSQITLNGSGIFPKTGGKFQVNAGQHLFMGGASA
NASAPELP------KAKPMQGALELLRSYGGDNFFKQNSYKVI--DSLGKQITGKLDGNGFAQVTGIAP-GP
AKVVFEKDNTSAWLQSSDFKRNYTWAEPVKSVQGLMKNALEAVGQNTMSQLQNNLLSTDKNSFKNLGKNTLD
NLAGQTVAQIKNQVTNTALNTVSKQLNLNLSAD----QMKSLGQMATNPSQSLEMLKEQGGDFLSDQMTAKL
FKTTNQESPIQQGDLDTFVRSKK
>15A34_5
-MFNNIFQILESFGFLSQHRSVYLQFSDASLNSQVFLQRIDGQHYLNQGMTAELICLSTNAHIPLKTFIGVQ
VAVDQVTDRGSFFRTTGIITGASQGQSDGALTLYKLAISDPTYLWHKRRNSRVFMNKSVKEISEILFQEWQG
KSPLFASSLTLDLSGLK-QTYDVRPFVMQLNESDYDFLTRLWRSEGISWLIDEAELTVASNMDNIQPQKLRL
IDDNNQYQALTRRAIRYHRSSATEQFDSMTSLMADRSLQPTSIFVQRWQPDVLQQTDGAGSVQSKHQHSTNY
DNQSLSLEEAWHFSPAWMQDLNGEDGATSASNQQLEKFNQNLSAYYDAQSKQFIAKTTVRDTQVGYWFELNE
HPEIDQ-HESTDKEFLIIGKNYYNQNNLPKDLNQQIQTLVQQSDWQASNTD-------ERQANQLILQRRYI
PTTPAYNPQTHSPVAHPQRAKVVGPEGEEIYVDEWGRIKVRFLFTRSDDHSHDGGAGTNNNDTDSAWIDVLT
PWAGEGYGARFLPRIGEIVVINFFNGDIDRPFVMGRVHEAQRHPTKFDNKGKLPDTKKLSGIRSKEVSGGGF
GQLRFDDTPGQISTQLQSSHGASQLNLGKLSHPKDKAESEDRGEGFELRTDQWGALRAGQGLLVSTHKQDNA
KGDHLDAEVAKKQLEGSQTNSKALSDIAKNQKTDEIESI---------------------------------
----------------------------------------EQLKDFASQIQQ--------------------
-------------------------------------------------------------------QIAKF
EKALLLLSSPDGIALSSSEDIHISADAQINQIAGDSINISTQKNVIAHAQNRLSLFAAQSGLKAVAAQGKVE
IQAQADALDVLSKLGITISSTDDKVIISSPKEVKITGGSSQITLNGSGIFPKTGGKFQVNAGQHLFMGGASA
NASAPELP------KAKPMQGALELLRSYGGDNFFKQNSYKVI--DSLGKQITGKLDGNGFAQVTGIAP-GP
AKVVFEKDNTSAWLQSSDFKRNYTWAEPVKSVQGLMKNALEAVGQNTMSQLQNNLLSTDKNSFKNLGKNTLD
NLAGQTVAQIKNQVTNTALNTVSKQLNLNLSAD----QMKSLGQMATNPSQSLEMLKEQGGDFLSDQMTAKL
FKTTNQESPIQQGDLDTFVRSKK
>15A5_5

-MFNNIFQILESFGFLSQHRSVYLQFSDASLNSQVFLQRIDGQHYLNQGMTAELICLSTNAHIPLKTFIGVQ
VAVDQVTDRGSFFRTTGIITGASQGQSDGALTLYKLAISDPTYLWHKRRNSRVFMNKSVKEISEILFQEWQG
KSPLFASSLTLDLSGLK-QTYDVRPFVMQLNESDYDFLTRLWRSEGISWLIDEAELTVASNMDNIQPQKLRL
IDDNNQYQALTRRAIRYHRSSATEQFDSMTSLMADRSLQPTSIFVQRWQPDVLQQTDGAGSVQSKHQHSTNY
DNQSLSLEEAWHFSPAWMQDLNGEDGATSASNQQLEKFNQNLSAYYDAQSKQFIAKTTVRDTQVGYWFELNE
HPEIDQ-HESTDKEFLIIGKNYYNQNNLPKDLNQQIQTLVQQSDWQASNTD-------ERQANQLILQRRYI
PTTPAYNPQTHSPVAHPQRAKVVGPEGEEIYVDEWGRIKVRFLFTRSDDHSHDGGAGTNNNDTDSAWIDVLT
PWAGEGYGARFLPRIGEIVVINFFNGDIDRPFVMGRVHEAQRHPTKFDNKGKLPDTKKLSGIRSKEVSGGGF
GQLRFDDTPGQISTQLQSSHGASQLNLGKLSHPKDKAESEDRGEGFELRTDQWGALRAGQGLLVSTHKQDNA
KGDHLDAEVAKKQLEGSQTNSKALSDIAKNQKTDEIESI---------------------------------
----------------------------------------EQLKDFASQIQQ--------------------
-------------------------------------------------------------------QIAKF
EKALLLLSSPDGIALSSSEDIHISADAQINQIAGDSINISTQKNVIAHAQNRLSLFAAQSGLKAVAAQGKVE
IQAQADALDVLSKLGITISSTDDKVIISSPKEVKITGGSSQITLNGSGIFPKTGGKFQVNAGQHLFMGGASA
NASAPELP------KAKPMQGALELLRSYGGDNFFKQNSYKVI--DSLGKQITGKLDGNGFAQVTGIAP-GP
AKVVFEKDNTSAWLQSSDFKRNYTWAEPVKSVQGLMKNALEAVGQNTMSQLQNNLLSTDKNSFKNLGKNTLD
NLAGQTVAQIKNQVTNTALNTVSKQLNLNLSAD----QMKSLGQMATNPSQSLEMLKEQGGDFLSDQMTAKL
FKTTNQESPIQQGDLDTFVRSKK
>3027STDY5784958_5
-MFNNIFQILESFGFLSQHRSVYLQFSDASLNSQVFLQRIDGQHYLNQGMTAELICLSTNAHIPLKTFIGVQ
VAVDQVTDRGSFFRTTGIITGASQGQSDGALTLYKLAISDPTYLWHKRRNSRVFMNKSVKEISEILFQEWQG
KSPLFASSLTLDLSGLK-QTYDVRPFVMQLNESDYDFLTRLWRSEGISWLIDEAELTVASNMDNIQPQKLRL
IDDNNQYQALTRRAIRYHRSSATEQFDSMTSLMADRSLQPTSIFVQRWQPDVLQQTDGAGSVQSKHQHSTNY
DNQSLSLEEAWHFSPAWMQDLNGEDGATSASNQQLEKFNQNLSAYYDAQSKQFIAKTTVRDTQVGYWFELNE
HPEIDQ-HESTDKEFLIIGKNYYNQNNLPKDLNQQIQTLVQQSDWQASNTD-------ERQANQLILQRRYI
PTTPAYNPQTHSPVAHPQRAKVVGPEGEEIYVDEWGRIKVRFLFTRSDDHSHDGGAGTNNNDTDSAWIDVLT
PWAGEGYGARFLPRIGEIVVINFFNGDIDRPFVMGRVHEAQRHPTKFDNKGKLPDTKKLSGIRSKEVSGGGF
GQLRFDDTPGQISTQLQSSHGASQLNLGKLSHPKDKAESEDRGEGFELRTDQWGALRAGQGLLVSTHKQDNA
KGDHLDAEVAKKQLEGSQTNSKALSDIAKNQKTDEIESI---------------------------------
----------------------------------------EQLKDFASQIQQ--------------------
-------------------------------------------------------------------QIAKF
EKALLLLSSPDGIALSSSEDIHISADAQINQIAGDSINISTQKNVIAHAQNRLSLFAAQSGLKAVAAQGKVE
IQAQADALDVLSKLGITISSTDDKVIISSPKEVKITGGSSQITLNGSGIFPKTGGKFQVNAGQHLFMGGASA

NASAPELP------KAKPMQGALELLRSYGGDNFFKQNSYKVI--DSLGKQITGKLDGNGFAQVTGIAP-GP
AKVVFEKDNTSAWLQSSDFKRNYTWAEPVKSVQGLMKNALEAVGQNTMSQLQNNLLSTDKNSFKNLGKNTLD
NLAGQTVAQIKNQVTNTALNTVSKQLNLNLSAD----QMKSLGQMATNPSQSLEMLKEQGGDFLSDQMTAKL
FKTTNQESPIQQGDLDTFVRSKK
>Ab4568_5
-MFNNIFQILESFGFLSQHRSVYLQFSDASLNSQVFLQRIDGQHYLNQGMTAELICLSTNAHIPLKTFIGVQ
VAVDQVTDRGSFFRTTGIITGASQGQSDGALTLYKLAISDPTYLWHKRRNSRVFMNKSVKEISEILFQEWQG
KSPLFASSLTLDLSGLK-QTYDVRPFVMQLNESDYDFLTRLWRSEGISWLIDEAELTVASNMDNIQPQKLRL
IDDNNQYQALTRRAIRYHRSSATEQFDSMTSLMADRSLQPTSIFVQRWQPDVLQQTDGAGSVQSKHQHSTNY
DNQSLSLEEAWHFSPAWMQDLNGEDGATSASNQQLEKFNQNLSAYYDAQSKQFIAKTTVRDTQVGYWFELNE
HPEIDQ-HESTDKEFLIIGKNYYNQNNLPKDLNQQIQTLVQQSDWQASNTD-------ERQANQLILQRRYI
PTTPAYNPQTHSPVAHPQRAKVVGPEGEEIYVDEWGRIKVRFLFTRSDDHSHDGGAGTNNNDTDSAWIDVLT
PWAGEGYGARFLPRIGEIVVINFFNGDIDRPFVMGRVHEAQRHPTKFDNKGKLPDTKKLSGIRSKEVSGGGF
GQLRFDDTPGQISTQLQSSHGASQLNLGKLSHPKDKAESEDRGEGFELRTDQWGALRAGQGLLVSTHKQDNA
KGDHLDAEVAKKQLEGSQTNSKALSDIAKNQKTDEIESI---------------------------------
---------------------------------------EQLKDFASQIQQ--------------------
-----------------------------------------------------------------QIAKF
EKALLLLSSPDGIALSSSEDIHISADAQINQIAGDSINISTQKNVIAHAQNRLSLFAAQSGLKAVAAQGKVE
IQAQADALDVLSKLGITISSTDDKVIISSPKEVKITGGSSQITLNGSGIFPKTGGKFQVNAGQHLFMGGASA
NASAPELP------KAKPMQGALELLRSYGGDNFFKQNSYKVI--DSLGKQITGKLDGNGFAQVTGIAP-GP
AKVVFEKDNTSAWLQSSDFKRNYTWAEPVKSVQGLMKNALEAVGQNTMSQLQNNLLSTDKNSFKNLGKNTLD
NLAGQTVAQIKNQVTNTALNTVSKQLNLNLSAD----QMKSLGQMATNPSQSLEMLKEQGGDFLSDQMTAKL
FKTTNQESPIQQGDLDTFVRSKK
>Ab4653_5
-MFNNIFQILESFGFLSQHRSVYLQFSDASLNSQVFLQRIDGQHYLNQGMTAELICLSTNAHIPLKTFIGVQ
VAVDQVTDRGSFFRTTGIITGASQGQSDGALTLYKLAISDPTYLWHKRRNSRVFMNKSVKEISEILFQEWQG
KSPLFASSLTLDLSGLK-QTYDVRPFVMQLNESDYDFLTRLWRSEGISWLIDEAELTVASNMDNIQPQKLRL
IDDNNQYQALTRRAIRYHRSSATEQFDSMTSLMADRSLQPTSIFVQRWQPDVLQQTDGAGSVQSKHQHSTNY
DNQSLSLEEAWHFSPAWMQDLNGEDGATSASNQQLEKFNQNLSAYYDAQSKQFIAKTTVRDTQVGYWFELNE
HPEIDQ-HESTDKEFLIIGKNYYNQNNLPKDLNQQIQTLVQQSDWQASNTD-------ERQANQLILQRRYI
PTTPAYNPQTHSPVAHPQRAKVVGPEGEEIYVDEWGRIKVRFLFTRSDDHSHDGGAGTNNNDTDSAWIDVLT
PWAGEGYGARFLPRIGEIVVINFFNGDIDRPFVMGRVHEAQRHPTKFDNKGKLPDTKKLSGIRSKEVSGGGF
GQLRFDDTPGQISTQLQSSHGASQLNLGKLSHPKDKAESEDRGEGFELRTDQWGALRAGQGLLVSTHKQDNA

KGDHLDAEVAKKQLEGSQTNSKALSDIAKNQKTDEIESI------------------------------------
----------------------------------------EQLKDFASQIQQ--------------------
----------------------------------------------------------------QIAKF
EKALLLLSSPDGIALSSSEDIHISADAQINQIAGDSINISTQKNVIAHAQNRLSLFAAQSGLKAVAAQGKVE
IQAQADALDVLSKLGITISSTDDKVIISSPKEVKITGGSSQITLNGSGIFPKTGGKFQVNAGQHLFMGGASA
NASAPELP------KAKPMQGALELLRSYGGDNFFKQNSYKVI--DSLGKQITGKLDGNGFAQVTGIAP-GP
AKVVFEKDNTSAWLQSSDFKRNYTWAEPVKSVQGLMKNALEAVGQNTMSQLQNNLLSTDKNSFKNLGKNTLD
NLAGQTVAQIKNQVTNTALNTVSKQLNLNLSAD----QMKSLGQMATNPSQSLEMLKEQGGDFLSDQMTAKL
FKTTNQESPIQQGDLDTFVRSKK
>Ab4977_5
-MFNNIFQILESFGFLSQHRSVYLQFSDASLNSQVFLQRIDGQHYLNQGMTAELICLSTNAHIPLKTFIGVQ
VAVDQVTDRGSFFRTTGIITGASQGQSDGALTLYKLAISDPTYLWHKRRNSRVFMNKSVKEISEILFQEWQG
KSPLFASSLTLDLSGLK-QTYDVRPFVMQLNESDYDFLTRLWRSEGISWLIDEAELTVASNMDNIQPQKLRL
IDDNNQYQALTRRAIRYHRSSATEQFDSMTSLMADRSLQPTSIFVQRWQPDVLQQTDGAGSVQSKHQHSTNY
DNQSLSLEEAWHFSPAWMQDLNGEDGATSASNQQLEKFNQNLSAYYDAQSKQFIAKTTVRDTQVGYWFELNE
HPEIDQ-HESTDKEFLIIGKNYYNQNNLPKDLNQQIQTLVQQSDWQASNTD-------ERQANQLILQRRYI
PTTPAYNPQTHSPVAHPQRAKVVGPEGEEIYVDEWGRIKVRFLFTRSDDHSHDGGAGTNNNDTDSAWIDVLT
PWAGEGYGARFLPRIGEIVVINFFNGDIDRPFVMGRVHEAQRHPTKFDNKGKLPDTKKLSGIRSKEVSGGGF
GQLRFDDTPGQISTQLQSSHGASQLNLGKLSHPKDKAESEDRGEGFELRTDQWGALRAGQGLLVSTHKQDNA
KGDHLDAEVAKKQLEGSQTNSKALSDIAKNQKTDEIESI------------------------------------
----------------------------------------EQLKDFASQIQQ--------------------
----------------------------------------------------------------QIAKF
EKALLLLSSPDGIALSSSEDIHISADAQINQIAGDSINISTQKNVIAHAQNRLSLFAAQSGLKAVAAQGKVE
IQAQADALDVLSKLGITISSTDDKVIISSPKEVKITGGSSQITLNGSGIFPKTGGKFQVNAGQHLFMGGASA
NASAPELP------KAKPMQGALELLRSYGGDNFFKQNSYKVI--DSLGKQITGKLDGNGFAQVTGIAP-GP
AKVVFEKDNTSAWLQSSDFKRNYTWAEPVKSVQGLMKNALEAVGQNTMSQLQNNLLSTDKNSFKNLGKNTLD
NLAGQTVAQIKNQVTNTALNTVSKQLNLNLSAD----QMKSLGQMATNPSQSLEMLKEQGGDFLSDQMTAKL
FKTTNQESPIQQGDLDTFVRSKK
>AF-673_5
-MFNNIFQILESFGFLSQHRSVYLQFSDASLNSQVFLQRIDGQHYLNQGMTAELICLSTNAHIPLKTFIGVQ
VAVDQVTDRGSFFRTTGIITGASQGQSDGALTLYKLAISDPTYLWHKRRNSRVFMNKSVKEISEILFQEWQG
KSPLFASSLTLDLSGLK-QTYDVRPFVMQLNESDYDFLTRLWRSEGISWLIDEAELTVASNMDNIQPQKLRL
IDDNNQYQALTRRAIRYHRSSATEQFDSMTSLMADRSLQPTSIFVQRWQPDVLQQTDGAGSVQSKHQHSTNY

DNQSLSLEEAWHFSPAWMQDLNGEDGATSASNQQLEKFNQNLSAYYDAQSKQFIAKTTVRDTQVGYWFELNE
HPEIDQ-HESTDKEFLIIGKNYYNQNNLPKDLNQQIQTLVQQSDWQASNTD-------ERQANQLILQRRYI
PTTPAYNPQTHSPVAHPQRAKVVGPEGEEIYVDEWGRIKVRFLFTRSDDHSHDGGAGTNNNDTDSAWIDVLT
PWAGEGYGARFLPRIGEIVVINFFNGDIDRPFVMGRVHEAQRHPTKFDNKGKLPDTKKLSGIRSKEVSGGGF
GQLRFDDTPGQISTQLQSSHGASQLNLGKLSHPKDKAESEDRGEGFELRTDQWGALRAGQGLLVSTHKQDNA
KGDHLDAEVAKKQLEGSQTNSKALSDIAKNQKTDEIESI-----------------------------------
----------------------------------------EQLKDFASQIQQ--------------------
-------------------------------------------------------------------QIAKF
EKALLLLSSPDGIALSSSEDIHISADAQINQIAGDSINISTQKNVIAHAQNRLSLFAAQSGLKAVAAQGKVE
IQAQADALDVLSKLGITISSTDDKVIISSPKEVKITGGSSQITLNGSGIFPKTGGKFQVNAGQHLFMGGASA
NASAPELP------KAKPMQGALELLRSYGGDNFFKQNSYKVI--DSLGKQITGKLDGNGFAQVTGIAP-GP
AKVVFEKDNTSAWLQSSDFKRNYTWAEPVKSVQGLMKNALEAVGQNTMSQLQNNLLSTDKNSFKNLGKNTLD
NLAGQTVAQIKNQVTNTALNTVSKQLNLNLSAD----QMKSLGQMATNPSQSLEMLKEQGGDFLSDQMTAKL
FKTTNQESPIQQGDLDTFVRSKK
>AR_0056_5
-MFNNIFQILESFGFLSQHRSVYLQFSDASLNSQVFLQRIDGQHYLNQGMTAELICLSTNAHIPLKTFIGVQ
VAVDQVTDRGSFFRTTGIITGASQGQSDGALTLYKLAISDPTYLWHKRRNSRVFMNKSVKEISEILFQEWQG
KSPLFASSLTLDLSGLK-QTYDVRPFVMQLNESDYDFLTRLWRSEGISWLIDEAELTVASNMDNIQPQKLRL
IDDNNQYQALTRRAIRYHRSSATEQFDSMTSLMADRSLQPTSIFVQRWQPDVLQQTDGAGSVQSKHQHSTNY
DNQSLSLEEAWHFSPAWMQDLNGEDGATSASNQQLEKFNQNLSAYYDAQSKQFIAKTTVRDTQVGYWFELNE
HPEIDQ-HESTDKEFLIIGKNYYNQNNLPKDLNQQIQTLVQQSDWQASNTD-------ERQANQLILQRRYI
PTTPAYNPQTHSPVAHPQRAKVVGPEGEEIYVDEWGRIKVRFLFTRSDDHSHDGGAGTNNNDTDSAWIDVLT
PWAGEGYGARFLPRIGEIVVINFFNGDIDRPFVMGRVHEAQRHPTKFDNKGKLPDTKKLSGIRSKEVSGGGF
GQLRFDDTPGQISTQLQSSHGASQLNLGKLSHPKDKAESEDRGEGFELRTDQWGALRAGQGLLVSTHKQDNA
KGDHLDAEVAKKQLEGSQTNSKALSDIAKNQKTDEIESI-----------------------------------
----------------------------------------EQLKDFASQIQQ--------------------
-------------------------------------------------------------------QIAKF
EKALLLLSSPDGIALSSSEDIHISADAQINQIAGDSINISTQKNVIAHAQNRLSLFAAQSGLKAVAAQGKVE
IQAQADALDVLSKLGITISSTDDKVIISSPKEVKITGGSSQITLNGSGIFPKTGGKFQVNAGQHLFMGGASA
NASAPELP------KAKPMQGALELLRSYGGDNFFKQNSYKVI--DSLGKQITGKLDGNGFAQVTGIAP-GP
AKVVFEKDNTSAWLQSSDFKRNYTWAEPVKSVQGLMKNALEAVGQNTMSQLQNNLLSTDKNSFKNLGKNTLD
NLAGQTVAQIKNQVTNTALNTVSKQLNLNLSAD----QMKSLGQMATNPSQSLEMLKEQGGDFLSDQMTAKL
FKTTNQESPIQQGDLDTFVRSKK

>AR_0102_5
-MFNNIFQILESFGFLSQHRSVYLQFSDASLNSQVFLQRIDGQHYLNQGMTAELICLSTNAHIPLKTFIGVQ
VAVDQVTDRGSFFRTTGIITGASQGQSDGALTLYKLAISDPTYLWHKRRNSRVFMNKSVKEISEILFQEWQG
KSPLFASSLTLDLSGLK-QTYDVRPFVMQLNESDYDFLTRLWRSEGISWLIDEAELTVASNMDNIQPQKLRL
IDDNNQYQALTRRAIRYHRSSATEQFDSMTSLMADRSLQPTSIFVQRWQPDVLQQTDGAGSVQSKHQHSTNY
DNQSLSLEEAWHFSPAWMQDLNGEDGATSASNQQLEKFNQNLSAYYDAQSKQFIAKTTVRDTQVGYWFELNE
HPEIDQ-HESTDKEFLIIGKNYYNQNNLPKDLNQQIQTLVQQSDWQASNTD-------ERQANQLILQRRYI
PTTPAYNPQTHSPVAHPQRAKVVGPEGEEIYVDEWGRIKVRFLFTRSDDHSHDGGAGTNNNDTDSAWIDVLT
PWAGEGYGARFLPRIGEIVVINFFNGDIDRPFVMGRVHEAQRHPTKFDNKGKLPDTKKLSGIRSKEVSGGGF
GQLRFDDTPGQISTQLQSSHGASQLNLGKLSHPKDKAESEDRGEGFELRTDQWGALRAGQGLLVSTHKQDNA
KGDHLDAEVAKKQLEGSQTNSKALSDIAKNQKTDEIESI---------------------------------
---------------------------------------EQLKDFASQIQQ--------------------
-------------------------------------------------------------------QIAKF
EKALLLLSSPDGIALSSSEDIHISADAQINQIAGDSINISTQKNVIAHAQNRLSLFAAQSGLKAVAAQGKVE
IQAQADALDVLSKLGITISSTDDKVIISSPKEVKITGGSSQITLNGSGIFPKTGGKFQVNAGQHLFMGGASA
NASAPELP------KAKPMQGALELLRSYGGDNFFKQNSYKVI--DSLGKQITGKLDGNGFAQVTGIAP-GP
AKVVFEKDNTSAWLQSSDFKRNYTWAEPVKSVQGLMKNALEAVGQNTMSQLQNNLLSTDKNSFKNLGKNTLD
NLAGQTVAQIKNQVTNTALNTVSKQLNLNLSAD----QMKSLGQMATNPSQSLEMLKEQGGDFLSDQMTAKL
FKTTNQESPIQQGDLDTFVRSKK
>BJAB07104_5
-MFNNIFQILESFGFLSQHRSVYLQFSDASLNSQVFLQRIDGQHYLNQGMTAELICLSTNAHIPLKTFIGVQ
VAVDQVTDRGSFFRTTGIITGASQGQSDGALTLYKLAISDPTYLWHKRRNSRVFMNKSVKEISEILFQEWQG
KSPLFASSLTLDLSGLK-QTYDVRPFVMQLNESDYDFLTRLWRSEGISWLIDEAELTVASNMDNIQPQKLRL
IDDNNQYQALTRRAIRYHRSSATEQFDSMTSLMADRSLQPTSIFVQRWQPDVLQQTDGAGSVQSKHQHSTNY
DNQSLSLEEAWHFSPAWMQDLNGEDGATSASNQQLEKFNQNLSAYYDAQSKQFIAKTTVRDTQVGYWFELNE
HPEIDQ-HESTDKEFLIIGKNYYNQNNLPKDLNQQIQTLVQQSDWQASNTD-------ERQANQLILQRRYI
PTTPAYNPQTHSPVAHPQRAKVVGPEGEEIYVDEWGRIKVRFLFTRSDDHSHDGGAGTNNNDTDSAWIDVLT
PWAGEGYGARFLPRIGEIVVINFFNGDIDRPFVMGRVHEAQRHPTKFDNKGKLPDTKKLSGIRSKEVSGGGF
GQLRFDDTPGQISTQLQSSHGASQLNLGKLSHPKDKAESEDRGEGFELRTDQWGALRAGQGLLVSTHKQDNA
KGDHLDAEVAKKQLEGSQTNSKALSDIAKNQKTDEIESI---------------------------------
---------------------------------------EQLKDFASQIQQ--------------------
-------------------------------------------------------------------QIAKF
EKALLLLSSPDGIALSSSEDIHISADAQINQIAGDSINISTQKNVIAHAQNRLSLFAAQSGLKAVAAQGKVE

IQAQADALDVLSKLGITISSTDDKVIISSPKEVKITGGSSQITLNGSGIFPKTGGKFQVNAGQHLFMGGASA
NASAPELP------KAKPMQGALELLRSYGGDNFFKQNSYKVI--DSLGKQITGKLDGNGFAQVTGIAP-GP
AKVVFEKDNTSAWLQSSDFKRNYTWAEPVKSVQGLMKNALEAVGQNTMSQLQNNLLSTDKNSFKNLGKNTLD
NLAGQTVAQIKNQVTNTALNTVSKQLNLNLSAD----QMKSLGQMATNPSQSLEMLKEQGGDFLSDQMTAKL
FKTTNQESPIQQGDLDTFVRSKK
>BJAB0868_5
-MFNNIFQILESFGFLSQHRSVYLQFSDASLNSQVFLQRIDGQHYLNQGMTAELICLSTNAHIPLKTFIGVQ
VAVDQVTDRGSFFRTTGIITGASQGQSDGALTLYKLAISDPTYLWHKRRNSRVFMNKSVKEISEILFQEWQG
KSPLFASSLTLDLSGLK-QTYDVRPFVMQLNESDYDFLTRLWRSEGISWLIDEAELTVASNMDNIQPQKLRL
IDDNNQYQALTRRAIRYHRSSATEQFDSMTSLMADRSLQPTSIFVQRWQPDVLQQTDGAGSVQSKHQHSTNY
DNQSLSLEEAWHFSPAWMQDLNGEDGATSASNQQLEKFNQNLSAYYDAQSKQFIAKTTVRDTQVGYWFELNE
HPEIDQ-HESTDKEFLIIGKNYYNQNNLPKDLNQQIQTLVQQSDWQASNTD-------ERQANQLILQRRYI
PTTPAYNPQTHSPVAHPQRAKVVGPEGEEIYVDEWGRIKVRFLFTRSDDHSHDGGAGTNNNDTDSAWIDVLT
PWAGEGYGARFLPRIGEIVVINFFNGDIDRPFVMGRVHEAQRHPTKFDNKGKLPDTKKLSGIRSKEVSGGGF
GQLRFDDTPGQISTQLQSSHGASQLNLGKLSHPKDKAESEDRGEGFELRTDQWGALRAGQGLLVSTHKQDNA
KGDHLDAEVAKKQLEGSQTNSKALSDIAKNQKTDEIESI--------------------------------
----------------------------------------EQLKDFASQIQQ--------------------
----------------------------------------------------------------QIAKF
EKALLLLSSPDGIALSSSEDIHISADAQINQIAGDSINISTQKNVIAHAQNRLSLFAAQSGLKAVAAQGKVE
IQAQADALDVLSKLGITISSTDDKVIISSPKEVKITGGSSQITLNGSGIFPKTGGKFQVNAGQHLFMGGASA
NASAPELP------KAKPMQGALELLRSYGGDNFFKQNSYKVI--DSLGKQITGKLDGNGFAQVTGIAP-GP
AKVVFEKDNTSAWLQSSDFKRNYTWAEPVKSVQGLMKNALEAVGQNTMSQLQNNLLSTDKNSFKNLGKNTLD
NLAGQTVAQIKNQVTNTALNTVSKQLNLNLSAD----QMKSLGQMATNPSQSLEMLKEQGGDFLSDQMTAKL
FKTTNQESPIQQGDLDTFVRSKK
>CMC-CR-MDR-Ab4_5
-MFNNIFQILESFGFLSQHRSVYLQFSDASLNSQVFLQRIDGQHYLNQGMTAELICLSTNAHIPLKTFIGVQ
VAVDQVTDRGSFFRTTGIITGASQGQSDGALTLYKLAISDPTYLWHKRRNSRVFMNKSVKEISEILFQEWQG
KSPLFASSLTLDLSGLK-QTYDVRPFVMQLNESDYDFLTRLWRSEGISWLIDEAELTVASNMDNIQPQKLRL
IDDNNQYQALTRRAIRYHRSSATEQFDSMTSLMADRSLQPTSIFVQRWQPDVLQQTDGAGSVQSKHQHSTNY
DNQSLSLEEAWHFSPAWMQDLNGEDGATSASNQQLEKFNQNLSAYYDAQSKQFIAKTTVRDTQVGYWFELNE
HPEIDQ-HESTDKEFLIIGKNYYNQNNLPKDLNQQIQTLVQQSDWQASNTD-------ERQANQLILQRRYI
PTTPAYNPQTHSPVAHPQRAKVVGPEGEEIYVDEWGRIKVRFLFTRSDDHSHDGGAGTNNNDTDSAWIDVLT
PWAGEGYGARFLPRIGEIVVINFFNGDIDRPFVMGRVHEAQRHPTKFDNKGKLPDTKKLSGIRSKEVSGGGF

GQLRFDDTPGQISTQLQSSHGASQLNLGKLSHPKDKAESEDRGEGFELRTDQWGALRAGQGLLVSTHKQDNA
KGDHLDAEVAKKQLEGSQTNSKALSDIAKNQKTDEIESI-----------------------------------
---------------------------------------EQLKDFASQIQQ--------------------
---------------------------------------------------------------------QIAKF
EKALLLLSSPDGIALSSSEDIHISADAQINQIAGDSINISTQKNVIAHAQNRLSLFAAQSGLKAVAAQGKVE
IQAQADALDVLSKLGITISSTDDKVIISSPKEVKITGGSSQITLNGSGIFPKTGGKFQVNAGQHLFMGGASA
NASAPELP------KAKPMQGALELLRSYGGDNFFKQNSYKVI--DSLGKQITGKLDGNGFAQVTGIAP-GP
AKVVFEKDNTSAWLQSSDFKRNYTWAEPVKSVQGLMKNALEAVGQNTMSQLQNNLLSTDKNSFKNLGKNTLD
NLAGQTVAQIKNQVTNTALNTVSKQLNLNLSAD----QMKSLGQMATNPSQSLEMLKEQGGDFLSDQMTAKL
FKTTNQESPIQQGDLDTFVRSKK
>CMC-CR-MDR-Ab66_5
-MFNNIFQILESFGFLSQHRSVYLQFSDASLNSQVFLQRIDGQHYLNQGMTAELICLSTNAHIPLKTFIGVQ
VAVDQVTDRGSFFRTTGIITGASQGQSDGALTLYKLAISDPTYLWHKRRNSRVFMNKSVKEISEILFQEWQG
KSPLFASSLTLDLSGLK-QTYDVRPFVMQLNESDYDFLTRLWRSEGISWLIDEAELTVASNMDNIQPQKLRL
IDDNNQYQALTRRAIRYHRSSATEQFDSMTSLMADRSLQPTSIFVQRWQPDVLQQTDGAGSVQSKHQHSTNY
DNQSLSLEEAWHFSPAWMQDLNGEDGATSASNQQLEKFNQNLSAYYDAQSKQFIAKTTVRDTQVGYWFELNE
HPEIDQ-HESTDKEFLIIGKNYYNQNNLPKDLNQQIQTLVQQSDWQASNTD-------ERQANQLILQRRYI
PTTPAYNPQTHSPVAHPQRAKVVGPEGEEIYVDEWGRIKVRFLFTRSDDHSHDGGAGTNNNDTDSAWIDVLT
PWAGEGYGARFLPRIGEIVVINFFNGDIDRPFVMGRVHEAQRHPTKFDNKGKLPDTKKLSGIRSKEVSGGGF
GQLRFDDTPGQISTQLQSSHGASQLNLGKLSHPKDKAESEDRGEGFELRTDQWGALRAGQGLLVSTHKQDNA
KGDHLDAEVAKKQLEGSQTNSKALSDIAKNQKTDEIESI-----------------------------------
---------------------------------------EQLKDFASQIQQ--------------------
---------------------------------------------------------------------QIAKF
EKALLLLSSPDGIALSSSEDIHISADAQINQIAGDSINISTQKNVIAHAQNRLSLFAAQSGLKAVAAQGKVE
IQAQADALDVLSKLGITISSTDDKVIISSPKEVKITGGSSQITLNGSGIFPKTGGKFQVNAGQHLFMGGASA
NASAPELP------KAKPMQGALELLRSYGGDNFFKQNSYKVI--DSLGKQITGKLDGNGFAQVTGIAP-GP
AKVVFEKDNTSAWLQSSDFKRNYTWAEPVKSVQGLMKNALEAVGQNTMSQLQNNLLSTDKNSFKNLGKNTLD
NLAGQTVAQIKNQVTNTALNTVSKQLNLNLSAD----QMKSLGQMATNPSQSLEMLKEQGGDFLSDQMTAKL
FKTTNQESPIQQGDLDTFVRSKK
>CMC-MDR-Ab59_5
-MFNNIFQILESFGFLSQHRSVYLQFSDASLNSQVFLQRIDGQHYLNQGMTAELICLSTNAHIPLKTFIGVQ
VAVDQVTDRGSFFRTTGIITGASQGQSDGALTLYKLAISDPTYLWHKRRNSRVFMNKSVKEISEILFQEWQG
KSPLFASSLTLDLSGLK-QTYDVRPFVMQLNESDYDFLTRLWRSEGISWLIDEAELTVASNMDNIQPQKLRL

IDDNNQYQALTRRAIRYHRSSATEQFDSMTSLMADRSLQPTSIFVQRWQPDVLQQTDGAGSVQSKHQHSTNY
DNQSLSLEEAWHFSPAWMQDLNGEDGATSASNQQLEKFNQNLSAYYDAQSKQFIAKTTVRDTQVGYWFELNE
HPEIDQ-HESTDKEFLIIGKNYYNQNNLPKDLNQQIQTLVQQSDWQASNTD-------ERQANQLILQRRYI
PTTPAYNPQTHSPVAHPQRAKVVGPEGEEIYVDEWGRIKVRFLFTRSDDHSHDGGAGTNNNDTDSAWIDVLT
PWAGEGYGARFLPRIGEIVVINFFNGDIDRPFVMGRVHEAQRHPTKFDNKGKLPDTKKLSGIRSKEVSGGGF
GQLRFDDTPGQISTQLQSSHGASQLNLGKLSHPKDKAESEDRGEGFELRTDQWGALRAGQGLLVSTHKQDNA
KGDHLDAEVAKKQLEGSQTNSKALSDIAKNQKTDEIESI---------------------------------
----------------------------------------EQLKDFASQIQQ--------------------
--------------------------------------------------------------------QIAKF
EKALLLLSSPDGIALSSSEDIHISADAQINQIAGDSINISTQKNVIAHAQNRLSLFAAQSGLKAVAAQGKVE
IQAQADALDVLSKLGITISSTDDKVIISSPKEVKITGGSSQITLNGSGIFPKTGGKFQVNAGQHLFMGGASA
NASAPELP------KAKPMQGALELLRSYGGDNFFKQNSYKVI--DSLGKQITGKLDGNGFAQVTGIAP-GP
AKVVFEKDNTSAWLQSSDFKRNYTWAEPVKSVQGLMKNALEAVGQNTMSQLQNNLLSTDKNSFKNLGKNTLD
NLAGQTVAQIKNQVTNTALNTVSKQLNLNLSAD----QMKSLGQMATNPSQSLEMLKEQGGDFLSDQMTAKL
FKTTNQESPIQQGDLDTFVRSKK
>HRAB-85_5
-MFNNIFQILESFGFLSQHRSVYLQFSDASLNSQVFLQRIDGQHYLNQGMTAELICLSTNAHIPLKTFIGVQ
VAVDQVTDRGSFFRTTGIITGASQGQSDGALTLYKLAISDPTYLWHKRRNSRVFMNKSVKEISEILFQEWQG
KSPLFASSLTLDLSGLK-QTYDVRPFVMQLNESDYDFLTRLWRSEGISWLIDEAELTVASNMDNIQPQKLRL
IDDNNQYQALTRRAIRYHRSSATEQFDSMTSLMADRSLQPTSIFVQRWQPDVLQQTDGAGSVQSKHQHSTNY
DNQSLSLEEAWHFSPAWMQDLNGEDGATSASNQQLEKFNQNLSAYYDAQSKQFIAKTTVRDTQVGYWFELNE
HPEIDQ-HESTDKEFLIIGKNYYNQNNLPKDLNQQIQTLVQQSDWQASNTD-------ERQANQLILQRRYI
PTTPAYNPQTHSPVAHPQRAKVVGPEGEEIYVDEWGRIKVRFLFTRSDDHSHDGGAGTNNNDTDSAWIDVLT
PWAGEGYGARFLPRIGEIVVINFFNGDIDRPFVMGRVHEAQRHPTKFDNKGKLPDTKKLSGIRSKEVSGGGF
GQLRFDDTPGQISTQLQSSHGASQLNLGKLSHPKDKAESEDRGEGFELRTDQWGALRAGQGLLVSTHKQDNA
KGDHLDAEVAKKQLEGSQTNSKALSDIAKNQKTDEIESI---------------------------------
----------------------------------------EQLKDFASQIQQ--------------------
--------------------------------------------------------------------QIAKF
EKALLLLSSPDGIALSSSEDIHISADAQINQIAGDSINISTQKNVIAHAQNRLSLFAAQSGLKAVAAQGKVE
IQAQADALDVLSKLGITISSTDDKVIISSPKEVKITGGSSQITLNGSGIFPKTGGKFQVNAGQHLFMGGASA
NASAPELP------KAKPMQGALELLRSYGGDNFFKQNSYKVI--DSLGKQITGKLDGNGFAQVTGIAP-GP
AKVVFEKDNTSAWLQSSDFKRNYTWAEPVKSVQGLMKNALEAVGQNTMSQLQNNLLSTDKNSFKNLGKNTLD
NLAGQTVAQIKNQVTNTALNTVSKQLNLNLSAD----QMKSLGQMATNPSQSLEMLKEQGGDFLSDQMTAKL

FKTTNQESPIQQGDLDTFVRSKK
>KBN10P02143_5
-MFNNIFQILESFGFLSQHRSVYLQFSDASLNSQVFLQRIDGQHYLNQGMTAELICLSTNAHIPLKTFIGVQ
VAVDQVTDRGSFFRTTGIITGASQGQSDGALTLYKLAISDPTYLWHKRRNSRVFMNKSVKEISEILFQEWQG
KSPLFASSLTLDLSGLK-QTYDVRPFVMQLNESDYDFLTRLWRSEGISWLIDEAELTVASNMDNIQPQKLRL
IDDNNQYQALTRRAIRYHRSSATEQFDSMTSLMADRSLQPTSIFVQRWQPDVLQQTDGAGSVQSKHQHSTNY
DNQSLSLEEAWHFSPAWMQDLNGEDGATSASNQQLEKFNQNLSAYYDAQSKQFIAKTTVRDTQVGYWFELNE
HPEIDQ-HESTDKEFLIIGKNYYNQNNLPKDLNQQIQTLVQQSDWQASNTD-------ERQANQLILQRRYI
PTTPAYNPQTHSPVAHPQRAKVVGPEGEEIYVDEWGRIKVRFLFTRSDDHSHDGGAGTNNNDTDSAWIDVLT
PWAGEGYGARFLPRIGEIVVINFFNGDIDRPFVMGRVHEAQRHPTKFDNKGKLPDTKKLSGIRSKEVSGGGF
GQLRFDDTPGQISTQLQSSHGASQLNLGKLSHPKDKAESEDRGEGFELRTDQWGALRAGQGLLVSTHKQDNA
KGDHLDAEVAKKQLEGSQTNSKALSDIAKNQKTDEIESI---------------------------------
----------------------------------------EQLKDFASQIQQ--------------------
-------------------------------------------------------------------QIAKF
EKALLLLSSPDGIALSSSEDIHISADAQINQIAGDSINISTQKNVIAHAQNRLSLFAAQSGLKAVAAQGKVE
IQAQADALDVLSKLGITISSTDDKVIISSPKEVKITGGSSQITLNGSGIFPKTGGKFQVNAGQHLFMGGASA
NASAPELP------KAKPMQGALELLRSYGGDNFFKQNSYKVI--DSLGKQITGKLDGNGFAQVTGIAP-GP
AKVVFEKDNTSAWLQSSDFKRNYTWAEPVKSVQGLMKNALEAVGQNTMSQLQNNLLSTDKNSFKNLGKNTLD
NLAGQTVAQIKNQVTNTALNTVSKQLNLNLSAD----QMKSLGQMATNPSQSLEMLKEQGGDFLSDQMTAKL
FKTTNQESPIQQGDLDTFVRSKK
>MDR-TJ_5
-MFNNIFQILESFGFLSQHRSVYLQFSDASLNSQVFLQRIDGQHYLNQGMTAELICLSTNAHIPLKTFIGVQ
VAVDQVTDRGSFFRTTGIITGASQGQSDGALTLYKLAISDPTYLWHKRRNSRVFMNKSVKEISEILFQEWQG
KSPLFASSLTLDLSGLK-QTYDVRPFVMQLNESDYDFLTRLWRSEGISWLIDEAELTVASNMDNIQPQKLRL
IDDNNQYQALTRRAIRYHRSSATEQFDSMTSLMADRSLQPTSIFVQRWQPDVLQQTDGAGSVQSKHQHSTNY
DNQSLSLEEAWHFSPAWMQDLNGEDGATSASNQQLEKFNQNLSAYYDAQSKQFIAKTTVRDTQVGYWFELNE
HPEIDQ-HESTDKEFLIIGKNYYNQNNLPKDLNQQIQTLVQQSDWQASNTD-------ERQANQLILQRRYI
PTTPAYNPQTHSPVAHPQRAKVVGPEGEEIYVDEWGRIKVRFLFTRSDDHSHDGGAGTNNNDTDSAWIDVLT
PWAGEGYGARFLPRIGEIVVINFFNGDIDRPFVMGRVHEAQRHPTKFDNKGKLPDTKKLSGIRSKEVSGGGF
GQLRFDDTPGQISTQLQSSHGASQLNLGKLSHPKDKAESEDRGEGFELRTDQWGALRAGQGLLVSTHKQDNA
KGDHLDAEVAKKQLEGSQTNSKALSDIAKNQKTDEIESI---------------------------------
----------------------------------------EQLKDFASQIQQ--------------------
-------------------------------------------------------------------QIAKF

EKALLLLSSPDGIALSSSEDIHISADAQINQIAGDSINISTQKNVIAHAQNRLSLFAAQSGLKAVAAQGKVE
IQAQADALDVLSKLGITISSTDDKVIISSPKEVKITGGSSQITLNGSGIFPKTGGKFQVNAGQHLFMGGASA
NASAPELP------KAKPMQGALELLRSYGGDNFFKQNSYKVI--DSLGKQITGKLDGNGFAQVTGIAP-GP
AKVVFEKDNTSAWLQSSDFKRNYTWAEPVKSVQGLMKNALEAVGQNTMSQLQNNLLSTDKNSFKNLGKNTLD
NLAGQTVAQIKNQVTNTALNTVSKQLNLNLSAD----QMKSLGQMATNPSQSLEMLKEQGGDFLSDQMTAKL
FKTTNQESPIQQGDLDTFVRSKK
>MDR-ZJ06_5
-MFNNIFQILESFGFLSQHRSVYLQFSDASLNSQVFLQRIDGQHYLNQGMTAELICLSTNAHIPLKTFIGVQ
VAVDQVTDRGSFFRTTGIITGASQGQSDGALTLYKLAISDPTYLWHKRRNSRVFMNKSVKEISEILFQEWQG
KSPLFASSLTLDLSGLK-QTYDVRPFVMQLNESDYDFLTRLWRSEGISWLIDEAELTVASNMDNIQPQKLRL
IDDNNQYQALTRRAIRYHRSSATEQFDSMTSLMADRSLQPTSIFVQRWQPDVLQQTDGAGSVQSKHQHSTNY
DNQSLSLEEAWHFSPAWMQDLNGEDGATSASNQQLEKFNQNLSAYYDAQSKQFIAKTTVRDTQVGYWFELNE
HPEIDQ-HESTDKEFLIIGKNYYNQNNLPKDLNQQIQTLVQQSDWQASNTD-------ERQANQLILQRRYI
PTTPAYNPQTHSPVAHPQRAKVVGPEGEEIYVDEWGRIKVRFLFTRSDDHSHDGGAGTNNNDTDSAWIDVLT
PWAGEGYGARFLPRIGEIVVINFFNGDIDRPFVMGRVHEAQRHPTKFDNKGKLPDTKKLSGIRSKEVSGGGF
GQLRFDDTPGQISTQLQSSHGASQLNLGKLSHPKDKAESEDRGEGFELRTDQWGALRAGQGLLVSTHKQDNA
KGDHLDAEVAKKQLEGSQTNSKALSDIAKNQKTDEIESI-------------------------------
---------------------------------------EQLKDFASQIQQ--------------------
-------------------------------------------------------------------QIAKF
EKALLLLSSPDGIALSSSEDIHISADAQINQIAGDSINISTQKNVIAHAQNRLSLFAAQSGLKAVAAQGKVE
IQAQADALDVLSKLGITISSTDDKVIISSPKEVKITGGSSQITLNGSGIFPKTGGKFQVNAGQHLFMGGASA
NASAPELP------KAKPMQGALELLRSYGGDNFFKQNSYKVI--DSLGKQITGKLDGNGFAQVTGIAP-GP
AKVVFEKDNTSAWLQSSDFKRNYTWAEPVKSVQGLMKNALEAVGQNTMSQLQNNLLSTDKNSFKNLGKNTLD
NLAGQTVAQIKNQVTNTALNTVSKQLNLNLSAD----QMKSLGQMATNPSQSLEMLKEQGGDFLSDQMTAKL
FKTTNQESPIQQGDLDTFVRSKK
>NCGM237_5
-MFNNIFQILESFGFLSQHRSVYLQFSDASLNSQVFLQRIDGQHYLNQGMTAELICLSTNAHIPLKTFIGVQ
VAVDQVTDRGSFFRTTGIITGASQGQSDGALTLYKLAISDPTYLWHKRRNSRVFMNKSVKEISEILFQEWQG
KSPLFASSLTLDLSGLK-QTYDVRPFVMQLNESDYDFLTRLWRSEGISWLIDEAELTVASNMDNIQPQKLRL
IDDNNQYQALTRRAIRYHRSSATEQFDSMTSLMADRSLQPTSIFVQRWQPDVLQQTDGAGSVQSKHQHSTNY
DNQSLSLEEAWHFSPAWMQDLNGEDGATSASNQQLEKFNQNLSAYYDAQSKQFIAKTTVRDTQVGYWFELNE
HPEIDQ-HESTDKEFLIIGKNYYNQNNLPKDLNQQIQTLVQQSDWQASNTD-------ERQANQLILQRRYI
PTTPAYNPQTHSPVAHPQRAKVVGPEGEEIYVDEWGRIKVRFLFTRSDDHSHDGGAGTNNNDTDSAWIDVLT

PWAGEGYGARFLPRIGEIVVINFFNGDIDRPFVMGRVHEAQRHPTKFDNKGKLPDTKKLSGIRSKEVSGGGF
GQLRFDDTPGQISTQLQSSHGASQLNLGKLSHPKDKAESEDRGEGFELRTDQWGALRAGQGLLVSTHKQDNA
KGDHLDAEVAKKQLEGSQTNSKALSDIAKNQKTDEIESI---------------------------------
----------------------------------------EQLKDFASQIQQ--------------------
-------------------------------------------------------------------QIAKF
EKALLLLSSPDGIALSSSEDIHISADAQINQIAGDSINISTQKNVIAHAQNRLSLFAAQSGLKAVAAQGKVE
IQAQADALDVLSKLGITISSTDDKVIISSPKEVKITGGSSQITLNGSGIFPKTGGKFQVNAGQHLFMGGASA
NASAPELP------KAKPMQGALELLRSYGGDNFFKQNSYKVI--DSLGKQITGKLDGNGFAQVTGIAP-GP
AKVVFEKDNTSAWLQSSDFKRNYTWAEPVKSVQGLMKNALEAVGQNTMSQLQNNLLSTDKNSFKNLGKNTLD
NLAGQTVAQIKNQVTNTALNTVSKQLNLNLSAD----QMKSLGQMATNPSQSLEMLKEQGGDFLSDQMTAKL
FKTTNQESPIQQGDLDTFVRSKK
>SSA6_5
-MFNNIFQILESFGFLSQHRSVYLQFSDASLNSQVFLQRIDGQHYLNQGMTAELICLSTNAHIPLKTFIGVQ
VAVDQVTDRGSFFRTTGIITGASQGQSDGALTLYKLAISDPTYLWHKRRNSRVFMNKSVKEISEILFQEWQG
KSPLFASSLTLDLSGLK-QTYDVRPFVMQLNESDYDFLTRLWRSEGISWLIDEAELTVASNMDNIQPQKLRL
IDDNNQYQALTRRAIRYHRSSATEQFDSMTSLMADRSLQPTSIFVQRWQPDVLQQTDGAGSVQSKHQHSTNY
DNQSLSLEEAWHFSPAWMQDLNGEDGATSASNQQLEKFNQNLSAYYDAQSKQFIAKTTVRDTQVGYWFELNE
HPEIDQ-HESTDKEFLIIGKNYYNQNNLPKDLNQQIQTLVQQSDWQASNTD-------ERQANQLILQRRYI
PTTPAYNPQTHSPVAHPQRAKVVGPEGEEIYVDEWGRIKVRFLFTRSDDHSHDGGAGTNNNDTDSAWIDVLT
PWAGEGYGARFLPRIGEIVVINFFNGDIDRPFVMGRVHEAQRHPTKFDNKGKLPDTKKLSGIRSKEVSGGGF
GQLRFDDTPGQISTQLQSSHGASQLNLGKLSHPKDKAESEDRGEGFELRTDQWGALRAGQGLLVSTHKQDNA
KGDHLDAEVAKKQLEGSQTNSKALSDIAKNQKTDEIESI---------------------------------
----------------------------------------EQLKDFASQIQQ--------------------
-------------------------------------------------------------------QIAKF
EKALLLLSSPDGIALSSSEDIHISADAQINQIAGDSINISTQKNVIAHAQNRLSLFAAQSGLKAVAAQGKVE
IQAQADALDVLSKLGITISSTDDKVIISSPKEVKITGGSSQITLNGSGIFPKTGGKFQVNAGQHLFMGGASA
NASAPELP------KAKPMQGALELLRSYGGDNFFKQNSYKVI--DSLGKQITGKLDGNGFAQVTGIAP-GP
AKVVFEKDNTSAWLQSSDFKRNYTWAEPVKSVQGLMKNALEAVGQNTMSQLQNNLLSTDKNSFKNLGKNTLD
NLAGQTVAQIKNQVTNTALNTVSKQLNLNLSAD----QMKSLGQMATNPSQSLEMLKEQGGDFLSDQMTAKL
FKTTNQESPIQQGDLDTFVRSKK
>TYTH-1_5
-MFNNIFQILESFGFLSQHRSVYLQFSDASLNSQVFLQRIDGQHYLNQGMTAELICLSTNAHIPLKTFIGVQ
VAVDQVTDRGSFFRTTGIITGASQGQSDGALTLYKLAISDPTYLWHKRRNSRVFMNKSVKEISEILFQEWQG

KSPLFASSLTLDLSGLK-QTYDVRPFVMQLNESDYDFLTRLWRSEGISWLIDEAELTVASNMDNIQPQKLRL
IDDNNQYQALTRRAIRYHRSSATEQFDSMTSLMADRSLQPTSIFVQRWQPDVLQQTDGAGSVQSKHQHSTNY
DNQSLSLEEAWHFSPAWMQDLNGEDGATSASNQQLEKFNQNLSAYYDAQSKQFIAKTTVRDTQVGYWFELNE
HPEIDQ-HESTDKEFLIIGKNYYNQNNLPKDLNQQIQTLVQQSDWQASNTD-------ERQANQLILQRRYI
PTTPAYNPQTHSPVAHPQRAKVVGPEGEEIYVDEWGRIKVRFLFTRSDDHSHDGGAGTNNNDTDSAWIDVLT
PWAGEGYGARFLPRIGEIVVINFFNGDIDRPFVMGRVHEAQRHPTKFDNKGKLPDTKKLSGIRSKEVSGGGF
GQLRFDDTPGQISTQLQSSHGASQLNLGKLSHPKDKAESEDRGEGFELRTDQWGALRAGQGLLVSTHKQDNA
KGDHLDAEVAKKQLEGSQTNSKALSDIAKNQKTDEIESI---------------------------------
----------------------------------------EQLKDFASQIQQ--------------------
-------------------------------------------------------------------QIAKF
EKALLLLSSPDGIALSSSEDIHISADAQINQIAGDSINISTQKNVIAHAQNRLSLFAAQSGLKAVAAQGKVE
IQAQADALDVLSKLGITISSTDDKVIISSPKEVKITGGSSQITLNGSGIFPKTGGKFQVNAGQHLFMGGASA
NASAPELP------KAKPMQGALELLRSYGGDNFFKQNSYKVI--DSLGKQITGKLDGNGFAQVTGIAP-GP
AKVVFEKDNTSAWLQSSDFKRNYTWAEPVKSVQGLMKNALEAVGQNTMSQLQNNLLSTDKNSFKNLGKNTLD
NLAGQTVAQIKNQVTNTALNTVSKQLNLNLSAD----QMKSLGQMATNPSQSLEMLKEQGGDFLSDQMTAKL
FKTTNQESPIQQGDLDTFVRSKK
>USA2_5
-MFNNIFQILESFGFLSQHRSVYLQFSDASLNSQVFLQRIDGQHYLNQGMTAELICLSTNAHIPLKTFIGVQ
VAVDQVTDRGSFFRTTGIITGASQGQSDGALTLYKLAISDPTYLWHKRRNSRVFMNKSVKEISEILFQEWQG
KSPLFASSLTLDLSGLK-QTYDVRPFVMQLNESDYDFLTRLWRSEGISWLIDEAELTVASNMDNIQPQKLRL
IDDNNQYQALTRRAIRYHRSSATEQFDSMTSLMADRSLQPTSIFVQRWQPDVLQQTDGAGSVQSKHQHSTNY
DNQSLSLEEAWHFSPAWMQDLNGEDGATSASNQQLEKFNQNLSAYYDAQSKQFIAKTTVRDTQVGYWFELNE
HPEIDQ-HESTDKEFLIIGKNYYNQNNLPKDLNQQIQTLVQQSDWQASNTD-------ERQANQLILQRRYI
PTTPAYNPQTHSPVAHPQRAKVVGPEGEEIYVDEWGRIKVRFLFTRSDDHSHDGGAGTNNNDTDSAWIDVLT
PWAGEGYGARFLPRIGEIVVINFFNGDIDRPFVMGRVHEAQRHPTKFDNKGKLPDTKKLSGIRSKEVSGGGF
GQLRFDDTPGQISTQLQSSHGASQLNLGKLSHPKDKAESEDRGEGFELRTDQWGALRAGQGLLVSTHKQDNA
KGDHLDAEVAKKQLEGSQTNSKALSDIAKNQKTDEIESI---------------------------------
----------------------------------------EQLKDFASQIQQ--------------------
-------------------------------------------------------------------QIAKF
EKALLLLSSPDGIALSSSEDIHISADAQINQIAGDSINISTQKNVIAHAQNRLSLFAAQSGLKAVAAQGKVE
IQAQADALDVLSKLGITISSTDDKVIISSPKEVKITGGSSQITLNGSGIFPKTGGKFQVNAGQHLFMGGASA
NASAPELP------KAKPMQGALELLRSYGGDNFFKQNSYKVI--DSLGKQITGKLDGNGFAQVTGIAP-GP
AKVVFEKDNTSAWLQSSDFKRNYTWAEPVKSVQGLMKNALEAVGQNTMSQLQNNLLSTDKNSFKNLGKNTLD

NLAGQTVAQIKNQVTNTALNTVSKQLNLNLSAD----QMKSLGQMATNPSQSLEMLKEQGGDFLSDQMTAKL
FKTTNQESPIQQGDLDTFVRSKK
>WCHAB005133_5
-MFNNIFQILESFGFLSQHRSVYLQFSDASLNSQVFLQRIDGQHYLNQGMTAELICLSTNAHIPLKTFIGVQ
VAVDQVTDRGSFFRTTGIITGASQGQSDGALTLYKLAISDPTYLWHKRRNSRVFMNKSVKEISEILFQEWQG
KSPLFASSLTLDLSGLK-QTYDVRPFVMQLNESDYDFLTRLWRSEGISWLIDEAELTVASNMDNIQPQKLRL
IDDNNQYQALTRRAIRYHRSSATEQFDSMTSLMADRSLQPTSIFVQRWQPDVLQQTDGAGSVQSKHQHSTNY
DNQSLSLEEAWHFSPAWMQDLNGEDGATSASNQQLEKFNQNLSAYYDAQSKQFIAKTTVRDTQVGYWFELNE
HPEIDQ-HESTDKEFLIIGKNYYNQNNLPKDLNQQIQTLVQQSDWQASNTD-------ERQANQLILQRRYI
PTTPAYNPQTHSPVAHPQRAKVVGPEGEEIYVDEWGRIKVRFLFTRSDDHSHDGGAGTNNNDTDSAWIDVLT
PWAGEGYGARFLPRIGEIVVINFFNGDIDRPFVMGRVHEAQRHPTKFDNKGKLPDTKKLSGIRSKEVSGGGF
GQLRFDDTPGQISTQLQSSHGASQLNLGKLSHPKDKAESEDRGEGFELRTDQWGALRAGQGLLVSTHKQDNA
KGDHLDAEVAKKQLEGSQTNSKALSDIAKNQKTDEIESI----------------------------------
---------------------------------------EQLKDFASQIQQ--------------------
-------------------------------------------------------------------QIAKF
EKALLLLSSPDGIALSSSEDIHISADAQINQIAGDSINISTQKNVIAHAQNRLSLFAAQSGLKAVAAQGKVE
IQAQADALDVLSKLGITISSTDDKVIISSPKEVKITGGSSQITLNGSGIFPKTGGKFQVNAGQHLFMGGASA
NASAPELP------KAKPMQGALELLRSYGGDNFFKQNSYKVI--DSLGKQITGKLDGNGFAQVTGIAP-GP
AKVVFEKDNTSAWLQSSDFKRNYTWAEPVKSVQGLMKNALEAVGQNTMSQLQNNLLSTDKNSFKNLGKNTLD
NLAGQTVAQIKNQVTNTALNTVSKQLNLNLSAD----QMKSLGQMATNPSQSLEMLKEQGGDFLSDQMTAKL
FKTTNQESPIQQGDLDTFVRSKK
>XDR-BJ83_5
-MFNNIFQILESFGFLSQHRSVYLQFSDASLNSQVFLQRIDGQHYLNQGMTAELICLSTNAHIPLKTFIGVQ
VAVDQVTDRGSFFRTTGIITGASQGQSDGALTLYKLAISDPTYLWHKRRNSRVFMNKSVKEISEILFQEWQG
KSPLFASSLTLDLSGLK-QTYDVRPFVMQLNESDYDFLTRLWRSEGISWLIDEAELTVASNMDNIQPQKLRL
IDDNNQYQALTRRAIRYHRSSATEQFDSMTSLMADRSLQPTSIFVQRWQPDVLQQTDGAGSVQSKHQHSTNY
DNQSLSLEEAWHFSPAWMQDLNGEDGATSASNQQLEKFNQNLSAYYDAQSKQFIAKTTVRDTQVGYWFELNE
HPEIDQ-HESTDKEFLIIGKNYYNQNNLPKDLNQQIQTLVQQSDWQASNTD-------ERQANQLILQRRYI
PTTPAYNPQTHSPVAHPQRAKVVGPEGEEIYVDEWGRIKVRFLFTRSDDHSHDGGAGTNNNDTDSAWIDVLT
PWAGEGYGARFLPRIGEIVVINFFNGDIDRPFVMGRVHEAQRHPTKFDNKGKLPDTKKLSGIRSKEVSGGGF
GQLRFDDTPGQISTQLQSSHGASQLNLGKLSHPKDKAESEDRGEGFELRTDQWGALRAGQGLLVSTHKQDNA
KGDHLDAEVAKKQLEGSQTNSKALSDIAKNQKTDEIESI----------------------------------
---------------------------------------EQLKDFASQIQQ--------------------

-------------------------------------------------------------------QIAKF
EKALLLLSSPDGIALSSSEDIHISADAQINQIAGDSINISTQKNVIAHAQNRLSLFAAQSGLKAVAAQGKVE
IQAQADALDVLSKLGITISSTDDKVIISSPKEVKITGGSSQITLNGSGIFPKTGGKFQVNAGQHLFMGGASA
NASAPELP------KAKPMQGALELLRSYGGDNFFKQNSYKVI--DSLGKQITGKLDGNGFAQVTGIAP-GP
AKVVFEKDNTSAWLQSSDFKRNYTWAEPVKSVQGLMKNALEAVGQNTMSQLQNNLLSTDKNSFKNLGKNTLD
NLAGQTVAQIKNQVTNTALNTVSKQLNLNLSAD----QMKSLGQMATNPSQSLEMLKEQGGDFLSDQMTAKL
FKTTNQESPIQQGDLDTFVRSKK
>XH386_5
-MFNNIFQILESFGFLSQHRSVYLQFSDASLNSQVFLQRIDGQHYLNQGMTAELICLSTNAHIPLKTFIGVQ
VAVDQVTDRGSFFRTTGIITGASQGQSDGALTLYKLAISDPTYLWHKRRNSRVFMNKSVKEISEILFQEWQG
KSPLFASSLTLDLSGLK-QTYDVRPFVMQLNESDYDFLTRLWRSEGISWLIDEAELTVASNMDNIQPQKLRL
IDDNNQYQALTRRAIRYHRSSATEQFDSMTSLMADRSLQPTSIFVQRWQPDVLQQTDGAGSVQSKHQHSTNY
DNQSLSLEEAWHFSPAWMQDLNGEDGATSASNQQLEKFNQNLSAYYDAQSKQFIAKTTVRDTQVGYWFELNE
HPEIDQ-HESTDKEFLIIGKNYYNQNNLPKDLNQQIQTLVQQSDWQASNTD-------ERQANQLILQRRYI
PTTPAYNPQTHSPVAHPQRAKVVGPEGEEIYVDEWGRIKVRFLFTRSDDHSHDGGAGTNNNDTDSAWIDVLT
PWAGEGYGARFLPRIGEIVVINFFNGDIDRPFVMGRVHEAQRHPTKFDNKGKLPDTKKLSGIRSKEVSGGGF
GQLRFDDTPGQISTQLQSSHGASQLNLGKLSHPKDKAESEDRGEGFELRTDQWGALRAGQGLLVSTHKQDNA
KGDHLDAEVAKKQLEGSQTNSKALSDIAKNQKTDEIESI---------------------------------
----------------------------------------EQLKDFASQIQQ--------------------
-------------------------------------------------------------------QIAKF
EKALLLLSSPDGIALSSSEDIHISADAQINQIAGDSINISTQKNVIAHAQNRLSLFAAQSGLKAVAAQGKVE
IQAQADALDVLSKLGITISSTDDKVIISSPKEVKITGGSSQITLNGSGIFPKTGGKFQVNAGQHLFMGGASA
NASAPELP------KAKPMQGALELLRSYGGDNFFKQNSYKVI--DSLGKQITGKLDGNGFAQVTGIAP-GP
AKVVFEKDNTSAWLQSSDFKRNYTWAEPVKSVQGLMKNALEAVGQNTMSQLQNNLLSTDKNSFKNLGKNTLD
NLAGQTVAQIKNQVTNTALNTVSKQLNLNLSAD----QMKSLGQMATNPSQSLEMLKEQGGDFLSDQMTAKL
FKTTNQESPIQQGDLDTFVRSKK
>XH856_5
-MFNNIFQILESFGFLSQHRSVYLQFSDASLNSQVFLQRIDGQHYLNQGMTAELICLSTNAHIPLKTFIGVQ
VAVDQVTDRGSFFRTTGIITGASQGQSDGALTLYKLAISDPTYLWHKRRNSRVFMNKSVKEISEILFQEWQG
KSPLFASSLTLDLSGLK-QTYDVRPFVMQLNESDYDFLTRLWRSEGISWLIDEAELTVASNMDNIQPQKLRL
IDDNNQYQALTRRAIRYHRSSATEQFDSMTSLMADRSLQPTSIFVQRWQPDVLQQTDGAGSVQSKHQHSTNY
DNQSLSLEEAWHFSPAWMQDLNGEDGATSASNQQLEKFNQNLSAYYDAQSKQFIAKTTVRDTQVGYWFELNE
HPEIDQ-HESTDKEFLIIGKNYYNQNNLPKDLNQQIQTLVQQSDWQASNTD-------ERQANQLILQRRYI

PTTPAYNPQTHSPVAHPQRAKVVGPEGEEIYVDEWGRIKVRFLFTRSDDHSHDGGAGTNNNDTDSAWIDVLT
PWAGEGYGARFLPRIGEIVVINFFNGDIDRPFVMGRVHEAQRHPTKFDNKGKLPDTKKLSGIRSKEVSGGGF
GQLRFDDTPGQISTQLQSSHGASQLNLGKLSHPKDKAESEDRGEGFELRTDQWGALRAGQGLLVSTHKQDNA
KGDHLDAEVAKKQLEGSQTNSKALSDIAKNQKTDEIESI---------------------------------
----------------------------------------EQLKDFASQIQQ--------------------
-------------------------------------------------------------------QIAKF
EKALLLLSSPDGIALSSSEDIHISADAQINQIAGDSINISTQKNVIAHAQNRLSLFAAQSGLKAVAAQGKVE
IQAQADALDVLSKLGITISSTDDKVIISSPKEVKITGGSSQITLNGSGIFPKTGGKFQVNAGQHLFMGGASA
NASAPELP------KAKPMQGALELLRSYGGDNFFKQNSYKVI--DSLGKQITGKLDGNGFAQVTGIAP-GP
AKVVFEKDNTSAWLQSSDFKRNYTWAEPVKSVQGLMKNALEAVGQNTMSQLQNNLLSTDKNSFKNLGKNTLD
NLAGQTVAQIKNQVTNTALNTVSKQLNLNLSAD----QMKSLGQMATNPSQSLEMLKEQGGDFLSDQMTAKL
FKTTNQESPIQQGDLDTFVRSKK
>XH859_5
-MFNNIFQILESFGFLSQHRSVYLQFSDASLNSQVFLQRIDGQHYLNQGMTAELICLSTNAHIPLKTFIGVQ
VAVDQVTDRGSFFRTTGIITGASQGQSDGALTLYKLAISDPTYLWHKRRNSRVFMNKSVKEISEILFQEWQG
KSPLFASSLTLDLSGLK-QTYDVRPFVMQLNESDYDFLTRLWRSEGISWLIDEAELTVASNMDNIQPQKLRL
IDDNNQYQALTRRAIRYHRSSATEQFDSMTSLMADRSLQPTSIFVQRWQPDVLQQTDGAGSVQSKHQHSTNY
DNQSLSLEEAWHFSPAWMQDLNGEDGATSASNQQLEKFNQNLSAYYDAQSKQFIAKTTVRDTQVGYWFELNE
HPEIDQ-HESTDKEFLIIGKNYYNQNNLPKDLNQQIQTLVQQSDWQASNTD-------ERQANQLILQRRYI
PTTPAYNPQTHSPVAHPQRAKVVGPEGEEIYVDEWGRIKVRFLFTRSDDHSHDGGAGTNNNDTDSAWIDVLT
PWAGEGYGARFLPRIGEIVVINFFNGDIDRPFVMGRVHEAQRHPTKFDNKGKLPDTKKLSGIRSKEVSGGGF
GQLRFDDTPGQISTQLQSSHGASQLNLGKLSHPKDKAESEDRGEGFELRTDQWGALRAGQGLLVSTHKQDNA
KGDHLDAEVAKKQLEGSQTNSKALSDIAKNQKTDEIESI---------------------------------
----------------------------------------EQLKDFASQIQQ--------------------
-------------------------------------------------------------------QIAKF
EKALLLLSSPDGIALSSSEDIHISADAQINQIAGDSINISTQKNVIAHAQNRLSLFAAQSGLKAVAAQGKVE
IQAQADALDVLSKLGITISSTDDKVIISSPKEVKITGGSSQITLNGSGIFPKTGGKFQVNAGQHLFMGGASA
NASAPELP------KAKPMQGALELLRSYGGDNFFKQNSYKVI--DSLGKQITGKLDGNGFAQVTGIAP-GP
AKVVFEKDNTSAWLQSSDFKRNYTWAEPVKSVQGLMKNALEAVGQNTMSQLQNNLLSTDKNSFKNLGKNTLD
NLAGQTVAQIKNQVTNTALNTVSKQLNLNLSAD----QMKSLGQMATNPSQSLEMLKEQGGDFLSDQMTAKL
FKTTNQESPIQQGDLDTFVRSKK
>YU-R612_5
-MFNNIFQILESFGFLSQHRSVYLQFSDASLNSQVFLQRIDGQHYLNQGMTAELICLSTNAHIPLKTFIGVQ

VAVDQVTDRGSFFRTTGIITGASQGQSDGALTLYKLAISDPTYLWHKRRNSRVFMNKSVKEISEILFQEWQG
KSPLFASSLTLDLSGLK-QTYDVRPFVMQLNESDYDFLTRLWRSEGISWLIDEAELTVASNMDNIQPQKLRL
IDDNNQYQALTRRAIRYHRSSATEQFDSMTSLMADRSLQPTSIFVQRWQPDVLQQTDGAGSVQSKHQHSTNY
DNQSLSLEEAWHFSPAWMQDLNGEDGATSASNQQLEKFNQNLSAYYDAQSKQFIAKTTVRDTQVGYWFELNE
HPEIDQ-HESTDKEFLIIGKNYYNQNNLPKDLNQQIQTLVQQSDWQASNTD-------ERQANQLILQRRYI
PTTPAYNPQTHSPVAHPQRAKVVGPEGEEIYVDEWGRIKVRFLFTRSDDHSHDGGAGTNNNDTDSAWIDVLT
PWAGEGYGARFLPRIGEIVVINFFNGDIDRPFVMGRVHEAQRHPTKFDNKGKLPDTKKLSGIRSKEVSGGGF
GQLRFDDTPGQISTQLQSSHGASQLNLGKLSHPKDKAESEDRGEGFELRTDQWGALRAGQGLLVSTHKQDNA
KGDHLDAEVAKKQLEGSQTNSKALSDIAKNQKTDEIESI---------------------------------
----------------------------------------EQLKDFASQIQQ--------------------
-------------------------------------------------------------------QIAKF
EKALLLLSSPDGIALSSSEDIHISADAQINQIAGDSINISTQKNVIAHAQNRLSLFAAQSGLKAVAAQGKVE
IQAQADALDVLSKLGITISSTDDKVIISSPKEVKITGGSSQITLNGSGIFPKTGGKFQVNAGQHLFMGGASA
NASAPELP------KAKPMQGALELLRSYGGDNFFKQNSYKVI--DSLGKQITGKLDGNGFAQVTGIAP-GP
AKVVFEKDNTSAWLQSSDFKRNYTWAEPVKSVQGLMKNALEAVGQNTMSQLQNNLLSTDKNSFKNLGKNTLD
NLAGQTVAQIKNQVTNTALNTVSKQLNLNLSAD----QMKSLGQMATNPSQSLEMLKEQGGDFLSDQMTAKL
FKTTNQESPIQQGDLDTFVRSKK
>AB031_0
-MFNNISQVLESLGFLSQHRSVHLQFSDASLNSQVFLQRIEGQHYLNEGMTAELICLSTNAHIALKTFIGVQ
VAIDQVTDQGQLFRTTGIITGASQGQSDGALTLYKLTISDPTYLWHKRRNSRVFMNKSVREITEILFQEWQG
KSPLFASSLTLDLSGLK-QAYDIRPFVMQLNQSDYDFLTRLWRSEGINWLIDEAELTVPSNTDHIQPQKLRL
IDDNSQYQALSRRTIRYHRSHATEQFDSMTSLMANRSLQPTSVFVQRWQSDVLQQTDGAGSVQSTHQHSTNY
DNQSLSLEDAWHFSPAWMQDLKGEDGATSASNQQLEKFNQNLSAFHDAQSKQFIAQTTVRDTQVGYWFELNE
HPEIDQ-HNGSDKEFLIIGKHYYNQNNLPKDLNQQIQALVRQSHWSINNEE--------RQGNELILQRRHI
KTVPEYQPLQDRPQASVQRARVVGPEGESIHVDQWGRIKVRFLFTRADDHRHDGGAGSNDNDTDSAWVDVLT
PWAGEGYGARFLPRIGEIVVIDFFDGNVDRPFVVGRIHEAERHPTQFDQKGQLPDTKKLSGIRSEEVDGKGF
NQLRFDDTTGQISAQLQSSHAASQLNLGNLSHPKEKAESDGRGEGFELRTDQWGAVRAGSGLYVSTHKQDQA
QGTHLDAKETKQQLDGNLNNAKALSDVAKNQQTDPLEHL---------------------------------
----------------------------------------ENLKTFLEQIEE--------------------
---------------------------------------------------------------QDQGKAAAF
KQALMVLTAPNSIALASNEDIHLSADGQLSQTAGDSISLSTQKNLIAHAQNKISLFAAQEGARLYAAKGKLE
VQAQGE------------------------KSLIVSF-------------------------QRLYKG----
------------------------------------------------------------------------

```
------------------------------------------------------------------------
------------------------------------------------------------------------
-----------------------
>AB031_00
-MFNNISQVLESLGFLSQHRSVHLQFSDASLNSQVFLQRIEGQHYLNEGMTAELICLSTNAHIALKTFIGVQ
VAIDQVTDQGQLFRTTGIITGASQGQSDGALTLYKLTISDPTYLWHKRRNSRVFMNKSVREITEILFQEWQG
KSPLFASSLTLDLSGLK-QAYDIRPFVMQLNQSDYDFLTRLWRSEGINWLIDEAELTVPSNTDHIQPQKLRL
IDDNSQYQALSRRTIRYHRSHATEQFDSMTSLMANRSLQPTSVFVQRWQSDVLQQTDGAGSVQSTHQHSTNY
DNQSLSLEDAWHFSPAWMQDLKGEDGATSASNQQLEKFNQNLSAFHDAQSKQFIAQTTVRDTQVGYWFELNE
HPEIDQ-HNGSDKEFLIIGKHYYNQNNLPKDLNQQIQALVRQSHWSINNEE--------RQGNELILQRRHI
KTVPEYQPLQDRPQASVQRARVVGPEGESIHVDQWGRIKVRFLFTRADDHRHDGGAGSNDNDTDSAWVDVLT
PWAGEGYGARFLPRIGEIVVIDFFDGNVDRPFVVGRIHEAERHPTQFDQKGQLPDTKKLSGIRSEEVDGKGF
NQLRFDDTTGQISAQLQSSHAASQLNLGNLSHPKEKAESDGRGEGFELRTDQWGAVRAGSGLYVSTHKQDQA
QGTHLDAKETKQQLDGNLNNAKALSDVAKNQQTDPLEHL---------------------------------
----------------------------------------ENLKTFLEQIEE--------------------
---------------------------------------------------------------QDQGKAAAF
KQALMVLTAPNSIALASNEDIHLSADGQLSQTAGDSISLSTQKNLIAHAQNKISLFAAQEGARLYAAKGKLE
VQAQGE------------------------KSLIVSF-------------------------QRLYKG----
------------------------------------------------------------------------
------------------------------------------------------------------------
------------------------------------------------------------------------
-----------------------
>ab736_0
-MFNNISQVLESLGFLSQHRSVHLQFSDASLNSQVFLQRIEGQHYLNEGMTAELICLSTNAHIALKTFIGVQ
VAIDQVTDQGQLFRTTGIITGASQGQSDGALTLYKLTISDPTYLWHKRRNSRVFMNKSVREITEILFQEWQG
KSPLFASSLTLDLSGLK-QAYDIRPFVMQLNQSDYDFLTRLWRSEGINWLIDEAELTVPSNTDHIQPQKLRL
IDDNSQYQALSRRTIRYHRSHATEQFDSMTSLMANRSLQPTSVFVQRWQSDVLQQTDGAGSVQSTHQHSTNY
DNQSLSLEDAWHFSPAWMQDLKGEDGATSASNQQLEKFNQNLSAFHDAQSKQFIAQTTVRDTQVGYWFELNE
HPEIDQ-HNGSDKEFLIIGKHYYNQNNLPKDLNQQIQALVRQSHWSINNEE--------RQGNELILQRRHI
KTVPEYQPLQDRPQASVQRARVVGPEGESIHVDQWGRIKVRFLFTRADDHRHDGGAGSNDNDTDSAWVDVLT
PWAGEGYGARFLPRIGEIVVIDFFDGNVDRPFVVGRIHEAERHPTQFDQKGQLPDTKKLSGIRSEEVDGKGF
NQLRFDDTTGQISAQLQSSHAASQLNLGNLSHPKEKAESDGRGEGFELRTDQWGAVRAGSGLYVSTHKQDQA
QGTHLDAKETKQQLDGNLNNAKALSDVAKNQQTDPLEHL---------------------------------
```

----------------------------------------ENLKTFLEQIEE--------------------
---------------------------------------------------------------QDQGKAAAF
KQALMVLTAPNSIALASNEDIHLSADGQLSQTAGDSISLSTQKNLIAHAQNKISLFAAQEGARLYAAKGKLE
VQAQGE------------------------KSLIVSF-------------------------QRLYKG----
------------------------------------------------------------------------
------------------------------------------------------------------------
------------------------------------------------------------------------
-----------------------
>ATCC19606_0
-MFNNISQVLESLGFLSQHRSVHLQFSDASLNSQVFLQRIEGQHYLNEGMTAELICLSTNAHIALKTFIGVQ
VAIDQVTDQGQLFRTTGIITGASQGQSDGALTLYKLTISDPTYLWHKRRNSRVFMNKSVREITEILFQEWQG
KSPLFASSLTLDLSGLK-QAYDIRPFVMQLNQSDYDFLTRLWRSEGINWLIDEAELTVPSNTDHIQPQKLRL
IDDNSQYQALSRRTIRYHRSHATEQFDSMTSLMANRSLQPTSVFVQRWQSDVLQQTDGAGSVQSTHQHSTNY
DNQSLSLEDAWHFSPAWMQDLKGEDGATSASNQQLEKFNQNLSAFHDAQSKQFIAQTTVRDTQVGYWFELNE
HPEIDQ-HNGSDKEFLIIGKHYYNQNNLPKDLNQQIQALVRQSHWSINNEE--------RQGNELILQRRHI
KTVPEYQPLQDRPQASVQRARVVGPEGESIHVDQWGRIKVRFLFTRADDHRHDGGAGSNDNDTDSAWVDVLT
PWAGEGYGARFLPRIGEIVVIDFFDGNVDRPFVVGRIHEAERHPTQFDQKGQLPDTKKLSGIRSEEVDGKGF
NQLRFDDTTGQISAQLQSSHAASQLNLGNLSHPKEKAESDGRGEGFELRTDQWGAVRAGSGLYVSTHKQDQA
QGTHLDAKETKQQLDGNLNNAKALSDVAKNQQTDPLEHL---------------------------------
----------------------------------------ENLKTFLEQIEE--------------------
---------------------------------------------------------------QDQGKAAAF
KQALMVLTAPNSIALASNEDIHLSADGQLSQTAGDSISLSTQKNLIAHAQNKISLFAAQEGARLYAAKGKLE
VQAQGE------------------------KSLIVSF-------------------------QRLYKG----
------------------------------------------------------------------------
------------------------------------------------------------------------
------------------------------------------------------------------------
-----------------------
>AR_0088_32
-MFNNISQVLESIGFLSQHRSVYLQFSDASLNSQVFLQRIDGQHYLNQGMTAELICLSTNAHIPLKTFIGLQ
VAVDQVTDQGQQFRTTGIITGASQGQSDGALTLYKLTISDPTYLWHKRRNSRVFMNKSVKEISEILFQEWQG
KSPLFASSLTLDLSGLK-QTYDVRPFVMQLNESDYDFLTRLWRSEGISWLIDEAELTVASNTDNIQPQKLRL
IDDNSQYQALTRRTIRYHRSSATEQFDSMTSLIAGRHLQPTSVFVQRWQSDVLQQTDGAGSVQSTHQHSASY
DNQSLSLEDAWHFSPAWMQDLNGEDGATSASNQQLEKFNQNLSAYHDAQSKQFVAQTTVRDTQVGYWFELNE

HPEIDL-HSGADKEFLIIGKHYYNQNNLPKDLNQQIQTLITQSHWQSSHTK-------ERQGNQLILQRRHI
KTVPEYQPLQDRPQAAVQRARVVGPEGESIYVDQWGRIKVRFLFTRANDHSHDGGAGSNDNDTDSAWVDVVT
PWAGKGYGARFLPRVGEIVVIDFFDGNIDRPFVVGRIHEAERHPAQFDQKGQLPDTKKLSGIRSEEVDGKGF
NQLRFDDTTGQISAQLQSSHAVSQLNLGNLSHPKDKAESDGRGEGFELRTDQWGAVRAGKGLLVSTHKQDQA
QGTHLDAADAKQQIEGGLNNAKALSEVAKNQQTDPLEML---------------------------------
----------------------------------------ENLKTFIEQIEQ--------------------
---------------------------------------------------------------QDKEKAAAF
KQALMILTAPNSIAVASNEDIHLSADGQLSQTAGDSINISTQKNLIAHAQNKLSLFAAQQGARLYAGKGKVE
IQAQGDGADLIARKGIQIISTEDKIEINASKEIVITSGSSQIKINSSGIFPVTGGKFEVKAGQHVFMGGGSA
SSTLPALP------QLNIPPKELELKYVYDDLKPVAQAPYKLI--FQDGSMQEGILDSNGYAKVQVPAD--K
VQPKVYYGFSNMEAKPDQPKQQNSFKDKKVMSIVEAEQLIEQYNKQELDHLLDEYFPDEIQAMIDGQGVEYD
DHINDYVEKDLEQQNNPDDAIADQAEEIPLNKQSTPHSGFGEGI----------------------------
-----------------------
>HWAB8_32
-MFNNISQVLESIGFLSQHRSVYLQFSDASLNSQVFLQRIDGQHYLNQGMTAELICLSTNAHIPLKTFIGLQ
VAVDQVTDQGQQFRTTGIITGASQGQSDGALTLYKLTISDPTYLWHKRRNSRVFMNKSVKEISEILFQEWQG
KSPLFASSLTLDLSGLK-QTYDVRPFVMQLNESDYDFLTRLWRSEGISWLIDEAELTVASNTDNIQPQKLRL
IDDNSQYQALTRRTIRYHRSSATEQFDSMTSLIAGRHLQPTSVFVQRWQSDVLQQTDGAGSVQSTHQHSASY
DNQSLSLEDAWHFSPAWMQDLNGEDGATSASNQQLEKFNQNLSAYHDAQSKQFVAQTTVRDTQVGYWFELNE
HPEIDL-HSGADKEFLIIGKHYYNQNNLPKDLNQQIQTLITQSHWQSSHTK-------ERQGNQLILQRRHI
KTVPEYQPLQDRPQAAVQRARVVGPEGESIYVDQWGRIKVRFLFTRANDHSHDGGAGSNDNDTDSAWVDVVT
PWAGKGYGARFLPRVGEIVVIDFFDGNIDRPFVVGRIHEAERHPAQFDQKGQLPDTKKLSGIRSEEVDGKGF
NQLRFDDTTGQISAQLQSSHAVSQLNLGNLSHPKDKAESDGRGEGFELRTDQWGAVRAGKGLLVSTHKQDQA
QGTHLDAADAKQQIEGGLNNAKALSEVAKNQQTDPLEML---------------------------------
----------------------------------------ENLKTFIEQIEQ--------------------
---------------------------------------------------------------QDKEKAAAF
KQALMILTAPNSIAVASNEDIHLSADGQLSQTAGDSINISTQKNLIAHAQNKLSLFAAQQGARLYAGKGKVE
IQAQGDGADLIARKGIQIISTEDKIEINASKEIVITSGSSQIKINSSGIFPVTGGKFEVKAGQHVFMGGGSA
SSTLPALP------QLNIPPKELELKYVYDDLKPVAQAPYKLI--FQDGSMQEGILDSNGYAKVQVPAD--K
VQPKVYYGFSNMEAKPDQPKQQNSFKDKKVMSIVEAEQLIEQYNKQELDHLLDEYFPDEIQAMIDGQGVEYD
DHINDYVEKDLEQQNNPDDAIADQAEEIPLNKQSTPHSGFGEGI----------------------------
-----------------------
>CIP70.10_2

-MFNNISQVLESIGFLSQHRSVYLQFSDASLNSQVFLQRIDGQHYLNQGMTAELICLSTNAHIPLKTFIGLQ
VAVDQVTDQGQQFRTTGIITGASQGQSDGALTLYKITISDPTYLWHKRRNSRVFMNKSVRDISEILFKEWQG
KSPLFASSLTLDLSGLK-QTYDVRPFVMQLNESDYDFLTRLWRSEGISWLIDEAELTVASNTDNIQPQKLRL
IDDNSQYQALTRRTIRYHRSSATEQFDSMTSLIAGRHLQPTSVFVQRWQSDVLQQTDGAGSVQSTHQHSTSY
DNQSLSLEDAWHFSPAWMQDLNGEDGATSASNQQLEKFNQNLSAYHDAQSKQFVAQTTVRDTQVGYWFELNE
HPEIDL-HSGADKEFLIIGKHYYNQNNLPKDLNQQIQTLITQSHWQSSHTK-------ERQGNQLILQRRHI
KTVPEYQPLQDRPQASVQRARVVGPEGESIYVDQWGRIKVRFLFTRANDHSHDGGAGSNDKDTDSAWVDVVT
PWAGKGYGARFLPRVGEIVVIDFFDGNIDRPFVVGRIHEAERHPAQFDQKGQLPDTKKLSGIRSEEVDGKGF
NQLRFDDTTGQISAQLQSSHAVSQLNLGNLSHPKDKAESDGRGEGFELRTDQWGAVRAGKGLLVSTHKQDQA
QGTHLNAADAKQQIEGGLNNAKALSEVAKNQQTDPLEML---------------------------------
----------------------------------------ENLKTFIEQIEQ--------------------
---------------------------------------------------------------QDKEKAATF
KQALMVLTAPNSIAVASNEDIHLSADGQLSQNAGDSISLSTQKNLIAHAQNKISLFAAQQGARLYAGKGKVE
IQAQGDGADLIARKGIQIISTEDKIEINASKEIVITSGGSQIKINSSGIFPVTGGKFEVKAGQHVFMGGAKA
DYVLPSLP------TQEIKTDDLLLEYLHSDGTPVKGADYEVL--LSDGSIRKGKLDASGKAIVSGVPA-GR
AKIQYGEDQSKDEFPALEVDDWFTQLGSSTKTGKEE------------------------------------
------------------------------------------------------------------------
-----------------------
>R2091_2
-MFNNISQVLESIGFLSQHRSVYLQFSDASLNSQVFLQRIDGQHYLNQGMTAELICLSTNAHIPLKTFIGLQ
VAVDQVTDQGQQFRTTGIITGASQGQSDGALTLYKITISDPTYLWHKRRNSRVFMNKSVRDISEILFKEWQG
KSPLFASSLTLDLSGLK-QTYDVRPFVMQLNESDYDFLTRLWRSEGISWLIDEAELTVASNTDNIQPQKLRL
IDDNSQYQALTRRTIRYHRSSATEQFDSMTSLIAGRHLQPTSVFVQRWQSDVLQQTDGAGSVQSTHQHSTSY
DNQSLSLEDAWHFSPAWMQDLNGEDGATSASNQQLEKFNQNLSAYHDAQSKQFVAQTTVRDTQVGYWFELNE
HPEIDL-HSGADKEFLIIGKHYYNQNNLPKDLNQQIQTLITQSHWQSSHTK-------ERQGNQLILQRRHI
KTVPEYQPLQDRPQASVQRARVVGPEGESIYVDQWGRIKVRFLFTRANDHSHDGGAGSNDKDTDSAWVDVVT
PWAGKGYGARFLPRVGEIVVIDFFDGNIDRPFVVGRIHEAERHPAQFDQKGQLPDTKKLSGIRSEEVDGKGF
NQLRFDDTTGQISAQLQSSHAVSQLNLGNLSHPKDKAESDGRGEGFELRTDQWGAVRAGKGLLVSTHKQDQA
QGTHLNAADAKQQIEGGLNNAKALSEVAKNQQTDPLEML---------------------------------
----------------------------------------ENLKTFIEQIEQ--------------------
---------------------------------------------------------------QDKEKAATF
KQALMVLTAPNSIAVASNEDIHLSADGQLSQNAGDSISLSTQKNLIAHAQNKISLFAAQQGARLYAGKGKVE
IQAQGDGADLIARKGIQIISTEDKIEINASKEIVITSGGSQIKINSSGIFPVTGGKFEVKAGQHVFMGGAKA

DYVLPSLP------TQEIKTDDLLLEYLHSDGTPVKGADYEVL--LSDGSIRKGKLDASGKAIVSGVPA-GR
AKIQYGEDQSKDEFPALEVDDWFTQLGSSTKTGKEE------------------------------------
------------------------------------------------------------------------
-----------------------

>ATCC17978-mff_2
-MFNNISQVLESIGFLSQHRSVYLQFSDASLNSQVFLQRIDGQHYLNQGMTAELICLSTNAHIPLKTFIGLQ
VAVDQVTDRGSFFRTTGIITGASQGQSDGALTLYKLAISDPTYLWHKRRNSRVFMNKSVKEISEILFQEWQG
KSPLFASSLTLDLSGLK-QTYDVRPFVMQLNESDYDFLTRLWRSEGISWLIDEAELTVASNTDNIQPQKLRL
IDDNSQYQTLTRRTIRYHRSSATEQFDSMTSLIAGRHLQPSSVFVQRWQSDVLQQTDGAGSVQSKHEHSSNY
DNQSLNLEDAWHFSPAWMQDLNGEDGATSASNQQLEKFNQNLSAYHDAQSKQFVAQTTVRDTQVGYWFELNE
HPEIDL-HSGADKEFLIIGKHYYNQNNLPKDLDQQIQTLTTQSHWQSSHTK-------ERQGNQLILQRRHI
KTVPEYQPLQDRPQASVQRARVVGPEGESIYVDQWGRIKVRFLFTRANDHSHDGGAGSNDNDTDSAWVDVLT
PWAGAGYGARFLPRVGEIVVIDFFDGNIDRPFVVGRIHEAERHPAQFDQKGQLPDTKKLSGIRSEEVDGKGF
NQLRFDDTTGQISAQLQSSHAVSQLNLGNLSHPKDKAESDGRGEGFELRTDQWGAVRAGKGLLVSTHKQDQA
QGTHLDAADAKQQIEGGLNNAKALSEVAKNQQTDPLEML---------------------------------
----------------------------------------ENLKTFIEQIEE--------------------
---------------------------------------------------------------KDQDKAATF
KQALMVLTAPNSIAVASNEDIHLSADGQLSQNAGDSISLSTQKNLIAHAQNKISLFAAQQGARLYAGKGKVE
IQAQGDGADLIARKGIQIISTEDKIEINASKEIVITSGGSQIKINSSGIFPVTGGKFEVKAGQHVFMGGAKA
DYVLPSLP------TQEIKTDDLLLEYLHSDGTPVKGADYEVL--LSDGSIRKGKLDASGKAIVSGVPA-GR
AKIQYGEDQSKDEFPALEVDDWFTQLGSSTKTGKEE------------------------------------
------------------------------------------------------------------------
-----------------------

>VgrG1Abay
-MLNNIHSVFEQLGFGALKRAIHVQFSNPLLNTQIFLQRIDGKHTLNEGLEVELTCLSINAHIALKQFIGCQ
VAIDQVTDTGQLARTSGIITRATQGQSDGALTLYTLNIQDSTALWHKRRNSRVFMNKSVAEISEILFKEWQN
RSSLFAASLTLDLSGLA-QNYDIRPFTMQINETDYEFLTRLWRSEGINWLVDEKNTVVTHPLQPIAPQKLRL
IDDNQQFLALSRGLIRFHRSHATEHFDSMTHLVAHRQLQSTAAYVQRWQADGLLQDEGSGSVLSAHQHSDQQ
SNESLSLEQAWSIGSAWTTDLTGADQATASSSQQIEKLNQNLTAYQERQAKYFIAQTTVRDAQVGYWFELQG
HPEIDQ-HDLADRQFLIYEKRFFNQNNLPKDIQQQFEYLLMHSALKTSNQQ-------ERQGNELTLIRRQV
KVVPEYQPLMHRPVVYPQRARVVGPENEEIYVDEWGRIKVRFLFTRTEDHAHDGGAGSNDNDTDSAWVDVLT
PWAGEGYGARFLPRVGELVVIDFFDGHVDRPFATGRIHEAQRSPTKFDIRGQLPATRNLSGIRSQEIAGSGF
NQLRFDDTTGQISTQLQSSHGASQLNLGNLSHPKNEETSQGRGEGFELRTDHYGAVRAGKGMLISTYAQEQA

IADHLEAAQAQSLLAQSHENMKTLSDIALKQQTDALNVIDRLPKFIQSLELKSTEQALDRTVNLFKDNLNKD
PINALKDCGSFIEDIGRLGGNTKGVVEEFDTFFTQNKDALENLKAFIENVEEHGTDVVKGRLASVKDDIQQN
PFASLQDIGKVLANVDIKDFDLMSNCGTFSKGSKLEVTPSKALSSLQGFMEGYTQGLESSSDAKQQEQGKIF
RQALMLLASPNGIALTTPENIILQASQDIAESASGSINLSAQKNIIGHAQDKVSLFAAQKGLSAYAAKGAVK
IQAQDDLVDIIAKKVIKLISTEDKIELTSTKEIDIKAGGSQLIVNGNGVFIKTGGKFEVRSGQHVFTSGEKV
SYEVPELP------NTSIFSNRLDIYDLFWASDF-SQLSYKAFV-PETSSFTSGSIDEHGRTGKISTSD--P
TKVQVLVGSNDEWGLVIDGFDEDDFMTDQNVNDSESDNNIEVRDLK------------------------
----------------------------------------------------------------------
----------------------
>ab736_9
-MLFNIFSVLEKIGLNAQKRAIHVQFSNELLNHQVFLQRIEGQHQLNGGLMAELICLSTNAQIALKQFIGVQ
VAVDQVTDSGQLFRTTGIVTEASYGQSDGALTLYKLTIEDATNLWHKRRNSRVFMNKSIVEITEVLFKEWQE
KSPLFAASLSLDLGGLS-QNYDIRPFTMQHNESDYDFLTRLWRSEGVSWLIDESKLFVPHFTAPIQGQKLRL
IDDNNQYQALARRSIRYHRSSATEYQDSITGFVAVRSLQPTAVHVQRWQPDALAQEEGNGSVVTTHTHSDNF
DSATLSLEQAWHVSPAWMQDLKGEDQATASSSSQLEKLNQHFTDMHASRAKYFKAYSSVRDTQVGYWFNLRE
HPEIDQ-HEGADQEFLIIAKNFYNQNNLPKDLHQQVSQLLTQSRWDQHSYD-----DIERQSNELTLIRRQI
KTAPEYNPEQHRPIAYPQRAKVVGPEGETIHVDEWGRIKVRFLFTRSDDHGHDGGAGSNDNDTDSAWVDVLT
PWAGEGYGARFLPRIGEVVVIDFFDGNIDRPFVTGRIHEAQRSPTKFDVKGQLPDTKKLSGIRSQEINGSGF
NQLRFDDTTGQISTQLQSSHAATQLNLGNLSHPKEQATSQGRGEGFELRTDAWGAVRAGKGLYISTHKQDQA
QGFHLNANETKQQLEGGLNNSKALSEVAKNQQTDPLEVL-------------------------------
---------------------------------------DHLKTFLDQIEQ-------------------
--------------------------------------------------------------RDKDKAAAF
KQAIMVLSAPNSMALTSNENIHVIADGQISQTADDSINLSTQKNFIAHAQNKISFLQLKMEQECMRARAKLS
YK----------HKVMVQISLHEK-----------------------------------GYRLFQQKIES
K---------------------------------------------------------------------
----------------------------------------------------------------------
----------------------------------------------------------------------
----------------------
>ATCC19606_9
-MLFNIFSVLEKIGLNAQKRAIHVQFSNELLNHQVFLQRIEGQHQLNGGLMAELICLSTNAQIALKQFIGVQ
VAVDQVTDSGQLFRTTGIVTEASYGQSDGALTLYKLTIEDATNLWHKRRNSRVFMNKSIVEITEVLFKEWQE
KSPLFAASLSLDLGGLS-QNYDIRPFTMQHNESDYDFLTRLWRSEGVSWLIDESKLFVPHFTAPIQGQKLRL
IDDNNQYQALARRSIRYHRSSATEYQDSITGFVAVRSLQPTAVHVQRWQPDALAQEEGNGSVVTTHTHSDNF

DSATLSLEQAWHVSPAWMQDLKGEDQATASSSSQLEKLNQHFTDMHASRAKYFKAYSSVRDTQVGYWFNLRE
HPEIDQ-HEGADQEFLIIAKNFYNQNNLPKDLHQQVSQLLTQSRWDQHSYD-----DIERQSNELTLIRRQI
KTAPEYNPEQHRPIAYPQRAKVVGPEGETIHVDEWGRIKVRFLFTRSDDHGHDGGAGSNDNDTDSAWVDVLT
PWAGEGYGARFLPRIGEVVVIDFFDGNIDRPFVTGRIHEAQRSPTKFDVKGQLPDTKKLSGIRSQEINGSGF
NQLRFDDTTGQISTQLQSSHAATQLNLGNLSHPKEQATSQGRGEGFELRTDAWGAVRAGKGLYISTHKQDQA
QGFHLNANETKQQLEGGLNNSKALSEVAKNQQTDPLEVL---------------------------------
----------------------------------------DHLKTFLDQIEQ--------------------
---------------------------------------------------------------RDKDKAAAF
KQAIMVLSAPNSMALTSNENIHVIADGQISQTADDSINLSTQKNFIAHAQNKISFLQLKMEQECMRARAKLS
YK----------HKVMVQISLHEK-------------------------------------GYRLFQQKIES
K-----------------------------------------------------------------------
------------------------------------------------------------------------
------------------------------------------------------------------------
-----------------------
>IOMTU433_0
--------------------------------------------------MAELICLSTNAQIALKQFIGVQ
VAVDQVTDSGQLFRTTGIVTEASYGQSDGALTLYKLTIEDATNLWHKRRNSRVFMNKSIVEITEVLFKEWQE
KSPLFAASLSLDLGGLS-QNYDIRPFTMQHNESDYDFLTRLWRSEGVSWLIDESELFVPHFTAPIQAQKLRL
IDDNNQYQALARRSIRYHRSSATEYQDSITGFVAVRSLQPTAVHVQRWQPDALAQEEGNGSVVTTHTHSDNF
DSATLSLEQAWHVSPAWMQDLKGEDQATASSSSQLEKLNQHFTDMHASRAKYFKAYSSVRDTQVGYWFNLRE
HPEIDQ-HEGADQEFLIIAKNFYNQNNLPKDLHQQVSQLLTQSRWDQHGYD-----DIERQGNELTLIRRQI
KTAPEYNPEQHRPIAYPQRAKVVGPEGETIHVDEWGRIKVRFLFTRSDDHGHDGGAGSNDNDTDSAWVDVLT
PWAGEGYGARFLPRIGEVVVIDFFDGNIDRPFVTGRIHEAQRSPTKFDVKGQLPDTKKLSGIRSQEINGSGF
NQLRFDDTTGQISTQLQSSHAATQLNLGNLSHPKEQATSQGRGEGFELRTDAWGAVRAGKGMLISTYAQEQA
IADHLEAAQAQSLLSQGYESMKMLSEVAAKQQTDALNVINRLPKFIQSLELKTTGQALNSTVNLFKEGINND
PIHALKDCGGFIQDIGALGGNAKGVVDEFNSFFSDAKDAVENLKAFIENVEEHGPDIVKGKLASIKDRIHKN
PFESIQEVGRVLANVETKDFDLMSTCGTFSKGSKLEVSPSKALSSLQGFMEGYTQGLESSSDTKQQEQGKIF
RQALMLLASPNGIALTTPENIILQASQDIAESASGSINLSAQKNIIGHAQDKISLFAAQKGLSAFAAKGPIK
VQAQTEGIEILSRKNIKILSVEDKIEIVGQKEIVLNAGGSQLTISDKGVFINTPRLFHAKAGQHKFDAGAII
NYSFPNLP--------SMYYGNFNITDK--NNNPIGGQKYKMT--LPSGKEILGFTDENGNTVTGYSGE---
-------DNQNLKLEIIEDLYQDIWYQPNSTYEYETIDDLELPVNFDKEVDEDE------------------
------------------------------------------------------------------------
-----------------------

```
>AB031_25
-MLFNIFSVLEKIGLNAQKRAIHVQFSNELLNNQIFLQRIEGQHQLNGGLMAELICLSTNAQIALKQFIGVQ
VAVDQVTDSGQLFRTTGIVTEASYGQSDGALTLYKLTIEDATNLWHKRRNSRVFMNKSIVEITEVLFKEWQE
KSPLFAASLSLDLGGLS-QNYDIRPFTMQHNESDYDFLTRLWRSEGISWLIDESELFVPHFTAPIQAQKLRL
IDDNSQYQALERRSIRYHRSSATEYQDSITGFVAVRSLQPTAVHVQRWQPDALAQEEGNGSVVTTHTHSDSF
DSATLSLEQAWHVSPAWMQDLKGEDQATASGSSQLEKLNQHFTDMHASRAKYFKAYSSVRDSQVGYWFNLRE
HPEIDQ-HEGADQEFLIIAKNFYNQNNLPKDLHQQVSQLLTQSHWDQHGYD-----DIERQGNELTLIRRQI
KTAPEYNPEQHRPIAYPQRAKVVGPQGETIHVDEWGRIKVRFLFTRSDDHGHDGGAGSNDNDTDSAWVDVLT
PWAGEGYGARFLPRIGEVVVIDFFDGNIDRPFVTGRIHEAQRSPTKFDIKGQLPATKKLSGIRSQEINGSGF
NQLRFDDTTGQISTQLQSSHAATQLNLGNLSHPKEQETSQGRGEGFELRTDAWGAVRAGKGMLISTYAQEQA
IADHLEAAQAQSLLSQGYESMKMLSEVAAKQQTDALNVINRLPKFIQSLELKTTGQALNSTVNLFKEGINND
PIHALKDCGGFIQDIGALGGNAKGVVDEFNAFFTDAKDAVENLKAFIENVEEHGADIVKGKLASIKDRIHKN
PFESIKEVGKVLANVETKDFDMMSVCGTFSKGSKLEVSPSKALSSLQGFMEGYTQGLESSSDTKQQEQGKIF
RQALMLLASPNGIALTTPENIILQASQDIAESASGSINLSAQKNIIGHAQDKISLFAAQKGLRAYAAKGKLE
LQAQDDAIEAIAKKVIKLISTEDKIELTSPKEIVLTAGGSQLKINANGVFSTTGGKFESKAGQHLFTSGATV
NAELPKMP------ESGIFSRRFDFSELINTNLLKDGFKYKAIN-HTKKTEYIGFLDEFARTRRIFSDN-PD
NIEILLLGKNDEHSDKLQLVEEIINDGQGKSSDETCCGGGDEHNHDHDSNEQIEDDDLKSDFENFRIKD---
--------------------------------------------------------------------------------
-------------------------
>3207_4
-MLFNIFSVLEKIGLNAQKRAIHVQFSNELLNHQVFLQRIEGQHQLNGGLMAELICLSTNAQIALKQFIGVQ
VAVDQVTDSGQLFRTTGIVTEASYGQSDGALTLYKLTIEDATNLWHKRRNSRVFMNKSIVEITEVLFKEWQE
KSPLFAASLSLDLGGLS-QNYDIRPFTMQHNESDYDFLTRLWRSEGVSWLIDESELFVPHFTAPIQAQKLKL
IDDNNQYQALARRSIRYHRSSATEYQDSITGFVAVRSLQPTAVHVQRWQPDALAQEEGNGSVVTTHTHSDNF
DSATLSLEQAWHVSPAWMQDLKGEDQATASSSSQLEKLNQHFTDMHASRAKYFKAYSSVRDTQVGYWFNLRE
HPEIDQ-HEGADQEFLIIAKNFYNQNNLPKDLQQQVSQLLTQSRWDQHGYD-----DIERQSNELTLIRRQI
KTAPEYNPEQHRPIAYPQRAKVVGPEGETIHVDEWGRIKVRFLFTRSDDHGHDGGAGSNDNDTDSAWVDVLT
PWAGEGYGARFLPRIGEVVVIDFFDGNIDRPFVTGRIHEAQRSPTKFDVKGQLPATKKLSGIRSQEINGSGF
NQLRFDDTTGQISTQLQSSHAATQLNLGNLSHPKEQETSQGRGEGFELRTDAWGAVRAGKGMLISTYAQEQA
IADHLEAAQAQSLLSQGYESMKMLSEVAAKQQTDALNVINRLPKFIQSLELKTTGQALNSTINLFKEGINND
PIHALKDCGGFIQDIGALGGNAKGVVDEFNAFFTDAKDAVENLKAFIENVEEHGADIVKGKLASIKDRIHKN
PFESIQEVGKVLANVETKDFDLMSTCGTFSKGSKLEVSPSKALSSLQGFMEGYTQGLESSSDTKQQEQGKIF
RQALMLLASPNGIALTTPENIILQASQDIAESASGSINLSAQKNIIGHAQDKISLFAAQKGLRAYAAKGKLE
```

LQAQDDAIEAIAKKVIKLISTEEKIELTSPKEIVLTAGGSQLKINANGVFSTTGGKFESKAGQHLFTSGAKV
SYEVPELP------SSGPFTKAFHFVSL--EGEKIENAIIQVY--EKINDSYKFKIGSKIDKSIFKLEE---
------SEENIEYKALVGFDDWVSWFEEEEGEEVEEIDLGEHYNPFDK------------------------
------------------------------------------------------------------------
-----------------------

>15A5_18
-MLFNIFSVLEKIGLNAQKRAIHVQFSNELLNHQVFLQRIEGQHQLNGGLMAELICLSTNAQIALKQFIGVQ
VAVDQVTDSGQLFRTTGIVTEASYGQSDGALTLYKLTIEDATNLWHKRRNSRVFMNKSIVEITEVLFKEWQE
KSPLFAASLSLDLGGLS-QNYDIRPFTMQHNEADYDFLTRLWRSEGVSWLIDESELFVPHFTAPIQAQKLRL
IDDNNQYQALARRSIRYHRSSATEYQDSITGFVAVRSLQPTAVHVQRWQPDALAQEEGNGSVVTTHTHSDNF
DSATLSLEQAWHVSPAWMQDLKGEDQATASSSSQLEKLNQHFTDMHASRAKYFKAYSSVRDTQVGYWFNLRE
HPEIDQ-HEGADQEFLIIAKNFYNQNNLPKDLQQQVSQLLTQSRWDQHGYD-----DIERQGNELTLIRRQI
KTAPEYNPEQHRPIAYPQRAKVVGPEGETIHVDEWGRIKVRFLFTRSDDHGHDGGAGSNDNDTDSAWVDVLT
PWAGEGYGARFLPRIGEVVVIDFFDGNIDRPFVTGRIHEAQRSPTKFDVKGQLPDTKKLSGIRSQEINGSGF
NQLRFDDTTGQISTQLQSSHAATQLNLGNLSHPKEQATSQGRGEGFELRTDAWGAVRAGKGMLISTYAQEQA
IADHLEAAQAQSLLSQGYESMKMLSEVAAKQQTDALNVINRLPKFIQSLELKTTGQALNSTVNLFKEGINND
PIHALKDCGGFIQDIGALGGNAKGVVDEFNSFFSDAKDAVENLKAFIENVEEHGPDIVKGKLASIKDRIHKN
PFESIQEVGKVLTNVETKDFDLMSTCGTFSKGSKLEVSPSKALSSLQGFMEGYTQGLESSSDTQQQEQGKIF
RQALMLLASPNGIALTTPENIILQASQDIAESASGSINLSAQKNIIGHAQDKISLFAAQKGLRAYAAKGKLE
LQAQDDAIEAIAKKVIKLISTEDKIELTSPKEIVLTAGGSQLKINANGVFSTTGGKFESKAGQHLFTGGAKV
SYEVPELP--------KNGPYAVDFLFASLAGNGIENAKIQLYEPDKKEIIWEGKTDLRGKSNLSLQNE---
---------SKRYEALIGFDDWSSIFDDEDDYEEEHEEEFEIGEHGMQAEENNLEE----------------
------------------------------------------------------------------------
-----------------------

>3027STDY5784958_18
-MLFNIFSVLEKIGLNAQKRAIHVQFSNELLNHQVFLQRIEGQHQLNGGLMAELICLSTNAQIALKQFIGVQ
VAVDQVTDSGQLFRTTGIVTEASYGQSDGALTLYKLTIEDATNLWHKRRNSRVFMNKSIVEITEVLFKEWQE
KSPLFAASLSLDLGGLS-QNYDIRPFTMQHNEADYDFLTRLWRSEGVSWLIDESELFVPHFTAPIQAQKLRL
IDDNNQYQALARRSIRYHRSSATEYQDSITGFVAVRSLQPTAVHVQRWQPDALAQEEGNGSVVTTHTHSDNF
DSATLSLEQAWHVSPAWMQDLKGEDQATASSSSQLEKLNQHFTDMHASRAKYFKAYSSVRDTQVGYWFNLRE
HPEIDQ-HEGADQEFLIIAKNFYNQNNLPKDLQQQVSQLLTQSRWDQHGYD-----DIERQGNELTLIRRQI
KTAPEYNPEQHRPIAYPQRAKVVGPEGETIHVDEWGRIKVRFLFTRSDDHGHDGGAGSNDNDTDSAWVDVLT
PWAGEGYGARFLPRIGEVVVIDFFDGNIDRPFVTGRIHEAQRSPTKFDVKGQLPDTKKLSGIRSQEINGSGF

NQLRFDDTTGQISTQLQSSHAATQLNLGNLSHPKEQATSQGRGEGFELRTDAWGAVRAGKGMLISTYAQEQA
IADHLEAAQAQSLLSQGYESMKMLSEVAAKQQTDALNVINRLPKFIQSLELKTTGQALNSTVNLFKEGINND
PIHALKDCGGFIQDIGALGGNAKGVVDEFNSFFSDAKDAVENLKAFIENVEEHGPDIVKGKLASIKDRIHKN
PFESIQEVGKVLTNVETKDFDLMSTCGTFSKGSKLEVSPSKALSSLQGFMEGYTQGLESSSDTQQQEQGKIF
RQALMLLASPNGIALTTPENIILQASQDIAESASGSINLSAQKNIIGHAQDKISLFAAQKGLRAYAAKGKLE
LQAQDDAIEAIAKKVIKLISTEDKIELTSPKEIVLTAGGSQLKINANGVFSTTGGKFESKAGQHLFTGGAKV
SYEVPELP--------KNGPYAVDFLFASLAGNGIENAKIQLYEPDKKEIIWEGKTDLRGKSNLSLQNE---
---------SKRYEALIGFDDWSSIFDDEDDYEEEHEEEFEIGEHGMQAEENNLEE----------------
------------------------------------------------------------------------
-----------------------
>AB07_18
-MLFNIFSVLEKIGLNAQKRAIHVQFSNELLNHQVFLQRIEGQHQLNGGLMAELICLSTNAQIALKQFIGVQ
VAVDQVTDSGQLFRTTGIVTEASYGQSDGALTLYKLTIEDATNLWHKRRNSRVFMNKSIVEITEVLFKEWQE
KSPLFAASLSLDLGGLS-QNYDIRPFTMQHNEADYDFLTRLWRSEGVSWLIDESELFVPHFTAPIQAQKLRL
IDDNNQYQALARRSIRYHRSSATEYQDSITGFVAVRSLQPTAVHVQRWQPDALAQEEGNGSVVTTHTHSDNF
DSATLSLEQAWHVSPAWMQDLKGEDQATASSSSQLEKLNQHFTDMHASRAKYFKAYSSVRDTQVGYWFNLRE
HPEIDQ-HEGADQEFLIIAKNFYNQNNLPKDLQQQVSQLLTQSRWDQHGYD-----DIERQGNELTLIRRQI
KTAPEYNPEQHRPIAYPQRAKVVGPEGETIHVDEWGRIKVRFLFTRSDDHGHDGGAGSNDNDTDSAWVDVLT
PWAGEGYGARFLPRIGEVVVIDFFDGNIDRPFVTGRIHEAQRSPTKFDVKGQLPDTKKLSGIRSQEINGSGF
NQLRFDDTTGQISTQLQSSHAATQLNLGNLSHPKEQATSQGRGEGFELRTDAWGAVRAGKGMLISTYAQEQA
IADHLEAAQAQSLLSQGYESMKMLSEVAAKQQTDALNVINRLPKFIQSLELKTTGQALNSTVNLFKEGINND
PIHALKDCGGFIQDIGALGGNAKGVVDEFNSFFSDAKDAVENLKAFIENVEEHGPDIVKGKLASIKDRIHKN
PFESIQEVGKVLTNVETKDFDLMSTCGTFSKGSKLEVSPSKALSSLQGFMEGYTQGLESSSDTQQQEQGKIF
RQALMLLASPNGIALTTPENIILQASQDIAESASGSINLSAQKNIIGHAQDKISLFAAQKGLRAYAAKGKLE
LQAQDDAIEAIAKKVIKLISTEDKIELTSPKEIVLTAGGSQLKINANGVFSTTGGKFESKAGQHLFTGGAKV
SYEVPELP--------KNGPYAVDFLFASLAGNGIENAKIQLYEPDKKEIIWEGKTDLRGKSNLSLQNE---
---------SKRYEALIGFDDWSSIFDDEDDYEEEHEEEFEIGEHGMQAEENNLEE----------------
------------------------------------------------------------------------
-----------------------
>AB34299_18
-MLFNIFSVLEKIGLNAQKRAIHVQFSNELLNHQVFLQRIEGQHQLNGGLMAELICLSTNAQIALKQFIGVQ
VAVDQVTDSGQLFRTTGIVTEASYGQSDGALTLYKLTIEDATNLWHKRRNSRVFMNKSIVEITEVLFKEWQE
KSPLFAASLSLDLGGLS-QNYDIRPFTMQHNEADYDFLTRLWRSEGVSWLIDESELFVPHFTAPIQAQKLRL

IDDNNQYQALARRSIRYHRSSATEYQDSITGFVAVRSLQPTAVHVQRWQPDALAQEEGNGSVVTTHTHSDNF
DSATLSLEQAWHVSPAWMQDLKGEDQATASSSSQLEKLNQHFTDMHASRAKYFKAYSSVRDTQVGYWFNLRE
HPEIDQ-HEGADQEFLIIAKNFYNQNNLPKDLQQQVSQLLTQSRWDQHGYD-----DIERQGNELTLIRRQI
KTAPEYNPEQHRPIAYPQRAKVVGPEGETIHVDEWGRIKVRFLFTRSDDHGHDGGAGSNDNDTDSAWVDVLT
PWAGEGYGARFLPRIGEVVVIDFFDGNIDRPFVTGRIHEAQRSPTKFDVKGQLPDTKKLSGIRSQEINGSGF
NQLRFDDTTGQISTQLQSSHAATQLNLGNLSHPKEQATSQGRGEGFELRTDAWGAVRAGKGMLISTYAQEQA
IADHLEAAQAQSLLSQGYESMKMLSEVAAKQQTDALNVINRLPKFIQSLELKTTGQALNSTVNLFKEGINND
PIHALKDCGGFIQDIGALGGNAKGVVDEFNSFFSDAKDAVENLKAFIENVEEHGPDIVKGKLASIKDRIHKN
PFESIQEVGKVLTNVETKDFDLMSTCGTFSKGSKLEVSPSKALSSLQGFMEGYTQGLESSSDTQQQEQGKIF
RQALMLLASPNGIALTTPENIILQASQDIAESASGSINLSAQKNIIGHAQDKISLFAAQKGLRAYAAKGKLE
LQAQDDAIEAIAKKVIKLISTEDKIELTSPKEIVLTAGGSQLKINANGVFSTTGGKFESKAGQHLFTGGAKV
SYEVPELP--------KNGPYAVDFLFASLAGNGIENAKIQLYEPDKKEIIWEGKTDLRGKSNLSLQNE---
---------SKRYEALIGFDDWSSIFDDEDDYEEEHEEEFEIGEHGMQAEENNLEE----------------
------------------------------------------------------------------------
-----------------------
>Ab4568_18
-MLFNIFSVLEKIGLNAQKRAIHVQFSNELLNHQVFLQRIEGQHQLNGGLMAELICLSTNAQIALKQFIGVQ
VAVDQVTDSGQLFRTTGIVTEASYGQSDGALTLYKLTIEDATNLWHKRRNSRVFMNKSIVEITEVLFKEWQE
KSPLFAASLSLDLGGLS-QNYDIRPFTMQHNEADYDFLTRLWRSEGVSWLIDESELFVPHFTAPIQAQKLRL
IDDNNQYQALARRSIRYHRSSATEYQDSITGFVAVRSLQPTAVHVQRWQPDALAQEEGNGSVVTTHTHSDNF
DSATLSLEQAWHVSPAWMQDLKGEDQATASSSSQLEKLNQHFTDMHASRAKYFKAYSSVRDTQVGYWFNLRE
HPEIDQ-HEGADQEFLIIAKNFYNQNNLPKDLQQQVSQLLTQSRWDQHGYD-----DIERQGNELTLIRRQI
KTAPEYNPEQHRPIAYPQRAKVVGPEGETIHVDEWGRIKVRFLFTRSDDHGHDGGAGSNDNDTDSAWVDVLT
PWAGEGYGARFLPRIGEVVVIDFFDGNIDRPFVTGRIHEAQRSPTKFDVKGQLPDTKKLSGIRSQEINGSGF
NQLRFDDTTGQISTQLQSSHAATQLNLGNLSHPKEQATSQGRGEGFELRTDAWGAVRAGKGMLISTYAQEQA
IADHLEAAQAQSLLSQGYESMKMLSEVAAKQQTDALNVINRLPKFIQSLELKTTGQALNSTVNLFKEGINND
PIHALKDCGGFIQDIGALGGNAKGVVDEFNSFFSDAKDAVENLKAFIENVEEHGPDIVKGKLASIKDRIHKN
PFESIQEVGKVLTNVETKDFDLMSTCGTFSKGSKLEVSPSKALSSLQGFMEGYTQGLESSSDTQQQEQGKIF
RQALMLLASPNGIALTTPENIILQASQDIAESASGSINLSAQKNIIGHAQDKISLFAAQKGLRAYAAKGKLE
LQAQDDAIEAIAKKVIKLISTEDKIELTSPKEIVLTAGGSQLKINANGVFSTTGGKFESKAGQHLFTGGAKV
SYEVPELP--------KNGPYAVDFLFASLAGNGIENAKIQLYEPDKKEIIWEGKTDLRGKSNLSLQNE---
---------SKRYEALIGFDDWSSIFDDEDDYEEEHEEEFEIGEHGMQAEENNLEE----------------
------------------------------------------------------------------------

-----------------------
>Ab4653_18
-MLFNIFSVLEKIGLNAQKRAIHVQFSNELLNHQVFLQRIEGQHQLNGGLMAELICLSTNAQIALKQFIGVQ
VAVDQVTDSGQLFRTTGIVTEASYGQSDGALTLYKLTIEDATNLWHKRRNSRVFMNKSIVEITEVLFKEWQE
KSPLFAASLSLDLGGLS-QNYDIRPFTMQHNEADYDFLTRLWRSEGVSWLIDESELFVPHFTAPIQAQKLRL
IDDNNQYQALARRSIRYHRSSATEYQDSITGFVAVRSLQPTAVHVQRWQPDALAQEEGNGSVVTTHTHSDNF
DSATLSLEQAWHVSPAWMQDLKGEDQATASSSSQLEKLNQHFTDMHASRAKYFKAYSSVRDTQVGYWFNLRE
HPEIDQ-HEGADQEFLIIAKNFYNQNNLPKDLQQQVSQLLTQSRWDQHGYD-----DIERQGNELTLIRRQI
KTAPEYNPEQHRPIAYPQRAKVVGPEGETIHVDEWGRIKVRFLFTRSDDHGHDGGAGSNDNDTDSAWVDVLT
PWAGEGYGARFLPRIGEVVVIDFFDGNIDRPFVTGRIHEAQRSPTKFDVKGQLPDTKKLSGIRSQEINGSGF
NQLRFDDTTGQISTQLQSSHAATQLNLGNLSHPKEQATSQGRGEGFELRTDAWGAVRAGKGMLISTYAQEQA
IADHLEAAQAQSLLSQGYESMKMLSEVAAKQQTDALNVINRLPKFIQSLELKTTGQALNSTVNLFKEGINND
PIHALKDCGGFIQDIGALGGNAKGVVDEFNSFFSDAKDAVENLKAFIENVEEHGPDIVKGKLASIKDRIHKN
PFESIQEVGKVLTNVETKDFDLMSTCGTFSKGSKLEVSPSKALSSLQGFMEGYTQGLESSSDTQQQEQGKIF
RQALMLLASPNGIALTTPENIILQASQDIAESASGSINLSAQKNIIGHAQDKISLFAAQKGLRAYAAKGKLE
LQAQDDAIEAIAKKVIKLISTEDKIELTSPKEIVLTAGGSQLKINANGVFSTTGGKFESKAGQHLFTGGAKV
SYEVPELP--------KNGPYAVDFLFASLAGNGIENAKIQLYEPDKKEIIWEGKTDLRGKSNLSLQNE---
---------SKRYEALIGFDDWSSIFDDEDDYEEEHEEEFEIGEHGMQAEENNLEE----------------
------------------------------------------------------------------------
-----------------------
>Ab4977_18
-MLFNIFSVLEKIGLNAQKRAIHVQFSNELLNHQVFLQRIEGQHQLNGGLMAELICLSTNAQIALKQFIGVQ
VAVDQVTDSGQLFRTTGIVTEASYGQSDGALTLYKLTIEDATNLWHKRRNSRVFMNKSIVEITEVLFKEWQE
KSPLFAASLSLDLGGLS-QNYDIRPFTMQHNEADYDFLTRLWRSEGVSWLIDESELFVPHFTAPIQAQKLRL
IDDNNQYQALARRSIRYHRSSATEYQDSITGFVAVRSLQPTAVHVQRWQPDALAQEEGNGSVVTTHTHSDNF
DSATLSLEQAWHVSPAWMQDLKGEDQATASSSSQLEKLNQHFTDMHASRAKYFKAYSSVRDTQVGYWFNLRE
HPEIDQ-HEGADQEFLIIAKNFYNQNNLPKDLQQQVSQLLTQSRWDQHGYD-----DIERQGNELTLIRRQI
KTAPEYNPEQHRPIAYPQRAKVVGPEGETIHVDEWGRIKVRFLFTRSDDHGHDGGAGSNDNDTDSAWVDVLT
PWAGEGYGARFLPRIGEVVVIDFFDGNIDRPFVTGRIHEAQRSPTKFDVKGQLPDTKKLSGIRSQEINGSGF
NQLRFDDTTGQISTQLQSSHAATQLNLGNLSHPKEQATSQGRGEGFELRTDAWGAVRAGKGMLISTYAQEQA
IADHLEAAQAQSLLSQGYESMKMLSEVAAKQQTDALNVINRLPKFIQSLELKTTGQALNSTVNLFKEGINND
PIHALKDCGGFIQDIGALGGNAKGVVDEFNSFFSDAKDAVENLKAFIENVEEHGPDIVKGKLASIKDRIHKN
PFESIQEVGKVLTNVETKDFDLMSTCGTFSKGSKLEVSPSKALSSLQGFMEGYTQGLESSSDTQQQEQGKIF

RQALMLLASPNGIALTTPENIILQASQDIAESASGSINLSAQKNIIGHAQDKISLFAAQKGLRAYAAKGKLE
LQAQDDAIEAIAKKVIKLISTEDKIELTSPKEIVLTAGGSQLKINANGVFSTTGGKFESKAGQHLFTGGAKV
SYEVPELP--------KNGPYAVDFLFASLAGNGIENAKIQLYEPDKKEIIWEGKTDLRGKSNLSLQNE---
---------SKRYEALIGFDDWSSIFDDEDDYEEEHEEEFEIGEHGMQAEENNLEE----------------
------------------------------------------------------------------------
-----------------------
>AC29_18
-MLFNIFSVLEKIGLNAQKRAIHVQFSNELLNHQVFLQRIEGQHQLNGGLMAELICLSTNAQIALKQFIGVQ
VAVDQVTDSGQLFRTTGIVTEASYGQSDGALTLYKLTIEDATNLWHKRRNSRVFMNKSIVEITEVLFKEWQE
KSPLFAASLSLDLGGLS-QNYDIRPFTMQHNEADYDFLTRLWRSEGVSWLIDESELFVPHFTAPIQAQKLRL
IDDNNQYQALARRSIRYHRSSATEYQDSITGFVAVRSLQPTAVHVQRWQPDALAQEEGNGSVVTTHTHSDNF
DSATLSLEQAWHVSPAWMQDLKGEDQATASSSSQLEKLNQHFTDMHASRAKYFKAYSSVRDTQVGYWFNLRE
HPEIDQ-HEGADQEFLIIAKNFYNQNNLPKDLQQQVSQLLTQSRWDQHGYD-----DIERQGNELTLIRRQI
KTAPEYNPEQHRPIAYPQRAKVVGPEGETIHVDEWGRIKVRFLFTRSDDHGHDGGAGSNDNDTDSAWVDVLT
PWAGEGYGARFLPRIGEVVVIDFFDGNIDRPFVTGRIHEAQRSPTKFDVKGQLPDTKKLSGIRSQEINGSGF
NQLRFDDTTGQISTQLQSSHAATQLNLGNLSHPKEQATSQGRGEGFELRTDAWGAVRAGKGMLISTYAQEQA
IADHLEAAQAQSLLSQGYESMKMLSEVAAKQQTDALNVINRLPKFIQSLELKTTGQALNSTVNLFKEGINND
PIHALKDCGGFIQDIGALGGNAKGVVDEFNSFFSDAKDAVENLKAFIENVEEHGPDIVKGKLASIKDRIHKN
PFESIQEVGKVLTNVETKDFDLMSTCGTFSKGSKLEVSPSKALSSLQGFMEGYTQGLESSSDTQQQEQGKIF
RQALMLLASPNGIALTTPENIILQASQDIAESASGSINLSAQKNIIGHAQDKISLFAAQKGLRAYAAKGKLE
LQAQDDAIEAIAKKVIKLISTEDKIELTSPKEIVLTAGGSQLKINANGVFSTTGGKFESKAGQHLFTGGAKV
SYEVPELP--------KNGPYAVDFLFASLAGNGIENAKIQLYEPDKKEIIWEGKTDLRGKSNLSLQNE---
---------SKRYEALIGFDDWSSIFDDEDDYEEEHEEEFEIGEHGMQAEENNLEE----------------
------------------------------------------------------------------------
-----------------------
>AC30_18
-MLFNIFSVLEKIGLNAQKRAIHVQFSNELLNHQVFLQRIEGQHQLNGGLMAELICLSTNAQIALKQFIGVQ
VAVDQVTDSGQLFRTTGIVTEASYGQSDGALTLYKLTIEDATNLWHKRRNSRVFMNKSIVEITEVLFKEWQE
KSPLFAASLSLDLGGLS-QNYDIRPFTMQHNEADYDFLTRLWRSEGVSWLIDESELFVPHFTAPIQAQKLRL
IDDNNQYQALARRSIRYHRSSATEYQDSITGFVAVRSLQPTAVHVQRWQPDALAQEEGNGSVVTTHTHSDNF
DSATLSLEQAWHVSPAWMQDLKGEDQATASSSSQLEKLNQHFTDMHASRAKYFKAYSSVRDTQVGYWFNLRE
HPEIDQ-HEGADQEFLIIAKNFYNQNNLPKDLQQQVSQLLTQSRWDQHGYD-----DIERQGNELTLIRRQI
KTAPEYNPEQHRPIAYPQRAKVVGPEGETIHVDEWGRIKVRFLFTRSDDHGHDGGAGSNDNDTDSAWVDVLT

PWAGEGYGARFLPRIGEVVVIDFFDGNIDRPFVTGRIHEAQRSPTKFDVKGQLPDTKKLSGIRSQEINGSGF
NQLRFDDTTGQISTQLQSSHAATQLNLGNLSHPKEQATSQGRGEGFELRTDAWGAVRAGKGMLISTYAQEQA
IADHLEAAQAQSLLSQGYESMKMLSEVAAKQQTDALNVINRLPKFIQSLELKTTGQALNSTVNLFKEGINND
PIHALKDCGGFIQDIGALGGNAKGVVDEFNSFFSDAKDAVENLKAFIENVEEHGPDIVKGKLASIKDRIHKN
PFESIQEVGKVLTNVETKDFDLMSTCGTFSKGSKLEVSPSKALSSLQGFMEGYTQGLESSSDTQQQEQGKIF
RQALMLLASPNGIALTTPENIILQASQDIAESASGSINLSAQKNIIGHAQDKISLFAAQKGLRAYAAKGKLE
LQAQDDAIEAIAKKVIKLISTEDKIELTSPKEIVLTAGGSQLKINANGVFSTTGGKFESKAGQHLFTGGAKV
SYEVPELP--------KNGPYAVDFLFASLAGNGIENAKIQLYEPDKKEIIWEGKTDLRGKSNLSLQNE---
---------SKRYEALIGFDDWSSIFDDEDDYEEEHEEEFEIGEHGMQAEENNLEE----------------
------------------------------------------------------------------------
-----------------------

>AF-673_18

-MLFNIFSVLEKIGLNAQKRAIHVQFSNELLNHQVFLQRIEGQHQLNGGLMAELICLSTNAQIALKQFIGVQ
VAVDQVTDSGQLFRTTGIVTEASYGQSDGALTLYKLTIEDATNLWHKRRNSRVFMNKSIVEITEVLFKEWQE
KSPLFAASLSLDLGGLS-QNYDIRPFTMQHNEADYDFLTRLWRSEGVSWLIDESELFVPHFTAPIQAQKLRL
IDDNNQYQALARRSIRYHRSSATEYQDSITGFVAVRSLQPTAVHVQRWQPDALAQEEGNGSVVTTHTHSDNF
DSATLSLEQAWHVSPAWMQDLKGEDQATASSSSQLEKLNQHFTDMHASRAKYFKAYSSVRDTQVGYWFNLRE
HPEIDQ-HEGADQEFLIIAKNFYNQNNLPKDLQQQVSQLLTQSRWDQHGYD-----DIERQGNELTLIRRQI
KTAPEYNPEQHRPIAYPQRAKVVGPEGETIHVDEWGRIKVRFLFTRSDDHGHDGGAGSNDNDTDSAWVDVLT
PWAGEGYGARFLPRIGEVVVIDFFDGNIDRPFVTGRIHEAQRSPTKFDVKGQLPDTKKLSGIRSQEINGSGF
NQLRFDDTTGQISTQLQSSHAATQLNLGNLSHPKEQATSQGRGEGFELRTDAWGAVRAGKGMLISTYAQEQA
IADHLEAAQAQSLLSQGYESMKMLSEVAAKQQTDALNVINRLPKFIQSLELKTTGQALNSTVNLFKEGINND
PIHALKDCGGFIQDIGALGGNAKGVVDEFNSFFSDAKDAVENLKAFIENVEEHGPDIVKGKLASIKDRIHKN
PFESIQEVGKVLTNVETKDFDLMSTCGTFSKGSKLEVSPSKALSSLQGFMEGYTQGLESSSDTQQQEQGKIF
RQALMLLASPNGIALTTPENIILQASQDIAESASGSINLSAQKNIIGHAQDKISLFAAQKGLRAYAAKGKLE
LQAQDDAIEAIAKKVIKLISTEDKIELTSPKEIVLTAGGSQLKINANGVFSTTGGKFESKAGQHLFTGGAKV
SYEVPELP--------KNGPYAVDFLFASLAGNGIENAKIQLYEPDKKEIIWEGKTDLRGKSNLSLQNE---
---------SKRYEALIGFDDWSSIFDDEDDYEEEHEEEFEIGEHGMQAEENNLEE----------------
------------------------------------------------------------------------
-----------------------

>AR_0102_18

-MLFNIFSVLEKIGLNAQKRAIHVQFSNELLNHQVFLQRIEGQHQLNGGLMAELICLSTNAQIALKQFIGVQ
VAVDQVTDSGQLFRTTGIVTEASYGQSDGALTLYKLTIEDATNLWHKRRNSRVFMNKSIVEITEVLFKEWQE

KSPLFAASLSLDLGGLS-QNYDIRPFTMQHNEADYDFLTRLWRSEGVSWLIDESELFVPHFTAPIQAQKLRL
IDDNNQYQALARRSIRYHRSSATEYQDSITGFVAVRSLQPTAVHVQRWQPDALAQEEGNGSVVTTHTHSDNF
DSATLSLEQAWHVSPAWMQDLKGEDQATASSSSQLEKLNQHFTDMHASRAKYFKAYSSVRDTQVGYWFNLRE
HPEIDQ-HEGADQEFLIIAKNFYNQNNLPKDLQQQVSQLLTQSRWDQHGYD-----DIERQGNELTLIRRQI
KTAPEYNPEQHRPIAYPQRAKVVGPEGETIHVDEWGRIKVRFLFTRSDDHGHDGGAGSNDNDTDSAWVDVLT
PWAGEGYGARFLPRIGEVVVIDFFDGNIDRPFVTGRIHEAQRSPTKFDVKGQLPDTKKLSGIRSQEINGSGF
NQLRFDDTTGQISTQLQSSHAATQLNLGNLSHPKEQATSQGRGEGFELRTDAWGAVRAGKGMLISTYAQEQA
IADHLEAAQAQSLLSQGYESMKMLSEVAAKQQTDALNVINRLPKFIQSLELKTTGQALNSTVNLFKEGINND
PIHALKDCGGFIQDIGALGGNAKGVVDEFNSFFSDAKDAVENLKAFIENVEEHGPDIVKGKLASIKDRIHKN
PFESIQEVGKVLTNVETKDFDLMSTCGTFSKGSKLEVSPSKALSSLQGFMEGYTQGLESSSDTQQQEQGKIF
RQALMLLASPNGIALTTPENIILQASQDIAESASGSINLSAQKNIIGHAQDKISLFAAQKGLRAYAAKGKLE
LQAQDDAIEAIAKKVIKLISTEDKIELTSPKEIVLTAGGSQLKINANGVFSTTGGKFESKAGQHLFTGGAKV
SYEVPELP--------KNGPYAVDFLFASLAGNGIENAKIQLYEPDKKEIIWEGKTDLRGKSNLSLQNE---
---------SKRYEALIGFDDWSSIFDDEDDYEEEHEEEFEIGEHGMQAEENNLEE----------------
------------------------------------------------------------------------
-----------------------
>AYP-A2_18
-MLFNIFSVLEKIGLNAQKRAIHVQFSNELLNHQVFLQRIEGQHQLNGGLMAELICLSTNAQIALKQFIGVQ
VAVDQVTDSGQLFRTTGIVTEASYGQSDGALTLYKLTIEDATNLWHKRRNSRVFMNKSIVEITEVLFKEWQE
KSPLFAASLSLDLGGLS-QNYDIRPFTMQHNEADYDFLTRLWRSEGVSWLIDESELFVPHFTAPIQAQKLRL
IDDNNQYQALARRSIRYHRSSATEYQDSITGFVAVRSLQPTAVHVQRWQPDALAQEEGNGSVVTTHTHSDNF
DSATLSLEQAWHVSPAWMQDLKGEDQATASSSSQLEKLNQHFTDMHASRAKYFKAYSSVRDTQVGYWFNLRE
HPEIDQ-HEGADQEFLIIAKNFYNQNNLPKDLQQQVSQLLTQSRWDQHGYD-----DIERQGNELTLIRRQI
KTAPEYNPEQHRPIAYPQRAKVVGPEGETIHVDEWGRIKVRFLFTRSDDHGHDGGAGSNDNDTDSAWVDVLT
PWAGEGYGARFLPRIGEVVVIDFFDGNIDRPFVTGRIHEAQRSPTKFDVKGQLPDTKKLSGIRSQEINGSGF
NQLRFDDTTGQISTQLQSSHAATQLNLGNLSHPKEQATSQGRGEGFELRTDAWGAVRAGKGMLISTYAQEQA
IADHLEAAQAQSLLSQGYESMKMLSEVAAKQQTDALNVINRLPKFIQSLELKTTGQALNSTVNLFKEGINND
PIHALKDCGGFIQDIGALGGNAKGVVDEFNSFFSDAKDAVENLKAFIENVEEHGPDIVKGKLASIKDRIHKN
PFESIQEVGKVLTNVETKDFDLMSTCGTFSKGSKLEVSPSKALSSLQGFMEGYTQGLESSSDTQQQEQGKIF
RQALMLLASPNGIALTTPENIILQASQDIAESASGSINLSAQKNIIGHAQDKISLFAAQKGLRAYAAKGKLE
LQAQDDAIEAIAKKVIKLISTEDKIELTSPKEIVLTAGGSQLKINANGVFSTTGGKFESKAGQHLFTGGAKV
SYEVPELP--------KNGPYAVDFLFASLAGNGIENAKIQLYEPDKKEIIWEGKTDLRGKSNLSLQNE---
---------SKRYEALIGFDDWSSIFDDEDDYEEEHEEEFEIGEHGMQAEENNLEE----------------

------------------------------------------------------------------------
-----------------------
>BJAB07104_18
-MLFNIFSVLEKIGLNAQKRAIHVQFSNELLNHQVFLQRIEGQHQLNGGLMAELICLSTNAQIALKQFIGVQ
VAVDQVTDSGQLFRTTGIVTEASYGQSDGALTLYKLTIEDATNLWHKRRNSRVFMNKSIVEITEVLFKEWQE
KSPLFAASLSLDLGGLS-QNYDIRPFTMQHNEADYDFLTRLWRSEGVSWLIDESELFVPHFTAPIQAQKLRL
IDDNNQYQALARRSIRYHRSSATEYQDSITGFVAVRSLQPTAVHVQRWQPDALAQEEGNGSVVTTHTHSDNF
DSATLSLEQAWHVSPAWMQDLKGEDQATASSSSQLEKLNQHFTDMHASRAKYFKAYSSVRDTQVGYWFNLRE
HPEIDQ-HEGADQEFLIIAKNFYNQNNLPKDLQQQVSQLLTQSRWDQHGYD-----DIERQGNELTLIRRQI
KTAPEYNPEQHRPIAYPQRAKVVGPEGETIHVDEWGRIKVRFLFTRSDDHGHDGGAGSNDNDTDSAWVDVLT
PWAGEGYGARFLPRIGEVVVIDFFDGNIDRPFVTGRIHEAQRSPTKFDVKGQLPDTKKLSGIRSQEINGSGF
NQLRFDDTTGQISTQLQSSHAATQLNLGNLSHPKEQATSQGRGEGFELRTDAWGAVRAGKGMLISTYAQEQA
IADHLEAAQAQSLLSQGYESMKMLSEVAAKQQTDALNVINRLPKFIQSLELKTTGQALNSTVNLFKEGINND
PIHALKDCGGFIQDIGALGGNAKGVVDEFNSFFSDAKDAVENLKAFIENVEEHGPDIVKGKLASIKDRIHKN
PFESIQEVGKVLTNVETKDFDLMSTCGTFSKGSKLEVSPSKALSSLQGFMEGYTQGLESSSDTQQQEQGKIF
RQALMLLASPNGIALTTPENIILQASQDIAESASGSINLSAQKNIIGHAQDKISLFAAQKGLRAYAAKGKLE
LQAQDDAIEAIAKKVIKLISTEDKIELTSPKEIVLTAGGSQLKINANGVFSTTGGKFESKAGQHLFTGGAKV
SYEVPELP--------KNGPYAVDFLFASLAGNGIENAKIQLYEPDKKEIIWEGKTDLRGKSNLSLQNE---
---------SKRYEALIGFDDWSSIFDDEDDYEEEHEEEFEIGEHGMQAEENNLEE----------------
------------------------------------------------------------------------
-----------------------
>BJAB0868_18
-MLFNIFSVLEKIGLNAQKRAIHVQFSNELLNHQVFLQRIEGQHQLNGGLMAELICLSTNAQIALKQFIGVQ
VAVDQVTDSGQLFRTTGIVTEASYGQSDGALTLYKLTIEDATNLWHKRRNSRVFMNKSIVEITEVLFKEWQE
KSPLFAASLSLDLGGLS-QNYDIRPFTMQHNEADYDFLTRLWRSEGVSWLIDESELFVPHFTAPIQAQKLRL
IDDNNQYQALARRSIRYHRSSATEYQDSITGFVAVRSLQPTAVHVQRWQPDALAQEEGNGSVVTTHTHSDNF
DSATLSLEQAWHVSPAWMQDLKGEDQATASSSSQLEKLNQHFTDMHASRAKYFKAYSSVRDTQVGYWFNLRE
HPEIDQ-HEGADQEFLIIAKNFYNQNNLPKDLQQQVSQLLTQSRWDQHGYD-----DIERQGNELTLIRRQI
KTAPEYNPEQHRPIAYPQRAKVVGPEGETIHVDEWGRIKVRFLFTRSDDHGHDGGAGSNDNDTDSAWVDVLT
PWAGEGYGARFLPRIGEVVVIDFFDGNIDRPFVTGRIHEAQRSPTKFDVKGQLPDTKKLSGIRSQEINGSGF
NQLRFDDTTGQISTQLQSSHAATQLNLGNLSHPKEQATSQGRGEGFELRTDAWGAVRAGKGMLISTYAQEQA
IADHLEAAQAQSLLSQGYESMKMLSEVAAKQQTDALNVINRLPKFIQSLELKTTGQALNSTVNLFKEGINND
PIHALKDCGGFIQDIGALGGNAKGVVDEFNSFFSDAKDAVENLKAFIENVEEHGPDIVKGKLASIKDRIHKN

PFESIQEVGKVLTNVETKDFDLMSTCGTFSKGSKLEVSPSKALSSLQGFMEGYTQGLESSSDTQQQEQGKIF
RQALMLLASPNGIALTTPENIILQASQDIAESASGSINLSAQKNIIGHAQDKISLFAAQKGLRAYAAKGKLE
LQAQDDAIEAIAKKVIKLISTEDKIELTSPKEIVLTAGGSQLKINANGVFSTTGGKFESKAGQHLFTGGAKV
SYEVPELP--------KNGPYAVDFLFASLAGNGIENAKIQLYEPDKKEIIWEGKTDLRGKSNLSLQNE---
---------SKRYEALIGFDDWSSIFDDEDDYEEEHEEEFEIGEHGMQAEENNLEE----------------
------------------------------------------------------------------------
-----------------------
>CBA7_18
-MLFNIFSVLEKIGLNAQKRAIHVQFSNELLNHQVFLQRIEGQHQLNGGLMAELICLSTNAQIALKQFIGVQ
VAVDQVTDSGQLFRTTGIVTEASYGQSDGALTLYKLTIEDATNLWHKRRNSRVFMNKSIVEITEVLFKEWQE
KSPLFAASLSLDLGGLS-QNYDIRPFTMQHNEADYDFLTRLWRSEGVSWLIDESELFVPHFTAPIQAQKLRL
IDDNNQYQALARRSIRYHRSSATEYQDSITGFVAVRSLQPTAVHVQRWQPDALAQEEGNGSVVTTHTHSDNF
DSATLSLEQAWHVSPAWMQDLKGEDQATASSSSQLEKLNQHFTDMHASRAKYFKAYSSVRDTQVGYWFNLRE
HPEIDQ-HEGADQEFLIIAKNFYNQNNLPKDLQQQVSQLLTQSRWDQHGYD-----DIERQGNELTLIRRQI
KTAPEYNPEQHRPIAYPQRAKVVGPEGETIHVDEWGRIKVRFLFTRSDDHGHDGGAGSNDNDTDSAWVDVLT
PWAGEGYGARFLPRIGEVVVIDFFDGNIDRPFVTGRIHEAQRSPTKFDVKGQLPDTKKLSGIRSQEINGSGF
NQLRFDDTTGQISTQLQSSHAATQLNLGNLSHPKEQATSQGRGEGFELRTDAWGAVRAGKGMLISTYAQEQA
IADHLEAAQAQSLLSQGYESMKMLSEVAAKQQTDALNVINRLPKFIQSLELKTTGQALNSTVNLFKEGINND
PIHALKDCGGFIQDIGALGGNAKGVVDEFNSFFSDAKDAVENLKAFIENVEEHGPDIVKGKLASIKDRIHKN
PFESIQEVGKVLTNVETKDFDLMSTCGTFSKGSKLEVSPSKALSSLQGFMEGYTQGLESSSDTQQQEQGKIF
RQALMLLASPNGIALTTPENIILQASQDIAESASGSINLSAQKNIIGHAQDKISLFAAQKGLRAYAAKGKLE
LQAQDDAIEAIAKKVIKLISTEDKIELTSPKEIVLTAGGSQLKINANGVFSTTGGKFESKAGQHLFTGGAKV
SYEVPELP--------KNGPYAVDFLFASLAGNGIENAKIQLYEPDKKEIIWEGKTDLRGKSNLSLQNE---
---------SKRYEALIGFDDWSSIFDDEDDYEEEHEEEFEIGEHGMQAEENNLEE----------------
------------------------------------------------------------------------
-----------------------
>CMC-CR-MDR-Ab4_18
-MLFNIFSVLEKIGLNAQKRAIHVQFSNELLNHQVFLQRIEGQHQLNGGLMAELICLSTNAQIALKQFIGVQ
VAVDQVTDSGQLFRTTGIVTEASYGQSDGALTLYKLTIEDATNLWHKRRNSRVFMNKSIVEITEVLFKEWQE
KSPLFAASLSLDLGGLS-QNYDIRPFTMQHNEADYDFLTRLWRSEGVSWLIDESELFVPHFTAPIQAQKLRL
IDDNNQYQALARRSIRYHRSSATEYQDSITGFVAVRSLQPTAVHVQRWQPDALAQEEGNGSVVTTHTHSDNF
DSATLSLEQAWHVSPAWMQDLKGEDQATASSSSQLEKLNQHFTDMHASRAKYFKAYSSVRDTQVGYWFNLRE
HPEIDQ-HEGADQEFLIIAKNFYNQNNLPKDLQQQVSQLLTQSRWDQHGYD-----DIERQGNELTLIRRQI

KTAPEYNPEQHRPIAYPQRAKVVGPEGETIHVDEWGRIKVRFLFTRSDDHGHDGGAGSNDNDTDSAWVDVLT
PWAGEGYGARFLPRIGEVVVIDFFDGNIDRPFVTGRIHEAQRSPTKFDVKGQLPDTKKLSGIRSQEINGSGF
NQLRFDDTTGQISTQLQSSHAATQLNLGNLSHPKEQATSQGRGEGFELRTDAWGAVRAGKGMLISTYAQEQA
IADHLEAAQAQSLLSQGYESMKMLSEVAAKQQTDALNVINRLPKFIQSLELKTTGQALNSTVNLFKEGINND
PIHALKDCGGFIQDIGALGGNAKGVVDEFNSFFSDAKDAVENLKAFIENVEEHGPDIVKGKLASIKDRIHKN
PFESIQEVGKVLTNVETKDFDLMSTCGTFSKGSKLEVSPSKALSSLQGFMEGYTQGLESSSDTQQQEQGKIF
RQALMLLASPNGIALTTPENIILQASQDIAESASGSINLSAQKNIIGHAQDKISLFAAQKGLRAYAAKGKLE
LQAQDDAIEAIAKKVIKLISTEDKIELTSPKEIVLTAGGSQLKINANGVFSTTGGKFESKAGQHLFTGGAKV
SYEVPELP--------KNGPYAVDFLFASLAGNGIENAKIQLYEPDKKEIIWEGKTDLRGKSNLSLQNE---
---------SKRYEALIGFDDWSSIFDDEDDYEEEHEEEFEIGEHGMQAEENNLEE----------------
------------------------------------------------------------------------
----------------------
>CMC-CR-MDR-Ab66_18
-MLFNIFSVLEKIGLNAQKRAIHVQFSNELLNHQVFLQRIEGQHQLNGGLMAELICLSTNAQIALKQFIGVQ
VAVDQVTDSGQLFRTTGIVTEASYGQSDGALTLYKLTIEDATNLWHKRRNSRVFMNKSIVEITEVLFKEWQE
KSPLFAASLSLDLGGLS-QNYDIRPFTMQHNEADYDFLTRLWRSEGVSWLIDESELFVPHFTAPIQAQKLRL
IDDNNQYQALARRSIRYHRSSATEYQDSITGFVAVRSLQPTAVHVQRWQPDALAQEEGNGSVVTTHTHSDNF
DSATLSLEQAWHVSPAWMQDLKGEDQATASSSSQLEKLNQHFTDMHASRAKYFKAYSSVRDTQVGYWFNLRE
HPEIDQ-HEGADQEFLIIAKNFYNQNNLPKDLQQQVSQLLTQSRWDQHGYD-----DIERQGNELTLIRRQI
KTAPEYNPEQHRPIAYPQRAKVVGPEGETIHVDEWGRIKVRFLFTRSDDHGHDGGAGSNDNDTDSAWVDVLT
PWAGEGYGARFLPRIGEVVVIDFFDGNIDRPFVTGRIHEAQRSPTKFDVKGQLPDTKKLSGIRSQEINGSGF
NQLRFDDTTGQISTQLQSSHAATQLNLGNLSHPKEQATSQGRGEGFELRTDAWGAVRAGKGMLISTYAQEQA
IADHLEAAQAQSLLSQGYESMKMLSEVAAKQQTDALNVINRLPKFIQSLELKTTGQALNSTVNLFKEGINND
PIHALKDCGGFIQDIGALGGNAKGVVDEFNSFFSDAKDAVENLKAFIENVEEHGPDIVKGKLASIKDRIHKN
PFESIQEVGKVLTNVETKDFDLMSTCGTFSKGSKLEVSPSKALSSLQGFMEGYTQGLESSSDTQQQEQGKIF
RQALMLLASPNGIALTTPENIILQASQDIAESASGSINLSAQKNIIGHAQDKISLFAAQKGLRAYAAKGKLE
LQAQDDAIEAIAKKVIKLISTEDKIELTSPKEIVLTAGGSQLKINANGVFSTTGGKFESKAGQHLFTGGAKV
SYEVPELP--------KNGPYAVDFLFASLAGNGIENAKIQLYEPDKKEIIWEGKTDLRGKSNLSLQNE---
---------SKRYEALIGFDDWSSIFDDEDDYEEEHEEEFEIGEHGMQAEENNLEE----------------
------------------------------------------------------------------------
----------------------
>CMC-MDR-Ab59_18
-MLFNIFSVLEKIGLNAQKRAIHVQFSNELLNHQVFLQRIEGQHQLNGGLMAELICLSTNAQIALKQFIGVQ

VAVDQVTDSGQLFRTTGIVTEASYGQSDGALTLYKLTIEDATNLWHKRRNSRVFMNKSIVEITEVLFKEWQE
KSPLFAASLSLDLGGLS-QNYDIRPFTMQHNEADYDFLTRLWRSEGVSWLIDESELFVPHFTAPIQAQKLRL
IDDNNQYQALARRSIRYHRSSATEYQDSITGFVAVRSLQPTAVHVQRWQPDALAQEEGNGSVVTTHTHSDNF
DSATLSLEQAWHVSPAWMQDLKGEDQATASSSSQLEKLNQHFTDMHASRAKYFKAYSSVRDTQVGYWFNLRE
HPEIDQ-HEGADQEFLIIAKNFYNQNNLPKDLQQQVSQLLTQSRWDQHGYD-----DIERQGNELTLIRRQI
KTAPEYNPEQHRPIAYPQRAKVVGPEGETIHVDEWGRIKVRFLFTRSDDHGHDGGAGSNDNDTDSAWVDVLT
PWAGEGYGARFLPRIGEVVVIDFFDGNIDRPFVTGRIHEAQRSPTKFDVKGQLPDTKKLSGIRSQEINGSGF
NQLRFDDTTGQISTQLQSSHAATQLNLGNLSHPKEQATSQGRGEGFELRTDAWGAVRAGKGMLISTYAQEQA
IADHLEAAQAQSLLSQGYESMKMLSEVAAKQQTDALNVINRLPKFIQSLELKTTGQALNSTVNLFKEGINND
PIHALKDCGGFIQDIGALGGNAKGVVDEFNSFFSDAKDAVENLKAFIENVEEHGPDIVKGKLASIKDRIHKN
PFESIQEVGKVLTNVETKDFDLMSTCGTFSKGSKLEVSPSKALSSLQGFMEGYTQGLESSSDTQQQEQGKIF
RQALMLLASPNGIALTTPENIILQASQDIAESASGSINLSAQKNIIGHAQDKISLFAAQKGLRAYAAKGKLE
LQAQDDAIEAIAKKVIKLISTEDKIELTSPKEIVLTAGGSQLKINANGVFSTTGGKFESKAGQHLFTGGAKV
SYEVPELP--------KNGPYAVDFLFASLAGNGIENAKIQLYEPDKKEIIWEGKTDLRGKSNLSLQNE---
---------SKRYEALIGFDDWSSIFDDEDDYEEEHEEEFEIGEHGMQAEENNLEE----------------
------------------------------------------------------------------------
-----------------------

>HRAB-85_18
-MLFNIFSVLEKIGLNAQKRAIHVQFSNELLNHQVFLQRIEGQHQLNGGLMAELICLSTNAQIALKQFIGVQ
VAVDQVTDSGQLFRTTGIVTEASYGQSDGALTLYKLTIEDATNLWHKRRNSRVFMNKSIVEITEVLFKEWQE
KSPLFAASLSLDLGGLS-QNYDIRPFTMQHNEADYDFLTRLWRSEGVSWLIDESELFVPHFTAPIQAQKLRL
IDDNNQYQALARRSIRYHRSSATEYQDSITGFVAVRSLQPTAVHVQRWQPDALAQEEGNGSVVTTHTHSDNF
DSATLSLEQAWHVSPAWMQDLKGEDQATASSSSQLEKLNQHFTDMHASRAKYFKAYSSVRDTQVGYWFNLRE
HPEIDQ-HEGADQEFLIIAKNFYNQNNLPKDLQQQVSQLLTQSRWDQHGYD-----DIERQGNELTLIRRQI
KTAPEYNPEQHRPIAYPQRAKVVGPEGETIHVDEWGRIKVRFLFTRSDDHGHDGGAGSNDNDTDSAWVDVLT
PWAGEGYGARFLPRIGEVVVIDFFDGNIDRPFVTGRIHEAQRSPTKFDVKGQLPDTKKLSGIRSQEINGSGF
NQLRFDDTTGQISTQLQSSHAATQLNLGNLSHPKEQATSQGRGEGFELRTDAWGAVRAGKGMLISTYAQEQA
IADHLEAAQAQSLLSQGYESMKMLSEVAAKQQTDALNVINRLPKFIQSLELKTTGQALNSTVNLFKEGINND
PIHALKDCGGFIQDIGALGGNAKGVVDEFNSFFSDAKDAVENLKAFIENVEEHGPDIVKGKLASIKDRIHKN
PFESIQEVGKVLTNVETKDFDLMSTCGTFSKGSKLEVSPSKALSSLQGFMEGYTQGLESSSDTQQQEQGKIF
RQALMLLASPNGIALTTPENIILQASQDIAESASGSINLSAQKNIIGHAQDKISLFAAQKGLRAYAAKGKLE
LQAQDDAIEAIAKKVIKLISTEDKIELTSPKEIVLTAGGSQLKINANGVFSTTGGKFESKAGQHLFTGGAKV
SYEVPELP--------KNGPYAVDFLFASLAGNGIENAKIQLYEPDKKEIIWEGKTDLRGKSNLSLQNE---

---------SKRYEALIGFDDWSSIFDDEDDYEEEHEEEFEIGEHGMQAEENNLEE----------------
------------------------------------------------------------------------
-----------------------
>JBA13_18
-MLFNIFSVLEKIGLNAQKRAIHVQFSNELLNHQVFLQRIEGQHQLNGGLMAELICLSTNAQIALKQFIGVQ
VAVDQVTDSGQLFRTTGIVTEASYGQSDGALTLYKLTIEDATNLWHKRRNSRVFMNKSIVEITEVLFKEWQE
KSPLFAASLSLDLGGLS-QNYDIRPFTMQHNEADYDFLTRLWRSEGVSWLIDESELFVPHFTAPIQAQKLRL
IDDNNQYQALARRSIRYHRSSATEYQDSITGFVAVRSLQPTAVHVQRWQPDALAQEEGNGSVVTTHTHSDNF
DSATLSLEQAWHVSPAWMQDLKGEDQATASSSSQLEKLNQHFTDMHASRAKYFKAYSSVRDTQVGYWFNLRE
HPEIDQ-HEGADQEFLIIAKNFYNQNNLPKDLQQQVSQLLTQSRWDQHGYD-----DIERQGNELTLIRRQI
KTAPEYNPEQHRPIAYPQRAKVVGPEGETIHVDEWGRIKVRFLFTRSDDHGHDGGAGSNDNDTDSAWVDVLT
PWAGEGYGARFLPRIGEVVVIDFFDGNIDRPFVTGRIHEAQRSPTKFDVKGQLPDTKKLSGIRSQEINGSGF
NQLRFDDTTGQISTQLQSSHAATQLNLGNLSHPKEQATSQGRGEGFELRTDAWGAVRAGKGMLISTYAQEQA
IADHLEAAQAQSLLSQGYESMKMLSEVAAKQQTDALNVINRLPKFIQSLELKTTGQALNSTVNLFKEGINND
PIHALKDCGGFIQDIGALGGNAKGVVDEFNSFFSDAKDAVENLKAFIENVEEHGPDIVKGKLASIKDRIHKN
PFESIQEVGKVLTNVETKDFDLMSTCGTFSKGSKLEVSPSKALSSLQGFMEGYTQGLESSSDTQQQEQGKIF
RQALMLLASPNGIALTTPENIILQASQDIAESASGSINLSAQKNIIGHAQDKISLFAAQKGLRAYAAKGKLE
LQAQDDAIEAIAKKVIKLISTEDKIELTSPKEIVLTAGGSQLKINANGVFSTTGGKFESKAGQHLFTGGAKV
SYEVPELP--------KNGPYAVDFLFASLAGNGIENAKIQLYEPDKKEIIWEGKTDLRGKSNLSLQNE---
---------SKRYEALIGFDDWSSIFDDEDDYEEEHEEEFEIGEHGMQAEENNLEE----------------
------------------------------------------------------------------------
-----------------------
>KAB02_18
-MLFNIFSVLEKIGLNAQKRAIHVQFSNELLNHQVFLQRIEGQHQLNGGLMAELICLSTNAQIALKQFIGVQ
VAVDQVTDSGQLFRTTGIVTEASYGQSDGALTLYKLTIEDATNLWHKRRNSRVFMNKSIVEITEVLFKEWQE
KSPLFAASLSLDLGGLS-QNYDIRPFTMQHNEADYDFLTRLWRSEGVSWLIDESELFVPHFTAPIQAQKLRL
IDDNNQYQALARRSIRYHRSSATEYQDSITGFVAVRSLQPTAVHVQRWQPDALAQEEGNGSVVTTHTHSDNF
DSATLSLEQAWHVSPAWMQDLKGEDQATASSSSQLEKLNQHFTDMHASRAKYFKAYSSVRDTQVGYWFNLRE
HPEIDQ-HEGADQEFLIIAKNFYNQNNLPKDLQQQVSQLLTQSRWDQHGYD-----DIERQGNELTLIRRQI
KTAPEYNPEQHRPIAYPQRAKVVGPEGETIHVDEWGRIKVRFLFTRSDDHGHDGGAGSNDNDTDSAWVDVLT
PWAGEGYGARFLPRIGEVVVIDFFDGNIDRPFVTGRIHEAQRSPTKFDVKGQLPDTKKLSGIRSQEINGSGF
NQLRFDDTTGQISTQLQSSHAATQLNLGNLSHPKEQATSQGRGEGFELRTDAWGAVRAGKGMLISTYAQEQA
IADHLEAAQAQSLLSQGYESMKMLSEVAAKQQTDALNVINRLPKFIQSLELKTTGQALNSTVNLFKEGINND

PIHALKDCGGFIQDIGALGGNAKGVVDEFNSFFSDAKDAVENLKAFIENVEEHGPDIVKGKLASIKDRIHKN
PFESIQEVGKVLTNVETKDFDLMSTCGTFSKGSKLEVSPSKALSSLQGFMEGYTQGLESSSDTQQQEQGKIF
RQALMLLASPNGIALTTPENIILQASQDIAESASGSINLSAQKNIIGHAQDKISLFAAQKGLRAYAAKGKLE
LQAQDDAIEAIAKKVIKLISTEDKIELTSPKEIVLTAGGSQLKINANGVFSTTGGKFESKAGQHLFTGGAKV
SYEVPELP--------KNGPYAVDFLFASLAGNGIENAKIQLYEPDKKEIIWEGKTDLRGKSNLSLQNE---
---------SKRYEALIGFDDWSSIFDDEDDYEEEHEEEFEIGEHGMQAEENNLEE----------------
------------------------------------------------------------------------
-----------------------
>KAB04_18
-MLFNIFSVLEKIGLNAQKRAIHVQFSNELLNHQVFLQRIEGQHQLNGGLMAELICLSTNAQIALKQFIGVQ
VAVDQVTDSGQLFRTTGIVTEASYGQSDGALTLYKLTIEDATNLWHKRRNSRVFMNKSIVEITEVLFKEWQE
KSPLFAASLSLDLGGLS-QNYDIRPFTMQHNEADYDFLTRLWRSEGVSWLIDESELFVPHFTAPIQAQKLRL
IDDNNQYQALARRSIRYHRSSATEYQDSITGFVAVRSLQPTAVHVQRWQPDALAQEEGNGSVVTTHTHSDNF
DSATLSLEQAWHVSPAWMQDLKGEDQATASSSSQLEKLNQHFTDMHASRAKYFKAYSSVRDTQVGYWFNLRE
HPEIDQ-HEGADQEFLIIAKNFYNQNNLPKDLQQQVSQLLTQSRWDQHGYD-----DIERQGNELTLIRRQI
KTAPEYNPEQHRPIAYPQRAKVVGPEGETIHVDEWGRIKVRFLFTRSDDHGHDGGAGSNDNDTDSAWVDVLT
PWAGEGYGARFLPRIGEVVVIDFFDGNIDRPFVTGRIHEAQRSPTKFDVKGQLPDTKKLSGIRSQEINGSGF
NQLRFDDTTGQISTQLQSSHAATQLNLGNLSHPKEQATSQGRGEGFELRTDAWGAVRAGKGMLISTYAQEQA
IADHLEAAQAQSLLSQGYESMKMLSEVAAKQQTDALNVINRLPKFIQSLELKTTGQALNSTVNLFKEGINND
PIHALKDCGGFIQDIGALGGNAKGVVDEFNSFFSDAKDAVENLKAFIENVEEHGPDIVKGKLASIKDRIHKN
PFESIQEVGKVLTNVETKDFDLMSTCGTFSKGSKLEVSPSKALSSLQGFMEGYTQGLESSSDTQQQEQGKIF
RQALMLLASPNGIALTTPENIILQASQDIAESASGSINLSAQKNIIGHAQDKISLFAAQKGLRAYAAKGKLE
LQAQDDAIEAIAKKVIKLISTEDKIELTSPKEIVLTAGGSQLKINANGVFSTTGGKFESKAGQHLFTGGAKV
SYEVPELP--------KNGPYAVDFLFASLAGNGIENAKIQLYEPDKKEIIWEGKTDLRGKSNLSLQNE---
---------SKRYEALIGFDDWSSIFDDEDDYEEEHEEEFEIGEHGMQAEENNLEE----------------
------------------------------------------------------------------------
-----------------------
>KAB06_18
-MLFNIFSVLEKIGLNAQKRAIHVQFSNELLNHQVFLQRIEGQHQLNGGLMAELICLSTNAQIALKQFIGVQ
VAVDQVTDSGQLFRTTGIVTEASYGQSDGALTLYKLTIEDATNLWHKRRNSRVFMNKSIVEITEVLFKEWQE
KSPLFAASLSLDLGGLS-QNYDIRPFTMQHNEADYDFLTRLWRSEGVSWLIDESELFVPHFTAPIQAQKLRL
IDDNNQYQALARRSIRYHRSSATEYQDSITGFVAVRSLQPTAVHVQRWQPDALAQEEGNGSVVTTHTHSDNF
DSATLSLEQAWHVSPAWMQDLKGEDQATASSSSQLEKLNQHFTDMHASRAKYFKAYSSVRDTQVGYWFNLRE

HPEIDQ-HEGADQEFLIIAKNFYNQNNLPKDLQQQVSQLLTQSRWDQHGYD-----DIERQGNELTLIRRQI
KTAPEYNPEQHRPIAYPQRAKVVGPEGETIHVDEWGRIKVRFLFTRSDDHGHDGGAGSNDNDTDSAWVDVLT
PWAGEGYGARFLPRIGEVVVIDFFDGNIDRPFVTGRIHEAQRSPTKFDVKGQLPDTKKLSGIRSQEINGSGF
NQLRFDDTTGQISTQLQSSHAATQLNLGNLSHPKEQATSQGRGEGFELRTDAWGAVRAGKGMLISTYAQEQA
IADHLEAAQAQSLLSQGYESMKMLSEVAAKQQTDALNVINRLPKFIQSLELKTTGQALNSTVNLFKEGINND
PIHALKDCGGFIQDIGALGGNAKGVVDEFNSFFSDAKDAVENLKAFIENVEEHGPDIVKGKLASIKDRIHKN
PFESIQEVGKVLTNVETKDFDLMSTCGTFSKGSKLEVSPSKALSSLQGFMEGYTQGLESSSDTQQQEQGKIF
RQALMLLASPNGIALTTPENIILQASQDIAESASGSINLSAQKNIIGHAQDKISLFAAQKGLRAYAAKGKLE
LQAQDDAIEAIAKKVIKLISTEDKIELTSPKEIVLTAGGSQLKINANGVFSTTGGKFESKAGQHLFTGGAKV
SYEVPELP--------KNGPYAVDFLFASLAGNGIENAKIQLYEPDKKEIIWEGKTDLRGKSNLSLQNE---
---------SKRYEALIGFDDWSSIFDDEDDYEEEHEEEFEIGEHGMQAEENNLEE----------------
------------------------------------------------------------------------
-----------------------
>KAB07_18
-MLFNIFSVLEKIGLNAQKRAIHVQFSNELLNHQVFLQRIEGQHQLNGGLMAELICLSTNAQIALKQFIGVQ
VAVDQVTDSGQLFRTTGIVTEASYGQSDGALTLYKLTIEDATNLWHKRRNSRVFMNKSIVEITEVLFKEWQE
KSPLFAASLSLDLGGLS-QNYDIRPFTMQHNEADYDFLTRLWRSEGVSWLIDESELFVPHFTAPIQAQKLRL
IDDNNQYQALARRSIRYHRSSATEYQDSITGFVAVRSLQPTAVHVQRWQPDALAQEEGNGSVVTTHTHSDNF
DSATLSLEQAWHVSPAWMQDLKGEDQATASSSSQLEKLNQHFTDMHASRAKYFKAYSSVRDTQVGYWFNLRE
HPEIDQ-HEGADQEFLIIAKNFYNQNNLPKDLQQQVSQLLTQSRWDQHGYD-----DIERQGNELTLIRRQI
KTAPEYNPEQHRPIAYPQRAKVVGPEGETIHVDEWGRIKVRFLFTRSDDHGHDGGAGSNDNDTDSAWVDVLT
PWAGEGYGARFLPRIGEVVVIDFFDGNIDRPFVTGRIHEAQRSPTKFDVKGQLPDTKKLSGIRSQEINGSGF
NQLRFDDTTGQISTQLQSSHAATQLNLGNLSHPKEQATSQGRGEGFELRTDAWGAVRAGKGMLISTYAQEQA
IADHLEAAQAQSLLSQGYESMKMLSEVAAKQQTDALNVINRLPKFIQSLELKTTGQALNSTVNLFKEGINND
PIHALKDCGGFIQDIGALGGNAKGVVDEFNSFFSDAKDAVENLKAFIENVEEHGPDIVKGKLASIKDRIHKN
PFESIQEVGKVLTNVETKDFDLMSTCGTFSKGSKLEVSPSKALSSLQGFMEGYTQGLESSSDTQQQEQGKIF
RQALMLLASPNGIALTTPENIILQASQDIAESASGSINLSAQKNIIGHAQDKISLFAAQKGLRAYAAKGKLE
LQAQDDAIEAIAKKVIKLISTEDKIELTSPKEIVLTAGGSQLKINANGVFSTTGGKFESKAGQHLFTGGAKV
SYEVPELP--------KNGPYAVDFLFASLAGNGIENAKIQLYEPDKKEIIWEGKTDLRGKSNLSLQNE---
---------SKRYEALIGFDDWSSIFDDEDDYEEEHEEEFEIGEHGMQAEENNLEE----------------
------------------------------------------------------------------------
-----------------------
>KAB08_18

-MLFNIFSVLEKIGLNAQKRAIHVQFSNELLNHQVFLQRIEGQHQLNGGLMAELICLSTNAQIALKQFIGVQ
VAVDQVTDSGQLFRTTGIVTEASYGQSDGALTLYKLTIEDATNLWHKRRNSRVFMNKSIVEITEVLFKEWQE
KSPLFAASLSLDLGGLS-QNYDIRPFTMQHNEADYDFLTRLWRSEGVSWLIDESELFVPHFTAPIQAQKLRL
IDDNNQYQALARRSIRYHRSSATEYQDSITGFVAVRSLQPTAVHVQRWQPDALAQEEGNGSVVTTHTHSDNF
DSATLSLEQAWHVSPAWMQDLKGEDQATASSSSQLEKLNQHFTDMHASRAKYFKAYSSVRDTQVGYWFNLRE
HPEIDQ-HEGADQEFLIIAKNFYNQNNLPKDLQQQVSQLLTQSRWDQHGYD-----DIERQGNELTLIRRQI
KTAPEYNPEQHRPIAYPQRAKVVGPEGETIHVDEWGRIKVRFLFTRSDDHGHDGGAGSNDNDTDSAWVDVLT
PWAGEGYGARFLPRIGEVVVIDFFDGNIDRPFVTGRIHEAQRSPTKFDVKGQLPDTKKLSGIRSQEINGSGF
NQLRFDDTTGQISTQLQSSHAATQLNLGNLSHPKEQATSQGRGEGFELRTDAWGAVRAGKGMLISTYAQEQA
IADHLEAAQAQSLLSQGYESMKMLSEVAAKQQTDALNVINRLPKFIQSLELKTTGQALNSTVNLFKEGINND
PIHALKDCGGFIQDIGALGGNAKGVVDEFNSFFSDAKDAVENLKAFIENVEEHGPDIVKGKLASIKDRIHKN
PFESIQEVGKVLTNVETKDFDLMSTCGTFSKGSKLEVSPSKALSSLQGFMEGYTQGLESSSDTQQQEQGKIF
RQALMLLASPNGIALTTPENIILQASQDIAESASGSINLSAQKNIIGHAQDKISLFAAQKGLRAYAAKGKLE
LQAQDDAIEAIAKKVIKLISTEDKIELTSPKEIVLTAGGSQLKINANGVFSTTGGKFESKAGQHLFTGGAKV
SYEVPELP--------KNGPYAVDFLFASLAGNGIENAKIQLYEPDKKEIIWEGKTDLRGKSNLSLQNE---
---------SKRYEALIGFDDWSSIFDDEDDYEEEHEEEFEIGEHGMQAEENNLEE----------------
------------------------------------------------------------------------
----------------------
>KBN10P02143_18
-MLFNIFSVLEKIGLNAQKRAIHVQFSNELLNHQVFLQRIEGQHQLNGGLMAELICLSTNAQIALKQFIGVQ
VAVDQVTDSGQLFRTTGIVTEASYGQSDGALTLYKLTIEDATNLWHKRRNSRVFMNKSIVEITEVLFKEWQE
KSPLFAASLSLDLGGLS-QNYDIRPFTMQHNEADYDFLTRLWRSEGVSWLIDESELFVPHFTAPIQAQKLRL
IDDNNQYQALARRSIRYHRSSATEYQDSITGFVAVRSLQPTAVHVQRWQPDALAQEEGNGSVVTTHTHSDNF
DSATLSLEQAWHVSPAWMQDLKGEDQATASSSSQLEKLNQHFTDMHASRAKYFKAYSSVRDTQVGYWFNLRE
HPEIDQ-HEGADQEFLIIAKNFYNQNNLPKDLQQQVSQLLTQSRWDQHGYD-----DIERQGNELTLIRRQI
KTAPEYNPEQHRPIAYPQRAKVVGPEGETIHVDEWGRIKVRFLFTRSDDHGHDGGAGSNDNDTDSAWVDVLT
PWAGEGYGARFLPRIGEVVVIDFFDGNIDRPFVTGRIHEAQRSPTKFDVKGQLPDTKKLSGIRSQEINGSGF
NQLRFDDTTGQISTQLQSSHAATQLNLGNLSHPKEQATSQGRGEGFELRTDAWGAVRAGKGMLISTYAQEQA
IADHLEAAQAQSLLSQGYESMKMLSEVAAKQQTDALNVINRLPKFIQSLELKTTGQALNSTVNLFKEGINND
PIHALKDCGGFIQDIGALGGNAKGVVDEFNSFFSDAKDAVENLKAFIENVEEHGPDIVKGKLASIKDRIHKN
PFESIQEVGKVLTNVETKDFDLMSTCGTFSKGSKLEVSPSKALSSLQGFMEGYTQGLESSSDTQQQEQGKIF
RQALMLLASPNGIALTTPENIILQASQDIAESASGSINLSAQKNIIGHAQDKISLFAAQKGLRAYAAKGKLE
LQAQDDAIEAIAKKVIKLISTEDKIELTSPKEIVLTAGGSQLKINANGVFSTTGGKFESKAGQHLFTGGAKV

SYEVPELP--------KNGPYAVDFLFASLAGNGIENAKIQLYEPDKKEIIWEGKTDLRGKSNLSLQNE---
---------SKRYEALIGFDDWSSIFDDEDDYEEEHEEEFEIGEHGMQAEENNLEE----------------
------------------------------------------------------------------------
-----------------------
>MDR-TJ_18
-MLFNIFSVLEKIGLNAQKRAIHVQFSNELLNHQVFLQRIEGQHQLNGGLMAELICLSTNAQIALKQFIGVQ
VAVDQVTDSGQLFRTTGIVTEASYGQSDGALTLYKLTIEDATNLWHKRRNSRVFMNKSIVEITEVLFKEWQE
KSPLFAASLSLDLGGLS-QNYDIRPFTMQHNEADYDFLTRLWRSEGVSWLIDESELFVPHFTAPIQAQKLRL
IDDNNQYQALARRSIRYHRSSATEYQDSITGFVAVRSLQPTAVHVQRWQPDALAQEEGNGSVVTTHTHSDNF
DSATLSLEQAWHVSPAWMQDLKGEDQATASSSSQLEKLNQHFTDMHASRAKYFKAYSSVRDTQVGYWFNLRE
HPEIDQ-HEGADQEFLIIAKNFYNQNNLPKDLQQQVSQLLTQSRWDQHGYD-----DIERQGNELTLIRRQI
KTAPEYNPEQHRPIAYPQRAKVVGPEGETIHVDEWGRIKVRFLFTRSDDHGHDGGAGSNDNDTDSAWVDVLT
PWAGEGYGARFLPRIGEVVVIDFFDGNIDRPFVTGRIHEAQRSPTKFDVKGQLPDTKKLSGIRSQEINGSGF
NQLRFDDTTGQISTQLQSSHAATQLNLGNLSHPKEQATSQGRGEGFELRTDAWGAVRAGKGMLISTYAQEQA
IADHLEAAQAQSLLSQGYESMKMLSEVAAKQQTDALNVINRLPKFIQSLELKTTGQALNSTVNLFKEGINND
PIHALKDCGGFIQDIGALGGNAKGVVDEFNSFFSDAKDAVENLKAFIENVEEHGPDIVKGKLASIKDRIHKN
PFESIQEVGKVLTNVETKDFDLMSTCGTFSKGSKLEVSPSKALSSLQGFMEGYTQGLESSSDTQQQEQGKIF
RQALMLLASPNGIALTTPENIILQASQDIAESASGSINLSAQKNIIGHAQDKISLFAAQKGLRAYAAKGKLE
LQAQDDAIEAIAKKVIKLISTEDKIELTSPKEIVLTAGGSQLKINANGVFSTTGGKFESKAGQHLFTGGAKV
SYEVPELP--------KNGPYAVDFLFASLAGNGIENAKIQLYEPDKKEIIWEGKTDLRGKSNLSLQNE---
---------SKRYEALIGFDDWSSIFDDEDDYEEEHEEEFEIGEHGMQAEENNLEE----------------
------------------------------------------------------------------------
-----------------------
>MDR-ZJ06_18
-MLFNIFSVLEKIGLNAQKRAIHVQFSNELLNHQVFLQRIEGQHQLNGGLMAELICLSTNAQIALKQFIGVQ
VAVDQVTDSGQLFRTTGIVTEASYGQSDGALTLYKLTIEDATNLWHKRRNSRVFMNKSIVEITEVLFKEWQE
KSPLFAASLSLDLGGLS-QNYDIRPFTMQHNEADYDFLTRLWRSEGVSWLIDESELFVPHFTAPIQAQKLRL
IDDNNQYQALARRSIRYHRSSATEYQDSITGFVAVRSLQPTAVHVQRWQPDALAQEEGNGSVVTTHTHSDNF
DSATLSLEQAWHVSPAWMQDLKGEDQATASSSSQLEKLNQHFTDMHASRAKYFKAYSSVRDTQVGYWFNLRE
HPEIDQ-HEGADQEFLIIAKNFYNQNNLPKDLQQQVSQLLTQSRWDQHGYD-----DIERQGNELTLIRRQI
KTAPEYNPEQHRPIAYPQRAKVVGPEGETIHVDEWGRIKVRFLFTRSDDHGHDGGAGSNDNDTDSAWVDVLT
PWAGEGYGARFLPRIGEVVVIDFFDGNIDRPFVTGRIHEAQRSPTKFDVKGQLPDTKKLSGIRSQEINGSGF
NQLRFDDTTGQISTQLQSSHAATQLNLGNLSHPKEQATSQGRGEGFELRTDAWGAVRAGKGMLISTYAQEQA

IADHLEAAQAQSLLSQGYESMKMLSEVAAKQQTDALNVINRLPKFIQSLELKTTGQALNSTVNLFKEGINND
PIHALKDCGGFIQDIGALGGNAKGVVDEFNSFFSDAKDAVENLKAFIENVEEHGPDIVKGKLASIKDRIHKN
PFESIQEVGKVLTNVETKDFDLMSTCGTFSKGSKLEVSPSKALSSLQGFMEGYTQGLESSSDTQQQEQGKIF
RQALMLLASPNGIALTTPENIILQASQDIAESASGSINLSAQKNIIGHAQDKISLFAAQKGLRAYAAKGKLE
LQAQDDAIEAIAKKVIKLISTEDKIELTSPKEIVLTAGGSQLKINANGVFSTTGGKFESKAGQHLFTGGAKV
SYEVPELP--------KNGPYAVDFLFASLAGNGIENAKIQLYEPDKKEIIWEGKTDLRGKSNLSLQNE---
---------SKRYEALIGFDDWSSIFDDEDDYEEEHEEEFEIGEHGMQAEENNLEE----------------
------------------------------------------------------------------------
-----------------------
>SAA14_18
-MLFNIFSVLEKIGLNAQKRAIHVQFSNELLNHQVFLQRIEGQHQLNGGLMAELICLSTNAQIALKQFIGVQ
VAVDQVTDSGQLFRTTGIVTEASYGQSDGALTLYKLTIEDATNLWHKRRNSRVFMNKSIVEITEVLFKEWQE
KSPLFAASLSLDLGGLS-QNYDIRPFTMQHNEADYDFLTRLWRSEGVSWLIDESELFVPHFTAPIQAQKLRL
IDDNNQYQALARRSIRYHRSSATEYQDSITGFVAVRSLQPTAVHVQRWQPDALAQEEGNGSVVTTHTHSDNF
DSATLSLEQAWHVSPAWMQDLKGEDQATASSSSQLEKLNQHFTDMHASRAKYFKAYSSVRDTQVGYWFNLRE
HPEIDQ-HEGADQEFLIIAKNFYNQNNLPKDLQQQVSQLLTQSRWDQHGYD-----DIERQGNELTLIRRQI
KTAPEYNPEQHRPIAYPQRAKVVGPEGETIHVDEWGRIKVRFLFTRSDDHGHDGGAGSNDNDTDSAWVDVLT
PWAGEGYGARFLPRIGEVVVIDFFDGNIDRPFVTGRIHEAQRSPTKFDVKGQLPDTKKLSGIRSQEINGSGF
NQLRFDDTTGQISTQLQSSHAATQLNLGNLSHPKEQATSQGRGEGFELRTDAWGAVRAGKGMLISTYAQEQA
IADHLEAAQAQSLLSQGYESMKMLSEVAAKQQTDALNVINRLPKFIQSLELKTTGQALNSTVNLFKEGINND
PIHALKDCGGFIQDIGALGGNAKGVVDEFNSFFSDAKDAVENLKAFIENVEEHGPDIVKGKLASIKDRIHKN
PFESIQEVGKVLTNVETKDFDLMSTCGTFSKGSKLEVSPSKALSSLQGFMEGYTQGLESSSDTQQQEQGKIF
RQALMLLASPNGIALTTPENIILQASQDIAESASGSINLSAQKNIIGHAQDKISLFAAQKGLRAYAAKGKLE
LQAQDDAIEAIAKKVIKLISTEDKIELTSPKEIVLTAGGSQLKINANGVFSTTGGKFESKAGQHLFTGGAKV
SYEVPELP--------KNGPYAVDFLFASLAGNGIENAKIQLYEPDKKEIIWEGKTDLRGKSNLSLQNE---
---------SKRYEALIGFDDWSSIFDDEDDYEEEHEEEFEIGEHGMQAEENNLEE----------------
------------------------------------------------------------------------
-----------------------
>SMC_Paed_Ab_BL01_18
-MLFNIFSVLEKIGLNAQKRAIHVQFSNELLNHQVFLQRIEGQHQLNGGLMAELICLSTNAQIALKQFIGVQ
VAVDQVTDSGQLFRTTGIVTEASYGQSDGALTLYKLTIEDATNLWHKRRNSRVFMNKSIVEITEVLFKEWQE
KSPLFAASLSLDLGGLS-QNYDIRPFTMQHNEADYDFLTRLWRSEGVSWLIDESELFVPHFTAPIQAQKLRL
IDDNNQYQALARRSIRYHRSSATEYQDSITGFVAVRSLQPTAVHVQRWQPDALAQEEGNGSVVTTHTHSDNF

DSATLSLEQAWHVSPAWMQDLKGEDQATASSSSQLEKLNQHFTDMHASRAKYFKAYSSVRDTQVGYWFNLRE
HPEIDQ-HEGADQEFLIIAKNFYNQNNLPKDLQQQVSQLLTQSRWDQHGYD-----DIERQGNELTLIRRQI
KTAPEYNPEQHRPIAYPQRAKVVGPEGETIHVDEWGRIKVRFLFTRSDDHGHDGGAGSNDNDTDSAWVDVLT
PWAGEGYGARFLPRIGEVVVIDFFDGNIDRPFVTGRIHEAQRSPTKFDVKGQLPDTKKLSGIRSQEINGSGF
NQLRFDDTTGQISTQLQSSHAATQLNLGNLSHPKEQATSQGRGEGFELRTDAWGAVRAGKGMLISTYAQEQA
IADHLEAAQAQSLLSQGYESMKMLSEVAAKQQTDALNVINRLPKFIQSLELKTTGQALNSTVNLFKEGINND
PIHALKDCGGFIQDIGALGGNAKGVVDEFNSFFSDAKDAVENLKAFIENVEEHGPDIVKGKLASIKDRIHKN
PFESIQEVGKVLTNVETKDFDLMSTCGTFSKGSKLEVSPSKALSSLQGFMEGYTQGLESSSDTQQQEQGKIF
RQALMLLASPNGIALTTPENIILQASQDIAESASGSINLSAQKNIIGHAQDKISLFAAQKGLRAYAAKGKLE
LQAQDDAIEAIAKKVIKLISTEDKIELTSPKEIVLTAGGSQLKINANGVFSTTGGKFESKAGQHLFTGGAKV
SYEVPELP--------KNGPYAVDFLFASLAGNGIENAKIQLYEPDKKEIIWEGKTDLRGKSNLSLQNE---
---------SKRYEALIGFDDWSSIFDDEDDYEEEHEEEFEIGEHGMQAEENNLEE----------------
-----------------------------------------------------------------------
-----------------------

>SSA12_18
-MLFNIFSVLEKIGLNAQKRAIHVQFSNELLNHQVFLQRIEGQHQLNGGLMAELICLSTNAQIALKQFIGVQ
VAVDQVTDSGQLFRTTGIVTEASYGQSDGALTLYKLTIEDATNLWHKRRNSRVFMNKSIVEITEVLFKEWQE
KSPLFAASLSLDLGGLS-QNYDIRPFTMQHNEADYDFLTRLWRSEGVSWLIDESELFVPHFTAPIQAQKLRL
IDDNNQYQALARRSIRYHRSSATEYQDSITGFVAVRSLQPTAVHVQRWQPDALAQEEGNGSVVTTHTHSDNF
DSATLSLEQAWHVSPAWMQDLKGEDQATASSSSQLEKLNQHFTDMHASRAKYFKAYSSVRDTQVGYWFNLRE
HPEIDQ-HEGADQEFLIIAKNFYNQNNLPKDLQQQVSQLLTQSRWDQHGYD-----DIERQGNELTLIRRQI
KTAPEYNPEQHRPIAYPQRAKVVGPEGETIHVDEWGRIKVRFLFTRSDDHGHDGGAGSNDNDTDSAWVDVLT
PWAGEGYGARFLPRIGEVVVIDFFDGNIDRPFVTGRIHEAQRSPTKFDVKGQLPDTKKLSGIRSQEINGSGF
NQLRFDDTTGQISTQLQSSHAATQLNLGNLSHPKEQATSQGRGEGFELRTDAWGAVRAGKGMLISTYAQEQA
IADHLEAAQAQSLLSQGYESMKMLSEVAAKQQTDALNVINRLPKFIQSLELKTTGQALNSTVNLFKEGINND
PIHALKDCGGFIQDIGALGGNAKGVVDEFNSFFSDAKDAVENLKAFIENVEEHGPDIVKGKLASIKDRIHKN
PFESIQEVGKVLTNVETKDFDLMSTCGTFSKGSKLEVSPSKALSSLQGFMEGYTQGLESSSDTQQQEQGKIF
RQALMLLASPNGIALTTPENIILQASQDIAESASGSINLSAQKNIIGHAQDKISLFAAQKGLRAYAAKGKLE
LQAQDDAIEAIAKKVIKLISTEDKIELTSPKEIVLTAGGSQLKINANGVFSTTGGKFESKAGQHLFTGGAKV
SYEVPELP--------KNGPYAVDFLFASLAGNGIENAKIQLYEPDKKEIIWEGKTDLRGKSNLSLQNE---
---------SKRYEALIGFDDWSSIFDDEDDYEEEHEEEFEIGEHGMQAEENNLEE----------------
-----------------------------------------------------------------------
-----------------------

>SSA6_18
-MLFNIFSVLEKIGLNAQKRAIHVQFSNELLNHQVFLQRIEGQHQLNGGLMAELICLSTNAQIALKQFIGVQ
VAVDQVTDSGQLFRTTGIVTEASYGQSDGALTLYKLTIEDATNLWHKRRNSRVFMNKSIVEITEVLFKEWQE
KSPLFAASLSLDLGGLS-QNYDIRPFTMQHNEADYDFLTRLWRSEGVSWLIDESELFVPHFTAPIQAQKLRL
IDDNNQYQALARRSIRYHRSSATEYQDSITGFVAVRSLQPTAVHVQRWQPDALAQEEGNGSVVTTHTHSDNF
DSATLSLEQAWHVSPAWMQDLKGEDQATASSSSQLEKLNQHFTDMHASRAKYFKAYSSVRDTQVGYWFNLRE
HPEIDQ-HEGADQEFLIIAKNFYNQNNLPKDLQQQVSQLLTQSRWDQHGYD-----DIERQGNELTLIRRQI
KTAPEYNPEQHRPIAYPQRAKVVGPEGETIHVDEWGRIKVRFLFTRSDDHGHDGGAGSNDNDTDSAWVDVLT
PWAGEGYGARFLPRIGEVVVIDFFDGNIDRPFVTGRIHEAQRSPTKFDVKGQLPDTKKLSGIRSQEINGSGF
NQLRFDDTTGQISTQLQSSHAATQLNLGNLSHPKEQATSQGRGEGFELRTDAWGAVRAGKGMLISTYAQEQA
IADHLEAAQAQSLLSQGYESMKMLSEVAAKQQTDALNVINRLPKFIQSLELKTTGQALNSTVNLFKEGINND
PIHALKDCGGFIQDIGALGGNAKGVVDEFNSFFSDAKDAVENLKAFIENVEEHGPDIVKGKLASIKDRIHKN
PFESIQEVGKVLTNVETKDFDLMSTCGTFSKGSKLEVSPSKALSSLQGFMEGYTQGLESSSDTQQQEQGKIF
RQALMLLASPNGIALTTPENIILQASQDIAESASGSINLSAQKNIIGHAQDKISLFAAQKGLRAYAAKGKLE
LQAQDDAIEAIAKKVIKLISTEDKIELTSPKEIVLTAGGSQLKINANGVFSTTGGKFESKAGQHLFTGGAKV
SYEVPELP--------KNGPYAVDFLFASLAGNGIENAKIQLYEPDKKEIIWEGKTDLRGKSNLSLQNE---
---------SKRYEALIGFDDWSSIFDDEDDYEEEHEEEFEIGEHGMQAEENNLEE----------------
------------------------------------------------------------------------
-----------------------
>TCDC-AB0715_18
---------LEKIGLNAQKRAIHVQFSNELLNHQVFLQRIEGQHQLNGGLMAELICLSTNAQIALKQFIGVQ
VAVDQVTDSGQLFRTTGIVTEASYGQSDGALTLYKLTIEDATNLWHKRRNSRVFMNKSIVEITEVLFKEWQE
KSPLFAASLSLDLGGLS-QNYDIRPFTMQHNEADYDFLTRLWRSEGVSWLIDESELFVPHFTAPIQAQKLRL
IDDNNQYQALARRSIRYHRSSATEYQDSITGFVAVRSLQPTAVHVQRWQPDALAQEEGNGSVVTTHTHSDNF
DSATLSLEQAWHVSPAWMQDLKGEDQATASSSSQLEKLNQHFTDMHASRAKYFKAYSSVRDTQVGYWFNLRE
HPEIDQ-HEGADQEFLIIAKNFYNQNNLPKDLQQQVSQLLTQSRWDQHGYD-----DIERQGNELTLIRRQI
KTAPEYNPEQHRPIAYPQRAKVVGPEGETIHVDEWGRIKVRFLFTRSDDHGHDGGAGSNDNDTDSAWVDVLT
PWAGEGYGARFLPRIGEVVVIDFFDGNIDRPFVTGRIHEAQRSPTKFDVKGQLPDTKKLSGIRSQEINGSGF
NQLRFDDTTGQISTQLQSSHAATQLNLGNLSHPKEQATSQGRGEGFELRTDAWGAVRAGKGMLISTYAQEQA
IADHLEAAQAQSLLSQGYESMKMLSEVAAKQQTDALNVINRLPKFIQSLELKTTGQALNSTVNLFKEGINND
PIHALKDCGGFIQDIGALGGNAKGVVDEFNSFFSDAKDAVENLKAFIENVEEHGPDIVKGKLASIKDRIHKN
PFESIQEVGKVLTNVETKDFDLMSTCGTFSKGSKLEVSPSKALSSLQGFMEGYTQGLESSSDTQQQEQGKIF
RQALMLLASPNGIALTTPENIILQASQDIAESASGSINLSAQKNIIGHAQDKISLFAAQKGLRAYAAKGKLE

LQAQDDAIEAIAKKVIKLISTEDKIELTSPKEIVLTAGGSQLKINANGVFSTTGGKFESKAGQHLFTGGAKV
SYEVPELP--------KNGPYAVDFLFASLAGNGIENAKIQLYEPDKKEIIWEGKTDLRGKSNLSLQNE---
---------SKRYEALIGFDDWSSIFDDEDDYEEEHEEEFEIGEHGMQAEENNLEE----------------
------------------------------------------------------------------------
-----------------------
>USA2_18
-MLFNIFSVLEKIGLNAQKRAIHVQFSNELLNHQVFLQRIEGQHQLNGGLMAELICLSTNAQIALKQFIGVQ
VAVDQVTDSGQLFRTTGIVTEASYGQSDGALTLYKLTIEDATNLWHKRRNSRVFMNKSIVEITEVLFKEWQE
KSPLFAASLSLDLGGLS-QNYDIRPFTMQHNEADYDFLTRLWRSEGVSWLIDESELFVPHFTAPIQAQKLRL
IDDNNQYQALARRSIRYHRSSATEYQDSITGFVAVRSLQPTAVHVQRWQPDALAQEEGNGSVVTTHTHSDNF
DSATLSLEQAWHVSPAWMQDLKGEDQATASSSSQLEKLNQHFTDMHASRAKYFKAYSSVRDTQVGYWFNLRE
HPEIDQ-HEGADQEFLIIAKNFYNQNNLPKDLQQQVSQLLTQSRWDQHGYD-----DIERQGNELTLIRRQI
KTAPEYNPEQHRPIAYPQRAKVVGPEGETIHVDEWGRIKVRFLFTRSDDHGHDGGAGSNDNDTDSAWVDVLT
PWAGEGYGARFLPRIGEVVVIDFFDGNIDRPFVTGRIHEAQRSPTKFDVKGQLPDTKKLSGIRSQEINGSGF
NQLRFDDTTGQISTQLQSSHAATQLNLGNLSHPKEQATSQGRGEGFELRTDAWGAVRAGKGMLISTYAQEQA
IADHLEAAQAQSLLSQGYESMKMLSEVAAKQQTDALNVINRLPKFIQSLELKTTGQALNSTVNLFKEGINND
PIHALKDCGGFIQDIGALGGNAKGVVDEFNSFFSDAKDAVENLKAFIENVEEHGPDIVKGKLASIKDRIHKN
PFESIQEVGKVLTNVETKDFDLMSTCGTFSKGSKLEVSPSKALSSLQGFMEGYTQGLESSSDTQQQEQGKIF
RQALMLLASPNGIALTTPENIILQASQDIAESASGSINLSAQKNIIGHAQDKISLFAAQKGLRAYAAKGKLE
LQAQDDAIEAIAKKVIKLISTEDKIELTSPKEIVLTAGGSQLKINANGVFSTTGGKFESKAGQHLFTGGAKV
SYEVPELP--------KNGPYAVDFLFASLAGNGIENAKIQLYEPDKKEIIWEGKTDLRGKSNLSLQNE---
---------SKRYEALIGFDDWSSIFDDEDDYEEEHEEEFEIGEHGMQAEENNLEE----------------
------------------------------------------------------------------------
-----------------------
>WCHAB005133_18
-MLFNIFSVLEKIGLNAQKRAIHVQFSNELLNHQVFLQRIEGQHQLNGGLMAELICLSTNAQIALKQFIGVQ
VAVDQVTDSGQLFRTTGIVTEASYGQSDGALTLYKLTIEDATNLWHKRRNSRVFMNKSIVEITEVLFKEWQE
KSPLFAASLSLDLGGLS-QNYDIRPFTMQHNEADYDFLTRLWRSEGVSWLIDESELFVPHFTAPIQAQKLRL
IDDNNQYQALARRSIRYHRSSATEYQDSITGFVAVRSLQPTAVHVQRWQPDALAQEEGNGSVVTTHTHSDNF
DSATLSLEQAWHVSPAWMQDLKGEDQATASSSSQLEKLNQHFTDMHASRAKYFKAYSSVRDTQVGYWFNLRE
HPEIDQ-HEGADQEFLIIAKNFYNQNNLPKDLQQQVSQLLTQSRWDQHGYD-----DIERQGNELTLIRRQI
KTAPEYNPEQHRPIAYPQRAKVVGPEGETIHVDEWGRIKVRFLFTRSDDHGHDGGAGSNDNDTDSAWVDVLT
PWAGEGYGARFLPRIGEVVVIDFFDGNIDRPFVTGRIHEAQRSPTKFDVKGQLPDTKKLSGIRSQEINGSGF

NQLRFDDTTGQISTQLQSSHAATQLNLGNLSHPKEQATSQGRGEGFELRTDAWGAVRAGKGMLISTYAQEQA
IADHLEAAQAQSLLSQGYESMKMLSEVAAKQQTDALNVINRLPKFIQSLELKTTGQALNSTVNLFKEGINND
PIHALKDCGGFIQDIGALGGNAKGVVDEFNSFFSDAKDAVENLKAFIENVEEHGPDIVKGKLASIKDRIHKN
PFESIQEVGKVLTNVETKDFDLMSTCGTFSKGSKLEVSPSKALSSLQGFMEGYTQGLESSSDTQQQEQGKIF
RQALMLLASPNGIALTTPENIILQASQDIAESASGSINLSAQKNIIGHAQDKISLFAAQKGLRAYAAKGKLE
LQAQDDAIEAIAKKVIKLISTEDKIELTSPKEIVLTAGGSQLKINANGVFSTTGGKFESKAGQHLFTGGAKV
SYEVPELP--------KNGPYAVDFLFASLAGNGIENAKIQLYEPDKKEIIWEGKTDLRGKSNLSLQNE---
---------SKRYEALIGFDDWSSIFDDEDDYEEEHEEEFEIGEHGMQAEENNLEE----------------
------------------------------------------------------------------------
-----------------------
>XDR-BJ83_18
-MLFNIFSVLEKIGLNAQKRAIHVQFSNELLNHQVFLQRIEGQHQLNGGLMAELICLSTNAQIALKQFIGVQ
VAVDQVTDSGQLFRTTGIVTEASYGQSDGALTLYKLTIEDATNLWHKRRNSRVFMNKSIVEITEVLFKEWQE
KSPLFAASLSLDLGGLS-QNYDIRPFTMQHNEADYDFLTRLWRSEGVSWLIDESELFVPHFTAPIQAQKLRL
IDDNNQYQALARRSIRYHRSSATEYQDSITGFVAVRSLQPTAVHVQRWQPDALAQEEGNGSVVTTHTHSDNF
DSATLSLEQAWHVSPAWMQDLKGEDQATASSSSQLEKLNQHFTDMHASRAKYFKAYSSVRDTQVGYWFNLRE
HPEIDQ-HEGADQEFLIIAKNFYNQNNLPKDLQQQVSQLLTQSRWDQHGYD-----DIERQGNELTLIRRQI
KTAPEYNPEQHRPIAYPQRAKVVGPEGETIHVDEWGRIKVRFLFTRSDDHGHDGGAGSNDNDTDSAWVDVLT
PWAGEGYGARFLPRIGEVVVIDFFDGNIDRPFVTGRIHEAQRSPTKFDVKGQLPDTKKLSGIRSQEINGSGF
NQLRFDDTTGQISTQLQSSHAATQLNLGNLSHPKEQATSQGRGEGFELRTDAWGAVRAGKGMLISTYAQEQA
IADHLEAAQAQSLLSQGYESMKMLSEVAAKQQTDALNVINRLPKFIQSLELKTTGQALNSTVNLFKEGINND
PIHALKDCGGFIQDIGALGGNAKGVVDEFNSFFSDAKDAVENLKAFIENVEEHGPDIVKGKLASIKDRIHKN
PFESIQEVGKVLTNVETKDFDLMSTCGTFSKGSKLEVSPSKALSSLQGFMEGYTQGLESSSDTQQQEQGKIF
RQALMLLASPNGIALTTPENIILQASQDIAESASGSINLSAQKNIIGHAQDKISLFAAQKGLRAYAAKGKLE
LQAQDDAIEAIAKKVIKLISTEDKIELTSPKEIVLTAGGSQLKINANGVFSTTGGKFESKAGQHLFTGGAKV
SYEVPELP--------KNGPYAVDFLFASLAGNGIENAKIQLYEPDKKEIIWEGKTDLRGKSNLSLQNE---
---------SKRYEALIGFDDWSSIFDDEDDYEEEHEEEFEIGEHGMQAEENNLEE----------------
------------------------------------------------------------------------
-----------------------
>XH386_18
-MLFNIFSVLEKIGLNAQKRAIHVQFSNELLNHQVFLQRIEGQHQLNGGLMAELICLSTNAQIALKQFIGVQ
VAVDQVTDSGQLFRTTGIVTEASYGQSDGALTLYKLTIEDATNLWHKRRNSRVFMNKSIVEITEVLFKEWQE
KSPLFAASLSLDLGGLS-QNYDIRPFTMQHNEADYDFLTRLWRSEGVSWLIDESELFVPHFTAPIQAQKLRL

IDDNNQYQALARRSIRYHRSSATEYQDSITGFVAVRSLQPTAVHVQRWQPDALAQEEGNGSVVTTHTHSDNF
DSATLSLEQAWHVSPAWMQDLKGEDQATASSSSQLEKLNQHFTDMHASRAKYFKAYSSVRDTQVGYWFNLRE
HPEIDQ-HEGADQEFLIIAKNFYNQNNLPKDLQQQVSQLLTQSRWDQHGYD-----DIERQGNELTLIRRQI
KTAPEYNPEQHRPIAYPQRAKVVGPEGETIHVDEWGRIKVRFLFTRSDDHGHDGGAGSNDNDTDSAWVDVLT
PWAGEGYGARFLPRIGEVVVIDFFDGNIDRPFVTGRIHEAQRSPTKFDVKGQLPDTKKLSGIRSQEINGSGF
NQLRFDDTTGQISTQLQSSHAATQLNLGNLSHPKEQATSQGRGEGFELRTDAWGAVRAGKGMLISTYAQEQA
IADHLEAAQAQSLLSQGYESMKMLSEVAAKQQTDALNVINRLPKFIQSLELKTTGQALNSTVNLFKEGINND
PIHALKDCGGFIQDIGALGGNAKGVVDEFNSFFSDAKDAVENLKAFIENVEEHGPDIVKGKLASIKDRIHKN
PFESIQEVGKVLTNVETKDFDLMSTCGTFSKGSKLEVSPSKALSSLQGFMEGYTQGLESSSDTQQQEQGKIF
RQALMLLASPNGIALTTPENIILQASQDIAESASGSINLSAQKNIIGHAQDKISLFAAQKGLRAYAAKGKLE
LQAQDDAIEAIAKKVIKLISTEDKIELTSPKEIVLTAGGSQLKINANGVFSTTGGKFESKAGQHLFTGGAKV
SYEVPELP--------KNGPYAVDFLFASLAGNGIENAKIQLYEPDKKEIIWEGKTDLRGKSNLSLQNE---
---------SKRYEALIGFDDWSSIFDDEDDYEEEHEEEFEIGEHGMQAEENNLEE----------------
------------------------------------------------------------------------
-----------------------
>XH856_18
-MLFNIFSVLEKIGLNAQKRAIHVQFSNELLNHQVFLQRIEGQHQLNGGLMAELICLSTNAQIALKQFIGVQ
VAVDQVTDSGQLFRTTGIVTEASYGQSDGALTLYKLTIEDATNLWHKRRNSRVFMNKSIVEITEVLFKEWQE
KSPLFAASLSLDLGGLS-QNYDIRPFTMQHNEADYDFLTRLWRSEGVSWLIDESELFVPHFTAPIQAQKLRL
IDDNNQYQALARRSIRYHRSSATEYQDSITGFVAVRSLQPTAVHVQRWQPDALAQEEGNGSVVTTHTHSDNF
DSATLSLEQAWHVSPAWMQDLKGEDQATASSSSQLEKLNQHFTDMHASRAKYFKAYSSVRDTQVGYWFNLRE
HPEIDQ-HEGADQEFLIIAKNFYNQNNLPKDLQQQVSQLLTQSRWDQHGYD-----DIERQGNELTLIRRQI
KTAPEYNPEQHRPIAYPQRAKVVGPEGETIHVDEWGRIKVRFLFTRSDDHGHDGGAGSNDNDTDSAWVDVLT
PWAGEGYGARFLPRIGEVVVIDFFDGNIDRPFVTGRIHEAQRSPTKFDVKGQLPDTKKLSGIRSQEINGSGF
NQLRFDDTTGQISTQLQSSHAATQLNLGNLSHPKEQATSQGRGEGFELRTDAWGAVRAGKGMLISTYAQEQA
IADHLEAAQAQSLLSQGYESMKMLSEVAAKQQTDALNVINRLPKFIQSLELKTTGQALNSTVNLFKEGINND
PIHALKDCGGFIQDIGALGGNAKGVVDEFNSFFSDAKDAVENLKAFIENVEEHGPDIVKGKLASIKDRIHKN
PFESIQEVGKVLTNVETKDFDLMSTCGTFSKGSKLEVSPSKALSSLQGFMEGYTQGLESSSDTQQQEQGKIF
RQALMLLASPNGIALTTPENIILQASQDIAESASGSINLSAQKNIIGHAQDKISLFAAQKGLRAYAAKGKLE
LQAQDDAIEAIAKKVIKLISTEDKIELTSPKEIVLTAGGSQLKINANGVFSTTGGKFESKAGQHLFTGGAKV
SYEVPELP--------KNGPYAVDFLFASLAGNGIENAKIQLYEPDKKEIIWEGKTDLRGKSNLSLQNE---
---------SKRYEALIGFDDWSSIFDDEDDYEEEHEEEFEIGEHGMQAEENNLEE----------------
------------------------------------------------------------------------

-----------------------
>XH859_18
-MLFNIFSVLEKIGLNAQKRAIHVQFSNELLNHQVFLQRIEGQHQLNGGLMAELICLSTNAQIALKQFIGVQ
VAVDQVTDSGQLFRTTGIVTEASYGQSDGALTLYKLTIEDATNLWHKRRNSRVFMNKSIVEITEVLFKEWQE
KSPLFAASLSLDLGGLS-QNYDIRPFTMQHNEADYDFLTRLWRSEGVSWLIDESELFVPHFTAPIQAQKLRL
IDDNNQYQALARRSIRYHRSSATEYQDSITGFVAVRSLQPTAVHVQRWQPDALAQEEGNGSVVTTHTHSDNF
DSATLSLEQAWHVSPAWMQDLKGEDQATASSSSQLEKLNQHFTDMHASRAKYFKAYSSVRDTQVGYWFNLRE
HPEIDQ-HEGADQEFLIIAKNFYNQNNLPKDLQQQVSQLLTQSRWDQHGYD-----DIERQGNELTLIRRQI
KTAPEYNPEQHRPIAYPQRAKVVGPEGETIHVDEWGRIKVRFLFTRSDDHGHDGGAGSNDNDTDSAWVDVLT
PWAGEGYGARFLPRIGEVVVIDFFDGNIDRPFVTGRIHEAQRSPTKFDVKGQLPDTKKLSGIRSQEINGSGF
NQLRFDDTTGQISTQLQSSHAATQLNLGNLSHPKEQATSQGRGEGFELRTDAWGAVRAGKGMLISTYAQEQA
IADHLEAAQAQSLLSQGYESMKMLSEVAAKQQTDALNVINRLPKFIQSLELKTTGQALNSTVNLFKEGINND
PIHALKDCGGFIQDIGALGGNAKGVVDEFNSFFSDAKDAVENLKAFIENVEEHGPDIVKGKLASIKDRIHKN
PFESIQEVGKVLTNVETKDFDLMSTCGTFSKGSKLEVSPSKALSSLQGFMEGYTQGLESSSDTQQQEQGKIF
RQALMLLASPNGIALTTPENIILQASQDIAESASGSINLSAQKNIIGHAQDKISLFAAQKGLRAYAAKGKLE
LQAQDDAIEAIAKKVIKLISTEDKIELTSPKEIVLTAGGSQLKINANGVFSTTGGKFESKAGQHLFTGGAKV
SYEVPELP--------KNGPYAVDFLFASLAGNGIENAKIQLYEPDKKEIIWEGKTDLRGKSNLSLQNE---
---------SKRYEALIGFDDWSSIFDDEDDYEEEHEEEFEIGEHGMQAEENNLEE----------------
------------------------------------------------------------------------
-----------------------
>YU-R612_18
-MLFNIFSVLEKIGLNAQKRAIHVQFSNELLNHQVFLQRIEGQHQLNGGLMAELICLSTNAQIALKQFIGVQ
VAVDQVTDSGQLFRTTGIVTEASYGQSDGALTLYKLTIEDATNLWHKRRNSRVFMNKSIVEITEVLFKEWQE
KSPLFAASLSLDLGGLS-QNYDIRPFTMQHNEADYDFLTRLWRSEGVSWLIDESELFVPHFTAPIQAQKLRL
IDDNNQYQALARRSIRYHRSSATEYQDSITGFVAVRSLQPTAVHVQRWQPDALAQEEGNGSVVTTHTHSDNF
DSATLSLEQAWHVSPAWMQDLKGEDQATASSSSQLEKLNQHFTDMHASRAKYFKAYSSVRDTQVGYWFNLRE
HPEIDQ-HEGADQEFLIIAKNFYNQNNLPKDLQQQVSQLLTQSRWDQHGYD-----DIERQGNELTLIRRQI
KTAPEYNPEQHRPIAYPQRAKVVGPEGETIHVDEWGRIKVRFLFTRSDDHGHDGGAGSNDNDTDSAWVDVLT
PWAGEGYGARFLPRIGEVVVIDFFDGNIDRPFVTGRIHEAQRSPTKFDVKGQLPDTKKLSGIRSQEINGSGF
NQLRFDDTTGQISTQLQSSHAATQLNLGNLSHPKEQATSQGRGEGFELRTDAWGAVRAGKGMLISTYAQEQA
IADHLEAAQAQSLLSQGYESMKMLSEVAAKQQTDALNVINRLPKFIQSLELKTTGQALNSTVNLFKEGINND
PIHALKDCGGFIQDIGALGGNAKGVVDEFNSFFSDAKDAVENLKAFIENVEEHGPDIVKGKLASIKDRIHKN
PFESIQEVGKVLTNVETKDFDLMSTCGTFSKGSKLEVSPSKALSSLQGFMEGYTQGLESSSDTQQQEQGKIF

RQALMLLASPNGIALTTPENIILQASQDIAESASGSINLSAQKNIIGHAQDKISLFAAQKGLRAYAAKGKLE
LQAQDDAIEAIAKKVIKLISTEDKIELTSPKEIVLTAGGSQLKINANGVFSTTGGKFESKAGQHLFTGGAKV
SYEVPELP--------KNGPYAVDFLFASLAGNGIENAKIQLYEPDKKEIIWEGKTDLRGKSNLSLQNE---
---------SKRYEALIGFDDWSSIFDDEDDYEEEHEEEFEIGEHGMQAEENNLEE----------------
------------------------------------------------------------------------
-----------------------

>AB030_24
-MLFNIFSVLEKIGLNAQKRAIHVQFSNELLNHQVFLQRIEGQHQLNGGLMAELICLSTNAQIALKQFIGVQ
VAVDQVTDSGQLFRTTGIVTEASYGQSDGALTLYKLTIEDATNLWHKRRNSRVFMNKSIVEITEVLFKEWQE
KSPLFAASLSLDLGGLS-QNYDIRPFTMQHNESDYDFLTRLWRSEGVSWLIDESELFVPHFTAPIQAQKLRL
IDDNNQYQALARRSIRYHRSNATEYQDSITGFVAVRSLQPTAVHVQRWQPDALAQEEGNGSVVTTHTHSDNF
DSATLSLEQAWHVSPAWMQDLKGEDQATASSSSQLEKLNQHFTDMHASRAKYFKAYSSVRDTQVGYWFNLRE
HPEIDQ-HEGADQEFLIIAKNFYNQNNLPKDLHQQVSQLLTQSRWDQHGYD-----DIERQSNELTLIRRQI
KTAPEYNPEQHRPIAYPQRAKVVGPEGETIHVDEWGRIKVRFLFTRSDDHGHDGGAGSNDNDTDSAWVSVLT
PWAGEGYGARFLPRIGEVVVIDFFDGNIDRPFVTGRIHEAQRSPTKFDVKGQLPDTKKLSGIRSQEVNGSGF
NQLRFDDTTGQISTQLQSSHAATQLNLGNLSHPKEQATSQGRGEGFELRTDAWGAVRAGKGMLISTYAQEQA
IADHLEAAQAQSLLSQGYESMKMLSEVAAKQQTDALNVINRLPKFIQSLELKTTGQALNSTVNLFKEGINND
PIHALKDCGGFIQDIGALGGNTKGVVDEFNSFFSDAKDAVENLKAFIENVEEHGPDIVKGKLASIKDRIHKN
PFESIQEVGKVLANVETKDFDLMSTCGTFSKGSKLEVSPSKALSSLQGFMEGYTQGLESSSDTQQQEQGKIF
RQALMLLASPNGIALTTPENIILQASQDIAESASGSINLSAQKNIIGHAQDKISLFAAQKGLRAYAAKGKLE
LQAQDDAIEAIAKKVIKLISTEEKIELTSPKEIVLTAGGSQLKINANGVFSTTGGKFESKAGQHLFMSGATV
NAELPKMP------ESGIFSRRFDFSDIFNLEKLKEEIRFKVIN-KTKNTEYIGMLDEMGRTPRIFSDS--S
DTIEIQFISNNENKSIIPTKELNEDIYNHDTGDFVDDGIQSQLAEALDDEYKDDLEDDFNKFGV--------
------------------------------------------------------------------------
-----------------------

>AbH12O-A2_24
-MLFNIFSVLEKIGLNAQKRAIHVQFSNELLNHQVFLQRIEGQHQLNGGLMAELICLSTNAQIALKQFIGVQ
VAVDQVTDSGQLFRTTGIVTEASYGQSDGALTLYKLTIEDATNLWHKRRNSRVFMNKSIVEITEVLFKEWQE
KSPLFAASLSLDLGGLS-QNYDIRPFTMQHNESDYDFLTRLWRSEGVSWLIDESELFVPHFTAPIQAQKLRL
IDDNNQYQALARRSIRYHRSNATEYQDSITGFVAVRSLQPTAVHVQRWQPDALAQEEGNGSVVTTHTHSDNF
DSATLSLEQAWHVSPAWMQDLKGEDQATASSSSQLEKLNQHFTDMHASRAKYFKAYSSVRDTQVGYWFNLRE
HPEIDQ-HEGADQEFLIIAKNFYNQNNLPKDLHQQVSQLLTQSRWDQHGYD-----DIERQSNELTLIRRQI
KTAPEYNPEQHRPIAYPQRAKVVGPEGETIHVDEWGRIKVRFLFTRSDDHGHDGGAGSNDNDTDSAWVSVLT

PWAGEGYGARFLPRIGEVVVIDFFDGNIDRPFVTGRIHEAQRSPTKFDVKGQLPDTKKLSGIRSQEVNGSGF
NQLRFDDTTGQISTQLQSSHAATQLNLGNLSHPKEQATSQGRGEGFELRTDAWGAVRAGKGMLISTYAQEQA
IADHLEAAQAQSLLSQGYESMKMLSEVAAKQQTDALNVINRLPKFIQSLELKTTGQALNSTVNLFKEGINND
PIHALKDCGGFIQDIGALGGNTKGVVDEFNSFFSDAKDAVENLKAFIENVEEHGPDIVKGKLASIKDRIHKN
PFESIQEVGKVLANVETKDFDLMSTCGTFSKGSKLEVSPSKALSSLQGFMEGYTQGLESSSDTQQQEQGKIF
RQALMLLASPNGIALTTPENIILQASQDIAESASGSINLSAQKNIIGHAQDKISLFAAQKGLRAYAAKGKLE
LQAQDDAIEAIAKKVIKLISTEEKIELTSPKEIVLTAGGSQLKINANGVFSTTGGKFESKAGQHLFMSGATV
NAELPKMP------ESGIFSRRFDFSDIFNLEKLKEEIRFKVIN-KTKNTEYIGMLDEMGRTPRIFSDS--S
DTIEIQFISNNENKSIIPTKELNEDIYNHDTGDFVDDGIQSQLAEALDDEYKDDLEDDFNKFGV--------
------------------------------------------------------------------------
-----------------------
>AF-401_24
-MLFNIFSVLEKIGLNAQKRAIHVQFSNELLNHQVFLQRIEGQHQLNGGLMAELICLSTNAQIALKQFIGVQ
VAVDQVTDSGQLFRTTGIVTEASYGQSDGALTLYKLTIEDATNLWHKRRNSRVFMNKSIVEITEVLFKEWQE
KSPLFAASLSLDLGGLS-QNYDIRPFTMQHNESDYDFLTRLWRSEGVSWLIDESELFVPHFTAPIQAQKLRL
IDDNNQYQALARRSIRYHRSNATEYQDSITGFVAVRSLQPTAVHVQRWQPDALAQEEGNGSVVTTHTHSDNF
DSATLSLEQAWHVSPAWMQDLKGEDQATASSSSQLEKLNQHFTDMHASRAKYFKAYSSVRDTQVGYWFNLRE
HPEIDQ-HEGADQEFLIIAKNFYNQNNLPKDLHQQVSQLLTQSRWDQHGYD-----DIERQSNELTLIRRQI
KTAPEYNPEQHRPIAYPQRAKVVGPEGETIHVDEWGRIKVRFLFTRSDDHGHDGGAGSNDNDTDSAWVSVLT
PWAGEGYGARFLPRIGEVVVIDFFDGNIDRPFVTGRIHEAQRSPTKFDVKGQLPDTKKLSGIRSQEVNGSGF
NQLRFDDTTGQISTQLQSSHAATQLNLGNLSHPKEQATSQGRGEGFELRTDAWGAVRAGKGMLISTYAQEQA
IADHLEAAQAQSLLSQGYESMKMLSEVAAKQQTDALNVINRLPKFIQSLELKTTGQALNSTVNLFKEGINND
PIHALKDCGGFIQDIGALGGNTKGVVDEFNSFFSDAKDAVENLKAFIENVEEHGPDIVKGKLASIKDRIHKN
PFESIQEVGKVLANVETKDFDLMSTCGTFSKGSKLEVSPSKALSSLQGFMEGYTQGLESSSDTQQQEQGKIF
RQALMLLASPNGIALTTPENIILQASQDIAESASGSINLSAQKNIIGHAQDKISLFAAQKGLRAYAAKGKLE
LQAQDDAIEAIAKKVIKLISTEEKIELTSPKEIVLTAGGSQLKINANGVFSTTGGKFESKAGQHLFMSGATV
NAELPKMP------ESGIFSRRFDFSDIFNLEKLKEEIRFKVIN-KTKNTEYIGMLDEMGRTPRIFSDS--S
DTIEIQFISNNENKSIIPTKELNEDIYNHDTGDFVDDGIQSQLAEALDDEYKDDLEDDFNKFGV--------
------------------------------------------------------------------------
-----------------------
>AR_0063_24
-MLFNIFSVLEKIGLNAQKRAIHVQFSNELLNHQVFLQRIEGQHQLNGGLMAELICLSTNAQIALKQFIGVQ
VAVDQVTDSGQLFRTTGIVTEASYGQSDGALTLYKLTIEDATNLWHKRRNSRVFMNKSIVEITEVLFKEWQE

```
KSPLFAASLSLDLGGLS-QNYDIRPFTMQHNESDYDFLTRLWRSEGVSWLIDESELFVPHFTAPIQAQKLRL
IDDNNQYQALARRSIRYHRSNATEYQDSITGFVAVRSLQPTAVHVQRWQPDALAQEEGNGSVVTTHTHSDNF
DSATLSLEQAWHVSPAWMQDLKGEDQATASSSSQLEKLNQHFTDMHASRAKYFKAYSSVRDTQVGYWFNLRE
HPEIDQ-HEGADQEFLIIAKNFYNQNNLPKDLHQQVSQLLTQSRWDQHGYD-----DIERQSNELTLIRRQI
KTAPEYNPEQHRPIAYPQRAKVVGPEGETIHVDEWGRIKVRFLFTRSDDHGHDGGAGSNDNDTDSAWVSVLT
PWAGEGYGARFLPRIGEVVVIDFFDGNIDRPFVTGRIHEAQRSPTKFDVKGQLPDTKKLSGIRSQEVNGSGF
NQLRFDDTTGQISTQLQSSHAATQLNLGNLSHPKEQATSQGRGEGFELRTDAWGAVRAGKGMLISTYAQEQA
IADHLEAAQAQSLLSQGYESMKMLSEVAAKQQTDALNVINRLPKFIQSLELKTTGQALNSTVNLFKEGINND
PIHALKDCGGFIQDIGALGGNTKGVVDEFNSFFSDAKDAVENLKAFIENVEEHGPDIVKGKLASIKDRIHKN
PFESIQEVGKVLANVETKDFDLMSTCGTFSKGSKLEVSPSKALSSLQGFMEGYTQGLESSSDTQQQEQGKIF
RQALMLLASPNGIALTTPENIILQASQDIAESASGSINLSAQKNIIGHAQDKISLFAAQKGLRAYAAKGKLE
LQAQDDAIEAIAKKVIKLISTEEKIELTSPKEIVLTAGGSQLKINANGVFSTTGGKFESKAGQHLFMSGATV
NAELPKMP------ESGIFSRRFDFSDIFNLEKLKEEIRFKVIN-KTKNTEYIGMLDEMGRTPRIFSDS--S
DTIEIQFISNNENKSIIPTKELNEDIYNHDTGDFVDDGIQSQLAEALDDEYKDDLEDDFNKFGV--------
------------------------------------------------------------------------
-----------------------
>AR_0101_24
-MLFNIFSVLEKIGLNAQKRAIHVQFSNELLNHQVFLQRIEGQHQLNGGLMAELICLSTNAQIALKQFIGVQ
VAVDQVTDSGQLFRTTGIVTEASYGQSDGALTLYKLTIEDATNLWHKRRNSRVFMNKSIVEITEVLFKEWQE
KSPLFAASLSLDLGGLS-QNYDIRPFTMQHNESDYDFLTRLWRSEGVSWLIDESELFVPHFTAPIQAQKLRL
IDDNNQYQALARRSIRYHRSNATEYQDSITGFVAVRSLQPTAVHVQRWQPDALAQEEGNGSVVTTHTHSDNF
DSATLSLEQAWHVSPAWMQDLKGEDQATASSSSQLEKLNQHFTDMHASRAKYFKAYSSVRDTQVGYWFNLRE
HPEIDQ-HEGADQEFLIIAKNFYNQNNLPKDLHQQVSQLLTQSRWDQHGYD-----DIERQSNELTLIRRQI
KTAPEYNPEQHRPIAYPQRAKVVGPEGETIHVDEWGRIKVRFLFTRSDDHGHDGGAGSNDNDTDSAWVSVLT
PWAGEGYGARFLPRIGEVVVIDFFDGNIDRPFVTGRIHEAQRSPTKFDVKGQLPDTKKLSGIRSQEVNGSGF
NQLRFDDTTGQISTQLQSSHAATQLNLGNLSHPKEQATSQGRGEGFELRTDAWGAVRAGKGMLISTYAQEQA
IADHLEAAQAQSLLSQGYESMKMLSEVAAKQQTDALNVINRLPKFIQSLELKTTGQALNSTVNLFKEGINND
PIHALKDCGGFIQDIGALGGNTKGVVDEFNSFFSDAKDAVENLKAFIENVEEHGPDIVKGKLASIKDRIHKN
PFESIQEVGKVLANVETKDFDLMSTCGTFSKGSKLEVSPSKALSSLQGFMEGYTQGLESSSDTQQQEQGKIF
RQALMLLASPNGIALTTPENIILQASQDIAESASGSINLSAQKNIIGHAQDKISLFAAQKGLRAYAAKGKLE
LQAQDDAIEAIAKKVIKLISTEEKIELTSPKEIVLTAGGSQLKINANGVFSTTGGKFESKAGQHLFMSGATV
NAELPKMP------ESGIFSRRFDFSDIFNLEKLKEEIRFKVIN-KTKNTEYIGMLDEMGRTPRIFSDS--S
DTIEIQFISNNENKSIIPTKELNEDIYNHDTGDFVDDGIQSQLAEALDDEYKDDLEDDFNKFGV--------
```

------------------------------------------------------------------------
-----------------------
>Ab04-mff_11
-MLFNIFSVLEKIGLNAQKRAIHVQFSNELLNHQVFLQRIEGQHQLNGGLMAELICLSTNAQIALKQFIGVQ
VAVDQVTDSGQLFRTTGIVTEASYGQSDGALTLYKLTIEDATNLWHKRRNSRVFMNKSIVEITEVLFKEWQE
KSPLFAASLSLDLGGLS-QNYDIRPFTMQHNESDYDFLTRLWRSEGVSWLIDESELFVPHFTAPIQAQKLKL
IDDNNQYQALARRSIRYHRSSATEYQDSITGFVAVRSLQPTAVHVQRWQPDALAQEEGNGSVVTTHTHSDNF
DSATLSLEQAWHVSPAWMQDLKGEDQATASSSSQLEKLNQHFTDMHASRAKYFKAYSSVRDTQVGYWFNLRE
HPEIDQ-HEGADQEFLIIAKNFYNQNNLPKDLHQQVSQLLTQSRWDQHGYD-----DIERQSNELTLIRRQI
KTAPEYNPEQHRPIAYPQRAKVVGPEGETIHVDEWGRIKVRFLFTRSDDHGHDGGAGSNDNDTDSAWVDVLT
PWAGEGYGARFLPRIGEVVVIDFFDGNIDRPFVTGRIHEAQRSPTKFDVKGQLPDTKKLSGIRSQEVNGSGF
NQLRFDDTTGQISTQLQSSHAATQLNLGNLSHPKEQATSQGRGEGFELRTDAWGAVRAGKGMLISTYAQEQA
IADHLEAAQAQSLLSQGYESMKMLSEVAAKQQTDALNVINRLPKFIQSLELKTTGQALNSTVNLFKEGINND
PIHALKDCGGFIQDIGALGGNAKGVVDEFNSFFSDAKDAVENLKAFIENVEEHGPDIVKGKLASIKDRIHKN
PFESIQEVGKVLANVETKDFDLMSTCGTFSKGSKLEVSPSKALSSLQGFMEGYTQGLESSSDTKQQEQGKIF
RQALMLLASPNGIALTTPENIILQASQDIAESASGSINLSAQKNIIGHAQDKISLFAAQKGLRAYAAKGKLE
LQAQDDAIEAIAKKVIKLISTEEKIELTSPKEIVLTAGGSQLKINANGVFSTTGGKFESKAGQHLFMSGATV
NAELPKMP------ETGMYSMRFDLSQIF-DTKILKNMEYKLIN-HSKKIEAVYEFEQESSARVYSDSV-DN
VELALVPGVYLTEIKELISEQEAESLDDEDIDSCGCGEEHEHD-----------------------------
------------------------------------------------------------------------
-----------------------
>BJAB0715_11
-MLFNIFSVLEKIGLNAQKRAIHVQFSNELLNHQVFLQRIEGQHQLNGGLMAELICLSTNAQIALKQFIGVQ
VAVDQVTDSGQLFRTTGIVTEASYGQSDGALTLYKLTIEDATNLWHKRRNSRVFMNKSIVEITEVLFKEWQE
KSPLFAASLSLDLGGLS-QNYDIRPFTMQHNESDYDFLTRLWRSEGVSWLIDESELFVPHFTAPIQAQKLKL
IDDNNQYQALARRSIRYHRSSATEYQDSITGFVAVRSLQPTAVHVQRWQPDALAQEEGNGSVVTTHTHSDNF
DSATLSLEQAWHVSPAWMQDLKGEDQATASSSSQLEKLNQHFTDMHASRAKYFKAYSSVRDTQVGYWFNLRE
HPEIDQ-HEGADQEFLIIAKNFYNQNNLPKDLHQQVSQLLTQSRWDQHGYD-----DIERQSNELTLIRRQI
KTAPEYNPEQHRPIAYPQRAKVVGPEGETIHVDEWGRIKVRFLFTRSDDHGHDGGAGSNDNDTDSAWVDVLT
PWAGEGYGARFLPRIGEVVVIDFFDGNIDRPFVTGRIHEAQRSPTKFDVKGQLPDTKKLSGIRSQEVNGSGF
NQLRFDDTTGQISTQLQSSHAATQLNLGNLSHPKEQATSQGRGEGFELRTDAWGAVRAGKGMLISTYAQEQA
IADHLEAAQAQSLLSQGYESMKMLSEVAAKQQTDALNVINRLPKFIQSLELKTTGQALNSTVNLFKEGINND
PIHALKDCGGFIQDIGALGGNAKGVVDEFNSFFSDAKDAVENLKAFIENVEEHGPDIVKGKLASIKDRIHKN

PFESIQEVGKVLANVETKDFDLMSTCGTFSKGSKLEVSPSKALSSLQGFMEGYTQGLESSSDTKQQEQGKIF
RQALMLLASPNGIALTTPENIILQASQDIAESASGSINLSAQKNIIGHAQDKISLFAAQKGLRAYAAKGKLE
LQAQDDAIEAIAKKVIKLISTEEKIELTSPKEIVLTAGGSQLKINANGVFSTTGGKFESKAGQHLFMSGATV
NAELPKMP------ETGMYSMRFDLSQIF-DTKILKNMEYKLIN-HSKKIEAVYEFEQESSARVYSDSV-DN
VELALVPGVYLTEIKELISEQEAESLDDEDIDSCGCGEEHEHD-----------------------------
------------------------------------------------------------------------
-----------------------
>WKA02_11
-MLFNIFSVLEKIGLNAQKRAIHVQFSNELLNHQVFLQRIEGQHQLNGGLMAELICLSTNAQIALKQFIGVQ
VAVDQVTDSGQLFRTTGIVTEASYGQSDGALTLYKLTIEDATNLWHKRRNSRVFMNKSIVEITEVLFKEWQE
KSPLFAASLSLDLGGLS-QNYDIRPFTMQHNESDYDFLTRLWRSEGVSWLIDESELFVPHFTAPIQAQKLKL
IDDNNQYQALARRSIRYHRSSATEYQDSITGFVAVRSLQPTAVHVQRWQPDALAQEEGNGSVVTTHTHSDNF
DSATLSLEQAWHVSPAWMQDLKGEDQATASSSSQLEKLNQHFTDMHASRAKYFKAYSSVRDTQVGYWFNLRE
HPEIDQ-HEGADQEFLIIAKNFYNQNNLPKDLHQQVSQLLTQSRWDQHGYD-----DIERQSNELTLIRRQI
KTAPEYNPEQHRPIAYPQRAKVVGPEGETIHVDEWGRIKVRFLFTRSDDHGHDGGAGSNDNDTDSAWVDVLT
PWAGEGYGARFLPRIGEVVVIDFFDGNIDRPFVTGRIHEAQRSPTKFDVKGQLPDTKKLSGIRSQEVNGSGF
NQLRFDDTTGQISTQLQSSHAATQLNLGNLSHPKEQATSQGRGEGFELRTDAWGAVRAGKGMLISTYAQEQA
IADHLEAAQAQSLLSQGYESMKMLSEVAAKQQTDALNVINRLPKFIQSLELKTTGQALNSTVNLFKEGINND
PIHALKDCGGFIQDIGALGGNAKGVVDEFNSFFSDAKDAVENLKAFIENVEEHGPDIVKGKLASIKDRIHKN
PFESIQEVGKVLANVETKDFDLMSTCGTFSKGSKLEVSPSKALSSLQGFMEGYTQGLESSSDTKQQEQGKIF
RQALMLLASPNGIALTTPENIILQASQDIAESASGSINLSAQKNIIGHAQDKISLFAAQKGLRAYAAKGKLE
LQAQDDAIEAIAKKVIKLISTEEKIELTSPKEIVLTAGGSQLKINANGVFSTTGGKFESKAGQHLFMSGATV
NAELPKMP------ETGMYSMRFDLSQIF-DTKILKNMEYKLIN-HSKKIEAVYEFEQESSARVYSDSV-DN
VELALVPGVYLTEIKELISEQEAESLDDEDIDSCGCGEEHEHD-----------------------------
------------------------------------------------------------------------
-----------------------
>XH858_11
-MLFNIFSVLEKIGLNAQKRAIHVQFSNELLNHQVFLQRIEGQHQLNGGLMAELICLSTNAQIALKQFIGVQ
VAVDQVTDSGQLFRTTGIVTEASYGQSDGALTLYKLTIEDATNLWHKRRNSRVFMNKSIVEITEVLFKEWQE
KSPLFAASLSLDLGGLS-QNYDIRPFTMQHNESDYDFLTRLWRSEGVSWLIDESELFVPHFTAPIQAQKLKL
IDDNNQYQALARRSIRYHRSSATEYQDSITGFVAVRSLQPTAVHVQRWQPDALAQEEGNGSVVTTHTHSDNF
DSATLSLEQAWHVSPAWMQDLKGEDQATASSSSQLEKLNQHFTDMHASRAKYFKAYSSVRDTQVGYWFNLRE
HPEIDQ-HEGADQEFLIIAKNFYNQNNLPKDLHQQVSQLLTQSRWDQHGYD-----DIERQSNELTLIRRQI

KTAPEYNPEQHRPIAYPQRAKVVGPEGETIHVDEWGRIKVRFLFTRSDDHGHDGGAGSNDNDTDSAWVDVLT
PWAGEGYGARFLPRIGEVVVIDFFDGNIDRPFVTGRIHEAQRSPTKFDVKGQLPDTKKLSGIRSQEVNGSGF
NQLRFDDTTGQISTQLQSSHAATQLNLGNLSHPKEQATSQGRGEGFELRTDAWGAVRAGKGMLISTYAQEQA
IADHLEAAQAQSLLSQGYESMKMLSEVAAKQQTDALNVINRLPKFIQSLELKTTGQALNSTVNLFKEGINND
PIHALKDCGGFIQDIGALGGNAKGVVDEFNSFFSDAKDAVENLKAFIENVEEHGPDIVKGKLASIKDRIHKN
PFESIQEVGKVLANVETKDFDLMSTCGTFSKGSKLEVSPSKALSSLQGFMEGYTQGLESSSDTKQQEQGKIF
RQALMLLASPNGIALTTPENIILQASQDIAESASGSINLSAQKNIIGHAQDKISLFAAQKGLRAYAAKGKLE
LQAQDDAIEAIAKKVIKLISTEEKIELTSPKEIVLTAGGSQLKINANGVFSTTGGKFESKAGQHLFMSGATV
NAELPKMP------ETGMYSMRFDLSQIF-DTKILKNMEYKLIN-HSKKIEAVYEFEQESSARVYSDSV-DN
VELALVPGVYLTEIKELISEQEAESLDDEDIDSCGCGEEHEHD-----------------------------
------------------------------------------------------------------------
-----------------------
>A1296_8
-MLFNIFSVLEKIGLNAQKRAIHVQFSNELLNHQVFLQRIEGQHQLNGGLMAELICLSTNAQIALKQFIGVQ
VAVDQVTDSGQLFRTTGIVTEASYGQSDGALTLYKLTIEDATNLWHKRRNSRVFMNKSIVEITEVLFKEWQE
KSPLFAASLSLDLGGLS-QNYDIRPFTMQHNESDYDFLTRLWRSEGGSWLIDESELFVPHFTAPIQAQKLKL
IDDNNQYQALARRSIRYHRSSATEYQDSITGFVAVRSLQPTAVHVQRWQPDALAQEEGNGSVVTTHTHSDNF
DSATLSLEQAWHVSPAWMQDLKGEDQATASSSSQLEKLNQHFTDMHASRAKYFKAYSSVRDTQVGYWFNLRE
HPEIDQ-HEGADQEFLIIAKNFYNQNNLPKDLQQQVSQLLTQSRWDQHGYD-----DIERQSNELTLIRRQI
KTAPEYNPEQHRPIAYPQRAKVVGPEGETIHVDEWGRIKVRFLFTRSDDHGHDGGAGSNDNDTDSAWVDVLT
PWAGEGYGARFLPRIGEVVVIDFFDGNIDRPFVTGRIHEAQRSPTKFDVKGQLPATKKLSGIRSQEINGSGF
NQLRFDDTTGQISTQLQSSHAATQLNLGNLSHPKEQEMSQGRGEGFELRTDAWGAVRAGKGMLISTYAQEQA
IADHLEAAQAQSLLSQGYESMKMLSEVAAKQQTDALNVINRLPKFIQSLELKTTGQALNSTVNLFKEGINND
PIHALKDCGGFIQDIGALGGNAKGVVDEFNSFFSDAKDAVENLKAFIENVEEHGPDIVKGKLASIKDRIHKN
PFESIQEVGKVLANVETKDFDLMSTCGTFSKGSKLEVSPSKALSSLQGFMEGYTQGLESSSDTKQQEQGKIF
RQALMLLASPNGIALTTPENIILQASQDIAESASGSINLSAQKNIIGHAQDKISLFAAQKGLRAYAAKGKLE
LQAQDDAIEAIAKKVIKLISTEDKIELTSPKEIVLTAGGSQLKINANGVFSTTGGKFESKAGQHLFTGGAKV
SMKLPHLP----VIQEGGFSQLFDLKDLSKDNPNLIGAPYKILLKKTGDILMVGTINDDLQTERVFTKE---
----------EEEIELILFETNYTDFNIENLDYLFGEE----------------------------------
------------------------------------------------------------------------
-----------------------
>D1279779_8
-MLFNIFSVLEKIGLNAQKRAIHVQFSNELLNPQVFLQRIEGQHQLNGGLMAELICLSTNAQIALKQFIGVQ

```
VAVDQVTDSGQLFRTTGIVTEASYGQSDGALTLYKLTIEDATNLWHKRRNSRVFMNKSIVEITEVLFKEWQE
KSPLFAASLSLDLGGLS-QNYDIRPFTMQHNESDYDFLTRLWRSEGVSWLIDESELFVPHFTAPIQAQKLRL
IDDNNQYQALARRSIRYHRSSATEYQDSITGFVAVRSLQPTAVHVQRWQPDALAQEEGNGSVVTTHTHSDNF
DSATLSLEQAWHVSPAWMQDLKGEDQATASSSSQLEKLNQHFTDMHASRAKYFKAYSSVRDTQVGYWFNLRE
HPEIDQ-HEGADQEFLIIAKNFYNQNNLPKDLHQQVSQLLTQSRWDQHGYD-----DIERQGNELTLIRRQI
KTAPEYNPEQHRPIAYPQRAKVVGPEGETIHVDEWGRIKVRFLFTRSDDHGHDGGAGSNDNDTDSAWVDVLT
PWAGEGYGARFLPRIGEVVVIDFFDGNIDRPFVTGRIHEAQRSPTKFDVKGQLPDTKKLSGIRSQEINGSGF
NQLRFDDTTGQISTQLQSSHAATQLNLGNLSHPKEQATSQGRGEGFELRTDAWGAVRAGKGMLISTYAQEQA
IADHLEAAQAQSLLSQGYESMKMLSEVAAKQQTDALNVINRLPKFIQSLELKTTGQALNSTVNLFKEGINND
PIHALKDCGGFIQDIGALGGNAKGVVDEFNSFFSDAKDAVENLKAFIENVEEHGPDIVKGKLASIKDRIHKN
PFESIQEVGKVLANVETKDFDLMSTCGTFSKGSKLEVSPSKALSSLQGFMEGYTQGLESSSDTKQQEQGKIF
RQALMLLASPNGIALTTPENIILQASQDIAESASGSINLSAQKNIIGHAQDKISLFAAQKGLRAYAAKGKLE
LQAQDDAIEAIAKKVIKLISTEEKIELTSPKEIVLTAGGSQLKINANGVFSTTGGKFESKAGQHLFTGGAKV
SMKLPHLP----VIQEGGFSQLFDLKDLSKDNPNLIGTPYKILLKKTGDILMVGTINDDLQTERVFTKE---
----------EEEIELILFETNYTDFNIENLDYLFGEE--------------------------------
----------------------------------------------------------------------
----------------------
>R2090_8
-MLFNIFSVLEKIGLNAQKRAIHVQFSNELLNPQVFLQRIEGQHQLNGGLMAELICLSTNAQIALKQFIGVQ
VAVDQVTDSGQLFRTTGIVTEASYGQSDGALTLYKLTIEDATNLWHKRRNSRVFMNKSIVEITEVLFKEWQE
KSPLFAASLSLDLGGLS-QNYDIRPFTMQHNESDYDFLTRLWRSEGVSWLIDESELFVPHFTAPIQAQKLRL
IDDNNQYQALARRSIRYHRSSATEYQDSITGFVAVRSLQPTAVHVQRWQPDALAQEEGNGSVVTTHTHSDNF
DSATLSLEQAWHVSPAWMQDLKGEDQATASSSSQLEKLNQHFTDMHASRAKYFKAYSSVRDTQVGYWFNLRE
HPEIDQ-HEGADQEFLIIAKNFYNQNNLPKDLHQQVSQLLTQSRWDQHGYD-----DIERQGNELTLIRRQI
KTAPEYNPEQHRPIAYPQRAKVVGPEGETIHVDEWGRIKVRFLFTRSDDHGHDGGAGSNDNDTDSAWVDVLT
PWAGEGYGARFLPRIGEVVVIDFFDGNIDRPFVTGRIHEAQRSPTKFDVKGQLPDTKKLSGIRSQEINGSGF
NQLRFDDTTGQISTQLQSSHAATQLNLGNLSHPKEQATSQGRGEGFELRTDAWGAVRAGKGMLISTYAQEQA
IADHLEAAQAQSLLSQGYESMKMLSEVAAKQQTDALNVINRLPKFIQSLELKTTGQALNSTVNLFKEGINND
PIHALKDCGGFIQDIGALGGNAKGVVDEFNSFFSDAKDAVENLKAFIENVEEHGPDIVKGKLASIKDRIHKN
PFESIQEVGKVLANVETKDFDLMSTCGTFSKGSKLEVSPSKALSSLQGFMEGYTQGLESSSDTKQQEQGKIF
RQALMLLASPNGIALTTPENIILQASQDIAESASGSINLSAQKNIIGHAQDKISLFAAQKGLRAYAAKGKLE
LQAQDDAIEAIAKKVIKLISTEEKIELTSPKEIVLTAGGSQLKINANGVFSTTGGKFESKAGQHLFTGGAKV
SMKLPHLP----VIQEGGFSQLFDLKDLSKDNPNLIGTPYKILLKKTGDILMVGTINDDLQTERVFTKE---
```

----------EEEIELILFETNYTDFNIENLDYLFGEE--------------------------------
----------------------------------------------------------------------
----------------------
>1656-2_8
-MLFNIFSVLEKIGLNAQKRAIHVQFSNELLNHQVFLQRIEGQHQLNGGLMAELICLSTNAQIALKQFIGVQ
VAVDQVTDSGQLFRTTGIVTEASYGQSDGALTLYKLTIEDATNLWHKRRNSRVFMNKSIVEITEVLFKEWQE
KSPLFAASLSLDLGGLS-QNYDIRPFTMQHNESDYDFLTRLWRSEGVSWLIDESELFVPHFTAPIQAQKLKL
IDDNNQYQALARRSIRYHRSSATEYQDSITGFVAVRSLQPTAVHVQRWQPDALAQEEGNGSVVTTHTHSDNF
DSATLSLEQAWHVSPAWMQDLKGEDQATASSSSQLEKLNQHFTDMHASRAKYFKAYSSVRDTQVGYWFNLRE
HPEIDQ-HEGADQEFLIIAKNFYNQNNLPKDLHQQVSQLLTQSRWDQHGYD-----EIERQGNELTLIRRQI
KTAPEYNPEQHRPIAYPQRAKVVGPEGETIHVDEWGRIKVRFLFTRSDDHGHDGGAGSNDNDTDSAWVDVLT
PWAGEGYGARFLPRIGEVVVIDFFDGNIDRPFVTGRIHEAQRSPTKFDVKGQLPDTKKLSGIRSQEVNGSGF
NQLRFDDTTGQISTQLQSSHAATQLNLGNLSHPKEQATSQGRGEGFELRTDAWGAVRAGKGMLISTYAQEQA
IADHLEAAQAQSLLSQGYESMKMLSEVAAKQQTDALNVINRLPKFIQSLELKTTGQALNSTVNLFKEGINND
PIHALKDCGGFIQDIGALGGNAKGVVDEFNSFFSDAKDAVENLKAFIENVEEHGPDIVKGKLASIKDRIHKN
PFESIQEVGKVLANVETKDFDLMSTCGTFSKGSKLEVSPSKALSSLQGFMEGYTQGLESSSDTKQQEQGKIF
RQALMLLASPNGIALTTPENIILQASQDIAESASGSINLSAQKNIIGHAQDKISLFAAQKGLRAYAAKGKLE
LQAQDDAIEAIAKKVIKLISTEDKIELTSPKEIVLTAGGSQLKINANGVFSTTGGKFESKAGQHLFTGGAKV
SMKLPHLP----VIQEGGFSQLFDLKDLSKDNPNLIGAPYKILLKKTGDILMVGTINDDLQTERVFTKE---
----------EEEIELILFETNYTDFNIENLDYLFGEE--------------------------------
----------------------------------------------------------------------
----------------------
>AbPK1_8
-MLFNIFSVLEKIGLNAQKRAIHVQFSNELLNHQVFLQRIEGQHQLNGGLMAELICLSTNAQIALKQFIGVQ
VAVDQVTDSGQLFRTTGIVTEASYGQSDGALTLYKLTIEDATNLWHKRRNSRVFMNKSIVEITEVLFKEWQE
KSPLFAASLSLDLGGLS-QNYDIRPFTMQHNESDYDFLTRLWRSEGVSWLIDESELFVPHFTAPIQAQKLKL
IDDNNQYQALARRSIRYHRSSATEYQDSITGFVAVRSLQPTAVHVQRWQPDALAQEEGNGSVVTTHTHSDNF
DSATLSLEQAWHVSPAWMQDLKGEDQATASSSSQLEKLNQHFTDMHASRAKYFKAYSSVRDTQVGYWFNLRE
HPEIDQ-HEGADQEFLIIAKNFYNQNNLPKDLHQQVSQLLTQSRWDQHGYD-----EIERQGNELTLIRRQI
KTAPEYNPEQHRPIAYPQRAKVVGPEGETIHVDEWGRIKVRFLFTRSDDHGHDGGAGSNDNDTDSAWVDVLT
PWAGEGYGARFLPRIGEVVVIDFFDGNIDRPFVTGRIHEAQRSPTKFDVKGQLPDTKKLSGIRSQEVNGSGF
NQLRFDDTTGQISTQLQSSHAATQLNLGNLSHPKEQATSQGRGEGFELRTDAWGAVRAGKGMLISTYAQEQA
IADHLEAAQAQSLLSQGYESMKMLSEVAAKQQTDALNVINRLPKFIQSLELKTTGQALNSTVNLFKEGINND

PIHALKDCGGFIQDIGALGGNAKGVVDEFNSFFSDAKDAVENLKAFIENVEEHGPDIVKGKLASIKDRIHKN
PFESIQEVGKVLANVETKDFDLMSTCGTFSKGSKLEVSPSKALSSLQGFMEGYTQGLESSSDTKQQEQGKIF
RQALMLLASPNGIALTTPENIILQASQDIAESASGSINLSAQKNIIGHAQDKISLFAAQKGLRAYAAKGKLE
LQAQDDAIEAIAKKVIKLISTEDKIELTSPKEIVLTAGGSQLKINANGVFSTTGGKFESKAGQHLFTGGAKV
SMKLPHLP----VIQEGGFSQLFDLKDLSKDNPNLIGAPYKILLKKTGDILMVGTINDDLQTERVFTKE---
----------EEEIELILFETNYTDFNIENLDYLFGEE----------------------------------
------------------------------------------------------------------------
-----------------------

>ACICU_8
-MLFNIFSVLEKIGLNAQKRAIHVQFSNELLNHQVFLQRIEGQHQLNGGLMAELICLSTNAQIALKQFIGVQ
VAVDQVTDSGQLFRTTGIVTEASYGQSDGALTLYKLTIEDATNLWHKRRNSRVFMNKSIVEITEVLFKEWQE
KSPLFAASLSLDLGGLS-QNYDIRPFTMQHNESDYDFLTRLWRSEGVSWLIDESELFVPHFTAPIQAQKLKL
IDDNNQYQALARRSIRYHRSSATEYQDSITGFVAVRSLQPTAVHVQRWQPDALAQEEGNGSVVTTHTHSDNF
DSATLSLEQAWHVSPAWMQDLKGEDQATASSSSQLEKLNQHFTDMHASRAKYFKAYSSVRDTQVGYWFNLRE
HPEIDQ-HEGADQEFLIIAKNFYNQNNLPKDLHQQVSQLLTQSRWDQHGYD-----EIERQGNELTLIRRQI
KTAPEYNPEQHRPIAYPQRAKVVGPEGETIHVDEWGRIKVRFLFTRSDDHGHDGGAGSNDNDTDSAWVDVLT
PWAGEGYGARFLPRIGEVVVIDFFDGNIDRPFVTGRIHEAQRSPTKFDVKGQLPDTKKLSGIRSQEVNGSGF
NQLRFDDTTGQISTQLQSSHAATQLNLGNLSHPKEQATSQGRGEGFELRTDAWGAVRAGKGMLISTYAQEQA
IADHLEAAQAQSLLSQGYESMKMLSEVAAKQQTDALNVINRLPKFIQSLELKTTGQALNSTVNLFKEGINND
PIHALKDCGGFIQDIGALGGNAKGVVDEFNSFFSDAKDAVENLKAFIENVEEHGPDIVKGKLASIKDRIHKN
PFESIQEVGKVLANVETKDFDLMSTCGTFSKGSKLEVSPSKALSSLQGFMEGYTQGLESSSDTKQQEQGKIF
RQALMLLASPNGIALTTPENIILQASQDIAESASGSINLSAQKNIIGHAQDKISLFAAQKGLRAYAAKGKLE
LQAQDDAIEAIAKKVIKLISTEDKIELTSPKEIVLTAGGSQLKINANGVFSTTGGKFESKAGQHLFTGGAKV
SMKLPHLP----VIQEGGFSQLFDLKDLSKDNPNLIGAPYKILLKKTGDILMVGTINDDLQTERVFTKE---
----------EEEIELILFETNYTDFNIENLDYLFGEE----------------------------------
------------------------------------------------------------------------
-----------------------

>AR_0056_8
-MLFNIFSVLEKIGLNAQKRAIHVQFSNELLNHQVFLQRIEGQHQLNGGLMAELICLSTNAQIALKQFIGVQ
VAVDQVTDSGQLFRTTGIVTEASYGQSDGALTLYKLTIEDATNLWHKRRNSRVFMNKSIVEITEVLFKEWQE
KSPLFAASLSLDLGGLS-QNYDIRPFTMQHNESDYDFLTRLWRSEGVSWLIDESELFVPHFTAPIQAQKLKL
IDDNNQYQALARRSIRYHRSSATEYQDSITGFVAVRSLQPTAVHVQRWQPDALAQEEGNGSVVTTHTHSDNF
DSATLSLEQAWHVSPAWMQDLKGEDQATASSSSQLEKLNQHFTDMHASRAKYFKAYSSVRDTQVGYWFNLRE

```
HPEIDQ-HEGADQEFLIIAKNFYNQNNLPKDLHQQVSQLLTQSRWDQHGYD-----EIERQGNELTLIRRQI
KTAPEYNPEQHRPIAYPQRAKVVGPEGETIHVDEWGRIKVRFLFTRSDDHGHDGGAGSNDNDTDSAWVDVLT
PWAGEGYGARFLPRIGEVVVIDFFDGNIDRPFVTGRIHEAQRSPTKFDVKGQLPDTKKLSGIRSQEVNGSGF
NQLRFDDTTGQISTQLQSSHAATQLNLGNLSHPKEQATSQGRGEGFELRTDAWGAVRAGKGMLISTYAQEQA
IADHLEAAQAQSLLSQGYESMKMLSEVAAKQQTDALNVINRLPKFIQSLELKTTGQALNSTVNLFKEGINND
PIHALKDCGGFIQDIGALGGNAKGVVDEFNSFFSDAKDAVENLKAFIENVEEHGPDIVKGKLASIKDRIHKN
PFESIQEVGKVLANVETKDFDLMSTCGTFSKGSKLEVSPSKALSSLQGFMEGYTQGLESSSDTKQQEQGKIF
RQALMLLASPNGIALTTPENIILQASQDIAESASGSINLSAQKNIIGHAQDKISLFAAQKGLRAYAAKGKLE
LQAQDDAIEAIAKKVIKLISTEDKIELTSPKEIVLTAGGSQLKINANGVFSTTGGKFESKAGQHLFTGGAKV
SMKLPHLP----VIQEGGFSQLFDLKDLSKDNPNLIGAPYKILLKKTGDILMVGTINDDLQTERVFTKE---
----------EEEIELILFETNYTDFNIENLDYLFGEE--------------------------------
----------------------------------------------------------------------
----------------------
>DU202_8
-MLFNIFSVLEKIGLNAQKRAIHVQFSNELLNHQVFLQRIEGQHQLNGGLMAELICLSTNAQIALKQFIGVQ
VAVDQVTDSGQLFRTTGIVTEASYGQSDGALTLYKLTIEDATNLWHKRRNSRVFMNKSIVEITEVLFKEWQE
KSPLFAASLSLDLGGLS-QNYDIRPFTMQHNESDYDFLTRLWRSEGVSWLIDESELFVPHFTAPIQAQKLKL
IDDNNQYQALARRSIRYHRSSATEYQDSITGFVAVRSLQPTAVHVQRWQPDALAQEEGNGSVVTTHTHSDNF
DSATLSLEQAWHVSPAWMQDLKGEDQATASSSSQLEKLNQHFTDMHASRAKYFKAYSSVRDTQVGYWFNLRE
HPEIDQ-HEGADQEFLIIAKNFYNQNNLPKDLHQQVSQLLTQSRWDQHGYD-----EIERQGNELTLIRRQI
KTAPEYNPEQHRPIAYPQRAKVVGPEGETIHVDEWGRIKVRFLFTRSDDHGHDGGAGSNDNDTDSAWVDVLT
PWAGEGYGARFLPRIGEVVVIDFFDGNIDRPFVTGRIHEAQRSPTKFDVKGQLPDTKKLSGIRSQEVNGSGF
NQLRFDDTTGQISTQLQSSHAATQLNLGNLSHPKEQATSQGRGEGFELRTDAWGAVRAGKGMLISTYAQEQA
IADHLEAAQAQSLLSQGYESMKMLSEVAAKQQTDALNVINRLPKFIQSLELKTTGQALNSTVNLFKEGINND
PIHALKDCGGFIQDIGALGGNAKGVVDEFNSFFSDAKDAVENLKAFIENVEEHGPDIVKGKLASIKDRIHKN
PFESIQEVGKVLANVETKDFDLMSTCGTFSKGSKLEVSPSKALSSLQGFMEGYTQGLESSSDTKQQEQGKIF
RQALMLLASPNGIALTTPENIILQASQDIAESASGSINLSAQKNIIGHAQDKISLFAAQKGLRAYAAKGKLE
LQAQDDAIEAIAKKVIKLISTEDKIELTSPKEIVLTAGGSQLKINANGVFSTTGGKFESKAGQHLFTGGAKV
SMKLPHLP----VIQEGGFSQLFDLKDLSKDNPNLIGAPYKILLKKTGDILMVGTINDDLQTERVFTKE---
----------EEEIELILFETNYTDFNIENLDYLFGEE--------------------------------
----------------------------------------------------------------------
----------------------
>KAB03_8
```

-MLFNIFSVLEKIGLNAQKRAIHVQFSNELLNHQVFLQRIEGQHQLNGGLMAELICLSTNAQIALKQFIGVQ
VAVDQVTDSGQLFRTTGIVTEASYGQSDGALTLYKLTIEDATNLWHKRRNSRVFMNKSIVEITEVLFKEWQE
KSPLFAASLSLDLGGLS-QNYDIRPFTMQHNESDYDFLTRLWRSEGVSWLIDESELFVPHFTAPIQAQKLKL
IDDNNQYQALARRSIRYHRSSATEYQDSITGFVAVRSLQPTAVHVQRWQPDALAQEEGNGSVVTTHTHSDNF
DSATLSLEQAWHVSPAWMQDLKGEDQATASSSSQLEKLNQHFTDMHASRAKYFKAYSSVRDTQVGYWFNLRE
HPEIDQ-HEGADQEFLIIAKNFYNQNNLPKDLHQQVSQLLTQSRWDQHGYD-----EIERQGNELTLIRRQI
KTAPEYNPEQHRPIAYPQRAKVVGPEGETIHVDEWGRIKVRFLFTRSDDHGHDGGAGSNDNDTDSAWVDVLT
PWAGEGYGARFLPRIGEVVVIDFFDGNIDRPFVTGRIHEAQRSPTKFDVKGQLPDTKKLSGIRSQEVNGSGF
NQLRFDDTTGQISTQLQSSHAATQLNLGNLSHPKEQATSQGRGEGFELRTDAWGAVRAGKGMLISTYAQEQA
IADHLEAAQAQSLLSQGYESMKMLSEVAAKQQTDALNVINRLPKFIQSLELKTTGQALNSTVNLFKEGINND
PIHALKDCGGFIQDIGALGGNAKGVVDEFNSFFSDAKDAVENLKAFIENVEEHGPDIVKGKLASIKDRIHKN
PFESIQEVGKVLANVETKDFDLMSTCGTFSKGSKLEVSPSKALSSLQGFMEGYTQGLESSSDTKQQEQGKIF
RQALMLLASPNGIALTTPENIILQASQDIAESASGSINLSAQKNIIGHAQDKISLFAAQKGLRAYAAKGKLE
LQAQDDAIEAIAKKVIKLISTEDKIELTSPKEIVLTAGGSQLKINANGVFSTTGGKFESKAGQHLFTGGAKV
SMKLPHLP----VIQEGGFSQLFDLKDLSKDNPNLIGAPYKILLKKTGDILMVGTINDDLQTERVFTKE---
----------EEEIELILFETNYTDFNIENLDYLFGEE----------------------------------
------------------------------------------------------------------------
-----------------------
>NCGM237_8
-MLFNIFSVLEKIGLNAQKRAIHVQFSNELLNHQVFLQRIEGQHQLNGGLMAELICLSTNAQIALKQFIGVQ
VAVDQVTDSGQLFRTTGIVTEASYGQSDGALTLYKLTIEDATNLWHKRRNSRVFMNKSIVEITEVLFKEWQE
KSPLFAASLSLDLGGLS-QNYDIRPFTMQHNESDYDFLTRLWRSEGVSWLIDESELFVPHFTAPIQAQKLKL
IDDNNQYQALARRSIRYHRSSATEYQDSITGFVAVRSLQPTAVHVQRWQPDALAQEEGNGSVVTTHTHSDNF
DSATLSLEQAWHVSPAWMQDLKGEDQATASSSSQLEKLNQHFTDMHASRAKYFKAYSSVRDTQVGYWFNLRE
HPEIDQ-HEGADQEFLIIAKNFYNQNNLPKDLHQQVSQLLTQSRWDQHGYD-----EIERQGNELTLIRRQI
KTAPEYNPEQHRPIAYPQRAKVVGPEGETIHVDEWGRIKVRFLFTRSDDHGHDGGAGSNDNDTDSAWVDVLT
PWAGEGYGARFLPRIGEVVVIDFFDGNIDRPFVTGRIHEAQRSPTKFDVKGQLPDTKKLSGIRSQEVNGSGF
NQLRFDDTTGQISTQLQSSHAATQLNLGNLSHPKEQATSQGRGEGFELRTDAWGAVRAGKGMLISTYAQEQA
IADHLEAAQAQSLLSQGYESMKMLSEVAAKQQTDALNVINRLPKFIQSLELKTTGQALNSTVNLFKEGINND
PIHALKDCGGFIQDIGALGGNAKGVVDEFNSFFSDAKDAVENLKAFIENVEEHGPDIVKGKLASIKDRIHKN
PFESIQEVGKVLANVETKDFDLMSTCGTFSKGSKLEVSPSKALSSLQGFMEGYTQGLESSSDTKQQEQGKIF
RQALMLLASPNGIALTTPENIILQASQDIAESASGSINLSAQKNIIGHAQDKISLFAAQKGLRAYAAKGKLE
LQAQDDAIEAIAKKVIKLISTEDKIELTSPKEIVLTAGGSQLKINANGVFSTTGGKFESKAGQHLFTGGAKV

SMKLPHLP----VIQEGGFSQLFDLKDLSKDNPNLIGAPYKILLKKTGDILMVGTINDDLQTERVFTKE---
----------EEEIELILFETNYTYFNIENLDYLFGEE----------------------------------
------------------------------------------------------------------------
-----------------------
>15A34_23
-MLFNIFSVLEKIGLNAQKRAIHVQFSNELLNPQVFLQRIEGQHQLNGGLMAELICLSTNAQIALKQFIGVQ
VAVDQVTDSGQLFRTTGIVTEASYGQSDGALTLYKLTIEDATNLWHKRRNSRVFMNKSIVEITEVLFKEWQE
KSPLFAASLSLDLGGLS-QNYDIRPFTMQHNESDYDFLTRLWRSEGVSWLIDESELFVPHFTAPIQAQKLKL
IDDNNQYQALARRSIRYHRSSATEYQDSITGFVAVRSLQPTAVHVQRWQPDALAQEEGNGSVVTTHTHSDNF
DSATLSLEQAWHVSPAWMQDLKGEDQATASGSSQLEKLNQHFTDMHASRAKYFKAYSSVRDSQVGYWFNLRE
HPEIDQ-HEGADQEFLIIAKNFYNQNNLPKDLHQQVSQLLTQSRWDQHGYD-----DIERQGNELTLIRRQI
KTAPEYNPEQHRPIAYPQRAKVVGPQGETIHVDEWGRIKVRFLFTRSDDHGHDGGAGSNDNDTDSAWVDVLT
PWAGEGYGARFLPRIGEVVVIDFFDGNIDRPFVTGRIHEAQRSPTKFDVKGQLPATKKLSGIRSQEINGSGF
NQLRFDDTTGQISTQLQSSHAATQLNLGNLSHPKEQATSQGRGEGFELRTDAWGAVRAGKGMLISTYAQEQA
IADHLEAAQAQSLLSQGYESMKMLSKVAAKQQTDALNVINRLPKFIQSLELKTTGQALNSTVNLFKEGINND
PIHALKDCGGFIQDIGALGGNAKGVVDEFNSFFSDAKDAVENLKAFIENVEEHGPDIVKGKLASIKDRIHKN
PFESIQEVGKVLANVETKDFDLMSTCGTFSKGSKLEVSPSKALSSLQGFMEGYTQGLESSSDTKQQEQGKIF
RQALMLLASPNGIALTTPENIILQASQDIAESASGSINLSAQKNIIGHAQDKISLFAAQKGLRAYAAKGKLE
LQAQDDAIEAIAKKVIKLISTEDKIELTSPKEIVLTAGGSQLKINANGVFSTTGGKFESKAGQHLFTSGAKV
SYEVPQLP------STMMYSNKLDVYDLFWDFDL-SRLSYVAK--FKNGRVSTGSLDENGRTARISSDS--S
EPAEVFVDTNTDWVVEIEEEVSQVESQNQDIK----------------------------------------
------------------------------------------------------------------------
-----------------------
>6200_17
-MLFNIFSVLEKIGLNAQKRAIHVQFSNELLNHQVFLQRIEGQHQLNGGLMAELICLSTNAQIALKQFIGVQ
VAVDQVTDSGQLFRTTGIVTEASYGQSDGALTLYKLTIEDATNLWHKRRNSRVFMNKSIVEITEVLFKEWQE
KSPLFAASLSLDLGGLS-QNYDIRPFTMQHNESDYDFLTRLWRSEGVSWLIDESELLVPHFTAPIQAQKLRL
IDDNNQYQALARRSIRYHRSSATEYQDSITGFVAVRSLQPTAVHVQRWQPDALAQEEGNGSVVTTHTHSDNF
DSATLSLEQAWHVSPAWMQDLKGEDQATASSSSQLEKLNQHFTDMHASRAKYFKAYSSVRDTQVGYWFNLRE
HPEIDQ-HEGADQEFLIIAKNFYNQNNLPKDLHQQVSQLLTQSRWDQHGYD-----DIERQSNELTLIRRQI
KTAPEYNPEQHRPIAYPQRAKVVGPEGETIHVDEWGRIKVRFLFTRSDDHGHDGGAGSNDNDTDSAWVDVLT
PWAGEGYGARFLPRIGEVVVIDFFDGNIDRPFVTGRIHEAQRSPTKFDVKGQLPDTKKLSGIRSQEINGSGF
NQLRFDDTTGQISTQLQSSHAATQLNLGNLSHPKEQATSQGRGEGFELRTDAWGAVRAGKGMLISTYAQEQA

```
IADHLEAAQAQSLLSQGYESMKMLSEVAAKQQTDALNVINRLPKFIQSLELKTTGQALNSTVNLFKEGINND
PIHALKDCGGFIQDIGALGGNAKGVVDEFNSFFSDAKDAVENLKAFIENVEEHGPDIVKGKLASIKDRIHKN
PFESIQEVGKVLANVETKDFDLMSTCGTFSKGSKLEVSPSKALSSLQGFMEGYTQGLESSSDTKQQEQGKIF
RQALMLLASPNGIALTTPENIILQASQDIAESASGSINLSAQKNIIGHAQDKISLFAAQKGLRAYAAKGKLE
LQAQDDAIEAIAKKVIKLISTEEKIELTSPKEIILTAGGSQLKINANGVFSTTGGKFESKAGQHLFTSGAKV
SYEVPELP------SSFDYSNRIDLYKIFQWSDF-ENIKYSAI--FENQRVTQGNLDELGRTERFKSSTPDK
MKVLVGYSADKWHKETEEFEDFDDYETNNDSNLNNNSN----------------------------------
------------------------------------------------------------------------
-----------------------
>ZW85-1_17
-MLFNIFSVLEKIGLNAQKRAIHVQFSNELLNHQVFLQRIEGQHQLNGGLMAELICLSTNAQIALKQFIGVQ
VAVDQVTDSGQLFRTTGIVTEASYGQSDGALTLYKLTIEDATNLWHKRRNSRVFMNKSIVEITEVLFKEWQE
KSPLFAASLSLDLDGLS-QNYDIRPFTMQHNESDYDFLTRLWRSEGVSWLIDESELFVPHFTAPIQAQKLRL
IDDNNQYQALARRSIRYHRSSATEYQDSITGFVAVRSLQPTAVHVQRWQPDALAQEEGNGSVVTTHTHSDNF
DSATLSLEQAWHVSPAWMQDLKGEDQATASSSSQLEKLNQHFTDMHASRAKYFKAYSSVRDTQVGYWFNLRE
HPEIDQ-HEGADQEFLIIAKNFYNQNNLPKDLHQQVSQLLTQSRWDQHGYD-----DIERQSNELTLIRRQI
KTAPEYNPEQHRPIAYPQRAKVVGPEGETIHVDEWGRIKVRFLFTRSDDHGHDGGAGSNDNDTDSAWVDVLT
PWAGEGYGARFLPRIGEVVVIDFFDGNIDRPFVTGRIHEAQRSPTKFDVKGQLPDTKKLSGIRSQEVNGSGF
NQLRFDDTTGQISTQLQSSHAATQLNLGNLSHPKEQATSQGRGEGFELRTDAWGAVRAGKGMLISTYAQEQA
IADHLEAAQAQSLLSQGYESMKMLSEVAAKQQTDALNVINRLPKFIQSLELKTTGQALNSTVNLFKEGINND
PIHALKDCGGFIQDIGALGENAKGVVDEFNSFFSDAKDAVENLKAFIENVEEHGPDIVKGKLASIKDRIHKN
PFESIQEVGRVLANVETKDFDLMSTCGTFSKGSKLEVSPSKALSSLQGFMEGYTQGLESSSDTKQQEQGKIF
RQALMLLASPNGIALTTPENIILQASQDIAESASGSINLSAQKNIIGHAQDKISLFAAQKGLRAYAAKGKLE
LQAQDDAIEAIAKKVIKLISTEEKIELTSPKEIVLTAGGSQLKINANGVFSTTGGKFESKAGQHLFTGGAKV
SYEVPELP------SSFDYSNRIDLYKIFQWSDF-ENIKYSAI--FENQRVTQGNLDELGRTERFKSSTPDK
MKVLVGYSADKWHKETEEFEDFDDYETNNDSNLNNNSN----------------------------------
------------------------------------------------------------------------
-----------------------
>A1_17
-MLFNIFSVLEKIGLNAQKRAIHVQFSNELLNHQVFLQRIEGQHQLNGGLMAELICLSTNAQIALKQFIGVQ
VAVDQVTDSGQLFRTTGIVTEASYGQSDGALTLYKLTIEDATNLWHKRRNSRVFMNKSIVEITEVLFKEWQE
KSPLFAASLSLDLGGLS-QNYDIRPFTMQHNESDYDFLTRLWRSEGVSWLIDESELFVPHFTAPIQAQKLRL
IDDNNQYQALARRSIRYHRSSATEYQDSITGFVAVRSLQPTAVHVQRWQPDALAQEEGNGSVVTTHTHSDNF
```

DSATLSLEQAWHVSPAWMQDLKGEDQATASSSSQLEKLNQHFTDMHASRAKYFKAYSSVRDTQVGYWFNLRE
HPEIDQ-HEGADQEFLIIAKNFYNQNNLPKDLHQQVSQLLTQSRWDQHGYD-----DIERQSNELTLIRRQI
KTAPEYNPEQHRPIAYPQRAKVVGPEGETIHVDEWGRIKVRFLFTRSDDHGHDGGAGSNDNDTDSAWVDVLT
PWAGEGYGARFLPRIGEVVVIDFFDGNIDRPFVTGRIHEAQRSPTKFDVKGQLPDTKKLSGIRSQEVNGSGF
NQLRFDDTTGQISTQLQSSHAATQLNLGNLSHPKEQATSQGRGEGFELRTDAWGAVRAGKGMLISTYAQEQA
IADHLEAAQAQSLLSQGYESMKMLSEVAAKQQTDALNVINRLPKFIQSLELKTTGQALNSTVNLFKEGINND
PIHALKDCGGFIQDIGALGGNAKGVVDEFNSFFSDAKDAVENLKAFIENVEEHGPDIVKGKLASIKDRIHKN
PFESIQEVGRVLANVETKDFDLMSTCGTFSKGSKLEVSPSKALSSLQGFMEGYTQGLESSSDTKQQEQGKIF
RQALMLLASPNGIALTTPENIILQASQDIAESASGSINLSAQKNIIGHAQDKISLFAAQKGLRAYAAKGKLE
LQAQDDAIEAIAKKVIKLISTEEKIELTSPKEIVLTAGGSQLKINANGVFSTTGGKFESKAGQHLFTSGAKV
SYEVPELP------SSFDYSNRIDLYKIFQWSDF-ENIKYSAI--FENQRVTQGNLDELGRTERFKSSMPDK
MKVLVGYSADKWHKETEEFEDFDDYETNNDSNLNNNSN----------------------------------
------------------------------------------------------------------------
-----------------------

>A388_17
-MLFNIFSVLEKIGLNAQKRAIHVQFSNELLNHQVFLQRIEGQHQLNGGLMAELICLSTNAQIALKQFIGVQ
VAVDQVTDSGQLFRTTGIVTEASYGQSDGALTLYKLTIEDATNLWHKRRNSRVFMNKSIVEITEVLFKEWQE
KSPLFAASLSLDLGGLS-QNYDIRPFTMQHNESDYDFLTRLWRSEGVSWLIDESELFVPHFTAPIQAQKLRL
IDDNNQYQALARRSIRYHRSSATEYQDSITGFVAVRSLQPTAVHVQRWQPDALAQEEGNGSVVTTHTHSDNF
DSATLSLEQAWHVSPAWMQDLKGEDQATASSSSQLEKLNQHFTDMHASRAKYFKAYSSVRDTQVGYWFNLRE
HPEIDQ-HEGADQEFLIIAKNFYNQNNLPKDLHQQVSQLLTQSRWDQHGYD-----DIERQSNELTLIRRQI
KTAPEYNPEQHRPIAYPQRAKVVGPEGETIHVDEWGRIKVRFLFTRSDDHGHDGGAGSNDNDTDSAWVDVLT
PWAGEGYGARFLPRIGEVVVIDFFDGNIDRPFVTGRIHEAQRSPTKFDVKGQLPDTKKLSGIRSQEVNGSGF
NQLRFDDTTGQISTQLQSSHAATQLNLGNLSHPKEQATSQGRGEGFELRTDAWGAVRAGKGMLISTYAQEQA
IADHLEAAQAQSLLSQGYESMKMLSEVAAKQQTDALNVINRLPKFIQSLELKTTGQALNSTVNLFKEGINND
PIHALKDCGGFIQDIGALGGNAKGVVDEFNSFFSDAKDAVENLKAFIENVEEHGPDIVKGKLASIKDRIHKN
PFESIQEVGRVLANVETKDFDLMSTCGTFSKGSKLEVSPSKALSSLQGFMEGYTQGLESSSDTKQQEQGKIF
RQALMLLASPNGIALTTPENIILQASQDIAESASGSINLSAQKNIIGHAQDKISLFAAQKGLRAYAAKGKLE
LQAQDDAIEAIAKKVIKLISTEEKIELTSPKEIVLTAGGSQLKINANGVFSTTGGKFESKAGQHLFTSGAKV
SYEVPELP------SSFDYSNRIDLYKIFQWSDF-ENIKYSAI--FENQRVTQGNLDELGRTERFKSSMPDK
MKVLVGYSADKWHKETEEFEDFDDYETNNDSNLNNNSN----------------------------------
------------------------------------------------------------------------
-----------------------

>A85_17
-MLFNIFSVLEKIGLNAQKRAIHVQFSNELLNHQVFLQRIEGQHQLNGGLMAELICLSTNAQIALKQFIGVQ
VAVDQVTDSGQLFRTTGIVTEASYGQSDGALTLYKLTIEDATNLWHKRRNSRVFMNKSIVEITEVLFKEWQE
KSPLFAASLSLDLGGLS-QNYDIRPFTMQHNESDYDFLTRLWRSEGVSWLIDESELFVPHFTAPIQAQKLRL
IDDNNQYQALARRSIRYHRSSATEYQDSITGFVAVRSLQPTAVHVQRWQPDALAQEEGNGSVVTTHTHSDNF
DSATLSLEQAWHVSPAWMQDLKGEDQATASSSSQLEKLNQHFTDMHASRAKYFKAYSSVRDTQVGYWFNLRE
HPEIDQ-HEGADQEFLIIAKNFYNQNNLPKDLHQQVSQLLTQSRWDQHGYD-----DIERQSNELTLIRRQI
KTAPEYNPEQHRPIAYPQRAKVVGPEGETIHVDEWGRIKVRFLFTRSDDHGHDGGAGSNDNDTDSAWVDVLT
PWAGEGYGARFLPRIGEVVVIDFFDGNIDRPFVTGRIHEAQRSPTKFDVKGQLPDTKKLSGIRSQEVNGSGF
NQLRFDDTTGQISTQLQSSHAATQLNLGNLSHPKEQATSQGRGEGFELRTDAWGAVRAGKGMLISTYAQEQA
IADHLEAAQAQSLLSQGYESMKMLSEVAAKQQTDALNVINRLPKFIQSLELKTTGQALNSTVNLFKEGINND
PIHALKDCGGFIQDIGALGGNAKGVVDEFNSFFSDAKDAVENLKAFIENVEEHGPDIVKGKLASIKDRIHKN
PFESIQEVGRVLANVETKDFDLMSTCGTFSKGSKLEVSPSKALSSLQGFMEGYTQGLESSSDTKQQEQGKIF
RQALMLLASPNGIALTTPENIILQASQDIAESASGSINLSAQKNIIGHAQDKISLFAAQKGLRAYAAKGKLE
LQAQDDAIEAIAKKVIKLISTEEKIELTSPKEIVLTAGGSQLKINANGVFSTTGGKFESKAGQHLFTSGAKV
SYEVPELP------SSFDYSNRIDLYKIFQWSDF-ENIKYSAI--FENQRVTQGNLDELGRTERFKSSMPDK
MKVLVGYSADKWHKETEEFEDFDDYETNNDSNLNNNSN----------------------------------
------------------------------------------------------------------------
-----------------------
>AB0057_17
-MLFNIFSVLEKIGLNAQKRAIHVQFSNELLNHQVFLQRIEGQHQLNGGLMAELICLSTNAQIALKQFIGVQ
VAVDQVTDSGQLFRTTGIVTEASYGQSDGALTLYKLTIEDATNLWHKRRNSRVFMNKSIVEITEVLFKEWQE
KSPLFAASLSLDLGGLS-QNYDIRPFTMQHNESDYDFLTRLWRSEGVSWLIDESELFVPHFTAPIQAQKLRL
IDDNNQYQALARRSIRYHRSSATEYQDSITGFVAVRSLQPTAVHVQRWQPDALAQEEGNGSVVTTHTHSDNF
DSATLSLEQAWHVSPAWMQDLKGEDQATASSSSQLEKLNQHFTDMHASRAKYFKAYSSVRDTQVGYWFNLRE
HPEIDQ-HEGADQEFLIIAKNFYNQNNLPKDLHQQVSQLLTQSRWDQHGYD-----DIERQSNELTLIRRQI
KTAPEYNPEQHRPIAYPQRAKVVGPEGETIHVDEWGRIKVRFLFTRSDDHGHDGGAGSNDNDTDSAWVDVLT
PWAGEGYGARFLPRIGEVVVIDFFDGNIDRPFVTGRIHEAQRSPTKFDVKGQLPDTKKLSGIRSQEVNGSGF
NQLRFDDTTGQISTQLQSSHAATQLNLGNLSHPKEQATSQGRGEGFELRTDAWGAVRAGKGMLISTYAQEQA
IADHLEAAQAQSLLSQGYESMKMLSEVAAKQQTDALNVINRLPKFIQSLELKTTGQALNSTVNLFKEGINND
PIHALKDCGGFIQDIGALGGNAKGVVDEFNSFFSDAKDAVENLKAFIENVEEHGPDIVKGKLASIKDRIHKN
PFESIQEVGRVLANVETKDFDLMSTCGTFSKGSKLEVSPSKALSSLQGFMEGYTQGLESSSDTKQQEQGKIF
RQALMLLASPNGIALTTPENIILQASQDIAESASGSINLSAQKNIIGHAQDKISLFAAQKGLRAYAAKGKLE

LQAQDDAIEAIAKKVIKLISTEEKIELTSPKEIVLTAGGSQLKINANGVFSTTGGKFESKAGQHLFTSGAKV
SYEVPELP------SSFDYSNRIDLYKIFQWSDF-ENIKYSAI--FENQRVTQGNLDELGRTERFKSSMPDK
MKVLVGYSADKWHKETEEFEDFDDYETNNDSNLNNNSN----------------------------------
------------------------------------------------------------------------
-----------------------
>AB307-0294_17
-MLFNIFSVLEKIGLNAQKRAIHVQFSNELLNHQVFLQRIEGQHQLNGGLMAELICLSTNAQIALKQFIGVQ
VAVDQVTDSGQLFRTTGIVTEASYGQSDGALTLYKLTIEDATNLWHKRRNSRVFMNKSIVEITEVLFKEWQE
KSPLFAASLSLDLGGLS-QNYDIRPFTMQHNESDYDFLTRLWRSEGVSWLIDESELFVPHFTAPIQAQKLRL
IDDNNQYQALARRSIRYHRSSATEYQDSITGFVAVRSLQPTAVHVQRWQPDALAQEEGNGSVVTTHTHSDNF
DSATLSLEQAWHVSPAWMQDLKGEDQATASSSSQLEKLNQHFTDMHASRAKYFKAYSSVRDTQVGYWFNLRE
HPEIDQ-HEGADQEFLIIAKNFYNQNNLPKDLHQQVSQLLTQSRWDQHGYD-----DIERQSNELTLIRRQI
KTAPEYNPEQHRPIAYPQRAKVVGPEGETIHVDEWGRIKVRFLFTRSDDHGHDGGAGSNDNDTDSAWVDVLT
PWAGEGYGARFLPRIGEVVVIDFFDGNIDRPFVTGRIHEAQRSPTKFDVKGQLPDTKKLSGIRSQEVNGSGF
NQLRFDDTTGQISTQLQSSHAATQLNLGNLSHPKEQATSQGRGEGFELRTDAWGAVRAGKGMLISTYAQEQA
IADHLEAAQAQSLLSQGYESMKMLSEVAAKQQTDALNVINRLPKFIQSLELKTTGQALNSTVNLFKEGINND
PIHALKDCGGFIQDIGALGGNAKGVVDEFNSFFSDAKDAVENLKAFIENVEEHGPDIVKGKLASIKDRIHKN
PFESIQEVGRVLANVETKDFDLMSTCGTFSKGSKLEVSPSKALSSLQGFMEGYTQGLESSSDTKQQEQGKIF
RQALMLLASPNGIALTTPENIILQASQDIAESASGSINLSAQKNIIGHAQDKISLFAAQKGLRAYAAKGKLE
LQAQDDAIEAIAKKVIKLISTEEKIELTSPKEIVLTAGGSQLKINANGVFSTTGGKFESKAGQHLFTSGAKV
SYEVPELP------SSFDYSNRIDLYKIFQWSDF-ENIKYSAI--FENQRVTQGNLDELGRTERFKSSMPDK
MKVLVGYSADKWHKETEEFEDFDDYETNNDSNLNNNSN----------------------------------
------------------------------------------------------------------------
-----------------------
>AB5075-UW_17
-MLFNIFSVLEKIGLNAQKRAIHVQFSNELLNHQVFLQRIEGQHQLNGGLMAELICLSTNAQIALKQFIGVQ
VAVDQVTDSGQLFRTTGIVTEASYGQSDGALTLYKLTIEDATNLWHKRRNSRVFMNKSIVEITEVLFKEWQE
KSPLFAASLSLDLGGLS-QNYDIRPFTMQHNESDYDFLTRLWRSEGVSWLIDESELFVPHFTAPIQAQKLRL
IDDNNQYQALARRSIRYHRSSATEYQDSITGFVAVRSLQPTAVHVQRWQPDALAQEEGNGSVVTTHTHSDNF
DSATLSLEQAWHVSPAWMQDLKGEDQATASSSSQLEKLNQHFTDMHASRAKYFKAYSSVRDTQVGYWFNLRE
HPEIDQ-HEGADQEFLIIAKNFYNQNNLPKDLHQQVSQLLTQSRWDQHGYD-----DIERQSNELTLIRRQI
KTAPEYNPEQHRPIAYPQRAKVVGPEGETIHVDEWGRIKVRFLFTRSDDHGHDGGAGSNDNDTDSAWVDVLT
PWAGEGYGARFLPRIGEVVVIDFFDGNIDRPFVTGRIHEAQRSPTKFDVKGQLPDTKKLSGIRSQEVNGSGF

NQLRFDDTTGQISTQLQSSHAATQLNLGNLSHPKEQATSQGRGEGFELRTDAWGAVRAGKGMLISTYAQEQA
IADHLEAAQAQSLLSQGYESMKMLSEVAAKQQTDALNVINRLPKFIQSLELKTTGQALNSTVNLFKEGINND
PIHALKDCGGFIQDIGALGGNAKGVVDEFNSFFSDAKDAVENLKAFIENVEEHGPDIVKGKLASIKDRIHKN
PFESIQEVGRVLANVETKDFDLMSTCGTFSKGSKLEVSPSKALSSLQGFMEGYTQGLESSSDTKQQEQGKIF
RQALMLLASPNGIALTTPENIILQASQDIAESASGSINLSAQKNIIGHAQDKISLFAAQKGLRAYAAKGKLE
LQAQDDAIEAIAKKVIKLISTEEKIELTSPKEIVLTAGGSQLKINANGVFSTTGGKFESKAGQHLFTSGAKV
SYEVPELP------SSFDYSNRIDLYKIFQWSDF-ENIKYSAI--FENQRVTQGNLDELGRTERFKSSMPDK
MKVLVGYSADKWHKETEEFEDFDDYETNNDSNLNNNSN----------------------------------
------------------------------------------------------------------------
-----------------------

>AR_0083_17
-MLFNIFSVLEKIGLNAQKRAIHVQFSNELLNHQVFLQRIEGQHQLNGGLMAELICLSTNAQIALKQFIGVQ
VAVDQVTDSGQLFRTTGIVTEASYGQSDGALTLYKLTIEDATNLWHKRRNSRVFMNKSIVEITEVLFKEWQE
KSPLFAASLSLDLGGLS-QNYDIRPFTMQHNESDYDFLTRLWRSEGVSWLIDESELFVPHFTAPIQAQKLRL
IDDNNQYQALARRSIRYHRSSATEYQDSITGFVAVRSLQPTAVHVQRWQPDALAQEEGNGSVVTTHTHSDNF
DSATLSLEQAWHVSPAWMQDLKGEDQATASSSSQLEKLNQHFTDMHASRAKYFKAYSSVRDTQVGYWFNLRE
HPEIDQ-HEGADQEFLIIAKNFYNQNNLPKDLHQQVSQLLTQSRWDQHGYD-----DIERQSNELTLIRRQI
KTAPEYNPEQHRPIAYPQRAKVVGPEGETIHVDEWGRIKVRFLFTRSDDHGHDGGAGSNDNDTDSAWVDVLT
PWAGEGYGARFLPRIGEVVVIDFFDGNIDRPFVTGRIHEAQRSPTKFDVKGQLPDTKKLSGIRSQEVNGSGF
NQLRFDDTTGQISTQLQSSHAATQLNLGNLSHPKEQATSQGRGEGFELRTDAWGAVRAGKGMLISTYAQEQA
IADHLEAAQAQSLLSQGYESMKMLSEVAAKQQTDALNVINRLPKFIQSLELKTTGQALNSTVNLFKEGINND
PIHALKDCGGFIQDIGALGGNAKGVVDEFNSFFSDAKDAVENLKAFIENVEEHGPDIVKGKLASIKDRIHKN
PFESIQEVGRVLANVETKDFDLMSTCGTFSKGSKLEVSPSKALSSLQGFMEGYTQGLESSSDTKQQEQGKIF
RQALMLLASPNGIALTTPENIILQASQDIAESASGSINLSAQKNIIGHAQDKISLFAAQKGLRAYAAKGKLE
LQAQDDAIEAIAKKVIKLISTEEKIELTSPKEIVLTAGGSQLKINANGVFSTTGGKFESKAGQHLFTSGAKV
SYEVPELP------SSFDYSNRIDLYKIFQWSDF-ENIKYSAI--FENQRVTQGNLDELGRTERFKSSMPDK
MKVLVGYSADKWHKETEEFEDFDDYETNNDSNLNNNSN----------------------------------
------------------------------------------------------------------------
-----------------------

>AYE_17
-MLFNIFSVLEKIGLNAQKRAIHVQFSNELLNHQVFLQRIEGQHQLNGGLMAELICLSTNAQIALKQFIGVQ
VAVDQVTDSGQLFRTTGIVTEASYGQSDGALTLYKLTIEDATNLWHKRRNSRVFMNKSIVEITEVLFKEWQE
KSPLFAASLSLDLGGLS-QNYDIRPFTMQHNESDYDFLTRLWRSEGVSWLIDESELFVPHFTAPIQAQKLRL

IDDNNQYQALARRSIRYHRSSATEYQDSITGFVAVRSLQPTAVHVQRWQPDALAQEEGNGSVVTTHTHSDNF
DSATLSLEQAWHVSPAWMQDLKGEDQATASSSSQLEKLNQHFTDMHASRAKYFKAYSSVRDTQVGYWFNLRE
HPEIDQ-HEGADQEFLIIAKNFYNQNNLPKDLHQQVSQLLTQSRWDQHGYD-----DIERQSNELTLIRRQI
KTAPEYNPEQHRPIAYPQRAKVVGPEGETIHVDEWGRIKVRFLFTRSDDHGHDGGAGSNDNDTDSAWVDVLT
PWAGEGYGARFLPRIGEVVVIDFFDGNIDRPFVTGRIHEAQRSPTKFDVKGQLPDTKKLSGIRSQEVNGSGF
NQLRFDDTTGQISTQLQSSHAATQLNLGNLSHPKEQATSQGRGEGFELRTDAWGAVRAGKGMLISTYAQEQA
IADHLEAAQAQSLLSQGYESMKMLSEVAAKQQTDALNVINRLPKFIQSLELKTTGQALNSTVNLFKEGINND
PIHALKDCGGFIQDIGALGGNAKGVVDEFNSFFSDAKDAVENLKAFIENVEEHGPDIVKGKLASIKDRIHKN
PFESIQEVGRVLANVETKDFDLMSTCGTFSKGSKLEVSPSKALSSLQGFMEGYTQGLESSSDTKQQEQGKIF
RQALMLLASPNGIALTTPENIILQASQDIAESASGSINLSAQKNIIGHAQDKISLFAAQKGLRAYAAKGKLE
LQAQDDAIEAIAKKVIKLISTEEKIELTSPKEIVLTAGGSQLKINANGVFSTTGGKFESKAGQHLFTSGAKV
SYEVPELP------SSFDYSNRIDLYKIFQWSDF-ENIKYSAI--FENQRVTQGNLDELGRTERFKSSMPDK
MKVLVGYSADKWHKETEEFEDFDDYETNNDSNLNNNSN----------------------------------
------------------------------------------------------------------------
-----------------------
>WCHAB005078_17
-MLFNIFSVLEKIGLNAQKRAIHVQFSNELLNHQVFLQRIEGQHQLNGGLMAELICLSTNAQIALKQFIGVQ
VAVDQVTDSGQLFRTTGIVTEASYGQSDGALTLYKLTIEDATNLWHKRRNSRVFMNKSIVEITEVLFKEWQE
KSPLFAASLSLDLGGLS-QNYDIRPFTMQHNESDYDFLTRLWRSEGVSWLIDESELFVPHFTAPIQAQKLRL
IDDNNQYQALARRSIRYHRSSATEYQDSITGFVAVRSLQPTAVHVQRWQPDALAQEEGNGSVVTTHTHSDNF
DSATLSLEQAWHVSPAWMQDLKGEDQATASSSSQLEKLNQHFTDMHASRAKYFKAYSSVRDTQVGYWFNLRE
HPEIDQ-HEGADQEFLIIAKNFYNQNNLPKDLHQQVSQLLTQSRWDQHGYD-----DIERQSNELTLIRRQI
KTAPEYNPEQHRPIAYPQRAKVVGPEGETIHVDEWGRIKVRFLFTRSDDHGHDGGAGSNDNDTDSAWVDVLT
PWAGEGYGARFLPRIGEVVVIDFFDGNIDRPFVTGRIHEAQRSPTKFDVKGQLPDTKKLSGIRSQEVNGSGF
NQLRFDDTTGQISTQLQSSHAATQLNLGNLSHPKEQATSQGRGEGFELRTDAWGAVRAGKGMLISTYAQEQA
IADHLEAAQAQSLLSQGYESMKMLSEVAAKQQTDALNVINRLPKFIQSLELKTTGQALNSTVNLFKEGINND
PIHALKDCGGFIQDIGALGGNAKGVVDEFNSFFSDAKDAVENLKAFIENVEEHGPDIVKGKLASIKDRIHKN
PFESIQEVGRVLANVETKDFDLMSTCGTFSKGSKLEVSPSKALSSLQGFMEGYTQGLESSSDTKQQEQGKIF
RQALMLLASPNGIALTTPENIILQASQDIAESASGSINLSAQKNIIGHAQDKISLFAAQKGLRAYAAKGKLE
LQAQDDAIEAIAKKVIKLISTEEKIELTSPKEIVLTAGGSQLKINANGVFSTTGGKFESKAGQHLFTSGAKV
SYEVPELP------SSFDYSNRIDLYKIFQWSDF-ENIKYSAI--FENQRVTQGNLDELGRTERFKSSMPDK
MKVLVGYSADKWHKETEEFEDFDDYETNNDSNLNNNSN----------------------------------
------------------------------------------------------------------------

```
-----------------------
>D36_17
-MLFNIFSVLEKIGLNAQKRAIHVQFSNELLNHQVFLQRIEGQHQLNGGLMAELICLSTNAQIALKQFIGVQ
VAVDQVTDSGQLFRTTGIVTEASYGQSDGALTLYKLTIEDATNLWHKRRNSRVFMNKSIVEITEVLFKEWQE
KSPLFAASLSLDLGGLS-QNYDIRPFTMQHNESDYDFLTRLWRSEGGSWLIDESELFVPHFTAPIQAQKLRL
IDDNNQYQALARRSIRYHRSSATEYQDSITGFVAVRSLQPTAVHVQRWQPDALAQEEGNGSVVTTHTHSDNF
DSATLSLEQAWHVSPAWMQDLKGEDQATASSSSQLEKLNQHFTDMHASRAKYFKAYSSVRDTQVGYWFNLRE
HPEIDQ-HEGADQEFLIIAKNFYNQNNLPKDLHQQVSQLLTQSRWDQHGYD-----DIERQSNELTLIRRQI
KTAPEYNPEQHRPIAYPQRAKVVGPEGETIHVDEWGRIKVRFLFTRSDDHGHDGGAGSNDNDTDSAWVDVLT
PWAGEGYGARFLPRIGEVVVIDFFDGNIDRPFVTGRIHEAQRSPTKFDVKGQLPDTKKLSGIRSQEVNGSGF
NQLRFDDTTGQISTQLQSSHAATQLNLGNLSHPKEQATSQGRGEGFELRTDAWGAVRAGKGMLISTYAQEQA
IADHLEAAQAQSLLSQGYESMKMLSEVAAKQQTDALNVINRLPKFIQSLELKTTGQALNSTVNLFKEGINND
PIHALKDCGGFIQDIGALGGNAKGVVDEFNSFFSDAKDAVENLKAFIENVEEHGPDIVKGKLASIKDRIHKN
PFESIQEVGRVLANVETKDFDLMSTCGTFSKGSKLEVSPSKALSSLQGFMEGYTQGLESSSDTKQQEQGKIF
RQALMLLASPNGIALTTPENIILQASQDIAESASGSINLSAQKNIIGHAQDKISLFAAQKGLRAYAAKGKLE
LQAQDDAIEAIAKKVIKLISTEEKIELTSPKEIVLTAGGSQLKINANGVFSTTGGKFESKAGQHLFTSGAKV
SYEVPELP------SSFDYSNRIDLYKIFQWSDF-ENIKYSAI--FENQRVTQGNLDELGRTERFKSSMPDK
MKVLVGYSADKWHKETEEFEDFDDYETNNDSNLNNNSN----------------------------------
------------------------------------------------------------------------
-----------------------
>AR_0088_14
-MLFNIFSVLEKIGLNAQKRAIHVQFSNELLNHQVFLQRIEGQHQLNGGLMAELICLSTNAQIALKQFIGVQ
VAVDQVTDSGQLFRTTGIVTEASYGQSDGALTLYKLTIEDATNLWHKRRNSRVFMNKSIVEITEVLFKEWQE
KSPLFAASLSLDLGGLS-QNYDIRPFTMQHNESDYDFLTRLWRSEGVSWLIDESELFVPHFTAPIQAQKLKL
IDDNNQYQALARRSIRYHRSSATEYQDSITGFVAVRSLQPTAVHVQRWQPDALAQEEGNGSVVTTHTHSDNF
DSATLSLEQAWHVSPAWMQDLKGEDQATASSSSQLEKLNQHFTDMHASRAKYFKAYSSVRDTQVGYWFNLRE
HPEIDQ-HEGADQEFLIIAKNFYNQNNLPKDLHQQVSQLLTQSRWDQHGYD-----DIERQSNELTLIRRQI
KTAPEYNPEQHRPIAYPQRAKVVGPEGETIHVDEWGRIKVRFLFTRSDDHGHDGGAGSNDNDTDSAWVDVLT
PWAGEGYGARFLPRIGEVVVIDFFDGNIDRPFVTGRIHEAQRSPTKFDVKGQLPDTKKLSGIRSQEVNGSGF
NQLRFDDTTGQISTQLQSSHAATQLNLGNLSHPKEQATSQGRGEGFELRTDAWGAVRAGKGMLISTYAQEQA
IADHLEAAQAQSLLSQGYESMKMLSEVAAKQQTDALNVINRLPKFIQSLELKTTGQALNSTVNLFKEGINND
PIHALKDCGGFIQDIGALGGNAKGVVDEFNSFFSDAKDAVENLKAFIENVEEHGPDIVKGKLASIKDRIHKN
PFESIQEVGRVLANVETKDFDLMSTCGTFSKGSKLEVSPSKALSSLQGFMEGYTQGLESSSDTKQQEQGKIF
```

RQALMLLASPNGIALTTPENIILQASQDIAESASGSINLSAQKNIIGHAQDKISLFAAQKGLRAYAAKGKLE
LQAQDDAIEAIAKKVIKLISTEEKIELTSPKEIVLTAGGSQLKINANGVFSTTGGKFESKAGQHLFTGGAKV
SYEVPELP------NTPTFSNRLDIYDLFWESDF-SKLSYKAFM-PETNSFTSGAVDEHGRTGKISSAD--P
TNVQVLIGSDDEWGLLIDSFDADDFLEDQSINGSNSEIDNNTEDRG--------------------------
------------------------------------------------------------------------
-----------------------
>CIP70.10_14
-MLFNIFSVLEKIGLNAQKRAIHVQFSNELLNHQVFLQRIEGQHQLNGGLMAELICLSTNAQIALKQFIGVQ
VAVDQVTDSGQLFRTTGIVTEASYGQSDGALTLYKLTIEDATNLWHKRRNSRVFMNKSIVEITEVLFKEWQE
KSPLFAASLSLDLGGLS-QNYDIRPFTMQHNESDYDFLTRLWRSEGVSWLIDESELFVPHFTAPIQAQKLKL
IDDNNQYQALARRSIRYHRSSATEYQDSITGFVAVRSLQPTAVHVQRWQPDALAQEEGNGSVVTTHTHSDNF
DSATLSLEQAWHVSPAWMQDLKGEDQATASSSSQLEKLNQHFTDMHASRAKYFKAYSSVRDTQVGYWFNLRE
HPEIDQ-HEGADQEFLIIAKNFYNQNNLPKDLHQQVSQLLTQSRWDQHGYD-----DIERQSNELTLIRRQI
KTAPEYNPEQHRPIAYPQRAKVVGPEGETIHVDEWGRIKVRFLFTRSDDHGHDGGAGSNDNDTDSAWVDVLT
PWAGEGYGARFLPRIGEVVVIDFFDGNIDRPFVTGRIHEAQRSPTKFDVKGQLPDTKKLSGIRSQEVNGSGF
NQLRFDDTTGQISTQLQSSHAATQLNLGNLSHPKEQATSQGRGEGFELRTDAWGAVRAGKGMLISTYAQEQA
IADHLEAAQAQSLLSQGYESMKMLSEVAAKQQTDALNVINRLPKFIQSLELKTTGQALNSTVNLFKEGINND
PIHALKDCGGFIQDIGALGGNAKGVVDEFNSFFSDAKDAVENLKAFIENVEEHGPDIVKGKLASIKDRIHKN
PFESIQEVGRVLANVETKDFDLMSTCGTFSKGSKLEVSPSKALSSLQGFMEGYTQGLESSSDTKQQEQGKIF
RQALMLLASPNGIALTTPENIILQASQDIAESASGSINLSAQKNIIGHAQDKISLFAAQKGLRAYAAKGKLE
LQAQDDAIEAIAKKVIKLISTEEKIELTSPKEIVLTAGGSQLKINANGVFSTTGGKFESKAGQHLFTGGAKV
SYEVPELP------NTPTFSNRLDIYDLFWESDF-SKLSYKAFM-PETNSFTSGAVDEHGRTGKISSAD--P
TNVQVLIGSDDEWGLLIDSFDADDFLEDQSINGSNSEIDNNTEDRG--------------------------
------------------------------------------------------------------------
-----------------------
>HWBA8_14
-MLFNIFSVLEKIGLNAQKRAIHVQFSNELLNHQVFLQRIEGQHQLNGGLMAELICLSTNAQIALKQFIGVQ
VAVDQVTDSGQLFRTTGIVTEASYGQSDGALTLYKLTIEDATNLWHKRRNSRVFMNKSIVEITEVLFKEWQE
KSPLFAASLSLDLGGLS-QNYDIRPFTMQHNESDYDFLTRLWRSEGVSWLIDESELFVPHFTAPIQAQKLKL
IDDNNQYQALARRSIRYHRSSATEYQDSITGFVAVRSLQPTAVHVQRWQPDALAQEEGNGSVVTTHTHSDNF
DSATLSLEQAWHVSPAWMQDLKGEDQATASSSSQLEKLNQHFTDMHASRAKYFKAYSSVRDTQVGYWFNLRE
HPEIDQ-HEGADQEFLIIAKNFYNQNNLPKDLHQQVSQLLTQSRWDQHGYD-----DIERQSNELTLIRRQI
KTAPEYNPEQHRPIAYPQRAKVVGPEGETIHVDEWGRIKVRFLFTRSDDHGHDGGAGSNDNDTDSAWVDVLT

PWAGEGYGARFLPRIGEVVVIDFFDGNIDRPFVTGRIHEAQRSPTKFDVKGQLPDTKKLSGIRSQEVNGSGF
NQLRFDDTTGQISTQLQSSHAATQLNLGNLSHPKEQATSQGRGEGFELRTDAWGAVRAGKGMLISTYAQEQA
IADHLEAAQAQSLLSQGYESMKMLSEVAAKQQTDALNVINRLPKFIQSLELKTTGQALNSTVNLFKEGINND
PIHALKDCGGFIQDIGALGGNAKGVVDEFNSFFSDAKDAVENLKAFIENVEEHGPDIVKGKLASIKDRIHKN
PFESIQEVGRVLANVETKDFDLMSTCGTFSKGSKLEVSPSKALSSLQGFMEGYTQGLESSSDTKQQEQGKIF
RQALMLLASPNGIALTTPENIILQASQDIAESASGSINLSAQKNIIGHAQDKISLFAAQKGLRAYAAKGKLE
LQAQDDAIEAIAKKVIKLISTEEKIELTSPKEIVLTAGGSQLKINANGVFSTTGGKFESKAGQHLFTGGAKV
SYEVPELP------NTPTFSNRLDIYDLFWESDF-SKLSYKAFM-PETNSFTSGAVDEHGRTGKISSAD--P
TNVQVLIGSDDEWGLLIDSFDADDFLEDQSINGSNSEIDNNTEDRG--------------------------
------------------------------------------------------------------------
-----------------------
>R2091_14
-MLFNIFSVLEKIGLNAQKRAIHVQFSNELLNHQVFLQRIEGQHQLNGGLMAELICLSTNAQIALKQFIGVQ
VAVDQVTDSGQLFRTTGIVTEASYGQSDGALTLYKLTIEDATNLWHKRRNSRVFMNKSIVEITEVLFKEWQE
KSPLFAASLSLDLGGLS-QNYDIRPFTMQHNESDYDFLTRLWRSEGVSWLIDESELFVPHFTAPIQAQKLKL
IDDNNQYQALARRSIRYHRSSATEYQDSITGFVAVRSLQPTAVHVQRWQPDALAQEEGNGSVVTTHTHSDNF
DSATLSLEQAWHVSPAWMQDLKGEDQATASSSSQLEKLNQHFTDMHASRAKYFKAYSSVRDTQVGYWFNLRE
HPEIDQ-HEGADQEFLIIAKNFYNQNNLPKDLHQQVSQLLTQSRWDQHGYD-----DIERQSNELTLIRRQI
KTAPEYNPEQHRPIAYPQRAKVVGPEGETIHVDEWGRIKVRFLFTRSDDHGHDGGAGSNDNDTDSAWVDVLT
PWAGEGYGARFLPRIGEVVVIDFFDGNIDRPFVTGRIHEAQRSPTKFDVKGQLPDTKKLSGIRSQEVNGSGF
NQLRFDDTTGQISTQLQSSHAATQLNLGNLSHPKEQATSQGRGEGFELRTDAWGAVRAGKGMLISTYAQEQA
IADHLEAAQAQSLLSQGYESMKMLSEVAAKQQTDALNVINRLPKFIQSLELKTTGQALNSTVNLFKEGINND
PIHALKDCGGFIQDIGALGGNAKGVVDEFNSFFSDAKDAVENLKAFIENVEEHGPDIVKGKLASIKDRIHKN
PFESIQEVGRVLANVETKDFDLMSTCGTFSKGSKLEVSPSKALSSLQGFMEGYTQGLESSSDTKQQEQGKIF
RQALMLLASPNGIALTTPENIILQASQDIAESASGSINLSAQKNIIGHAQDKISLFAAQKGLRAYAAKGKLE
LQAQDDAIEAIAKKVIKLISTEEKIELTSPKEIVLTAGGSQLKINANGVFSTTGGKFESKAGQHLFTGGAKV
SYEVPELP------NTPTFSNRLDIYDLFWESDF-SKLSYKAFM-PETNSFTSGAVDEHGRTGKISSAD--P
TNVQVLIGSDDEWGLLIDSFDADDFLEDQSINGSNSEIDNNTEDRG--------------------------
------------------------------------------------------------------------
-----------------------
>AR_0078_29
-MLFNIFSVLEKIGLNAQKRAIHVQFSNELLNHQVFLQRIEGQHQLNGGLMAELICLSTNAQIALKQFIGVQ
VAVDQVTDSGQLFRTTGIVTEASYGQSDGALTLYKLTIEDATNLWHKRRNSRVFMNKSIVEITEVLFKEWQE

KSPLFAASLSLDLGGLS-QNYDIRPFTMQHNESDYDFLTRLWRSEGVSWLIDESELFVPHFTAPIQAQKLRL
IDDNNQNQALARRSIRYHRSSATEYQDSITGFVAVRSLQPTAVHVQRWQPDALAQEEGNGSVVTTHTHSDNF
DSATLSLEQAWHVSPAWMQDLKGEDQATASSSSQLEKLNQHFTDMHASRAKYFKAYSSVRDTQVGYWFNLRE
HPEIDQ-HEGADQEFLIIAKNFYNQNNLPKDLHQQVSQLLTQSRWDQHGYD-----DIERQSNELTLIRRQI
KTAPEYNPEQHRPIAYPQRAKVVGPEGETIHVDEWGRIKVRFLFTRSDDHGHDGGAGSNDNDTDSAWVDVLT
PWAGEGYGARFLPRIGEVVVIDFFDGNIDRPFVTGRIHEAQRSPTKFDVKGQLPDTKKLSGIRSQEINGSGF
NQLRFDDTTGQISTQLQSSHAATQLNLGNLSHPKEQATSQGRGEGFELRTDAWGAVRAGKGMLISTYAQEQA
IADHLEAAQAQSLLSQGYESMKMLSEVAAKQQTDALNVINRLPKFIQSLELKTTGQALNSTVNLFKEGINND
PIHALKDCGGFIQDIGALGGNAKGVVDEFNSFFSDAKDAVENLKAFIENVEEHGPDIVKGKLASIKDRIHKN
PFESIQEVGKVLANVETKDFDLMSTCGTFSKGSKLEVSPSKALSSLQGFMEGYTQGLESSSDTKQQEQGKIF
RQALMLLASPNGIALTTPENIILQASQDIAESASGSINLSAQKNIIGHAQDKISLFAAQKGLRAYAAKGKLE
LQAQDDAIEAIAKKVIKLISTEEKIELTSPKEIVLTAGGSQLKINANGVFSTTGGKFESKAGQHLFAGGAKV
SYEVPELP------NTPTFSNRLDIYDLFWESDF-SKLSYKAFM-PETNSFTSGAIDEHGRTGKIGTPD--P
TNVQVLIGSDDEWGLLIDSFDEDEILEDQSINSNNSAIDNNIEDRG--------------------------
------------------------------------------------------------------------
-----------------------

>ab736_10
-MLNNIFDFLDKIGFASQKRALHIYFTNPEINNQVFLQRIDGFHSINEGLSAELICLSINPYISLKQFIGSQ
VAVEQVTDSGKLFRTTGIITGASQGQSDGALCLYKLTMQDATSLWYKRRNSRVFMNKSAVEIIEIIFKEWQN
KSSLFAASLQLDSSGLT-KNYDIRPFSMQSNESDYAYLTRLMREEGINWLIDESESMVLSNSQTIEPQKLRL
IDHNNEYKVLERRAIRYHRSDATEQNDSITSFIAQRQLQPTAIHLQRWQADNLSQEDASGSVLSAHQHSSQR
DNESLSLEQAWTIAPAWITDLNGEDQATTSSNSQIEKLNKQLNQYQALQAKYFIARSSVRDAQVGYWFKLNE
HPELERNHESSDKEFLILSKRFYNQNNLPKDIRDQVEQLLSLSHWQ-VSHD----RAQERQANELMVVRRYI
DIVPEFDPLIHRPSAHVQRAKVVGLDGEEIHVDEWGRIKVRFLFTRTDDHQHDGGAGSNDNDTDSAWVDVLT
PWAGEGYGARFLPRVGEIVVIDFFDGNIDRPFVTGRVHEGQRSPTKFDIKGQLPDTKKLSGIRTKEVSGSGF
NQLRFDDTTGQISTQLHSSHGASQLNLGNLSYPKDSATSEGWGEGFELRTDQWGAVRAGDGLLISTYKQKNA
AHDALNLEQTIADLKTHKEWNTVLEDNVKEHKVMAL------------------------------------
----------------------------------------DALATLTKSIEA--------------------
-----------------------------------------------------------LEAAGKETEVQTL
KEAIIILTSPADITLNSSKNIFLQSQSNTHISAKENINLMSDQNTVLASQKKLSLFSYEDFMQIISAKGKLE
IQTHEKSIEMIANEVVKILSVKSNIELTSPEEITLTAKESQIKINGSGVFITTNGKFEVKAGQHVFLSGAKA
D-----LP-------------KLTWSFKEKTASCMKNA-------SESHEAFVGFEG---------------
------------------------------------------------------------------------

------------------------------------------------------------------------
-----------------------
>ATCC19606_10
-MLNNIFDFLDKIGFASQKRALHIYFTNPEINNQVFLQRIDGFHSINEGLSAELICLSINPYISLKQFIGSQ
VAVEQVTDSGKLFRTTGIITGASQGQSDGALCLYKLTMQDATSLWYKRRNSRVFMNKSAVEIIEIIFKEWQN
KSSLFAASLQLDSSGLT-KNYDIRPFSMQSNESDYAYLTRLMREEGINWLIDESESMVLSNSQTIEPQKLRL
IDHNNEYKVLERRAIRYHRSDATEQNDSITSFIAQRQLQPTAIHLQRWQADNLSQEDASGSVLSAHQHSSQR
DNESLSLEQAWTIAPAWITDLNGEDQATTSSNSQIEKLNKQLNQYQALQAKYFIARSSVRDAQVGYWFKLNE
HPELERNHESSDKEFLILSKRFYNQNNLPKDIRDQVEQLLSLSHWQ-VSHD----RAQERQANELMVVRRYI
DIVPEFDPLIHRPSAHVQRAKVVGLDGEEIHVDEWGRIKVRFLFTRTDDHQHDGGAGSNDNDTDSAWVDVLT
PWAGEGYGARFLPRVGEIVVIDFFDGNIDRPFVTGRVHEGQRSPTKFDIKGQLPDTKKLSGIRTKEVSGSGF
NQLRFDDTTGQISTQLHSSHGASQLNLGNLSYPKDSATSEGWGEGFELRTDQWGAVRAGDGLLISTYKQKNA
AHDALNLEQTIADLKTHKEWNTVLEDNVKEHKVMAL------------------------------------
----------------------------------------DALATLTKSIEA--------------------
-----------------------------------------------------------LEAAGKETEVQTL
KEAIIILTSPADITLNSSKNIFLQSQSNTHISAKENINLMSDQNTVLASQKKLSLFSYEDFMQIISAKGKLE
IQTHEKSIEMIANEVVKILSVKSNIELTSPEEITLTAKESQIKINGSGVFITTNGKFEVKAGQHVFLSGAKA
D-----LP-------------KLTWSFKEKTASCMKNA-------SESHEAFVGFEG---------------
------------------------------------------------------------------------
------------------------------------------------------------------------
-----------------------
>VgrG4Abay
-MLNSIFGILDKLGFNQQKRAISIQFSNQELNHHIMLQRIDGYHAINDGLSVELICISTNPYLSLKQFIGCQ
AAIDQVTDTGKLFRTTGIITEASQGQSDGALTLYRLRLQDATSLWHKRRNSRVFMNKSVVEIIEIVFREWQS
KSPLFAATLKLDTSHLTNKQYDIRPFSMQSNESDYAYLTRLMREEAINWLIDESNYLVMSQTQAIEPQYLRL
VDQNTEYSCLERSSIRYHRSHATEQQDSITSLIAQRQLQSTAIYVQRWQADALSQEDASGSVLSNHQHSQQH
GNESLSLEQAWNISPAWMTDLNGEDQATASDSRQLEKLNKQLHLYQALQAKSFTAYSSVRDTQVGYWFRLVD
HPELDKNHDESDKEFLIVSKHFYNQNNLPKDIQDQIQQLLSLNHWS-IYQD----SFQERQANKLTLIRRNI
DIVPEYDPLQHRAIAHVQRARVVGSETEEIHVDEWGRIKVRFLFTRIEDHEHDGGAGSNDSDTDSAWVDVLT
PWAGEGYGARFLPRVGEIVVIDFFDGNIDRPFVTGRIHEAHRSPTKFDIKGQLPETKKLSGIRSKEVAGEGY
NQLRFDDTTGQISAQLHSSHGTTQLNLGHLSHPKDKAESEGRGEGFELRTDDWGAIRASKGILLTSEASEQA
QGEQLTRSSIKENLHFHTQSNQYFKELAQAHEVDEPDL----------------------------------
----------------------------------------EAQKSLKEKFES--------------------

--------------------------------------------------------------------WADN
NDALVALHSESALILDSKESLQLTSKDNIELSTPKNLQFFTGKSWIAKALDKISLFAKHAGIKIKSGEGDIE
VAAQKGKMTLSSKQQMHVYSLNDFVRIESGKGILITAGGGYIKIQNGNIEIACPGLMELKAGQIQAKSGASL
SSELPAMP-------ELKSQYDEHFILHYPDGEPAKNLKYRIT--AEDGQVFEGVSGEDGKTTVFTKDA---
-----------------MTALNIEVFSPE-------------------------------------------
------------------------------------------------------------------------

---

**Supplementary file S4g**

>PAAR1
MTSPIFLHELPIEQLQQMSQEDIEQTLKAEQLYFQHRPKAMYYLAVNGSKTKQGGLVKASATQGKIGDLSIA
RIGDDVIY-ADGTIAKIISGAGKVCIVEGLSAALVGSQLDNGDEIIDSPNRTVAIN----------------
------------------------------------------------------------------------
----------------------IFKGETLPEGFLIPEGTDHG------------------------------
---------------------------------------
>PAAR3
MSRP--------------------------------------YITI-GSPTTGGGKVITANRRFLINGKAIA
CVGDKATCPTHKTVATIIAGDNHMLVM-GKAAAQHNSPLSCGCKCIADQSLTVGNN----------------
------------------------------------------------------------------------
------------------------------------------------------------------------
---------------------------------------
>15A34_B
MTTP--------------------------------------YITI-GCPTSGGGQVISGNNMFLIDGIPVA
CVGDKATCPTHKVVATIVSGDPCMQIF-GKAAARVNDSLSCGCKLLPQQNLVVQDNGGGAASSAAKSSSAPI
SQKQPATDSFVKDEYENYYIEQNTTKYVKFTNGIFPYDEDKKNLFGAISQAVSGVCTFIVTYILKGQELFVT
VSMLPPTLSGDATIFPYATLDLVHNNQSLGK---TRLEKGKG---VWSTENSKEPVGQCKVTLPKPDLSTIE
ATLTMGYTAKFDG--GTVRPNPPHTRFSFTLNSASRRKS
>15A5_B
MTTP--------------------------------------YITI-GCPTSGGGQVISGNNMFLIDGIPVA
CVGDKATCPTHKVVATIVSGDPCMQIF-GKAAARVNDSLSCGCKLLPQQNLVVQDNGGGAASSAAKSSSAPI
SQKQPATDSFVKDEYENYYIEQNTTKYVKFTNGIFPYDEDKKNLFGAISQAVSGVCTFIVTYILKGQELFVT
VSMLPPTLSGDATIFPYATLDLVHNNQSLGK---TRLEKGKG---VWSTENSKEPVGQCKVTLPKPDLSTIE
ATLTMGYTAKFDG--GTVRPNPPHTRFSFTLNSASRRKS
>3027STDY5784958_B

MTTP--------------------------------------YITI-GCPTSGGGQVISGNNMFLIDGIPVA
CVGDKATCPTHKVVATIVSGDPCMQIF-GKAAARVNDSLSCGCKLLPQQNLVVQDNGGGAASSAAKSSSAPI
SQKQPATDSFVKDEYENYYIEQNTTKYVKFTNGIFPYDEDKKNLFGAISQAVSGVCTFIVTYILKGQELFVT
VSMLPPTLSGDATIFPYATLDLVHNNQSLGK---TRLEKGKG---VWSTENSKEPVGQCKVTLPKPDLSTIE
ATLTMGYTAKFDG--GTVRPNPPHTRFSFTLNSASRRKS
>AB34299_B
MTTP--------------------------------------YITI-GCPTSGGGQVISGNNMFLIDGIPVA
CVGDKATCPTHKVVATIVSGDPCMQIF-GKAAARVNDSLSCGCKLLPQQNLVVQDNGGGAASSAAKSSSAPI
SQKQPATDSFVKDEYENYYIEQNTTKYVKFTNGIFPYDEDKKNLFGAISQAVSGVCTFIVTYILKGQELFVT
VSMLPPTLSGDATIFPYATLDLVHNNQSLGK---TRLEKGKG---VWSTENSKEPVGQCKVTLPKPDLSTIE
ATLTMGYTAKFDG--GTVRPNPPHTRFSFTLNSASRRKS
>Ab4568_B
MTTP--------------------------------------YITI-GCPTSGGGQVISGNNMFLIDGIPVA
CVGDKATCPTHKVVATIVSGDPCMQIF-GKAAARVNDSLSCGCKLLPQQNLVVQDNGGGAASSAAKSSSAPI
SQKQPATDSFVKDEYENYYIEQNTTKYVKFTNGIFPYDEDKKNLFGAISQAVSGVCTFIVTYILKGQELFVT
VSMLPPTLSGDATIFPYATLDLVHNNQSLGK---TRLEKGKG---VWSTENSKEPVGQCKVTLPKPDLSTIE
ATLTMGYTAKFDG--GTVRPNPPHTRFSFTLNSASRRKS
>Ab4653_B
MTTP--------------------------------------YITI-GCPTSGGGQVISGNNMFLIDGIPVA
CVGDKATCPTHKVVATIVSGDPCMQIF-GKAAARVNDSLSCGCKLLPQQNLVVQDNGGGAASSAAKSSSAPI
SQKQPATDSFVKDEYENYYIEQNTTKYVKFTNGIFPYDEDKKNLFGAISQAVSGVCTFIVTYILKGQELFVT
VSMLPPTLSGDATIFPYATLDLVHNNQSLGK---TRLEKGKG---VWSTENSKEPVGQCKVTLPKPDLSTIE
ATLTMGYTAKFDG--GTVRPNPPHTRFSFTLNSASRRKS
>Ab4977_B
MTTP--------------------------------------YITI-GCPTSGGGQVISGNNMFLIDGIPVA
CVGDKATCPTHKVVATIVSGDPCMQIF-GKAAARVNDSLSCGCKLLPQQNLVVQDNGGGAASSAAKSSSAPI
SQKQPATDSFVKDEYENYYIEQNTTKYVKFTNGIFPYDEDKKNLFGAISQAVSGVCTFIVTYILKGQELFVT
VSMLPPTLSGDATIFPYATLDLVHNNQSLGK---TRLEKGKG---VWSTENSKEPVGQCKVTLPKPDLSTIE
ATLTMGYTAKFDG--GTVRPNPPHTRFSFTLNSASRRKS
>AbPK1_B
MTTP--------------------------------------YITI-GCPTSGGGQVISGNNMFLIDGIPVA
CVGDKATCPTHKVVATIVSGDPCMQIF-GKAAARVNDSLSCGCKLLPQQNLVVQDNGGGAASSAAKSSSAPI
SQKQPATDSFVKDEYENYYIEQNTTKYVKFTNGIFPYDEDKKNLFGAISQAVSGVCTFIVTYILKGQELFVT

VSMLPPTLSGDATIFPYATLDLVHNNQSLGK---TRLEKGKG---VWSTENSKEPVGQCKVTLPKPDLSTIE
ATLTMGYTAKFDG--GTVRPNPPHTRFSFTLNSASRRKS
>AC29_B
MTTP--------------------------------------YITI-GCPTSGGGQVISGNNMFLIDGIPVA
CVGDKATCPTHKVVATIVSGDPCMQIF-GKAAARVNDSLSCGCKLLPQQNLVVQDNGGGAASSAAKSSSAPI
SQKQPATDSFVKDEYENYYIEQNTTKYVKFTNGIFPYDEDKKNLFGAISQAVSGVCTFIVTYILKGQELFVT
VSMLPPTLSGDATIFPYATLDLVHNNQSLGK---TRLEKGKG---VWSTENSKEPVGQCKVTLPKPDLSTIE
ATLTMGYTAKFDG--GTVRPNPPHTRFSFTLNSASRRKS
>AF-673_B
MTTP--------------------------------------YITI-GCPTSGGGQVISGNNMFLIDGIPVA
CVGDKATCPTHKVVATIVSGDPCMQIF-GKAAARVNDSLSCGCKLLPQQNLVVQDNGGGAASSAAKSSSAPI
SQKQPATDSFVKDEYENYYIEQNTTKYVKFTNGIFPYDEDKKNLFGAISQAVSGVCTFIVTYILKGQELFVT
VSMLPPTLSGDATIFPYATLDLVHNNQSLGK---TRLEKGKG---VWSTENSKEPVGQCKVTLPKPDLSTIE
ATLTMGYTAKFDG--GTVRPNPPHTRFSFTLNSASRRKS
>AR_0056_B
MTTP--------------------------------------YITI-GCPTSGGGQVISGNNMFLIDGIPVA
CVGDKATCPTHKVVATIVSGDPCMQIF-GKAAARVNDSLSCGCKLLPQQNLVVQDNGGGAASSAAKSSSAPI
SQKQPATDSFVKDEYENYYIEQNTTKYVKFTNGIFPYDEDKKNLFGAISQAVSGVCTFIVTYILKGQELFVT
VSMLPPTLSGDATIFPYATLDLVHNNQSLGK---TRLEKGKG---VWSTENSKEPVGQCKVTLPKPDLSTIE
ATLTMGYTAKFDG--GTVRPNPPHTRFSFTLNSASRRKS
>AR_0102_B
MTTP--------------------------------------YITI-GCPTSGGGQVISGNNMFLIDGIPVA
CVGDKATCPTHKVVATIVSGDPCMQIF-GKAAARVNDSLSCGCKLLPQQNLVVQDNGGGAASSAAKSSSAPI
SQKQPATDSFVKDEYENYYIEQNTTKYVKFTNGIFPYDEDKKNLFGAISQAVSGVCTFIVTYILKGQELFVT
VSMLPPTLSGDATIFPYATLDLVHNNQSLGK---TRLEKGKG---VWSTENSKEPVGQCKVTLPKPDLSTIE
ATLTMGYTAKFDG--GTVRPNPPHTRFSFTLNSASRRKS
>AYP-A2_B
MTTP--------------------------------------YITI-GCPTSGGGQVISGNNMFLIDGIPVA
CVGDKATCPTHKVVATIVSGDPCMQIF-GKAAARVNDSLSCGCKLLPQQNLVVQDNGGGAASSAAKSSSAPI
SQKQPATDSFVKDEYENYYIEQNTTKYVKFTNGIFPYDEDKKNLFGAISQAVSGVCTFIVTYILKGQELFVT
VSMLPPTLSGDATIFPYATLDLVHNNQSLGK---TRLEKGKG---VWSTENSKEPVGQCKVTLPKPDLSTIE
ATLTMGYTAKFDG--GTVRPNPPHTRFSFTLNSASRRKS
>CBA7_B

MTTP--------------------------------------YITI-GCPTSGGGQVISGNNMFLIDGIPVA
CVGDKATCPTHKVVATIVSGDPCMQIF-GKAAARVNDSLSCGCKLLPQQNLVVQDNGGGAASSAAKSSSAPI
SQKQPATDSFVKDEYENYYIEQNTTKYVKFTNGIFPYDEDKKNLFGAISQAVSGVCTFIVTYILKGQELFVT
VSMLPPTLSGDATIFPYATLDLVHNNQSLGK---TRLEKGKG---VWSTENSKEPVGQCKVTLPKPDLSTIE
ATLTMGYTAKFDG--GTVRPNPPHTRFSFTLNSASRRKS
>CMC-CR-MDR-Ab4_B
MTTP--------------------------------------YITI-GCPTSGGGQVISGNNMFLIDGIPVA
CVGDKATCPTHKVVATIVSGDPCMQIF-GKAAARVNDSLSCGCKLLPQQNLVVQDNGGGAASSAAKSSSAPI
SQKQPATDSFVKDEYENYYIEQNTTKYVKFTNGIFPYDEDKKNLFGAISQAVSGVCTFIVTYILKGQELFVT
VSMLPPTLSGDATIFPYATLDLVHNNQSLGK---TRLEKGKG---VWSTENSKEPVGQCKVTLPKPDLSTIE
ATLTMGYTAKFDG--GTVRPNPPHTRFSFTLNSASRRKS
>CMC-CR-MDR-Ab66_B
MTTP--------------------------------------YITI-GCPTSGGGQVISGNNMFLIDGIPVA
CVGDKATCPTHKVVATIVSGDPCMQIF-GKAAARVNDSLSCGCKLLPQQNLVVQDNGGGAASSAAKSSSAPI
SQKQPATDSFVKDEYENYYIEQNTTKYVKFTNGIFPYDEDKKNLFGAISQAVSGVCTFIVTYILKGQELFVT
VSMLPPTLSGDATIFPYATLDLVHNNQSLGK---TRLEKGKG---VWSTENSKEPVGQCKVTLPKPDLSTIE
ATLTMGYTAKFDG--GTVRPNPPHTRFSFTLNSASRRKS
>CMC-MDR-Ab59_B
MTTP--------------------------------------YITI-GCPTSGGGQVISGNNMFLIDGIPVA
CVGDKATCPTHKVVATIVSGDPCMQIF-GKAAARVNDSLSCGCKLLPQQNLVVQDNGGGAASSAAKSSSAPI
SQKQPATDSFVKDEYENYYIEQNTTKYVKFTNGIFPYDEDKKNLFGAISQAVSGVCTFIVTYILKGQELFVT
VSMLPPTLSGDATIFPYATLDLVHNNQSLGK---TRLEKGKG---VWSTENSKEPVGQCKVTLPKPDLSTIE
ATLTMGYTAKFDG--GTVRPNPPHTRFSFTLNSASRRKS
>DU202_B
MTTP--------------------------------------YITI-GCPTSGGGQVISGNNMFLIDGIPVA
CVGDKATCPTHKVVATIVSGDPCMQIF-GKAAARVNDSLSCGCKLLPQQNLVVQDNGGGAASSAAKSSSAPI
SQKQPATDSFVKDEYENYYIEQNTTKYVKFTNGIFPYDEDKKNLFGAISQAVSGVCTFIVTYILKGQELFVT
VSMLPPTLSGDATIFPYATLDLVHNNQSLGK---TRLEKGKG---VWSTENSKEPVGQCKVTLPKPDLSTIE
ATLTMGYTAKFDG--GTVRPNPPHTRFSFTLNSASRRKS
>HRAB-85_B
MTTP--------------------------------------YITI-GCPTSGGGQVISGNNMFLIDGIPVA
CVGDKATCPTHKVVATIVSGDPCMQIF-GKAAARVNDSLSCGCKLLPQQNLVVQDNGGGAASSAAKSSSAPI
SQKQPATDSFVKDEYENYYIEQNTTKYVKFTNGIFPYDEDKKNLFGAISQAVSGVCTFIVTYILKGQELFVT

VSMLPPTLSGDATIFPYATLDLVHNNQSLGK---TRLEKGKG---VWSTENSKEPVGQCKVTLPKPDLSTIE
ATLTMGYTAKFDG--GTVRPNPPHTRFSFTLNSASRRKS
>JBA13_B
MTTP--------------------------------------YITI-GCPTSGGGQVISGNNMFLIDGIPVA
CVGDKATCPTHKVVATIVSGDPCMQIF-GKAAARVNDSLSCGCKLLPQQNLVVQDNGGGAASSAAKSSSAPI
SQKQPATDSFVKDEYENYYIEQNTTKYVKFTNGIFPYDEDKKNLFGAISQAVSGVCTFIVTYILKGQELFVT
VSMLPPTLSGDATIFPYATLDLVHNNQSLGK---TRLEKGKG---VWSTENSKEPVGQCKVTLPKPDLSTIE
ATLTMGYTAKFDG--GTVRPNPPHTRFSFTLNSASRRKS
>KAB01_B
MTTP--------------------------------------YITI-GCPTSGGGQVISGNNMFLIDGIPVA
CVGDKATCPTHKVVATIVSGDPCMQIF-GKAAARVNDSLSCGCKLLPQQNLVVQDNGGGAASSAAKSSSAPI
SQKQPATDSFVKDEYENYYIEQNTTKYVKFTNGIFPYDEDKKNLFGAISQAVSGVCTFIVTYILKGQELFVT
VSMLPPTLSGDATIFPYATLDLVHNNQSLGK---TRLEKGKG---VWSTENSKEPVGQCKVTLPKPDLSTIE
ATLTMGYTAKFDG--GTVRPNPPHTRFSFTLNSASRRKS
>KAB02_B
MTTP--------------------------------------YITI-GCPTSGGGQVISGNNMFLIDGIPVA
CVGDKATCPTHKVVATIVSGDPCMQIF-GKAAARVNDSLSCGCKLLPQQNLVVQDNGGGAASSAAKSSSAPI
SQKQPATDSFVKDEYENYYIEQNTTKYVKFTNGIFPYDEDKKNLFGAISQAVSGVCTFIVTYILKGQELFVT
VSMLPPTLSGDATIFPYATLDLVHNNQSLGK---TRLEKGKG---VWSTENSKEPVGQCKVTLPKPDLSTIE
ATLTMGYTAKFDG--GTVRPNPPHTRFSFTLNSASRRKS
>KAB03_B
MTTP--------------------------------------YITI-GCPTSGGGQVISGNNMFLIDGIPVA
CVGDKATCPTHKVVATIVSGDPCMQIF-GKAAARVNDSLSCGCKLLPQQNLVVQDNGGGAASSAAKSSSAPI
SQKQPATDSFVKDEYENYYIEQNTTKYVKFTNGIFPYDEDKKNLFGAISQAVSGVCTFIVTYILKGQELFVT
VSMLPPTLSGDATIFPYATLDLVHNNQSLGK---TRLEKGKG---VWSTENSKEPVGQCKVTLPKPDLSTIE
ATLTMGYTAKFDG--GTVRPNPPHTRFSFTLNSASRRKS
>KAB04_B
MTTP--------------------------------------YITI-GCPTSGGGQVISGNNMFLIDGIPVA
CVGDKATCPTHKVVATIVSGDPCMQIF-GKAAARVNDSLSCGCKLLPQQNLVVQDNGGGAASSAAKSSSAPI
SQKQPATDSFVKDEYENYYIEQNTTKYVKFTNGIFPYDEDKKNLFGAISQAVSGVCTFIVTYILKGQELFVT
VSMLPPTLSGDATIFPYATLDLVHNNQSLGK---TRLEKGKG---VWSTENSKEPVGQCKVTLPKPDLSTIE
ATLTMGYTAKFDG--GTVRPNPPHTRFSFTLNSASRRKS
>KAB05_B

MTTP--------------------------------------YITI-GCPTSGGGQVISGNNMFLIDGIPVA
CVGDKATCPTHKVVATIVSGDPCMQIF-GKAAARVNDSLSCGCKLLPQQNLVVQDNGGGAASSAAKSSSAPI
SQKQPATDSFVKDEYENYYIEQNTTKYVKFTNGIFPYDEDKKNLFGAISQAVSGVCTFIVTYILKGQELFVT
VSMLPPTLSGDATIFPYATLDLVHNNQSLGK---TRLEKGKG---VWSTENSKEPVGQCKVTLPKPDLSTIE
ATLTMGYTAKFDG--GTVRPNPPHTRFSFTLNSASRRKS
>KAB06_B
MTTP--------------------------------------YITI-GCPTSGGGQVISGNNMFLIDGIPVA
CVGDKATCPTHKVVATIVSGDPCMQIF-GKAAARVNDSLSCGCKLLPQQNLVVQDNGGGAASSAAKSSSAPI
SQKQPATDSFVKDEYENYYIEQNTTKYVKFTNGIFPYDEDKKNLFGAISQAVSGVCTFIVTYILKGQELFVT
VSMLPPTLSGDATIFPYATLDLVHNNQSLGK---TRLEKGKG---VWSTENSKEPVGQCKVTLPKPDLSTIE
ATLTMGYTAKFDG--GTVRPNPPHTRFSFTLNSASRRKS
>KAB07_B
MTTP--------------------------------------YITI-GCPTSGGGQVISGNNMFLIDGIPVA
CVGDKATCPTHKVVATIVSGDPCMQIF-GKAAARVNDSLSCGCKLLPQQNLVVQDNGGGAASSAAKSSSAPI
SQKQPATDSFVKDEYENYYIEQNTTKYVKFTNGIFPYDEDKKNLFGAISQAVSGVCTFIVTYILKGQELFVT
VSMLPPTLSGDATIFPYATLDLVHNNQSLGK---TRLEKGKG---VWSTENSKEPVGQCKVTLPKPDLSTIE
ATLTMGYTAKFDG--GTVRPNPPHTRFSFTLNSASRRKS
>KAB08_B
MTTP--------------------------------------YITI-GCPTSGGGQVISGNNMFLIDGIPVA
CVGDKATCPTHKVVATIVSGDPCMQIF-GKAAARVNDSLSCGCKLLPQQNLVVQDNGGGAASSAAKSSSAPI
SQKQPATDSFVKDEYENYYIEQNTTKYVKFTNGIFPYDEDKKNLFGAISQAVSGVCTFIVTYILKGQELFVT
VSMLPPTLSGDATIFPYATLDLVHNNQSLGK---TRLEKGKG---VWSTENSKEPVGQCKVTLPKPDLSTIE
ATLTMGYTAKFDG--GTVRPNPPHTRFSFTLNSASRRKS
>KBN10P02143_B
MTTP--------------------------------------YITI-GCPTSGGGQVISGNNMFLIDGIPVA
CVGDKATCPTHKVVATIVSGDPCMQIF-GKAAARVNDSLSCGCKLLPQQNLVVQDNGGGAASSAAKSSSAPI
SQKQPATDSFVKDEYENYYIEQNTTKYVKFTNGIFPYDEDKKNLFGAISQAVSGVCTFIVTYILKGQELFVT
VSMLPPTLSGDATIFPYATLDLVHNNQSLGK---TRLEKGKG---VWSTENSKEPVGQCKVTLPKPDLSTIE
ATLTMGYTAKFDG--GTVRPNPPHTRFSFTLNSASRRKS
>SAA14_B
MTTP--------------------------------------YITI-GCPTSGGGQVISGNNMFLIDGIPVA
CVGDKATCPTHKVVATIVSGDPCMQIF-GKAAARVNDSLSCGCKLLPQQNLVVQDNGGGAASSAAKSSSAPI
SQKQPATDSFVKDEYENYYIEQNTTKYVKFTNGIFPYDEDKKNLFGAISQAVSGVCTFIVTYILKGQELFVT

VSMLPPTLSGDATIFPYATLDLVHNNQSLGK---TRLEKGKG---VWSTENSKEPVGQCKVTLPKPDLSTIE
ATLTMGYTAKFDG--GTVRPNPPHTRFSFTLNSASRRKS
>SMC_Paed_Ab_BL01_B
MTTP--------------------------------------YITI-GCPTSGGGQVISGNNMFLIDGIPVA
CVGDKATCPTHKVVATIVSGDPCMQIF-GKAAARVNDSLSCGCKLLPQQNLVVQDNGGGAASSAAKSSSAPI
SQKQPATDSFVKDEYENYYIEQNTTKYVKFTNGIFPYDEDKKNLFGAISQAVSGVCTFIVTYILKGQELFVT
VSMLPPTLSGDATIFPYATLDLVHNNQSLGK---TRLEKGKG---VWSTENSKEPVGQCKVTLPKPDLSTIE
ATLTMGYTAKFDG--GTVRPNPPHTRFSFTLNSASRRKS
>SSA12_B
MTTP--------------------------------------YITI-GCPTSGGGQVISGNNMFLIDGIPVA
CVGDKATCPTHKVVATIVSGDPCMQIF-GKAAARVNDSLSCGCKLLPQQNLVVQDNGGGAASSAAKSSSAPI
SQKQPATDSFVKDEYENYYIEQNTTKYVKFTNGIFPYDEDKKNLFGAISQAVSGVCTFIVTYILKGQELFVT
VSMLPPTLSGDATIFPYATLDLVHNNQSLGK---TRLEKGKG---VWSTENSKEPVGQCKVTLPKPDLSTIE
ATLTMGYTAKFDG--GTVRPNPPHTRFSFTLNSASRRKS
>SSA6_B
MTTP--------------------------------------YITI-GCPTSGGGQVISGNNMFLIDGIPVA
CVGDKATCPTHKVVATIVSGDPCMQIF-GKAAARVNDSLSCGCKLLPQQNLVVQDNGGGAASSAAKSSSAPI
SQKQPATDSFVKDEYENYYIEQNTTKYVKFTNGIFPYDEDKKNLFGAISQAVSGVCTFIVTYILKGQELFVT
VSMLPPTLSGDATIFPYATLDLVHNNQSLGK---TRLEKGKG---VWSTENSKEPVGQCKVTLPKPDLSTIE
ATLTMGYTAKFDG--GTVRPNPPHTRFSFTLNSASRRKS
>SSMA17_B
MTTP--------------------------------------YITI-GCPTSGGGQVISGNNMFLIDGIPVA
CVGDKATCPTHKVVATIVSGDPCMQIF-GKAAARVNDSLSCGCKLLPQQNLVVQDNGGGAASSAAKSSSAPI
SQKQPATDSFVKDEYENYYIEQNTTKYVKFTNGIFPYDEDKKNLFGAISQAVSGVCTFIVTYILKGQELFVT
VSMLPPTLSGDATIFPYATLDLVHNNQSLGK---TRLEKGKG---VWSTENSKEPVGQCKVTLPKPDLSTIE
ATLTMGYTAKFDG--GTVRPNPPHTRFSFTLNSASRRKS
>USA2_B
MTTP--------------------------------------YITI-GCPTSGGGQVISGNNMFLIDGIPVA
CVGDKATCPTHKVVATIVSGDPCMQIF-GKAAARVNDSLSCGCKLLPQQNLVVQDNGGGAASSAAKSSSAPI
SQKQPATDSFVKDEYENYYIEQNTTKYVKFTNGIFPYDEDKKNLFGAISQAVSGVCTFIVTYILKGQELFVT
VSMLPPTLSGDATIFPYATLDLVHNNQSLGK---TRLEKGKG---VWSTENSKEPVGQCKVTLPKPDLSTIE
ATLTMGYTAKFDG--GTVRPNPPHTRFSFTLNSASRRKS
>WCHAB005133_B

MTTP--------------------------------------YITI-GCPTSGGGQVISGNNMFLIDGIPVA
CVGDKATCPTHKVVATIVSGDPCMQIF-GKAAARVNDSLSCGCKLLPQQNLVVQDNGGGAASSAAKSSSAPI
SQKQPATDSFVKDEYENYYIEQNTTKYVKFTNGIFPYDEDKKNLFGAISQAVSGVCTFIVTYILKGQELFVT
VSMLPPTLSGDATIFPYATLDLVHNNQSLGK---TRLEKGKG---VWSTENSKEPVGQCKVTLPKPDLSTIE
ATLTMGYTAKFDG--GTVRPNPPHTRFSFTLNSASRRKS
>XDR-BJ83_B
MTTP--------------------------------------YITI-GCPTSGGGQVISGNNMFLIDGIPVA
CVGDKATCPTHKVVATIVSGDPCMQIF-GKAAARVNDSLSCGCKLLPQQNLVVQDNGGGAASSAAKSSSAPI
SQKQPATDSFVKDEYENYYIEQNTTKYVKFTNGIFPYDEDKKNLFGAISQAVSGVCTFIVTYILKGQELFVT
VSMLPPTLSGDATIFPYATLDLVHNNQSLGK---TRLEKGKG---VWSTENSKEPVGQCKVTLPKPDLSTIE
ATLTMGYTAKFDG--GTVRPNPPHTRFSFTLNSASRRKS
>XH386_B
MTTP--------------------------------------YITI-GCPTSGGGQVISGNNMFLIDGIPVA
CVGDKATCPTHKVVATIVSGDPCMQIF-GKAAARVNDSLSCGCKLLPQQNLVVQDNGGGAASSAAKSSSAPI
SQKQPATDSFVKDEYENYYIEQNTTKYVKFTNGIFPYDEDKKNLFGAISQAVSGVCTFIVTYILKGQELFVT
VSMLPPTLSGDATIFPYATLDLVHNNQSLGK---TRLEKGKG---VWSTENSKEPVGQCKVTLPKPDLSTIE
ATLTMGYTAKFDG--GTVRPNPPHTRFSFTLNSASRRKS
>XH857_B
MTTP--------------------------------------YITI-GCPTSGGGQVISGNNMFLIDGIPVA
CVGDKATCPTHKVVATIVSGDPCMQIF-GKAAARVNDSLSCGCKLLPQQNLVVQDNGGGAASSAAKSSSAPI
SQKQPATDSFVKDEYENYYIEQNTTKYVKFTNGIFPYDEDKKNLFGAISQAVSGVCTFIVTYILKGQELFVT
VSMLPPTLSGDATIFPYATLDLVHNNQSLGK---TRLEKGKG---VWSTENSKEPVGQCKVTLPKPDLSTIE
ATLTMGYTAKFDG--GTVRPNPPHTRFSFTLNSASRRKS
>XH859_B
MTTP--------------------------------------YITI-GCPTSGGGQVISGNNMFLIDGIPVA
CVGDKATCPTHKVVATIVSGDPCMQIF-GKAAARVNDSLSCGCKLLPQQNLVVQDNGGGAASSAAKSSSAPI
SQKQPATDSFVKDEYENYYIEQNTTKYVKFTNGIFPYDEDKKNLFGAISQAVSGVCTFIVTYILKGQELFVT
VSMLPPTLSGDATIFPYATLDLVHNNQSLGK---TRLEKGKG---VWSTENSKEPVGQCKVTLPKPDLSTIE
ATLTMGYTAKFDG--GTVRPNPPHTRFSFTLNSASRRKS
>XH860_B
MTTP--------------------------------------YITI-GCPTSGGGQVISGNNMFLIDGIPVA
CVGDKATCPTHKVVATIVSGDPCMQIF-GKAAARVNDSLSCGCKLLPQQNLVVQDNGGGAASSAAKSSSAPI
SQKQPATDSFVKDEYENYYIEQNTTKYVKFTNGIFPYDEDKKNLFGAISQAVSGVCTFIVTYILKGQELFVT

VSMLPPTLSGDATIFPYATLDLVHNNQSLGK---TRLEKGKG---VWSTENSKEPVGQCKVTLPKPDLSTIE
ATLTMGYTAKFDG--GTVRPNPPHTRFSFTLNSASRRKS
>YU-R612_B
MTTP---------------------------------------YITI-GCPTSGGGQVISGNNMFLIDGIPVA
CVGDKATCPTHKVVATIVSGDPCMQIF-GKAAARVNDSLSCGCKLLPQQNLVVQDNGGGAASSAAKSSSAPI
SQKQPATDSFVKDEYENYYIEQNTTKYVKFTNGIFPYDEDKKNLFGAISQAVSGVCTFIVTYILKGQELFVT
VSMLPPTLSGDATIFPYATLDLVHNNQSLGK---TRLEKGKG---VWSTENSKEPVGQCKVTLPKPDLSTIE
ATLTMGYTAKFDG--GTVRPNPPHTRFSFTLNSASRRKS
>1656-2_B
LTTP---------------------------------------YITI-GCPTSGGGQVISGNNMFLIDGIPVA
CVGDKATCPTHKVVATIVSGDPCMQIF-GKAAARVNDSLSCGCKLLPQQNLVVQDNGGGAASSAAKSSSAPI
SQKQPATDSFVKDEYENYYIEQNTTKYVKFTNGIFPYDEDKKNLFGAISQAVSGVCTFIVTYILKGQELFVT
VSMLPPTLSGDATIFPYATLDLVHNNQSLGK---TRLEKGKG---VWSTENSKEPVGQCKVTLPKPDLSTIE
ATLTMGYTAKFDG--GTVRPNPPHTRFSFTLNSASRRKS
>AC30_B
LTTP---------------------------------------YITI-GCPTSGGGQVISGNNMFLIDGIPVA
CVGDKATCPTHKVVATIVSGDPCMQIF-GKAAARVNDSLSCGCKLLPQQNLVVQDNGGGAASSAAKSSSAPI
SQKQPATDSFVKDEYENYYIEQNTTKYVKFTNGIFPYDEDKKNLFGAISQAVSGVCTFIVTYILKGQELFVT
VSMLPPTLSGDATIFPYATLDLVHNNQSLGK---TRLEKGKG---VWSTENSKEPVGQCKVTLPKPDLSTIE
ATLTMGYTAKFDG--GTVRPNPPHTRFSFTLNSASRRKS
>ACICU_B
LTTP---------------------------------------YITI-GCPTSGGGQVISGNNMFLIDGIPVA
CVGDKATCPTHKVVATIVSGDPCMQIF-GKAAARVNDSLSCGCKLLPQQNLVVQDNGGGAASSAAKSSSAPI
SQKQPATDSFVKDEYENYYIEQNTTKYVKFTNGIFPYDEDKKNLFGAISQAVSGVCTFIVTYILKGQELFVT
VSMLPPTLSGDATIFPYATLDLVHNNQSLGK---TRLEKGKG---VWSTENSKEPVGQCKVTLPKPDLSTIE
ATLTMGYTAKFDG--GTVRPNPPHTRFSFTLNSASRRKS
>BJAB07104_B
LTTP---------------------------------------YITI-GCPTSGGGQVISGNNMFLIDGIPVA
CVGDKATCPTHKVVATIVSGDPCMQIF-GKAAARVNDSLSCGCKLLPQQNLVVQDNGGGAASSAAKSSSAPI
SQKQPATDSFVKDEYENYYIEQNTTKYVKFTNGIFPYDEDKKNLFGAISQAVSGVCTFIVTYILKGQELFVT
VSMLPPTLSGDATIFPYATLDLVHNNQSLGK---TRLEKGKG---VWSTENSKEPVGQCKVTLPKPDLSTIE
ATLTMGYTAKFDG--GTVRPNPPHTRFSFTLNSASRRKS
>BJAB0868_B

LTTP--------------------------------------YITI-GCPTSGGGQVISGNNMFLIDGIPVA
CVGDKATCPTHKVVATIVSGDPCMQIF-GKAAARVNDSLSCGCKLLPQQNLVVQDNGGGAASSAAKSSSAPI
SQKQPATDSFVKDEYENYYIEQNTTKYVKFTNGIFPYDEDKKNLFGAISQAVSGVCTFIVTYILKGQELFVT
VSMLPPTLSGDATIFPYATLDLVHNNQSLGK---TRLEKGKG---VWSTENSKEPVGQCKVTLPKPDLSTIE
ATLTMGYTAKFDG--GTVRPNPPHTRFSFTLNSASRRKS
>MDR-TJ_B
LTTP--------------------------------------YITI-GCPTSGGGQVISGNNMFLIDGIPVA
CVGDKATCPTHKVVATIVSGDPCMQIF-GKAAARVNDSLSCGCKLLPQQNLVVQDNGGGAASSAAKSSSAPI
SQKQPATDSFVKDEYENYYIEQNTTKYVKFTNGIFPYDEDKKNLFGAISQAVSGVCTFIVTYILKGQELFVT
VSMLPPTLSGDATIFPYATLDLVHNNQSLGK---TRLEKGKG---VWSTENSKEPVGQCKVTLPKPDLSTIE
ATLTMGYTAKFDG--GTVRPNPPHTRFSFTLNSASRRKS
>MDR-ZJ06_B
LTTP--------------------------------------YITI-GCPTSGGGQVISGNNMFLIDGIPVA
CVGDKATCPTHKVVATIVSGDPCMQIF-GKAAARVNDSLSCGCKLLPQQNLVVQDNGGGAASSAAKSSSAPI
SQKQPATDSFVKDEYENYYIEQNTTKYVKFTNGIFPYDEDKKNLFGAISQAVSGVCTFIVTYILKGQELFVT
VSMLPPTLSGDATIFPYATLDLVHNNQSLGK---TRLEKGKG---VWSTENSKEPVGQCKVTLPKPDLSTIE
ATLTMGYTAKFDG--GTVRPNPPHTRFSFTLNSASRRKS
>NCGM237_B
LTTP--------------------------------------YITI-GCPTSGGGQVISGNNMFLIDGIPVA
CVGDKATCPTHKVVATIVSGDPCMQIF-GKAAARVNDSLSCGCKLLPQQNLVVQDNGGGAASSAAKSSSAPI
SQKQPATDSFVKDEYENYYIEQNTTKYVKFTNGIFPYDEDKKNLFGAISQAVSGVCTFIVTYILKGQELFVT
VSMLPPTLSGDATIFPYATLDLVHNNQSLGK---TRLEKGKG---VWSTENSKEPVGQCKVTLPKPDLSTIE
ATLTMGYTAKFDG--GTVRPNPPHTRFSFTLNSASRRKS
>TCDC-0715_B
LTTP--------------------------------------YITI-GCPTSGGGQVISGNNMFLIDGIPVA
CVGDKATCPTHKVVATIVSGDPCMQIF-GKAAARVNDSLSCGCKLLPQQNLVVQDNGGGAASSAAKSSSAPI
SQKQPATDSFVKDEYENYYIEQNTTKYVKFTNGIFPYDEDKKNLFGAISQAVSGVCTFIVTYILKGQELFVT
VSMLPPTLSGDATIFPYATLDLVHNNQSLGK---TRLEKGKG---VWSTENSKEPVGQCKVTLPKPDLSTIE
ATLTMGYTAKFDG--GTVRPNPPHTRFSFTLNSASRRKS
>AB030_I
MATP--------------------------------------YITI-GCPTSGGGQVISGNSMFLIDGIPVA
CVGDKATCPTHKVVATIVSGDPYMQIF-GKAAARVNDSLSCGCKLLPQQNLVVQDNGGGSAQSLPAS-----
--KAQTQDSFVEDKSKEY----------------------------------------GIQFQLKDEKTQKV

FSEIPYSI-------------IYKKDGKVET--------------GWTDKEGKTHVINAD------------
--------------------SPDEVEFQTIDASKPLPPL
>AR_0088_I
MATP--------------------------------------YITI-GCPTSGGGQVISGNSMFLIDGIPVA
CVGDKATCPTHKVVATIVSGDPYMQIF-GKAAARVNDSLSCGCKLLPQQNLVVQDNGGGSAQSLPAS-----
--KAQTQDSFVEDKSKEY----------------------------------------GIQFQLKDEKTQKV
FSEIPYSI-------------IYKKDGKVET--------------GWTDKEGKTHVINAD------------
--------------------SPDEVEFQTIDASKPLPPL
>HWAB8_I
MATP--------------------------------------YITI-GCPTSGGGQVISGNSMFLIDGIPVA
CVGDKATCPTHKVVATIVSGDPYMQIF-GKAAARVNDSLSCGCKLLPQQNLVVQDNGGGSAQSLPAS-----
--KAQTQDSFVEDKSKEY----------------------------------------GIQFQLKDEKTQKV
FSEIPYSI-------------IYKKDGKVET--------------GWTDKEGKTHVINAD------------
--------------------SPDEVEFQTIDASKPLPPL
>B8342_G
MATP--------------------------------------YITI-GCPTSGGGQVISGNSMFLIDGIPVA
CVGDKATCPTHKVVATIVSGDPYMQIF-GKAAARVNDSLSCGCKLLPQQNLVVQDNGGGSAQSSPVS-----
--KAQTQDSFVENKSKDIHKIQFK----------LVDDETKKPLTELMYEI----------YSKDKGQLLV-
----------------------------------------QG---YTDKSG---------------------
--LTALYESNYTAESVEVILVDLSKPIEPI---------
>A388_J
MATP--------------------------------------YITI-GCPTTGGGQVISGNSMFLIDGIPVA
CVGDKATCPTHKVVATIVSGDPYMQIF-GKAAARVNDSLSCGCKLLPQQNLVVQDNGGGAVQGSQASNATQD
SFMPQTDEHGIKFQLKDQETGK-------------PLAQQYFKLQGPDGSEIEG------------------
----------------------FTDENGFTE----LIKTGTE---------------AKEIDLTTFDLSQPM
AKWE-----------------------------------
>A85_J
MATP--------------------------------------YITI-GCPTTGGGQVISGNSMFLIDGIPVA
CVGDKATCPTHKVVATIVSGDPYMQIF-GKAAARVNDSLSCGCKLLPQQNLVVQDNGGGAVQGSQASNATQD
SFMPQTDEHGIKFQLKDQETGK-------------PLAQQYFKLQGPDGSEIEG------------------
----------------------FTDENGFTE----LIKTGTE---------------AKEIDLTTFDLSQPM
AKWE-----------------------------------
>AB0057_J

MATP--------------------------------------YITI-GCPTTGGGQVISGNSMFLIDGIPVA
CVGDKATCPTHKVVATIVSGDPYMQIF-GKAAARVNDSLSCGCKLLPQQNLVVQDNGGGAVQGSQASNATQD
SFMPQTDEHGIKFQLKDQETGK-------------PLAQQYFKLQGPDGSEIEG------------------
----------------------FTDENGFTE----LIKTGTE---------------AKEIDLTTFDLSQPM
AKWE-----------------------------------
>15A34_J
MATP--------------------------------------YITI-GCPTTGGGQVISGNSMFLIDGIPVA
CVGDKATCPTHKVVATIVSGDPYMQIF-GKAAARVNDSLSCGCKLLPQQNLVVQDNGGGAVQGSQASNATQD
SFMPQTDEHGIKFQLKDQETGK-------------PLAQQYFKLKGPDGSEIEG------------------
----------------------FTDKNGFTE----LIKTGTE---------------AKEIDLTTFDLSQPM
AKWE-----------------------------------
>LAC-4_A
MATP--------------------------------------YITI-GCPTTGGGQVISGNSLFQIDGIPVA
CTGDKATCPTHKVVATIVSGDPCMQIF-GKAAARVNDSLSCGCKLLPKQNLVVQDNGSGAASSAAKSSPAPM
SQKQPATDSFVKDEYENYYIEQNKTTMVPFKTMLMPYDQDRTNLFGVMSQMVSGACNFEVTHQVKKDQLFVT
ATLLPPTVRADATIIPRAVLRLFKKDKQISD--TITLKVGKG---YWNTANDKQPVGSCEVKLPAPDLEVIK
AKLTMKYDAKFDG--GVVVTSPPDVTYEFTITSAARRKA
>6200_A
MATP--------------------------------------YITI-GCPTTGGGQVISGNSLFLIDGIAVA
CVGDKATCPTHKIVATIVSGDPNMQIF-GKAAARVNDSLSCGCKLLPKQHLVVQDNGGGSASSAANSSPAPM
SQKQPTTDSFVKDEYENYYIEQNKTTMVPFKTMLMPYDQDRTNLFGVMSQIVSGACNFEVTHRVKKDQLFVT
ATLLPPTVRADATIIPRAVLRLFKKDKQISD--TITLKVGKG---YWNTANDKQPVGSCEIKLPAPDLEVIK
AKLTMKYDAKFDG--GVVVTSPPDVTYEFTITSAARRKA
>3027STDY5784958_A
MATP--------------------------------------YITI-GCPTTGGGQVISGNSLFLIDGIAVA
CVGDKATCPTHKIVATIVSGDPNMQIF-GKAAARVNDSLSCGCKLLPKQHLVVQDNGGGSASSAANSSPAPM
SQKQPTTDSFVKDEYENYYIEQNKTTMVPFKTMLMPYDQDRTNLFGVMSQIVSGACNFEVTHRVKKDQLFVT
ATLLPPTVRADATIIPRAVLRLFKKDKQISD--TITLKVGKG---YWNTANDKQPVGSCEIKLPAPDLEVIK
AKLTMKYDAKFDG--GVVVTSPPDVTYEFTITSAARRKA
>Ab4568_A
MATP--------------------------------------YITI-GCPTTGGGQVISGNSLFLIDGIAVA
CVGDKATCPTHKIVATIVSGDPNMQIF-GKAAARVNDSLSCGCKLLPKQHLVVQDNGGGSASSAANSSPAPM
SQKQPTTDSFVKDEYENYYIEQNKTTMVPFKTMLMPYDQDRTNLFGVMSQIVSGACNFEVTHRVKKDQLFVT

ATLLPPTVRADATIIPRAVLRLFKKDKQISD--TITLKVGKG---YWNTANDKQPVGSCEIKLPAPDLEVIK
AKLTMKYDAKFDG--GVVVTSPPDVTYEFTITSAARRKA
>Ab4977_A
MATP--------------------------------------YITI-GCPTTGGGQVISGNSLFLIDGIAVA
CVGDKATCPTHKIVATIVSGDPNMQIF-GKAAARVNDSLSCGCKLLPKQHLVVQDNGGGSASSAANSSPAPM
SQKQPTTDSFVKDEYENYYIEQNKTTMVPFKTMLMPYDQDRTNLFGVMSQIVSGACNFEVTHRVKKDQLFVT
ATLLPPTVRADATIIPRAVLRLFKKDKQISD--TITLKVGKG---YWNTANDKQPVGSCEIKLPAPDLEVIK
AKLTMKYDAKFDG--GVVVTSPPDVTYEFTITSAARRKA
>AF-673_A
MATP--------------------------------------YITI-GCPTTGGGQVISGNSLFLIDGIAVA
CVGDKATCPTHKIVATIVSGDPNMQIF-GKAAARVNDSLSCGCKLLPKQHLVVQDNGGGSASSAANSSPAPM
SQKQPTTDSFVKDEYENYYIEQNKTTMVPFKTMLMPYDQDRTNLFGVMSQIVSGACNFEVTHRVKKDQLFVT
ATLLPPTVRADATIIPRAVLRLFKKDKQISD--TITLKVGKG---YWNTANDKQPVGSCEIKLPAPDLEVIK
AKLTMKYDAKFDG--GVVVTSPPDVTYEFTITSAARRKA
>AR_0102_A
MATP--------------------------------------YITI-GCPTTGGGQVISGNSLFLIDGIAVA
CVGDKATCPTHKIVATIVSGDPNMQIF-GKAAARVNDSLSCGCKLLPKQHLVVQDNGGGSASSAANSSPAPM
SQKQPTTDSFVKDEYENYYIEQNKTTMVPFKTMLMPYDQDRTNLFGVMSQIVSGACNFEVTHRVKKDQLFVT
ATLLPPTVRADATIIPRAVLRLFKKDKQISD--TITLKVGKG---YWNTANDKQPVGSCEIKLPAPDLEVIK
AKLTMKYDAKFDG--GVVVTSPPDVTYEFTITSAARRKA
>CBA7_A
MATP--------------------------------------YITI-GCPTTGGGQVISGNSLFLIDGIAVA
CVGDKATCPTHKIVATIVSGDPNMQIF-GKAAARVNDSLSCGCKLLPKQHLVVQDNGGGSASSAANSSPAPM
SQKQPTTDSFVKDEYENYYIEQNKTTMVPFKTMLMPYDQDRTNLFGVMSQIVSGACNFEVTHRVKKDQLFVT
ATLLPPTVRADATIIPRAVLRLFKKDKQISD--TITLKVGKG---YWNTANDKQPVGSCEIKLPAPDLEVIK
AKLTMKYDAKFDG--GVVVTSPPDVTYEFTITSAARRKA
>CMC-CR-MDR-Ab4_A
MATP--------------------------------------YITI-GCPTTGGGQVISGNSLFLIDGIAVA
CVGDKATCPTHKIVATIVSGDPNMQIF-GKAAARVNDSLSCGCKLLPKQHLVVQDNGGGSASSAANSSPAPM
SQKQPTTDSFVKDEYENYYIEQNKTTMVPFKTMLMPYDQDRTNLFGVMSQIVSGACNFEVTHRVKKDQLFVT
ATLLPPTVRADATIIPRAVLRLFKKDKQISD--TITLKVGKG---YWNTANDKQPVGSCEIKLPAPDLEVIK
AKLTMKYDAKFDG--GVVVTSPPDVTYEFTITSAARRKA
>CMC-CR-MDR-Ab66_A

MATP--------------------------------------YITI-GCPTTGGGQVISGNSLFLIDGIAVA
CVGDKATCPTHKIVATIVSGDPNMQIF-GKAAARVNDSLSCGCKLLPKQHLVVQDNGGGSASSAANSSPAPM
SQKQPTTDSFVKDEYENYYIEQNKTTMVPFKTMLMPYDQDRTNLFGVMSQIVSGACNFEVTHRVKKDQLFVT
ATLLPPTVRADATIIPRAVLRLFKKDKQISD--TITLKVGKG---YWNTANDKQPVGSCEIKLPAPDLEVIK
AKLTMKYDAKFDG--GVVVTSPPDVTYEFTITSAARRKA
>CMC-MDR-Ab59_A
MATP--------------------------------------YITI-GCPTTGGGQVISGNSLFLIDGIAVA
CVGDKATCPTHKIVATIVSGDPNMQIF-GKAAARVNDSLSCGCKLLPKQHLVVQDNGGGSASSAANSSPAPM
SQKQPTTDSFVKDEYENYYIEQNKTTMVPFKTMLMPYDQDRTNLFGVMSQIVSGACNFEVTHRVKKDQLFVT
ATLLPPTVRADATIIPRAVLRLFKKDKQISD--TITLKVGKG---YWNTANDKQPVGSCEIKLPAPDLEVIK
AKLTMKYDAKFDG--GVVVTSPPDVTYEFTITSAARRKA
>JBA13_A
MATP--------------------------------------YITI-GCPTTGGGQVISGNSLFLIDGIAVA
CVGDKATCPTHKIVATIVSGDPNMQIF-GKAAARVNDSLSCGCKLLPKQHLVVQDNGGGSASSAANSSPAPM
SQKQPTTDSFVKDEYENYYIEQNKTTMVPFKTMLMPYDQDRTNLFGVMSQIVSGACNFEVTHRVKKDQLFVT
ATLLPPTVRADATIIPRAVLRLFKKDKQISD--TITLKVGKG---YWNTANDKQPVGSCEIKLPAPDLEVIK
AKLTMKYDAKFDG--GVVVTSPPDVTYEFTITSAARRKA
>KAB02_A
MATP--------------------------------------YITI-GCPTTGGGQVISGNSLFLIDGIAVA
CVGDKATCPTHKIVATIVSGDPNMQIF-GKAAARVNDSLSCGCKLLPKQHLVVQDNGGGSASSAANSSPAPM
SQKQPTTDSFVKDEYENYYIEQNKTTMVPFKTMLMPYDQDRTNLFGVMSQIVSGACNFEVTHRVKKDQLFVT
ATLLPPTVRADATIIPRAVLRLFKKDKQISD--TITLKVGKG---YWNTANDKQPVGSCEIKLPAPDLEVIK
AKLTMKYDAKFDG--GVVVTSPPDVTYEFTITSAARRKA
>KAB04_A
MATP--------------------------------------YITI-GCPTTGGGQVISGNSLFLIDGIAVA
CVGDKATCPTHKIVATIVSGDPNMQIF-GKAAARVNDSLSCGCKLLPKQHLVVQDNGGGSASSAANSSPAPM
SQKQPTTDSFVKDEYENYYIEQNKTTMVPFKTMLMPYDQDRTNLFGVMSQIVSGACNFEVTHRVKKDQLFVT
ATLLPPTVRADATIIPRAVLRLFKKDKQISD--TITLKVGKG---YWNTANDKQPVGSCEIKLPAPDLEVIK
AKLTMKYDAKFDG--GVVVTSPPDVTYEFTITSAARRKA
>KAB05_A
MATP--------------------------------------YITI-GCPTTGGGQVISGNSLFLIDGIAVA
CVGDKATCPTHKIVATIVSGDPNMQIF-GKAAARVNDSLSCGCKLLPKQHLVVQDNGGGSASSAANSSPAPM
SQKQPTTDSFVKDEYENYYIEQNKTTMVPFKTMLMPYDQDRTNLFGVMSQIVSGACNFEVTHRVKKDQLFVT

ATLLPPTVRADATIIPRAVLRLFKKDKQISD--TITLKVGKG---YWNTANDKQPVGSCEIKLPAPDLEVIK
AKLTMKYDAKFDG--GVVVTSPPDVTYEFTITSAARRKA
>KAB06_A
MATP--------------------------------------YITI-GCPTTGGGQVISGNSLFLIDGIAVA
CVGDKATCPTHKIVATIVSGDPNMQIF-GKAAARVNDSLSCGCKLLPKQHLVVQDNGGGSASSAANSSPAPM
SQKQPTTDSFVKDEYENYYIEQNKTTMVPFKTMLMPYDQDRTNLFGVMSQIVSGACNFEVTHRVKKDQLFVT
ATLLPPTVRADATIIPRAVLRLFKKDKQISD--TITLKVGKG---YWNTANDKQPVGSCEIKLPAPDLEVIK
AKLTMKYDAKFDG--GVVVTSPPDVTYEFTITSAARRKA
>KAB07_A
MATP--------------------------------------YITI-GCPTTGGGQVISGNSLFLIDGIAVA
CVGDKATCPTHKIVATIVSGDPNMQIF-GKAAARVNDSLSCGCKLLPKQHLVVQDNGGGSASSAANSSPAPM
SQKQPTTDSFVKDEYENYYIEQNKTTMVPFKTMLMPYDQDRTNLFGVMSQIVSGACNFEVTHRVKKDQLFVT
ATLLPPTVRADATIIPRAVLRLFKKDKQISD--TITLKVGKG---YWNTANDKQPVGSCEIKLPAPDLEVIK
AKLTMKYDAKFDG--GVVVTSPPDVTYEFTITSAARRKA
>KAB08_A
MATP--------------------------------------YITI-GCPTTGGGQVISGNSLFLIDGIAVA
CVGDKATCPTHKIVATIVSGDPNMQIF-GKAAARVNDSLSCGCKLLPKQHLVVQDNGGGSASSAANSSPAPM
SQKQPTTDSFVKDEYENYYIEQNKTTMVPFKTMLMPYDQDRTNLFGVMSQIVSGACNFEVTHRVKKDQLFVT
ATLLPPTVRADATIIPRAVLRLFKKDKQISD--TITLKVGKG---YWNTANDKQPVGSCEIKLPAPDLEVIK
AKLTMKYDAKFDG--GVVVTSPPDVTYEFTITSAARRKA
>MDR-ZJ06_A
MATP--------------------------------------YITI-GCPTTGGGQVISGNSLFLIDGIAVA
CVGDKATCPTHKIVATIVSGDPNMQIF-GKAAARVNDSLSCGCKLLPKQHLVVQDNGGGSASSAANSSPAPM
SQKQPTTDSFVKDEYENYYIEQNKTTMVPFKTMLMPYDQDRTNLFGVMSQIVSGACNFEVTHRVKKDQLFVT
ATLLPPTVRADATIIPRAVLRLFKKDKQISD--TITLKVGKG---YWNTANDKQPVGSCEIKLPAPDLEVIK
AKLTMKYDAKFDG--GVVVTSPPDVTYEFTITSAARRKA
>SAA14_A
MATP--------------------------------------YITI-GCPTTGGGQVISGNSLFLIDGIAVA
CVGDKATCPTHKIVATIVSGDPNMQIF-GKAAARVNDSLSCGCKLLPKQHLVVQDNGGGSASSAANSSPAPM
SQKQPTTDSFVKDEYENYYIEQNKTTMVPFKTMLMPYDQDRTNLFGVMSQIVSGACNFEVTHRVKKDQLFVT
ATLLPPTVRADATIIPRAVLRLFKKDKQISD--TITLKVGKG---YWNTANDKQPVGSCEIKLPAPDLEVIK
AKLTMKYDAKFDG--GVVVTSPPDVTYEFTITSAARRKA
>SSA12_A

MATP--------------------------------------YITI-GCPTTGGGQVISGNSLFLIDGIAVA
CVGDKATCPTHKIVATIVSGDPNMQIF-GKAAARVNDSLSCGCKLLPKQHLVVQDNGGGSASSAANSSPAPM
SQKQPTTDSFVKDEYENYYIEQNKTTMVPFKTMLMPYDQDRTNLFGVMSQIVSGACNFEVTHRVKKDQLFVT
ATLLPPTVRADATIIPRAVLRLFKKDKQISD--TITLKVGKG---YWNTANDKQPVGSCEIKLPAPDLEVIK
AKLTMKYDAKFDG--GVVVTSPPDVTYEFTITSAARRKA
>SSMA17_A
MATP--------------------------------------YITI-GCPTTGGGQVISGNSLFLIDGIAVA
CVGDKATCPTHKIVATIVSGDPNMQIF-GKAAARVNDSLSCGCKLLPKQHLVVQDNGGGSASSAANSSPAPM
SQKQPTTDSFVKDEYENYYIEQNKTTMVPFKTMLMPYDQDRTNLFGVMSQIVSGACNFEVTHRVKKDQLFVT
ATLLPPTVRADATIIPRAVLRLFKKDKQISD--TITLKVGKG---YWNTANDKQPVGSCEIKLPAPDLEVIK
AKLTMKYDAKFDG--GVVVTSPPDVTYEFTITSAARRKA
>TYTH-1_A
MATP--------------------------------------YITI-GCPTTGGGQVISGNSLFLIDGIAVA
CVGDKATCPTHKIVATIVSGDPNMQIF-GKAAARVNDSLSCGCKLLPKQHLVVQDNGGGSASSAANSSPAPM
SQKQPTTDSFVKDEYENYYIEQNKTTMVPFKTMLMPYDQDRTNLFGVMSQIVSGACNFEVTHRVKKDQLFVT
ATLLPPTVRADATIIPRAVLRLFKKDKQISD--TITLKVGKG---YWNTANDKQPVGSCEIKLPAPDLEVIK
AKLTMKYDAKFDG--GVVVTSPPDVTYEFTITSAARRKA
>WKA02_A
MATP--------------------------------------YITI-GCPTTGGGQVISGNSLFLIDGIAVA
CVGDKATCPTHKIVATIVSGDPNMQIF-GKAAARVNDSLSCGCKLLPKQHLVVQDNGGGSASSAANSSPAPM
SQKQPTTDSFVKDEYENYYIEQNKTTMVPFKTMLMPYDQDRTNLFGVMSQIVSGACNFEVTHRVKKDQLFVT
ATLLPPTVRADATIIPRAVLRLFKKDKQISD--TITLKVGKG---YWNTANDKQPVGSCEIKLPAPDLEVIK
AKLTMKYDAKFDG--GVVVTSPPDVTYEFTITSAARRKA
>XDR-BJ83_A
MATP--------------------------------------YITI-GCPTTGGGQVISGNSLFLIDGIAVA
CVGDKATCPTHKIVATIVSGDPNMQIF-GKAAARVNDSLSCGCKLLPKQHLVVQDNGGGSASSAANSSPAPM
SQKQPTTDSFVKDEYENYYIEQNKTTMVPFKTMLMPYDQDRTNLFGVMSQIVSGACNFEVTHRVKKDQLFVT
ATLLPPTVRADATIIPRAVLRLFKKDKQISD--TITLKVGKG---YWNTANDKQPVGSCEIKLPAPDLEVIK
AKLTMKYDAKFDG--GVVVTSPPDVTYEFTITSAARRKA
>XH386_A
MATP--------------------------------------YITI-GCPTTGGGQVISGNSLFLIDGIAVA
CVGDKATCPTHKIVATIVSGDPNMQIF-GKAAARVNDSLSCGCKLLPKQHLVVQDNGGGSASSAANSSPAPM
SQKQPTTDSFVKDEYENYYIEQNKTTMVPFKTMLMPYDQDRTNLFGVMSQIVSGACNFEVTHRVKKDQLFVT

ATLLPPTVRADATIIPRAVLRLFKKDKQISD--TITLKVGKG---YWNTANDKQPVGSCEIKLPAPDLEVIK
AKLTMKYDAKFDG--GVVVTSPPDVTYEFTITSAARRKA
>XH856_A
MATP--------------------------------------YITI-GCPTTGGGQVISGNSLFLIDGIAVA
CVGDKATCPTHKIVATIVSGDPNMQIF-GKAAARVNDSLSCGCKLLPKQHLVVQDNGGGSASSAANSSPAPM
SQKQPTTDSFVKDEYENYYIEQNKTTMVPFKTMLMPYDQDRTNLFGVMSQIVSGACNFEVTHRVKKDQLFVT
ATLLPPTVRADATIIPRAVLRLFKKDKQISD--TITLKVGKG---YWNTANDKQPVGSCEIKLPAPDLEVIK
AKLTMKYDAKFDG--GVVVTSPPDVTYEFTITSAARRKA
>XH857_A
MATP--------------------------------------YITI-GCPTTGGGQVISGNSLFLIDGIAVA
CVGDKATCPTHKIVATIVSGDPNMQIF-GKAAARVNDSLSCGCKLLPKQHLVVQDNGGGSASSAANSSPAPM
SQKQPTTDSFVKDEYENYYIEQNKTTMVPFKTMLMPYDQDRTNLFGVMSQIVSGACNFEVTHRVKKDQLFVT
ATLLPPTVRADATIIPRAVLRLFKKDKQISD--TITLKVGKG---YWNTANDKQPVGSCEIKLPAPDLEVIK
AKLTMKYDAKFDG--GVVVTSPPDVTYEFTITSAARRKA
>XH858_A
MATP--------------------------------------YITI-GCPTTGGGQVISGNSLFLIDGIAVA
CVGDKATCPTHKIVATIVSGDPNMQIF-GKAAARVNDSLSCGCKLLPKQHLVVQDNGGGSASSAANSSPAPM
SQKQPTTDSFVKDEYENYYIEQNKTTMVPFKTMLMPYDQDRTNLFGVMSQIVSGACNFEVTHRVKKDQLFVT
ATLLPPTVRADATIIPRAVLRLFKKDKQISD--TITLKVGKG---YWNTANDKQPVGSCEIKLPAPDLEVIK
AKLTMKYDAKFDG--GVVVTSPPDVTYEFTITSAARRKA
>XH859_A
MATP--------------------------------------YITI-GCPTTGGGQVISGNSLFLIDGIAVA
CVGDKATCPTHKIVATIVSGDPNMQIF-GKAAARVNDSLSCGCKLLPKQHLVVQDNGGGSASSAANSSPAPM
SQKQPTTDSFVKDEYENYYIEQNKTTMVPFKTMLMPYDQDRTNLFGVMSQIVSGACNFEVTHRVKKDQLFVT
ATLLPPTVRADATIIPRAVLRLFKKDKQISD--TITLKVGKG---YWNTANDKQPVGSCEIKLPAPDLEVIK
AKLTMKYDAKFDG--GVVVTSPPDVTYEFTITSAARRKA
>BJAB07104_A
LATP--------------------------------------YITI-GCPTTGGGQVISGNSLFLIDGIAVA
CVGDKATCPTHKIVATIVSGDPNMQIF-GKAAARVNDSLSCGCKLLPKQHLVVQDNGGGSASSAANSSPAPM
SQKQPTTDSFVKDEYENYYIEQNKTTMVPFKTMLMPYDQDRTNLFGVMSQIVSGACNFEVTHRVKKDQLFVT
ATLLPPTVRADATIIPRAVLRLFKKDKQISD--TITLKVGKG---YWNTANDKQPVGSCEIKLPAPDLEVIK
AKLTMKYDAKFDG--GVVVTSPPDVTYEFTITSAARRKA
>BJAB0715_A

LATP--------------------------------------YITI-GCPTTGGGQVISGNSLFLIDGIAVA
CVGDKATCPTHKIVATIVSGDPNMQIF-GKAAARVNDSLSCGCKLLPKQHLVVQDNGGGSASSAANSSPAPM
SQKQPTTDSFVKDEYENYYIEQNKTTMVPFKTMLMPYDQDRTNLFGVMSQIVSGACNFEVTHRVKKDQLFVT
ATLLPPTVRADATIIPRAVLRLFKKDKQISD--TITLKVGKG---YWNTANDKQPVGSCEIKLPAPDLEVIK
AKLTMKYDAKFDG--GVVVTSPPDVTYEFTITSAARRKA
>BJAB0868_A
LATP--------------------------------------YITI-GCPTTGGGQVISGNSLFLIDGIAVA
CVGDKATCPTHKIVATIVSGDPNMQIF-GKAAARVNDSLSCGCKLLPKQHLVVQDNGGGSASSAANSSPAPM
SQKQPTTDSFVKDEYENYYIEQNKTTMVPFKTMLMPYDQDRTNLFGVMSQIVSGACNFEVTHRVKKDQLFVT
ATLLPPTVRADATIIPRAVLRLFKKDKQISD--TITLKVGKG---YWNTANDKQPVGSCEIKLPAPDLEVIK
AKLTMKYDAKFDG--GVVVTSPPDVTYEFTITSAARRKA
>AB34299_A
MATP--------------------------------------YITI-GCPTTGGGQVISGNSLFLIDGIAVA
CVGDKATCPTHKIVATIVSGDPNMQIF-GKAAARVNDSLSCGCKLLPKQHLVVQDNGGGSASSAAKSSPAPM
SQKQPTTDSFVKDEYENYYIEQNKTTMVPFKTMLMPYDQDRTNLFGVMSQIVSGACNFEVTHRVKKDQLFVT
ATLLPPTVRADATIIPRAVLRLFKKDKQISD--TITLKVGKG---YWNTANEKQPVGSCEIKLPAPDLEVIK
AKLTMKYDAKFDG--GVVVTSPPDVTYEFTITSAARRKA
>AC29_A
MATP--------------------------------------YITI-GCPTTGGGQVISGNSLFLIDGIAVA
CVGDKATCPTHKIVATIVSGDPNMQIF-GKAAARVNDSLSCGCKLLPKQHLVVQDNGGGSASSAAKSSPAPM
SQKQPTTDSFVKDEYENYYIEQNKTTMVPFKTMLMPYDQDRTNLFGVMSQIVSGACNFEVTHRVKKDQLFVT
ATLLPPTVRADATIIPRAVLRLFKKDKQISD--TITLKVGKG---YWNTANEKQPVGSCEIKLPAPDLEVIK
AKLTMKYDAKFDG--GVVVTSPPDVTYEFTITSAARRKA
>AC30_A
MATP--------------------------------------YITI-GCPTTGGGQVISGNSLFLIDGIAVA
CVGDKATCPTHKIVATIVSGDPNMQIF-GKAAARVNDSLSCGCKLLPKQHLVVQDNGGGSASSAAKSSPAPM
SQKQPTTDSFVKDEYENYYIEQNKTTMVPFKTMLMPYDQDRTNLFGVMSQIVSGACNFEVTHRVKKDQLFVT
ATLLPPTVRADATIIPRAVLRLFKKDKQISD--TITLKVGKG---YWNTANEKQPVGSCEIKLPAPDLEVIK
AKLTMKYDAKFDG--GVVVTSPPDVTYEFTITSAARRKA
>KAB01_A
MATP--------------------------------------YITI-GCPTTGGGQVISGNSLFLIDGIAVA
CVGDKATCPTHKIVATIVSGDPNMQIF-GKAAARVNDSLSCGCKLLPKQHLVVQDNGGGSASSAAKSSPAPM
SQKQPTTDSFVKDEYENYYIEQNKTTMVPFKTMLMPYDQDRTNLFGVMSQIVSGACNFEVTHRVKKDQLFVT

ATLLPPTVRADATIIPRAVLRLFKKDKQISD--TITLKVGKG---YWNTANEKQPVGSCEIKLPAPDLEVIK
AKLTMKYDAKFDG--GVVVTSPPDVTYEFTITSAARRKA
>KAB03_A
MATP--------------------------------------YITI-GCPTTGGGQVISGNSLFLIDGIAVA
CVGDKATCPTHKIVATIVSGDPNMQIF-GKAAARVNDSLSCGCKLLPKQHLVVQDNGGGSASSAAKSSPAPM
SQKQPTTDSFVKDEYENYYIEQNKTTMVPFKTMLMPYDQDRTNLFGVMSQIVSGACNFEVTHRVKKDQLFVT
ATLLPPTVRADATIIPRAVLRLFKKDKQISD--TITLKVGKG---YWNTANEKQPVGSCEIKLPAPDLEVIK
AKLTMKYDAKFDG--GVVVTSPPDVTYEFTITSAARRKA
>AB07_A
LATP--------------------------------------YITI-GCPTTGGGQVISGNSLFLIDGIAVA
CVGDKATCPTHKIVATIVSGDPNMQIF-GKAAARVNDSLSCGCKLLPKQHLVVQDNGGGSASSAAKSSPAPM
SQKQPTTDSFVKDEYENYYIEQNKTTMVPFKTMLMPYDQDRTNLFGVMSQIVSGACNFEVTHRVKKDQLFVT
ATLLPPTVRADATIIPRAVLRLFKKDKQISD--TITLKVGKG---YWNTANEKQPVGSCEIKLPAPDLEVIK
AKLTMKYDAKFDG--GVVVTSPPDVTYEFTITSAARRKA
>SDF_M
MASP--------------------------------------YIMI-GCPTTGGGQVISGNSAFLIEGITIA
CVGDKATCPAHKTVSTIVSGDPNMQVM-GKAAARVNDSLSCGCKLLPKQSLV--------------------
------------------------------------------------------------------------
---------------------------------------NQG------------------------------
---------------------------------------
>IOMTU433_M
MASP--------------------------------------YITI-GCPTTGGGQVISGNSAFLIEGIAIA
CVGDKATCPAHKTVSTIVSGDPNMQVM-GKAAARVNDTLSCGCKLLPKQSLV--------------------
------------------------------------------------------------------------
---------------------------------------NQG------------------------------
---------------------------------------
>TYTH-1_M
MASP--------------------------------------YITI-GCPTTGGGQVISGNSAFLIEGIAIA
CVGDKATCPAHKTVSTIVSGDPNMQVM-GKAAARVNDSLSCGCKLLPKQSLV--------------------
------------------------------------------------------------------------
---------------------------------------NQG------------------------------
---------------------------------------
>15A34_M

MASP--------------------------------------YITI-GCPTTGGGQVISGNSAFLIEGIAIA
CVGDKATCPAHKTVSTIVSGDPNMQVM-GKAAARVNDSLSCGCKLLPKQSLV--------------------
------------------------------------------------------------------------
---------------------------------------NQG------------------------------
---------------------------------------
>1656-2_M
MASP--------------------------------------YITI-GCPTTGGGQVISGNSAFLIEGIAIA
CVGDKATCPAHKTVSTIVSGDPNMQVM-GKAAARVNDSLSCGCKLLPKQSLV--------------------
------------------------------------------------------------------------
---------------------------------------NQG------------------------------
---------------------------------------
>A1_M
MASP--------------------------------------YITI-GCPTTGGGQVISGNSAFLIEGIAIA
CVGDKATCPAHKTVSTIVSGDPNMQVM-GKAAARVNDSLSCGCKLLPKQSLV--------------------
------------------------------------------------------------------------
---------------------------------------NQG------------------------------
---------------------------------------
>A388_M
MASP--------------------------------------YITI-GCPTTGGGQVISGNSAFLIEGIAIA
CVGDKATCPAHKTVSTIVSGDPNMQVM-GKAAARVNDSLSCGCKLLPKQSLV--------------------
------------------------------------------------------------------------
---------------------------------------NQG------------------------------
---------------------------------------
>A85_M
MASP--------------------------------------YITI-GCPTTGGGQVISGNSAFLIEGIAIA
CVGDKATCPAHKTVSTIVSGDPNMQVM-GKAAARVNDSLSCGCKLLPKQSLV--------------------
------------------------------------------------------------------------
---------------------------------------NQG------------------------------
---------------------------------------
>AB0057_M
MASP--------------------------------------YITI-GCPTTGGGQVISGNSAFLIEGIAIA
CVGDKATCPAHKTVSTIVSGDPNMQVM-GKAAARVNDSLSCGCKLLPKQSLV--------------------
------------------------------------------------------------------------

---------------------------------------NQG------------------------------
---------------------------------------
>AB07_M
MASP--------------------------------------YITI-GCPTTGGGQVISGNSAFLIEGIAIA
CVGDKATCPAHKTVSTIVSGDPNMQVM-GKAAARVNDSLSCGCKLLPKQSLV--------------------
------------------------------------------------------------------------
---------------------------------------NQG------------------------------
---------------------------------------
>AB307-0294_M
MASP--------------------------------------YITI-GCPTTGGGQVISGNSAFLIEGIAIA
CVGDKATCPAHKTVSTIVSGDPNMQVM-GKAAARVNDSLSCGCKLLPKQSLV--------------------
------------------------------------------------------------------------
---------------------------------------NQG------------------------------
---------------------------------------
>AB34299_M
MASP--------------------------------------YITI-GCPTTGGGQVISGNSAFLIEGIAIA
CVGDKATCPAHKTVSTIVSGDPNMQVM-GKAAARVNDSLSCGCKLLPKQSLV--------------------
------------------------------------------------------------------------
---------------------------------------NQG------------------------------
---------------------------------------
>Ab4653_M
MASP--------------------------------------YITI-GCPTTGGGQVISGNSAFLIEGIAIA
CVGDKATCPAHKTVSTIVSGDPNMQVM-GKAAARVNDSLSCGCKLLPKQSLV--------------------
------------------------------------------------------------------------
---------------------------------------NQG------------------------------
---------------------------------------
>AB5075-UW_M
MASP--------------------------------------YITI-GCPTTGGGQVISGNSAFLIEGIAIA
CVGDKATCPAHKTVSTIVSGDPNMQVM-GKAAARVNDSLSCGCKLLPKQSLV--------------------
------------------------------------------------------------------------
---------------------------------------NQG------------------------------
---------------------------------------
>AbPK1_M

MASP--------------------------------------YITI-GCPTTGGGQVISGNSAFLIEGIAIA
CVGDKATCPAHKTVSTIVSGDPNMQVM-GKAAARVNDSLSCGCKLLPKQSLV--------------------
------------------------------------------------------------------------
---------------------------------------NQG------------------------------
---------------------------------------
>AC29_M
MASP--------------------------------------YITI-GCPTTGGGQVISGNSAFLIEGIAIA
CVGDKATCPAHKTVSTIVSGDPNMQVM-GKAAARVNDSLSCGCKLLPKQSLV--------------------
------------------------------------------------------------------------
---------------------------------------NQG------------------------------
---------------------------------------
>AC30_M
MASP--------------------------------------YITI-GCPTTGGGQVISGNSAFLIEGIAIA
CVGDKATCPAHKTVSTIVSGDPNMQVM-GKAAARVNDSLSCGCKLLPKQSLV--------------------
------------------------------------------------------------------------
---------------------------------------NQG------------------------------
---------------------------------------
>ACICU_M
MASP--------------------------------------YITI-GCPTTGGGQVISGNSAFLIEGIAIA
CVGDKATCPAHKTVSTIVSGDPNMQVM-GKAAARVNDSLSCGCKLLPKQSLV--------------------
------------------------------------------------------------------------
---------------------------------------NQG------------------------------
---------------------------------------
>AF-673_M
MASP--------------------------------------YITI-GCPTTGGGQVISGNSAFLIEGIAIA
CVGDKATCPAHKTVSTIVSGDPNMQVM-GKAAARVNDSLSCGCKLLPKQSLV--------------------
------------------------------------------------------------------------
---------------------------------------NQG------------------------------
---------------------------------------
>AR_0056_M
MASP--------------------------------------YITI-GCPTTGGGQVISGNSAFLIEGIAIA
CVGDKATCPAHKTVSTIVSGDPNMQVM-GKAAARVNDSLSCGCKLLPKQSLV--------------------
------------------------------------------------------------------------

---------------------------------------NQG------------------------------
---------------------------------------
>AR_0078_M
MASP--------------------------------------YITI-GCPTTGGGQVISGNSAFLIEGIAIA
CVGDKATCPAHKTVSTIVSGDPNMQVM-GKAAARVNDSLSCGCKLLPKQSLV--------------------
------------------------------------------------------------------------
---------------------------------------NQG------------------------------
---------------------------------------
>AR_0083_M
MASP--------------------------------------YITI-GCPTTGGGQVISGNSAFLIEGIAIA
CVGDKATCPAHKTVSTIVSGDPNMQVM-GKAAARVNDSLSCGCKLLPKQSLV--------------------
------------------------------------------------------------------------
---------------------------------------NQG------------------------------
---------------------------------------
>AR_0088_M
MASP--------------------------------------YITI-GCPTTGGGQVISGNSAFLIEGIAIA
CVGDKATCPAHKTVSTIVSGDPNMQVM-GKAAARVNDSLSCGCKLLPKQSLV--------------------
------------------------------------------------------------------------
---------------------------------------NQG------------------------------
---------------------------------------
>AYE_M
MASP--------------------------------------YITI-GCPTTGGGQVISGNSAFLIEGIAIA
CVGDKATCPAHKTVSTIVSGDPNMQVM-GKAAARVNDSLSCGCKLLPKQSLV--------------------
------------------------------------------------------------------------
---------------------------------------NQG------------------------------
---------------------------------------
>AYP-A2_M
MASP--------------------------------------YITI-GCPTTGGGQVISGNSAFLIEGIAIA
CVGDKATCPAHKTVSTIVSGDPNMQVM-GKAAARVNDSLSCGCKLLPKQSLV--------------------
------------------------------------------------------------------------
---------------------------------------NQG------------------------------
---------------------------------------
>BJAB07104_M

MASP--------------------------------------YITI-GCPTTGGGQVISGNSAFLIEGIAIA
CVGDKATCPAHKTVSTIVSGDPNMQVM-GKAAARVNDSLSCGCKLLPKQSLV--------------------
------------------------------------------------------------------------
---------------------------------------NQG------------------------------
---------------------------------------
>BJAB0715_M
MASP--------------------------------------YITI-GCPTTGGGQVISGNSAFLIEGIAIA
CVGDKATCPAHKTVSTIVSGDPNMQVM-GKAAARVNDSLSCGCKLLPKQSLV--------------------
------------------------------------------------------------------------
---------------------------------------NQG------------------------------
---------------------------------------
>BJAB0868_M
MASP--------------------------------------YITI-GCPTTGGGQVISGNSAFLIEGIAIA
CVGDKATCPAHKTVSTIVSGDPNMQVM-GKAAARVNDSLSCGCKLLPKQSLV--------------------
------------------------------------------------------------------------
---------------------------------------NQG------------------------------
---------------------------------------
>CBA7_M
MASP--------------------------------------YITI-GCPTTGGGQVISGNSAFLIEGIAIA
CVGDKATCPAHKTVSTIVSGDPNMQVM-GKAAARVNDSLSCGCKLLPKQSLV--------------------
------------------------------------------------------------------------
---------------------------------------NQG------------------------------
---------------------------------------
>CIP70.10_M
MASP--------------------------------------YITI-GCPTTGGGQVISGNSAFLIEGIAIA
CVGDKATCPAHKTVSTIVSGDPNMQVM-GKAAARVNDSLSCGCKLLPKQSLV--------------------
------------------------------------------------------------------------
---------------------------------------NQG------------------------------
---------------------------------------
>CMC-CR-MDR-Ab4_M
MASP--------------------------------------YITI-GCPTTGGGQVISGNSAFLIEGIAIA
CVGDKATCPAHKTVSTIVSGDPNMQVM-GKAAARVNDSLSCGCKLLPKQSLV--------------------
------------------------------------------------------------------------

---------------------------------------NQG------------------------------
---------------------------------------
>CMC-CR-MDR-Ab66_M
MASP--------------------------------------YITI-GCPTTGGGQVISGNSAFLIEGIAIA
CVGDKATCPAHKTVSTIVSGDPNMQVM-GKAAARVNDSLSCGCKLLPKQSLV--------------------
------------------------------------------------------------------------
---------------------------------------NQG------------------------------
---------------------------------------
>CMC-MDR-Ab59_M
MASP--------------------------------------YITI-GCPTTGGGQVISGNSAFLIEGIAIA
CVGDKATCPAHKTVSTIVSGDPNMQVM-GKAAARVNDSLSCGCKLLPKQSLV--------------------
------------------------------------------------------------------------
---------------------------------------NQG------------------------------
---------------------------------------
>D36_M
MASP--------------------------------------YITI-GCPTTGGGQVISGNSAFLIEGIAIA
CVGDKATCPAHKTVSTIVSGDPNMQVM-GKAAARVNDSLSCGCKLLPKQSLV--------------------
------------------------------------------------------------------------
---------------------------------------NQG------------------------------
---------------------------------------
>DU202_M
MASP--------------------------------------YITI-GCPTTGGGQVISGNSAFLIEGIAIA
CVGDKATCPAHKTVSTIVSGDPNMQVM-GKAAARVNDSLSCGCKLLPKQSLV--------------------
------------------------------------------------------------------------
---------------------------------------NQG------------------------------
---------------------------------------
>HRAB-85_M
MASP--------------------------------------YITI-GCPTTGGGQVISGNSAFLIEGIAIA
CVGDKATCPAHKTVSTIVSGDPNMQVM-GKAAARVNDSLSCGCKLLPKQSLV--------------------
------------------------------------------------------------------------
---------------------------------------NQG------------------------------
---------------------------------------
>HWAB8_M

MASP--------------------------------------YITI-GCPTTGGGQVISGNSAFLIEGIAIA
CVGDKATCPAHKTVSTIVSGDPNMQVM-GKAAARVNDSLSCGCKLLPKQSLV--------------------
------------------------------------------------------------------------
---------------------------------------NQG------------------------------
---------------------------------------
>KAB01_M
MASP--------------------------------------YITI-GCPTTGGGQVISGNSAFLIEGIAIA
CVGDKATCPAHKTVSTIVSGDPNMQVM-GKAAARVNDSLSCGCKLLPKQSLV--------------------
------------------------------------------------------------------------
---------------------------------------NQG------------------------------
---------------------------------------
>KAB03_M
MASP--------------------------------------YITI-GCPTTGGGQVISGNSAFLIEGIAIA
CVGDKATCPAHKTVSTIVSGDPNMQVM-GKAAARVNDSLSCGCKLLPKQSLV--------------------
------------------------------------------------------------------------
---------------------------------------NQG------------------------------
---------------------------------------
>MDR-TJ_M
MASP--------------------------------------YITI-GCPTTGGGQVISGNSAFLIEGIAIA
CVGDKATCPAHKTVSTIVSGDPNMQVM-GKAAARVNDSLSCGCKLLPKQSLV--------------------
------------------------------------------------------------------------
---------------------------------------NQG------------------------------
---------------------------------------
>MDR-ZJ06_M
MASP--------------------------------------YITI-GCPTTGGGQVISGNSAFLIEGIAIA
CVGDKATCPAHKTVSTIVSGDPNMQVM-GKAAARVNDSLSCGCKLLPKQSLV--------------------
------------------------------------------------------------------------
---------------------------------------NQG------------------------------
---------------------------------------
>NCGM237_M
MASP--------------------------------------YITI-GCPTTGGGQVISGNSAFLIEGIAIA
CVGDKATCPAHKTVSTIVSGDPNMQVM-GKAAARVNDSLSCGCKLLPKQSLV--------------------
------------------------------------------------------------------------

---------------------------------------NQG------------------------------
---------------------------------------
>R2090_M
MASP--------------------------------------YITI-GCPTTGGGQVISGNSAFLIEGIAIA
CVGDKATCPAHKTVSTIVSGDPNMQVM-GKAAARVNDSLSCGCKLLPKQSLV--------------------
------------------------------------------------------------------------
---------------------------------------NQG------------------------------
---------------------------------------
>R2091_M
MASP--------------------------------------YITI-GCPTTGGGQVISGNSAFLIEGIAIA
CVGDKATCPAHKTVSTIVSGDPNMQVM-GKAAARVNDSLSCGCKLLPKQSLV--------------------
------------------------------------------------------------------------
---------------------------------------NQG------------------------------
---------------------------------------
>SSA6_M
MASP--------------------------------------YITI-GCPTTGGGQVISGNSAFLIEGIAIA
CVGDKATCPAHKTVSTIVSGDPNMQVM-GKAAARVNDSLSCGCKLLPKQSLV--------------------
------------------------------------------------------------------------
---------------------------------------NQG------------------------------
---------------------------------------
>TCDC-0715_M
MASP--------------------------------------YITI-GCPTTGGGQVISGNSAFLIEGIAIA
CVGDKATCPAHKTVSTIVSGDPNMQVM-GKAAARVNDSLSCGCKLLPKQSLV--------------------
------------------------------------------------------------------------
---------------------------------------NQG------------------------------
---------------------------------------
>USA15_M
MASP--------------------------------------YITI-GCPTTGGGQVISGNSAFLIEGIAIA
CVGDKATCPAHKTVSTIVSGDPNMQVM-GKAAARVNDSLSCGCKLLPKQSLV--------------------
------------------------------------------------------------------------
---------------------------------------NQG------------------------------
---------------------------------------
>USA2_M

MASP--------------------------------------YITI-GCPTTGGGQVISGNSAFLIEGIAIA
CVGDKATCPAHKTVSTIVSGDPNMQVM-GKAAARVNDSLSCGCKLLPKQSLV--------------------
------------------------------------------------------------------------
---------------------------------------NQG------------------------------
---------------------------------------
>WCHAB005078_M
MASP--------------------------------------YITI-GCPTTGGGQVISGNSAFLIEGIAIA
CVGDKATCPAHKTVSTIVSGDPNMQVM-GKAAARVNDSLSCGCKLLPKQSLV--------------------
------------------------------------------------------------------------
---------------------------------------NQG------------------------------
---------------------------------------
>WCHAB005133_M
MASP--------------------------------------YITI-GCPTTGGGQVISGNSAFLIEGIAIA
CVGDKATCPAHKTVSTIVSGDPNMQVM-GKAAARVNDSLSCGCKLLPKQSLV--------------------
------------------------------------------------------------------------
---------------------------------------NQG------------------------------
---------------------------------------
>XDR-BJ83_M
MASP--------------------------------------YITI-GCPTTGGGQVISGNSAFLIEGIAIA
CVGDKATCPAHKTVSTIVSGDPNMQVM-GKAAARVNDSLSCGCKLLPKQSLV--------------------
------------------------------------------------------------------------
---------------------------------------NQG------------------------------
---------------------------------------
>XH386_M
MASP--------------------------------------YITI-GCPTTGGGQVISGNSAFLIEGIAIA
CVGDKATCPAHKTVSTIVSGDPNMQVM-GKAAARVNDSLSCGCKLLPKQSLV--------------------
------------------------------------------------------------------------
---------------------------------------NQG------------------------------
---------------------------------------
>XH856_M
MASP--------------------------------------YITI-GCPTTGGGQVISGNSAFLIEGIAIA
CVGDKATCPAHKTVSTIVSGDPNMQVM-GKAAARVNDSLSCGCKLLPKQSLV--------------------
------------------------------------------------------------------------

---------------------------------------NQG------------------------------
---------------------------------------
>XH857_M
MASP--------------------------------------YITI-GCPTTGGGQVISGNSAFLIEGIAIA
CVGDKATCPAHKTVSTIVSGDPNMQVM-GKAAARVNDSLSCGCKLLPKQSLV--------------------
------------------------------------------------------------------------
---------------------------------------NQG------------------------------
---------------------------------------
>XH858_M
MASP--------------------------------------YITI-GCPTTGGGQVISGNSAFLIEGIAIA
CVGDKATCPAHKTVSTIVSGDPNMQVM-GKAAARVNDSLSCGCKLLPKQSLV--------------------
------------------------------------------------------------------------
---------------------------------------NQG------------------------------
---------------------------------------
>XH859_M
MASP--------------------------------------YITI-GCPTTGGGQVISGNSAFLIEGIAIA
CVGDKATCPAHKTVSTIVSGDPNMQVM-GKAAARVNDSLSCGCKLLPKQSLV--------------------
------------------------------------------------------------------------
---------------------------------------NQG------------------------------
---------------------------------------
>XH860_M
MASP--------------------------------------YITI-GCPTTGGGQVISGNSAFLIEGIAIA
CVGDKATCPAHKTVSTIVSGDPNMQVM-GKAAARVNDSLSCGCKLLPKQSLV--------------------
------------------------------------------------------------------------
---------------------------------------NQG------------------------------
---------------------------------------
>ZW85-1_M
MASP--------------------------------------YITI-GCPTTGGGQVISGNSAFLIEGIAIA
CVGDKATCPAHKTVSTIVSGDPNMQVM-GKAAARVNDSLSCGCKLLPKQSLV--------------------
------------------------------------------------------------------------
---------------------------------------NQG------------------------------
---------------------------------------
>3207_M

MASP--------------------------------------YITI-GCPTTGGGQVISGNSAFLIDGIAIA
CVGDKATCPAHKTVSTIVSGDPNMQVM-GKAAARVNDSLSCGCKLLPKQSLV--------------------
------------------------------------------------------------------------
---------------------------------------NQG------------------------------
---------------------------------------
>AB030_M
MASP--------------------------------------YITI-GCPTTGGGQVISGNSAFLIDGIAIA
CVGDKATCPAHKTVSTIVSGDPNMQVM-GKAAARVNDSLSCGCKLLPKQSLV--------------------
------------------------------------------------------------------------
---------------------------------------NQG------------------------------
---------------------------------------
>AB042_M
MASP--------------------------------------YITI-GCPTTGGGQVISGNSAFLIDGIAIA
CVGDKATCPAHKTVSTIVSGDPNMQVM-GKAAARVNDSLSCGCKLLPKQSLV--------------------
------------------------------------------------------------------------
---------------------------------------NQG------------------------------
---------------------------------------
>Ab04-mff_M
MASP--------------------------------------YITI-GCPTTGGGQVISGNSAFLIDGIAIA
CVGDKATCPAHKTVSTIVSGDPNMQVM-GKAAARVNDSLSCGCKLLPKQSLV--------------------
------------------------------------------------------------------------
---------------------------------------NQG------------------------------
---------------------------------------
>ab736_M
MASP--------------------------------------YITI-GCPTTGGGQVISGNSAFLIDGIAIA
CVGDKATCPAHKTVSTIVSGDPNMQVM-GKAAARVNDSLSCGCKLLPKQSLV--------------------
------------------------------------------------------------------------
---------------------------------------NQG------------------------------
---------------------------------------
>AbH12O-A2_M
MASP--------------------------------------YITI-GCPTTGGGQVISGNSAFLIDGIAIA
CVGDKATCPAHKTVSTIVSGDPNMQVM-GKAAARVNDSLSCGCKLLPKQSLV--------------------
------------------------------------------------------------------------

---------------------------------------NQG------------------------------
---------------------------------------
>AF-401_M
MASP--------------------------------------YITI-GCPTTGGGQVISGNSAFLIDGIAIA
CVGDKATCPAHKTVSTIVSGDPNMQVM-GKAAARVNDSLSCGCKLLPKQSLV--------------------
------------------------------------------------------------------------
---------------------------------------NQG------------------------------
---------------------------------------
>AR_0063_M
MASP--------------------------------------YITI-GCPTTGGGQVISGNSAFLIDGIAIA
CVGDKATCPAHKTVSTIVSGDPNMQVM-GKAAARVNDSLSCGCKLLPKQSLV--------------------
------------------------------------------------------------------------
---------------------------------------NQG------------------------------
---------------------------------------
>AR_0101_M
MASP--------------------------------------YITI-GCPTTGGGQVISGNSAFLIDGIAIA
CVGDKATCPAHKTVSTIVSGDPNMQVM-GKAAARVNDSLSCGCKLLPKQSLV--------------------
------------------------------------------------------------------------
---------------------------------------NQG------------------------------
---------------------------------------
>ATCC17978-mff_M
MASP--------------------------------------YITI-GCPTTGGGQVISGNSAFLIDGIAIA
CVGDKATCPAHKTVSTIVSGDPNMQVM-GKAAARVNDSLSCGCKLLPKQSLV--------------------
------------------------------------------------------------------------
---------------------------------------NQG------------------------------
---------------------------------------
>ATCC19606_M
MASP--------------------------------------YITI-GCPTTGGGQVISGNSAFLIDGIAIA
CVGDKATCPAHKTVSTIVSGDPNMQVM-GKAAARVNDSLSCGCKLLPKQSLV--------------------
------------------------------------------------------------------------
---------------------------------------NQG------------------------------
---------------------------------------
>WKA02_M

MASP--------------------------------------YITI-GCPTTGGGQVISGNSAFLIDGIAIA
CVGDKATCPAHKTVSTIVSGDPNMQVM-GKAAARVNDSLSCGCKLLPKQSLV--------------------
------------------------------------------------------------------------
---------------------------------------NQG------------------------------
---------------------------------------
>AB307-0294_C
MATP--------------------------------------YITI-GCPTSGGGQVISGNSMFLIDGIAVA
CVGDKATCPTHKVVATIISGDPNMQIF-GKAAARVNDSLSCGCKLLPQQNLVVQDN-GGAASSGAKSSTAPM
SQKQQTTNSFREEKYENYYIEQYKTDVYISHKAVLLGDEGVTPL--------DGAVSYFLNYKVQGKELFLS
VVINAAPLSHKGTVYPFGTAIVSREGKEITR---AKLKSDKG---YWPTDKNKAPLGSCTIKLPEPNLQLVD
VELELGYTAVISDTVGSVHPMPPIKKYKFSLNSAARKV-
>AbH12O-A2_C
LATP--------------------------------------YITI-GCPTTGGGQVISGNSMFLIDGIPVA
CVGDKATCPTHKVVATIVSGDPNMQIF-GKAAARVNDSLSCGCKLLPQQNLVVQDNGGGAASSASKSSSAPI
SQKQQTTDSFREEKYENYYIEQYKTDVYISHKAVLLGDEGVTPL--------DGAVSYFLNYKVQGKELFLS
VVINAAPLSHKGTVYPFGTAIVSREGKEIAR---TKLKSDQG---YWPTDKNKAPLGSCTIKLPEPNLQLVD
VELELGYTAVISDTVGSVHPMPPIKKYKFSLNSAARKV-
>AB030_C
MATP--------------------------------------YITI-GCPTTGGGQVISGNSMFLIDGIPVA
CVGDKATCPTHKVVATIVSGDPNMQIF-GKAAARVNDSLSCGCKLLPQQNLVVQDNGGGAASSASKSSSAPI
SQKQQTTDSFREEKYENYYIEQYKTDVYISHKAVLLGDEGVTPL--------DGAVSYFLNYKVQGKELFLS
VVINAAPLSHKGTVYPFGTAIVSREGKEIAR---TKLKSDQG---YWPTDKNKAPLGSCTIKLPEPNLQLVD
VELELGYTAVISDTVGSVHPMPPIKKYKFSLNSAARKV-
>AF-401_C
MATP--------------------------------------YITI-GCPTTGGGQVISGNSMFLIDGIPVA
CVGDKATCPTHKVVATIVSGDPNMQIF-GKAAARVNDSLSCGCKLLPQQNLVVQDNGGGAASSASKSSSAPI
SQKQQTTDSFREEKYENYYIEQYKTDVYISHKAVLLGDEGVTPL--------DGAVSYFLNYKVQGKELFLS
VVINAAPLSHKGTVYPFGTAIVSREGKEIAR---TKLKSDQG---YWPTDKNKAPLGSCTIKLPEPNLQLVD
VELELGYTAVISDTVGSVHPMPPIKKYKFSLNSAARKV-
>AR_0063_C
MATP--------------------------------------YITI-GCPTTGGGQVISGNSMFLIDGIPVA
CVGDKATCPTHKVVATIVSGDPNMQIF-GKAAARVNDSLSCGCKLLPQQNLVVQDNGGGAASSASKSSSAPI
SQKQQTTDSFREEKYENYYIEQYKTDVYISHKAVLLGDEGVTPL--------DGAVSYFLNYKVQGKELFLS

VVINAAPLSHKGTVYPFGTAIVSREGKEIAR---TKLKSDQG---YWPTDKNKAPLGSCTIKLPEPNLQLVD
VELELGYTAVISDTVGSVHPMPPIKKYKFSLNSAARKV-
>AR_0101_C
MATP--------------------------------------YITI-GCPTTGGGQVISGNSMFLIDGIPVA
CVGDKATCPTHKVVATIVSGDPNMQIF-GKAAARVNDSLSCGCKLLPQQNLVVQDNGGGAASSASKSSSAPI
SQKQQTTDSFREEKYENYYIEQYKTDVYISHKAVLLGDEGVTPL--------DGAVSYFLNYKVQGKELFLS
VVINAAPLSHKGTVYPFGTAIVSREGKEIAR---TKLKSDQG---YWPTDKNKAPLGSCTIKLPEPNLQLVD
VELELGYTAVISDTVGSVHPMPPIKKYKFSLNSAARKV-
>A1268_C
MATP--------------------------------------YITI-GCPTTGGGQVISGNSMFLIDGIPIA
CVGDKATCPTHKVVATIVSGDPNMQIF-GKAAARVNDSLSCGCKLLPQQNLVVQDN-GGAASSGAKSSSAPM
SQKQQTTDSFREEKYENYYIEQYKTDVYISHKAVLLGDEGVTPL--------DGAVSYFLNYKVQGKELFLS
VVINAAPLSHKGTVYPFGTAIVSREGKEIAR---TKLKSDQG---YWPTDKNKAPLGSCTIKLPEPNLQLVD
VELELGYTAVISDTVGSVHPMPPIKKYKFSLNSAARKV-
>AB042_C
MATP--------------------------------------YITI-GCPTTGGGQVISGNSMFLIDGIPVA
CVGDKATCPTHKVVATIVSGDPNMQIF-GKAAARVNDSLSCGCKLLPQQNLVVQDN-GGAASSGAKSSSAPM
SQKQQTTDSFREEKYENYYIEQYKTDVYISHKAVLLGDEGVTPL--------DGAVSYFLNYKVQGKELFLS
VVINAAPLSHKGTVYPFGTAIVSREGKEIAR---TKLKSDQG---YWPTDKNKAPLGSCTIKLPEPNLQLVD
VELELGYTAVISDTVGSVHPMPPIKKYKFSLNSAARKV-
>AR_0078_C
MATP--------------------------------------YITI-GCPTTGGGQVISGNSMFLIDGIPVA
CVGDKATCPTHKVVATIVSGDPNMQIF-GKAAARVNDSLSCGCKLLPQQNLVVQDN-GGAASSGAKSSSAPM
SQKQQTTDSFREEKYENYYIEQYKTDVYISHKAVLLGDEGVTPL--------DGAVSYFLNYKVQGKELFLS
VVINAAPLSHKGTVYPFGTAIVSREGKEIAR---TKLKSDQG---YWPTDKNKAPLGSCTIKLPEPNLQLVD
VELELGYTAVISDTVGSVHPMPPIKKYKFSLNSAARKV-
>ATCC17978-mff_C
MATP--------------------------------------YITI-GCPTTGGGQVISGNSMFLIDGIPVA
CVGDKATCPTHKVVATIVSGDPNMQIF-GKAAARVNDSLSCGCKLLPQQNLVVQDN-GGAASSGAKSSSAPM
SQKQQTTDSFREEKYENYYIEQYKTDVYISHKAVLLGDEGVTPL--------DGAVSYFLNYKVQGKELFLS
VVINAAPLSHKGTVYPFGTAIVSREGKEIAR---TKLKSDQG---YWPTDKNKAPLGSCTIKLPEPNLQLVD
VELELGYTAVISDTVGSVHPMPPIKKYKFSLNSAARKV-
>SDF_D

```
------------------------------------------------------------------MGNLFV
RAGDGHMCPKCKCWSVVIKSHDHMIMD-GKPVAYAGDKLTCGATIQPQQSHVVGDS-VSPYSYASTTSQSLS
PQNQLTTNSFVKDEYENYYIDNSKEERVEFKNFLFPYDQDKGNLIGIIAQGVSGKCTFVVNRKNQNTSLFVS
VTLLPPVLKNDAKIYPKASIQLLKKGKPLGP--IEPMPIGKG---FWNTEKDMQPVGSIELTLPPPDLEPVT
VKLRLSYTGWIDG--GQVVPNPSYINHEFTITSAARRKA
>ACICU_H
MAKS--------------------------------------FAIN-MAPTNHGGVVPATQSRTSQEGNLFV
RAGDGHMCPKCKCWSVVIKSHDHVIMD-GKPVAYVGDKLSCGATIMPQQFHVVGDSGSPYSSAASSAAPVQN
SLVEDKSNEIHKVQFKLVDVDTDQ------------------------------------------------
---------------PLSAMLYEIHSKESGKLLVQGYTDKNGMTAIYESEHTPESVQLITVDLSKPLDPL--
---------------------------------------
>SDF_H
MSKS--------------------------------------FAVH-MAPTNHGGMIPATQSRSSQQGNLFV
RAGDGHMCPKCKCWSVVIKSHDHVIFD-GKPVAYVGDSLSCGAKIVLQQFHVVGDSGSPYSSAASSTAPVQD
SLVEDKSNEIHKVQFKLVDVDTNQ------------------------------------------------
---------------PLSAMLYEIHSKDSGKLLVQGYTDKNGMTAIYESEHTPESVQLITVDLSKPLDPL--
---------------------------------------
>PAAR2
MAKG--------------------------------------FAIH-NAPTNHGGIIPSTQVRGSQQGNLFV
RAGDGHFCPKCKCWSTVIKSHDHVIMD-GKPVAYAGDKLTCGATIQPQQSHVVGDS-GSYYNGSTNFSQNLL
DSQKGIFDEQIQVEVNNSFSDLF----------------------------------LGLAYRLKVDGNVIE
GTLDSNG----------KTLRFETKKQSQVTEFEIFFKENIK---YFSDLEGEE------------------
---------------------------------------
>SDF_K
-MKG--------------------------------------FAIH-NAITDHGGIIPSTQMRTSQMGNLFV
RAGDGHMCPKCKCWSVVIKSHDHIIMD-GKPVAYAGDKLSCGATIQPQQSHVVGDSGSPYSYAASSAAPA--
------QDSFVEEKKE-----------------------------------------YGIQFQLKDEKTQKI
FSDIPYSI-------------IYKKDGKIET--------------GWTDKEGKTHV----------------
----------------INAATPDEVEFQTIDASKPLPPL
>ab736_L
MAKG--------------------------------------FAIH-NAITDHGGVIPSTQAISSQMGNLFV
RAGDGHMCPKCRCWSVVIKSHDHVIFD-GKPVAYVGDKLSCGATIQPQQSHVVGESGSPYSRSSDNTNNSSF
VPTL---------QYGQRFLLQDELTGEPLSNICYEIEKGGNIIHGKTDE--NGFTDLITSENKEEIQIHI-
```

```
----------------------------------IYEEHDHG------------------------------
---------------------------------------
>ATCC19606_L
MAKG--------------------------------------FAIH-NAITDHGGVIPSTQAISSQMGNLFV
RAGDGHMCPKCRCWSVVIKSHDHVIFD-GKPVAYVGDKLSCGATIQPQQSHVVGESGSPYSRSSDNTNNSSF
VPTL---------QYGQRFLLQDELTGEPLSNICYEIEKGGNIIHGKTDE--NGFTDLITSENKEEIQIHI-
----------------------------------IYEEHDHG------------------------------
---------------------------------------
>6200_E
MAKG--------------------------------------FAIH-NAITDHGGVIPSTQSRSSQMGNLFV
RAGDGHMCPKCRCWSVVIKSHDHVIFD-GKPVAYAGDKLSCGATIQPQQSHVVGESGSPYSGKDSNTQSQKY
DEQLKITFNDETDELLKYF-----------------------------------DCRVKIGENFRNANLDIT
G----------------KTNRFYTEGKEPITEIELILKDNII---YFDEL----------------------
---------------------------------------
>A1268_F
MAKG--------------------------------------FAIH-NAITDHGGVIPSTQSRSSQMGNLFV
RAGDGHMCPKCRCWSVVIKSHDHIIMD-GKPVAYAGDKLSCGATIQPQQSHVVGDSGSPYSAKETTQKNNFV
ENNIEKKVTEITWSYGNNFNPLS----------------DKSRFFNDLN------IHVKTTGYSQGENVSIS
---IEPENSDISTFEPFAITIKVDSN-------------GEG---ILKDVFSGK------------------
---TLIIDTEY----------------------------
```
